# Selection of allosteric dnazymes that can sense phenylalanine by expression-SELEX

**DOI:** 10.1093/nar/gkad424

**Published:** 2023-05-19

**Authors:** Binfen Chen, Xinmei Yu, Ting Gao, Yaoyao Wu, Xiaojun Zhang, Sanshu Li

**Affiliations:** Medical School, Huaqiao University, Xiamen 361021, P.R. China; Medical School, Huaqiao University, Xiamen 361021, P.R. China; Medical School, Huaqiao University, Xiamen 361021, P.R. China; Medical School, Huaqiao University, Xiamen 361021, P.R. China; Chemical Engineering Institute, Huaqiao University, Xiamen 361021, P.R. China; Medical School, Engineering Research Center of Molecular Medicine of Ministry of Education, Key Laboratory of Precision Medicine and Molecular Diagnosis of Fujian Universities, Institute of Genomics, Huaqiao University, Xiamen 361021, P.R. China

## Abstract

Aptamers are ligand-binding RNA or DNA molecules and have been widely examined as biosensors, diagnostic tools, and therapeutic agents. The application of aptamers as biosensors commonly requires an expression platform to produce a signal to report the aptamer-ligand binding event. Traditionally, aptamer selection and expression platform integration are two independent steps and the aptamer selection requires the immobilization of either the aptamer or the ligand. These drawbacks can be easily overcome through the selection of allosteric DNAzymes (aptazymes). Herein, we used the technique of Expression-SELEX developed in our laboratory to select for aptazymes that can be specifically activated by low concentrations of l-phenylalanine. We chose a previous DNA-cleaving DNAzyme known as II-R1 as the expression platform for its low cleavage rate and used stringent selection conditions to drive the selection of high-performance aptazyme candidates. Three aptazymes were chosen for detailed characterization and these DNAzymes were found to exhibit a dissociation constant for l-phenylalanine as low as 4.8 μM, a catalytic rate constant improvement as high as 20 000-fold in the presence of l-phenylalanine, and the ability to discriminate against closely related l-phenylalanine analogs including d-phenylalanine. This work has established the Expression-SELEX as an effective SELEX method to enrich high-quality ligand-responsive aptazymes.

## Graphical Abstract

**Graphical Abstract:**
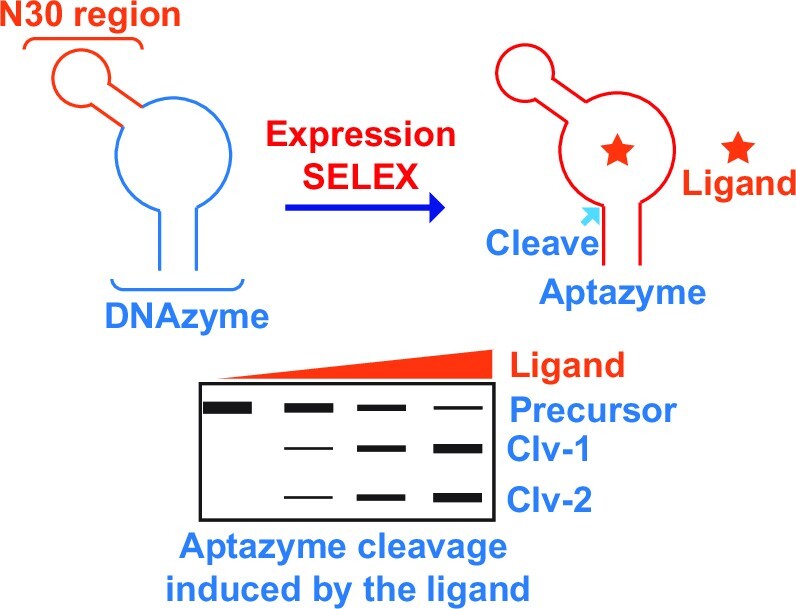

## INTRODUCTION

Aptamers are ssDNA or RNA that can bind to a variety of ligands, such as proteins, peptides, carbohydrates, and other molecules. Aptamers have been engineered and applied in various research fields, including biosensing (1–4), diagnostics and therapeutics (5), regulation of gene expression (6–9), logic gate operations (10,11) and the development of novel synthetic and biological tools (12,13). The theophylline aptamer and the VEGF aptamer are two well-known aptamers used as biosensors and gene-expression regulators (5,14).

SELEX was invented in 1990 to select aptamers that can bind to T4 DNA polymerase (15) or organic dye molecules (16) or to select ribozymes that can cleave single stranded DNA (17). Later, different improved SELEX techniques have been invented, including negative SELEX (18), counter SELEX (14), capillary electrophoresis SELEX (CE-SELEX) (19), Cell SELEX (20–22), *in vivo* SELEX (23) and Capture-SELEX (24). Based on these techniques, many aptamers have been generated. However, the application of aptamers commonly requires an expression platform to receive the signal from the aptamer-ligand binding event.

Many aptamers have been combined with an expression such as a DNAzyme, a ribozyme, a ribosome-binding site (RBS), a Rho-independent terminator, or a poly (A) signaling sequence to facilitate their applications (8,12,13). An ATP aptamer was fused with the hammerhead ribozyme to form the first allosteric ribozyme whose cleavage can be controlled by ATP (2). Moreover, a natural c-di-GMP class I riboswitch aptamer was joined to a hammerhead ribozyme to sense bacterial c-di-GMP (25). Recently, a TPP riboswitch aptamer was fused with a hammerhead ribozyme to detect the blood concentration of thiamine pyrophosphate, a potential biomarker for Alzheimer's disease (26).

The first DNAzyme was isolated by Breaker and Joyce in 1994 by SELEX using a library of ssDNA with a target ribonucleotide in the middle (27). Later, two more classes of self-cleaving DNAs were produced, one of which requires Cu^2+^ and ascorbate as co-effectors, while the other requires Cu^2+^ only (28). Recently, small highly-active DNAzymes that require Zn^2+^ have been generated (29). Additionally, an L-RNA cleaving DNAzyme has been isolated and fused with an ATP-binding aptamer to form an allosteric DNAzyme (30). These DNAzymes can potentially be used as expression platforms for DNA aptamers.

Historically, the selection of an aptamer and the fusion of an expression platform with the aptamer are two separate steps. Recently, we integrated a highly active DNAzyme (I-R3), which was generated by the Breaker's lab in 2013 (29), as an expression platform into the SELEX cycle (termed Expression-SELEX (31)) to merge these two steps into one step. Expression-SELEX does not require immobilizing either the random sequence library or the ligand. The embedded DNAzyme I-R3 also facilitates the isolation of the cleavage product for the next cycle selection without the need to immobilize either ligands or DNA libraries. However, the previously isolated allosteric DNAzyme (aptazyme) bound to the ligand weakly and induced only slight DNA cleavage.

In this study, we improved Expression-SELEX further by the use of a new expression platform II-R1 DNAzyme, the application of an exonuclease to digest the dsDNA to obtain higher quality ssDNA, and the increment of negative incubation time to eliminate self-cleaving DNA aptazymes in the absence of a ligand. With these improvements, within six rounds of selection, we isolated several allosteric DNAzymes that can bind to the amino acid l-phenylalanine, which is the biomarker of phenylketonuria with high affinity and high inducible cleavage. The improved Expression-SELEX can be applied to select aptazymes that can bind to various ligands with high affinity in a few selection cycles.

## MATERIALS AND METHODS

### Expression-SELEX screening process

Step 1: Negative selection. The single-stranded DNA (ssDNA) random library II-R1 random-sequence library was dissolved with molecules up to 1 × 10^15^ in HEPES buffer (0.05 M HEPES, 0.1 M NaCl, 0.04 M MgCl_2_, 1 mM ZnCl_2_, pH 7.05), and then incubated at 37°C for 12 h; Full-length ssDNA was separated by 10% urea-denaturing polyacrylamide gel electrophoresis (PAGE) and the corresponding ssDNA band was isolated; the gel pieces were crushed and soaked in the elution buffer (Tris–HCl 10 mM, pH 7.5 at 23°C, 200 mM NaCl and 1 mM EDTA) and recovered by ethanol precipitation. Step 2: Positive selection. The recovered full-length ssDNA was dissolved in HEPES buffer containing the ligand L-Phenylalanine (different ligand concentrations in different rounds) and incubated at 37°C for 10–30 min. Step 3: Selection of cleaved products. The 3′cleavage product (80 nt) was separated by 10% PAGE and the corresponding ssDNA band was isolated and purified. Step 4: PCR amplification using Taq DNA polymerase (ABM company). Primers II-forward (10 μM) and II-reverse (10 μM, containing a 5′phosphate group) (Supplementary file No. S1Supplementary Table S1) were used to amplify the 3′cleavage product. Step 5: Exonuclease digestion to obtain single-strand DNA (Supplementary files No. S1Supplementary Figure S1). The purified PCR product (5 μg) was incubated with 5 units of lambda exonuclease (New England Biolabs) at 37°C for 30 min to digest the DNA antisense strand with the 5′phosphate group. The purified full-length ssDNA was used for the next round of screening. When the screening was conducted on the 19th and 20th rounds, a phenylalanine analog (4-fluoro-dl-phenylalanine) was used for counter-selection.

### Next-generation sequencing (NGS) of the SELEX-enriched aptazymes

Sequencing was performed using a previously described method (31). Briefly, the P5-forward (10 μM) and P7-reverse (10 μM) (Supplementary file No. S1, Supplementary Table S1) from the Illumina platform were appended to the end of the ssDNA by PCR using Taq DNA polymerase. The PCR product was purified by an agarose gel DNA recovery kit (Sangon Biotech) and sent to Beijing Novogene Company for sequencing.

### Analysis of the secondary structures of the allosteric DNAzyme candidates

The analysis method is similar to the one described previously (32). We used perl scripts to analyze the high-through-put sequencing results for these enriched libraries to list all unique reads in ranked order (Supplementary files No. S2–S4). To search for homologous sequences and structures that are similar to those top-enriched sequences, we first extracted the 30 nt random regions of top 1000 sequences (Supplementary files No. S5), use BLAST to look for higher than 90% similarity between each top sequence and the top 1000 sequences (Supplementary files No. S6). Therefore, each top sequence and its homologues form a group of similar sequences. The full-length of these sequences were obtained by a perl script (Supplementary files No. S7), and then they were aligned by Clustal Omega (33) (Supplementary files No. S8). The resulting alignment files were used for RNAalifold (34) to predict their structures (Supplementary files No. S9) based on minimal free energy and nucleotide covariance (34). The resulting Stockholm files containing structural information were used for R2R to draw the secondary structures (35) (Figure 2 and Supplementary files No. S1Supplementary Figure S3).

### DNA aptazyme cleavage assays

Cleavage assays were performed under conditions similar to those described previously (31). Briefly, approximately 100 ng of ssDNA was incubated with or without the ligand L-Phenylalanine in cleavage buffer (0.05 M HEPES, 0.1 M NaCl, 0.04 M MgCl_2_, 1 mM ZnCl_2_, pH 7.05) in a final volume of 20 μl. Cleavage products were separated on the 15% PAGE gel and visualized by staining with SYBR Gold (Invitrogen). The fraction of cleavage was determined as the intensity of the 3′ cleavage DNA products divided by the total ssDNA.

### Dissociation constant (*K*_D_) measurements

The apparent *K*_D_ values were determined by a previously described method (31). The *K*_D_ was calculated using GraphPad Prism with the function of specific binding with Hill slope 1.0 and the equation: *Y* = *B*_max_ × *X*/(*K*_D_ + *X*), where *B*_max_ is the maximum-specific binding.

### Observed rate constant (*k*_obs_) measurements

Measurements of *k*_obs_ values were performed as described previously (31). Briefly, we performed cleavage of the ssDNA aptazymes with and without a ligand in the cleavage buffer. The fraction of cleavage at each time point was calculated as described above. The *k*_obs_ values for each reaction were measured using GraphPad Software with one phase decay and the equation: *Y* = (*Y*_0_ – plateau) × exp (–*k*_obs_ × *X*) + plateau, where *Y*_0_ is the *Y* value when *X* (time) is zero, Plateau is the *Y* value at infinite times, and *k*_obs_ is the rate constant.

## RESULTS

### Integrating the II-R1 DNA enzyme (DNAzyme) into the random library

**Figure 1.**
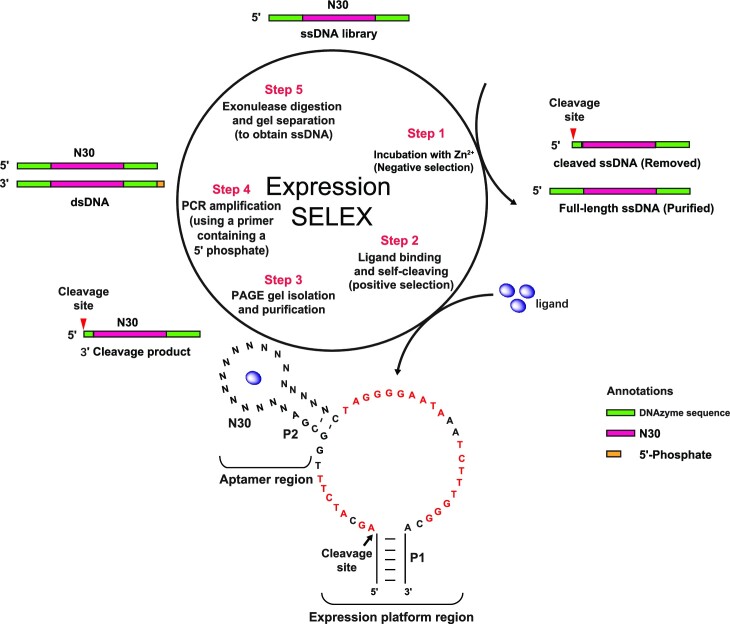
Selection cycles of Expression-SELEX. Step 1: Negative selection. The library was incubated with buffer containing 1 mM Zn^2+^ for 12 h, and the uncleaved full-length ssDNA was purified with 10% PAGE gel. Step 2: Positive selection. The purified full-length ssDNA was incubated with l-phenylalanine to induce the cleavage of the embedded DNAzyme II-R1. Step 3: Isolation of the 3′cleavage product by a PAGE gel. Step 4: Amplification by PCR (using Taq DNA polymerase) with specific primers. Step 5: Digestion of antisense DNA strand with Lambda exonuclease to purify the aptazymes. The purified aptazymes were used for the next selection cycle.

We chose the II-R1 DNAzyme as an expression platform for its slow cleavage rate. We expected that the binding of ligands would enable the DNAzyme to reorganize and stabilize the structure and the cleavage rate would be improved, would facilitate the isolation of a ligand-responsive DNAzyme. The new DNAzymes to be selected will be called allosteric DNAzymes or aptazymes in this work. The initial library included ssDNA sequences in which 30 random nucleotides were integrated into the P2 stem of the II-R1 DNAzyme (the aptazyme in Figure 1) and the new II-R1 aptazymes were expected to perform self-cleaving reaction upon ligand binding. The cleavage products were then isolated by denatured PAGE and used for PCR amplification.

### Enrichment of aptazymes by expression-SELEX

The double-stranded (ds) PCR amplicons of the cleaved products were digested by the lambda exonuclease to obtain full-length ssDNA aptazyme candidates. Our experiment showed that exonuclease digestion produced a highly pure ssDNA with a single band on the gel, while the traditional method using embedded RNA nucleotide in the dsDNA and degraded by NaOH could not (Supplementary Figure S1, all supplementary figures are in the Supplementary file No. S1). In addition, we extended the negative selection step up to 12 h to obtain full-length ssDNA with extremely low background cleavage activity. More detailed steps (Figure 1) are described in the method.

In addition, the ligand concentration and incubation time were varied during different cycles to obtain high affinity and specificity of ligand binding by the ssDNA aptazymes (Supplemary2 Supplementary Figure S2). The first round started from the ssDNA library commercially acquired. Compared to the cleavage of the second-round ssDNA pool, the cleavage of the ssDNA pool for each round in the following cycles increased gradually. The sixth round reached the first enrichment peak. Approximately 10% of the total ssDNA can respond to the ligand induction and cleave itself. We then reduced the concentration of the ligand from 1 mM to 0.5 mM, and after another four rounds of selection, the fraction of cleavage had increased to approximately 20% in the 10th round. From this point, we reduced the incubation time from 20 min to 10 min and further decreased the ligand concentration to 0.25 mM.

### Identification of enriched self-cleaving aptazymes and their family members

High-throughput sequencing was employed to analyze the ssDNA sequences in the enriched libraries from the 6th, 14th and 20th round selection (Supplementary file No. S2–S4). The results showed that the top four most abundant sequences in these three libraries are the same (Supplementary file No. S1Supplementary Table S2). The most abundant sequence (II-R1-1) was enriched from 0.9% to 45.6%, the second most abundant sequence (II-R1-2) was enriched from 0.14% to 20.9%, and the third most abundant sequence (II-R1-3) was enriched from 0.07% to 7.1% (Supplementary file No. S2–S4).

**Figure 2.**
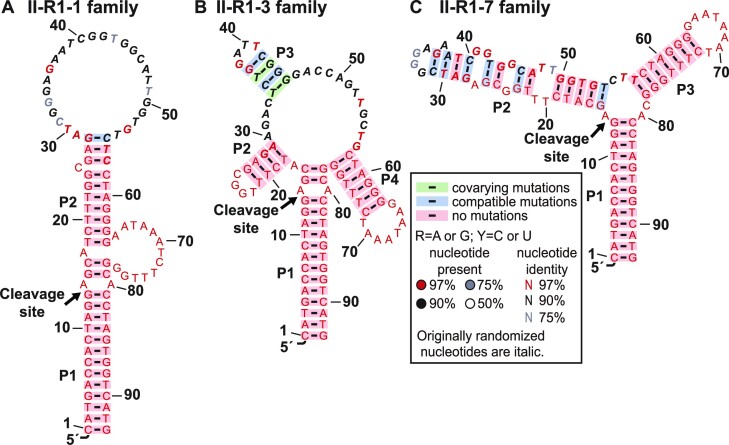
Predicted structures and sequence consensus of enriched aptazymes that can self-cleave upon ligand-binding. Three randomly picked aptazyme candidates II-R1-1, II-R1-3 and II-R1-7 were aligned to top 1000 enriched sequences with more than 90 percent similarity. The secondary structures were analyzed by RNAalifold. (**A**) Consensus sequence and secondary structure of 209 homologs of the II-R1-1aptazyme family in the 20th round library. (**B**) Consensus sequence and secondary structure of 82 homologs of the II-R1-3 aptazyme family. (**C**) Consensus sequence and secondary structure of 19 homologs of the II-R1-7 aptazyme family. Annotations are embedded in the box. 30 nucleotides originated from the random-region of the DNA library are in italic font.

We arbitrarily picked candidates II-R1-1, II-R1-3 and II-R1-7 among the top 10 enriched candidates (Supplementary Figure S3A–C) for cleavage assay under the ligand induction. The initial results showed that all of them can self-cleave when the ligand was present while almost no cleavage happened when the ligand was absent, suggesting that they are all allosteric DNAzymes. Subsequently, we used these candidates as starting sequences to search for their homologs among the top 1000 enriched sequences. First, we used BLAST to search for the sequences that are >90% similar to the starting sequence (for 30 nt random region). Then, full-length (93 nt) homologs were aligned by the Clustal Omega (33). Finally, the secondary structure was predicted by the RNAalifold algorithm (34) based on the minimal free-energy and nucleotide covariance. To visualize the secondary structure from a Stockholm file obtained from RNAalifold, R2R program (35) was applied to draw the figure (Figure 2). II-R1-1 aptazyme family contains a big loop at the top and an internal loop between P1 and P2 stems (Figure 2A and Supplementary Figure S3A). II-R1-3 aptazyme family is consisted of four stems forming a four-way junction with two covariations in the P3 stem (Figure 2B and Supplementary Figure S3A). II-R1-7 aptazyme family possesses three stems with two internal loops in the P2 stem (Figure 2C and Supplementary Figure S3C). These top candidates contain complex secondary structures, including several stems, loops, and multi-way junctions. These variable structures suggest that the library contains many sequences that can cooperate with the embedded DNAzymes in a way to sense the ligand and induce the cleavage.

### Binding specificity of the aptazymes

**Figure 3:**
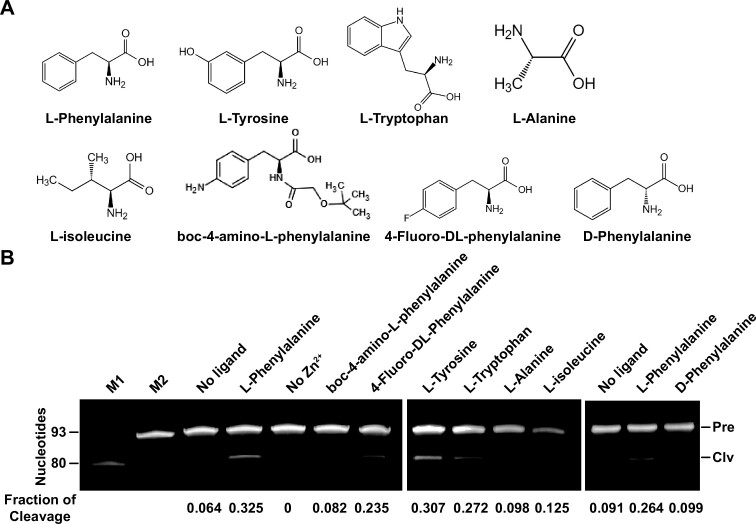
Cleavage activity of the II-R1-3 DNA aptazyme with different ligands. (**A**) Analogs of l-phenylalanine. (**B**) Induction of DNA cleavage of II-R1-3 by these analogs. The concentration of the tested ligands was 100 μM, and the incubation time was 30 min. The fraction of DNA cleavage was the 3′ cleavage product (band intensity) divided by the sum of the remaining full-length DNA and the 3′ cleavage product. Pre and Clv represent the precursor and the 3′ end cleavage product of the aptazyme, respectively. M1 and M2 are ssDNA markers of 93 and 80 nt, respectively. Note that the three images are from three gels with three sets of ligands.

To investigate the binding specificity, several l-phenylalanine analogs, including closely related analogs (such as l-tyrosine, l-tryptophan, 4-fluoro-dl-phenylalanine, and boc-4-amino-l-phenylalanine, d-phenylalanine) and less closely related analogs (such as alanine and l-isoleucine) were used for comparison (Figure 3A). The analogs containing a benzyl group appeared to be able to induce the highest percentage of cleavage for the aptazyme II-R1-3. For example, l-phenylalanine, l-tyrosine, and 4-fluoro-dl-phenylalanine induced a fraction of cleavage of 0.325, 0.307 and 0.235, respectively, whereas alanine and l-isoleucine only induced a smaller fraction of cleavage of 0.098 and 0.125, respectively (Figure 3B and Supplementary Figure S3B). The test of different compounds on the induction of the aptazymes II-R1-7 (Supplementary Figure S3C and S4) and II-R1-1 (Supplementary Figure S3A and S5) produced similar results, except that the II-R1-1 had a higher fraction of cleavage for isoleucine (Supplementary Figure S5). These results indicate that the benzyl group in these compounds is important for recognition by all three aptazymes. However, in an additional experiment, when d-phenylalanine was incubated with the aptazyme II-R1-3, no cleavage was observed (Figure 3B), suggesting that the aptazyme can distinguish the difference between the d and l enantiomers of phenylalanine.

### Binding affinity (*K*_D_) of the aptazymes

**Figure 4:**
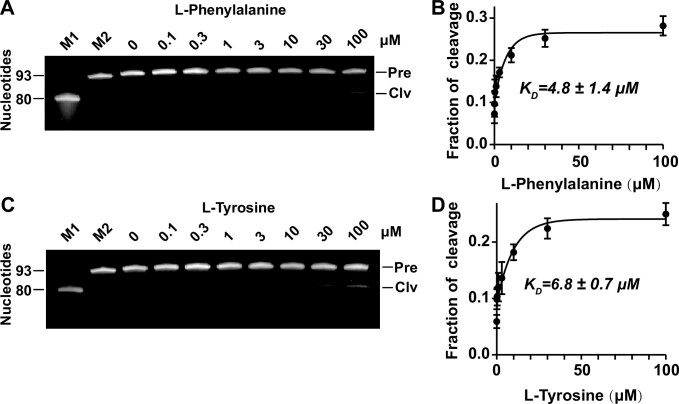
Binding affinity of II-R1-3 for l-phenylalanine and l-tyrosine. (**A**) PAGE gel analysis of II-R1-3 self-cleavage with l-phenylalanine concentrations ranging from 0.1 to 100 μM. (**B**) Dissociation constants (*K*_D_) of II-R1-3 for l-phenylalanine. (**C**) PAGE gel analysis of II-R1-3 self-cleavage with different concentrations of L-tyrosine. (**D**) *K*_D_ of II-R1-3 for l-tyrosine. The *K*_D_ values are the mean of three independent experiments with standard deviation. Other notes are the same as those listed in the Figure 1.

To investigate the binding affinity of II-R1-3, we incubated the aptazyme with l-phenylalanine and l-tyrosine with concentrations ranging from 0.1 to 100 μM. The apparent dissociation constant (*K*_D_), measured using a previously described method (31), was the concentration of the ligand that was required to induce 50% of the maximum cleavage. The *K*_D_ value for II-R1-3 was 4.8 ± 1.4 μM (mean ± standard deviation) for l-phenylalanine and 6.8 ± 0.7 μM for l-tyrosine (Figure 4). Similar tests were conducted for II-R1-7 (Supplementary Figure S6) and II-R1-1 (Supplementary Figure S7). The *K*_D_ values for II-R1-7 were 19.3 ± 2.4 and 118.9 ± 11.7 μM for l-phenylalanine and L-tyrosine, respectively (Supplementary Figure S6) and those for II-R1-1 were 20.3 ± 1.6 and 71.6 ± 9.8 μM, respectively (Supplementary Figure S7). These results indicate that II-R1-7 and II-R1-1 exhibit a higher binding affinity for l-phenylalanine than for l-tyrosine, while II-R1-3 shows a similar binding affinity for these two ligands.

### Cleavage rate constant of the featured aptazymes

**Figure 5:**
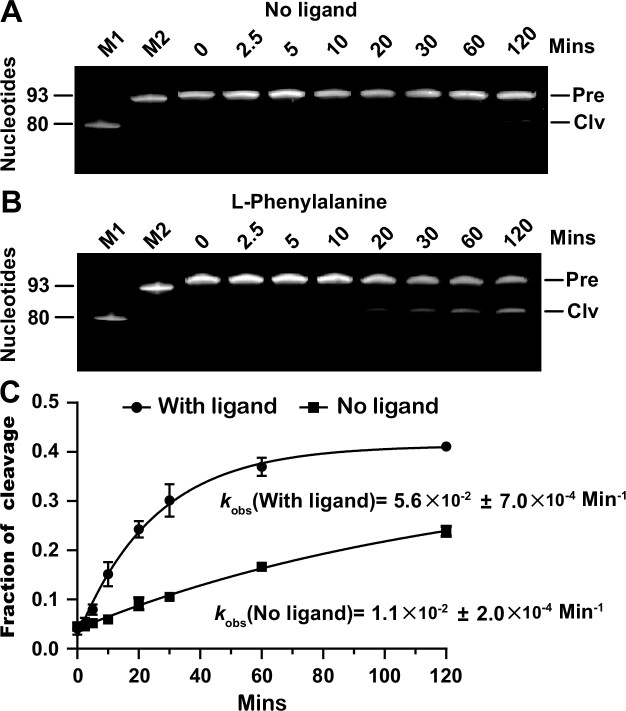
Induction of II-R1-3 self-cleavage by L-phenylalanine. The aptazyme was incubated in the cleavage buffer for different time points in the presence or absence of L-phenylalanine. M1 and M2 are ssDNA markers of 93 nt and 80 nt, respectively (Supplementary file No. 1 Supplementary Table S1). PAGE gel-based analysis of self-cleavage of II-R1-3 in the absence of l-phenylalanine (**A**) and in the presence of 100 μM l-phenylalanine (**B**) as well as the *k*_obs_ values under these conditions (**C**). The *k*_obs_ values are the mean of three independent experiments with standard deviation (SD). Please note that the graph was made by GraphPad Prism. If the error bar would be shorter than the size of the symbol, Prism would not draw it.

To investigate catalytic properties of II-R1-1, II-R1-3 and II-R1-7, we determined their cleavage rate constant (the observed rate constant or *kobs*). We incubated each aptazyme with or without a ligand (100 μM l-phenylalanine) and measured the fraction cleavage at different time points (Figure 5A and B). The *kobs* values were then estimated by GraphPad Prism. The results show that the ligand significantly improved the cleavage rate. The amplitude of cleavage in the presence of the ligand is about 0.4 for II-R1-3 (Figure 5C), 0.35 for II-R1-7 (Supplementary Figure S8C) and 0.22 for II-R1-1 (Supplementary Figure S7B). The *kobs* was found to be 1.1 × 10^−2^ ± 2.0 × 10^−4^ min^−1^ for II-R1-3 without any ligand but changed to 5.6 × 10^−2^ ± 7.0 × 10^−4^ min^−1^ in the presence of L-phenylalanine (Figure 5C), resulting in a 5-fold improvement. The *kobs* values for II-R1-7 were improved by 17-fold upon ligand induction (from 2.0 × 10^−3^ ± 1.0 × 10^−3^ min^−1^ to 3.4 × 10^−2^ ± 3.0 × 10^−3^ min^−1^; Supplementary Figure S8). In contrast, the *kobs* values for II-R1-1 were improved by >20 000-fold (from 1.6 × 10^−6^ ± 6.2 × 10^−8^ min^−1^ to 3.6 × 10^−2^ ± 5.0 × 10^−3^ min^−1^ upon induction with L-phenylalanine (Supplementary Figure S9). These results show that the self-cleavage of all three aptazymes can be significantly induced by l-phenylalanine and II-R1-7 exhibits dramatic induction by l-phenylalanine.

## DISCUSSION

### The allosteric DNAzymes obtained by expression-SELEX enable the direct detection of ligand by the cleavage assay

Through the Expression-SELEX cycles, we obtained several aptazymes that can be induced by l-phenylalanine. These allosteric DNAzymes uses ligand binding to trigger their self-cleavage, and the level of self-cleavage correlates with the concentration of the ligand. They can be designed into various sensors as the cleavage reaction itself can function as a reporter. However, common aptamers need to attach to both a fluorescent group and a quencher to produce a fluorescent signal (36), or to conjugate with a G-quadruplex/hemin DNAzyme to generate a chemiluminescent signal (37) or a color (38), or to connect to a set of electrochemical equipment to yield a electric signal (39) before they can be applied to measure the ligand concentration.

### Improving expression-SELEX to obtain high-quality allosteric DNAzymes for phenylalanine recognition

Previously, we implemented a highly active DNAzyme I-R1 in the random library in the SELEX cycle to select an aptamer with an expression platform, namely, Expression-SELEX (31). The isolated allosteric DNAzyme (aptazyme) IR3-I-DNA could bind L-allo-isoleucine with a low dissociation constant (*K*_D_) of 0.57 mM (31). In this study, we made a few improvement steps to obtain aptazymes with higher quality. First, we chose the II-R1 DNAzyme as an expression platform because of its slow cleavage rate, probably due to its structure deficiency. If the binding of ligands enables the aptazyme to reorganize and stabilize the structure, the cleavage rate will be improved and a ligand-responsive allosteric aptazyme can be obtained. Second, we used lambda exonuclease to digest the PCR product to obtain highly pure full-length ssDNA during the selection cycle (Supplementary Figure S1). Third, we extended the incubation time for negative selection up to 12 h instead of 2 h to eliminate those ssDNA molecules that can self-cleave in the absence of a ligand. Fourth, we chose l-phenylalanine as the ligand. l-phenylalanine contains a phenyl group that can easily be recognized and bound by the aptamer in riboswitches (40,41). Finally, we reduced the ligand incubation time and lowered the ligand concentration during the selection cycles. Through these improvements, we obtained many aptazymes that can self-cleave upon ligand binding. We arbitrarily picked three candidates among the top 10 enriched candidates for characterization.

The *K*_D_ values for these aptazymes for l-phenylalanine are as low as 4.8 μM while they are 118.9 μM for the analog l-tyrosine. These results imply that these aptazymes have a high binding affinity for l-phenylalanine and they can elucidate the subtle difference between l-phenylalanine and l-tyrosine. In addition, our experiment showed that l-phenylalanine but not d-phenylalanine could induce significant cleavage of the aptazyme, suggesting that the aptazyme could tell the difference between d and l enantiomers.

### Cleavage of the selected aptazymes by expression-SELEX is highly inducible by the ligand

The *k_obs_* values for the cleavage of these three aptazymes are very low (<0.011 min^−1^), especially for the aptazyme II-R1-1, which has a *k*_obs_ value of around 10^−6^ min^−1^. However, when the ligand l-phenylalanine is present, the values can be improved up to 5.6 × 10^−2^, 3.6 × 10^−2^ and 3.4 × 10^−2^ min^−1^ for these three aptazymes, and one of which were improved by approximately 20 000-fold. These results suggest that the cleavage of these aptazymes could be induced dramatically by the ligand.

### Low no-ligand self-cleavage and high ligand-induced self-cleavage might facilitate the enrichment of these allosteric DNAzymes

The II-R1-1 aptazyme is the most abundant ssDNA candidate in the pool during the selection cycles. In the 6th round, it occupied only 0.92% of the total reads. However, it occupied up to 45.61% in the 14th round and up to 73.85% in the 20th round. The reason for its high enrichment is unknown. We noticed that the self-cleavage of II-R1-1 is the lowest when the ligand is not present. However, the improvement of the cleavage induced by the ligand was the highest among all of these tested aptazymes. Therefore, low self-cleavage and high ligand-induced cleavage might account for its high enrichment.

### Optimized approach can select various ligand-binding aptazymes without the need to immobilize either ligands or aptazymes

The SELEX procedure, invented in 1990, commonly requires the immobilization of either the ligand or the aptamer to facilitate the selection (15,16,28). However, the chemical modifications of both small ligands and aptamers for immobilization are complicated and may interfere with the binding of the aptamer to its ligand. The Expression-SELEX described in this study takes advantage of the self-cleaving DNAzyme and does not require immobilization. When the ligand binds to the aptazyme, the binding induces cleavage, and the cleavage products could be separated and purified by PAGE gel. After PCR amplification using Taq DNA polymerase (ABM company) and ssDNA separation by the exonuclease digestion, the aptazyme candidates can be enriched during each SELEX cycle.

### Application of allosteric DNAzymes to measure phenylalanine for phenylketonuria

The blood of a health individual usually contains 50–110 μM phenylalanine, while that of an individual with a phenylketonuria has a concentration >120 μM. Given the *K*_D_ value for the II-R1-3 allosteric DNAzyme is 4.8 μM, it is well suited for measuring the blood phenylalanine concentration, which constitutes a future research objective.

## CONCLUSIONS

Aptamers are widely applied for biosensors, diagnostic tools, and therapeutic agents. The application of aptamers usually requires an expression platform to generate a signal to report the aptamer-ligand binding event. Traditionally, aptamer selection and expression platform fusion are two separate steps and the aptamer selection requires the immobilization of either the aptamer or the ligand during the selection cycle. These shortcomings can be easily overcome by merging a DNAzyme with the random-sequence and selecting self-cleaving aptazymes under ligand-induction. Herein, we applied the technique of Expression-SELEX developed in our laboratory to select for aptazymes that can be specifically activated by l-phenylalanine. We integrated the II-R1 DNAzyme into the DNA library and used stringent selection conditions and special purification approach to drive the selection of high-quality aptazymes that can self-cleave upon ligand binding. We obtained at least three enriched candidates that exhibit a dissociation constant for l-phenylalanine as low as 4.8 μM, and a catalytic rate constant improvement as high as 20 000-fold, and discriminate against closely related analogs as well as d- and l-phenylalanine enantiomers. Therefore, we have established an efficient and reliable Expression-SELEX to enrich high-quality ligand-responsive aptazymes.

## DATA AVAILABILITY

The data underlying this article are available in the article and in its online supplementary material.

## Supplementary Material

gkad424_Supplemental_Files
