# Supplementary material for: Selection of allosteric dnazymes that can sense phenylalanine by expression-SELEX
Source: Nucleic Acids Res. 2023 May 19;51(11):e66. doi: 10.1093/nar/gkad424 (PMC10287898; doi:10.1093/nar/gkad424)
Supplement: gkad424_Supplemental_Files [file gkad424_supplemental_files.zip › Supplementary file No. 1 Figures-tables-revised-clean.docx]

Selection of allosteric DNAzymes that can sense phenylalanine by Expression-SELEX

Binfen Chen^1^, Xinmei Yu^1^, Ting Gao^1^, Yaoyao Wu^1^, Xiaojun Zhang^2^, Sanshu Li^3, *^

^1^ Medical School, Huaqiao University, Xiamen, 361021, P. R. China

^2^ Chemical Engineering Institute, Huaqiao University, Xiamen, 361021, P. R. China

^3^ Medical School, Engineering Research Center of Molecular Medicine of Ministry of Education, Key Laboratory of Precision Medicine and Molecular Diagnosis of Fujian Universities, Institute of Genomics, Huaqiao University, Xiamen, 361021, P. R. China

* To whom correspondence should be addressed. Email: sanshuli@hqu.edu.cn


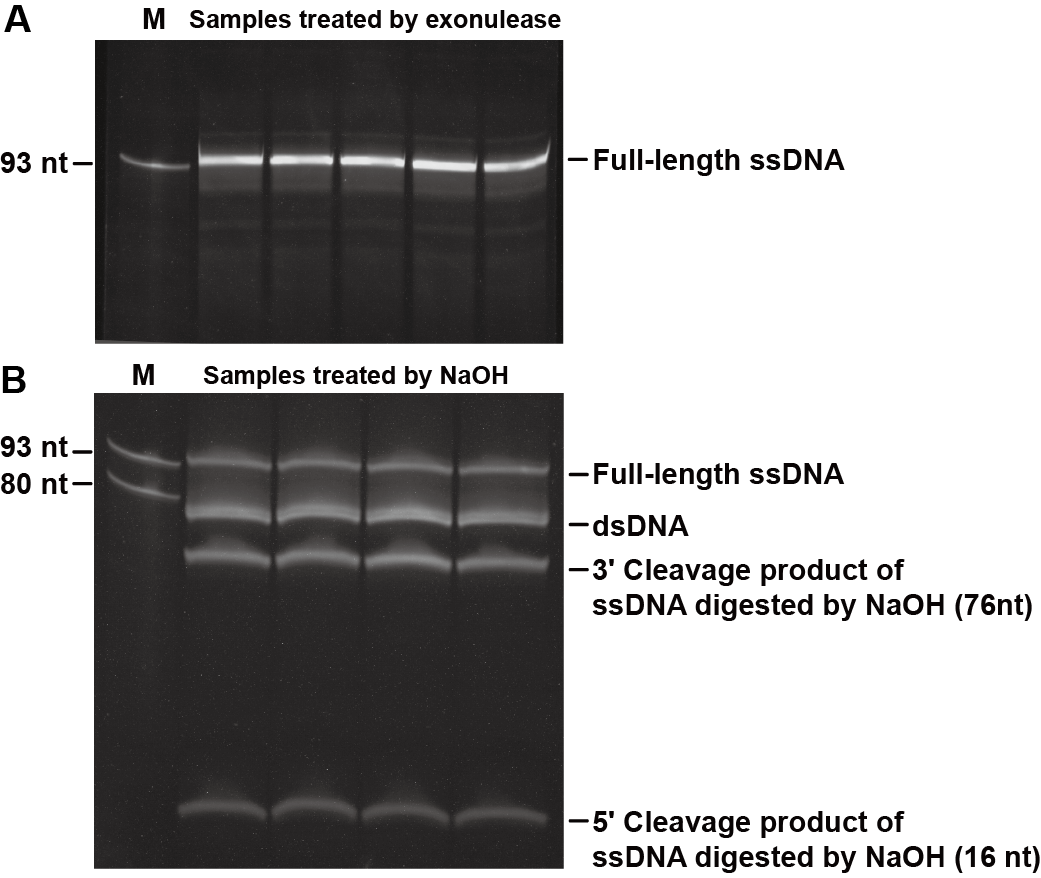


Figure S1 Different treatment methods to obtain single-stranded aptamer DNA during the SELEX cycles. A. Double stranded PCR products were treated by lambda exonuclease to digest the antisense strand with the phosphate group and separated by the PAGE gel. The brightest band is the full-length aptamer (sense strand). B. Double stranded PCR products were treated by NaOH, and separated on the PAGE gel. Several bands were observed, however, only the weak top band is the full-length aptamer (sense strand).


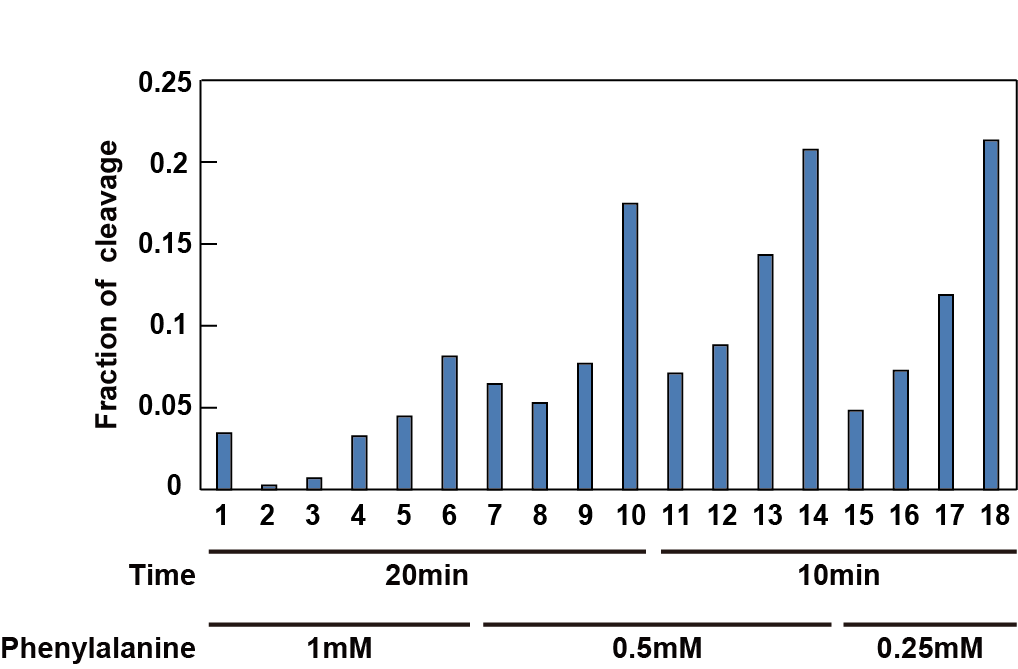


Figure S2 The enrichment of the phenylalanine binding aptamers by the Expression-SELEX. The initial incubation time is 20 minutes and the ligand concentration is 1 mM. The incubation time was reduced to 10 mins and the concentration of L-Phenylalanine was gradually reduced to 0.25 mM during the cycles. The fraction of cleavage of the ssDNA library was measured by incubating the library with ligand for the time indicated. The 19^th^ and 20^th^ cycles were counter-selection cycles using the analog 4-fluoro-DL-Phenylalanine (the enrichment is not shown on the graph) and were not shown in the graph. Candidate aptamers from the 6^th^, 14^th,^ and 20^th^ SELEX cycle were analyzed by high throughput sequencing.


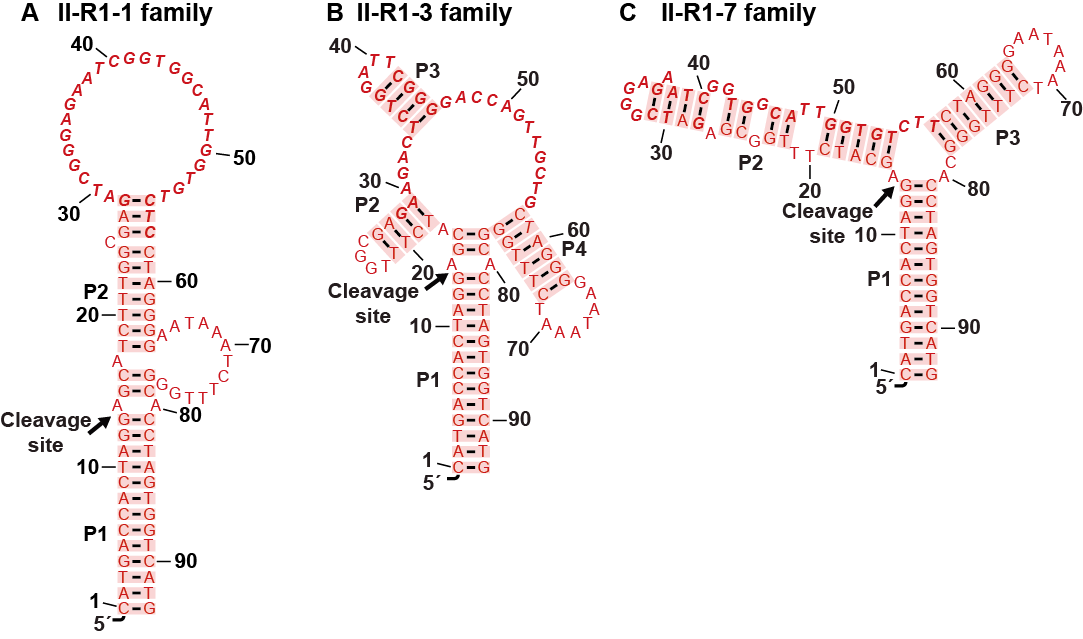


Figure S3 Three enriched aptazyme candidates. (A) II-R1-1; (B) II-R1-3; (C) II-R1-7. 30 nucleotides originated from the random-region of the DNA library are in italic font. Sequences and alignments are in the Supplementary file No. 6-17.


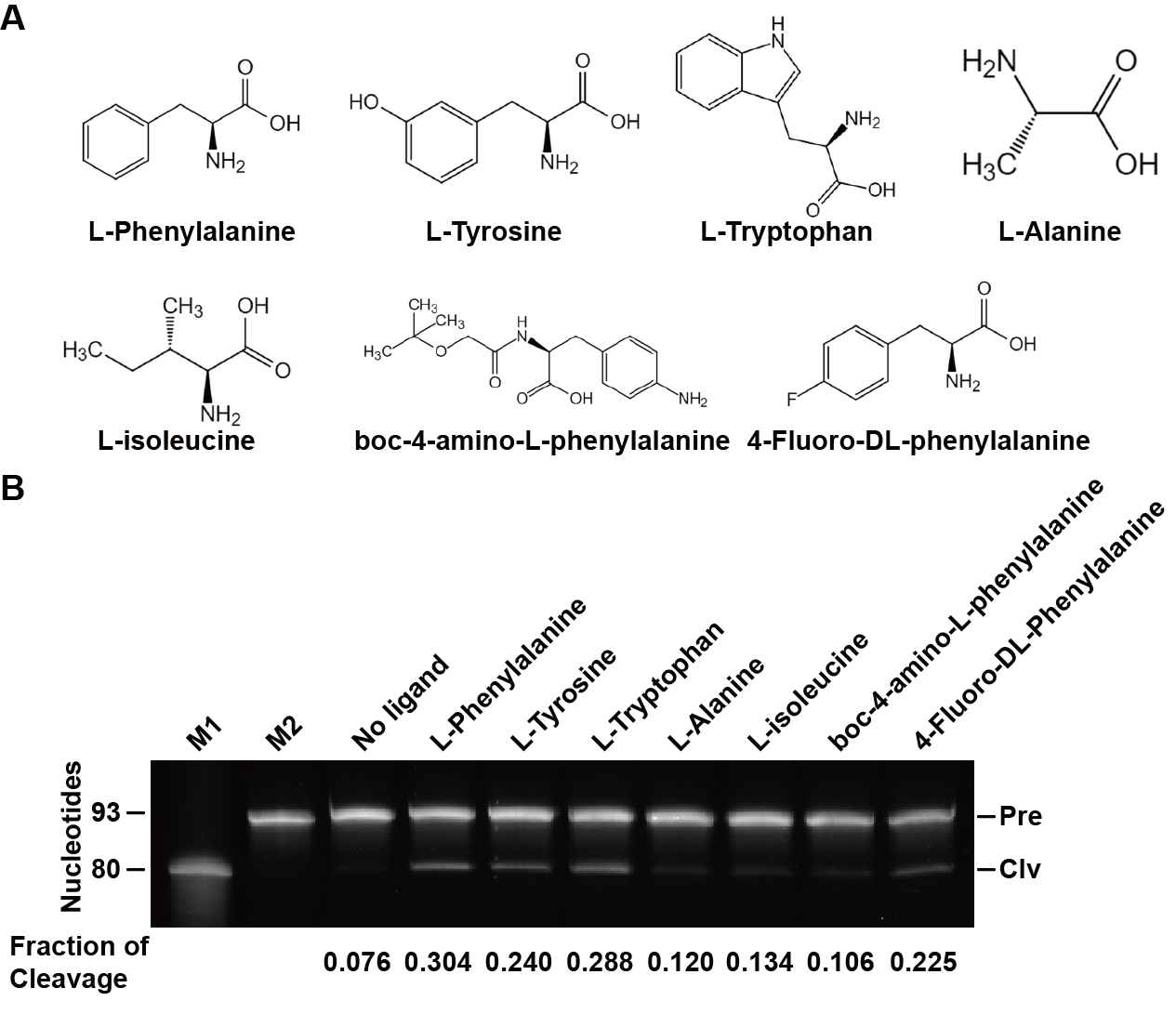


Figure S4 Binding specificity of the II-R1-7 aptamer. A. The structures of analogues of L-Phenylalanine. B. The induction of DNA cleavage by these analogues. The concentration of the analogues was 100μM, and incubation time was 30 mins. The fraction of DNA cleavage was the 3' cleavage product (band intensity) divided by the sum of the remaining full-length DNA and 3' cleavage product. Note that the 5' cleavage product is only 13 nucleotides, its band intensity is too weak. Therefore, its intensity is not included in the calculation.


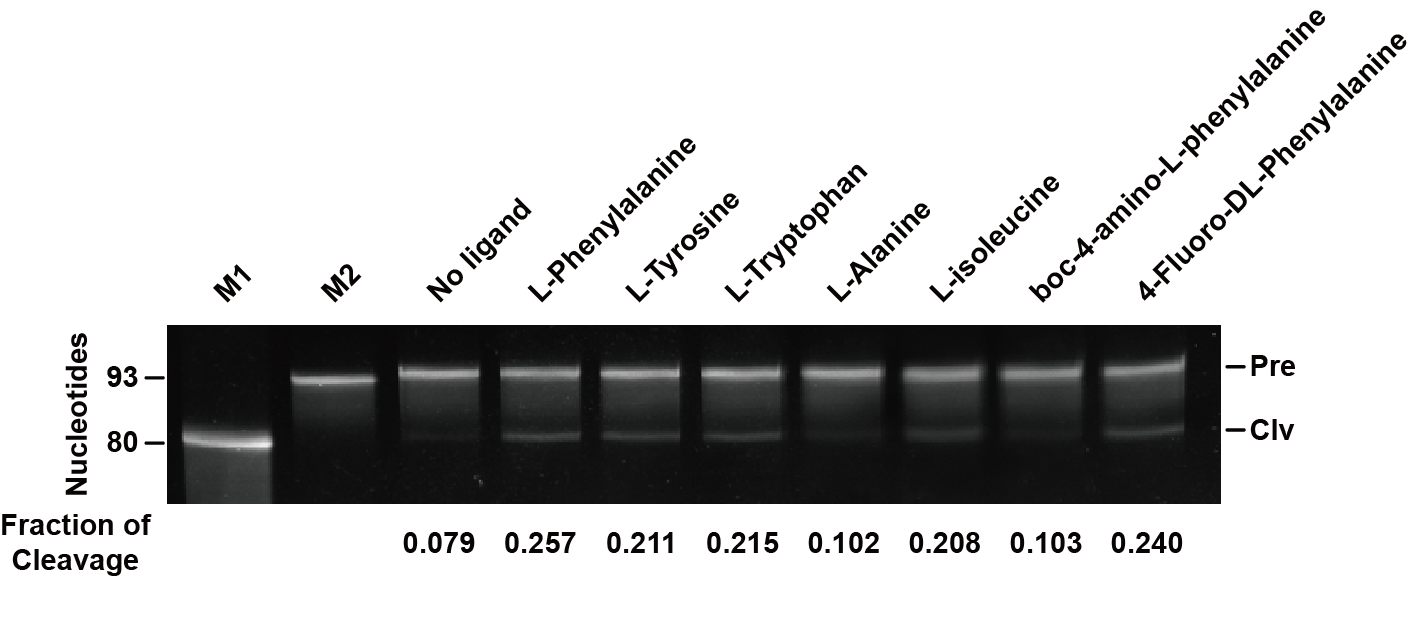


Figure S5 Binding specificity of the II-R1-1 aptamer. The assay method and the figure annotation are as described in the Figure S2.


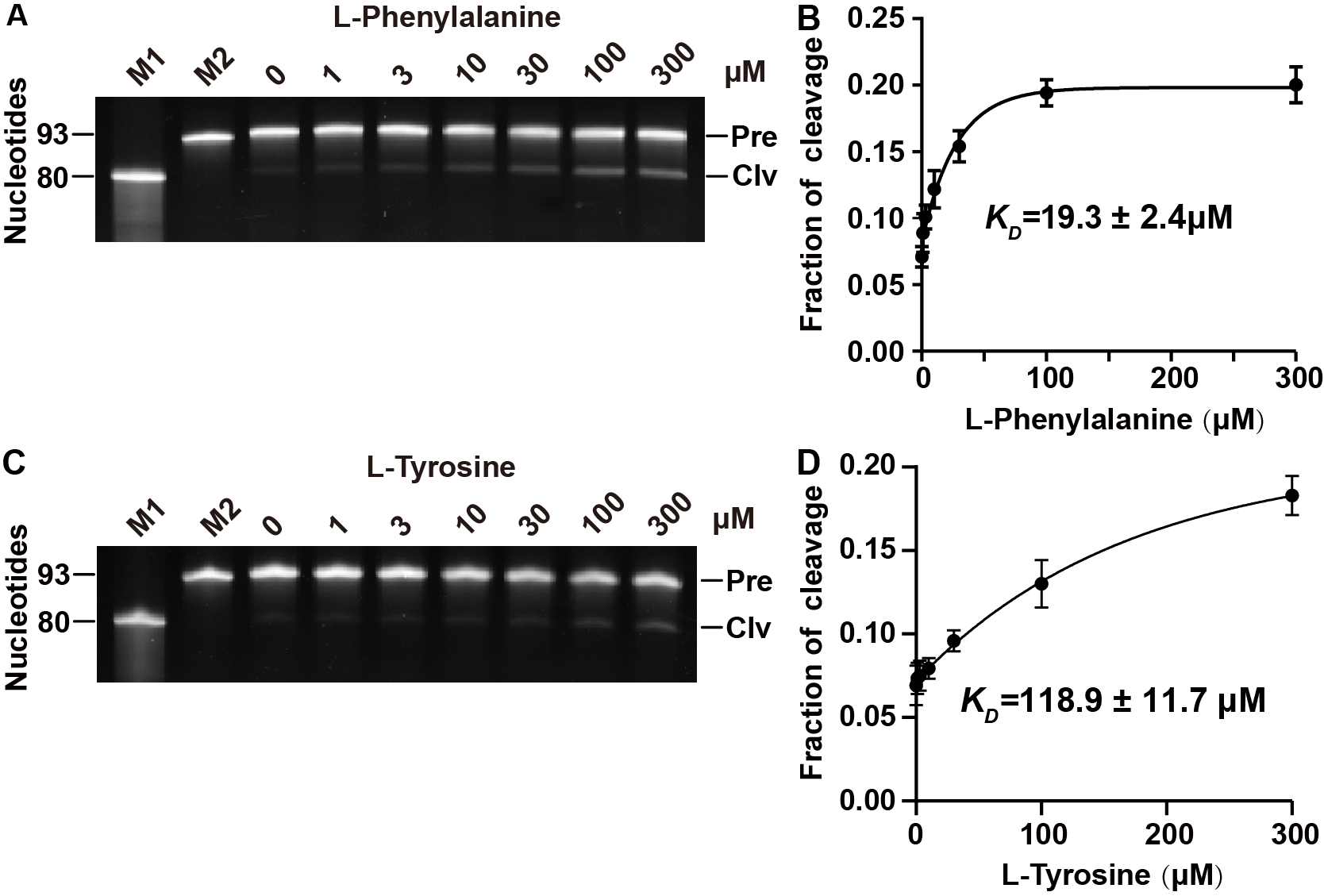


Figure S6 Dissociation constants (*K*_D_) of the II-R1-7 DNA aptamer. A. The aptamer was incubated with L-Phenylalanine. B. *K*_D_ measurement with ligand L-Phenylalanine. C. The aptamer was incubated with L-Tyrosine. D. *K*_D_ measurement with ligand L-Tyrosine. The *K*_D_ values are the mean of three independent experiments with standard deviation (SD).


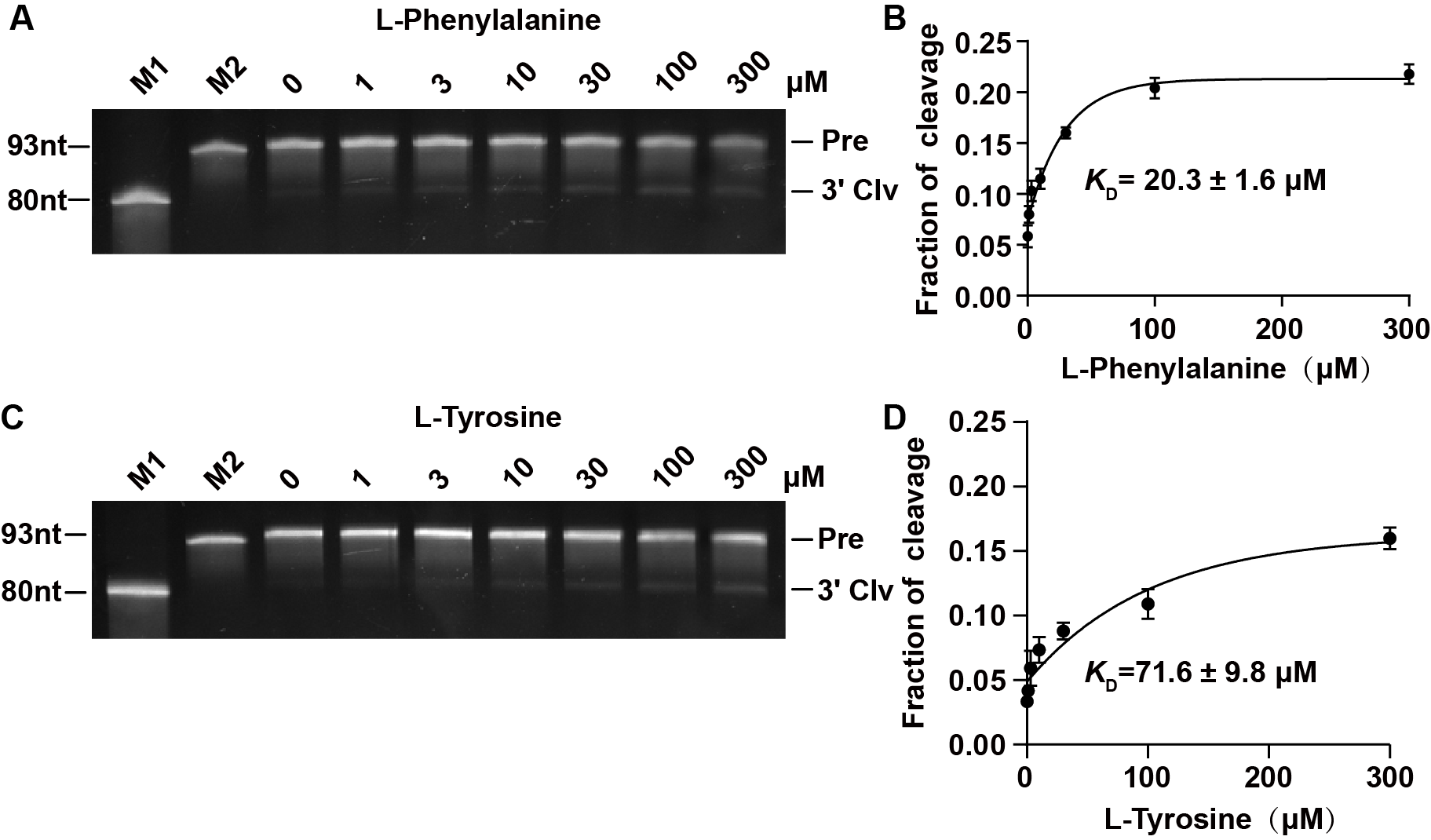


Figure S7 Dissociation constants (KD) of the II-R1-1 DNA aptamer. A. The aptamer was incubated with L-Phenylalanine. B. KD measurement with ligand L-Phenylalanine. C. The aptamer was incubated with L-Tyrosine. D. KD measurement with ligand L-Tyrosine. The KD values are the mean of three independent experiments with standard deviation (SD).


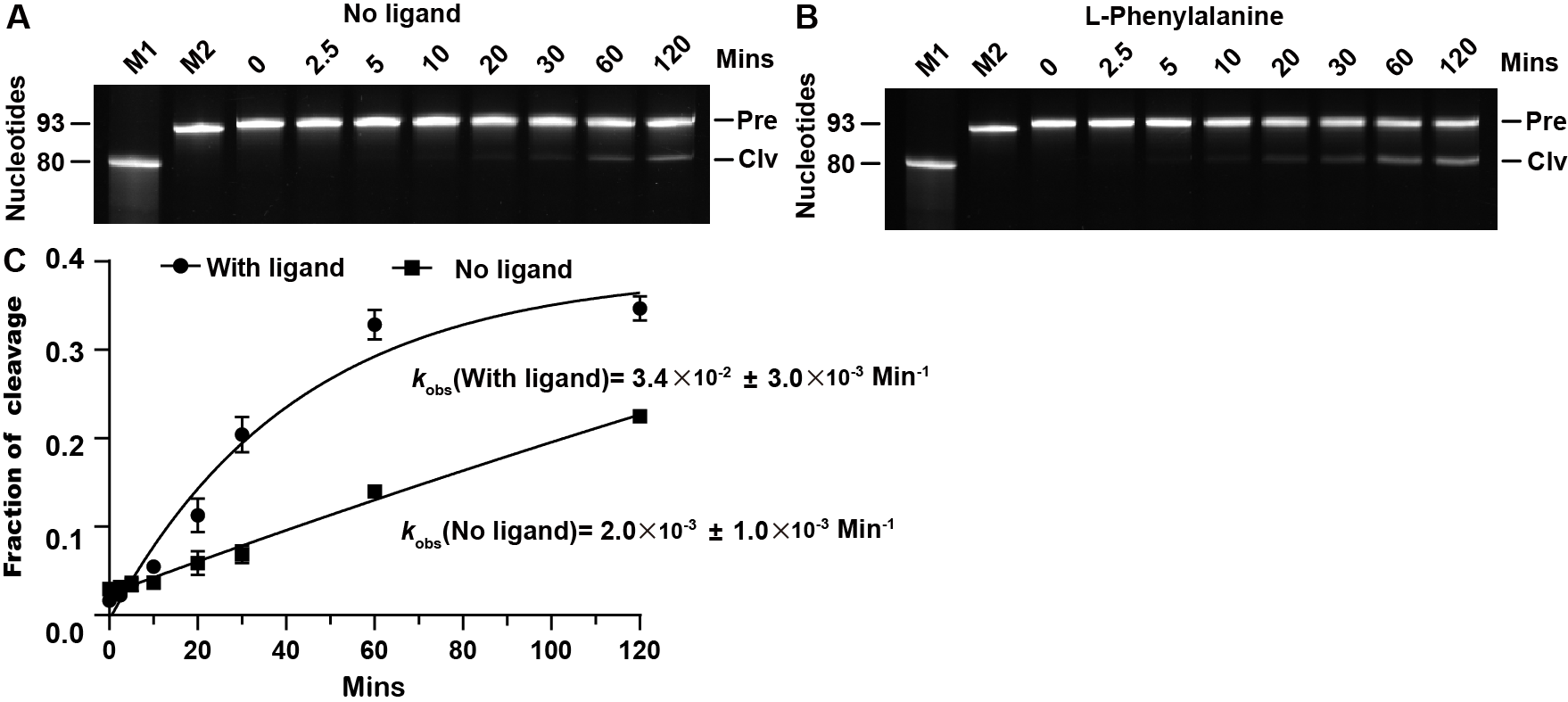


Figure S8 The *k_obs_* of the II-R1-7 DNA aptamer with or without the ligand L-Phenylalanine. II-R1-7 DNA aptamer was incubated in the cleavage buffer for different time points. A. Without a ligand. B. With the ligand L-Phenylalanine (100 μM). C. The values of *k_obs_* were measured by the time-course experiments up to 120 mins. The *k_obs_* values are the mean of three independent experiments with standard deviation (SD). Please note that the graph was made by GraphPad Prism. If the error bar would be shorter than the size of the symbol, Prism would not draw it.


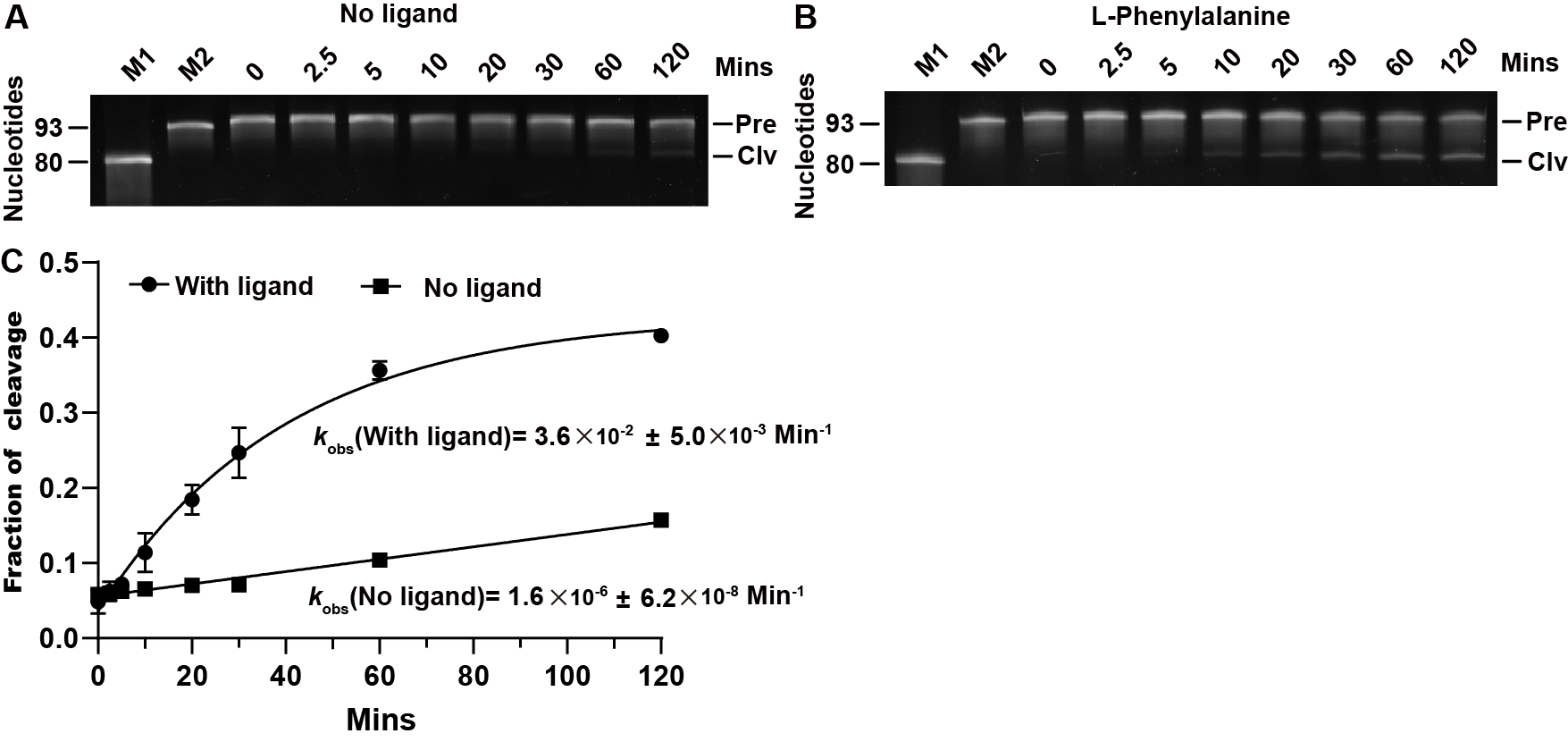


Figure S9 The kobs of II-R1-1 DNA aptamer with or without the ligand L-Phenylalanine. II-R1-1 DNA aptamer was incubated in the cleavage buffer for different time points. A. Without a ligand. B. With the ligand L-Phenylalanine (100 μM). C. The values of kobs were measured by the time-course experiments up to 120 mins. The kobs values are the mean of three independent experiments with standard deviation (SD). Please note that the graph was made by GraphPad Prism. If the error bar would be shorter than the size of the symbol, Prism would not draw it.

Table S1 Primers and oligos

| Name | sequences | notes |
| --- | --- | --- |
| II-random-library | 5'-CATGACCACTAGGAGCATCTTTGGCGA-N30-CTAGGGGAATAAATCTTTGGGCACCTAGTGGTCATG | 93nt (Random sequence library) |
| Marker M1 | 5'-GGAATCGTAGGTTATATCGGTCAGCTTGATGCGAAGGAAATTTTACTGAAAGGGTTAGAAAAGCTTGAGTACCGCGGCTATGATTCTGCTGGT | 93nt (from the ORF of the GlmS gene) |
| Marker M2 | 5'-GGAATCGTAGGTTATATCGGTCAGCTTGATGCGAAGGAAATTTTACTGAAAGGGTTAGAAAAGCTTGAGTACCGCGGCTA | 80 nt (from the ORF of the GlmS gene) |
| II-forward | 5'-CATGACCACTAGGAGCATCTTTGGCGA | Use II-forward and II-reverse to amplify the aptazyme |
| II-reverse | 5'-CATGACCACTAGGTGCC | Use II-forward and II-reverse to amplify the aptazyme |
| II-reverse-R | 5'-CATGACCACTAGGTGCC_r_ | The last nucleotide is a ribonucleic acid. |
| II-reverse-P | 5'-PO3-CATGACCACTAGGTGCC | Use II-forward and II-reverse-P to amplify the aptazyme with a phosphate group |
| P5-forward | 5'-AATGATACGGCGACCACCGAGATCTACACTCTTTCCCTACACGACGCTCTTCCGATCTCATGACCACTAGGAGCATCTTTGGCG | Use P5-forward and P7-Reverse primers to construct sequencing library |
| P7-reverse | 5'-CAAGCAGAAGACGGCATACGAGATGCATATGTGACTGGAGTTCAGACGTGTGCTCTTCCGATCCATGACCACTAGGTGCCCAAAG | To construct the sequencing library(the letters in red are index sequences) |
| II-R1-3-DNA | 5'-CATGACCACTAGGAGCATCTTTGGCGAGAAGACTCTGGATTCGGGGACCAGTTGCTGCTAGGGGAATAAATCTTTGGGCACCTAGTGGTCATG | 93 nt |
| II-R1-7-DNA | 5'-CATGACCACTAGGAGCATCTTTGGCGATCCTCGTCAGATGGTGAAGCAGACGTTTGGCTAGGGGAATAAATCTTTGGGCACCTAGTGGTCATG | 93 nt |
| II-R1-1-DNA | 5'-CATGACCACTAGGAGCATCTTTGGCGAGATCGGGAGAATCGGTGGCATTGGTGTCTCCTAGGGGAATAAATCTTTGGGCACCTAGTGGTCATG | 93 nt |

Table S2 Top enriched-sequences in the library during different cycles of selection

| ROUND 6 | ROUND 14 | ROUND 20 |
| --- | --- | --- |
| Top-1 | Top-1 | Top-1 |
| Top-2 | Top-2 | Top-2 |
| Top-3 | Top-3 | Top-3 |
| Top-4 | Top-4 | Top-4 |
| Top-5 | Top-6 | Top-10 |
| Top-6 | Top-5 | Top-9 |
| Top-7 | Top-15 | Top-125 |
| Top-8 | Top-7 | Top-74 |
| Top-9 | Top-14 | Top-5 |
| Top-10 | Top-9 | Top-28 |

Note: The top10 enriched sequences in the sixth round are also found in the round 14th and round 20th. The number of total reads of each library and the reads for each sequence are listed in the Supplementary file No. 2-4 for round_6, round_14 and round_20.
