# Supplementary material for: Selection of allosteric dnazymes that can sense phenylalanine by expression-SELEX
Source: Nucleic Acids Res. 2023 May 19;51(11):e66. doi: 10.1093/nar/gkad424 (PMC10287898; doi:10.1093/nar/gkad424)
Supplement: gkad424_Supplemental_Files [file gkad424_supplemental_files.zip › Supplementary file No. 2 count_most_represented_seqs_for_round-6-top1000.docx]

Note: total reads of the library are 1575421. The reads are on the left, and the sequence is on the right. Top 1000 sequences are shown here only, since the original file is too large (135M bits).

14448 CATGACCACTAGGAGCATCTTTGGCGAGATCGGGAGAATCGGTGGCATTGGTGTCTCCTAGGGGAATAAATCTTTGGGCACCTAGTGGTCATG

2277 CATGACCACTAGGAGCATCTTTGGCGAACGGCAGGTGTTGCGGTGGTCTGTGAATCCCTAGGGGAATAAATCTTTGGGCACCTAGTGGTCATG

1130 CATGACCACTAGGAGCATCTTTGGCGAGAAGACTCTGGATTCGGGGACCAGTTGCTGCTAGGGGAATAAATCTTTGGGCACCTAGTGGTCATG

409 CATGACCACTAGGAGCATCTTTGGCGAAAGTCGGTAGAACAGGGTGGGGTGCTGTCCCTAGGGGAATAAATCTTTGGGCACCTAGTGGTCATG

271 CATGACCACTAGGAGCATCTTTGGCGAGCCTCGCTTGGGAGGTTGCTCCACCAGTTCCTAGGGGAATAAATCTTTGGGCACCTAGTGGTCATG

248 CATGACCACTAGGAGCATCTTTGGCGAATGGCCGGCACGGCCTTCTAGTCCTCGGTACTAGGGGAATAAATCTTTGGGCACCTAGTGGTCATG

207 CATGACCACTAGGAGCATCTTTGGCGATCAGGCCGCCAGGGCGTACGAAGTGTGGTTCTAGGGGAATAAATCTTTGGGCACCTAGTGGTCATG

126 CATGACCACTAGGAGCATCTTTGGCGATCCTCGTCAGATGGTGAAGCAGACGTTTGGCTAGGGGAATAAATCTTTGGGCACCTAGTGGTCATG

120 CATGACCACTAGGAGCATCTTTGGCGAGATCGGGAGAATCGGCGGCATTGGTGTCTCCTAGGGGAATAAATCTTTGGGCACCTAGTGGTCATG

111 CATGACCACTAGGAGCATCTTTGGCGATGGGTGCTGACGGCCGCCGCTGCGGCTACACTAGGGGAATAAATCTTTGGGCACCTAGTGGTCATG

90 CATGACCACTAGGAGCATCTTTGGCGAAGCGTGTTGCAAGGTCCGCGAGTGTGGACCCTAGGGGAATAAATCTTTGGGCACCTAGTGGTCATG

85 CATGACCACTAGGAGCATCTTTGGCGATGGGTGCTGACGGGGCTAGTGAGCCCTACACTAGGGGAATAAATCTTTGGGCACCTAGTGGTCATG

84 CATGACCACTAGGAGCATCTTTGGCGACGGTGTGGGGAACTTGTTTCGGCGGTGCTACTAGGGGAATAAATCTTTGGGCACCTAGTGGTCATG

67 CATGACCACTAGGAGCATCTTTGGCGACGGCGTGGGGCCATCTGTGCGGCGGATCCCCTAGGGGAATAAATCTTTGGGCACCTAGTGGTCATG

63 CATGACCACTAGGAGCATCTTTGGCGAGGGAGGGCGCCGGCAGCGGTGTGAATGCGCCTAGGGGAATAAATCTTTGGGCACCTAGTGGTCATG

55 CATGACCACTAGGAGCATCTTTGGCGATGGGGCCGTGCTATCTGCACACTCCGCGGGCTAGGGGAATAAATCTTTGGGCACCTAGTGGTCATG

54 CATGACCACTAGGAGCATCTTTGGCGACGGCGTGGGGCTATTGATGCGGCGGCACTCCTAGGGGAATAAATCTTTGGGCACCTAGTGGTCATG

49 CATGACCACTAGGAGCATCTTTGGCGAAGGGTGTAGGACTTCAAGTGGATCTCATAGCTAGGGGAATAAATCTTTGGGCACCTAGTGGTCATG

47 CATGACCACTAGGAGCATCTTTGGCGACGTAGTGGGCTGGCAGGGAGTACTTGTTGCCTAGGGGAATAAATCTTTGGGCACCTAGTGGTCATG

45 CATGACCACTAGGAGCATCTTTGGCGAAGCAGTGCGACGGGCTATGGGTTCCTCGAGCTAGGGGAATAAATCTTTGGGCACCTAGTGGTCATG

43 CATGACCACTAGGAGCATCTTTGGCGAGGCGAGTCCAGCGGCCTTCTTGGCCTGCGACTAGGGGAATAAATCTTTGGGCACCTAGTGGTCATG

42 CATGACCACTAGGAGCATCTTTGGCGATAGGCGTGTACGGTCGAGCGAAAGCGATAGCTAGGGGAATAAATCTTTGGGCACCTAGTGGTCATG

38 CATGACCACTAGGAGCATCTTTGGCGACGAGGGGCACGGGTGGTCATTGCGAGAAGACTAGGGGAATAAATCTTTGGGCACCTAGTGGTCATG

37 CATGACCACTAGGAGCATCTTTGGCGAGGGGTCATGGGAATGTAATTGCGCTTAAGACTAGGGGAATAAATCTTTGGGCACCTAGTGGTCATG

37 CATGACCACTAGGAGCATCTTTGGCGACCGTAGGAAAACAAAAGTGGCCTTGCTAGCCTAGGGGAATAAATCTTTGGGCACCTAGTGGTCATG

34 CATGACCACTAGGAGCATCTTTGGCGAGATCGGGAGAATCGGTGGCATCGGTGTCTCCTAGGGGAATAAATCTTTGGGCACCTAGTGGTCATG

31 CATGACCACTAGGAGCATCTTTGGCGAGATCGTGAGAATCGGTGGCATTGGTGTCTCCTAGGGGAATAAATCTTTGGGCACCTAGTGGTCATG

30 CATGACCACTAGGAGCATCTTTGGCGAGGGAGTGGTCGGCCATGTGGGGGGTCGGGGCTAGGGGAATAAATCTTTGGGCACCTAGTGGTCATG

29 CATGACCACTAGGAGCATCTTTGGCGAAAGCGCAGGGCATGCGACCTCCAATTGGTCCTAGGGGAATAAATCTTTGGGCACCTAGTGGTCATG

29 CATGACCACTAGGAGCATCTTTGGCGAGAAGGGGATGGTTGGAGCAGCTCTGGGGGTCTAGGGGAATAAATCTTTGGGCACCTAGTGGTCATG

29 CATGACCACTAGGAGCATCTTTGGCGACTGTGGTCTCGTCCTAATTGTGCGGGCGTCCTAGGGGAATAAATCTTTGGGCACCTAGTGGTCATG

29 CATGACCACTAGGAGCATCTTTGGCGACAGGGGATGCCGATGCGCTGGACGACCGAGCTAGGGGAATAAATCTTTGGGCACCTAGTGGTCATG

28 CATGACCACTAGGAGCATCTTTGGCGAACGTGCAAGATATGCCGCCTGGAGCGTAGGCTAGGGGAATAAATCTTTGGGCACCTAGTGGTCATG

27 CATGACCACTAGGAGCATCTTTGGCGACGGTGTGGGGACCGCAGGTTCGGCGGGTACCTAGGGGAATAAATCTTTGGGCACCTAGTGGTCATG

27 CATGACCACTAGGAGCATCTTTGGCGACAGCGGCGTAGGCTGCCTTGGGTGGTGGCCCTAGGGGAATAAATCTTTGGGCACCTAGTGGTCATG

27 CATGACCACTAGGAGCATCTTTGGCGAGCCGATTGTGCAACAATGTGTTCTGGGTAACTAGGGGAATAAATCTTTGGGCACCTAGTGGTCATG

26 CATGACCACTAGGAGCATCTTTGGCGAAGTACAACGGCAGGCATTGTGTCTCGGTAGCTAGGGGAATAAATCTTTGGGCACCTAGTGGTCATG

26 CATGACCACTAGGAGCATCTTTGGCGAGTATTGGCTGGTAGGTTGCGTATTGGGGAGCTAGGGGAATAAATCTTTGGGCACCTAGTGGTCATG

26 CATGACCACTAGGAGCATCTTTGGCGACGGTGTGGGTGCAGGGCCAGGCGGTTGATCCTAGGGGAATAAATCTTTGGGCACCTAGTGGTCATG

25 CATGACCACTAGGAGCATCTTTGGCGAACCTGTACGGTGATGAGGTCGGTATCAGCCCTAGGGGAATAAATCTTTGGGCACCTAGTGGTCATG

25 CATGACCACTAGGAGCATCTTTGGCGAGATCGGGGGAATCGGTGGCATTGGTGTCTCCTAGGGGAATAAATCTTTGGGCACCTAGTGGTCATG

25 CATGACCACTAGGAGCATCTTTGGCGATCAGCATAGCATATGCGCCTGGAGCGTAGGCTAGGGGAATAAATCTTTGGGCACCTAGTGGTCATG

25 CATGACCACTAGGAGCATCTTTGGCGACGACTTCGGAGTGGGTGCGCCGCTGGCATACTAGGGGAATAAATCTTTGGGCACCTAGTGGTCATG

24 CATGACCACTAGGAGCATCTTTGGCGAACGGCAGGTGTTGCGGTGGTTTGTGAATCCCTAGGGGAATAAATCTTTGGGCACCTAGTGGTCATG

23 CATGACCACTAGGAGCATCTTTGGCGAGATCAGGAGAATCGGTGGCATTGGTGTCTCCTAGGGGAATAAATCTTTGGGCACCTAGTGGTCATG

22 CATGACCACTAGGAGCATCTTTGGCGATGGGTGCAGACGAGTGCGCTTGCAATTACACTAGGGGAATAAATCTTTGGGCACCTAGTGGTCATG

22 CATGACCACTAGGAGCATCTTTGGCGAGGTTGTAGCTCGCGCGTAGGGTCGGCAGTGCTAGGGGAATAAATCTTTGGGCACCTAGTGGTCATG

21 CATGACCACTAGGAGCATCTTTGGCGACTCGTGGTCCTTACCCCCGTAGAGGGTTGTCTAGGGGAATAAATCTTTGGGCACCTAGTGGTCATG

21 CATGACCACTAGGAGCATCTTTGGCGAGAGCTTGAAAAAGGTGCGTTGTTGTCGCATCTAGGGGAATAAATCTTTGGGCACCTAGTGGTCATG

20 CATGACCACTAGGAGCATCTTTGGCGAGATCGGGAGAATCGGTGGCATTGGTGTCCCCTAGGGGAATAAATCTTTGGGCACCTAGTGGTCATG

19 CATGACCACTAGGAGCATCTTTGGCGAGATCGAGAGAATCGGTGGCATTGGTGTCTCCTAGGGGAATAAATCTTTGGGCACCTAGTGGTCATG

19 CATGACCACTAGGAGCATCTTTGGCGAGTTGTCGTAGAGCCACGGGACACCCGTCAGCTAGGGGAATAAATCTTTGGGCACCTAGTGGTCATG

19 CATGACCACTAGGAGCATCTTTGGCGAGTTTCGCTGTGCTTGGAATGGGGCCACCCTCTAGGGGAATAAATCTTTGGGCACCTAGTGGTCATG

19 CATGACCACTAGGAGCATCTTTGGCGAACAGGCCGTGGCTGCGGGTGGGTGCGCACCCTAGGGGAATAAATCTTTGGGCACCTAGTGGTCATG

18 CATGACCACTAGGAGCATCTTTGGCGAGTGTTGCCCGGTACACGAGCAGACGTTTGGCTAGGGGAATAAATCTTTGGGCACCTAGTGGTCATG

18 CATGACCACTAGGAGCATCTTTGGCGAGATCGGGAGAATCGGTGGCATTGGTGCCTCCTAGGGGAATAAATCTTTGGGCACCTAGTGGTCATG

18 CATGACCACTAGGAGCATCTTTGGCGACGAGATTTGGTAGGTAGATGTGCTCTAGCACTAGGGGAATAAATCTTTGGGCACCTAGTGGTCATG

18 CATGACCACTAGGAGCATCTTTGGCGATGACGTCGTCGTAAATAGAGGCCTCGCGACCTAGGGGAATAAATCTTTGGGCACCTAGTGGTCATG

17 CATGACCACTAGGAGCATCTTTGGCGAGGAGTGGCTGAGGGCGGTGGGTAGGTCGCGCTAGGGGAATAAATCTTTGGGCACCTAGTGGTCATG

17 CATGACCACTAGGAGCATCTTTGGCGAGATCGGGAGAGTCGGTGGCATTGGTGTCTCCTAGGGGAATAAATCTTTGGGCACCTAGTGGTCATG

17 CATGACCACTAGGAGCATCTTTGGCGATAGCGAAGTGCTGTGAACAGTGGTTAGCACCTAGGGGAATAAATCTTTGGGCACCTAGTGGTCATG

16 CATGACCACTAGGAGCATCTTTGGCGAACTCATTGGAGTGGGTGTAACGAATCCGTACTAGGGGAATAAATCTTTGGGCACCTAGTGGTCATG

16 CATGACCACTAGGAGCATCTTTGGCGAAGACGACACAGTCGATCGGGATAGAGGAAGCTAGGGGAATAAATCTTTGGGCACCTAGTGGTCATG

15 CATGACCACTAGGAGCATCTTTGGCGAAGTGATCGGAGATTACAAGCAGACGTTGGGCTAGGGGAATAAATCTTTGGGCACCTAGTGGTCATG

15 CATGACCACTAGGAGCATCTTTGGCGAGATCGCGAGAATCGGTGGCATTGGTGTCTCCTAGGGGAATAAATCTTTGGGCACCTAGTGGTCATG

15 CATGACCACTAGGAGCATCTTTGGCGACGGTGTGGGGAAGTTATATCGGCGGATGCACTAGGGGAATAAATCTTTGGGCACCTAGTGGTCATG

15 CATGACCACTAGGAGCATCTTTGGCGAGATCGGGAGAATCGGAGGCATTGGTGTCTCCTAGGGGAATAAATCTTTGGGCACCTAGTGGTCATG

15 CATGACCACTAGGAGCATCTTTGGCGACGGGATCGTGTCGGCAGATAGCGTACTTGGCTAGGGGAATAAATCTTTGGGCACCTAGTGGTCATG

15 CATGACCACTAGGAGCATCTTTGGCGAGGGGTAGGGGGCCGCTCGCGCCTGCGTGCGCTAGGGGAATAAATCTTTGGGCACCTAGTGGTCATG

14 CATGACCACTAGGAGCATCTTTGGCGAAACGACCTTGTCGTTGGGGACCAGTTGCTGCTAGGGGAATAAATCTTTGGGCACCTAGTGGTCATG

14 CATGACCACTAGGAGCATCTTTGGCGAGATAGGGAGAATCGGTGGCATTGGTGTCTCCTAGGGGAATAAATCTTTGGGCACCTAGTGGTCATG

14 CATGACCACTAGGAGCATCTTTGGCGAGTGGTGGTCGGACGGAAAGCATACACGAGACTAGGGGAATAAATCTTTGGGCACCTAGTGGTCATG

14 CATGACCACTAGGAGCATCTTTGGCGAAGTGCGGTGGGCATGTGGGTGGTAGCGACGCTAGGGGAATAAATCTTTGGGCACCTAGTGGTCATG

14 CATGACCACTAGGAGCATCTTTGGCGAGATCGGGAGAATCGTTGGCATTGGTGTCTCCTAGGGGAATAAATCTTTGGGCACCTAGTGGTCATG

14 CATGACCACTAGGAGCATCTTTGGCGAGTGTGGTCAGCGTCACCGACGACACAGCGACTAGGGGAATAAATCTTTGGGCACCTAGTGGTCATG

13 CATGACCACTAGGAGCATCTTTGGCGAGAGCCGCCACGGGTTGGGCCGGTGCGACAGCTAGGGGAATAAATCTTTGGGCACCTAGTGGTCATG

13 CATGACCACTAGGAGCATCTTTGGCGACGGCGTGGGGCATCTCTCGCGGCGGTTATTCTAGGGGAATAAATCTTTGGGCACCTAGTGGTCATG

13 CATGACCACTAGGAGCATCTTTGGCGACGTGGGCTTAAAGGGGGAGGTTAGGGGTGGCTAGGGGAATAAATCTTTGGGCACCTAGTGGTCATG

13 CATGACCACTAGGAGCATCTTTGGCGAAAGTGGGCGATCTGGGAGAGGTCAATCATGCTAGGGGAATAAATCTTTGGGCACCTAGTGGTCATG

13 CATGACCACTAGGAGCATCTTTGGCGAGATCGGGAGAATCGGTGGCACTGGTGTCTCCTAGGGGAATAAATCTTTGGGCACCTAGTGGTCATG

13 CATGACCACTAGGAGCATCTTTGGCGACAATGGGGCTGGTAGGAAAGTACTTATAGCCTAGGGGAATAAATCTTTGGGCACCTAGTGGTCATG

12 CATGACCACTAGGAGCATCTTTGGCGACGTGGGCTTGAAGGGGGAGGTTAGGGGTGGCTAGGGGAATAAATCTTTGGGCACCTAGTGGTCATG

12 CATGACCACTAGGAGCATCTTTGGCGACGGAGTGGGGCTCATTGTGCGGCGGTAATCCTAGGGGAATAAATCTTTGGGCACCTAGTGGTCATG

12 CATGACCACTAGGAGCATCTTTGGCGACAGCCGTAGCCGTAAGGTTGGTATAAGGCCCTAGGGGAATAAATCTTTGGGCACCTAGTGGTCATG

12 CATGACCACTAGGAGCATCTTTGGCGAGAAGACCCTGGATTCGGGGACCAGTTGCTGCTAGGGGAATAAATCTTTGGGCACCTAGTGGTCATG

12 CATGACCACTAGGAGCATCTTTGGCGAAGATCCGATGGGCTTGTGACCCGCCTCGGCCTAGGGGAATAAATCTTTGGGCACCTAGTGGTCATG

12 CATGACCACTAGGAGCATCTTTGGCGATATCGGGAGAATCGGTGGCATTGGTGTCTCCTAGGGGAATAAATCTTTGGGCACCTAGTGGTCATG

12 CATGACCACTAGGAGCATCTTTGGCGAACGGCAGGTGTTGCGGTGGTCTATGAATCCCTAGGGGAATAAATCTTTGGGCACCTAGTGGTCATG

12 CATGACCACTAGGAGCATCTTTGGCGAGATCGGGAGAATCGGTGGCAGTGGTGTCTCCTAGGGGAATAAATCTTTGGGCACCTAGTGGTCATG

12 CATGACCACTAGGAGCATCTTTGGCGATACAGGTGATCGGTGCTCGGGTGCCTGGCCCTAGGGGAATAAATCTTTGGGCACCTAGTGGTCATG

12 CATGACCACTAGGAGCATCTTTGGCGAGATCTGGAGAATCGGTGGCATTGGTGTCTCCTAGGGGAATAAATCTTTGGGCACCTAGTGGTCATG

12 CATGACCACTAGGAGCATCTTTGGCGACGGGGTGGGGCCGAAGTGCGGCGGCGACTCCTAGGGGAATAAATCTTTGGGCACCTAGTGGTCATG

12 CATGACCACTAGGAGCATCTTTGGCGACACGTGGTCCTTGGGGCAATCATACGAATGCTAGGGGAATAAATCTTTGGGCACCTAGTGGTCATG

11 CATGACCACTAGGAGCATCTTTGGCGAGCTCTACGCGTCCTTGAGTGCCGACGTACACTAGGGGAATAAATCTTTGGGCACCTAGTGGTCATG

11 CATGACCACTAGGAGCATCTTTGGCGAGATCGGGAGAATCGGTGGCATTGGTGTCTACTAGGGGAATAAATCTTTGGGCACCTAGTGGTCATG

11 CATGACCACTAGGAGCATCTTTGGCGACTTTGGGAGCAAGTGCGTCGGAGTGGTCGCCTAGGGGAATAAATCTTTGGGCACCTAGTGGTCATG

11 CATGACCACTAGGAGCATCTTTGGCGAGGTGTCTCAGTTCGACATAAGGGGCGAGCACTAGGGGAATAAATCTTTGGGCACCTAGTGGTCATG

11 CATGACCACTAGGAGCATCTTTGGCGAGATCGGGAGAATCGGTGGCATTGGCGTCTCCTAGGGGAATAAATCTTTGGGCACCTAGTGGTCATG

11 CATGACCACTAGGAGCATCTTTGGCGACATGTACACCACCTTTGCCTGGAGCGTAGGCTAGGGGAATAAATCTTTGGGCACCTAGTGGTCATG

11 CATGACCACTAGGAGCATCTTTGGCGAGATCGGGAGAATCGGTGGCATTGATGTCTCCTAGGGGAATAAATCTTTGGGCACCTAGTGGTCATG

10 CATGACCACTAGGAGCATCTTTGGCGAGGACCGGTAAACTAGCATTAGCGTTGCGCTCTAGGGGAATAAATCTTTGGGCACCTAGTGGTCATG

10 CATGACCACTAGGAGCATCTTTGGCGAGATCGGGAGGATCGGTGGCATTGGTGTCTCCTAGGGGAATAAATCTTTGGGCACCTAGTGGTCATG

10 CATGACCACTAGGAGCATCTTTGGCGAGATCGGGAGAATCGGTGGCATTGTTGTCTCCTAGGGGAATAAATCTTTGGGCACCTAGTGGTCATG

10 CATGACCACTAGGAGCATCTTTGGCGAGGGGGCGAGTGGGCGAGGTGTTATAGGGAGCTAGGGGAATAAATCTTTGGGCACCTAGTGGTCATG

10 CATGACCACTAGGAGCATCTTTGGCGACGTGGGCTGGGAGGATGTGGTACTTCTCGACTAGGGGAATAAATCTTTGGGCACCTAGTGGTCATG

10 CATGACCACTAGGAGCATCTTTGGCGACGTCTGTCCTTGGTAGTTACGGCCAATCTCCTAGGGGAATAAATCTTTGGGCACCTAGTGGTCATG

10 CATGACCACTAGGAGCATCTTTGGCGAGATCGGGAGAATCGGTGGCATTAGTGTCTCCTAGGGGAATAAATCTTTGGGCACCTAGTGGTCATG

10 CATGACCACTAGGAGCATCTTTGGCGACGGGGCCGGACGGGACGGACACATCGCGGCCTAGGGGAATAAATCTTTGGGCACCTAGTGGTCATG

10 CATGACCACTAGGAGCATCTTTGGCGACTCGACCGGGGCCCTAGGGCTGCGGCCCACCTAGGGGAATAAATCTTTGGGCACCTAGTGGTCATG

10 CATGACCACTAGGAGCATCTTTGGCGAGATCGGGAGAATAGGTGGCATTGGTGTCTCCTAGGGGAATAAATCTTTGGGCACCTAGTGGTCATG

10 CATGACCACTAGGAGCATCTTTGGCGATCCGGGGGCACCTATGTGCGACGCTGTGGGCTAGGGGAATAAATCTTTGGGCACCTAGTGGTCATG

10 CATGACCACTAGGAGCATCTTTGGCGACGGATACGGTAGGTTTGTGTGCCTAGCCTGCTAGGGGAATAAATCTTTGGGCACCTAGTGGTCATG

10 CATGACCACTAGGAGCATCTTTGGCGAAATCGGGAGAATCGGTGGCATTGGTGTCTCCTAGGGGAATAAATCTTTGGGCACCTAGTGGTCATG

9 CATGACCACTAGGAGCATCTTTGGCGATCCGGGGGTCACAAGTGACGGCGCATAGGGCTAGGGGAATAAATCTTTGGGCACCTAGTGGTCATG

9 CATGACCACTAGGAGCATCTTTGGCGAGATGGTGTACACCGTCGAGCAGACGTTGGGCTAGGGGAATAAATCTTTGGGCACCTAGTGGTCATG

9 CATGACCACTAGGAGCATCTTTGGCGAGAACGGGAGAATCGGTGGCATTGGTGTCTCCTAGGGGAATAAATCTTTGGGCACCTAGTGGTCATG

9 CATGACCACTAGGAGCATCTTTGGCGACGTGGTGCGAAAGGGGGAGGTTAGGGGTGGCTAGGGGAATAAATCTTTGGGCACCTAGTGGTCATG

9 CATGACCACTAGGAGCATCTTTGGCGAGATTGGGAGAATCGGTGGCATTGGTGTCTCCTAGGGGAATAAATCTTTGGGCACCTAGTGGTCATG

9 CATGACCACTAGGAGCATCTTTGGCGAGACCGGGAGAATCGGTGGCATTGGTGTCTCCTAGGGGAATAAATCTTTGGGCACCTAGTGGTCATG

9 CATGACCACTAGGAGCATCTTTGGCGAGATCGGGAGAATCGGTGGCATAGGTGTCTCCTAGGGGAATAAATCTTTGGGCACCTAGTGGTCATG

9 CATGACCACTAGGAGCATCTTTGGCGAGATCGGGAGAATCGGTGGCGTTGGTGTCTCCTAGGGGAATAAATCTTTGGGCACCTAGTGGTCATG

9 CATGACCACTAGGAGCATCTTTGGCGAGAAGACTCCGGATTCGGGGACCAGTTGCTGCTAGGGGAATAAATCTTTGGGCACCTAGTGGTCATG

9 CATGACCACTAGGAGCATCTTTGGCGACTCCAGTTCATCTGTCGGGCAGTTTAAGGACTAGGGGAATAAATCTTTGGGCACCTAGTGGTCATG

9 CATGACCACTAGGAGCATCTTTGGCGATTTGGGAGTCGCAGTATGCAGGCAGTGAGACTAGGGGAATAAATCTTTGGGCACCTAGTGGTCATG

9 CATGACCACTAGGAGCATCTTTGGCGACGTGGGCACAAGGAACAGAGAACACAGAGACTAGGGGAATAAATCTTTGGGCACCTAGTGGTCATG

9 CATGACCACTAGGAGCATCTTTGGCGAATGGCCGGCACGGCCTTCTAGTCCTTGGTACTAGGGGAATAAATCTTTGGGCACCTAGTGGTCATG

9 CATGACCACTAGGAGCATCTTTGGCGAGGTCGCGTTAGCGATTGCGGCGGGCCTCGACTAGGGGAATAAATCTTTGGGCACCTAGTGGTCATG

9 CATGACCACTAGGAGCATCTTTGGCGAGATCGGGAGACTCGGTGGCATTGGTGTCTCCTAGGGGAATAAATCTTTGGGCACCTAGTGGTCATG

9 CATGACCACTAGGAGCATCTTTGGCGAGTAGGGGGCACCGGTAATCGACAGACGAGCCTAGGGGAATAAATCTTTGGGCACCTAGTGGTCATG

8 CATGACCACTAGGAGCATCTTTGGCGAGATCGGGAGAATCGGTGGCATTGGTGTCGCCTAGGGGAATAAATCTTTGGGCACCTAGTGGTCATG

8 CATGACCACTAGGAGCATCTTTGGCGACGTGGTGTGTTGCCCAAGTGGCTGAGTTGACTAGGGGAATAAATCTTTGGGCACCTAGTGGTCATG

8 CATGACCACTAGGAGCATCTTTGGCGAGATCGGGAGAATCTGTGGCATTGGTGTCTCCTAGGGGAATAAATCTTTGGGCACCTAGTGGTCATG

8 CATGACCACTAGGAGCATCTTTGGCGAACGGCAGGTGTTGCGGTGGTCCGTGAATCCCTAGGGGAATAAATCTTTGGGCACCTAGTGGTCATG

8 CATGACCACTAGGAGCATCTTTGGCGAAGTGGGCATGCGCTGGGTGCTTAACGCAGACTAGGGGAATAAATCTTTGGGCACCTAGTGGTCATG

8 CATGACCACTAGGAGCATCTTTGGCGAGGGGATCTGTCGGGCCGGAGTGCTCCAACGCTAGGGGAATAAATCTTTGGGCACCTAGTGGTCATG

8 CATGACCACTAGGAGCATCTTTGGCGACGTTATGAGTTGGGAAGCCTGGCGGCCAGACTAGGGGAATAAATCTTTGGGCACCTAGTGGTCATG

8 CATGACCACTAGGAGCATCTTTGGCGAGAAGACTTTGGATTCGGGGACCAGTTGCTGCTAGGGGAATAAATCTTTGGGCACCTAGTGGTCATG

8 CATGACCACTAGGAGCATCTTTGGCGAGATCGGGAGAATCGGTGGCATTGGTGTCTTCTAGGGGAATAAATCTTTGGGCACCTAGTGGTCATG

8 CATGACCACTAGGAGCATCTTTGGCGAAGATCGCTCGTCGACTCGGGTCCGAGGAAGCTAGGGGAATAAATCTTTGGGCACCTAGTGGTCATG

8 CATGACCACTAGGAGCATCTTTGGCGATCTGTGCTGAAAAGCCCCGTGGAGGGTTGTCTAGGGGAATAAATCTTTGGGCACCTAGTGGTCATG

8 CATGACCACTAGGAGCATCTTTGGCGAGTCAGGTCATGCAGTAAGAAAAAAACGAGACTAGGGGAATAAATCTTTGGGCACCTAGTGGTCATG

8 CATGACCACTAGGAGCATCTTTGGCGAGTGCGGGCAACACGCAGTCCTGGTGTGCGACTAGGGGAATAAATCTTTGGGCACCTAGTGGTCATG

8 CATGACCACTAGGAGCATCTTTGGCGAGATCGGAAGAATCGGTGGCATTGGTGTCTCCTAGGGGAATAAATCTTTGGGCACCTAGTGGTCATG

8 CATGACCACTAGGAGCATCTTTGGCGAACGGCAGGTGTTGCGGTGGTCTGTGGATCCCTAGGGGAATAAATCTTTGGGCACCTAGTGGTCATG

8 CATGACCACTAGGAGCATCTTTGGCGACCCTGGCGCGGGGATCCTGGTTGCGGCGACCTAGGGGAATAAATCTTTGGGCACCTAGTGGTCATG

7 CATGACCACTAGGAGCATCTTTGGCGAGATCGGGAGAATCGGTAGCATTGGTGTCTCCTAGGGGAATAAATCTTTGGGCACCTAGTGGTCATG

7 CATGACCACTAGGAGCATCTTTGGCGAACGGCAGGTGTTGCGGTGGTCTGTGAACCCCTAGGGGAATAAATCTTTGGGCACCTAGTGGTCATG

7 CATGACCACTAGGAGCATCTTTGGCGAAGGGCGCTAGTGATTCGGTGGTTGCCCCCGCTAGGGGAATAAATCTTTGGGCACCTAGTGGTCATG

7 CATGACCACTAGGAGCATCTTTGGCGAAGTGCGGCGTGCCGAGTCGTCCTGCGACTCCTAGGGGAATAAATCTTTGGGCACCTAGTGGTCATG

7 CATGACCACTAGGAGCATCTTTGGCGACAATGTTAATGTGGTGTGATGCTTAGAATTCTAGGGGAATAAATCTTTGGGCACCTAGTGGTCATG

7 CATGACCACTAGGAGCATCTTTGGCGATGTGATCGACGCGCTATAGGTTGCGGTAAGCTAGGGGAATAAATCTTTGGGCACCTAGTGGTCATG

7 CATGACCACTAGGAGCATCTTTGGCGAACGGCAGGTGCTGCGGTGGTCTGTGAATCCCTAGGGGAATAAATCTTTGGGCACCTAGTGGTCATG

7 CATGACCACTAGGAGCATCTTTGGCGACGGGGTGGGGCCTAAGTGCGGCGGCTGATTCTAGGGGAATAAATCTTTGGGCACCTAGTGGTCATG

7 CATGACCACTAGGAGCATCTTTGGCGAGGCGATATGAAGCGACGAGCAGACGTTTGGCTAGGGGAATAAATCTTTGGGCACCTAGTGGTCATG

7 CATGACCACTAGGAGCATCTTTGGCGACTAGGGGGGATGGACAGTGACATAAGTCGACTAGGGGAATAAATCTTTGGGCACCTAGTGGTCATG

7 CATGACCACTAGGAGCATCTTTGGCGAGGGATGGGAGCCAGGGGGGTTCGGGGTCCGCTAGGGGAATAAATCTTTGGGCACCTAGTGGTCATG

7 CATGACCACTAGGAGCATCTTTGGCGAGATCGGGAGAATCGGTGGCATTGGAGTCTCCTAGGGGAATAAATCTTTGGGCACCTAGTGGTCATG

7 CATGACCACTAGGAGCATCTTTGGCGAGATCGGGAGAATCGGTGGTATTGGTGTCTCCTAGGGGAATAAATCTTTGGGCACCTAGTGGTCATG

7 CATGACCACTAGGAGCATCTTTGGCGAGTAGTGGGTCTGACAAGGGTAAAAACGAGACTAGGGGAATAAATCTTTGGGCACCTAGTGGTCATG

7 CATGACCACTAGGAGCATCTTTGGCGACGGTGTGGGGAACAAGGGTTCGGCGGCTCTCTAGGGGAATAAATCTTTGGGCACCTAGTGGTCATG

7 CATGACCACTAGGAGCATCTTTGGCGAGGCTTGGGCGGGGCGTTGCGTTGCCGAGCTCTAGGGGAATAAATCTTTGGGCACCTAGTGGTCATG

7 CATGACCACTAGGAGCATCTTTGGCGAACGGCAGGTGTTGCGGTGGTCTGTGTATCCCTAGGGGAATAAATCTTTGGGCACCTAGTGGTCATG

7 CATGACCACTAGGAGCATCTTTGGCGAGATCGGGAGAATCGGTGGAATTGGTGTCTCCTAGGGGAATAAATCTTTGGGCACCTAGTGGTCATG

7 CATGACCACTAGGAGCATCTTTGGCGATCAGATACGGGACGGGCAGACAAGGATCGACTAGGGGAATAAATCTTTGGGCACCTAGTGGTCATG

7 CATGACCACTAGGAGCATCTTTGGCGAGATCGGGAGAACCGGTGGCATTGGTGTCTCCTAGGGGAATAAATCTTTGGGCACCTAGTGGTCATG

7 CATGACCACTAGGAGCATCTTTGGCGAGGCTTTCGGTGGTAAGGACTACGTCTAGAGCTAGGGGAATAAATCTTTGGGCACCTAGTGGTCATG

7 CATGACCACTAGGAGCATCTTTGGCGAGATCGGGAGAAACGGTGGCATTGGTGTCTCCTAGGGGAATAAATCTTTGGGCACCTAGTGGTCATG

7 CATGACCACTAGGAGCATCTTTGGCGAATGCGAGGTTGGTTACGATGTGGCTGAGGCCTAGGGGAATAAATCTTTGGGCACCTAGTGGTCATG

7 CATGACCACTAGGAGCATCTTTGGCGAGTGGGGGGCGACGGCCGGTAGTGGGTGAGACTAGGGGAATAAATCTTTGGGCACCTAGTGGTCATG

7 CATGACCACTAGGAGCATCTTTGGCGAGGGTCCCTAGGGCCCTATAGAGTTTGTGAGCTAGGGGAATAAATCTTTGGGCACCTAGTGGTCATG

7 CATGACCACTAGGAGCATCTTTGGCGAGATCGGGAGAATCGGTGGCATTGGTGTTTCCTAGGGGAATAAATCTTTGGGCACCTAGTGGTCATG

7 CATGACCACTAGGAGCATCTTTGGCGAATGAGCCTGTAGGCCAGAGCAGACGTTTGGCTAGGGGAATAAATCTTTGGGCACCTAGTGGTCATG

7 CATGACCACTAGGAGCATCTTTGGCGAGATCGGGAGAATCGGTGGCCTTGGTGTCTCCTAGGGGAATAAATCTTTGGGCACCTAGTGGTCATG

7 CATGACCACTAGGAGCATCTTTGGCGAGATCGGGAGAATCGATGGCATTGGTGTCTCCTAGGGGAATAAATCTTTGGGCACCTAGTGGTCATG

6 CATGACCACTAGGAGCATCTTTGGCGATACGTCGTGTGGTGGTTCAAGGTGAGCTAGCTAGGGGAATAAATCTTTGGGCACCTAGTGGTCATG

6 CATGACCACTAGGAGCATCTTTGGCGAGCCGCTCTGGTTGGTCAGGCCGCTTGTGAACTAGGGGAATAAATCTTTGGGCACCTAGTGGTCATG

6 CATGACCACTAGGAGCATCTTTGGCGAAGCGCAGGGTGTGAGAGGGGCGGGTCCATGCTAGGGGAATAAATCTTTGGGCACCTAGTGGTCATG

6 CATGACCACTAGGAGCATCTTTGGCGAGTAGGGGTCGTGTGCTAAGTACGGACGAGACTAGGGGAATAAATCTTTGGGCACCTAGTGGTCATG

6 CATGACCACTAGGAGCATCTTTGGCGACTAGCGGGCAGCTGTAGGACATACGTCGCTCTAGGGGAATAAATCTTTGGGCACCTAGTGGTCATG

6 CATGACCACTAGGAGCATCTTTGGCGAACTTGAACGTCGAAGTGGCAGAGAAAGCGACTAGGGGAATAAATCTTTGGGCACCTAGTGGTCATG

6 CATGACCACTAGGAGCATCTTTGGCGACGTGGCATGGAGATCGGAAGTAAGAGTCGACTAGGGGAATAAATCTTTGGGCACCTAGTGGTCATG

6 CATGACCACTAGGAGCATCTTTGGCGATTACGAGGCCGCTCAGAATGCGAAGTGGAGCTAGGGGAATAAATCTTTGGGCACCTAGTGGTCATG

6 CATGACCACTAGGAGCATCTTTGGCGAGGGGCGCAAGGCCGTGGGCAAAGGTCGAGACTAGGGGAATAAATCTTTGGGCACCTAGTGGTCATG

6 CATGACCACTAGGAGCATCTTTGGCGAAGTGTGGAACATGCCGGTTTTGGTTGCACCCTAGGGGAATAAATCTTTGGGCACCTAGTGGTCATG

6 CATGACCACTAGGAGCATCTTTGGCGAGTAAGGTGTGGGCGGTGAGAAACAATACGACTAGGGGAATAAATCTTTGGGCACCTAGTGGTCATG

6 CATGACCACTAGGAGCATCTTTGGCGACGGCGTGGGGCCTCTAGCGCGGCGGGGATCCTAGGGGAATAAATCTTTGGGCACCTAGTGGTCATG

6 CATGACCACTAGGAGCATCTTTGGCGACGGGGGCATGCACAGGATCGAGACAGTCGACTAGGGGAATAAATCTTTGGGCACCTAGTGGTCATG

6 CATGACCACTAGGAGCATCTTTGGCGAAGCTTGGTTAGGCGACGGGCCTGGGCCACTCTAGGGGAATAAATCTTTGGGCACCTAGTGGTCATG

6 CATGACCACTAGGAGCATCTTTGGCGACGAGTTGGGCGTTGGATAGTTGGCAGTCGACTAGGGGAATAAATCTTTGGGCACCTAGTGGTCATG

6 CATGACCACTAGGAGCATCTTTGGCGAAGAGCTGTGCACGTGGGTGTGGTCCGGGCCCTAGGGGAATAAATCTTTGGGCACCTAGTGGTCATG

6 CATGACCACTAGGAGCATCTTTGGCGAAGAGTGGTCGGTCGTCAAATAGAACTGAGCCTAGGGGAATAAATCTTTGGGCACCTAGTGGTCATG

6 CATGACCACTAGGAGCATCTTTGGCGACCGAGTGGGCTGGGAGGAAGTATTTCGGAGCTAGGGGAATAAATCTTTGGGCACCTAGTGGTCATG

6 CATGACCACTAGGAGCATCTTTGGCGAGTGGACTCAATCATACGAGCAGACGTTTGGCTAGGGGAATAAATCTTTGGGCACCTAGTGGTCATG

6 CATGACCACTAGGAGCATCTTTGGCGACTGGATACGGAAGGCAAGTGTGCCACTAGCCTAGGGGAATAAATCTTTGGGCACCTAGTGGTCATG

6 CATGACCACTAGGAGCATCTTTGGCGATCTGGGGCAGCCAGGTCAGGAAAGGATCGACTAGGGGAATAAATCTTTGGGCACCTAGTGGTCATG

6 CATGACCACTAGGAGCATCTTTGGCGAGGTCGGGAGAATCGGTGGCATTGGTGTCTCCTAGGGGAATAAATCTTTGGGCACCTAGTGGTCATG

6 CATGACCACTAGGAGCATCTTTGGCGACGTGGGCTTGCAGGGGGAGGTTAGGGGTGGCTAGGGGAATAAATCTTTGGGCACCTAGTGGTCATG

6 CATGACCACTAGGAGCATCTTTGGCGACCACGAAGGTACAAATAGCCAAGGGTACGACTAGGGGAATAAATCTTTGGGCACCTAGTGGTCATG

6 CATGACCACTAGGAGCATCTTTGGCGAGGTTGTTCGAAGTTATCACCCTTAAGGGTGCTAGGGGAATAAATCTTTGGGCACCTAGTGGTCATG

6 CATGACCACTAGGAGCATCTTTGGCGAGAAGACTCTGGATTCGGGGACCAGTTGCTCCTAGGGGAATAAATCTTTGGGCACCTAGTGGTCATG

6 CATGACCACTAGGAGCATCTTTGGCGATAGAGGTCAGTCATGTAGGGCAGTCTTCGCCTAGGGGAATAAATCTTTGGGCACCTAGTGGTCATG

6 CATGACCACTAGGAGCATCTTTGGCGAATGCTGCCGTGGGCAAGCGGGTGTGAGGAACTAGGGGAATAAATCTTTGGGCACCTAGTGGTCATG

6 CATGACCACTAGGAGCATCTTTGGCGACTCGTGGGCATATATGGGCCACGTAGTGGACTAGGGGAATAAATCTTTGGGCACCTAGTGGTCATG

6 CATGACCACTAGGAGCATCTTTGGCGAACCCGCGCGCGCTATAGGTTGCTCGAGCTCCTAGGGGAATAAATCTTTGGGCACCTAGTGGTCATG

6 CATGACCACTAGGAGCATCTTTGGCGAGCGGCAGGTGTTGCGGTGGTCTGTGAATCCCTAGGGGAATAAATCTTTGGGCACCTAGTGGTCATG

6 CATGACCACTAGGAGCATCTTTGGCGACGTGGCTGGTAGGGTAAGTACTGTGTCGATCTAGGGGAATAAATCTTTGGGCACCTAGTGGTCATG

6 CATGACCACTAGGAGCATCTTTGGCGACATTGACAGCTAGGGTGAGGTTGTGTGCGACTAGGGGAATAAATCTTTGGGCACCTAGTGGTCATG

6 CATGACCACTAGGAGCATCTTTGGCGACTAGGGTCCCGGGGATGCAGCACGAGTCGACTAGGGGAATAAATCTTTGGGCACCTAGTGGTCATG

6 CATGACCACTAGGAGCATCTTTGGCGAACACGAGAGTCAAGTGCGTATAGCGTGGAGCTAGGGGAATAAATCTTTGGGCACCTAGTGGTCATG

6 CATGACCACTAGGAGCATCTTTGGCGAGATCGGCAGAATCGGTGGCATTGGTGTCTCCTAGGGGAATAAATCTTTGGGCACCTAGTGGTCATG

6 CATGACCACTAGGAGCATCTTTGGCGACTCGGTGGGCACTGCCGTATACACGGGCGACTAGGGGAATAAATCTTTGGGCACCTAGTGGTCATG

6 CATGACCACTAGGAGCATCTTTGGCGAACGGTAGGTGTTGCGGTGGTCTGTGAATCCCTAGGGGAATAAATCTTTGGGCACCTAGTGGTCATG

6 CATGACCACTAGGAGCATCTTTGGCGACCGAGGCAGCGGGGGAAATATGCGCAGGTCCTAGGGGAATAAATCTTTGGGCACCTAGTGGTCATG

6 CATGACCACTAGGAGCATCTTTGGCGACCTCGGCTGGTAGGTAATGTATTGGGCGATCTAGGGGAATAAATCTTTGGGCACCTAGTGGTCATG

6 CATGACCACTAGGAGCATCTTTGGCGAGTCAGTGGGCGGGCGATACTGGGTACGAGACTAGGGGAATAAATCTTTGGGCACCTAGTGGTCATG

6 CATGACCACTAGGAGCATCTTTGGCGACTACGGGCTAGAGTTACTCGTGTTAATAAGCTAGGGGAATAAATCTTTGGGCACCTAGTGGTCATG

6 CATGACCACTAGGAGCATCTTTGGCGAGTGTGGGCTGGCAGTGCGTACTTGATCGCTCTAGGGGAATAAATCTTTGGGCACCTAGTGGTCATG

6 CATGACCACTAGGAGCATCTTTGGCGACTACGTGGGCTAGGCATTCGGAACAGGTCGCTAGGGGAATAAATCTTTGGGCACCTAGTGGTCATG

6 CATGACCACTAGGAGCATCTTTGGCGAAGCACCCGGCTCACCAGGGAAACCGTTCGGCTAGGGGAATAAATCTTTGGGCACCTAGTGGTCATG

6 CATGACCACTAGGAGCATCTTTGGCGACATTGTGGGCATGCGCGAATAAGAGGTCGACTAGGGGAATAAATCTTTGGGCACCTAGTGGTCATG

6 CATGACCACTAGGAGCATCTTTGGCGACACCTTTAGAGCGGCGGCATTGTTGCGTGCCTAGGGGAATAAATCTTTGGGCACCTAGTGGTCATG

5 CATGACCACTAGGAGCATCTTTGGCGACTCGACGGGTTGAGGAGGGATGCGAGGCGACTAGGGGAATAAATCTTTGGGCACCTAGTGGTCATG

5 CATGACCACTAGGAGCATCTTTGGCGACGCAAGTGGGCCAGCAAAAGTCACAGGAGCCTAGGGGAATAAATCTTTGGGCACCTAGTGGTCATG

5 CATGACCACTAGGAGCATCTTTGGCGACGTCGTGGCTGGTAGAGTGTATTTGACAAGCTAGGGGAATAAATCTTTGGGCACCTAGTGGTCATG

5 CATGACCACTAGGAGCATCTTTGGCGACGATCACGAATCAGCAGTCAAAAGAGGCGACTAGGGGAATAAATCTTTGGGCACCTAGTGGTCATG

5 CATGACCACTAGGAGCATCTTTGGCGATCCTCGCGGGGGTGCCCTTTATGGCCCTAGCTAGGGGAATAAATCTTTGGGCACCTAGTGGTCATG

5 CATGACCACTAGGAGCATCTTTGGCGAATTCGAGAGTCCGCGGCGGACCCAATTCGGCTAGGGGAATAAATCTTTGGGCACCTAGTGGTCATG

5 CATGACCACTAGGAGCATCTTTGGCGATGGTCTAGGCGTCATCGGGCCTAGTACATCCTAGGGGAATAAATCTTTGGGCACCTAGTGGTCATG

5 CATGACCACTAGGAGCATCTTTGGCGATCATTTAGGGCACGGACGAATGTGGATCGCCTAGGGGAATAAATCTTTGGGCACCTAGTGGTCATG

5 CATGACCACTAGGAGCATCTTTGGCGACGTAGTGGGCCTTGGCACATATGTGAGAGCCTAGGGGAATAAATCTTTGGGCACCTAGTGGTCATG

5 CATGACCACTAGGAGCATCTTTGGCGACCGAGTGGTAAAGCGCGGAGAAGAGGAAGACTAGGGGAATAAATCTTTGGGCACCTAGTGGTCATG

5 CATGACCACTAGGAGCATCTTTGGCGAGTGTGCATGTTGTAGATAAAGTAGGTTCGCCTAGGGGAATAAATCTTTGGGCACCTAGTGGTCATG

5 CATGACCACTAGGAGCATCTTTGGCGACGGACAAGGCATGAGCCCGAGGGAGTCAGACTAGGGGAATAAATCTTTGGGCACCTAGTGGTCATG

5 CATGACCACTAGGAGCATCTTTGGCGACCGTAAGACCTGAGCGGGGATGAGCCCACACTAGGGGAATAAATCTTTGGGCACCTAGTGGTCATG

5 CATGACCACTAGGAGCATCTTTGGCGATATTGGCGTTAGCTAACATCGTAGGATGTTCTAGGGGAATAAATCTTTGGGCACCTAGTGGTCATG

5 CATGACCACTAGGAGCATCTTTGGCGATGTTCCTGTCGGTCATTCAGAACGATCCGCCTAGGGGAATAAATCTTTGGGCACCTAGTGGTCATG

5 CATGACCACTAGGAGCATCTTTGGCGATGGGTGCTGACGACCGTCCACACGGTTACTCTAGGGGAATAAATCTTTGGGCACCTAGTGGTCATG

5 CATGACCACTAGGAGCATCTTTGGCGAAATCCCGAGCAAGGGAATCCCGGGATGCAGCTAGGGGAATAAATCTTTGGGCACCTAGTGGTCATG

5 CATGACCACTAGGAGCATCTTTGGCGACTGGTGGCGCTGTTGAGCGGTAGGCCGAGACTAGGGGAATAAATCTTTGGGCACCTAGTGGTCATG

5 CATGACCACTAGGAGCATCTTTGGCGACGTCGGCTGGTAGAGACTGTATTGAGGAACCTAGGGGAATAAATCTTTGGGCACCTAGTGGTCATG

5 CATGACCACTAGGAGCATCTTTGGCGAGGGGGCATGGCGACATTGGCTAGGGCTCGACTAGGGGAATAAATCTTTGGGCACCTAGTGGTCATG

5 CATGACCACTAGGAGCATCTTTGGCGAGATCGGGATAATCGGTGGCATTGGTGTCTCCTAGGGGAATAAATCTTTGGGCACCTAGTGGTCATG

5 CATGACCACTAGGAGCATCTTTGGCGACGCGGGTGCAACAAGCTAGCAGACGTTTGGCTAGGGGAATAAATCTTTGGGCACCTAGTGGTCATG

5 CATGACCACTAGGAGCATCTTTGGCGAGGGAGCGGGACACAGATGCGACGGGTCGAGCTAGGGGAATAAATCTTTGGGCACCTAGTGGTCATG

5 CATGACCACTAGGAGCATCTTTGGCGAGATCGGGAGAATCGGTGGCATTGGTGTCACCTAGGGGAATAAATCTTTGGGCACCTAGTGGTCATG

5 CATGACCACTAGGAGCATCTTTGGCGAGGACGTATGCGGCTTTACGCCGAAGGTCGCCTAGGGGAATAAATCTTTGGGCACCTAGTGGTCATG

5 CATGACCACTAGGAGCATCTTTGGCGACTTGTGGGCAATTAGATATCGGTACGTCGACTAGGGGAATAAATCTTTGGGCACCTAGTGGTCATG

5 CATGACCACTAGGAGCATCTTTGGCGAGAGCCCGGTGTGGGTGCGCAAGCTATATGCCTAGGGGAATAAATCTTTGGGCACCTAGTGGTCATG

5 CATGACCACTAGGAGCATCTTTGGCGACGGTGTGGGGAAGTTATATCGGCGGATGCTCTAGGGGAATAAATCTTTGGGCACCTAGTGGTCATG

5 CATGACCACTAGGAGCATCTTTGGCGACTCTGGGTCATGCTCGGCGTAAGAGTTCGACTAGGGGAATAAATCTTTGGGCACCTAGTGGTCATG

5 CATGACCACTAGGAGCATCTTTGGCGATTGAAGCCGGTGCGGTGCGGCGAGATGCGACTAGGGGAATAAATCTTTGGGCACCTAGTGGTCATG

5 CATGACCACTAGGAGCATCTTTGGCGACGTGGGCACTACAGAACAGAGGCGTCGACGCTAGGGGAATAAATCTTTGGGCACCTAGTGGTCATG

5 CATGACCACTAGGAGCATCTTTGGCGACGGTGTGGGGCTCCATGATGCGGCGGACTCCTAGGGGAATAAATCTTTGGGCACCTAGTGGTCATG

5 CATGACCACTAGGAGCATCTTTGGCGACGTCGTAAAGTGCGTAGAGTGGTAAGTCGACTAGGGGAATAAATCTTTGGGCACCTAGTGGTCATG

5 CATGACCACTAGGAGCATCTTTGGCGACATGGGCGACCTAAGAGAGGAGAAGTCGAACTAGGGGAATAAATCTTTGGGCACCTAGTGGTCATG

5 CATGACCACTAGGAGCATCTTTGGCGAAGTTCCATGGATAGCTGGTAAGAACGATGACTAGGGGAATAAATCTTTGGGCACCTAGTGGTCATG

5 CATGACCACTAGGAGCATCTTTGGCGAAGTATCGACTAGATTCGAGCAGACGTTTGGCTAGGGGAATAAATCTTTGGGCACCTAGTGGTCATG

5 CATGACCACTAGGAGCATCTTTGGCGAAATTGGATGGCCGATTGCCATACGATGGCGCTAGGGGAATAAATCTTTGGGCACCTAGTGGTCATG

5 CATGACCACTAGGAGCATCTTTGGCGATCAAGTGCGCTTGGTAATTCCTGATGCGATCTAGGGGAATAAATCTTTGGGCACCTAGTGGTCATG

5 CATGACCACTAGGAGCATCTTTGGCGACCCCCTCCGTTGCGGGTTTGACGAGGTTTGCTAGGGGAATAAATCTTTGGGCACCTAGTGGTCATG

5 CATGACCACTAGGAGCATCTTTGGCGACGTAGGGGGCATGGGTCAGAAAGTCGCCAGCTAGGGGAATAAATCTTTGGGCACCTAGTGGTCATG

5 CATGACCACTAGGAGCATCTTTGGCGAGCCGTGACAGCGGGACAGCACGAGTGCGAACTAGGGGAATAAATCTTTGGGCACCTAGTGGTCATG

5 CATGACCACTAGGAGCATCTTTGGCGAGCGTGAAGGCCTTAGACTGGTATACGGTCGCTAGGGGAATAAATCTTTGGGCACCTAGTGGTCATG

5 CATGACCACTAGGAGCATCTTTGGCGATGGGTTGCTGGTAGGTGGTACGACCTCGATCTAGGGGAATAAATCTTTGGGCACCTAGTGGTCATG

5 CATGACCACTAGGAGCATCTTTGGCGATGTGGTCAGTAGGGTCAATGCGGAGATCGCCTAGGGGAATAAATCTTTGGGCACCTAGTGGTCATG

5 CATGACCACTAGGAGCATCTTTGGCGACTAGTGGGGTCCGAGAAAGTCAGTAGTCGACTAGGGGAATAAATCTTTGGGCACCTAGTGGTCATG

5 CATGACCACTAGGAGCATCTTTGGCGAGTCATGTAAGGACAGCTACGACTGCGGCGCCTAGGGGAATAAATCTTTGGGCACCTAGTGGTCATG

5 CATGACCACTAGGAGCATCTTTGGCGACATATGTTTGCGAGGTCCAGGCGTGTCGCTCTAGGGGAATAAATCTTTGGGCACCTAGTGGTCATG

5 CATGACCACTAGGAGCATCTTTGGCGAGCGTGGGGCACGTTATCGAGTCACACCTAGCTAGGGGAATAAATCTTTGGGCACCTAGTGGTCATG

5 CATGACCACTAGGAGCATCTTTGGCGACCCCAGTACGCGTTAGCTATACGGGGTGGACTAGGGGAATAAATCTTTGGGCACCTAGTGGTCATG

5 CATGACCACTAGGAGCATCTTTGGCGACTAGAGGGGCGTGGCTAGGTCATTAGTCGACTAGGGGAATAAATCTTTGGGCACCTAGTGGTCATG

5 CATGACCACTAGGAGCATCTTTGGCGACTATACGTTATCGCGAGGTCGTTGCGAGCCCTAGGGGAATAAATCTTTGGGCACCTAGTGGTCATG

5 CATGACCACTAGGAGCATCTTTGGCGAGTAGTGCGTCTCGGGTAAGGTGCACCTCGACTAGGGGAATAAATCTTTGGGCACCTAGTGGTCATG

5 CATGACCACTAGGAGCATCTTTGGCGATAGTCGGTAGAACAGGGTGGGGTGCTGTCCCTAGGGGAATAAATCTTTGGGCACCTAGTGGTCATG

5 CATGACCACTAGGAGCATCTTTGGCGAGGAGTGTGGCATGGACAGAAACACCGAAGACTAGGGGAATAAATCTTTGGGCACCTAGTGGTCATG

5 CATGACCACTAGGAGCATCTTTGGCGACGAGCATGTGTGTGCGAGAAGTGTTTGGTGCTAGGGGAATAAATCTTTGGGCACCTAGTGGTCATG

5 CATGACCACTAGGAGCATCTTTGGCGACGTGTGGGCACGGGGGATGGAAACACTAGCCTAGGGGAATAAATCTTTGGGCACCTAGTGGTCATG

5 CATGACCACTAGGAGCATCTTTGGCGAGTAGGGTCCAAACGTAAGCTATGCACGAGACTAGGGGAATAAATCTTTGGGCACCTAGTGGTCATG

5 CATGACCACTAGGAGCATCTTTGGCGATCACAGGTCTACAGGAGCGACAGACCGCGCCTAGGGGAATAAATCTTTGGGCACCTAGTGGTCATG

5 CATGACCACTAGGAGCATCTTTGGCGAAAGCGCATAGACGGGTCGTTGTACGGTCTACTAGGGGAATAAATCTTTGGGCACCTAGTGGTCATG

5 CATGACCACTAGGAGCATCTTTGGCGACGGGCGGGCGCCCTAGGGTGTGAGTGTTGCCTAGGGGAATAAATCTTTGGGCACCTAGTGGTCATG

5 CATGACCACTAGGAGCATCTTTGGCGAGATCGGGAGAATCGGTGTCATTGGTGTCTCCTAGGGGAATAAATCTTTGGGCACCTAGTGGTCATG

5 CATGACCACTAGGAGCATCTTTGGCGACGTGGGCTTGCTGGGGGAGGTTAGGGGTGGCTAGGGGAATAAATCTTTGGGCACCTAGTGGTCATG

5 CATGACCACTAGGAGCATCTTTGGCGACTTTGCCGGGCAGACCGCGCCGTTAGTTGTCTAGGGGAATAAATCTTTGGGCACCTAGTGGTCATG

5 CATGACCACTAGGAGCATCTTTGGCGACGAGTTGACCTATGGGTCTGCTGGGAGCGCCTAGGGGAATAAATCTTTGGGCACCTAGTGGTCATG

5 CATGACCACTAGGAGCATCTTTGGCGACTCTGGGGTCATAGGCTAGGACTGGTCGCTCTAGGGGAATAAATCTTTGGGCACCTAGTGGTCATG

5 CATGACCACTAGGAGCATCTTTGGCGAACGGCAGGGGTTGCGGTGGTCTGTGAATCCCTAGGGGAATAAATCTTTGGGCACCTAGTGGTCATG

5 CATGACCACTAGGAGCATCTTTGGCGACGAGAGTGGGTCATGGTGGAATAAAGTCGACTAGGGGAATAAATCTTTGGGCACCTAGTGGTCATG

5 CATGACCACTAGGAGCATCTTTGGCGACCACGGGCCAAGGTCCAGGGGGATGTTCGACTAGGGGAATAAATCTTTGGGCACCTAGTGGTCATG

5 CATGACCACTAGGAGCATCTTTGGCGAGGCGGAGGGTCCGCCGGGGACCAGTTGCTGCTAGGGGAATAAATCTTTGGGCACCTAGTGGTCATG

5 CATGACCACTAGGAGCATCTTTGGCGACGAGTCCTGGACCTGTCCCCGAGGGGAGATCTAGGGGAATAAATCTTTGGGCACCTAGTGGTCATG

5 CATGACCACTAGGAGCATCTTTGGCGACATCGATTGCCGTACTGCAAGCGTTGTGGACTAGGGGAATAAATCTTTGGGCACCTAGTGGTCATG

5 CATGACCACTAGGAGCATCTTTGGCGACTTGCGGCTCCCGGCCACTCTAAGTCGGCGCTAGGGGAATAAATCTTTGGGCACCTAGTGGTCATG

5 CATGACCACTAGGAGCATCTTTGGCGACGTGTGGGCCGCACTATGTGTCGAAGTCGACTAGGGGAATAAATCTTTGGGCACCTAGTGGTCATG

5 CATGACCACTAGGAGCATCTTTGGCGACTAGGGGGGTAACGGCCGACAGGTAGTCGACTAGGGGAATAAATCTTTGGGCACCTAGTGGTCATG

5 CATGACCACTAGGAGCATCTTTGGCGAACTTGTGGGCTCGAGCGGCAACGCTTTCGACTAGGGGAATAAATCTTTGGGCACCTAGTGGTCATG

5 CATGACCACTAGGAGCATCTTTGGCGACATGGGCCGGAAGGATGGTATTTGTAGCCGCTAGGGGAATAAATCTTTGGGCACCTAGTGGTCATG

5 CATGACCACTAGGAGCATCTTTGGCGAGTCTGGGGGCGTGTCGTAAGAAGGACGAGACTAGGGGAATAAATCTTTGGGCACCTAGTGGTCATG

5 CATGACCACTAGGAGCATCTTTGGCGAGCATCGAATGGGGGTCAAGGGTGCGCGAGACTAGGGGAATAAATCTTTGGGCACCTAGTGGTCATG

5 CATGACCACTAGGAGCATCTTTGGCGAGATCGGTAGAATCGGTGGCATTGGTGTCTCCTAGGGGAATAAATCTTTGGGCACCTAGTGGTCATG

5 CATGACCACTAGGAGCATCTTTGGCGATCCGGGGGGCTGTGCAGCCGACGCAAAGGGCTAGGGGAATAAATCTTTGGGCACCTAGTGGTCATG

5 CATGACCACTAGGAGCATCTTTGGCGATAGTGACGGGAGTCGACCCCTGGTTGGGTCCTAGGGGAATAAATCTTTGGGCACCTAGTGGTCATG

5 CATGACCACTAGGAGCATCTTTGGCGACTCATGTGGTCCCGTACCACAGGTAGTGGACTAGGGGAATAAATCTTTGGGCACCTAGTGGTCATG

5 CATGACCACTAGGAGCATCTTTGGCGACCTAGTGCGTCACGTCAGACGCGAAGGAGGCTAGGGGAATAAATCTTTGGGCACCTAGTGGTCATG

5 CATGACCACTAGGAGCATCTTTGGCGACCGGTCAGAATGGCTGCAACCAAAGGGAGACTAGGGGAATAAATCTTTGGGCACCTAGTGGTCATG

5 CATGACCACTAGGAGCATCTTTGGCGAGGGTATCCGTCTGTGGACAGTGGTCGAGTACTAGGGGAATAAATCTTTGGGCACCTAGTGGTCATG

5 CATGACCACTAGGAGCATCTTTGGCGACGGCGTGGGGGAGAAATCCGGCGGTGACTGCTAGGGGAATAAATCTTTGGGCACCTAGTGGTCATG

4 CATGACCACTAGGAGCATCTTTGGCGAGGAGGCAAAGCGCAACCGGAGTTAAGTGAGCTAGGGGAATAAATCTTTGGGCACCTAGTGGTCATG

4 CATGACCACTAGGAGCATCTTTGGCGATTCAGTGGGCATGGTCGTTGGAAAATCGCTCTAGGGGAATAAATCTTTGGGCACCTAGTGGTCATG

4 CATGACCACTAGGAGCATCTTTGGCGAAGAGACGACGTTAGTAGGATGAACGAGCTCCTAGGGGAATAAATCTTTGGGCACCTAGTGGTCATG

4 CATGACCACTAGGAGCATCTTTGGCGAGTGGGCACCATGTGCCAGTGCGTGAGCCGCCTAGGGGAATAAATCTTTGGGCACCTAGTGGTCATG

4 CATGACCACTAGGAGCATCTTTGGCGAAATATTGTCTAAAATACATAGGTAAATTCGCTAGGGGAATAAATCTTTGGGCACCTAGTGGTCATG

4 CATGACCACTAGGAGCATCTTTGGCGAGCAGGGACGCCGAATGAGGCCGTAGAGAGACTAGGGGAATAAATCTTTGGGCACCTAGTGGTCATG

4 CATGACCACTAGGAGCATCTTTGGCGAACGGTGGGCTGCTGGGGTGAACCGGACGCTCTAGGGGAATAAATCTTTGGGCACCTAGTGGTCATG

4 CATGACCACTAGGAGCATCTTTGGCGAAGGTCGGGCGGGTGTCGTTGACCGTTGCCGCTAGGGGAATAAATCTTTGGGCACCTAGTGGTCATG

4 CATGACCACTAGGAGCATCTTTGGCGACGACGGGGGCACTCAGGGTACAAATGTCGACTAGGGGAATAAATCTTTGGGCACCTAGTGGTCATG

4 CATGACCACTAGGAGCATCTTTGGCGACGAGTGGGCTCCGAGGAAACCGGCAGTCGACTAGGGGAATAAATCTTTGGGCACCTAGTGGTCATG

4 CATGACCACTAGGAGCATCTTTGGCGAGGGCATGGGGTGCGGTGACAGGTTCGCCATCTAGGGGAATAAATCTTTGGGCACCTAGTGGTCATG

4 CATGACCACTAGGAGCATCTTTGGCGACTAGAGATGGTCACGATGGACTGCGCTCGCCTAGGGGAATAAATCTTTGGGCACCTAGTGGTCATG

4 CATGACCACTAGGAGCATCTTTGGCGACGTAGGTGGGCAGTCTAAGGTTCCGTCGCTCTAGGGGAATAAATCTTTGGGCACCTAGTGGTCATG

4 CATGACCACTAGGAGCATCTTTGGCGACCGTGGATATTGTCATTGCAGATCGGGGCGCTAGGGGAATAAATCTTTGGGCACCTAGTGGTCATG

4 CATGACCACTAGGAGCATCTTTGGCGACTAGGGGGCACCGCGACCCGGAAGGCGCATCTAGGGGAATAAATCTTTGGGCACCTAGTGGTCATG

4 CATGACCACTAGGAGCATCTTTGGCGACGGTGTGGGGTCTTTCAATGCGGCGGTATACTAGGGGAATAAATCTTTGGGCACCTAGTGGTCATG

4 CATGACCACTAGGAGCATCTTTGGCGATACGGTCGTCGGATTTTCTAAGACATCGCCCTAGGGGAATAAATCTTTGGGCACCTAGTGGTCATG

4 CATGACCACTAGGAGCATCTTTGGCGAAGGGGCGCCGGTGGGTCGAGCACGTGTCGCCTAGGGGAATAAATCTTTGGGCACCTAGTGGTCATG

4 CATGACCACTAGGAGCATCTTTGGCGAGGAGGTGTCTATTAGTTGGTCGAACGAGTTCTAGGGGAATAAATCTTTGGGCACCTAGTGGTCATG

4 CATGACCACTAGGAGCATCTTTGGCGACGTAAGGTCAGGAACAGAGGAAGGAGTCGACTAGGGGAATAAATCTTTGGGCACCTAGTGGTCATG

4 CATGACCACTAGGAGCATCTTTGGCGAGTACGACCGTCACAGGAAGTATGCTCATAGCTAGGGGAATAAATCTTTGGGCACCTAGTGGTCATG

4 CATGACCACTAGGAGCATCTTTGGCGAGTGGTCACGTTGGGCGCATGGCGGTCCGAGCTAGGGGAATAAATCTTTGGGCACCTAGTGGTCATG

4 CATGACCACTAGGAGCATCTTTGGCGATCAGTGGGCATGTGGGCACTAAAGGAGAGACTAGGGGAATAAATCTTTGGGCACCTAGTGGTCATG

4 CATGACCACTAGGAGCATCTTTGGCGACAGTATCGGGGGGAGTGAGAGTACTGCTCGCTAGGGGAATAAATCTTTGGGCACCTAGTGGTCATG

4 CATGACCACTAGGAGCATCTTTGGCGAGGTAGTGGGACATGGAGAAGAAGACCTCGACTAGGGGAATAAATCTTTGGGCACCTAGTGGTCATG

4 CATGACCACTAGGAGCATCTTTGGCGAGTCACCAGGGGTGTGAGTGTAGAGGATCGCCTAGGGGAATAAATCTTTGGGCACCTAGTGGTCATG

4 CATGACCACTAGGAGCATCTTTGGCGACCAGTTGCAAACGATTCAGTGGCCGAGCGCCTAGGGGAATAAATCTTTGGGCACCTAGTGGTCATG

4 CATGACCACTAGGAGCATCTTTGGCGAGCAGATCACCTGATCTCGTTACGCAAAATACTAGGGGAATAAATCTTTGGGCACCTAGTGGTCATG

4 CATGACCACTAGGAGCATCTTTGGCGACCATGGTGGAATACGTGTATATGTGGGCGACTAGGGGAATAAATCTTTGGGCACCTAGTGGTCATG

4 CATGACCACTAGGAGCATCTTTGGCGAACTAGGCCGGGCCCCCCGCGACAGGTCGCTCTAGGGGAATAAATCTTTGGGCACCTAGTGGTCATG

4 CATGACCACTAGGAGCATCTTTGGCGAGAGGAGGCACGTTATTAGCGGAAGGCTTTACTAGGGGAATAAATCTTTGGGCACCTAGTGGTCATG

4 CATGACCACTAGGAGCATCTTTGGCGACCAGCGTTACGTGAGTGAATGATGGTGCTACTAGGGGAATAAATCTTTGGGCACCTAGTGGTCATG

4 CATGACCACTAGGAGCATCTTTGGCGAAAGGCAGGTGTTGCGGTGGTCTGTGAATCCCTAGGGGAATAAATCTTTGGGCACCTAGTGGTCATG

4 CATGACCACTAGGAGCATCTTTGGCGAGAGCAGAGCTCAAACACCATTAGAAAACGCCTAGGGGAATAAATCTTTGGGCACCTAGTGGTCATG

4 CATGACCACTAGGAGCATCTTTGGCGACGAAGGCACTGTAAGACTGGGCGGGGTCGTCTAGGGGAATAAATCTTTGGGCACCTAGTGGTCATG

4 CATGACCACTAGGAGCATCTTTGGCGATAGCGCTGGCACAAGGTCCTGCGGCTAAGACTAGGGGAATAAATCTTTGGGCACCTAGTGGTCATG

4 CATGACCACTAGGAGCATCTTTGGCGATGAGGGACGCTGAGCTAGAGTGCAAGTCGCCTAGGGGAATAAATCTTTGGGCACCTAGTGGTCATG

4 CATGACCACTAGGAGCATCTTTGGCGACTGGTCGTGCTCAACGTGGCAGAGTGAAGCCTAGGGGAATAAATCTTTGGGCACCTAGTGGTCATG

4 CATGACCACTAGGAGCATCTTTGGCGACTCGGAGTGCGTCAAGGGGGCGTAAGTCGACTAGGGGAATAAATCTTTGGGCACCTAGTGGTCATG

4 CATGACCACTAGGAGCATCTTTGGCGACCTCTTGGGGAGCGTGCTTTGGGTTCGACACTAGGGGAATAAATCTTTGGGCACCTAGTGGTCATG

4 CATGACCACTAGGAGCATCTTTGGCGACGTGGGCAGTTTAGAAGATCGCCTAGTACGCTAGGGGAATAAATCTTTGGGCACCTAGTGGTCATG

4 CATGACCACTAGGAGCATCTTTGGCGAACTGCGTACGTTGTTGGCTAACGGAGAAGCCTAGGGGAATAAATCTTTGGGCACCTAGTGGTCATG

4 CATGACCACTAGGAGCATCTTTGGCGATCGAATCGGGGGAAAGCAGGCAATGATCGACTAGGGGAATAAATCTTTGGGCACCTAGTGGTCATG

4 CATGACCACTAGGAGCATCTTTGGCGACGAACCGTGGTCGTGGGGCTACGTTCAAGACTAGGGGAATAAATCTTTGGGCACCTAGTGGTCATG

4 CATGACCACTAGGAGCATCTTTGGCGAACATGATCGGGGCAACGCCGCGAGTTTGTCCTAGGGGAATAAATCTTTGGGCACCTAGTGGTCATG

4 CATGACCACTAGGAGCATCTTTGGCGAGCAGCCTGGATAACTTTGAAATGGGTCGAGCTAGGGGAATAAATCTTTGGGCACCTAGTGGTCATG

4 CATGACCACTAGGAGCATCTTTGGCGATGTAGTATATTACGGAGTACTGCTCTATCGCTAGGGGAATAAATCTTTGGGCACCTAGTGGTCATG

4 CATGACCACTAGGAGCATCTTTGGCGAGTCCGGAAGAAAACGAGGGTCGGGACGCGACTAGGGGAATAAATCTTTGGGCACCTAGTGGTCATG

4 CATGACCACTAGGAGCATCTTTGGCGAAGGGCACGGCTAGGGTGGTAATTGTGGCGACTAGGGGAATAAATCTTTGGGCACCTAGTGGTCATG

4 CATGACCACTAGGAGCATCTTTGGCGAGCTCGCCGTAAGGCGGACGAAGTGGAGCTCCTAGGGGAATAAATCTTTGGGCACCTAGTGGTCATG

4 CATGACCACTAGGAGCATCTTTGGCGAGACGCACGGGAGTGGCGGATGATTCGAGCACTAGGGGAATAAATCTTTGGGCACCTAGTGGTCATG

4 CATGACCACTAGGAGCATCTTTGGCGATCATAACGGGTGACCTGGAAATTGATCGGTCTAGGGGAATAAATCTTTGGGCACCTAGTGGTCATG

4 CATGACCACTAGGAGCATCTTTGGCGACCTTGTGGGCACAACCAGTTGGCAAGGAGACTAGGGGAATAAATCTTTGGGCACCTAGTGGTCATG

4 CATGACCACTAGGAGCATCTTTGGCGACTGGGGTCTAGGTATCGGTGTCCAGGCTGACTAGGGGAATAAATCTTTGGGCACCTAGTGGTCATG

4 CATGACCACTAGGAGCATCTTTGGCGACGAACAGACGGCACTGGTTCTGAGCTCGCTCTAGGGGAATAAATCTTTGGGCACCTAGTGGTCATG

4 CATGACCACTAGGAGCATCTTTGGCGAACACGTAGTTTCACGTCTCGAGGATGGCGCCTAGGGGAATAAATCTTTGGGCACCTAGTGGTCATG

4 CATGACCACTAGGAGCATCTTTGGCGACAGGGCACGTGCATAGTCACGGAGTAGCGACTAGGGGAATAAATCTTTGGGCACCTAGTGGTCATG

4 CATGACCACTAGGAGCATCTTTGGCGACCGGACGATGGTTCGTACGTCCTGTAATGCCTAGGGGAATAAATCTTTGGGCACCTAGTGGTCATG

4 CATGACCACTAGGAGCATCTTTGGCGACAGTGGTCTGAGCGGCAACCGTAACGTCGACTAGGGGAATAAATCTTTGGGCACCTAGTGGTCATG

4 CATGACCACTAGGAGCATCTTTGGCGACAAGCGATCACCTGGTGACGTCTGGAGCTACTAGGGGAATAAATCTTTGGGCACCTAGTGGTCATG

4 CATGACCACTAGGAGCATCTTTGGCGAACATAAGGGGCCCTGGGCCAGGTTGGTAGCCTAGGGGAATAAATCTTTGGGCACCTAGTGGTCATG

4 CATGACCACTAGGAGCATCTTTGGCGACGCATGATGATAACGGAATCTATGTCTACGCTAGGGGAATAAATCTTTGGGCACCTAGTGGTCATG

4 CATGACCACTAGGAGCATCTTTGGCGATGTGGATAGGGGTCGTCCCTGCCTTATCGACTAGGGGAATAAATCTTTGGGCACCTAGTGGTCATG

4 CATGACCACTAGGAGCATCTTTGGCGACGGCGTGGGGGTCGAATCCGGCGGTGATCTCTAGGGGAATAAATCTTTGGGCACCTAGTGGTCATG

4 CATGACCACTAGGAGCATCTTTGGCGACTCCCGCGTCATGTAGAGGCGAGTGTGTAGCTAGGGGAATAAATCTTTGGGCACCTAGTGGTCATG

4 CATGACCACTAGGAGCATCTTTGGCGACAGAGGCCTGGGGGAAATGGATTCAGTCGCCTAGGGGAATAAATCTTTGGGCACCTAGTGGTCATG

4 CATGACCACTAGGAGCATCTTTGGCGAGCTCGGGACACATGTATGGAACGGAAGAGACTAGGGGAATAAATCTTTGGGCACCTAGTGGTCATG

4 CATGACCACTAGGAGCATCTTTGGCGAGTCGCAGGTCGTATCAAGACTCAAGGTAGCCTAGGGGAATAAATCTTTGGGCACCTAGTGGTCATG

4 CATGACCACTAGGAGCATCTTTGGCGACCGGATGGGGTCATGATGAAGATAAGGCGCCTAGGGGAATAAATCTTTGGGCACCTAGTGGTCATG

4 CATGACCACTAGGAGCATCTTTGGCGAAGGTGTACACCGGGCGAACATAGCCTGAGCCTAGGGGAATAAATCTTTGGGCACCTAGTGGTCATG

4 CATGACCACTAGGAGCATCTTTGGCGAAGCGCATAGGCACAGTGGCTGCGTGGACTACTAGGGGAATAAATCTTTGGGCACCTAGTGGTCATG

4 CATGACCACTAGGAGCATCTTTGGCGATAAGGGTGGAAGGCAATGCTTCCAATCGAGCTAGGGGAATAAATCTTTGGGCACCTAGTGGTCATG

4 CATGACCACTAGGAGCATCTTTGGCGAATGTGGTCCTGGTTGCTGTGGCAGAAGTCGCTAGGGGAATAAATCTTTGGGCACCTAGTGGTCATG

4 CATGACCACTAGGAGCATCTTTGGCGATTACCCAGGGGGCACCTGGACAATCGACAGCTAGGGGAATAAATCTTTGGGCACCTAGTGGTCATG

4 CATGACCACTAGGAGCATCTTTGGCGATTCGAGAGTCCGATCCTCGTGAGCGTGATACTAGGGGAATAAATCTTTGGGCACCTAGTGGTCATG

4 CATGACCACTAGGAGCATCTTTGGCGAGGGTGGACGCGCACCGTATCGCTCCTCGCACTAGGGGAATAAATCTTTGGGCACCTAGTGGTCATG

4 CATGACCACTAGGAGCATCTTTGGCGAGGGCCATTTGTGTCAAGTGGACGGGCGCTCCTAGGGGAATAAATCTTTGGGCACCTAGTGGTCATG

4 CATGACCACTAGGAGCATCTTTGGCGACTAACCTAGTGGGCTGGACAGGATAGACGCCTAGGGGAATAAATCTTTGGGCACCTAGTGGTCATG

4 CATGACCACTAGGAGCATCTTTGGCGACCCGGCAACGGCGTGGCGTATAGGGGCAGACTAGGGGAATAAATCTTTGGGCACCTAGTGGTCATG

4 CATGACCACTAGGAGCATCTTTGGCGAGATGGTACATCACACATAGAAATTGCGAGCCTAGGGGAATAAATCTTTGGGCACCTAGTGGTCATG

4 CATGACCACTAGGAGCATCTTTGGCGACGAGGGGGCATAGCATTCGGGTAAGTCGCTCTAGGGGAATAAATCTTTGGGCACCTAGTGGTCATG

4 CATGACCACTAGGAGCATCTTTGGCGAACGAGTATGTGTGCCTGGGTCGAGGTGCGACTAGGGGAATAAATCTTTGGGCACCTAGTGGTCATG

4 CATGACCACTAGGAGCATCTTTGGCGAGAGCGACCGCGAATCTCCTGCGCTCTGGATCTAGGGGAATAAATCTTTGGGCACCTAGTGGTCATG

4 CATGACCACTAGGAGCATCTTTGGCGACTGAAGGCAACGTCCTATGGATTACATAGCCTAGGGGAATAAATCTTTGGGCACCTAGTGGTCATG

4 CATGACCACTAGGAGCATCTTTGGCGACTGAACACGGGGCAACGTGCTAAAGGGCGACTAGGGGAATAAATCTTTGGGCACCTAGTGGTCATG

4 CATGACCACTAGGAGCATCTTTGGCGAGTTTCGCTCCGTCATACGGGTGTGACTCGGCTAGGGGAATAAATCTTTGGGCACCTAGTGGTCATG

4 CATGACCACTAGGAGCATCTTTGGCGACTGGGGCACCGTTGCAGAACAGGGAGGCGACTAGGGGAATAAATCTTTGGGCACCTAGTGGTCATG

4 CATGACCACTAGGAGCATCTTTGGCGACTAGTTACAGAGGTGAATCTCGGGAGCCAGCTAGGGGAATAAATCTTTGGGCACCTAGTGGTCATG

4 CATGACCACTAGGAGCATCTTTGGCGACGGTTGTGGCAACAGTGAGAAGGGTGTCGACTAGGGGAATAAATCTTTGGGCACCTAGTGGTCATG

4 CATGACCACTAGGAGCATCTTTGGCGACCGGCAATTTGACGACTCAGTCAGGTCGAACTAGGGGAATAAATCTTTGGGCACCTAGTGGTCATG

4 CATGACCACTAGGAGCATCTTTGGCGATCCGAGACGGGTTCACTGCCTGGTTGCCGCCTAGGGGAATAAATCTTTGGGCACCTAGTGGTCATG

4 CATGACCACTAGGAGCATCTTTGGCGAGAAGGCTCTGGATTCGGGGACCAGTTGCTGCTAGGGGAATAAATCTTTGGGCACCTAGTGGTCATG

4 CATGACCACTAGGAGCATCTTTGGCGACGGGGAAGGATACAGTCCGTAAAGCGGCGCCTAGGGGAATAAATCTTTGGGCACCTAGTGGTCATG

4 CATGACCACTAGGAGCATCTTTGGCGAAGCGTAGTTGCGACAGTGGCCGATAGCAAGCTAGGGGAATAAATCTTTGGGCACCTAGTGGTCATG

4 CATGACCACTAGGAGCATCTTTGGCGATCACACTAGGTATGACCTTGATTCAATCGCCTAGGGGAATAAATCTTTGGGCACCTAGTGGTCATG

4 CATGACCACTAGGAGCATCTTTGGCGAGTGCGGGCCTTGCAACAGTAGCCTCGAGATCTAGGGGAATAAATCTTTGGGCACCTAGTGGTCATG

4 CATGACCACTAGGAGCATCTTTGGCGAGTTGTCGGAGAGCCAGGTGTCAAACCTCAGCTAGGGGAATAAATCTTTGGGCACCTAGTGGTCATG

4 CATGACCACTAGGAGCATCTTTGGCGATGGGGCCGTGGCATCGCTACACTGCGCGGCCTAGGGGAATAAATCTTTGGGCACCTAGTGGTCATG

4 CATGACCACTAGGAGCATCTTTGGCGAAGTGCGGGATGGCCCGCATGCGGTAGCAGCCTAGGGGAATAAATCTTTGGGCACCTAGTGGTCATG

4 CATGACCACTAGGAGCATCTTTGGCGAGGATCGAGACACGACCCGTGTTGGGGGTCGCTAGGGGAATAAATCTTTGGGCACCTAGTGGTCATG

4 CATGACCACTAGGAGCATCTTTGGCGACGACAGTCAGCGTGATCGTTCGCGGGCTGCCTAGGGGAATAAATCTTTGGGCACCTAGTGGTCATG

4 CATGACCACTAGGAGCATCTTTGGCGAACACGGGCGGTCACGCGAGGGGTTTGCACGCTAGGGGAATAAATCTTTGGGCACCTAGTGGTCATG

4 CATGACCACTAGGAGCATCTTTGGCGACCGGGTGGCAGAGAGCTCGATATACGGTCACTAGGGGAATAAATCTTTGGGCACCTAGTGGTCATG

4 CATGACCACTAGGAGCATCTTTGGCGACGCTCGGGGTCAGGCTAAAAGAAAGGTCGCCTAGGGGAATAAATCTTTGGGCACCTAGTGGTCATG

4 CATGACCACTAGGAGCATCTTTGGCGAGTCGGCACCTAAAGTACGCCATAGCCCTCGCTAGGGGAATAAATCTTTGGGCACCTAGTGGTCATG

4 CATGACCACTAGGAGCATCTTTGGCGAGGATGTTCGGAGGCGGTAGAGTGGTCGAGACTAGGGGAATAAATCTTTGGGCACCTAGTGGTCATG

4 CATGACCACTAGGAGCATCTTTGGCGATGTGGGCTTCGTAGGCTCAGGTGTACAGCGCTAGGGGAATAAATCTTTGGGCACCTAGTGGTCATG

4 CATGACCACTAGGAGCATCTTTGGCGATCCTATGGGTCCGTCGTTATATGGAATCGACTAGGGGAATAAATCTTTGGGCACCTAGTGGTCATG

4 CATGACCACTAGGAGCATCTTTGGCGACGGTTGGTAGTTCGATTGTGAACTCTGAGACTAGGGGAATAAATCTTTGGGCACCTAGTGGTCATG

4 CATGACCACTAGGAGCATCTTTGGCGACTCGTGAAGGGGTCATCCCTACTTTGTCGACTAGGGGAATAAATCTTTGGGCACCTAGTGGTCATG

4 CATGACCACTAGGAGCATCTTTGGCGAATAGTCTATACTGGGCTTCATTGCTATTGCCTAGGGGAATAAATCTTTGGGCACCTAGTGGTCATG

4 CATGACCACTAGGAGCATCTTTGGCGAGGACAATAGCTCCTGGAAGTCTTGCTCGCACTAGGGGAATAAATCTTTGGGCACCTAGTGGTCATG

4 CATGACCACTAGGAGCATCTTTGGCGACATACCGGAGTGGGCTTTCGGGCAGGTCGGCTAGGGGAATAAATCTTTGGGCACCTAGTGGTCATG

4 CATGACCACTAGGAGCATCTTTGGCGACAGTCTGCGCACAGGGCCACCGAAGACGAGCTAGGGGAATAAATCTTTGGGCACCTAGTGGTCATG

4 CATGACCACTAGGAGCATCTTTGGCGACGCGGGGTCATGGGTTGCAAGTAGAAGTCGCTAGGGGAATAAATCTTTGGGCACCTAGTGGTCATG

4 CATGACCACTAGGAGCATCTTTGGCGAGACGCCGTAATAGTGCGTCTTTGCTCGCGCCTAGGGGAATAAATCTTTGGGCACCTAGTGGTCATG

4 CATGACCACTAGGAGCATCTTTGGCGAACTATCATCAGCTGGGATTTGTTTGGTCGTCTAGGGGAATAAATCTTTGGGCACCTAGTGGTCATG

4 CATGACCACTAGGAGCATCTTTGGCGATCGAGGGCAACGGTGGGAAGCGAAGGTCGACTAGGGGAATAAATCTTTGGGCACCTAGTGGTCATG

4 CATGACCACTAGGAGCATCTTTGGCGAGTAGTGGGCTCGTGGCGGGTAACTAGTCGACTAGGGGAATAAATCTTTGGGCACCTAGTGGTCATG

4 CATGACCACTAGGAGCATCTTTGGCGACCGGGGTTGCACCTAGTCTCCAACGTCGCGCTAGGGGAATAAATCTTTGGGCACCTAGTGGTCATG

4 CATGACCACTAGGAGCATCTTTGGCGACACAGTACTCTAGCTGGGGGAGTCGTCGCACTAGGGGAATAAATCTTTGGGCACCTAGTGGTCATG

4 CATGACCACTAGGAGCATCTTTGGCGATCGAGCAGGGCCAGGTTGACATCGGATCGACTAGGGGAATAAATCTTTGGGCACCTAGTGGTCATG

4 CATGACCACTAGGAGCATCTTTGGCGACCAATGGGTCATGTAGGCTAAGGTAGTCGCCTAGGGGAATAAATCTTTGGGCACCTAGTGGTCATG

4 CATGACCACTAGGAGCATCTTTGGCGACGTAAATGCGCCCGACAGGGCGTTGATCGCCTAGGGGAATAAATCTTTGGGCACCTAGTGGTCATG

4 CATGACCACTAGGAGCATCTTTGGCGAGCCGTGATGGCTTATTGGGTATCGCTCGCACTAGGGGAATAAATCTTTGGGCACCTAGTGGTCATG

4 CATGACCACTAGGAGCATCTTTGGCGAGCTTAGGGTGGTCGGGTACACACTAAGGTCCTAGGGGAATAAATCTTTGGGCACCTAGTGGTCATG

4 CATGACCACTAGGAGCATCTTTGGCGACAGTGCGAGGTTGGTTCAGTGGTCTGCGATCTAGGGGAATAAATCTTTGGGCACCTAGTGGTCATG

4 CATGACCACTAGGAGCATCTTTGGCGACGTGGGCCGGACAAGCACATAGATAGGAGCCTAGGGGAATAAATCTTTGGGCACCTAGTGGTCATG

4 CATGACCACTAGGAGCATCTTTGGCGACTTGCGCGGGGTGCCTGAGCCGGATGTCGACTAGGGGAATAAATCTTTGGGCACCTAGTGGTCATG

4 CATGACCACTAGGAGCATCTTTGGCGACTAGCAAGGCGGAAGCAGCTCGATGGTCGACTAGGGGAATAAATCTTTGGGCACCTAGTGGTCATG

4 CATGACCACTAGGAGCATCTTTGGCGAGGAGGGCTTGCCGTGTGGCCCGCGTGGAGCCTAGGGGAATAAATCTTTGGGCACCTAGTGGTCATG

4 CATGACCACTAGGAGCATCTTTGGCGACGTGGTTCTAAAGGGGGAGGTTAGGGGTGGCTAGGGGAATAAATCTTTGGGCACCTAGTGGTCATG

4 CATGACCACTAGGAGCATCTTTGGCGACGGTGGTGTCAACTGATCCTGGCTAGCCAGCTAGGGGAATAAATCTTTGGGCACCTAGTGGTCATG

4 CATGACCACTAGGAGCATCTTTGGCGAGCTGTACTTGTTCGGCTGTAGAAAGTCGCACTAGGGGAATAAATCTTTGGGCACCTAGTGGTCATG

4 CATGACCACTAGGAGCATCTTTGGCGAGTCCGACGGGGGCCAGGATTCGACGAGCTTCTAGGGGAATAAATCTTTGGGCACCTAGTGGTCATG

4 CATGACCACTAGGAGCATCTTTGGCGAACGGGGTGGTTGCGGTGGCGCAGAGAGCTCCTAGGGGAATAAATCTTTGGGCACCTAGTGGTCATG

4 CATGACCACTAGGAGCATCTTTGGCGATGGGACGGACCACTCGCGTCACAAGAGCTTCTAGGGGAATAAATCTTTGGGCACCTAGTGGTCATG

4 CATGACCACTAGGAGCATCTTTGGCGAGTCTCGAATGAAACAAGAAGAGAGATAAGTCTAGGGGAATAAATCTTTGGGCACCTAGTGGTCATG

4 CATGACCACTAGGAGCATCTTTGGCGAGCGTCACAGACTGAGTATAGCACAGATCGACTAGGGGAATAAATCTTTGGGCACCTAGTGGTCATG

4 CATGACCACTAGGAGCATCTTTGGCGAATGATAAGGCGAGCCGGCGATCGTCGCCATCTAGGGGAATAAATCTTTGGGCACCTAGTGGTCATG

4 CATGACCACTAGGAGCATCTTTGGCGAGCGAAATGAGTGTACGGTCCGATGTCGCTCCTAGGGGAATAAATCTTTGGGCACCTAGTGGTCATG

4 CATGACCACTAGGAGCATCTTTGGCGACTGTGTGGGCTGTCCATCTGTGTAAGTCGACTAGGGGAATAAATCTTTGGGCACCTAGTGGTCATG

4 CATGACCACTAGGAGCATCTTTGGCGACTGTGGGATCGAGGTATGCCAGTGTGGAGACTAGGGGAATAAATCTTTGGGCACCTAGTGGTCATG

4 CATGACCACTAGGAGCATCTTTGGCGACTTTCACATATGCAAATGGCGAGCTGCTCACTAGGGGAATAAATCTTTGGGCACCTAGTGGTCATG

4 CATGACCACTAGGAGCATCTTTGGCGAGAGATCGCTGTGAACCAGAGGGTTCAGTAACTAGGGGAATAAATCTTTGGGCACCTAGTGGTCATG

4 CATGACCACTAGGAGCATCTTTGGCGACGAAATTGGCATGTCACCGGCGTCAGTCGCCTAGGGGAATAAATCTTTGGGCACCTAGTGGTCATG

4 CATGACCACTAGGAGCATCTTTGGCGATTCTGCTGGTAGGTCATGTATGAGGTAGCTCTAGGGGAATAAATCTTTGGGCACCTAGTGGTCATG

4 CATGACCACTAGGAGCATCTTTGGCGAGAATAGTAAAGCAGCTTGGACACAGAGCCTCTAGGGGAATAAATCTTTGGGCACCTAGTGGTCATG

4 CATGACCACTAGGAGCATCTTTGGCGAACGTAGAGCGTTAGGTGTGCTCACCGTGAACTAGGGGAATAAATCTTTGGGCACCTAGTGGTCATG

4 CATGACCACTAGGAGCATCTTTGGCGAACTAGCACGGTATCGAGAGACTAGGCTCGACTAGGGGAATAAATCTTTGGGCACCTAGTGGTCATG

4 CATGACCACTAGGAGCATCTTTGGCGAACTCGGCGGAGAGTCTCGCGGTAAAATCGCCTAGGGGAATAAATCTTTGGGCACCTAGTGGTCATG

4 CATGACCACTAGGAGCATCTTTGGCGACGTGGTGGGCATTGCTGGATTATGAGGAGCCTAGGGGAATAAATCTTTGGGCACCTAGTGGTCATG

4 CATGACCACTAGGAGCATCTTTGGCGATCGTACAGTGAAGGCCAGCCGTGAAGATTGCTAGGGGAATAAATCTTTGGGCACCTAGTGGTCATG

4 CATGACCACTAGGAGCATCTTTGGCGAACTGGGGGGTCATGCAGTTGACCGGTGCGACTAGGGGAATAAATCTTTGGGCACCTAGTGGTCATG

4 CATGACCACTAGGAGCATCTTTGGCGAACTGTGAATGCTCACGCAAGATGTTTGAGACTAGGGGAATAAATCTTTGGGCACCTAGTGGTCATG

4 CATGACCACTAGGAGCATCTTTGGCGACGTGAGTGGGAAGAATCAAGGGGTCGTCGACTAGGGGAATAAATCTTTGGGCACCTAGTGGTCATG

4 CATGACCACTAGGAGCATCTTTGGCGACAGTCTGGGTTGGTCGCATCGGTTTAACCGCTAGGGGAATAAATCTTTGGGCACCTAGTGGTCATG

4 CATGACCACTAGGAGCATCTTTGGCGACGAGTGGCACGCAAACATAATGCACAGAGCCTAGGGGAATAAATCTTTGGGCACCTAGTGGTCATG

4 CATGACCACTAGGAGCATCTTTGGCGAACGGCAGGTGTTGAGGTGGTCTGTGAATCCCTAGGGGAATAAATCTTTGGGCACCTAGTGGTCATG

4 CATGACCACTAGGAGCATCTTTGGCGACGTCGGAGGTGCGAAGTTCCGACGAGTGATCTAGGGGAATAAATCTTTGGGCACCTAGTGGTCATG

4 CATGACCACTAGGAGCATCTTTGGCGACGTGGGTGGCTTGGGCACTCACAAATCCGCCTAGGGGAATAAATCTTTGGGCACCTAGTGGTCATG

4 CATGACCACTAGGAGCATCTTTGGCGAGTGGGCCTCGAACATCTTCGTGCAGGTCGCCTAGGGGAATAAATCTTTGGGCACCTAGTGGTCATG

4 CATGACCACTAGGAGCATCTTTGGCGACTGGTGGCGGTTCCACTGTGCCTCCGACGACTAGGGGAATAAATCTTTGGGCACCTAGTGGTCATG

4 CATGACCACTAGGAGCATCTTTGGCGAATAAACAAGGGAGATTAGCATATAATGAGCCTAGGGGAATAAATCTTTGGGCACCTAGTGGTCATG

4 CATGACCACTAGGAGCATCTTTGGCGACGTGGTCTCGTTAGAACTCTGAGAGAGGAGCTAGGGGAATAAATCTTTGGGCACCTAGTGGTCATG

4 CATGACCACTAGGAGCATCTTTGGCGATCTTACCTGGAAATGGCGATGTAGAGGCGACTAGGGGAATAAATCTTTGGGCACCTAGTGGTCATG

4 CATGACCACTAGGAGCATCTTTGGCGACGACGTGGGCGTCGGAAACGTAAAAGGCGCCTAGGGGAATAAATCTTTGGGCACCTAGTGGTCATG

4 CATGACCACTAGGAGCATCTTTGGCGACGACCGACGCAAGCTCAGCGATGGGTCGCTCTAGGGGAATAAATCTTTGGGCACCTAGTGGTCATG

4 CATGACCACTAGGAGCATCTTTGGCGACCCAGCGTAGAATGCGCCTGTGGGTGGGTCCTAGGGGAATAAATCTTTGGGCACCTAGTGGTCATG

4 CATGACCACTAGGAGCATCTTTGGCGAGATCGGGAGAATCGGTGGCATTGGTGACTCCTAGGGGAATAAATCTTTGGGCACCTAGTGGTCATG

4 CATGACCACTAGGAGCATCTTTGGCGACGAACATTTGTCCTTCGGATGGGAGTCGGTCTAGGGGAATAAATCTTTGGGCACCTAGTGGTCATG

4 CATGACCACTAGGAGCATCTTTGGCGACTCTAGTGGTCCAAAGACAAAACGAGGTCGCTAGGGGAATAAATCTTTGGGCACCTAGTGGTCATG

4 CATGACCACTAGGAGCATCTTTGGCGAGTGTAGCGGACTTTGATACCGGCTAAGCTCCTAGGGGAATAAATCTTTGGGCACCTAGTGGTCATG

4 CATGACCACTAGGAGCATCTTTGGCGACGGTGTGGGGGCGTCAGCCCGGCGGTCATACTAGGGGAATAAATCTTTGGGCACCTAGTGGTCATG

4 CATGACCACTAGGAGCATCTTTGGCGACCCGGTGGTCATTGCACAGTGGGGTGGCGCCTAGGGGAATAAATCTTTGGGCACCTAGTGGTCATG

4 CATGACCACTAGGAGCATCTTTGGCGAGGGCCTAGGCAGTACGGAAGGGGTCTCGCACTAGGGGAATAAATCTTTGGGCACCTAGTGGTCATG

4 CATGACCACTAGGAGCATCTTTGGCGACGGATCTCCACAGTCATGTGGCTCAGCCAGCTAGGGGAATAAATCTTTGGGCACCTAGTGGTCATG

4 CATGACCACTAGGAGCATCTTTGGCGATCGGCGCAGGATGGGCTAGACTGAGATCGACTAGGGGAATAAATCTTTGGGCACCTAGTGGTCATG

4 CATGACCACTAGGAGCATCTTTGGCGACATGTCTACTGACGCAGTGGAGAGTCGCAACTAGGGGAATAAATCTTTGGGCACCTAGTGGTCATG

4 CATGACCACTAGGAGCATCTTTGGCGACGGAGACTGTCGTTAGGGGGCTCCCGACGGCTAGGGGAATAAATCTTTGGGCACCTAGTGGTCATG

4 CATGACCACTAGGAGCATCTTTGGCGAATTCGCCATCGGCTTCATAAGCAAGCGCCACTAGGGGAATAAATCTTTGGGCACCTAGTGGTCATG

4 CATGACCACTAGGAGCATCTTTGGCGATATGGTCATAGCAGGCAGTCCGACTACGAGCTAGGGGAATAAATCTTTGGGCACCTAGTGGTCATG

4 CATGACCACTAGGAGCATCTTTGGCGACAGGGGGCGCTAGTTTAGCCGTGCAAACTACTAGGGGAATAAATCTTTGGGCACCTAGTGGTCATG

4 CATGACCACTAGGAGCATCTTTGGCGAGCACGGGAGGGCCTCTTCAAAAAGTGCCGACTAGGGGAATAAATCTTTGGGCACCTAGTGGTCATG

4 CATGACCACTAGGAGCATCTTTGGCGAGACAAGTCGTCGTATTCGCGCCGGGGTCGCCTAGGGGAATAAATCTTTGGGCACCTAGTGGTCATG

4 CATGACCACTAGGAGCATCTTTGGCGACGTAGGGGGCATTGGGATTCAGAGAGTCGACTAGGGGAATAAATCTTTGGGCACCTAGTGGTCATG

4 CATGACCACTAGGAGCATCTTTGGCGACAGAGTGGGCATTGTGGTAGAGTGTCGCTGCTAGGGGAATAAATCTTTGGGCACCTAGTGGTCATG

4 CATGACCACTAGGAGCATCTTTGGCGAAGTCATAGCCGAAGGCGGAGCCTGCACTTCCTAGGGGAATAAATCTTTGGGCACCTAGTGGTCATG

4 CATGACCACTAGGAGCATCTTTGGCGAAGTACTGCATCGTAAGGGCGGTTCTGCGGCCTAGGGGAATAAATCTTTGGGCACCTAGTGGTCATG

4 CATGACCACTAGGAGCATCTTTGGCGATGTGGGTAGAACCACCCCATACGGTGTCAGCTAGGGGAATAAATCTTTGGGCACCTAGTGGTCATG

4 CATGACCACTAGGAGCATCTTTGGCGAGGCTCGTTCGGCTAAAGTGACTGGGAGCCGCTAGGGGAATAAATCTTTGGGCACCTAGTGGTCATG

4 CATGACCACTAGGAGCATCTTTGGCGAGAGCACGTTCGGCTCAGAACTGGCGAAAGACTAGGGGAATAAATCTTTGGGCACCTAGTGGTCATG

4 CATGACCACTAGGAGCATCTTTGGCGAGGGCCGGGATGTCGTGCGGCAAAGGACGAGCTAGGGGAATAAATCTTTGGGCACCTAGTGGTCATG

4 CATGACCACTAGGAGCATCTTTGGCGAGATCGGGAGAATCGGTGGCAATGGTGTCTCCTAGGGGAATAAATCTTTGGGCACCTAGTGGTCATG

4 CATGACCACTAGGAGCATCTTTGGCGAACTAGGGTCTTGCGTCACCGGCTCGTTTCGCTAGGGGAATAAATCTTTGGGCACCTAGTGGTCATG

4 CATGACCACTAGGAGCATCTTTGGCGACTCGGTCATGGATTACTGAGCTTAGTCGGCCTAGGGGAATAAATCTTTGGGCACCTAGTGGTCATG

4 CATGACCACTAGGAGCATCTTTGGCGACCAGGCTTAGCGCTCAGAGCGCAGGACGCACTAGGGGAATAAATCTTTGGGCACCTAGTGGTCATG

4 CATGACCACTAGGAGCATCTTTGGCGAGTGGTCCTGTTCAAACCGGAGGACGTTGGGCTAGGGGAATAAATCTTTGGGCACCTAGTGGTCATG

4 CATGACCACTAGGAGCATCTTTGGCGATAGCATAAGTGCACCTGATGTCCTTCGCCACTAGGGGAATAAATCTTTGGGCACCTAGTGGTCATG

4 CATGACCACTAGGAGCATCTTTGGCGACTCAAATCCCCGTCGGGGAGGTAGCGGAGACTAGGGGAATAAATCTTTGGGCACCTAGTGGTCATG

4 CATGACCACTAGGAGCATCTTTGGCGATCACCGAATGCACTGCAGGCGGCGTGGAAGCTAGGGGAATAAATCTTTGGGCACCTAGTGGTCATG

4 CATGACCACTAGGAGCATCTTTGGCGAGGACCGTGCGGCTAGGCCAGCGCTAATTCGCTAGGGGAATAAATCTTTGGGCACCTAGTGGTCATG

4 CATGACCACTAGGAGCATCTTTGGCGAGGTATGGACGTGGCGAAACGTACAACCCGACTAGGGGAATAAATCTTTGGGCACCTAGTGGTCATG

4 CATGACCACTAGGAGCATCTTTGGCGATCTGGTGGGCACTGGAATATAGGAGAGCGCCTAGGGGAATAAATCTTTGGGCACCTAGTGGTCATG

4 CATGACCACTAGGAGCATCTTTGGCGACTGTGGGCTCGTGGATCTAGGGTGACGGAGCTAGGGGAATAAATCTTTGGGCACCTAGTGGTCATG

4 CATGACCACTAGGAGCATCTTTGGCGACGAGGATCGTGCTCGTTGGACACAGGAGCCCTAGGGGAATAAATCTTTGGGCACCTAGTGGTCATG

4 CATGACCACTAGGAGCATCTTTGGCGACGTTGGCACAGCAAGTGTGTTCGTAGCCTTCTAGGGGAATAAATCTTTGGGCACCTAGTGGTCATG

4 CATGACCACTAGGAGCATCTTTGGCGATTCCGGAGAGGTAATCGGCCGGGGCGCCTCCTAGGGGAATAAATCTTTGGGCACCTAGTGGTCATG

4 CATGACCACTAGGAGCATCTTTGGCGAGCTCATAATCTTTAGAAGGTGCGCGCTATGCTAGGGGAATAAATCTTTGGGCACCTAGTGGTCATG

4 CATGACCACTAGGAGCATCTTTGGCGAGCGTCAATGATCGATAGTGGTTAGCTAGCTCTAGGGGAATAAATCTTTGGGCACCTAGTGGTCATG

4 CATGACCACTAGGAGCATCTTTGGCGAAAGAGCCGACGCTAGGAGCTAGTGTAACTACTAGGGGAATAAATCTTTGGGCACCTAGTGGTCATG

4 CATGACCACTAGGAGCATCTTTGGCGAGGGACGCTCGATCCGGTCGTCAAAAACAGCCTAGGGGAATAAATCTTTGGGCACCTAGTGGTCATG

4 CATGACCACTAGGAGCATCTTTGGCGACGACCGAGTAAACAAAGCTACGACTGGAGACTAGGGGAATAAATCTTTGGGCACCTAGTGGTCATG

4 CATGACCACTAGGAGCATCTTTGGCGAGACATTATGAAATCAGAGGTATTATATCGCCTAGGGGAATAAATCTTTGGGCACCTAGTGGTCATG

4 CATGACCACTAGGAGCATCTTTGGCGAGTAGCGAGGGTACGGGGTGGAGCGACTCACCTAGGGGAATAAATCTTTGGGCACCTAGTGGTCATG

4 CATGACCACTAGGAGCATCTTTGGCGAGTGGATCTAAGTGAGCGTGAAGATACTCGACTAGGGGAATAAATCTTTGGGCACCTAGTGGTCATG

4 CATGACCACTAGGAGCATCTTTGGCGAACTTCATTGAGAGGAAGGATCTTCAACTAACTAGGGGAATAAATCTTTGGGCACCTAGTGGTCATG

4 CATGACCACTAGGAGCATCTTTGGCGAGGTTCGCGGACTCGTCGGTGCGCAACTAGACTAGGGGAATAAATCTTTGGGCACCTAGTGGTCATG

4 CATGACCACTAGGAGCATCTTTGGCGATACGACGGTTGTGTGTTAACTGCCGATCGACTAGGGGAATAAATCTTTGGGCACCTAGTGGTCATG

4 CATGACCACTAGGAGCATCTTTGGCGAACGACCGTTATAACGAAGCGTGCAGCGCATCTAGGGGAATAAATCTTTGGGCACCTAGTGGTCATG

4 CATGACCACTAGGAGCATCTTTGGCGACGTCGTGGGTGGCAAGGGGTATCAGTTCGCCTAGGGGAATAAATCTTTGGGCACCTAGTGGTCATG

4 CATGACCACTAGGAGCATCTTTGGCGACGTCAGGGATGTAAGGTACTAGTGGTCGCTCTAGGGGAATAAATCTTTGGGCACCTAGTGGTCATG

4 CATGACCACTAGGAGCATCTTTGGCGAGCATCAGGATAGAGTTTACGGAGATGCGACCTAGGGGAATAAATCTTTGGGCACCTAGTGGTCATG

4 CATGACCACTAGGAGCATCTTTGGCGACCGGTCAGTCCGTTGGAGTCTCGAGTCGCACTAGGGGAATAAATCTTTGGGCACCTAGTGGTCATG

4 CATGACCACTAGGAGCATCTTTGGCGAGGATCACGACCGACTGTTGTGAGACGGCCTCTAGGGGAATAAATCTTTGGGCACCTAGTGGTCATG

4 CATGACCACTAGGAGCATCTTTGGCGACTCCTGGGTGCTCGGATGGGTCAATGTCGACTAGGGGAATAAATCTTTGGGCACCTAGTGGTCATG

4 CATGACCACTAGGAGCATCTTTGGCGAAAATCGGAGAAAGGTAATAAGTATTTTTGCCTAGGGGAATAAATCTTTGGGCACCTAGTGGTCATG

4 CATGACCACTAGGAGCATCTTTGGCGAGAGGCATACGCGGGTACGAAAATGCCCTCACTAGGGGAATAAATCTTTGGGCACCTAGTGGTCATG

4 CATGACCACTAGGAGCATCTTTGGCGAAGAGAAGCGGGCCGATCGCTATATTTTCGACTAGGGGAATAAATCTTTGGGCACCTAGTGGTCATG

4 CATGACCACTAGGAGCATCTTTGGCGACATCGTCTCTGGGAGGGGTAACGTGTCGCTCTAGGGGAATAAATCTTTGGGCACCTAGTGGTCATG

4 CATGACCACTAGGAGCATCTTTGGCGAGGAACGGTTGTCACAAGAGCTTCAAACCAGCTAGGGGAATAAATCTTTGGGCACCTAGTGGTCATG

4 CATGACCACTAGGAGCATCTTTGGCGAGGTACGAAGCCGAGGCTGTGGCTGAGTTTACTAGGGGAATAAATCTTTGGGCACCTAGTGGTCATG

4 CATGACCACTAGGAGCATCTTTGGCGACTCGGTGGGCACCGGCAAACGTGAGGAGATCTAGGGGAATAAATCTTTGGGCACCTAGTGGTCATG

4 CATGACCACTAGGAGCATCTTTGGCGAGTCCCGTTGCTCATAACAAGATATACTCGACTAGGGGAATAAATCTTTGGGCACCTAGTGGTCATG

4 CATGACCACTAGGAGCATCTTTGGCGACCAGATATGGTTGGTAATGTGCTGAGGAGGCTAGGGGAATAAATCTTTGGGCACCTAGTGGTCATG

4 CATGACCACTAGGAGCATCTTTGGCGACTCATCGTGGCTGTAGGCGAGGTACGTCGTCTAGGGGAATAAATCTTTGGGCACCTAGTGGTCATG

4 CATGACCACTAGGAGCATCTTTGGCGACGCAAATGATCGTGAAGGGACTGCGGAGAGCTAGGGGAATAAATCTTTGGGCACCTAGTGGTCATG

4 CATGACCACTAGGAGCATCTTTGGCGACGGACATAGGCTATCAATCGGCGGTTGACACTAGGGGAATAAATCTTTGGGCACCTAGTGGTCATG

4 CATGACCACTAGGAGCATCTTTGGCGAGTCGCCTGTCGTCCGGCCAGGCGTTGACAACTAGGGGAATAAATCTTTGGGCACCTAGTGGTCATG

4 CATGACCACTAGGAGCATCTTTGGCGATAGACTTGTAATGGGCAGCATAGAATATCGCTAGGGGAATAAATCTTTGGGCACCTAGTGGTCATG

4 CATGACCACTAGGAGCATCTTTGGCGACGAGGGGGCATTGTAATACGGTAAAAGGAGCTAGGGGAATAAATCTTTGGGCACCTAGTGGTCATG

4 CATGACCACTAGGAGCATCTTTGGCGAGGAGATGGTTTAGACGCCAAGGGCGAGACACTAGGGGAATAAATCTTTGGGCACCTAGTGGTCATG

4 CATGACCACTAGGAGCATCTTTGGCGATAAGCATATCTATCGGAATAGTAATCGCTGCTAGGGGAATAAATCTTTGGGCACCTAGTGGTCATG

4 CATGACCACTAGGAGCATCTTTGGCGAGTGCACGCGGTGACCGGTGCATCTGACTGTCTAGGGGAATAAATCTTTGGGCACCTAGTGGTCATG

4 CATGACCACTAGGAGCATCTTTGGCGAGTGTCCGAGTGGCGAGGGTGTGCCTCGAGACTAGGGGAATAAATCTTTGGGCACCTAGTGGTCATG

4 CATGACCACTAGGAGCATCTTTGGCGAGATTGGGACACTATTGCTGTCGCGTGAGCCCTAGGGGAATAAATCTTTGGGCACCTAGTGGTCATG

4 CATGACCACTAGGAGCATCTTTGGCGAGGACCGTCCGGACCGTAAGTAGGGAGCAGCCTAGGGGAATAAATCTTTGGGCACCTAGTGGTCATG

4 CATGACCACTAGGAGCATCTTTGGCGACGAAGGCAGAGCTCCGGTGTCCGGCGGAGACTAGGGGAATAAATCTTTGGGCACCTAGTGGTCATG

4 CATGACCACTAGGAGCATCTTTGGCGAGGGGCGTGGACGCTGTAACCGGAGTGCTCGCTAGGGGAATAAATCTTTGGGCACCTAGTGGTCATG

4 CATGACCACTAGGAGCATCTTTGGCGACCTCGAGAGGGGTGTCTCCGTGGGCCGCATCTAGGGGAATAAATCTTTGGGCACCTAGTGGTCATG

4 CATGACCACTAGGAGCATCTTTGGCGACGATGTGGGCTAGGATGGAGAAGACGTCGACTAGGGGAATAAATCTTTGGGCACCTAGTGGTCATG

4 CATGACCACTAGGAGCATCTTTGGCGATAGACCGCGGCCCGCTCCGCGTGTGACTCGCTAGGGGAATAAATCTTTGGGCACCTAGTGGTCATG

4 CATGACCACTAGGAGCATCTTTGGCGAGGCACCGGGTGTGGCACGGTGGTTACGAGACTAGGGGAATAAATCTTTGGGCACCTAGTGGTCATG

4 CATGACCACTAGGAGCATCTTTGGCGATGTAAAGCTTGGCAGTATGGTAACATCTCGCTAGGGGAATAAATCTTTGGGCACCTAGTGGTCATG

4 CATGACCACTAGGAGCATCTTTGGCGACCCAGTGGGCTAATTGCATGAGGTGGTCGACTAGGGGAATAAATCTTTGGGCACCTAGTGGTCATG

4 CATGACCACTAGGAGCATCTTTGGCGAGTGTGGTCTTTGGCCTCGCCACTCAATCGACTAGGGGAATAAATCTTTGGGCACCTAGTGGTCATG

4 CATGACCACTAGGAGCATCTTTGGCGACGGCATTTGTTAGGCTCCGGACAGTGTCACCTAGGGGAATAAATCTTTGGGCACCTAGTGGTCATG

4 CATGACCACTAGGAGCATCTTTGGCGAACGGCAGGTGTTGCGGTGGTCTGTGAGTCCCTAGGGGAATAAATCTTTGGGCACCTAGTGGTCATG

4 CATGACCACTAGGAGCATCTTTGGCGACGGTGGTCCCGGAGAACCGGTATGAGGAGACTAGGGGAATAAATCTTTGGGCACCTAGTGGTCATG

4 CATGACCACTAGGAGCATCTTTGGCGATCGCCTACGCTATATGCAGGTGTGATCGCTCTAGGGGAATAAATCTTTGGGCACCTAGTGGTCATG

4 CATGACCACTAGGAGCATCTTTGGCGACTTAGTGGCTGAGAGGCCGAGAAACTTAGCCTAGGGGAATAAATCTTTGGGCACCTAGTGGTCATG

4 CATGACCACTAGGAGCATCTTTGGCGATCCGACGCAGTAGGTGTGGCCGCAGGCAGCCTAGGGGAATAAATCTTTGGGCACCTAGTGGTCATG

4 CATGACCACTAGGAGCATCTTTGGCGACGGGCGTGGTCGGTTCTGCCAACTATAGGTCTAGGGGAATAAATCTTTGGGCACCTAGTGGTCATG

4 CATGACCACTAGGAGCATCTTTGGCGATGGGCAGTGGGCACGGCAACTTGAGATCGACTAGGGGAATAAATCTTTGGGCACCTAGTGGTCATG

4 CATGACCACTAGGAGCATCTTTGGCGACACCGGTGGGCATGTGCTGGTGAGGTGTGCCTAGGGGAATAAATCTTTGGGCACCTAGTGGTCATG

4 CATGACCACTAGGAGCATCTTTGGCGAAGGGCACGGCATATGGACTGGCCCAGCCTACTAGGGGAATAAATCTTTGGGCACCTAGTGGTCATG

4 CATGACCACTAGGAGCATCTTTGGCGACGGGTGGTGTGACTACCCCACGGTGGTCGACTAGGGGAATAAATCTTTGGGCACCTAGTGGTCATG

4 CATGACCACTAGGAGCATCTTTGGCGACTTCAGATGTCGAAGCAACAAGAGCTCAATCTAGGGGAATAAATCTTTGGGCACCTAGTGGTCATG

4 CATGACCACTAGGAGCATCTTTGGCGAGCAGTACAGAGCGAAGGAGCACCTGCGAGACTAGGGGAATAAATCTTTGGGCACCTAGTGGTCATG

4 CATGACCACTAGGAGCATCTTTGGCGATCTGTGGTCTATCGGGAAGCCACCGGTCGACTAGGGGAATAAATCTTTGGGCACCTAGTGGTCATG

4 CATGACCACTAGGAGCATCTTTGGCGACGTAAAAAATCGGTGTGTTATACGTTCGCGCTAGGGGAATAAATCTTTGGGCACCTAGTGGTCATG

4 CATGACCACTAGGAGCATCTTTGGCGATGAGTGGCGTGGGACATACGCAACGCCTCGCTAGGGGAATAAATCTTTGGGCACCTAGTGGTCATG

4 CATGACCACTAGGAGCATCTTTGGCGACACACGACGTAGGGAGGCAGCGGATGGCGACTAGGGGAATAAATCTTTGGGCACCTAGTGGTCATG

4 CATGACCACTAGGAGCATCTTTGGCGATCCATATGGCCTCGTGGCAGAGATAGTCGACTAGGGGAATAAATCTTTGGGCACCTAGTGGTCATG

4 CATGACCACTAGGAGCATCTTTGGCGATCGAGCGTAAGGCCGTGCGGCCCTTGTCGCCTAGGGGAATAAATCTTTGGGCACCTAGTGGTCATG

4 CATGACCACTAGGAGCATCTTTGGCGATCGTTTTAGTCTAAACCCCCCGGAGGTCCTCTAGGGGAATAAATCTTTGGGCACCTAGTGGTCATG

4 CATGACCACTAGGAGCATCTTTGGCGACGTCGCGTTAGGTGGTAACAATGGCGTTCGCTAGGGGAATAAATCTTTGGGCACCTAGTGGTCATG

4 CATGACCACTAGGAGCATCTTTGGCGACGGGGGGCGTTCATATGTCCGTGAGTTGCTCTAGGGGAATAAATCTTTGGGCACCTAGTGGTCATG

4 CATGACCACTAGGAGCATCTTTGGCGATCGTGAGGGCGCGGTGGAACATCTCGATCCCTAGGGGAATAAATCTTTGGGCACCTAGTGGTCATG

4 CATGACCACTAGGAGCATCTTTGGCGATCCGGGGGCCGTGGATAGGCGACGTTTAGGCTAGGGGAATAAATCTTTGGGCACCTAGTGGTCATG

4 CATGACCACTAGGAGCATCTTTGGCGAGACGGCGGCTAGCAATGCTCACGATGCGAGCTAGGGGAATAAATCTTTGGGCACCTAGTGGTCATG

4 CATGACCACTAGGAGCATCTTTGGCGAACGTCCAGCGGCAAGCGCAGGAGTACGACACTAGGGGAATAAATCTTTGGGCACCTAGTGGTCATG

4 CATGACCACTAGGAGCATCTTTGGCGACGGTGGTCCAAGCGTAGTATGGCTGGTCGACTAGGGGAATAAATCTTTGGGCACCTAGTGGTCATG

4 CATGACCACTAGGAGCATCTTTGGCGAGCGGGAACTATATGCCTAAGGGCACCGTAGCTAGGGGAATAAATCTTTGGGCACCTAGTGGTCATG

4 CATGACCACTAGGAGCATCTTTGGCGAGCCTACCGCGTGGGTGGGCATGGTGCGAGACTAGGGGAATAAATCTTTGGGCACCTAGTGGTCATG

4 CATGACCACTAGGAGCATCTTTGGCGAGCCTTGCTTGGGAGGTTGCTCCACCAGTTCCTAGGGGAATAAATCTTTGGGCACCTAGTGGTCATG

4 CATGACCACTAGGAGCATCTTTGGCGACGAGGGGCTGCCGGAGTAGGGATAAGTCGACTAGGGGAATAAATCTTTGGGCACCTAGTGGTCATG

4 CATGACCACTAGGAGCATCTTTGGCGAGCAGAAGGGGGTGTCGAAACGCCGCTGCCGCTAGGGGAATAAATCTTTGGGCACCTAGTGGTCATG

4 CATGACCACTAGGAGCATCTTTGGCGACGCGAGGGGACCGCAATGTGGCCTAGTCGACTAGGGGAATAAATCTTTGGGCACCTAGTGGTCATG

4 CATGACCACTAGGAGCATCTTTGGCGACGGTGGGCGTTTGGGAGAAGGATGAGTCGACTAGGGGAATAAATCTTTGGGCACCTAGTGGTCATG

4 CATGACCACTAGGAGCATCTTTGGCGACTACACAGGTGAGGCTCCGTAAAAGGCGCACTAGGGGAATAAATCTTTGGGCACCTAGTGGTCATG

4 CATGACCACTAGGAGCATCTTTGGCGACACGTACAAGCCTGGTCTGTACGGGTGGACCTAGGGGAATAAATCTTTGGGCACCTAGTGGTCATG

4 CATGACCACTAGGAGCATCTTTGGCGACATGAGTGGGCAGGGCTGGTTGGTCGTCGACTAGGGGAATAAATCTTTGGGCACCTAGTGGTCATG

4 CATGACCACTAGGAGCATCTTTGGCGACGAGGGAACGCTACTCGTTGAGGTTCGAGCCTAGGGGAATAAATCTTTGGGCACCTAGTGGTCATG

4 CATGACCACTAGGAGCATCTTTGGCGAGTGGTCACGCGTGTCGCGATCAACAATTGCCTAGGGGAATAAATCTTTGGGCACCTAGTGGTCATG

4 CATGACCACTAGGAGCATCTTTGGCGACACTGTCCCACGCGTTATAGGATGCTCGAGCTAGGGGAATAAATCTTTGGGCACCTAGTGGTCATG

4 CATGACCACTAGGAGCATCTTTGGCGACGGTGGTCGCATGGCAAGCCGAAACGTCCGCTAGGGGAATAAATCTTTGGGCACCTAGTGGTCATG

4 CATGACCACTAGGAGCATCTTTGGCGACTGGACATACACGATAGATCGTGGATCCGCCTAGGGGAATAAATCTTTGGGCACCTAGTGGTCATG

4 CATGACCACTAGGAGCATCTTTGGCGAGGCATATAGTCTGTGGTTGTGTGTTACCGCCTAGGGGAATAAATCTTTGGGCACCTAGTGGTCATG

4 CATGACCACTAGGAGCATCTTTGGCGATGTTGCAGGGACCAACGGTACTTGAGCCATCTAGGGGAATAAATCTTTGGGCACCTAGTGGTCATG

4 CATGACCACTAGGAGCATCTTTGGCGATTCGGCACGGCGGGTCCGTATAGGAATCGACTAGGGGAATAAATCTTTGGGCACCTAGTGGTCATG

4 CATGACCACTAGGAGCATCTTTGGCGAGCACTTGTAGAGGGTCCGCTCGAGGCAAGCCTAGGGGAATAAATCTTTGGGCACCTAGTGGTCATG

4 CATGACCACTAGGAGCATCTTTGGCGATGGAGTGCGCGTACGGGGCTCCTTCCGCCACTAGGGGAATAAATCTTTGGGCACCTAGTGGTCATG

4 CATGACCACTAGGAGCATCTTTGGCGAGGGAGCAGGTAGAGATGCGGTAGGTACGAGCTAGGGGAATAAATCTTTGGGCACCTAGTGGTCATG

4 CATGACCACTAGGAGCATCTTTGGCGATTGAGGAGGCGCGTGCGATTACTGAATTGACTAGGGGAATAAATCTTTGGGCACCTAGTGGTCATG

4 CATGACCACTAGGAGCATCTTTGGCGAGTTTGTGGCGGCGCGCGACACTGGATACGCCTAGGGGAATAAATCTTTGGGCACCTAGTGGTCATG

4 CATGACCACTAGGAGCATCTTTGGCGACCCATGTGTGGTGGTCTACACCTGAAGGAGCTAGGGGAATAAATCTTTGGGCACCTAGTGGTCATG

4 CATGACCACTAGGAGCATCTTTGGCGACTATGTGGGTCTAGAGCGAAAACTGGTCGACTAGGGGAATAAATCTTTGGGCACCTAGTGGTCATG

4 CATGACCACTAGGAGCATCTTTGGCGACTACGTGGGGCGTACGACCTTAACGAGCGACTAGGGGAATAAATCTTTGGGCACCTAGTGGTCATG

4 CATGACCACTAGGAGCATCTTTGGCGAGGCACCGGCGCGACAACGGGATGCTCCGAGCTAGGGGAATAAATCTTTGGGCACCTAGTGGTCATG

4 CATGACCACTAGGAGCATCTTTGGCGATCGGACGTTGTGTTAGAACCAGAGAAGCCTCTAGGGGAATAAATCTTTGGGCACCTAGTGGTCATG

4 CATGACCACTAGGAGCATCTTTGGCGACAAGAGGTGGCTTGTATAGTCTGTGTCGACCTAGGGGAATAAATCTTTGGGCACCTAGTGGTCATG

4 CATGACCACTAGGAGCATCTTTGGCGAGTGTCGGCAGGGCCAAGGAACAGACGTCGACTAGGGGAATAAATCTTTGGGCACCTAGTGGTCATG

4 CATGACCACTAGGAGCATCTTTGGCGAGAGGCTCCGCTGTGTTTCTATGCTGATCGACTAGGGGAATAAATCTTTGGGCACCTAGTGGTCATG

4 CATGACCACTAGGAGCATCTTTGGCGACTTTGGTCTAGGGTCGAAAACTAAAGGAGCCTAGGGGAATAAATCTTTGGGCACCTAGTGGTCATG

4 CATGACCACTAGGAGCATCTTTGGCGATCTCCAGTGGGGGAATAAGTTCCCTTTCGACTAGGGGAATAAATCTTTGGGCACCTAGTGGTCATG

4 CATGACCACTAGGAGCATCTTTGGCGAATAGGACAGGCTTGGATCTACTGTGATCGACTAGGGGAATAAATCTTTGGGCACCTAGTGGTCATG

4 CATGACCACTAGGAGCATCTTTGGCGATAGGTGGTCAGACGCCTGGTTGTAAAACGCCTAGGGGAATAAATCTTTGGGCACCTAGTGGTCATG

4 CATGACCACTAGGAGCATCTTTGGCGACCGCTCGTGGGTGTCTCTCGAGTATTGCCACTAGGGGAATAAATCTTTGGGCACCTAGTGGTCATG

4 CATGACCACTAGGAGCATCTTTGGCGAACGTGGTCGTGCATGGATGGGAAAGTGCGCCTAGGGGAATAAATCTTTGGGCACCTAGTGGTCATG

4 CATGACCACTAGGAGCATCTTTGGCGACTAGGGTCTTATGCGAACGCACCGGTCGATCTAGGGGAATAAATCTTTGGGCACCTAGTGGTCATG

4 CATGACCACTAGGAGCATCTTTGGCGATCCGGGGGCACGAACCGTGCGACGTTTAGGCTAGGGGAATAAATCTTTGGGCACCTAGTGGTCATG

4 CATGACCACTAGGAGCATCTTTGGCGAACTGTCGTGCTGGAGCGCGTACATATCGATCTAGGGGAATAAATCTTTGGGCACCTAGTGGTCATG

4 CATGACCACTAGGAGCATCTTTGGCGATCGGGACTGCGACGGGACTGCAGAGCGCCGCTAGGGGAATAAATCTTTGGGCACCTAGTGGTCATG

4 CATGACCACTAGGAGCATCTTTGGCGAGTGGTGGGCTTGGGGAGAAGGACAACGAGACTAGGGGAATAAATCTTTGGGCACCTAGTGGTCATG

4 CATGACCACTAGGAGCATCTTTGGCGATAGAGGTGCTCTCGGCCCACTTATTAATCGCTAGGGGAATAAATCTTTGGGCACCTAGTGGTCATG

4 CATGACCACTAGGAGCATCTTTGGCGACGGTGGCCGATCATTGGTCTCAATAGGGAGCTAGGGGAATAAATCTTTGGGCACCTAGTGGTCATG

4 CATGACCACTAGGAGCATCTTTGGCGATCGAAACGGGTGACGGCAGGGTAGATCGCACTAGGGGAATAAATCTTTGGGCACCTAGTGGTCATG

4 CATGACCACTAGGAGCATCTTTGGCGACCTCGGACCCACGGAAGTGGGAAAGTGGAGCTAGGGGAATAAATCTTTGGGCACCTAGTGGTCATG

4 CATGACCACTAGGAGCATCTTTGGCGACTGAGGTGGGCAAACGATACAACTGTCGCTCTAGGGGAATAAATCTTTGGGCACCTAGTGGTCATG

4 CATGACCACTAGGAGCATCTTTGGCGAGGGTACCCAAGGAACGACAGGGCTATGTCACTAGGGGAATAAATCTTTGGGCACCTAGTGGTCATG

4 CATGACCACTAGGAGCATCTTTGGCGAGCGGCGGGGAGCTGGACCCGCGGGGCGCGCCTAGGGGAATAAATCTTTGGGCACCTAGTGGTCATG

4 CATGACCACTAGGAGCATCTTTGGCGACGGTAAAAGGAATTACCGCACTGAGGGCGCCTAGGGGAATAAATCTTTGGGCACCTAGTGGTCATG

4 CATGACCACTAGGAGCATCTTTGGCGATTGACCCGTTTACGAGCGTCTGGTCTGTCGCTAGGGGAATAAATCTTTGGGCACCTAGTGGTCATG

4 CATGACCACTAGGAGCATCTTTGGCGACGTATGTTGTCATATAGAGTACTGTCGCATCTAGGGGAATAAATCTTTGGGCACCTAGTGGTCATG

4 CATGACCACTAGGAGCATCTTTGGCGACAGCATAGGTCGGGCAATGCTGATCCTCGACTAGGGGAATAAATCTTTGGGCACCTAGTGGTCATG

4 CATGACCACTAGGAGCATCTTTGGCGAGGCGTGTACGCACCGTGGGCCGCGTCTCGACTAGGGGAATAAATCTTTGGGCACCTAGTGGTCATG

4 CATGACCACTAGGAGCATCTTTGGCGACGGTGGGCTCTCGGCTAGGCCCTACGCCGACTAGGGGAATAAATCTTTGGGCACCTAGTGGTCATG

4 CATGACCACTAGGAGCATCTTTGGCGACGGGTGGGCGTTGGGAGTTTAACCCGAAGACTAGGGGAATAAATCTTTGGGCACCTAGTGGTCATG

4 CATGACCACTAGGAGCATCTTTGGCGAATACCCGGACTGAGCAAGGCTGTTAATCGACTAGGGGAATAAATCTTTGGGCACCTAGTGGTCATG

4 CATGACCACTAGGAGCATCTTTGGCGACGGTGTAGGACGCGATGAGTACCCCGGAGACTAGGGGAATAAATCTTTGGGCACCTAGTGGTCATG

4 CATGACCACTAGGAGCATCTTTGGCGAAAACCGTAGGCATGTGCCGCAGTAGTAGAGCTAGGGGAATAAATCTTTGGGCACCTAGTGGTCATG

4 CATGACCACTAGGAGCATCTTTGGCGAGCGTACTGCAGGTAAGGAGGGCAGCGATGACTAGGGGAATAAATCTTTGGGCACCTAGTGGTCATG

4 CATGACCACTAGGAGCATCTTTGGCGACACCTTGGCATGCTATGGCCATAGTGGCCTCTAGGGGAATAAATCTTTGGGCACCTAGTGGTCATG

4 CATGACCACTAGGAGCATCTTTGGCGACGATGGTTGGGAGGGATGTATTTCGGGCGACTAGGGGAATAAATCTTTGGGCACCTAGTGGTCATG

4 CATGACCACTAGGAGCATCTTTGGCGAGCTATGGCCGGACAAGGGGAGGACGCTAGCCTAGGGGAATAAATCTTTGGGCACCTAGTGGTCATG

4 CATGACCACTAGGAGCATCTTTGGCGACTTGTGGTTCTAAATTTCGGGGTTAGGCGCCTAGGGGAATAAATCTTTGGGCACCTAGTGGTCATG

4 CATGACCACTAGGAGCATCTTTGGCGAGCGGGCGGCGAGCGGACAGAGGTAATGCTGCTAGGGGAATAAATCTTTGGGCACCTAGTGGTCATG

4 CATGACCACTAGGAGCATCTTTGGCGAGAAGACTCTGGGTTCGGGGACCAGTTGCTGCTAGGGGAATAAATCTTTGGGCACCTAGTGGTCATG

4 CATGACCACTAGGAGCATCTTTGGCGAGGAAAAAAAGGTCGTTGCGTACGACGAGCACTAGGGGAATAAATCTTTGGGCACCTAGTGGTCATG

4 CATGACCACTAGGAGCATCTTTGGCGAAACGGTGCCCGCGGAAAGCATGCGTTCGCCCTAGGGGAATAAATCTTTGGGCACCTAGTGGTCATG

4 CATGACCACTAGGAGCATCTTTGGCGACTCGGTGGGCAGTGGAGTGCCGGAAGGAGACTAGGGGAATAAATCTTTGGGCACCTAGTGGTCATG

4 CATGACCACTAGGAGCATCTTTGGCGACCCAGCCTACCCGCACTCGAGGTGGTCGAACTAGGGGAATAAATCTTTGGGCACCTAGTGGTCATG

4 CATGACCACTAGGAGCATCTTTGGCGACTACACAGGTCATGCACCGTGTCTAGTTGACTAGGGGAATAAATCTTTGGGCACCTAGTGGTCATG

4 CATGACCACTAGGAGCATCTTTGGCGAAAGAGATCGTAGGATGATCGCGATATCGCACTAGGGGAATAAATCTTTGGGCACCTAGTGGTCATG

4 CATGACCACTAGGAGCATCTTTGGCGAGCGTAGCGTGGGCACGGTGCGGGTTTCCTCCTAGGGGAATAAATCTTTGGGCACCTAGTGGTCATG

4 CATGACCACTAGGAGCATCTTTGGCGACATAGTTGGTCAGGTCACACACTGGTTCGCCTAGGGGAATAAATCTTTGGGCACCTAGTGGTCATG

4 CATGACCACTAGGAGCATCTTTGGCGAGTTTACCGTTGGCCGCCGGCCAAACTGCGACTAGGGGAATAAATCTTTGGGCACCTAGTGGTCATG

4 CATGACCACTAGGAGCATCTTTGGCGATGGGTCGATCTACCTCCGACTAAGGTAAGCCTAGGGGAATAAATCTTTGGGCACCTAGTGGTCATG

4 CATGACCACTAGGAGCATCTTTGGCGAGACACTGGGGTGTGCGATATTGTGTTCCGCCTAGGGGAATAAATCTTTGGGCACCTAGTGGTCATG

4 CATGACCACTAGGAGCATCTTTGGCGACAAGGCGGTGCCTGTCAAGGTCCGACCAGACTAGGGGAATAAATCTTTGGGCACCTAGTGGTCATG

4 CATGACCACTAGGAGCATCTTTGGCGACCCACGGTAAAGCAGTGTACGTGTGGTGGACTAGGGGAATAAATCTTTGGGCACCTAGTGGTCATG

4 CATGACCACTAGGAGCATCTTTGGCGACTGGCGGGAGTATGTGACCGAGCGCTGAGACTAGGGGAATAAATCTTTGGGCACCTAGTGGTCATG

4 CATGACCACTAGGAGCATCTTTGGCGAGGCACACAAACGGGATGGGTTGTCGCAGTCCTAGGGGAATAAATCTTTGGGCACCTAGTGGTCATG

4 CATGACCACTAGGAGCATCTTTGGCGACTGGGCCACGCGGAGCGGGGGTAATTATCGCTAGGGGAATAAATCTTTGGGCACCTAGTGGTCATG

4 CATGACCACTAGGAGCATCTTTGGCGAACGGCAGGTGTTGCGGTGGTCAGTGAATCCCTAGGGGAATAAATCTTTGGGCACCTAGTGGTCATG

4 CATGACCACTAGGAGCATCTTTGGCGACGCAGTGGGCTTCGGTATGGAGTTGAGGAGCTAGGGGAATAAATCTTTGGGCACCTAGTGGTCATG

4 CATGACCACTAGGAGCATCTTTGGCGACGCAGGGGGCATGGTTCAATCGGGCGGAGCCTAGGGGAATAAATCTTTGGGCACCTAGTGGTCATG

4 CATGACCACTAGGAGCATCTTTGGCGACGAAGGCTGTTTTCGTAACAGATGTCTAGACTAGGGGAATAAATCTTTGGGCACCTAGTGGTCATG

4 CATGACCACTAGGAGCATCTTTGGCGACCCGTCGTATAAGGGACATACACGGGTGGACTAGGGGAATAAATCTTTGGGCACCTAGTGGTCATG

4 CATGACCACTAGGAGCATCTTTGGCGAGTACTCACGGTTGCTCAACCGGCGCGACGCCTAGGGGAATAAATCTTTGGGCACCTAGTGGTCATG

4 CATGACCACTAGGAGCATCTTTGGCGATTGCTCGGACTGGGTTGCAAGACAGCGCCGCTAGGGGAATAAATCTTTGGGCACCTAGTGGTCATG

4 CATGACCACTAGGAGCATCTTTGGCGACCTCGGCGGCGACGTCCGCCGATCAGTCGGCTAGGGGAATAAATCTTTGGGCACCTAGTGGTCATG

4 CATGACCACTAGGAGCATCTTTGGCGACATAGTGGATCGCAGTCCCGAAAAGGACGCCTAGGGGAATAAATCTTTGGGCACCTAGTGGTCATG

4 CATGACCACTAGGAGCATCTTTGGCGAGGGTAGTGGGCAGGTAGATACATCGCACTACTAGGGGAATAAATCTTTGGGCACCTAGTGGTCATG

4 CATGACCACTAGGAGCATCTTTGGCGAGTGTATCGTTGGGCGTCCCGGGACTCTCGCCTAGGGGAATAAATCTTTGGGCACCTAGTGGTCATG

4 CATGACCACTAGGAGCATCTTTGGCGATCCAGGCTCTGATAAGCAGCTTCGTCGCCGCTAGGGGAATAAATCTTTGGGCACCTAGTGGTCATG

4 CATGACCACTAGGAGCATCTTTGGCGATTGTCCTGGGTTCACTTGAACGCAACAGAGCTAGGGGAATAAATCTTTGGGCACCTAGTGGTCATG

4 CATGACCACTAGGAGCATCTTTGGCGAAGTGAGGCTCTGAGGTGGCGCTGTGGTAGCCTAGGGGAATAAATCTTTGGGCACCTAGTGGTCATG

4 CATGACCACTAGGAGCATCTTTGGCGAGTGCGTGGCTCCGTGGAACCATAGATCTCGCTAGGGGAATAAATCTTTGGGCACCTAGTGGTCATG

4 CATGACCACTAGGAGCATCTTTGGCGATGGAGATGCGCACGGGTGGTCGTAAATCGCCTAGGGGAATAAATCTTTGGGCACCTAGTGGTCATG

4 CATGACCACTAGGAGCATCTTTGGCGAATCCCGGATCGGGACAAGTAAGGGATGTGACTAGGGGAATAAATCTTTGGGCACCTAGTGGTCATG

4 CATGACCACTAGGAGCATCTTTGGCGATAGGCGACCCGGGGGCGGCCCGGGCGCTCGCTAGGGGAATAAATCTTTGGGCACCTAGTGGTCATG

4 CATGACCACTAGGAGCATCTTTGGCGAGACGATGTCGGCTCACGGTTAAGTCTGCAGCTAGGGGAATAAATCTTTGGGCACCTAGTGGTCATG

4 CATGACCACTAGGAGCATCTTTGGCGATTGCGAGGACGTCGGGGACGTCACGAGAGACTAGGGGAATAAATCTTTGGGCACCTAGTGGTCATG

4 CATGACCACTAGGAGCATCTTTGGCGACTAGATAACAACTATGGATATCCAAACAGCCTAGGGGAATAAATCTTTGGGCACCTAGTGGTCATG

4 CATGACCACTAGGAGCATCTTTGGCGAGATGGTCATAGGGGGAAGAGGGCCAGTCGCCTAGGGGAATAAATCTTTGGGCACCTAGTGGTCATG

4 CATGACCACTAGGAGCATCTTTGGCGACGTCCTTGGGTGTAATCCTCGGCTAGGCGCCTAGGGGAATAAATCTTTGGGCACCTAGTGGTCATG

4 CATGACCACTAGGAGCATCTTTGGCGACGACACTGGAGATCCGTCGGAAGGAGCCAGCTAGGGGAATAAATCTTTGGGCACCTAGTGGTCATG

4 CATGACCACTAGGAGCATCTTTGGCGATTACAGGCGTGCATGGTCGACCTGGTATCGCTAGGGGAATAAATCTTTGGGCACCTAGTGGTCATG

4 CATGACCACTAGGAGCATCTTTGGCGAATAGTCCTGGGTGAAATGTCTCTGCATTCGCTAGGGGAATAAATCTTTGGGCACCTAGTGGTCATG

4 CATGACCACTAGGAGCATCTTTGGCGAACCCGAACGGTTGAGCGAACGGGATCTCGACTAGGGGAATAAATCTTTGGGCACCTAGTGGTCATG

4 CATGACCACTAGGAGCATCTTTGGCGACGGGCAGGGCGCAGGTTAGCCCCGTTGCTCCTAGGGGAATAAATCTTTGGGCACCTAGTGGTCATG

4 CATGACCACTAGGAGCATCTTTGGCGACAACGGTAGGACAGGGCACCAAGATGGCGACTAGGGGAATAAATCTTTGGGCACCTAGTGGTCATG

4 CATGACCACTAGGAGCATCTTTGGCGAACTGGGTGGTCTGTGAGGCTGGTGAGTGAGCTAGGGGAATAAATCTTTGGGCACCTAGTGGTCATG

4 CATGACCACTAGGAGCATCTTTGGCGACGTAGGGGGCATGGGTCAGAAGGTCGCCAGCTAGGGGAATAAATCTTTGGGCACCTAGTGGTCATG

4 CATGACCACTAGGAGCATCTTTGGCGACTAGGGTCACTCGATCGGAAGGGTAGTCGACTAGGGGAATAAATCTTTGGGCACCTAGTGGTCATG

4 CATGACCACTAGGAGCATCTTTGGCGAGCTCGTGCGCAGCTGGGTGAAGGGCTCGATCTAGGGGAATAAATCTTTGGGCACCTAGTGGTCATG

4 CATGACCACTAGGAGCATCTTTGGCGAGAGTGGCAAGGGACGGCCTCCTCTAAACGCCTAGGGGAATAAATCTTTGGGCACCTAGTGGTCATG

4 CATGACCACTAGGAGCATCTTTGGCGATCAGCCGGGTAGAGACGAGCTAGTGGTCGCCTAGGGGAATAAATCTTTGGGCACCTAGTGGTCATG

4 CATGACCACTAGGAGCATCTTTGGCGACGGGGGTCCATATATCGTAATAACGTTCGCCTAGGGGAATAAATCTTTGGGCACCTAGTGGTCATG

4 CATGACCACTAGGAGCATCTTTGGCGAACTCCGGAGGGAAGATAGTCACTTAGGTCGCTAGGGGAATAAATCTTTGGGCACCTAGTGGTCATG

4 CATGACCACTAGGAGCATCTTTGGCGAATGGTGGTCTGGTGGTAATAACGTATGTAGCTAGGGGAATAAATCTTTGGGCACCTAGTGGTCATG

4 CATGACCACTAGGAGCATCTTTGGCGACCTCGGTGGGCATGGCTGGGGCCGAGGCAGCTAGGGGAATAAATCTTTGGGCACCTAGTGGTCATG

4 CATGACCACTAGGAGCATCTTTGGCGACCATTAATTTGCTCTTAGTAGGCGCTTCGCCTAGGGGAATAAATCTTTGGGCACCTAGTGGTCATG

4 CATGACCACTAGGAGCATCTTTGGCGATCGACGCTGGTATGGGGGTTCGCGGTCGCTCTAGGGGAATAAATCTTTGGGCACCTAGTGGTCATG

4 CATGACCACTAGGAGCATCTTTGGCGATCAGGCCGCCAGGGCGTACGAAGCGTGGTTCTAGGGGAATAAATCTTTGGGCACCTAGTGGTCATG

4 CATGACCACTAGGAGCATCTTTGGCGAATAGTGATGCGCTGATGAACGCATTTCGAACTAGGGGAATAAATCTTTGGGCACCTAGTGGTCATG

4 CATGACCACTAGGAGCATCTTTGGCGACTTGTCTAACAATGTTCATTGAATATCGCCCTAGGGGAATAAATCTTTGGGCACCTAGTGGTCATG

4 CATGACCACTAGGAGCATCTTTGGCGAGACCTTGCGGCGTAGAGCGCTGGGTTCGCGCTAGGGGAATAAATCTTTGGGCACCTAGTGGTCATG

4 CATGACCACTAGGAGCATCTTTGGCGAGATCGGGAGAATCAGTGGCATTGGTGTCTCCTAGGGGAATAAATCTTTGGGCACCTAGTGGTCATG

4 CATGACCACTAGGAGCATCTTTGGCGAACGGCAGGTGTTGCGGTGGTCTGTGAAACCCTAGGGGAATAAATCTTTGGGCACCTAGTGGTCATG

4 CATGACCACTAGGAGCATCTTTGGCGAGCGCTGAACGCACAGATCAACGGGCGCTGCCTAGGGGAATAAATCTTTGGGCACCTAGTGGTCATG

4 CATGACCACTAGGAGCATCTTTGGCGAACTGGGGTGCCGCGAGTTCACGCACGTCGCCTAGGGGAATAAATCTTTGGGCACCTAGTGGTCATG

4 CATGACCACTAGGAGCATCTTTGGCGATGAGTCAGCAAAGCGTCTAAGGGCGAGCGCCTAGGGGAATAAATCTTTGGGCACCTAGTGGTCATG

4 CATGACCACTAGGAGCATCTTTGGCGACGAGATGGTGAAGACCGCCACGGGAGGCGACTAGGGGAATAAATCTTTGGGCACCTAGTGGTCATG

4 CATGACCACTAGGAGCATCTTTGGCGAGGCGCAACGAGGACGTCGAGCGCTCCGAGTCTAGGGGAATAAATCTTTGGGCACCTAGTGGTCATG

4 CATGACCACTAGGAGCATCTTTGGCGAGCTCCGAGCAAGCGAACCGCGAAAAGTCGGCTAGGGGAATAAATCTTTGGGCACCTAGTGGTCATG

4 CATGACCACTAGGAGCATCTTTGGCGATCTCGCCAAAAACGTGCTAAATATTGCTGTCTAGGGGAATAAATCTTTGGGCACCTAGTGGTCATG

4 CATGACCACTAGGAGCATCTTTGGCGACGCAGGGTATGGGTATCGATACCCAAGTGTCTAGGGGAATAAATCTTTGGGCACCTAGTGGTCATG

4 CATGACCACTAGGAGCATCTTTGGCGAGACGTGCGTCGGCGAGAGCCGTAGGGTCGCCTAGGGGAATAAATCTTTGGGCACCTAGTGGTCATG

4 CATGACCACTAGGAGCATCTTTGGCGACTAAGCCGGGAACCAGCTTCCAACAATCGACTAGGGGAATAAATCTTTGGGCACCTAGTGGTCATG

4 CATGACCACTAGGAGCATCTTTGGCGACGTCTGTGGTCGGGAAGCGAGCGTTGCCATCTAGGGGAATAAATCTTTGGGCACCTAGTGGTCATG

4 CATGACCACTAGGAGCATCTTTGGCGAGGGTACTGTTGGACTCCAGAGGGCCGAGATCTAGGGGAATAAATCTTTGGGCACCTAGTGGTCATG

4 CATGACCACTAGGAGCATCTTTGGCGACACCACGGGGTGGGCTAGACACTGGTGCGACTAGGGGAATAAATCTTTGGGCACCTAGTGGTCATG

4 CATGACCACTAGGAGCATCTTTGGCGATACGGCCACGCTCTGTAATGTACACAGCCTCTAGGGGAATAAATCTTTGGGCACCTAGTGGTCATG

4 CATGACCACTAGGAGCATCTTTGGCGATAGTTCATCTATGAGTCACAGTATGTGCATCTAGGGGAATAAATCTTTGGGCACCTAGTGGTCATG

4 CATGACCACTAGGAGCATCTTTGGCGACCGGCCAGCGGGCATCGGCTCGAAGATCGACTAGGGGAATAAATCTTTGGGCACCTAGTGGTCATG

4 CATGACCACTAGGAGCATCTTTGGCGACTGAGAGGTCCTAACCAGAGGAAAGGGCGCCTAGGGGAATAAATCTTTGGGCACCTAGTGGTCATG

4 CATGACCACTAGGAGCATCTTTGGCGAAAGTGGTCGTGCGTTCGAGAGACCATACGCCTAGGGGAATAAATCTTTGGGCACCTAGTGGTCATG

4 CATGACCACTAGGAGCATCTTTGGCGACGCACCAAGTGATAGTGAGTTCGCCGTCGTCTAGGGGAATAAATCTTTGGGCACCTAGTGGTCATG

4 CATGACCACTAGGAGCATCTTTGGCGACTTCAGCGATACAGATAACCGGGCTGTCGACTAGGGGAATAAATCTTTGGGCACCTAGTGGTCATG

4 CATGACCACTAGGAGCATCTTTGGCGAAAGTTAGTTTACGTGTAAAGGTAAGTTTGCCTAGGGGAATAAATCTTTGGGCACCTAGTGGTCATG

4 CATGACCACTAGGAGCATCTTTGGCGAATCGGTCGAGGGGCACTTTGGCGAGGTGAGCTAGGGGAATAAATCTTTGGGCACCTAGTGGTCATG

4 CATGACCACTAGGAGCATCTTTGGCGAAGCGAGACTCGCAGGAGGTAGTGGATTCGACTAGGGGAATAAATCTTTGGGCACCTAGTGGTCATG

4 CATGACCACTAGGAGCATCTTTGGCGACATGATGAGAATGGCGGTGCTAACCGTTGACTAGGGGAATAAATCTTTGGGCACCTAGTGGTCATG

4 CATGACCACTAGGAGCATCTTTGGCGACCATACTGGTGGTGCGACCAGCGACATAGCCTAGGGGAATAAATCTTTGGGCACCTAGTGGTCATG

4 CATGACCACTAGGAGCATCTTTGGCGACATAACCAGGGTTAGTCGAGGGAGGTCGCTCTAGGGGAATAAATCTTTGGGCACCTAGTGGTCATG

4 CATGACCACTAGGAGCATCTTTGGCGATACCATAACGGGCTGTGCCGTTATGGACGGCTAGGGGAATAAATCTTTGGGCACCTAGTGGTCATG

4 CATGACCACTAGGAGCATCTTTGGCGAGAGTGACACGTTCTAGTGCCTTCTTGGAGCCTAGGGGAATAAATCTTTGGGCACCTAGTGGTCATG

4 CATGACCACTAGGAGCATCTTTGGCGACAGCTGCTTCAGGTTATCCGGTGTCGGCTTCTAGGGGAATAAATCTTTGGGCACCTAGTGGTCATG

4 CATGACCACTAGGAGCATCTTTGGCGACCGTAGTACCTAGGCAAAGTACAGGGGGAGCTAGGGGAATAAATCTTTGGGCACCTAGTGGTCATG

4 CATGACCACTAGGAGCATCTTTGGCGAACCGGACAGCAGGAATGAGTGTCGGGGAGACTAGGGGAATAAATCTTTGGGCACCTAGTGGTCATG

4 CATGACCACTAGGAGCATCTTTGGCGAGATCGGGAGAATCGGTGGCATTGGTATCTCCTAGGGGAATAAATCTTTGGGCACCTAGTGGTCATG

4 CATGACCACTAGGAGCATCTTTGGCGATAGCCTCTGATCGGTACGATAGGGCTTACGCTAGGGGAATAAATCTTTGGGCACCTAGTGGTCATG

4 CATGACCACTAGGAGCATCTTTGGCGATATCTGGCTGGCAGGTTCGTACTGGTTCGACTAGGGGAATAAATCTTTGGGCACCTAGTGGTCATG

4 CATGACCACTAGGAGCATCTTTGGCGACACGAGGCCCATGTGGCCCAGATCTCACGCCTAGGGGAATAAATCTTTGGGCACCTAGTGGTCATG

4 CATGACCACTAGGAGCATCTTTGGCGATGGGGGGAACGTATGTTCCAGGGTAGACAGCTAGGGGAATAAATCTTTGGGCACCTAGTGGTCATG

4 CATGACCACTAGGAGCATCTTTGGCGAAGGTAAAGGTGTGATGTGTGGTCGATTCGCCTAGGGGAATAAATCTTTGGGCACCTAGTGGTCATG

4 CATGACCACTAGGAGCATCTTTGGCGACTTGTGGGCTATGCGGCTCAAGAACGTCGACTAGGGGAATAAATCTTTGGGCACCTAGTGGTCATG

4 CATGACCACTAGGAGCATCTTTGGCGATGCGTCGACGCAAAACGTCAGGAGGTGAGCCTAGGGGAATAAATCTTTGGGCACCTAGTGGTCATG

4 CATGACCACTAGGAGCATCTTTGGCGAGGGTAAGTATGAACGTTTCTTGTAGAGAGCCTAGGGGAATAAATCTTTGGGCACCTAGTGGTCATG

4 CATGACCACTAGGAGCATCTTTGGCGACGGTCATAGTTTGTCTGGGTAGTTGTACGCCTAGGGGAATAAATCTTTGGGCACCTAGTGGTCATG

4 CATGACCACTAGGAGCATCTTTGGCGACAACTGGCGGTAATGTCTAATAGCTGAGTCCTAGGGGAATAAATCTTTGGGCACCTAGTGGTCATG

4 CATGACCACTAGGAGCATCTTTGGCGACGGGCGGGCGCCCAAGGGTGTGAGTGTTGCCTAGGGGAATAAATCTTTGGGCACCTAGTGGTCATG

4 CATGACCACTAGGAGCATCTTTGGCGACTGGGTCACTATGAGCGGCGGTGATGTCGACTAGGGGAATAAATCTTTGGGCACCTAGTGGTCATG

4 CATGACCACTAGGAGCATCTTTGGCGACGGACGCACTCGGCACTTGGTAATGGAGCCCTAGGGGAATAAATCTTTGGGCACCTAGTGGTCATG

4 CATGACCACTAGGAGCATCTTTGGCGACGGTTGGTCATTGGAACAGGGGATAAGTCGCTAGGGGAATAAATCTTTGGGCACCTAGTGGTCATG

4 CATGACCACTAGGAGCATCTTTGGCGAGCATCCTTCGTCGGCGGACGCATGGTGATCCTAGGGGAATAAATCTTTGGGCACCTAGTGGTCATG

4 CATGACCACTAGGAGCATCTTTGGCGACCGTGGTCATATGCGAATCTGGGCAGTCGCCTAGGGGAATAAATCTTTGGGCACCTAGTGGTCATG

4 CATGACCACTAGGAGCATCTTTGGCGAATAGGGGTCGCTACGCTACCGTCCACAGAGCTAGGGGAATAAATCTTTGGGCACCTAGTGGTCATG

4 CATGACCACTAGGAGCATCTTTGGCGACCAATCACGGTTTACCGTGGTAAAGTGGAGCTAGGGGAATAAATCTTTGGGCACCTAGTGGTCATG

4 CATGACCACTAGGAGCATCTTTGGCGAGCTAACGTGACAGAGAACGCGTTGTCGAGACTAGGGGAATAAATCTTTGGGCACCTAGTGGTCATG

4 CATGACCACTAGGAGCATCTTTGGCGAGGACGGTGGGCCGGCTAGCCACGGGAATCGCTAGGGGAATAAATCTTTGGGCACCTAGTGGTCATG

4 CATGACCACTAGGAGCATCTTTGGCGAATAGGACAGGAATGGTACTGTATGCGAGCTCTAGGGGAATAAATCTTTGGGCACCTAGTGGTCATG

4 CATGACCACTAGGAGCATCTTTGGCGAATCAAGTTGCACACACGCGAAATGAGCGAACTAGGGGAATAAATCTTTGGGCACCTAGTGGTCATG

4 CATGACCACTAGGAGCATCTTTGGCGATACTGACCGGTTAGGTTGGGCCAGGAAAGCCTAGGGGAATAAATCTTTGGGCACCTAGTGGTCATG

4 CATGACCACTAGGAGCATCTTTGGCGACAGTGGGCTCGAGACTCCACGGTAGTCCGCCTAGGGGAATAAATCTTTGGGCACCTAGTGGTCATG

4 CATGACCACTAGGAGCATCTTTGGCGACTGATCTCGACAGGGTCAAGCAAACGTTGCCTAGGGGAATAAATCTTTGGGCACCTAGTGGTCATG

4 CATGACCACTAGGAGCATCTTTGGCGAGAACGTCGGCTTAGCAGGAAAAGTCTCTCGCTAGGGGAATAAATCTTTGGGCACCTAGTGGTCATG

4 CATGACCACTAGGAGCATCTTTGGCGACCTGAGTAACATCGAGGATACGTCAGGTCCCTAGGGGAATAAATCTTTGGGCACCTAGTGGTCATG

4 CATGACCACTAGGAGCATCTTTGGCGAACTCGCAACGGGCTGTTGACTAAGTAGGTCCTAGGGGAATAAATCTTTGGGCACCTAGTGGTCATG

4 CATGACCACTAGGAGCATCTTTGGCGAGGCTAATATGTAATGTAATCGCAGATCGCTCTAGGGGAATAAATCTTTGGGCACCTAGTGGTCATG

4 CATGACCACTAGGAGCATCTTTGGCGAGGTCCGGCAGACCGTCTTTAAAATAGGCGCCTAGGGGAATAAATCTTTGGGCACCTAGTGGTCATG

4 CATGACCACTAGGAGCATCTTTGGCGACGCGGTAGGTCATACAGCATCAGGTGTCGACTAGGGGAATAAATCTTTGGGCACCTAGTGGTCATG

4 CATGACCACTAGGAGCATCTTTGGCGACACCGCTGACGAAGGACTCGGCTTGGCGTCCTAGGGGAATAAATCTTTGGGCACCTAGTGGTCATG

4 CATGACCACTAGGAGCATCTTTGGCGAGTACGCATCGCGGGTTGGTTAGGTTAGCTCCTAGGGGAATAAATCTTTGGGCACCTAGTGGTCATG

4 CATGACCACTAGGAGCATCTTTGGCGAGTACAGCGATGTAGAGAAGCCGGGGGCGAGCTAGGGGAATAAATCTTTGGGCACCTAGTGGTCATG

4 CATGACCACTAGGAGCATCTTTGGCGACGTTGGGAGCGAAGTCCCAGCGAAGTGGACCTAGGGGAATAAATCTTTGGGCACCTAGTGGTCATG

4 CATGACCACTAGGAGCATCTTTGGCGACAAGGCCAGGATGGATGAGTGGCTGAGGGCCTAGGGGAATAAATCTTTGGGCACCTAGTGGTCATG

4 CATGACCACTAGGAGCATCTTTGGCGACAGTGGGCACCTGGGGCGAATCGGTGTCGACTAGGGGAATAAATCTTTGGGCACCTAGTGGTCATG

4 CATGACCACTAGGAGCATCTTTGGCGAGGTGAAACAGAGGGCAAGGGCTCACATCGACTAGGGGAATAAATCTTTGGGCACCTAGTGGTCATG

4 CATGACCACTAGGAGCATCTTTGGCGACCGTGTCTTTCTCGCTGGATTGCAGGTTGGCTAGGGGAATAAATCTTTGGGCACCTAGTGGTCATG

4 CATGACCACTAGGAGCATCTTTGGCGACGTGGTCAAGTGGGCGGGACCGCCAGACGCCTAGGGGAATAAATCTTTGGGCACCTAGTGGTCATG

4 CATGACCACTAGGAGCATCTTTGGCGACCACCAAGCGCAGTATCGCTACCGACACGCCTAGGGGAATAAATCTTTGGGCACCTAGTGGTCATG

4 CATGACCACTAGGAGCATCTTTGGCGAGGGGCGCTTGTCGGCCGTGTGCGGAACCGCCTAGGGGAATAAATCTTTGGGCACCTAGTGGTCATG

4 CATGACCACTAGGAGCATCTTTGGCGACGCGTGGGCATTGTCTGGTGTGAAAGGCGCCTAGGGGAATAAATCTTTGGGCACCTAGTGGTCATG

4 CATGACCACTAGGAGCATCTTTGGCGACAACTGGGCTGGAATACACCGGGGAGCCGTCTAGGGGAATAAATCTTTGGGCACCTAGTGGTCATG

4 CATGACCACTAGGAGCATCTTTGGCGAATGGTATAGGGTGACGAACGCGTATATGAGCTAGGGGAATAAATCTTTGGGCACCTAGTGGTCATG

4 CATGACCACTAGGAGCATCTTTGGCGAATAGTGGTCGTTTGTCCGAGTAGCACACGCCTAGGGGAATAAATCTTTGGGCACCTAGTGGTCATG

4 CATGACCACTAGGAGCATCTTTGGCGATCCAGGGTAAGCCAGAGGCTAGATATCGCTCTAGGGGAATAAATCTTTGGGCACCTAGTGGTCATG

4 CATGACCACTAGGAGCATCTTTGGCGAGAAGCCGACAACCGATGGTGTTAAGGTCTACTAGGGGAATAAATCTTTGGGCACCTAGTGGTCATG

4 CATGACCACTAGGAGCATCTTTGGCGACTAGGGGGCTGTGCTTCAATGGAGCGTCGACTAGGGGAATAAATCTTTGGGCACCTAGTGGTCATG

4 CATGACCACTAGGAGCATCTTTGGCGATTCGACGGCAGGCCCTGCAGAGTTTAGCCACTAGGGGAATAAATCTTTGGGCACCTAGTGGTCATG

4 CATGACCACTAGGAGCATCTTTGGCGAGTAGTGGGCTCTATCGCAGTAACGAATCGACTAGGGGAATAAATCTTTGGGCACCTAGTGGTCATG

4 CATGACCACTAGGAGCATCTTTGGCGAGCAAGCGCGGTCCCTCGGACCTCACAGTTGCTAGGGGAATAAATCTTTGGGCACCTAGTGGTCATG

4 CATGACCACTAGGAGCATCTTTGGCGAATAAGGGCTCGTCAGTTCACCAGACCCGCACTAGGGGAATAAATCTTTGGGCACCTAGTGGTCATG

4 CATGACCACTAGGAGCATCTTTGGCGAACGAGCGCAGTCGGGGGCATGGCGGTGAGACTAGGGGAATAAATCTTTGGGCACCTAGTGGTCATG

4 CATGACCACTAGGAGCATCTTTGGCGACGTGTGCGGATCGTCGAGGTAGCAAGCAGCCTAGGGGAATAAATCTTTGGGCACCTAGTGGTCATG

4 CATGACCACTAGGAGCATCTTTGGCGAGGCTATCGGGGCCCGACATCGGCCGTGGCTCTAGGGGAATAAATCTTTGGGCACCTAGTGGTCATG

4 CATGACCACTAGGAGCATCTTTGGCGAACGGTAACGTAGTAACGTCGAAGTAGGCGCCTAGGGGAATAAATCTTTGGGCACCTAGTGGTCATG

4 CATGACCACTAGGAGCATCTTTGGCGACACGTGGTGGTGGCTTAGGGCTCTGGTCGACTAGGGGAATAAATCTTTGGGCACCTAGTGGTCATG

4 CATGACCACTAGGAGCATCTTTGGCGATGTAGATCGGTCGTGAGTAGTTGAGAGCGCCTAGGGGAATAAATCTTTGGGCACCTAGTGGTCATG

4 CATGACCACTAGGAGCATCTTTGGCGACGGCTATAGGGTGAACGTTACACAGCGGCGCTAGGGGAATAAATCTTTGGGCACCTAGTGGTCATG

4 CATGACCACTAGGAGCATCTTTGGCGAGGGGTCAGGACTCAGGGGTGAAGCACTCGTCTAGGGGAATAAATCTTTGGGCACCTAGTGGTCATG

4 CATGACCACTAGGAGCATCTTTGGCGAGCCGTCGGCAGCGTCGTCTAGAAAGGCCGCCTAGGGGAATAAATCTTTGGGCACCTAGTGGTCATG

3 CATGACCACTAGGAGCATCTTTGGCGAGGGGTGTGGGGGTACCTATGATACCTCGCTCTAGGGGAATAAATCTTTGGGCACCTAGTGGTCATG

3 CATGACCACTAGGAGCATCTTTGGCGAGAACGTCAGCAGAGCCGCCTCGTGTCTAGCCTAGGGGAATAAATCTTTGGGCACCTAGTGGTCATG

3 CATGACCACTAGGAGCATCTTTGGCGAGTCAGGCCTAAGTTCGCTGCCCGGACGAGACTAGGGGAATAAATCTTTGGGCACCTAGTGGTCATG

3 CATGACCACTAGGAGCATCTTTGGCGAGGACGCATCGCATGGCATAGTTAGCTCCGCCTAGGGGAATAAATCTTTGGGCACCTAGTGGTCATG

3 CATGACCACTAGGAGCATCTTTGGCGACTGGTGGTGTGGTCACACAGTTAAAGCTATCTAGGGGAATAAATCTTTGGGCACCTAGTGGTCATG

3 CATGACCACTAGGAGCATCTTTGGCGACGCAGTGGGCTAATCGACTATAGGCGCGCTCTAGGGGAATAAATCTTTGGGCACCTAGTGGTCATG

3 CATGACCACTAGGAGCATCTTTGGCGACCGGCGAAACGGTTTACGTTACGTCCGTGACTAGGGGAATAAATCTTTGGGCACCTAGTGGTCATG

3 CATGACCACTAGGAGCATCTTTGGCGAGGTGCTGTCCGCTAGTTCACGTGCTCATCGCTAGGGGAATAAATCTTTGGGCACCTAGTGGTCATG

3 CATGACCACTAGGAGCATCTTTGGCGACGGCTGGCTGGCCCGGGCCGATATGTCGTCCTAGGGGAATAAATCTTTGGGCACCTAGTGGTCATG

3 CATGACCACTAGGAGCATCTTTGGCGAGAGGTGGAGCAGTGAGGGTGGACCCACTCGCTAGGGGAATAAATCTTTGGGCACCTAGTGGTCATG

3 CATGACCACTAGGAGCATCTTTGGCGACTGTCCTGAGTTCGAAGATTACGACGTCGACTAGGGGAATAAATCTTTGGGCACCTAGTGGTCATG

3 CATGACCACTAGGAGCATCTTTGGCGAACGGCGTATAGTGGGAGTAAAATGGTGAGCCTAGGGGAATAAATCTTTGGGCACCTAGTGGTCATG

3 CATGACCACTAGGAGCATCTTTGGCGAATGACGGGATATCGGATAGTGCGTATTCGACTAGGGGAATAAATCTTTGGGCACCTAGTGGTCATG

3 CATGACCACTAGGAGCATCTTTGGCGACGGGCTGAGCGTGTGAAGTAAACACGACGCCTAGGGGAATAAATCTTTGGGCACCTAGTGGTCATG

3 CATGACCACTAGGAGCATCTTTGGCGAGGGGCCTGGGGGGGACCAGACGCACCTCGGCTAGGGGAATAAATCTTTGGGCACCTAGTGGTCATG

3 CATGACCACTAGGAGCATCTTTGGCGACGGCAGAAAAATAACTCGCGGCTGATGCCACTAGGGGAATAAATCTTTGGGCACCTAGTGGTCATG

3 CATGACCACTAGGAGCATCTTTGGCGATCCCTCGTGACAGTGGTTCGTAAGGGATGACTAGGGGAATAAATCTTTGGGCACCTAGTGGTCATG

3 CATGACCACTAGGAGCATCTTTGGCGACCGACGTCCAGTGCTATATCCTGGGTCGTACTAGGGGAATAAATCTTTGGGCACCTAGTGGTCATG

3 CATGACCACTAGGAGCATCTTTGGCGAACCGGGAAGCAGTGCTTAACTAGACTGAGCCTAGGGGAATAAATCTTTGGGCACCTAGTGGTCATG

3 CATGACCACTAGGAGCATCTTTGGCGACGAGAGTGGCCAAGCGGGACCGGGAGTCGACTAGGGGAATAAATCTTTGGGCACCTAGTGGTCATG

3 CATGACCACTAGGAGCATCTTTGGCGAGTAGGGGACTAAGCTCGGTCCCAGTGTCGGCTAGGGGAATAAATCTTTGGGCACCTAGTGGTCATG

3 CATGACCACTAGGAGCATCTTTGGCGAACGAGTGGTTATGAGCATCCGACACGTCGCCTAGGGGAATAAATCTTTGGGCACCTAGTGGTCATG

3 CATGACCACTAGGAGCATCTTTGGCGAGCCGTTCGCTACTTATTAGTACACATGCGACTAGGGGAATAAATCTTTGGGCACCTAGTGGTCATG

3 CATGACCACTAGGAGCATCTTTGGCGATCCGTTACCAGATGGTGGGCGAATATCGCGCTAGGGGAATAAATCTTTGGGCACCTAGTGGTCATG

3 CATGACCACTAGGAGCATCTTTGGCGACGCAGACTAGTTCCACCGGTAGAGTGCTAGCTAGGGGAATAAATCTTTGGGCACCTAGTGGTCATG

3 CATGACCACTAGGAGCATCTTTGGCGAACCGGCCAGGGTGACTGACGGTGCTACGCTCTAGGGGAATAAATCTTTGGGCACCTAGTGGTCATG

3 CATGACCACTAGGAGCATCTTTGGCGACGATCCGGGAGTGATAGACGGCGACGCGAGCTAGGGGAATAAATCTTTGGGCACCTAGTGGTCATG

3 CATGACCACTAGGAGCATCTTTGGCGAGTTCAATCTCCGAAGTGGATCGATAACTAGCTAGGGGAATAAATCTTTGGGCACCTAGTGGTCATG

3 CATGACCACTAGGAGCATCTTTGGCGACAGTAAACGTCAGTACGACGGAGGACTAAGCTAGGGGAATAAATCTTTGGGCACCTAGTGGTCATG

3 CATGACCACTAGGAGCATCTTTGGCGAATCGCGATGGGGCACTTATGCCGGATTCGACTAGGGGAATAAATCTTTGGGCACCTAGTGGTCATG

3 CATGACCACTAGGAGCATCTTTGGCGACTCGCTAGGGCGGTAAATGGGAGCGAGTTGCTAGGGGAATAAATCTTTGGGCACCTAGTGGTCATG

3 CATGACCACTAGGAGCATCTTTGGCGACTCGAGTGACGTGCTTATACCGAGTTCGCGCTAGGGGAATAAATCTTTGGGCACCTAGTGGTCATG

3 CATGACCACTAGGAGCATCTTTGGCGACGGGAAAAGTAATCTGTTGCGCAGGAGCCTCTAGGGGAATAAATCTTTGGGCACCTAGTGGTCATG

3 CATGACCACTAGGAGCATCTTTGGCGAGTGTGAGCGGCTGAGCTCATGCCACTAAGACTAGGGGAATAAATCTTTGGGCACCTAGTGGTCATG

3 CATGACCACTAGGAGCATCTTTGGCGAGGGGCTGCGCCGTGGTGCATCGCCAGACGCCTAGGGGAATAAATCTTTGGGCACCTAGTGGTCATG

3 CATGACCACTAGGAGCATCTTTGGCGAAGATAGTATATAACGATGATCATTTCTCGCCTAGGGGAATAAATCTTTGGGCACCTAGTGGTCATG

3 CATGACCACTAGGAGCATCTTTGGCGACCTCGCTAGCGGCAGGTGCAAGCTATGGAGCTAGGGGAATAAATCTTTGGGCACCTAGTGGTCATG

3 CATGACCACTAGGAGCATCTTTGGCGACAGCATATCGGAACGATAGCATTAGTGGAGCTAGGGGAATAAATCTTTGGGCACCTAGTGGTCATG

3 CATGACCACTAGGAGCATCTTTGGCGACGTGTTGAGCGGTGAGCGACAACAAGTGGACTAGGGGAATAAATCTTTGGGCACCTAGTGGTCATG

3 CATGACCACTAGGAGCATCTTTGGCGACTCGGCTTCGCGTTCCGTTCCGTCTACAGCCTAGGGGAATAAATCTTTGGGCACCTAGTGGTCATG

3 CATGACCACTAGGAGCATCTTTGGCGAACAACGGAGATAACGTGTATACGGGGACGCCTAGGGGAATAAATCTTTGGGCACCTAGTGGTCATG

3 CATGACCACTAGGAGCATCTTTGGCGACCTTCGTAGAGTACATCAGAGAAGACGCTCCTAGGGGAATAAATCTTTGGGCACCTAGTGGTCATG

3 CATGACCACTAGGAGCATCTTTGGCGAGCCGACGGGCACTGTGACTGGAGACGGCTACTAGGGGAATAAATCTTTGGGCACCTAGTGGTCATG

3 CATGACCACTAGGAGCATCTTTGGCGACTATTCCGGCTCCGTGTGACGGGAATCGTCCTAGGGGAATAAATCTTTGGGCACCTAGTGGTCATG

3 CATGACCACTAGGAGCATCTTTGGCGACGGTCATAGACATTCAAACAAATTGCACGCCTAGGGGAATAAATCTTTGGGCACCTAGTGGTCATG

3 CATGACCACTAGGAGCATCTTTGGCGACGTGGAGCTTACTCGCGCTGTACGAGCTAACTAGGGGAATAAATCTTTGGGCACCTAGTGGTCATG

3 CATGACCACTAGGAGCATCTTTGGCGACGGCATTGTGCAAACTCCCAGTGCTGGAGACTAGGGGAATAAATCTTTGGGCACCTAGTGGTCATG

3 CATGACCACTAGGAGCATCTTTGGCGACAGTGGGCGGACCGGGACCCAAGGGGCGGACTAGGGGAATAAATCTTTGGGCACCTAGTGGTCATG

3 CATGACCACTAGGAGCATCTTTGGCGAGACGTAGAACACGGGCAGGGTTATCTCGCACTAGGGGAATAAATCTTTGGGCACCTAGTGGTCATG

3 CATGACCACTAGGAGCATCTTTGGCGATGGCCCTACAGTTGTAGAGGGGGTCCGAGTCTAGGGGAATAAATCTTTGGGCACCTAGTGGTCATG

3 CATGACCACTAGGAGCATCTTTGGCGATTGGTCCATGACTAGAGATGATAGATTCGCCTAGGGGAATAAATCTTTGGGCACCTAGTGGTCATG

3 CATGACCACTAGGAGCATCTTTGGCGATGGCACAGTGCAACGTCCAATGCCAGTCACCTAGGGGAATAAATCTTTGGGCACCTAGTGGTCATG

3 CATGACCACTAGGAGCATCTTTGGCGAGCGGTCGGTGGTGTGGCGCAGGAGCGCAGTCTAGGGGAATAAATCTTTGGGCACCTAGTGGTCATG

3 CATGACCACTAGGAGCATCTTTGGCGAACGAGCACGCGCGGCAAGATAGATCGCCTACTAGGGGAATAAATCTTTGGGCACCTAGTGGTCATG

3 CATGACCACTAGGAGCATCTTTGGCGATTGGAAGTCCCCGCCCTAAGTGGTCAATCGCTAGGGGAATAAATCTTTGGGCACCTAGTGGTCATG

3 CATGACCACTAGGAGCATCTTTGGCGACGAGGGGTCGTTAGGATGCAAAGGAAGTCGCTAGGGGAATAAATCTTTGGGCACCTAGTGGTCATG

3 CATGACCACTAGGAGCATCTTTGGCGAAAGCGGAGTCTCGGTTCCAGATCCAGCCGCCTAGGGGAATAAATCTTTGGGCACCTAGTGGTCATG

3 CATGACCACTAGGAGCATCTTTGGCGACTATCCGCGCGGGCCTGAAGGAGGATCGAGCTAGGGGAATAAATCTTTGGGCACCTAGTGGTCATG

3 CATGACCACTAGGAGCATCTTTGGCGAGACACGGGCGGTTGGGCGTGACCGTGCGGCCTAGGGGAATAAATCTTTGGGCACCTAGTGGTCATG

3 CATGACCACTAGGAGCATCTTTGGCGATTCCACAGCTGGACCGGAGGACAGGAACGCCTAGGGGAATAAATCTTTGGGCACCTAGTGGTCATG

3 CATGACCACTAGGAGCATCTTTGGCGATGGGCCTCGGGCCGCAGGTTGGGAACGAGACTAGGGGAATAAATCTTTGGGCACCTAGTGGTCATG

3 CATGACCACTAGGAGCATCTTTGGCGACGGCTGGGGTTCATCCCTGTGGACGGCGAGCTAGGGGAATAAATCTTTGGGCACCTAGTGGTCATG

3 CATGACCACTAGGAGCATCTTTGGCGAAGTAGCATGAATATGTTAGTCGGCTTCGTACTAGGGGAATAAATCTTTGGGCACCTAGTGGTCATG

3 CATGACCACTAGGAGCATCTTTGGCGAGCCGTGCGGGTAACCCGAGCGACGGTGCGTCTAGGGGAATAAATCTTTGGGCACCTAGTGGTCATG

3 CATGACCACTAGGAGCATCTTTGGCGACGGAAGACGAACATACAGGAGTCTGAGCCACTAGGGGAATAAATCTTTGGGCACCTAGTGGTCATG

3 CATGACCACTAGGAGCATCTTTGGCGACCCACAGCGCAATGCCATGTGGCTGAACGCCTAGGGGAATAAATCTTTGGGCACCTAGTGGTCATG

3 CATGACCACTAGGAGCATCTTTGGCGAGTCATTGTGCGCGTCCAGGAGCATCGAGCCCTAGGGGAATAAATCTTTGGGCACCTAGTGGTCATG

3 CATGACCACTAGGAGCATCTTTGGCGAAGAGCAATGAAGTGGAGACTTTGGCTGGAGCTAGGGGAATAAATCTTTGGGCACCTAGTGGTCATG

3 CATGACCACTAGGAGCATCTTTGGCGACTGGAAGTGGGCAGGCAAATAGTCGTCGCTCTAGGGGAATAAATCTTTGGGCACCTAGTGGTCATG

3 CATGACCACTAGGAGCATCTTTGGCGAGGGGAAGAGGCCTGCAGCCTGTCCGGCGAGCTAGGGGAATAAATCTTTGGGCACCTAGTGGTCATG

3 CATGACCACTAGGAGCATCTTTGGCGATGTGGTGTCTAGGGAAGCGCGTAGCATCGCCTAGGGGAATAAATCTTTGGGCACCTAGTGGTCATG

3 CATGACCACTAGGAGCATCTTTGGCGACTCGAAGGGATAGCCGGGAATGAGTGTCGACTAGGGGAATAAATCTTTGGGCACCTAGTGGTCATG

3 CATGACCACTAGGAGCATCTTTGGCGACGCAAACCCTGATGGGGTATGTGGGACGCTCTAGGGGAATAAATCTTTGGGCACCTAGTGGTCATG

3 CATGACCACTAGGAGCATCTTTGGCGAATGACGATGTCCAGGCGACTGAGATTCTCGCTAGGGGAATAAATCTTTGGGCACCTAGTGGTCATG

3 CATGACCACTAGGAGCATCTTTGGCGAAGGATACTGCTTGGTGTCCAGATCTGATGCCTAGGGGAATAAATCTTTGGGCACCTAGTGGTCATG

3 CATGACCACTAGGAGCATCTTTGGCGAATCCGGTGTGAATCGGCCAGGGTAGATCGCCTAGGGGAATAAATCTTTGGGCACCTAGTGGTCATG

3 CATGACCACTAGGAGCATCTTTGGCGAGCCGACTGGGGGGCCAATGGTTGCGCGAGACTAGGGGAATAAATCTTTGGGCACCTAGTGGTCATG

3 CATGACCACTAGGAGCATCTTTGGCGACCTGGTGTGTGGCGTTCCTGTCGAAGAAGCCTAGGGGAATAAATCTTTGGGCACCTAGTGGTCATG

3 CATGACCACTAGGAGCATCTTTGGCGAGTCTGGGAGAATGGAGATGAGGTGACTCGACTAGGGGAATAAATCTTTGGGCACCTAGTGGTCATG

3 CATGACCACTAGGAGCATCTTTGGCGACCATAGCATGTCATCGGCGAGACGGGAGAACTAGGGGAATAAATCTTTGGGCACCTAGTGGTCATG

3 CATGACCACTAGGAGCATCTTTGGCGACGAACACATCATGGATCGTGCGCTCGCCGGCTAGGGGAATAAATCTTTGGGCACCTAGTGGTCATG

3 CATGACCACTAGGAGCATCTTTGGCGAAATTCTACATCGTAGTATTATTGGTATACGCTAGGGGAATAAATCTTTGGGCACCTAGTGGTCATG

3 CATGACCACTAGGAGCATCTTTGGCGAAGGAGCTACGCTCGTTGGGTGTAGTGCCAGCTAGGGGAATAAATCTTTGGGCACCTAGTGGTCATG

3 CATGACCACTAGGAGCATCTTTGGCGACGCGGGGTGACAGTGCCTTTAGTGGCTCGCCTAGGGGAATAAATCTTTGGGCACCTAGTGGTCATG

3 CATGACCACTAGGAGCATCTTTGGCGAGTCACGTGGACTCCATTGCACGTAACTTAGCTAGGGGAATAAATCTTTGGGCACCTAGTGGTCATG

3 CATGACCACTAGGAGCATCTTTGGCGACCGTTGGTAGACGGAGCACTTGTGCAGACACTAGGGGAATAAATCTTTGGGCACCTAGTGGTCATG

3 CATGACCACTAGGAGCATCTTTGGCGATCGTTTCAAGCATGGTCTGAGAACGCATACCTAGGGGAATAAATCTTTGGGCACCTAGTGGTCATG

3 CATGACCACTAGGAGCATCTTTGGCGACGCGGATTGGGAAGGAGGCTCCTCAGGCGCCTAGGGGAATAAATCTTTGGGCACCTAGTGGTCATG

3 CATGACCACTAGGAGCATCTTTGGCGATCCGGGGGTCAGATAAGACGGCGCGTTGGGCTAGGGGAATAAATCTTTGGGCACCTAGTGGTCATG

3 CATGACCACTAGGAGCATCTTTGGCGAACCGGTCCCGAGACGCCATTCTAAACAGTACTAGGGGAATAAATCTTTGGGCACCTAGTGGTCATG

3 CATGACCACTAGGAGCATCTTTGGCGACACGAAAGGGCCTAGGATACTGTGAGTCGACTAGGGGAATAAATCTTTGGGCACCTAGTGGTCATG

3 CATGACCACTAGGAGCATCTTTGGCGAAGGGAAGACTGCAGGGCAGTGTCTTTCCGCCTAGGGGAATAAATCTTTGGGCACCTAGTGGTCATG

3 CATGACCACTAGGAGCATCTTTGGCGAAGCGTAAACAGCGTACTTGGGGTGACTGTTCTAGGGGAATAAATCTTTGGGCACCTAGTGGTCATG

3 CATGACCACTAGGAGCATCTTTGGCGAGTAGACGGCGCGACTCACGCTTGTGTTCGCCTAGGGGAATAAATCTTTGGGCACCTAGTGGTCATG

3 CATGACCACTAGGAGCATCTTTGGCGAGCGGACCAGGGGGTCCTAAACAGGCGACGACTAGGGGAATAAATCTTTGGGCACCTAGTGGTCATG

3 CATGACCACTAGGAGCATCTTTGGCGAGTAGGTGGAACACTTGAACATTATCTTCGACTAGGGGAATAAATCTTTGGGCACCTAGTGGTCATG

3 CATGACCACTAGGAGCATCTTTGGCGAGGGCTTAATCGTTCTGTAGGGCACCCGAGCCTAGGGGAATAAATCTTTGGGCACCTAGTGGTCATG

3 CATGACCACTAGGAGCATCTTTGGCGATATCGAGCAAGGCTAGTATGTCAGATCGCTCTAGGGGAATAAATCTTTGGGCACCTAGTGGTCATG

3 CATGACCACTAGGAGCATCTTTGGCGATTTGACGTGAGGACGGTGTGATGTAGGTCGCTAGGGGAATAAATCTTTGGGCACCTAGTGGTCATG

3 CATGACCACTAGGAGCATCTTTGGCGACGATTGTTTCATCTGGGGGGCAGGCGTCACCTAGGGGAATAAATCTTTGGGCACCTAGTGGTCATG

3 CATGACCACTAGGAGCATCTTTGGCGACGTCGGGACGCAGTGCAGGGAAAGGGTCGACTAGGGGAATAAATCTTTGGGCACCTAGTGGTCATG

3 CATGACCACTAGGAGCATCTTTGGCGACAGCGTATGAACGGCTTGGAAGGCTGCGAACTAGGGGAATAAATCTTTGGGCACCTAGTGGTCATG

3 CATGACCACTAGGAGCATCTTTGGCGAGATCTACATCGGTACGCATGTGTATCACGGCTAGGGGAATAAATCTTTGGGCACCTAGTGGTCATG

3 CATGACCACTAGGAGCATCTTTGGCGAGAATCACAAATGCACTGCGTCTAGGGCCGCCTAGGGGAATAAATCTTTGGGCACCTAGTGGTCATG

3 CATGACCACTAGGAGCATCTTTGGCGAAGCATGGAGCGCTGACGAGACATGGACTGCCTAGGGGAATAAATCTTTGGGCACCTAGTGGTCATG

3 CATGACCACTAGGAGCATCTTTGGCGATGTTGAGAGCATTGCAGGCTCACTTCTAGCCTAGGGGAATAAATCTTTGGGCACCTAGTGGTCATG

3 CATGACCACTAGGAGCATCTTTGGCGACTTGTTGAACCACGTCCTGCGGGGTGCTCGCTAGGGGAATAAATCTTTGGGCACCTAGTGGTCATG

3 CATGACCACTAGGAGCATCTTTGGCGATACAATCGTTTGTGTCAAATGTGTAGCGATCTAGGGGAATAAATCTTTGGGCACCTAGTGGTCATG

3 CATGACCACTAGGAGCATCTTTGGCGAGGGTCGCGTACGGCCATGGGTTGAGGCCAGCTAGGGGAATAAATCTTTGGGCACCTAGTGGTCATG

3 CATGACCACTAGGAGCATCTTTGGCGACACGTAAAGAAAAGCAATTAGTTGCAGAGCCTAGGGGAATAAATCTTTGGGCACCTAGTGGTCATG

3 CATGACCACTAGGAGCATCTTTGGCGACGGTAAGTGGTCCGAGTATTGAACCGTCCGCTAGGGGAATAAATCTTTGGGCACCTAGTGGTCATG

3 CATGACCACTAGGAGCATCTTTGGCGAGCCTCTGCAAAAAGAATCGCGGAGGGACGCCTAGGGGAATAAATCTTTGGGCACCTAGTGGTCATG

3 CATGACCACTAGGAGCATCTTTGGCGATCCAGTGGTCCGAGATGGGTAGTGATCGCGCTAGGGGAATAAATCTTTGGGCACCTAGTGGTCATG

3 CATGACCACTAGGAGCATCTTTGGCGATCTGAGGTCTATTGTGTACCGGCGATCCGCCTAGGGGAATAAATCTTTGGGCACCTAGTGGTCATG

3 CATGACCACTAGGAGCATCTTTGGCGAGTGATGGTCTCGCTACGTGGAGGATACTCGCTAGGGGAATAAATCTTTGGGCACCTAGTGGTCATG

3 CATGACCACTAGGAGCATCTTTGGCGACAGGAGTAGGGCTGAAAGTACTCCGGTTGACTAGGGGAATAAATCTTTGGGCACCTAGTGGTCATG

3 CATGACCACTAGGAGCATCTTTGGCGATTTGCTACGCCAGACATGTTCCAACATCGACTAGGGGAATAAATCTTTGGGCACCTAGTGGTCATG

3 CATGACCACTAGGAGCATCTTTGGCGACGTGTGGGCTCGGCTAACTGTGGGGTTCGCCTAGGGGAATAAATCTTTGGGCACCTAGTGGTCATG

3 CATGACCACTAGGAGCATCTTTGGCGAGGTGTTTTGACTTCGTTGAGTCCGAGACATCTAGGGGAATAAATCTTTGGGCACCTAGTGGTCATG

3 CATGACCACTAGGAGCATCTTTGGCGACTAACATGGGGCATAAATGTGCTCGGCGCTCTAGGGGAATAAATCTTTGGGCACCTAGTGGTCATG

3 CATGACCACTAGGAGCATCTTTGGCGACCCTTCGGGGTGCAGTTTACTGCTTATTCGCTAGGGGAATAAATCTTTGGGCACCTAGTGGTCATG

3 CATGACCACTAGGAGCATCTTTGGCGAACACACACATAGAGGGGGACGAATGGGCGACTAGGGGAATAAATCTTTGGGCACCTAGTGGTCATG

3 CATGACCACTAGGAGCATCTTTGGCGACCGAGGACACGACGTTGGTGTTGGGCGCCGCTAGGGGAATAAATCTTTGGGCACCTAGTGGTCATG

3 CATGACCACTAGGAGCATCTTTGGCGACGGCGCGATCCTAGTTCCGTCCGTAGGAGCCTAGGGGAATAAATCTTTGGGCACCTAGTGGTCATG

3 CATGACCACTAGGAGCATCTTTGGCGACGATCCAGGGGCCCTGCGCCCCTTTGAGTCCTAGGGGAATAAATCTTTGGGCACCTAGTGGTCATG

3 CATGACCACTAGGAGCATCTTTGGCGACTGACTGTCACACTCGGGTACACTCGGAGACTAGGGGAATAAATCTTTGGGCACCTAGTGGTCATG

3 CATGACCACTAGGAGCATCTTTGGCGAGTCGGCGCAGCACAAACCAGTCAAGGCGAGCTAGGGGAATAAATCTTTGGGCACCTAGTGGTCATG

3 CATGACCACTAGGAGCATCTTTGGCGACGCGGTGAGTTTCGGTCGACCGCCTCGCTACTAGGGGAATAAATCTTTGGGCACCTAGTGGTCATG

3 CATGACCACTAGGAGCATCTTTGGCGAGACACGTAATGTGTGGAACCGGGATAGCGCCTAGGGGAATAAATCTTTGGGCACCTAGTGGTCATG

3 CATGACCACTAGGAGCATCTTTGGCGAGGCAGATATGTGACATTTACGTGTACGAGCCTAGGGGAATAAATCTTTGGGCACCTAGTGGTCATG

3 CATGACCACTAGGAGCATCTTTGGCGAGGCAGTGGCCGACCGGGTGTATGGTCGCCGCTAGGGGAATAAATCTTTGGGCACCTAGTGGTCATG

3 CATGACCACTAGGAGCATCTTTGGCGAGGCAACTAAGGGCGTGTGGACATGCCGATCCTAGGGGAATAAATCTTTGGGCACCTAGTGGTCATG

3 CATGACCACTAGGAGCATCTTTGGCGAGTTCTGTGACTCAAATGTCACTTTTTCGAGCTAGGGGAATAAATCTTTGGGCACCTAGTGGTCATG

3 CATGACCACTAGGAGCATCTTTGGCGACCGTGGGCATTAGGCTATACAGGTCCGCTGCTAGGGGAATAAATCTTTGGGCACCTAGTGGTCATG

3 CATGACCACTAGGAGCATCTTTGGCGAAGCAATACGGCCAGACGCGTATGTCTTTGGCTAGGGGAATAAATCTTTGGGCACCTAGTGGTCATG

3 CATGACCACTAGGAGCATCTTTGGCGATGGGGAGCTACTGCATATTGACTAAGCCAGCTAGGGGAATAAATCTTTGGGCACCTAGTGGTCATG

3 CATGACCACTAGGAGCATCTTTGGCGACCGGAAGATCAGAACCTAGCGCAAGGTCGACTAGGGGAATAAATCTTTGGGCACCTAGTGGTCATG

3 CATGACCACTAGGAGCATCTTTGGCGATGGCATCTGGCTAATGAGTCGTCCGTCGCGCTAGGGGAATAAATCTTTGGGCACCTAGTGGTCATG

3 CATGACCACTAGGAGCATCTTTGGCGAGATGCGACGGCGTGGTAGGTCAAGAATCGCCTAGGGGAATAAATCTTTGGGCACCTAGTGGTCATG

3 CATGACCACTAGGAGCATCTTTGGCGAGTGCTGACGTGGGAATTCCGGCGGGTCGAGCTAGGGGAATAAATCTTTGGGCACCTAGTGGTCATG

3 CATGACCACTAGGAGCATCTTTGGCGACGAAGGACCTTGGAACGAAAGAGAACGGCGCTAGGGGAATAAATCTTTGGGCACCTAGTGGTCATG

3 CATGACCACTAGGAGCATCTTTGGCGAAGGACTAAGTGTGAATCTGTGTACGGAGCACTAGGGGAATAAATCTTTGGGCACCTAGTGGTCATG

3 CATGACCACTAGGAGCATCTTTGGCGACGAGACAGGGGGCTCAGTGCTTAGTCGCCGCTAGGGGAATAAATCTTTGGGCACCTAGTGGTCATG

3 CATGACCACTAGGAGCATCTTTGGCGAGGTGCGGGCACGAGGAGCGGACCGCAGCCCCTAGGGGAATAAATCTTTGGGCACCTAGTGGTCATG

3 CATGACCACTAGGAGCATCTTTGGCGACCGAACTCTGTCGTTGGGGGACCAGGGAGACTAGGGGAATAAATCTTTGGGCACCTAGTGGTCATG

3 CATGACCACTAGGAGCATCTTTGGCGACTATGGCGGCGTCGTCAGAGTCAGCGCCTACTAGGGGAATAAATCTTTGGGCACCTAGTGGTCATG

3 CATGACCACTAGGAGCATCTTTGGCGAGTCGATGTAGTCAACGAGTAAAACGGTCGCCTAGGGGAATAAATCTTTGGGCACCTAGTGGTCATG

3 CATGACCACTAGGAGCATCTTTGGCGAACGACATAGTGGTCTCCGGGTGAGAGTGAGCTAGGGGAATAAATCTTTGGGCACCTAGTGGTCATG

3 CATGACCACTAGGAGCATCTTTGGCGAAGTGGTCCAGTGCTCCACTCGGGAGATCGCCTAGGGGAATAAATCTTTGGGCACCTAGTGGTCATG

3 CATGACCACTAGGAGCATCTTTGGCGACGGGAGGATCGCGGGCGTCATTCCTTCGCACTAGGGGAATAAATCTTTGGGCACCTAGTGGTCATG

3 CATGACCACTAGGAGCATCTTTGGCGACTGGTGGACTGCAACATGTGGCCCTCCGAGCTAGGGGAATAAATCTTTGGGCACCTAGTGGTCATG

3 CATGACCACTAGGAGCATCTTTGGCGAGGGCGCGTGGCGTGGGCGCCGCGGCCGGGCCTAGGGGAATAAATCTTTGGGCACCTAGTGGTCATG

3 CATGACCACTAGGAGCATCTTTGGCGAATGGGCGATGTGTGTCATGGACCCGGTAGACTAGGGGAATAAATCTTTGGGCACCTAGTGGTCATG

3 CATGACCACTAGGAGCATCTTTGGCGAGCGGACGGTGGGCATGCGCAGGGAAGCCGACTAGGGGAATAAATCTTTGGGCACCTAGTGGTCATG

3 CATGACCACTAGGAGCATCTTTGGCGAGCATCGGCCTGAGGAGACGGAAATGCGCCTCTAGGGGAATAAATCTTTGGGCACCTAGTGGTCATG

3 CATGACCACTAGGAGCATCTTTGGCGAGGGTAACGAGGCTTCTCTGGCTCCCGAGAACTAGGGGAATAAATCTTTGGGCACCTAGTGGTCATG

3 CATGACCACTAGGAGCATCTTTGGCGAATCAGACTGGTGTTTTTTATCATCGCTGCGCTAGGGGAATAAATCTTTGGGCACCTAGTGGTCATG

3 CATGACCACTAGGAGCATCTTTGGCGATCCGGGTGGGCAGCGCCATATATAGATCGACTAGGGGAATAAATCTTTGGGCACCTAGTGGTCATG

3 CATGACCACTAGGAGCATCTTTGGCGACACTCGAACTGGCACGTTCAGGAGAGCAAGCTAGGGGAATAAATCTTTGGGCACCTAGTGGTCATG

3 CATGACCACTAGGAGCATCTTTGGCGAATGAGTAATATTGGGACATGGATGGATCGCCTAGGGGAATAAATCTTTGGGCACCTAGTGGTCATG

3 CATGACCACTAGGAGCATCTTTGGCGACCGCCGCGTTGTTGAGCAGTAAGGTGCCAGCTAGGGGAATAAATCTTTGGGCACCTAGTGGTCATG

3 CATGACCACTAGGAGCATCTTTGGCGAAGGCACCGTTGATAAAAGGGCCCTACTCGACTAGGGGAATAAATCTTTGGGCACCTAGTGGTCATG

3 CATGACCACTAGGAGCATCTTTGGCGATGTTTGACGCTCTGCGGCGTTAGGGTACGCCTAGGGGAATAAATCTTTGGGCACCTAGTGGTCATG

3 CATGACCACTAGGAGCATCTTTGGCGATTAGTGCAAATTTATTTGCATCATTATGGTCTAGGGGAATAAATCTTTGGGCACCTAGTGGTCATG

3 CATGACCACTAGGAGCATCTTTGGCGAGTCAGGAAGGCGTATTCGCGGATGACTTGACTAGGGGAATAAATCTTTGGGCACCTAGTGGTCATG

3 CATGACCACTAGGAGCATCTTTGGCGAGATCCGTGACGAGCGAAGGTGTGCGACCTACTAGGGGAATAAATCTTTGGGCACCTAGTGGTCATG

3 CATGACCACTAGGAGCATCTTTGGCGAAGGTCCCTGTTCGACTGGGAGAGTCCTCGCCTAGGGGAATAAATCTTTGGGCACCTAGTGGTCATG

3 CATGACCACTAGGAGCATCTTTGGCGAGGAAGGAGCAAGGGATAGTGTGACGAGCCTCTAGGGGAATAAATCTTTGGGCACCTAGTGGTCATG

3 CATGACCACTAGGAGCATCTTTGGCGACGGGCGTAGGTCGCGTATAGAGCTGTGTCGCTAGGGGAATAAATCTTTGGGCACCTAGTGGTCATG

3 CATGACCACTAGGAGCATCTTTGGCGACCATCGGACGGGCAACGGCTGAGAATCGCTCTAGGGGAATAAATCTTTGGGCACCTAGTGGTCATG

3 CATGACCACTAGGAGCATCTTTGGCGACGGGCAGCTCCTGCAGAGGGAAGGCACGAGCTAGGGGAATAAATCTTTGGGCACCTAGTGGTCATG

3 CATGACCACTAGGAGCATCTTTGGCGACCGAGGAGGCCTAACAGGCCGGTGGATCGACTAGGGGAATAAATCTTTGGGCACCTAGTGGTCATG

3 CATGACCACTAGGAGCATCTTTGGCGAGACGAGGAGTCGTGCACGAGGGATCGAGCACTAGGGGAATAAATCTTTGGGCACCTAGTGGTCATG

3 CATGACCACTAGGAGCATCTTTGGCGAACGAGGCGGATCAAGGGTAGTCCGTTTGCCCTAGGGGAATAAATCTTTGGGCACCTAGTGGTCATG

3 CATGACCACTAGGAGCATCTTTGGCGATGTCGCCGGGTTGGGACTCCGGCGATAATCCTAGGGGAATAAATCTTTGGGCACCTAGTGGTCATG

3 CATGACCACTAGGAGCATCTTTGGCGATACCTCTACACTGGTACTGGGGTTGAAGCACTAGGGGAATAAATCTTTGGGCACCTAGTGGTCATG

3 CATGACCACTAGGAGCATCTTTGGCGAGTACGTGTAGCAAGAAGGTTGCCGTACGCGCTAGGGGAATAAATCTTTGGGCACCTAGTGGTCATG

3 CATGACCACTAGGAGCATCTTTGGCGATGAGGCCGTCGTGCTCCACAGGGAGTCATGCTAGGGGAATAAATCTTTGGGCACCTAGTGGTCATG

3 CATGACCACTAGGAGCATCTTTGGCGACGCGAGGGGGGAAGTGTGACCTCGCCGTGACTAGGGGAATAAATCTTTGGGCACCTAGTGGTCATG

3 CATGACCACTAGGAGCATCTTTGGCGAGGAAAAACCGACGTTTGAACATAGTTTAGCCTAGGGGAATAAATCTTTGGGCACCTAGTGGTCATG

3 CATGACCACTAGGAGCATCTTTGGCGAAGGAGGCTGTCCGAGACAGACATAGGGAGCCTAGGGGAATAAATCTTTGGGCACCTAGTGGTCATG

3 CATGACCACTAGGAGCATCTTTGGCGAACGGGCGGCGCGCGTGGATGCAGCGTTCGGCTAGGGGAATAAATCTTTGGGCACCTAGTGGTCATG

3 CATGACCACTAGGAGCATCTTTGGCGAAGATCGATCTTGCCTATAGATCTCGGCCAGCTAGGGGAATAAATCTTTGGGCACCTAGTGGTCATG

3 CATGACCACTAGGAGCATCTTTGGCGAGGGTCTTCCCCAGGCATGGGGATTTGGCGCCTAGGGGAATAAATCTTTGGGCACCTAGTGGTCATG

3 CATGACCACTAGGAGCATCTTTGGCGACTCGAGGAAAGGATTTACTCGAATGGCGACCTAGGGGAATAAATCTTTGGGCACCTAGTGGTCATG

3 CATGACCACTAGGAGCATCTTTGGCGATCTTATTTGTCGTCGAGACGACTGAGTCGACTAGGGGAATAAATCTTTGGGCACCTAGTGGTCATG

3 CATGACCACTAGGAGCATCTTTGGCGAGATCACGGATCCACATCTGTAAGTGCGCCGCTAGGGGAATAAATCTTTGGGCACCTAGTGGTCATG

3 CATGACCACTAGGAGCATCTTTGGCGACGAATCCGCGTGACAATGGTATTAGGGCGCCTAGGGGAATAAATCTTTGGGCACCTAGTGGTCATG
