# Supplementary material for: Selection of allosteric dnazymes that can sense phenylalanine by expression-SELEX
Source: Nucleic Acids Res. 2023 May 19;51(11):e66. doi: 10.1093/nar/gkad424 (PMC10287898; doi:10.1093/nar/gkad424)
Supplement: gkad424_Supplemental_Files [file gkad424_supplemental_files.zip › Supplementary file No. 3 count_most_represented_seqs_for_round-14.docx]

Note: total reads of the library are 698005. The reads are on the left, and the sequence is on the right.

318341 CATGACCACTAGGAGCATCTTTGGCGAGATCGGGAGAATCGGTGGCATTGGTGTCTCCTAGGGGAATAAATCTTTGGGCACCTAGTGGTCATG

146251 CATGACCACTAGGAGCATCTTTGGCGAACGGCAGGTGTTGCGGTGGTCTGTGAATCCCTAGGGGAATAAATCTTTGGGCACCTAGTGGTCATG

49546 CATGACCACTAGGAGCATCTTTGGCGAGAAGACTCTGGATTCGGGGACCAGTTGCTGCTAGGGGAATAAATCTTTGGGCACCTAGTGGTCATG

27395 CATGACCACTAGGAGCATCTTTGGCGAAAGTCGGTAGAACAGGGTGGGGTGCTGTCCCTAGGGGAATAAATCTTTGGGCACCTAGTGGTCATG

21702 CATGACCACTAGGAGCATCTTTGGCGAATGGCCGGCACGGCCTTCTAGTCCTCGGTACTAGGGGAATAAATCTTTGGGCACCTAGTGGTCATG

14861 CATGACCACTAGGAGCATCTTTGGCGAGCCTCGCTTGGGAGGTTGCTCCACCAGTTCCTAGGGGAATAAATCTTTGGGCACCTAGTGGTCATG

3765 CATGACCACTAGGAGCATCTTTGGCGATCCTCGTCAGATGGTGAAGCAGACGTTTGGCTAGGGGAATAAATCTTTGGGCACCTAGTGGTCATG

3423 CATGACCACTAGGAGCATCTTTGGCGATGGGGCCGTGCTATCTGCACACTCCGCGGGCTAGGGGAATAAATCTTTGGGCACCTAGTGGTCATG

3298 CATGACCACTAGGAGCATCTTTGGCGATGGGTGCTGACGGCCGCCGCTGCGGCTACACTAGGGGAATAAATCTTTGGGCACCTAGTGGTCATG

3205 CATGACCACTAGGAGCATCTTTGGCGAAGCGTGTTGCAAGGTCCGCGAGTGTGGACCCTAGGGGAATAAATCTTTGGGCACCTAGTGGTCATG

2744 CATGACCACTAGGAGCATCTTTGGCGAAGGGTGTAGGACTTCAAGTGGATCTCATAGCTAGGGGAATAAATCTTTGGGCACCTAGTGGTCATG

2556 CATGACCACTAGGAGCATCTTTGGCGAGGGAGGGCGCCGGCAGCGGTGTGAATGCGCCTAGGGGAATAAATCTTTGGGCACCTAGTGGTCATG

2376 CATGACCACTAGGAGCATCTTTGGCGAGCCTTGCTTGGGAGGTTGCTCCACCAGTTCCTAGGGGAATAAATCTTTGGGCACCTAGTGGTCATG

2267 CATGACCACTAGGAGCATCTTTGGCGAGATCGGGAGAATCGGCGGCATTGGTGTCTCCTAGGGGAATAAATCTTTGGGCACCTAGTGGTCATG

2241 CATGACCACTAGGAGCATCTTTGGCGATCAGGCCGCCAGGGCGTACGAAGTGTGGTTCTAGGGGAATAAATCTTTGGGCACCTAGTGGTCATG

1577 CATGACCACTAGGAGCATCTTTGGCGACGGCGTGGGGCCATCTGTGCGGCGGATCCCCTAGGGGAATAAATCTTTGGGCACCTAGTGGTCATG

1503 CATGACCACTAGGAGCATCTTTGGCGAACGGCAGGTGTTGCGGTGGTTTGTGAATCCCTAGGGGAATAAATCTTTGGGCACCTAGTGGTCATG

1460 CATGACCACTAGGAGCATCTTTGGCGACGGTGTGGGGAACTTGTTTCGGCGGTGCTACTAGGGGAATAAATCTTTGGGCACCTAGTGGTCATG

1434 CATGACCACTAGGAGCATCTTTGGCGATGGGTGCTGACGGGGCTAGTGAGCCCTACACTAGGGGAATAAATCTTTGGGCACCTAGTGGTCATG

1341 CATGACCACTAGGAGCATCTTTGGCGAACGTGCAAGATATGCCGCCTGGAGCGTAGGCTAGGGGAATAAATCTTTGGGCACCTAGTGGTCATG

1282 CATGACCACTAGGAGCATCTTTGGCGAATGGCCGGCACGGCCTTCTAGTCCTTGGTACTAGGGGAATAAATCTTTGGGCACCTAGTGGTCATG

1281 CATGACCACTAGGAGCATCTTTGGCGACAGGGGATGCCGATGCGCTGGACGACCGAGCTAGGGGAATAAATCTTTGGGCACCTAGTGGTCATG

1280 CATGACCACTAGGAGCATCTTTGGCGAGGCGAGTCCAGCGGCCTTCTTGGCCTGCGACTAGGGGAATAAATCTTTGGGCACCTAGTGGTCATG

1085 CATGACCACTAGGAGCATCTTTGGCGACGGCGTGGGGCTATTGATGCGGCGGCACTCCTAGGGGAATAAATCTTTGGGCACCTAGTGGTCATG

1045 CATGACCACTAGGAGCATCTTTGGCGAACGGCAGGTGTTGCGGTGGTCTGTGGATCCCTAGGGGAATAAATCTTTGGGCACCTAGTGGTCATG

972 CATGACCACTAGGAGCATCTTTGGCGAGTATTGGCTGGTAGGTTGCGTATTGGGGAGCTAGGGGAATAAATCTTTGGGCACCTAGTGGTCATG

932 CATGACCACTAGGAGCATCTTTGGCGATAGGCGTGTACGGTCGAGCGAAAGCGATAGCTAGGGGAATAAATCTTTGGGCACCTAGTGGTCATG

910 CATGACCACTAGGAGCATCTTTGGCGAGATCGGGAGAATCGGTGGCATCGGTGTCTCCTAGGGGAATAAATCTTTGGGCACCTAGTGGTCATG

845 CATGACCACTAGGAGCATCTTTGGCGACCGTAGGAAAACAAAAGTGGCCTTGCTAGCCTAGGGGAATAAATCTTTGGGCACCTAGTGGTCATG

814 CATGACCACTAGGAGCATCTTTGGCGAGTGGGGGGCGACGGCCGGTAGTGGGTGAGACTAGGGGAATAAATCTTTGGGCACCTAGTGGTCATG

807 CATGACCACTAGGAGCATCTTTGGCGAGGGGTAGGGGGCCGCTCGCGCCTGCGTGCGCTAGGGGAATAAATCTTTGGGCACCTAGTGGTCATG

799 CATGACCACTAGGAGCATCTTTGGCGAGATCAGGAGAATCGGTGGCATTGGTGTCTCCTAGGGGAATAAATCTTTGGGCACCTAGTGGTCATG

706 CATGACCACTAGGAGCATCTTTGGCGAGATCGTGAGAATCGGTGGCATTGGTGTCTCCTAGGGGAATAAATCTTTGGGCACCTAGTGGTCATG

667 CATGACCACTAGGAGCATCTTTGGCGAAGCAGTGCGACGGGCTATGGGTTCCTCGAGCTAGGGGAATAAATCTTTGGGCACCTAGTGGTCATG

605 CATGACCACTAGGAGCATCTTTGGCGAGATCGAGAGAATCGGTGGCATTGGTGTCTCCTAGGGGAATAAATCTTTGGGCACCTAGTGGTCATG

582 CATGACCACTAGGAGCATCTTTGGCGAGAAGACTTTGGATTCGGGGACCAGTTGCTGCTAGGGGAATAAATCTTTGGGCACCTAGTGGTCATG

547 CATGACCACTAGGAGCATCTTTGGCGACGGTGTGGGGACCGCAGGTTCGGCGGGTACCTAGGGGAATAAATCTTTGGGCACCTAGTGGTCATG

536 CATGACCACTAGGAGCATCTTTGGCGACAGCGGCGTAGGCTGCCTTGGGTGGTGGCCCTAGGGGAATAAATCTTTGGGCACCTAGTGGTCATG

530 CATGACCACTAGGAGCATCTTTGGCGAACGGCAGGTGTTGCGGTGGTCTGTGTATCCCTAGGGGAATAAATCTTTGGGCACCTAGTGGTCATG

504 CATGACCACTAGGAGCATCTTTGGCGAGCCGATTGTGCAACAATGTGTTCTGGGTAACTAGGGGAATAAATCTTTGGGCACCTAGTGGTCATG

488 CATGACCACTAGGAGCATCTTTGGCGAGGTTGTAGCTCGCGCGTAGGGTCGGCAGTGCTAGGGGAATAAATCTTTGGGCACCTAGTGGTCATG

488 CATGACCACTAGGAGCATCTTTGGCGAGAAGACTCTGGATTCGGGGACCAGTTGCTCCTAGGGGAATAAATCTTTGGGCACCTAGTGGTCATG

469 CATGACCACTAGGAGCATCTTTGGCGAGGAGTGGCTGAGGGCGGTGGGTAGGTCGCGCTAGGGGAATAAATCTTTGGGCACCTAGTGGTCATG

465 CATGACCACTAGGAGCATCTTTGGCGAGATCGGGGGAATCGGTGGCATTGGTGTCTCCTAGGGGAATAAATCTTTGGGCACCTAGTGGTCATG

465 CATGACCACTAGGAGCATCTTTGGCGAACGGCAGGTGTTGCGGTGGTCTGTGAACCCCTAGGGGAATAAATCTTTGGGCACCTAGTGGTCATG

460 CATGACCACTAGGAGCATCTTTGGCGAGATCGGGAGAATCGGTGGCATTGGTGTCCCCTAGGGGAATAAATCTTTGGGCACCTAGTGGTCATG

458 CATGACCACTAGGAGCATCTTTGGCGAGGGGTCATGGGAATGTAATTGCGCTTAAGACTAGGGGAATAAATCTTTGGGCACCTAGTGGTCATG

438 CATGACCACTAGGAGCATCTTTGGCGAGATCGCGAGAATCGGTGGCATTGGTGTCTCCTAGGGGAATAAATCTTTGGGCACCTAGTGGTCATG

429 CATGACCACTAGGAGCATCTTTGGCGAGATCGGGAGAATCGGTGGCATTGGTGCCTCCTAGGGGAATAAATCTTTGGGCACCTAGTGGTCATG

421 CATGACCACTAGGAGCATCTTTGGCGATAGTCGGTAGAACAGGGTGGGGTGCTGTCCCTAGGGGAATAAATCTTTGGGCACCTAGTGGTCATG

390 CATGACCACTAGGAGCATCTTTGGCGAGATCGGGAGAATCGGTGGCATTGGCGTCTCCTAGGGGAATAAATCTTTGGGCACCTAGTGGTCATG

389 CATGACCACTAGGAGCATCTTTGGCGAGATCTGGAGAATCGGTGGCATTGGTGTCTCCTAGGGGAATAAATCTTTGGGCACCTAGTGGTCATG

383 CATGACCACTAGGAGCATCTTTGGCGATTTGGGAGTCGCAGTATGCAGGCAGTGAGACTAGGGGAATAAATCTTTGGGCACCTAGTGGTCATG

377 CATGACCACTAGGAGCATCTTTGGCGAACGGCAGGTGTTGCGGTGGTCTATGAATCCCTAGGGGAATAAATCTTTGGGCACCTAGTGGTCATG

373 CATGACCACTAGGAGCATCTTTGGCGAACGGCAGGTGTTGCGGTGGTCCGTGAATCCCTAGGGGAATAAATCTTTGGGCACCTAGTGGTCATG

368 CATGACCACTAGGAGCATCTTTGGCGAGAAGACTCCGGATTCGGGGACCAGTTGCTGCTAGGGGAATAAATCTTTGGGCACCTAGTGGTCATG

341 CATGACCACTAGGAGCATCTTTGGCGACGACTTCGGAGTGGGTGCGCCGCTGGCATACTAGGGGAATAAATCTTTGGGCACCTAGTGGTCATG

331 CATGACCACTAGGAGCATCTTTGGCGAGATCGGGAGGATCGGTGGCATTGGTGTCTCCTAGGGGAATAAATCTTTGGGCACCTAGTGGTCATG

326 CATGACCACTAGGAGCATCTTTGGCGACGAGATTTGGTAGGTAGATGTGCTCTAGCACTAGGGGAATAAATCTTTGGGCACCTAGTGGTCATG

320 CATGACCACTAGGAGCATCTTTGGCGAACGGCAGGTGTTGCGGTGGTCTGTGAAACCCTAGGGGAATAAATCTTTGGGCACCTAGTGGTCATG

317 CATGACCACTAGGAGCATCTTTGGCGAGAAGACCCTGGATTCGGGGACCAGTTGCTGCTAGGGGAATAAATCTTTGGGCACCTAGTGGTCATG

295 CATGACCACTAGGAGCATCTTTGGCGAGATCGGGAGAATCGGTGGCATTGATGTCTCCTAGGGGAATAAATCTTTGGGCACCTAGTGGTCATG

295 CATGACCACTAGGAGCATCTTTGGCGACAGCCGTAGCCGTAAGGTTGGTATAAGGCCCTAGGGGAATAAATCTTTGGGCACCTAGTGGTCATG

295 CATGACCACTAGGAGCATCTTTGGCGAGATCGGGAGAGTCGGTGGCATTGGTGTCTCCTAGGGGAATAAATCTTTGGGCACCTAGTGGTCATG

290 CATGACCACTAGGAGCATCTTTGGCGAATGGCCGGCACGGCCTCCTAGTCCTCGGTACTAGGGGAATAAATCTTTGGGCACCTAGTGGTCATG

284 CATGACCACTAGGAGCATCTTTGGCGAGATCGGGAGAATCGGTGGCATTGTTGTCTCCTAGGGGAATAAATCTTTGGGCACCTAGTGGTCATG

283 CATGACCACTAGGAGCATCTTTGGCGAGATCGGGAGAATCGGAGGCATTGGTGTCTCCTAGGGGAATAAATCTTTGGGCACCTAGTGGTCATG

273 CATGACCACTAGGAGCATCTTTGGCGAAGATCCGATGGGCTTGTGACCCGCCTCGGCCTAGGGGAATAAATCTTTGGGCACCTAGTGGTCATG

271 CATGACCACTAGGAGCATCTTTGGCGAGATCGGTAGAATCGGTGGCATTGGTGTCTCCTAGGGGAATAAATCTTTGGGCACCTAGTGGTCATG

267 CATGACCACTAGGAGCATCTTTGGCGACTCCAGTTCATCTGTCGGGCAGTTTAAGGACTAGGGGAATAAATCTTTGGGCACCTAGTGGTCATG

263 CATGACCACTAGGAGCATCTTTGGCGAGCGGCAGGTGTTGCGGTGGTCTGTGAATCCCTAGGGGAATAAATCTTTGGGCACCTAGTGGTCATG

248 CATGACCACTAGGAGCATCTTTGGCGAGATCGGAAGAATCGGTGGCATTGGTGTCTCCTAGGGGAATAAATCTTTGGGCACCTAGTGGTCATG

232 CATGACCACTAGGAGCATCTTTGGCGAGATCGGGAGAATCGGTGGCAGTGGTGTCTCCTAGGGGAATAAATCTTTGGGCACCTAGTGGTCATG

231 CATGACCACTAGGAGCATCTTTGGCGAGTTGTCGTAGAGCCACGGGACACCCGTCAGCTAGGGGAATAAATCTTTGGGCACCTAGTGGTCATG

228 CATGACCACTAGGAGCATCTTTGGCGAGGTCGGGAGAATCGGTGGCATTGGTGTCTCCTAGGGGAATAAATCTTTGGGCACCTAGTGGTCATG

226 CATGACCACTAGGAGCATCTTTGGCGAGCAAGCGCGGTCCCTCGGACCTCACAGTTGCTAGGGGAATAAATCTTTGGGCACCTAGTGGTCATG

226 CATGACCACTAGGAGCATCTTTGGCGAACGGCCGGCACGGCCTTCTAGTCCTCGGTACTAGGGGAATAAATCTTTGGGCACCTAGTGGTCATG

225 CATGACCACTAGGAGCATCTTTGGCGAGATCGGGAGAATCGGTGGCGTTGGTGTCTCCTAGGGGAATAAATCTTTGGGCACCTAGTGGTCATG

223 CATGACCACTAGGAGCATCTTTGGCGAAAGCGCAGGGCATGCGACCTCCAATTGGTCCTAGGGGAATAAATCTTTGGGCACCTAGTGGTCATG

221 CATGACCACTAGGAGCATCTTTGGCGAACGGCAGGGGTTGCGGTGGTCTGTGAATCCCTAGGGGAATAAATCTTTGGGCACCTAGTGGTCATG

221 CATGACCACTAGGAGCATCTTTGGCGATCAGCATAGCATATGCGCCTGGAGCGTAGGCTAGGGGAATAAATCTTTGGGCACCTAGTGGTCATG

220 CATGACCACTAGGAGCATCTTTGGCGATGGGTGCAGACGAGTGCGCTTGCAATTACACTAGGGGAATAAATCTTTGGGCACCTAGTGGTCATG

219 CATGACCACTAGGAGCATCTTTGGCGATGACGTCGTCGTAAATAGAGGCCTCGCGACCTAGGGGAATAAATCTTTGGGCACCTAGTGGTCATG

218 CATGACCACTAGGAGCATCTTTGGCGAGATCGGGAGAATCGGTGGCATAGGTGTCTCCTAGGGGAATAAATCTTTGGGCACCTAGTGGTCATG

216 CATGACCACTAGGAGCATCTTTGGCGAGACCGGGAGAATCGGTGGCATTGGTGTCTCCTAGGGGAATAAATCTTTGGGCACCTAGTGGTCATG

214 CATGACCACTAGGAGCATCTTTGGCGAGATAGGGAGAATCGGTGGCATTGGTGTCTCCTAGGGGAATAAATCTTTGGGCACCTAGTGGTCATG

209 CATGACCACTAGGAGCATCTTTGGCGAGATCGGGAGAATCGGTGGAATTGGTGTCTCCTAGGGGAATAAATCTTTGGGCACCTAGTGGTCATG

208 CATGACCACTAGGAGCATCTTTGGCGACGTAGTGGGCTGGCAGGGAGTACTTGTTGCCTAGGGGAATAAATCTTTGGGCACCTAGTGGTCATG

208 CATGACCACTAGGAGCATCTTTGGCGAATGGCAGGTGTTGCGGTGGTCTGTGAATCCCTAGGGGAATAAATCTTTGGGCACCTAGTGGTCATG

207 CATGACCACTAGGAGCATCTTTGGCGAGATCGGGAGAATCGGTGGCACTGGTGTCTCCTAGGGGAATAAATCTTTGGGCACCTAGTGGTCATG

206 CATGACCACTAGGAGCATCTTTGGCGAACCTGTACGGTGATGAGGTCGGTATCAGCCCTAGGGGAATAAATCTTTGGGCACCTAGTGGTCATG

205 CATGACCACTAGGAGCATCTTTGGCGAACAGGCCGTGGCTGCGGGTGGGTGCGCACCCTAGGGGAATAAATCTTTGGGCACCTAGTGGTCATG

204 CATGACCACTAGGAGCATCTTTGGCGATACAGGTGATCGGTGCTCGGGTGCCTGGCCCTAGGGGAATAAATCTTTGGGCACCTAGTGGTCATG

204 CATGACCACTAGGAGCATCTTTGGCGAACGGTAGGTGTTGCGGTGGTCTGTGAATCCCTAGGGGAATAAATCTTTGGGCACCTAGTGGTCATG

198 CATGACCACTAGGAGCATCTTTGGCGAAGACGACACAGTCGATCGGGATAGAGGAAGCTAGGGGAATAAATCTTTGGGCACCTAGTGGTCATG

195 CATGACCACTAGGAGCATCTTTGGCGAACACGAGAGTCAAGTGCGTATAGCGTGGAGCTAGGGGAATAAATCTTTGGGCACCTAGTGGTCATG

194 CATGACCACTAGGAGCATCTTTGGCGAGATTGGGAGAATCGGTGGCATTGGTGTCTCCTAGGGGAATAAATCTTTGGGCACCTAGTGGTCATG

190 CATGACCACTAGGAGCATCTTTGGCGAGATCGGGAGAATCGTTGGCATTGGTGTCTCCTAGGGGAATAAATCTTTGGGCACCTAGTGGTCATG

189 CATGACCACTAGGAGCATCTTTGGCGAGTTTCGCTGTGCTTGGAATGGGGCCACCCTCTAGGGGAATAAATCTTTGGGCACCTAGTGGTCATG

189 CATGACCACTAGGAGCATCTTTGGCGAGAGCTTGAAAAAGGTGCGTTGTTGTCGCATCTAGGGGAATAAATCTTTGGGCACCTAGTGGTCATG

188 CATGACCACTAGGAGCATCTTTGGCGAGATCGGGAGAATCTGTGGCATTGGTGTCTCCTAGGGGAATAAATCTTTGGGCACCTAGTGGTCATG

186 CATGACCACTAGGAGCATCTTTGGCGAGATCGGGAGAATCGGTGGCATTGGTGTCTACTAGGGGAATAAATCTTTGGGCACCTAGTGGTCATG

178 CATGACCACTAGGAGCATCTTTGGCGAACGGCAGGTGTTGCGGTGGTCTGTGAATCACTAGGGGAATAAATCTTTGGGCACCTAGTGGTCATG

173 CATGACCACTAGGAGCATCTTTGGCGAGATCGGCAGAATCGGTGGCATTGGTGTCTCCTAGGGGAATAAATCTTTGGGCACCTAGTGGTCATG

172 CATGACCACTAGGAGCATCTTTGGCGAGATCGGGAGAATCGGTGGCATTGCTGTCTCCTAGGGGAATAAATCTTTGGGCACCTAGTGGTCATG

172 CATGACCACTAGGAGCATCTTTGGCGATGGGTTGCTGGTAGGTGGTACGACCTCGATCTAGGGGAATAAATCTTTGGGCACCTAGTGGTCATG

162 CATGACCACTAGGAGCATCTTTGGCGAGATCGGGAGAATAGGTGGCATTGGTGTCTCCTAGGGGAATAAATCTTTGGGCACCTAGTGGTCATG

160 CATGACCACTAGGAGCATCTTTGGCGAGATCGGGAGAATCAGTGGCATTGGTGTCTCCTAGGGGAATAAATCTTTGGGCACCTAGTGGTCATG

160 CATGACCACTAGGAGCATCTTTGGCGAGATCGGGAGAATCGGTGTCATTGGTGTCTCCTAGGGGAATAAATCTTTGGGCACCTAGTGGTCATG

155 CATGACCACTAGGAGCATCTTTGGCGACGGCGTGGGGCCATTTGTGCGGCGGATCCCCTAGGGGAATAAATCTTTGGGCACCTAGTGGTCATG

154 CATGACCACTAGGAGCATCTTTGGCGAATGGCCGGTACGGCCTTCTAGTCCTCGGTACTAGGGGAATAAATCTTTGGGCACCTAGTGGTCATG

152 CATGACCACTAGGAGCATCTTTGGCGATAGCGAAGTGCTGTGAACAGTGGTTAGCACCTAGGGGAATAAATCTTTGGGCACCTAGTGGTCATG

152 CATGACCACTAGGAGCATCTTTGGCGACGTCGGCTGGTAGAGACTGTATTGAGGAACCTAGGGGAATAAATCTTTGGGCACCTAGTGGTCATG

152 CATGACCACTAGGAGCATCTTTGGCGAGATCGGGAGAATCGGTGGCATTGGTGTCTTCTAGGGGAATAAATCTTTGGGCACCTAGTGGTCATG

150 CATGACCACTAGGAGCATCTTTGGCGAAGTGCGGTGGGCATGTGGGTGGTAGCGACGCTAGGGGAATAAATCTTTGGGCACCTAGTGGTCATG

149 CATGACCACTAGGAGCATCTTTGGCGATATCGGGAGAATCGGTGGCATTGGTGTCTCCTAGGGGAATAAATCTTTGGGCACCTAGTGGTCATG

148 CATGACCACTAGGAGCATCTTTGGCGAGATCGGGAGAATCGGTGGCATTGGTGACTCCTAGGGGAATAAATCTTTGGGCACCTAGTGGTCATG

147 CATGACCACTAGGAGCATCTTTGGCGAGATCGGGAGAATCGGTGGCATTTGTGTCTCCTAGGGGAATAAATCTTTGGGCACCTAGTGGTCATG

147 CATGACCACTAGGAGCATCTTTGGCGAGATCGGGAGAAACGGTGGCATTGGTGTCTCCTAGGGGAATAAATCTTTGGGCACCTAGTGGTCATG

146 CATGACCACTAGGAGCATCTTTGGCGAGATCGGGAGAATCGGTGGTATTGGTGTCTCCTAGGGGAATAAATCTTTGGGCACCTAGTGGTCATG

143 CATGACCACTAGGAGCATCTTTGGCGACCGAGTGGGCTGGGAGGAAGTATTTCGGAGCTAGGGGAATAAATCTTTGGGCACCTAGTGGTCATG

140 CATGACCACTAGGAGCATCTTTGGCGACGTTATGAGTTGGGAAGCCTGGCGGCCAGACTAGGGGAATAAATCTTTGGGCACCTAGTGGTCATG

140 CATGACCACTAGGAGCATCTTTGGCGAGATCGGGAGAATCGGTGGCATTGGTGTATCCTAGGGGAATAAATCTTTGGGCACCTAGTGGTCATG

140 CATGACCACTAGGAGCATCTTTGGCGAACGGCAGGTGTTGCGGTGGCCTGTGAATCCCTAGGGGAATAAATCTTTGGGCACCTAGTGGTCATG

140 CATGACCACTAGGAGCATCTTTGGCGAACGGCAGGTGTTGCGGTGGTCTGCGAATCCCTAGGGGAATAAATCTTTGGGCACCTAGTGGTCATG

139 CATGACCACTAGGAGCATCTTTGGCGAACGGCAGGTGTTGCGGTGGTCTGTGAGTCCCTAGGGGAATAAATCTTTGGGCACCTAGTGGTCATG

139 CATGACCACTAGGAGCATCTTTGGCGAAGCGTGTTGCGAGGTCCGCGAGTGTGGACCCTAGGGGAATAAATCTTTGGGCACCTAGTGGTCATG

138 CATGACCACTAGGAGCATCTTTGGCGAACGGCAGGTGCTGCGGTGGTCTGTGAATCCCTAGGGGAATAAATCTTTGGGCACCTAGTGGTCATG

137 CATGACCACTAGGAGCATCTTTGGCGAACGGCGGGTGTTGCGGTGGTCTGTGAATCCCTAGGGGAATAAATCTTTGGGCACCTAGTGGTCATG

136 CATGACCACTAGGAGCATCTTTGGCGAGATCGGGAGAATCGGTGGCATTAGTGTCTCCTAGGGGAATAAATCTTTGGGCACCTAGTGGTCATG

135 CATGACCACTAGGAGCATCTTTGGCGAAATCGGGAGAATCGGTGGCATTGGTGTCTCCTAGGGGAATAAATCTTTGGGCACCTAGTGGTCATG

135 CATGACCACTAGGAGCATCTTTGGCGAGATCGGGAGAATCGGTGGCATTGGAGTCTCCTAGGGGAATAAATCTTTGGGCACCTAGTGGTCATG

133 CATGACCACTAGGAGCATCTTTGGCGACGATGGTTGGGAGGGATGTATTTCGGGCGACTAGGGGAATAAATCTTTGGGCACCTAGTGGTCATG

133 CATGACCACTAGGAGCATCTTTGGCGAGATCGGGAGACTCGGTGGCATTGGTGTCTCCTAGGGGAATAAATCTTTGGGCACCTAGTGGTCATG

133 CATGACCACTAGGAGCATCTTTGGCGAGGTGTCTCAGTTCGACATAAGGGGCGAGCACTAGGGGAATAAATCTTTGGGCACCTAGTGGTCATG

131 CATGACCACTAGGAGCATCTTTGGCGAACGGCAGGTGTTGCGGTGGTCTTTGAATCCCTAGGGGAATAAATCTTTGGGCACCTAGTGGTCATG

130 CATGACCACTAGGAGCATCTTTGGCGAGATCGGGAGAACCGGTGGCATTGGTGTCTCCTAGGGGAATAAATCTTTGGGCACCTAGTGGTCATG

128 CATGACCACTAGGAGCATCTTTGGCGAGAGTCGGTAGAACAGGGTGGGGTGCTGTCCCTAGGGGAATAAATCTTTGGGCACCTAGTGGTCATG

127 CATGACCACTAGGAGCATCTTTGGCGATTAGGCCGCCAGGGCGTACGAAGTGTGGTTCTAGGGGAATAAATCTTTGGGCACCTAGTGGTCATG

127 CATGACCACTAGGAGCATCTTTGGCGAACGGCAGGCGTTGCGGTGGTCTGTGAATCCCTAGGGGAATAAATCTTTGGGCACCTAGTGGTCATG

127 CATGACCACTAGGAGCATCTTTGGCGAGATCGGGAGAATCGGTGGCATTGGTTTCTCCTAGGGGAATAAATCTTTGGGCACCTAGTGGTCATG

126 CATGACCACTAGGAGCATCTTTGGCGAGGGTCCCTAGGGCCCTATAGAGTTTGTGAGCTAGGGGAATAAATCTTTGGGCACCTAGTGGTCATG

121 CATGACCACTAGGAGCATCTTTGGCGAGATCGGGATAATCGGTGGCATTGGTGTCTCCTAGGGGAATAAATCTTTGGGCACCTAGTGGTCATG

121 CATGACCACTAGGAGCATCTTTGGCGAATAGGCCGTGGCTGCGGGTGGGTGCGCACCCTAGGGGAATAAATCTTTGGGCACCTAGTGGTCATG

121 CATGACCACTAGGAGCATCTTTGGCGACTGGATACGGAAGGCAAGTGTGCCACTAGCCTAGGGGAATAAATCTTTGGGCACCTAGTGGTCATG

121 CATGACCACTAGGAGCATCTTTGGCGAGATCGGGAGAATCGGTAGCATTGGTGTCTCCTAGGGGAATAAATCTTTGGGCACCTAGTGGTCATG

120 CATGACCACTAGGAGCATCTTTGGCGACGGTGTGGGTGCAGGGCCAGGCGGTTGATCCTAGGGGAATAAATCTTTGGGCACCTAGTGGTCATG

119 CATGACCACTAGGAGCATCTTTGGCGAAGTGTGGAACATGCCGGTTTTGGTTGCACCCTAGGGGAATAAATCTTTGGGCACCTAGTGGTCATG

118 CATGACCACTAGGAGCATCTTTGGCGAGATCGGGAGAATCGGTGGCATTGGTGTCACCTAGGGGAATAAATCTTTGGGCACCTAGTGGTCATG

118 CATGACCACTAGGAGCATCTTTGGCGAGATCGGGAGAATCGGTGGCATTGGTATCTCCTAGGGGAATAAATCTTTGGGCACCTAGTGGTCATG

116 CATGACCACTAGGAGCATCTTTGGCGAACGGAAGGTGTTGCGGTGGTCTGTGAATCCCTAGGGGAATAAATCTTTGGGCACCTAGTGGTCATG

116 CATGACCACTAGGAGCATCTTTGGCGAACGGCAGGTGTCGCGGTGGTCTGTGAATCCCTAGGGGAATAAATCTTTGGGCACCTAGTGGTCATG

116 CATGACCACTAGGAGCATCTTTGGCGAGCTCTACGCGTCCTTGAGTGCCGACGTACACTAGGGGAATAAATCTTTGGGCACCTAGTGGTCATG

115 CATGACCACTAGGAGCATCTTTGGCGAGTGTTGCCCGGTACACGAGCAGACGTTTGGCTAGGGGAATAAATCTTTGGGCACCTAGTGGTCATG

114 CATGACCACTAGGAGCATCTTTGGCGAGAAGACTCTGTATTCGGGGACCAGTTGCTGCTAGGGGAATAAATCTTTGGGCACCTAGTGGTCATG

110 CATGACCACTAGGAGCATCTTTGGCGAGATCGGGAGAATCGGTGACATTGGTGTCTCCTAGGGGAATAAATCTTTGGGCACCTAGTGGTCATG

107 CATGACCACTAGGAGCATCTTTGGCGAACGGCAGGTGTTGCGGCGGTCTGTGAATCCCTAGGGGAATAAATCTTTGGGCACCTAGTGGTCATG

106 CATGACCACTAGGAGCATCTTTGGCGAAGTACAACGGCAGGCATTGTGTCTCGGTAGCTAGGGGAATAAATCTTTGGGCACCTAGTGGTCATG

105 CATGACCACTAGGAGCATCTTTGGCGAGAAGACTCAGGATTCGGGGACCAGTTGCTGCTAGGGGAATAAATCTTTGGGCACCTAGTGGTCATG

104 CATGACCACTAGGAGCATCTTTGGCGAGATCGGGAGAATCGGTTGCATTGGTGTCTCCTAGGGGAATAAATCTTTGGGCACCTAGTGGTCATG

102 CATGACCACTAGGAGCATCTTTGGCGAGATCGGGTGAATCGGTGGCATTGGTGTCTCCTAGGGGAATAAATCTTTGGGCACCTAGTGGTCATG

101 CATGACCACTAGGAGCATCTTTGGCGAGAGCCGCCACGGGTTGGGCCGGTGCGACAGCTAGGGGAATAAATCTTTGGGCACCTAGTGGTCATG

100 CATGACCACTAGGAGCATCTTTGGCGATCAAGTGCGCTTGGTAATTCCTGATGCGATCTAGGGGAATAAATCTTTGGGCACCTAGTGGTCATG

100 CATGACCACTAGGAGCATCTTTGGCGAACGGCAGGTGTTGAGGTGGTCTGTGAATCCCTAGGGGAATAAATCTTTGGGCACCTAGTGGTCATG

97 CATGACCACTAGGAGCATCTTTGGCGAGAACGGGAGAATCGGTGGCATTGGTGTCTCCTAGGGGAATAAATCTTTGGGCACCTAGTGGTCATG

96 CATGACCACTAGGAGCATCTTTGGCGAAAGGCAGGTGTTGCGGTGGTCTGTGAATCCCTAGGGGAATAAATCTTTGGGCACCTAGTGGTCATG

95 CATGACCACTAGGAGCATCTTTGGCGAGATCGGGAGAATCGATGGCATTGGTGTCTCCTAGGGGAATAAATCTTTGGGCACCTAGTGGTCATG

95 CATGACCACTAGGAGCATCTTTGGCGAGAAGGCTCTGGATTCGGGGACCAGTTGCTGCTAGGGGAATAAATCTTTGGGCACCTAGTGGTCATG

93 CATGACCACTAGGAGCATCTTTGGCGAATGAGCCTGTAGGCCAGAGCAGACGTTTGGCTAGGGGAATAAATCTTTGGGCACCTAGTGGTCATG

93 CATGACCACTAGGAGCATCTTTGGCGACGAGGGGCACGGGTGGTCATTGCGAGAAGACTAGGGGAATAAATCTTTGGGCACCTAGTGGTCATG

92 CATGACCACTAGGAGCATCTTTGGCGAAAGTCGATAGAACAGGGTGGGGTGCTGTCCCTAGGGGAATAAATCTTTGGGCACCTAGTGGTCATG

92 CATGACCACTAGGAGCATCTTTGGCGATCAGGCCGCCAGGGCGTACGAAGCGTGGTTCTAGGGGAATAAATCTTTGGGCACCTAGTGGTCATG

92 CATGACCACTAGGAGCATCTTTGGCGAAAGTCGGCAGAACAGGGTGGGGTGCTGTCCCTAGGGGAATAAATCTTTGGGCACCTAGTGGTCATG

92 CATGACCACTAGGAGCATCTTTGGCGAGGGGGCGAGTGGGCGAGGTGTTATAGGGAGCTAGGGGAATAAATCTTTGGGCACCTAGTGGTCATG

91 CATGACCACTAGGAGCATCTTTGGCGAACGGCAGGTGTTGCGGTGGTCTGTGAATACCTAGGGGAATAAATCTTTGGGCACCTAGTGGTCATG

90 CATGACCACTAGGAGCATCTTTGGCGAGATCGGGAGAATTGGTGGCATTGGTGTCTCCTAGGGGAATAAATCTTTGGGCACCTAGTGGTCATG

90 CATGACCACTAGGAGCATCTTTGGCGACCCACGGTAAAGCAGTGTACGTGTGGTGGACTAGGGGAATAAATCTTTGGGCACCTAGTGGTCATG

89 CATGACCACTAGGAGCATCTTTGGCGAAGCGCAGGGTGTGAGAGGGGCGTGTCCATGCTAGGGGAATAAATCTTTGGGCACCTAGTGGTCATG

88 CATGACCACTAGGAGCATCTTTGGCGAAAGTCGGTAGAACAGGGTGGGGCGCTGTCCCTAGGGGAATAAATCTTTGGGCACCTAGTGGTCATG

85 CATGACCACTAGGAGCATCTTTGGCGAGGGAGGGCGCCGGCAGCGGTGTGAATGCGACTAGGGGAATAAATCTTTGGGCACCTAGTGGTCATG

84 CATGACCACTAGGAGCATCTTTGGCGACGTCTGTCCTTGGTAGTTACGGCCAATCTCCTAGGGGAATAAATCTTTGGGCACCTAGTGGTCATG

83 CATGACCACTAGGAGCATCTTTGGCGAACGTGCAAGAAATGCCGCCTGGAGCGTAGGCTAGGGGAATAAATCTTTGGGCACCTAGTGGTCATG

82 CATGACCACTAGGAGCATCTTTGGCGAAGTATCGACTAGATTCGAGCAGACGTTTGGCTAGGGGAATAAATCTTTGGGCACCTAGTGGTCATG

81 CATGACCACTAGGAGCATCTTTGGCGAGATCGGGAGAATCGGTGGCAATGGTGTCTCCTAGGGGAATAAATCTTTGGGCACCTAGTGGTCATG

80 CATGACCACTAGGAGCATCTTTGGCGACTCGTGGTCCTTACCCCCGTAGAGGGTTGTCTAGGGGAATAAATCTTTGGGCACCTAGTGGTCATG

79 CATGACCACTAGGAGCATCTTTGGCGAATGGCCGGCATGGCCTTCTAGTCCTCGGTACTAGGGGAATAAATCTTTGGGCACCTAGTGGTCATG

79 CATGACCACTAGGAGCATCTTTGGCGAGATCTTAACGAAGTGAATAGCGGCAGGGGCCTAGGGGAATAAATCTTTGGGCACCTAGTGGTCATG

79 CATGACCACTAGGAGCATCTTTGGCGAGAGGACTCTGGATTCGGGGACCAGTTGCTGCTAGGGGAATAAATCTTTGGGCACCTAGTGGTCATG

78 CATGACCACTAGGAGCATCTTTGGCGAAGACGACACAGTCGATCGGGTTAGAGGAAGCTAGGGGAATAAATCTTTGGGCACCTAGTGGTCATG

78 CATGACCACTAGGAGCATCTTTGGCGAACTGCAGGTGTTGCGGTGGTCTGTGAATCCCTAGGGGAATAAATCTTTGGGCACCTAGTGGTCATG

78 CATGACCACTAGGAGCATCTTTGGCGAATGGCCGGCGCGGCCTTCTAGTCCTCGGTACTAGGGGAATAAATCTTTGGGCACCTAGTGGTCATG

78 CATGACCACTAGGAGCATCTTTGGCGAGATCGGGAGAATCGGTGGCATTGGTGTTTCCTAGGGGAATAAATCTTTGGGCACCTAGTGGTCATG

77 CATGACCACTAGGAGCATCTTTGGCGAAAGTCGTTAGAACAGGGTGGGGTGCTGTCCCTAGGGGAATAAATCTTTGGGCACCTAGTGGTCATG

76 CATGACCACTAGGAGCATCTTTGGCGAACGGCAGGTGGTGCGGTGGTCTGTGAATCCCTAGGGGAATAAATCTTTGGGCACCTAGTGGTCATG

75 CATGACCACTAGGAGCATCTTTGGCGACGGTGTGGGGCCATCTGTGCGGCGGATCCCCTAGGGGAATAAATCTTTGGGCACCTAGTGGTCATG

75 CATGACCACTAGGAGCATCTTTGGCGAGAAGACTCTGGGTTCGGGGACCAGTTGCTGCTAGGGGAATAAATCTTTGGGCACCTAGTGGTCATG

74 CATGACCACTAGGAGCATCTTTGGCGAACGGCAGGTGTTGCGGTGGTATGTGAATCCCTAGGGGAATAAATCTTTGGGCACCTAGTGGTCATG

74 CATGACCACTAGGAGCATCTTTGGCGAAGTGCGGCGTGCCGAGTCGTCCTGCGACTCCTAGGGGAATAAATCTTTGGGCACCTAGTGGTCATG

73 CATGACCACTAGGAGCATCTTTGGCGAGATCGGGAAAATCGGTGGCATTGGTGTCTCCTAGGGGAATAAATCTTTGGGCACCTAGTGGTCATG

72 CATGACCACTAGGAGCATCTTTGGCGAACGGCAGGTGTTGCTGTGGTCTGTGAATCCCTAGGGGAATAAATCTTTGGGCACCTAGTGGTCATG

72 CATGACCACTAGGAGCATCTTTGGCGAGGTGTTTCAGTTCGACATAAGGGGCGAGCACTAGGGGAATAAATCTTTGGGCACCTAGTGGTCATG

70 CATGACCACTAGGAGCATCTTTGGCGAACGGCAGGTGTTGCGTTGGTCTGTGAATCCCTAGGGGAATAAATCTTTGGGCACCTAGTGGTCATG

69 CATGACCACTAGGAGCATCTTTGGCGAACGGCAGGTGTTGCGGTGGTCTGTGAATCTCTAGGGGAATAAATCTTTGGGCACCTAGTGGTCATG

68 CATGACCACTAGGAGCATCTTTGGCGAGAGCCCGGTGTGGGTGCGCAAGCTATATGCCTAGGGGAATAAATCTTTGGGCACCTAGTGGTCATG

68 CATGACCACTAGGAGCATCTTTGGCGAAGTGATCGGAGATTACAAGCAGACGTTGGGCTAGGGGAATAAATCTTTGGGCACCTAGTGGTCATG

68 CATGACCACTAGGAGCATCTTTGGCGAGGGGTCGGTTGGAAGGACGTATTCCTGAGGCTAGGGGAATAAATCTTTGGGCACCTAGTGGTCATG

67 CATGACCACTAGGAGCATCTTTGGCGAACGGCAGGTGTTTCGGTGGTCTGTGAATCCCTAGGGGAATAAATCTTTGGGCACCTAGTGGTCATG

67 CATGACCACTAGGAGCATCTTTGGCGACGGAGTGGGGCTCATTGTGCGGCGGTAATCCTAGGGGAATAAATCTTTGGGCACCTAGTGGTCATG

66 CATGACCACTAGGAGCATCTTTGGCGACGGCGTGGGGCATCTCTCGCGGCGGTTATTCTAGGGGAATAAATCTTTGGGCACCTAGTGGTCATG

66 CATGACCACTAGGAGCATCTTTGGCGAGATCGGGAGAATCGGTGGCCTTGGTGTCTCCTAGGGGAATAAATCTTTGGGCACCTAGTGGTCATG

66 CATGACCACTAGGAGCATCTTTGGCGAACGGCAGGTGTTGCGGTGGTCAGTGAATCCCTAGGGGAATAAATCTTTGGGCACCTAGTGGTCATG

66 CATGACCACTAGGAGCATCTTTGGCGACATGGGCGACCTAAGAGAGGAGAAGTCGAACTAGGGGAATAAATCTTTGGGCACCTAGTGGTCATG

66 CATGACCACTAGGAGCATCTTTGGCGAACTCATTGGAGTGGGTGTAACGAATCCGTACTAGGGGAATAAATCTTTGGGCACCTAGTGGTCATG

66 CATGACCACTAGGAGCATCTTTGGCGAGGGGATCTGTCGGGCCGGAGTGCTCCAACGCTAGGGGAATAAATCTTTGGGCACCTAGTGGTCATG

65 CATGACCACTAGGAGCATCTTTGGCGAAACGGATCGGGAGGCGTGGCGGATTGTGTGCTAGGGGAATAAATCTTTGGGCACCTAGTGGTCATG

65 CATGACCACTAGGAGCATCTTTGGCGAACGGCAGGTGTTGCAGTGGTCTGTGAATCCCTAGGGGAATAAATCTTTGGGCACCTAGTGGTCATG

65 CATGACCACTAGGAGCATCTTTGGCGAGAAGACTCTGGATTCGGGGACCAGTTGCTACTAGGGGAATAAATCTTTGGGCACCTAGTGGTCATG

64 CATGACCACTAGGAGCATCTTTGGCGATTTGGGAGTCGCATTATGCAGGCAGTGAGACTAGGGGAATAAATCTTTGGGCACCTAGTGGTCATG

63 CATGACCACTAGGAGCATCTTTGGCGAACGTCGGTAGAACAGGGTGGGGTGCTGTCCCTAGGGGAATAAATCTTTGGGCACCTAGTGGTCATG

63 CATGACCACTAGGAGCATCTTTGGCGAGAAGACTCTGGATTCGGGGACCAGTTGCCGCTAGGGGAATAAATCTTTGGGCACCTAGTGGTCATG

63 CATGACCACTAGGAGCATCTTTGGCGAACGGCAGGTGTTGCGGAGGTCTGTGAATCCCTAGGGGAATAAATCTTTGGGCACCTAGTGGTCATG

63 CATGACCACTAGGAGCATCTTTGGCGACCGGCAGGTGTTGCGGTGGTCTGTGAATCCCTAGGGGAATAAATCTTTGGGCACCTAGTGGTCATG

63 CATGACCACTAGGAGCATCTTTGGCGAAGATCGCTCGTCGACTCGGGTCCGAGGAAGCTAGGGGAATAAATCTTTGGGCACCTAGTGGTCATG

62 CATGACCACTAGGAGCATCTTTGGCGAACGGCAGGTGTTGCGGTGGTCTGAGAATCCCTAGGGGAATAAATCTTTGGGCACCTAGTGGTCATG

62 CATGACCACTAGGAGCATCTTTGGCGAACGGCAGGTGTTGCGGTGTTCTGTGAATCCCTAGGGGAATAAATCTTTGGGCACCTAGTGGTCATG

62 CATGACCACTAGGAGCATCTTTGGCGAGATCCGGAGAATCGGTGGCATTGGTGTCTCCTAGGGGAATAAATCTTTGGGCACCTAGTGGTCATG

61 CATGACCACTAGGAGCATCTTTGGCGAACGGCATGTGTTGCGGTGGTCTGTGAATCCCTAGGGGAATAAATCTTTGGGCACCTAGTGGTCATG

61 CATGACCACTAGGAGCATCTTTGGCGAGAATACTCTGGATTCGGGGACCAGTTGCTGCTAGGGGAATAAATCTTTGGGCACCTAGTGGTCATG

61 CATGACCACTAGGAGCATCTTTGGCGAGGTACGAAGCCGAGGCTGTGGCTGAGTTTACTAGGGGAATAAATCTTTGGGCACCTAGTGGTCATG

61 CATGACCACTAGGAGCATCTTTGGCGAACGGCAGGTGTTGCGGTGGTCTGTGCATCCCTAGGGGAATAAATCTTTGGGCACCTAGTGGTCATG

59 CATGACCACTAGGAGCATCTTTGGCGAACGGCAGGTGTTGCGGTGGTCTCTGAATCCCTAGGGGAATAAATCTTTGGGCACCTAGTGGTCATG

59 CATGACCACTAGGAGCATCTTTGGCGATGGGGCCGTGCTATTTGCACACTCCGCGGGCTAGGGGAATAAATCTTTGGGCACCTAGTGGTCATG

59 CATGACCACTAGGAGCATCTTTGGCGACAATGGGGCTGGTAGGAAAGTACTTATAGCCTAGGGGAATAAATCTTTGGGCACCTAGTGGTCATG

58 CATGACCACTAGGAGCATCTTTGGCGACGGCGTGGGGGAGAAATCCGGCGGTGACTGCTAGGGGAATAAATCTTTGGGCACCTAGTGGTCATG

58 CATGACCACTAGGAGCATCTTTGGCGAGATCGGGCGAATCGGTGGCATTGGTGTCTCCTAGGGGAATAAATCTTTGGGCACCTAGTGGTCATG

58 CATGACCACTAGGAGCATCTTTGGCGAACGGCAGGTGTTGCGGTGGTCTGTAAATCCCTAGGGGAATAAATCTTTGGGCACCTAGTGGTCATG

57 CATGACCACTAGGAGCATCTTTGGCGAGAAGACTCTGGATTCGGGGGCCAGTTGCTGCTAGGGGAATAAATCTTTGGGCACCTAGTGGTCATG

57 CATGACCACTAGGAGCATCTTTGGCGAACAGCAGGTGTTGCGGTGGTCTGTGAATCCCTAGGGGAATAAATCTTTGGGCACCTAGTGGTCATG

57 CATGACCACTAGGAGCATCTTTGGCGACCAGATATGGTAGGTAATGTGCTGAGGAGGCTAGGGGAATAAATCTTTGGGCACCTAGTGGTCATG

57 CATGACCACTAGGAGCATCTTTGGCGAGATCGGGAGTATCGGTGGCATTGGTGTCTCCTAGGGGAATAAATCTTTGGGCACCTAGTGGTCATG

57 CATGACCACTAGGAGCATCTTTGGCGAACGTCAGGTGTTGCGGTGGTCTGTGAATCCCTAGGGGAATAAATCTTTGGGCACCTAGTGGTCATG

57 CATGACCACTAGGAGCATCTTTGGCGAACGGCAGGTGTTGCGGGGGTCTGTGAATCCCTAGGGGAATAAATCTTTGGGCACCTAGTGGTCATG

56 CATGACCACTAGGAGCATCTTTGGCGAGGGGGGGCGCCGGCAGCGGTGTGAATGCGCCTAGGGGAATAAATCTTTGGGCACCTAGTGGTCATG

56 CATGACCACTAGGAGCATCTTTGGCGAACGGCAAGTGTTGCGGTGGTCTGTGAATCCCTAGGGGAATAAATCTTTGGGCACCTAGTGGTCATG

55 CATGACCACTAGGAGCATCTTTGGCGAATGGCTGGCACGGCCTTCTAGTCCTCGGTACTAGGGGAATAAATCTTTGGGCACCTAGTGGTCATG

55 CATGACCACTAGGAGCATCTTTGGCGATAGCGGCGTAGGCTGCCTTGGGTGGTGGCCCTAGGGGAATAAATCTTTGGGCACCTAGTGGTCATG

55 CATGACCACTAGGAGCATCTTTGGCGAAAGTGGGCGATCTGGGAGAGGTCAATCATGCTAGGGGAATAAATCTTTGGGCACCTAGTGGTCATG

55 CATGACCACTAGGAGCATCTTTGGCGAACGACAGGTGTTGCGGTGGTCTGTGAATCCCTAGGGGAATAAATCTTTGGGCACCTAGTGGTCATG

54 CATGACCACTAGGAGCATCTTTGGCGACATTGACAGCTAGGGTGAGGTTGTGTGCGACTAGGGGAATAAATCTTTGGGCACCTAGTGGTCATG

54 CATGACCACTAGGAGCATCTTTGGCGAGAAGACTATGGATTCGGGGACCAGTTGCTGCTAGGGGAATAAATCTTTGGGCACCTAGTGGTCATG

53 CATGACCACTAGGAGCATCTTTGGCGACAGTCGGTAGAACAGGGTGGGGTGCTGTCCCTAGGGGAATAAATCTTTGGGCACCTAGTGGTCATG

53 CATGACCACTAGGAGCATCTTTGGCGAACGGCAGGTGTTACGGTGGTCTGTGAATCCCTAGGGGAATAAATCTTTGGGCACCTAGTGGTCATG

53 CATGACCACTAGGAGCATCTTTGGCGAACGGCAGTTGTTGCGGTGGTCTGTGAATCCCTAGGGGAATAAATCTTTGGGCACCTAGTGGTCATG

53 CATGACCACTAGGAGCATCTTTGGCGAACGGCAGGTGTTGCGGTGGTCGGTGAATCCCTAGGGGAATAAATCTTTGGGCACCTAGTGGTCATG

52 CATGACCACTAGGAGCATCTTTGGCGACGGGGCCGGACGGGACGGACACATCGCGGCCTAGGGGAATAAATCTTTGGGCACCTAGTGGTCATG

52 CATGACCACTAGGAGCATCTTTGGCGAGAGCGGGAGAATCGGTGGCATTGGTGTCTCCTAGGGGAATAAATCTTTGGGCACCTAGTGGTCATG

52 CATGACCACTAGGAGCATCTTTGGCGAACGGCAGGTGTTGCGGTAGTCTGTGAATCCCTAGGGGAATAAATCTTTGGGCACCTAGTGGTCATG

51 CATGACCACTAGGAGCATCTTTGGCGAACGGCAGGTGTTGCGGTGGTCTGTGAATTCCTAGGGGAATAAATCTTTGGGCACCTAGTGGTCATG

51 CATGACCACTAGGAGCATCTTTGGCGACTTTGGGAGCAAGTGCGTCGGAGTGGTCGCCTAGGGGAATAAATCTTTGGGCACCTAGTGGTCATG

51 CATGACCACTAGGAGCATCTTTGGCGACGGGCGGGCGCCCTAGGGTGTGAGTGTTGCCTAGGGGAATAAATCTTTGGGCACCTAGTGGTCATG

51 CATGACCACTAGGAGCATCTTTGGCGAACGGCAGGTGTTGCGGTGATCTGTGAATCCCTAGGGGAATAAATCTTTGGGCACCTAGTGGTCATG

51 CATGACCACTAGGAGCATCTTTGGCGAACGGCAGGTGTTGCGGTGGACTGTGAATCCCTAGGGGAATAAATCTTTGGGCACCTAGTGGTCATG

51 CATGACCACTAGGAGCATCTTTGGCGACAGCGGCGTAGGCCGCCTTGGGTGGTGGCCCTAGGGGAATAAATCTTTGGGCACCTAGTGGTCATG

51 CATGACCACTAGGAGCATCTTTGGCGATGGGGCCGTGCTATCTGCACACTTCGCGGGCTAGGGGAATAAATCTTTGGGCACCTAGTGGTCATG

51 CATGACCACTAGGAGCATCTTTGGCGAGGGTGGGCGCCGGCAGCGGTGTGAATGCGCCTAGGGGAATAAATCTTTGGGCACCTAGTGGTCATG

50 CATGACCACTAGGAGCATCTTTGGCGAGCCACGGGCGTAGACCGCTGTTCTGGGTAACTAGGGGAATAAATCTTTGGGCACCTAGTGGTCATG

49 CATGACCACTAGGAGCATCTTTGGCGATATCGGCGTTAGCTAACATCGTAGGATGTTCTAGGGGAATAAATCTTTGGGCACCTAGTGGTCATG

49 CATGACCACTAGGAGCATCTTTGGCGAGATCGGGAGAATCGGTGGCATGGGTGTCTCCTAGGGGAATAAATCTTTGGGCACCTAGTGGTCATG

49 CATGACCACTAGGAGCATCTTTGGCGAGGACCGGTAAACTAGCATTAGCGTTGCGCTCTAGGGGAATAAATCTTTGGGCACCTAGTGGTCATG

49 CATGACCACTAGGAGCATCTTTGGCGAACGGCAGGTGATGCGGTGGTCTGTGAATCCCTAGGGGAATAAATCTTTGGGCACCTAGTGGTCATG

49 CATGACCACTAGGAGCATCTTTGGCGAGAAGACTCTGGACTCGGGGACCAGTTGCTGCTAGGGGAATAAATCTTTGGGCACCTAGTGGTCATG

48 CATGACCACTAGGAGCATCTTTGGCGAACGGCAGGTGTTGTGGTGGTCTGTGAATCCCTAGGGGAATAAATCTTTGGGCACCTAGTGGTCATG

48 CATGACCACTAGGAGCATCTTTGGCGACTGTGGTCTCGTCCTAATTGTGCGGGCGTCCTAGGGGAATAAATCTTTGGGCACCTAGTGGTCATG

47 CATGACCACTAGGAGCATCTTTGGCGAAAGTCGGTAGAACAGGGTGGGGTACTGTCCCTAGGGGAATAAATCTTTGGGCACCTAGTGGTCATG

47 CATGACCACTAGGAGCATCTTTGGCGACTTTGCCGGGCAGACCGCGCCGTTAGTTGTCTAGGGGAATAAATCTTTGGGCACCTAGTGGTCATG

46 CATGACCACTAGGAGCATCTTTGGCGAATGGCCGGCACGGCTTTCTAGTCCTCGGTACTAGGGGAATAAATCTTTGGGCACCTAGTGGTCATG

46 CATGACCACTAGGAGCATCTTTGGCGAAGGGGGTCTCGGGGTGATAACGATGTAGCCCTAGGGGAATAAATCTTTGGGCACCTAGTGGTCATG

46 CATGACCACTAGGAGCATCTTTGGCGAACGGCAGGTGTAGCGGTGGTCTGTGAATCCCTAGGGGAATAAATCTTTGGGCACCTAGTGGTCATG

46 CATGACCACTAGGAGCATCTTTGGCGAACGGCAGGTGTTGCGGTGGTCTGTTAATCCCTAGGGGAATAAATCTTTGGGCACCTAGTGGTCATG

45 CATGACCACTAGGAGCATCTTTGGCGAATGGCCGGCACGGTCTTCTAGTCCTCGGTACTAGGGGAATAAATCTTTGGGCACCTAGTGGTCATG

45 CATGACCACTAGGAGCATCTTTGGCGAGAAGACTCTGGATTCGGGGACCAGTTGATGCTAGGGGAATAAATCTTTGGGCACCTAGTGGTCATG

45 CATGACCACTAGGAGCATCTTTGGCGAGCCTCGCTTGGGAGGTTGCTTCACCAGTTCCTAGGGGAATAAATCTTTGGGCACCTAGTGGTCATG

45 CATGACCACTAGGAGCATCTTTGGCGACTAGGCGTCAGCACCGGGTGGGCTCGGTCCCTAGGGGAATAAATCTTTGGGCACCTAGTGGTCATG

44 CATGACCACTAGGAGCATCTTTGGCGAACGGCAGGTGTTGCGGTGGGCTGTGAATCCCTAGGGGAATAAATCTTTGGGCACCTAGTGGTCATG

44 CATGACCACTAGGAGCATCTTTGGCGAACGGCAGATGTTGCGGTGGTCTGTGAATCCCTAGGGGAATAAATCTTTGGGCACCTAGTGGTCATG

44 CATGACCACTAGGAGCATCTTTGGCGAGGGTAGGCGTCAGGATGATGTGTATTTGCTCTAGGGGAATAAATCTTTGGGCACCTAGTGGTCATG

44 CATGACCACTAGGAGCATCTTTGGCGAGAAGACTCTGAATTCGGGGACCAGTTGCTGCTAGGGGAATAAATCTTTGGGCACCTAGTGGTCATG

43 CATGACCACTAGGAGCATCTTTGGCGAGAAGACTCTGGATTCGGGGACCAGCTGCTGCTAGGGGAATAAATCTTTGGGCACCTAGTGGTCATG

43 CATGACCACTAGGAGCATCTTTGGCGAACGGCAGGAGTTGCGGTGGTCTGTGAATCCCTAGGGGAATAAATCTTTGGGCACCTAGTGGTCATG

43 CATGACCACTAGGAGCATCTTTGGCGAACAGGCCTTGGCTGCGGGTGGGTGCGCACCCTAGGGGAATAAATCTTTGGGCACCTAGTGGTCATG

43 CATGACCACTAGGAGCATCTTTGGCGAGAAGACACTGGATTCGGGGACCAGTTGCTGCTAGGGGAATAAATCTTTGGGCACCTAGTGGTCATG

43 CATGACCACTAGGAGCATCTTTGGCGAGGCGATATGAAGCGACGAGCAGACGTTTGGCTAGGGGAATAAATCTTTGGGCACCTAGTGGTCATG

42 CATGACCACTAGGAGCATCTTTGGCGACGGGGTGGGGCCTAAGTGCGGCGGCTGATTCTAGGGGAATAAATCTTTGGGCACCTAGTGGTCATG

42 CATGACCACTAGGAGCATCTTTGGCGACGTCGTAAAGTGCGTAGAGTGGTAAGTCGACTAGGGGAATAAATCTTTGGGCACCTAGTGGTCATG

42 CATGACCACTAGGAGCATCTTTGGCGACGGGCGGGCGCCCAAGGGTGTGAGTGTTGCCTAGGGGAATAAATCTTTGGGCACCTAGTGGTCATG

41 CATGACCACTAGGAGCATCTTTGGCGAACGGCAGGTGTTGCGGTTGTCTGTGAATCCCTAGGGGAATAAATCTTTGGGCACCTAGTGGTCATG

41 CATGACCACTAGGAGCATCTTTGGCGATCTGTGCTGAAAAGCCCCGTGGAGGGTTGTCTAGGGGAATAAATCTTTGGGCACCTAGTGGTCATG

41 CATGACCACTAGGAGCATCTTTGGCGAGATCGGGAGAATCGGTGGCATTGGGGTCTCCTAGGGGAATAAATCTTTGGGCACCTAGTGGTCATG

40 CATGACCACTAGGAGCATCTTTGGCGAGAGAATCGGTAGGCAGAGTGCTCCAGCCAGCTAGGGGAATAAATCTTTGGGCACCTAGTGGTCATG

40 CATGACCACTAGGAGCATCTTTGGCGACGGTGTGGGGAAGTTATATCGGCGGATGCACTAGGGGAATAAATCTTTGGGCACCTAGTGGTCATG

40 CATGACCACTAGGAGCATCTTTGGCGACCCTGGCGCGGGGATCCTGGTTGCGGCGACCTAGGGGAATAAATCTTTGGGCACCTAGTGGTCATG

40 CATGACCACTAGGAGCATCTTTGGCGAGATCGGGAGAATCGGTGGCATTGGTGTCTGCTAGGGGAATAAATCTTTGGGCACCTAGTGGTCATG

40 CATGACCACTAGGAGCATCTTTGGCGAGCACCCGACGAGGGGGGAGCAGACGTTTGGCTAGGGGAATAAATCTTTGGGCACCTAGTGGTCATG

39 CATGACCACTAGGAGCATCTTTGGCGAATGGCCAGCACGGCCTTCTAGTCCTCGGTACTAGGGGAATAAATCTTTGGGCACCTAGTGGTCATG

39 CATGACCACTAGGAGCATCTTTGGCGATGGGTGCTGACGGCCGCTGCTGCGGCTACACTAGGGGAATAAATCTTTGGGCACCTAGTGGTCATG

39 CATGACCACTAGGAGCATCTTTGGCGATTTGGGAGTCGCAGTATGCAGGCAGTGAGGCTAGGGGAATAAATCTTTGGGCACCTAGTGGTCATG

39 CATGACCACTAGGAGCATCTTTGGCGATGGGGAGCGAGAGGACTGAGTAGGAATAAGCTAGGGGAATAAATCTTTGGGCACCTAGTGGTCATG

39 CATGACCACTAGGAGCATCTTTGGCGACGGGATCGTGTCGGCAGATAGCGTACTTGGCTAGGGGAATAAATCTTTGGGCACCTAGTGGTCATG

39 CATGACCACTAGGAGCATCTTTGGCGACTACGGGCTAGAGTTACTCGTGTTAATAAGCTAGGGGAATAAATCTTTGGGCACCTAGTGGTCATG

39 CATGACCACTAGGAGCATCTTTGGCGAATGGCCGACACGGCCTTCTAGTCCTCGGTACTAGGGGAATAAATCTTTGGGCACCTAGTGGTCATG

39 CATGACCACTAGGAGCATCTTTGGCGAGATCGGGAGAATCGCTGGCATTGGTGTCTCCTAGGGGAATAAATCTTTGGGCACCTAGTGGTCATG

39 CATGACCACTAGGAGCATCTTTGGCGACTCGACCGGGGCCCTAGGGCTGCGGCCCACCTAGGGGAATAAATCTTTGGGCACCTAGTGGTCATG

39 CATGACCACTAGGAGCATCTTTGGCGAGATCGGGAGAATCGGTGCCATTGGTGTCTCCTAGGGGAATAAATCTTTGGGCACCTAGTGGTCATG

39 CATGACCACTAGGAGCATCTTTGGCGAGAAGACTCTGGATCCGGGGACCAGTTGCTGCTAGGGGAATAAATCTTTGGGCACCTAGTGGTCATG

39 CATGACCACTAGGAGCATCTTTGGCGAGAAGACTCTGGATTCGGGGACCGGTTGCTGCTAGGGGAATAAATCTTTGGGCACCTAGTGGTCATG

39 CATGACCACTAGGAGCATCTTTGGCGAGATGGTGTACACCGTCGAGCAGACGTTGGGCTAGGGGAATAAATCTTTGGGCACCTAGTGGTCATG

39 CATGACCACTAGGAGCATCTTTGGCGATCCTCGTTAGATGGTGAAGCAGACGTTTGGCTAGGGGAATAAATCTTTGGGCACCTAGTGGTCATG

39 CATGACCACTAGGAGCATCTTTGGCGAGGAGCCCTGTTGCGAGCAACCCGAGGCGAGCTAGGGGAATAAATCTTTGGGCACCTAGTGGTCATG

38 CATGACCACTAGGAGCATCTTTGGCGAATGATGACTAGGTCAATCGGGCTAGAGGAACTAGGGGAATAAATCTTTGGGCACCTAGTGGTCATG

38 CATGACCACTAGGAGCATCTTTGGCGAGGGGCCTAAGTGCGTATCTGTGGTCGAGCTCTAGGGGAATAAATCTTTGGGCACCTAGTGGTCATG

37 CATGACCACTAGGAGCATCTTTGGCGAACGGCTGGTGTTGCGGTGGTCTGTGAATCCCTAGGGGAATAAATCTTTGGGCACCTAGTGGTCATG

37 CATGACCACTAGGAGCATCTTTGGCGACGGTGTGGGGAAGTTATATCGGCGGATGCTCTAGGGGAATAAATCTTTGGGCACCTAGTGGTCATG

37 CATGACCACTAGGAGCATCTTTGGCGAAGCGCAGGGTGTGAGAGGGGCGGGTCCATGCTAGGGGAATAAATCTTTGGGCACCTAGTGGTCATG

36 CATGACCACTAGGAGCATCTTTGGCGAACGGCAGGTTTTGCGGTGGTCTGTGAATCCCTAGGGGAATAAATCTTTGGGCACCTAGTGGTCATG

36 CATGACCACTAGGAGCATCTTTGGCGACCTCGGCTGGTAGGTAATGTATTGGGCGATCTAGGGGAATAAATCTTTGGGCACCTAGTGGTCATG

36 CATGACCACTAGGAGCATCTTTGGCGACGGCGTGGGGCCTCTAGCGCGGCGGGGATCCTAGGGGAATAAATCTTTGGGCACCTAGTGGTCATG

35 CATGACCACTAGGAGCATCTTTGGCGAACGGCAGGTATTGCGGTGGTCTGTGAATCCCTAGGGGAATAAATCTTTGGGCACCTAGTGGTCATG

35 CATGACCACTAGGAGCATCTTTGGCGAGTGTGGGCTGGCAGTGCGTACTTGATCGCTCTAGGGGAATAAATCTTTGGGCACCTAGTGGTCATG

35 CATGACCACTAGGAGCATCTTTGGCGAATGGCCGGCACGGCCTTCTAGTCTTCGGTACTAGGGGAATAAATCTTTGGGCACCTAGTGGTCATG

35 CATGACCACTAGGAGCATCTTTGGCGACGGCGTGGGGCTATAGATGCGGCGGCACTCCTAGGGGAATAAATCTTTGGGCACCTAGTGGTCATG

35 CATGACCACTAGGAGCATCTTTGGCGAAACGACCTTGTCGTTGGGGACCAGTTGCTGCTAGGGGAATAAATCTTTGGGCACCTAGTGGTCATG

35 CATGACCACTAGGAGCATCTTTGGCGACGTTGGGAGCGAAGTCCCAGCGAAGTGGACCTAGGGGAATAAATCTTTGGGCACCTAGTGGTCATG

35 CATGACCACTAGGAGCATCTTTGGCGAGCTCCGAGCAAGCGAACCGCGAAAAGTCGGCTAGGGGAATAAATCTTTGGGCACCTAGTGGTCATG

34 CATGACCACTAGGAGCATCTTTGGCGACTAAGTGGTCAACGCGTTAAATGGGGTAGACTAGGGGAATAAATCTTTGGGCACCTAGTGGTCATG

34 CATGACCACTAGGAGCATCTTTGGCGAATGGTCGGCACGGCCTTCTAGTCCTCGGTACTAGGGGAATAAATCTTTGGGCACCTAGTGGTCATG

34 CATGACCACTAGGAGCATCTTTGGCGAGGAGACTCTGGATTCGGGGACCAGTTGCTGCTAGGGGAATAAATCTTTGGGCACCTAGTGGTCATG

34 CATGACCACTAGGAGCATCTTTGGCGAAGGGGGTCTCGGGGTGATAACGATGTAGCTCTAGGGGAATAAATCTTTGGGCACCTAGTGGTCATG

34 CATGACCACTAGGAGCATCTTTGGCGATACGTCGTGTGGTGGTTCAAGGTGAGCTAGCTAGGGGAATAAATCTTTGGGCACCTAGTGGTCATG

33 CATGACCACTAGGAGCATCTTTGGCGAGTTCGGGAGAATCGGTGGCATTGGTGTCTCCTAGGGGAATAAATCTTTGGGCACCTAGTGGTCATG

33 CATGACCACTAGGAGCATCTTTGGCGAACGGCAGGTGTTGCGATGGTCTGTGAATCCCTAGGGGAATAAATCTTTGGGCACCTAGTGGTCATG

33 CATGACCACTAGGAGCATCTTTGGCGAGAAGACTCTGGATTCTGGGACCAGTTGCTGCTAGGGGAATAAATCTTTGGGCACCTAGTGGTCATG

33 CATGACCACTAGGAGCATCTTTGGCGAAAGTCGGTAGAACAGGGTGGGGTGCTGCCCCTAGGGGAATAAATCTTTGGGCACCTAGTGGTCATG

33 CATGACCACTAGGAGCATCTTTGGCGACGGCGTGGGGCTATTGATGCGGCGGTACTCCTAGGGGAATAAATCTTTGGGCACCTAGTGGTCATG

33 CATGACCACTAGGAGCATCTTTGGCGAAAGTCGGTAGAACAGGGTGGGGTGCTGTCACTAGGGGAATAAATCTTTGGGCACCTAGTGGTCATG

33 CATGACCACTAGGAGCATCTTTGGCGACACAGTCGGCTGGTAGAATGTATTTGTGAGCTAGGGGAATAAATCTTTGGGCACCTAGTGGTCATG

32 CATGACCACTAGGAGCATCTTTGGCGAAGCGTGTTGCAAGGTCCGCGGGTGTGGACCCTAGGGGAATAAATCTTTGGGCACCTAGTGGTCATG

32 CATGACCACTAGGAGCATCTTTGGCGACTTGTCGAGCCGTTCGGCGTTGAGGAAGTGCTAGGGGAATAAATCTTTGGGCACCTAGTGGTCATG

32 CATGACCACTAGGAGCATCTTTGGCGAGAAGACTCTGGATTCGGGGACCAGTCGCTGCTAGGGGAATAAATCTTTGGGCACCTAGTGGTCATG

32 CATGACCACTAGGAGCATCTTTGGCGACTAGGCGTCAGCACCGGGTGGGTTCGGTCCCTAGGGGAATAAATCTTTGGGCACCTAGTGGTCATG

32 CATGACCACTAGGAGCATCTTTGGCGAGAAGACTCTGGATTCGGGGACCAGTTTCTGCTAGGGGAATAAATCTTTGGGCACCTAGTGGTCATG

32 CATGACCACTAGGAGCATCTTTGGCGAGAAGATTCTGGATTCGGGGACCAGTTGCTGCTAGGGGAATAAATCTTTGGGCACCTAGTGGTCATG

31 CATGACCACTAGGAGCATCTTTGGCGAATGGCCGGCACGGCCTTCTAGTCCTCGGCACTAGGGGAATAAATCTTTGGGCACCTAGTGGTCATG

31 CATGACCACTAGGAGCATCTTTGGCGAGATCGGGAGAATCGGTGGCATTGGTGGCTCCTAGGGGAATAAATCTTTGGGCACCTAGTGGTCATG

31 CATGACCACTAGGAGCATCTTTGGCGAGAAGACTCTGGATTCGGGGACCAGATGCTGCTAGGGGAATAAATCTTTGGGCACCTAGTGGTCATG

31 CATGACCACTAGGAGCATCTTTGGCGAGATCGGGAGAATCGGTGGGATTGGTGTCTCCTAGGGGAATAAATCTTTGGGCACCTAGTGGTCATG

31 CATGACCACTAGGAGCATCTTTGGCGAGATCGGGAGAATCGGTGGCATTGGTGTCGCCTAGGGGAATAAATCTTTGGGCACCTAGTGGTCATG

30 CATGACCACTAGGAGCATCTTTGGCGAGAAGACTCTAGATTCGGGGACCAGTTGCTGCTAGGGGAATAAATCTTTGGGCACCTAGTGGTCATG

30 CATGACCACTAGGAGCATCTTTGGCGATCAGGCCGTGGCTGCGGGTGGGTGCGCACCCTAGGGGAATAAATCTTTGGGCACCTAGTGGTCATG

30 CATGACCACTAGGAGCATCTTTGGCGAGTGTCCGAGTGGCGAGGGTGTGCCTCGAGACTAGGGGAATAAATCTTTGGGCACCTAGTGGTCATG

29 CATGACCACTAGGAGCATCTTTGGCGAATGCGAGGTTGGTTACGATGTGGCTGAGGCCTAGGGGAATAAATCTTTGGGCACCTAGTGGTCATG

29 CATGACCACTAGGAGCATCTTTGGCGAGATCGGGAGAATCGGGGGCATTGGTGTCTCCTAGGGGAATAAATCTTTGGGCACCTAGTGGTCATG

29 CATGACCACTAGGAGCATCTTTGGCGAGAAGACTCTGGATTCGGGGACCAGTAGCTGCTAGGGGAATAAATCTTTGGGCACCTAGTGGTCATG

29 CATGACCACTAGGAGCATCTTTGGCGATGGGGCCGTGCTATCCGCACACTCCGCGGGCTAGGGGAATAAATCTTTGGGCACCTAGTGGTCATG

29 CATGACCACTAGGAGCATCTTTGGCGATTGGATACGGAAGGCAAGTGTGCCACTAGCCTAGGGGAATAAATCTTTGGGCACCTAGTGGTCATG

29 CATGACCACTAGGAGCATCTTTGGCGAGAAGACTCTGGATTCGGGTACCAGTTGCTGCTAGGGGAATAAATCTTTGGGCACCTAGTGGTCATG

28 CATGACCACTAGGAGCATCTTTGGCGACGGCGTGGGGCCATCTGTGCGGCGGATCCTCTAGGGGAATAAATCTTTGGGCACCTAGTGGTCATG

28 CATGACCACTAGGAGCATCTTTGGCGAAAGTCGGTAGAACAGGGTGGGGTGCCGTCCCTAGGGGAATAAATCTTTGGGCACCTAGTGGTCATG

28 CATGACCACTAGGAGCATCTTTGGCGACGCGGGTGCAACAAGCTAGCAGACGTTTGGCTAGGGGAATAAATCTTTGGGCACCTAGTGGTCATG

28 CATGACCACTAGGAGCATCTTTGGCGAATGGCGTGGCTCAGCCCACGTGAGGACTGGCTAGGGGAATAAATCTTTGGGCACCTAGTGGTCATG

28 CATGACCACTAGGAGCATCTTTGGCGAATGGCCGGCTCGGCCTTCTAGTCCTCGGTACTAGGGGAATAAATCTTTGGGCACCTAGTGGTCATG

27 CATGACCACTAGGAGCATCTTTGGCGACAATGTTAATGTGGTGTGATGCTTAGAATTCTAGGGGAATAAATCTTTGGGCACCTAGTGGTCATG

27 CATGACCACTAGGAGCATCTTTGGCGATTGAAGCCGGTGCGGTGCGGCGAGATGCGACTAGGGGAATAAATCTTTGGGCACCTAGTGGTCATG

27 CATGACCACTAGGAGCATCTTTGGCGAGAAGACTCTGGATTTGGGGACCAGTTGCTGCTAGGGGAATAAATCTTTGGGCACCTAGTGGTCATG

27 CATGACCACTAGGAGCATCTTTGGCGAGAAGACTCTGGATTAGGGGACCAGTTGCTGCTAGGGGAATAAATCTTTGGGCACCTAGTGGTCATG

27 CATGACCACTAGGAGCATCTTTGGCGATTTCGATGGCCACGACCTTGCGGCCGCAACCTAGGGGAATAAATCTTTGGGCACCTAGTGGTCATG

27 CATGACCACTAGGAGCATCTTTGGCGAGAAGACTCTGGATTCGGGGACAAGTTGCTGCTAGGGGAATAAATCTTTGGGCACCTAGTGGTCATG

27 CATGACCACTAGGAGCATCTTTGGCGATGTGATCGACGCGCTATAGGTTGCGGTAAGCTAGGGGAATAAATCTTTGGGCACCTAGTGGTCATG

26 CATGACCACTAGGAGCATCTTTGGCGAATGGCCGGCACGGCCTTCTAGTCCCCGGTACTAGGGGAATAAATCTTTGGGCACCTAGTGGTCATG

26 CATGACCACTAGGAGCATCTTTGGCGAAAGTCGGTAGAAAAGGGTGGGGTGCTGTCCCTAGGGGAATAAATCTTTGGGCACCTAGTGGTCATG

26 CATGACCACTAGGAGCATCTTTGGCGATGGGTGCTGACGACCGTCCACACGGTTACTCTAGGGGAATAAATCTTTGGGCACCTAGTGGTCATG

26 CATGACCACTAGGAGCATCTTTGGCGATTGGTAGTCGCGGATGCAGGCAGTGATGCTCTAGGGGAATAAATCTTTGGGCACCTAGTGGTCATG

26 CATGACCACTAGGAGCATCTTTGGCGACGGCTGGCTGGCAGTGCGTACTAGTAGTCACTAGGGGAATAAATCTTTGGGCACCTAGTGGTCATG

26 CATGACCACTAGGAGCATCTTTGGCGACGTGGGCTGGGAGGATGTGGTACTTCTCGACTAGGGGAATAAATCTTTGGGCACCTAGTGGTCATG

26 CATGACCACTAGGAGCATCTTTGGCGACGGTGTGGGGACCGTAGGTTCGGCGGGTACCTAGGGGAATAAATCTTTGGGCACCTAGTGGTCATG

26 CATGACCACTAGGAGCATCTTTGGCGAACGTGCATGATATGCCGCCTGGAGCGTAGGCTAGGGGAATAAATCTTTGGGCACCTAGTGGTCATG

26 CATGACCACTAGGAGCATCTTTGGCGAGAAAACTCTGGATTCGGGGACCAGTTGCTGCTAGGGGAATAAATCTTTGGGCACCTAGTGGTCATG

26 CATGACCACTAGGAGCATCTTTGGCGAGAACGCGAACGCGTAAAAGCAGACGTTTGGCTAGGGGAATAAATCTTTGGGCACCTAGTGGTCATG

25 CATGACCACTAGGAGCATCTTTGGCGAAAGTCGGTAGAGCAGGGTGGGGTGCTGTCCCTAGGGGAATAAATCTTTGGGCACCTAGTGGTCATG

25 CATGACCACTAGGAGCATCTTTGGCGAATGGCCGGCACGGCCTTCTATTCCTCGGTACTAGGGGAATAAATCTTTGGGCACCTAGTGGTCATG

25 CATGACCACTAGGAGCATCTTTGGCGACACCTTTAGAGCGGCGGCATTGTTGCGTGCCTAGGGGAATAAATCTTTGGGCACCTAGTGGTCATG

25 CATGACCACTAGGAGCATCTTTGGCGAGGGGGTCTGTCGGGCCGGAGTGCTCCAACGCTAGGGGAATAAATCTTTGGGCACCTAGTGGTCATG

25 CATGACCACTAGGAGCATCTTTGGCGAAAGTCGGTAGAACAGGGTGTGGTGCTGTCCCTAGGGGAATAAATCTTTGGGCACCTAGTGGTCATG

25 CATGACCACTAGGAGCATCTTTGGCGAGAAGAATCTGGATTCGGGGACCAGTTGCTGCTAGGGGAATAAATCTTTGGGCACCTAGTGGTCATG

25 CATGACCACTAGGAGCATCTTTGGCGATCGGCAGGTGTTGCGGTGGTCTGTGAATCCCTAGGGGAATAAATCTTTGGGCACCTAGTGGTCATG

25 CATGACCACTAGGAGCATCTTTGGCGAAAGTCGGTAGAACAGGGCGGGGTGCTGTCCCTAGGGGAATAAATCTTTGGGCACCTAGTGGTCATG

25 CATGACCACTAGGAGCATCTTTGGCGATTAGGCGTCAGCACCGGGTGGGCTCGGTCCCTAGGGGAATAAATCTTTGGGCACCTAGTGGTCATG

25 CATGACCACTAGGAGCATCTTTGGCGACCGTAAGACCTGAGCGGGGATGAGCCCACACTAGGGGAATAAATCTTTGGGCACCTAGTGGTCATG

24 CATGACCACTAGGAGCATCTTTGGCGAAAGTCAGTAGAACAGGGTGGGGTGCTGTCCCTAGGGGAATAAATCTTTGGGCACCTAGTGGTCATG

24 CATGACCACTAGGAGCATCTTTGGCGACGAGCTGGGTGCGCGTTAGATTGGGATAGCCTAGGGGAATAAATCTTTGGGCACCTAGTGGTCATG

24 CATGACCACTAGGAGCATCTTTGGCGACCCGGGACGCACTTGATGGACCGTGGTGGACTAGGGGAATAAATCTTTGGGCACCTAGTGGTCATG

24 CATGACCACTAGGAGCATCTTTGGCGAGAAGACTCTGGATTCGTGGACCAGTTGCTGCTAGGGGAATAAATCTTTGGGCACCTAGTGGTCATG

24 CATGACCACTAGGAGCATCTTTGGCGACTGAGGGACGGCACGTCATGTCGAAGTGGACTAGGGGAATAAATCTTTGGGCACCTAGTGGTCATG

24 CATGACCACTAGGAGCATCTTTGGCGACAGTGCGAGGTTGGTTCAGTGGTCTGCGATCTAGGGGAATAAATCTTTGGGCACCTAGTGGTCATG

24 CATGACCACTAGGAGCATCTTTGGCGAAGCGTGTTGCAAGGTCCGTGAGTGTGGACCCTAGGGGAATAAATCTTTGGGCACCTAGTGGTCATG

24 CATGACCACTAGGAGCATCTTTGGCGATCAGGCCGCCAGGGCGTACGAAGTGTGGTACTAGGGGAATAAATCTTTGGGCACCTAGTGGTCATG

24 CATGACCACTAGGAGCATCTTTGGCGATGGGGCCGTGCTACCTGCACACTCCGCGGGCTAGGGGAATAAATCTTTGGGCACCTAGTGGTCATG

24 CATGACCACTAGGAGCATCTTTGGCGAGAAGACTCTGGATTCAGGGACCAGTTGCTGCTAGGGGAATAAATCTTTGGGCACCTAGTGGTCATG

24 CATGACCACTAGGAGCATCTTTGGCGATGAGCTCGGCGGCGGTCGTCGGTTCCGCCCCTAGGGGAATAAATCTTTGGGCACCTAGTGGTCATG

24 CATGACCACTAGGAGCATCTTTGGCGACGGATACGGTAGGTTTGTGTGCCTAGCCTGCTAGGGGAATAAATCTTTGGGCACCTAGTGGTCATG

23 CATGACCACTAGGAGCATCTTTGGCGAAGGGTGTAGGACTTTAAGTGGATCTCATAGCTAGGGGAATAAATCTTTGGGCACCTAGTGGTCATG

23 CATGACCACTAGGAGCATCTTTGGCGACCCCCTCCGTTGCGGGTTTGACGAGGTTTGCTAGGGGAATAAATCTTTGGGCACCTAGTGGTCATG

23 CATGACCACTAGGAGCATCTTTGGCGAGGTAGCTATCGAAGTTATTACCTTTAGGTACTAGGGGAATAAATCTTTGGGCACCTAGTGGTCATG

23 CATGACCACTAGGAGCATCTTTGGCGAAAGCATAGCTGTGCGTGTCGGTCTAGCATCCTAGGGGAATAAATCTTTGGGCACCTAGTGGTCATG

23 CATGACCACTAGGAGCATCTTTGGCGACCCACACGAGGACTCGAGTAGTGTGGTGGACTAGGGGAATAAATCTTTGGGCACCTAGTGGTCATG

23 CATGACCACTAGGAGCATCTTTGGCGACCCAGACTGATGTAATCTGTAATGGGTGGACTAGGGGAATAAATCTTTGGGCACCTAGTGGTCATG

23 CATGACCACTAGGAGCATCTTTGGCGAGGCTTTCGGTGGTAAGGACTACGTCTAGAGCTAGGGGAATAAATCTTTGGGCACCTAGTGGTCATG

23 CATGACCACTAGGAGCATCTTTGGCGACGATTGGTCGGAAGGCAAGTGTTCGGATGACTAGGGGAATAAATCTTTGGGCACCTAGTGGTCATG

23 CATGACCACTAGGAGCATCTTTGGCGAGTGGACTCAATCATACGAGCAGACGTTTGGCTAGGGGAATAAATCTTTGGGCACCTAGTGGTCATG

22 CATGACCACTAGGAGCATCTTTGGCGAAATTCGGTAGAACAGGGTGGGGTGCTGTCCCTAGGGGAATAAATCTTTGGGCACCTAGTGGTCATG

22 CATGACCACTAGGAGCATCTTTGGCGACGGTGTGGGGACCGCAGGCTCGGCGGGTACCTAGGGGAATAAATCTTTGGGCACCTAGTGGTCATG

22 CATGACCACTAGGAGCATCTTTGGCGAATGGCCGGCACGGCCTTCTAGTCATCGGTACTAGGGGAATAAATCTTTGGGCACCTAGTGGTCATG

22 CATGACCACTAGGAGCATCTTTGGCGAGAAGACTCTGGATTCGGGGACTAGTTGCTGCTAGGGGAATAAATCTTTGGGCACCTAGTGGTCATG

22 CATGACCACTAGGAGCATCTTTGGCGACATCCTGAGCTGGCCCAGATCAGGTGTGGACTAGGGGAATAAATCTTTGGGCACCTAGTGGTCATG

22 CATGACCACTAGGAGCATCTTTGGCGAATGGCCGGCACGGCCCTCTAGTCCTCGGTACTAGGGGAATAAATCTTTGGGCACCTAGTGGTCATG

22 CATGACCACTAGGAGCATCTTTGGCGAGGGAGGGCGCCGGTAGCGGTGTGAATGCGCCTAGGGGAATAAATCTTTGGGCACCTAGTGGTCATG

22 CATGACCACTAGGAGCATCTTTGGCGAGATCGGGAGAATCGGTGGCATTCGTGTCTCCTAGGGGAATAAATCTTTGGGCACCTAGTGGTCATG

22 CATGACCACTAGGAGCATCTTTGGCGAGATCGGGAGAATCGGTGGCTTTGGTGTCTCCTAGGGGAATAAATCTTTGGGCACCTAGTGGTCATG

22 CATGACCACTAGGAGCATCTTTGGCGAGCCTCGCTTGGGAGGTTGCTCCACCAGCTCCTAGGGGAATAAATCTTTGGGCACCTAGTGGTCATG

21 CATGACCACTAGGAGCATCTTTGGCGAGAAGACTCTGGATTCGAGGACCAGTTGCTGCTAGGGGAATAAATCTTTGGGCACCTAGTGGTCATG

21 CATGACCACTAGGAGCATCTTTGGCGAGTTGTCGGAGAGCCAGGTGTCAAACCTCAGCTAGGGGAATAAATCTTTGGGCACCTAGTGGTCATG

21 CATGACCACTAGGAGCATCTTTGGCGATCCTGTAGGAAGAGTAGCGGCTTCGTGAGACTAGGGGAATAAATCTTTGGGCACCTAGTGGTCATG

21 CATGACCACTAGGAGCATCTTTGGCGAATGTAGGTTGACTGTAAGGTAGGTTAGTTCCTAGGGGAATAAATCTTTGGGCACCTAGTGGTCATG

21 CATGACCACTAGGAGCATCTTTGGCGACGGTGTGGGGAACAAGGGTTCGGCGGCTCTCTAGGGGAATAAATCTTTGGGCACCTAGTGGTCATG

21 CATGACCACTAGGAGCATCTTTGGCGAATGGCAGGCACGGCCTTCTAGTCCTCGGTACTAGGGGAATAAATCTTTGGGCACCTAGTGGTCATG

21 CATGACCACTAGGAGCATCTTTGGCGAATGGCCGGCACGGCCTTCTAGCCCTCGGTACTAGGGGAATAAATCTTTGGGCACCTAGTGGTCATG

21 CATGACCACTAGGAGCATCTTTGGCGAAGGTCGGTAGAACAGGGTGGGGTGCTGTCCCTAGGGGAATAAATCTTTGGGCACCTAGTGGTCATG

21 CATGACCACTAGGAGCATCTTTGGCGACAGTCGGGTGCGAGATAAAAAATCGGCCAGCTAGGGGAATAAATCTTTGGGCACCTAGTGGTCATG

21 CATGACCACTAGGAGCATCTTTGGCGAATGGCCGGCACGGCCTACTAGTCCTCGGTACTAGGGGAATAAATCTTTGGGCACCTAGTGGTCATG

21 CATGACCACTAGGAGCATCTTTGGCGAAAGTCGGTAGAACAGTGTGGGGTGCTGTCCCTAGGGGAATAAATCTTTGGGCACCTAGTGGTCATG

21 CATGACCACTAGGAGCATCTTTGGCGAGTTGGGGGCACGACCTTGGTAGGTATAAAGCTAGGGGAATAAATCTTTGGGCACCTAGTGGTCATG

21 CATGACCACTAGGAGCATCTTTGGCGAACCTCGTCAGATGGTGAAGCAGACGTTTGGCTAGGGGAATAAATCTTTGGGCACCTAGTGGTCATG

21 CATGACCACTAGGAGCATCTTTGGCGAATGGCCGGCACGGCCTTCTAGTCCTCAGTACTAGGGGAATAAATCTTTGGGCACCTAGTGGTCATG

21 CATGACCACTAGGAGCATCTTTGGCGAGAAGACTCTGGATTCGGGGACCAGTTGCTTCTAGGGGAATAAATCTTTGGGCACCTAGTGGTCATG

21 CATGACCACTAGGAGCATCTTTGGCGACAGCCGTAGCCGTAAGGTTGGTATAAGGTCCTAGGGGAATAAATCTTTGGGCACCTAGTGGTCATG

20 CATGACCACTAGGAGCATCTTTGGCGAATGGCCGGCACGGCCTTCTAGTACTCGGTACTAGGGGAATAAATCTTTGGGCACCTAGTGGTCATG

20 CATGACCACTAGGAGCATCTTTGGCGAACGGCAGGTGTGGCGGTGGTCTGTGAATCCCTAGGGGAATAAATCTTTGGGCACCTAGTGGTCATG

20 CATGACCACTAGGAGCATCTTTGGCGACGGCGTGGGGCTATTGATGCGGCGGCACTTCTAGGGGAATAAATCTTTGGGCACCTAGTGGTCATG

20 CATGACCACTAGGAGCATCTTTGGCGAGTGGTGGTCGGACGGAAAGCATACACGAGACTAGGGGAATAAATCTTTGGGCACCTAGTGGTCATG

20 CATGACCACTAGGAGCATCTTTGGCGACTCGACCGGGGCCCTAGGGTTGCGGCCCACCTAGGGGAATAAATCTTTGGGCACCTAGTGGTCATG

20 CATGACCACTAGGAGCATCTTTGGCGACCCATGGAATGCCACATTCAATGCGGTGGACTAGGGGAATAAATCTTTGGGCACCTAGTGGTCATG

20 CATGACCACTAGGAGCATCTTTGGCGAGTGGGGGGCGCCGGCCGGTAGTGGGTGAGACTAGGGGAATAAATCTTTGGGCACCTAGTGGTCATG

20 CATGACCACTAGGAGCATCTTTGGCGACGGTGTGGGGCTATTGATGCGGCGGCACTCCTAGGGGAATAAATCTTTGGGCACCTAGTGGTCATG

20 CATGACCACTAGGAGCATCTTTGGCGACACATTGACGTCAACCAATGCGAAGTGGAGCTAGGGGAATAAATCTTTGGGCACCTAGTGGTCATG

20 CATGACCACTAGGAGCATCTTTGGCGAATGGCCGGCAAGGCCTTCTAGTCCTCGGTACTAGGGGAATAAATCTTTGGGCACCTAGTGGTCATG

20 CATGACCACTAGGAGCATCTTTGGCGAGTCGGGGATTGGCAGGGGCGTTGACTGGCACTAGGGGAATAAATCTTTGGGCACCTAGTGGTCATG

20 CATGACCACTAGGAGCATCTTTGGCGAGGAAGATGGTAGGGTAATGTGTCGGCGCCGCTAGGGGAATAAATCTTTGGGCACCTAGTGGTCATG

20 CATGACCACTAGGAGCATCTTTGGCGAATGGCCGGAACGGCCTTCTAGTCCTCGGTACTAGGGGAATAAATCTTTGGGCACCTAGTGGTCATG

20 CATGACCACTAGGAGCATCTTTGGCGATGGGTGTTGACGGCCGCCGCTGCGGCTACACTAGGGGAATAAATCTTTGGGCACCTAGTGGTCATG

20 CATGACCACTAGGAGCATCTTTGGCGAAGCGAAGGAGCTCGTAAGGCGGCGTACCGGCTAGGGGAATAAATCTTTGGGCACCTAGTGGTCATG

20 CATGACCACTAGGAGCATCTTTGGCGATAAGACTCTGGATTCGGGGACCAGTTGCTGCTAGGGGAATAAATCTTTGGGCACCTAGTGGTCATG

20 CATGACCACTAGGAGCATCTTTGGCGAAGCGTAGTCGTTAGATTGGTCAGGTTGATCCTAGGGGAATAAATCTTTGGGCACCTAGTGGTCATG

20 CATGACCACTAGGAGCATCTTTGGCGACGGGGTGGGGCCGAAGTGCGGCGGCGACTCCTAGGGGAATAAATCTTTGGGCACCTAGTGGTCATG

20 CATGACCACTAGGAGCATCTTTGGCGAAGGGCGCTAGTGATTCGGTGGTTGCCCCCGCTAGGGGAATAAATCTTTGGGCACCTAGTGGTCATG

20 CATGACCACTAGGAGCATCTTTGGCGACTGTGGGTTGGTAGGGGTGTACCTGTGGCGCTAGGGGAATAAATCTTTGGGCACCTAGTGGTCATG

19 CATGACCACTAGGAGCATCTTTGGCGACCCGTGGTACAGTGTAACCCACGTGGTGGACTAGGGGAATAAATCTTTGGGCACCTAGTGGTCATG

19 CATGACCACTAGGAGCATCTTTGGCGAGCCCCACAACGAATGTATGTGTTTGGTCGCCTAGGGGAATAAATCTTTGGGCACCTAGTGGTCATG

19 CATGACCACTAGGAGCATCTTTGGCGATCCTCGCTTGGGAGGTTGCTCCACCAGTTCCTAGGGGAATAAATCTTTGGGCACCTAGTGGTCATG

19 CATGACCACTAGGAGCATCTTTGGCGAGAAGACTCTGGATACGGGGACCAGTTGCTGCTAGGGGAATAAATCTTTGGGCACCTAGTGGTCATG

19 CATGACCACTAGGAGCATCTTTGGCGAAAGTCGGTAGGACAGGGTGGGGTGCTGTCCCTAGGGGAATAAATCTTTGGGCACCTAGTGGTCATG

19 CATGACCACTAGGAGCATCTTTGGCGAGGTTGTTCGAAGTTATCACCCTTAAGGGTGCTAGGGGAATAAATCTTTGGGCACCTAGTGGTCATG

19 CATGACCACTAGGAGCATCTTTGGCGAAGTGCGGCGTGCCGTTGTCCGTGCGGCAACCTAGGGGAATAAATCTTTGGGCACCTAGTGGTCATG

19 CATGACCACTAGGAGCATCTTTGGCGAGCCTCGCTCGGGAGGTTGCTCCACCAGTTCCTAGGGGAATAAATCTTTGGGCACCTAGTGGTCATG

19 CATGACCACTAGGAGCATCTTTGGCGAGTGGCCGGCACGGCCTTCTAGTCCTCGGTACTAGGGGAATAAATCTTTGGGCACCTAGTGGTCATG

19 CATGACCACTAGGAGCATCTTTGGCGAGGTTGGTGCAGGCTCTGCCTGTCGTCGAGACTAGGGGAATAAATCTTTGGGCACCTAGTGGTCATG

19 CATGACCACTAGGAGCATCTTTGGCGAGATCGGGAGAATCGGTGGCATTGGTCTCTCCTAGGGGAATAAATCTTTGGGCACCTAGTGGTCATG

19 CATGACCACTAGGAGCATCTTTGGCGAGGGGTAGGGGGCCGCTCGCGCCTGCGGGCGCTAGGGGAATAAATCTTTGGGCACCTAGTGGTCATG

19 CATGACCACTAGGAGCATCTTTGGCGAATGGCCGTCACGGCCTTCTAGTCCTCGGTACTAGGGGAATAAATCTTTGGGCACCTAGTGGTCATG

18 CATGACCACTAGGAGCATCTTTGGCGATCCTCGTCAGACGGTGAAGCAGACGTTTGGCTAGGGGAATAAATCTTTGGGCACCTAGTGGTCATG

18 CATGACCACTAGGAGCATCTTTGGCGACATAGTGGATCGCAGTCCCGAAAAGGACGCCTAGGGGAATAAATCTTTGGGCACCTAGTGGTCATG

18 CATGACCACTAGGAGCATCTTTGGCGAAAGTCGGTAGAACAGGGTGGGGTGCTGTTCCTAGGGGAATAAATCTTTGGGCACCTAGTGGTCATG

18 CATGACCACTAGGAGCATCTTTGGCGAATGGCCGGCACGGCCTCCTAGTCCTTGGTACTAGGGGAATAAATCTTTGGGCACCTAGTGGTCATG

18 CATGACCACTAGGAGCATCTTTGGCGAAGAGCTGTGCACGTGGGTGTGGTCCGGGCCCTAGGGGAATAAATCTTTGGGCACCTAGTGGTCATG

18 CATGACCACTAGGAGCATCTTTGGCGATAAGATATGGAAGGTAAATGTGCTTAATAGCTAGGGGAATAAATCTTTGGGCACCTAGTGGTCATG

18 CATGACCACTAGGAGCATCTTTGGCGACGTGGCTGGTAGGGTAAGTACTGTGTCGATCTAGGGGAATAAATCTTTGGGCACCTAGTGGTCATG

18 CATGACCACTAGGAGCATCTTTGGCGAGATCGGGAGAAGCGGTGGCATTGGTGTCTCCTAGGGGAATAAATCTTTGGGCACCTAGTGGTCATG

18 CATGACCACTAGGAGCATCTTTGGCGATGGGGGGCGAGAGGACTGAGTAGGAATAAGCTAGGGGAATAAATCTTTGGGCACCTAGTGGTCATG

18 CATGACCACTAGGAGCATCTTTGGCGAGAAGACTCTGGATTCGGGGATCAGTTGCTGCTAGGGGAATAAATCTTTGGGCACCTAGTGGTCATG

18 CATGACCACTAGGAGCATCTTTGGCGAAAGTCGGTAGAACAGGGTGGGGTGCTATCCCTAGGGGAATAAATCTTTGGGCACCTAGTGGTCATG

18 CATGACCACTAGGAGCATCTTTGGCGAGCGGTTCGTCCCGGCCGATGCTCGGACAAGCTAGGGGAATAAATCTTTGGGCACCTAGTGGTCATG

18 CATGACCACTAGGAGCATCTTTGGCGAGAAGACTCTGGATTCGGTGACCAGTTGCTGCTAGGGGAATAAATCTTTGGGCACCTAGTGGTCATG

18 CATGACCACTAGGAGCATCTTTGGCGAAAACAAAAGACTATGCTAAGAGAAATTCGCCTAGGGGAATAAATCTTTGGGCACCTAGTGGTCATG

18 CATGACCACTAGGAGCATCTTTGGCGAGAAGACTCTGGAGTCGGGGACCAGTTGCTGCTAGGGGAATAAATCTTTGGGCACCTAGTGGTCATG

18 CATGACCACTAGGAGCATCTTTGGCGACGGCGTGGGGCCATCTGTGCGGCGGATCTCCTAGGGGAATAAATCTTTGGGCACCTAGTGGTCATG

18 CATGACCACTAGGAGCATCTTTGGCGACGGGATCGTGTCGGCGGATAGCGTACTTGGCTAGGGGAATAAATCTTTGGGCACCTAGTGGTCATG

18 CATGACCACTAGGAGCATCTTTGGCGAGCGGAGCGGAGTTTACGTTGGTGGGTAGCCCTAGGGGAATAAATCTTTGGGCACCTAGTGGTCATG

18 CATGACCACTAGGAGCATCTTTGGCGATCAGCATAGTATATGCGCCTGGAGCGTAGGCTAGGGGAATAAATCTTTGGGCACCTAGTGGTCATG

17 CATGACCACTAGGAGCATCTTTGGCGACCCGAAAGGAGTGGGAGAGGCGAAGGTCGACTAGGGGAATAAATCTTTGGGCACCTAGTGGTCATG

17 CATGACCACTAGGAGCATCTTTGGCGAACGGCAGGTGTTGCGGTGGTCTGTGACTCCCTAGGGGAATAAATCTTTGGGCACCTAGTGGTCATG

17 CATGACCACTAGGAGCATCTTTGGCGACGGCGTGGGGCTATTGATGCGGCGGCACCCCTAGGGGAATAAATCTTTGGGCACCTAGTGGTCATG

17 CATGACCACTAGGAGCATCTTTGGCGATGGGTGTTGACGGGGCTAGTGAGCCCTACACTAGGGGAATAAATCTTTGGGCACCTAGTGGTCATG

17 CATGACCACTAGGAGCATCTTTGGCGATGGGACCCCGTCCTCTAACGGGCAACGGTCCTAGGGGAATAAATCTTTGGGCACCTAGTGGTCATG

17 CATGACCACTAGGAGCATCTTTGGCGAAAAGACTCTGGATTCGGGGACCAGTTGCTGCTAGGGGAATAAATCTTTGGGCACCTAGTGGTCATG

17 CATGACCACTAGGAGCATCTTTGGCGACGGTGTGGGGGCGACGGCCGGCGGTTACCTCTAGGGGAATAAATCTTTGGGCACCTAGTGGTCATG

17 CATGACCACTAGGAGCATCTTTGGCGACGGCGTGGGGCTATTGATGCGGCGGCATTCCTAGGGGAATAAATCTTTGGGCACCTAGTGGTCATG

17 CATGACCACTAGGAGCATCTTTGGCGACCGTAGGAAAACAAAAGTGCCCTTGCTAGCCTAGGGGAATAAATCTTTGGGCACCTAGTGGTCATG

17 CATGACCACTAGGAGCATCTTTGGCGACTTAACAGGTCCGTTGTGATCGCGAGGACCCTAGGGGAATAAATCTTTGGGCACCTAGTGGTCATG

17 CATGACCACTAGGAGCATCTTTGGCGAGCCTCGCTTGGGAGGTTGCACCACCAGTTCCTAGGGGAATAAATCTTTGGGCACCTAGTGGTCATG

17 CATGACCACTAGGAGCATCTTTGGCGACCGGCGGAGACATGCATGCGCGTTGTGGAGCTAGGGGAATAAATCTTTGGGCACCTAGTGGTCATG

17 CATGACCACTAGGAGCATCTTTGGCGACCGTAGGAGAACAAAAGTGGCCTTGCTAGCCTAGGGGAATAAATCTTTGGGCACCTAGTGGTCATG

17 CATGACCACTAGGAGCATCTTTGGCGAGGGAGTGGTCGGCCATGTGGGGGGTCGGGGCTAGGGGAATAAATCTTTGGGCACCTAGTGGTCATG

17 CATGACCACTAGGAGCATCTTTGGCGATGGATACGGGAGGACAGTGTGCCAGAATGCCTAGGGGAATAAATCTTTGGGCACCTAGTGGTCATG

17 CATGACCACTAGGAGCATCTTTGGCGATCCTCGTCAGGTGGTGAAGCAGACGTTTGGCTAGGGGAATAAATCTTTGGGCACCTAGTGGTCATG

17 CATGACCACTAGGAGCATCTTTGGCGAGCCGACTCGGGGGCCGAGTATGCCTGGGTCCTAGGGGAATAAATCTTTGGGCACCTAGTGGTCATG

17 CATGACCACTAGGAGCATCTTTGGCGAAAGCGAAGTGCGTCGTCGTGGCTGAGGCACCTAGGGGAATAAATCTTTGGGCACCTAGTGGTCATG

16 CATGACCACTAGGAGCATCTTTGGCGAAGCGTGTTGCAAGGTCCGCGTGTGTGGACCCTAGGGGAATAAATCTTTGGGCACCTAGTGGTCATG

16 CATGACCACTAGGAGCATCTTTGGCGACGTGGGCTGGAAGGATGTGGTACTTCTCGACTAGGGGAATAAATCTTTGGGCACCTAGTGGTCATG

16 CATGACCACTAGGAGCATCTTTGGCGAGATCGGGAGAATCGGTCGCATTGGTGTCTCCTAGGGGAATAAATCTTTGGGCACCTAGTGGTCATG

16 CATGACCACTAGGAGCATCTTTGGCGAGAAGACTCTTGATTCGGGGACCAGTTGCTGCTAGGGGAATAAATCTTTGGGCACCTAGTGGTCATG

16 CATGACCACTAGGAGCATCTTTGGCGAGGGGTAGGGGGCCGCTCGCGCCAGCGTGCGCTAGGGGAATAAATCTTTGGGCACCTAGTGGTCATG

16 CATGACCACTAGGAGCATCTTTGGCGAGCCTCGCTTGGGAGGTTGCTACACCAGTTCCTAGGGGAATAAATCTTTGGGCACCTAGTGGTCATG

16 CATGACCACTAGGAGCATCTTTGGCGAATGGCCGGCACGGCCTTCTGGTCCTCGGTACTAGGGGAATAAATCTTTGGGCACCTAGTGGTCATG

16 CATGACCACTAGGAGCATCTTTGGCGATCTAGTGGACACGCGTTATCATTGGGAAGGCTAGGGGAATAAATCTTTGGGCACCTAGTGGTCATG

16 CATGACCACTAGGAGCATCTTTGGCGAGGCGGAGGGTCCGCCGGGGACCAGTTGCTGCTAGGGGAATAAATCTTTGGGCACCTAGTGGTCATG

16 CATGACCACTAGGAGCATCTTTGGCGACGTGGCTGGCAGGATGGTATTCGTTCGCTGCTAGGGGAATAAATCTTTGGGCACCTAGTGGTCATG

16 CATGACCACTAGGAGCATCTTTGGCGAGCCTTCGTATTTGTACGTGCTGTCTGGGTCCTAGGGGAATAAATCTTTGGGCACCTAGTGGTCATG

16 CATGACCACTAGGAGCATCTTTGGCGAAAGTCGGTAGAACAGGGTGGGGTGATGTCCCTAGGGGAATAAATCTTTGGGCACCTAGTGGTCATG

16 CATGACCACTAGGAGCATCTTTGGCGAGAAGACTCTGGATTCGGGAACCAGTTGCTGCTAGGGGAATAAATCTTTGGGCACCTAGTGGTCATG

16 CATGACCACTAGGAGCATCTTTGGCGACCCGGTCTTACGAAGGAATGCCGTGGTGGACTAGGGGAATAAATCTTTGGGCACCTAGTGGTCATG

16 CATGACCACTAGGAGCATCTTTGGCGACGGCATTTGTTAGGCTCCGGACAGTGTCACCTAGGGGAATAAATCTTTGGGCACCTAGTGGTCATG

16 CATGACCACTAGGAGCATCTTTGGCGAAGGGTGCAGGACTTCAAGTGGATCTCATAGCTAGGGGAATAAATCTTTGGGCACCTAGTGGTCATG

16 CATGACCACTAGGAGCATCTTTGGCGATCCCCCTTGGCACGGGTCTTAATGGATTTGCTAGGGGAATAAATCTTTGGGCACCTAGTGGTCATG

16 CATGACCACTAGGAGCATCTTTGGCGATGGGTGCTGACGGCCGCCGCCGCGGCTACACTAGGGGAATAAATCTTTGGGCACCTAGTGGTCATG

16 CATGACCACTAGGAGCATCTTTGGCGACGGCGTGGCTGGCACCAATAGTTTACGCACCTAGGGGAATAAATCTTTGGGCACCTAGTGGTCATG

16 CATGACCACTAGGAGCATCTTTGGCGAGTTCTCCGCCGAGAATGAGCAGACGTTTGGCTAGGGGAATAAATCTTTGGGCACCTAGTGGTCATG

16 CATGACCACTAGGAGCATCTTTGGCGAATGGCCGGCACGGCATTCTAGTCCTCGGTACTAGGGGAATAAATCTTTGGGCACCTAGTGGTCATG

16 CATGACCACTAGGAGCATCTTTGGCGAAAGTCGGTAGAACCGGGTGGGGTGCTGTCCCTAGGGGAATAAATCTTTGGGCACCTAGTGGTCATG

15 CATGACCACTAGGAGCATCTTTGGCGAACCTCGCTTGGGAGGTTGCTCCACCAGTTCCTAGGGGAATAAATCTTTGGGCACCTAGTGGTCATG

15 CATGACCACTAGGAGCATCTTTGGCGAGATCGGGAGAATCCGTGGCATTGGTGTCTCCTAGGGGAATAAATCTTTGGGCACCTAGTGGTCATG

15 CATGACCACTAGGAGCATCTTTGGCGAACGGCAGGTGTTGCGGTGGTCTGGGAATCCCTAGGGGAATAAATCTTTGGGCACCTAGTGGTCATG

15 CATGACCACTAGGAGCATCTTTGGCGATAAAAGAGATAAAGTGACATAGATATTCGCCTAGGGGAATAAATCTTTGGGCACCTAGTGGTCATG

15 CATGACCACTAGGAGCATCTTTGGCGACGGTCGGTCGGTAGGATAGTATTCCGTTGGCTAGGGGAATAAATCTTTGGGCACCTAGTGGTCATG

15 CATGACCACTAGGAGCATCTTTGGCGATGGGCGAAAGAGACAACTGAGGAGACCGCCCTAGGGGAATAAATCTTTGGGCACCTAGTGGTCATG

15 CATGACCACTAGGAGCATCTTTGGCGAATGGCCGGCACGGCCTTCTAGTCCTCGTTACTAGGGGAATAAATCTTTGGGCACCTAGTGGTCATG

15 CATGACCACTAGGAGCATCTTTGGCGAGGCTCCGTGTGAGGCAGAGCAGACGTTTGGCTAGGGGAATAAATCTTTGGGCACCTAGTGGTCATG

15 CATGACCACTAGGAGCATCTTTGGCGAACGGCAGGTGTTGCGGTGGTCTGTGAATCGCTAGGGGAATAAATCTTTGGGCACCTAGTGGTCATG

15 CATGACCACTAGGAGCATCTTTGGCGACACTGTCCCACGCGTTATAGGTTGCTCGAGCTAGGGGAATAAATCTTTGGGCACCTAGTGGTCATG

15 CATGACCACTAGGAGCATCTTTGGCGATGGGGCCGTGCTATCAGCACACTCCGCGGGCTAGGGGAATAAATCTTTGGGCACCTAGTGGTCATG

15 CATGACCACTAGGAGCATCTTTGGCGAATGCTGCCGTGGGCAAGCGGGTGTGAGGAACTAGGGGAATAAATCTTTGGGCACCTAGTGGTCATG

15 CATGACCACTAGGAGCATCTTTGGCGACGCAACAAGTACGTAGGACGTTGTCGTGGACTAGGGGAATAAATCTTTGGGCACCTAGTGGTCATG

15 CATGACCACTAGGAGCATCTTTGGCGAGAAGACTCTGGATTCGGGGACCAGTTACTGCTAGGGGAATAAATCTTTGGGCACCTAGTGGTCATG

15 CATGACCACTAGGAGCATCTTTGGCGATGGGTGCTGACGGCCGCCCCTGCGGCTACACTAGGGGAATAAATCTTTGGGCACCTAGTGGTCATG

15 CATGACCACTAGGAGCATCTTTGGCGAAGTTCGTTGGGCCTGGGCCCGGTTGGAGACCTAGGGGAATAAATCTTTGGGCACCTAGTGGTCATG

15 CATGACCACTAGGAGCATCTTTGGCGATGGGGATGGCAGAGCAATGGGGAGTTGGGGCTAGGGGAATAAATCTTTGGGCACCTAGTGGTCATG

15 CATGACCACTAGGAGCATCTTTGGCGAGATGGGGAGAATCGGTGGCATTGGTGTCTCCTAGGGGAATAAATCTTTGGGCACCTAGTGGTCATG

15 CATGACCACTAGGAGCATCTTTGGCGAGCACTACGTAGCGGTAGGCATTGTTGTCTCCTAGGGGAATAAATCTTTGGGCACCTAGTGGTCATG

15 CATGACCACTAGGAGCATCTTTGGCGAATGGCCGGCACGGCCTTCCAGTCCTCGGTACTAGGGGAATAAATCTTTGGGCACCTAGTGGTCATG

15 CATGACCACTAGGAGCATCTTTGGCGACTGTGGCTGGTAGGTTCGTACTATAGGCGGCTAGGGGAATAAATCTTTGGGCACCTAGTGGTCATG

15 CATGACCACTAGGAGCATCTTTGGCGAAAGTCGGTGGAACAGGGTGGGGTGCTGTCCCTAGGGGAATAAATCTTTGGGCACCTAGTGGTCATG

15 CATGACCACTAGGAGCATCTTTGGCGATGGGTGCTGACGGCCGCCGTTGCGGCTACACTAGGGGAATAAATCTTTGGGCACCTAGTGGTCATG

15 CATGACCACTAGGAGCATCTTTGGCGATTGGTAGTCGCGGATGCAGGTAGTGATGCTCTAGGGGAATAAATCTTTGGGCACCTAGTGGTCATG

15 CATGACCACTAGGAGCATCTTTGGCGACGGTGTGGGGAACTCGTTTCGGCGGTGCTACTAGGGGAATAAATCTTTGGGCACCTAGTGGTCATG

15 CATGACCACTAGGAGCATCTTTGGCGAATGGCCGGCACGGCCTTCTAGTCCTCGGAACTAGGGGAATAAATCTTTGGGCACCTAGTGGTCATG

15 CATGACCACTAGGAGCATCTTTGGCGAGGTCCGAGCGATGCGTGCCGTGGTCGAGCTCTAGGGGAATAAATCTTTGGGCACCTAGTGGTCATG

15 CATGACCACTAGGAGCATCTTTGGCGACGGTGTGGGGAACTTGTCTCGGCGGTGCTACTAGGGGAATAAATCTTTGGGCACCTAGTGGTCATG

15 CATGACCACTAGGAGCATCTTTGGCGAGAAGACCTTGGATTCGGGGACCAGTTGCTGCTAGGGGAATAAATCTTTGGGCACCTAGTGGTCATG

15 CATGACCACTAGGAGCATCTTTGGCGAAACGAGCGCTCGAGTGTATCCGAAGTGGAGCTAGGGGAATAAATCTTTGGGCACCTAGTGGTCATG

15 CATGACCACTAGGAGCATCTTTGGCGACGGTGTGGGGAACTTGTTTCGGCGGTGCTCCTAGGGGAATAAATCTTTGGGCACCTAGTGGTCATG

15 CATGACCACTAGGAGCATCTTTGGCGAAAGTCGGTAGAACAGGGTGGGGTTCTGTCCCTAGGGGAATAAATCTTTGGGCACCTAGTGGTCATG

15 CATGACCACTAGGAGCATCTTTGGCGAGTGCGGGCAACACGCAGTCCTGGTGTGCGACTAGGGGAATAAATCTTTGGGCACCTAGTGGTCATG

14 CATGACCACTAGGAGCATCTTTGGCGAGGGCTTTGGCTGGTAGGGCGTACTCCCTTGCTAGGGGAATAAATCTTTGGGCACCTAGTGGTCATG

14 CATGACCACTAGGAGCATCTTTGGCGAAAGTCGGTATAACAGGGTGGGGTGCTGTCCCTAGGGGAATAAATCTTTGGGCACCTAGTGGTCATG

14 CATGACCACTAGGAGCATCTTTGGCGAATGGACGGCACGGCCTTCTAGTCCTCGGTACTAGGGGAATAAATCTTTGGGCACCTAGTGGTCATG

14 CATGACCACTAGGAGCATCTTTGGCGATGGGGCCGTGCTATCTGCACACTCTGCGGGCTAGGGGAATAAATCTTTGGGCACCTAGTGGTCATG

14 CATGACCACTAGGAGCATCTTTGGCGATGGGGCCGTGCTGTCTGCACACTCCGCGGGCTAGGGGAATAAATCTTTGGGCACCTAGTGGTCATG

14 CATGACCACTAGGAGCATCTTTGGCGAGGTGTTGGAAGCGGAAGCCAGGGGCGAGCTCTAGGGGAATAAATCTTTGGGCACCTAGTGGTCATG

14 CATGACCACTAGGAGCATCTTTGGCGAACGGCCGGCACGGCCTTCTAGTCCTTGGTACTAGGGGAATAAATCTTTGGGCACCTAGTGGTCATG

14 CATGACCACTAGGAGCATCTTTGGCGAGCCTCGCTTGGGAGGTTGCTCCGCCAGTTCCTAGGGGAATAAATCTTTGGGCACCTAGTGGTCATG

14 CATGACCACTAGGAGCATCTTTGGCGAGAAGCCTCTGGATTCGGGGACCAGTTGCTGCTAGGGGAATAAATCTTTGGGCACCTAGTGGTCATG

14 CATGACCACTAGGAGCATCTTTGGCGACCAGCTGAGGCGTCATGCAGCGGAGTGGAACTAGGGGAATAAATCTTTGGGCACCTAGTGGTCATG

14 CATGACCACTAGGAGCATCTTTGGCGACCTGGTGGGACATGCTCAAACCAGTGTGGACTAGGGGAATAAATCTTTGGGCACCTAGTGGTCATG

14 CATGACCACTAGGAGCATCTTTGGCGAAGCGTACGAGGCAGGTCGACGTATAAGACCCTAGGGGAATAAATCTTTGGGCACCTAGTGGTCATG

14 CATGACCACTAGGAGCATCTTTGGCGATGTGATCGACGCGCTGTAGGTTGCGGTAAGCTAGGGGAATAAATCTTTGGGCACCTAGTGGTCATG

14 CATGACCACTAGGAGCATCTTTGGCGACGTCGTGGCTGGTAGAGTGTATTTGACAAGCTAGGGGAATAAATCTTTGGGCACCTAGTGGTCATG

14 CATGACCACTAGGAGCATCTTTGGCGAAGACGACGCAGTCGATCGGGATAGAGGAAGCTAGGGGAATAAATCTTTGGGCACCTAGTGGTCATG

14 CATGACCACTAGGAGCATCTTTGGCGATTGGATATCCAAGACAGAGCAGACGTTTGGCTAGGGGAATAAATCTTTGGGCACCTAGTGGTCATG

14 CATGACCACTAGGAGCATCTTTGGCGAAAGTCGGTAGAACGGGGTGGGGTGCTGTCCCTAGGGGAATAAATCTTTGGGCACCTAGTGGTCATG

14 CATGACCACTAGGAGCATCTTTGGCGACGGTGTGGGGACCGCAGGTTCGGCGGCTACCTAGGGGAATAAATCTTTGGGCACCTAGTGGTCATG

14 CATGACCACTAGGAGCATCTTTGGCGACACGACATTAGGAACGAATGCCGCTGTGGACTAGGGGAATAAATCTTTGGGCACCTAGTGGTCATG

14 CATGACCACTAGGAGCATCTTTGGCGAATGGCCGGCACGGCCTTTTAGTCCTCGGTACTAGGGGAATAAATCTTTGGGCACCTAGTGGTCATG

14 CATGACCACTAGGAGCATCTTTGGCGAAAGTCGGTAGAACAGGGAGGGGTGCTGTCCCTAGGGGAATAAATCTTTGGGCACCTAGTGGTCATG

14 CATGACCACTAGGAGCATCTTTGGCGAGAAGACTCTGGATTCGGAGACCAGTTGCTGCTAGGGGAATAAATCTTTGGGCACCTAGTGGTCATG

14 CATGACCACTAGGAGCATCTTTGGCGAAGCTTGGTTAGGCGACGGGCCTGGGCCACTCTAGGGGAATAAATCTTTGGGCACCTAGTGGTCATG

14 CATGACCACTAGGAGCATCTTTGGCGAGAAGACTGTGGATTCGGGGACCAGTTGCTGCTAGGGGAATAAATCTTTGGGCACCTAGTGGTCATG

13 CATGACCACTAGGAGCATCTTTGGCGATGGGTGCAGACGAGTGCGCATGCAATTACACTAGGGGAATAAATCTTTGGGCACCTAGTGGTCATG

13 CATGACCACTAGGAGCATCTTTGGCGAAAGTCGGTAGAACAGGGTGGGGTCCTGTCCCTAGGGGAATAAATCTTTGGGCACCTAGTGGTCATG

13 CATGACCACTAGGAGCATCTTTGGCGACTAGTCATAGAGAACAAAGGTTAAGAGTCGCTAGGGGAATAAATCTTTGGGCACCTAGTGGTCATG

13 CATGACCACTAGGAGCATCTTTGGCGATTAGCGTCTTGCGAGCGGGTGGTTCGCTCCCTAGGGGAATAAATCTTTGGGCACCTAGTGGTCATG

13 CATGACCACTAGGAGCATCTTTGGCGAAAGAAGATGGTAGGTTATGTGTGTTGAGCACTAGGGGAATAAATCTTTGGGCACCTAGTGGTCATG

13 CATGACCACTAGGAGCATCTTTGGCGAAAGTCGGTAGAACAGGGTGAGGTGCTGTCCCTAGGGGAATAAATCTTTGGGCACCTAGTGGTCATG

13 CATGACCACTAGGAGCATCTTTGGCGAAAGTCGGTAGAACAGGGTGGGGTGCTGACCCTAGGGGAATAAATCTTTGGGCACCTAGTGGTCATG

13 CATGACCACTAGGAGCATCTTTGGCGAAGCGTGTTGTAAGGTCCGCGAGTGTGGACCCTAGGGGAATAAATCTTTGGGCACCTAGTGGTCATG

13 CATGACCACTAGGAGCATCTTTGGCGAGCCTCGCTTGGGGGGTTGCTCCACCAGTTCCTAGGGGAATAAATCTTTGGGCACCTAGTGGTCATG

13 CATGACCACTAGGAGCATCTTTGGCGACTAAGTGGCCAACGCGTTAAATGGGGTAGACTAGGGGAATAAATCTTTGGGCACCTAGTGGTCATG

13 CATGACCACTAGGAGCATCTTTGGCGAACGCCAGGTGTTGCGGTGGTCTGTGAATCCCTAGGGGAATAAATCTTTGGGCACCTAGTGGTCATG

13 CATGACCACTAGGAGCATCTTTGGCGACATGCTTGATACGTTGGTTGCAGGGCCCGACTAGGGGAATAAATCTTTGGGCACCTAGTGGTCATG

13 CATGACCACTAGGAGCATCTTTGGCGACGGGGTGGGGCCTAAGTGCGGCGGTTGATTCTAGGGGAATAAATCTTTGGGCACCTAGTGGTCATG

13 CATGACCACTAGGAGCATCTTTGGCGACGATTGGTCGGAAGGTAAGTGTTCGGATGACTAGGGGAATAAATCTTTGGGCACCTAGTGGTCATG

13 CATGACCACTAGGAGCATCTTTGGCGACTCGTGGGCATATATGGGCCACGTAGTGGACTAGGGGAATAAATCTTTGGGCACCTAGTGGTCATG

13 CATGACCACTAGGAGCATCTTTGGCGAGCCGCTCTGGTTGGTCAGGCCGCTTGTGAACTAGGGGAATAAATCTTTGGGCACCTAGTGGTCATG

13 CATGACCACTAGGAGCATCTTTGGCGATTTGGGAGTCGCAGTACGCAGGCAGTGAGACTAGGGGAATAAATCTTTGGGCACCTAGTGGTCATG

13 CATGACCACTAGGAGCATCTTTGGCGAATGGCCGGCACGGCCTTCTAGACCTCGGTACTAGGGGAATAAATCTTTGGGCACCTAGTGGTCATG

13 CATGACCACTAGGAGCATCTTTGGCGAACGGCAGGTGTTGCGGTGGTGTGTGAATCCCTAGGGGAATAAATCTTTGGGCACCTAGTGGTCATG

13 CATGACCACTAGGAGCATCTTTGGCGAGGTCGCGTTAGCGATTGCGGCGGGCCTCGACTAGGGGAATAAATCTTTGGGCACCTAGTGGTCATG

13 CATGACCACTAGGAGCATCTTTGGCGAATGTCCGGCACGGCCTTCTAGTCCTCGGTACTAGGGGAATAAATCTTTGGGCACCTAGTGGTCATG

13 CATGACCACTAGGAGCATCTTTGGCGATAGCCGTAGCCGTAAGGTTGGTATAAGGCCCTAGGGGAATAAATCTTTGGGCACCTAGTGGTCATG

13 CATGACCACTAGGAGCATCTTTGGCGAACGGTAACGTAGTAACGTCGAAGTAGGCGCCTAGGGGAATAAATCTTTGGGCACCTAGTGGTCATG

13 CATGACCACTAGGAGCATCTTTGGCGAACGGCCGGTGTTGCGGTGGTCTGTGAATCCCTAGGGGAATAAATCTTTGGGCACCTAGTGGTCATG

13 CATGACCACTAGGAGCATCTTTGGCGAAAGTCTGTAGAACAGGGTGGGGTGCTGTCCCTAGGGGAATAAATCTTTGGGCACCTAGTGGTCATG

13 CATGACCACTAGGAGCATCTTTGGCGACGGGTTGCTGGTAGGTGGTACGACCTCGATCTAGGGGAATAAATCTTTGGGCACCTAGTGGTCATG

13 CATGACCACTAGGAGCATCTTTGGCGAAAGTCGGTAGAACAGGGTGGTGTGCTGTCCCTAGGGGAATAAATCTTTGGGCACCTAGTGGTCATG

13 CATGACCACTAGGAGCATCTTTGGCGAGCCTCGCTTGGGAGGTCGCTCCACCAGTTCCTAGGGGAATAAATCTTTGGGCACCTAGTGGTCATG

13 CATGACCACTAGGAGCATCTTTGGCGAGAAGACTCTGGATTCGGGGACCAATTGCTGCTAGGGGAATAAATCTTTGGGCACCTAGTGGTCATG

13 CATGACCACTAGGAGCATCTTTGGCGAACGGGAGGTGTTGCGGTGGTCTGTGAATCCCTAGGGGAATAAATCTTTGGGCACCTAGTGGTCATG

13 CATGACCACTAGGAGCATCTTTGGCGAATGGCCTGCACGGCCTTCTAGTCCTCGGTACTAGGGGAATAAATCTTTGGGCACCTAGTGGTCATG

13 CATGACCACTAGGAGCATCTTTGGCGAAAGACGGTAGAACAGGGTGGGGTGCTGTCCCTAGGGGAATAAATCTTTGGGCACCTAGTGGTCATG

13 CATGACCACTAGGAGCATCTTTGGCGAAAGTCGGTAGAACAGGGTGGGTTGCTGTCCCTAGGGGAATAAATCTTTGGGCACCTAGTGGTCATG

13 CATGACCACTAGGAGCATCTTTGGCGAAAGTCGGAAGAACAGGGTGGGGTGCTGTCCCTAGGGGAATAAATCTTTGGGCACCTAGTGGTCATG

13 CATGACCACTAGGAGCATCTTTGGCGATCTGCGTGGTCGTCGGATTAAGTTACGCCGCTAGGGGAATAAATCTTTGGGCACCTAGTGGTCATG

13 CATGACCACTAGGAGCATCTTTGGCGAGCCTCGCTTGGGAGGTTGCTCTACCAGTTCCTAGGGGAATAAATCTTTGGGCACCTAGTGGTCATG

13 CATGACCACTAGGAGCATCTTTGGCGAGCCTCGCTTGGGAGGTTGCCCCACCAGTTCCTAGGGGAATAAATCTTTGGGCACCTAGTGGTCATG

13 CATGACCACTAGGAGCATCTTTGGCGAGTTGGTGGCCGGGTGGGACTTCAGCGTAGACTAGGGGAATAAATCTTTGGGCACCTAGTGGTCATG

13 CATGACCACTAGGAGCATCTTTGGCGAAAGTCGGTAGAACAGGTTGGGGTGCTGTCCCTAGGGGAATAAATCTTTGGGCACCTAGTGGTCATG

13 CATGACCACTAGGAGCATCTTTGGCGATGGGGCCGTGGCATCGCTACACCGCGCGGCCTAGGGGAATAAATCTTTGGGCACCTAGTGGTCATG

13 CATGACCACTAGGAGCATCTTTGGCGAACCCGCGCGCGCTATAGGTTGCTCGAGCTCCTAGGGGAATAAATCTTTGGGCACCTAGTGGTCATG

13 CATGACCACTAGGAGCATCTTTGGCGAGCCTCGCTTGGGAGGTTGCTCCACCAGTCCCTAGGGGAATAAATCTTTGGGCACCTAGTGGTCATG

13 CATGACCACTAGGAGCATCTTTGGCGATTCTGCTGGTAGGTCATGTATGAGGTAGCTCTAGGGGAATAAATCTTTGGGCACCTAGTGGTCATG

12 CATGACCACTAGGAGCATCTTTGGCGAGCCTCGCTTGGGAGGTTGCTCCACCAGATCCTAGGGGAATAAATCTTTGGGCACCTAGTGGTCATG

12 CATGACCACTAGGAGCATCTTTGGCGATGGGTGCTGACGGCCGCCACTGCGGCTACACTAGGGGAATAAATCTTTGGGCACCTAGTGGTCATG

12 CATGACCACTAGGAGCATCTTTGGCGAATCGAGCTTGTCGGTGGTGTGGTTCCGACCCTAGGGGAATAAATCTTTGGGCACCTAGTGGTCATG

12 CATGACCACTAGGAGCATCTTTGGCGAGAAGACTCTGGATTCGGGGAACAGTTGCTGCTAGGGGAATAAATCTTTGGGCACCTAGTGGTCATG

12 CATGACCACTAGGAGCATCTTTGGCGAGAAGACTCTGGATTCGGGGACCAGTTGCGGCTAGGGGAATAAATCTTTGGGCACCTAGTGGTCATG

12 CATGACCACTAGGAGCATCTTTGGCGAAAGTAGGTAGAACAGGGTGGGGTGCTGTCCCTAGGGGAATAAATCTTTGGGCACCTAGTGGTCATG

12 CATGACCACTAGGAGCATCTTTGGCGAATGGCCGGCACTGCCTTCTAGTCCTCGGTACTAGGGGAATAAATCTTTGGGCACCTAGTGGTCATG

12 CATGACCACTAGGAGCATCTTTGGCGAGAAGACTCTGGATTCGGGGACCAGTTGTTGCTAGGGGAATAAATCTTTGGGCACCTAGTGGTCATG

12 CATGACCACTAGGAGCATCTTTGGCGAATGGCCGGCACAGCCTTCTAGTCCTCGGTACTAGGGGAATAAATCTTTGGGCACCTAGTGGTCATG

12 CATGACCACTAGGAGCATCTTTGGCGAGCCTCGCTTGGGAGGTTGATCCACCAGTTCCTAGGGGAATAAATCTTTGGGCACCTAGTGGTCATG

12 CATGACCACTAGGAGCATCTTTGGCGACGTGGGCGTTTCGGCCGTTCCGGCGTGTGCCTAGGGGAATAAATCTTTGGGCACCTAGTGGTCATG

12 CATGACCACTAGGAGCATCTTTGGCGAGAAGACTCTGGATTCGGGGACCAGTTGCAGCTAGGGGAATAAATCTTTGGGCACCTAGTGGTCATG

12 CATGACCACTAGGAGCATCTTTGGCGAATGGCCGGCACGGCCTTCTAGTCCTCTGTACTAGGGGAATAAATCTTTGGGCACCTAGTGGTCATG

12 CATGACCACTAGGAGCATCTTTGGCGATTAGCGTCTTGCGAGCGGGTGGGTTCGCTCCTAGGGGAATAAATCTTTGGGCACCTAGTGGTCATG

12 CATGACCACTAGGAGCATCTTTGGCGAGAAGACTCTGGATTCGGGGACCCGTTGCTGCTAGGGGAATAAATCTTTGGGCACCTAGTGGTCATG

12 CATGACCACTAGGAGCATCTTTGGCGAATGGCCGGCACGGCCTTCTAGTCCTCGGTGCTAGGGGAATAAATCTTTGGGCACCTAGTGGTCATG

12 CATGACCACTAGGAGCATCTTTGGCGACGAAGAGCGAACGTTTATGCCGCGAGGAAGCTAGGGGAATAAATCTTTGGGCACCTAGTGGTCATG

12 CATGACCACTAGGAGCATCTTTGGCGACAGTGGGCGGACCGGGACCCAAGGGGCGGACTAGGGGAATAAATCTTTGGGCACCTAGTGGTCATG

12 CATGACCACTAGGAGCATCTTTGGCGAGGCGTGTTGCAAGGTCCGCGAGTGTGGACCCTAGGGGAATAAATCTTTGGGCACCTAGTGGTCATG

12 CATGACCACTAGGAGCATCTTTGGCGACCCATACAACTTGCAAAAGTTTTGGGTGGACTAGGGGAATAAATCTTTGGGCACCTAGTGGTCATG

12 CATGACCACTAGGAGCATCTTTGGCGACGATGGTTGGGAGGGAAGTATTTCGGGCGACTAGGGGAATAAATCTTTGGGCACCTAGTGGTCATG

12 CATGACCACTAGGAGCATCTTTGGCGAGAAGACTCTGGATTCGGGGACCATTTGCTGCTAGGGGAATAAATCTTTGGGCACCTAGTGGTCATG

12 CATGACCACTAGGAGCATCTTTGGCGATCCTTGTCAGATGGTGAAGCAGACGTTTGGCTAGGGGAATAAATCTTTGGGCACCTAGTGGTCATG

12 CATGACCACTAGGAGCATCTTTGGCGAGATCGGGACAATCGGTGGCATTGGTGTCTCCTAGGGGAATAAATCTTTGGGCACCTAGTGGTCATG

12 CATGACCACTAGGAGCATCTTTGGCGAGGACCAAGGTGTGCATCCCGTACAGGCTGCCTAGGGGAATAAATCTTTGGGCACCTAGTGGTCATG

12 CATGACCACTAGGAGCATCTTTGGCGACAGCAGCGTAGGCTGCCTTGGGTGGTGGCCCTAGGGGAATAAATCTTTGGGCACCTAGTGGTCATG

12 CATGACCACTAGGAGCATCTTTGGCGATCAAGCCTGGGCGCGCTATATTGGGTAGATCTAGGGGAATAAATCTTTGGGCACCTAGTGGTCATG

12 CATGACCACTAGGAGCATCTTTGGCGAACCTACAGGAGCACCGCGAATCGGATGAGACTAGGGGAATAAATCTTTGGGCACCTAGTGGTCATG

12 CATGACCACTAGGAGCATCTTTGGCGATGGGTGCTGACGGCTGTTTCATCAGCTACACTAGGGGAATAAATCTTTGGGCACCTAGTGGTCATG

12 CATGACCACTAGGAGCATCTTTGGCGAGGGATGGGAGCCGATGGGGGTTCGGGGTTTCTAGGGGAATAAATCTTTGGGCACCTAGTGGTCATG

12 CATGACCACTAGGAGCATCTTTGGCGATTTGGGAGTCGCAGTAAGCAGGCAGTGAGACTAGGGGAATAAATCTTTGGGCACCTAGTGGTCATG

11 CATGACCACTAGGAGCATCTTTGGCGAGCCTCGCTAGGGAGGTTGCTCCACCAGTTCCTAGGGGAATAAATCTTTGGGCACCTAGTGGTCATG

11 CATGACCACTAGGAGCATCTTTGGCGACGAAGGTCTTTAGTGGGCCCGAGGGCCGACCTAGGGGAATAAATCTTTGGGCACCTAGTGGTCATG

11 CATGACCACTAGGAGCATCTTTGGCGACCGAGGCAGCGGGGGAAATATGCGCAGGTCCTAGGGGAATAAATCTTTGGGCACCTAGTGGTCATG

11 CATGACCACTAGGAGCATCTTTGGCGAATGGCCGGCACGGCCTTCTAGTCCTCGGTCCTAGGGGAATAAATCTTTGGGCACCTAGTGGTCATG

11 CATGACCACTAGGAGCATCTTTGGCGATTTGGGAGTCGCACTATGCAGGCAGTGAGACTAGGGGAATAAATCTTTGGGCACCTAGTGGTCATG

11 CATGACCACTAGGAGCATCTTTGGCGATAGGCGTGTACGGTCGAGCGAAGGCGATAGCTAGGGGAATAAATCTTTGGGCACCTAGTGGTCATG

11 CATGACCACTAGGAGCATCTTTGGCGAATGTCGGTAGAACAGGGTGGGGTGCTGTCCCTAGGGGAATAAATCTTTGGGCACCTAGTGGTCATG

11 CATGACCACTAGGAGCATCTTTGGCGACGTTAAGTTGGGAAATGTGATATGTTCAGACTAGGGGAATAAATCTTTGGGCACCTAGTGGTCATG

11 CATGACCACTAGGAGCATCTTTGGCGATGGGTGCTGACGGGGCTAGCGAGCCCTACACTAGGGGAATAAATCTTTGGGCACCTAGTGGTCATG

11 CATGACCACTAGGAGCATCTTTGGCGACGACGAAGGTGCAATGACTGAGGATTCGCCCTAGGGGAATAAATCTTTGGGCACCTAGTGGTCATG

11 CATGACCACTAGGAGCATCTTTGGCGACGTACGGAACCAACAGTCGATAAAGTCGCTCTAGGGGAATAAATCTTTGGGCACCTAGTGGTCATG

11 CATGACCACTAGGAGCATCTTTGGCGAGAAGACTCTGGATTCGGGGACCAGGTGCTGCTAGGGGAATAAATCTTTGGGCACCTAGTGGTCATG

11 CATGACCACTAGGAGCATCTTTGGCGAGGCGAGTCCAGCGGCCTTCTTGGCCTGTGACTAGGGGAATAAATCTTTGGGCACCTAGTGGTCATG

11 CATGACCACTAGGAGCATCTTTGGCGATGGGGCCGTGCCATCTGCACACTCCGCGGGCTAGGGGAATAAATCTTTGGGCACCTAGTGGTCATG

11 CATGACCACTAGGAGCATCTTTGGCGAACGGCAGGTGTTGCGGTGGTCTGTCAATCCCTAGGGGAATAAATCTTTGGGCACCTAGTGGTCATG

11 CATGACCACTAGGAGCATCTTTGGCGAAGCGTGTTGCAAGGTCTGCGAGTGTGGACCCTAGGGGAATAAATCTTTGGGCACCTAGTGGTCATG

11 CATGACCACTAGGAGCATCTTTGGCGATACAGGTGATCGGTGCTTGGGTGCCTGGCCCTAGGGGAATAAATCTTTGGGCACCTAGTGGTCATG

11 CATGACCACTAGGAGCATCTTTGGCGAGCCTCGCTTGGGAGGTTGCTCCACCGGTTCCTAGGGGAATAAATCTTTGGGCACCTAGTGGTCATG

11 CATGACCACTAGGAGCATCTTTGGCGAGTGGTGGGCTTGGGGAGAAGGACAACGAGACTAGGGGAATAAATCTTTGGGCACCTAGTGGTCATG

11 CATGACCACTAGGAGCATCTTTGGCGAGATCGGGAGCATCGGTGGCATTGGTGTCTCCTAGGGGAATAAATCTTTGGGCACCTAGTGGTCATG

11 CATGACCACTAGGAGCATCTTTGGCGACATCAGCTCTGCGGTCGGGGACATAAGGGACTAGGGGAATAAATCTTTGGGCACCTAGTGGTCATG

11 CATGACCACTAGGAGCATCTTTGGCGACTCCCAGGCAGCGTGCTGCTTGGGTGTGGACTAGGGGAATAAATCTTTGGGCACCTAGTGGTCATG

11 CATGACCACTAGGAGCATCTTTGGCGAAAGTCGGTAGAACAAGGTGGGGTGCTGTCCCTAGGGGAATAAATCTTTGGGCACCTAGTGGTCATG

11 CATGACCACTAGGAGCATCTTTGGCGAAGGGCAGGTGTTGCGGTGGTCTGTGAATCCCTAGGGGAATAAATCTTTGGGCACCTAGTGGTCATG

11 CATGACCACTAGGAGCATCTTTGGCGATCCTCGTCAGATGGAGAAGCAGACGTTTGGCTAGGGGAATAAATCTTTGGGCACCTAGTGGTCATG

11 CATGACCACTAGGAGCATCTTTGGCGAAAGGCCGGCACGGCCTTCTAGTCCTCGGTACTAGGGGAATAAATCTTTGGGCACCTAGTGGTCATG

11 CATGACCACTAGGAGCATCTTTGGCGAGTCGGGGCATGCCGGGTCGTTGATTGCCTGCTAGGGGAATAAATCTTTGGGCACCTAGTGGTCATG

11 CATGACCACTAGGAGCATCTTTGGCGAGTAGACTCTGGATTCGGGGACCAGTTGCTGCTAGGGGAATAAATCTTTGGGCACCTAGTGGTCATG

11 CATGACCACTAGGAGCATCTTTGGCGAATGGCCGGCACGGCCTTCTAGTCCACGGTACTAGGGGAATAAATCTTTGGGCACCTAGTGGTCATG

11 CATGACCACTAGGAGCATCTTTGGCGACCCCAGTACGCGTTAGCTATACGGGGTGGACTAGGGGAATAAATCTTTGGGCACCTAGTGGTCATG

11 CATGACCACTAGGAGCATCTTTGGCGACGAGGAGCGGACGTTTATTGCGCTAGTCGACTAGGGGAATAAATCTTTGGGCACCTAGTGGTCATG

11 CATGACCACTAGGAGCATCTTTGGCGAGCCTCGCTTGGGAGGCTGCTCCACCAGTTCCTAGGGGAATAAATCTTTGGGCACCTAGTGGTCATG

11 CATGACCACTAGGAGCATCTTTGGCGATTTCAGACATTGAAAGCAGCAGACGTTTGGCTAGGGGAATAAATCTTTGGGCACCTAGTGGTCATG

11 CATGACCACTAGGAGCATCTTTGGCGACCCGAATCGTAAACACGACAGAGCGGTGGACTAGGGGAATAAATCTTTGGGCACCTAGTGGTCATG

11 CATGACCACTAGGAGCATCTTTGGCGATCGAGTGGGCTGGGAGGAAGTATTTCGGAGCTAGGGGAATAAATCTTTGGGCACCTAGTGGTCATG

11 CATGACCACTAGGAGCATCTTTGGCGATGTTGGCTGGTAGGGAGTACTGATATCGACCTAGGGGAATAAATCTTTGGGCACCTAGTGGTCATG

11 CATGACCACTAGGAGCATCTTTGGCGACGGTGGGCTGGAAGGGCGTATTCTCGACGCCTAGGGGAATAAATCTTTGGGCACCTAGTGGTCATG

11 CATGACCACTAGGAGCATCTTTGGCGAGCCTCGCTTGTGAGGTTGCTCCACCAGTTCCTAGGGGAATAAATCTTTGGGCACCTAGTGGTCATG

11 CATGACCACTAGGAGCATCTTTGGCGAATGGCCGGCACGGCCATCTAGTCCTCGGTACTAGGGGAATAAATCTTTGGGCACCTAGTGGTCATG

11 CATGACCACTAGGAGCATCTTTGGCGAAAGTCGGTAGAACAGGGGGGGGTGCTGTCCCTAGGGGAATAAATCTTTGGGCACCTAGTGGTCATG

11 CATGACCACTAGGAGCATCTTTGGCGAACGGCAGGTGTTGCCGTGGTCTGTGAATCCCTAGGGGAATAAATCTTTGGGCACCTAGTGGTCATG

11 CATGACCACTAGGAGCATCTTTGGCGATCTGTCGGTTGGTGCGTCAGGTACGGGTAGCTAGGGGAATAAATCTTTGGGCACCTAGTGGTCATG

10 CATGACCACTAGGAGCATCTTTGGCGAGGGGTAGGGGGCCGCTCGCGCCTACGTGCGCTAGGGGAATAAATCTTTGGGCACCTAGTGGTCATG

10 CATGACCACTAGGAGCATCTTTGGCGAGGCGAGTCCAGCGGCCCTCTTGGCCTGCGACTAGGGGAATAAATCTTTGGGCACCTAGTGGTCATG

10 CATGACCACTAGGAGCATCTTTGGCGATCAAGTGACTTAGTGTGCCTGGAGCGAAGGCTAGGGGAATAAATCTTTGGGCACCTAGTGGTCATG

10 CATGACCACTAGGAGCATCTTTGGCGATATCGGCGTAGCCAACCAATGCTAATGGTTCTAGGGGAATAAATCTTTGGGCACCTAGTGGTCATG

10 CATGACCACTAGGAGCATCTTTGGCGAATGGCCGGCACGGCCTTCTAGTCTTTGGTACTAGGGGAATAAATCTTTGGGCACCTAGTGGTCATG

10 CATGACCACTAGGAGCATCTTTGGCGACGAAGGAAACTTTGTTGACTGATAGTGCGACTAGGGGAATAAATCTTTGGGCACCTAGTGGTCATG

10 CATGACCACTAGGAGCATCTTTGGCGACTAAGTGGTCAACGCGTTAAGTGGGGTAGACTAGGGGAATAAATCTTTGGGCACCTAGTGGTCATG

10 CATGACCACTAGGAGCATCTTTGGCGAAAGTGCGATAGCAGGTTTACGAAGTGGAGCCTAGGGGAATAAATCTTTGGGCACCTAGTGGTCATG

10 CATGACCACTAGGAGCATCTTTGGCGATGGGGCCGTGGCATCGCTACACTGCGCGGCCTAGGGGAATAAATCTTTGGGCACCTAGTGGTCATG

10 CATGACCACTAGGAGCATCTTTGGCGATTTGCTGGGAGGTCGCTCGGGTACGGGTAGCTAGGGGAATAAATCTTTGGGCACCTAGTGGTCATG

10 CATGACCACTAGGAGCATCTTTGGCGATTAGTCGGGCCATGGGATACTTGCGGCCCGCTAGGGGAATAAATCTTTGGGCACCTAGTGGTCATG

10 CATGACCACTAGGAGCATCTTTGGCGATAGCGTCTTGCGAGCGGGTGGGTTCGCTCCCTAGGGGAATAAATCTTTGGGCACCTAGTGGTCATG

10 CATGACCACTAGGAGCATCTTTGGCGAGTAGGGGGCACCGGTAATCGACAGACGAGCCTAGGGGAATAAATCTTTGGGCACCTAGTGGTCATG

10 CATGACCACTAGGAGCATCTTTGGCGAGGAGATGGTTTAGACGCCAAGGGCGAGACACTAGGGGAATAAATCTTTGGGCACCTAGTGGTCATG

10 CATGACCACTAGGAGCATCTTTGGCGAACGGCAGGTGTTGCGGTCGTCTGTGAATCCCTAGGGGAATAAATCTTTGGGCACCTAGTGGTCATG

10 CATGACCACTAGGAGCATCTTTGGCGAAAGTCGGTAGAACAGGGTGGAGTGCTGTCCCTAGGGGAATAAATCTTTGGGCACCTAGTGGTCATG

10 CATGACCACTAGGAGCATCTTTGGCGAGAGCCGAGGTTGGTTTCGTGGGGATCAGCCCTAGGGGAATAAATCTTTGGGCACCTAGTGGTCATG

10 CATGACCACTAGGAGCATCTTTGGCGAGCCTCGCTGGGGAGGTTGCTCCACCAGTTCCTAGGGGAATAAATCTTTGGGCACCTAGTGGTCATG

10 CATGACCACTAGGAGCATCTTTGGCGATCCTCGTCAGATGGTGAAGCAGACGGTTGGCTAGGGGAATAAATCTTTGGGCACCTAGTGGTCATG

10 CATGACCACTAGGAGCATCTTTGGCGAGCGAAGGTAACGGTGTGCGTAAAGGAGCTCCTAGGGGAATAAATCTTTGGGCACCTAGTGGTCATG

10 CATGACCACTAGGAGCATCTTTGGCGAGCCTTGCTTGGGAGGTTGCTTCACCAGTTCCTAGGGGAATAAATCTTTGGGCACCTAGTGGTCATG

10 CATGACCACTAGGAGCATCTTTGGCGACCTTGGCGCGGGGATCCTGGTTGCGGCGACCTAGGGGAATAAATCTTTGGGCACCTAGTGGTCATG

10 CATGACCACTAGGAGCATCTTTGGCGAAAGTCGGTAGAACAGGGTGGGGTGCAGTCCCTAGGGGAATAAATCTTTGGGCACCTAGTGGTCATG

10 CATGACCACTAGGAGCATCTTTGGCGACCCATCCGAACAGCAGGGAGGTGTGGTGGACTAGGGGAATAAATCTTTGGGCACCTAGTGGTCATG

10 CATGACCACTAGGAGCATCTTTGGCGAGGCGAGTCCAGCGGCCTTCCTGGCCTGCGACTAGGGGAATAAATCTTTGGGCACCTAGTGGTCATG

10 CATGACCACTAGGAGCATCTTTGGCGACGGGGCCGTGCTATCTGCACACTCCGCGGGCTAGGGGAATAAATCTTTGGGCACCTAGTGGTCATG

10 CATGACCACTAGGAGCATCTTTGGCGAGGGGATATGGTAGGAGGATGTGCCTCTTGACTAGGGGAATAAATCTTTGGGCACCTAGTGGTCATG

10 CATGACCACTAGGAGCATCTTTGGCGAGCTCGGGAGAATCGGTGGCATTGGTGTCTCCTAGGGGAATAAATCTTTGGGCACCTAGTGGTCATG

10 CATGACCACTAGGAGCATCTTTGGCGACGTGGGCGTGTCGGCCGTGTGGCGTAGTAGCTAGGGGAATAAATCTTTGGGCACCTAGTGGTCATG

10 CATGACCACTAGGAGCATCTTTGGCGATCGGGCCGCCAGGGCGTACGAAGTGTGGTTCTAGGGGAATAAATCTTTGGGCACCTAGTGGTCATG

10 CATGACCACTAGGAGCATCTTTGGCGAAACGCACATAAACTGCGATCGTAGTGGAGACTAGGGGAATAAATCTTTGGGCACCTAGTGGTCATG

10 CATGACCACTAGGAGCATCTTTGGCGATGGGTGCTGACTGCCGCCGCTGCGGCTACACTAGGGGAATAAATCTTTGGGCACCTAGTGGTCATG

10 CATGACCACTAGGAGCATCTTTGGCGACCCGTCACGACAATGCTCGTACGTGGTGGACTAGGGGAATAAATCTTTGGGCACCTAGTGGTCATG

10 CATGACCACTAGGAGCATCTTTGGCGAAGTGAAGAGTGCGTGTAGTGGTCTGTTCGCCTAGGGGAATAAATCTTTGGGCACCTAGTGGTCATG

10 CATGACCACTAGGAGCATCTTTGGCGATCAGCAATGGGATTGACCTCCTTGTAGGTCCTAGGGGAATAAATCTTTGGGCACCTAGTGGTCATG

10 CATGACCACTAGGAGCATCTTTGGCGAGAAGACTCTGGCTTCGGGGACCAGTTGCTGCTAGGGGAATAAATCTTTGGGCACCTAGTGGTCATG

10 CATGACCACTAGGAGCATCTTTGGCGACGGCGTGGGGACTCGGTCTCGGCGGTTTCCCTAGGGGAATAAATCTTTGGGCACCTAGTGGTCATG

10 CATGACCACTAGGAGCATCTTTGGCGAATGAATGCGACGGTCATGGGTGTGAGACCGCTAGGGGAATAAATCTTTGGGCACCTAGTGGTCATG

10 CATGACCACTAGGAGCATCTTTGGCGAAGGTGGCCGAGATCCGATCGGTAGTGTTGCCTAGGGGAATAAATCTTTGGGCACCTAGTGGTCATG

10 CATGACCACTAGGAGCATCTTTGGCGAAGTGGTATGCATTGTGGTCTTGGTCCAACCCTAGGGGAATAAATCTTTGGGCACCTAGTGGTCATG

10 CATGACCACTAGGAGCATCTTTGGCGATGGCCGTCTGGAAGGTTTGTGAGGCGGTAGCTAGGGGAATAAATCTTTGGGCACCTAGTGGTCATG

10 CATGACCACTAGGAGCATCTTTGGCGATACGACGAGCCGGAGATCGCAGACGTTTGGCTAGGGGAATAAATCTTTGGGCACCTAGTGGTCATG

10 CATGACCACTAGGAGCATCTTTGGCGATCAGCATAACATATGCGCCTGGAGCGTAGGCTAGGGGAATAAATCTTTGGGCACCTAGTGGTCATG

10 CATGACCACTAGGAGCATCTTTGGCGAATGGCTGGCACGGCCTTCTAGTCCTTGGTACTAGGGGAATAAATCTTTGGGCACCTAGTGGTCATG

10 CATGACCACTAGGAGCATCTTTGGCGAGCCTCGCGTGGGAGGTTGCTCCACCAGTTCCTAGGGGAATAAATCTTTGGGCACCTAGTGGTCATG

10 CATGACCACTAGGAGCATCTTTGGCGAATGGCCGGCACGGCCTTCAAGTCCTCGGTACTAGGGGAATAAATCTTTGGGCACCTAGTGGTCATG

10 CATGACCACTAGGAGCATCTTTGGCGAATGGGGGGCGACGGCCGGTAGTGGGTGAGACTAGGGGAATAAATCTTTGGGCACCTAGTGGTCATG

10 CATGACCACTAGGAGCATCTTTGGCGAACTGCTGTTCCGGCGCTCCAGAAGATAAAGCTAGGGGAATAAATCTTTGGGCACCTAGTGGTCATG

9 CATGACCACTAGGAGCATCTTTGGCGAAAGTCGGTAGAATAGGGTGGGGTGCTGTCCCTAGGGGAATAAATCTTTGGGCACCTAGTGGTCATG

9 CATGACCACTAGGAGCATCTTTGGCGATGGTGGGTGGAACCATGCTAGGGTCGTCGACTAGGGGAATAAATCTTTGGGCACCTAGTGGTCATG

9 CATGACCACTAGGAGCATCTTTGGCGACCCGACTACGGCAGTATAGCGGCGGGTGGACTAGGGGAATAAATCTTTGGGCACCTAGTGGTCATG

9 CATGACCACTAGGAGCATCTTTGGCGACTTCGAGGCTCTGCCGACAACCGAAGTGGACTAGGGGAATAAATCTTTGGGCACCTAGTGGTCATG

9 CATGACCACTAGGAGCATCTTTGGCGATCAGGCCGCCAGGGCGTACGAAGTGTGGTCCTAGGGGAATAAATCTTTGGGCACCTAGTGGTCATG

9 CATGACCACTAGGAGCATCTTTGGCGACCTTTGGTACGGGGTGGGATCCGCTGTGCCCTAGGGGAATAAATCTTTGGGCACCTAGTGGTCATG

9 CATGACCACTAGGAGCATCTTTGGCGAAAGTCGGTAGACCAGGGTGGGGTGCTGTCCCTAGGGGAATAAATCTTTGGGCACCTAGTGGTCATG

9 CATGACCACTAGGAGCATCTTTGGCGAAAGTCGGTCGAACAGGGTGGGGTGCTGTCCCTAGGGGAATAAATCTTTGGGCACCTAGTGGTCATG

9 CATGACCACTAGGAGCATCTTTGGCGAAGAGAGGAAAGGTCGGGAACAGTGCGTTCACTAGGGGAATAAATCTTTGGGCACCTAGTGGTCATG

9 CATGACCACTAGGAGCATCTTTGGCGACACAAGATGTGACCTAGATAAACGTCGCCGCTAGGGGAATAAATCTTTGGGCACCTAGTGGTCATG

9 CATGACCACTAGGAGCATCTTTGGCGACGAGAGTGGGTCATGGTGGAATAAAGTCGACTAGGGGAATAAATCTTTGGGCACCTAGTGGTCATG

9 CATGACCACTAGGAGCATCTTTGGCGAGAGGGGATGCCGATGCGCTGGACGACCGAGCTAGGGGAATAAATCTTTGGGCACCTAGTGGTCATG

9 CATGACCACTAGGAGCATCTTTGGCGATAGCCCGGTGTGGGTGCGCAAGCTATATGCCTAGGGGAATAAATCTTTGGGCACCTAGTGGTCATG

9 CATGACCACTAGGAGCATCTTTGGCGACGGTGTTGACCTTATAGGTGTTTGGAGGCCCTAGGGGAATAAATCTTTGGGCACCTAGTGGTCATG

9 CATGACCACTAGGAGCATCTTTGGCGAGGCGAGTCCAGCGGCCTCCTTGGCCTGCGACTAGGGGAATAAATCTTTGGGCACCTAGTGGTCATG

9 CATGACCACTAGGAGCATCTTTGGCGAATGGCCGGCACGGCCTTCTAATCCTCGGTACTAGGGGAATAAATCTTTGGGCACCTAGTGGTCATG

9 CATGACCACTAGGAGCATCTTTGGCGAGAACATCGAATGTTCGGGGACCAGTTGCAGCTAGGGGAATAAATCTTTGGGCACCTAGTGGTCATG

9 CATGACCACTAGGAGCATCTTTGGCGAGCATCGCTTGGGAGGTTGCTCCACCAGTTCCTAGGGGAATAAATCTTTGGGCACCTAGTGGTCATG

9 CATGACCACTAGGAGCATCTTTGGCGAATGGCCGGCACGGACTTCTAGTCCTCGGTACTAGGGGAATAAATCTTTGGGCACCTAGTGGTCATG

9 CATGACCACTAGGAGCATCTTTGGCGACTACTGTAAGCGAAAGAAGCAACAGTGGAGCTAGGGGAATAAATCTTTGGGCACCTAGTGGTCATG

9 CATGACCACTAGGAGCATCTTTGGCGAACGTGCAAAATATGCCGCCTGGAGCGTAGGCTAGGGGAATAAATCTTTGGGCACCTAGTGGTCATG

9 CATGACCACTAGGAGCATCTTTGGCGAATAGCCGGCACGGCCTTCTAGTCCTCGGTACTAGGGGAATAAATCTTTGGGCACCTAGTGGTCATG

9 CATGACCACTAGGAGCATCTTTGGCGAGCCTCGCTTGGGAGGTTGCTCAACCAGTTCCTAGGGGAATAAATCTTTGGGCACCTAGTGGTCATG

9 CATGACCACTAGGAGCATCTTTGGCGACCCGGAATAGCGAAGTCAAGTCTGGGTGGACTAGGGGAATAAATCTTTGGGCACCTAGTGGTCATG

9 CATGACCACTAGGAGCATCTTTGGCGAGGGAGGGCGCCGGCAGCGGTGTGAATGCGTCTAGGGGAATAAATCTTTGGGCACCTAGTGGTCATG

9 CATGACCACTAGGAGCATCTTTGGCGAGCCCCCCGAGTGCGATCACGATTGGGCCGACTAGGGGAATAAATCTTTGGGCACCTAGTGGTCATG

9 CATGACCACTAGGAGCATCTTTGGCGAAGGGATCTGTCGGGCCGGAGTGCTCCAACGCTAGGGGAATAAATCTTTGGGCACCTAGTGGTCATG

9 CATGACCACTAGGAGCATCTTTGGCGATCCTCGACAGATGGTGAAGCAGACGTTTGGCTAGGGGAATAAATCTTTGGGCACCTAGTGGTCATG

9 CATGACCACTAGGAGCATCTTTGGCGAACTTGATCGAGACGATCGAGCGAAGTTGGACTAGGGGAATAAATCTTTGGGCACCTAGTGGTCATG

9 CATGACCACTAGGAGCATCTTTGGCGAAGCGTGTTGAGGCTCCGACAGATTCTAGGACTAGGGGAATAAATCTTTGGGCACCTAGTGGTCATG

9 CATGACCACTAGGAGCATCTTTGGCGAGTAGTGGGTCTGACAAGGGTAAAAACGAGACTAGGGGAATAAATCTTTGGGCACCTAGTGGTCATG

9 CATGACCACTAGGAGCATCTTTGGCGAGGTCGAGAAGAACTGAGGATACAGGTGCCTCTAGGGGAATAAATCTTTGGGCACCTAGTGGTCATG

9 CATGACCACTAGGAGCATCTTTGGCGAGTAAGGTGTGGGCGGTGAGAAACAATACGACTAGGGGAATAAATCTTTGGGCACCTAGTGGTCATG

9 CATGACCACTAGGAGCATCTTTGGCGACGCGTTTACGCACCGATGAGGCGTTTGGTCCTAGGGGAATAAATCTTTGGGCACCTAGTGGTCATG

9 CATGACCACTAGGAGCATCTTTGGCGACGACTTTGGAGTGGGTGCGCCGCTGGCATACTAGGGGAATAAATCTTTGGGCACCTAGTGGTCATG

9 CATGACCACTAGGAGCATCTTTGGCGAACGGCAGGTGTTGCGGTGGTCTGTGTACCCCTAGGGGAATAAATCTTTGGGCACCTAGTGGTCATG

9 CATGACCACTAGGAGCATCTTTGGCGACCCGACATCACGTAAGGATGTAGAGGTGGACTAGGGGAATAAATCTTTGGGCACCTAGTGGTCATG

9 CATGACCACTAGGAGCATCTTTGGCGACGATGGTGGGCTAGAGACCTCCGAAGTGGACTAGGGGAATAAATCTTTGGGCACCTAGTGGTCATG

9 CATGACCACTAGGAGCATCTTTGGCGAGGGGACCCGCTTCTAGGATGGGCTTTGTCACTAGGGGAATAAATCTTTGGGCACCTAGTGGTCATG

9 CATGACCACTAGGAGCATCTTTGGCGACCGAGCATTTGGCGCGCTAAGTGGGCGTAGCTAGGGGAATAAATCTTTGGGCACCTAGTGGTCATG

9 CATGACCACTAGGAGCATCTTTGGCGAACCTGGTGGACTATGTGAGCAGACGTTTGGCTAGGGGAATAAATCTTTGGGCACCTAGTGGTCATG

9 CATGACCACTAGGAGCATCTTTGGCGAAGATCGCTTGTCGACTCGGGTCCGAGGAAGCTAGGGGAATAAATCTTTGGGCACCTAGTGGTCATG

9 CATGACCACTAGGAGCATCTTTGGCGACTCGTGGTTCTTACCCCCGTAGAGGGTTGTCTAGGGGAATAAATCTTTGGGCACCTAGTGGTCATG

9 CATGACCACTAGGAGCATCTTTGGCGAATTGCCGGCACGGCCTTCTAGTCCTCGGTACTAGGGGAATAAATCTTTGGGCACCTAGTGGTCATG

9 CATGACCACTAGGAGCATCTTTGGCGATCAGCATAGCATATGTGCCTGGAGCGTAGGCTAGGGGAATAAATCTTTGGGCACCTAGTGGTCATG

8 CATGACCACTAGGAGCATCTTTGGCGAGGGGACGTAGGGCACGCAGCCGGGGGCGAGCTAGGGGAATAAATCTTTGGGCACCTAGTGGTCATG

8 CATGACCACTAGGAGCATCTTTGGCGAGCCTCGCTTGGGAGTTTGCTCCACCAGTTCCTAGGGGAATAAATCTTTGGGCACCTAGTGGTCATG

8 CATGACCACTAGGAGCATCTTTGGCGAACCGTCCAGCGCAGGACGACGAAGTGGAGCCTAGGGGAATAAATCTTTGGGCACCTAGTGGTCATG

8 CATGACCACTAGGAGCATCTTTGGCGAAAGTCGGTAGAACAGGGTGGGGTGCTGTCTCTAGGGGAATAAATCTTTGGGCACCTAGTGGTCATG

8 CATGACCACTAGGAGCATCTTTGGCGACCCTGCATACATTGGGGAGCAGACGTTTGGCTAGGGGAATAAATCTTTGGGCACCTAGTGGTCATG

8 CATGACCACTAGGAGCATCTTTGGCGAGGAGAAGCCCGCCAAGCGGAAATGGGCGAGCTAGGGGAATAAATCTTTGGGCACCTAGTGGTCATG

8 CATGACCACTAGGAGCATCTTTGGCGACCTGGCTGGAAGGGAGTATTGGATAAGCCTCTAGGGGAATAAATCTTTGGGCACCTAGTGGTCATG

8 CATGACCACTAGGAGCATCTTTGGCGAGCCACGCTTGGGAGGTTGCTCCACCAGTTCCTAGGGGAATAAATCTTTGGGCACCTAGTGGTCATG

8 CATGACCACTAGGAGCATCTTTGGCGAACGGCAGGTGTTGCGCTGGTCTGTGAATCCCTAGGGGAATAAATCTTTGGGCACCTAGTGGTCATG

8 CATGACCACTAGGAGCATCTTTGGCGACCCGGAAGGATGCAATCTTAATCGGGTGGACTAGGGGAATAAATCTTTGGGCACCTAGTGGTCATG

8 CATGACCACTAGGAGCATCTTTGGCGAATGGCCGGCACGGCCTTATAGTCCTCGGTACTAGGGGAATAAATCTTTGGGCACCTAGTGGTCATG

8 CATGACCACTAGGAGCATCTTTGGCGATTGGTGGTACACGGTGGGTAGGCAGGTAGACTAGGGGAATAAATCTTTGGGCACCTAGTGGTCATG

8 CATGACCACTAGGAGCATCTTTGGCGACGGTGTGGGGAACCTGTTTCGGCGGTGCTACTAGGGGAATAAATCTTTGGGCACCTAGTGGTCATG

8 CATGACCACTAGGAGCATCTTTGGCGATCGGTTGGCATTGGTGTCTCCCTTGTGACGCTAGGGGAATAAATCTTTGGGCACCTAGTGGTCATG

8 CATGACCACTAGGAGCATCTTTGGCGAGCCTCGCTTGGGAGGTTGCTCCACCAGTTACTAGGGGAATAAATCTTTGGGCACCTAGTGGTCATG

8 CATGACCACTAGGAGCATCTTTGGCGAGAAGACTCTGGATGCGGGGACCAGTTGCTGCTAGGGGAATAAATCTTTGGGCACCTAGTGGTCATG

8 CATGACCACTAGGAGCATCTTTGGCGACTGGCCGGCACGGCCTTCTAGTCCTCGGTACTAGGGGAATAAATCTTTGGGCACCTAGTGGTCATG

8 CATGACCACTAGGAGCATCTTTGGCGATTTGGGAGTCGCGGTATGCAGGCAGTGAGACTAGGGGAATAAATCTTTGGGCACCTAGTGGTCATG

8 CATGACCACTAGGAGCATCTTTGGCGAACGGCACGTGTTGCGGTGGTCTGTGAATCCCTAGGGGAATAAATCTTTGGGCACCTAGTGGTCATG

8 CATGACCACTAGGAGCATCTTTGGCGATGGGTGCTGACGACCGTCCATACGGTTACTCTAGGGGAATAAATCTTTGGGCACCTAGTGGTCATG

8 CATGACCACTAGGAGCATCTTTGGCGATCCACGTCAGATGGTGAAGCAGACGTTTGGCTAGGGGAATAAATCTTTGGGCACCTAGTGGTCATG

8 CATGACCACTAGGAGCATCTTTGGCGATGGGTGCTGACGGGGCTGGTGAGCCCTACACTAGGGGAATAAATCTTTGGGCACCTAGTGGTCATG

8 CATGACCACTAGGAGCATCTTTGGCGACGTTATGAGTTGGGAAGCTTGGCGGCCAGACTAGGGGAATAAATCTTTGGGCACCTAGTGGTCATG

8 CATGACCACTAGGAGCATCTTTGGCGAGGTCACCTGGAGGCTCGAGAGGCTTTGTCACTAGGGGAATAAATCTTTGGGCACCTAGTGGTCATG

8 CATGACCACTAGGAGCATCTTTGGCGACGACAAAGGTTGCGCGTCGTGGAGGAGACACTAGGGGAATAAATCTTTGGGCACCTAGTGGTCATG

8 CATGACCACTAGGAGCATCTTTGGCGAGCCTAGCTTGGGAGGTTGCTCCACCAGTTCCTAGGGGAATAAATCTTTGGGCACCTAGTGGTCATG

8 CATGACCACTAGGAGCATCTTTGGCGAAAGCCCGGTGTGGGTGCGCAAGCTATATGCCTAGGGGAATAAATCTTTGGGCACCTAGTGGTCATG

8 CATGACCACTAGGAGCATCTTTGGCGAGCCTCTCTTGGGAGGTTGCTCCACCAGTTCCTAGGGGAATAAATCTTTGGGCACCTAGTGGTCATG

8 CATGACCACTAGGAGCATCTTTGGCGACCCATCACTGATAAGCATCATGTGGGTGGACTAGGGGAATAAATCTTTGGGCACCTAGTGGTCATG

8 CATGACCACTAGGAGCATCTTTGGCGAAAGTCGGTAGAACAGGGTTGGGTGCTGTCCCTAGGGGAATAAATCTTTGGGCACCTAGTGGTCATG

8 CATGACCACTAGGAGCATCTTTGGCGAAGGACGGTATAGCGAAGCGGAGTGTGTCACCTAGGGGAATAAATCTTTGGGCACCTAGTGGTCATG

8 CATGACCACTAGGAGCATCTTTGGCGATGGGTGCAGACGAGTGCGCTAGCAATTACACTAGGGGAATAAATCTTTGGGCACCTAGTGGTCATG

8 CATGACCACTAGGAGCATCTTTGGCGACGAAAGTGGCCATCTCAAGCGCGTGTGATCCTAGGGGAATAAATCTTTGGGCACCTAGTGGTCATG

8 CATGACCACTAGGAGCATCTTTGGCGACGGTGTGGGGAACTTGTTTCGGCGGTGCTGCTAGGGGAATAAATCTTTGGGCACCTAGTGGTCATG

8 CATGACCACTAGGAGCATCTTTGGCGATCTGCGTGGTCGTCGGATCAAGTTACGCCGCTAGGGGAATAAATCTTTGGGCACCTAGTGGTCATG

8 CATGACCACTAGGAGCATCTTTGGCGAACGGCAGGTGTTGGGGTGGTCTGTGAATCCCTAGGGGAATAAATCTTTGGGCACCTAGTGGTCATG

8 CATGACCACTAGGAGCATCTTTGGCGATTAGGCCGCCAGGGCGTACGAAGCGTGGTTCTAGGGGAATAAATCTTTGGGCACCTAGTGGTCATG

8 CATGACCACTAGGAGCATCTTTGGCGACGGCGTGGGGCATCTTTCGCGGCGGTTATTCTAGGGGAATAAATCTTTGGGCACCTAGTGGTCATG

8 CATGACCACTAGGAGCATCTTTGGCGAGCCCCGCTTGGGAGGTTGCTCCACCAGTTCCTAGGGGAATAAATCTTTGGGCACCTAGTGGTCATG

8 CATGACCACTAGGAGCATCTTTGGCGAGTATCGGCTGGTAGGTTGCGTATTGGGGAGCTAGGGGAATAAATCTTTGGGCACCTAGTGGTCATG

8 CATGACCACTAGGAGCATCTTTGGCGATGGGGCCGTGCTATCTGCACACTACGCGGGCTAGGGGAATAAATCTTTGGGCACCTAGTGGTCATG

8 CATGACCACTAGGAGCATCTTTGGCGAGCCTGCCGATATGGCCGAGCAGACGTTTGGCTAGGGGAATAAATCTTTGGGCACCTAGTGGTCATG

8 CATGACCACTAGGAGCATCTTTGGCGACTCATGTGGTCCCGTACCACAGGTAGTGGACTAGGGGAATAAATCTTTGGGCACCTAGTGGTCATG

8 CATGACCACTAGGAGCATCTTTGGCGATGTGGGTAGAACCACCCCATACGGTGTCAGCTAGGGGAATAAATCTTTGGGCACCTAGTGGTCATG

8 CATGACCACTAGGAGCATCTTTGGCGACACGCGTGCGCTGCAACGCCGCGTTGTGGACTAGGGGAATAAATCTTTGGGCACCTAGTGGTCATG

8 CATGACCACTAGGAGCATCTTTGGCGAATGGCCGCCACGGCCTTCTAGTCCTCGGTACTAGGGGAATAAATCTTTGGGCACCTAGTGGTCATG

8 CATGACCACTAGGAGCATCTTTGGCGACATGGGCCGGAAGGATGGTATTTGTAGCCGCTAGGGGAATAAATCTTTGGGCACCTAGTGGTCATG

8 CATGACCACTAGGAGCATCTTTGGCGAGGGGTGTAGGACTTCAAGTGGATCTCATAGCTAGGGGAATAAATCTTTGGGCACCTAGTGGTCATG

8 CATGACCACTAGGAGCATCTTTGGCGACTCCGACATGCAGCGTCAGGGCGGTGTGGACTAGGGGAATAAATCTTTGGGCACCTAGTGGTCATG

8 CATGACCACTAGGAGCATCTTTGGCGACGGGTGTAGGACTTCAAGTGGATCTCATAGCTAGGGGAATAAATCTTTGGGCACCTAGTGGTCATG

8 CATGACCACTAGGAGCATCTTTGGCGAGAAGTGGCGCTTGGGCCGTTTAGTCCAGAGCTAGGGGAATAAATCTTTGGGCACCTAGTGGTCATG

8 CATGACCACTAGGAGCATCTTTGGCGAGTGGGGTCGTGGTTAAATCCGTGCTCGAGCCTAGGGGAATAAATCTTTGGGCACCTAGTGGTCATG

8 CATGACCACTAGGAGCATCTTTGGCGAGCCTCACTTGGGAGGTTGCTCCACCAGTTCCTAGGGGAATAAATCTTTGGGCACCTAGTGGTCATG

8 CATGACCACTAGGAGCATCTTTGGCGACAGGGGATGCCGATGCGCTGGACGACTGAGCTAGGGGAATAAATCTTTGGGCACCTAGTGGTCATG

8 CATGACCACTAGGAGCATCTTTGGCGATCAGGCCGCCAGGGCGTACGGAGTGTGGTTCTAGGGGAATAAATCTTTGGGCACCTAGTGGTCATG

8 CATGACCACTAGGAGCATCTTTGGCGAAAGTTGGTAGAACAGGGTGGGGTGCTGTCCCTAGGGGAATAAATCTTTGGGCACCTAGTGGTCATG

8 CATGACCACTAGGAGCATCTTTGGCGACCCGTAAGACATAGTTGCACGAGGTGGAGACTAGGGGAATAAATCTTTGGGCACCTAGTGGTCATG

8 CATGACCACTAGGAGCATCTTTGGCGACCAGGCCGCCAGGGCGTACGAAGTGTGGTTCTAGGGGAATAAATCTTTGGGCACCTAGTGGTCATG

8 CATGACCACTAGGAGCATCTTTGGCGAACGGCAGGTGTTGCGGTGGTCTGTGATTCCCTAGGGGAATAAATCTTTGGGCACCTAGTGGTCATG

8 CATGACCACTAGGAGCATCTTTGGCGACGCGGTGTCATCGTTTGGGGCAAGTTGCTGCTAGGGGAATAAATCTTTGGGCACCTAGTGGTCATG

8 CATGACCACTAGGAGCATCTTTGGCGAGAAGACTTCGGATTCGGGGACCAGTTGCTGCTAGGGGAATAAATCTTTGGGCACCTAGTGGTCATG

8 CATGACCACTAGGAGCATCTTTGGCGACCCGGGATCGGGCAACGAACACGTGGTGGACTAGGGGAATAAATCTTTGGGCACCTAGTGGTCATG

8 CATGACCACTAGGAGCATCTTTGGCGACTGGACGAGGGTAACACGAGGTTGTTTGGCCTAGGGGAATAAATCTTTGGGCACCTAGTGGTCATG

8 CATGACCACTAGGAGCATCTTTGGCGAGTGCGAGCTGGTAGGATGTATCTGAAACGACTAGGGGAATAAATCTTTGGGCACCTAGTGGTCATG

8 CATGACCACTAGGAGCATCTTTGGCGAAAGTCGGTAGAACAGGATGGGGTGCTGTCCCTAGGGGAATAAATCTTTGGGCACCTAGTGGTCATG

8 CATGACCACTAGGAGCATCTTTGGCGAGATCAGGAGAATCGGCGGCATTGGTGTCTCCTAGGGGAATAAATCTTTGGGCACCTAGTGGTCATG

8 CATGACCACTAGGAGCATCTTTGGCGAAAGTCGGTAGAACATGGTGGGGTGCTGTCCCTAGGGGAATAAATCTTTGGGCACCTAGTGGTCATG

8 CATGACCACTAGGAGCATCTTTGGCGAACGGCAGGTCTTGCGGTGGTCTGTGAATCCCTAGGGGAATAAATCTTTGGGCACCTAGTGGTCATG

8 CATGACCACTAGGAGCATCTTTGGCGAGCCTCGCTTGGGAGGTTGCTCCACAAGTTCCTAGGGGAATAAATCTTTGGGCACCTAGTGGTCATG

8 CATGACCACTAGGAGCATCTTTGGCGACCCTCGTCAGATGGTGAAGCAGACGTTTGGCTAGGGGAATAAATCTTTGGGCACCTAGTGGTCATG

8 CATGACCACTAGGAGCATCTTTGGCGAAAGTCGGTAGAACAGGGTGGGGTGCTTTCCCTAGGGGAATAAATCTTTGGGCACCTAGTGGTCATG

8 CATGACCACTAGGAGCATCTTTGGCGAGGGGTAGGGGGCCGCTCGCGCCTGTGTGCGCTAGGGGAATAAATCTTTGGGCACCTAGTGGTCATG

8 CATGACCACTAGGAGCATCTTTGGCGAGTCGGGGATTTCCGGAGTCGTTGACTGGTCCTAGGGGAATAAATCTTTGGGCACCTAGTGGTCATG

8 CATGACCACTAGGAGCATCTTTGGCGATCAGATCCCGGGAGTGTGAGAGGCCCAGCGCTAGGGGAATAAATCTTTGGGCACCTAGTGGTCATG

8 CATGACCACTAGGAGCATCTTTGGCGAACGGCAGGTGTTGCGGTGCTCTGTGAATCCCTAGGGGAATAAATCTTTGGGCACCTAGTGGTCATG

7 CATGACCACTAGGAGCATCTTTGGCGACTTTCCTTATGCTCACCCGCAGAGGGTTGTCTAGGGGAATAAATCTTTGGGCACCTAGTGGTCATG

7 CATGACCACTAGGAGCATCTTTGGCGACGAAAGCGAAGGTACGCTAAGCGAAGTGGACTAGGGGAATAAATCTTTGGGCACCTAGTGGTCATG

7 CATGACCACTAGGAGCATCTTTGGCGAAACGGTTCGGCAGGAATCCGGTAAGTGGAGCTAGGGGAATAAATCTTTGGGCACCTAGTGGTCATG

7 CATGACCACTAGGAGCATCTTTGGCGAGCCTCGCTTGGGAGGTAGCTCCACCAGTTCCTAGGGGAATAAATCTTTGGGCACCTAGTGGTCATG

7 CATGACCACTAGGAGCATCTTTGGCGACGTTGTGGGCCCACATGTGGCACCGTGGAGCTAGGGGAATAAATCTTTGGGCACCTAGTGGTCATG

7 CATGACCACTAGGAGCATCTTTGGCGAGCCTCGCTTGGGAGGTTGCTCCACCAGTACCTAGGGGAATAAATCTTTGGGCACCTAGTGGTCATG

7 CATGACCACTAGGAGCATCTTTGGCGACGTGCGGAGTGAACAGATGCTCTTTGCTCGCTAGGGGAATAAATCTTTGGGCACCTAGTGGTCATG

7 CATGACCACTAGGAGCATCTTTGGCGAACGGCAGCTGTTGCGGTGGTCTGTGAATCCCTAGGGGAATAAATCTTTGGGCACCTAGTGGTCATG

7 CATGACCACTAGGAGCATCTTTGGCGACCCGGGAGCCCTTGTGGATCTCGTGGTGGACTAGGGGAATAAATCTTTGGGCACCTAGTGGTCATG

7 CATGACCACTAGGAGCATCTTTGGCGACCCCGTTAGTCGAGACGACACGGTGGTGGACTAGGGGAATAAATCTTTGGGCACCTAGTGGTCATG

7 CATGACCACTAGGAGCATCTTTGGCGAGAAGACTCGGGATTCGGGGACCAGTTGCTGCTAGGGGAATAAATCTTTGGGCACCTAGTGGTCATG

7 CATGACCACTAGGAGCATCTTTGGCGAACGGCAGGTGTTGCGGTGGTTCGTGAATCCCTAGGGGAATAAATCTTTGGGCACCTAGTGGTCATG

7 CATGACCACTAGGAGCATCTTTGGCGACGGTGTGGGTGCAGGGCCAGGCGGTTGATTCTAGGGGAATAAATCTTTGGGCACCTAGTGGTCATG

7 CATGACCACTAGGAGCATCTTTGGCGAAAGTCGGTAGAACAGAGTGGGGTGCTGTCCCTAGGGGAATAAATCTTTGGGCACCTAGTGGTCATG

7 CATGACCACTAGGAGCATCTTTGGCGAATCGTGTTGCAAGGTCCGCGAGTGTGGACCCTAGGGGAATAAATCTTTGGGCACCTAGTGGTCATG

7 CATGACCACTAGGAGCATCTTTGGCGATCAAGTGCGCTTGGTAATTCCTGATGCGACCTAGGGGAATAAATCTTTGGGCACCTAGTGGTCATG

7 CATGACCACTAGGAGCATCTTTGGCGAACGGCAGGTGTTGCGGTGGTCTGTGAATGCCTAGGGGAATAAATCTTTGGGCACCTAGTGGTCATG

7 CATGACCACTAGGAGCATCTTTGGCGAGCCTCGCTTGGGAGGTTTCTCCACCAGTTCCTAGGGGAATAAATCTTTGGGCACCTAGTGGTCATG

7 CATGACCACTAGGAGCATCTTTGGCGATTGAGCTGGGAGGGCAGGTATTTATCGCCGCTAGGGGAATAAATCTTTGGGCACCTAGTGGTCATG

7 CATGACCACTAGGAGCATCTTTGGCGATAGGCGTGTACGGTCGAGCGAGAGCGATAGCTAGGGGAATAAATCTTTGGGCACCTAGTGGTCATG

7 CATGACCACTAGGAGCATCTTTGGCGAGAAGACTCTGGATTCGGGGACCAGTGTCTCCTAGGGGAATAAATCTTTGGGCACCTAGTGGTCATG

7 CATGACCACTAGGAGCATCTTTGGCGATCCGTAGGAATGGCAGGCATACTGCGTCGCCTAGGGGAATAAATCTTTGGGCACCTAGTGGTCATG

7 CATGACCACTAGGAGCATCTTTGGCGAGGGAATGTAGTTGACAGAGCAGACGTTTGGCTAGGGGAATAAATCTTTGGGCACCTAGTGGTCATG

7 CATGACCACTAGGAGCATCTTTGGCGACAGTACGGTGCGAGATCCAAAATCGGCCAGCTAGGGGAATAAATCTTTGGGCACCTAGTGGTCATG

7 CATGACCACTAGGAGCATCTTTGGCGAGAAGACTTTGGATTCGGGGACCAGTTGCTCCTAGGGGAATAAATCTTTGGGCACCTAGTGGTCATG

7 CATGACCACTAGGAGCATCTTTGGCGAGGAGTGGTTGAGGGCGGTGGGTAGGTCGCGCTAGGGGAATAAATCTTTGGGCACCTAGTGGTCATG

7 CATGACCACTAGGAGCATCTTTGGCGACAGGGGATGCCGATGCGCCGGACGACCGAGCTAGGGGAATAAATCTTTGGGCACCTAGTGGTCATG

7 CATGACCACTAGGAGCATCTTTGGCGAGATCGGGAGAATCGGCGGCATTGGTGCCTCCTAGGGGAATAAATCTTTGGGCACCTAGTGGTCATG

7 CATGACCACTAGGAGCATCTTTGGCGACTGGGCTGGCAGGATGGTATTGTGTTCGCACTAGGGGAATAAATCTTTGGGCACCTAGTGGTCATG

7 CATGACCACTAGGAGCATCTTTGGCGACCGTAGGAAAACAAAGGTGGCCTTGCTAGCCTAGGGGAATAAATCTTTGGGCACCTAGTGGTCATG

7 CATGACCACTAGGAGCATCTTTGGCGAGCCTTGCTTGGGAGGTCGCTCCACCAGTTCCTAGGGGAATAAATCTTTGGGCACCTAGTGGTCATG

7 CATGACCACTAGGAGCATCTTTGGCGACCCATCACGGCGCGTGCGCTAATGGGTGGACTAGGGGAATAAATCTTTGGGCACCTAGTGGTCATG

7 CATGACCACTAGGAGCATCTTTGGCGACTGGCGGCGGAGAGGTTATTGCAGTGGGGCCTAGGGGAATAAATCTTTGGGCACCTAGTGGTCATG

7 CATGACCACTAGGAGCATCTTTGGCGATCCCCTCCGTTGCGGGTTTGACGAGGTTTGCTAGGGGAATAAATCTTTGGGCACCTAGTGGTCATG

7 CATGACCACTAGGAGCATCTTTGGCGAGAAGTGGGCAAGGGGCAAAGTGTGTCGAGCCTAGGGGAATAAATCTTTGGGCACCTAGTGGTCATG

7 CATGACCACTAGGAGCATCTTTGGCGAGGACAAAGTACTGGGCATGTGGTCGAGCTCCTAGGGGAATAAATCTTTGGGCACCTAGTGGTCATG

7 CATGACCACTAGGAGCATCTTTGGCGACCAGATATGGTTGGTAATGTGCTGAGGAGGCTAGGGGAATAAATCTTTGGGCACCTAGTGGTCATG

7 CATGACCACTAGGAGCATCTTTGGCGAAGGGTGTAGGGCTTCAAGTGGATCTCATAGCTAGGGGAATAAATCTTTGGGCACCTAGTGGTCATG

7 CATGACCACTAGGAGCATCTTTGGCGATCTCGTACTGGGATTGTCCAATGTCCCTCACTAGGGGAATAAATCTTTGGGCACCTAGTGGTCATG

7 CATGACCACTAGGAGCATCTTTGGCGAAGAGAGGAAAGGTCGGGAACGGTGCGTTCACTAGGGGAATAAATCTTTGGGCACCTAGTGGTCATG

7 CATGACCACTAGGAGCATCTTTGGCGATGGGCGAAGCGTGGTCGCTGAGGAGACCGACTAGGGGAATAAATCTTTGGGCACCTAGTGGTCATG

7 CATGACCACTAGGAGCATCTTTGGCGACTAGCGTAGGACGGTACCGGGTGTAGGTGACTAGGGGAATAAATCTTTGGGCACCTAGTGGTCATG

7 CATGACCACTAGGAGCATCTTTGGCGAATCTACGATCATCGTAGCGTGGGTAATCGCCTAGGGGAATAAATCTTTGGGCACCTAGTGGTCATG

7 CATGACCACTAGGAGCATCTTTGGCGACGGCGTGGGGCATTTCTCGCGGCGGTTATTCTAGGGGAATAAATCTTTGGGCACCTAGTGGTCATG

7 CATGACCACTAGGAGCATCTTTGGCGAGTGCCGTGGCACAGTAGAGCAGACGTTTGGCTAGGGGAATAAATCTTTGGGCACCTAGTGGTCATG

7 CATGACCACTAGGAGCATCTTTGGCGACCCGGATTTAGAGTATCTAAACAGGGTGGACTAGGGGAATAAATCTTTGGGCACCTAGTGGTCATG

7 CATGACCACTAGGAGCATCTTTGGCGAAGGTGCGACAACGGTGTACATGATATTCGCCTAGGGGAATAAATCTTTGGGCACCTAGTGGTCATG

7 CATGACCACTAGGAGCATCTTTGGCGATAGTGACGGGAGTCGACCCCTGGTTGGGTCCTAGGGGAATAAATCTTTGGGCACCTAGTGGTCATG

7 CATGACCACTAGGAGCATCTTTGGCGAATGACCGGCACGGCCTTCTAGTCCTCGGTACTAGGGGAATAAATCTTTGGGCACCTAGTGGTCATG

7 CATGACCACTAGGAGCATCTTTGGCGAACGGCAGGTGTTCCGGTGGTCTGTGAATCCCTAGGGGAATAAATCTTTGGGCACCTAGTGGTCATG

7 CATGACCACTAGGAGCATCTTTGGCGACCCGACTGCGTGACAATGCCAGCGGGTGGACTAGGGGAATAAATCTTTGGGCACCTAGTGGTCATG

7 CATGACCACTAGGAGCATCTTTGGCGAAAGTCGGTAGAACAGGGTGGGGAGCTGTCCCTAGGGGAATAAATCTTTGGGCACCTAGTGGTCATG

7 CATGACCACTAGGAGCATCTTTGGCGACCTGTAGGTATGTGCAAACGGCCTTGCTAGCTAGGGGAATAAATCTTTGGGCACCTAGTGGTCATG

7 CATGACCACTAGGAGCATCTTTGGCGATGGGTTGCTGGTAGGTGGTACGACTTCGATCTAGGGGAATAAATCTTTGGGCACCTAGTGGTCATG

7 CATGACCACTAGGAGCATCTTTGGCGATAAGGCCGCCAGGGCGTACGAAGTGTGGTTCTAGGGGAATAAATCTTTGGGCACCTAGTGGTCATG

7 CATGACCACTAGGAGCATCTTTGGCGAGAAGACTCTGGAATCGGGGACCAGTTGCTGCTAGGGGAATAAATCTTTGGGCACCTAGTGGTCATG

7 CATGACCACTAGGAGCATCTTTGGCGACGGGGGCATGCACAGGATCGAGACAGTCGACTAGGGGAATAAATCTTTGGGCACCTAGTGGTCATG

7 CATGACCACTAGGAGCATCTTTGGCGAATGGCCGGTACGGCCTTCTAGTCCTTGGTACTAGGGGAATAAATCTTTGGGCACCTAGTGGTCATG

7 CATGACCACTAGGAGCATCTTTGGCGACGACAGTCAGCGTGATCGTTCGCGGGCTGCCTAGGGGAATAAATCTTTGGGCACCTAGTGGTCATG

7 CATGACCACTAGGAGCATCTTTGGCGAAGGGTGTAGGACCTCAAGTGGATCTCATAGCTAGGGGAATAAATCTTTGGGCACCTAGTGGTCATG

7 CATGACCACTAGGAGCATCTTTGGCGAAAGTCGGTAGAACAGGGTGGGATGCTGTCCCTAGGGGAATAAATCTTTGGGCACCTAGTGGTCATG

7 CATGACCACTAGGAGCATCTTTGGCGAGCCTCGCTTGGGAGGTTGCTCCAACAGTTCCTAGGGGAATAAATCTTTGGGCACCTAGTGGTCATG

7 CATGACCACTAGGAGCATCTTTGGCGACAGTTCGTGCGAGTATGCAAATACGGCCTACTAGGGGAATAAATCTTTGGGCACCTAGTGGTCATG

7 CATGACCACTAGGAGCATCTTTGGCGAAGGGTGTAGGACTCCAAGTGGATCTCATAGCTAGGGGAATAAATCTTTGGGCACCTAGTGGTCATG

7 CATGACCACTAGGAGCATCTTTGGCGATAGCCTGGAAGACGGCAGGAAGTGTGTCCACTAGGGGAATAAATCTTTGGGCACCTAGTGGTCATG

7 CATGACCACTAGGAGCATCTTTGGCGAGCTTCGCTTGGGAGGTTGCTCCACCAGTTCCTAGGGGAATAAATCTTTGGGCACCTAGTGGTCATG

7 CATGACCACTAGGAGCATCTTTGGCGACGGTGTGGGGGCGAGAGTCCCGGCGGTTTCCTAGGGGAATAAATCTTTGGGCACCTAGTGGTCATG

7 CATGACCACTAGGAGCATCTTTGGCGAATGGCCGGCACGGCCTTCTAGTCCTCGATACTAGGGGAATAAATCTTTGGGCACCTAGTGGTCATG

7 CATGACCACTAGGAGCATCTTTGGCGACACTGGTCATCCTGTGTTCGGGTAGGTGCGCTAGGGGAATAAATCTTTGGGCACCTAGTGGTCATG

7 CATGACCACTAGGAGCATCTTTGGCGATGGGGCCGTGCTATATGCACACTCCGCGGGCTAGGGGAATAAATCTTTGGGCACCTAGTGGTCATG

7 CATGACCACTAGGAGCATCTTTGGCGAGAGCGGCGTAGGCTGCCTTGGGTGGTGGCCCTAGGGGAATAAATCTTTGGGCACCTAGTGGTCATG

7 CATGACCACTAGGAGCATCTTTGGCGAGTAGTGGGGCATCCAGTGGCTGAGTCTCGACTAGGGGAATAAATCTTTGGGCACCTAGTGGTCATG

7 CATGACCACTAGGAGCATCTTTGGCGATTGGCCGGCACGGCCTTCTAGTCCTCGGTACTAGGGGAATAAATCTTTGGGCACCTAGTGGTCATG

7 CATGACCACTAGGAGCATCTTTGGCGAGGGTTGCGGGCTCCTCGGAGTGTGGGTGGCCTAGGGGAATAAATCTTTGGGCACCTAGTGGTCATG

7 CATGACCACTAGGAGCATCTTTGGCGAGCTCGCCGTAAGGCGGACGAAGTGGAGCTCCTAGGGGAATAAATCTTTGGGCACCTAGTGGTCATG

7 CATGACCACTAGGAGCATCTTTGGCGAACTTGAACGTCGAAGTGGCAGAGAAAGCGACTAGGGGAATAAATCTTTGGGCACCTAGTGGTCATG

7 CATGACCACTAGGAGCATCTTTGGCGAGCCTCGATTGGGAGGTTGCTCCACCAGTTCCTAGGGGAATAAATCTTTGGGCACCTAGTGGTCATG

7 CATGACCACTAGGAGCATCTTTGGCGAGACGAAGGTGCCAAGTGTGGCAGAGCTCGACTAGGGGAATAAATCTTTGGGCACCTAGTGGTCATG

7 CATGACCACTAGGAGCATCTTTGGCGATAGTAGCTCCGTTACATTGGTTGTTTGCTGCTAGGGGAATAAATCTTTGGGCACCTAGTGGTCATG

6 CATGACCACTAGGAGCATCTTTGGCGATCCTCGTCGGATGGTGAAGCAGACGTTTGGCTAGGGGAATAAATCTTTGGGCACCTAGTGGTCATG

6 CATGACCACTAGGAGCATCTTTGGCGACGGAGTGGGGCTCATTGTGCGGCGGTAACCCTAGGGGAATAAATCTTTGGGCACCTAGTGGTCATG

6 CATGACCACTAGGAGCATCTTTGGCGAAGTGCATTGGGTATGTGACTCGGTCGGGCCCTAGGGGAATAAATCTTTGGGCACCTAGTGGTCATG

6 CATGACCACTAGGAGCATCTTTGGCGAAGCGTGTTGCAAGGTCCGCGATTGTGGACCCTAGGGGAATAAATCTTTGGGCACCTAGTGGTCATG

6 CATGACCACTAGGAGCATCTTTGGCGAGCCTTGCTTGGGAGGTTGCTCTACCAGTTCCTAGGGGAATAAATCTTTGGGCACCTAGTGGTCATG

6 CATGACCACTAGGAGCATCTTTGGCGACGGCGTGGGGCCACCTGTGCGGCGGATCCCCTAGGGGAATAAATCTTTGGGCACCTAGTGGTCATG

6 CATGACCACTAGGAGCATCTTTGGCGAAGCAGTGCGACGGGTTATGGGTTCCTCGAGCTAGGGGAATAAATCTTTGGGCACCTAGTGGTCATG

6 CATGACCACTAGGAGCATCTTTGGCGACGAGGTCAAGGGAATCCGTATCGAAGTGGACTAGGGGAATAAATCTTTGGGCACCTAGTGGTCATG

6 CATGACCACTAGGAGCATCTTTGGCGACTCCGAAAAACGCAGGTGTTTCGGTGTGGACTAGGGGAATAAATCTTTGGGCACCTAGTGGTCATG

6 CATGACCACTAGGAGCATCTTTGGCGAAGATGTCTCCACAAGTCATTTGCTTCGCCGCTAGGGGAATAAATCTTTGGGCACCTAGTGGTCATG

6 CATGACCACTAGGAGCATCTTTGGCGATCGATCCGACGATCGGGAGCAGACGTTTGGCTAGGGGAATAAATCTTTGGGCACCTAGTGGTCATG

6 CATGACCACTAGGAGCATCTTTGGCGAAAGTCGGTAAAACAGGGTGGGGTGCTGTCCCTAGGGGAATAAATCTTTGGGCACCTAGTGGTCATG

6 CATGACCACTAGGAGCATCTTTGGCGACGGGATCGTGTCGGCAGATAGCGTACTTGCCTAGGGGAATAAATCTTTGGGCACCTAGTGGTCATG

6 CATGACCACTAGGAGCATCTTTGGCGACCCGTAGTAAGATGGAATCTCACGGGTGGACTAGGGGAATAAATCTTTGGGCACCTAGTGGTCATG

6 CATGACCACTAGGAGCATCTTTGGCGACCCGTAGTAACGTAGGAACGCCCGGGTGGACTAGGGGAATAAATCTTTGGGCACCTAGTGGTCATG

6 CATGACCACTAGGAGCATCTTTGGCGATACGCGGATGAGTCGCGGCCGAAGTGGAGCCTAGGGGAATAAATCTTTGGGCACCTAGTGGTCATG

6 CATGACCACTAGGAGCATCTTTGGCGATGGGTGCTGACGGCCGCCGCTGCGGCTACGCTAGGGGAATAAATCTTTGGGCACCTAGTGGTCATG

6 CATGACCACTAGGAGCATCTTTGGCGAAGGAGAATCGGGAGGTAGGAGTGCTCCAAACTAGGGGAATAAATCTTTGGGCACCTAGTGGTCATG

6 CATGACCACTAGGAGCATCTTTGGCGAATGGCCGGCATGGCCTTCTAGTCCTTGGTACTAGGGGAATAAATCTTTGGGCACCTAGTGGTCATG

6 CATGACCACTAGGAGCATCTTTGGCGATATCTGGCTGGCAGGTTCGTACTGGTTCGACTAGGGGAATAAATCTTTGGGCACCTAGTGGTCATG

6 CATGACCACTAGGAGCATCTTTGGCGAGCCTCGCCTGGGAGGTTGCTCCACCAGTTCCTAGGGGAATAAATCTTTGGGCACCTAGTGGTCATG

6 CATGACCACTAGGAGCATCTTTGGCGAGAAGACGCTGGATTCGGGGACCAGTTGCTGCTAGGGGAATAAATCTTTGGGCACCTAGTGGTCATG

6 CATGACCACTAGGAGCATCTTTGGCGATGAGGCCGCCAGGGCGTACGAAGTGTGGTTCTAGGGGAATAAATCTTTGGGCACCTAGTGGTCATG

6 CATGACCACTAGGAGCATCTTTGGCGATGGGTGCTGACGGCCGCCGCAGCGGCTACACTAGGGGAATAAATCTTTGGGCACCTAGTGGTCATG

6 CATGACCACTAGGAGCATCTTTGGCGACATCAGCTCCGCGGTCGGGGACATAAGGGACTAGGGGAATAAATCTTTGGGCACCTAGTGGTCATG

6 CATGACCACTAGGAGCATCTTTGGCGACGTGGTGTGTTGCCCAAGTGGCTGAGTTGACTAGGGGAATAAATCTTTGGGCACCTAGTGGTCATG

6 CATGACCACTAGGAGCATCTTTGGCGAAAGTCGCTAGAACAGGGTGGGGTGCTGTCCCTAGGGGAATAAATCTTTGGGCACCTAGTGGTCATG

6 CATGACCACTAGGAGCATCTTTGGCGAGGGAGGGCGCCGGCAGTGGTGTGAATGCGCCTAGGGGAATAAATCTTTGGGCACCTAGTGGTCATG

6 CATGACCACTAGGAGCATCTTTGGCGATCATGCCGCCAGGGCGTACGAAGTGTGGTTCTAGGGGAATAAATCTTTGGGCACCTAGTGGTCATG

6 CATGACCACTAGGAGCATCTTTGGCGAGCCTACGCCATACGGCGCAATGCCTGGGTCCTAGGGGAATAAATCTTTGGGCACCTAGTGGTCATG

6 CATGACCACTAGGAGCATCTTTGGCGATCAGGCCGCCAGGGCGTACGAAGGGTGGTTCTAGGGGAATAAATCTTTGGGCACCTAGTGGTCATG

6 CATGACCACTAGGAGCATCTTTGGCGACAAGCTGGTATTACCAGTCCTGAAGTGGACCTAGGGGAATAAATCTTTGGGCACCTAGTGGTCATG

6 CATGACCACTAGGAGCATCTTTGGCGAAAGCCGGTAGAACAGGGTGGGGTGCTGTCCCTAGGGGAATAAATCTTTGGGCACCTAGTGGTCATG

6 CATGACCACTAGGAGCATCTTTGGCGAAGCGTGTTGCAAGGTCCGCGAGTGTGTACCCTAGGGGAATAAATCTTTGGGCACCTAGTGGTCATG

6 CATGACCACTAGGAGCATCTTTGGCGACCCGGGCATCGTTTACGATCCAGTGGTGGACTAGGGGAATAAATCTTTGGGCACCTAGTGGTCATG

6 CATGACCACTAGGAGCATCTTTGGCGAGAGGGGGACAAAGTGCGGGTGGTCGAGCCTCTAGGGGAATAAATCTTTGGGCACCTAGTGGTCATG

6 CATGACCACTAGGAGCATCTTTGGCGAGGTCGAGAAGAATTGAGGATACAGGTGCCTCTAGGGGAATAAATCTTTGGGCACCTAGTGGTCATG

6 CATGACCACTAGGAGCATCTTTGGCGAATGCGAAGGGCTCTGGGACGTAATGAGCCCCTAGGGGAATAAATCTTTGGGCACCTAGTGGTCATG

6 CATGACCACTAGGAGCATCTTTGGCGATAGCGCAGGGCATGCGACCTCCAATTGGTCCTAGGGGAATAAATCTTTGGGCACCTAGTGGTCATG

6 CATGACCACTAGGAGCATCTTTGGCGAACTGGCGAAAGCCAATGCCTGGAGCGCAGGCTAGGGGAATAAATCTTTGGGCACCTAGTGGTCATG

6 CATGACCACTAGGAGCATCTTTGGCGAATGGCGGGCACGGCCTTCTAGTCCTCGGTACTAGGGGAATAAATCTTTGGGCACCTAGTGGTCATG

6 CATGACCACTAGGAGCATCTTTGGCGAGGGAGGGCGCCGGCAGCGGTGTGAATACGCCTAGGGGAATAAATCTTTGGGCACCTAGTGGTCATG

6 CATGACCACTAGGAGCATCTTTGGCGACTCCAGTTCATCTGTCGGGCAGTTCAAGGACTAGGGGAATAAATCTTTGGGCACCTAGTGGTCATG

6 CATGACCACTAGGAGCATCTTTGGCGAAAATCGGTAGAACAGGGTGGGGTGCTGTCCCTAGGGGAATAAATCTTTGGGCACCTAGTGGTCATG

6 CATGACCACTAGGAGCATCTTTGGCGAGCCTCGCTTTGGAGGTTGCTCCACCAGTTCCTAGGGGAATAAATCTTTGGGCACCTAGTGGTCATG

6 CATGACCACTAGGAGCATCTTTGGCGACCCCTGGCTGGTAGTGCGTACTCTGTCGAACTAGGGGAATAAATCTTTGGGCACCTAGTGGTCATG

6 CATGACCACTAGGAGCATCTTTGGCGACTGAAGCACTGTGAGAACGGTGCCAGTGGACTAGGGGAATAAATCTTTGGGCACCTAGTGGTCATG

6 CATGACCACTAGGAGCATCTTTGGCGAGGGAAGTGGTCAAGCGGCAGCTCCCGTAGACTAGGGGAATAAATCTTTGGGCACCTAGTGGTCATG

6 CATGACCACTAGGAGCATCTTTGGCGACTATAAGTGGGATCTACGGATGGATGGTGCCTAGGGGAATAAATCTTTGGGCACCTAGTGGTCATG

6 CATGACCACTAGGAGCATCTTTGGCGAGATCGGGAGAATGGGTGGCATTGGTGTCTCCTAGGGGAATAAATCTTTGGGCACCTAGTGGTCATG

6 CATGACCACTAGGAGCATCTTTGGCGAGAAGACTCTGGTTTCGGGGACCAGTTGCTGCTAGGGGAATAAATCTTTGGGCACCTAGTGGTCATG

6 CATGACCACTAGGAGCATCTTTGGCGACCACATGTGTGCTAAACATATGGTGGTGGACTAGGGGAATAAATCTTTGGGCACCTAGTGGTCATG

6 CATGACCACTAGGAGCATCTTTGGCGACGGTTGGTCGGAAGGCAAGTGTTCGGATGACTAGGGGAATAAATCTTTGGGCACCTAGTGGTCATG

6 CATGACCACTAGGAGCATCTTTGGCGACCCGTAACGAGCTTCAGCCGCGCGGGTGGACTAGGGGAATAAATCTTTGGGCACCTAGTGGTCATG

6 CATGACCACTAGGAGCATCTTTGGCGAGATCGGGAGAATCGGTGGCATTGGTGTGTCCTAGGGGAATAAATCTTTGGGCACCTAGTGGTCATG

6 CATGACCACTAGGAGCATCTTTGGCGAAGCGTGTTGCAAGGCCCGCGAGTGTGGACCCTAGGGGAATAAATCTTTGGGCACCTAGTGGTCATG

6 CATGACCACTAGGAGCATCTTTGGCGAGGGAGGGCGCCGGCAGCGGTGTGTATGCGCCTAGGGGAATAAATCTTTGGGCACCTAGTGGTCATG

6 CATGACCACTAGGAGCATCTTTGGCGAGGCGAGTCCAGCGGCCTTCTCGGCCTGCGACTAGGGGAATAAATCTTTGGGCACCTAGTGGTCATG

6 CATGACCACTAGGAGCATCTTTGGCGAAGCGTGTTGCAAGGTCCGCGAGCGTGGACCCTAGGGGAATAAATCTTTGGGCACCTAGTGGTCATG

6 CATGACCACTAGGAGCATCTTTGGCGACATCCCTGCGGTGTAACAAGCGGGTGTGGACTAGGGGAATAAATCTTTGGGCACCTAGTGGTCATG

6 CATGACCACTAGGAGCATCTTTGGCGAGAAGACTCTGGATTCGGGGTCCAGTTGCTGCTAGGGGAATAAATCTTTGGGCACCTAGTGGTCATG

6 CATGACCACTAGGAGCATCTTTGGCGAGCCTCGCTTGGGAGGTTACTCCACCAGTTCCTAGGGGAATAAATCTTTGGGCACCTAGTGGTCATG

6 CATGACCACTAGGAGCATCTTTGGCGAGCCTCGCTTGGGAGGTTGCTCCACTAGTTCCTAGGGGAATAAATCTTTGGGCACCTAGTGGTCATG

6 CATGACCACTAGGAGCATCTTTGGCGATGAGCTGTGCACGTGGGTGTGGTCCGGGCCCTAGGGGAATAAATCTTTGGGCACCTAGTGGTCATG

6 CATGACCACTAGGAGCATCTTTGGCGAAACGCCGTAGTCGGCAGATTGGTCGAGGTCCTAGGGGAATAAATCTTTGGGCACCTAGTGGTCATG

6 CATGACCACTAGGAGCATCTTTGGCGACAGGCGCTGGAAGGTTGTGTATGTTTTCGACTAGGGGAATAAATCTTTGGGCACCTAGTGGTCATG

6 CATGACCACTAGGAGCATCTTTGGCGACGATGTGGGCAAGGGATCGGATAGAGTCGACTAGGGGAATAAATCTTTGGGCACCTAGTGGTCATG

6 CATGACCACTAGGAGCATCTTTGGCGACCCGTAAGAGCTCCGCGTAGACGAGGTGGACTAGGGGAATAAATCTTTGGGCACCTAGTGGTCATG

6 CATGACCACTAGGAGCATCTTTGGCGAGCCTCGCTTGGGAGGTTGCTCCACCAATTCCTAGGGGAATAAATCTTTGGGCACCTAGTGGTCATG

6 CATGACCACTAGGAGCATCTTTGGCGAACGCGGCTGGCAGGGATGTATTAAGTGGAGCTAGGGGAATAAATCTTTGGGCACCTAGTGGTCATG

6 CATGACCACTAGGAGCATCTTTGGCGAGAAGTCTCTGGATTCGGGGACCAGTTGCTGCTAGGGGAATAAATCTTTGGGCACCTAGTGGTCATG

6 CATGACCACTAGGAGCATCTTTGGCGAGAGCCGTAGCCGTAAGGTTGGTATAAGGCCCTAGGGGAATAAATCTTTGGGCACCTAGTGGTCATG

6 CATGACCACTAGGAGCATCTTTGGCGAGCTGGCGAAAGCCAATGCCTGGAGCGCAGGCTAGGGGAATAAATCTTTGGGCACCTAGTGGTCATG

6 CATGACCACTAGGAGCATCTTTGGCGACGGCGTGAACGTTGCCGTGAGTTTAGTCCTCTAGGGGAATAAATCTTTGGGCACCTAGTGGTCATG

6 CATGACCACTAGGAGCATCTTTGGCGATACGGCCTGCACGCCTGGGTGCCGCGGGGCCTAGGGGAATAAATCTTTGGGCACCTAGTGGTCATG

6 CATGACCACTAGGAGCATCTTTGGCGAGAAGACTCTGGATTCGGGGACCTGTTGCTGCTAGGGGAATAAATCTTTGGGCACCTAGTGGTCATG

6 CATGACCACTAGGAGCATCTTTGGCGACGGTACTGTTTACGGCCGGTTGGCGAGAGACTAGGGGAATAAATCTTTGGGCACCTAGTGGTCATG

6 CATGACCACTAGGAGCATCTTTGGCGACAAGTGAAGAGGAACGAAGCGCTAGTGGAGCTAGGGGAATAAATCTTTGGGCACCTAGTGGTCATG

6 CATGACCACTAGGAGCATCTTTGGCGAATGACCGAAGACATGTGGCAGAGAGGTCGCCTAGGGGAATAAATCTTTGGGCACCTAGTGGTCATG

6 CATGACCACTAGGAGCATCTTTGGCGAGCCTCGCTTGGGAGGTTGTTCCACCAGTTCCTAGGGGAATAAATCTTTGGGCACCTAGTGGTCATG

6 CATGACCACTAGGAGCATCTTTGGCGAGACTCGCTTGGGAGGTTGCTCCACCAGTTCCTAGGGGAATAAATCTTTGGGCACCTAGTGGTCATG

6 CATGACCACTAGGAGCATCTTTGGCGAGATAGGGAGAATCGGTGGAATTGGTGTCTCCTAGGGGAATAAATCTTTGGGCACCTAGTGGTCATG

6 CATGACCACTAGGAGCATCTTTGGCGATACGGGGTGTTGCGCTGGTGGTTGGGGTCGCTAGGGGAATAAATCTTTGGGCACCTAGTGGTCATG

6 CATGACCACTAGGAGCATCTTTGGCGATTGGCTGGGAGAAGGGCTCTAAGTATTCAGCTAGGGGAATAAATCTTTGGGCACCTAGTGGTCATG

6 CATGACCACTAGGAGCATCTTTGGCGACCCAATCTGTAAGAATTACGAGTGGGTGGACTAGGGGAATAAATCTTTGGGCACCTAGTGGTCATG

6 CATGACCACTAGGAGCATCTTTGGCGACAACGGGGTGTGAGGAGCTAAGGTGCGACACTAGGGGAATAAATCTTTGGGCACCTAGTGGTCATG

6 CATGACCACTAGGAGCATCTTTGGCGAGCGATAGCAGCTAAACATTGAGGCTCGCTCCTAGGGGAATAAATCTTTGGGCACCTAGTGGTCATG

6 CATGACCACTAGGAGCATCTTTGGCGAGATCGGGAGAATCGGTGGCATTGGAGACTCCTAGGGGAATAAATCTTTGGGCACCTAGTGGTCATG

6 CATGACCACTAGGAGCATCTTTGGCGATGGAGGTGTGTACGTTAGGGGCGACACGCACTAGGGGAATAAATCTTTGGGCACCTAGTGGTCATG

6 CATGACCACTAGGAGCATCTTTGGCGACGGTGTGGGGACCTTGTTTCGGCGGTGCTACTAGGGGAATAAATCTTTGGGCACCTAGTGGTCATG

6 CATGACCACTAGGAGCATCTTTGGCGATTTGGGAGTCGCAGCATGCAGGCAGTGAGACTAGGGGAATAAATCTTTGGGCACCTAGTGGTCATG

6 CATGACCACTAGGAGCATCTTTGGCGACCCACATCAACGCAGTATATGGTGGGTGGACTAGGGGAATAAATCTTTGGGCACCTAGTGGTCATG

6 CATGACCACTAGGAGCATCTTTGGCGACCCGGTCACGGCAAGTAGTACCGTGGTGGACTAGGGGAATAAATCTTTGGGCACCTAGTGGTCATG

6 CATGACCACTAGGAGCATCTTTGGCGACGTTTGGCTGGGAGGTCAAGTATTAGGCGGCTAGGGGAATAAATCTTTGGGCACCTAGTGGTCATG

6 CATGACCACTAGGAGCATCTTTGGCGAGCCCTGCTTGGGAGGTTGCTCCACCAGTTCCTAGGGGAATAAATCTTTGGGCACCTAGTGGTCATG

6 CATGACCACTAGGAGCATCTTTGGCGATAGGCGTGTACGGTCGAGCGGAAGCGATAGCTAGGGGAATAAATCTTTGGGCACCTAGTGGTCATG

6 CATGACCACTAGGAGCATCTTTGGCGACAGGCGTGTACGGTCGAGCGAAAGCGATAGCTAGGGGAATAAATCTTTGGGCACCTAGTGGTCATG

6 CATGACCACTAGGAGCATCTTTGGCGACGGTGTGGGGCATCTCTCGCGGCGGTTATTCTAGGGGAATAAATCTTTGGGCACCTAGTGGTCATG

6 CATGACCACTAGGAGCATCTTTGGCGATTGGGACGTCCCATTTGGGACACTACGTTCCTAGGGGAATAAATCTTTGGGCACCTAGTGGTCATG

6 CATGACCACTAGGAGCATCTTTGGCGAGGGATAGAATCCGCGGATTTTTTGTCGAGCCTAGGGGAATAAATCTTTGGGCACCTAGTGGTCATG

6 CATGACCACTAGGAGCATCTTTGGCGACCCAGAGACCGACGAAGCGCTATGGGTGGACTAGGGGAATAAATCTTTGGGCACCTAGTGGTCATG

6 CATGACCACTAGGAGCATCTTTGGCGATGGGTGTAGGACTTCAAGTGGATCTCATAGCTAGGGGAATAAATCTTTGGGCACCTAGTGGTCATG

6 CATGACCACTAGGAGCATCTTTGGCGATGGGTGCTGACGGGGCTAGTAAGCCCTACACTAGGGGAATAAATCTTTGGGCACCTAGTGGTCATG

6 CATGACCACTAGGAGCATCTTTGGCGATGGGATCTGTCGGGCCGGAGTGCTCCAACGCTAGGGGAATAAATCTTTGGGCACCTAGTGGTCATG

6 CATGACCACTAGGAGCATCTTTGGCGACGTGGGCGTTTCGGCCGTCTCGGCGTGTGCCTAGGGGAATAAATCTTTGGGCACCTAGTGGTCATG

6 CATGACCACTAGGAGCATCTTTGGCGAAGCGTGTTGCAAGGTCCGCGACTGTGGACCCTAGGGGAATAAATCTTTGGGCACCTAGTGGTCATG

6 CATGACCACTAGGAGCATCTTTGGCGACCGAGTCCGACGAGTGGATGTGTTGGAGAGCTAGGGGAATAAATCTTTGGGCACCTAGTGGTCATG

6 CATGACCACTAGGAGCATCTTTGGCGACCGAGCTGGAAACGCGTTAAGTTGGCGAAGCTAGGGGAATAAATCTTTGGGCACCTAGTGGTCATG

6 CATGACCACTAGGAGCATCTTTGGCGATTGGCGTGTACGGTCGAGCGAAAGCGATAGCTAGGGGAATAAATCTTTGGGCACCTAGTGGTCATG

6 CATGACCACTAGGAGCATCTTTGGCGAACGTGCAATATATGCCGCCTGGAGCGTAGGCTAGGGGAATAAATCTTTGGGCACCTAGTGGTCATG

6 CATGACCACTAGGAGCATCTTTGGCGATCCACGTCGGGGTGTAGTTGCGCAGGCACGCTAGGGGAATAAATCTTTGGGCACCTAGTGGTCATG

6 CATGACCACTAGGAGCATCTTTGGCGATGGGTGCTGACGGGGCTAGAGAGCCCTACACTAGGGGAATAAATCTTTGGGCACCTAGTGGTCATG

6 CATGACCACTAGGAGCATCTTTGGCGATGAGTCATATGATACAAAATTTATACATCGCTAGGGGAATAAATCTTTGGGCACCTAGTGGTCATG

6 CATGACCACTAGGAGCATCTTTGGCGAAGGCATGGCTGGCAGGAAAGTATTGTCTCGCTAGGGGAATAAATCTTTGGGCACCTAGTGGTCATG

6 CATGACCACTAGGAGCATCTTTGGCGACCCGGTGGAACATCGTCAACAGCGGGTGGACTAGGGGAATAAATCTTTGGGCACCTAGTGGTCATG

5 CATGACCACTAGGAGCATCTTTGGCGACGGGGGTTCCCCTCATCCAGGATGTCCCTCCTAGGGGAATAAATCTTTGGGCACCTAGTGGTCATG

5 CATGACCACTAGGAGCATCTTTGGCGACCCGGAACGGGTACCGTGATACCGGGTGGACTAGGGGAATAAATCTTTGGGCACCTAGTGGTCATG

5 CATGACCACTAGGAGCATCTTTGGCGAGTTGTCGTAGAGCCACGGGATACCCGTCAGCTAGGGGAATAAATCTTTGGGCACCTAGTGGTCATG

5 CATGACCACTAGGAGCATCTTTGGCGACTGAACCTGCCAGGAATGGCAGGCAGTGGACTAGGGGAATAAATCTTTGGGCACCTAGTGGTCATG

5 CATGACCACTAGGAGCATCTTTGGCGACCCGACGGCTGGCTACGGCCGAGTGGTGGACTAGGGGAATAAATCTTTGGGCACCTAGTGGTCATG

5 CATGACCACTAGGAGCATCTTTGGCGAGCCCGACTGGACGTCCTGAGATCTGGGATTCTAGGGGAATAAATCTTTGGGCACCTAGTGGTCATG

5 CATGACCACTAGGAGCATCTTTGGCGATCCTCGTCAAATGGTGAAGCAGACGTTTGGCTAGGGGAATAAATCTTTGGGCACCTAGTGGTCATG

5 CATGACCACTAGGAGCATCTTTGGCGAATCGGAGGTGGCTCGGTACAATGATGGCAGCTAGGGGAATAAATCTTTGGGCACCTAGTGGTCATG

5 CATGACCACTAGGAGCATCTTTGGCGAGTCAGTGGGCGGGCGATACTGGGTACGAGACTAGGGGAATAAATCTTTGGGCACCTAGTGGTCATG

5 CATGACCACTAGGAGCATCTTTGGCGACCCAAACGGGTGCGACGCACATTGGGTGGACTAGGGGAATAAATCTTTGGGCACCTAGTGGTCATG

5 CATGACCACTAGGAGCATCTTTGGCGACACTGTCCCACGCGTTATAGGATGCTCGAGCTAGGGGAATAAATCTTTGGGCACCTAGTGGTCATG

5 CATGACCACTAGGAGCATCTTTGGCGACACAGATGTTGATAGATGTGACAAGTGGAGCTAGGGGAATAAATCTTTGGGCACCTAGTGGTCATG

5 CATGACCACTAGGAGCATCTTTGGCGAAGCGTGTTGCAAGGTCAGCGAGTGTGGACCCTAGGGGAATAAATCTTTGGGCACCTAGTGGTCATG

5 CATGACCACTAGGAGCATCTTTGGCGAGTCTCGCTTGGGAGGTTGCTCCACCAGTTCCTAGGGGAATAAATCTTTGGGCACCTAGTGGTCATG

5 CATGACCACTAGGAGCATCTTTGGCGAGTATTGGCTGGTAGGCTGCGTATTGGGGAGCTAGGGGAATAAATCTTTGGGCACCTAGTGGTCATG

5 CATGACCACTAGGAGCATCTTTGGCGACCTCGCTGGTTGGTTAAGTATGGGGTAGATCTAGGGGAATAAATCTTTGGGCACCTAGTGGTCATG

5 CATGACCACTAGGAGCATCTTTGGCGACCCACAATGGACAACTCCATGTAGGGTGGACTAGGGGAATAAATCTTTGGGCACCTAGTGGTCATG

5 CATGACCACTAGGAGCATCTTTGGCGAAGACTGGTTGAGATGCGGGGTGGTCGAGCTCTAGGGGAATAAATCTTTGGGCACCTAGTGGTCATG

5 CATGACCACTAGGAGCATCTTTGGCGATAGCAGAGATCGTACATAGAATTTAATCGCCTAGGGGAATAAATCTTTGGGCACCTAGTGGTCATG

5 CATGACCACTAGGAGCATCTTTGGCGAAAGGCGTGTACGGTCGAGCGAAAGCGATAGCTAGGGGAATAAATCTTTGGGCACCTAGTGGTCATG

5 CATGACCACTAGGAGCATCTTTGGCGATGACTGAGTAGGAGCGAAGAGGGGTACCGCCTAGGGGAATAAATCTTTGGGCACCTAGTGGTCATG

5 CATGACCACTAGGAGCATCTTTGGCGATAGCCGTCAGGGGCGGGTGGGGTTCGCCCCCTAGGGGAATAAATCTTTGGGCACCTAGTGGTCATG

5 CATGACCACTAGGAGCATCTTTGGCGAGCCATTAGGTGTATAGCCGTTGCCTGGGTCCTAGGGGAATAAATCTTTGGGCACCTAGTGGTCATG

5 CATGACCACTAGGAGCATCTTTGGCGACGGGTGGGCGCCCTAGGGTGTGAGTGTTGCCTAGGGGAATAAATCTTTGGGCACCTAGTGGTCATG

5 CATGACCACTAGGAGCATCTTTGGCGAAAGCGCAGGGCATGCGACCTCCAGTTGGTCCTAGGGGAATAAATCTTTGGGCACCTAGTGGTCATG

5 CATGACCACTAGGAGCATCTTTGGCGATCCTCGTCAGATGGTGAAGCAAACGTTTGGCTAGGGGAATAAATCTTTGGGCACCTAGTGGTCATG

5 CATGACCACTAGGAGCATCTTTGGCGATGGGTGCTGACGGCCGCCTCTGCGGCTACACTAGGGGAATAAATCTTTGGGCACCTAGTGGTCATG

5 CATGACCACTAGGAGCATCTTTGGCGACTTGTGGGAGTGGGAAGGGTAGGGCGGAGACTAGGGGAATAAATCTTTGGGCACCTAGTGGTCATG

5 CATGACCACTAGGAGCATCTTTGGCGAGCCTCGCTTGGGAGGTTGCTCCACCATTTCCTAGGGGAATAAATCTTTGGGCACCTAGTGGTCATG

5 CATGACCACTAGGAGCATCTTTGGCGATCCTCGTCAGAGGGTGAAGCAGACGTTTGGCTAGGGGAATAAATCTTTGGGCACCTAGTGGTCATG

5 CATGACCACTAGGAGCATCTTTGGCGACATCCGGGCACCGGGTGTGCACGGTGTGGACTAGGGGAATAAATCTTTGGGCACCTAGTGGTCATG

5 CATGACCACTAGGAGCATCTTTGGCGACACAGGTGATCGGTGCTCGGGTGCCTGGCCCTAGGGGAATAAATCTTTGGGCACCTAGTGGTCATG

5 CATGACCACTAGGAGCATCTTTGGCGATGTTCCGTCGCGCTGTAGGTTGCTCAGTAGCTAGGGGAATAAATCTTTGGGCACCTAGTGGTCATG

5 CATGACCACTAGGAGCATCTTTGGCGACCCAGGAGGCTTGTTCCGACCTGAGGTGGACTAGGGGAATAAATCTTTGGGCACCTAGTGGTCATG

5 CATGACCACTAGGAGCATCTTTGGCGAGTTGCTTGCAAAAGCTATCGTATGATCAGACTAGGGGAATAAATCTTTGGGCACCTAGTGGTCATG

5 CATGACCACTAGGAGCATCTTTGGCGAATGGCGGACACGTGTGTTCGAGAGGACTGGCTAGGGGAATAAATCTTTGGGCACCTAGTGGTCATG

5 CATGACCACTAGGAGCATCTTTGGCGACGGCGTGGGGCCATCTGCGCGGCGGATCCCCTAGGGGAATAAATCTTTGGGCACCTAGTGGTCATG

5 CATGACCACTAGGAGCATCTTTGGCGATCCTCCCGTTCCGGGCCTACGAGGGGTTTGCTAGGGGAATAAATCTTTGGGCACCTAGTGGTCATG

5 CATGACCACTAGGAGCATCTTTGGCGAGAAGACTCTGGATTCGGGGAGCAGTTGCTGCTAGGGGAATAAATCTTTGGGCACCTAGTGGTCATG

5 CATGACCACTAGGAGCATCTTTGGCGATGGGGCCGTGTTATCTGCACACTCCGCGGGCTAGGGGAATAAATCTTTGGGCACCTAGTGGTCATG

5 CATGACCACTAGGAGCATCTTTGGCGACGGCGTGGGGCCATTTGTGCGGCGGATCCTCTAGGGGAATAAATCTTTGGGCACCTAGTGGTCATG

5 CATGACCACTAGGAGCATCTTTGGCGAGAAGACTCTGTATTCGTGGACCAGTTGCTGCTAGGGGAATAAATCTTTGGGCACCTAGTGGTCATG

5 CATGACCACTAGGAGCATCTTTGGCGAATGGTTTTCAAACCTCCGAGTCCTTGGGATCTAGGGGAATAAATCTTTGGGCACCTAGTGGTCATG

5 CATGACCACTAGGAGCATCTTTGGCGACATGGGCGATGTCCGGCGAGGAAACGTCACCTAGGGGAATAAATCTTTGGGCACCTAGTGGTCATG

5 CATGACCACTAGGAGCATCTTTGGCGAGATCGGGGGAATCGGCGGCATTGGTGTCTCCTAGGGGAATAAATCTTTGGGCACCTAGTGGTCATG

5 CATGACCACTAGGAGCATCTTTGGCGAAAGCGCAGGGCATGCGACCTCTAATTGGTCCTAGGGGAATAAATCTTTGGGCACCTAGTGGTCATG

5 CATGACCACTAGGAGCATCTTTGGCGAGGCAGGTGGTCATAATAACCAGGGTGGTAGCTAGGGGAATAAATCTTTGGGCACCTAGTGGTCATG

5 CATGACCACTAGGAGCATCTTTGGCGAAGCGTGTTGCAAGGTCCGCGAGTGTGTCTCCTAGGGGAATAAATCTTTGGGCACCTAGTGGTCATG

5 CATGACCACTAGGAGCATCTTTGGCGACGAGATTTGGTAGGCAGATGTGCTCTAGCACTAGGGGAATAAATCTTTGGGCACCTAGTGGTCATG

5 CATGACCACTAGGAGCATCTTTGGCGAAGGGCAAGGTGACCAAGACGAGGAGATCGCCTAGGGGAATAAATCTTTGGGCACCTAGTGGTCATG

5 CATGACCACTAGGAGCATCTTTGGCGACCCTTTGGGAGTGGATCGGCAGAGAGCCGACTAGGGGAATAAATCTTTGGGCACCTAGTGGTCATG

5 CATGACCACTAGGAGCATCTTTGGCGACCCGGAGCCACGTTAGAGGCCCGCGGTGGACTAGGGGAATAAATCTTTGGGCACCTAGTGGTCATG

5 CATGACCACTAGGAGCATCTTTGGCGAGGAGTGGCTGAGGGCGGTGGGTAGGTCGCACTAGGGGAATAAATCTTTGGGCACCTAGTGGTCATG

5 CATGACCACTAGGAGCATCTTTGGCGATCCTCGTCAGATGGTGGAGCAGACGTTTGGCTAGGGGAATAAATCTTTGGGCACCTAGTGGTCATG

5 CATGACCACTAGGAGCATCTTTGGCGAGGGTAGGCGTCAGGATGATGTGTATTCGCTCTAGGGGAATAAATCTTTGGGCACCTAGTGGTCATG

5 CATGACCACTAGGAGCATCTTTGGCGATGGGTGCTGACGGGGCTAATGAGCCCTACACTAGGGGAATAAATCTTTGGGCACCTAGTGGTCATG

5 CATGACCACTAGGAGCATCTTTGGCGAGATCAGGGGAATCGGTGGCATTGGTGTCTCCTAGGGGAATAAATCTTTGGGCACCTAGTGGTCATG

5 CATGACCACTAGGAGCATCTTTGGCGAAGTGCGGCGGGCCGTGGACTCCGTCCACCACTAGGGGAATAAATCTTTGGGCACCTAGTGGTCATG

5 CATGACCACTAGGAGCATCTTTGGCGACCCCGGTGCGAGAGCAATCGAAGGGGTGGACTAGGGGAATAAATCTTTGGGCACCTAGTGGTCATG

5 CATGACCACTAGGAGCATCTTTGGCGACGAGGTATGGTCAGCGATCTCCGAAGTGGACTAGGGGAATAAATCTTTGGGCACCTAGTGGTCATG

5 CATGACCACTAGGAGCATCTTTGGCGACAGGTTCGTTAGGCGGTGGCCGCGGGAGATCTAGGGGAATAAATCTTTGGGCACCTAGTGGTCATG

5 CATGACCACTAGGAGCATCTTTGGCGATCCGACGAAGTGGCAGAGGCAGAATATCGACTAGGGGAATAAATCTTTGGGCACCTAGTGGTCATG

5 CATGACCACTAGGAGCATCTTTGGCGAGAAGAGTCTGGATTCGGGGACCAGTTGCTGCTAGGGGAATAAATCTTTGGGCACCTAGTGGTCATG

5 CATGACCACTAGGAGCATCTTTGGCGACTAGGGATAGGGTGTGCGAAGAAACCGCCACTAGGGGAATAAATCTTTGGGCACCTAGTGGTCATG

5 CATGACCACTAGGAGCATCTTTGGCGAGATCTGGATAATCGGTGGCATTGGTGTCTCCTAGGGGAATAAATCTTTGGGCACCTAGTGGTCATG

5 CATGACCACTAGGAGCATCTTTGGCGACGGCATTTGTTAGGCTCTGGACAGTGTCACCTAGGGGAATAAATCTTTGGGCACCTAGTGGTCATG

5 CATGACCACTAGGAGCATCTTTGGCGATCGAGTATCGGTGGTCTGATTCAGATGCGCCTAGGGGAATAAATCTTTGGGCACCTAGTGGTCATG

5 CATGACCACTAGGAGCATCTTTGGCGAATGGCCGGGACGGCCTTCTAGTCCTCGGTACTAGGGGAATAAATCTTTGGGCACCTAGTGGTCATG

5 CATGACCACTAGGAGCATCTTTGGCGATCCTCGTCAGATGGTGAAGAAGACGTTTGGCTAGGGGAATAAATCTTTGGGCACCTAGTGGTCATG

5 CATGACCACTAGGAGCATCTTTGGCGAAGCGCATAGGCACAGTGGCTGCGTGGACTACTAGGGGAATAAATCTTTGGGCACCTAGTGGTCATG

5 CATGACCACTAGGAGCATCTTTGGCGACCCGCTGAGGCGAAGTAATAGTCGGGTGGACTAGGGGAATAAATCTTTGGGCACCTAGTGGTCATG

5 CATGACCACTAGGAGCATCTTTGGCGAGATCGGGAGATTCGGTGGCATTGGTGTCTCCTAGGGGAATAAATCTTTGGGCACCTAGTGGTCATG

5 CATGACCACTAGGAGCATCTTTGGCGATATCGGCGTTAGCTTAGGCATGGAGGCCTACTAGGGGAATAAATCTTTGGGCACCTAGTGGTCATG

5 CATGACCACTAGGAGCATCTTTGGCGACAGTTTGTGCGACGCCTGTTGGCGGGCCAGCTAGGGGAATAAATCTTTGGGCACCTAGTGGTCATG

5 CATGACCACTAGGAGCATCTTTGGCGACCCTTGGGATCAGGATAGACCAAGGTGTGACTAGGGGAATAAATCTTTGGGCACCTAGTGGTCATG

5 CATGACCACTAGGAGCATCTTTGGCGATATCGGCGTCAGCCAAGTCCATATGGACTTCTAGGGGAATAAATCTTTGGGCACCTAGTGGTCATG

5 CATGACCACTAGGAGCATCTTTGGCGAGGGGAGGCGCGAAGCTGACGCCCCCTAAGACTAGGGGAATAAATCTTTGGGCACCTAGTGGTCATG

5 CATGACCACTAGGAGCATCTTTGGCGACTCAAACTTAGTGCGTTAGAATTGGGACAGCTAGGGGAATAAATCTTTGGGCACCTAGTGGTCATG

5 CATGACCACTAGGAGCATCTTTGGCGACGGTTGGTCAGGCCCCCAAGGGCCGCTGAGCTAGGGGAATAAATCTTTGGGCACCTAGTGGTCATG

5 CATGACCACTAGGAGCATCTTTGGCGAGAGCCGCCTCGGGTTGGGCCGGTGCGACAGCTAGGGGAATAAATCTTTGGGCACCTAGTGGTCATG

5 CATGACCACTAGGAGCATCTTTGGCGAGGATGACCGAGCGTCGTCCCGAAGTCGGACCTAGGGGAATAAATCTTTGGGCACCTAGTGGTCATG

5 CATGACCACTAGGAGCATCTTTGGCGAGGCGGCCCGGGCCGTATTGTATCGTAATGCCTAGGGGAATAAATCTTTGGGCACCTAGTGGTCATG

5 CATGACCACTAGGAGCATCTTTGGCGACCCGACGTGATATCATCATCTCGCGGTGGACTAGGGGAATAAATCTTTGGGCACCTAGTGGTCATG

5 CATGACCACTAGGAGCATCTTTGGCGAATGGCAGGTGTTGCGGTGGTTTGTGAATCCCTAGGGGAATAAATCTTTGGGCACCTAGTGGTCATG

5 CATGACCACTAGGAGCATCTTTGGCGAAGGCTCTCGGGTCGGGCTGGACTGAACGCTCTAGGGGAATAAATCTTTGGGCACCTAGTGGTCATG

5 CATGACCACTAGGAGCATCTTTGGCGAAGCCCAAGGCAACTCTGTGATATTGCGGGTCTAGGGGAATAAATCTTTGGGCACCTAGTGGTCATG

5 CATGACCACTAGGAGCATCTTTGGCGACGGAGTGGGGCTCATTGTGCGGCGGTGATCCTAGGGGAATAAATCTTTGGGCACCTAGTGGTCATG

5 CATGACCACTAGGAGCATCTTTGGCGAAGCACCCGGCTCACCAGGGAAACCGTTCGGCTAGGGGAATAAATCTTTGGGCACCTAGTGGTCATG

5 CATGACCACTAGGAGCATCTTTGGCGAGGAACGGTTGTCGCAAGAGCTTCAAACCAGCTAGGGGAATAAATCTTTGGGCACCTAGTGGTCATG

5 CATGACCACTAGGAGCATCTTTGGCGACGTTAGGCACCAAATAGAGTGGCCGAGCGCCTAGGGGAATAAATCTTTGGGCACCTAGTGGTCATG

5 CATGACCACTAGGAGCATCTTTGGCGATTTGGGAGTCGCATTATGCAGGCAGTGAGGCTAGGGGAATAAATCTTTGGGCACCTAGTGGTCATG

5 CATGACCACTAGGAGCATCTTTGGCGACAGCCGTAGCCGTAAGGTTGGTATAGGGCCCTAGGGGAATAAATCTTTGGGCACCTAGTGGTCATG

5 CATGACCACTAGGAGCATCTTTGGCGAGCCTCGCTTGGGAGGTTGCTCCATCAGTTCCTAGGGGAATAAATCTTTGGGCACCTAGTGGTCATG

5 CATGACCACTAGGAGCATCTTTGGCGAAGGCATGTGTGGGACTTGAACCGAATCGATCTAGGGGAATAAATCTTTGGGCACCTAGTGGTCATG

5 CATGACCACTAGGAGCATCTTTGGCGACAAGGGTACCGTTGCGAAGTGGATAAGCGCCTAGGGGAATAAATCTTTGGGCACCTAGTGGTCATG

5 CATGACCACTAGGAGCATCTTTGGCGAGAAGACTCTGGATTCGGGGCCCAGTTGCTGCTAGGGGAATAAATCTTTGGGCACCTAGTGGTCATG

5 CATGACCACTAGGAGCATCTTTGGCGACGAGCATGTGTGTGCGAGAAGTGTTTGGTGCTAGGGGAATAAATCTTTGGGCACCTAGTGGTCATG

5 CATGACCACTAGGAGCATCTTTGGCGACCCGGTTGCGTAAGCATGCAGGAGTGGAGACTAGGGGAATAAATCTTTGGGCACCTAGTGGTCATG

5 CATGACCACTAGGAGCATCTTTGGCGAGTAGTGGATCCAAGGAACTCTTGTGCTCGCCTAGGGGAATAAATCTTTGGGCACCTAGTGGTCATG

5 CATGACCACTAGGAGCATCTTTGGCGACGGCGTGGGGACCGCAGGTTCGGCGGGTACCTAGGGGAATAAATCTTTGGGCACCTAGTGGTCATG

5 CATGACCACTAGGAGCATCTTTGGCGACGTAGGTCGTACGTAGATGACCGAAGTGGACTAGGGGAATAAATCTTTGGGCACCTAGTGGTCATG

5 CATGACCACTAGGAGCATCTTTGGCGAGCAGCTTCAAAATGGTTAGGTTGTTGTAAGCTAGGGGAATAAATCTTTGGGCACCTAGTGGTCATG

5 CATGACCACTAGGAGCATCTTTGGCGAAACATGTGACGAAGTTGCAATGAAGTGGAGCTAGGGGAATAAATCTTTGGGCACCTAGTGGTCATG

5 CATGACCACTAGGAGCATCTTTGGCGAAGCGTGTTGCAAGGTTCGCGAGTGTGGACCCTAGGGGAATAAATCTTTGGGCACCTAGTGGTCATG

5 CATGACCACTAGGAGCATCTTTGGCGAAGCGTGCTGCAAGGTCCGCGAGTGTGGACCCTAGGGGAATAAATCTTTGGGCACCTAGTGGTCATG

5 CATGACCACTAGGAGCATCTTTGGCGACTCCAGTTCATCTGTCGGGCAGTCTAAGGACTAGGGGAATAAATCTTTGGGCACCTAGTGGTCATG

5 CATGACCACTAGGAGCATCTTTGGCGACTCGGGCTGGGGGGTTGGTACCTGGGTAGACTAGGGGAATAAATCTTTGGGCACCTAGTGGTCATG

5 CATGACCACTAGGAGCATCTTTGGCGACGGCGTGGGGCCATCCGTGCGGCGGATCCCCTAGGGGAATAAATCTTTGGGCACCTAGTGGTCATG

5 CATGACCACTAGGAGCATCTTTGGCGACCCAGGCGCGGCACGCGGCCGGAAGTGGAGCTAGGGGAATAAATCTTTGGGCACCTAGTGGTCATG

5 CATGACCACTAGGAGCATCTTTGGCGAAGATCGCTCGTCGACTCGGGTCTGAGGAAGCTAGGGGAATAAATCTTTGGGCACCTAGTGGTCATG

5 CATGACCACTAGGAGCATCTTTGGCGAACGGCAGGTGTTGCGGTGGTCTGTGAAGCCCTAGGGGAATAAATCTTTGGGCACCTAGTGGTCATG

5 CATGACCACTAGGAGCATCTTTGGCGACACGACTCGCATAAGCGTGGGCGTTGTGGACTAGGGGAATAAATCTTTGGGCACCTAGTGGTCATG

5 CATGACCACTAGGAGCATCTTTGGCGAAAGTCGGTAGAACAGGGTGGGGTGTTGTCCCTAGGGGAATAAATCTTTGGGCACCTAGTGGTCATG

5 CATGACCACTAGGAGCATCTTTGGCGATTAGCGTCGTCCGGGTCCCGGCCAGGTGACCTAGGGGAATAAATCTTTGGGCACCTAGTGGTCATG

5 CATGACCACTAGGAGCATCTTTGGCGACAGACGCGGCTCGGGCGGATCCAGGGCCTTCTAGGGGAATAAATCTTTGGGCACCTAGTGGTCATG

5 CATGACCACTAGGAGCATCTTTGGCGAGCCTCGCTTGGGAGGATGCTCCACCAGTTCCTAGGGGAATAAATCTTTGGGCACCTAGTGGTCATG

5 CATGACCACTAGGAGCATCTTTGGCGAGGAACGGTTGTCACAAGAGCTTCAAACCAGCTAGGGGAATAAATCTTTGGGCACCTAGTGGTCATG

5 CATGACCACTAGGAGCATCTTTGGCGACCCATTGGGCGAATGCAAACAGTGGGTGGACTAGGGGAATAAATCTTTGGGCACCTAGTGGTCATG

5 CATGACCACTAGGAGCATCTTTGGCGAGGGAGGGCGCCGGCAGCGGTGTGGATGCGCCTAGGGGAATAAATCTTTGGGCACCTAGTGGTCATG

5 CATGACCACTAGGAGCATCTTTGGCGATGAAAGTGTCATTGAAGAGCAGACGTTTGGCTAGGGGAATAAATCTTTGGGCACCTAGTGGTCATG

5 CATGACCACTAGGAGCATCTTTGGCGACACACAAGGATGCGTACGTGTGGGTAGAGCCTAGGGGAATAAATCTTTGGGCACCTAGTGGTCATG

5 CATGACCACTAGGAGCATCTTTGGCGAATGGCCGGCACGGTCTTCTAGTCCTTGGTACTAGGGGAATAAATCTTTGGGCACCTAGTGGTCATG

5 CATGACCACTAGGAGCATCTTTGGCGAACGGTGGCATCGATGTCTAGCTCCATTTAGCTAGGGGAATAAATCTTTGGGCACCTAGTGGTCATG

5 CATGACCACTAGGAGCATCTTTGGCGACCGAGGTAGCGGGGGAAATATGCGCAGGTCCTAGGGGAATAAATCTTTGGGCACCTAGTGGTCATG

5 CATGACCACTAGGAGCATCTTTGGCGAAGGGCACGGCTAGGGTGGTAATTGTGGCGACTAGGGGAATAAATCTTTGGGCACCTAGTGGTCATG

5 CATGACCACTAGGAGCATCTTTGGCGACCCTGTAAGCTGACCAGCGACGAGGGTGGACTAGGGGAATAAATCTTTGGGCACCTAGTGGTCATG

5 CATGACCACTAGGAGCATCTTTGGCGAGATCGTGAGAATCGGTGTCATTGGTGTCTCCTAGGGGAATAAATCTTTGGGCACCTAGTGGTCATG

5 CATGACCACTAGGAGCATCTTTGGCGAGTGTGGTCAGCGTCACCGACGACACAGCGACTAGGGGAATAAATCTTTGGGCACCTAGTGGTCATG

5 CATGACCACTAGGAGCATCTTTGGCGAGTGGACTCAGTCATACGAGCAGACGTTTGGCTAGGGGAATAAATCTTTGGGCACCTAGTGGTCATG

5 CATGACCACTAGGAGCATCTTTGGCGACCCGCGCAGAGCGTAGCCTCGCGTGGTGGACTAGGGGAATAAATCTTTGGGCACCTAGTGGTCATG

5 CATGACCACTAGGAGCATCTTTGGCGATCCTCGTCAGAAGGTGAAGCAGACGTTTGGCTAGGGGAATAAATCTTTGGGCACCTAGTGGTCATG

5 CATGACCACTAGGAGCATCTTTGGCGAGGACCAAGGTGTGAGAATACGTGCAACTGCCTAGGGGAATAAATCTTTGGGCACCTAGTGGTCATG

5 CATGACCACTAGGAGCATCTTTGGCGAGCCTCGCATGGGAGGTTGCTCCACCAGTTCCTAGGGGAATAAATCTTTGGGCACCTAGTGGTCATG

5 CATGACCACTAGGAGCATCTTTGGCGAAGACTCGTGGTTGGTTGTGTGGATCGATAGCTAGGGGAATAAATCTTTGGGCACCTAGTGGTCATG

5 CATGACCACTAGGAGCATCTTTGGCGAGGGAGGGCGCCGGCAGCGGTGTGAATGAGCCTAGGGGAATAAATCTTTGGGCACCTAGTGGTCATG

5 CATGACCACTAGGAGCATCTTTGGCGAGTTGTCGTAGAGCCACGGGGCACCCGTCAGCTAGGGGAATAAATCTTTGGGCACCTAGTGGTCATG

5 CATGACCACTAGGAGCATCTTTGGCGAAAGTCGGTAGAACAGGGTAGGGTGCTGTCCCTAGGGGAATAAATCTTTGGGCACCTAGTGGTCATG

5 CATGACCACTAGGAGCATCTTTGGCGACTCCAGCTAGCTGGCCAGCGCTGGTGTGGACTAGGGGAATAAATCTTTGGGCACCTAGTGGTCATG

5 CATGACCACTAGGAGCATCTTTGGCGATATTGGCGTTAGCTAACATCGTAGGATGTTCTAGGGGAATAAATCTTTGGGCACCTAGTGGTCATG

4 CATGACCACTAGGAGCATCTTTGGCGAGCCTTGCTTGGGAGGTTGCTCCACCGGTTCCTAGGGGAATAAATCTTTGGGCACCTAGTGGTCATG

4 CATGACCACTAGGAGCATCTTTGGCGACGTGCGGGGCGGCGGCCCGGTAGGAAGGGCCTAGGGGAATAAATCTTTGGGCACCTAGTGGTCATG

4 CATGACCACTAGGAGCATCTTTGGCGATCAAGTGACTTAGCGTGCCTGGAGCGAAGGCTAGGGGAATAAATCTTTGGGCACCTAGTGGTCATG

4 CATGACCACTAGGAGCATCTTTGGCGACGGCGTGGGGCCATATGTGCGGCGGATCCCCTAGGGGAATAAATCTTTGGGCACCTAGTGGTCATG

4 CATGACCACTAGGAGCATCTTTGGCGATCCGGGGCGAGCGAACGAGGTTTTGAAGCTCTAGGGGAATAAATCTTTGGGCACCTAGTGGTCATG

4 CATGACCACTAGGAGCATCTTTGGCGAAAGTCGGTAGAACAGGCTGGGGTGCTGTCCCTAGGGGAATAAATCTTTGGGCACCTAGTGGTCATG

4 CATGACCACTAGGAGCATCTTTGGCGACGAGGGGCATGGGTGGTCATTGCGAGAAGACTAGGGGAATAAATCTTTGGGCACCTAGTGGTCATG

4 CATGACCACTAGGAGCATCTTTGGCGACTCGCATATCCGTAAGGTGGGCGTAGTGGACTAGGGGAATAAATCTTTGGGCACCTAGTGGTCATG

4 CATGACCACTAGGAGCATCTTTGGCGAGCCTCGCTTGAGAGGTTGCTCCACCAGTTCCTAGGGGAATAAATCTTTGGGCACCTAGTGGTCATG

4 CATGACCACTAGGAGCATCTTTGGCGATGGAGGTAACGCGAGGGAGCAGACGTTGGGCTAGGGGAATAAATCTTTGGGCACCTAGTGGTCATG

4 CATGACCACTAGGAGCATCTTTGGCGAGATCGGGAGAAACGGTGGCATTGGTGTATCCTAGGGGAATAAATCTTTGGGCACCTAGTGGTCATG

4 CATGACCACTAGGAGCATCTTTGGCGACCGAGACGTTGCTAAAGTCGTACAGTGGAGCTAGGGGAATAAATCTTTGGGCACCTAGTGGTCATG

4 CATGACCACTAGGAGCATCTTTGGCGACTCATAGTGCGAGCGCACGTATGTAGTGGACTAGGGGAATAAATCTTTGGGCACCTAGTGGTCATG

4 CATGACCACTAGGAGCATCTTTGGCGAGTATTGGCTGGTAGGATGCGTATTGGGGAGCTAGGGGAATAAATCTTTGGGCACCTAGTGGTCATG

4 CATGACCACTAGGAGCATCTTTGGCGAACGGAAGGTGTTGCGGTGGTCTGTGAATCACTAGGGGAATAAATCTTTGGGCACCTAGTGGTCATG

4 CATGACCACTAGGAGCATCTTTGGCGACCCAACTGCGCCTGGCGAAGGTGTGGTGGACTAGGGGAATAAATCTTTGGGCACCTAGTGGTCATG

4 CATGACCACTAGGAGCATCTTTGGCGACCCGTTTGAGGGTGAAACCAAGCGGGTGGACTAGGGGAATAAATCTTTGGGCACCTAGTGGTCATG

4 CATGACCACTAGGAGCATCTTTGGCGACAAAGGTGTTCCGCAAGTGGCACTGTGGAGCTAGGGGAATAAATCTTTGGGCACCTAGTGGTCATG

4 CATGACCACTAGGAGCATCTTTGGCGAGATCAAGAGAATCGGTGGCATTGGTGTCTCCTAGGGGAATAAATCTTTGGGCACCTAGTGGTCATG

4 CATGACCACTAGGAGCATCTTTGGCGAGGGAGGGCGCCGGCAGAGGTGTGAATGCGCCTAGGGGAATAAATCTTTGGGCACCTAGTGGTCATG

4 CATGACCACTAGGAGCATCTTTGGCGACTTAAAGTTGGCCTGCTAACGCGAAGTGGACTAGGGGAATAAATCTTTGGGCACCTAGTGGTCATG

4 CATGACCACTAGGAGCATCTTTGGCGACGAAGGAACGGTAAGGACTGATTGTGGAGACTAGGGGAATAAATCTTTGGGCACCTAGTGGTCATG

4 CATGACCACTAGGAGCATCTTTGGCGACCCAGGAGCCTCGTAGAACGACTGGGTGGACTAGGGGAATAAATCTTTGGGCACCTAGTGGTCATG

4 CATGACCACTAGGAGCATCTTTGGCGAGATCGGGAGAATCGGTGGCATAGTTGTCTCCTAGGGGAATAAATCTTTGGGCACCTAGTGGTCATG

4 CATGACCACTAGGAGCATCTTTGGCGAGATCGGGAGAATCGTAGGCATTGGTGTCTCCTAGGGGAATAAATCTTTGGGCACCTAGTGGTCATG

4 CATGACCACTAGGAGCATCTTTGGCGACCCCTGTCGCTGGACATGCGACGGAGTGGACTAGGGGAATAAATCTTTGGGCACCTAGTGGTCATG

4 CATGACCACTAGGAGCATCTTTGGCGAGTGTTGCCCGGCACACGAGCAGACGTTTGGCTAGGGGAATAAATCTTTGGGCACCTAGTGGTCATG

4 CATGACCACTAGGAGCATCTTTGGCGAGGTGTAAACTTCGCGTACGGTGGCCGAGCTCTAGGGGAATAAATCTTTGGGCACCTAGTGGTCATG

4 CATGACCACTAGGAGCATCTTTGGCGAGGTGGGTGGAACCCTTGTTAAGGGCGCGCCCTAGGGGAATAAATCTTTGGGCACCTAGTGGTCATG

4 CATGACCACTAGGAGCATCTTTGGCGATTAGCGTCTGCGAGCGGGTGGGTTCGCTCCCTAGGGGAATAAATCTTTGGGCACCTAGTGGTCATG

4 CATGACCACTAGGAGCATCTTTGGCGACCCATCACCAGGCATCTGCGTATGGGTGGACTAGGGGAATAAATCTTTGGGCACCTAGTGGTCATG

4 CATGACCACTAGGAGCATCTTTGGCGAGATCGGGAGAATCTGTGTCATTGGTGTCTCCTAGGGGAATAAATCTTTGGGCACCTAGTGGTCATG

4 CATGACCACTAGGAGCATCTTTGGCGAAGTCACAAGAGTAACAATGAGCATATTCGCCTAGGGGAATAAATCTTTGGGCACCTAGTGGTCATG

4 CATGACCACTAGGAGCATCTTTGGCGACTGAACACCGGCGCTGTCCGGGGCAGTGGACTAGGGGAATAAATCTTTGGGCACCTAGTGGTCATG

4 CATGACCACTAGGAGCATCTTTGGCGACCAGCTAAGCGTTGTAGGAATCCTTGCTAGCTAGGGGAATAAATCTTTGGGCACCTAGTGGTCATG

4 CATGACCACTAGGAGCATCTTTGGCGAACGGCAGGTGTTGCGGTGGTTTGTGTATCCCTAGGGGAATAAATCTTTGGGCACCTAGTGGTCATG

4 CATGACCACTAGGAGCATCTTTGGCGACAGGGGATGCCGATGCGCTGGACGGCCGAGCTAGGGGAATAAATCTTTGGGCACCTAGTGGTCATG

4 CATGACCACTAGGAGCATCTTTGGCGAAGGCGAAGCACGCTGGTCCTGGTGGCGTCCCTAGGGGAATAAATCTTTGGGCACCTAGTGGTCATG

4 CATGACCACTAGGAGCATCTTTGGCGAGACTATGTCGGCAGGGGGTTGGCGCCGTCGCTAGGGGAATAAATCTTTGGGCACCTAGTGGTCATG

4 CATGACCACTAGGAGCATCTTTGGCGATATCGGGAGAATCGGTGGCATTTGTGTCTCCTAGGGGAATAAATCTTTGGGCACCTAGTGGTCATG

4 CATGACCACTAGGAGCATCTTTGGCGACGTTGACAAGCCATGTGGCTGAGTCGGAGACTAGGGGAATAAATCTTTGGGCACCTAGTGGTCATG

4 CATGACCACTAGGAGCATCTTTGGCGACTGCGTGGGGCCATCTGTGCGGCGGATCCCCTAGGGGAATAAATCTTTGGGCACCTAGTGGTCATG

4 CATGACCACTAGGAGCATCTTTGGCGACAGGGGATGCCGATGCGCAGGACGACCGAGCTAGGGGAATAAATCTTTGGGCACCTAGTGGTCATG

4 CATGACCACTAGGAGCATCTTTGGCGAACACTGGAGGAAGCAGTGGCAGAAGTGGAGCTAGGGGAATAAATCTTTGGGCACCTAGTGGTCATG

4 CATGACCACTAGGAGCATCTTTGGCGAACGCAGCGCGGCCGTAAGGTGCAAGTGGAGCTAGGGGAATAAATCTTTGGGCACCTAGTGGTCATG

4 CATGACCACTAGGAGCATCTTTGGCGACGTACAACGGCAGGCATTGTGTCTCGGTAGCTAGGGGAATAAATCTTTGGGCACCTAGTGGTCATG

4 CATGACCACTAGGAGCATCTTTGGCGACGGTGTGGGGAACTTGTTTCGGCGGTGCCACTAGGGGAATAAATCTTTGGGCACCTAGTGGTCATG

4 CATGACCACTAGGAGCATCTTTGGCGAATGGCCGGTGTTGCGGTGGTCTGTGAATCCCTAGGGGAATAAATCTTTGGGCACCTAGTGGTCATG

4 CATGACCACTAGGAGCATCTTTGGCGACCCTCGAGACGTAGTTGTTCGCAGGGTGGACTAGGGGAATAAATCTTTGGGCACCTAGTGGTCATG

4 CATGACCACTAGGAGCATCTTTGGCGAGGGCACGCAGGATGCGAGAGTGGTCGAGTACTAGGGGAATAAATCTTTGGGCACCTAGTGGTCATG

4 CATGACCACTAGGAGCATCTTTGGCGAACCGGGTCGTCCGAAGACCCAGAGGTGGAGCTAGGGGAATAAATCTTTGGGCACCTAGTGGTCATG

4 CATGACCACTAGGAGCATCTTTGGCGACGCGGTGTGTGGATAGGGTATGGTACGCGACTAGGGGAATAAATCTTTGGGCACCTAGTGGTCATG

4 CATGACCACTAGGAGCATCTTTGGCGAGGTTGTTACTAACGCTGAGCAGACGTTTGGCTAGGGGAATAAATCTTTGGGCACCTAGTGGTCATG

4 CATGACCACTAGGAGCATCTTTGGCGAACGTGCAGGATATGCCGCCTGGAGCGTAGGCTAGGGGAATAAATCTTTGGGCACCTAGTGGTCATG

4 CATGACCACTAGGAGCATCTTTGGCGACGTTGGCGGCCTAAGTGGACTTAACGCTGACTAGGGGAATAAATCTTTGGGCACCTAGTGGTCATG

4 CATGACCACTAGGAGCATCTTTGGCGAGAAGACTCTGGATTCGGGGACCAGTTGCGACTAGGGGAATAAATCTTTGGGCACCTAGTGGTCATG

4 CATGACCACTAGGAGCATCTTTGGCGACGGCGTGGGGCTATCGATGCGGCGGCACTCCTAGGGGAATAAATCTTTGGGCACCTAGTGGTCATG

4 CATGACCACTAGGAGCATCTTTGGCGACCCAGTCTGCCGAATGGCAGCAAGGGTGGACTAGGGGAATAAATCTTTGGGCACCTAGTGGTCATG

4 CATGACCACTAGGAGCATCTTTGGCGATCCTCGTCAGATGGTGAAGCAGACGTATGGCTAGGGGAATAAATCTTTGGGCACCTAGTGGTCATG

4 CATGACCACTAGGAGCATCTTTGGCGACCCAACATGAGGTACTCCTGTTGCGGTGGACTAGGGGAATAAATCTTTGGGCACCTAGTGGTCATG

4 CATGACCACTAGGAGCATCTTTGGCGATGGGGCCGTGCTAACTGCACACTCCGCGGGCTAGGGGAATAAATCTTTGGGCACCTAGTGGTCATG

4 CATGACCACTAGGAGCATCTTTGGCGACAGGGGATGCCGATGCGCTGGACGACCAAGCTAGGGGAATAAATCTTTGGGCACCTAGTGGTCATG

4 CATGACCACTAGGAGCATCTTTGGCGATCCTAGTCAGATGGTGAAGCAGACGTTTGGCTAGGGGAATAAATCTTTGGGCACCTAGTGGTCATG

4 CATGACCACTAGGAGCATCTTTGGCGACGGCGTGGGGCCATCTGTGCGGCGGTTCCCCTAGGGGAATAAATCTTTGGGCACCTAGTGGTCATG

4 CATGACCACTAGGAGCATCTTTGGCGACGACAAGGATCGTTGATCAAAATTAATCGCCTAGGGGAATAAATCTTTGGGCACCTAGTGGTCATG

4 CATGACCACTAGGAGCATCTTTGGCGAATGGTGTAGGACTTCAAGTGGATCTCATAGCTAGGGGAATAAATCTTTGGGCACCTAGTGGTCATG

4 CATGACCACTAGGAGCATCTTTGGCGACCCGTCGTATAAGGGACATACACGGGTGGACTAGGGGAATAAATCTTTGGGCACCTAGTGGTCATG

4 CATGACCACTAGGAGCATCTTTGGCGACTACCGGAGCGAAGTACTGTGGCTGAGTACCTAGGGGAATAAATCTTTGGGCACCTAGTGGTCATG

4 CATGACCACTAGGAGCATCTTTGGCGATGGGGCCGTGCAATCTGCACACTCCGCGGGCTAGGGGAATAAATCTTTGGGCACCTAGTGGTCATG

4 CATGACCACTAGGAGCATCTTTGGCGAGTGGTCGGGTTGGGAGGGCGTGGCTATCGGCTAGGGGAATAAATCTTTGGGCACCTAGTGGTCATG

4 CATGACCACTAGGAGCATCTTTGGCGAGATGGTGTACACTGTCGAGCAGACGTTGGGCTAGGGGAATAAATCTTTGGGCACCTAGTGGTCATG

4 CATGACCACTAGGAGCATCTTTGGCGACATGGGCGACTTAAGAGAGGAGAAGTCGAACTAGGGGAATAAATCTTTGGGCACCTAGTGGTCATG

4 CATGACCACTAGGAGCATCTTTGGCGACGGTGTGGGTGCAGGGTCAGGCGGTTGATCCTAGGGGAATAAATCTTTGGGCACCTAGTGGTCATG

4 CATGACCACTAGGAGCATCTTTGGCGAACGGCAGGTGTTGCGGTGGTTTGTGAAACCCTAGGGGAATAAATCTTTGGGCACCTAGTGGTCATG

4 CATGACCACTAGGAGCATCTTTGGCGAGAGGAGCACTCGCTCGTCACGAAGTGGAGCCTAGGGGAATAAATCTTTGGGCACCTAGTGGTCATG

4 CATGACCACTAGGAGCATCTTTGGCGACCCAGGCGTACACGCGTGGCACTGGGTGGACTAGGGGAATAAATCTTTGGGCACCTAGTGGTCATG

4 CATGACCACTAGGAGCATCTTTGGCGAAAGCGCAGGGTATGCGACCTCCAATTGGTCCTAGGGGAATAAATCTTTGGGCACCTAGTGGTCATG

4 CATGACCACTAGGAGCATCTTTGGCGAAACTGCGCATGAAGGTGCGAAGAAGTGGAGCTAGGGGAATAAATCTTTGGGCACCTAGTGGTCATG

4 CATGACCACTAGGAGCATCTTTGGCGAGCCTCGTCAGATGGTGAAGCAGACGTTTGGCTAGGGGAATAAATCTTTGGGCACCTAGTGGTCATG

4 CATGACCACTAGGAGCATCTTTGGCGAGGATCAAGGTGTGACTCACGAAGTGCTCTGCTAGGGGAATAAATCTTTGGGCACCTAGTGGTCATG

4 CATGACCACTAGGAGCATCTTTGGCGACACGGAGTGGAATCAAGGGGTAAGAAGCCACTAGGGGAATAAATCTTTGGGCACCTAGTGGTCATG

4 CATGACCACTAGGAGCATCTTTGGCGAGTGGATTCAATCATACGAGCAGACGTTTGGCTAGGGGAATAAATCTTTGGGCACCTAGTGGTCATG

4 CATGACCACTAGGAGCATCTTTGGCGACACGGTTGGTCAGCGGCCAAACGTTGTGGACTAGGGGAATAAATCTTTGGGCACCTAGTGGTCATG

4 CATGACCACTAGGAGCATCTTTGGCGAAACTCCCGGCAGTGCAGAACAAAGTTGCGACTAGGGGAATAAATCTTTGGGCACCTAGTGGTCATG

4 CATGACCACTAGGAGCATCTTTGGCGACCCGAGCAGTCAGAGTTCGCTCGTGGTGGACTAGGGGAATAAATCTTTGGGCACCTAGTGGTCATG

4 CATGACCACTAGGAGCATCTTTGGCGATAGGCGTGTGCGGTCGAGCGAAAGCGATAGCTAGGGGAATAAATCTTTGGGCACCTAGTGGTCATG

4 CATGACCACTAGGAGCATCTTTGGCGAGGGGTGGGGGGCCGCTCGCGCCTGCGTGCGCTAGGGGAATAAATCTTTGGGCACCTAGTGGTCATG

4 CATGACCACTAGGAGCATCTTTGGCGAGGGCGGGCGCCGGCAGCGGTGTGAATGCGCCTAGGGGAATAAATCTTTGGGCACCTAGTGGTCATG

4 CATGACCACTAGGAGCATCTTTGGCGAAGAATCGCAGATGGGACCCGTCGGGGTTACCTAGGGGAATAAATCTTTGGGCACCTAGTGGTCATG

4 CATGACCACTAGGAGCATCTTTGGCGAGGGGTCATGGGACTGTAATTGCGCTTAAGACTAGGGGAATAAATCTTTGGGCACCTAGTGGTCATG

4 CATGACCACTAGGAGCATCTTTGGCGAGATGACTCTGGATTCGGGGACCAGTTGCTGCTAGGGGAATAAATCTTTGGGCACCTAGTGGTCATG

4 CATGACCACTAGGAGCATCTTTGGCGAATGGTCGGCACGGCCTTCTAGTCCTTGGTACTAGGGGAATAAATCTTTGGGCACCTAGTGGTCATG

4 CATGACCACTAGGAGCATCTTTGGCGAATGGCCGGCGCGGCCTTCTAGTCCTTGGTACTAGGGGAATAAATCTTTGGGCACCTAGTGGTCATG

4 CATGACCACTAGGAGCATCTTTGGCGAAAGTCCGTAGAACAGGGTGGGGTGCTGTCCCTAGGGGAATAAATCTTTGGGCACCTAGTGGTCATG

4 CATGACCACTAGGAGCATCTTTGGCGAGTTCGTAAGGCCGCGCTCCTTGTGGCCGTGCTAGGGGAATAAATCTTTGGGCACCTAGTGGTCATG

4 CATGACCACTAGGAGCATCTTTGGCGACAGGGGATGCCGGTGCGCTGGACGACCGAGCTAGGGGAATAAATCTTTGGGCACCTAGTGGTCATG

4 CATGACCACTAGGAGCATCTTTGGCGAGATCGGGAGAATCGTTGTCATTGGTGTCTCCTAGGGGAATAAATCTTTGGGCACCTAGTGGTCATG

4 CATGACCACTAGGAGCATCTTTGGCGAATGGCCGGCACGGCCGTCTAGTCCTCGGTACTAGGGGAATAAATCTTTGGGCACCTAGTGGTCATG

4 CATGACCACTAGGAGCATCTTTGGCGAAGGGCGCTAGTGGTTCGGTGGTTGCCCCCGCTAGGGGAATAAATCTTTGGGCACCTAGTGGTCATG

4 CATGACCACTAGGAGCATCTTTGGCGATCGAAGTAATCGCGCAGGGGCTTGTGGTTCCTAGGGGAATAAATCTTTGGGCACCTAGTGGTCATG

4 CATGACCACTAGGAGCATCTTTGGCGAACGGCCGGCACGGCCTCCTAGTCCTCGGTACTAGGGGAATAAATCTTTGGGCACCTAGTGGTCATG

4 CATGACCACTAGGAGCATCTTTGGCGACAGCCCGGTGTGGGTGCGCAAGCTATATGCCTAGGGGAATAAATCTTTGGGCACCTAGTGGTCATG

4 CATGACCACTAGGAGCATCTTTGGCGACCCAGTTGTCCTGTACAGGAAATGGGTGGACTAGGGGAATAAATCTTTGGGCACCTAGTGGTCATG

4 CATGACCACTAGGAGCATCTTTGGCGAGGGTGGGAGTTGTGGCAGTATGTGTGGCGCCTAGGGGAATAAATCTTTGGGCACCTAGTGGTCATG

4 CATGACCACTAGGAGCATCTTTGGCGATATAGGGAGAATCGGTGGCATTGGTGTCTCCTAGGGGAATAAATCTTTGGGCACCTAGTGGTCATG

4 CATGACCACTAGGAGCATCTTTGGCGACAGCGTTTGCGGGCCCGACATAGTGGGACCCTAGGGGAATAAATCTTTGGGCACCTAGTGGTCATG

4 CATGACCACTAGGAGCATCTTTGGCGAGTAGTGGCTGAGGTATGTTCACGTACGAAACTAGGGGAATAAATCTTTGGGCACCTAGTGGTCATG

4 CATGACCACTAGGAGCATCTTTGGCGAAAGTCGGTAGAACAGGGTGGGGTGCTCTCCCTAGGGGAATAAATCTTTGGGCACCTAGTGGTCATG

4 CATGACCACTAGGAGCATCTTTGGCGACAGGGGCTGGTATGGAGTACTCGGGCAAGCCTAGGGGAATAAATCTTTGGGCACCTAGTGGTCATG

4 CATGACCACTAGGAGCATCTTTGGCGAACGGCAGGTGATGCGGTGGTCTGTGAATCACTAGGGGAATAAATCTTTGGGCACCTAGTGGTCATG

4 CATGACCACTAGGAGCATCTTTGGCGACAGGGGATGCCGATGCGCTGGACAACCGAGCTAGGGGAATAAATCTTTGGGCACCTAGTGGTCATG

4 CATGACCACTAGGAGCATCTTTGGCGATGGGGCCGTGCTATCTGCACACTTTGCGGGCTAGGGGAATAAATCTTTGGGCACCTAGTGGTCATG

4 CATGACCACTAGGAGCATCTTTGGCGAACCGTGGTGCCGTGCACGACGTGGTGGAGACTAGGGGAATAAATCTTTGGGCACCTAGTGGTCATG

4 CATGACCACTAGGAGCATCTTTGGCGACATAGGGGGCATGCCGGGAAGGAGAGGCGCCTAGGGGAATAAATCTTTGGGCACCTAGTGGTCATG

4 CATGACCACTAGGAGCATCTTTGGCGATGGGGCCGTGCTATCTGCGCACTCCGCGGGCTAGGGGAATAAATCTTTGGGCACCTAGTGGTCATG

4 CATGACCACTAGGAGCATCTTTGGCGATCCCTCTGCGTAAGCGCTGAGGTGTGGAGACTAGGGGAATAAATCTTTGGGCACCTAGTGGTCATG

4 CATGACCACTAGGAGCATCTTTGGCGAATCGGCGGCAAGTCGTTGAAGGATGTCAGTCTAGGGGAATAAATCTTTGGGCACCTAGTGGTCATG

4 CATGACCACTAGGAGCATCTTTGGCGATCCTCGTCAGATGGTGAAGCAGACGTTTGTCTAGGGGAATAAATCTTTGGGCACCTAGTGGTCATG

4 CATGACCACTAGGAGCATCTTTGGCGAACGGCAGGTGTCGCGGTGGTCTGCGAATCCCTAGGGGAATAAATCTTTGGGCACCTAGTGGTCATG

4 CATGACCACTAGGAGCATCTTTGGCGACGGCGTGGGGCTACTGATGCGGCGGCACTCCTAGGGGAATAAATCTTTGGGCACCTAGTGGTCATG

4 CATGACCACTAGGAGCATCTTTGGCGACATCCGTTGATGATTCAGCAATGGTGTGGACTAGGGGAATAAATCTTTGGGCACCTAGTGGTCATG

4 CATGACCACTAGGAGCATCTTTGGCGACAGGACTAGCATAGAACAGCGAAGTGGAGACTAGGGGAATAAATCTTTGGGCACCTAGTGGTCATG

4 CATGACCACTAGGAGCATCTTTGGCGACGTAGGTCGGAAGGGTCGCGCCGCGGCACACTAGGGGAATAAATCTTTGGGCACCTAGTGGTCATG

4 CATGACCACTAGGAGCATCTTTGGCGAATGGCCGGCACGGCCTTCTAGTTCTCGGTACTAGGGGAATAAATCTTTGGGCACCTAGTGGTCATG

4 CATGACCACTAGGAGCATCTTTGGCGACCCGTCTATAGGTGTAAACCTGCGGGTGGACTAGGGGAATAAATCTTTGGGCACCTAGTGGTCATG

4 CATGACCACTAGGAGCATCTTTGGCGAGCCTCGCTTGGGAGGGTGCTCCACCAGTTCCTAGGGGAATAAATCTTTGGGCACCTAGTGGTCATG

4 CATGACCACTAGGAGCATCTTTGGCGAAATACAACGGCGGCATTGATTGTCAATCGACTAGGGGAATAAATCTTTGGGCACCTAGTGGTCATG

4 CATGACCACTAGGAGCATCTTTGGCGAGCCCGAACAACGTAGTGGGCGGTGTGGAGACTAGGGGAATAAATCTTTGGGCACCTAGTGGTCATG

4 CATGACCACTAGGAGCATCTTTGGCGACGGAGTGGGGCTCATTATGCGGCGGTAATCCTAGGGGAATAAATCTTTGGGCACCTAGTGGTCATG

4 CATGACCACTAGGAGCATCTTTGGCGACAGAGGGTGCGGGGCAGGACCAGTTGAAAGCTAGGGGAATAAATCTTTGGGCACCTAGTGGTCATG

4 CATGACCACTAGGAGCATCTTTGGCGAGCCTTGCTTGGGAGGTTGCTCCACCAGTTACTAGGGGAATAAATCTTTGGGCACCTAGTGGTCATG

4 CATGACCACTAGGAGCATCTTTGGCGAGCAAGCGCGGTCCCCCGGACCTCACAGTTGCTAGGGGAATAAATCTTTGGGCACCTAGTGGTCATG

4 CATGACCACTAGGAGCATCTTTGGCGACCCAGGGCGCGAAGAGCAAGGCGTGGAAGACTAGGGGAATAAATCTTTGGGCACCTAGTGGTCATG

4 CATGACCACTAGGAGCATCTTTGGCGACGTGGCTGGGAAAGGGTTCGGGTGTGGAGACTAGGGGAATAAATCTTTGGGCACCTAGTGGTCATG

4 CATGACCACTAGGAGCATCTTTGGCGAAGGGTGTAGGACTTCAAGTGGATCTCACAGCTAGGGGAATAAATCTTTGGGCACCTAGTGGTCATG

4 CATGACCACTAGGAGCATCTTTGGCGACCTTGGTCGGTAGGCTGGTGTTAGTGGAGCCTAGGGGAATAAATCTTTGGGCACCTAGTGGTCATG

4 CATGACCACTAGGAGCATCTTTGGCGAGGTTACTCCCGAAGGTGGTGTGGTCGAGAACTAGGGGAATAAATCTTTGGGCACCTAGTGGTCATG

4 CATGACCACTAGGAGCATCTTTGGCGAGGGAGGGCGCCGGCGGCGGTGTGAATGCGCCTAGGGGAATAAATCTTTGGGCACCTAGTGGTCATG

4 CATGACCACTAGGAGCATCTTTGGCGACGTAGTGGGCTGGTAGGGAGTACTTGTTGCCTAGGGGAATAAATCTTTGGGCACCTAGTGGTCATG

4 CATGACCACTAGGAGCATCTTTGGCGATCCGGGGCGACCGTACGGAAGGGCTCGTAGCTAGGGGAATAAATCTTTGGGCACCTAGTGGTCATG

4 CATGACCACTAGGAGCATCTTTGGCGAGCAGCGCAGACTGGTTGATTAGGTTTGCTCCTAGGGGAATAAATCTTTGGGCACCTAGTGGTCATG

4 CATGACCACTAGGAGCATCTTTGGCGAGATCGTGAGAATCGGAGGCATTGGTGTCTCCTAGGGGAATAAATCTTTGGGCACCTAGTGGTCATG

4 CATGACCACTAGGAGCATCTTTGGCGAGCCTCGCTTGGGAGGTTGCTCCACCAGGTCCTAGGGGAATAAATCTTTGGGCACCTAGTGGTCATG

4 CATGACCACTAGGAGCATCTTTGGCGATGGGTGCAGACGGCCGCCGCTGCGGCTACACTAGGGGAATAAATCTTTGGGCACCTAGTGGTCATG

4 CATGACCACTAGGAGCATCTTTGGCGAGTAGTAGCATCCTATTTGTCGGTTGTTTCCCTAGGGGAATAAATCTTTGGGCACCTAGTGGTCATG

4 CATGACCACTAGGAGCATCTTTGGCGACGACAGTGGTGAGTGGCCTCCGGGAGGAGACTAGGGGAATAAATCTTTGGGCACCTAGTGGTCATG

4 CATGACCACTAGGAGCATCTTTGGCGACTCAGTGGTAGGACCACCCGGAATGGTTGTCTAGGGGAATAAATCTTTGGGCACCTAGTGGTCATG

4 CATGACCACTAGGAGCATCTTTGGCGATACCGGGCGCGAAGCGCGCGTGGTGGAAGACTAGGGGAATAAATCTTTGGGCACCTAGTGGTCATG

4 CATGACCACTAGGAGCATCTTTGGCGAAGTGCGGCGTGCCGAGTCGCCCTGCGACTCCTAGGGGAATAAATCTTTGGGCACCTAGTGGTCATG

4 CATGACCACTAGGAGCATCTTTGGCGACGAGTCCTGGACCTGTCCCCGAGGGGAGATCTAGGGGAATAAATCTTTGGGCACCTAGTGGTCATG

4 CATGACCACTAGGAGCATCTTTGGCGATCCTCATCAGATGGTGAAGCAGACGTTTGGCTAGGGGAATAAATCTTTGGGCACCTAGTGGTCATG

4 CATGACCACTAGGAGCATCTTTGGCGATCCTCGTCAGATGGTAAAGCAGACGTTTGGCTAGGGGAATAAATCTTTGGGCACCTAGTGGTCATG

4 CATGACCACTAGGAGCATCTTTGGCGAAGGGTGTAGGACTTCAAGTGGATCTTATAGCTAGGGGAATAAATCTTTGGGCACCTAGTGGTCATG

4 CATGACCACTAGGAGCATCTTTGGCGAGGGGTAGGGGGCCGCTAGCGCCTGCGTGCGCTAGGGGAATAAATCTTTGGGCACCTAGTGGTCATG

4 CATGACCACTAGGAGCATCTTTGGCGACTTGCACGGGCCAGAGACCGTCGAAGTGGACTAGGGGAATAAATCTTTGGGCACCTAGTGGTCATG

4 CATGACCACTAGGAGCATCTTTGGCGACCCGGAATTCAGCATGCTATACCGGGTGGACTAGGGGAATAAATCTTTGGGCACCTAGTGGTCATG

4 CATGACCACTAGGAGCATCTTTGGCGATTGAGCTGGCAGTGGCCCGGGGGTTTGCGCCTAGGGGAATAAATCTTTGGGCACCTAGTGGTCATG

4 CATGACCACTAGGAGCATCTTTGGCGACCCGATAGAAGGGACTGCTAGCAGGGTGGACTAGGGGAATAAATCTTTGGGCACCTAGTGGTCATG

4 CATGACCACTAGGAGCATCTTTGGCGACCCGGACTGCGCAGTAGAACGGATGTGGAGCTAGGGGAATAAATCTTTGGGCACCTAGTGGTCATG

4 CATGACCACTAGGAGCATCTTTGGCGACGAAAGAGTTGCGGGTATGGGGAGTGTCGACTAGGGGAATAAATCTTTGGGCACCTAGTGGTCATG

4 CATGACCACTAGGAGCATCTTTGGCGAGATCGGGAGAATCTGTGGAATTGGTGTCTCCTAGGGGAATAAATCTTTGGGCACCTAGTGGTCATG

4 CATGACCACTAGGAGCATCTTTGGCGATCATCGAAGTGGACGAAGCTAAACAAGCGCCTAGGGGAATAAATCTTTGGGCACCTAGTGGTCATG

4 CATGACCACTAGGAGCATCTTTGGCGACCCTAACTACGATCAGGAGTGTTGGGTGGACTAGGGGAATAAATCTTTGGGCACCTAGTGGTCATG

4 CATGACCACTAGGAGCATCTTTGGCGACCCGGGCAGCGAAGCTAAGACGCGGGTGGACTAGGGGAATAAATCTTTGGGCACCTAGTGGTCATG

4 CATGACCACTAGGAGCATCTTTGGCGACTGAGCTCAGAGAGTCACGTGCGAAGTGGACTAGGGGAATAAATCTTTGGGCACCTAGTGGTCATG

4 CATGACCACTAGGAGCATCTTTGGCGAACGCGAGGTTGGTTACGATGTGGCTGAGGCCTAGGGGAATAAATCTTTGGGCACCTAGTGGTCATG

4 CATGACCACTAGGAGCATCTTTGGCGAGAAGACTCTGGATTCGGGCACCAGTTGCTGCTAGGGGAATAAATCTTTGGGCACCTAGTGGTCATG

4 CATGACCACTAGGAGCATCTTTGGCGACCCAGCGAGTGTGCACGATAGGTGGGTGGACTAGGGGAATAAATCTTTGGGCACCTAGTGGTCATG

4 CATGACCACTAGGAGCATCTTTGGCGAATGGCCGGCACGGCATTCTAGTCCTTGGTACTAGGGGAATAAATCTTTGGGCACCTAGTGGTCATG

4 CATGACCACTAGGAGCATCTTTGGCGATGGGTGCTGGCGGCCGCCGCTGCGGCTACACTAGGGGAATAAATCTTTGGGCACCTAGTGGTCATG

4 CATGACCACTAGGAGCATCTTTGGCGAAACCAGGGCGGACGCCCATGTTAGTGGAGACTAGGGGAATAAATCTTTGGGCACCTAGTGGTCATG

4 CATGACCACTAGGAGCATCTTTGGCGACGCTCCTGCGCGTTGTTGGACGTTCTGCTACTAGGGGAATAAATCTTTGGGCACCTAGTGGTCATG

4 CATGACCACTAGGAGCATCTTTGGCGATCATGGATCGCATCAGCGCTCGGGTGGCGTCTAGGGGAATAAATCTTTGGGCACCTAGTGGTCATG

4 CATGACCACTAGGAGCATCTTTGGCGAGCATTGCTTGGGAGGTTGCTCCACCAGTTCCTAGGGGAATAAATCTTTGGGCACCTAGTGGTCATG

4 CATGACCACTAGGAGCATCTTTGGCGACAGCTCTGGGTGAGACCCGTACTGTGGGTCCTAGGGGAATAAATCTTTGGGCACCTAGTGGTCATG

4 CATGACCACTAGGAGCATCTTTGGCGAGAGCTTTGGTTGGTAGGCAGTATTGCGACGCTAGGGGAATAAATCTTTGGGCACCTAGTGGTCATG

4 CATGACCACTAGGAGCATCTTTGGCGAGATTGTAGCTCGCGCGTAGGGTCGGCAGTGCTAGGGGAATAAATCTTTGGGCACCTAGTGGTCATG

4 CATGACCACTAGGAGCATCTTTGGCGAACGGCAGGTGTTGCGGTGGTTTGTGAATCACTAGGGGAATAAATCTTTGGGCACCTAGTGGTCATG

4 CATGACCACTAGGAGCATCTTTGGCGAGGACCGGTAAACTAGCGTTAGCGTTGCGCTCTAGGGGAATAAATCTTTGGGCACCTAGTGGTCATG

4 CATGACCACTAGGAGCATCTTTGGCGAGCCTCGCTTGGTAGGTTGCTCCACCAGTTCCTAGGGGAATAAATCTTTGGGCACCTAGTGGTCATG

4 CATGACCACTAGGAGCATCTTTGGCGATGGGTGCTGACGGGGCTAGTGGGCCCTACACTAGGGGAATAAATCTTTGGGCACCTAGTGGTCATG

4 CATGACCACTAGGAGCATCTTTGGCGACGGTGTGGGGCTCCATGATGCGGCGGATTCCTAGGGGAATAAATCTTTGGGCACCTAGTGGTCATG

4 CATGACCACTAGGAGCATCTTTGGCGAGATCGGGAGAATCGGCGGCATTGGTGTCTACTAGGGGAATAAATCTTTGGGCACCTAGTGGTCATG

4 CATGACCACTAGGAGCATCTTTGGCGACGTAGGTAAGTAAAGATGTAAGTGTCGCCGCTAGGGGAATAAATCTTTGGGCACCTAGTGGTCATG

4 CATGACCACTAGGAGCATCTTTGGCGAGATCGGGAGAAACGGAGGCATTGGTGTCTCCTAGGGGAATAAATCTTTGGGCACCTAGTGGTCATG

4 CATGACCACTAGGAGCATCTTTGGCGACCCAGCGCAGGTACCTCGCAAATGGGTGGACTAGGGGAATAAATCTTTGGGCACCTAGTGGTCATG

4 CATGACCACTAGGAGCATCTTTGGCGAAGGGTGTAGGACTTCAAGTGGATCTCATATCTAGGGGAATAAATCTTTGGGCACCTAGTGGTCATG

4 CATGACCACTAGGAGCATCTTTGGCGATGGGTGCTGACGGCCGCCGCTGCGGCTATACTAGGGGAATAAATCTTTGGGCACCTAGTGGTCATG

4 CATGACCACTAGGAGCATCTTTGGCGAGATCGGGAGAATCTGTGGCATTGGAGTCTCCTAGGGGAATAAATCTTTGGGCACCTAGTGGTCATG

4 CATGACCACTAGGAGCATCTTTGGCGATCACGGGGCGAGCACAGAGAGGGTTTGCAGCTAGGGGAATAAATCTTTGGGCACCTAGTGGTCATG

4 CATGACCACTAGGAGCATCTTTGGCGAGGTATTTACTCTGTATGGGCTGGAAGATAGCTAGGGGAATAAATCTTTGGGCACCTAGTGGTCATG

4 CATGACCACTAGGAGCATCTTTGGCGATATCGGCGTAGCCCATCCTGTCAGGGTATGCTAGGGGAATAAATCTTTGGGCACCTAGTGGTCATG

4 CATGACCACTAGGAGCATCTTTGGCGATGGTAGCAGTGGGCGTAGGGTCTGGTCAGCCTAGGGGAATAAATCTTTGGGCACCTAGTGGTCATG

4 CATGACCACTAGGAGCATCTTTGGCGAGTGGCGCTCTGGGTGAGACCTTCATTGGTCCTAGGGGAATAAATCTTTGGGCACCTAGTGGTCATG

4 CATGACCACTAGGAGCATCTTTGGCGACGGTGTGGGGACCGCAGGTTCGGCGGGTATCTAGGGGAATAAATCTTTGGGCACCTAGTGGTCATG

4 CATGACCACTAGGAGCATCTTTGGCGAAGATCTGATGGGCTTGTGACCCGCCTCGGCCTAGGGGAATAAATCTTTGGGCACCTAGTGGTCATG

4 CATGACCACTAGGAGCATCTTTGGCGATAGGGGATGCCGATGCGCTGGACGACCGAGCTAGGGGAATAAATCTTTGGGCACCTAGTGGTCATG

4 CATGACCACTAGGAGCATCTTTGGCGAGATCGGGAGAATCGGTTGAATTGGTGTCTCCTAGGGGAATAAATCTTTGGGCACCTAGTGGTCATG

4 CATGACCACTAGGAGCATCTTTGGCGAAGCGTGTTGCAAGGTCCGCGAGTGCGGACCCTAGGGGAATAAATCTTTGGGCACCTAGTGGTCATG

4 CATGACCACTAGGAGCATCTTTGGCGAATGGCCGGCAGGGCCTTCTAGTCCTCGGTACTAGGGGAATAAATCTTTGGGCACCTAGTGGTCATG

4 CATGACCACTAGGAGCATCTTTGGCGACGCGGTTTACGGAGACGGAACCGACGTGGACTAGGGGAATAAATCTTTGGGCACCTAGTGGTCATG

4 CATGACCACTAGGAGCATCTTTGGCGAGGGAGGGCGCCGGCAGCGGTGTGAATTCGCCTAGGGGAATAAATCTTTGGGCACCTAGTGGTCATG

4 CATGACCACTAGGAGCATCTTTGGCGATCGGTCGGTAGGAGACTCGGTACTGAAGCACTAGGGGAATAAATCTTTGGGCACCTAGTGGTCATG

4 CATGACCACTAGGAGCATCTTTGGCGAAGCTTGGTTAGGCGACGGGCTTGGGCCACTCTAGGGGAATAAATCTTTGGGCACCTAGTGGTCATG

4 CATGACCACTAGGAGCATCTTTGGCGACCCACCCGGGAGAAACGCGGGTGAGGTGGACTAGGGGAATAAATCTTTGGGCACCTAGTGGTCATG

4 CATGACCACTAGGAGCATCTTTGGCGACGGCGTGGGGCCATCAGTGCGGCGGATCCCCTAGGGGAATAAATCTTTGGGCACCTAGTGGTCATG

4 CATGACCACTAGGAGCATCTTTGGCGAATTCGGTTGGTGGAGTGTCGTCTGTCGTGACTAGGGGAATAAATCTTTGGGCACCTAGTGGTCATG

4 CATGACCACTAGGAGCATCTTTGGCGACCGGGCAAGCCGGCGATCATCTTGCGTATCCTAGGGGAATAAATCTTTGGGCACCTAGTGGTCATG

4 CATGACCACTAGGAGCATCTTTGGCGAACTACCACGCGAGTTCGGAGCGGGTAGCGACTAGGGGAATAAATCTTTGGGCACCTAGTGGTCATG

4 CATGACCACTAGGAGCATCTTTGGCGAAGCAGTGCGACGGGCTACGGGTTCCTCGAGCTAGGGGAATAAATCTTTGGGCACCTAGTGGTCATG

4 CATGACCACTAGGAGCATCTTTGGCGAGGAAAAGGACGACGCCAATGCGGCGAGCCTCTAGGGGAATAAATCTTTGGGCACCTAGTGGTCATG

4 CATGACCACTAGGAGCATCTTTGGCGAATGGCCGGCACGGCCTTCTAGTCCTAGGTACTAGGGGAATAAATCTTTGGGCACCTAGTGGTCATG

4 CATGACCACTAGGAGCATCTTTGGCGAACGTGCAAGATATGCAGCCTGGAGCGTAGGCTAGGGGAATAAATCTTTGGGCACCTAGTGGTCATG

4 CATGACCACTAGGAGCATCTTTGGCGACGATTGGTTGGAAGGCAAGTGTTCGGATGACTAGGGGAATAAATCTTTGGGCACCTAGTGGTCATG

4 CATGACCACTAGGAGCATCTTTGGCGAAGCGTGTTGAAAGGTCCGCGAGTGTGGACCCTAGGGGAATAAATCTTTGGGCACCTAGTGGTCATG

4 CATGACCACTAGGAGCATCTTTGGCGACGGTGTGGGGCTCCATGATGCGGCGGTCTCCTAGGGGAATAAATCTTTGGGCACCTAGTGGTCATG

4 CATGACCACTAGGAGCATCTTTGGCGAGTTGTCGTAGAGCTACGGGACACCCGTCAGCTAGGGGAATAAATCTTTGGGCACCTAGTGGTCATG

4 CATGACCACTAGGAGCATCTTTGGCGAAGGGTGTAGGACTTCAGGTGGATCTCATAGCTAGGGGAATAAATCTTTGGGCACCTAGTGGTCATG

4 CATGACCACTAGGAGCATCTTTGGCGACTCCCGTGGGCGGCAGGCACACGGAGTGGACTAGGGGAATAAATCTTTGGGCACCTAGTGGTCATG

4 CATGACCACTAGGAGCATCTTTGGCGAGCCTCGCTTAGGAGGTTGCTCCACCAGTTCCTAGGGGAATAAATCTTTGGGCACCTAGTGGTCATG

4 CATGACCACTAGGAGCATCTTTGGCGACCCAAGATAGTCATAGCTTGGAGTGTTGCTCTAGGGGAATAAATCTTTGGGCACCTAGTGGTCATG

4 CATGACCACTAGGAGCATCTTTGGCGAACGGAAGGTGTTGAGGTGGTCTGTGAATCCCTAGGGGAATAAATCTTTGGGCACCTAGTGGTCATG

4 CATGACCACTAGGAGCATCTTTGGCGAGATCGGGAGAATCGGCGGCATTGGTATCTCCTAGGGGAATAAATCTTTGGGCACCTAGTGGTCATG

4 CATGACCACTAGGAGCATCTTTGGCGAGCCTGGCTTGGGAGGTTGCTCCACCAGTTCCTAGGGGAATAAATCTTTGGGCACCTAGTGGTCATG

4 CATGACCACTAGGAGCATCTTTGGCGAGAAGACTCTGGATTCGCGGACCAGTTGCTGCTAGGGGAATAAATCTTTGGGCACCTAGTGGTCATG

4 CATGACCACTAGGAGCATCTTTGGCGAGTCGGGGCCGGTACGACGGGTCGTTGACTGCTAGGGGAATAAATCTTTGGGCACCTAGTGGTCATG

4 CATGACCACTAGGAGCATCTTTGGCGACGACGGGGTTGTGGCCTTGTGGGGAGGCGACTAGGGGAATAAATCTTTGGGCACCTAGTGGTCATG

4 CATGACCACTAGGAGCATCTTTGGCGACTCCAGTTTATCTGTCGGGCAGTTTAAGGACTAGGGGAATAAATCTTTGGGCACCTAGTGGTCATG

4 CATGACCACTAGGAGCATCTTTGGCGAGTATTGGCCGGTAGGTTGCGTATTGGGGAGCTAGGGGAATAAATCTTTGGGCACCTAGTGGTCATG

4 CATGACCACTAGGAGCATCTTTGGCGACCCGTAACGTAGCGACTGCGCACGGGTGGACTAGGGGAATAAATCTTTGGGCACCTAGTGGTCATG

4 CATGACCACTAGGAGCATCTTTGGCGACAAACGGGATGCAAATAGTGGCAGAGACGCCTAGGGGAATAAATCTTTGGGCACCTAGTGGTCATG

4 CATGACCACTAGGAGCATCTTTGGCGAAACGATAGGTACGCCCGGAGGCTCGCTCCGCTAGGGGAATAAATCTTTGGGCACCTAGTGGTCATG

4 CATGACCACTAGGAGCATCTTTGGCGAGAAGACTCTGGATTCGGGGACCAGTGGCTGCTAGGGGAATAAATCTTTGGGCACCTAGTGGTCATG

4 CATGACCACTAGGAGCATCTTTGGCGACGGGGTGGGGCCGAAGTGCGGCGGTGACTCCTAGGGGAATAAATCTTTGGGCACCTAGTGGTCATG

4 CATGACCACTAGGAGCATCTTTGGCGAGGGGGCGAGTGCGCGAGGTGTTATAGGGAGCTAGGGGAATAAATCTTTGGGCACCTAGTGGTCATG

4 CATGACCACTAGGAGCATCTTTGGCGAGTTGGGAGTCGCAGTATGCAGGCAGTGAGACTAGGGGAATAAATCTTTGGGCACCTAGTGGTCATG

4 CATGACCACTAGGAGCATCTTTGGCGAGCCTCGGTTGGGAGGTTGCTCCACCAGTTCCTAGGGGAATAAATCTTTGGGCACCTAGTGGTCATG

4 CATGACCACTAGGAGCATCTTTGGCGATCGGCTGGTAGGATGCGGGTGGTATTGGAGCTAGGGGAATAAATCTTTGGGCACCTAGTGGTCATG

4 CATGACCACTAGGAGCATCTTTGGCGAAAGTCGGTAGAACAGGGTGGGGTGCTGTACCTAGGGGAATAAATCTTTGGGCACCTAGTGGTCATG

4 CATGACCACTAGGAGCATCTTTGGCGAGCATACGGGCTATCTCGGTAAAGTGCGACGCTAGGGGAATAAATCTTTGGGCACCTAGTGGTCATG

4 CATGACCACTAGGAGCATCTTTGGCGAGCACTACGTAGCGGTAGGCATTGGTGTCTCCTAGGGGAATAAATCTTTGGGCACCTAGTGGTCATG

4 CATGACCACTAGGAGCATCTTTGGCGACCCTGGCTCGCGCTAGCGAACTGCGGTGGACTAGGGGAATAAATCTTTGGGCACCTAGTGGTCATG

4 CATGACCACTAGGAGCATCTTTGGCGACCCGCATAAACAAGTGATTTGCGAGGTGGACTAGGGGAATAAATCTTTGGGCACCTAGTGGTCATG

4 CATGACCACTAGGAGCATCTTTGGCGAGCCTTGCTAGGGAGGTTGCTCCACCAGTTCCTAGGGGAATAAATCTTTGGGCACCTAGTGGTCATG

4 CATGACCACTAGGAGCATCTTTGGCGAACAGGCCGCCAGGGCGTACGAAGTGTGGTTCTAGGGGAATAAATCTTTGGGCACCTAGTGGTCATG

3 CATGACCACTAGGAGCATCTTTGGCGAGCTAGCGCGGTCCCTCGGACCTCACAGTTGCTAGGGGAATAAATCTTTGGGCACCTAGTGGTCATG

3 CATGACCACTAGGAGCATCTTTGGCGACACGTTATAGGCGGAAAATAACGTTGTGGACTAGGGGAATAAATCTTTGGGCACCTAGTGGTCATG

3 CATGACCACTAGGAGCATCTTTGGCGAGATCGGGAGAAACGTTGGCATTGGTGTCTCCTAGGGGAATAAATCTTTGGGCACCTAGTGGTCATG

3 CATGACCACTAGGAGCATCTTTGGCGAACCGCAGGTGTTGCGGTGGTCTGTGAATCCCTAGGGGAATAAATCTTTGGGCACCTAGTGGTCATG

3 CATGACCACTAGGAGCATCTTTGGCGAAGGGTGTATGACTTCAAGTGGATCTCATAGCTAGGGGAATAAATCTTTGGGCACCTAGTGGTCATG

3 CATGACCACTAGGAGCATCTTTGGCGATGTGGGCTGGGAGGATGTGGTACTTCTCGACTAGGGGAATAAATCTTTGGGCACCTAGTGGTCATG

3 CATGACCACTAGGAGCATCTTTGGCGACGAAAAAGATCGCGTGTCGTGGATGTTCGCCTAGGGGAATAAATCTTTGGGCACCTAGTGGTCATG

3 CATGACCACTAGGAGCATCTTTGGCGACACGTCTGGTCGTCAGACCTGCGTTGTGGACTAGGGGAATAAATCTTTGGGCACCTAGTGGTCATG

3 CATGACCACTAGGAGCATCTTTGGCGAGATCGTGAGAATCGGTGGCATTGGTGCCTCCTAGGGGAATAAATCTTTGGGCACCTAGTGGTCATG

3 CATGACCACTAGGAGCATCTTTGGCGACATCCAGTGAAGGCGAATCACTGGTGTGGACTAGGGGAATAAATCTTTGGGCACCTAGTGGTCATG

3 CATGACCACTAGGAGCATCTTTGGCGAGATCGCGAGAATCGGCGGCATTGGTGTCTCCTAGGGGAATAAATCTTTGGGCACCTAGTGGTCATG

3 CATGACCACTAGGAGCATCTTTGGCGACCGAGGTGTAGCTAAATGCGTATAGTGGAGCTAGGGGAATAAATCTTTGGGCACCTAGTGGTCATG

3 CATGACCACTAGGAGCATCTTTGGCGATGTGACGCACGTCACCGAAGTGGCAAGAGCCTAGGGGAATAAATCTTTGGGCACCTAGTGGTCATG

3 CATGACCACTAGGAGCATCTTTGGCGAACACGAGAGTCAAGTGCGTACAGCGTGGAGCTAGGGGAATAAATCTTTGGGCACCTAGTGGTCATG

3 CATGACCACTAGGAGCATCTTTGGCGAGATCGGGAGAATAGGTGGCATTGGTGTCTACTAGGGGAATAAATCTTTGGGCACCTAGTGGTCATG

3 CATGACCACTAGGAGCATCTTTGGCGAGTAGTGGACGCAAGGGGATGAGCACACCGCCTAGGGGAATAAATCTTTGGGCACCTAGTGGTCATG

3 CATGACCACTAGGAGCATCTTTGGCGAATGGCCAGCACGGCCTTCTAGTCCTTGGTACTAGGGGAATAAATCTTTGGGCACCTAGTGGTCATG

3 CATGACCACTAGGAGCATCTTTGGCGAAGGGTGTAGGACTTCAATTGGATCTCATAGCTAGGGGAATAAATCTTTGGGCACCTAGTGGTCATG

3 CATGACCACTAGGAGCATCTTTGGCGACACGTGCGAAGGGTACGTAGCCGCTGTGGACTAGGGGAATAAATCTTTGGGCACCTAGTGGTCATG

3 CATGACCACTAGGAGCATCTTTGGCGAAAGTCGGTAGATCAGGGTGGGGTGCTGTCCCTAGGGGAATAAATCTTTGGGCACCTAGTGGTCATG

3 CATGACCACTAGGAGCATCTTTGGCGAGTCGAAGGGCGGCAGGTATTGTTATCGGCTCTAGGGGAATAAATCTTTGGGCACCTAGTGGTCATG

3 CATGACCACTAGGAGCATCTTTGGCGAGGGAGGGCGCCGGCAGCGGTGTGAAAGCGCCTAGGGGAATAAATCTTTGGGCACCTAGTGGTCATG

3 CATGACCACTAGGAGCATCTTTGGCGAGGGCGTGGGGCTATTGATGCGGCGGCACTCCTAGGGGAATAAATCTTTGGGCACCTAGTGGTCATG

3 CATGACCACTAGGAGCATCTTTGGCGACAGAGTGCGCATTGAGATCATAAGTCGCCGCTAGGGGAATAAATCTTTGGGCACCTAGTGGTCATG

3 CATGACCACTAGGAGCATCTTTGGCGACGTTGGTCATTGACGTGTATGGGTAGGAGACTAGGGGAATAAATCTTTGGGCACCTAGTGGTCATG

3 CATGACCACTAGGAGCATCTTTGGCGAGATCTGTAGAATCGGTGGCATTGGTGTCTCCTAGGGGAATAAATCTTTGGGCACCTAGTGGTCATG

3 CATGACCACTAGGAGCATCTTTGGCGAAGCGTGTCGCAAGGTCCGCGAGTGTGGACCCTAGGGGAATAAATCTTTGGGCACCTAGTGGTCATG

3 CATGACCACTAGGAGCATCTTTGGCGACCCGTCATATGATGAAATAGGAGTGGAAGACTAGGGGAATAAATCTTTGGGCACCTAGTGGTCATG

3 CATGACCACTAGGAGCATCTTTGGCGACTGAGTGGGCTGGGAGGAAGTATTTCGGAGCTAGGGGAATAAATCTTTGGGCACCTAGTGGTCATG

3 CATGACCACTAGGAGCATCTTTGGCGAGCTCTGCGGTAGCAGACTACGAAGTGGAGCCTAGGGGAATAAATCTTTGGGCACCTAGTGGTCATG

3 CATGACCACTAGGAGCATCTTTGGCGACCCGGCAAAACGTGTTATTGGCAGGGTGGACTAGGGGAATAAATCTTTGGGCACCTAGTGGTCATG

3 CATGACCACTAGGAGCATCTTTGGCGATAGAGGTCGCTAAGGTGCGAGAATCTTAGCCTAGGGGAATAAATCTTTGGGCACCTAGTGGTCATG

3 CATGACCACTAGGAGCATCTTTGGCGACAGTGGATGCCGATGCGCTGGACGACCGAGCTAGGGGAATAAATCTTTGGGCACCTAGTGGTCATG

3 CATGACCACTAGGAGCATCTTTGGCGATCAGGCCGCCAGGGCGTACGTAGTGTGGTTCTAGGGGAATAAATCTTTGGGCACCTAGTGGTCATG

3 CATGACCACTAGGAGCATCTTTGGCGATGGGGAGCAAGAGGACTGAGTAGGAATAAGCTAGGGGAATAAATCTTTGGGCACCTAGTGGTCATG

3 CATGACCACTAGGAGCATCTTTGGCGATGGGTGCTGACGACCGTTCACACGGTTACTCTAGGGGAATAAATCTTTGGGCACCTAGTGGTCATG

3 CATGACCACTAGGAGCATCTTTGGCGAGGGAGGGCGCCGGCAGCGGCGTGAATGCGCCTAGGGGAATAAATCTTTGGGCACCTAGTGGTCATG

3 CATGACCACTAGGAGCATCTTTGGCGAGCCTTGCTTGGGAGGTTTCTCCACCAGTTCCTAGGGGAATAAATCTTTGGGCACCTAGTGGTCATG

3 CATGACCACTAGGAGCATCTTTGGCGATGGGTGCTGACGGCCGCCGCTGAGGCTACACTAGGGGAATAAATCTTTGGGCACCTAGTGGTCATG

3 CATGACCACTAGGAGCATCTTTGGCGAGATCGGTAGAAACGGTGGCATTGGTGTCTCCTAGGGGAATAAATCTTTGGGCACCTAGTGGTCATG

3 CATGACCACTAGGAGCATCTTTGGCGACAGGGGATGCCGATGCGCTGGGCGACCGAGCTAGGGGAATAAATCTTTGGGCACCTAGTGGTCATG

3 CATGACCACTAGGAGCATCTTTGGCGAGGAGTGGCTGAGGGCGGTGGTTAGGTCGCGCTAGGGGAATAAATCTTTGGGCACCTAGTGGTCATG

3 CATGACCACTAGGAGCATCTTTGGCGAAAGCGCAGGGCGTGCGACCTCCAATTGGTCCTAGGGGAATAAATCTTTGGGCACCTAGTGGTCATG

3 CATGACCACTAGGAGCATCTTTGGCGAGTAGAGCTGGTAGGTTACGTATTTAAAAGACTAGGGGAATAAATCTTTGGGCACCTAGTGGTCATG

3 CATGACCACTAGGAGCATCTTTGGCGACGTAGGTGGCATCGTGGTAGAGCTACTAGACTAGGGGAATAAATCTTTGGGCACCTAGTGGTCATG

3 CATGACCACTAGGAGCATCTTTGGCGAACCTGTATGGTGATGAGGTCGGTATCAGCCCTAGGGGAATAAATCTTTGGGCACCTAGTGGTCATG

3 CATGACCACTAGGAGCATCTTTGGCGAGATCGTGAGAATCGGTGGCATTGGTGTATCCTAGGGGAATAAATCTTTGGGCACCTAGTGGTCATG

3 CATGACCACTAGGAGCATCTTTGGCGAGATCGGGAGAATCGGAGGCATTGTTGTCTCCTAGGGGAATAAATCTTTGGGCACCTAGTGGTCATG

3 CATGACCACTAGGAGCATCTTTGGCGAAGTAGAAGAGGACTCCATATGATTTACGAGCTAGGGGAATAAATCTTTGGGCACCTAGTGGTCATG

3 CATGACCACTAGGAGCATCTTTGGCGAGGTGGTCAGGGGGGCGTAGAGGCTGCACGCCTAGGGGAATAAATCTTTGGGCACCTAGTGGTCATG

3 CATGACCACTAGGAGCATCTTTGGCGAGTATCGCCGATGCGACGACGCATCGCAGAGCTAGGGGAATAAATCTTTGGGCACCTAGTGGTCATG

3 CATGACCACTAGGAGCATCTTTGGCGAGCCTTGCTTGGGAGGTTGCTCCACTAGTTCCTAGGGGAATAAATCTTTGGGCACCTAGTGGTCATG

3 CATGACCACTAGGAGCATCTTTGGCGATGTAATAGAGTGGCTGAGCACGAGTATCGACTAGGGGAATAAATCTTTGGGCACCTAGTGGTCATG

3 CATGACCACTAGGAGCATCTTTGGCGAGGGGTAGGGGGCCGCTCGCGCCTGAGTGCGCTAGGGGAATAAATCTTTGGGCACCTAGTGGTCATG

3 CATGACCACTAGGAGCATCTTTGGCGATGGGGCCGTGCTCTCTGCACACTCCGCGGGCTAGGGGAATAAATCTTTGGGCACCTAGTGGTCATG

3 CATGACCACTAGGAGCATCTTTGGCGAGCCATATAGGGGTATCCCGTTGCCTGGGTCCTAGGGGAATAAATCTTTGGGCACCTAGTGGTCATG

3 CATGACCACTAGGAGCATCTTTGGCGACCGTAGGTAAACAAAAGTGGCCTTGCTAGCCTAGGGGAATAAATCTTTGGGCACCTAGTGGTCATG

3 CATGACCACTAGGAGCATCTTTGGCGAATGGCCGGCACGTCCTTCTAGTCCTCGGTACTAGGGGAATAAATCTTTGGGCACCTAGTGGTCATG

3 CATGACCACTAGGAGCATCTTTGGCGAAACGGGGACCGGATCCCGGAGGTGTGTAGCCTAGGGGAATAAATCTTTGGGCACCTAGTGGTCATG

3 CATGACCACTAGGAGCATCTTTGGCGAAGGGTATAGGACTTCAAGTGGATCTCATAGCTAGGGGAATAAATCTTTGGGCACCTAGTGGTCATG

3 CATGACCACTAGGAGCATCTTTGGCGAAGACGACACAGTCGATCGGGGTAGAGGAAGCTAGGGGAATAAATCTTTGGGCACCTAGTGGTCATG

3 CATGACCACTAGGAGCATCTTTGGCGATCCGTGCTGAAAAGCCCCGTGGAGGGTTGTCTAGGGGAATAAATCTTTGGGCACCTAGTGGTCATG

3 CATGACCACTAGGAGCATCTTTGGCGACCCAATACTTCGCGAGACGTTATGGGTGGACTAGGGGAATAAATCTTTGGGCACCTAGTGGTCATG

3 CATGACCACTAGGAGCATCTTTGGCGATGTGGGCTGGAAGGATGTGGTACTTCTCGACTAGGGGAATAAATCTTTGGGCACCTAGTGGTCATG

3 CATGACCACTAGGAGCATCTTTGGCGATCAGGCCGCCAGTGCGTACGAAGTGTGGTTCTAGGGGAATAAATCTTTGGGCACCTAGTGGTCATG

3 CATGACCACTAGGAGCATCTTTGGCGACTTACGAGCGGTACGCGTGTTGGAAGTGGACTAGGGGAATAAATCTTTGGGCACCTAGTGGTCATG

3 CATGACCACTAGGAGCATCTTTGGCGATGGGGCCGTGCTATCTGCACACACCGCGGGCTAGGGGAATAAATCTTTGGGCACCTAGTGGTCATG

3 CATGACCACTAGGAGCATCTTTGGCGAGCCTTGCTTGGGAGGTTGCCCCACCAGTTCCTAGGGGAATAAATCTTTGGGCACCTAGTGGTCATG

3 CATGACCACTAGGAGCATCTTTGGCGACTAGGACTGGTAGCAAAGAGGTCTAGCGCCCTAGGGGAATAAATCTTTGGGCACCTAGTGGTCATG

3 CATGACCACTAGGAGCATCTTTGGCGACCGAGTGGTAAAGCGCGGAGAAGAGGAAGACTAGGGGAATAAATCTTTGGGCACCTAGTGGTCATG

3 CATGACCACTAGGAGCATCTTTGGCGACGGCGTGGGGCCATCTGTACGGCGGATCCCCTAGGGGAATAAATCTTTGGGCACCTAGTGGTCATG

3 CATGACCACTAGGAGCATCTTTGGCGAAGTAGTGGGATAACGGGTATGTATCGAAGCCTAGGGGAATAAATCTTTGGGCACCTAGTGGTCATG

3 CATGACCACTAGGAGCATCTTTGGCGACCCGGCAGTAAGAGATTAACTCCGGGTGGACTAGGGGAATAAATCTTTGGGCACCTAGTGGTCATG

3 CATGACCACTAGGAGCATCTTTGGCGAGGAGTGACTGAGGGCGGTGGGTAGGTCGCGCTAGGGGAATAAATCTTTGGGCACCTAGTGGTCATG

3 CATGACCACTAGGAGCATCTTTGGCGAGGCGGTGTGAATGCTAATGAGGAGGCGAGACTAGGGGAATAAATCTTTGGGCACCTAGTGGTCATG

3 CATGACCACTAGGAGCATCTTTGGCGAAGGCGATTGGTGTGGATCGTGGTGGGGTAGCTAGGGGAATAAATCTTTGGGCACCTAGTGGTCATG

3 CATGACCACTAGGAGCATCTTTGGCGACAGTGGCTACGCAATATGGCGGTGTGTCGACTAGGGGAATAAATCTTTGGGCACCTAGTGGTCATG

3 CATGACCACTAGGAGCATCTTTGGCGAGGGAGGGCGCCGGCAGCGGTGTGACTGCGCCTAGGGGAATAAATCTTTGGGCACCTAGTGGTCATG

3 CATGACCACTAGGAGCATCTTTGGCGATAGCGAAGTGCGTCGTCGTGGCTGAGGCACCTAGGGGAATAAATCTTTGGGCACCTAGTGGTCATG

3 CATGACCACTAGGAGCATCTTTGGCGAACGTGCAAGATATGCCGCCTGTAGCGTAGGCTAGGGGAATAAATCTTTGGGCACCTAGTGGTCATG

3 CATGACCACTAGGAGCATCTTTGGCGATGGGTGCTGACGGCCGCCGATGCGGCTACACTAGGGGAATAAATCTTTGGGCACCTAGTGGTCATG

3 CATGACCACTAGGAGCATCTTTGGCGACCCATCCGGCAGGGACGATGATGTGGTGGACTAGGGGAATAAATCTTTGGGCACCTAGTGGTCATG

3 CATGACCACTAGGAGCATCTTTGGCGATAGGCAGGTTGGTGGGTAACGTGTGGGCAGCTAGGGGAATAAATCTTTGGGCACCTAGTGGTCATG

3 CATGACCACTAGGAGCATCTTTGGCGACACTACAGGCCACGGGACTGAAGTTGTGGACTAGGGGAATAAATCTTTGGGCACCTAGTGGTCATG

3 CATGACCACTAGGAGCATCTTTGGCGATTAGATGGACTGGGATACACTGCAACTCGACTAGGGGAATAAATCTTTGGGCACCTAGTGGTCATG

3 CATGACCACTAGGAGCATCTTTGGCGACGTAGTGGACTTGGGAATCGACACAGTCGACTAGGGGAATAAATCTTTGGGCACCTAGTGGTCATG

3 CATGACCACTAGGAGCATCTTTGGCGACTAGGGCCCGGTCCACTGGCAATAGTTAGACTAGGGGAATAAATCTTTGGGCACCTAGTGGTCATG

3 CATGACCACTAGGAGCATCTTTGGCGAGATCGGGAGAATCGGTGGCATTGTTGTCTACTAGGGGAATAAATCTTTGGGCACCTAGTGGTCATG

3 CATGACCACTAGGAGCATCTTTGGCGAATACTGTCGTCGGCGAGCGACGCAGGGAGACTAGGGGAATAAATCTTTGGGCACCTAGTGGTCATG

3 CATGACCACTAGGAGCATCTTTGGCGAGGACTGCTTGCATACTCGGTTGCTGGGCGACTAGGGGAATAAATCTTTGGGCACCTAGTGGTCATG

3 CATGACCACTAGGAGCATCTTTGGCGACATTGGTGCACGTCTTACGTGTTCTTAAGCCTAGGGGAATAAATCTTTGGGCACCTAGTGGTCATG

3 CATGACCACTAGGAGCATCTTTGGCGATGTCCCGCTCAGCGTTATAGGATGCTCGAGCTAGGGGAATAAATCTTTGGGCACCTAGTGGTCATG

3 CATGACCACTAGGAGCATCTTTGGCGAATAGATTCGGAAGGTGGAGTGCTAGAGCGACTAGGGGAATAAATCTTTGGGCACCTAGTGGTCATG

3 CATGACCACTAGGAGCATCTTTGGCGAGATCGGGAGAATAGGTTGCATTGGTGTCTCCTAGGGGAATAAATCTTTGGGCACCTAGTGGTCATG

3 CATGACCACTAGGAGCATCTTTGGCGAGGTCGAGGAGAACTGAGGATACAGGTGCCTCTAGGGGAATAAATCTTTGGGCACCTAGTGGTCATG

3 CATGACCACTAGGAGCATCTTTGGCGATCCTCGTCAGATGGTGAAGCAGACGTGTGGCTAGGGGAATAAATCTTTGGGCACCTAGTGGTCATG

3 CATGACCACTAGGAGCATCTTTGGCGAGATCGGGAGAATCGGCGGCATTGGCGTCTCCTAGGGGAATAAATCTTTGGGCACCTAGTGGTCATG

3 CATGACCACTAGGAGCATCTTTGGCGAGATTACGCGGCTCGGGGCCCTGCGGTCAGACTAGGGGAATAAATCTTTGGGCACCTAGTGGTCATG

3 CATGACCACTAGGAGCATCTTTGGCGACCCGACATACCGCACGGGACGGCGGGTGGACTAGGGGAATAAATCTTTGGGCACCTAGTGGTCATG

3 CATGACCACTAGGAGCATCTTTGGCGAAAGGGGATGCCGATGCGCTGGACGACCGAGCTAGGGGAATAAATCTTTGGGCACCTAGTGGTCATG

3 CATGACCACTAGGAGCATCTTTGGCGACTTAGTACTGCGTCAGTAGAACGAAGTGGACTAGGGGAATAAATCTTTGGGCACCTAGTGGTCATG

3 CATGACCACTAGGAGCATCTTTGGCGATCGGCTGGTAGGATGCGGGTAGTATTGGAGCTAGGGGAATAAATCTTTGGGCACCTAGTGGTCATG

3 CATGACCACTAGGAGCATCTTTGGCGAGATCGGGAGAAACGGTGGCATTGGTGACTCCTAGGGGAATAAATCTTTGGGCACCTAGTGGTCATG

3 CATGACCACTAGGAGCATCTTTGGCGAGGGGTGCTGACGGCCGCCGCTGCGGCTACACTAGGGGAATAAATCTTTGGGCACCTAGTGGTCATG

3 CATGACCACTAGGAGCATCTTTGGCGATCCGGCCGCCAGGGCGTACGAAGTGTGGTTCTAGGGGAATAAATCTTTGGGCACCTAGTGGTCATG

3 CATGACCACTAGGAGCATCTTTGGCGATAGACGGTAGGGATCCAGTTTTTGTAGCTACTAGGGGAATAAATCTTTGGGCACCTAGTGGTCATG

3 CATGACCACTAGGAGCATCTTTGGCGACCCGATCCCCAGCATGGGTAGACGGGTGGACTAGGGGAATAAATCTTTGGGCACCTAGTGGTCATG

3 CATGACCACTAGGAGCATCTTTGGCGAGTTCTCCGCTGAGAATGAGCAGACGTTTGGCTAGGGGAATAAATCTTTGGGCACCTAGTGGTCATG

3 CATGACCACTAGGAGCATCTTTGGCGACCGGGGTCATGCGTCTGCAATGGGGTTAGACTAGGGGAATAAATCTTTGGGCACCTAGTGGTCATG

3 CATGACCACTAGGAGCATCTTTGGCGAGATCGGAAGAATCGGCGGCATTGGTGTCTCCTAGGGGAATAAATCTTTGGGCACCTAGTGGTCATG

3 CATGACCACTAGGAGCATCTTTGGCGAAGCGTGTTGCAAGGTCCGCGAGTGTGGACACTAGGGGAATAAATCTTTGGGCACCTAGTGGTCATG

3 CATGACCACTAGGAGCATCTTTGGCGAAGCGTGTTGCAAGGTCCGCGAGTGAGGACCCTAGGGGAATAAATCTTTGGGCACCTAGTGGTCATG

3 CATGACCACTAGGAGCATCTTTGGCGAAGCGTGTTGCAAGGTCCTCGAGTGTGGACCCTAGGGGAATAAATCTTTGGGCACCTAGTGGTCATG

3 CATGACCACTAGGAGCATCTTTGGCGATCCGGGGGTCCTGCGTGACGACGCAAAGGGCTAGGGGAATAAATCTTTGGGCACCTAGTGGTCATG

3 CATGACCACTAGGAGCATCTTTGGCGAGATCGAGAGAATCGGCGGCATTGGTGTCTCCTAGGGGAATAAATCTTTGGGCACCTAGTGGTCATG

3 CATGACCACTAGGAGCATCTTTGGCGAGATCGGGAGAATCGGCGGCATTTGTGTCTCCTAGGGGAATAAATCTTTGGGCACCTAGTGGTCATG

3 CATGACCACTAGGAGCATCTTTGGCGACCCGTGTGGGGAAATCCACATCGTGGTGGACTAGGGGAATAAATCTTTGGGCACCTAGTGGTCATG

3 CATGACCACTAGGAGCATCTTTGGCGAGCCTCGCTTGGGAGGTTGCTCCTCCAGTTCCTAGGGGAATAAATCTTTGGGCACCTAGTGGTCATG

3 CATGACCACTAGGAGCATCTTTGGCGACGGGGGATGCCGATGCGCTGGACGACCGAGCTAGGGGAATAAATCTTTGGGCACCTAGTGGTCATG

3 CATGACCACTAGGAGCATCTTTGGCGACGGGTGCTGACGGCCGCCGCTGCGGCTACACTAGGGGAATAAATCTTTGGGCACCTAGTGGTCATG

3 CATGACCACTAGGAGCATCTTTGGCGAACCGGGTCTTCCTGGGGCGCAGACGTTTGGCTAGGGGAATAAATCTTTGGGCACCTAGTGGTCATG

3 CATGACCACTAGGAGCATCTTTGGCGATCCTCGTCAGATGGTGAAGCAGACGTTCGGCTAGGGGAATAAATCTTTGGGCACCTAGTGGTCATG

3 CATGACCACTAGGAGCATCTTTGGCGACAAAGCGACGTAAGTGTGCTGATAGTGCGACTAGGGGAATAAATCTTTGGGCACCTAGTGGTCATG

3 CATGACCACTAGGAGCATCTTTGGCGATGGATGCTGACGGCCGCCGCTGCGGCTACACTAGGGGAATAAATCTTTGGGCACCTAGTGGTCATG

3 CATGACCACTAGGAGCATCTTTGGCGACCCAGTGTCCGGGAGGACATCTGTGGTGGACTAGGGGAATAAATCTTTGGGCACCTAGTGGTCATG

3 CATGACCACTAGGAGCATCTTTGGCGACGGCGTGGGGAACTTGTTTCGGCGGTGCTACTAGGGGAATAAATCTTTGGGCACCTAGTGGTCATG

3 CATGACCACTAGGAGCATCTTTGGCGAGCCTTGCTGGGGAGGTTGCTCCACCAGTTCCTAGGGGAATAAATCTTTGGGCACCTAGTGGTCATG

3 CATGACCACTAGGAGCATCTTTGGCGATGGGTGATGACGGCCGCCGCTGCGGCTACACTAGGGGAATAAATCTTTGGGCACCTAGTGGTCATG

3 CATGACCACTAGGAGCATCTTTGGCGACAGTGTGTGCGAGCATCGACATGCGGCCAGCTAGGGGAATAAATCTTTGGGCACCTAGTGGTCATG

3 CATGACCACTAGGAGCATCTTTGGCGATCCGGGGCGACTCCCAAGGAGGTTTTGTAGCTAGGGGAATAAATCTTTGGGCACCTAGTGGTCATG

3 CATGACCACTAGGAGCATCTTTGGCGAGCTAATGTTCGTCAGGCAAGACTGAATCGCCTAGGGGAATAAATCTTTGGGCACCTAGTGGTCATG

3 CATGACCACTAGGAGCATCTTTGGCGAGTTCTATTGGGAAGCTGCGTGGCGGCCAAGCTAGGGGAATAAATCTTTGGGCACCTAGTGGTCATG

3 CATGACCACTAGGAGCATCTTTGGCGAGCAGAGGGAGGTACACCAGTGCGCGAGCCGCTAGGGGAATAAATCTTTGGGCACCTAGTGGTCATG

3 CATGACCACTAGGAGCATCTTTGGCGACCCGTAGGCGCGTGTCGACTACGCGGTGGACTAGGGGAATAAATCTTTGGGCACCTAGTGGTCATG

3 CATGACCACTAGGAGCATCTTTGGCGATCCTCGTCAGATGGTGAAGCAGACGCTTGGCTAGGGGAATAAATCTTTGGGCACCTAGTGGTCATG

3 CATGACCACTAGGAGCATCTTTGGCGATTTGGGAGTCGCAATATGCAGGCAGTGAGACTAGGGGAATAAATCTTTGGGCACCTAGTGGTCATG

3 CATGACCACTAGGAGCATCTTTGGCGAAGCGGATGGCGAGTATGTGGGCAGCGGAGACTAGGGGAATAAATCTTTGGGCACCTAGTGGTCATG

3 CATGACCACTAGGAGCATCTTTGGCGACCGAGGATGCGTGTGATGTGGTCGAGCAAGCTAGGGGAATAAATCTTTGGGCACCTAGTGGTCATG

3 CATGACCACTAGGAGCATCTTTGGCGACTACAGGGTCATGGCAGTCGGTTTAGTCGACTAGGGGAATAAATCTTTGGGCACCTAGTGGTCATG

3 CATGACCACTAGGAGCATCTTTGGCGAAAGTCGGTAGAACAGGGTGGGGTGCTGTGCCTAGGGGAATAAATCTTTGGGCACCTAGTGGTCATG

3 CATGACCACTAGGAGCATCTTTGGCGACTCACAGACAGCATGTCACTGTGTAGTGGACTAGGGGAATAAATCTTTGGGCACCTAGTGGTCATG

3 CATGACCACTAGGAGCATCTTTGGCGAACAGGCCTCGGCTGCGGGTGGGTGCGCACCCTAGGGGAATAAATCTTTGGGCACCTAGTGGTCATG

3 CATGACCACTAGGAGCATCTTTGGCGAATGGATACGGAAGGCAAGTGTGCCACTAGCCTAGGGGAATAAATCTTTGGGCACCTAGTGGTCATG

3 CATGACCACTAGGAGCATCTTTGGCGAGCAGGGTAGATAAAGATGTGAGAAGCGAGACTAGGGGAATAAATCTTTGGGCACCTAGTGGTCATG

3 CATGACCACTAGGAGCATCTTTGGCGATCTTCGTCAGATGGTGAAGCAGACGTTTGGCTAGGGGAATAAATCTTTGGGCACCTAGTGGTCATG

3 CATGACCACTAGGAGCATCTTTGGCGACCCAAAATCCGAAGGATAGATTTGGGTGGACTAGGGGAATAAATCTTTGGGCACCTAGTGGTCATG

3 CATGACCACTAGGAGCATCTTTGGCGACCCACGACGCTCAGCGTATCGTGAGGTGGACTAGGGGAATAAATCTTTGGGCACCTAGTGGTCATG

3 CATGACCACTAGGAGCATCTTTGGCGAACGGAAGGTGTTTCGGTGGTCTGTGAATCCCTAGGGGAATAAATCTTTGGGCACCTAGTGGTCATG

3 CATGACCACTAGGAGCATCTTTGGCGAAGCGTGTTGCAATGTCCGCGAGTGTGGACCCTAGGGGAATAAATCTTTGGGCACCTAGTGGTCATG

3 CATGACCACTAGGAGCATCTTTGGCGAACCGTCAAGCGGAGCTGATAACGGGTGGCTCTAGGGGAATAAATCTTTGGGCACCTAGTGGTCATG

3 CATGACCACTAGGAGCATCTTTGGCGAGATCGGGAGAATCGGCGGCATTGGTGACTCCTAGGGGAATAAATCTTTGGGCACCTAGTGGTCATG

3 CATGACCACTAGGAGCATCTTTGGCGATCCTCGTCAGATGGTGAAACAGACGTTTGGCTAGGGGAATAAATCTTTGGGCACCTAGTGGTCATG

3 CATGACCACTAGGAGCATCTTTGGCGAGTTGGGTAGGAATAGGTGGGTCTATGGTGGCTAGGGGAATAAATCTTTGGGCACCTAGTGGTCATG

3 CATGACCACTAGGAGCATCTTTGGCGACCCGTGTCATCGTAATGAGACGGAGTGTTGCTAGGGGAATAAATCTTTGGGCACCTAGTGGTCATG

3 CATGACCACTAGGAGCATCTTTGGCGAGCCTTGCTCGGGAGGTTGCTCCACCAGTTCCTAGGGGAATAAATCTTTGGGCACCTAGTGGTCATG

3 CATGACCACTAGGAGCATCTTTGGCGACGAGGGGGCATTGGGAAATGTAAACGTCGACTAGGGGAATAAATCTTTGGGCACCTAGTGGTCATG

3 CATGACCACTAGGAGCATCTTTGGCGAGAAGACTCTGGATTCGGCGACCAGTTGCTGCTAGGGGAATAAATCTTTGGGCACCTAGTGGTCATG

3 CATGACCACTAGGAGCATCTTTGGCGAGGAAAACGATTTGAGATGAAAGGGCGAAGCCTAGGGGAATAAATCTTTGGGCACCTAGTGGTCATG

3 CATGACCACTAGGAGCATCTTTGGCGACACGTGGTTCGTGAAACAAGTGTAGTGGAGCTAGGGGAATAAATCTTTGGGCACCTAGTGGTCATG

3 CATGACCACTAGGAGCATCTTTGGCGACAGGGGTTGCCGATGCGCTGGACGACCGAGCTAGGGGAATAAATCTTTGGGCACCTAGTGGTCATG

3 CATGACCACTAGGAGCATCTTTGGCGAGATCGGGAGAATCGGTGGCATCGGTGTCCCCTAGGGGAATAAATCTTTGGGCACCTAGTGGTCATG

3 CATGACCACTAGGAGCATCTTTGGCGACCCGAGTCAATGTATGCGAGGAGTGGATCGCTAGGGGAATAAATCTTTGGGCACCTAGTGGTCATG

3 CATGACCACTAGGAGCATCTTTGGCGAATGGCCGGCACGGCTTTCTAGTCCTTGGTACTAGGGGAATAAATCTTTGGGCACCTAGTGGTCATG

3 CATGACCACTAGGAGCATCTTTGGCGAGGGTTAGGGTACGGCTGCTTCGATAGACGCCTAGGGGAATAAATCTTTGGGCACCTAGTGGTCATG

3 CATGACCACTAGGAGCATCTTTGGCGAAGCATGGGGGGGCAAGCCCCCCCGCGGCGACTAGGGGAATAAATCTTTGGGCACCTAGTGGTCATG

3 CATGACCACTAGGAGCATCTTTGGCGACGGTGTGGGGCCATTTGTGCGGCGGATCCCCTAGGGGAATAAATCTTTGGGCACCTAGTGGTCATG

3 CATGACCACTAGGAGCATCTTTGGCGATCCTCGTCAGATGGTGAAGCGGACGTTTGGCTAGGGGAATAAATCTTTGGGCACCTAGTGGTCATG

3 CATGACCACTAGGAGCATCTTTGGCGAGTATTGGCTGGTAGGTCGCGTATTGGGGAGCTAGGGGAATAAATCTTTGGGCACCTAGTGGTCATG

3 CATGACCACTAGGAGCATCTTTGGCGACACCAATCCTGGAAGCTGATTGGTCGTGGACTAGGGGAATAAATCTTTGGGCACCTAGTGGTCATG

3 CATGACCACTAGGAGCATCTTTGGCGAAAGGGCGCTAGTGGTCGTGGTTGCCCGTCCCTAGGGGAATAAATCTTTGGGCACCTAGTGGTCATG

3 CATGACCACTAGGAGCATCTTTGGCGACGGGGCCGGACGGGACGAACACATCGCGGCCTAGGGGAATAAATCTTTGGGCACCTAGTGGTCATG

3 CATGACCACTAGGAGCATCTTTGGCGACGGTGTGGACATTAACGCACGCTAGTGCAACTAGGGGAATAAATCTTTGGGCACCTAGTGGTCATG

3 CATGACCACTAGGAGCATCTTTGGCGAGCCTTGCTTGGGAGGTTGCTCCACCAGCTCCTAGGGGAATAAATCTTTGGGCACCTAGTGGTCATG

3 CATGACCACTAGGAGCATCTTTGGCGACCCGAGGCAATGTGCACCTCGGTGTGGGAGCTAGGGGAATAAATCTTTGGGCACCTAGTGGTCATG

3 CATGACCACTAGGAGCATCTTTGGCGAACGGCGGGTGTTGCGGTGGTCTGTGGATCCCTAGGGGAATAAATCTTTGGGCACCTAGTGGTCATG

3 CATGACCACTAGGAGCATCTTTGGCGATATGGACACATAACGAGGTCGATGTTAGCCCTAGGGGAATAAATCTTTGGGCACCTAGTGGTCATG

3 CATGACCACTAGGAGCATCTTTGGCGAGATCGGGAGAAACGGTGGCATTGGAGTCTCCTAGGGGAATAAATCTTTGGGCACCTAGTGGTCATG

3 CATGACCACTAGGAGCATCTTTGGCGACGGCGTGGGGGAGAAATCCGGCGGTGATTGCTAGGGGAATAAATCTTTGGGCACCTAGTGGTCATG

3 CATGACCACTAGGAGCATCTTTGGCGAGATCGGGAGAATCGGTGGCATATGTGTCTCCTAGGGGAATAAATCTTTGGGCACCTAGTGGTCATG

3 CATGACCACTAGGAGCATCTTTGGCGATTGGGAAGGGCCAAGGGCCCATCTACGTTCCTAGGGGAATAAATCTTTGGGCACCTAGTGGTCATG

3 CATGACCACTAGGAGCATCTTTGGCGACATGTTTCGCAACGCGGTAACAAAGTGGAGCTAGGGGAATAAATCTTTGGGCACCTAGTGGTCATG

3 CATGACCACTAGGAGCATCTTTGGCGACCCAGGTAGGGTGAAAAACCCCTGGGTGGACTAGGGGAATAAATCTTTGGGCACCTAGTGGTCATG

3 CATGACCACTAGGAGCATCTTTGGCGACCCAGACATGCGTGCATGCATCGGTGTGGCCTAGGGGAATAAATCTTTGGGCACCTAGTGGTCATG

3 CATGACCACTAGGAGCATCTTTGGCGAGTGGGGGGCGACGGACGGTAGTGGGTGAGACTAGGGGAATAAATCTTTGGGCACCTAGTGGTCATG

3 CATGACCACTAGGAGCATCTTTGGCGAGTCTACGCTGGCAGGATGTACGTGGGATAGCTAGGGGAATAAATCTTTGGGCACCTAGTGGTCATG

3 CATGACCACTAGGAGCATCTTTGGCGACCCGTCGCAAAGCGAATCGGAGTGTTGCCTCTAGGGGAATAAATCTTTGGGCACCTAGTGGTCATG

3 CATGACCACTAGGAGCATCTTTGGCGAAGCGTGTTGCAAGGTCCGCGAGTATGGACCCTAGGGGAATAAATCTTTGGGCACCTAGTGGTCATG

3 CATGACCACTAGGAGCATCTTTGGCGAGGGAGGTCGCCGGCAGCGGTGTGAATGCGCCTAGGGGAATAAATCTTTGGGCACCTAGTGGTCATG

3 CATGACCACTAGGAGCATCTTTGGCGATAGGTGCTGACGGCCGCCGCTGCGGCTACACTAGGGGAATAAATCTTTGGGCACCTAGTGGTCATG

3 CATGACCACTAGGAGCATCTTTGGCGAACGGCAGGTGTTGAGGTGGTCTGTGAATCACTAGGGGAATAAATCTTTGGGCACCTAGTGGTCATG

3 CATGACCACTAGGAGCATCTTTGGCGACCCGGTTCAGGCAAGCCGACACTGGGTGGACTAGGGGAATAAATCTTTGGGCACCTAGTGGTCATG

3 CATGACCACTAGGAGCATCTTTGGCGACGGTGTTGACCTTGTAGGTGTTTGGAGGCCCTAGGGGAATAAATCTTTGGGCACCTAGTGGTCATG

3 CATGACCACTAGGAGCATCTTTGGCGAGGATGGGCTGGGAGAACGGTATTCCGGTGCCTAGGGGAATAAATCTTTGGGCACCTAGTGGTCATG

3 CATGACCACTAGGAGCATCTTTGGCGACGTAGAGGCAGTCCCGGCCTTCGAAGTGGACTAGGGGAATAAATCTTTGGGCACCTAGTGGTCATG

3 CATGACCACTAGGAGCATCTTTGGCGACCCGACAGTTCGACGATATCTGCGGGTGGACTAGGGGAATAAATCTTTGGGCACCTAGTGGTCATG

3 CATGACCACTAGGAGCATCTTTGGCGAGGCGAGCCCAGCGGCCTTCTTGGCCTGCGACTAGGGGAATAAATCTTTGGGCACCTAGTGGTCATG

3 CATGACCACTAGGAGCATCTTTGGCGATCCAATCCGGTTTACCGGGAGGAGTGGAGCCTAGGGGAATAAATCTTTGGGCACCTAGTGGTCATG

3 CATGACCACTAGGAGCATCTTTGGCGATGGGTGCTGACGGCTGCCGCTGCGGCTACACTAGGGGAATAAATCTTTGGGCACCTAGTGGTCATG

3 CATGACCACTAGGAGCATCTTTGGCGACCCCGGACGTGTAACGATCGAGGGTGGAGACTAGGGGAATAAATCTTTGGGCACCTAGTGGTCATG

3 CATGACCACTAGGAGCATCTTTGGCGAACGTGCAAGATATGCCGACTGGAGCGTAGGCTAGGGGAATAAATCTTTGGGCACCTAGTGGTCATG

3 CATGACCACTAGGAGCATCTTTGGCGAGATCGTGAGAATCGGCGGCATTGGTGTCTCCTAGGGGAATAAATCTTTGGGCACCTAGTGGTCATG

3 CATGACCACTAGGAGCATCTTTGGCGATGGGGCCGTGCTATCTACACACTCCGCGGGCTAGGGGAATAAATCTTTGGGCACCTAGTGGTCATG

3 CATGACCACTAGGAGCATCTTTGGCGACTCCAGTTCATCTGTCGGGCAGCTTAAGGACTAGGGGAATAAATCTTTGGGCACCTAGTGGTCATG

3 CATGACCACTAGGAGCATCTTTGGCGACTCGACAGGGAATGCTCGTACCTAAGTCGACTAGGGGAATAAATCTTTGGGCACCTAGTGGTCATG

3 CATGACCACTAGGAGCATCTTTGGCGACGTGGGCAGTTTAGAAGATCGCCTAGTACGCTAGGGGAATAAATCTTTGGGCACCTAGTGGTCATG

3 CATGACCACTAGGAGCATCTTTGGCGATCCTTGCTTGGGAGGTTGCTCCACCAGTTCCTAGGGGAATAAATCTTTGGGCACCTAGTGGTCATG

3 CATGACCACTAGGAGCATCTTTGGCGACGGTGTGGGGAACTTGTTTCGGCTGTGCTACTAGGGGAATAAATCTTTGGGCACCTAGTGGTCATG

3 CATGACCACTAGGAGCATCTTTGGCGAGCCGGCTTGTACCAAGCATGATCTGGGTAACTAGGGGAATAAATCTTTGGGCACCTAGTGGTCATG

3 CATGACCACTAGGAGCATCTTTGGCGAGGTTGTAGCTCTCGCGTAGGGTCGGCAGTGCTAGGGGAATAAATCTTTGGGCACCTAGTGGTCATG

3 CATGACCACTAGGAGCATCTTTGGCGAATGGCCGGCACGGCCTTCTTGTCCTCGGTACTAGGGGAATAAATCTTTGGGCACCTAGTGGTCATG

3 CATGACCACTAGGAGCATCTTTGGCGACGAGTGGGCAGTACGGGAATCAGCCGGAGCCTAGGGGAATAAATCTTTGGGCACCTAGTGGTCATG

3 CATGACCACTAGGAGCATCTTTGGCGAAAGCGCAGGGCATGCGACCTCCAATCGGTCCTAGGGGAATAAATCTTTGGGCACCTAGTGGTCATG

3 CATGACCACTAGGAGCATCTTTGGCGAGATCGGTAGAATCGGTGGCATTTGTGTCTCCTAGGGGAATAAATCTTTGGGCACCTAGTGGTCATG

3 CATGACCACTAGGAGCATCTTTGGCGATTAGCGTTTGCGAGCGGGTGGGTTCGCTCCCTAGGGGAATAAATCTTTGGGCACCTAGTGGTCATG

3 CATGACCACTAGGAGCATCTTTGGCGACCCACAGGGCCACGTCCTGTGGAGTGTAGACTAGGGGAATAAATCTTTGGGCACCTAGTGGTCATG

3 CATGACCACTAGGAGCATCTTTGGCGACCCGAGTAATTGTCTCAAATACCGGGTGGACTAGGGGAATAAATCTTTGGGCACCTAGTGGTCATG

3 CATGACCACTAGGAGCATCTTTGGCGACCCTTCTCGCAGGCTGCGGGAAGAGGTGGACTAGGGGAATAAATCTTTGGGCACCTAGTGGTCATG

3 CATGACCACTAGGAGCATCTTTGGCGAGATCGTGAGAATCTGTGGCATTGGTGTCTCCTAGGGGAATAAATCTTTGGGCACCTAGTGGTCATG

3 CATGACCACTAGGAGCATCTTTGGCGACGTGGCATGGAGATCGGAAGTAAGAGTCGACTAGGGGAATAAATCTTTGGGCACCTAGTGGTCATG

3 CATGACCACTAGGAGCATCTTTGGCGAGATCGGGAGAATCGGCTGCATTGGTGTCTCCTAGGGGAATAAATCTTTGGGCACCTAGTGGTCATG

3 CATGACCACTAGGAGCATCTTTGGCGAAGTGATTGGAGATTACAAGCAGACGTTGGGCTAGGGGAATAAATCTTTGGGCACCTAGTGGTCATG

3 CATGACCACTAGGAGCATCTTTGGCGATAGCGAACATGCGACCAGAGTGGTTAAGTGCTAGGGGAATAAATCTTTGGGCACCTAGTGGTCATG

3 CATGACCACTAGGAGCATCTTTGGCGACGGCGTGGGGCCATCTGTGCGGCGGATCACCTAGGGGAATAAATCTTTGGGCACCTAGTGGTCATG

3 CATGACCACTAGGAGCATCTTTGGCGACGACAATGATAATCCAAGATTGTGAAGCGCCTAGGGGAATAAATCTTTGGGCACCTAGTGGTCATG

3 CATGACCACTAGGAGCATCTTTGGCGAACGACTAGACTGTCATGTCGGAATCCTGCACTAGGGGAATAAATCTTTGGGCACCTAGTGGTCATG

3 CATGACCACTAGGAGCATCTTTGGCGACCCGGATCTGCGGAGTAAGTCCGAGGTGGACTAGGGGAATAAATCTTTGGGCACCTAGTGGTCATG

3 CATGACCACTAGGAGCATCTTTGGCGACTGTGGGCCAGAGGTGTAATTATGAGGAGCCTAGGGGAATAAATCTTTGGGCACCTAGTGGTCATG

3 CATGACCACTAGGAGCATCTTTGGCGAAGCGTGTTGCAGGGTCCGCGAGTGTGGACCCTAGGGGAATAAATCTTTGGGCACCTAGTGGTCATG

3 CATGACCACTAGGAGCATCTTTGGCGACACCGTCGGCAGAACATGCGCGTTGTGGAGCTAGGGGAATAAATCTTTGGGCACCTAGTGGTCATG

3 CATGACCACTAGGAGCATCTTTGGCGATCAGGCCGCCAGGGCGTACGAAGTGGGGTTCTAGGGGAATAAATCTTTGGGCACCTAGTGGTCATG

3 CATGACCACTAGGAGCATCTTTGGCGAAACGTCCCGTGAGCCGTGGCTGAGTAGACACTAGGGGAATAAATCTTTGGGCACCTAGTGGTCATG

3 CATGACCACTAGGAGCATCTTTGGCGACGGCGTGGGGCCCATTGTGCGGCGGGGACTCTAGGGGAATAAATCTTTGGGCACCTAGTGGTCATG

3 CATGACCACTAGGAGCATCTTTGGCGAGGGAATCCCCTACATGTAGGGGCTATGTCACTAGGGGAATAAATCTTTGGGCACCTAGTGGTCATG

3 CATGACCACTAGGAGCATCTTTGGCGAGATCGGGAGAATCGGCGGCATTGGTGTCCCCTAGGGGAATAAATCTTTGGGCACCTAGTGGTCATG

3 CATGACCACTAGGAGCATCTTTGGCGAGCCTCGCTTGGAAGGTTGCTCCACCAGTTCCTAGGGGAATAAATCTTTGGGCACCTAGTGGTCATG

3 CATGACCACTAGGAGCATCTTTGGCGACAGTGGGCTAGGACCACACGAGGACTCAGGCTAGGGGAATAAATCTTTGGGCACCTAGTGGTCATG

3 CATGACCACTAGGAGCATCTTTGGCGAGGGGTAGGGGGCCGCTCGCGCCTCCGTGCGCTAGGGGAATAAATCTTTGGGCACCTAGTGGTCATG

3 CATGACCACTAGGAGCATCTTTGGCGACGTGGGCACAAGGAACAGAGAACACAGAGACTAGGGGAATAAATCTTTGGGCACCTAGTGGTCATG

3 CATGACCACTAGGAGCATCTTTGGCGATGGGGCCGTGCTATCTGCACACCCCGCGGGCTAGGGGAATAAATCTTTGGGCACCTAGTGGTCATG

3 CATGACCACTAGGAGCATCTTTGGCGATAGGCGTGTACGGTCGAGCGAATGCGATAGCTAGGGGAATAAATCTTTGGGCACCTAGTGGTCATG

3 CATGACCACTAGGAGCATCTTTGGCGAGATCGGGAGAATCGTTTGCATTGGTGTCTCCTAGGGGAATAAATCTTTGGGCACCTAGTGGTCATG

3 CATGACCACTAGGAGCATCTTTGGCGAAGCGTGATGCAAGGTCCGCGAGTGTGGACCCTAGGGGAATAAATCTTTGGGCACCTAGTGGTCATG

3 CATGACCACTAGGAGCATCTTTGGCGAGATCGGGAGAATCGGTGGAATTGTTGTCTCCTAGGGGAATAAATCTTTGGGCACCTAGTGGTCATG

3 CATGACCACTAGGAGCATCTTTGGCGATCCACTGCAGGAGGCAGTAGGTGTGGACGACTAGGGGAATAAATCTTTGGGCACCTAGTGGTCATG

3 CATGACCACTAGGAGCATCTTTGGCGAGCCTTGCTTGGGAGGTTGCACCACCAGTTCCTAGGGGAATAAATCTTTGGGCACCTAGTGGTCATG

3 CATGACCACTAGGAGCATCTTTGGCGACCCAGAGACATGTGCTCAGGGAGTGGTAGCCTAGGGGAATAAATCTTTGGGCACCTAGTGGTCATG

3 CATGACCACTAGGAGCATCTTTGGCGAAAGAGGTGTCTACGTTCGGGGGCGAATCGACTAGGGGAATAAATCTTTGGGCACCTAGTGGTCATG

3 CATGACCACTAGGAGCATCTTTGGCGAAGTGTGGAACATGCCGGTCTTGGTTGCACCCTAGGGGAATAAATCTTTGGGCACCTAGTGGTCATG

3 CATGACCACTAGGAGCATCTTTGGCGACTATACGTTATCGCGAGGTCGTTGCGAGCCCTAGGGGAATAAATCTTTGGGCACCTAGTGGTCATG

3 CATGACCACTAGGAGCATCTTTGGCGACCCGTGACCATGCAAATGGTTCGTGGTGGACTAGGGGAATAAATCTTTGGGCACCTAGTGGTCATG

3 CATGACCACTAGGAGCATCTTTGGCGATTTGGCCGCCAGGGCGTACGAAGTGTGGTTCTAGGGGAATAAATCTTTGGGCACCTAGTGGTCATG

3 CATGACCACTAGGAGCATCTTTGGCGAGATCGGGAGAATCGGTGGCATTGGTGACACCTAGGGGAATAAATCTTTGGGCACCTAGTGGTCATG

3 CATGACCACTAGGAGCATCTTTGGCGACTGTGCCGGGGTAGGCCTCAGCCCGTCGGCCTAGGGGAATAAATCTTTGGGCACCTAGTGGTCATG

3 CATGACCACTAGGAGCATCTTTGGCGACGGGGTGGGGCCGAAGTGCGGCGGCGATTCCTAGGGGAATAAATCTTTGGGCACCTAGTGGTCATG

3 CATGACCACTAGGAGCATCTTTGGCGAAGAAACCGGCAAATTAGCAATGCGTTAGAGCTAGGGGAATAAATCTTTGGGCACCTAGTGGTCATG

3 CATGACCACTAGGAGCATCTTTGGCGATCCTCGTCAGATGGTGAAGCAGATGTTTGGCTAGGGGAATAAATCTTTGGGCACCTAGTGGTCATG

3 CATGACCACTAGGAGCATCTTTGGCGAGATCGGGAGAATCGGTGGCATAGGAGTCTCCTAGGGGAATAAATCTTTGGGCACCTAGTGGTCATG

3 CATGACCACTAGGAGCATCTTTGGCGATATCTGGAGAATCGGTGGCATTGGTGTCTCCTAGGGGAATAAATCTTTGGGCACCTAGTGGTCATG

3 CATGACCACTAGGAGCATCTTTGGCGATCGCGACTGGGTTGCGACGCAGACGTTTGGCTAGGGGAATAAATCTTTGGGCACCTAGTGGTCATG

3 CATGACCACTAGGAGCATCTTTGGCGATGAGCTGTGGGCGTAGCCGGTTGTTTGCCGCTAGGGGAATAAATCTTTGGGCACCTAGTGGTCATG

3 CATGACCACTAGGAGCATCTTTGGCGAGACGGCACGATTGGATGGAACGATCGAGCACTAGGGGAATAAATCTTTGGGCACCTAGTGGTCATG

3 CATGACCACTAGGAGCATCTTTGGCGAGCCAACGCCACCGTCGCGTATGTCTGGGTCCTAGGGGAATAAATCTTTGGGCACCTAGTGGTCATG

3 CATGACCACTAGGAGCATCTTTGGCGAATGAGTGTAGACGGAAATCCGAACTAAAGCCTAGGGGAATAAATCTTTGGGCACCTAGTGGTCATG

3 CATGACCACTAGGAGCATCTTTGGCGAAGCAGCGCGACGGGCTATGGGTTCCTCGAGCTAGGGGAATAAATCTTTGGGCACCTAGTGGTCATG

3 CATGACCACTAGGAGCATCTTTGGCGATTTGCTCGCCGCTGGCCGCGGAGTAAAAGCCTAGGGGAATAAATCTTTGGGCACCTAGTGGTCATG

3 CATGACCACTAGGAGCATCTTTGGCGACCCGGCGGGATAGTGCCAGCGACGGGTGGACTAGGGGAATAAATCTTTGGGCACCTAGTGGTCATG

3 CATGACCACTAGGAGCATCTTTGGCGAATAGCTGTGCACGTGGGTGTGGTCCGGGCCCTAGGGGAATAAATCTTTGGGCACCTAGTGGTCATG

3 CATGACCACTAGGAGCATCTTTGGCGACACGAAGAGACGGAGTCAAGCGAAGTGGACCTAGGGGAATAAATCTTTGGGCACCTAGTGGTCATG

3 CATGACCACTAGGAGCATCTTTGGCGAGGCGAGTCCAGCGGCCTACTTGGCCTGCGACTAGGGGAATAAATCTTTGGGCACCTAGTGGTCATG

3 CATGACCACTAGGAGCATCTTTGGCGACAACGTCGATGAGATAGCGACGCCGTGGAGCTAGGGGAATAAATCTTTGGGCACCTAGTGGTCATG

3 CATGACCACTAGGAGCATCTTTGGCGACCCTCATGGGGTTGTGACCAGAGAACAGTCCTAGGGGAATAAATCTTTGGGCACCTAGTGGTCATG

3 CATGACCACTAGGAGCATCTTTGGCGACCCGGCATACCTGTATGTACGGAGTGGAGACTAGGGGAATAAATCTTTGGGCACCTAGTGGTCATG

3 CATGACCACTAGGAGCATCTTTGGCGAGGGAGGGCGCCGACAGCGGTGTGAATGCGCCTAGGGGAATAAATCTTTGGGCACCTAGTGGTCATG

3 CATGACCACTAGGAGCATCTTTGGCGACTACCGTAGAAGTATTTCGTACGGAGTGGACTAGGGGAATAAATCTTTGGGCACCTAGTGGTCATG

3 CATGACCACTAGGAGCATCTTTGGCGAATGTGCCTTTGGCACTCCTAGTCCTTGGTACTAGGGGAATAAATCTTTGGGCACCTAGTGGTCATG

3 CATGACCACTAGGAGCATCTTTGGCGATGGGTGCCGACGGCCGCCGCTGCGGCTACACTAGGGGAATAAATCTTTGGGCACCTAGTGGTCATG

3 CATGACCACTAGGAGCATCTTTGGCGACTATGCAAGTGGTCACCGTCGGAAGTCGCTCTAGGGGAATAAATCTTTGGGCACCTAGTGGTCATG

3 CATGACCACTAGGAGCATCTTTGGCGAGATCGGGAGAATCGGTGGCAGTGGGGTCTCCTAGGGGAATAAATCTTTGGGCACCTAGTGGTCATG

3 CATGACCACTAGGAGCATCTTTGGCGACCCATCAGTATAGCATCATCTGTGGGTGGACTAGGGGAATAAATCTTTGGGCACCTAGTGGTCATG

3 CATGACCACTAGGAGCATCTTTGGCGAGGTGTTTCAGTTCGACATAAGGGGCGAGCGCTAGGGGAATAAATCTTTGGGCACCTAGTGGTCATG

3 CATGACCACTAGGAGCATCTTTGGCGACCCTGCGCAGTACGTAGTATCCAGGGTGGACTAGGGGAATAAATCTTTGGGCACCTAGTGGTCATG

3 CATGACCACTAGGAGCATCTTTGGCGAGAAGACTTAGGATTCGGGGACCAGTTGCTGCTAGGGGAATAAATCTTTGGGCACCTAGTGGTCATG

3 CATGACCACTAGGAGCATCTTTGGCGACCCGCTACTGCATCGTAACGTACGGGTGGACTAGGGGAATAAATCTTTGGGCACCTAGTGGTCATG

3 CATGACCACTAGGAGCATCTTTGGCGACAAGGGGGCAGTGTGGTGATAGGGTGTCGACTAGGGGAATAAATCTTTGGGCACCTAGTGGTCATG

3 CATGACCACTAGGAGCATCTTTGGCGAGTGTCAGGAGGCAAATATGGTGCAACGAGACTAGGGGAATAAATCTTTGGGCACCTAGTGGTCATG

3 CATGACCACTAGGAGCATCTTTGGCGAGTGGGGGGCGACGGCCGGTCGTGGGTGAGACTAGGGGAATAAATCTTTGGGCACCTAGTGGTCATG

3 CATGACCACTAGGAGCATCTTTGGCGAACGTGCAAGATAAGCCGCCTGGAGCGTAGGCTAGGGGAATAAATCTTTGGGCACCTAGTGGTCATG

3 CATGACCACTAGGAGCATCTTTGGCGAATGGCCGGCACGGCCTTCTAGTCCCTGGTACTAGGGGAATAAATCTTTGGGCACCTAGTGGTCATG

3 CATGACCACTAGGAGCATCTTTGGCGAGACGACTCTGGATTCGGGGACCAGTTGCTGCTAGGGGAATAAATCTTTGGGCACCTAGTGGTCATG

3 CATGACCACTAGGAGCATCTTTGGCGACCCGGCCGGCGTAAGACGCGCTAGGGTGGACTAGGGGAATAAATCTTTGGGCACCTAGTGGTCATG

3 CATGACCACTAGGAGCATCTTTGGCGAGCCTTGCTTGGGAGGTTGCTCCACCAGTCCCTAGGGGAATAAATCTTTGGGCACCTAGTGGTCATG

3 CATGACCACTAGGAGCATCTTTGGCGACAAGACCCTATATGTAGGGGCTAAGTGGAGCTAGGGGAATAAATCTTTGGGCACCTAGTGGTCATG

3 CATGACCACTAGGAGCATCTTTGGCGAGACTCTGCGGACCCTCACGATTCGTGGGTCCTAGGGGAATAAATCTTTGGGCACCTAGTGGTCATG

3 CATGACCACTAGGAGCATCTTTGGCGAGATAGGGAGAATCGGCGGCATTGGTGTCTCCTAGGGGAATAAATCTTTGGGCACCTAGTGGTCATG

3 CATGACCACTAGGAGCATCTTTGGCGACATCCGTGGGTGGCGACCCATCGGTGTGGACTAGGGGAATAAATCTTTGGGCACCTAGTGGTCATG

3 CATGACCACTAGGAGCATCTTTGGCGAACGCAAAAGGGCACAAAATATAAGAGTTCGCTAGGGGAATAAATCTTTGGGCACCTAGTGGTCATG

3 CATGACCACTAGGAGCATCTTTGGCGATCCGGAAAGCAGCGCAAAACGGTGTGGAGCCTAGGGGAATAAATCTTTGGGCACCTAGTGGTCATG

3 CATGACCACTAGGAGCATCTTTGGCGACAACAGTGCGTAGCACATAGATAAATGAGCCTAGGGGAATAAATCTTTGGGCACCTAGTGGTCATG

3 CATGACCACTAGGAGCATCTTTGGCGAGGGTACGCATGCCGCAGGGTCAAGGAGCTGCTAGGGGAATAAATCTTTGGGCACCTAGTGGTCATG

3 CATGACCACTAGGAGCATCTTTGGCGACGAGCACATCCCATGTGCGGTAGTGGAGCTCTAGGGGAATAAATCTTTGGGCACCTAGTGGTCATG

3 CATGACCACTAGGAGCATCTTTGGCGACGAGTGCTGCACCGGACAATAAAACGTCGACTAGGGGAATAAATCTTTGGGCACCTAGTGGTCATG

3 CATGACCACTAGGAGCATCTTTGGCGAGATCGGGAGAATCTGTGGCATTGGTGTATCCTAGGGGAATAAATCTTTGGGCACCTAGTGGTCATG

3 CATGACCACTAGGAGCATCTTTGGCGAAACGTGACGAGCTCACGTGGGCGGGGTGCCCTAGGGGAATAAATCTTTGGGCACCTAGTGGTCATG

3 CATGACCACTAGGAGCATCTTTGGCGAGCCGGAGAGTTGTGCGATCATGCCTGGGTCCTAGGGGAATAAATCTTTGGGCACCTAGTGGTCATG

3 CATGACCACTAGGAGCATCTTTGGCGACCCGTACTTGATGTGCAAGTATGTGGTGGACTAGGGGAATAAATCTTTGGGCACCTAGTGGTCATG

3 CATGACCACTAGGAGCATCTTTGGCGAGGGAGGGCGACGGCAGCGGTGTGAATGCGCCTAGGGGAATAAATCTTTGGGCACCTAGTGGTCATG

3 CATGACCACTAGGAGCATCTTTGGCGACTGCGGTAGAACTCGAACTGCCGAAGTGGACTAGGGGAATAAATCTTTGGGCACCTAGTGGTCATG

3 CATGACCACTAGGAGCATCTTTGGCGAAAGTCGGTAGTACAGGGTGGGGTGCTGTCCCTAGGGGAATAAATCTTTGGGCACCTAGTGGTCATG

3 CATGACCACTAGGAGCATCTTTGGCGATGTAAAGTTGGGTCTGCTCAGAACTGTCGCCTAGGGGAATAAATCTTTGGGCACCTAGTGGTCATG

3 CATGACCACTAGGAGCATCTTTGGCGACCCAAGTGCAAGACGCGCACTAAGGGTGGACTAGGGGAATAAATCTTTGGGCACCTAGTGGTCATG

3 CATGACCACTAGGAGCATCTTTGGCGACGCCGATTGGATGCGCGTGGTGGTCGGCGCCTAGGGGAATAAATCTTTGGGCACCTAGTGGTCATG

3 CATGACCACTAGGAGCATCTTTGGCGAGGAGACATAGGAGGCTACCCGGTGGTCGAGCTAGGGGAATAAATCTTTGGGCACCTAGTGGTCATG

3 CATGACCACTAGGAGCATCTTTGGCGATGGCGTGGGGCCATCTGTGCGGCGGATCCCCTAGGGGAATAAATCTTTGGGCACCTAGTGGTCATG

3 CATGACCACTAGGAGCATCTTTGGCGAGACTGTGCAAGGCAGAGTACGAAGTGGAGCCTAGGGGAATAAATCTTTGGGCACCTAGTGGTCATG

3 CATGACCACTAGGAGCATCTTTGGCGACGCGAGGTGATAGTAGCATCTCGACGTGGACTAGGGGAATAAATCTTTGGGCACCTAGTGGTCATG

3 CATGACCACTAGGAGCATCTTTGGCGACAACCCGCACGGAGTGGAGCAGACGTTTGGCTAGGGGAATAAATCTTTGGGCACCTAGTGGTCATG

3 CATGACCACTAGGAGCATCTTTGGCGAAGTTAAGGTTGGGATTAGTGTAACTACAAGCTAGGGGAATAAATCTTTGGGCACCTAGTGGTCATG

3 CATGACCACTAGGAGCATCTTTGGCGATGGGGCCGTGCTATCTGCACACTCCTCGGGCTAGGGGAATAAATCTTTGGGCACCTAGTGGTCATG

3 CATGACCACTAGGAGCATCTTTGGCGACCCGATCACGGGACTCCGTGACGAGGTGGACTAGGGGAATAAATCTTTGGGCACCTAGTGGTCATG

3 CATGACCACTAGGAGCATCTTTGGCGACACGGACACGCTCGTCGAGTCCGCTGTGGACTAGGGGAATAAATCTTTGGGCACCTAGTGGTCATG

3 CATGACCACTAGGAGCATCTTTGGCGACAGCGGCGTAAGCTGCCTTGGGTGGTGGCCCTAGGGGAATAAATCTTTGGGCACCTAGTGGTCATG

3 CATGACCACTAGGAGCATCTTTGGCGATACTTCTTTGGACTAGGACTATGTCCATTACTAGGGGAATAAATCTTTGGGCACCTAGTGGTCATG

3 CATGACCACTAGGAGCATCTTTGGCGATCCTCGTCAGATGGTGTAGCAGACGTTTGGCTAGGGGAATAAATCTTTGGGCACCTAGTGGTCATG

3 CATGACCACTAGGAGCATCTTTGGCGACCCGGTGTTCCGAAGAAACATCAGGGTGGACTAGGGGAATAAATCTTTGGGCACCTAGTGGTCATG

3 CATGACCACTAGGAGCATCTTTGGCGAAGATCCGATGGGCTTGTGACCCGCCTTGGCCTAGGGGAATAAATCTTTGGGCACCTAGTGGTCATG

3 CATGACCACTAGGAGCATCTTTGGCGATCAGGCCGCAAGGGCGTACGAAGTGTGGTTCTAGGGGAATAAATCTTTGGGCACCTAGTGGTCATG

3 CATGACCACTAGGAGCATCTTTGGCGATGAGTCCTGCTCTAGCCCGAGGAGAGCCTCCTAGGGGAATAAATCTTTGGGCACCTAGTGGTCATG

3 CATGACCACTAGGAGCATCTTTGGCGAGGCGAGTCCAGCGGCCTTCTAGGCCTGCGACTAGGGGAATAAATCTTTGGGCACCTAGTGGTCATG

3 CATGACCACTAGGAGCATCTTTGGCGAGTTGCTCTCAGCTAACCGTGAGGTTCAGCACTAGGGGAATAAATCTTTGGGCACCTAGTGGTCATG

3 CATGACCACTAGGAGCATCTTTGGCGATCCTCGTCAGATGGTGAAGCAGAGGTTTGGCTAGGGGAATAAATCTTTGGGCACCTAGTGGTCATG

3 CATGACCACTAGGAGCATCTTTGGCGATCCCCGTCAGATGGTGAAGCAGACGTTTGGCTAGGGGAATAAATCTTTGGGCACCTAGTGGTCATG

3 CATGACCACTAGGAGCATCTTTGGCGACGTAAGGTCAGGAACAGAGGAAGGAGTCGACTAGGGGAATAAATCTTTGGGCACCTAGTGGTCATG

3 CATGACCACTAGGAGCATCTTTGGCGACGGGCTGGTAGGTTCAAGGGTATCTTCGCACTAGGGGAATAAATCTTTGGGCACCTAGTGGTCATG

3 CATGACCACTAGGAGCATCTTTGGCGAACGGAAGGTGTTGCGGTGGTCGGTGAATCCCTAGGGGAATAAATCTTTGGGCACCTAGTGGTCATG

3 CATGACCACTAGGAGCATCTTTGGCGAAAGTCGGTAGAACAGGGTGGGGTTCGCTCCCTAGGGGAATAAATCTTTGGGCACCTAGTGGTCATG

3 CATGACCACTAGGAGCATCTTTGGCGACCCGGCGACCGATGTAAGCAGACGGGTGGACTAGGGGAATAAATCTTTGGGCACCTAGTGGTCATG

3 CATGACCACTAGGAGCATCTTTGGCGACAGCGAAGTGCTGTGAACAGTGGTTAGCACCTAGGGGAATAAATCTTTGGGCACCTAGTGGTCATG

3 CATGACCACTAGGAGCATCTTTGGCGATAGACACAGCATTGAAACCAAGGATCGCCGCTAGGGGAATAAATCTTTGGGCACCTAGTGGTCATG

3 CATGACCACTAGGAGCATCTTTGGCGATGTGTGCTGACGGCCGCCGCTGCGGCTACACTAGGGGAATAAATCTTTGGGCACCTAGTGGTCATG

3 CATGACCACTAGGAGCATCTTTGGCGAGAAGACTCCGGATTCGGGGACCAGTTGCTCCTAGGGGAATAAATCTTTGGGCACCTAGTGGTCATG

3 CATGACCACTAGGAGCATCTTTGGCGAAGTGGGCTGGTAGGATCGTATTATTATCGACTAGGGGAATAAATCTTTGGGCACCTAGTGGTCATG

3 CATGACCACTAGGAGCATCTTTGGCGACTGATGAGTCATGGTAATGAATCCAGTGGACTAGGGGAATAAATCTTTGGGCACCTAGTGGTCATG

3 CATGACCACTAGGAGCATCTTTGGCGAGTTTCGCTGTGCTTGGAATGGGGTCACCCTCTAGGGGAATAAATCTTTGGGCACCTAGTGGTCATG

3 CATGACCACTAGGAGCATCTTTGGCGACCGAAAGCAGCCGTGAGTAGACTTGTCGCCCTAGGGGAATAAATCTTTGGGCACCTAGTGGTCATG

3 CATGACCACTAGGAGCATCTTTGGCGAAACTGCGAGGAGGGGAACGCGCAGGCCCGCCTAGGGGAATAAATCTTTGGGCACCTAGTGGTCATG

3 CATGACCACTAGGAGCATCTTTGGCGATTCTCGTCAGATGGTGAAGCAGACGTTTGGCTAGGGGAATAAATCTTTGGGCACCTAGTGGTCATG

3 CATGACCACTAGGAGCATCTTTGGCGATGCGAGTCCAGCGGCCTTCTTGGCCTGCGACTAGGGGAATAAATCTTTGGGCACCTAGTGGTCATG

3 CATGACCACTAGGAGCATCTTTGGCGAGATCGGGAGAGTCGGCGGCATTGGTGTCTCCTAGGGGAATAAATCTTTGGGCACCTAGTGGTCATG

3 CATGACCACTAGGAGCATCTTTGGCGATCCTCGGCAGATGGTGAAGCAGACGTTTGGCTAGGGGAATAAATCTTTGGGCACCTAGTGGTCATG

3 CATGACCACTAGGAGCATCTTTGGCGAGATCGGGAGAATCGGTGTCATTGTTGTCTCCTAGGGGAATAAATCTTTGGGCACCTAGTGGTCATG

3 CATGACCACTAGGAGCATCTTTGGCGAACGGCAGGTGTTGCGGTGGTCTGTGAAACACTAGGGGAATAAATCTTTGGGCACCTAGTGGTCATG

3 CATGACCACTAGGAGCATCTTTGGCGAGATCGGGAGAATCGGTGGCATAGGTGACTCCTAGGGGAATAAATCTTTGGGCACCTAGTGGTCATG

3 CATGACCACTAGGAGCATCTTTGGCGAGCCTCGCTTGGGATGTTGCTCCACCAGTTCCTAGGGGAATAAATCTTTGGGCACCTAGTGGTCATG

3 CATGACCACTAGGAGCATCTTTGGCGAAGCGAGTCCAGCGGCCTTCTTGGCCTGCGACTAGGGGAATAAATCTTTGGGCACCTAGTGGTCATG

3 CATGACCACTAGGAGCATCTTTGGCGAGTCGGGGTAAACGCGGGTAGTTGACTGCCGCTAGGGGAATAAATCTTTGGGCACCTAGTGGTCATG

3 CATGACCACTAGGAGCATCTTTGGCGAATGGGCGGCACGGCCTTCTAGTCCTCGGTACTAGGGGAATAAATCTTTGGGCACCTAGTGGTCATG

3 CATGACCACTAGGAGCATCTTTGGCGAAGGTACGGGTCCTGCCGGTAGGTGCGAGCGCTAGGGGAATAAATCTTTGGGCACCTAGTGGTCATG

3 CATGACCACTAGGAGCATCTTTGGCGAGGGGACCTGTCGGGCCGGAGTGCTCCAACGCTAGGGGAATAAATCTTTGGGCACCTAGTGGTCATG

3 CATGACCACTAGGAGCATCTTTGGCGAAGGGTGTAGGACTTCAAGTGGATCTCATAACTAGGGGAATAAATCTTTGGGCACCTAGTGGTCATG

3 CATGACCACTAGGAGCATCTTTGGCGATACATTACGGGCACAGATGCCCAAGTGGAGCTAGGGGAATAAATCTTTGGGCACCTAGTGGTCATG

3 CATGACCACTAGGAGCATCTTTGGCGAGATCGGGAGAATCGACGGCATTGGTGTCTCCTAGGGGAATAAATCTTTGGGCACCTAGTGGTCATG

3 CATGACCACTAGGAGCATCTTTGGCGAGGCAATAAGATGACTAATAGTGGCTGAGACCTAGGGGAATAAATCTTTGGGCACCTAGTGGTCATG

3 CATGACCACTAGGAGCATCTTTGGCGAAGGGTGTAGGACTTAAAGTGGATCTCATAGCTAGGGGAATAAATCTTTGGGCACCTAGTGGTCATG

3 CATGACCACTAGGAGCATCTTTGGCGAAGGGTGTAGGACTTCGAGTGGATCTCATAGCTAGGGGAATAAATCTTTGGGCACCTAGTGGTCATG

3 CATGACCACTAGGAGCATCTTTGGCGACGGTGTGGGGAACTTGTTTCGGCGGTGTTACTAGGGGAATAAATCTTTGGGCACCTAGTGGTCATG

3 CATGACCACTAGGAGCATCTTTGGCGACGAGTTTCACGGTAGTGGTACGAAGTGGACCTAGGGGAATAAATCTTTGGGCACCTAGTGGTCATG

3 CATGACCACTAGGAGCATCTTTGGCGAGCCGAAGAAAAATTTAGTCCTATAAGCGAGCTAGGGGAATAAATCTTTGGGCACCTAGTGGTCATG

3 CATGACCACTAGGAGCATCTTTGGCGAATGGTTGGTCTGACTTCCAGTCACAGGAGACTAGGGGAATAAATCTTTGGGCACCTAGTGGTCATG

3 CATGACCACTAGGAGCATCTTTGGCGAGGGGCGCTCTGGGTGAGACCTTCATTGGTCCTAGGGGAATAAATCTTTGGGCACCTAGTGGTCATG

3 CATGACCACTAGGAGCATCTTTGGCGATCCTCGTCAGATGGTGAAGCAGACGTTTGACTAGGGGAATAAATCTTTGGGCACCTAGTGGTCATG

3 CATGACCACTAGGAGCATCTTTGGCGAGAAGACTCTGGATTCCGGGACCAGTTGCTGCTAGGGGAATAAATCTTTGGGCACCTAGTGGTCATG

3 CATGACCACTAGGAGCATCTTTGGCGAGTGTGGTCGGGATCCTGGGCGCCTTCGAGCCTAGGGGAATAAATCTTTGGGCACCTAGTGGTCATG

3 CATGACCACTAGGAGCATCTTTGGCGACCCGGTGGCGCGTCAGCGCGCCGCGGTGGACTAGGGGAATAAATCTTTGGGCACCTAGTGGTCATG

3 CATGACCACTAGGAGCATCTTTGGCGATGGGGCCGCGCTATCTGCACACTCCGCGGGCTAGGGGAATAAATCTTTGGGCACCTAGTGGTCATG

3 CATGACCACTAGGAGCATCTTTGGCGAAGTTAAGATCAAGTGACAAATGTTATTCGCCTAGGGGAATAAATCTTTGGGCACCTAGTGGTCATG

3 CATGACCACTAGGAGCATCTTTGGCGATCAGCTGAGGCGTCATGCAGCGGAGTGGAACTAGGGGAATAAATCTTTGGGCACCTAGTGGTCATG

3 CATGACCACTAGGAGCATCTTTGGCGAGCCCGTTACGAAAGTAGCAAACGGCGTGGACTAGGGGAATAAATCTTTGGGCACCTAGTGGTCATG

3 CATGACCACTAGGAGCATCTTTGGCGACCTTCATGGGGTTGTGACCAGAGAACAGTCCTAGGGGAATAAATCTTTGGGCACCTAGTGGTCATG

3 CATGACCACTAGGAGCATCTTTGGCGAGAAGACTCTGGATTCGGGGACGAGTTGCTGCTAGGGGAATAAATCTTTGGGCACCTAGTGGTCATG

3 CATGACCACTAGGAGCATCTTTGGCGAGGTAGGCAGCCAGGACGCGGGGACGCCGAGCTAGGGGAATAAATCTTTGGGCACCTAGTGGTCATG

3 CATGACCACTAGGAGCATCTTTGGCGACAGTGGGCTGGAACATACCGGGAATGTCGACTAGGGGAATAAATCTTTGGGCACCTAGTGGTCATG

3 CATGACCACTAGGAGCATCTTTGGCGACGGTGTGGGGAATTTGTTTCGGCGGTGCTACTAGGGGAATAAATCTTTGGGCACCTAGTGGTCATG

3 CATGACCACTAGGAGCATCTTTGGCGAATGGCCGGCACGACCTTCTAGTCCTCGGTACTAGGGGAATAAATCTTTGGGCACCTAGTGGTCATG

3 CATGACCACTAGGAGCATCTTTGGCGACGGCAAAGGTTCGACGTGGTTGTGTCGCCGCTAGGGGAATAAATCTTTGGGCACCTAGTGGTCATG

3 CATGACCACTAGGAGCATCTTTGGCGACCCGGAGCGCTTGCGCGTACTGCGGGTGGACTAGGGGAATAAATCTTTGGGCACCTAGTGGTCATG

3 CATGACCACTAGGAGCATCTTTGGCGAGATCGGGAGAATCGGTGGCATTGGTGCCCCCTAGGGGAATAAATCTTTGGGCACCTAGTGGTCATG

3 CATGACCACTAGGAGCATCTTTGGCGATGGGTGCTAACGGGGCTAGTGAGCCCTACACTAGGGGAATAAATCTTTGGGCACCTAGTGGTCATG

3 CATGACCACTAGGAGCATCTTTGGCGAGACCGGAGGATGCGGGAAGGCACGGTGAGCCTAGGGGAATAAATCTTTGGGCACCTAGTGGTCATG

3 CATGACCACTAGGAGCATCTTTGGCGATCTACACGAAGTGTGCGAAGTGGACAACGACTAGGGGAATAAATCTTTGGGCACCTAGTGGTCATG

3 CATGACCACTAGGAGCATCTTTGGCGATCCGAGGCAGGCGTTTGGTAACAGCGAGCACTAGGGGAATAAATCTTTGGGCACCTAGTGGTCATG

3 CATGACCACTAGGAGCATCTTTGGCGACGGCATTTGTTAGGCTTTGGACAGTGTCACCTAGGGGAATAAATCTTTGGGCACCTAGTGGTCATG

3 CATGACCACTAGGAGCATCTTTGGCGACTGCGGACGTATGGTTCGCTCCGTAGTGGACTAGGGGAATAAATCTTTGGGCACCTAGTGGTCATG

3 CATGACCACTAGGAGCATCTTTGGCGAAAGTGCAAGATATGCCGCCTGGAGCGTAGGCTAGGGGAATAAATCTTTGGGCACCTAGTGGTCATG

3 CATGACCACTAGGAGCATCTTTGGCGAGATAGGGAGAATCGGTGGCATAGGTGTCTCCTAGGGGAATAAATCTTTGGGCACCTAGTGGTCATG

3 CATGACCACTAGGAGCATCTTTGGCGATACGGTGCGGTACGATCCGTAAAGTGGAGACTAGGGGAATAAATCTTTGGGCACCTAGTGGTCATG

3 CATGACCACTAGGAGCATCTTTGGCGAAGCGTACGAGGTAGGTCGACGTATAAGACCCTAGGGGAATAAATCTTTGGGCACCTAGTGGTCATG

3 CATGACCACTAGGAGCATCTTTGGCGAAGACTAAGATAAGATCCAAATTAAATTCGCCTAGGGGAATAAATCTTTGGGCACCTAGTGGTCATG

3 CATGACCACTAGGAGCATCTTTGGCGAGCCGATTGTGTAACAATGTGTTCTGGGTAACTAGGGGAATAAATCTTTGGGCACCTAGTGGTCATG

3 CATGACCACTAGGAGCATCTTTGGCGAACCGGGCAGGCAGGCCTGCTCGAGGTGGAGCTAGGGGAATAAATCTTTGGGCACCTAGTGGTCATG

3 CATGACCACTAGGAGCATCTTTGGCGATGGGTGCTGGCGGGGCTAGTGAGCCCTACACTAGGGGAATAAATCTTTGGGCACCTAGTGGTCATG

3 CATGACCACTAGGAGCATCTTTGGCGATAGCCAGTGATGTGAGTAGTGGCCGATCGCCTAGGGGAATAAATCTTTGGGCACCTAGTGGTCATG

3 CATGACCACTAGGAGCATCTTTGGCGACATCGGGAGAATCGGTGGCATTGGTGTCTCCTAGGGGAATAAATCTTTGGGCACCTAGTGGTCATG

3 CATGACCACTAGGAGCATCTTTGGCGACTCGTGGGCTGGTAGGCGGTACTTGAGTCACTAGGGGAATAAATCTTTGGGCACCTAGTGGTCATG

3 CATGACCACTAGGAGCATCTTTGGCGAAATGGACGGACAGGTCTCGACTGAAATCGCCTAGGGGAATAAATCTTTGGGCACCTAGTGGTCATG

3 CATGACCACTAGGAGCATCTTTGGCGATCCTCGTCAGATGGTGAGGCAGACGTTTGGCTAGGGGAATAAATCTTTGGGCACCTAGTGGTCATG

3 CATGACCACTAGGAGCATCTTTGGCGACGTAGGGGGCATGGGTCAGAAGGTCGCCAGCTAGGGGAATAAATCTTTGGGCACCTAGTGGTCATG

3 CATGACCACTAGGAGCATCTTTGGCGAACGGCCGGCACGGCCTTCTAGTCCTCGGCACTAGGGGAATAAATCTTTGGGCACCTAGTGGTCATG

3 CATGACCACTAGGAGCATCTTTGGCGACGGCGTGGGGCCATCTGTGTGGCGGATCCCCTAGGGGAATAAATCTTTGGGCACCTAGTGGTCATG

3 CATGACCACTAGGAGCATCTTTGGCGACCCGTGTGAAGCTGTGCTATTACGGGTGGACTAGGGGAATAAATCTTTGGGCACCTAGTGGTCATG

3 CATGACCACTAGGAGCATCTTTGGCGAACGTGTACGGCAGTGAACGCGAAGTGGAGCCTAGGGGAATAAATCTTTGGGCACCTAGTGGTCATG

3 CATGACCACTAGGAGCATCTTTGGCGAGATCGGGAGAATCGGTGGCATAGGTGTCTACTAGGGGAATAAATCTTTGGGCACCTAGTGGTCATG

3 CATGACCACTAGGAGCATCTTTGGCGAGGGGTAGGGGGCCGCTCGCGCCGGCGTGCGCTAGGGGAATAAATCTTTGGGCACCTAGTGGTCATG

3 CATGACCACTAGGAGCATCTTTGGCGACCCGCAAAGCTCCGTTCGTTCGCGGGTGGACTAGGGGAATAAATCTTTGGGCACCTAGTGGTCATG

3 CATGACCACTAGGAGCATCTTTGGCGAGGGTAGTGGGCAGGTAGATACATCGCACTACTAGGGGAATAAATCTTTGGGCACCTAGTGGTCATG

3 CATGACCACTAGGAGCATCTTTGGCGAGTAGGGTCCAAACGTAAGCTATGCACGAGACTAGGGGAATAAATCTTTGGGCACCTAGTGGTCATG

3 CATGACCACTAGGAGCATCTTTGGCGACCCGAGACGTGCTTGCCGTTACAGGGTGGACTAGGGGAATAAATCTTTGGGCACCTAGTGGTCATG

3 CATGACCACTAGGAGCATCTTTGGCGAGAGACTAAAAGATACAAGTGGCCGAGCCTGCTAGGGGAATAAATCTTTGGGCACCTAGTGGTCATG

3 CATGACCACTAGGAGCATCTTTGGCGATCAGGCCGCCAGGGCGTACGACGTGTGGTTCTAGGGGAATAAATCTTTGGGCACCTAGTGGTCATG

3 CATGACCACTAGGAGCATCTTTGGCGAAGGTTGTAGGACTTCAAGTGGATCTCATAGCTAGGGGAATAAATCTTTGGGCACCTAGTGGTCATG

3 CATGACCACTAGGAGCATCTTTGGCGAACGGAATGTGTTGCGGTGGTCTGTGAATCCCTAGGGGAATAAATCTTTGGGCACCTAGTGGTCATG

3 CATGACCACTAGGAGCATCTTTGGCGAGGGGTCACGGGAATGTAATTGCGCTTAAGACTAGGGGAATAAATCTTTGGGCACCTAGTGGTCATG

3 CATGACCACTAGGAGCATCTTTGGCGAGTATTGGCTGGTAGGTTGCGTAGTGGGGAGCTAGGGGAATAAATCTTTGGGCACCTAGTGGTCATG

3 CATGACCACTAGGAGCATCTTTGGCGACTCGTGGTCTTTACCCCCGTAGAGGGTTGTCTAGGGGAATAAATCTTTGGGCACCTAGTGGTCATG

3 CATGACCACTAGGAGCATCTTTGGCGATGGGTTGCTCATGGTTGTGCGCAGGCGGAGCTAGGGGAATAAATCTTTGGGCACCTAGTGGTCATG

3 CATGACCACTAGGAGCATCTTTGGCGACCCTGGCGTGGGGATCCTGGTTGCGGCGACCTAGGGGAATAAATCTTTGGGCACCTAGTGGTCATG

3 CATGACCACTAGGAGCATCTTTGGCGACGTGGTAGCAGCACGGTCACTGGTAGCGCCCTAGGGGAATAAATCTTTGGGCACCTAGTGGTCATG

3 CATGACCACTAGGAGCATCTTTGGCGAGATCGGGAGAATCGATGGCATTGGTGCCTCCTAGGGGAATAAATCTTTGGGCACCTAGTGGTCATG

3 CATGACCACTAGGAGCATCTTTGGCGAGCCTTGCTTGGGAGGTTGCTCAACCAGTTCCTAGGGGAATAAATCTTTGGGCACCTAGTGGTCATG

3 CATGACCACTAGGAGCATCTTTGGCGAGGTCGCGCGGAGTGGGTTCGTATAGACTAGCTAGGGGAATAAATCTTTGGGCACCTAGTGGTCATG

3 CATGACCACTAGGAGCATCTTTGGCGAGTAGTGGCAGAGAGAGATCCACATCGCAGGCTAGGGGAATAAATCTTTGGGCACCTAGTGGTCATG

3 CATGACCACTAGGAGCATCTTTGGCGAGGGATAGGCGTAACACGCTTTTGACGAGTCCTAGGGGAATAAATCTTTGGGCACCTAGTGGTCATG

3 CATGACCACTAGGAGCATCTTTGGCGACGACATAGAGAAGGGCAGATAGATAGTCGACTAGGGGAATAAATCTTTGGGCACCTAGTGGTCATG

3 CATGACCACTAGGAGCATCTTTGGCGAATGGCCGGCACGGCCTTCTAGTCCTTTGTACTAGGGGAATAAATCTTTGGGCACCTAGTGGTCATG

3 CATGACCACTAGGAGCATCTTTGGCGACACCAGACGGCTAAGAGGTGGAAAGTGGAGCTAGGGGAATAAATCTTTGGGCACCTAGTGGTCATG

3 CATGACCACTAGGAGCATCTTTGGCGAACGGCAGGTGTTGCGGTGGTTTGTGAATACCTAGGGGAATAAATCTTTGGGCACCTAGTGGTCATG

3 CATGACCACTAGGAGCATCTTTGGCGAGTGGTCTCTGGATAGCTCGTCTCCCCTTGCCTAGGGGAATAAATCTTTGGGCACCTAGTGGTCATG

3 CATGACCACTAGGAGCATCTTTGGCGACCCATCTAGGTGTAAACAGATGAGGTGGCTCTAGGGGAATAAATCTTTGGGCACCTAGTGGTCATG

3 CATGACCACTAGGAGCATCTTTGGCGAGTACGCATCGCGGGTTGGTTAGGTTAGCTCCTAGGGGAATAAATCTTTGGGCACCTAGTGGTCATG

3 CATGACCACTAGGAGCATCTTTGGCGATAGTCGTGTACGGTCGAGCGAAAGCGATAGCTAGGGGAATAAATCTTTGGGCACCTAGTGGTCATG

3 CATGACCACTAGGAGCATCTTTGGCGACCCGCATGGAGGGGAACGACGCGAGGTGGACTAGGGGAATAAATCTTTGGGCACCTAGTGGTCATG

3 CATGACCACTAGGAGCATCTTTGGCGAGCGTGCTCACTGACGCGTGTGGTCGAGCTCCTAGGGGAATAAATCTTTGGGCACCTAGTGGTCATG

3 CATGACCACTAGGAGCATCTTTGGCGACCCGAGCTTAAGAAGGATGTCTCGGGTGGACTAGGGGAATAAATCTTTGGGCACCTAGTGGTCATG

3 CATGACCACTAGGAGCATCTTTGGCGAGGGAGGGCTCCGGCAGCGGTGTGAATGCGCCTAGGGGAATAAATCTTTGGGCACCTAGTGGTCATG

3 CATGACCACTAGGAGCATCTTTGGCGACGGGCAGGGCAGCGGTCCTCAACTAGTCGACTAGGGGAATAAATCTTTGGGCACCTAGTGGTCATG

3 CATGACCACTAGGAGCATCTTTGGCGAGAATACTCCGGATTCGGGGACCAGTTGCTGCTAGGGGAATAAATCTTTGGGCACCTAGTGGTCATG

3 CATGACCACTAGGAGCATCTTTGGCGACCCGTGTAACAGGGTACTCGGTTGTGGAGCCTAGGGGAATAAATCTTTGGGCACCTAGTGGTCATG

3 CATGACCACTAGGAGCATCTTTGGCGACGTAGGGGGCATTGGGATTCAGAGAGTCGACTAGGGGAATAAATCTTTGGGCACCTAGTGGTCATG

3 CATGACCACTAGGAGCATCTTTGGCGACTAAGTGGCCAGCGCGTTAAATGGGGTAGACTAGGGGAATAAATCTTTGGGCACCTAGTGGTCATG

3 CATGACCACTAGGAGCATCTTTGGCGAAGCGAACAGCATCAGCAAGTGGATTGTCTGCTAGGGGAATAAATCTTTGGGCACCTAGTGGTCATG

3 CATGACCACTAGGAGCATCTTTGGCGAGTGGTCAGAGATGTCAGTCGCAGTGACTCGCTAGGGGAATAAATCTTTGGGCACCTAGTGGTCATG

3 CATGACCACTAGGAGCATCTTTGGCGACTTGGGTTGGTAGGATGAGTGTTAGTCGAACTAGGGGAATAAATCTTTGGGCACCTAGTGGTCATG

3 CATGACCACTAGGAGCATCTTTGGCGAGCCTTCGTATGTGTACGTGCTGTCTGGGTCCTAGGGGAATAAATCTTTGGGCACCTAGTGGTCATG

3 CATGACCACTAGGAGCATCTTTGGCGACATCGATCACTATGAGTGAGACGTTGTGGACTAGGGGAATAAATCTTTGGGCACCTAGTGGTCATG

3 CATGACCACTAGGAGCATCTTTGGCGATTAAGTGGTCAACGCGTTAAATGGGGTAGACTAGGGGAATAAATCTTTGGGCACCTAGTGGTCATG

3 CATGACCACTAGGAGCATCTTTGGCGAGATCGGTAGAATAGGTGGCATTGGTGTCTCCTAGGGGAATAAATCTTTGGGCACCTAGTGGTCATG

3 CATGACCACTAGGAGCATCTTTGGCGAGATCGGGAGAATAGTTGGCATTGGTGTCTCCTAGGGGAATAAATCTTTGGGCACCTAGTGGTCATG

3 CATGACCACTAGGAGCATCTTTGGCGAACGGCAGGTGTTGCGGTGGTCTGTGGATCACTAGGGGAATAAATCTTTGGGCACCTAGTGGTCATG

3 CATGACCACTAGGAGCATCTTTGGCGATCAATCGGTTGGAAGGGAGTATTTTGTCGGCTAGGGGAATAAATCTTTGGGCACCTAGTGGTCATG

3 CATGACCACTAGGAGCATCTTTGGCGACGATGATGATGGGCAGGCTGGGGAGTGTTGCTAGGGGAATAAATCTTTGGGCACCTAGTGGTCATG

3 CATGACCACTAGGAGCATCTTTGGCGACCTGGTGCGGCAGAGTTAGTTGGGGGTCGACTAGGGGAATAAATCTTTGGGCACCTAGTGGTCATG

3 CATGACCACTAGGAGCATCTTTGGCGATCCCAGGCGGTGTCAACCCCTGGCGTGGAGCTAGGGGAATAAATCTTTGGGCACCTAGTGGTCATG

3 CATGACCACTAGGAGCATCTTTGGCGACCCTTAGCGTTGACTAACGCAAGAGGTGGACTAGGGGAATAAATCTTTGGGCACCTAGTGGTCATG

3 CATGACCACTAGGAGCATCTTTGGCGAAGTGCAAATATGCGTACCATAGTTAATTCGCTAGGGGAATAAATCTTTGGGCACCTAGTGGTCATG

3 CATGACCACTAGGAGCATCTTTGGCGAAGGTGTGGGGAACTTGTTTCGGCGGTGCTACTAGGGGAATAAATCTTTGGGCACCTAGTGGTCATG

3 CATGACCACTAGGAGCATCTTTGGCGACCCGCTTACCGTATTTATCGTGCGGGTGGACTAGGGGAATAAATCTTTGGGCACCTAGTGGTCATG

3 CATGACCACTAGGAGCATCTTTGGCGACCGTAGGAAACCAAAAGTGGCCTTGCTAGCCTAGGGGAATAAATCTTTGGGCACCTAGTGGTCATG

3 CATGACCACTAGGAGCATCTTTGGCGACTCATGTGGTCTCGTACCACAGGTAGTGGACTAGGGGAATAAATCTTTGGGCACCTAGTGGTCATG

3 CATGACCACTAGGAGCATCTTTGGCGAGCCGATTGTGCAACAATGTGTCCTGGGTAACTAGGGGAATAAATCTTTGGGCACCTAGTGGTCATG

3 CATGACCACTAGGAGCATCTTTGGCGAGAAGACTCTGGATTCGGGGACCAGTTGGTGCTAGGGGAATAAATCTTTGGGCACCTAGTGGTCATG

3 CATGACCACTAGGAGCATCTTTGGCGAACGTCGTCGGCAACGAGGCGATAAGTGGAGCTAGGGGAATAAATCTTTGGGCACCTAGTGGTCATG

3 CATGACCACTAGGAGCATCTTTGGCGAGACTAGAGGAGAGCAGCGATCTCGAGTGGCCTAGGGGAATAAATCTTTGGGCACCTAGTGGTCATG

3 CATGACCACTAGGAGCATCTTTGGCGACCCGTGAGGTGAACACTGGTAACGGGTGGACTAGGGGAATAAATCTTTGGGCACCTAGTGGTCATG

3 CATGACCACTAGGAGCATCTTTGGCGATCAGCATAGCGAAGTCAGAGGAGACGGGGCCTAGGGGAATAAATCTTTGGGCACCTAGTGGTCATG

3 CATGACCACTAGGAGCATCTTTGGCGACGTCAGATATGCATATAGATGGGAAGTGGACTAGGGGAATAAATCTTTGGGCACCTAGTGGTCATG

3 CATGACCACTAGGAGCATCTTTGGCGATCTGGAGTGGGTGACCAAACGTTCAGAAGCCTAGGGGAATAAATCTTTGGGCACCTAGTGGTCATG

3 CATGACCACTAGGAGCATCTTTGGCGACGAGGGGCACGGGTGGTCATTGCGGGAAGACTAGGGGAATAAATCTTTGGGCACCTAGTGGTCATG

3 CATGACCACTAGGAGCATCTTTGGCGAGGACCGGTAAACTAGCATTGGCGTTGCGCTCTAGGGGAATAAATCTTTGGGCACCTAGTGGTCATG

3 CATGACCACTAGGAGCATCTTTGGCGATCAGGCCGCCAAGGCGTACGAAGTGTGGTTCTAGGGGAATAAATCTTTGGGCACCTAGTGGTCATG

3 CATGACCACTAGGAGCATCTTTGGCGAGAAGACTCTGGATTCGGGGACCACTTGCTGCTAGGGGAATAAATCTTTGGGCACCTAGTGGTCATG

3 CATGACCACTAGGAGCATCTTTGGCGAGCATCAGACGTAGTCCGAGCAGACGTTTGGCTAGGGGAATAAATCTTTGGGCACCTAGTGGTCATG

3 CATGACCACTAGGAGCATCTTTGGCGACGAGTGGGGTGTACACCCCATCGAAGTGGACTAGGGGAATAAATCTTTGGGCACCTAGTGGTCATG

3 CATGACCACTAGGAGCATCTTTGGCGAGCATAAGATGGGCATGGTGGCAGAGCTCGACTAGGGGAATAAATCTTTGGGCACCTAGTGGTCATG

3 CATGACCACTAGGAGCATCTTTGGCGATTGGCCGGCACGGCCTCCTAGTCCTCGGTACTAGGGGAATAAATCTTTGGGCACCTAGTGGTCATG

3 CATGACCACTAGGAGCATCTTTGGCGACGTGGGTTGGCTGGTAGTATTGCGTGCGCTCTAGGGGAATAAATCTTTGGGCACCTAGTGGTCATG

3 CATGACCACTAGGAGCATCTTTGGCGACACTGTTTAACTGTGTTAAACGGTTGTGGACTAGGGGAATAAATCTTTGGGCACCTAGTGGTCATG

3 CATGACCACTAGGAGCATCTTTGGCGACTACTGTCGACAGGTTGCGGGAACTGGGAGCTAGGGGAATAAATCTTTGGGCACCTAGTGGTCATG

3 CATGACCACTAGGAGCATCTTTGGCGATGCCCCCTCGGGGTCTGGATGCAGTTTTAGCTAGGGGAATAAATCTTTGGGCACCTAGTGGTCATG

3 CATGACCACTAGGAGCATCTTTGGCGAGGCGAGTCCAACGGCCTTCTTGGCCTGCGACTAGGGGAATAAATCTTTGGGCACCTAGTGGTCATG

3 CATGACCACTAGGAGCATCTTTGGCGACCCAGTTGGCTCCGCAGCAACTTGGGTGGACTAGGGGAATAAATCTTTGGGCACCTAGTGGTCATG

3 CATGACCACTAGGAGCATCTTTGGCGAACGGTAGGTGTTGCGGTGGTCTGTGTATCCCTAGGGGAATAAATCTTTGGGCACCTAGTGGTCATG

3 CATGACCACTAGGAGCATCTTTGGCGACGTGGGCAACAAGGGGAGCGAGCAAGGAGACTAGGGGAATAAATCTTTGGGCACCTAGTGGTCATG

3 CATGACCACTAGGAGCATCTTTGGCGATAGGCTTGTACGGTCGAGCGAAAGCGATAGCTAGGGGAATAAATCTTTGGGCACCTAGTGGTCATG

3 CATGACCACTAGGAGCATCTTTGGCGAGATCGGTAGAATCTGTGGCATTGGTGTCTCCTAGGGGAATAAATCTTTGGGCACCTAGTGGTCATG

3 CATGACCACTAGGAGCATCTTTGGCGACCAAATGGACCGCGGTCCAATGGAGTGGAGCTAGGGGAATAAATCTTTGGGCACCTAGTGGTCATG

3 CATGACCACTAGGAGCATCTTTGGCGAAGCGCGTTGCAAGGTCCGCGAGTGTGGACCCTAGGGGAATAAATCTTTGGGCACCTAGTGGTCATG

3 CATGACCACTAGGAGCATCTTTGGCGACGGTGTGGGTACTTTAGTTAGGCGGTTAGTCTAGGGGAATAAATCTTTGGGCACCTAGTGGTCATG

3 CATGACCACTAGGAGCATCTTTGGCGAGACCGAGAGAATCGGTGGCATTGGTGTCTCCTAGGGGAATAAATCTTTGGGCACCTAGTGGTCATG

3 CATGACCACTAGGAGCATCTTTGGCGACGGCGTGGGGCTATTGATGCGGCGGCAATCCTAGGGGAATAAATCTTTGGGCACCTAGTGGTCATG

3 CATGACCACTAGGAGCATCTTTGGCGACCCAGTAGGAGTGATAGCCCACTGGGTGGACTAGGGGAATAAATCTTTGGGCACCTAGTGGTCATG

3 CATGACCACTAGGAGCATCTTTGGCGACAGCGCAGGGCATGCGACCTCCAATTGGTCCTAGGGGAATAAATCTTTGGGCACCTAGTGGTCATG

3 CATGACCACTAGGAGCATCTTTGGCGATCGACAAAGTCTCGAATGTGATCTGAGCCGCTAGGGGAATAAATCTTTGGGCACCTAGTGGTCATG

3 CATGACCACTAGGAGCATCTTTGGCGACGAGGCGTGTCTGGTTATGGCGGGCGGAACCTAGGGGAATAAATCTTTGGGCACCTAGTGGTCATG

3 CATGACCACTAGGAGCATCTTTGGCGACACGTGGTCCTTGGGGCAATCATACGAATGCTAGGGGAATAAATCTTTGGGCACCTAGTGGTCATG

3 CATGACCACTAGGAGCATCTTTGGCGATTAGGCCGCCAGGGCGTACGAAGTGTGGTACTAGGGGAATAAATCTTTGGGCACCTAGTGGTCATG

3 CATGACCACTAGGAGCATCTTTGGCGATCCTCGTCAGATGGTGACGCAGACGTTTGGCTAGGGGAATAAATCTTTGGGCACCTAGTGGTCATG

3 CATGACCACTAGGAGCATCTTTGGCGATATTGTTGAAGCGGGTTGCCGACGCTGGTTCTAGGGGAATAAATCTTTGGGCACCTAGTGGTCATG

3 CATGACCACTAGGAGCATCTTTGGCGACACGTGGTCGGCTCGGTATATGTGCTTTCTCTAGGGGAATAAATCTTTGGGCACCTAGTGGTCATG

3 CATGACCACTAGGAGCATCTTTGGCGAGTAAGCGCGGTCCCTCGGACCTCACAGTTGCTAGGGGAATAAATCTTTGGGCACCTAGTGGTCATG

3 CATGACCACTAGGAGCATCTTTGGCGAAGCGTGTTGCGAGGTCCGCGTGTGTGGACCCTAGGGGAATAAATCTTTGGGCACCTAGTGGTCATG

3 CATGACCACTAGGAGCATCTTTGGCGAGATAGTGAGAATCGGTGGCATTGGTGTCTCCTAGGGGAATAAATCTTTGGGCACCTAGTGGTCATG

3 CATGACCACTAGGAGCATCTTTGGCGACGCGCAACGGACGGGTGCGTTGTAAGTGGACTAGGGGAATAAATCTTTGGGCACCTAGTGGTCATG

3 CATGACCACTAGGAGCATCTTTGGCGACCCATCCGTGTGTATACCCGGTGTGGTGGACTAGGGGAATAAATCTTTGGGCACCTAGTGGTCATG

3 CATGACCACTAGGAGCATCTTTGGCGAGATCGGGAGAATCGGTGGAATTGGAGTCTCCTAGGGGAATAAATCTTTGGGCACCTAGTGGTCATG

3 CATGACCACTAGGAGCATCTTTGGCGAGATCGGGATAATCGGTGGCATTGTTGTCTCCTAGGGGAATAAATCTTTGGGCACCTAGTGGTCATG

2 CATGACCACTAGGAGCATCTTTGGCGACGGAGGGTCATCGGGACGTGGTCTAGTCGACTAGGGGAATAAATCTTTGGGCACCTAGTGGTCATG

2 CATGACCACTAGGAGCATCTTTGGCGAGTGGGCCCACCTAGCTGTGGCTGGCCCGAGCTAGGGGAATAAATCTTTGGGCACCTAGTGGTCATG

2 CATGACCACTAGGAGCATCTTTGGCGAGATCGGGAGAGTCGGTGGCATTTGTGTCTCCTAGGGGAATAAATCTTTGGGCACCTAGTGGTCATG

2 CATGACCACTAGGAGCATCTTTGGCGAGTCGACAGGGATGCAACGCGTGGACGAGAACTAGGGGAATAAATCTTTGGGCACCTAGTGGTCATG

2 CATGACCACTAGGAGCATCTTTGGCGACCCGACACTGAGCAGCTGTAGTAGGGTGGACTAGGGGAATAAATCTTTGGGCACCTAGTGGTCATG

2 CATGACCACTAGGAGCATCTTTGGCGAGATCGGGAGAATCGGTGGCATTGTTTTCTCCTAGGGGAATAAATCTTTGGGCACCTAGTGGTCATG

2 CATGACCACTAGGAGCATCTTTGGCGAACACGAGAGTCAAGTGCGTATGGCGTGGAGCTAGGGGAATAAATCTTTGGGCACCTAGTGGTCATG

2 CATGACCACTAGGAGCATCTTTGGCGAGAGGGTGCGGTAGAACCAGTAACGGCGAGACTAGGGGAATAAATCTTTGGGCACCTAGTGGTCATG

2 CATGACCACTAGGAGCATCTTTGGCGATTGGCGTGACTGTCCGCTAAAGCTGTTCGCCTAGGGGAATAAATCTTTGGGCACCTAGTGGTCATG

2 CATGACCACTAGGAGCATCTTTGGCGACGGACGAACCAACGAGTTGCTAAGAGGCGCCTAGGGGAATAAATCTTTGGGCACCTAGTGGTCATG

2 CATGACCACTAGGAGCATCTTTGGCGACAAGGATGGTCGGCCGTTAGCTGAAGGAGCCTAGGGGAATAAATCTTTGGGCACCTAGTGGTCATG

2 CATGACCACTAGGAGCATCTTTGGCGATCGCACAGGTTGGAACGGGTAGTATATCGCCTAGGGGAATAAATCTTTGGGCACCTAGTGGTCATG

2 CATGACCACTAGGAGCATCTTTGGCGATTCGCCGGTGCCGCGGCACTGTCAGATTGCCTAGGGGAATAAATCTTTGGGCACCTAGTGGTCATG

2 CATGACCACTAGGAGCATCTTTGGCGAGATCGGGGGAATCGGTGGCATTGGTGTCTACTAGGGGAATAAATCTTTGGGCACCTAGTGGTCATG

2 CATGACCACTAGGAGCATCTTTGGCGAGACCAAGTGTCCGCACTACGTGGCTGAGCTCTAGGGGAATAAATCTTTGGGCACCTAGTGGTCATG

2 CATGACCACTAGGAGCATCTTTGGCGATGTGGTCAGGCAAATTACTGGTACGAAGAGCTAGGGGAATAAATCTTTGGGCACCTAGTGGTCATG

2 CATGACCACTAGGAGCATCTTTGGCGAACGAGGTAAGACGGACGATGGTTCAGTGAGCTAGGGGAATAAATCTTTGGGCACCTAGTGGTCATG

2 CATGACCACTAGGAGCATCTTTGGCGAGGGGTCATTGGAATGTAATTGCGCTTAAGACTAGGGGAATAAATCTTTGGGCACCTAGTGGTCATG

2 CATGACCACTAGGAGCATCTTTGGCGAGATCGTGAGAATCGGTGGCATTGGTGTCTACTAGGGGAATAAATCTTTGGGCACCTAGTGGTCATG

2 CATGACCACTAGGAGCATCTTTGGCGAACGGCCGGCGCGGCCTTCTAGTCCTCGGTACTAGGGGAATAAATCTTTGGGCACCTAGTGGTCATG

2 CATGACCACTAGGAGCATCTTTGGCGACGGTGTGGGGAAGTTATATCGGCGGACGCACTAGGGGAATAAATCTTTGGGCACCTAGTGGTCATG

2 CATGACCACTAGGAGCATCTTTGGCGATCGGCCGTTGGGAGGGAGTACGGTTCGACACTAGGGGAATAAATCTTTGGGCACCTAGTGGTCATG

2 CATGACCACTAGGAGCATCTTTGGCGAGCCGGCCGCTATAGTTGGCTTGTCTGGGTCCTAGGGGAATAAATCTTTGGGCACCTAGTGGTCATG

2 CATGACCACTAGGAGCATCTTTGGCGAGCGTGCAAGATATGCCGCCTGGAGCGTAGGCTAGGGGAATAAATCTTTGGGCACCTAGTGGTCATG

2 CATGACCACTAGGAGCATCTTTGGCGACCCCGCTAGGAGTTCACAACAGCGGGTGGACTAGGGGAATAAATCTTTGGGCACCTAGTGGTCATG

2 CATGACCACTAGGAGCATCTTTGGCGAGAACGGGAGAATCGGTGGCATTTGTGTCTCCTAGGGGAATAAATCTTTGGGCACCTAGTGGTCATG

2 CATGACCACTAGGAGCATCTTTGGCGACACGTCTAGCAGGGATGTAGCAATGCGCGCCTAGGGGAATAAATCTTTGGGCACCTAGTGGTCATG

2 CATGACCACTAGGAGCATCTTTGGCGAATAGGTGATAAAGCCAGCTTTATAATGAGACTAGGGGAATAAATCTTTGGGCACCTAGTGGTCATG

2 CATGACCACTAGGAGCATCTTTGGCGAGGCGAGTCCAGTGGCCTTCTTGGCCTGCGACTAGGGGAATAAATCTTTGGGCACCTAGTGGTCATG

2 CATGACCACTAGGAGCATCTTTGGCGAAGTGCGGTGGGCGTGTGGGTGGTAGCGACGCTAGGGGAATAAATCTTTGGGCACCTAGTGGTCATG

2 CATGACCACTAGGAGCATCTTTGGCGACACGTCGGCGCAGCAAAAGGACGTTGTGGACTAGGGGAATAAATCTTTGGGCACCTAGTGGTCATG

2 CATGACCACTAGGAGCATCTTTGGCGAGGCGAGTCCAGCGGCCTTCTTGGCATGCGACTAGGGGAATAAATCTTTGGGCACCTAGTGGTCATG

2 CATGACCACTAGGAGCATCTTTGGCGAATGGCCGGTTCGGCCTTCTAGTCCTCGGTACTAGGGGAATAAATCTTTGGGCACCTAGTGGTCATG

2 CATGACCACTAGGAGCATCTTTGGCGACCCTTTATGGTGCAACCAAAGGCGTGTCGTCTAGGGGAATAAATCTTTGGGCACCTAGTGGTCATG

2 CATGACCACTAGGAGCATCTTTGGCGATCCATACCTGGGAAGTCAATGGTGTGGAGCCTAGGGGAATAAATCTTTGGGCACCTAGTGGTCATG

2 CATGACCACTAGGAGCATCTTTGGCGAAGGGTGCTGTAAGCGCCGTCAGAGGAGCGCCTAGGGGAATAAATCTTTGGGCACCTAGTGGTCATG

2 CATGACCACTAGGAGCATCTTTGGCGATAGCGAAGTGCTGTGAACAGTGGTCAGCACCTAGGGGAATAAATCTTTGGGCACCTAGTGGTCATG

2 CATGACCACTAGGAGCATCTTTGGCGACCCGTGATGTCTAGCTATCAAACGGGTGGACTAGGGGAATAAATCTTTGGGCACCTAGTGGTCATG

2 CATGACCACTAGGAGCATCTTTGGCGAGACGGCAGGGTTCGTACAAAAGCGGATCGACTAGGGGAATAAATCTTTGGGCACCTAGTGGTCATG

2 CATGACCACTAGGAGCATCTTTGGCGAAGCAGTGCGGCGGGCTATGGGTTCCTCGAGCTAGGGGAATAAATCTTTGGGCACCTAGTGGTCATG

2 CATGACCACTAGGAGCATCTTTGGCGATACATTATATGCCTTGTAATAGAATGTCGCCTAGGGGAATAAATCTTTGGGCACCTAGTGGTCATG

2 CATGACCACTAGGAGCATCTTTGGCGAGGAGGTGCGACGGGGGCATGAGGGTCGAGACTAGGGGAATAAATCTTTGGGCACCTAGTGGTCATG

2 CATGACCACTAGGAGCATCTTTGGCGAGACCGGGAGAATCGGCGGCATTGGTGTCTCCTAGGGGAATAAATCTTTGGGCACCTAGTGGTCATG

2 CATGACCACTAGGAGCATCTTTGGCGAAGGCGAAGCACGCTGGTCGTGGTGGCGTCCCTAGGGGAATAAATCTTTGGGCACCTAGTGGTCATG

2 CATGACCACTAGGAGCATCTTTGGCGACCCAGCTTAGGATCATGCTGGAGTGTTGCACTAGGGGAATAAATCTTTGGGCACCTAGTGGTCATG

2 CATGACCACTAGGAGCATCTTTGGCGAGGGAGGGCGCCGGTAGCGGTGTGAATGCGACTAGGGGAATAAATCTTTGGGCACCTAGTGGTCATG

2 CATGACCACTAGGAGCATCTTTGGCGAGGGTGTCACGAAGTCACGAGTACGGCGAGACTAGGGGAATAAATCTTTGGGCACCTAGTGGTCATG

2 CATGACCACTAGGAGCATCTTTGGCGACGTAGGGGGCATGGGTCAGAAAGTCGCCAGCTAGGGGAATAAATCTTTGGGCACCTAGTGGTCATG

2 CATGACCACTAGGAGCATCTTTGGCGAGGTACGGCAGTATGCCGGGAGTAAATCGCACTAGGGGAATAAATCTTTGGGCACCTAGTGGTCATG

2 CATGACCACTAGGAGCATCTTTGGCGAGGTCCGAAACTGTAGGGCAGAAGATCGAGCCTAGGGGAATAAATCTTTGGGCACCTAGTGGTCATG

2 CATGACCACTAGGAGCATCTTTGGCGAGGGAGGGCGCCGGCAGCGGTGTGAATGTGCCTAGGGGAATAAATCTTTGGGCACCTAGTGGTCATG

2 CATGACCACTAGGAGCATCTTTGGCGAGGGTTCTAGCACCGGGTCCGCGACACGAGCCTAGGGGAATAAATCTTTGGGCACCTAGTGGTCATG

2 CATGACCACTAGGAGCATCTTTGGCGAGATCGGGAGAATCGTTGGAATTGGTGTCTCCTAGGGGAATAAATCTTTGGGCACCTAGTGGTCATG

2 CATGACCACTAGGAGCATCTTTGGCGAACCGATCCTGGCGGATTATCAGGGTTTTGCCTAGGGGAATAAATCTTTGGGCACCTAGTGGTCATG

2 CATGACCACTAGGAGCATCTTTGGCGAGCCTTGCTTGGGGGGTTGCTCCACCAGTTCCTAGGGGAATAAATCTTTGGGCACCTAGTGGTCATG

2 CATGACCACTAGGAGCATCTTTGGCGACCCAGACAGCGGTGAGTCGTCTAGGGTGGACTAGGGGAATAAATCTTTGGGCACCTAGTGGTCATG

2 CATGACCACTAGGAGCATCTTTGGCGACTCGGTTCTGCTCAGTAGAACTGTAGTGGACTAGGGGAATAAATCTTTGGGCACCTAGTGGTCATG

2 CATGACCACTAGGAGCATCTTTGGCGACTACCATATCATCGCGAATATGGAGTGGAGCTAGGGGAATAAATCTTTGGGCACCTAGTGGTCATG

2 CATGACCACTAGGAGCATCTTTGGCGAGAGGGAATCGGTGGGAAGAGTGCCCTAAGACTAGGGGAATAAATCTTTGGGCACCTAGTGGTCATG

2 CATGACCACTAGGAGCATCTTTGGCGACGGTGAGGGGAACTTGTTTCGGCGGTGCTACTAGGGGAATAAATCTTTGGGCACCTAGTGGTCATG

2 CATGACCACTAGGAGCATCTTTGGCGACGCAGTGGGCTAAGTAATCGGGGGCGTCGACTAGGGGAATAAATCTTTGGGCACCTAGTGGTCATG

2 CATGACCACTAGGAGCATCTTTGGCGACCCTGCCCCCGCGAAGTAGGCAGAGGTGGACTAGGGGAATAAATCTTTGGGCACCTAGTGGTCATG

2 CATGACCACTAGGAGCATCTTTGGCGAGGAAGTCGGCTAGTTGCGTCAGTGGTCGAGCTAGGGGAATAAATCTTTGGGCACCTAGTGGTCATG

2 CATGACCACTAGGAGCATCTTTGGCGAACGGGTAGTGGCATGAGTATGGTTCTGTCCCTAGGGGAATAAATCTTTGGGCACCTAGTGGTCATG

2 CATGACCACTAGGAGCATCTTTGGCGATAGGGCAGGGCAGCGAGTCCTGTTTAACGCCTAGGGGAATAAATCTTTGGGCACCTAGTGGTCATG

2 CATGACCACTAGGAGCATCTTTGGCGACCCACTACGCATAGCATGTGTGAGGGTGGACTAGGGGAATAAATCTTTGGGCACCTAGTGGTCATG

2 CATGACCACTAGGAGCATCTTTGGCGACGGTGTACCAGGCACGGACGGCGGTCGCCTCTAGGGGAATAAATCTTTGGGCACCTAGTGGTCATG

2 CATGACCACTAGGAGCATCTTTGGCGACATAGTGGGCAATACATCGGGCTAAGTCGACTAGGGGAATAAATCTTTGGGCACCTAGTGGTCATG

2 CATGACCACTAGGAGCATCTTTGGCGAGATCGAAACGTGGCGACGTCGAAGTGGAGACTAGGGGAATAAATCTTTGGGCACCTAGTGGTCATG

2 CATGACCACTAGGAGCATCTTTGGCGAGATCGCGAGAATCGGTGGCATTGGTGTCTACTAGGGGAATAAATCTTTGGGCACCTAGTGGTCATG

2 CATGACCACTAGGAGCATCTTTGGCGAACGGAAGGTGTTGCGGTGGTTTGTGAATCCCTAGGGGAATAAATCTTTGGGCACCTAGTGGTCATG

2 CATGACCACTAGGAGCATCTTTGGCGATCCGGGCTGGTAGGGGGTACTCAGGTAGCTCTAGGGGAATAAATCTTTGGGCACCTAGTGGTCATG

2 CATGACCACTAGGAGCATCTTTGGCGACCCGGCAACAGGCACAGGCAGCAGGGTGGACTAGGGGAATAAATCTTTGGGCACCTAGTGGTCATG

2 CATGACCACTAGGAGCATCTTTGGCGATGGGGCCGTGCTATCTCCACACTCCGCGGGCTAGGGGAATAAATCTTTGGGCACCTAGTGGTCATG

2 CATGACCACTAGGAGCATCTTTGGCGATGGGTGCTTACGGCCGCCGCTGCGGCTACACTAGGGGAATAAATCTTTGGGCACCTAGTGGTCATG

2 CATGACCACTAGGAGCATCTTTGGCGACTTGGGGGCTGACTAGCAGGACAAAGACGACTAGGGGAATAAATCTTTGGGCACCTAGTGGTCATG

2 CATGACCACTAGGAGCATCTTTGGCGACCCTCCTGCGGGTGTGGGATGCGCTGTCGTCTAGGGGAATAAATCTTTGGGCACCTAGTGGTCATG

2 CATGACCACTAGGAGCATCTTTGGCGACGAGCAGTTCCCGGAACCTCGCGAAGTGGACTAGGGGAATAAATCTTTGGGCACCTAGTGGTCATG

2 CATGACCACTAGGAGCATCTTTGGCGAACACCCGACGAGGGGGGAGCAGACGTTTGGCTAGGGGAATAAATCTTTGGGCACCTAGTGGTCATG

2 CATGACCACTAGGAGCATCTTTGGCGATTACGTCGTCGTAAATAGAGGCCTCGCGACCTAGGGGAATAAATCTTTGGGCACCTAGTGGTCATG

2 CATGACCACTAGGAGCATCTTTGGCGAAAGGCAGGTGTAGCGGTGGTCTGTGAATCCCTAGGGGAATAAATCTTTGGGCACCTAGTGGTCATG

2 CATGACCACTAGGAGCATCTTTGGCGAGGGACACGGCCTCACTGAAGGTATACGAGACTAGGGGAATAAATCTTTGGGCACCTAGTGGTCATG

2 CATGACCACTAGGAGCATCTTTGGCGAGAGCATGCGCGTAGTGGCAAATATCGAGTACTAGGGGAATAAATCTTTGGGCACCTAGTGGTCATG

2 CATGACCACTAGGAGCATCTTTGGCGACCCAGTGCGGCTTGAACGGACTGTGGTGGACTAGGGGAATAAATCTTTGGGCACCTAGTGGTCATG

2 CATGACCACTAGGAGCATCTTTGGCGATGGGTGTGTAGCTGATGCGGACAGATGCGCCTAGGGGAATAAATCTTTGGGCACCTAGTGGTCATG

2 CATGACCACTAGGAGCATCTTTGGCGAACGGCAGGTGTTGCGGTGGTCTGTGGGTCCCTAGGGGAATAAATCTTTGGGCACCTAGTGGTCATG

2 CATGACCACTAGGAGCATCTTTGGCGAGTGGCTGAGGGCAAGGGATGGCGGTTGGAGCTAGGGGAATAAATCTTTGGGCACCTAGTGGTCATG

2 CATGACCACTAGGAGCATCTTTGGCGACTCCAGTTCAACTGTCGGGCAGTTTAAGGACTAGGGGAATAAATCTTTGGGCACCTAGTGGTCATG

2 CATGACCACTAGGAGCATCTTTGGCGAAGTCCAGGGGCACAGGGATGACGGAGCCAGCTAGGGGAATAAATCTTTGGGCACCTAGTGGTCATG

2 CATGACCACTAGGAGCATCTTTGGCGATTGGGATGTCCCATTTGGGACACTACGTTCCTAGGGGAATAAATCTTTGGGCACCTAGTGGTCATG

2 CATGACCACTAGGAGCATCTTTGGCGATCCTCGTCTGATGGTGAAGCAGACGTTTGGCTAGGGGAATAAATCTTTGGGCACCTAGTGGTCATG

2 CATGACCACTAGGAGCATCTTTGGCGAACACCGGAGTCGAAGGTGGACGTAGTGGAGCTAGGGGAATAAATCTTTGGGCACCTAGTGGTCATG

2 CATGACCACTAGGAGCATCTTTGGCGAGGTACAATATGTTGCGTTTTGTATCGCCTACTAGGGGAATAAATCTTTGGGCACCTAGTGGTCATG

2 CATGACCACTAGGAGCATCTTTGGCGAACGGCAGGTGTGGAGGTGGTCTGTGAATCCCTAGGGGAATAAATCTTTGGGCACCTAGTGGTCATG

2 CATGACCACTAGGAGCATCTTTGGCGACCCGACGACTAATGGAGTCCGGAGTGGAAGCTAGGGGAATAAATCTTTGGGCACCTAGTGGTCATG

2 CATGACCACTAGGAGCATCTTTGGCGACAATGTGGGCATCGCACCCTGAAAAGTCGACTAGGGGAATAAATCTTTGGGCACCTAGTGGTCATG

2 CATGACCACTAGGAGCATCTTTGGCGAGTTATGGTGCTGCCATCTCACGGAGTCAGACTAGGGGAATAAATCTTTGGGCACCTAGTGGTCATG

2 CATGACCACTAGGAGCATCTTTGGCGACGTAAGTGGGCATGCAACTGCATAGCTCCGCTAGGGGAATAAATCTTTGGGCACCTAGTGGTCATG

2 CATGACCACTAGGAGCATCTTTGGCGAAGGAACATGACTGGATGCAGCACTCTGAGACTAGGGGAATAAATCTTTGGGCACCTAGTGGTCATG

2 CATGACCACTAGGAGCATCTTTGGCGAAGCGTGTTGCAAGGTCCGCAAGTGTGGACCCTAGGGGAATAAATCTTTGGGCACCTAGTGGTCATG

2 CATGACCACTAGGAGCATCTTTGGCGACTGGCTTCGCTGCGAGATGACCTGATCGTACTAGGGGAATAAATCTTTGGGCACCTAGTGGTCATG

2 CATGACCACTAGGAGCATCTTTGGCGATGACGTCGTCGTGAATAGAGGCCTCGCGACCTAGGGGAATAAATCTTTGGGCACCTAGTGGTCATG

2 CATGACCACTAGGAGCATCTTTGGCGACCCGAATGACTACGATCTCGTGCGGGTGGACTAGGGGAATAAATCTTTGGGCACCTAGTGGTCATG

2 CATGACCACTAGGAGCATCTTTGGCGACCCGGGACGCGTGTGGACCGGAAGTGGAGACTAGGGGAATAAATCTTTGGGCACCTAGTGGTCATG

2 CATGACCACTAGGAGCATCTTTGGCGAAAGCCAGTGATGTCGAGTAGTGGCTGAGCTCTAGGGGAATAAATCTTTGGGCACCTAGTGGTCATG

2 CATGACCACTAGGAGCATCTTTGGCGACGAGCCTGGGCACGCGGCAGCTGCCGTCGACTAGGGGAATAAATCTTTGGGCACCTAGTGGTCATG

2 CATGACCACTAGGAGCATCTTTGGCGATCGGGTCCCAGTGATTGCTCATATGATCGACTAGGGGAATAAATCTTTGGGCACCTAGTGGTCATG

2 CATGACCACTAGGAGCATCTTTGGCGAACTAAACGGAGCACGAACGCAAATAGGAGCCTAGGGGAATAAATCTTTGGGCACCTAGTGGTCATG

2 CATGACCACTAGGAGCATCTTTGGCGACGGGGTGGGCCGCAGCGCGAGGGCAGTCGACTAGGGGAATAAATCTTTGGGCACCTAGTGGTCATG

2 CATGACCACTAGGAGCATCTTTGGCGATGGGGCCGTGCTATCTGCATACTCCGCGGGCTAGGGGAATAAATCTTTGGGCACCTAGTGGTCATG

2 CATGACCACTAGGAGCATCTTTGGCGACCCAGAATGTATGACAGTATACTGGGTGGACTAGGGGAATAAATCTTTGGGCACCTAGTGGTCATG

2 CATGACCACTAGGAGCATCTTTGGCGAGCGGAGCTGTACGGACCGAAATTGTGCGAGCTAGGGGAATAAATCTTTGGGCACCTAGTGGTCATG

2 CATGACCACTAGGAGCATCTTTGGCGAGATCGGGAGAATAGGTGGCATTGGTGTATCCTAGGGGAATAAATCTTTGGGCACCTAGTGGTCATG

2 CATGACCACTAGGAGCATCTTTGGCGAACTGCAGGGGTTGCGGTGGTCTGTGAATCCCTAGGGGAATAAATCTTTGGGCACCTAGTGGTCATG

2 CATGACCACTAGGAGCATCTTTGGCGACCCTGTTCCGGGACGGATAAAAAGGGTGGACTAGGGGAATAAATCTTTGGGCACCTAGTGGTCATG

2 CATGACCACTAGGAGCATCTTTGGCGAAGATACGATGGGCTTGTGACCCGCCTCGGCCTAGGGGAATAAATCTTTGGGCACCTAGTGGTCATG

2 CATGACCACTAGGAGCATCTTTGGCGACGAGTCGAGTCAGGACATCGACTAAGTGGACTAGGGGAATAAATCTTTGGGCACCTAGTGGTCATG

2 CATGACCACTAGGAGCATCTTTGGCGAGCGGTCAAGGTGCGGTCATAGACGATTCGACTAGGGGAATAAATCTTTGGGCACCTAGTGGTCATG

2 CATGACCACTAGGAGCATCTTTGGCGACACCGGAGGTCGTGGAAAGGTCTCAGTCGACTAGGGGAATAAATCTTTGGGCACCTAGTGGTCATG

2 CATGACCACTAGGAGCATCTTTGGCGATAAGACGTGAGGCGTGATGTCCCGGTGAGACTAGGGGAATAAATCTTTGGGCACCTAGTGGTCATG

2 CATGACCACTAGGAGCATCTTTGGCGACCCTTGGTGACATAGTACAGGTGTGGGAGACTAGGGGAATAAATCTTTGGGCACCTAGTGGTCATG

2 CATGACCACTAGGAGCATCTTTGGCGACTATGGAGATAACCGTTAATACATAAAAGACTAGGGGAATAAATCTTTGGGCACCTAGTGGTCATG

2 CATGACCACTAGGAGCATCTTTGGCGACGGCGTGGGGCCTCTAGCGCGGCGGGGATTCTAGGGGAATAAATCTTTGGGCACCTAGTGGTCATG

2 CATGACCACTAGGAGCATCTTTGGCGAGTATTGGCTGGTAGGTTGCGTATTCGGGAGCTAGGGGAATAAATCTTTGGGCACCTAGTGGTCATG

2 CATGACCACTAGGAGCATCTTTGGCGACCCGGAGGTTGCATCGCGTCAACGGGTGGACTAGGGGAATAAATCTTTGGGCACCTAGTGGTCATG

2 CATGACCACTAGGAGCATCTTTGGCGAATGGCCGGCACGGCCTTCAAGTCCTTGGTACTAGGGGAATAAATCTTTGGGCACCTAGTGGTCATG

2 CATGACCACTAGGAGCATCTTTGGCGAAAGGCAGGTGTTGCGGTGGTCTGTTAATCCCTAGGGGAATAAATCTTTGGGCACCTAGTGGTCATG

2 CATGACCACTAGGAGCATCTTTGGCGACCCGACCATTGAAAAAAGCGAGCGGGTGGACTAGGGGAATAAATCTTTGGGCACCTAGTGGTCATG

2 CATGACCACTAGGAGCATCTTTGGCGAGGAGTGGGCAAGGGGCAAAGTGTGTCGAGCCTAGGGGAATAAATCTTTGGGCACCTAGTGGTCATG

2 CATGACCACTAGGAGCATCTTTGGCGACGAAGGGGGCAGGGAAGGCTTGATGAGGAGCTAGGGGAATAAATCTTTGGGCACCTAGTGGTCATG

2 CATGACCACTAGGAGCATCTTTGGCGACAACCGGAGTAAGACTCCGGGATAGTGGAGCTAGGGGAATAAATCTTTGGGCACCTAGTGGTCATG

2 CATGACCACTAGGAGCATCTTTGGCGACTCTGTCCGGTACGGGCCCGCTATGGTTGTCTAGGGGAATAAATCTTTGGGCACCTAGTGGTCATG

2 CATGACCACTAGGAGCATCTTTGGCGACCCTGACGAGTGGTACGACGACAGCGGTGGCTAGGGGAATAAATCTTTGGGCACCTAGTGGTCATG

2 CATGACCACTAGGAGCATCTTTGGCGAGGAACAACATGATGCCGCGAGAGGACGAGACTAGGGGAATAAATCTTTGGGCACCTAGTGGTCATG

2 CATGACCACTAGGAGCATCTTTGGCGATGGGTGCTGACTGGGCTAGTGAGCCCTACACTAGGGGAATAAATCTTTGGGCACCTAGTGGTCATG

2 CATGACCACTAGGAGCATCTTTGGCGACCCCGGGCGTCAACGGCGTACGGTGGTGGACTAGGGGAATAAATCTTTGGGCACCTAGTGGTCATG

2 CATGACCACTAGGAGCATCTTTGGCGAGGGAGGGCGCCGGCAGCGGTGCGAATGCGCCTAGGGGAATAAATCTTTGGGCACCTAGTGGTCATG

2 CATGACCACTAGGAGCATCTTTGGCGACCTGGGGCGACTTGTTCGCCCTAGTGTGGACTAGGGGAATAAATCTTTGGGCACCTAGTGGTCATG

2 CATGACCACTAGGAGCATCTTTGGCGACCAGACGTGTCGTCGATCAGATGTGTAGCGCTAGGGGAATAAATCTTTGGGCACCTAGTGGTCATG

2 CATGACCACTAGGAGCATCTTTGGCGAGTCGGTGTGAACGGGTCCTGCGCAGCGAGTCTAGGGGAATAAATCTTTGGGCACCTAGTGGTCATG

2 CATGACCACTAGGAGCATCTTTGGCGACCCTAAGCATGCCATGCGTCAGAGGGTGGACTAGGGGAATAAATCTTTGGGCACCTAGTGGTCATG

2 CATGACCACTAGGAGCATCTTTGGCGATACCGAGGTGTGGCGCGAGCGACGGAGAGACTAGGGGAATAAATCTTTGGGCACCTAGTGGTCATG

2 CATGACCACTAGGAGCATCTTTGGCGACGGCGTGGGGCCATCTGTGCGGCGGACCCCCTAGGGGAATAAATCTTTGGGCACCTAGTGGTCATG

2 CATGACCACTAGGAGCATCTTTGGCGAAGGGACGGCCCACGGGTGCAGGTCCGCAGACTAGGGGAATAAATCTTTGGGCACCTAGTGGTCATG

2 CATGACCACTAGGAGCATCTTTGGCGACAGTGCGGGCTCAGGGGTCCGGCGAGGAGACTAGGGGAATAAATCTTTGGGCACCTAGTGGTCATG

2 CATGACCACTAGGAGCATCTTTGGCGACCCGCATCGTTGTTCACACTGCGCGGTGGACTAGGGGAATAAATCTTTGGGCACCTAGTGGTCATG

2 CATGACCACTAGGAGCATCTTTGGCGAGGGTCCCTAGGGCCCTACAGAGTTTGTGAGCTAGGGGAATAAATCTTTGGGCACCTAGTGGTCATG

2 CATGACCACTAGGAGCATCTTTGGCGACCCGTGTTGCGATAGAAAACAAGTGGTGGACTAGGGGAATAAATCTTTGGGCACCTAGTGGTCATG

2 CATGACCACTAGGAGCATCTTTGGCGACCCACGGTAAAGCAATGTACGTGTGGTGGACTAGGGGAATAAATCTTTGGGCACCTAGTGGTCATG

2 CATGACCACTAGGAGCATCTTTGGCGACTGTACGGGAGTCAATCGTGGCCGAGTCGACTAGGGGAATAAATCTTTGGGCACCTAGTGGTCATG

2 CATGACCACTAGGAGCATCTTTGGCGACTCGACGGCGTATAGCGATGTCGTAGTGGACTAGGGGAATAAATCTTTGGGCACCTAGTGGTCATG

2 CATGACCACTAGGAGCATCTTTGGCGACAGAATGGGCAACGGCTAACTAGAATGCGCCTAGGGGAATAAATCTTTGGGCACCTAGTGGTCATG

2 CATGACCACTAGGAGCATCTTTGGCGAGATAGGGAGAATCGGTGGCAATGGTGTCTCCTAGGGGAATAAATCTTTGGGCACCTAGTGGTCATG

2 CATGACCACTAGGAGCATCTTTGGCGATCGGCAACGGGCGGACATGTGAGCCGGAGACTAGGGGAATAAATCTTTGGGCACCTAGTGGTCATG

2 CATGACCACTAGGAGCATCTTTGGCGAACAGCGCGAGGACTACGTAACCGTGTTAGCCTAGGGGAATAAATCTTTGGGCACCTAGTGGTCATG

2 CATGACCACTAGGAGCATCTTTGGCGACCCGCAATAGGAGGAACGGCAGCGGGTGGACTAGGGGAATAAATCTTTGGGCACCTAGTGGTCATG

2 CATGACCACTAGGAGCATCTTTGGCGAGGGAGGGCGCCGGAAGCGGTGTGAATGCGCCTAGGGGAATAAATCTTTGGGCACCTAGTGGTCATG

2 CATGACCACTAGGAGCATCTTTGGCGACGTGAGCAGCGCGGTGTCAGGGTAGGCGCACTAGGGGAATAAATCTTTGGGCACCTAGTGGTCATG

2 CATGACCACTAGGAGCATCTTTGGCGACGACCGAGGACTACTCAGACTCGGCGTGGACTAGGGGAATAAATCTTTGGGCACCTAGTGGTCATG

2 CATGACCACTAGGAGCATCTTTGGCGACACTCTGCTAGCTTGCGCAGGTAAGTGGACCTAGGGGAATAAATCTTTGGGCACCTAGTGGTCATG

2 CATGACCACTAGGAGCATCTTTGGCGACGTATGCAGGCGCGTAGTCCTGGAGTGGACCTAGGGGAATAAATCTTTGGGCACCTAGTGGTCATG

2 CATGACCACTAGGAGCATCTTTGGCGAATAATGAGCGCAATACGAGTGGCTGAGTATCTAGGGGAATAAATCTTTGGGCACCTAGTGGTCATG

2 CATGACCACTAGGAGCATCTTTGGCGAGCCTCGTTTGGGAGGTTGCTCCACCAGTTCCTAGGGGAATAAATCTTTGGGCACCTAGTGGTCATG

2 CATGACCACTAGGAGCATCTTTGGCGAGGGGTCAGTGTGCGGGAGCAAGGGGCGAGACTAGGGGAATAAATCTTTGGGCACCTAGTGGTCATG

2 CATGACCACTAGGAGCATCTTTGGCGACCCGTTCTCCGCAGTGCTGCAACGGGTGGACTAGGGGAATAAATCTTTGGGCACCTAGTGGTCATG

2 CATGACCACTAGGAGCATCTTTGGCGACCTGTGGTTGGTGCAAGAAGAGACGGAAGCCTAGGGGAATAAATCTTTGGGCACCTAGTGGTCATG

2 CATGACCACTAGGAGCATCTTTGGCGACTAAATGGGCATGGCAGAGGGGAAAGTCGACTAGGGGAATAAATCTTTGGGCACCTAGTGGTCATG

2 CATGACCACTAGGAGCATCTTTGGCGATAGGCGTGTAAGGTCGAGCGAAAGCGATAGCTAGGGGAATAAATCTTTGGGCACCTAGTGGTCATG

2 CATGACCACTAGGAGCATCTTTGGCGAGTGTGGGGGTTCAGAGGTCTATAGCCCGAGCTAGGGGAATAAATCTTTGGGCACCTAGTGGTCATG

2 CATGACCACTAGGAGCATCTTTGGCGAACATGCGGGTGTAGGTCACGGCGTTGTGAGCTAGGGGAATAAATCTTTGGGCACCTAGTGGTCATG

2 CATGACCACTAGGAGCATCTTTGGCGACTGATATGGAGGGGTGGGATCCGCTATGCCCTAGGGGAATAAATCTTTGGGCACCTAGTGGTCATG

2 CATGACCACTAGGAGCATCTTTGGCGACGTAGTGGGCTGGGAACGGAAAAGCGTCGCCTAGGGGAATAAATCTTTGGGCACCTAGTGGTCATG

2 CATGACCACTAGGAGCATCTTTGGCGATCCGCGTAACCTGACGGTTATCGGCCAGCACTAGGGGAATAAATCTTTGGGCACCTAGTGGTCATG

2 CATGACCACTAGGAGCATCTTTGGCGAACATCGAAGGCAAGGTGAGGCAGAGTTCGACTAGGGGAATAAATCTTTGGGCACCTAGTGGTCATG

2 CATGACCACTAGGAGCATCTTTGGCGAACGGCAGGTGTTGCGGGGGTCTGAGAATCCCTAGGGGAATAAATCTTTGGGCACCTAGTGGTCATG

2 CATGACCACTAGGAGCATCTTTGGCGACACGCTTGGAGGCACCCAGGCGTCGTGGAGCTAGGGGAATAAATCTTTGGGCACCTAGTGGTCATG

2 CATGACCACTAGGAGCATCTTTGGCGATCTGTACGTGCCGACGCACGGGCGTAGAGACTAGGGGAATAAATCTTTGGGCACCTAGTGGTCATG

2 CATGACCACTAGGAGCATCTTTGGCGACCCTCGAGACGTAGTTGTTCGTAGGGTGGACTAGGGGAATAAATCTTTGGGCACCTAGTGGTCATG

2 CATGACCACTAGGAGCATCTTTGGCGACTGAGGGCTCCAGTGCTCTAGCAATATCGCCTAGGGGAATAAATCTTTGGGCACCTAGTGGTCATG

2 CATGACCACTAGGAGCATCTTTGGCGAAAGGAAGGTGTTGCGGTGGTCTGTGAATCCCTAGGGGAATAAATCTTTGGGCACCTAGTGGTCATG

2 CATGACCACTAGGAGCATCTTTGGCGACTGTAGTGGTCGTGCGAACCGCGAAGTGGACTAGGGGAATAAATCTTTGGGCACCTAGTGGTCATG

2 CATGACCACTAGGAGCATCTTTGGCGAGATCGGTATAATCGGTGGCATTGGTGTCTCCTAGGGGAATAAATCTTTGGGCACCTAGTGGTCATG

2 CATGACCACTAGGAGCATCTTTGGCGAGCCGGCGTGTAGTGGTGAGCAGACGTTTGGCTAGGGGAATAAATCTTTGGGCACCTAGTGGTCATG

2 CATGACCACTAGGAGCATCTTTGGCGACCCAGCGTGGTTTACCCGTCGATGGGTGGACTAGGGGAATAAATCTTTGGGCACCTAGTGGTCATG

2 CATGACCACTAGGAGCATCTTTGGCGACCCAGGAAGCGAGCAGACTACCTGGGTGGACTAGGGGAATAAATCTTTGGGCACCTAGTGGTCATG

2 CATGACCACTAGGAGCATCTTTGGCGACCCTCGAAGAACGTGTAATTCGTGGGTGGACTAGGGGAATAAATCTTTGGGCACCTAGTGGTCATG

2 CATGACCACTAGGAGCATCTTTGGCGAGGCTTCTTGGGCGGAGGTATGTGCCCTGCGCTAGGGGAATAAATCTTTGGGCACCTAGTGGTCATG

2 CATGACCACTAGGAGCATCTTTGGCGACCAGGAACTAGAGTGACGATGCTTAGGTCGCTAGGGGAATAAATCTTTGGGCACCTAGTGGTCATG

2 CATGACCACTAGGAGCATCTTTGGCGAGGGGTAGGGGGCCGCTCGCGCCTTCGTGCGCTAGGGGAATAAATCTTTGGGCACCTAGTGGTCATG

2 CATGACCACTAGGAGCATCTTTGGCGAAGTGGTCCGTTTGGGAATGTCTGACGAAGCCTAGGGGAATAAATCTTTGGGCACCTAGTGGTCATG

2 CATGACCACTAGGAGCATCTTTGGCGACGAGGACCGGACAACCGCGCTTAAGGTCGACTAGGGGAATAAATCTTTGGGCACCTAGTGGTCATG

2 CATGACCACTAGGAGCATCTTTGGCGAACCCGAAACCCGGCTGTGCGAGCGGAGAGCCTAGGGGAATAAATCTTTGGGCACCTAGTGGTCATG

2 CATGACCACTAGGAGCATCTTTGGCGAGTTGTGGGCACAGGTCTTAGAAGACTCGACCTAGGGGAATAAATCTTTGGGCACCTAGTGGTCATG

2 CATGACCACTAGGAGCATCTTTGGCGAAACGCAGTTGGTGTAATGCGGATAGTGGAGCTAGGGGAATAAATCTTTGGGCACCTAGTGGTCATG

2 CATGACCACTAGGAGCATCTTTGGCGACCACGTTCAGAGACCTGGGAGAAGGGGCGACTAGGGGAATAAATCTTTGGGCACCTAGTGGTCATG

2 CATGACCACTAGGAGCATCTTTGGCGACTCAGGGGGTTGCGAAGGTGCTCAGTCGCTCTAGGGGAATAAATCTTTGGGCACCTAGTGGTCATG

2 CATGACCACTAGGAGCATCTTTGGCGAACGGCAGGTGTTGCGGTGGTTTGTTAATCCCTAGGGGAATAAATCTTTGGGCACCTAGTGGTCATG

2 CATGACCACTAGGAGCATCTTTGGCGAGTTTCGCTGTGCTTGGAATGGGGCTACCCTCTAGGGGAATAAATCTTTGGGCACCTAGTGGTCATG

2 CATGACCACTAGGAGCATCTTTGGCGAGGGGCGAGGGATACGAGGGGTATTTGTCGACTAGGGGAATAAATCTTTGGGCACCTAGTGGTCATG

2 CATGACCACTAGGAGCATCTTTGGCGACCCGAGTATGCCTGTCAATACGGGGTGGCTCTAGGGGAATAAATCTTTGGGCACCTAGTGGTCATG

2 CATGACCACTAGGAGCATCTTTGGCGAGAAGACCCTGGATTCGGGGACCAGTTGCTACTAGGGGAATAAATCTTTGGGCACCTAGTGGTCATG

2 CATGACCACTAGGAGCATCTTTGGCGAGATCGGGAGAATCTGTGGCATAGGTGTCTACTAGGGGAATAAATCTTTGGGCACCTAGTGGTCATG

2 CATGACCACTAGGAGCATCTTTGGCGACTTAAGTGGTTGAGATGTCTGGGCAGTCGACTAGGGGAATAAATCTTTGGGCACCTAGTGGTCATG

2 CATGACCACTAGGAGCATCTTTGGCGACGTCCTCGGTGGGGCGTGCTGGCCCGGCGCCTAGGGGAATAAATCTTTGGGCACCTAGTGGTCATG

2 CATGACCACTAGGAGCATCTTTGGCGACCATTTGTCGGGCCGACTGTGGATCCGGAGCTAGGGGAATAAATCTTTGGGCACCTAGTGGTCATG

2 CATGACCACTAGGAGCATCTTTGGCGATCCTCGTCATATGGTGAAGCAGACGTTTGGCTAGGGGAATAAATCTTTGGGCACCTAGTGGTCATG

2 CATGACCACTAGGAGCATCTTTGGCGAAGACAACGCGGCGTCAAGCATGCTCGAAGACTAGGGGAATAAATCTTTGGGCACCTAGTGGTCATG

2 CATGACCACTAGGAGCATCTTTGGCGACGAGTGGGCTCCGAGGAAACCGGCAGTCGACTAGGGGAATAAATCTTTGGGCACCTAGTGGTCATG

2 CATGACCACTAGGAGCATCTTTGGCGACCTGCCTAGCGAAGCGGGGCACGAGTTCGGCTAGGGGAATAAATCTTTGGGCACCTAGTGGTCATG

2 CATGACCACTAGGAGCATCTTTGGCGATCCGAAAGCGATGGAGAATCGGCGTGGAAGCTAGGGGAATAAATCTTTGGGCACCTAGTGGTCATG

2 CATGACCACTAGGAGCATCTTTGGCGATGGGTGCTGACGGGGCTAGTGAGACCTACACTAGGGGAATAAATCTTTGGGCACCTAGTGGTCATG

2 CATGACCACTAGGAGCATCTTTGGCGACTAAGGGCGGCCAGGCAGACCTAAGTGGAGCTAGGGGAATAAATCTTTGGGCACCTAGTGGTCATG

2 CATGACCACTAGGAGCATCTTTGGCGAGCCTTGCTTGGGAAGTTGCTCCACCAGTTCCTAGGGGAATAAATCTTTGGGCACCTAGTGGTCATG

2 CATGACCACTAGGAGCATCTTTGGCGATGTACTTCGTAGTTGGGGCTGGAATCAAGACTAGGGGAATAAATCTTTGGGCACCTAGTGGTCATG

2 CATGACCACTAGGAGCATCTTTGGCGAGGAAACGGCGTCGGGCCAAGGGCGATGCCACTAGGGGAATAAATCTTTGGGCACCTAGTGGTCATG

2 CATGACCACTAGGAGCATCTTTGGCGAATGGCCGGCACGGCCTTCTAGTCGTCGGTACTAGGGGAATAAATCTTTGGGCACCTAGTGGTCATG

2 CATGACCACTAGGAGCATCTTTGGCGAGAAGACTCTGGATTCGTGGACCAGTTGATGCTAGGGGAATAAATCTTTGGGCACCTAGTGGTCATG

2 CATGACCACTAGGAGCATCTTTGGCGAAGAAGATGGTAGGGTAATGTGTCGGCGCCGCTAGGGGAATAAATCTTTGGGCACCTAGTGGTCATG

2 CATGACCACTAGGAGCATCTTTGGCGAACGTGCAAGATATGCCGCCTGGATCGTAGGCTAGGGGAATAAATCTTTGGGCACCTAGTGGTCATG

2 CATGACCACTAGGAGCATCTTTGGCGAGATCGAGAGAATCGGTGGCATAGGTGTCTCCTAGGGGAATAAATCTTTGGGCACCTAGTGGTCATG

2 CATGACCACTAGGAGCATCTTTGGCGACAGCGGCGTAGGCTGCCTCGGGTGGTGGCCCTAGGGGAATAAATCTTTGGGCACCTAGTGGTCATG

2 CATGACCACTAGGAGCATCTTTGGCGAACGGCAGGTGTTGCGGTGGTATGTGAATCACTAGGGGAATAAATCTTTGGGCACCTAGTGGTCATG

2 CATGACCACTAGGAGCATCTTTGGCGACGTGGGAAGTCTGAGGATTTCCTAGCGGAGCTAGGGGAATAAATCTTTGGGCACCTAGTGGTCATG

2 CATGACCACTAGGAGCATCTTTGGCGATTTGCCTCGGTGCAGCCTGGCAAGTAGCCGCTAGGGGAATAAATCTTTGGGCACCTAGTGGTCATG

2 CATGACCACTAGGAGCATCTTTGGCGACACATTGTAACTTGTTTCAATGTTGTGGAGCTAGGGGAATAAATCTTTGGGCACCTAGTGGTCATG

2 CATGACCACTAGGAGCATCTTTGGCGACTTGGGTTCCGGTGACGGCGGTCTTGCAAGCTAGGGGAATAAATCTTTGGGCACCTAGTGGTCATG

2 CATGACCACTAGGAGCATCTTTGGCGAGGGAAACGAAGATTGAGTTGCGTCCCTAGACTAGGGGAATAAATCTTTGGGCACCTAGTGGTCATG

2 CATGACCACTAGGAGCATCTTTGGCGACTACTGGCCGACGCCGGCCTAGAAGTGGAGCTAGGGGAATAAATCTTTGGGCACCTAGTGGTCATG

2 CATGACCACTAGGAGCATCTTTGGCGACCCAGATTGATGTAATCTGTAATGGGTGGACTAGGGGAATAAATCTTTGGGCACCTAGTGGTCATG

2 CATGACCACTAGGAGCATCTTTGGCGACTAGGGGGGTAACGGCCGACAGGTAGTCGACTAGGGGAATAAATCTTTGGGCACCTAGTGGTCATG

2 CATGACCACTAGGAGCATCTTTGGCGACGTGTTGAGCGGTGAGCGACAACAAGTGGACTAGGGGAATAAATCTTTGGGCACCTAGTGGTCATG

2 CATGACCACTAGGAGCATCTTTGGCGAATGGCCGGCACGGCCTTCTGGTCCTTGGTACTAGGGGAATAAATCTTTGGGCACCTAGTGGTCATG

2 CATGACCACTAGGAGCATCTTTGGCGACTGAGGCAAGTGGCGCGTCACCGAAGTGGACTAGGGGAATAAATCTTTGGGCACCTAGTGGTCATG

2 CATGACCACTAGGAGCATCTTTGGCGAAGCGACGGGTGGACATCCTTGGAATTGATGCTAGGGGAATAAATCTTTGGGCACCTAGTGGTCATG

2 CATGACCACTAGGAGCATCTTTGGCGACGGCGTGGGGCTATTGATGCGGAGGCACTCCTAGGGGAATAAATCTTTGGGCACCTAGTGGTCATG

2 CATGACCACTAGGAGCATCTTTGGCGACTGAGGATGGTGCATAGAACATCCAGTGGCCTAGGGGAATAAATCTTTGGGCACCTAGTGGTCATG

2 CATGACCACTAGGAGCATCTTTGGCGACTGTGGTGTGCCTAGATCTCGGGATGTCGACTAGGGGAATAAATCTTTGGGCACCTAGTGGTCATG

2 CATGACCACTAGGAGCATCTTTGGCGACTAAGTGGCTAACGCGTTAAATGGGGTAGACTAGGGGAATAAATCTTTGGGCACCTAGTGGTCATG

2 CATGACCACTAGGAGCATCTTTGGCGACCGCAGGAAAACAAAAGTGGCCTTGCTAGCCTAGGGGAATAAATCTTTGGGCACCTAGTGGTCATG

2 CATGACCACTAGGAGCATCTTTGGCGAAGCATTCTATGGAGCGGTGTGGTGGCTCCCCTAGGGGAATAAATCTTTGGGCACCTAGTGGTCATG

2 CATGACCACTAGGAGCATCTTTGGCGACTAGGCTTTACCGACGTTCCGATTGGGTAGCTAGGGGAATAAATCTTTGGGCACCTAGTGGTCATG

2 CATGACCACTAGGAGCATCTTTGGCGAGGTGTCTCAGTTCGACATAGGGGGCGAGCACTAGGGGAATAAATCTTTGGGCACCTAGTGGTCATG

2 CATGACCACTAGGAGCATCTTTGGCGAGTTGTCTACCGCGAGGAAGACTATCCGCTACTAGGGGAATAAATCTTTGGGCACCTAGTGGTCATG

2 CATGACCACTAGGAGCATCTTTGGCGAACGTTGACAGTCACGTAGTACTCAGTGGAGCTAGGGGAATAAATCTTTGGGCACCTAGTGGTCATG

2 CATGACCACTAGGAGCATCTTTGGCGATTAAGTTGTGCGATACGGATTGAAGATCGACTAGGGGAATAAATCTTTGGGCACCTAGTGGTCATG

2 CATGACCACTAGGAGCATCTTTGGCGACGTAGTGGGCTGGCAGGGAGTACTTGTTGACTAGGGGAATAAATCTTTGGGCACCTAGTGGTCATG

2 CATGACCACTAGGAGCATCTTTGGCGAGCACAGGTGGGGTTCGGGAAGATAGCGAGACTAGGGGAATAAATCTTTGGGCACCTAGTGGTCATG

2 CATGACCACTAGGAGCATCTTTGGCGAAGCGTGTTGCAAGGTCCGCGAGAGTGGACCCTAGGGGAATAAATCTTTGGGCACCTAGTGGTCATG

2 CATGACCACTAGGAGCATCTTTGGCGACACACAAGGATGCGTACGCGTGGGTAGAGCCTAGGGGAATAAATCTTTGGGCACCTAGTGGTCATG

2 CATGACCACTAGGAGCATCTTTGGCGAATGGCAGGTGTTGCGGTGGTCTGTGAACCCCTAGGGGAATAAATCTTTGGGCACCTAGTGGTCATG

2 CATGACCACTAGGAGCATCTTTGGCGAGAAGACTTTGGGTTCGGGGACCAGTTGCTGCTAGGGGAATAAATCTTTGGGCACCTAGTGGTCATG

2 CATGACCACTAGGAGCATCTTTGGCGACCCCTCGTGAAATGGCACCGAGGCGGTGGACTAGGGGAATAAATCTTTGGGCACCTAGTGGTCATG

2 CATGACCACTAGGAGCATCTTTGGCGAGGAGATGTATCAACATAGTGTGGGCGAGTACTAGGGGAATAAATCTTTGGGCACCTAGTGGTCATG

2 CATGACCACTAGGAGCATCTTTGGCGAGTAGTGGGCGTGCTACAGGAAAATACGAGACTAGGGGAATAAATCTTTGGGCACCTAGTGGTCATG

2 CATGACCACTAGGAGCATCTTTGGCGACAGAAGGTTGCTTGGGTATATGAGTCGCCGCTAGGGGAATAAATCTTTGGGCACCTAGTGGTCATG

2 CATGACCACTAGGAGCATCTTTGGCGACTCAGGGGCATGGCAGTAAAGGGTAGTCGACTAGGGGAATAAATCTTTGGGCACCTAGTGGTCATG

2 CATGACCACTAGGAGCATCTTTGGCGACCGTAGGAAAACAATAGTGGCCTTGCTAGCCTAGGGGAATAAATCTTTGGGCACCTAGTGGTCATG

2 CATGACCACTAGGAGCATCTTTGGCGATACCCGTTCGGGTAGTCACTGGTCTGCGCCCTAGGGGAATAAATCTTTGGGCACCTAGTGGTCATG

2 CATGACCACTAGGAGCATCTTTGGCGATGACGAAGGTGCGGACTGGTCGGAAGAGCTCTAGGGGAATAAATCTTTGGGCACCTAGTGGTCATG

2 CATGACCACTAGGAGCATCTTTGGCGACCGCGGAGGCGTAGCCGTCCGGAAGTGGAGCTAGGGGAATAAATCTTTGGGCACCTAGTGGTCATG

2 CATGACCACTAGGAGCATCTTTGGCGACGACGTGGCGCTAGACTATTGAGACGGAGACTAGGGGAATAAATCTTTGGGCACCTAGTGGTCATG

2 CATGACCACTAGGAGCATCTTTGGCGAGGGGACACATTCGGCTTCGACGGGTATCGACTAGGGGAATAAATCTTTGGGCACCTAGTGGTCATG

2 CATGACCACTAGGAGCATCTTTGGCGAGATCGGGAGAATCGGTGTCAATGGTGTCTCCTAGGGGAATAAATCTTTGGGCACCTAGTGGTCATG

2 CATGACCACTAGGAGCATCTTTGGCGACCCGGAGGTGATCATCATCGCCGTGGTGGACTAGGGGAATAAATCTTTGGGCACCTAGTGGTCATG

2 CATGACCACTAGGAGCATCTTTGGCGACTAGTTATCGTGCTTGGGTGTTGCGGTCGCCTAGGGGAATAAATCTTTGGGCACCTAGTGGTCATG

2 CATGACCACTAGGAGCATCTTTGGCGACAGAGTGGGTGTAACGCACGCGTAGTGGAGCTAGGGGAATAAATCTTTGGGCACCTAGTGGTCATG

2 CATGACCACTAGGAGCATCTTTGGCGACCCGCTACTGTGCAAGCAAAGTCGGGTGGACTAGGGGAATAAATCTTTGGGCACCTAGTGGTCATG

2 CATGACCACTAGGAGCATCTTTGGCGAGGCATGGAGCCGGGTAGACTTGTAAGCGCGCTAGGGGAATAAATCTTTGGGCACCTAGTGGTCATG

2 CATGACCACTAGGAGCATCTTTGGCGAGCGTCAGCATACCGATTCTGGGCGTTTGGTCTAGGGGAATAAATCTTTGGGCACCTAGTGGTCATG

2 CATGACCACTAGGAGCATCTTTGGCGAGATCTGAAGAATCGGTGGCATTGGTGTCTCCTAGGGGAATAAATCTTTGGGCACCTAGTGGTCATG

2 CATGACCACTAGGAGCATCTTTGGCGAGGGCGTCTGTCCACGAAGGGTGGTGTCCCGCTAGGGGAATAAATCTTTGGGCACCTAGTGGTCATG

2 CATGACCACTAGGAGCATCTTTGGCGATAGTGTAACGTGGCTTAAAAATTAATTCGCCTAGGGGAATAAATCTTTGGGCACCTAGTGGTCATG

2 CATGACCACTAGGAGCATCTTTGGCGACGTGCGAGTGTCGGTTCGAGGTTGCGACGCCTAGGGGAATAAATCTTTGGGCACCTAGTGGTCATG

2 CATGACCACTAGGAGCATCTTTGGCGACGAGACTCCTGTGGAGGCACAAAGGTCGCTCTAGGGGAATAAATCTTTGGGCACCTAGTGGTCATG

2 CATGACCACTAGGAGCATCTTTGGCGAGCCTAAGCGCTGTGCCGCTCTGACTGGGTCCTAGGGGAATAAATCTTTGGGCACCTAGTGGTCATG

2 CATGACCACTAGGAGCATCTTTGGCGACGAGTGGGGCCGGTAAGGGACGAGATTAGCCTAGGGGAATAAATCTTTGGGCACCTAGTGGTCATG

2 CATGACCACTAGGAGCATCTTTGGCGAAGGGTTTAGGACTTCAAGTGGATCTCATAGCTAGGGGAATAAATCTTTGGGCACCTAGTGGTCATG

2 CATGACCACTAGGAGCATCTTTGGCGACTAAATGTGACGGGCAGGATCACGAGGCGACTAGGGGAATAAATCTTTGGGCACCTAGTGGTCATG

2 CATGACCACTAGGAGCATCTTTGGCGATGTGTGGTCTGTGATGGTAGAAATCATCGACTAGGGGAATAAATCTTTGGGCACCTAGTGGTCATG

2 CATGACCACTAGGAGCATCTTTGGCGACTGTGACTGGGACTGGACTACGGTGGTCGCCTAGGGGAATAAATCTTTGGGCACCTAGTGGTCATG

2 CATGACCACTAGGAGCATCTTTGGCGACCCGGCCGTGTAAGACACGGATGTGGTGGACTAGGGGAATAAATCTTTGGGCACCTAGTGGTCATG

2 CATGACCACTAGGAGCATCTTTGGCGAGGCAATGCGACGGGCTATGGGTTCCTCGAGCTAGGGGAATAAATCTTTGGGCACCTAGTGGTCATG

2 CATGACCACTAGGAGCATCTTTGGCGACGTTGTGGGGACCGTAGGTTCGGCGGGTACCTAGGGGAATAAATCTTTGGGCACCTAGTGGTCATG

2 CATGACCACTAGGAGCATCTTTGGCGAACACATGACTGTACTGCATGTAGCAGGCGCCTAGGGGAATAAATCTTTGGGCACCTAGTGGTCATG

2 CATGACCACTAGGAGCATCTTTGGCGATGGGTGCTGACGTCCGCCGCTGCGGCTACACTAGGGGAATAAATCTTTGGGCACCTAGTGGTCATG

2 CATGACCACTAGGAGCATCTTTGGCGACGTGGGCTTGTATAATAGTCAGGAAGGAGACTAGGGGAATAAATCTTTGGGCACCTAGTGGTCATG

2 CATGACCACTAGGAGCATCTTTGGCGACGACCGGGGGCGAAGCTAACCCGGCGTGGACTAGGGGAATAAATCTTTGGGCACCTAGTGGTCATG

2 CATGACCACTAGGAGCATCTTTGGCGATCCGTTCGCGGCTGCGAATGCGTGTGGAGCCTAGGGGAATAAATCTTTGGGCACCTAGTGGTCATG

2 CATGACCACTAGGAGCATCTTTGGCGAAGATGGAACGCTGGGTCCCGGAATCGTCGCCTAGGGGAATAAATCTTTGGGCACCTAGTGGTCATG

2 CATGACCACTAGGAGCATCTTTGGCGAAAGTCGGTAGAACAGTGTGGGTTGCTGTCCCTAGGGGAATAAATCTTTGGGCACCTAGTGGTCATG

2 CATGACCACTAGGAGCATCTTTGGCGAGATCGGGAGAATCGTTGGCATAGGTGTCTCCTAGGGGAATAAATCTTTGGGCACCTAGTGGTCATG

2 CATGACCACTAGGAGCATCTTTGGCGAGGGGCCTTGCTATAGCTGCAAACTGAGCATCTAGGGGAATAAATCTTTGGGCACCTAGTGGTCATG

2 CATGACCACTAGGAGCATCTTTGGCGAATGGCTGGCACGGCCTCCTAGTCCTCGGTACTAGGGGAATAAATCTTTGGGCACCTAGTGGTCATG

2 CATGACCACTAGGAGCATCTTTGGCGAGCGGCAGGTGTTGCGGTGGTCTGTGTATCCCTAGGGGAATAAATCTTTGGGCACCTAGTGGTCATG

2 CATGACCACTAGGAGCATCTTTGGCGAGCCTTGCTTGGGAGGCTGCTCCACCAGTTCCTAGGGGAATAAATCTTTGGGCACCTAGTGGTCATG

2 CATGACCACTAGGAGCATCTTTGGCGACGTTACTAGTTGGGAAGCATTCGCGGTTAGCTAGGGGAATAAATCTTTGGGCACCTAGTGGTCATG

2 CATGACCACTAGGAGCATCTTTGGCGAAGATCGCTCGTCGACTCGGGTCCGAGAAAGCTAGGGGAATAAATCTTTGGGCACCTAGTGGTCATG

2 CATGACCACTAGGAGCATCTTTGGCGAGATAGGGAGAATCGGAGGCATTGGTGTCTCCTAGGGGAATAAATCTTTGGGCACCTAGTGGTCATG

2 CATGACCACTAGGAGCATCTTTGGCGATGAGTCTAGTACGCGTTAGGTTGTTTGCCGCTAGGGGAATAAATCTTTGGGCACCTAGTGGTCATG

2 CATGACCACTAGGAGCATCTTTGGCGAGACGACGCGTGACACCGGGTTAGTGCGAGACTAGGGGAATAAATCTTTGGGCACCTAGTGGTCATG

2 CATGACCACTAGGAGCATCTTTGGCGACTGGGGGTCTCCTCGCAGGGTTCGCGCCAGCTAGGGGAATAAATCTTTGGGCACCTAGTGGTCATG

2 CATGACCACTAGGAGCATCTTTGGCGAATCATGGGTTGGCAGGGGGTATTTGGGCGACTAGGGGAATAAATCTTTGGGCACCTAGTGGTCATG

2 CATGACCACTAGGAGCATCTTTGGCGAAAATGGCGGTCGCTCGAACCCAGAGTCGCGCTAGGGGAATAAATCTTTGGGCACCTAGTGGTCATG

2 CATGACCACTAGGAGCATCTTTGGCGAGATCTGGAGAATCTGTGGCATTGGTGTCTCCTAGGGGAATAAATCTTTGGGCACCTAGTGGTCATG

2 CATGACCACTAGGAGCATCTTTGGCGATAGGTGGCGGGTGCGTATGGGGCGTTGGAGCTAGGGGAATAAATCTTTGGGCACCTAGTGGTCATG

2 CATGACCACTAGGAGCATCTTTGGCGAAGTCGAGGATAGCTCACAAGTTTAATTCGCCTAGGGGAATAAATCTTTGGGCACCTAGTGGTCATG

2 CATGACCACTAGGAGCATCTTTGGCGAGCCTCGCTTGGGAGGTTGCTCCACCAGTTTCTAGGGGAATAAATCTTTGGGCACCTAGTGGTCATG

2 CATGACCACTAGGAGCATCTTTGGCGACAGTTGGGCTTGGAGAATGATACTGGCAGCCTAGGGGAATAAATCTTTGGGCACCTAGTGGTCATG

2 CATGACCACTAGGAGCATCTTTGGCGACGAGAGAAAGCTGCGGAAAGCACCAAGTGGCTAGGGGAATAAATCTTTGGGCACCTAGTGGTCATG

2 CATGACCACTAGGAGCATCTTTGGCGACCCGGGCGATAAGGGACAGCCCGCGGTGGACTAGGGGAATAAATCTTTGGGCACCTAGTGGTCATG

2 CATGACCACTAGGAGCATCTTTGGCGATCAGGCTATCGAGGGATGGCACTGATAAGACTAGGGGAATAAATCTTTGGGCACCTAGTGGTCATG

2 CATGACCACTAGGAGCATCTTTGGCGACAGAGCAGCGACGGTCGGCGGTCGAGGAGACTAGGGGAATAAATCTTTGGGCACCTAGTGGTCATG

2 CATGACCACTAGGAGCATCTTTGGCGAGGGGCGAGACGTCAGCGAGGTGTTAGGTGCCTAGGGGAATAAATCTTTGGGCACCTAGTGGTCATG

2 CATGACCACTAGGAGCATCTTTGGCGACTCCGGGGAGCATCGCGAGACCGGTGTGGACTAGGGGAATAAATCTTTGGGCACCTAGTGGTCATG

2 CATGACCACTAGGAGCATCTTTGGCGAGGTGTTTCAGTTCGACACAAGGGGCGAGCACTAGGGGAATAAATCTTTGGGCACCTAGTGGTCATG

2 CATGACCACTAGGAGCATCTTTGGCGACCCGACCTGAAGAAACTTCATACGGGTGGACTAGGGGAATAAATCTTTGGGCACCTAGTGGTCATG

2 CATGACCACTAGGAGCATCTTTGGCGACCCGTAGATCGAGTAGATCTAATGGGTGGACTAGGGGAATAAATCTTTGGGCACCTAGTGGTCATG

2 CATGACCACTAGGAGCATCTTTGGCGATATCGGGAGAATCGGTGGAATTGGTGTCTCCTAGGGGAATAAATCTTTGGGCACCTAGTGGTCATG

2 CATGACCACTAGGAGCATCTTTGGCGAACGGCAGGTGTTGCGGTGGTCTCTGAAACCCTAGGGGAATAAATCTTTGGGCACCTAGTGGTCATG

2 CATGACCACTAGGAGCATCTTTGGCGACGCATCAGAGTCACACGCTGATGTCGTGGACTAGGGGAATAAATCTTTGGGCACCTAGTGGTCATG

2 CATGACCACTAGGAGCATCTTTGGCGAGGTCACACAGTGATACGCGCAGACGTTTGGCTAGGGGAATAAATCTTTGGGCACCTAGTGGTCATG

2 CATGACCACTAGGAGCATCTTTGGCGACGAAGTGGTCGGAGGCGCGTTAATGAGGAGCTAGGGGAATAAATCTTTGGGCACCTAGTGGTCATG

2 CATGACCACTAGGAGCATCTTTGGCGAACGGCAGGTGTTGCGGTGGTTTGTGCATCCCTAGGGGAATAAATCTTTGGGCACCTAGTGGTCATG

2 CATGACCACTAGGAGCATCTTTGGCGACGCGCTGACGGAAAGTTCGAGGACACCAGGCTAGGGGAATAAATCTTTGGGCACCTAGTGGTCATG

2 CATGACCACTAGGAGCATCTTTGGCGACATGGGGGCAGTGTACGAGGCAATGTCAGACTAGGGGAATAAATCTTTGGGCACCTAGTGGTCATG

2 CATGACCACTAGGAGCATCTTTGGCGATGGTGCCGTGCTATCTGCACACTCCGCGGGCTAGGGGAATAAATCTTTGGGCACCTAGTGGTCATG

2 CATGACCACTAGGAGCATCTTTGGCGAACGGGCGACGAGATGTATCGGAAGTGAAGACTAGGGGAATAAATCTTTGGGCACCTAGTGGTCATG

2 CATGACCACTAGGAGCATCTTTGGCGAAGAGTGGTCGGTCGTCAAATAGAACTGAGCCTAGGGGAATAAATCTTTGGGCACCTAGTGGTCATG

2 CATGACCACTAGGAGCATCTTTGGCGATACGATTTGGAAGGCGAAAGTGCGTGAGACCTAGGGGAATAAATCTTTGGGCACCTAGTGGTCATG

2 CATGACCACTAGGAGCATCTTTGGCGACGGCGTGGGGCACCTCTCGCGGCGGTTATTCTAGGGGAATAAATCTTTGGGCACCTAGTGGTCATG

2 CATGACCACTAGGAGCATCTTTGGCGAATAGGCGTCAGCACCGGGTGGGCTCGGTCCCTAGGGGAATAAATCTTTGGGCACCTAGTGGTCATG

2 CATGACCACTAGGAGCATCTTTGGCGACGTAGTGGGCTGGCAGGGAGTACTTGTAGCCTAGGGGAATAAATCTTTGGGCACCTAGTGGTCATG

2 CATGACCACTAGGAGCATCTTTGGCGACCTAACCTGCGCAAGTAGTGTTAGTGTGGACTAGGGGAATAAATCTTTGGGCACCTAGTGGTCATG

2 CATGACCACTAGGAGCATCTTTGGCGAGAGACTGAGATGTGCAATGTGGTCGAGCATCTAGGGGAATAAATCTTTGGGCACCTAGTGGTCATG

2 CATGACCACTAGGAGCATCTTTGGCGATACACGTTGAGACCACACCCAGGTAATCGCCTAGGGGAATAAATCTTTGGGCACCTAGTGGTCATG

2 CATGACCACTAGGAGCATCTTTGGCGACTTCAGTGTGCGGCTGCGCACTTAGTGGAGCTAGGGGAATAAATCTTTGGGCACCTAGTGGTCATG

2 CATGACCACTAGGAGCATCTTTGGCGATGGGTTCTGACGGCCGCCGCTGCGGCTACACTAGGGGAATAAATCTTTGGGCACCTAGTGGTCATG

2 CATGACCACTAGGAGCATCTTTGGCGAGGCGGCTCGTCATCACGTACGGCGATCTAGCTAGGGGAATAAATCTTTGGGCACCTAGTGGTCATG

2 CATGACCACTAGGAGCATCTTTGGCGAGATCGGGAGAATCGGTGGCATTGGTGGACCCTAGGGGAATAAATCTTTGGGCACCTAGTGGTCATG

2 CATGACCACTAGGAGCATCTTTGGCGATGGGTGCTGACGGGGCTAGTGAGCCATACACTAGGGGAATAAATCTTTGGGCACCTAGTGGTCATG

2 CATGACCACTAGGAGCATCTTTGGCGACCCGAGCTCGGGACGAGTCAATCGGGTGGACTAGGGGAATAAATCTTTGGGCACCTAGTGGTCATG

2 CATGACCACTAGGAGCATCTTTGGCGACCCCGACGGCGAAGCTCGGTCGGTGGTGGACTAGGGGAATAAATCTTTGGGCACCTAGTGGTCATG

2 CATGACCACTAGGAGCATCTTTGGCGACCCGATATGTGGGCAACAGAACCGGGTGGACTAGGGGAATAAATCTTTGGGCACCTAGTGGTCATG

2 CATGACCACTAGGAGCATCTTTGGCGAGATCGGGAGAATCGTCGGCATTGGTGTCTCCTAGGGGAATAAATCTTTGGGCACCTAGTGGTCATG

2 CATGACCACTAGGAGCATCTTTGGCGAAGGGTGAAGGACTTCAAGTGGATCTCATAGCTAGGGGAATAAATCTTTGGGCACCTAGTGGTCATG

2 CATGACCACTAGGAGCATCTTTGGCGACGACGTAGGGCACATAGGGACACGCAAAGCCTAGGGGAATAAATCTTTGGGCACCTAGTGGTCATG

2 CATGACCACTAGGAGCATCTTTGGCGACGGCGTGGGGCTATTGATGCGGCGGCTCTCCTAGGGGAATAAATCTTTGGGCACCTAGTGGTCATG

2 CATGACCACTAGGAGCATCTTTGGCGACAGGGGACGCCGATGCGCTGGACGACCGAGCTAGGGGAATAAATCTTTGGGCACCTAGTGGTCATG

2 CATGACCACTAGGAGCATCTTTGGCGATCGGCAGATCGGGTTGGGTGTCGCTATGCCCTAGGGGAATAAATCTTTGGGCACCTAGTGGTCATG

2 CATGACCACTAGGAGCATCTTTGGCGATCGTGGCTGAGCGGTCTAGTAGGGAATCGCCTAGGGGAATAAATCTTTGGGCACCTAGTGGTCATG

2 CATGACCACTAGGAGCATCTTTGGCGACGCGGCTGCAGGGCAACGTGCGCAGTGGAGCTAGGGGAATAAATCTTTGGGCACCTAGTGGTCATG

2 CATGACCACTAGGAGCATCTTTGGCGACGAGTGGGCATTGGGCGATGTCGAGGCAGACTAGGGGAATAAATCTTTGGGCACCTAGTGGTCATG

2 CATGACCACTAGGAGCATCTTTGGCGACTGTGGGTAGGACTGTGAGGGTCTCGGTCGCTAGGGGAATAAATCTTTGGGCACCTAGTGGTCATG

2 CATGACCACTAGGAGCATCTTTGGCGACGCAGCGCGTGTGTCCCAGTGTTGTTGGACCTAGGGGAATAAATCTTTGGGCACCTAGTGGTCATG

2 CATGACCACTAGGAGCATCTTTGGCGACGTGGGTGGTCAAAGCAGTTTGGTTGTCGCCTAGGGGAATAAATCTTTGGGCACCTAGTGGTCATG

2 CATGACCACTAGGAGCATCTTTGGCGACCCTGCGCGAAATGGGCAAAGGTGTGGCGACTAGGGGAATAAATCTTTGGGCACCTAGTGGTCATG

2 CATGACCACTAGGAGCATCTTTGGCGAGAGCCACGAGGTCGCTTCGCTTATCGATGCCTAGGGGAATAAATCTTTGGGCACCTAGTGGTCATG

2 CATGACCACTAGGAGCATCTTTGGCGACGGTGGCACATAGGGCGCTCCAAGGCTCGCCTAGGGGAATAAATCTTTGGGCACCTAGTGGTCATG

2 CATGACCACTAGGAGCATCTTTGGCGAGCCATTGGTATTGCTTGCGCAGCTGCGAGACTAGGGGAATAAATCTTTGGGCACCTAGTGGTCATG

2 CATGACCACTAGGAGCATCTTTGGCGAGTGGGGTCGTGTGAATCGTTGTCTCCTCGTCTAGGGGAATAAATCTTTGGGCACCTAGTGGTCATG

2 CATGACCACTAGGAGCATCTTTGGCGATGGGGTCGTGCTATCTGCACACTCCGCGGGCTAGGGGAATAAATCTTTGGGCACCTAGTGGTCATG

2 CATGACCACTAGGAGCATCTTTGGCGAAAGGCAGGTGTTGCGGTGTTCTGTGAATCCCTAGGGGAATAAATCTTTGGGCACCTAGTGGTCATG

2 CATGACCACTAGGAGCATCTTTGGCGACCCGCATAGACCTGTCGCTAGCGTGGTGGACTAGGGGAATAAATCTTTGGGCACCTAGTGGTCATG

2 CATGACCACTAGGAGCATCTTTGGCGAGGGGTGGGGGGACGGCATGAATGGGCTAGACTAGGGGAATAAATCTTTGGGCACCTAGTGGTCATG

2 CATGACCACTAGGAGCATCTTTGGCGAACGGCAGGTGTTGCGGTGGTCTATGTATCCCTAGGGGAATAAATCTTTGGGCACCTAGTGGTCATG

2 CATGACCACTAGGAGCATCTTTGGCGACGGCAAGCCACGGGGATAAAGGCTCCGAGACTAGGGGAATAAATCTTTGGGCACCTAGTGGTCATG

2 CATGACCACTAGGAGCATCTTTGGCGAACGGCAGGTGTTGAGGTGGTCTGAGAATCCCTAGGGGAATAAATCTTTGGGCACCTAGTGGTCATG

2 CATGACCACTAGGAGCATCTTTGGCGACGTAATGGGCTGGCAGGGAGTACTTGTTGCCTAGGGGAATAAATCTTTGGGCACCTAGTGGTCATG

2 CATGACCACTAGGAGCATCTTTGGCGATCGATCAGGGGCACGAACCGGGGAAGTCGCCTAGGGGAATAAATCTTTGGGCACCTAGTGGTCATG

2 CATGACCACTAGGAGCATCTTTGGCGACCCGGAATCTTGTGGTAGCAACCGGGTGGACTAGGGGAATAAATCTTTGGGCACCTAGTGGTCATG

2 CATGACCACTAGGAGCATCTTTGGCGATTCGGGCGGCCCGGGCGGGGCCCGCACAGCCTAGGGGAATAAATCTTTGGGCACCTAGTGGTCATG

2 CATGACCACTAGGAGCATCTTTGGCGACGATGGGGGGCAGGCGGAACCAGCAGTAGACTAGGGGAATAAATCTTTGGGCACCTAGTGGTCATG

2 CATGACCACTAGGAGCATCTTTGGCGAGTATACCGGCAAGGAAGGGAAACGTTGCGACTAGGGGAATAAATCTTTGGGCACCTAGTGGTCATG

2 CATGACCACTAGGAGCATCTTTGGCGATCCCGTACTATATGGAAAGTACGGTGTGGCCTAGGGGAATAAATCTTTGGGCACCTAGTGGTCATG

2 CATGACCACTAGGAGCATCTTTGGCGACCGTGGTGGTCCGAGCTTCTGGGATGTCGACTAGGGGAATAAATCTTTGGGCACCTAGTGGTCATG

2 CATGACCACTAGGAGCATCTTTGGCGAACGCAGATGGGCCGTGGTTGAGCGTGCAGACTAGGGGAATAAATCTTTGGGCACCTAGTGGTCATG

2 CATGACCACTAGGAGCATCTTTGGCGATGGTGTGGGGACCGCAGGTTCGGCGGGTACCTAGGGGAATAAATCTTTGGGCACCTAGTGGTCATG

2 CATGACCACTAGGAGCATCTTTGGCGACTGTTGGCTGGTAGCATGGTATTAAAGAGGCTAGGGGAATAAATCTTTGGGCACCTAGTGGTCATG

2 CATGACCACTAGGAGCATCTTTGGCGACGAGGGGCACGGGTGGTTATTGCGAGAAGACTAGGGGAATAAATCTTTGGGCACCTAGTGGTCATG

2 CATGACCACTAGGAGCATCTTTGGCGACGTGGGCAGCGAGGAAGAAATGTACGTCGACTAGGGGAATAAATCTTTGGGCACCTAGTGGTCATG

2 CATGACCACTAGGAGCATCTTTGGCGACTGTGGCACATCGGCGGGAGCAACAGTGGACTAGGGGAATAAATCTTTGGGCACCTAGTGGTCATG

2 CATGACCACTAGGAGCATCTTTGGCGAGCTCCGCTGTGGTCGACGGAAAGCGATGCCCTAGGGGAATAAATCTTTGGGCACCTAGTGGTCATG

2 CATGACCACTAGGAGCATCTTTGGCGATAGACAGAGCGGGGCATGGGTTGTTATCGCCTAGGGGAATAAATCTTTGGGCACCTAGTGGTCATG

2 CATGACCACTAGGAGCATCTTTGGCGACAAGTGGTGGATATGTGAGATGCTGTCGCTCTAGGGGAATAAATCTTTGGGCACCTAGTGGTCATG

2 CATGACCACTAGGAGCATCTTTGGCGAGATCAGGAGAATCGCTGGCATTGGTGTCTCCTAGGGGAATAAATCTTTGGGCACCTAGTGGTCATG

2 CATGACCACTAGGAGCATCTTTGGCGAACGGCAGGTGTTGCGGTGGTTTGTGGATCCCTAGGGGAATAAATCTTTGGGCACCTAGTGGTCATG

2 CATGACCACTAGGAGCATCTTTGGCGACAGGGGATGCCGAGGCGCTGGACGACCGAGCTAGGGGAATAAATCTTTGGGCACCTAGTGGTCATG

2 CATGACCACTAGGAGCATCTTTGGCGAGGTCGTAGCTCGCGCGTAGGGTCGGCAGTGCTAGGGGAATAAATCTTTGGGCACCTAGTGGTCATG

2 CATGACCACTAGGAGCATCTTTGGCGACCCCGGACAGGACTGTGTCAGAGGGGTGGACTAGGGGAATAAATCTTTGGGCACCTAGTGGTCATG

2 CATGACCACTAGGAGCATCTTTGGCGATGGTGTGGGGAACTTGTTTCGGCGGTGCTACTAGGGGAATAAATCTTTGGGCACCTAGTGGTCATG

2 CATGACCACTAGGAGCATCTTTGGCGACACCACTTACGCATGGCGTATGGTTGTGGACTAGGGGAATAAATCTTTGGGCACCTAGTGGTCATG

2 CATGACCACTAGGAGCATCTTTGGCGACCCGATTGACGCATCGGCGATCGAGGTGGACTAGGGGAATAAATCTTTGGGCACCTAGTGGTCATG

2 CATGACCACTAGGAGCATCTTTGGCGACCCCGTGCGCACGCACGTGGAGTGTTGACACTAGGGGAATAAATCTTTGGGCACCTAGTGGTCATG

2 CATGACCACTAGGAGCATCTTTGGCGATCCTCGTCAGATGGTGAAGCATACGTTTGGCTAGGGGAATAAATCTTTGGGCACCTAGTGGTCATG

2 CATGACCACTAGGAGCATCTTTGGCGAAGCGTGTTACAAGGTCCGCGAGTGTGGACCCTAGGGGAATAAATCTTTGGGCACCTAGTGGTCATG

2 CATGACCACTAGGAGCATCTTTGGCGAACGGGGAGTTGACTGGATAGTCGGGGGAAGCTAGGGGAATAAATCTTTGGGCACCTAGTGGTCATG

2 CATGACCACTAGGAGCATCTTTGGCGACCCGTACCTGTGGCTACACAAGCGGGTGGACTAGGGGAATAAATCTTTGGGCACCTAGTGGTCATG

2 CATGACCACTAGGAGCATCTTTGGCGACCCGGGCATTACTGATATGACTGTGGTGGACTAGGGGAATAAATCTTTGGGCACCTAGTGGTCATG

2 CATGACCACTAGGAGCATCTTTGGCGAGTTCTCCGCCGAGAATGAGCAGACGTTCGGCTAGGGGAATAAATCTTTGGGCACCTAGTGGTCATG

2 CATGACCACTAGGAGCATCTTTGGCGAACGTTAAGATGGACATACGAATATAACTAGCTAGGGGAATAAATCTTTGGGCACCTAGTGGTCATG

2 CATGACCACTAGGAGCATCTTTGGCGAACGACGCAGTCAACACAAGGGAAAGAGCGACTAGGGGAATAAATCTTTGGGCACCTAGTGGTCATG

2 CATGACCACTAGGAGCATCTTTGGCGACATATGCAACTACGTTGCCGAAGTGGACGACTAGGGGAATAAATCTTTGGGCACCTAGTGGTCATG

2 CATGACCACTAGGAGCATCTTTGGCGAGAGGGACCGAACCTGCAGATTGCTCGATGCCTAGGGGAATAAATCTTTGGGCACCTAGTGGTCATG

2 CATGACCACTAGGAGCATCTTTGGCGACTGAGGGGCGGACGCTCGTCACGAAGTGGACTAGGGGAATAAATCTTTGGGCACCTAGTGGTCATG

2 CATGACCACTAGGAGCATCTTTGGCGACAGCTGTCGACGCGTTAGGGGCTGCGTGAGCTAGGGGAATAAATCTTTGGGCACCTAGTGGTCATG

2 CATGACCACTAGGAGCATCTTTGGCGAGTATTGGCTGGCAGGTTGCGTATTGGGGAGCTAGGGGAATAAATCTTTGGGCACCTAGTGGTCATG

2 CATGACCACTAGGAGCATCTTTGGCGAACGTGCAAGATATGCCGCCTGGAGCGTATGCTAGGGGAATAAATCTTTGGGCACCTAGTGGTCATG

2 CATGACCACTAGGAGCATCTTTGGCGAGGAGTGGCAGAGGGCGGTGGGTAGGTCGCGCTAGGGGAATAAATCTTTGGGCACCTAGTGGTCATG

2 CATGACCACTAGGAGCATCTTTGGCGAGTTCTACCGCTGAGGGCGCCCGGGCCGAGACTAGGGGAATAAATCTTTGGGCACCTAGTGGTCATG

2 CATGACCACTAGGAGCATCTTTGGCGACGGAGTGGGGCTCATTGTGCGGCGGTAATGCTAGGGGAATAAATCTTTGGGCACCTAGTGGTCATG

2 CATGACCACTAGGAGCATCTTTGGCGAGATCGGTAGAATCGGTGGCATTGTTGTCTCCTAGGGGAATAAATCTTTGGGCACCTAGTGGTCATG

2 CATGACCACTAGGAGCATCTTTGGCGACTCCAATGGTTCACCCATTGGGATGTGGAGCTAGGGGAATAAATCTTTGGGCACCTAGTGGTCATG

2 CATGACCACTAGGAGCATCTTTGGCGAAGTGTGTAGGACTTCAAGTGGATCTCATAGCTAGGGGAATAAATCTTTGGGCACCTAGTGGTCATG

2 CATGACCACTAGGAGCATCTTTGGCGAAAAAACGGGTACAAGGTGGTCCGTTGTCGCCTAGGGGAATAAATCTTTGGGCACCTAGTGGTCATG

2 CATGACCACTAGGAGCATCTTTGGCGACGCATGTACATATGGTTGATGTACCGGTTTCTAGGGGAATAAATCTTTGGGCACCTAGTGGTCATG

2 CATGACCACTAGGAGCATCTTTGGCGAGGGGAGGCTTTAGTGGTCGTGGAGTGGCGCCTAGGGGAATAAATCTTTGGGCACCTAGTGGTCATG

2 CATGACCACTAGGAGCATCTTTGGCGAGGGGGGACTCCGAAACGAATAGGGACGAGCCTAGGGGAATAAATCTTTGGGCACCTAGTGGTCATG

2 CATGACCACTAGGAGCATCTTTGGCGAAACCTATCAGGCGGAGACAAATTGGTTTCCCTAGGGGAATAAATCTTTGGGCACCTAGTGGTCATG

2 CATGACCACTAGGAGCATCTTTGGCGAGTGGGGGTCGGCCAGGGCTGTGGTGGGAGGCTAGGGGAATAAATCTTTGGGCACCTAGTGGTCATG

2 CATGACCACTAGGAGCATCTTTGGCGAACGCGAATTGACGTCCAGGGTATGACTCAACTAGGGGAATAAATCTTTGGGCACCTAGTGGTCATG

2 CATGACCACTAGGAGCATCTTTGGCGAGAGGACTCTGGACTCGGGGACCAGTTGCTGCTAGGGGAATAAATCTTTGGGCACCTAGTGGTCATG

2 CATGACCACTAGGAGCATCTTTGGCGACACCGTCCAATCTGTGAGACGGTTGTGGAGCTAGGGGAATAAATCTTTGGGCACCTAGTGGTCATG

2 CATGACCACTAGGAGCATCTTTGGCGACTAAACTCGTACGCGAGTGGCCCGGAGAGCCTAGGGGAATAAATCTTTGGGCACCTAGTGGTCATG

2 CATGACCACTAGGAGCATCTTTGGCGAGATCCTGAGAATCGGTGGCATTGGTGTCTCCTAGGGGAATAAATCTTTGGGCACCTAGTGGTCATG

2 CATGACCACTAGGAGCATCTTTGGCGAGACGTGTGGGGACCGTCGGGAGAAGTGAGCCTAGGGGAATAAATCTTTGGGCACCTAGTGGTCATG

2 CATGACCACTAGGAGCATCTTTGGCGAGTGAGCGGGAGGAACGCCGCGGAGAAACGCCTAGGGGAATAAATCTTTGGGCACCTAGTGGTCATG

2 CATGACCACTAGGAGCATCTTTGGCGAGATCGTGAGAATCGGTGGCACTGGTGTCTCCTAGGGGAATAAATCTTTGGGCACCTAGTGGTCATG

2 CATGACCACTAGGAGCATCTTTGGCGAGATAGGGAGAATCGGTGGCATTGGTTTCTCCTAGGGGAATAAATCTTTGGGCACCTAGTGGTCATG

2 CATGACCACTAGGAGCATCTTTGGCGACCCGCGACTGGCAAGGATGCGCGGTGGCTTCTAGGGGAATAAATCTTTGGGCACCTAGTGGTCATG

2 CATGACCACTAGGAGCATCTTTGGCGAAGGCGGGCTGGTAGGTGCGTACTTCTGGAGCTAGGGGAATAAATCTTTGGGCACCTAGTGGTCATG

2 CATGACCACTAGGAGCATCTTTGGCGAGGGGGCGAGTGGGCGAGGTGTTGTAGGGAGCTAGGGGAATAAATCTTTGGGCACCTAGTGGTCATG

2 CATGACCACTAGGAGCATCTTTGGCGAAGAGCGCAATGGTCGTGTTGACTGAGCCTACTAGGGGAATAAATCTTTGGGCACCTAGTGGTCATG

2 CATGACCACTAGGAGCATCTTTGGCGATGTTCGAACTCGGGTGTGGTAGGATTGCGACTAGGGGAATAAATCTTTGGGCACCTAGTGGTCATG

2 CATGACCACTAGGAGCATCTTTGGCGAAGGTCAATAAATGCGAGAATCTTAGCTGAGCTAGGGGAATAAATCTTTGGGCACCTAGTGGTCATG

2 CATGACCACTAGGAGCATCTTTGGCGAAGCGGGAAGGCACGCCGACTAAGCTTGCGACTAGGGGAATAAATCTTTGGGCACCTAGTGGTCATG

2 CATGACCACTAGGAGCATCTTTGGCGACCGGCTGTTATGCGTAATTGAACGGTTTGCCTAGGGGAATAAATCTTTGGGCACCTAGTGGTCATG

2 CATGACCACTAGGAGCATCTTTGGCGACTAGGGTCTGCAAGGCATGCTTCAAGGCGCCTAGGGGAATAAATCTTTGGGCACCTAGTGGTCATG

2 CATGACCACTAGGAGCATCTTTGGCGATGGGTGCTGAAGGGGCTAGTGAGCCCTACACTAGGGGAATAAATCTTTGGGCACCTAGTGGTCATG

2 CATGACCACTAGGAGCATCTTTGGCGAGGTCGGCTGGTAGAGACTGTATTGAGGAACCTAGGGGAATAAATCTTTGGGCACCTAGTGGTCATG

2 CATGACCACTAGGAGCATCTTTGGCGAAAGCGAAAGGGGTCACGGCTCGCGCAACGACTAGGGGAATAAATCTTTGGGCACCTAGTGGTCATG

2 CATGACCACTAGGAGCATCTTTGGCGATGTACTTGCGTCCGAACTGTTGTCGATCGCCTAGGGGAATAAATCTTTGGGCACCTAGTGGTCATG

2 CATGACCACTAGGAGCATCTTTGGCGACTAAGTGCAGACGGCGACACCGTGAGGCGACTAGGGGAATAAATCTTTGGGCACCTAGTGGTCATG

2 CATGACCACTAGGAGCATCTTTGGCGATCGCATGTGGGCAATTGATCGGACGAGCGACTAGGGGAATAAATCTTTGGGCACCTAGTGGTCATG

2 CATGACCACTAGGAGCATCTTTGGCGAGATCGGGAGAATCTGAGGCATTGGTGTCTCCTAGGGGAATAAATCTTTGGGCACCTAGTGGTCATG

2 CATGACCACTAGGAGCATCTTTGGCGATCCTCGTCAGATGGTGAAGCAGACGTCTGGCTAGGGGAATAAATCTTTGGGCACCTAGTGGTCATG

2 CATGACCACTAGGAGCATCTTTGGCGAGAGCGCGGTAACTGGAGGGTACTAGAGCCGCTAGGGGAATAAATCTTTGGGCACCTAGTGGTCATG

2 CATGACCACTAGGAGCATCTTTGGCGAATTGGTTGGCCTTAGCAAAGAGTGATTCGCCTAGGGGAATAAATCTTTGGGCACCTAGTGGTCATG

2 CATGACCACTAGGAGCATCTTTGGCGAGAAGACTCTGGATTCTGGGACCAGTTTCTGCTAGGGGAATAAATCTTTGGGCACCTAGTGGTCATG

2 CATGACCACTAGGAGCATCTTTGGCGACGGGGGTACATGTATGATGCTTAAAAGGCGCTAGGGGAATAAATCTTTGGGCACCTAGTGGTCATG

2 CATGACCACTAGGAGCATCTTTGGCGACACCGGAGTTAAAGGCATGCTGGCGTGGAGCTAGGGGAATAAATCTTTGGGCACCTAGTGGTCATG

2 CATGACCACTAGGAGCATCTTTGGCGACCACGGTGGGCGGATAATGAAGAAGGTCGACTAGGGGAATAAATCTTTGGGCACCTAGTGGTCATG

2 CATGACCACTAGGAGCATCTTTGGCGATATCGGGAGAATCGTTGGCATTGGTGTCTCCTAGGGGAATAAATCTTTGGGCACCTAGTGGTCATG

2 CATGACCACTAGGAGCATCTTTGGCGATGGGGCCGGGCGCACAGACCACCTCGCGGCCTAGGGGAATAAATCTTTGGGCACCTAGTGGTCATG

2 CATGACCACTAGGAGCATCTTTGGCGAGCGAACCGGGCGGGACCATACGGCGCGAAACTAGGGGAATAAATCTTTGGGCACCTAGTGGTCATG

2 CATGACCACTAGGAGCATCTTTGGCGACGAAGCAGCGATCGGTGCATGCGAAGTGGACTAGGGGAATAAATCTTTGGGCACCTAGTGGTCATG

2 CATGACCACTAGGAGCATCTTTGGCGAAGCGAGGGTAAGTGCATGGCGGCGGGCCATCTAGGGGAATAAATCTTTGGGCACCTAGTGGTCATG

2 CATGACCACTAGGAGCATCTTTGGCGATGAGCTGTGATGGTTGTGTACTATTTTGAGCTAGGGGAATAAATCTTTGGGCACCTAGTGGTCATG

2 CATGACCACTAGGAGCATCTTTGGCGAAAGCGCAGGGCATGCGACCTCCAAGTGGTCCTAGGGGAATAAATCTTTGGGCACCTAGTGGTCATG

2 CATGACCACTAGGAGCATCTTTGGCGATCAGTAGGACGGGAGCACGGTATGGATCGACTAGGGGAATAAATCTTTGGGCACCTAGTGGTCATG

2 CATGACCACTAGGAGCATCTTTGGCGAAGCGCAGGGTGTGAGAGGGGCGGGGCCATGCTAGGGGAATAAATCTTTGGGCACCTAGTGGTCATG

2 CATGACCACTAGGAGCATCTTTGGCGACCCTCGTGGTTACCTATAAAGGTGTGGAGCCTAGGGGAATAAATCTTTGGGCACCTAGTGGTCATG

2 CATGACCACTAGGAGCATCTTTGGCGACGACGTTGGGTCAGGTGGTACGAGAGGAGACTAGGGGAATAAATCTTTGGGCACCTAGTGGTCATG

2 CATGACCACTAGGAGCATCTTTGGCGACACGCGGTTCTGAGAGGCAGTGTAGTGGAGCTAGGGGAATAAATCTTTGGGCACCTAGTGGTCATG

2 CATGACCACTAGGAGCATCTTTGGCGACCCAGCACGTTGAGGGATGCGTTGTGGAGACTAGGGGAATAAATCTTTGGGCACCTAGTGGTCATG

2 CATGACCACTAGGAGCATCTTTGGCGACAGGGGGTGCCGATGCGCTGGACGACCGAGCTAGGGGAATAAATCTTTGGGCACCTAGTGGTCATG

2 CATGACCACTAGGAGCATCTTTGGCGAACGGCAGGTGTTGCGGCGGTTTGTGAATCCCTAGGGGAATAAATCTTTGGGCACCTAGTGGTCATG

2 CATGACCACTAGGAGCATCTTTGGCGACTGGGGGCACGGCAACAGCACTACCGGAGACTAGGGGAATAAATCTTTGGGCACCTAGTGGTCATG

2 CATGACCACTAGGAGCATCTTTGGCGACGGAGTGGGGCCATCTGTGCGGCGGATCCCCTAGGGGAATAAATCTTTGGGCACCTAGTGGTCATG

2 CATGACCACTAGGAGCATCTTTGGCGAGAGCGTGAGAATCGGTGGCATTGGTGTCTCCTAGGGGAATAAATCTTTGGGCACCTAGTGGTCATG

2 CATGACCACTAGGAGCATCTTTGGCGACCCAGTATGCGTGTCAGCATCTGTGGTGGACTAGGGGAATAAATCTTTGGGCACCTAGTGGTCATG

2 CATGACCACTAGGAGCATCTTTGGCGAAGTATAAGATCGGTCTCAAGTATTTTTCGCCTAGGGGAATAAATCTTTGGGCACCTAGTGGTCATG

2 CATGACCACTAGGAGCATCTTTGGCGAGCTATGAGGTCAGGAAATAGTGAAACGAGCCTAGGGGAATAAATCTTTGGGCACCTAGTGGTCATG

2 CATGACCACTAGGAGCATCTTTGGCGACAAGATAAAGTAGAACATAATCGAAGTGGACTAGGGGAATAAATCTTTGGGCACCTAGTGGTCATG

2 CATGACCACTAGGAGCATCTTTGGCGACCCATGCGAACGTAAGGGAGCAAGGTGTGACTAGGGGAATAAATCTTTGGGCACCTAGTGGTCATG

2 CATGACCACTAGGAGCATCTTTGGCGAGATCGTGAGAATCGGTGGCATTGGTGTCCCCTAGGGGAATAAATCTTTGGGCACCTAGTGGTCATG

2 CATGACCACTAGGAGCATCTTTGGCGACAAGTGAGCGACGTCATGGAGAGATAGCGCCTAGGGGAATAAATCTTTGGGCACCTAGTGGTCATG

2 CATGACCACTAGGAGCATCTTTGGCGACACGCATATCCTAGATATGGCGTTGTGGCTCTAGGGGAATAAATCTTTGGGCACCTAGTGGTCATG

2 CATGACCACTAGGAGCATCTTTGGCGACCCTGCCGACGTAAGTGGTAGGAGTGTTGACTAGGGGAATAAATCTTTGGGCACCTAGTGGTCATG

2 CATGACCACTAGGAGCATCTTTGGCGACCCGGAGCCGCGGACGGCTTCCGAGGTGGACTAGGGGAATAAATCTTTGGGCACCTAGTGGTCATG

2 CATGACCACTAGGAGCATCTTTGGCGAATTAACTCCCCAGACCTGGGAGTAAGTAGGCTAGGGGAATAAATCTTTGGGCACCTAGTGGTCATG

2 CATGACCACTAGGAGCATCTTTGGCGAAGGGTGTAGGACTTCAAGTGGAGCTCATAGCTAGGGGAATAAATCTTTGGGCACCTAGTGGTCATG

2 CATGACCACTAGGAGCATCTTTGGCGAGGGGTAGGGGGCCGCTCGCGCCTGCGTTCGCTAGGGGAATAAATCTTTGGGCACCTAGTGGTCATG

2 CATGACCACTAGGAGCATCTTTGGCGATGGGTGCTGACGGCCGCCGCTGCGGCCACACTAGGGGAATAAATCTTTGGGCACCTAGTGGTCATG

2 CATGACCACTAGGAGCATCTTTGGCGACAGCGTCGTGGCGTGTGTGGGTTGCCGCAGCTAGGGGAATAAATCTTTGGGCACCTAGTGGTCATG

2 CATGACCACTAGGAGCATCTTTGGCGACAAGAGGAAACCACACTGCGGAGAGGTCGACTAGGGGAATAAATCTTTGGGCACCTAGTGGTCATG

2 CATGACCACTAGGAGCATCTTTGGCGAACGTGCAAGATATGCCGCCTGGAGCGAAGGCTAGGGGAATAAATCTTTGGGCACCTAGTGGTCATG

2 CATGACCACTAGGAGCATCTTTGGCGAGGCTTTCGGTGGTAAGGACTGCGTCTAGAGCTAGGGGAATAAATCTTTGGGCACCTAGTGGTCATG

2 CATGACCACTAGGAGCATCTTTGGCGAGCGTACGTGAGCCTGAATGCGAGTAGCTCGCTAGGGGAATAAATCTTTGGGCACCTAGTGGTCATG

2 CATGACCACTAGGAGCATCTTTGGCGACCAGTGTGGCAGAGATCGAGTGTAGGTCGACTAGGGGAATAAATCTTTGGGCACCTAGTGGTCATG

2 CATGACCACTAGGAGCATCTTTGGCGAGGGGCAGGGGGCCGCTCGCGCCTGCGTGCGCTAGGGGAATAAATCTTTGGGCACCTAGTGGTCATG

2 CATGACCACTAGGAGCATCTTTGGCGAAGCAGAGCGACGGGCTATGGGTTCCTCGAGCTAGGGGAATAAATCTTTGGGCACCTAGTGGTCATG

2 CATGACCACTAGGAGCATCTTTGGCGACACTGGGTAAGGAACCGTGGCATACGTCGACTAGGGGAATAAATCTTTGGGCACCTAGTGGTCATG

2 CATGACCACTAGGAGCATCTTTGGCGAGAGGAACACCGGGGAGCTAAGGGGTCGAGACTAGGGGAATAAATCTTTGGGCACCTAGTGGTCATG

2 CATGACCACTAGGAGCATCTTTGGCGATTCGTATTAGATAACTCAGAATAGTCCGCCCTAGGGGAATAAATCTTTGGGCACCTAGTGGTCATG

2 CATGACCACTAGGAGCATCTTTGGCGAACAGGTGCGCTGTCCCGGTTGCGACGTGAGCTAGGGGAATAAATCTTTGGGCACCTAGTGGTCATG

2 CATGACCACTAGGAGCATCTTTGGCGACGGGGCCGGACGGGACGTGCACATCGCGGCCTAGGGGAATAAATCTTTGGGCACCTAGTGGTCATG

2 CATGACCACTAGGAGCATCTTTGGCGACGGGTCATCGCACGGCCTACGAAAAGTCGACTAGGGGAATAAATCTTTGGGCACCTAGTGGTCATG

2 CATGACCACTAGGAGCATCTTTGGCGAGGGGTAGGGGGCCGCTCGCGCCTGCGTGCACTAGGGGAATAAATCTTTGGGCACCTAGTGGTCATG

2 CATGACCACTAGGAGCATCTTTGGCGAGTTGCGACGGTTGGAAACCCGGACCACCGCCTAGGGGAATAAATCTTTGGGCACCTAGTGGTCATG

2 CATGACCACTAGGAGCATCTTTGGCGAGATCGGGAGAGTCGGTGGCATTGGTGTCTACTAGGGGAATAAATCTTTGGGCACCTAGTGGTCATG

2 CATGACCACTAGGAGCATCTTTGGCGAAGCGTTTTGCAAGGTCCGCGAGTGTGGACCCTAGGGGAATAAATCTTTGGGCACCTAGTGGTCATG

2 CATGACCACTAGGAGCATCTTTGGCGAGCGGCCTGAGGAACGATCGCAGACGTTTGGCTAGGGGAATAAATCTTTGGGCACCTAGTGGTCATG

2 CATGACCACTAGGAGCATCTTTGGCGAACCATATAACGTATGTGCTTAAGCGATCGACTAGGGGAATAAATCTTTGGGCACCTAGTGGTCATG

2 CATGACCACTAGGAGCATCTTTGGCGAATCTTGATAGGGATCGTAGAAGAAGTGAGACTAGGGGAATAAATCTTTGGGCACCTAGTGGTCATG

2 CATGACCACTAGGAGCATCTTTGGCGATGGTCGGTAGGATGGTTCGGTATTTCGTCTCTAGGGGAATAAATCTTTGGGCACCTAGTGGTCATG

2 CATGACCACTAGGAGCATCTTTGGCGACAGGGGTACCAGTCAGCACGTTGGGTCGCTCTAGGGGAATAAATCTTTGGGCACCTAGTGGTCATG

2 CATGACCACTAGGAGCATCTTTGGCGAACGTCCCGACAGCGAATTCAAAGAGAGCCGCTAGGGGAATAAATCTTTGGGCACCTAGTGGTCATG

2 CATGACCACTAGGAGCATCTTTGGCGAGAAGACTCTGCATTCGGGGACCAGTTGCTGCTAGGGGAATAAATCTTTGGGCACCTAGTGGTCATG

2 CATGACCACTAGGAGCATCTTTGGCGACTAGAGGGCCGGCACAGCGACGAGAGGAGACTAGGGGAATAAATCTTTGGGCACCTAGTGGTCATG

2 CATGACCACTAGGAGCATCTTTGGCGAGGTGTCTCAGTTCGACATAAGGGGCGAGCGCTAGGGGAATAAATCTTTGGGCACCTAGTGGTCATG

2 CATGACCACTAGGAGCATCTTTGGCGACGCGGTAGACCACGAGGTGTAACCGTCGACCTAGGGGAATAAATCTTTGGGCACCTAGTGGTCATG

2 CATGACCACTAGGAGCATCTTTGGCGAGGGACGGGGTCAGCTCGACCAATAGCGAGCCTAGGGGAATAAATCTTTGGGCACCTAGTGGTCATG

2 CATGACCACTAGGAGCATCTTTGGCGAAAGTCGGTTGAACAGGGTGGGGTGCTGTCCCTAGGGGAATAAATCTTTGGGCACCTAGTGGTCATG

2 CATGACCACTAGGAGCATCTTTGGCGACAGCGTAGGCGGATGCTTGGCTAGGACATTCTAGGGGAATAAATCTTTGGGCACCTAGTGGTCATG

2 CATGACCACTAGGAGCATCTTTGGCGAGATCAGGAGAATCGGTGGCATTGGTGCCTCCTAGGGGAATAAATCTTTGGGCACCTAGTGGTCATG

2 CATGACCACTAGGAGCATCTTTGGCGACGGACGATTCCGAAGGAGTTCTAGTTGCCGCTAGGGGAATAAATCTTTGGGCACCTAGTGGTCATG

2 CATGACCACTAGGAGCATCTTTGGCGACCCGGGTAAGCGGAACCTAAACCGGGTGGACTAGGGGAATAAATCTTTGGGCACCTAGTGGTCATG

2 CATGACCACTAGGAGCATCTTTGGCGAGTATTGGCTGGAAGGTTGCGTATTGGGGAGCTAGGGGAATAAATCTTTGGGCACCTAGTGGTCATG

2 CATGACCACTAGGAGCATCTTTGGCGACAGCGGCGTAGGCTGACTTGGGTGGTGGCCCTAGGGGAATAAATCTTTGGGCACCTAGTGGTCATG

2 CATGACCACTAGGAGCATCTTTGGCGAGCACGCCGTAAGGCGGACGAAGTGGAGCTCCTAGGGGAATAAATCTTTGGGCACCTAGTGGTCATG

2 CATGACCACTAGGAGCATCTTTGGCGATGGTTGCTGACGGGGCTAGTGAGCCCTACACTAGGGGAATAAATCTTTGGGCACCTAGTGGTCATG

2 CATGACCACTAGGAGCATCTTTGGCGACCTTGCATACATTGGGGAGCAGACGTTTGGCTAGGGGAATAAATCTTTGGGCACCTAGTGGTCATG

2 CATGACCACTAGGAGCATCTTTGGCGATAGGCGCGTACGGTCGAGCGAAAGCGATAGCTAGGGGAATAAATCTTTGGGCACCTAGTGGTCATG

2 CATGACCACTAGGAGCATCTTTGGCGAAAGAGCCGACGCTAGGAGCTAGTGTAACTACTAGGGGAATAAATCTTTGGGCACCTAGTGGTCATG

2 CATGACCACTAGGAGCATCTTTGGCGACTAGCGGGCAGCTGTAGGACATACGTCGCTCTAGGGGAATAAATCTTTGGGCACCTAGTGGTCATG

2 CATGACCACTAGGAGCATCTTTGGCGAGTAGGAACGGCTAGTGACAGACCAGATCGACTAGGGGAATAAATCTTTGGGCACCTAGTGGTCATG

2 CATGACCACTAGGAGCATCTTTGGCGACAGGGGTGGTCGGCCACGGTGAATTGTCGACTAGGGGAATAAATCTTTGGGCACCTAGTGGTCATG

2 CATGACCACTAGGAGCATCTTTGGCGACGGTGTGGGGAACTTGTTTCGGCGGTCCTACTAGGGGAATAAATCTTTGGGCACCTAGTGGTCATG

2 CATGACCACTAGGAGCATCTTTGGCGACACCCGTGTGCTCGCGCACGGAGGTGTGGACTAGGGGAATAAATCTTTGGGCACCTAGTGGTCATG

2 CATGACCACTAGGAGCATCTTTGGCGACGACTATGATTGTGTCAGCCGGGAGGTAGACTAGGGGAATAAATCTTTGGGCACCTAGTGGTCATG

2 CATGACCACTAGGAGCATCTTTGGCGAGCCGATTGTGCGACAATGTGTTCTGGGTAACTAGGGGAATAAATCTTTGGGCACCTAGTGGTCATG

2 CATGACCACTAGGAGCATCTTTGGCGACGACGGGGGCACTCAGGGTACAAATGTCGACTAGGGGAATAAATCTTTGGGCACCTAGTGGTCATG

2 CATGACCACTAGGAGCATCTTTGGCGAGGGGACCAGTGCAACGAACAGATCCCTCCGCTAGGGGAATAAATCTTTGGGCACCTAGTGGTCATG

2 CATGACCACTAGGAGCATCTTTGGCGACAGTTGTCGGGAATATACCTCGTATAGGTGCTAGGGGAATAAATCTTTGGGCACCTAGTGGTCATG

2 CATGACCACTAGGAGCATCTTTGGCGACGGCGTGGGGCAATCTGTGCGGCGGATCCCCTAGGGGAATAAATCTTTGGGCACCTAGTGGTCATG

2 CATGACCACTAGGAGCATCTTTGGCGATAGTGGTCTGATATAGAATTAACGTCAAGCCTAGGGGAATAAATCTTTGGGCACCTAGTGGTCATG

2 CATGACCACTAGGAGCATCTTTGGCGAAAAGGGCGAGTAATCCGAGGAAAAATAGGTCTAGGGGAATAAATCTTTGGGCACCTAGTGGTCATG

2 CATGACCACTAGGAGCATCTTTGGCGAGTAGGGGGCAACCGACGACAGTAAACGAGACTAGGGGAATAAATCTTTGGGCACCTAGTGGTCATG

2 CATGACCACTAGGAGCATCTTTGGCGACAGTGTGGAGCATCTATGCTGCGTGCATAGCTAGGGGAATAAATCTTTGGGCACCTAGTGGTCATG

2 CATGACCACTAGGAGCATCTTTGGCGACGCGGGACGCATGCAATGCTCCGACGTGGACTAGGGGAATAAATCTTTGGGCACCTAGTGGTCATG

2 CATGACCACTAGGAGCATCTTTGGCGACCCGAGACCCGTAGGGTCGTTCCGGGTGGACTAGGGGAATAAATCTTTGGGCACCTAGTGGTCATG

2 CATGACCACTAGGAGCATCTTTGGCGACGATATAGTACAGGGATACGGCATACTCGCCTAGGGGAATAAATCTTTGGGCACCTAGTGGTCATG

2 CATGACCACTAGGAGCATCTTTGGCGAGTATTGGCTGGTAGGTTGCGTATTAGGGAGCTAGGGGAATAAATCTTTGGGCACCTAGTGGTCATG

2 CATGACCACTAGGAGCATCTTTGGCGACGCCAGAACACGAGGTGTATCTGGTGTGGACTAGGGGAATAAATCTTTGGGCACCTAGTGGTCATG

2 CATGACCACTAGGAGCATCTTTGGCGACGTTATAGTGGGCATGTACCGGCCGGTCGCCTAGGGGAATAAATCTTTGGGCACCTAGTGGTCATG

2 CATGACCACTAGGAGCATCTTTGGCGAAGGTTGGCTGGGAGCTGGACGTAACCGAGACTAGGGGAATAAATCTTTGGGCACCTAGTGGTCATG

2 CATGACCACTAGGAGCATCTTTGGCGACGAGGCGGGTCGCTCCGCAGCAGGAGGAGACTAGGGGAATAAATCTTTGGGCACCTAGTGGTCATG

2 CATGACCACTAGGAGCATCTTTGGCGACCCAGCCGTGTGCACGGTTGGAGTGTTGCTCTAGGGGAATAAATCTTTGGGCACCTAGTGGTCATG

2 CATGACCACTAGGAGCATCTTTGGCGACCCGACAGCGTAACGTCGTCTGCGGGTGGACTAGGGGAATAAATCTTTGGGCACCTAGTGGTCATG

2 CATGACCACTAGGAGCATCTTTGGCGAGCGGCCGGCACGGCCTTCTAGTCCTCGGTACTAGGGGAATAAATCTTTGGGCACCTAGTGGTCATG

2 CATGACCACTAGGAGCATCTTTGGCGAGCCGATTGTGCAGCAATGTGTTCTGGGTAACTAGGGGAATAAATCTTTGGGCACCTAGTGGTCATG

2 CATGACCACTAGGAGCATCTTTGGCGAGCCTGAGGGGACCGGGAAAGGGCGTACCATCTAGGGGAATAAATCTTTGGGCACCTAGTGGTCATG

2 CATGACCACTAGGAGCATCTTTGGCGATCGTGGGCTGGAAGGTATGTACTTTGAACGCTAGGGGAATAAATCTTTGGGCACCTAGTGGTCATG

2 CATGACCACTAGGAGCATCTTTGGCGAGGGTTCATGGGAATGTAATTGCGCTTAAGACTAGGGGAATAAATCTTTGGGCACCTAGTGGTCATG

2 CATGACCACTAGGAGCATCTTTGGCGATGGGTGCTGACGGCCGCCGCTGTGGCTACACTAGGGGAATAAATCTTTGGGCACCTAGTGGTCATG

2 CATGACCACTAGGAGCATCTTTGGCGACGTCGTAAAGTGCGTAGAGTAGTAAGTCGACTAGGGGAATAAATCTTTGGGCACCTAGTGGTCATG

2 CATGACCACTAGGAGCATCTTTGGCGACAGACGCCGGACACGACGTCATAAGTGGAGCTAGGGGAATAAATCTTTGGGCACCTAGTGGTCATG

2 CATGACCACTAGGAGCATCTTTGGCGACCCGACGCCCGACGGGCGGGACAGGGTGGACTAGGGGAATAAATCTTTGGGCACCTAGTGGTCATG

2 CATGACCACTAGGAGCATCTTTGGCGAGTGATCGGATCCCGCCAAGAATGGTCGAGACTAGGGGAATAAATCTTTGGGCACCTAGTGGTCATG

2 CATGACCACTAGGAGCATCTTTGGCGAGGTTGTGGCTCGCGCGTAGGGTCGGCAGTGCTAGGGGAATAAATCTTTGGGCACCTAGTGGTCATG

2 CATGACCACTAGGAGCATCTTTGGCGATGGGGCTGTGCTATCTGCACACTCCGCGGGCTAGGGGAATAAATCTTTGGGCACCTAGTGGTCATG

2 CATGACCACTAGGAGCATCTTTGGCGAAGCGGTGCGACGGGCTATGGGTTCCTCGAGCTAGGGGAATAAATCTTTGGGCACCTAGTGGTCATG

2 CATGACCACTAGGAGCATCTTTGGCGATACGTCGACGAGCTGAGCGCTGGCCGTCGCCTAGGGGAATAAATCTTTGGGCACCTAGTGGTCATG

2 CATGACCACTAGGAGCATCTTTGGCGAGGGCCCGGGGTGCGTGCTTGCAGGGTCGAGCTAGGGGAATAAATCTTTGGGCACCTAGTGGTCATG

2 CATGACCACTAGGAGCATCTTTGGCGAGTTGTCGGAGAGCGCCCGCATGGGTCAGCCCTAGGGGAATAAATCTTTGGGCACCTAGTGGTCATG

2 CATGACCACTAGGAGCATCTTTGGCGACGGTGTGGAAAAGGATCGTAAGATCGTATACTAGGGGAATAAATCTTTGGGCACCTAGTGGTCATG

2 CATGACCACTAGGAGCATCTTTGGCGACATGGGCGACCTAAGAGAGGAAAAGTCGAACTAGGGGAATAAATCTTTGGGCACCTAGTGGTCATG

2 CATGACCACTAGGAGCATCTTTGGCGATCAGATCCCGGGAGTGTGAGAGGTCCAGCGCTAGGGGAATAAATCTTTGGGCACCTAGTGGTCATG

2 CATGACCACTAGGAGCATCTTTGGCGAGGGAGGGCGCCGGCTGCGGTGTGAATGCGCCTAGGGGAATAAATCTTTGGGCACCTAGTGGTCATG

2 CATGACCACTAGGAGCATCTTTGGCGAGAACACTCTGGATTCGGGGACCAGTTGCTGCTAGGGGAATAAATCTTTGGGCACCTAGTGGTCATG

2 CATGACCACTAGGAGCATCTTTGGCGAAGCGGAGGAGCTCGTAAGGCGGCGTACCGGCTAGGGGAATAAATCTTTGGGCACCTAGTGGTCATG

2 CATGACCACTAGGAGCATCTTTGGCGACGGCGTGGGGCTATTGATGCGGCGGTACCCCTAGGGGAATAAATCTTTGGGCACCTAGTGGTCATG

2 CATGACCACTAGGAGCATCTTTGGCGAACGGCAGGTGTTGCGGTGGTCTTTGAATACCTAGGGGAATAAATCTTTGGGCACCTAGTGGTCATG

2 CATGACCACTAGGAGCATCTTTGGCGACGGGTTGTGGTCAGCTCTGAACCGCTTAAACTAGGGGAATAAATCTTTGGGCACCTAGTGGTCATG

2 CATGACCACTAGGAGCATCTTTGGCGAGTATTGGCTGGTAGGTTGTGTATTGGGGAGCTAGGGGAATAAATCTTTGGGCACCTAGTGGTCATG

2 CATGACCACTAGGAGCATCTTTGGCGACGAGGGGGCTGTGTGGAATGGCGTAGTCGACTAGGGGAATAAATCTTTGGGCACCTAGTGGTCATG

2 CATGACCACTAGGAGCATCTTTGGCGAGACGTAGGCTGAGTCCGAGGTTGACGAGCTCTAGGGGAATAAATCTTTGGGCACCTAGTGGTCATG

2 CATGACCACTAGGAGCATCTTTGGCGAGAACGCGAACGCGTTAAAGCAGACGTTTGGCTAGGGGAATAAATCTTTGGGCACCTAGTGGTCATG

2 CATGACCACTAGGAGCATCTTTGGCGATACTCGTCAGATGGTGAAGCAGACGTTTGGCTAGGGGAATAAATCTTTGGGCACCTAGTGGTCATG

2 CATGACCACTAGGAGCATCTTTGGCGACGCGCGAGGCCTAGATCGAAGCGACGTGGACTAGGGGAATAAATCTTTGGGCACCTAGTGGTCATG

2 CATGACCACTAGGAGCATCTTTGGCGATCCTCGTCAGATGTTGAAGCAGACGTTTGGCTAGGGGAATAAATCTTTGGGCACCTAGTGGTCATG

2 CATGACCACTAGGAGCATCTTTGGCGAGTTAGAACACACACGAAGAGGTCCGAGCCACTAGGGGAATAAATCTTTGGGCACCTAGTGGTCATG

2 CATGACCACTAGGAGCATCTTTGGCGATCCGGAGAGTGCCAGCGACCGTGGTGGAGCCTAGGGGAATAAATCTTTGGGCACCTAGTGGTCATG

2 CATGACCACTAGGAGCATCTTTGGCGACCCGGTGTGGCGGAGCCTAGCCGTGGTGGACTAGGGGAATAAATCTTTGGGCACCTAGTGGTCATG

2 CATGACCACTAGGAGCATCTTTGGCGACGGTGTGGGGAACTTGTTTCGGCGATGCTACTAGGGGAATAAATCTTTGGGCACCTAGTGGTCATG

2 CATGACCACTAGGAGCATCTTTGGCGACGGAGTGGCTGAGAAGCGTGTTAACGGCGACTAGGGGAATAAATCTTTGGGCACCTAGTGGTCATG

2 CATGACCACTAGGAGCATCTTTGGCGAGGGGTAGGTGTTGCGGTGGTCTGTGAATCCCTAGGGGAATAAATCTTTGGGCACCTAGTGGTCATG

2 CATGACCACTAGGAGCATCTTTGGCGACCCGTATCGGCTTGCGTGATACCGGGTGGACTAGGGGAATAAATCTTTGGGCACCTAGTGGTCATG

2 CATGACCACTAGGAGCATCTTTGGCGAGATCGGGAGAAACGGTGGCATAGGTGTCTCCTAGGGGAATAAATCTTTGGGCACCTAGTGGTCATG

2 CATGACCACTAGGAGCATCTTTGGCGACGGCGTGGGGCCATCTGTGCGGCGGGTCCCCTAGGGGAATAAATCTTTGGGCACCTAGTGGTCATG

2 CATGACCACTAGGAGCATCTTTGGCGAAGAGCCCTGTTGCGAGCAACCCGAGGCGAGCTAGGGGAATAAATCTTTGGGCACCTAGTGGTCATG

2 CATGACCACTAGGAGCATCTTTGGCGAATATTGGCTGGTAGGTTGCGTATTGGGGAGCTAGGGGAATAAATCTTTGGGCACCTAGTGGTCATG

2 CATGACCACTAGGAGCATCTTTGGCGACCCGACTGAGCCGAGCGACGTTGTGGTGGACTAGGGGAATAAATCTTTGGGCACCTAGTGGTCATG

2 CATGACCACTAGGAGCATCTTTGGCGAGTCCACGAGCGAATGCGGCCCTGCACTGCACTAGGGGAATAAATCTTTGGGCACCTAGTGGTCATG

2 CATGACCACTAGGAGCATCTTTGGCGATGAGCTCCGGGGGTTCAGAAGGCGAAGAGACTAGGGGAATAAATCTTTGGGCACCTAGTGGTCATG

2 CATGACCACTAGGAGCATCTTTGGCGATCTACGGGGTCATGGGACAACTAGAATCGACTAGGGGAATAAATCTTTGGGCACCTAGTGGTCATG

2 CATGACCACTAGGAGCATCTTTGGCGAAGCTTGTTGCAAGGTCCGCGAGTGTGGACCCTAGGGGAATAAATCTTTGGGCACCTAGTGGTCATG

2 CATGACCACTAGGAGCATCTTTGGCGAATGGGCCTTTGGCACTCCTAGTCCTTGGTACTAGGGGAATAAATCTTTGGGCACCTAGTGGTCATG

2 CATGACCACTAGGAGCATCTTTGGCGATGGGGGCCAAGCAGGGCTACACAAATTCGACTAGGGGAATAAATCTTTGGGCACCTAGTGGTCATG

2 CATGACCACTAGGAGCATCTTTGGCGAATCCACAGGCACGCGAAACAGGAGATGAGACTAGGGGAATAAATCTTTGGGCACCTAGTGGTCATG

2 CATGACCACTAGGAGCATCTTTGGCGAGGGAGTTACGGGAAGCGATACGACACGAGACTAGGGGAATAAATCTTTGGGCACCTAGTGGTCATG

2 CATGACCACTAGGAGCATCTTTGGCGAAGCGTGTTGCAAGGTCCGCGAGTGTGGGCCCTAGGGGAATAAATCTTTGGGCACCTAGTGGTCATG

2 CATGACCACTAGGAGCATCTTTGGCGACGGCGCGGGGCCATCTGTGCGGCGGATCCCCTAGGGGAATAAATCTTTGGGCACCTAGTGGTCATG

2 CATGACCACTAGGAGCATCTTTGGCGAGGTCCTGTGGAGGTGAACCAGTCGTAAGCTCTAGGGGAATAAATCTTTGGGCACCTAGTGGTCATG

2 CATGACCACTAGGAGCATCTTTGGCGAGCGCCAGTGACGGGGAAGGCTGGGTCGAGACTAGGGGAATAAATCTTTGGGCACCTAGTGGTCATG

2 CATGACCACTAGGAGCATCTTTGGCGACCCTAATGCGACCACTGGTCGTGGGGTGGACTAGGGGAATAAATCTTTGGGCACCTAGTGGTCATG

2 CATGACCACTAGGAGCATCTTTGGCGACGTTGTGGGCTGGCAGGGAGTACTTGTTGCCTAGGGGAATAAATCTTTGGGCACCTAGTGGTCATG

2 CATGACCACTAGGAGCATCTTTGGCGACTTAAGGGGTCATCGGCGAGTTGGAGTCGACTAGGGGAATAAATCTTTGGGCACCTAGTGGTCATG

2 CATGACCACTAGGAGCATCTTTGGCGAGATCGGGAGAATCGGCGGCATTGGTTTCTCCTAGGGGAATAAATCTTTGGGCACCTAGTGGTCATG

2 CATGACCACTAGGAGCATCTTTGGCGACTCATTTTGAACGTTGCAAAATGTAGTGGACTAGGGGAATAAATCTTTGGGCACCTAGTGGTCATG

2 CATGACCACTAGGAGCATCTTTGGCGATGGGGCCGTGCTATCTGCACACTCCGAGGGCTAGGGGAATAAATCTTTGGGCACCTAGTGGTCATG

2 CATGACCACTAGGAGCATCTTTGGCGAGGTGAGTCCAGCGGCCTTCTTGGCCTGCGACTAGGGGAATAAATCTTTGGGCACCTAGTGGTCATG

2 CATGACCACTAGGAGCATCTTTGGCGACAAAGCTGTGGCAACTTATCGAGATGTCGACTAGGGGAATAAATCTTTGGGCACCTAGTGGTCATG

2 CATGACCACTAGGAGCATCTTTGGCGACAGTGGTCGGGTCTTAGGCATAAGACGTCGCTAGGGGAATAAATCTTTGGGCACCTAGTGGTCATG

2 CATGACCACTAGGAGCATCTTTGGCGATCACCGAATGCACTGCAGGCGGCGTGGAAGCTAGGGGAATAAATCTTTGGGCACCTAGTGGTCATG

2 CATGACCACTAGGAGCATCTTTGGCGACGAGCTGGGCATGAAGAAACCGGAGGCCGCCTAGGGGAATAAATCTTTGGGCACCTAGTGGTCATG

2 CATGACCACTAGGAGCATCTTTGGCGATGGAGGAACGGTAGGTCACTGGCGCCTCGCCTAGGGGAATAAATCTTTGGGCACCTAGTGGTCATG

2 CATGACCACTAGGAGCATCTTTGGCGACGTAGGGTCGGGCTACTAAGACGATCGCCTCTAGGGGAATAAATCTTTGGGCACCTAGTGGTCATG

2 CATGACCACTAGGAGCATCTTTGGCGACTGTGGCGTTTTGGGTATGGTCTAGGAGCTCTAGGGGAATAAATCTTTGGGCACCTAGTGGTCATG

2 CATGACCACTAGGAGCATCTTTGGCGAGAAGACACTGGATTCGGGGACCAGTTGCTCCTAGGGGAATAAATCTTTGGGCACCTAGTGGTCATG

2 CATGACCACTAGGAGCATCTTTGGCGACCCGGATGCAGACTTGTCATCGAGGGTGGACTAGGGGAATAAATCTTTGGGCACCTAGTGGTCATG

2 CATGACCACTAGGAGCATCTTTGGCGAGAGCTTGAAAAAGGTGCGTTGTTGTCGCAACTAGGGGAATAAATCTTTGGGCACCTAGTGGTCATG

2 CATGACCACTAGGAGCATCTTTGGCGAATGGCCGGCACGGCCTTCTAGTCCTGGGTACTAGGGGAATAAATCTTTGGGCACCTAGTGGTCATG

2 CATGACCACTAGGAGCATCTTTGGCGACAAGTGGGCTAAGGAGCCCCTATAGTGGAGCTAGGGGAATAAATCTTTGGGCACCTAGTGGTCATG

2 CATGACCACTAGGAGCATCTTTGGCGAATGGCCGGCACGGCCTTCTAGACCTTGGTACTAGGGGAATAAATCTTTGGGCACCTAGTGGTCATG

2 CATGACCACTAGGAGCATCTTTGGCGACGTTGGCTGGTAGGCGATCGTGTTAAGTGCCTAGGGGAATAAATCTTTGGGCACCTAGTGGTCATG

2 CATGACCACTAGGAGCATCTTTGGCGACGTGCAGATGAGAAAGTGTCCTATAGGCGCCTAGGGGAATAAATCTTTGGGCACCTAGTGGTCATG

2 CATGACCACTAGGAGCATCTTTGGCGAGCAAGCGCGGTCCCTCGGACCTTACAGTTGCTAGGGGAATAAATCTTTGGGCACCTAGTGGTCATG

2 CATGACCACTAGGAGCATCTTTGGCGACGATGGTTGGGAGGGACGTATTTCGGGCGACTAGGGGAATAAATCTTTGGGCACCTAGTGGTCATG

2 CATGACCACTAGGAGCATCTTTGGCGAAGACTAAGGAGCGAGTGCGTGGTCGACCGCCTAGGGGAATAAATCTTTGGGCACCTAGTGGTCATG

2 CATGACCACTAGGAGCATCTTTGGCGACAGGGTTTGGAAGCGTAGCCTATGTGTCGACTAGGGGAATAAATCTTTGGGCACCTAGTGGTCATG

2 CATGACCACTAGGAGCATCTTTGGCGAACCACACGAGGACTCGAGTAGTGTGGTGGACTAGGGGAATAAATCTTTGGGCACCTAGTGGTCATG

2 CATGACCACTAGGAGCATCTTTGGCGACCCGAGGGGAGTAGCCGAGGTGTGGAAAGCCTAGGGGAATAAATCTTTGGGCACCTAGTGGTCATG

2 CATGACCACTAGGAGCATCTTTGGCGACGATGTGGAAAGACTCGGGATGAGAGGAGACTAGGGGAATAAATCTTTGGGCACCTAGTGGTCATG

2 CATGACCACTAGGAGCATCTTTGGCGAGATCGGGAGAGTCGGTGGCATTGGCGTCTCCTAGGGGAATAAATCTTTGGGCACCTAGTGGTCATG

2 CATGACCACTAGGAGCATCTTTGGCGAACGGCAGGTGTTGCGGAGGTCTGTGAATCACTAGGGGAATAAATCTTTGGGCACCTAGTGGTCATG

2 CATGACCACTAGGAGCATCTTTGGCGATACAGGTGATCGGTGCTCGGTTGCCTGGCCCTAGGGGAATAAATCTTTGGGCACCTAGTGGTCATG

2 CATGACCACTAGGAGCATCTTTGGCGAGTCGTCGAAGAGCGCGTCGTGGGTTCGAGCCTAGGGGAATAAATCTTTGGGCACCTAGTGGTCATG

2 CATGACCACTAGGAGCATCTTTGGCGAACCGGAACGGGGTGGATAAGGAGCGTGAGACTAGGGGAATAAATCTTTGGGCACCTAGTGGTCATG

2 CATGACCACTAGGAGCATCTTTGGCGAACGGCAGGGGTTGCGGTGGTCTGTGAATCACTAGGGGAATAAATCTTTGGGCACCTAGTGGTCATG

2 CATGACCACTAGGAGCATCTTTGGCGACTAGTACGGGCAGGGGTCTCAATAAGTCGACTAGGGGAATAAATCTTTGGGCACCTAGTGGTCATG

2 CATGACCACTAGGAGCATCTTTGGCGACCCGTTACGATCAGAACGTGACAGGGTGGACTAGGGGAATAAATCTTTGGGCACCTAGTGGTCATG

2 CATGACCACTAGGAGCATCTTTGGCGATCACCGGCAGAGTGGAAACCAGGGTGTCGACTAGGGGAATAAATCTTTGGGCACCTAGTGGTCATG

2 CATGACCACTAGGAGCATCTTTGGCGATCCGTGCTGGAAAGCCCCGTGGAGGGTTGTCTAGGGGAATAAATCTTTGGGCACCTAGTGGTCATG

2 CATGACCACTAGGAGCATCTTTGGCGAGATCTGGAGAATCGGTGGCATTGGTGCCTCCTAGGGGAATAAATCTTTGGGCACCTAGTGGTCATG

2 CATGACCACTAGGAGCATCTTTGGCGACCCTATGCTCAGAGCTCAGCAGAGGGTGGACTAGGGGAATAAATCTTTGGGCACCTAGTGGTCATG

2 CATGACCACTAGGAGCATCTTTGGCGAGATCGGGAGAATCGGTGGCGTTGGTGTCTACTAGGGGAATAAATCTTTGGGCACCTAGTGGTCATG

2 CATGACCACTAGGAGCATCTTTGGCGAAGCAGTGCGACGGGCTATGGGCTCCTCGAGCTAGGGGAATAAATCTTTGGGCACCTAGTGGTCATG

2 CATGACCACTAGGAGCATCTTTGGCGACTTATAAAGCCGTGATTGGAGTCCGGTCCGCTAGGGGAATAAATCTTTGGGCACCTAGTGGTCATG

2 CATGACCACTAGGAGCATCTTTGGCGAATTGGTAACGCTTAACGCGTGGATGAGTCGCTAGGGGAATAAATCTTTGGGCACCTAGTGGTCATG

2 CATGACCACTAGGAGCATCTTTGGCGACCGGCGAAGTGGATCTTTGGCGCGGAGAGACTAGGGGAATAAATCTTTGGGCACCTAGTGGTCATG

2 CATGACCACTAGGAGCATCTTTGGCGACCGTCAAATGATCATATTGTGGACGAGCCTCTAGGGGAATAAATCTTTGGGCACCTAGTGGTCATG

2 CATGACCACTAGGAGCATCTTTGGCGACCCGTCCCAGGGACTGGATACACGGGTGGACTAGGGGAATAAATCTTTGGGCACCTAGTGGTCATG

2 CATGACCACTAGGAGCATCTTTGGCGAGGAGTGGGCGCATGTAATATAAACCCGAGACTAGGGGAATAAATCTTTGGGCACCTAGTGGTCATG

2 CATGACCACTAGGAGCATCTTTGGCGACGTCGTGGGGCTATTGATGCGGCGGCACTCCTAGGGGAATAAATCTTTGGGCACCTAGTGGTCATG

2 CATGACCACTAGGAGCATCTTTGGCGAGATCGGGAGAATCGGTGGAAGTGGTGTCTCCTAGGGGAATAAATCTTTGGGCACCTAGTGGTCATG

2 CATGACCACTAGGAGCATCTTTGGCGAGTAGTGGCTGAGAGCACCAAGTGTCCGAGACTAGGGGAATAAATCTTTGGGCACCTAGTGGTCATG

2 CATGACCACTAGGAGCATCTTTGGCGACAGTCCCAAGTGCTGTAGTGTGGATGGAGACTAGGGGAATAAATCTTTGGGCACCTAGTGGTCATG

2 CATGACCACTAGGAGCATCTTTGGCGAGATCTGGAGAATAGGTGGCATTGGTGTCTCCTAGGGGAATAAATCTTTGGGCACCTAGTGGTCATG

2 CATGACCACTAGGAGCATCTTTGGCGAAGTGCGGCGTGCCGTTGTCCGTGCGACAACCTAGGGGAATAAATCTTTGGGCACCTAGTGGTCATG

2 CATGACCACTAGGAGCATCTTTGGCGACACAGTCTGTTTAAGAGTGCACGTGGAGAACTAGGGGAATAAATCTTTGGGCACCTAGTGGTCATG

2 CATGACCACTAGGAGCATCTTTGGCGACGAGGGGGCAAGAGGGGACAGGCGAGGCGACTAGGGGAATAAATCTTTGGGCACCTAGTGGTCATG

2 CATGACCACTAGGAGCATCTTTGGCGACCGAAGGAAAACAAAAGTGGCCTTGCTAGCCTAGGGGAATAAATCTTTGGGCACCTAGTGGTCATG

2 CATGACCACTAGGAGCATCTTTGGCGATGTAATACGCCATAGCAGTGGCTGAGTCGCCTAGGGGAATAAATCTTTGGGCACCTAGTGGTCATG

2 CATGACCACTAGGAGCATCTTTGGCGAGTATTAGCTGGTAGGTTGCGTATTGGGGAGCTAGGGGAATAAATCTTTGGGCACCTAGTGGTCATG

2 CATGACCACTAGGAGCATCTTTGGCGAACTGGGCCGCAGGGGCAAGCCTGAGGTCGACTAGGGGAATAAATCTTTGGGCACCTAGTGGTCATG

2 CATGACCACTAGGAGCATCTTTGGCGAACAGGATGTGGGCAAGAGTGATACTGGAGACTAGGGGAATAAATCTTTGGGCACCTAGTGGTCATG

2 CATGACCACTAGGAGCATCTTTGGCGAGGCTCAAGCACGTCCGGGTCTGGCCGAGAGCTAGGGGAATAAATCTTTGGGCACCTAGTGGTCATG

2 CATGACCACTAGGAGCATCTTTGGCGACAAGTGGACTTAGTCGTGAAAATGTCGACTCTAGGGGAATAAATCTTTGGGCACCTAGTGGTCATG

2 CATGACCACTAGGAGCATCTTTGGCGACCCTCGCCAATATGGCGAAGGAGTGTTGCTCTAGGGGAATAAATCTTTGGGCACCTAGTGGTCATG

2 CATGACCACTAGGAGCATCTTTGGCGACACAATGGTAAACATCGGGTAAGATGTCGACTAGGGGAATAAATCTTTGGGCACCTAGTGGTCATG

2 CATGACCACTAGGAGCATCTTTGGCGATGTGAAACGGTTCGATGATGCGTGGGTCGCCTAGGGGAATAAATCTTTGGGCACCTAGTGGTCATG

2 CATGACCACTAGGAGCATCTTTGGCGAACGGCAGGAGTTGCGGTGGTCTGTGAAACCCTAGGGGAATAAATCTTTGGGCACCTAGTGGTCATG

2 CATGACCACTAGGAGCATCTTTGGCGAACGGCAGGTGTCGCGGTGGTCTGTGAACCCCTAGGGGAATAAATCTTTGGGCACCTAGTGGTCATG

2 CATGACCACTAGGAGCATCTTTGGCGAAAGTCGGTAGAACAGGGTGGGGGGCTGTCCCTAGGGGAATAAATCTTTGGGCACCTAGTGGTCATG

2 CATGACCACTAGGAGCATCTTTGGCGAGATCGGTAGAACCGGTGGCATTGGTGTCTCCTAGGGGAATAAATCTTTGGGCACCTAGTGGTCATG

2 CATGACCACTAGGAGCATCTTTGGCGATCCACCGTCTGTAGCATCGTGGAGTGGCGCCTAGGGGAATAAATCTTTGGGCACCTAGTGGTCATG

2 CATGACCACTAGGAGCATCTTTGGCGACACAGCGACCGCACGGGCAAGTAAGTGCGACTAGGGGAATAAATCTTTGGGCACCTAGTGGTCATG

2 CATGACCACTAGGAGCATCTTTGGCGAGCAAGCGCGGTCCCTCGGACCTCATAGTTGCTAGGGGAATAAATCTTTGGGCACCTAGTGGTCATG

2 CATGACCACTAGGAGCATCTTTGGCGAGGGGTGGTCTGTCAGCGTGGTGCCCGAAGCCTAGGGGAATAAATCTTTGGGCACCTAGTGGTCATG

2 CATGACCACTAGGAGCATCTTTGGCGACGAGGGCAGTCAAGGTTGGTCACTAGGAGACTAGGGGAATAAATCTTTGGGCACCTAGTGGTCATG

2 CATGACCACTAGGAGCATCTTTGGCGACGGAAGTAAGATTGATGGGACTTCCGAAGACTAGGGGAATAAATCTTTGGGCACCTAGTGGTCATG

2 CATGACCACTAGGAGCATCTTTGGCGAACGGCAGGTGCGGCGGTGGTCTGTGAATCCCTAGGGGAATAAATCTTTGGGCACCTAGTGGTCATG

2 CATGACCACTAGGAGCATCTTTGGCGATGAGGACCAGTCCGATAAGTGGCCGAGCGACTAGGGGAATAAATCTTTGGGCACCTAGTGGTCATG

2 CATGACCACTAGGAGCATCTTTGGCGAGAAGACTCTGGATTCGGGGACCAGCTGCTCCTAGGGGAATAAATCTTTGGGCACCTAGTGGTCATG

2 CATGACCACTAGGAGCATCTTTGGCGAAAGAGGTGTCTATGTTCGGGGGCGAATCGACTAGGGGAATAAATCTTTGGGCACCTAGTGGTCATG

2 CATGACCACTAGGAGCATCTTTGGCGAGCCAAGGTAAAAAGATATCAACATAGCTCGCTAGGGGAATAAATCTTTGGGCACCTAGTGGTCATG

2 CATGACCACTAGGAGCATCTTTGGCGAGGGTCAGTGGGCATCGCGGGTACTCCGAGACTAGGGGAATAAATCTTTGGGCACCTAGTGGTCATG

2 CATGACCACTAGGAGCATCTTTGGCGACCCGAAGCGTGCGACCGGATCGTGGATCGACTAGGGGAATAAATCTTTGGGCACCTAGTGGTCATG

2 CATGACCACTAGGAGCATCTTTGGCGAAGGGCGTAGGACTTCAAGTGGATCTCATAGCTAGGGGAATAAATCTTTGGGCACCTAGTGGTCATG

2 CATGACCACTAGGAGCATCTTTGGCGAGTCCATGGAGCTACCTCTGGTGGCTGGAACCTAGGGGAATAAATCTTTGGGCACCTAGTGGTCATG

2 CATGACCACTAGGAGCATCTTTGGCGAACAGGCTGTGGCTGCGGGTGGGTGCGCACCCTAGGGGAATAAATCTTTGGGCACCTAGTGGTCATG

2 CATGACCACTAGGAGCATCTTTGGCGACAGCGGCGTAGGCTGCCTTGGGTGCTGTCCCTAGGGGAATAAATCTTTGGGCACCTAGTGGTCATG

2 CATGACCACTAGGAGCATCTTTGGCGATGTATCGACTAGATTCGAGCAGACGTTTGGCTAGGGGAATAAATCTTTGGGCACCTAGTGGTCATG

2 CATGACCACTAGGAGCATCTTTGGCGACATGGGCGATGTCTGGCGAGGAAACGTCACCTAGGGGAATAAATCTTTGGGCACCTAGTGGTCATG

2 CATGACCACTAGGAGCATCTTTGGCGAGATCGGGAGGATCGGCGGCATTGGTGTCTCCTAGGGGAATAAATCTTTGGGCACCTAGTGGTCATG

2 CATGACCACTAGGAGCATCTTTGGCGACGGGTGTATGGGCAACGGTGAGCGAGTCGCCTAGGGGAATAAATCTTTGGGCACCTAGTGGTCATG

2 CATGACCACTAGGAGCATCTTTGGCGACATGCGTAGTCCATCTACGTCGAAGTGGAGCTAGGGGAATAAATCTTTGGGCACCTAGTGGTCATG

2 CATGACCACTAGGAGCATCTTTGGCGATGGGGCCGTGCTATCTGTACACTCCGCGGGCTAGGGGAATAAATCTTTGGGCACCTAGTGGTCATG

2 CATGACCACTAGGAGCATCTTTGGCGAGGAGGGGGCATGGCCTGGCGAGGTCGAAGCCTAGGGGAATAAATCTTTGGGCACCTAGTGGTCATG

2 CATGACCACTAGGAGCATCTTTGGCGAAGGGTGTAGGACTTCAAGTGGATCACATAGCTAGGGGAATAAATCTTTGGGCACCTAGTGGTCATG

2 CATGACCACTAGGAGCATCTTTGGCGAGGAGTGGCTGAGGGCGGTGGCTAGGTCGCGCTAGGGGAATAAATCTTTGGGCACCTAGTGGTCATG

2 CATGACCACTAGGAGCATCTTTGGCGACGGAGTGGGGCCCATTGTGCGGCGGTAATCCTAGGGGAATAAATCTTTGGGCACCTAGTGGTCATG

2 CATGACCACTAGGAGCATCTTTGGCGACCCTACTGGACTTGTCAATGTTAGGGTGGACTAGGGGAATAAATCTTTGGGCACCTAGTGGTCATG

2 CATGACCACTAGGAGCATCTTTGGCGACGCGGGGGCAACAAGCTAGCAGACGTTTGGCTAGGGGAATAAATCTTTGGGCACCTAGTGGTCATG

2 CATGACCACTAGGAGCATCTTTGGCGACTGTGGGAACTCGCGACAAATAACAGGAGACTAGGGGAATAAATCTTTGGGCACCTAGTGGTCATG

2 CATGACCACTAGGAGCATCTTTGGCGATCCGGGTCAAGTGAGCCGCGCGTGGTGCGCCTAGGGGAATAAATCTTTGGGCACCTAGTGGTCATG

2 CATGACCACTAGGAGCATCTTTGGCGACGTCACGCACACGAAGAGACGAAGTGGAGCCTAGGGGAATAAATCTTTGGGCACCTAGTGGTCATG

2 CATGACCACTAGGAGCATCTTTGGCGACGTGTTGGTCTGGGTTGCGTCGGGACTCGCCTAGGGGAATAAATCTTTGGGCACCTAGTGGTCATG

2 CATGACCACTAGGAGCATCTTTGGCGACGGCACAGGGTTGGGATAGGAGTGGTATCGCTAGGGGAATAAATCTTTGGGCACCTAGTGGTCATG

2 CATGACCACTAGGAGCATCTTTGGCGAGTCGTCTGCGAAGGAACGGATACAACGAGACTAGGGGAATAAATCTTTGGGCACCTAGTGGTCATG

2 CATGACCACTAGGAGCATCTTTGGCGAGAACGGGGTTCGAACGGGGGTTTGCGAGAGCTAGGGGAATAAATCTTTGGGCACCTAGTGGTCATG

2 CATGACCACTAGGAGCATCTTTGGCGACCCGATATCGTCAGAGTACTATCGGGTGGACTAGGGGAATAAATCTTTGGGCACCTAGTGGTCATG

2 CATGACCACTAGGAGCATCTTTGGCGATCCATAGGATTATGGTTCGTAGGGATTCGACTAGGGGAATAAATCTTTGGGCACCTAGTGGTCATG

2 CATGACCACTAGGAGCATCTTTGGCGAGATCGGTAGAATCGGCGGCATTGGTGTCTCCTAGGGGAATAAATCTTTGGGCACCTAGTGGTCATG

2 CATGACCACTAGGAGCATCTTTGGCGAAGGCAAGCATGCGGAACGTGGGCAAGCCAGCTAGGGGAATAAATCTTTGGGCACCTAGTGGTCATG

2 CATGACCACTAGGAGCATCTTTGGCGAGCAAGGGTTGGGATGCGATCGGCGCAGCCACTAGGGGAATAAATCTTTGGGCACCTAGTGGTCATG

2 CATGACCACTAGGAGCATCTTTGGCGATGTTAGCTCGCCAGTGGTTTCGTTCTGGCCCTAGGGGAATAAATCTTTGGGCACCTAGTGGTCATG

2 CATGACCACTAGGAGCATCTTTGGCGATTAAGGCACGCGCGCTCGCAAAGGGCCCGCCTAGGGGAATAAATCTTTGGGCACCTAGTGGTCATG

2 CATGACCACTAGGAGCATCTTTGGCGACATCGTCGGTCAATAGTGGCTGAGTCGCCTCTAGGGGAATAAATCTTTGGGCACCTAGTGGTCATG

2 CATGACCACTAGGAGCATCTTTGGCGACTCTAACGGACACGGAGAGCGAAGTGGAGCCTAGGGGAATAAATCTTTGGGCACCTAGTGGTCATG

2 CATGACCACTAGGAGCATCTTTGGCGAACGGCAGGTGTTGCGGTGGTCTGAGAAACCCTAGGGGAATAAATCTTTGGGCACCTAGTGGTCATG

2 CATGACCACTAGGAGCATCTTTGGCGAGCCAGAATGTGCTGGCCTTTAGAACCGAGCCTAGGGGAATAAATCTTTGGGCACCTAGTGGTCATG

2 CATGACCACTAGGAGCATCTTTGGCGACGGTGTTGGGAACTTGTTTCGGCGGTGCTACTAGGGGAATAAATCTTTGGGCACCTAGTGGTCATG

2 CATGACCACTAGGAGCATCTTTGGCGAACGTGCAAGATATGCCGCCTGGAGAGTAGGCTAGGGGAATAAATCTTTGGGCACCTAGTGGTCATG

2 CATGACCACTAGGAGCATCTTTGGCGACTCATGGGTTGGCAGGGGGTATTTGGGCGACTAGGGGAATAAATCTTTGGGCACCTAGTGGTCATG

2 CATGACCACTAGGAGCATCTTTGGCGAAGATGGACCCCGCGTGCGGGTCCCGTCTGCCTAGGGGAATAAATCTTTGGGCACCTAGTGGTCATG

2 CATGACCACTAGGAGCATCTTTGGCGACCCAGCGGGCGGTGCCCGTGAGTGTGGAGACTAGGGGAATAAATCTTTGGGCACCTAGTGGTCATG

2 CATGACCACTAGGAGCATCTTTGGCGATGGGTGCTAACGGCCGCCGCTGCGGCTACACTAGGGGAATAAATCTTTGGGCACCTAGTGGTCATG

2 CATGACCACTAGGAGCATCTTTGGCGAGTACCCGGGGGAATCGTCGTGCTGTCGAGCCTAGGGGAATAAATCTTTGGGCACCTAGTGGTCATG

2 CATGACCACTAGGAGCATCTTTGGCGACAAGGTGGGAGTGTACTGTTGCTGTCGCCGCTAGGGGAATAAATCTTTGGGCACCTAGTGGTCATG

2 CATGACCACTAGGAGCATCTTTGGCGACCTAAGACGGGGATGCGACGTAATGGTCGACTAGGGGAATAAATCTTTGGGCACCTAGTGGTCATG

2 CATGACCACTAGGAGCATCTTTGGCGACTAGGGTAACAGGAAGCTACTGAACGTCGACTAGGGGAATAAATCTTTGGGCACCTAGTGGTCATG

2 CATGACCACTAGGAGCATCTTTGGCGAACGGCAGGGGTTGCGGTGGTCTGTGAACCCCTAGGGGAATAAATCTTTGGGCACCTAGTGGTCATG

2 CATGACCACTAGGAGCATCTTTGGCGAGGTTTGTAGGCCTGGCTCGGCGGCCGACGTCTAGGGGAATAAATCTTTGGGCACCTAGTGGTCATG

2 CATGACCACTAGGAGCATCTTTGGCGAGCAAATCGCACAAGAGGACACCGCGAGCCGCTAGGGGAATAAATCTTTGGGCACCTAGTGGTCATG

2 CATGACCACTAGGAGCATCTTTGGCGATGGGGAGGTAGTGGGGACGGTATAGATCGCCTAGGGGAATAAATCTTTGGGCACCTAGTGGTCATG

2 CATGACCACTAGGAGCATCTTTGGCGAGGAGGTACTCCTGCGATGTGGTCGAGCTTACTAGGGGAATAAATCTTTGGGCACCTAGTGGTCATG

2 CATGACCACTAGGAGCATCTTTGGCGACACGACAGGCCTTAAGGACGTCGCTGTGGACTAGGGGAATAAATCTTTGGGCACCTAGTGGTCATG

2 CATGACCACTAGGAGCATCTTTGGCGACACCCTATGTTGAGAAACTAGGGTTGTGGACTAGGGGAATAAATCTTTGGGCACCTAGTGGTCATG

2 CATGACCACTAGGAGCATCTTTGGCGACGCCGTAGAGTGGCTGAGAGCTAGAGTCGACTAGGGGAATAAATCTTTGGGCACCTAGTGGTCATG

2 CATGACCACTAGGAGCATCTTTGGCGAAGTGTGGAACATGCTGGTTTTGGTTGCACCCTAGGGGAATAAATCTTTGGGCACCTAGTGGTCATG

2 CATGACCACTAGGAGCATCTTTGGCGACTAAGGTGTCATACGCGTTCAGTAGGGAAGCTAGGGGAATAAATCTTTGGGCACCTAGTGGTCATG

2 CATGACCACTAGGAGCATCTTTGGCGAGAAGGTAACTGTAGCCTCTGTGGTCGAGCTCTAGGGGAATAAATCTTTGGGCACCTAGTGGTCATG

2 CATGACCACTAGGAGCATCTTTGGCGATTCGACGTGGCGGGTAACGAGGAGAAGCGACTAGGGGAATAAATCTTTGGGCACCTAGTGGTCATG

2 CATGACCACTAGGAGCATCTTTGGCGACTTGGAGAGTCTGATCGTATGACTGTCGCACTAGGGGAATAAATCTTTGGGCACCTAGTGGTCATG

2 CATGACCACTAGGAGCATCTTTGGCGACCCGTAGCGTCTGGTGCGCTGCGAGGTGGACTAGGGGAATAAATCTTTGGGCACCTAGTGGTCATG

2 CATGACCACTAGGAGCATCTTTGGCGATGGTCGGTAGAATGGTTCGGTATTTCGTCTCTAGGGGAATAAATCTTTGGGCACCTAGTGGTCATG

2 CATGACCACTAGGAGCATCTTTGGCGATCCGAGGCACAGACAGTCGGCACAGAGCGCCTAGGGGAATAAATCTTTGGGCACCTAGTGGTCATG

2 CATGACCACTAGGAGCATCTTTGGCGACGTGGTCGGGTAAGAGGTAGTAGGTGTCGCCTAGGGGAATAAATCTTTGGGCACCTAGTGGTCATG

2 CATGACCACTAGGAGCATCTTTGGCGACCCGTGGCTGAAGCTAATCGGACGGGTGGACTAGGGGAATAAATCTTTGGGCACCTAGTGGTCATG

2 CATGACCACTAGGAGCATCTTTGGCGAACCTGAGGATACAATCCGGACTGAGGCTCGCTAGGGGAATAAATCTTTGGGCACCTAGTGGTCATG

2 CATGACCACTAGGAGCATCTTTGGCGATGGGTGCGAAGGTCGTTGAGCAGTACTTGCCTAGGGGAATAAATCTTTGGGCACCTAGTGGTCATG

2 CATGACCACTAGGAGCATCTTTGGCGACACCGTCAGTACGATGATGGTCAAGTGGAGCTAGGGGAATAAATCTTTGGGCACCTAGTGGTCATG

2 CATGACCACTAGGAGCATCTTTGGCGAGATCGGGAGAATCGGTGGCATTGGTTACTCCTAGGGGAATAAATCTTTGGGCACCTAGTGGTCATG

2 CATGACCACTAGGAGCATCTTTGGCGAGGCTGGAGGGGACGCCTCCGAACGGACCGCCTAGGGGAATAAATCTTTGGGCACCTAGTGGTCATG

2 CATGACCACTAGGAGCATCTTTGGCGAGGAATGGAAGCTTGCGCTAAGGGACATCGACTAGGGGAATAAATCTTTGGGCACCTAGTGGTCATG

2 CATGACCACTAGGAGCATCTTTGGCGACCCTGATAAGTATAGCGTATCAGCGGTGGACTAGGGGAATAAATCTTTGGGCACCTAGTGGTCATG

2 CATGACCACTAGGAGCATCTTTGGCGAAGACGACACAGTCGATCGGGATAGTGGAAGCTAGGGGAATAAATCTTTGGGCACCTAGTGGTCATG

2 CATGACCACTAGGAGCATCTTTGGCGACCCTGCCAGTGAGGCGGCAGGAGTGTTGCTCTAGGGGAATAAATCTTTGGGCACCTAGTGGTCATG

2 CATGACCACTAGGAGCATCTTTGGCGATCCGGGGGGCTGTGCAGCCGACGCAAAGGGCTAGGGGAATAAATCTTTGGGCACCTAGTGGTCATG

2 CATGACCACTAGGAGCATCTTTGGCGACTCAAGCCGTGCAGGATGCATGCGAGTTGGCTAGGGGAATAAATCTTTGGGCACCTAGTGGTCATG

2 CATGACCACTAGGAGCATCTTTGGCGACAGTGGCTGGCAGGGTAGTATCATTTCGCTCTAGGGGAATAAATCTTTGGGCACCTAGTGGTCATG

2 CATGACCACTAGGAGCATCTTTGGCGACACACAATTGTGTAACAGGGTGTCGTGGAGCTAGGGGAATAAATCTTTGGGCACCTAGTGGTCATG

2 CATGACCACTAGGAGCATCTTTGGCGAGAACGGGAGAATCGGTGGCATTGGGGTCTCCTAGGGGAATAAATCTTTGGGCACCTAGTGGTCATG

2 CATGACCACTAGGAGCATCTTTGGCGAGATCTCGAGAATCGGTGGCATTGGTGTCTCCTAGGGGAATAAATCTTTGGGCACCTAGTGGTCATG

2 CATGACCACTAGGAGCATCTTTGGCGATGTGGTTGGTCGTGGGGAGGGAAGGTGTAGCTAGGGGAATAAATCTTTGGGCACCTAGTGGTCATG

2 CATGACCACTAGGAGCATCTTTGGCGACGCCGAGGCAGCACACGGGCGGATCGACAGCTAGGGGAATAAATCTTTGGGCACCTAGTGGTCATG

2 CATGACCACTAGGAGCATCTTTGGCGACGACGTGGTGGAGTACGATAAACGTCGAGACTAGGGGAATAAATCTTTGGGCACCTAGTGGTCATG

2 CATGACCACTAGGAGCATCTTTGGCGACCCGGTAGGGCCTGTAGGCACACGGGTGGACTAGGGGAATAAATCTTTGGGCACCTAGTGGTCATG

2 CATGACCACTAGGAGCATCTTTGGCGAACGGCAGGTGTTGCGGTGGTCTGCGGATCCCTAGGGGAATAAATCTTTGGGCACCTAGTGGTCATG

2 CATGACCACTAGGAGCATCTTTGGCGAGGTCGGCTGGCAGGTTGTATTAACGAGATACTAGGGGAATAAATCTTTGGGCACCTAGTGGTCATG

2 CATGACCACTAGGAGCATCTTTGGCGAACCAGTGAGCGTAGCTCTCTGAGGTGGAGACTAGGGGAATAAATCTTTGGGCACCTAGTGGTCATG

2 CATGACCACTAGGAGCATCTTTGGCGAAGTCCCAACAGCGCACAGATAACGACAAGCCTAGGGGAATAAATCTTTGGGCACCTAGTGGTCATG

2 CATGACCACTAGGAGCATCTTTGGCGAGTGGATACGGAAGGCAAGTGTGCCACTAGCCTAGGGGAATAAATCTTTGGGCACCTAGTGGTCATG

2 CATGACCACTAGGAGCATCTTTGGCGAATGTGTCGAGCTCGCTGGGAATGATGTCGCCTAGGGGAATAAATCTTTGGGCACCTAGTGGTCATG

2 CATGACCACTAGGAGCATCTTTGGCGACCCTGGGCGCAAGCGTAAAGACAGGGTGGACTAGGGGAATAAATCTTTGGGCACCTAGTGGTCATG

2 CATGACCACTAGGAGCATCTTTGGCGACACGGTAGTCATGAAGTCATCAATCGTCGCCTAGGGGAATAAATCTTTGGGCACCTAGTGGTCATG

2 CATGACCACTAGGAGCATCTTTGGCGATGGGTGCTGACGGAGCTAGTGAGCCCTACACTAGGGGAATAAATCTTTGGGCACCTAGTGGTCATG

2 CATGACCACTAGGAGCATCTTTGGCGAAGGGTGTAGGACTTCAAGTGGATCTCGTAGCTAGGGGAATAAATCTTTGGGCACCTAGTGGTCATG

2 CATGACCACTAGGAGCATCTTTGGCGAAGCGAGATGTATATATCCTGAGGATTCGCCCTAGGGGAATAAATCTTTGGGCACCTAGTGGTCATG

2 CATGACCACTAGGAGCATCTTTGGCGACAAAAGAGGTCAAATATTTATACTAGTCGACTAGGGGAATAAATCTTTGGGCACCTAGTGGTCATG

2 CATGACCACTAGGAGCATCTTTGGCGAAGTGCGGCGTGCCGTGACTGAAAGGATCACCTAGGGGAATAAATCTTTGGGCACCTAGTGGTCATG

2 CATGACCACTAGGAGCATCTTTGGCGAGAAGGTATAGGGCGCCAGGATGGGTCGAGACTAGGGGAATAAATCTTTGGGCACCTAGTGGTCATG

2 CATGACCACTAGGAGCATCTTTGGCGACCGAATAGAGACAGGAAGGTTACAGGGAGACTAGGGGAATAAATCTTTGGGCACCTAGTGGTCATG

2 CATGACCACTAGGAGCATCTTTGGCGATTATATGTTATCGCGAGGTCGTTGCGAGCCCTAGGGGAATAAATCTTTGGGCACCTAGTGGTCATG

2 CATGACCACTAGGAGCATCTTTGGCGAGGGAGTACAAAGTGCGTAGTGGCCGAGCTACTAGGGGAATAAATCTTTGGGCACCTAGTGGTCATG

2 CATGACCACTAGGAGCATCTTTGGCGACGGCATTTGTTAGGCTCAGGACAGTGTCACCTAGGGGAATAAATCTTTGGGCACCTAGTGGTCATG

2 CATGACCACTAGGAGCATCTTTGGCGACGTGATCGGAGATTACAAGCAGACGTTGGGCTAGGGGAATAAATCTTTGGGCACCTAGTGGTCATG

2 CATGACCACTAGGAGCATCTTTGGCGACCCGGATAGGTACTATCCTGTGAGTGGCGACTAGGGGAATAAATCTTTGGGCACCTAGTGGTCATG

2 CATGACCACTAGGAGCATCTTTGGCGATCGACGGCGGTTCGCACGGAGCGGGGTTCCCTAGGGGAATAAATCTTTGGGCACCTAGTGGTCATG

2 CATGACCACTAGGAGCATCTTTGGCGACCCATGGCAGAGCACTCGCATATGGGTGGACTAGGGGAATAAATCTTTGGGCACCTAGTGGTCATG

2 CATGACCACTAGGAGCATCTTTGGCGACGGCGTGGGGCATCTCTTGCGGCGGTTATTCTAGGGGAATAAATCTTTGGGCACCTAGTGGTCATG

2 CATGACCACTAGGAGCATCTTTGGCGACGACGGGGGCACGGCTTAAATGGTAGTCGACTAGGGGAATAAATCTTTGGGCACCTAGTGGTCATG

2 CATGACCACTAGGAGCATCTTTGGCGAAAGGTGGGCCCGTATTGATCATGTCTGGCGCTAGGGGAATAAATCTTTGGGCACCTAGTGGTCATG

2 CATGACCACTAGGAGCATCTTTGGCGAGGGCGGTGGCTGCGAGGAGTGGTTGGGTGCCTAGGGGAATAAATCTTTGGGCACCTAGTGGTCATG

2 CATGACCACTAGGAGCATCTTTGGCGACGCAAGCGGAATGCCGGCATAGTTGCGAGACTAGGGGAATAAATCTTTGGGCACCTAGTGGTCATG

2 CATGACCACTAGGAGCATCTTTGGCGAGATCGGGAGAATCGGAGGCATTGGTGTCTACTAGGGGAATAAATCTTTGGGCACCTAGTGGTCATG

2 CATGACCACTAGGAGCATCTTTGGCGACGAGGTGACCAGCGGGACGGCCCGAGGAACCTAGGGGAATAAATCTTTGGGCACCTAGTGGTCATG

2 CATGACCACTAGGAGCATCTTTGGCGAACGACAGGTGTTGCGGTGGTTTGTGAATCCCTAGGGGAATAAATCTTTGGGCACCTAGTGGTCATG

2 CATGACCACTAGGAGCATCTTTGGCGACCCGCCTCGTGTTAACGAGATCGTGGTGGACTAGGGGAATAAATCTTTGGGCACCTAGTGGTCATG

2 CATGACCACTAGGAGCATCTTTGGCGAACGGCAGGTGTTGCTGGGGTCTGTGAATCCCTAGGGGAATAAATCTTTGGGCACCTAGTGGTCATG

2 CATGACCACTAGGAGCATCTTTGGCGATAGGACCGGCTGGCCTGCACGAGTAGTCGCCTAGGGGAATAAATCTTTGGGCACCTAGTGGTCATG

2 CATGACCACTAGGAGCATCTTTGGCGACATCCCATAATGGGACGAAATGGGTGTGGACTAGGGGAATAAATCTTTGGGCACCTAGTGGTCATG

2 CATGACCACTAGGAGCATCTTTGGCGACTAGGGTGTCGAGCAGTGCAAGTAGGTCGCCTAGGGGAATAAATCTTTGGGCACCTAGTGGTCATG

2 CATGACCACTAGGAGCATCTTTGGCGACTTGGGTGGTCAAAGCAGTTTGGTTGTCGCCTAGGGGAATAAATCTTTGGGCACCTAGTGGTCATG

2 CATGACCACTAGGAGCATCTTTGGCGACGCACCGAGGTCTGGGTACCAGCGGCAAGACTAGGGGAATAAATCTTTGGGCACCTAGTGGTCATG

2 CATGACCACTAGGAGCATCTTTGGCGACACGTCGTGTGGTGGTTCAAGGTGAGCTAGCTAGGGGAATAAATCTTTGGGCACCTAGTGGTCATG

2 CATGACCACTAGGAGCATCTTTGGCGAGCGGGCGTAACCCGAGCAAGTACTGATCGACTAGGGGAATAAATCTTTGGGCACCTAGTGGTCATG

2 CATGACCACTAGGAGCATCTTTGGCGAAAGTCGGGAGAACAGGGTGGGGTGCTGTCCCTAGGGGAATAAATCTTTGGGCACCTAGTGGTCATG

2 CATGACCACTAGGAGCATCTTTGGCGAGTCTAACAGGCCCAAATAATCGACGAGCTGCTAGGGGAATAAATCTTTGGGCACCTAGTGGTCATG

2 CATGACCACTAGGAGCATCTTTGGCGAGCTTTACGCGTCCTTGAGTGCCGACGTACGCTAGGGGAATAAATCTTTGGGCACCTAGTGGTCATG

2 CATGACCACTAGGAGCATCTTTGGCGAGGGTGGGCGCCGGCAGCGGTGTGAATGCGACTAGGGGAATAAATCTTTGGGCACCTAGTGGTCATG

2 CATGACCACTAGGAGCATCTTTGGCGACGTTGGGAGTGAAGTCCCAGCGAAGTGGACCTAGGGGAATAAATCTTTGGGCACCTAGTGGTCATG

2 CATGACCACTAGGAGCATCTTTGGCGAAACAGCATCCGGGGGGTAGGCTGTGAGAGACTAGGGGAATAAATCTTTGGGCACCTAGTGGTCATG

2 CATGACCACTAGGAGCATCTTTGGCGACCCGCAACGCGCAGCGACGCATAGGGTGGACTAGGGGAATAAATCTTTGGGCACCTAGTGGTCATG

2 CATGACCACTAGGAGCATCTTTGGCGAGTGTAACCGATCCAATAGCTACAGTCGAGACTAGGGGAATAAATCTTTGGGCACCTAGTGGTCATG

2 CATGACCACTAGGAGCATCTTTGGCGACCCGCATGAACTATGTTTAGAGCGGGTGGACTAGGGGAATAAATCTTTGGGCACCTAGTGGTCATG

2 CATGACCACTAGGAGCATCTTTGGCGACTCCCATGTTAGGAGGCTACGCGCGGGAAACTAGGGGAATAAATCTTTGGGCACCTAGTGGTCATG

2 CATGACCACTAGGAGCATCTTTGGCGAGTGTTGCCTGGTACACGAGCAGACGTTTGGCTAGGGGAATAAATCTTTGGGCACCTAGTGGTCATG

2 CATGACCACTAGGAGCATCTTTGGCGACACGATGGACGGAGGTTTATCGTAGTGGAGCTAGGGGAATAAATCTTTGGGCACCTAGTGGTCATG

2 CATGACCACTAGGAGCATCTTTGGCGACTAGGGCGTCACGTGAAGCTCGATACGTCGCTAGGGGAATAAATCTTTGGGCACCTAGTGGTCATG

2 CATGACCACTAGGAGCATCTTTGGCGATTGTAGGTCACTACGACTACACTGTATCGACTAGGGGAATAAATCTTTGGGCACCTAGTGGTCATG

2 CATGACCACTAGGAGCATCTTTGGCGACTCGAATAGTGGCAAAGGGATCTCAGTCGACTAGGGGAATAAATCTTTGGGCACCTAGTGGTCATG

2 CATGACCACTAGGAGCATCTTTGGCGATGAAGATGGTAGGGTAATGTGTCGGCGCCGCTAGGGGAATAAATCTTTGGGCACCTAGTGGTCATG

2 CATGACCACTAGGAGCATCTTTGGCGAGTCAGGACGATTAACTCGTTCGTAGTGGAGCTAGGGGAATAAATCTTTGGGCACCTAGTGGTCATG

2 CATGACCACTAGGAGCATCTTTGGCGACGGGGGGATATGTGCGCGCAGGGCTGTCGACTAGGGGAATAAATCTTTGGGCACCTAGTGGTCATG

2 CATGACCACTAGGAGCATCTTTGGCGACGGTGTGGGGCCTAAGTGCGGCGGCTGATTCTAGGGGAATAAATCTTTGGGCACCTAGTGGTCATG

2 CATGACCACTAGGAGCATCTTTGGCGAATGTAGGTTGACTGCAAGGTAGGTTAGTTCCTAGGGGAATAAATCTTTGGGCACCTAGTGGTCATG

2 CATGACCACTAGGAGCATCTTTGGCGACCACGTCATGGTGTTGGGAAAAAAGGGCGCCTAGGGGAATAAATCTTTGGGCACCTAGTGGTCATG

2 CATGACCACTAGGAGCATCTTTGGCGAATCGTATGTTACCTAAGAGGTGCCGTTCGACTAGGGGAATAAATCTTTGGGCACCTAGTGGTCATG

2 CATGACCACTAGGAGCATCTTTGGCGAGTCGCAACAGGGAACTGATCGATGTATCGCCTAGGGGAATAAATCTTTGGGCACCTAGTGGTCATG

2 CATGACCACTAGGAGCATCTTTGGCGACAGTGGGCGGGCATCAGTTACCAGTGTCGACTAGGGGAATAAATCTTTGGGCACCTAGTGGTCATG

2 CATGACCACTAGGAGCATCTTTGGCGAGCAGCTCCAAAATGGTTAGGTTGTTGTAAGCTAGGGGAATAAATCTTTGGGCACCTAGTGGTCATG

2 CATGACCACTAGGAGCATCTTTGGCGAGCGGTGATGGTCGGCACTAAGGGGTCGAGACTAGGGGAATAAATCTTTGGGCACCTAGTGGTCATG

2 CATGACCACTAGGAGCATCTTTGGCGAGATCGGGAGAATCTGTGGCAATGGTGTCTCCTAGGGGAATAAATCTTTGGGCACCTAGTGGTCATG

2 CATGACCACTAGGAGCATCTTTGGCGAACGTGCAAGATATGCCGCATGGAGCGTAGGCTAGGGGAATAAATCTTTGGGCACCTAGTGGTCATG

2 CATGACCACTAGGAGCATCTTTGGCGAGATCGGGAGTATCGGCGGCATTGGTGTCTCCTAGGGGAATAAATCTTTGGGCACCTAGTGGTCATG

2 CATGACCACTAGGAGCATCTTTGGCGACATGTACACTACCTTTGCCTGGAGCGTAGGCTAGGGGAATAAATCTTTGGGCACCTAGTGGTCATG

2 CATGACCACTAGGAGCATCTTTGGCGACAGGGGATGCCGATGCGCTGGAAGACCGAGCTAGGGGAATAAATCTTTGGGCACCTAGTGGTCATG

2 CATGACCACTAGGAGCATCTTTGGCGACGTCGAAGGCTAGTATAGGGGTATAGTCGACTAGGGGAATAAATCTTTGGGCACCTAGTGGTCATG

2 CATGACCACTAGGAGCATCTTTGGCGACCCAGAGCGCGCGAGTTTGCGTTGGGTGGACTAGGGGAATAAATCTTTGGGCACCTAGTGGTCATG

2 CATGACCACTAGGAGCATCTTTGGCGAGAAGACTCTCGATTCGGGGACCAGTTGCTGCTAGGGGAATAAATCTTTGGGCACCTAGTGGTCATG

2 CATGACCACTAGGAGCATCTTTGGCGATGGTAGCGTCGCTGACCGCCTGGACTGCTACTAGGGGAATAAATCTTTGGGCACCTAGTGGTCATG

2 CATGACCACTAGGAGCATCTTTGGCGACCATCCCTGGTACGGGCTTCTGTGGGTTTACTAGGGGAATAAATCTTTGGGCACCTAGTGGTCATG

2 CATGACCACTAGGAGCATCTTTGGCGATGGGGCCGTGCGATCTGCACACTCCGCGGGCTAGGGGAATAAATCTTTGGGCACCTAGTGGTCATG

2 CATGACCACTAGGAGCATCTTTGGCGACGAAGTGGGAAAGTATGGGCAGATAGTCGACTAGGGGAATAAATCTTTGGGCACCTAGTGGTCATG

2 CATGACCACTAGGAGCATCTTTGGCGACATGGGATGCCGATGCGCTGGACGACCGAGCTAGGGGAATAAATCTTTGGGCACCTAGTGGTCATG

2 CATGACCACTAGGAGCATCTTTGGCGACCCGCGGATTGCCTGAGCAATGCGGGTGGACTAGGGGAATAAATCTTTGGGCACCTAGTGGTCATG

2 CATGACCACTAGGAGCATCTTTGGCGAGACGATATGAAGCGACGAGCAGACGTTTGGCTAGGGGAATAAATCTTTGGGCACCTAGTGGTCATG

2 CATGACCACTAGGAGCATCTTTGGCGACCCACGGAGCGAGTATCGACGTGTGGTGGACTAGGGGAATAAATCTTTGGGCACCTAGTGGTCATG

2 CATGACCACTAGGAGCATCTTTGGCGACGGCATTTGTTAGGCTTCGGACAGTGTCACCTAGGGGAATAAATCTTTGGGCACCTAGTGGTCATG

2 CATGACCACTAGGAGCATCTTTGGCGATTAGCCTCGTGACGTGGCCGAGAGTATCGACTAGGGGAATAAATCTTTGGGCACCTAGTGGTCATG

2 CATGACCACTAGGAGCATCTTTGGCGATCTGGGGCAGCCAGGTCAGGAAAGGATCGACTAGGGGAATAAATCTTTGGGCACCTAGTGGTCATG

2 CATGACCACTAGGAGCATCTTTGGCGACATTCGACATAGATCGGCAGGGCGTCGCCGCTAGGGGAATAAATCTTTGGGCACCTAGTGGTCATG

2 CATGACCACTAGGAGCATCTTTGGCGAGGAGTGAGGGCCTGTACAAATACACTGAGACTAGGGGAATAAATCTTTGGGCACCTAGTGGTCATG

2 CATGACCACTAGGAGCATCTTTGGCGAGCTAGACCGAGATCTCGACTAGGGGCGAGACTAGGGGAATAAATCTTTGGGCACCTAGTGGTCATG

2 CATGACCACTAGGAGCATCTTTGGCGACGTTGGCTGGTAGAGACTGTATTGAGGAACCTAGGGGAATAAATCTTTGGGCACCTAGTGGTCATG

2 CATGACCACTAGGAGCATCTTTGGCGATACGGACGCAGGTGCTCCGTGGCAGAGAGACTAGGGGAATAAATCTTTGGGCACCTAGTGGTCATG

2 CATGACCACTAGGAGCATCTTTGGCGACGGTGTGGGGGCGTCAGCCCGGCGGTCATACTAGGGGAATAAATCTTTGGGCACCTAGTGGTCATG

2 CATGACCACTAGGAGCATCTTTGGCGACACAGTGTAATCCATGTAGCTGTCGTGGAGCTAGGGGAATAAATCTTTGGGCACCTAGTGGTCATG

2 CATGACCACTAGGAGCATCTTTGGCGACCCAGTGTGTCATCGACCCAGGAGTGGAGCCTAGGGGAATAAATCTTTGGGCACCTAGTGGTCATG

2 CATGACCACTAGGAGCATCTTTGGCGACGTGGGCACTCAGGCAAGAAGTGTGGTCGACTAGGGGAATAAATCTTTGGGCACCTAGTGGTCATG

2 CATGACCACTAGGAGCATCTTTGGCGAGCGTAGGTGCTCAGCTCAATGGGAAGCCGACTAGGGGAATAAATCTTTGGGCACCTAGTGGTCATG

2 CATGACCACTAGGAGCATCTTTGGCGACTGCGTAGGCGGATGCTTGGCTAGGACATTCTAGGGGAATAAATCTTTGGGCACCTAGTGGTCATG

2 CATGACCACTAGGAGCATCTTTGGCGAAACGGGTCGGTGACCTGCGTCGAAGTGGAGCTAGGGGAATAAATCTTTGGGCACCTAGTGGTCATG

2 CATGACCACTAGGAGCATCTTTGGCGAGGGTACCTCGTATAAGAATGTGGCCGAGCACTAGGGGAATAAATCTTTGGGCACCTAGTGGTCATG

2 CATGACCACTAGGAGCATCTTTGGCGAGTATTGGTTGGTAGGTTGCGTATTGGGGAGCTAGGGGAATAAATCTTTGGGCACCTAGTGGTCATG

2 CATGACCACTAGGAGCATCTTTGGCGAGATCGGGAGAATCGGTGGCCGTGGTGTCTCCTAGGGGAATAAATCTTTGGGCACCTAGTGGTCATG

2 CATGACCACTAGGAGCATCTTTGGCGACAGGACGCGATGCGGGATCCAGCGGTCGCACTAGGGGAATAAATCTTTGGGCACCTAGTGGTCATG

2 CATGACCACTAGGAGCATCTTTGGCGAACGGCAGTTGTTGCGGTGGTCGGTGAATCCCTAGGGGAATAAATCTTTGGGCACCTAGTGGTCATG

2 CATGACCACTAGGAGCATCTTTGGCGACTCGTGGCTGAGAAGCGATATACCGAGCGACTAGGGGAATAAATCTTTGGGCACCTAGTGGTCATG

2 CATGACCACTAGGAGCATCTTTGGCGACAGGGAATGCCGATGCGCTGGACGACCGAGCTAGGGGAATAAATCTTTGGGCACCTAGTGGTCATG

2 CATGACCACTAGGAGCATCTTTGGCGACGCCTCAGGGGGGTACCCGGTAGGGCGTAGCTAGGGGAATAAATCTTTGGGCACCTAGTGGTCATG

2 CATGACCACTAGGAGCATCTTTGGCGACTAAGCTCGAGGAATTGAAGAGCCAGTTGGCTAGGGGAATAAATCTTTGGGCACCTAGTGGTCATG

2 CATGACCACTAGGAGCATCTTTGGCGAGATCGGGAGAATCGGCGGCATAGGTGTCTCCTAGGGGAATAAATCTTTGGGCACCTAGTGGTCATG

2 CATGACCACTAGGAGCATCTTTGGCGAGGTTGTAGCTCGCGCGCAGGGTCGGCAGTGCTAGGGGAATAAATCTTTGGGCACCTAGTGGTCATG

2 CATGACCACTAGGAGCATCTTTGGCGAGAAGACTCAGGATTCGGGGACCAGTTGCTCCTAGGGGAATAAATCTTTGGGCACCTAGTGGTCATG

2 CATGACCACTAGGAGCATCTTTGGCGACGTCCGTGGGTATCGGGGACCTGAAGTCGACTAGGGGAATAAATCTTTGGGCACCTAGTGGTCATG

2 CATGACCACTAGGAGCATCTTTGGCGACCCAGCGACCGATCAGGGAGCACGGGTGGACTAGGGGAATAAATCTTTGGGCACCTAGTGGTCATG

2 CATGACCACTAGGAGCATCTTTGGCGACGACGTGGGCGAAGGATAAAAGACCGTCGACTAGGGGAATAAATCTTTGGGCACCTAGTGGTCATG

2 CATGACCACTAGGAGCATCTTTGGCGACGGTGTGGGTGCGGGGCCAGGCGGTTGATCCTAGGGGAATAAATCTTTGGGCACCTAGTGGTCATG

2 CATGACCACTAGGAGCATCTTTGGCGAACGTGCAAGATATGCTGCCTGGAGCGTAGGCTAGGGGAATAAATCTTTGGGCACCTAGTGGTCATG

2 CATGACCACTAGGAGCATCTTTGGCGAGAGGCGGGAAGGGCGCTGGGTTGGGGAAGACTAGGGGAATAAATCTTTGGGCACCTAGTGGTCATG

2 CATGACCACTAGGAGCATCTTTGGCGAGATCGGGAGAAACGGCGGCATTGGTGTCTCCTAGGGGAATAAATCTTTGGGCACCTAGTGGTCATG

2 CATGACCACTAGGAGCATCTTTGGCGATAAAAGGCATCGGAGGTAAGTGCTTATCGACTAGGGGAATAAATCTTTGGGCACCTAGTGGTCATG

2 CATGACCACTAGGAGCATCTTTGGCGACGGAATGTGTGACGTGCGTGGCAGAGTCGACTAGGGGAATAAATCTTTGGGCACCTAGTGGTCATG

2 CATGACCACTAGGAGCATCTTTGGCGAGAGCTTGAAAAGGGTGCGTTGTTGTCGCATCTAGGGGAATAAATCTTTGGGCACCTAGTGGTCATG

2 CATGACCACTAGGAGCATCTTTGGCGACGTCGTGGACCTGGCTTCCGGGCAGGAGCTCTAGGGGAATAAATCTTTGGGCACCTAGTGGTCATG

2 CATGACCACTAGGAGCATCTTTGGCGAGCCTTGCTTGGGAGGTTGCTCCGCCAGTTCCTAGGGGAATAAATCTTTGGGCACCTAGTGGTCATG

2 CATGACCACTAGGAGCATCTTTGGCGATCCCGGGCGCCGTGGGCGATCGTGGTGGCTCTAGGGGAATAAATCTTTGGGCACCTAGTGGTCATG

2 CATGACCACTAGGAGCATCTTTGGCGACGTCATCAGATGACAGGTTTGCGACCGAGCCTAGGGGAATAAATCTTTGGGCACCTAGTGGTCATG

2 CATGACCACTAGGAGCATCTTTGGCGAACGACAAGGTTGGGCGCCTTGTGCTCCGCACTAGGGGAATAAATCTTTGGGCACCTAGTGGTCATG

2 CATGACCACTAGGAGCATCTTTGGCGACCGGCAGGGGTTAGCGTCACTAGTTCAGCTCTAGGGGAATAAATCTTTGGGCACCTAGTGGTCATG

2 CATGACCACTAGGAGCATCTTTGGCGAAGCGTAGTCGTTAGATTGGTTAGGTTGATCCTAGGGGAATAAATCTTTGGGCACCTAGTGGTCATG

2 CATGACCACTAGGAGCATCTTTGGCGAGATCGGGAGAATCGGTGGCATTGGTGACTACTAGGGGAATAAATCTTTGGGCACCTAGTGGTCATG

2 CATGACCACTAGGAGCATCTTTGGCGAAGGGTGTAGGACTTCAAGTTGATCTCATAGCTAGGGGAATAAATCTTTGGGCACCTAGTGGTCATG

2 CATGACCACTAGGAGCATCTTTGGCGACACTTGTGGGCTGGTGGGATCGTTTGTCGACTAGGGGAATAAATCTTTGGGCACCTAGTGGTCATG

2 CATGACCACTAGGAGCATCTTTGGCGATGAGGGATGGTTCGTAGAGAGTGCTCTCGACTAGGGGAATAAATCTTTGGGCACCTAGTGGTCATG

2 CATGACCACTAGGAGCATCTTTGGCGAACGTGCAAGATATGCCGCCTGGGGCGTAGGCTAGGGGAATAAATCTTTGGGCACCTAGTGGTCATG

2 CATGACCACTAGGAGCATCTTTGGCGAGCGATCGGCTGGTAGGGAGTATTGAGTTAGCTAGGGGAATAAATCTTTGGGCACCTAGTGGTCATG

2 CATGACCACTAGGAGCATCTTTGGCGACACGCGTAACGCGGGGTGGATGGATGTCGACTAGGGGAATAAATCTTTGGGCACCTAGTGGTCATG

2 CATGACCACTAGGAGCATCTTTGGCGAGACGAGGATCGGTAGTATAAGGAGCTCGAGCTAGGGGAATAAATCTTTGGGCACCTAGTGGTCATG

2 CATGACCACTAGGAGCATCTTTGGCGAGGGAGGGCGCCGGCAGCGGTGTGAATGCGGCTAGGGGAATAAATCTTTGGGCACCTAGTGGTCATG

2 CATGACCACTAGGAGCATCTTTGGCGAGAGCGCAGGGCATGCGACCTCCAATTGGTCCTAGGGGAATAAATCTTTGGGCACCTAGTGGTCATG

2 CATGACCACTAGGAGCATCTTTGGCGACTTGGGGGCGTCTCGAGCAAACACGTTCGACTAGGGGAATAAATCTTTGGGCACCTAGTGGTCATG

2 CATGACCACTAGGAGCATCTTTGGCGACAAAGTAGCGTGTTGTGGCATATTTGACGCCTAGGGGAATAAATCTTTGGGCACCTAGTGGTCATG

2 CATGACCACTAGGAGCATCTTTGGCGACTGACGGATAGCCGGCTATTCCGAAGTGGACTAGGGGAATAAATCTTTGGGCACCTAGTGGTCATG

2 CATGACCACTAGGAGCATCTTTGGCGAACGGCAGGTGTTGCGTTGGTCTGTGAAACCCTAGGGGAATAAATCTTTGGGCACCTAGTGGTCATG

2 CATGACCACTAGGAGCATCTTTGGCGACGTCGAAGAGTACGCAAGGTGCAAGGGAGCCTAGGGGAATAAATCTTTGGGCACCTAGTGGTCATG

2 CATGACCACTAGGAGCATCTTTGGCGACCCGACGTGATATCATCATCTCGTGGTGGACTAGGGGAATAAATCTTTGGGCACCTAGTGGTCATG

2 CATGACCACTAGGAGCATCTTTGGCGATAGGCGTGTACGGTCGAGCGAAAGCGCTAGCTAGGGGAATAAATCTTTGGGCACCTAGTGGTCATG

2 CATGACCACTAGGAGCATCTTTGGCGAGGAGACGTTCAGTCTATGGTTCAGAGGTAGCTAGGGGAATAAATCTTTGGGCACCTAGTGGTCATG

2 CATGACCACTAGGAGCATCTTTGGCGAGGGGAACTGTCGGGCCGGAGTGCTCCAACGCTAGGGGAATAAATCTTTGGGCACCTAGTGGTCATG

2 CATGACCACTAGGAGCATCTTTGGCGAGACATGACTGGCCAAGGCAATAGGCCGACACTAGGGGAATAAATCTTTGGGCACCTAGTGGTCATG

2 CATGACCACTAGGAGCATCTTTGGCGACGGTGTAGGCGTTGAGTTGGTGTTCCGAGGCTAGGGGAATAAATCTTTGGGCACCTAGTGGTCATG

2 CATGACCACTAGGAGCATCTTTGGCGACACAAGGGTTGGGCAGTCGAAGGGTGGCGACTAGGGGAATAAATCTTTGGGCACCTAGTGGTCATG

2 CATGACCACTAGGAGCATCTTTGGCGATGGGGCCGTGCTATCTGCACGCTCCGCGGGCTAGGGGAATAAATCTTTGGGCACCTAGTGGTCATG

2 CATGACCACTAGGAGCATCTTTGGCGACGTAGGTGTCAGGGCGGCTCAGTTTGTCGACTAGGGGAATAAATCTTTGGGCACCTAGTGGTCATG

2 CATGACCACTAGGAGCATCTTTGGCGAACAGGCCATGGCTGCGGGTGGGTGCGCACCCTAGGGGAATAAATCTTTGGGCACCTAGTGGTCATG

2 CATGACCACTAGGAGCATCTTTGGCGAATTGGAAAATAGCTTTGTTGGGCTCGGGCCCTAGGGGAATAAATCTTTGGGCACCTAGTGGTCATG

2 CATGACCACTAGGAGCATCTTTGGCGAAGCGAAGTGCGTCACTGTGTGGCTTGTCACCTAGGGGAATAAATCTTTGGGCACCTAGTGGTCATG

2 CATGACCACTAGGAGCATCTTTGGCGATCTAAGGGCGCGCAAGGTGCAGAAGATCGCCTAGGGGAATAAATCTTTGGGCACCTAGTGGTCATG

2 CATGACCACTAGGAGCATCTTTGGCGAATGGCCGGCACGGCCATCAAGTCCTCGGTACTAGGGGAATAAATCTTTGGGCACCTAGTGGTCATG

2 CATGACCACTAGGAGCATCTTTGGCGACAAGAAAGAAGGCGGTAGCCGAAATACAGCCTAGGGGAATAAATCTTTGGGCACCTAGTGGTCATG

2 CATGACCACTAGGAGCATCTTTGGCGAGTATTGGCTGGTCGGTTGCGTATTGGGGAGCTAGGGGAATAAATCTTTGGGCACCTAGTGGTCATG

2 CATGACCACTAGGAGCATCTTTGGCGAGTAGGGTCGAAGAACAGCCGCCGAATGAGCCTAGGGGAATAAATCTTTGGGCACCTAGTGGTCATG

2 CATGACCACTAGGAGCATCTTTGGCGAGATCGGGAGAATCGGTGGCATCGGTTTCTCCTAGGGGAATAAATCTTTGGGCACCTAGTGGTCATG

2 CATGACCACTAGGAGCATCTTTGGCGACTATCGAGATAAGACAGGCAATGAAGTCGACTAGGGGAATAAATCTTTGGGCACCTAGTGGTCATG

2 CATGACCACTAGGAGCATCTTTGGCGACGGTGTGGGGAACTGGTTTCGGCGGTGCTACTAGGGGAATAAATCTTTGGGCACCTAGTGGTCATG

2 CATGACCACTAGGAGCATCTTTGGCGACCACGTCGGCAGGATGTCGAATCTGTGGAGCTAGGGGAATAAATCTTTGGGCACCTAGTGGTCATG

2 CATGACCACTAGGAGCATCTTTGGCGACCCTCAGCGCAACGCAGGTAGATGGGTGGACTAGGGGAATAAATCTTTGGGCACCTAGTGGTCATG

2 CATGACCACTAGGAGCATCTTTGGCGATCAGGCCGCCAGGGCGTACTAAGTGTGGTTCTAGGGGAATAAATCTTTGGGCACCTAGTGGTCATG

2 CATGACCACTAGGAGCATCTTTGGCGAACGTAGTCGAAGTGGAGGTGCTACTGAGCTCTAGGGGAATAAATCTTTGGGCACCTAGTGGTCATG

2 CATGACCACTAGGAGCATCTTTGGCGAATGGCCGGCACGGCCTTCTAGTCCGTGGTACTAGGGGAATAAATCTTTGGGCACCTAGTGGTCATG

2 CATGACCACTAGGAGCATCTTTGGCGAAGACGGTCAGGATGATCCGACACTCGGCGCCTAGGGGAATAAATCTTTGGGCACCTAGTGGTCATG

2 CATGACCACTAGGAGCATCTTTGGCGAGGCGAGTCCAGCGGCCTTCTTGACCTGCGACTAGGGGAATAAATCTTTGGGCACCTAGTGGTCATG

2 CATGACCACTAGGAGCATCTTTGGCGACCCGCAGACTCCTAGGTCAGGCGTGGTGGACTAGGGGAATAAATCTTTGGGCACCTAGTGGTCATG

2 CATGACCACTAGGAGCATCTTTGGCGAGATAGGGAGAATCGGTGGCATTGGTGTATCCTAGGGGAATAAATCTTTGGGCACCTAGTGGTCATG

2 CATGACCACTAGGAGCATCTTTGGCGACAGGGGATGCCTATGCGCTGGACGACCGAGCTAGGGGAATAAATCTTTGGGCACCTAGTGGTCATG

2 CATGACCACTAGGAGCATCTTTGGCGACAGACACCTGGAGCCTATGGTGGCCGAGCTCTAGGGGAATAAATCTTTGGGCACCTAGTGGTCATG

2 CATGACCACTAGGAGCATCTTTGGCGACGACGGGGATGAGACGCGAGAGGAGTTCGACTAGGGGAATAAATCTTTGGGCACCTAGTGGTCATG

2 CATGACCACTAGGAGCATCTTTGGCGATTAGCGTGTACGGTCGAGCGAAAGCGATAGCTAGGGGAATAAATCTTTGGGCACCTAGTGGTCATG

2 CATGACCACTAGGAGCATCTTTGGCGACAGCGGAAGGGGGTATTCTCGAGCTCCTGCCTAGGGGAATAAATCTTTGGGCACCTAGTGGTCATG

2 CATGACCACTAGGAGCATCTTTGGCGAGAAGACTCTGGATTCGGAGACCAGTTGCTCCTAGGGGAATAAATCTTTGGGCACCTAGTGGTCATG

2 CATGACCACTAGGAGCATCTTTGGCGACGAAGGTGGGATCGTCAGGCGGTGAGGCGACTAGGGGAATAAATCTTTGGGCACCTAGTGGTCATG

2 CATGACCACTAGGAGCATCTTTGGCGAGGGAGTGCGCCGGCAGCGGTGTGAATGCGCCTAGGGGAATAAATCTTTGGGCACCTAGTGGTCATG

2 CATGACCACTAGGAGCATCTTTGGCGAGTACGTGGTCGCGGGCAAGGCGAAACGAGACTAGGGGAATAAATCTTTGGGCACCTAGTGGTCATG

2 CATGACCACTAGGAGCATCTTTGGCGAACCACAGACGAGCATGATGCTCCGGTGAAGCTAGGGGAATAAATCTTTGGGCACCTAGTGGTCATG

2 CATGACCACTAGGAGCATCTTTGGCGATCCGCGTAAGGGCGAAGCGTCGTGGTGGAGCTAGGGGAATAAATCTTTGGGCACCTAGTGGTCATG

2 CATGACCACTAGGAGCATCTTTGGCGACGCGATATGCGAGAGACTGAGCCACAAGCACTAGGGGAATAAATCTTTGGGCACCTAGTGGTCATG

2 CATGACCACTAGGAGCATCTTTGGCGAGGGGTCATGGGAATGTAATTGTGCTTAAGACTAGGGGAATAAATCTTTGGGCACCTAGTGGTCATG

2 CATGACCACTAGGAGCATCTTTGGCGAGGGGTAGGGGGCCGCTCGCGCTTGCGTGCGCTAGGGGAATAAATCTTTGGGCACCTAGTGGTCATG

2 CATGACCACTAGGAGCATCTTTGGCGACCCAGGCACCGATAGTGGCCTCGTGGTGGACTAGGGGAATAAATCTTTGGGCACCTAGTGGTCATG

2 CATGACCACTAGGAGCATCTTTGGCGACAGACTCGGGCAGAGTCTACGAAGTGGCGACTAGGGGAATAAATCTTTGGGCACCTAGTGGTCATG

2 CATGACCACTAGGAGCATCTTTGGCGAACTGAGAGATCGGTTTAGACCAGAGAGAGCCTAGGGGAATAAATCTTTGGGCACCTAGTGGTCATG

2 CATGACCACTAGGAGCATCTTTGGCGACCCCGGACGGTGCGAAGTTCCGGTGGTGGACTAGGGGAATAAATCTTTGGGCACCTAGTGGTCATG

2 CATGACCACTAGGAGCATCTTTGGCGAAGATCCAATGGGCTTGTGACCCGCCTCGGCCTAGGGGAATAAATCTTTGGGCACCTAGTGGTCATG

2 CATGACCACTAGGAGCATCTTTGGCGACGGTGGGCTTCGGCCGTATGGGATAGTCGACTAGGGGAATAAATCTTTGGGCACCTAGTGGTCATG

2 CATGACCACTAGGAGCATCTTTGGCGAAGTCTGCTGGTAGGAGTGTACGGGATCGCACTAGGGGAATAAATCTTTGGGCACCTAGTGGTCATG

2 CATGACCACTAGGAGCATCTTTGGCGAGACACGTGCAACGAGTAGTCAACTGTCGATCTAGGGGAATAAATCTTTGGGCACCTAGTGGTCATG

2 CATGACCACTAGGAGCATCTTTGGCGACGAGGGGGCGGGAGATGTGGGGAACGGAGACTAGGGGAATAAATCTTTGGGCACCTAGTGGTCATG

2 CATGACCACTAGGAGCATCTTTGGCGATCGTGTGGAGGAAACAGTCGGGCACGATGCCTAGGGGAATAAATCTTTGGGCACCTAGTGGTCATG

2 CATGACCACTAGGAGCATCTTTGGCGAACGGTAGTAGGGCATAATGCCCAAGTGGAGCTAGGGGAATAAATCTTTGGGCACCTAGTGGTCATG

2 CATGACCACTAGGAGCATCTTTGGCGAGATAGGTGGGACCAACGTGTCCTCCTAAGCCTAGGGGAATAAATCTTTGGGCACCTAGTGGTCATG

2 CATGACCACTAGGAGCATCTTTGGCGACCTAGGAGGACTAAGTGCCGTGATGGGAGACTAGGGGAATAAATCTTTGGGCACCTAGTGGTCATG

2 CATGACCACTAGGAGCATCTTTGGCGAGCCTTGCGTGGGAGGTTGCTCCACCAGTTCCTAGGGGAATAAATCTTTGGGCACCTAGTGGTCATG

2 CATGACCACTAGGAGCATCTTTGGCGATGAGTGGGCACGTAGATCTACGCCTCAACGCTAGGGGAATAAATCTTTGGGCACCTAGTGGTCATG

2 CATGACCACTAGGAGCATCTTTGGCGACAGTGGGCGTTGGGGCGACGACACGGAGAGCTAGGGGAATAAATCTTTGGGCACCTAGTGGTCATG

2 CATGACCACTAGGAGCATCTTTGGCGACTATGTTGGGAATATAGCAGGAGCAAGTCGCTAGGGGAATAAATCTTTGGGCACCTAGTGGTCATG

2 CATGACCACTAGGAGCATCTTTGGCGAATCGTGGTTGCCTCGATGTGGGAGATGAGACTAGGGGAATAAATCTTTGGGCACCTAGTGGTCATG

2 CATGACCACTAGGAGCATCTTTGGCGAGAAGACTCTGGATTCGGTGACCAGTTGATGCTAGGGGAATAAATCTTTGGGCACCTAGTGGTCATG

2 CATGACCACTAGGAGCATCTTTGGCGAAGGGTGTAGGACTTCAAGTGGACCTCATAGCTAGGGGAATAAATCTTTGGGCACCTAGTGGTCATG

2 CATGACCACTAGGAGCATCTTTGGCGACAGATCTTAGTACTGCAACCGGAGAGTCGACTAGGGGAATAAATCTTTGGGCACCTAGTGGTCATG

2 CATGACCACTAGGAGCATCTTTGGCGATATCGGCGTTAGCTAACATTGTAGGATGTTCTAGGGGAATAAATCTTTGGGCACCTAGTGGTCATG

2 CATGACCACTAGGAGCATCTTTGGCGACGAGTCGACAGGACTCTCGCAGACGTTTGGCTAGGGGAATAAATCTTTGGGCACCTAGTGGTCATG

2 CATGACCACTAGGAGCATCTTTGGCGACTGCCGAGGATGAGTTAAGTGGTCGAGCGCCTAGGGGAATAAATCTTTGGGCACCTAGTGGTCATG

2 CATGACCACTAGGAGCATCTTTGGCGATTGGAAGTCGCCCGATGGCAGGCAAGTGAGCTAGGGGAATAAATCTTTGGGCACCTAGTGGTCATG

2 CATGACCACTAGGAGCATCTTTGGCGATGGGCGCTCTGGGTGAGACCTTCATTGGTCCTAGGGGAATAAATCTTTGGGCACCTAGTGGTCATG

2 CATGACCACTAGGAGCATCTTTGGCGAACATGCAAGATATGCCGCCTGGAGCGTAGGCTAGGGGAATAAATCTTTGGGCACCTAGTGGTCATG

2 CATGACCACTAGGAGCATCTTTGGCGAACGGCAGGTGTTGCGTTGGTCTGAGAATCCCTAGGGGAATAAATCTTTGGGCACCTAGTGGTCATG

2 CATGACCACTAGGAGCATCTTTGGCGACCCACTGGGCTGAAAAGCATGTGTGGTGGACTAGGGGAATAAATCTTTGGGCACCTAGTGGTCATG

2 CATGACCACTAGGAGCATCTTTGGCGACGACGGATGTAAGCAGCACGCGGGAGGCGACTAGGGGAATAAATCTTTGGGCACCTAGTGGTCATG

2 CATGACCACTAGGAGCATCTTTGGCGAGCGGCAGGTGTTGCGGTGGTCTGTGGATCCCTAGGGGAATAAATCTTTGGGCACCTAGTGGTCATG

2 CATGACCACTAGGAGCATCTTTGGCGACGAAGGTCTTTTAGTGGCCCGAGGGCCGACCTAGGGGAATAAATCTTTGGGCACCTAGTGGTCATG

2 CATGACCACTAGGAGCATCTTTGGCGATGGGTAGGGGGCCGCTCGCGCCTGCGTGCGCTAGGGGAATAAATCTTTGGGCACCTAGTGGTCATG

2 CATGACCACTAGGAGCATCTTTGGCGACTAAGGTGGTTGAGTCCACCAGTGGGAAGACTAGGGGAATAAATCTTTGGGCACCTAGTGGTCATG

2 CATGACCACTAGGAGCATCTTTGGCGACGGTGTGGGTGCAGGGCCAGGCGGTTGACCCTAGGGGAATAAATCTTTGGGCACCTAGTGGTCATG

2 CATGACCACTAGGAGCATCTTTGGCGAAACGTGCAGACAATATGTGCTAGTGTGGAGCTAGGGGAATAAATCTTTGGGCACCTAGTGGTCATG

2 CATGACCACTAGGAGCATCTTTGGCGACCCCGGTTGGCTGCACAAACCAGGGGTGGACTAGGGGAATAAATCTTTGGGCACCTAGTGGTCATG

2 CATGACCACTAGGAGCATCTTTGGCGACAGCGAGCATAGTTGCGATCGGTATCCCGACTAGGGGAATAAATCTTTGGGCACCTAGTGGTCATG

2 CATGACCACTAGGAGCATCTTTGGCGAACTGCAGGTGGTGCGGTGGTCTGTGAATCCCTAGGGGAATAAATCTTTGGGCACCTAGTGGTCATG

2 CATGACCACTAGGAGCATCTTTGGCGATGGGGCCGTGCTATCTGCACACTCCCCGGGCTAGGGGAATAAATCTTTGGGCACCTAGTGGTCATG

2 CATGACCACTAGGAGCATCTTTGGCGATGGGTGCTGACGGCCGTCGCTGCGGCTACACTAGGGGAATAAATCTTTGGGCACCTAGTGGTCATG

2 CATGACCACTAGGAGCATCTTTGGCGAGGGAGGGCGCCGGCAGCGGAGTGAATGCGCCTAGGGGAATAAATCTTTGGGCACCTAGTGGTCATG

2 CATGACCACTAGGAGCATCTTTGGCGATGGGGCCGTGCTATTCGCACACTCCGCGGGCTAGGGGAATAAATCTTTGGGCACCTAGTGGTCATG

2 CATGACCACTAGGAGCATCTTTGGCGACCTCGTGCGAAGTGGCTTGAGAATGGACGACTAGGGGAATAAATCTTTGGGCACCTAGTGGTCATG

2 CATGACCACTAGGAGCATCTTTGGCGAATGTCCGGCACGGCCTACTAGTCCTCGGTACTAGGGGAATAAATCTTTGGGCACCTAGTGGTCATG

2 CATGACCACTAGGAGCATCTTTGGCGACCCTGAAGAGGATACCAATTCAGCGGTGGACTAGGGGAATAAATCTTTGGGCACCTAGTGGTCATG

2 CATGACCACTAGGAGCATCTTTGGCGACTACGTGGGCCAGGCACTGATTCCAGTCGACTAGGGGAATAAATCTTTGGGCACCTAGTGGTCATG

2 CATGACCACTAGGAGCATCTTTGGCGATCCTCGTCAGATGGTGAAGTAGACGTTTGGCTAGGGGAATAAATCTTTGGGCACCTAGTGGTCATG

2 CATGACCACTAGGAGCATCTTTGGCGACTAAGTGGGCTGGACCGAAAACGAGCGCGCCTAGGGGAATAAATCTTTGGGCACCTAGTGGTCATG

2 CATGACCACTAGGAGCATCTTTGGCGACATTTGATAGCTGCTTAGCTAAATAGCCATCTAGGGGAATAAATCTTTGGGCACCTAGTGGTCATG

2 CATGACCACTAGGAGCATCTTTGGCGAAGTCCGAGATGTGACTAAATGATATTTCGCCTAGGGGAATAAATCTTTGGGCACCTAGTGGTCATG

2 CATGACCACTAGGAGCATCTTTGGCGACCCAGACACGGTCTGTGCGTGGGGCAGAGCCTAGGGGAATAAATCTTTGGGCACCTAGTGGTCATG

2 CATGACCACTAGGAGCATCTTTGGCGACCCTTCAGTGTGGAACATCACAGGGGTGGACTAGGGGAATAAATCTTTGGGCACCTAGTGGTCATG

2 CATGACCACTAGGAGCATCTTTGGCGATAGGCGTTTACGGTCGAGCGAAAGCGATAGCTAGGGGAATAAATCTTTGGGCACCTAGTGGTCATG

2 CATGACCACTAGGAGCATCTTTGGCGATGGGGCCTTGCTATCTGCACATTCCGCGGGCTAGGGGAATAAATCTTTGGGCACCTAGTGGTCATG

2 CATGACCACTAGGAGCATCTTTGGCGACGTCGGTTGGATGGTAGTATTAAGTAAGTCCTAGGGGAATAAATCTTTGGGCACCTAGTGGTCATG

2 CATGACCACTAGGAGCATCTTTGGCGAGCGGTAGAGTATATGTTCACCCGCAGTCGCCTAGGGGAATAAATCTTTGGGCACCTAGTGGTCATG

2 CATGACCACTAGGAGCATCTTTGGCGATGGGGCCGTGCTATCTGCACACTCCGCGGTCTAGGGGAATAAATCTTTGGGCACCTAGTGGTCATG

2 CATGACCACTAGGAGCATCTTTGGCGATATCGGGAGAATCGGTGGCATTGGTTTCTACTAGGGGAATAAATCTTTGGGCACCTAGTGGTCATG

2 CATGACCACTAGGAGCATCTTTGGCGACCGGGGGGAGCGCCCACGAGTGGCTGAGTGCTAGGGGAATAAATCTTTGGGCACCTAGTGGTCATG

2 CATGACCACTAGGAGCATCTTTGGCGAGTGAGGGCGTTCGGGGTTCTTAGGACCGACCTAGGGGAATAAATCTTTGGGCACCTAGTGGTCATG

2 CATGACCACTAGGAGCATCTTTGGCGAACGGCAGGTGTTGCGGTGGTCTGTGTAACCCTAGGGGAATAAATCTTTGGGCACCTAGTGGTCATG

2 CATGACCACTAGGAGCATCTTTGGCGAGTCGATATGAAGCGACGAGCAGACGTTTGGCTAGGGGAATAAATCTTTGGGCACCTAGTGGTCATG

2 CATGACCACTAGGAGCATCTTTGGCGATACGGGAGACTGCTACGGCGTTCTCATCGACTAGGGGAATAAATCTTTGGGCACCTAGTGGTCATG

2 CATGACCACTAGGAGCATCTTTGGCGAAGGGGCCGTGCTATCTGCACACTCCGCGGGCTAGGGGAATAAATCTTTGGGCACCTAGTGGTCATG

2 CATGACCACTAGGAGCATCTTTGGCGACCCTGACAGGCTGGGCTGAGTTCTGGGAGACTAGGGGAATAAATCTTTGGGCACCTAGTGGTCATG

2 CATGACCACTAGGAGCATCTTTGGCGAGTTTGAGTAGGCCCTCGGGACGCGCTGAGTCTAGGGGAATAAATCTTTGGGCACCTAGTGGTCATG

2 CATGACCACTAGGAGCATCTTTGGCGAGGAGTGGGCGCATGAGAACGAACCAAGCCGCTAGGGGAATAAATCTTTGGGCACCTAGTGGTCATG

2 CATGACCACTAGGAGCATCTTTGGCGAACGGCAGGTGTTGCGGTGGCTTGTGAATCCCTAGGGGAATAAATCTTTGGGCACCTAGTGGTCATG

2 CATGACCACTAGGAGCATCTTTGGCGATGGGGCCGTGCTATCTGCACCCTCCGCGGGCTAGGGGAATAAATCTTTGGGCACCTAGTGGTCATG

2 CATGACCACTAGGAGCATCTTTGGCGACCCGGCAACAGTGGAACCGAGCCGGAGTGACTAGGGGAATAAATCTTTGGGCACCTAGTGGTCATG

2 CATGACCACTAGGAGCATCTTTGGCGACGGCGTGGGGCTATAGACGCGGCGGCACTCCTAGGGGAATAAATCTTTGGGCACCTAGTGGTCATG

2 CATGACCACTAGGAGCATCTTTGGCGAGATCGTGAGAATCGGTGGCATTGTTGTCTCCTAGGGGAATAAATCTTTGGGCACCTAGTGGTCATG

2 CATGACCACTAGGAGCATCTTTGGCGACGTGGGTAGGCGGGTAGTGTGGCGGAGAGACTAGGGGAATAAATCTTTGGGCACCTAGTGGTCATG

2 CATGACCACTAGGAGCATCTTTGGCGAACGGCAAGTGTTGCGGTGGTCTGTGTATCCCTAGGGGAATAAATCTTTGGGCACCTAGTGGTCATG

2 CATGACCACTAGGAGCATCTTTGGCGAACGGCAGGGGTTGCGGGGGTCTGTGAATCCCTAGGGGAATAAATCTTTGGGCACCTAGTGGTCATG

2 CATGACCACTAGGAGCATCTTTGGCGAGGGGACGGGACTGGTTGACCCGCCCAAAGCCTAGGGGAATAAATCTTTGGGCACCTAGTGGTCATG

2 CATGACCACTAGGAGCATCTTTGGCGACGTAGTGCATAAGCTGCGGGAATTGGTCGACTAGGGGAATAAATCTTTGGGCACCTAGTGGTCATG

2 CATGACCACTAGGAGCATCTTTGGCGAGATCGGGAGAATCGGAGGCAGTGGTGTCTCCTAGGGGAATAAATCTTTGGGCACCTAGTGGTCATG

2 CATGACCACTAGGAGCATCTTTGGCGATCCCGTAGGGTGTGGACATAAACAGATCGACTAGGGGAATAAATCTTTGGGCACCTAGTGGTCATG

2 CATGACCACTAGGAGCATCTTTGGCGACCCGTAGTGTTCTGAACATGCGCGGGTGGACTAGGGGAATAAATCTTTGGGCACCTAGTGGTCATG

2 CATGACCACTAGGAGCATCTTTGGCGAAAGAGGCGATTGACCTACTGATGCTTTTCGCTAGGGGAATAAATCTTTGGGCACCTAGTGGTCATG

2 CATGACCACTAGGAGCATCTTTGGCGACTAGGGGGCGGTCCGACCCCGTGCGTGGAGCTAGGGGAATAAATCTTTGGGCACCTAGTGGTCATG

2 CATGACCACTAGGAGCATCTTTGGCGAGATCGGGAGAATCGGTGGCAGTGGTGTCCCCTAGGGGAATAAATCTTTGGGCACCTAGTGGTCATG

2 CATGACCACTAGGAGCATCTTTGGCGAAAGTCGGTAGAACAGGGTCGGGTGCTGTCCCTAGGGGAATAAATCTTTGGGCACCTAGTGGTCATG

2 CATGACCACTAGGAGCATCTTTGGCGACACGGCACGGCAGGCAAAACCAGATGTCGACTAGGGGAATAAATCTTTGGGCACCTAGTGGTCATG

2 CATGACCACTAGGAGCATCTTTGGCGAGTGAGCGCGAGGTACAGCGTATGCAGCCAGCTAGGGGAATAAATCTTTGGGCACCTAGTGGTCATG

2 CATGACCACTAGGAGCATCTTTGGCGAGGCATGTGCCAGCACCAAATAAGCCGTTGCCTAGGGGAATAAATCTTTGGGCACCTAGTGGTCATG

2 CATGACCACTAGGAGCATCTTTGGCGACACACGAAGCCGTGGCGTTCGTGCTGTGGACTAGGGGAATAAATCTTTGGGCACCTAGTGGTCATG

2 CATGACCACTAGGAGCATCTTTGGCGACCCGTGAAAACGTAGTAGCAGGCGGGTGGACTAGGGGAATAAATCTTTGGGCACCTAGTGGTCATG

2 CATGACCACTAGGAGCATCTTTGGCGACGGGTGGGCGCCCAAGGGTGTGAGTGTTGCCTAGGGGAATAAATCTTTGGGCACCTAGTGGTCATG

2 CATGACCACTAGGAGCATCTTTGGCGACCGAGCATTTGGCGCGTTAAGTGGGCGTAGCTAGGGGAATAAATCTTTGGGCACCTAGTGGTCATG

2 CATGACCACTAGGAGCATCTTTGGCGAGATCGGGAGACTCGGTGGCAGTGGTGTCTCCTAGGGGAATAAATCTTTGGGCACCTAGTGGTCATG

2 CATGACCACTAGGAGCATCTTTGGCGAATACAGGCATGGTGTAAATCAGGAATGCGCCTAGGGGAATAAATCTTTGGGCACCTAGTGGTCATG

2 CATGACCACTAGGAGCATCTTTGGCGACCCGGCATATGTGGTGGGCGTTGATGTCGCCTAGGGGAATAAATCTTTGGGCACCTAGTGGTCATG

2 CATGACCACTAGGAGCATCTTTGGCGAAAGGCAGGTGTTTCGGTGGTCTGTGAATCCCTAGGGGAATAAATCTTTGGGCACCTAGTGGTCATG

2 CATGACCACTAGGAGCATCTTTGGCGACCCACGGTAAAGCAGCGTACGTGTGGTGGACTAGGGGAATAAATCTTTGGGCACCTAGTGGTCATG

2 CATGACCACTAGGAGCATCTTTGGCGAAGAATGCAGCCATATCTTGCGCGTGTGTTACTAGGGGAATAAATCTTTGGGCACCTAGTGGTCATG

2 CATGACCACTAGGAGCATCTTTGGCGATGGGGCCGTGCTATCTGCACACTCCGCGAGCTAGGGGAATAAATCTTTGGGCACCTAGTGGTCATG

2 CATGACCACTAGGAGCATCTTTGGCGACGGTGTGGGTGCAGTGCCAGGCGGTTGATCCTAGGGGAATAAATCTTTGGGCACCTAGTGGTCATG

2 CATGACCACTAGGAGCATCTTTGGCGACCAGTTGCAAACGATTCAGTGGCCGAGCGCCTAGGGGAATAAATCTTTGGGCACCTAGTGGTCATG

2 CATGACCACTAGGAGCATCTTTGGCGACAGTGGGCTCAAAGGTAAGACGAACAGTCGCTAGGGGAATAAATCTTTGGGCACCTAGTGGTCATG

2 CATGACCACTAGGAGCATCTTTGGCGATTGAGCTGGCAGTGGCCGGGGGGTTTGCGCCTAGGGGAATAAATCTTTGGGCACCTAGTGGTCATG

2 CATGACCACTAGGAGCATCTTTGGCGAACTGCAGGTGTTGCGGTGGTATGTGAATCCCTAGGGGAATAAATCTTTGGGCACCTAGTGGTCATG

2 CATGACCACTAGGAGCATCTTTGGCGAGCCATGCTTGGGAGGTTGCTCCACCAGATCCTAGGGGAATAAATCTTTGGGCACCTAGTGGTCATG

2 CATGACCACTAGGAGCATCTTTGGCGAGGAACACAGGGCGATGCGGCAATGCCGAGACTAGGGGAATAAATCTTTGGGCACCTAGTGGTCATG

2 CATGACCACTAGGAGCATCTTTGGCGACGGTGTGGGGCGAGTCCTTCGCGGGAAGCTCTAGGGGAATAAATCTTTGGGCACCTAGTGGTCATG

2 CATGACCACTAGGAGCATCTTTGGCGAGATCGGGATAATCGGTGTCATTGGTGTCTCCTAGGGGAATAAATCTTTGGGCACCTAGTGGTCATG

2 CATGACCACTAGGAGCATCTTTGGCGACGAGTGGGGGGCAAGCAACGCGTTTGTCGACTAGGGGAATAAATCTTTGGGCACCTAGTGGTCATG

2 CATGACCACTAGGAGCATCTTTGGCGACCATGTCACGGCTAAGCAAATATTGTCGCTCTAGGGGAATAAATCTTTGGGCACCTAGTGGTCATG

2 CATGACCACTAGGAGCATCTTTGGCGACTGAGGAGCGAAGTCCTACCGTTAGTGGACCTAGGGGAATAAATCTTTGGGCACCTAGTGGTCATG

2 CATGACCACTAGGAGCATCTTTGGCGACTAGGGAAAATAACTGGCCTCGTCAGTCGACTAGGGGAATAAATCTTTGGGCACCTAGTGGTCATG

2 CATGACCACTAGGAGCATCTTTGGCGAAGGCATGTGTGGGACTCGAACCGAATCGATCTAGGGGAATAAATCTTTGGGCACCTAGTGGTCATG

2 CATGACCACTAGGAGCATCTTTGGCGAGTATGGTCGCGCGGCATTGCCTGTTCGAGCCTAGGGGAATAAATCTTTGGGCACCTAGTGGTCATG

2 CATGACCACTAGGAGCATCTTTGGCGAATGGCCGGCACGGCCTTCTAGTCCTCCGTACTAGGGGAATAAATCTTTGGGCACCTAGTGGTCATG

2 CATGACCACTAGGAGCATCTTTGGCGAGAAGACTCTGGATTCGGGGACCAGTTGGTACTAGGGGAATAAATCTTTGGGCACCTAGTGGTCATG

2 CATGACCACTAGGAGCATCTTTGGCGAACAGGCCGTGGCTGCGGGTGGGTGCGCCCCCTAGGGGAATAAATCTTTGGGCACCTAGTGGTCATG

2 CATGACCACTAGGAGCATCTTTGGCGAGATCGGGAGAATCGGTGGCATTTTTGTCTCCTAGGGGAATAAATCTTTGGGCACCTAGTGGTCATG

2 CATGACCACTAGGAGCATCTTTGGCGACACGGGTGGACCAGGTGAGAAAAGGGTCGACTAGGGGAATAAATCTTTGGGCACCTAGTGGTCATG

2 CATGACCACTAGGAGCATCTTTGGCGATCTGTACAAGGGCAGACACGAGCCGATCGACTAGGGGAATAAATCTTTGGGCACCTAGTGGTCATG

2 CATGACCACTAGGAGCATCTTTGGCGAACGGCAGGGGTTGAGGTGGTCTGTGAATCCCTAGGGGAATAAATCTTTGGGCACCTAGTGGTCATG

2 CATGACCACTAGGAGCATCTTTGGCGAGCCTTCGTATTCGTACGTGCTGTCTGGGTCCTAGGGGAATAAATCTTTGGGCACCTAGTGGTCATG

2 CATGACCACTAGGAGCATCTTTGGCGAGATCGCGAGAATCGGTGGCATCGGTGTCTCCTAGGGGAATAAATCTTTGGGCACCTAGTGGTCATG

2 CATGACCACTAGGAGCATCTTTGGCGACACAGACATAAGTATGCGAGTGGTGGCGTCCTAGGGGAATAAATCTTTGGGCACCTAGTGGTCATG

2 CATGACCACTAGGAGCATCTTTGGCGACGGGGCAAGCCGGCGATCATCTTGCGTATCCTAGGGGAATAAATCTTTGGGCACCTAGTGGTCATG

2 CATGACCACTAGGAGCATCTTTGGCGAGCGATGGCTGGTAGGTAGTATTCTGTGAGGCTAGGGGAATAAATCTTTGGGCACCTAGTGGTCATG

2 CATGACCACTAGGAGCATCTTTGGCGACCCAGACATGCGTGCACGCATCGGTGTGGCCTAGGGGAATAAATCTTTGGGCACCTAGTGGTCATG

2 CATGACCACTAGGAGCATCTTTGGCGAAAGGTGTCTAGCAGACACCGGGCATTCGTGCTAGGGGAATAAATCTTTGGGCACCTAGTGGTCATG

2 CATGACCACTAGGAGCATCTTTGGCGAACTAGCACGAAGGTCGCGTAATGTAGTCGACTAGGGGAATAAATCTTTGGGCACCTAGTGGTCATG

2 CATGACCACTAGGAGCATCTTTGGCGACTAGGGTCCCGGGGATGCAGCACGAGTCGACTAGGGGAATAAATCTTTGGGCACCTAGTGGTCATG

2 CATGACCACTAGGAGCATCTTTGGCGAACCGGACGGAGCGAGCCGGCTGAGGTGGGCCTAGGGGAATAAATCTTTGGGCACCTAGTGGTCATG

2 CATGACCACTAGGAGCATCTTTGGCGATTGGCACAACGCGTGTAAGGTGGCCGAGCTCTAGGGGAATAAATCTTTGGGCACCTAGTGGTCATG

2 CATGACCACTAGGAGCATCTTTGGCGAGATCGGGAGTATCGGTGGCAGTGGTGTCTCCTAGGGGAATAAATCTTTGGGCACCTAGTGGTCATG

2 CATGACCACTAGGAGCATCTTTGGCGAATAGAACCAACAGATTGATTAGATGAGCCGCTAGGGGAATAAATCTTTGGGCACCTAGTGGTCATG

2 CATGACCACTAGGAGCATCTTTGGCGAGCGTAGTGACATAGAGGGTCTGGGTCGAGACTAGGGGAATAAATCTTTGGGCACCTAGTGGTCATG

2 CATGACCACTAGGAGCATCTTTGGCGAGGTCAAGGATGCGGAGGGTGGTCGAGCCTCCTAGGGGAATAAATCTTTGGGCACCTAGTGGTCATG

2 CATGACCACTAGGAGCATCTTTGGCGAAAGGCAGGTGTTGCGGTGGTCTGTGAATACCTAGGGGAATAAATCTTTGGGCACCTAGTGGTCATG

2 CATGACCACTAGGAGCATCTTTGGCGAGGACTGCTTGTGAGACCTGGTTGCTGGTCGCTAGGGGAATAAATCTTTGGGCACCTAGTGGTCATG

2 CATGACCACTAGGAGCATCTTTGGCGAACAGAGCGGCGGGACGCGTGCAATGGTCGACTAGGGGAATAAATCTTTGGGCACCTAGTGGTCATG

2 CATGACCACTAGGAGCATCTTTGGCGATCCTCGTCAGATGGTGAAGCAGAAGTTTGGCTAGGGGAATAAATCTTTGGGCACCTAGTGGTCATG

2 CATGACCACTAGGAGCATCTTTGGCGACAGAAGGGCGTAACGAAAGGTGGTCGCCAGCTAGGGGAATAAATCTTTGGGCACCTAGTGGTCATG

2 CATGACCACTAGGAGCATCTTTGGCGACGAGGGGCGCAGTGGAACAAGGAAGGATGCCTAGGGGAATAAATCTTTGGGCACCTAGTGGTCATG

2 CATGACCACTAGGAGCATCTTTGGCGACGTGGGCATTGGAGTACTAGTGAAAGTCGACTAGGGGAATAAATCTTTGGGCACCTAGTGGTCATG

2 CATGACCACTAGGAGCATCTTTGGCGACGGTGACAGGTGATCAGGCGAGGACTCAGGCTAGGGGAATAAATCTTTGGGCACCTAGTGGTCATG

2 CATGACCACTAGGAGCATCTTTGGCGACCCAGCTGACGTAGTCTGCACGTGGTGGAGCTAGGGGAATAAATCTTTGGGCACCTAGTGGTCATG

2 CATGACCACTAGGAGCATCTTTGGCGAGATCGGGAGAATCGTTGGCATTGGTGACTCCTAGGGGAATAAATCTTTGGGCACCTAGTGGTCATG

2 CATGACCACTAGGAGCATCTTTGGCGACGAAGAGTGGGCAACGACAGTGAGCGGAGACTAGGGGAATAAATCTTTGGGCACCTAGTGGTCATG

2 CATGACCACTAGGAGCATCTTTGGCGAACTATCGGAGAAGAACGATGAAATAGGCGACTAGGGGAATAAATCTTTGGGCACCTAGTGGTCATG

2 CATGACCACTAGGAGCATCTTTGGCGAGATCGAGAGAATCGGTGGCATTGGTGCCTCCTAGGGGAATAAATCTTTGGGCACCTAGTGGTCATG

2 CATGACCACTAGGAGCATCTTTGGCGATCGGGTGCAGCCGGCAGAACCCGGATTCGACTAGGGGAATAAATCTTTGGGCACCTAGTGGTCATG

2 CATGACCACTAGGAGCATCTTTGGCGAGATCGGGAGAATCTGTGGCATAGGTGTCTCCTAGGGGAATAAATCTTTGGGCACCTAGTGGTCATG

2 CATGACCACTAGGAGCATCTTTGGCGAGTGTCAGGAGACAAATATGGTGCAACGAGACTAGGGGAATAAATCTTTGGGCACCTAGTGGTCATG

2 CATGACCACTAGGAGCATCTTTGGCGACGACATCCACAACACGTCAGGTGAGTCTAGCTAGGGGAATAAATCTTTGGGCACCTAGTGGTCATG

2 CATGACCACTAGGAGCATCTTTGGCGACTCGTGGGCAGGGAACATTGTAGGAGTCGACTAGGGGAATAAATCTTTGGGCACCTAGTGGTCATG

2 CATGACCACTAGGAGCATCTTTGGCGATCAAGCCGCCAGGGCGTACGAAGTGTGGTTCTAGGGGAATAAATCTTTGGGCACCTAGTGGTCATG

2 CATGACCACTAGGAGCATCTTTGGCGAAGCGGAGTTGCGATATAGTGGTTGAGCTACCTAGGGGAATAAATCTTTGGGCACCTAGTGGTCATG

2 CATGACCACTAGGAGCATCTTTGGCGAGGTCAATGTGATGTGATGTCTAGGTCGAGACTAGGGGAATAAATCTTTGGGCACCTAGTGGTCATG

2 CATGACCACTAGGAGCATCTTTGGCGATCCAGAGCGGCAGCTCGGAGTGGATAGACACTAGGGGAATAAATCTTTGGGCACCTAGTGGTCATG

2 CATGACCACTAGGAGCATCTTTGGCGACTACAGGGACTTAGATGTAAATAATGGCGCCTAGGGGAATAAATCTTTGGGCACCTAGTGGTCATG

2 CATGACCACTAGGAGCATCTTTGGCGAACTCGACGTTTGAGTGCTCATTAGAGGCGCCTAGGGGAATAAATCTTTGGGCACCTAGTGGTCATG

2 CATGACCACTAGGAGCATCTTTGGCGACCCGGCGGCTCATAGGGCTGCCGCGGTGGACTAGGGGAATAAATCTTTGGGCACCTAGTGGTCATG

2 CATGACCACTAGGAGCATCTTTGGCGACGTTCTAGCTAAGCGAATTGCTACGTCCGACTAGGGGAATAAATCTTTGGGCACCTAGTGGTCATG

2 CATGACCACTAGGAGCATCTTTGGCGACTACCGGGGCAACTCTTGCTCCGGAGTGGACTAGGGGAATAAATCTTTGGGCACCTAGTGGTCATG

2 CATGACCACTAGGAGCATCTTTGGCGAGCCGGGTGATGCCTCGTTTCGGATTTCCGACTAGGGGAATAAATCTTTGGGCACCTAGTGGTCATG

2 CATGACCACTAGGAGCATCTTTGGCGACAGTGGGCTGGTCGAGAGTAGGTCGTCGCTCTAGGGGAATAAATCTTTGGGCACCTAGTGGTCATG

2 CATGACCACTAGGAGCATCTTTGGCGATCCTCGTCAGATTGTGAAGCAGACGTTTGGCTAGGGGAATAAATCTTTGGGCACCTAGTGGTCATG

2 CATGACCACTAGGAGCATCTTTGGCGAGATGGTGTACGCCGTCGAGCAGACGTTGGGCTAGGGGAATAAATCTTTGGGCACCTAGTGGTCATG

2 CATGACCACTAGGAGCATCTTTGGCGACTTACGTGGGCGTGATAAATGGGCAAGGAGCTAGGGGAATAAATCTTTGGGCACCTAGTGGTCATG

2 CATGACCACTAGGAGCATCTTTGGCGACGCTCGTGGTCAATCCACGCCGAAGTGGAGCTAGGGGAATAAATCTTTGGGCACCTAGTGGTCATG

2 CATGACCACTAGGAGCATCTTTGGCGACAAGTGGAAGGCATGATGTAAGAAATGTCGCTAGGGGAATAAATCTTTGGGCACCTAGTGGTCATG

2 CATGACCACTAGGAGCATCTTTGGCGACCGCGGTAACACTGTTGCCGGGCGTGGAGACTAGGGGAATAAATCTTTGGGCACCTAGTGGTCATG

2 CATGACCACTAGGAGCATCTTTGGCGACCACGAAGGTACAAATAGCCAAGGGTACGACTAGGGGAATAAATCTTTGGGCACCTAGTGGTCATG

2 CATGACCACTAGGAGCATCTTTGGCGAAATATTCGGCGTTAACAGCGCCAAGTGGAGCTAGGGGAATAAATCTTTGGGCACCTAGTGGTCATG

2 CATGACCACTAGGAGCATCTTTGGCGAACGGCAGGTGTTGCGGTGGTCTGTGAATAACTAGGGGAATAAATCTTTGGGCACCTAGTGGTCATG

2 CATGACCACTAGGAGCATCTTTGGCGACACGACGAGGACTGCACAAGGTAGTAGAGACTAGGGGAATAAATCTTTGGGCACCTAGTGGTCATG

2 CATGACCACTAGGAGCATCTTTGGCGAGGCGAGTCCAGCGGCCTTCTTGGTCTGCGACTAGGGGAATAAATCTTTGGGCACCTAGTGGTCATG

2 CATGACCACTAGGAGCATCTTTGGCGACATGTACACCACCTTTGCCTGGAGCGTAGGCTAGGGGAATAAATCTTTGGGCACCTAGTGGTCATG

2 CATGACCACTAGGAGCATCTTTGGCGACATAGTGGACAGTTCACGCACGTTGGAGATCTAGGGGAATAAATCTTTGGGCACCTAGTGGTCATG

2 CATGACCACTAGGAGCATCTTTGGCGACCCAAGTAGCGACTGCTACACTGTGGTGGACTAGGGGAATAAATCTTTGGGCACCTAGTGGTCATG

2 CATGACCACTAGGAGCATCTTTGGCGATAAGCGGCAACGTGCAGAATCGAAATTCGCCTAGGGGAATAAATCTTTGGGCACCTAGTGGTCATG

2 CATGACCACTAGGAGCATCTTTGGCGACGGGGGTCTCACTGTACACGGCAAGTCGCGCTAGGGGAATAAATCTTTGGGCACCTAGTGGTCATG

2 CATGACCACTAGGAGCATCTTTGGCGACGTTGCGGAACATAGTTCTGCAGAAGTGGACTAGGGGAATAAATCTTTGGGCACCTAGTGGTCATG

2 CATGACCACTAGGAGCATCTTTGGCGACGTGGCATGGGAACTGAACCTCAACGGAGACTAGGGGAATAAATCTTTGGGCACCTAGTGGTCATG

2 CATGACCACTAGGAGCATCTTTGGCGAACATCCCCTCGAACGATGTGGGACGGTAGCCTAGGGGAATAAATCTTTGGGCACCTAGTGGTCATG

2 CATGACCACTAGGAGCATCTTTGGCGAAACGACCTCGTCGTTGGGGACCAGTTGCTGCTAGGGGAATAAATCTTTGGGCACCTAGTGGTCATG

2 CATGACCACTAGGAGCATCTTTGGCGAAGGGTGTGGGACTTCAAGTGGATCTCATAGCTAGGGGAATAAATCTTTGGGCACCTAGTGGTCATG

2 CATGACCACTAGGAGCATCTTTGGCGAGATCGTGAGAATCGTTGGCATTGGTGTCTCCTAGGGGAATAAATCTTTGGGCACCTAGTGGTCATG

2 CATGACCACTAGGAGCATCTTTGGCGAGGGAGTGGTATACCACCGGCGAACGCTGTCCTAGGGGAATAAATCTTTGGGCACCTAGTGGTCATG

2 CATGACCACTAGGAGCATCTTTGGCGAGATCGGGAGAATCGGTGGCATTGGAGTCACCTAGGGGAATAAATCTTTGGGCACCTAGTGGTCATG

2 CATGACCACTAGGAGCATCTTTGGCGAGGTTGCAGCTCGCGCGTAGGGTCGGCAGTGCTAGGGGAATAAATCTTTGGGCACCTAGTGGTCATG

2 CATGACCACTAGGAGCATCTTTGGCGAAGGGACTCTCTACCGACCGAACATCTGCGCCTAGGGGAATAAATCTTTGGGCACCTAGTGGTCATG

2 CATGACCACTAGGAGCATCTTTGGCGACCCAGGAGGAAGTTATTTCGAATGGGTGGACTAGGGGAATAAATCTTTGGGCACCTAGTGGTCATG

2 CATGACCACTAGGAGCATCTTTGGCGACCCAAACGGTGTAACCGTTGGAGTGTTGCTCTAGGGGAATAAATCTTTGGGCACCTAGTGGTCATG

2 CATGACCACTAGGAGCATCTTTGGCGACGACGGGGTCTATCGATCCCGATAGTGGAGCTAGGGGAATAAATCTTTGGGCACCTAGTGGTCATG

2 CATGACCACTAGGAGCATCTTTGGCGAAGGCGAAAGGGCATCCGCGGGCCGACGAGACTAGGGGAATAAATCTTTGGGCACCTAGTGGTCATG

2 CATGACCACTAGGAGCATCTTTGGCGACCCGAACTGCGTAGCAGGGCCGTGGTGGCTCTAGGGGAATAAATCTTTGGGCACCTAGTGGTCATG

2 CATGACCACTAGGAGCATCTTTGGCGACGGCGTGGGGCCATCTGTGCGGCGGATTCCCTAGGGGAATAAATCTTTGGGCACCTAGTGGTCATG

2 CATGACCACTAGGAGCATCTTTGGCGACTTAGTGGGGTAGGAGTTCATTGCGGTCGCCTAGGGGAATAAATCTTTGGGCACCTAGTGGTCATG

2 CATGACCACTAGGAGCATCTTTGGCGACACCATAAACGCAGTGGGATGGTCGTGGAGCTAGGGGAATAAATCTTTGGGCACCTAGTGGTCATG

2 CATGACCACTAGGAGCATCTTTGGCGAACGGCAGGTGTTGCGGTGGTCTATGGATCCCTAGGGGAATAAATCTTTGGGCACCTAGTGGTCATG

2 CATGACCACTAGGAGCATCTTTGGCGACAAGCCAGGACTGCGTGAGTGGCTGACTGCCTAGGGGAATAAATCTTTGGGCACCTAGTGGTCATG

2 CATGACCACTAGGAGCATCTTTGGCGACTCCGGTAGTGCTCGTACTACTGGTGTGGACTAGGGGAATAAATCTTTGGGCACCTAGTGGTCATG

2 CATGACCACTAGGAGCATCTTTGGCGACAGCGGCGTAGGCTGCCTGGGGTGGTGGCCCTAGGGGAATAAATCTTTGGGCACCTAGTGGTCATG

2 CATGACCACTAGGAGCATCTTTGGCGATCAGGCCGCCAGGTCGTACGAAGTGTGGTTCTAGGGGAATAAATCTTTGGGCACCTAGTGGTCATG

2 CATGACCACTAGGAGCATCTTTGGCGAGAAGACTCTGTATTCGGGGACCAGTTGCTCCTAGGGGAATAAATCTTTGGGCACCTAGTGGTCATG

2 CATGACCACTAGGAGCATCTTTGGCGACCCGGGGGCAGAATGCACTACCGAGGTGGACTAGGGGAATAAATCTTTGGGCACCTAGTGGTCATG

2 CATGACCACTAGGAGCATCTTTGGCGAGAAGTGCGTCTTAGATGGTTGTGGTCGAGACTAGGGGAATAAATCTTTGGGCACCTAGTGGTCATG

2 CATGACCACTAGGAGCATCTTTGGCGACTGGCAGGTGTTGCGGTGGTCTGTGAATCCCTAGGGGAATAAATCTTTGGGCACCTAGTGGTCATG

2 CATGACCACTAGGAGCATCTTTGGCGAGGAGAAACACGTACGAAGATAGCACTCGCTCTAGGGGAATAAATCTTTGGGCACCTAGTGGTCATG

2 CATGACCACTAGGAGCATCTTTGGCGACTGAGTGGGCAACGCGTTAAATGGGGTAGACTAGGGGAATAAATCTTTGGGCACCTAGTGGTCATG

2 CATGACCACTAGGAGCATCTTTGGCGAAAGCGCAGGGCATGCGACCTTCAATTGGTCCTAGGGGAATAAATCTTTGGGCACCTAGTGGTCATG

2 CATGACCACTAGGAGCATCTTTGGCGAACGGCAGTTGTTGCGGAGGTCTGTGAATCCCTAGGGGAATAAATCTTTGGGCACCTAGTGGTCATG

2 CATGACCACTAGGAGCATCTTTGGCGATGGGTGCTGACGACCGTCTACACGGTTACTCTAGGGGAATAAATCTTTGGGCACCTAGTGGTCATG

2 CATGACCACTAGGAGCATCTTTGGCGACCCGATAGTAAATAATACATAGCGGGTGGACTAGGGGAATAAATCTTTGGGCACCTAGTGGTCATG

2 CATGACCACTAGGAGCATCTTTGGCGAACGCGCAGGGCATGCGACCTCCAATTGGTCCTAGGGGAATAAATCTTTGGGCACCTAGTGGTCATG

2 CATGACCACTAGGAGCATCTTTGGCGATCCGGGGGAATTGCCACGGGTGTGGAGCGACTAGGGGAATAAATCTTTGGGCACCTAGTGGTCATG

2 CATGACCACTAGGAGCATCTTTGGCGATAGGCGTGTACGCTCGAGCGAAAGCGATAGCTAGGGGAATAAATCTTTGGGCACCTAGTGGTCATG

2 CATGACCACTAGGAGCATCTTTGGCGATCTGGCCGCCAGGGCGTACGAAGTGTGGTTCTAGGGGAATAAATCTTTGGGCACCTAGTGGTCATG

2 CATGACCACTAGGAGCATCTTTGGCGATTGGTTAGGTCTGGCACTGCCGTGTAGTCGCTAGGGGAATAAATCTTTGGGCACCTAGTGGTCATG

2 CATGACCACTAGGAGCATCTTTGGCGACCCTTGGCTGGTAGTGCGTACTCTGTCGAACTAGGGGAATAAATCTTTGGGCACCTAGTGGTCATG

2 CATGACCACTAGGAGCATCTTTGGCGAACCAAGCGCAGTGCGGCTCCAGGGGGTCGACTAGGGGAATAAATCTTTGGGCACCTAGTGGTCATG

2 CATGACCACTAGGAGCATCTTTGGCGACGTGGCTGGCAGGGTAAGTACTGTGTCGATCTAGGGGAATAAATCTTTGGGCACCTAGTGGTCATG

2 CATGACCACTAGGAGCATCTTTGGCGAACGTTGGGTCATGTAGGAAGAAAACTGAGACTAGGGGAATAAATCTTTGGGCACCTAGTGGTCATG

2 CATGACCACTAGGAGCATCTTTGGCGAGTAGTGCTGGCTAACTAAGTGTGGAACTCGCTAGGGGAATAAATCTTTGGGCACCTAGTGGTCATG

2 CATGACCACTAGGAGCATCTTTGGCGACCCGCCTCGTCATCGCGAGCGCGTGGTGGACTAGGGGAATAAATCTTTGGGCACCTAGTGGTCATG

2 CATGACCACTAGGAGCATCTTTGGCGAGAACGGGAGAATCGGTGGCATTGGTGACTCCTAGGGGAATAAATCTTTGGGCACCTAGTGGTCATG

2 CATGACCACTAGGAGCATCTTTGGCGACCCTGGACGAGGTGACTCGCGGAGGGTGGACTAGGGGAATAAATCTTTGGGCACCTAGTGGTCATG

2 CATGACCACTAGGAGCATCTTTGGCGACAGGGCGTGGGACACAAGTCGCGGAGGCGCCTAGGGGAATAAATCTTTGGGCACCTAGTGGTCATG

2 CATGACCACTAGGAGCATCTTTGGCGACACACAATAAGGACGGTCTGGGCGATGTCGCTAGGGGAATAAATCTTTGGGCACCTAGTGGTCATG

2 CATGACCACTAGGAGCATCTTTGGCGATACGTAACGGCCAGTTCCGGCAACGAGCGACTAGGGGAATAAATCTTTGGGCACCTAGTGGTCATG

2 CATGACCACTAGGAGCATCTTTGGCGAGAGGAACAATGGCGCCGGGCTACTCGAAGACTAGGGGAATAAATCTTTGGGCACCTAGTGGTCATG

2 CATGACCACTAGGAGCATCTTTGGCGACCGAGCGGCATACCTGACGAGGAAGTGGAGCTAGGGGAATAAATCTTTGGGCACCTAGTGGTCATG

2 CATGACCACTAGGAGCATCTTTGGCGACCACGTTTATCTAAGATATAGTGTGGTGGACTAGGGGAATAAATCTTTGGGCACCTAGTGGTCATG

2 CATGACCACTAGGAGCATCTTTGGCGAAGGCCTCAGAAGCGTCAACAATGCCGCAGACTAGGGGAATAAATCTTTGGGCACCTAGTGGTCATG

2 CATGACCACTAGGAGCATCTTTGGCGACGCAAACAGACGGACGTCGGTTGTCGTGGACTAGGGGAATAAATCTTTGGGCACCTAGTGGTCATG

2 CATGACCACTAGGAGCATCTTTGGCGAGTGGTGTAGTACAGGTGGGTCTTGTGGTGGCTAGGGGAATAAATCTTTGGGCACCTAGTGGTCATG

2 CATGACCACTAGGAGCATCTTTGGCGACGAGGCTTAGGGGCCGAGAAGTGTTTGGCTCTAGGGGAATAAATCTTTGGGCACCTAGTGGTCATG

2 CATGACCACTAGGAGCATCTTTGGCGAGTGATTAAGTCGGGTCCCCCGCGTCCTCGACTAGGGGAATAAATCTTTGGGCACCTAGTGGTCATG

2 CATGACCACTAGGAGCATCTTTGGCGAGCAGGAACTGGCTTCGACGTGGCTGAGCGACTAGGGGAATAAATCTTTGGGCACCTAGTGGTCATG

2 CATGACCACTAGGAGCATCTTTGGCGATCCGTCAGGGTGGCGACTTGTACGACAAGACTAGGGGAATAAATCTTTGGGCACCTAGTGGTCATG

2 CATGACCACTAGGAGCATCTTTGGCGAAGCGCGGCCGTGACGCATCCCGAAGTGGATCTAGGGGAATAAATCTTTGGGCACCTAGTGGTCATG

2 CATGACCACTAGGAGCATCTTTGGCGACGTGGGAATTGCCGGGAAACAAGACGTCGACTAGGGGAATAAATCTTTGGGCACCTAGTGGTCATG

2 CATGACCACTAGGAGCATCTTTGGCGAATGGCCGGCACGGCCTTCTAGTCCTCGCTACTAGGGGAATAAATCTTTGGGCACCTAGTGGTCATG

2 CATGACCACTAGGAGCATCTTTGGCGATAGGCGTGTACGGTTGAGCGAAAGCGATAGCTAGGGGAATAAATCTTTGGGCACCTAGTGGTCATG

2 CATGACCACTAGGAGCATCTTTGGCGACCCACGGTAAGGCAGTGTACGTGTGGTGGACTAGGGGAATAAATCTTTGGGCACCTAGTGGTCATG

2 CATGACCACTAGGAGCATCTTTGGCGAGCGGGGTTGCTTCGTGTGTAGTACCGTCTACTAGGGGAATAAATCTTTGGGCACCTAGTGGTCATG

2 CATGACCACTAGGAGCATCTTTGGCGAAGTGCGGTGGGCATGTGGGAGGTAGCGACGCTAGGGGAATAAATCTTTGGGCACCTAGTGGTCATG

2 CATGACCACTAGGAGCATCTTTGGCGACGGTGTGGGGCTATTGATGCGGCGGTACTCCTAGGGGAATAAATCTTTGGGCACCTAGTGGTCATG

2 CATGACCACTAGGAGCATCTTTGGCGACGGCGTGGGGGTCGAATCCGGCGGTGATCTCTAGGGGAATAAATCTTTGGGCACCTAGTGGTCATG

2 CATGACCACTAGGAGCATCTTTGGCGAAGGGTGTAGGACTTCAAGCGGATCTCATAGCTAGGGGAATAAATCTTTGGGCACCTAGTGGTCATG

2 CATGACCACTAGGAGCATCTTTGGCGATCGTAGGAAAACAAAAGTGGCCTTGCTAGCCTAGGGGAATAAATCTTTGGGCACCTAGTGGTCATG

2 CATGACCACTAGGAGCATCTTTGGCGACATGGGCGATGCCCGGCGAGGAAACGTCACCTAGGGGAATAAATCTTTGGGCACCTAGTGGTCATG

2 CATGACCACTAGGAGCATCTTTGGCGAGTGGCGTCGCGCGGTTGGTAGAGGAACTCGCTAGGGGAATAAATCTTTGGGCACCTAGTGGTCATG

2 CATGACCACTAGGAGCATCTTTGGCGATCAGGCCACCAGGGCGTACGAAGTGTGGTTCTAGGGGAATAAATCTTTGGGCACCTAGTGGTCATG

2 CATGACCACTAGGAGCATCTTTGGCGATACGCAGTAGCCGGAGTGGTCGAGTGTAGCCTAGGGGAATAAATCTTTGGGCACCTAGTGGTCATG

2 CATGACCACTAGGAGCATCTTTGGCGACCCGGGTGCGAGGAAGCTCAGAAGCGGCGACTAGGGGAATAAATCTTTGGGCACCTAGTGGTCATG

2 CATGACCACTAGGAGCATCTTTGGCGAGTGAAGCGGGCCGGCGAGCGACAACGAAGCCTAGGGGAATAAATCTTTGGGCACCTAGTGGTCATG

2 CATGACCACTAGGAGCATCTTTGGCGACCCACTTGTAGGAGAACAAGTGGAGTGAGACTAGGGGAATAAATCTTTGGGCACCTAGTGGTCATG

2 CATGACCACTAGGAGCATCTTTGGCGACGAGGTCGGTAGGCAAGTGTTCATAGGAAGCTAGGGGAATAAATCTTTGGGCACCTAGTGGTCATG

2 CATGACCACTAGGAGCATCTTTGGCGAGGGAGGGCGCCGGCAGCGGTGTGAATGCCCCTAGGGGAATAAATCTTTGGGCACCTAGTGGTCATG

2 CATGACCACTAGGAGCATCTTTGGCGACAGGGTAACAGGAGAGGACAACAACTGCGCCTAGGGGAATAAATCTTTGGGCACCTAGTGGTCATG

2 CATGACCACTAGGAGCATCTTTGGCGAAGGGCGAGGGTAAAGACCCCGAGGAGATGTCTAGGGGAATAAATCTTTGGGCACCTAGTGGTCATG

2 CATGACCACTAGGAGCATCTTTGGCGAGGGAGGGCGTCGGCAGCGGTGTGAATGCGCCTAGGGGAATAAATCTTTGGGCACCTAGTGGTCATG

2 CATGACCACTAGGAGCATCTTTGGCGACCCGCGTGAATGACAGAACGCTAGGGTGGACTAGGGGAATAAATCTTTGGGCACCTAGTGGTCATG

2 CATGACCACTAGGAGCATCTTTGGCGACCCACTGCGGGGAACACGTAAGGGGGTGGACTAGGGGAATAAATCTTTGGGCACCTAGTGGTCATG

2 CATGACCACTAGGAGCATCTTTGGCGATGAGGCCGTGCTATCTGCACACTCCGCGGGCTAGGGGAATAAATCTTTGGGCACCTAGTGGTCATG

2 CATGACCACTAGGAGCATCTTTGGCGACCCGTCAGGTGTGGAGTCAATCGCTGTCGACTAGGGGAATAAATCTTTGGGCACCTAGTGGTCATG

2 CATGACCACTAGGAGCATCTTTGGCGAGAATAATCTGGATTCGGGGACCAGTTGCTGCTAGGGGAATAAATCTTTGGGCACCTAGTGGTCATG

2 CATGACCACTAGGAGCATCTTTGGCGAAGTCTGGGGGATGGCCGGGAAGAAAGTCGACTAGGGGAATAAATCTTTGGGCACCTAGTGGTCATG

2 CATGACCACTAGGAGCATCTTTGGCGACGGCGTGGGGCCATTGATGCGGCGGCACTCCTAGGGGAATAAATCTTTGGGCACCTAGTGGTCATG

2 CATGACCACTAGGAGCATCTTTGGCGATGGTTGCTGACGGCCGCCGCTGCGGCTACACTAGGGGAATAAATCTTTGGGCACCTAGTGGTCATG

2 CATGACCACTAGGAGCATCTTTGGCGACATCTAAGCCTATCATATAGGAATGTTGCTCTAGGGGAATAAATCTTTGGGCACCTAGTGGTCATG

2 CATGACCACTAGGAGCATCTTTGGCGACAACTCCAGCGGCGGGCGGTCGGTGGTCGACTAGGGGAATAAATCTTTGGGCACCTAGTGGTCATG

2 CATGACCACTAGGAGCATCTTTGGCGAGAGCAAGTCTCCGGGACTTCACGAGTAGTGCTAGGGGAATAAATCTTTGGGCACCTAGTGGTCATG

2 CATGACCACTAGGAGCATCTTTGGCGAGACAACGGAACGTCAGAGGTTAGACTCGCACTAGGGGAATAAATCTTTGGGCACCTAGTGGTCATG

2 CATGACCACTAGGAGCATCTTTGGCGAGCTGGAAGATAGAGGAACAACTGCGCTCGACTAGGGGAATAAATCTTTGGGCACCTAGTGGTCATG

2 CATGACCACTAGGAGCATCTTTGGCGACGGCGTGGGGCCTAAGTGCGGCGGCTGATTCTAGGGGAATAAATCTTTGGGCACCTAGTGGTCATG

2 CATGACCACTAGGAGCATCTTTGGCGAGGCCTAAGGTGTACCGTAGAAGGAGCGGGCCTAGGGGAATAAATCTTTGGGCACCTAGTGGTCATG

2 CATGACCACTAGGAGCATCTTTGGCGAAAAGGTACGCGCTCTACACATGCTTGCAGCCTAGGGGAATAAATCTTTGGGCACCTAGTGGTCATG

2 CATGACCACTAGGAGCATCTTTGGCGAAGGGTGTAGGACTTCAAGTGCATCTCATAGCTAGGGGAATAAATCTTTGGGCACCTAGTGGTCATG

2 CATGACCACTAGGAGCATCTTTGGCGAATGGCTAGCACGGCCTTCTAGTCCTCGGTACTAGGGGAATAAATCTTTGGGCACCTAGTGGTCATG

2 CATGACCACTAGGAGCATCTTTGGCGAGATCGGGAGAATCGGAGGCATAGGTGTCTCCTAGGGGAATAAATCTTTGGGCACCTAGTGGTCATG

2 CATGACCACTAGGAGCATCTTTGGCGAGTAGTGGCGTAGGACGGGAGCGGGGCGAGCCTAGGGGAATAAATCTTTGGGCACCTAGTGGTCATG

2 CATGACCACTAGGAGCATCTTTGGCGAGATCGGGGGAATCGGTGGCATCGGTGTCTCCTAGGGGAATAAATCTTTGGGCACCTAGTGGTCATG

2 CATGACCACTAGGAGCATCTTTGGCGACATCGTGGCTAGCTATAGATTCTGTGGAGACTAGGGGAATAAATCTTTGGGCACCTAGTGGTCATG

2 CATGACCACTAGGAGCATCTTTGGCGAAAGTCGGTAGAACAGGGTGGGGTGCTGGCCCTAGGGGAATAAATCTTTGGGCACCTAGTGGTCATG

2 CATGACCACTAGGAGCATCTTTGGCGACGTTGGCTGGTAGGCGATCGTATTAAGTGCCTAGGGGAATAAATCTTTGGGCACCTAGTGGTCATG

2 CATGACCACTAGGAGCATCTTTGGCGATCAGTGGTCGAGCCTAGGGAAGGACGCCAGCTAGGGGAATAAATCTTTGGGCACCTAGTGGTCATG

2 CATGACCACTAGGAGCATCTTTGGCGAGGTGGTCATAGGTCTCGACCGGGCTCGAGGCTAGGGGAATAAATCTTTGGGCACCTAGTGGTCATG

2 CATGACCACTAGGAGCATCTTTGGCGACCCGACAAGGACTATGTATCGACGGGTGGACTAGGGGAATAAATCTTTGGGCACCTAGTGGTCATG

2 CATGACCACTAGGAGCATCTTTGGCGACCCACGGGGTGGAGTGACTCCAATGGGCGACTAGGGGAATAAATCTTTGGGCACCTAGTGGTCATG

2 CATGACCACTAGGAGCATCTTTGGCGATTCAGAGTTGGTAGGGTAAAGTGCTGGATCCTAGGGGAATAAATCTTTGGGCACCTAGTGGTCATG

2 CATGACCACTAGGAGCATCTTTGGCGAAGGGTGTAAGACTTCAAGTGGATCTCATAGCTAGGGGAATAAATCTTTGGGCACCTAGTGGTCATG

2 CATGACCACTAGGAGCATCTTTGGCGACCGAGTGGGCTGGGAGGAAGTATTTCGTAGCTAGGGGAATAAATCTTTGGGCACCTAGTGGTCATG

2 CATGACCACTAGGAGCATCTTTGGCGAACGAGTGGGGCAGGAAGTCTCCACTGGAAGCTAGGGGAATAAATCTTTGGGCACCTAGTGGTCATG

2 CATGACCACTAGGAGCATCTTTGGCGAGATACAAGGTCTGCAAGGTTGGCTCGAGTCCTAGGGGAATAAATCTTTGGGCACCTAGTGGTCATG

2 CATGACCACTAGGAGCATCTTTGGCGAGGCTGACTAGACCGGGGGCATTGTTGTCTACTAGGGGAATAAATCTTTGGGCACCTAGTGGTCATG

2 CATGACCACTAGGAGCATCTTTGGCGACCCGAGCCCAGGTAGGGCCTCAAGGGTGGACTAGGGGAATAAATCTTTGGGCACCTAGTGGTCATG

2 CATGACCACTAGGAGCATCTTTGGCGAGCGTGGTCATCAGGGCAGAACACGAGGAGACTAGGGGAATAAATCTTTGGGCACCTAGTGGTCATG

2 CATGACCACTAGGAGCATCTTTGGCGACACTAAGTGTAAGCGATGTGTGAAGTGGAGCTAGGGGAATAAATCTTTGGGCACCTAGTGGTCATG

2 CATGACCACTAGGAGCATCTTTGGCGAGTGCGTAGCGCGCTTGCCTGTGGCCGAGCTCTAGGGGAATAAATCTTTGGGCACCTAGTGGTCATG

2 CATGACCACTAGGAGCATCTTTGGCGAGATCGGGAGAATCGGTGTCATAGGTGTCTCCTAGGGGAATAAATCTTTGGGCACCTAGTGGTCATG

2 CATGACCACTAGGAGCATCTTTGGCGAAGCGTGTTGCAAGGTCCGCGAGTGTGGAACCTAGGGGAATAAATCTTTGGGCACCTAGTGGTCATG

2 CATGACCACTAGGAGCATCTTTGGCGACAACCGCTTACGTCCGTCAAGCGGTGTGGACTAGGGGAATAAATCTTTGGGCACCTAGTGGTCATG

2 CATGACCACTAGGAGCATCTTTGGCGACGCGGTGGTCATCTGACCAAAGGGATTCGCCTAGGGGAATAAATCTTTGGGCACCTAGTGGTCATG

2 CATGACCACTAGGAGCATCTTTGGCGAAGGGGTCTGTCGGGCCGGAGTGCTCCAACGCTAGGGGAATAAATCTTTGGGCACCTAGTGGTCATG

2 CATGACCACTAGGAGCATCTTTGGCGATAGCAACAAGGATGGGCAGGTAAATATCGACTAGGGGAATAAATCTTTGGGCACCTAGTGGTCATG

2 CATGACCACTAGGAGCATCTTTGGCGAGACAGTGAGCAACACCGGGGCAGTCTCGAACTAGGGGAATAAATCTTTGGGCACCTAGTGGTCATG

2 CATGACCACTAGGAGCATCTTTGGCGAGCCTGTTCGAACACTGATGCGACGAGCCTGCTAGGGGAATAAATCTTTGGGCACCTAGTGGTCATG

2 CATGACCACTAGGAGCATCTTTGGCGAGTATTGGCTGGTAGGTTGCGGATTGGGGAGCTAGGGGAATAAATCTTTGGGCACCTAGTGGTCATG

2 CATGACCACTAGGAGCATCTTTGGCGAATGGCAGGTGTTGCGGTGGTCTGCGAATCCCTAGGGGAATAAATCTTTGGGCACCTAGTGGTCATG

2 CATGACCACTAGGAGCATCTTTGGCGATTGTGGTGTGGGAGGGTGGGCTAGACGCCGCTAGGGGAATAAATCTTTGGGCACCTAGTGGTCATG

2 CATGACCACTAGGAGCATCTTTGGCGACCCGGATGAGAGTATCGAATCGTGGTGGAGCTAGGGGAATAAATCTTTGGGCACCTAGTGGTCATG

2 CATGACCACTAGGAGCATCTTTGGCGATCCACTCGGACTAGATCATGGGGTAAGCGACTAGGGGAATAAATCTTTGGGCACCTAGTGGTCATG

2 CATGACCACTAGGAGCATCTTTGGCGACGTGATTTAAGCCGGGCTGAGAGGAGCCAGCTAGGGGAATAAATCTTTGGGCACCTAGTGGTCATG

2 CATGACCACTAGGAGCATCTTTGGCGACACCAGACGGGTGCATATCAGAGCTGTCGACTAGGGGAATAAATCTTTGGGCACCTAGTGGTCATG

2 CATGACCACTAGGAGCATCTTTGGCGAGCCTCGCTTGGGAGCTTGCTCCACCAGTTCCTAGGGGAATAAATCTTTGGGCACCTAGTGGTCATG

2 CATGACCACTAGGAGCATCTTTGGCGACGTCGTGGTCTCATGTAAGCGGTAGGAGCTCTAGGGGAATAAATCTTTGGGCACCTAGTGGTCATG

2 CATGACCACTAGGAGCATCTTTGGCGAGATCGGTAGAATCGGTTGCATTGGTGTCTCCTAGGGGAATAAATCTTTGGGCACCTAGTGGTCATG

2 CATGACCACTAGGAGCATCTTTGGCGACAATGAGGCGAATGCCTTAAAAGATGTTGCCTAGGGGAATAAATCTTTGGGCACCTAGTGGTCATG

2 CATGACCACTAGGAGCATCTTTGGCGACTTTCGGGGGTCATGATAAGTCCGAGTCGACTAGGGGAATAAATCTTTGGGCACCTAGTGGTCATG

2 CATGACCACTAGGAGCATCTTTGGCGACGCGTTGCGTGGTCGAGGTACTCTTGCGTCCTAGGGGAATAAATCTTTGGGCACCTAGTGGTCATG

2 CATGACCACTAGGAGCATCTTTGGCGAAGTGCGGTGGGCATGTGGGTGGCAGCGACGCTAGGGGAATAAATCTTTGGGCACCTAGTGGTCATG

2 CATGACCACTAGGAGCATCTTTGGCGAAGACGACACAGTCGAGCGGGTTAGAGGAAGCTAGGGGAATAAATCTTTGGGCACCTAGTGGTCATG

2 CATGACCACTAGGAGCATCTTTGGCGACATGAGTGGGCAGGGCTGGTTGGTCGTCGACTAGGGGAATAAATCTTTGGGCACCTAGTGGTCATG

2 CATGACCACTAGGAGCATCTTTGGCGACATGGCGAAAGAGATGTGGTTGAGCATCGCCTAGGGGAATAAATCTTTGGGCACCTAGTGGTCATG

2 CATGACCACTAGGAGCATCTTTGGCGACTGGTGGTTGCGCAAACCACTGAAGTGGAGCTAGGGGAATAAATCTTTGGGCACCTAGTGGTCATG

2 CATGACCACTAGGAGCATCTTTGGCGACACCAATCATTGGTTGGACGAAGTGGACGACTAGGGGAATAAATCTTTGGGCACCTAGTGGTCATG

2 CATGACCACTAGGAGCATCTTTGGCGACCATGGGTGGGCTCAACTAAGGAAGGGCGACTAGGGGAATAAATCTTTGGGCACCTAGTGGTCATG

2 CATGACCACTAGGAGCATCTTTGGCGACCCATTGACGGATAGCGCTCAAGGGGTGGACTAGGGGAATAAATCTTTGGGCACCTAGTGGTCATG

2 CATGACCACTAGGAGCATCTTTGGCGAGTATTGGCTGGTAGGTTGCGTCTTGGGGAGCTAGGGGAATAAATCTTTGGGCACCTAGTGGTCATG

2 CATGACCACTAGGAGCATCTTTGGCGACGGCGGGTGTCTGAGAATGGAGCCCGACGCCTAGGGGAATAAATCTTTGGGCACCTAGTGGTCATG

2 CATGACCACTAGGAGCATCTTTGGCGAGATCGGGAGAATAGGTGGCATTGTTGTCTCCTAGGGGAATAAATCTTTGGGCACCTAGTGGTCATG

2 CATGACCACTAGGAGCATCTTTGGCGAGGGAGGGCGCCGGCAGCGGTGTAAATGCGCCTAGGGGAATAAATCTTTGGGCACCTAGTGGTCATG

2 CATGACCACTAGGAGCATCTTTGGCGACGGCGTGGGGGTCTCGATCCGGCGGAGGTCCTAGGGGAATAAATCTTTGGGCACCTAGTGGTCATG

2 CATGACCACTAGGAGCATCTTTGGCGACTCAAATCGGTCAATCCAATTTGTAGTGGACTAGGGGAATAAATCTTTGGGCACCTAGTGGTCATG

2 CATGACCACTAGGAGCATCTTTGGCGAGACGACCTTGTCGTTGGGGACCAGTTGCTGCTAGGGGAATAAATCTTTGGGCACCTAGTGGTCATG

2 CATGACCACTAGGAGCATCTTTGGCGACCCGCTGACTGAGGTCAGCGCGAGTGGAGACTAGGGGAATAAATCTTTGGGCACCTAGTGGTCATG

2 CATGACCACTAGGAGCATCTTTGGCGACGACGGGGGCAGGGGATAGTAGGGAGTCGACTAGGGGAATAAATCTTTGGGCACCTAGTGGTCATG

2 CATGACCACTAGGAGCATCTTTGGCGACGTCGGCTGGTAGGGACTGTATTGAGGAACCTAGGGGAATAAATCTTTGGGCACCTAGTGGTCATG

2 CATGACCACTAGGAGCATCTTTGGCGAATCTACGGGGCGGCGTCGCGCGGATTCGAACTAGGGGAATAAATCTTTGGGCACCTAGTGGTCATG

2 CATGACCACTAGGAGCATCTTTGGCGACGAGGTGGGAGGCTAAATGTGAGAAGGAGACTAGGGGAATAAATCTTTGGGCACCTAGTGGTCATG

2 CATGACCACTAGGAGCATCTTTGGCGAACGGCAGTTGTTGCTGTGGTCTGTGAATCCCTAGGGGAATAAATCTTTGGGCACCTAGTGGTCATG

2 CATGACCACTAGGAGCATCTTTGGCGAGGCGTGTCCTGGCGGGGGCCTTGAGGTCGACTAGGGGAATAAATCTTTGGGCACCTAGTGGTCATG

2 CATGACCACTAGGAGCATCTTTGGCGAACGGCAGGTGTTGCTGTGGTCTGTGAATCACTAGGGGAATAAATCTTTGGGCACCTAGTGGTCATG

2 CATGACCACTAGGAGCATCTTTGGCGACACCCGGCATGTGGAATGCTAAGGTGTGGACTAGGGGAATAAATCTTTGGGCACCTAGTGGTCATG

2 CATGACCACTAGGAGCATCTTTGGCGACGAGGAGTCATGGGTGCAGGGCAGAGTCGACTAGGGGAATAAATCTTTGGGCACCTAGTGGTCATG

2 CATGACCACTAGGAGCATCTTTGGCGAGGCTCATAAGTTATTTCCCGGGTCGAGACGCTAGGGGAATAAATCTTTGGGCACCTAGTGGTCATG

2 CATGACCACTAGGAGCATCTTTGGCGAGGTCGGGAGAATCGTTGGCATTGGTGTCTCCTAGGGGAATAAATCTTTGGGCACCTAGTGGTCATG

2 CATGACCACTAGGAGCATCTTTGGCGACCCGCGGAGTGAGATGAGCGGAGAGTGGAGCTAGGGGAATAAATCTTTGGGCACCTAGTGGTCATG

2 CATGACCACTAGGAGCATCTTTGGCGACAGTGGCTGCCTGGCAACAATACTGCTCGACTAGGGGAATAAATCTTTGGGCACCTAGTGGTCATG

2 CATGACCACTAGGAGCATCTTTGGCGAGGGAGGACGCCGGCAGCGGTGTGAATGCGCCTAGGGGAATAAATCTTTGGGCACCTAGTGGTCATG

2 CATGACCACTAGGAGCATCTTTGGCGAAAGGTGGGACAAGTAGGTCAAGGTACTCGCCTAGGGGAATAAATCTTTGGGCACCTAGTGGTCATG

2 CATGACCACTAGGAGCATCTTTGGCGACGCAGGGGGCAGCTATGAGGTAAAAGTCGACTAGGGGAATAAATCTTTGGGCACCTAGTGGTCATG

2 CATGACCACTAGGAGCATCTTTGGCGACGATGACTGAGGATTCTCGGACAGGAGCCGCTAGGGGAATAAATCTTTGGGCACCTAGTGGTCATG

2 CATGACCACTAGGAGCATCTTTGGCGACCGACCGGTGGGGTAGGATGCATAGGGAGACTAGGGGAATAAATCTTTGGGCACCTAGTGGTCATG

2 CATGACCACTAGGAGCATCTTTGGCGACTCGTGTAGGTTGCGTCGACCGCCCGGTGACTAGGGGAATAAATCTTTGGGCACCTAGTGGTCATG

2 CATGACCACTAGGAGCATCTTTGGCGACCGAACGACAGTGCGTTAAGTGGGGCAGATCTAGGGGAATAAATCTTTGGGCACCTAGTGGTCATG

2 CATGACCACTAGGAGCATCTTTGGCGATCCTCGTCAGATGGTGAAGCAGACGTTTAGCTAGGGGAATAAATCTTTGGGCACCTAGTGGTCATG

2 CATGACCACTAGGAGCATCTTTGGCGACCCCGCAGGGAGTGGGCTAGGTCTGTTCGCCTAGGGGAATAAATCTTTGGGCACCTAGTGGTCATG

2 CATGACCACTAGGAGCATCTTTGGCGAGTAGCGTTCAAAGTGGCTCCGTAGCTACCGCTAGGGGAATAAATCTTTGGGCACCTAGTGGTCATG

2 CATGACCACTAGGAGCATCTTTGGCGACTAGGCCGTGGCTGCGGGTGGGTGCGCACCCTAGGGGAATAAATCTTTGGGCACCTAGTGGTCATG

2 CATGACCACTAGGAGCATCTTTGGCGACAGCCGTAGCCGTAAGGGTGGTATAAGGCCCTAGGGGAATAAATCTTTGGGCACCTAGTGGTCATG

2 CATGACCACTAGGAGCATCTTTGGCGAATGAAAAAGATCTAGCCAATAAGTATTCGCCTAGGGGAATAAATCTTTGGGCACCTAGTGGTCATG

2 CATGACCACTAGGAGCATCTTTGGCGACGTCGTGGGGCGAGATACTAAGAGCGTCGACTAGGGGAATAAATCTTTGGGCACCTAGTGGTCATG

2 CATGACCACTAGGAGCATCTTTGGCGAGGGGGCGAGTGCGCGAGGTGTTATGGGGAGCTAGGGGAATAAATCTTTGGGCACCTAGTGGTCATG

2 CATGACCACTAGGAGCATCTTTGGCGACTCGGGGTGCAGCAGGCTCACCGTAGTGGACTAGGGGAATAAATCTTTGGGCACCTAGTGGTCATG

2 CATGACCACTAGGAGCATCTTTGGCGAGATCGGGAGAATCGCAGGCATTGGTGTCTCCTAGGGGAATAAATCTTTGGGCACCTAGTGGTCATG

2 CATGACCACTAGGAGCATCTTTGGCGACCCAGTTCGTCAAGTATCGAAGTGGGTGGACTAGGGGAATAAATCTTTGGGCACCTAGTGGTCATG

2 CATGACCACTAGGAGCATCTTTGGCGATCCCGGGGAAGCCATGCGAGCGGCGAGCGACTAGGGGAATAAATCTTTGGGCACCTAGTGGTCATG

2 CATGACCACTAGGAGCATCTTTGGCGACGACGGGGGCATGGGCGGGAGAGGAGTCGACTAGGGGAATAAATCTTTGGGCACCTAGTGGTCATG

2 CATGACCACTAGGAGCATCTTTGGCGACGGCGTGGGGCCTCTAGCGCGGCGGGGACCCTAGGGGAATAAATCTTTGGGCACCTAGTGGTCATG

2 CATGACCACTAGGAGCATCTTTGGCGACTGGGGTGGGCGTGGACCGGGAGACGTCGACTAGGGGAATAAATCTTTGGGCACCTAGTGGTCATG

2 CATGACCACTAGGAGCATCTTTGGCGACGGAGGGGGCATAGGTATTGCGAAGTTCGCCTAGGGGAATAAATCTTTGGGCACCTAGTGGTCATG

2 CATGACCACTAGGAGCATCTTTGGCGACCCATGAGGCTGAGGCTGTAATGTGGTGGACTAGGGGAATAAATCTTTGGGCACCTAGTGGTCATG

2 CATGACCACTAGGAGCATCTTTGGCGATACAGGTGGTCGGTGCTCGGGTGCCTGGCCCTAGGGGAATAAATCTTTGGGCACCTAGTGGTCATG

2 CATGACCACTAGGAGCATCTTTGGCGACTGGGGGTCGGACTGGTTAAGAATAGGAGCCTAGGGGAATAAATCTTTGGGCACCTAGTGGTCATG

2 CATGACCACTAGGAGCATCTTTGGCGATCCCCCTTGGCACGGGTCTTAATAGATTTGCTAGGGGAATAAATCTTTGGGCACCTAGTGGTCATG

2 CATGACCACTAGGAGCATCTTTGGCGACAAGACGTGGCCGGGCAATAGGAGGGTCGCCTAGGGGAATAAATCTTTGGGCACCTAGTGGTCATG

2 CATGACCACTAGGAGCATCTTTGGCGATAGCGAAGTGCTGTGAACAGTGGCTAGCACCTAGGGGAATAAATCTTTGGGCACCTAGTGGTCATG

2 CATGACCACTAGGAGCATCTTTGGCGATGACAGTGGCTGAGCATGAGCTATTAGCTCCTAGGGGAATAAATCTTTGGGCACCTAGTGGTCATG

2 CATGACCACTAGGAGCATCTTTGGCGAAACTCGGTAGAACAGGGTGGGGTGCTGTCCCTAGGGGAATAAATCTTTGGGCACCTAGTGGTCATG

2 CATGACCACTAGGAGCATCTTTGGCGAGGCAAAGAGGTGGTTATATGTGGTCGAGCTCTAGGGGAATAAATCTTTGGGCACCTAGTGGTCATG

2 CATGACCACTAGGAGCATCTTTGGCGAACGTCAGGTGTTGCGGTGGTCTGTGAATACCTAGGGGAATAAATCTTTGGGCACCTAGTGGTCATG

2 CATGACCACTAGGAGCATCTTTGGCGACCGACCGGATGCTTGGGAACAAGGAGGCGACTAGGGGAATAAATCTTTGGGCACCTAGTGGTCATG

2 CATGACCACTAGGAGCATCTTTGGCGAAGATCGCTCGTCGACTCGGGTTTGAGGAAGCTAGGGGAATAAATCTTTGGGCACCTAGTGGTCATG

2 CATGACCACTAGGAGCATCTTTGGCGATAGGCTCGATTGGGCACGGATGACATCGCTCTAGGGGAATAAATCTTTGGGCACCTAGTGGTCATG

2 CATGACCACTAGGAGCATCTTTGGCGACATGCGTGTTCCAGGGTCTCCGGGTGTCGACTAGGGGAATAAATCTTTGGGCACCTAGTGGTCATG

2 CATGACCACTAGGAGCATCTTTGGCGAGCAGCACAGCGCACAAGAAGAGAGCAGCTCCTAGGGGAATAAATCTTTGGGCACCTAGTGGTCATG

2 CATGACCACTAGGAGCATCTTTGGCGAGGCATCGGTGCCTGACGGGGATGGCGAGGACTAGGGGAATAAATCTTTGGGCACCTAGTGGTCATG

2 CATGACCACTAGGAGCATCTTTGGCGAATTTGGCTGGTAGGTCGCGTACTAGGTAGCCTAGGGGAATAAATCTTTGGGCACCTAGTGGTCATG

2 CATGACCACTAGGAGCATCTTTGGCGACTCCAGTTCATCTGTCGGGCAGTATAAGGACTAGGGGAATAAATCTTTGGGCACCTAGTGGTCATG

2 CATGACCACTAGGAGCATCTTTGGCGACGGTATGAAGTGGGATACGCATTGTCGCCGCTAGGGGAATAAATCTTTGGGCACCTAGTGGTCATG

2 CATGACCACTAGGAGCATCTTTGGCGACCCGCGCGGGCACGACAAGCGCGAGGTGGACTAGGGGAATAAATCTTTGGGCACCTAGTGGTCATG

2 CATGACCACTAGGAGCATCTTTGGCGAGGCGAAGCCATCGAAGATATGCTTCGGAGACTAGGGGAATAAATCTTTGGGCACCTAGTGGTCATG

2 CATGACCACTAGGAGCATCTTTGGCGAAGTTGAAGACGGTCAATAAGTGCTTCGCCGCTAGGGGAATAAATCTTTGGGCACCTAGTGGTCATG

2 CATGACCACTAGGAGCATCTTTGGCGAGCCATGGTGTGGGACTAGCGGAGATTCGACCTAGGGGAATAAATCTTTGGGCACCTAGTGGTCATG

2 CATGACCACTAGGAGCATCTTTGGCGAAGGGTTACAAGTGGATCTCGTGGTCGAAGCCTAGGGGAATAAATCTTTGGGCACCTAGTGGTCATG

2 CATGACCACTAGGAGCATCTTTGGCGAGAAGCACAAAGCAAATGAATTACGATCTCGCTAGGGGAATAAATCTTTGGGCACCTAGTGGTCATG

2 CATGACCACTAGGAGCATCTTTGGCGACTGCGGGCGTGGTATGCCCGGACAATCGCTCTAGGGGAATAAATCTTTGGGCACCTAGTGGTCATG

2 CATGACCACTAGGAGCATCTTTGGCGAAGTGAGCATTGGAACGGCTGGCTCCACTCGCTAGGGGAATAAATCTTTGGGCACCTAGTGGTCATG

2 CATGACCACTAGGAGCATCTTTGGCGAACGGCAGGTGTTGCGTTGGTCTGTGAATCACTAGGGGAATAAATCTTTGGGCACCTAGTGGTCATG

2 CATGACCACTAGGAGCATCTTTGGCGACCCAGGGGACCACGTCTCAAGCTGGGTGGACTAGGGGAATAAATCTTTGGGCACCTAGTGGTCATG

2 CATGACCACTAGGAGCATCTTTGGCGAGGCTAACAGAGGTCGTCGGTTGCTGTCGAGCTAGGGGAATAAATCTTTGGGCACCTAGTGGTCATG

2 CATGACCACTAGGAGCATCTTTGGCGAAGTCGTGGTTGGTTCAGTGGATGTGGCCAGCTAGGGGAATAAATCTTTGGGCACCTAGTGGTCATG

2 CATGACCACTAGGAGCATCTTTGGCGACGGTCTGGGCGATGCGTGAGAGCAAGGAGACTAGGGGAATAAATCTTTGGGCACCTAGTGGTCATG

2 CATGACCACTAGGAGCATCTTTGGCGAGATCGGGAGAATCGGCGGTATTGGTGTCTCCTAGGGGAATAAATCTTTGGGCACCTAGTGGTCATG

2 CATGACCACTAGGAGCATCTTTGGCGAGATCGGTGGTCGTGGAAAACTGAGAGCGCCCTAGGGGAATAAATCTTTGGGCACCTAGTGGTCATG

2 CATGACCACTAGGAGCATCTTTGGCGACTGAGGTGGTCATAGAATGGGGCGTCGCCGCTAGGGGAATAAATCTTTGGGCACCTAGTGGTCATG

2 CATGACCACTAGGAGCATCTTTGGCGAGAGAGTCGGTAGGCAGAGTGCTCCAGCCAGCTAGGGGAATAAATCTTTGGGCACCTAGTGGTCATG

2 CATGACCACTAGGAGCATCTTTGGCGATCCCGGGGGACCTGTCCACCGGTGTGGAGACTAGGGGAATAAATCTTTGGGCACCTAGTGGTCATG

2 CATGACCACTAGGAGCATCTTTGGCGACCTTGGCGGGGACAAGAGACGAAGGGGCGACTAGGGGAATAAATCTTTGGGCACCTAGTGGTCATG

2 CATGACCACTAGGAGCATCTTTGGCGACCGGCTGGGGCAGGGGGAGCAGAGAGGGAGCTAGGGGAATAAATCTTTGGGCACCTAGTGGTCATG

2 CATGACCACTAGGAGCATCTTTGGCGATCCTCGCCAGATGGTGAAGCAGACGTTTGGCTAGGGGAATAAATCTTTGGGCACCTAGTGGTCATG

2 CATGACCACTAGGAGCATCTTTGGCGAACGGCAGGTGTTACGGTGGTCTGTGTATCCCTAGGGGAATAAATCTTTGGGCACCTAGTGGTCATG

2 CATGACCACTAGGAGCATCTTTGGCGAGTGGTGGGCGACGGCCGGTAGTGGGTGAGACTAGGGGAATAAATCTTTGGGCACCTAGTGGTCATG

2 CATGACCACTAGGAGCATCTTTGGCGAAACAGGCGCATCCGTAGGCCTGAAGTGGAGCTAGGGGAATAAATCTTTGGGCACCTAGTGGTCATG

2 CATGACCACTAGGAGCATCTTTGGCGAGATCGGCAGAATCGGTGGCATTGGTGTCCCCTAGGGGAATAAATCTTTGGGCACCTAGTGGTCATG

2 CATGACCACTAGGAGCATCTTTGGCGAACTACGTGAGCGGACGAGGCGTCAGTGGAGCTAGGGGAATAAATCTTTGGGCACCTAGTGGTCATG

2 CATGACCACTAGGAGCATCTTTGGCGACAGGGGGCATGGACGTAGAAAAACAGTCGCCTAGGGGAATAAATCTTTGGGCACCTAGTGGTCATG

2 CATGACCACTAGGAGCATCTTTGGCGAACGCGTAGGACTGTTTCGGGCTGGATCGACCTAGGGGAATAAATCTTTGGGCACCTAGTGGTCATG

2 CATGACCACTAGGAGCATCTTTGGCGACTGCCAGGGTGCGTTGTAAGAGTTAGTCGCCTAGGGGAATAAATCTTTGGGCACCTAGTGGTCATG

2 CATGACCACTAGGAGCATCTTTGGCGACTCAATGGTAGATGGAAGCAGAGGTGTCGACTAGGGGAATAAATCTTTGGGCACCTAGTGGTCATG

2 CATGACCACTAGGAGCATCTTTGGCGATGGGGGCGTGCTATCTGCACACTCCGCGGGCTAGGGGAATAAATCTTTGGGCACCTAGTGGTCATG

2 CATGACCACTAGGAGCATCTTTGGCGAGATCAGGAGAATAGGTGGCATTGGTGTCTCCTAGGGGAATAAATCTTTGGGCACCTAGTGGTCATG

2 CATGACCACTAGGAGCATCTTTGGCGACACGGAACGGCTAGCGCCGACCGCTGTGGACTAGGGGAATAAATCTTTGGGCACCTAGTGGTCATG

2 CATGACCACTAGGAGCATCTTTGGCGACACGCACGAGAGATGCCCATTGGTGTGGAGCTAGGGGAATAAATCTTTGGGCACCTAGTGGTCATG

2 CATGACCACTAGGAGCATCTTTGGCGAGACTACTCTGGATTCGGGGACCAGTTGCTGCTAGGGGAATAAATCTTTGGGCACCTAGTGGTCATG

2 CATGACCACTAGGAGCATCTTTGGCGACCCAAGAGAATCGGCGGTGGGCTTGGGCGACTAGGGGAATAAATCTTTGGGCACCTAGTGGTCATG

2 CATGACCACTAGGAGCATCTTTGGCGATAGGCGTGTACGGTCGAGCGAAAGCGATGGCTAGGGGAATAAATCTTTGGGCACCTAGTGGTCATG

2 CATGACCACTAGGAGCATCTTTGGCGACAGCGTGTACGTGTGAACGCAGCGCACAGACTAGGGGAATAAATCTTTGGGCACCTAGTGGTCATG

2 CATGACCACTAGGAGCATCTTTGGCGACTTGGGGGCACACGTCGGAGAAAGAGTCGACTAGGGGAATAAATCTTTGGGCACCTAGTGGTCATG

2 CATGACCACTAGGAGCATCTTTGGCGATCGCTGTAGCGCACGTATAAGAAGAGGGGCCTAGGGGAATAAATCTTTGGGCACCTAGTGGTCATG

2 CATGACCACTAGGAGCATCTTTGGCGAATGGCCGGCACAGCCTTCTAGTCCTTGGTACTAGGGGAATAAATCTTTGGGCACCTAGTGGTCATG

2 CATGACCACTAGGAGCATCTTTGGCGAAACGATCTTGTCGTTGGGGACCAGTTGCTGCTAGGGGAATAAATCTTTGGGCACCTAGTGGTCATG

2 CATGACCACTAGGAGCATCTTTGGCGACACGCAGTTAGTAAACTAACAGAAATGAGACTAGGGGAATAAATCTTTGGGCACCTAGTGGTCATG

2 CATGACCACTAGGAGCATCTTTGGCGATAGTGCGGTAGGCGGCGTCGGCCTATCGACCTAGGGGAATAAATCTTTGGGCACCTAGTGGTCATG

2 CATGACCACTAGGAGCATCTTTGGCGAGGTCGAGGGCGCCGGAATGTCGACTGCTGCCTAGGGGAATAAATCTTTGGGCACCTAGTGGTCATG

2 CATGACCACTAGGAGCATCTTTGGCGAGGAGCTGTGCACGTGGGTGTGGTCCGGGCCCTAGGGGAATAAATCTTTGGGCACCTAGTGGTCATG

2 CATGACCACTAGGAGCATCTTTGGCGACTCGACTAAGCACAGTGATGTCGTAGTGGACTAGGGGAATAAATCTTTGGGCACCTAGTGGTCATG

2 CATGACCACTAGGAGCATCTTTGGCGACGTGTGGGCTACGACGCGGTCTTGAGGCGCCTAGGGGAATAAATCTTTGGGCACCTAGTGGTCATG

2 CATGACCACTAGGAGCATCTTTGGCGAGTGGGGGGCGACAGCCGGTAGTGGGTGAGACTAGGGGAATAAATCTTTGGGCACCTAGTGGTCATG

2 CATGACCACTAGGAGCATCTTTGGCGATTGGCAGGCACGGCCTTCTAGTCCTCGGTACTAGGGGAATAAATCTTTGGGCACCTAGTGGTCATG

2 CATGACCACTAGGAGCATCTTTGGCGATAATCTGGGCGGCAGGTTGTAGGAGATCGACTAGGGGAATAAATCTTTGGGCACCTAGTGGTCATG

2 CATGACCACTAGGAGCATCTTTGGCGAGAGTGAAGGATGCGACATAAGGACATCGAGCTAGGGGAATAAATCTTTGGGCACCTAGTGGTCATG

2 CATGACCACTAGGAGCATCTTTGGCGACGGGCGGGCGCCCTAGGGTGTGAGTGTTGTCTAGGGGAATAAATCTTTGGGCACCTAGTGGTCATG

2 CATGACCACTAGGAGCATCTTTGGCGATAGTTTTGTTGGACCGGGGAATCCTTGGTCCTAGGGGAATAAATCTTTGGGCACCTAGTGGTCATG

2 CATGACCACTAGGAGCATCTTTGGCGAACTAGAGGGCACCGTATAGACCAAGGAAGCCTAGGGGAATAAATCTTTGGGCACCTAGTGGTCATG

2 CATGACCACTAGGAGCATCTTTGGCGACGGGAGGGCATGTGTTGTTTGGGCAGGCGCCTAGGGGAATAAATCTTTGGGCACCTAGTGGTCATG

2 CATGACCACTAGGAGCATCTTTGGCGATCCGGTGGGTGCTGCTAAGAGGCGGCAAGCCTAGGGGAATAAATCTTTGGGCACCTAGTGGTCATG

2 CATGACCACTAGGAGCATCTTTGGCGAGAACGCGAACGCGTAAGAGCAGACGTTTGGCTAGGGGAATAAATCTTTGGGCACCTAGTGGTCATG

2 CATGACCACTAGGAGCATCTTTGGCGACCAGTAGCTGCGGAGTATAAGGTAGGTCGACTAGGGGAATAAATCTTTGGGCACCTAGTGGTCATG

2 CATGACCACTAGGAGCATCTTTGGCGACGCGGCAACCGAAGGGGTGGACGACGTGGACTAGGGGAATAAATCTTTGGGCACCTAGTGGTCATG

2 CATGACCACTAGGAGCATCTTTGGCGACTAAGTATCCCTGCCTGTCAGCGGTCGCCTCTAGGGGAATAAATCTTTGGGCACCTAGTGGTCATG

2 CATGACCACTAGGAGCATCTTTGGCGACGAATGAAGCAACGCTAAATGCTTGTGGAGCTAGGGGAATAAATCTTTGGGCACCTAGTGGTCATG

2 CATGACCACTAGGAGCATCTTTGGCGAGATCGGTAGAATCGGTGGCATTGGCGTCTCCTAGGGGAATAAATCTTTGGGCACCTAGTGGTCATG

2 CATGACCACTAGGAGCATCTTTGGCGAACGGTAGGTGTTGCGGTGGTCTGTGGATCCCTAGGGGAATAAATCTTTGGGCACCTAGTGGTCATG

2 CATGACCACTAGGAGCATCTTTGGCGACGACTGCGTGCATGGCATGCCGCTACGAGACTAGGGGAATAAATCTTTGGGCACCTAGTGGTCATG

2 CATGACCACTAGGAGCATCTTTGGCGACACAACGAAGTGCACGCAGTGTTGTGGATCCTAGGGGAATAAATCTTTGGGCACCTAGTGGTCATG

2 CATGACCACTAGGAGCATCTTTGGCGAGTGGTAACAGGGCTGGGGTGCCGAACGGAGCTAGGGGAATAAATCTTTGGGCACCTAGTGGTCATG

2 CATGACCACTAGGAGCATCTTTGGCGAATAGCGAAGTGCGAGAATCTTGAGAGTCACCTAGGGGAATAAATCTTTGGGCACCTAGTGGTCATG

2 CATGACCACTAGGAGCATCTTTGGCGACTGTGGTCTGGCCGGTGGCTGTAGAGTCGACTAGGGGAATAAATCTTTGGGCACCTAGTGGTCATG

2 CATGACCACTAGGAGCATCTTTGGCGAGGAACAAGCGGTGGTGCGGAGTGGTCGAGACTAGGGGAATAAATCTTTGGGCACCTAGTGGTCATG

2 CATGACCACTAGGAGCATCTTTGGCGAGCGGTCCGGGTATCGGCAAATGGAGATCGACTAGGGGAATAAATCTTTGGGCACCTAGTGGTCATG

2 CATGACCACTAGGAGCATCTTTGGCGAAAGTCGGTAGAACAGGGTGCGGTGCTGTCCCTAGGGGAATAAATCTTTGGGCACCTAGTGGTCATG

2 CATGACCACTAGGAGCATCTTTGGCGACGGGCGGGGGGCATGGTCTCTGGGAGTCGACTAGGGGAATAAATCTTTGGGCACCTAGTGGTCATG

2 CATGACCACTAGGAGCATCTTTGGCGATCAGGCCGCCAGGGCGTGCGAAGTGTGGTTCTAGGGGAATAAATCTTTGGGCACCTAGTGGTCATG

2 CATGACCACTAGGAGCATCTTTGGCGATAGATACAAGGCGGGTGCCATACTATCGCACTAGGGGAATAAATCTTTGGGCACCTAGTGGTCATG

2 CATGACCACTAGGAGCATCTTTGGCGATCCGGTAATGGCATGCCTTCCGGGTGGAGCCTAGGGGAATAAATCTTTGGGCACCTAGTGGTCATG

2 CATGACCACTAGGAGCATCTTTGGCGAAAGAAACGAAGATTGCGTAGACCTTGGAGCCTAGGGGAATAAATCTTTGGGCACCTAGTGGTCATG

2 CATGACCACTAGGAGCATCTTTGGCGACCCGGAAGCCCCGCGGGATGGAGTGGTGGACTAGGGGAATAAATCTTTGGGCACCTAGTGGTCATG

2 CATGACCACTAGGAGCATCTTTGGCGAGAAGACTTTGGATTCGGGGACCAGTTGCCGCTAGGGGAATAAATCTTTGGGCACCTAGTGGTCATG

2 CATGACCACTAGGAGCATCTTTGGCGACTGTGAGGCGTGGAGGGTAGGACAGTCAGACTAGGGGAATAAATCTTTGGGCACCTAGTGGTCATG

2 CATGACCACTAGGAGCATCTTTGGCGAGGGGACCTGATGCCTACCGGTGGTCGAGTACTAGGGGAATAAATCTTTGGGCACCTAGTGGTCATG

2 CATGACCACTAGGAGCATCTTTGGCGAGCCGATTGAGCAACAATGTGTTCTGGGTAACTAGGGGAATAAATCTTTGGGCACCTAGTGGTCATG

2 CATGACCACTAGGAGCATCTTTGGCGAGACAGGGTCTGGGCTAGATTTGGTCGACGCCTAGGGGAATAAATCTTTGGGCACCTAGTGGTCATG

2 CATGACCACTAGGAGCATCTTTGGCGATTGGACAAGCTCGTCTAATGCGAAGGTCGCCTAGGGGAATAAATCTTTGGGCACCTAGTGGTCATG

2 CATGACCACTAGGAGCATCTTTGGCGAAGGGTGTAGGAATTCAAGTGGATCTCATAGCTAGGGGAATAAATCTTTGGGCACCTAGTGGTCATG

2 CATGACCACTAGGAGCATCTTTGGCGACCCGTCAGGATGCAATTACAGGCGGGTGGACTAGGGGAATAAATCTTTGGGCACCTAGTGGTCATG

2 CATGACCACTAGGAGCATCTTTGGCGACACAGGTGGCAGCCCTTAAGTGAAGTGGAGCTAGGGGAATAAATCTTTGGGCACCTAGTGGTCATG

2 CATGACCACTAGGAGCATCTTTGGCGACCTGGAGGGCCCATGGCCGTCCAGCGTGGACTAGGGGAATAAATCTTTGGGCACCTAGTGGTCATG

2 CATGACCACTAGGAGCATCTTTGGCGACGGCGTGGGGCTATTGATGCGGCGGCACACCTAGGGGAATAAATCTTTGGGCACCTAGTGGTCATG

2 CATGACCACTAGGAGCATCTTTGGCGATCAGCATGGCATATGCGCCTGGAGCGTAGGCTAGGGGAATAAATCTTTGGGCACCTAGTGGTCATG

2 CATGACCACTAGGAGCATCTTTGGCGAAGGGCGATGAATGGTTGGCGTGGTGGGCCGCTAGGGGAATAAATCTTTGGGCACCTAGTGGTCATG

2 CATGACCACTAGGAGCATCTTTGGCGAAGAGTGTAGGACTTCAAGTGGATCTCATAGCTAGGGGAATAAATCTTTGGGCACCTAGTGGTCATG

2 CATGACCACTAGGAGCATCTTTGGCGACAGTGGGGCTGGTAGGAAAGTACTTATAGCCTAGGGGAATAAATCTTTGGGCACCTAGTGGTCATG

2 CATGACCACTAGGAGCATCTTTGGCGACAGTATAAAAGATACAATTGATTTGTGCGCCTAGGGGAATAAATCTTTGGGCACCTAGTGGTCATG

2 CATGACCACTAGGAGCATCTTTGGCGAGCCTTAGGAGCGCTTCGTGGGCTTGGGCCCCTAGGGGAATAAATCTTTGGGCACCTAGTGGTCATG

2 CATGACCACTAGGAGCATCTTTGGCGACCCAGGCGCGGCACGCGGCCGGTAGTGGAGCTAGGGGAATAAATCTTTGGGCACCTAGTGGTCATG

2 CATGACCACTAGGAGCATCTTTGGCGACTAGTTGGGAATGGTTATTGGCGCTAGGAGCTAGGGGAATAAATCTTTGGGCACCTAGTGGTCATG

2 CATGACCACTAGGAGCATCTTTGGCGACGATGGGACGAGTTACGAAGGGAGAGGCGACTAGGGGAATAAATCTTTGGGCACCTAGTGGTCATG

2 CATGACCACTAGGAGCATCTTTGGCGAGTAGTGTGGCACTTCAGATCAAGAGACGAGCTAGGGGAATAAATCTTTGGGCACCTAGTGGTCATG

2 CATGACCACTAGGAGCATCTTTGGCGACTCGTCATCACGCAGCTGAGACGATGTGGACTAGGGGAATAAATCTTTGGGCACCTAGTGGTCATG

2 CATGACCACTAGGAGCATCTTTGGCGAGGGACTACTCAAACAAAGAATTTGTCCTCGCTAGGGGAATAAATCTTTGGGCACCTAGTGGTCATG

2 CATGACCACTAGGAGCATCTTTGGCGAGTAGTGGGCAAAATGGCCGTGGGCCCGAGACTAGGGGAATAAATCTTTGGGCACCTAGTGGTCATG

2 CATGACCACTAGGAGCATCTTTGGCGACTAGGAACGTGGCGTGCTCAGTGTTGGAGACTAGGGGAATAAATCTTTGGGCACCTAGTGGTCATG

2 CATGACCACTAGGAGCATCTTTGGCGACGTGGTCCATAAGCGGAAAGTGAGAGTCGACTAGGGGAATAAATCTTTGGGCACCTAGTGGTCATG

2 CATGACCACTAGGAGCATCTTTGGCGAGTCCTCACGCCGGGCAAATGGACTAAGCATCTAGGGGAATAAATCTTTGGGCACCTAGTGGTCATG

2 CATGACCACTAGGAGCATCTTTGGCGATAGTCGGCAGAACAGGGTGGGGTGCTGTCCCTAGGGGAATAAATCTTTGGGCACCTAGTGGTCATG

2 CATGACCACTAGGAGCATCTTTGGCGACGTCGTGGGGCCATCTGTGCGGCGGATCCCCTAGGGGAATAAATCTTTGGGCACCTAGTGGTCATG

2 CATGACCACTAGGAGCATCTTTGGCGACCCGAGCCGCGACAAGCGCGATTGGGTGGACTAGGGGAATAAATCTTTGGGCACCTAGTGGTCATG

2 CATGACCACTAGGAGCATCTTTGGCGACTGAGGCGTGAAAAACGGATCCGAAGTGGACTAGGGGAATAAATCTTTGGGCACCTAGTGGTCATG

2 CATGACCACTAGGAGCATCTTTGGCGACCGTAGGGAAACAAAAGTGGCCTTGCTAGCCTAGGGGAATAAATCTTTGGGCACCTAGTGGTCATG

2 CATGACCACTAGGAGCATCTTTGGCGACGGGAGTCGGTCATGGTGTTGTGGAGTCGCCTAGGGGAATAAATCTTTGGGCACCTAGTGGTCATG

2 CATGACCACTAGGAGCATCTTTGGCGAGTTCCCAAAAGGGACAACAGTGAAGGAAGCCTAGGGGAATAAATCTTTGGGCACCTAGTGGTCATG

2 CATGACCACTAGGAGCATCTTTGGCGAGATAGGGAGAATCGGTGGCAGTGGTGTCTCCTAGGGGAATAAATCTTTGGGCACCTAGTGGTCATG

2 CATGACCACTAGGAGCATCTTTGGCGATGGGTGCTGACGGGGCTAGTGAGCCTTACACTAGGGGAATAAATCTTTGGGCACCTAGTGGTCATG

2 CATGACCACTAGGAGCATCTTTGGCGACGGTGTGGGGGACTTGTTTCGGCGGTGCTACTAGGGGAATAAATCTTTGGGCACCTAGTGGTCATG

2 CATGACCACTAGGAGCATCTTTGGCGACAGGCCGAGGGATCATGGTAGTACGGTCGACTAGGGGAATAAATCTTTGGGCACCTAGTGGTCATG

2 CATGACCACTAGGAGCATCTTTGGCGACACGGTGCGAAGGAGCTCCGTCCGTGGAACCTAGGGGAATAAATCTTTGGGCACCTAGTGGTCATG

2 CATGACCACTAGGAGCATCTTTGGCGAAGCGTGTTGCAAGGTCCGCGAGTGTGAACCCTAGGGGAATAAATCTTTGGGCACCTAGTGGTCATG

2 CATGACCACTAGGAGCATCTTTGGCGACTTGTGCGATGTCGGGGGGTAATGTCGCTTCTAGGGGAATAAATCTTTGGGCACCTAGTGGTCATG

2 CATGACCACTAGGAGCATCTTTGGCGAAGGGTGTAGGACTACAAGTGGATCTCATAGCTAGGGGAATAAATCTTTGGGCACCTAGTGGTCATG

2 CATGACCACTAGGAGCATCTTTGGCGAGACAACGAACAAGCTAATAAAGGATCGCTGCTAGGGGAATAAATCTTTGGGCACCTAGTGGTCATG

2 CATGACCACTAGGAGCATCTTTGGCGAAGTGAAGTATGGCTGTATTAGTGTAGCGAGCTAGGGGAATAAATCTTTGGGCACCTAGTGGTCATG

2 CATGACCACTAGGAGCATCTTTGGCGACAAAATCGATGACGAAAAATTTGTTGCCTGCTAGGGGAATAAATCTTTGGGCACCTAGTGGTCATG

2 CATGACCACTAGGAGCATCTTTGGCGAGTCGTGGGCTAATGGGACGGGGTTTCGAGCCTAGGGGAATAAATCTTTGGGCACCTAGTGGTCATG

2 CATGACCACTAGGAGCATCTTTGGCGAGCGCTGATGTGTTGGCACCTTAGAGCTGGCCTAGGGGAATAAATCTTTGGGCACCTAGTGGTCATG

2 CATGACCACTAGGAGCATCTTTGGCGATTGAAGTTCGACTGAAGGGCGAACCAGTGCCTAGGGGAATAAATCTTTGGGCACCTAGTGGTCATG

2 CATGACCACTAGGAGCATCTTTGGCGACGCATGATTAATCGTAAGGTAATAGGCGCGCTAGGGGAATAAATCTTTGGGCACCTAGTGGTCATG

2 CATGACCACTAGGAGCATCTTTGGCGAAGACGGATGAGTGTTTAGAACAGTCGAAGACTAGGGGAATAAATCTTTGGGCACCTAGTGGTCATG

2 CATGACCACTAGGAGCATCTTTGGCGAGATCGGGAGAATCGGTGGCATTGGTTTTTCCTAGGGGAATAAATCTTTGGGCACCTAGTGGTCATG

2 CATGACCACTAGGAGCATCTTTGGCGACCCAGTTACCTACGATGGTACTGGCGTGAGCTAGGGGAATAAATCTTTGGGCACCTAGTGGTCATG

2 CATGACCACTAGGAGCATCTTTGGCGACGAGGGGCGCGAAGCTTTCGGGAAGGAGTACTAGGGGAATAAATCTTTGGGCACCTAGTGGTCATG

2 CATGACCACTAGGAGCATCTTTGGCGAGAGTCAAAAAGGAAACAAGTGGCCGAGCTTCTAGGGGAATAAATCTTTGGGCACCTAGTGGTCATG

2 CATGACCACTAGGAGCATCTTTGGCGACCAAGCGACCGACGTGGCCGAGCTTTGCGACTAGGGGAATAAATCTTTGGGCACCTAGTGGTCATG

2 CATGACCACTAGGAGCATCTTTGGCGAGGATGTGGTCCTAGGCAGTGCTGGTCGAGACTAGGGGAATAAATCTTTGGGCACCTAGTGGTCATG

2 CATGACCACTAGGAGCATCTTTGGCGAAGTGCGGTGGGCATGTGGGTGTTAGCGACGCTAGGGGAATAAATCTTTGGGCACCTAGTGGTCATG

2 CATGACCACTAGGAGCATCTTTGGCGAAACAGGTGATCGGTGCTCGGGTGCCTGGCCCTAGGGGAATAAATCTTTGGGCACCTAGTGGTCATG

2 CATGACCACTAGGAGCATCTTTGGCGATTATTGGCTGGTAGGTTGCGTATTGGGGAGCTAGGGGAATAAATCTTTGGGCACCTAGTGGTCATG

2 CATGACCACTAGGAGCATCTTTGGCGACTGGGGTCTCGTACACAATGTCTGCATCGCCTAGGGGAATAAATCTTTGGGCACCTAGTGGTCATG

2 CATGACCACTAGGAGCATCTTTGGCGAAGCAGTGCGACGGGCTATGGGATCCTCGAGCTAGGGGAATAAATCTTTGGGCACCTAGTGGTCATG

2 CATGACCACTAGGAGCATCTTTGGCGACGGCGTGGGGCCATCTGTGCGGCGGATACCCTAGGGGAATAAATCTTTGGGCACCTAGTGGTCATG

2 CATGACCACTAGGAGCATCTTTGGCGAGCCGAGGATGTCTACTTAGAGAGAGGTCGACTAGGGGAATAAATCTTTGGGCACCTAGTGGTCATG

2 CATGACCACTAGGAGCATCTTTGGCGACGGGCGGGCGCCTAAGGGTGTGAGTGTTGCCTAGGGGAATAAATCTTTGGGCACCTAGTGGTCATG

2 CATGACCACTAGGAGCATCTTTGGCGACCGTGGTAAAGTGTCAACGAGGTTAGGCGACTAGGGGAATAAATCTTTGGGCACCTAGTGGTCATG

2 CATGACCACTAGGAGCATCTTTGGCGACACAGTGGTACAGTCTGTAACGACTGCTCGCTAGGGGAATAAATCTTTGGGCACCTAGTGGTCATG

2 CATGACCACTAGGAGCATCTTTGGCGAGTCCAGTGGCCGAGCAGCATCGGAGTGCGACTAGGGGAATAAATCTTTGGGCACCTAGTGGTCATG

2 CATGACCACTAGGAGCATCTTTGGCGAAGTCAATAATGCGTACTGCGTGGAATTCGACTAGGGGAATAAATCTTTGGGCACCTAGTGGTCATG

2 CATGACCACTAGGAGCATCTTTGGCGAACTGGCAGCGTTGCGATGTCTGGAATAGCACTAGGGGAATAAATCTTTGGGCACCTAGTGGTCATG

2 CATGACCACTAGGAGCATCTTTGGCGACAGCTGGCTGGCAGTGCGTACTAGTAGTCACTAGGGGAATAAATCTTTGGGCACCTAGTGGTCATG

2 CATGACCACTAGGAGCATCTTTGGCGAGCAGTCCCGGGTCCGGACGACAGGCATAGCCTAGGGGAATAAATCTTTGGGCACCTAGTGGTCATG

2 CATGACCACTAGGAGCATCTTTGGCGACCGGGGATGCCGATGCGCTGGACGACCGAGCTAGGGGAATAAATCTTTGGGCACCTAGTGGTCATG

2 CATGACCACTAGGAGCATCTTTGGCGAGATCGGGAGAATCTGTGGCATTGGTTTCTCCTAGGGGAATAAATCTTTGGGCACCTAGTGGTCATG

2 CATGACCACTAGGAGCATCTTTGGCGATACGACAGTGGCTGAGTATAGTGAAATCGACTAGGGGAATAAATCTTTGGGCACCTAGTGGTCATG

2 CATGACCACTAGGAGCATCTTTGGCGACCCATGGTGCATCGCATCCGCTTGGGTGGACTAGGGGAATAAATCTTTGGGCACCTAGTGGTCATG

2 CATGACCACTAGGAGCATCTTTGGCGACGGCGTGGGGCCATCTGTGCGGCGGATCCACTAGGGGAATAAATCTTTGGGCACCTAGTGGTCATG

2 CATGACCACTAGGAGCATCTTTGGCGACGTGTGGGCTCAAGCCAGGTGTAAACGAGACTAGGGGAATAAATCTTTGGGCACCTAGTGGTCATG

2 CATGACCACTAGGAGCATCTTTGGCGAGCCGATTGCGCAACAATGTGTTCTGGGTAACTAGGGGAATAAATCTTTGGGCACCTAGTGGTCATG

2 CATGACCACTAGGAGCATCTTTGGCGACGAGTCAACGGTATCAGCTCGGGATGGAGCCTAGGGGAATAAATCTTTGGGCACCTAGTGGTCATG

2 CATGACCACTAGGAGCATCTTTGGCGAGTTCGTCCGGGGCGGAACTGACGAATCGATCTAGGGGAATAAATCTTTGGGCACCTAGTGGTCATG

2 CATGACCACTAGGAGCATCTTTGGCGATTACCGTCGGCCTGGACCGCGGTGTGGAGACTAGGGGAATAAATCTTTGGGCACCTAGTGGTCATG

2 CATGACCACTAGGAGCATCTTTGGCGAGAGGAGACGCAATGATTGCGGCAAGTGGAGCTAGGGGAATAAATCTTTGGGCACCTAGTGGTCATG

2 CATGACCACTAGGAGCATCTTTGGCGAACTCTAAATTCGAAGAGTATGTAGGGCAGACTAGGGGAATAAATCTTTGGGCACCTAGTGGTCATG

2 CATGACCACTAGGAGCATCTTTGGCGAGATCGGGAGAATCGTTGGCATTGGAGTCTCCTAGGGGAATAAATCTTTGGGCACCTAGTGGTCATG

2 CATGACCACTAGGAGCATCTTTGGCGATCAGGTCGCCAGGGCGTACGAAGTGTGGTTCTAGGGGAATAAATCTTTGGGCACCTAGTGGTCATG

2 CATGACCACTAGGAGCATCTTTGGCGACGTAACTGGAGGGACATGTGCGTGAGGCGACTAGGGGAATAAATCTTTGGGCACCTAGTGGTCATG

2 CATGACCACTAGGAGCATCTTTGGCGACTGTGACCGTCTAGCAGTGCAACCAGGCGACTAGGGGAATAAATCTTTGGGCACCTAGTGGTCATG

2 CATGACCACTAGGAGCATCTTTGGCGAGATCGGTGGAATCGGTGGCATTGGTGTCTCCTAGGGGAATAAATCTTTGGGCACCTAGTGGTCATG

2 CATGACCACTAGGAGCATCTTTGGCGACCGAGATCGGCTAAGTGTCGAGACAAGCCTCTAGGGGAATAAATCTTTGGGCACCTAGTGGTCATG

2 CATGACCACTAGGAGCATCTTTGGCGAGGGAGTGGGTACAATGCGTGGAGTCCGAGACTAGGGGAATAAATCTTTGGGCACCTAGTGGTCATG

2 CATGACCACTAGGAGCATCTTTGGCGATCAGGCCTTGGCTGCGGGTGGGTGCGCACCCTAGGGGAATAAATCTTTGGGCACCTAGTGGTCATG

2 CATGACCACTAGGAGCATCTTTGGCGATCCCTGCGAGGGTGAAGGAGAGGTGAGAGACTAGGGGAATAAATCTTTGGGCACCTAGTGGTCATG

2 CATGACCACTAGGAGCATCTTTGGCGAGATAGGGAGAATCTGAGGCATTGGTGTCTCCTAGGGGAATAAATCTTTGGGCACCTAGTGGTCATG

2 CATGACCACTAGGAGCATCTTTGGCGATCCGACCTGTCGGTGTGTGACAACGTCAGCCTAGGGGAATAAATCTTTGGGCACCTAGTGGTCATG

2 CATGACCACTAGGAGCATCTTTGGCGAATGGCCGGCACGGCGTTCTAGTCCTCGGTACTAGGGGAATAAATCTTTGGGCACCTAGTGGTCATG

2 CATGACCACTAGGAGCATCTTTGGCGAGGCGAGTCCAGCGGCTTTCTTGGCCTGCGACTAGGGGAATAAATCTTTGGGCACCTAGTGGTCATG

2 CATGACCACTAGGAGCATCTTTGGCGACCTCCAGTACTGGAAGTACAAGGTGTGGAGCTAGGGGAATAAATCTTTGGGCACCTAGTGGTCATG

2 CATGACCACTAGGAGCATCTTTGGCGACTAAGTGGCCCACGCGTTAAATGGGGTAGACTAGGGGAATAAATCTTTGGGCACCTAGTGGTCATG

2 CATGACCACTAGGAGCATCTTTGGCGATGGGGCCGTGATATCTGCACACTCCGCGGGCTAGGGGAATAAATCTTTGGGCACCTAGTGGTCATG

2 CATGACCACTAGGAGCATCTTTGGCGAGCAAGGTAGTCGACGGGTCGCGGCCCGAGCCTAGGGGAATAAATCTTTGGGCACCTAGTGGTCATG

2 CATGACCACTAGGAGCATCTTTGGCGACATTGTGGGCATGCGCGAATAAGAGGTCGACTAGGGGAATAAATCTTTGGGCACCTAGTGGTCATG

2 CATGACCACTAGGAGCATCTTTGGCGAGCCGATTGTGCAACAATGTGTTTTGGGTAACTAGGGGAATAAATCTTTGGGCACCTAGTGGTCATG

2 CATGACCACTAGGAGCATCTTTGGCGACGTTCCTGGTACGGGTTCGAGAACGGCGCACTAGGGGAATAAATCTTTGGGCACCTAGTGGTCATG

2 CATGACCACTAGGAGCATCTTTGGCGACCCGTCTGTGCAAACAGCGGTAGTGGGCGACTAGGGGAATAAATCTTTGGGCACCTAGTGGTCATG

2 CATGACCACTAGGAGCATCTTTGGCGACAGGGGATGCCGATGAGCTGGACGACCGAGCTAGGGGAATAAATCTTTGGGCACCTAGTGGTCATG

2 CATGACCACTAGGAGCATCTTTGGCGACAGTATATGGAGGTGAGGGATGCTGGCGCTCTAGGGGAATAAATCTTTGGGCACCTAGTGGTCATG

2 CATGACCACTAGGAGCATCTTTGGCGACCCGCGTTGCACGAAGAGGCGGGGTGGAGCCTAGGGGAATAAATCTTTGGGCACCTAGTGGTCATG

2 CATGACCACTAGGAGCATCTTTGGCGATGGGTGCTGACGGCCGCCGCTGCGGCTACTCTAGGGGAATAAATCTTTGGGCACCTAGTGGTCATG

2 CATGACCACTAGGAGCATCTTTGGCGAGGAGAGTCCAGCGGCCTTCTTGGCCTGCGACTAGGGGAATAAATCTTTGGGCACCTAGTGGTCATG

2 CATGACCACTAGGAGCATCTTTGGCGACCTTAACGAGAAAAGCTCGATAAGTGTGGACTAGGGGAATAAATCTTTGGGCACCTAGTGGTCATG

2 CATGACCACTAGGAGCATCTTTGGCGACGGACTGAGGACCGGGGTCATCCGGAAGCTCTAGGGGAATAAATCTTTGGGCACCTAGTGGTCATG

2 CATGACCACTAGGAGCATCTTTGGCGAACAGCGGAACGGCGCGGATGGAACGTGAGACTAGGGGAATAAATCTTTGGGCACCTAGTGGTCATG

2 CATGACCACTAGGAGCATCTTTGGCGACGACGTGGTAGCGGATGGTTACGAAGTCGACTAGGGGAATAAATCTTTGGGCACCTAGTGGTCATG

2 CATGACCACTAGGAGCATCTTTGGCGATGGGTGCTGACGGACGCCGCTGCGGCTACACTAGGGGAATAAATCTTTGGGCACCTAGTGGTCATG

2 CATGACCACTAGGAGCATCTTTGGCGACGTGAGTAGCTTAGTGGCTGAGCTCCTAGACTAGGGGAATAAATCTTTGGGCACCTAGTGGTCATG

2 CATGACCACTAGGAGCATCTTTGGCGAGTAGTGGCCGAGAGCGTCTAGTGGGACGAGCTAGGGGAATAAATCTTTGGGCACCTAGTGGTCATG

2 CATGACCACTAGGAGCATCTTTGGCGAGATCGTGAGAAACGGTGGCATTGGTGTCTCCTAGGGGAATAAATCTTTGGGCACCTAGTGGTCATG

2 CATGACCACTAGGAGCATCTTTGGCGATGGGTCCGTGCTATCTGCACACTCCGCGGGCTAGGGGAATAAATCTTTGGGCACCTAGTGGTCATG

2 CATGACCACTAGGAGCATCTTTGGCGAACGTGCAAGATATGCCGCCTGGAGCGCAGGCTAGGGGAATAAATCTTTGGGCACCTAGTGGTCATG

2 CATGACCACTAGGAGCATCTTTGGCGACACGTGTCTCGAAAGATGTCCGCCGTGGAGCTAGGGGAATAAATCTTTGGGCACCTAGTGGTCATG

2 CATGACCACTAGGAGCATCTTTGGCGACGGTGTGGGGAACTTGCTTCGGCGGTGCTACTAGGGGAATAAATCTTTGGGCACCTAGTGGTCATG

2 CATGACCACTAGGAGCATCTTTGGCGAGATCGGGAGAAACGGTGTCATTGGTGTCTCCTAGGGGAATAAATCTTTGGGCACCTAGTGGTCATG

2 CATGACCACTAGGAGCATCTTTGGCGAAAGTGGTGTGGCATCCAGTAGACTCATCGCCTAGGGGAATAAATCTTTGGGCACCTAGTGGTCATG

2 CATGACCACTAGGAGCATCTTTGGCGAACGGCAGGTGTTGCGTAGGTCTGTGAATCCCTAGGGGAATAAATCTTTGGGCACCTAGTGGTCATG

2 CATGACCACTAGGAGCATCTTTGGCGAACTTGTGGTCCAAATCTGAGGGGAGGTCGCCTAGGGGAATAAATCTTTGGGCACCTAGTGGTCATG

2 CATGACCACTAGGAGCATCTTTGGCGAAATCGGAAGAATCGGTGGCATTGGTGTCTCCTAGGGGAATAAATCTTTGGGCACCTAGTGGTCATG

2 CATGACCACTAGGAGCATCTTTGGCGACCCGTGCCATACGTGTAAGTGCCGGGTGGACTAGGGGAATAAATCTTTGGGCACCTAGTGGTCATG

2 CATGACCACTAGGAGCATCTTTGGCGAGATCTGGAGAATCGGTGGCATTTGTGTCTCCTAGGGGAATAAATCTTTGGGCACCTAGTGGTCATG

2 CATGACCACTAGGAGCATCTTTGGCGACGGGACTGTGAGCCGTGGTTGCACCGGAGACTAGGGGAATAAATCTTTGGGCACCTAGTGGTCATG

2 CATGACCACTAGGAGCATCTTTGGCGATGAACCCGGTATAGGAGGTTACATCATCGACTAGGGGAATAAATCTTTGGGCACCTAGTGGTCATG

2 CATGACCACTAGGAGCATCTTTGGCGACCCGATGGCGATAGCCTATGGAGTGGAGCACTAGGGGAATAAATCTTTGGGCACCTAGTGGTCATG

2 CATGACCACTAGGAGCATCTTTGGCGACGGAGGTGGTCCAGAATACACGATCGCCAGCTAGGGGAATAAATCTTTGGGCACCTAGTGGTCATG

2 CATGACCACTAGGAGCATCTTTGGCGACCCACTCGCTTAGTAGAGTGTGAGTGGCGACTAGGGGAATAAATCTTTGGGCACCTAGTGGTCATG

2 CATGACCACTAGGAGCATCTTTGGCGAGATCGTGGGAATCGGTGGCATTGGTGTCTCCTAGGGGAATAAATCTTTGGGCACCTAGTGGTCATG

2 CATGACCACTAGGAGCATCTTTGGCGATCCGACCTGGCGAGAGACAGCGGAGGTGGACTAGGGGAATAAATCTTTGGGCACCTAGTGGTCATG

2 CATGACCACTAGGAGCATCTTTGGCGAACGGCAGGTGTTGCGGTGGTCTATGAAACCCTAGGGGAATAAATCTTTGGGCACCTAGTGGTCATG

2 CATGACCACTAGGAGCATCTTTGGCGATCCGATTCCCTCCGGGAACGAGCGTGGAGCCTAGGGGAATAAATCTTTGGGCACCTAGTGGTCATG

2 CATGACCACTAGGAGCATCTTTGGCGAAGAGTGTTGCAAGGTCCGCGAGTGTGGACCCTAGGGGAATAAATCTTTGGGCACCTAGTGGTCATG

2 CATGACCACTAGGAGCATCTTTGGCGAGAAGGCTTTGGATTCGGGGACCAGTTGCTGCTAGGGGAATAAATCTTTGGGCACCTAGTGGTCATG

2 CATGACCACTAGGAGCATCTTTGGCGACCCAGCCGAGGGAGCACCGAGATGGGTGGACTAGGGGAATAAATCTTTGGGCACCTAGTGGTCATG

2 CATGACCACTAGGAGCATCTTTGGCGAGTGGCCGAGGGATACTAATCCTACTCTCGACTAGGGGAATAAATCTTTGGGCACCTAGTGGTCATG

2 CATGACCACTAGGAGCATCTTTGGCGAAGGGACATGTCCGAACGGTGTGAGCCACGCCTAGGGGAATAAATCTTTGGGCACCTAGTGGTCATG

2 CATGACCACTAGGAGCATCTTTGGCGATCGATACGGTCGGCGTTTGGTAATGTGAGACTAGGGGAATAAATCTTTGGGCACCTAGTGGTCATG

2 CATGACCACTAGGAGCATCTTTGGCGAGTAGCGTAAGTGCACGCGTCGGAGTGAAGCCTAGGGGAATAAATCTTTGGGCACCTAGTGGTCATG

2 CATGACCACTAGGAGCATCTTTGGCGACGGCGTGGGGCTATTTATGCGGCGGCACTCCTAGGGGAATAAATCTTTGGGCACCTAGTGGTCATG

2 CATGACCACTAGGAGCATCTTTGGCGACGGGTACCGTAAAAGTTCCAAGTAAGTCGACTAGGGGAATAAATCTTTGGGCACCTAGTGGTCATG

2 CATGACCACTAGGAGCATCTTTGGCGACGTAGGTCGGAAGGGTTGCGCCGCGGCACACTAGGGGAATAAATCTTTGGGCACCTAGTGGTCATG

2 CATGACCACTAGGAGCATCTTTGGCGAGGCGAGTCCGGCGGCCTTCTTGGCCTGCGACTAGGGGAATAAATCTTTGGGCACCTAGTGGTCATG

2 CATGACCACTAGGAGCATCTTTGGCGAGTCATAGGCTGGGAGGTAAGTATTGTGAAGCTAGGGGAATAAATCTTTGGGCACCTAGTGGTCATG

2 CATGACCACTAGGAGCATCTTTGGCGATATGTGGCAGAGTAAAGCGGTTTAGATCGACTAGGGGAATAAATCTTTGGGCACCTAGTGGTCATG

2 CATGACCACTAGGAGCATCTTTGGCGAGTCGGGCGAGTCTGGGGTGTTGACTGCCAGCTAGGGGAATAAATCTTTGGGCACCTAGTGGTCATG

2 CATGACCACTAGGAGCATCTTTGGCGACATTGAATGATGGTGACTGGAAGATGTCGACTAGGGGAATAAATCTTTGGGCACCTAGTGGTCATG

2 CATGACCACTAGGAGCATCTTTGGCGAGGCTACAGGGGAAGCAGAGCAGACGTTTGGCTAGGGGAATAAATCTTTGGGCACCTAGTGGTCATG

2 CATGACCACTAGGAGCATCTTTGGCGACATTCGTGCAAGGCATCGAAAGAAGTGGAGCTAGGGGAATAAATCTTTGGGCACCTAGTGGTCATG

2 CATGACCACTAGGAGCATCTTTGGCGAGCAGGGCGCGCCCCGCGCAACCCTCGAGATCTAGGGGAATAAATCTTTGGGCACCTAGTGGTCATG

2 CATGACCACTAGGAGCATCTTTGGCGACGGCGTGTGGCTATTGATGCGGCGGCACTCCTAGGGGAATAAATCTTTGGGCACCTAGTGGTCATG

2 CATGACCACTAGGAGCATCTTTGGCGACGACTTCGGAGTGGGTGCGCTGCTGGCATACTAGGGGAATAAATCTTTGGGCACCTAGTGGTCATG

2 CATGACCACTAGGAGCATCTTTGGCGACCCACGGAGCAGGTACCTCCGATGGGTGGACTAGGGGAATAAATCTTTGGGCACCTAGTGGTCATG

2 CATGACCACTAGGAGCATCTTTGGCGACGCGCACGCGGCCGACAGCGGGCGCAACGACTAGGGGAATAAATCTTTGGGCACCTAGTGGTCATG

2 CATGACCACTAGGAGCATCTTTGGCGACTTCGATACAAATGTAATCGTCGAAGTGGACTAGGGGAATAAATCTTTGGGCACCTAGTGGTCATG

2 CATGACCACTAGGAGCATCTTTGGCGAGATCTGGAGAATCGGTGGAATTGGTGTCTCCTAGGGGAATAAATCTTTGGGCACCTAGTGGTCATG

2 CATGACCACTAGGAGCATCTTTGGCGACCCGCTGCACATAGCATTGCGTCGGGTGGACTAGGGGAATAAATCTTTGGGCACCTAGTGGTCATG

2 CATGACCACTAGGAGCATCTTTGGCGAGATCTGGAGAATCGTTGGCATTGGTGTCTCCTAGGGGAATAAATCTTTGGGCACCTAGTGGTCATG

2 CATGACCACTAGGAGCATCTTTGGCGATGGGTTCTGACGGGGCTAGTGAGCCCTACACTAGGGGAATAAATCTTTGGGCACCTAGTGGTCATG

2 CATGACCACTAGGAGCATCTTTGGCGAGTGGGGGGCGACGGCCGTTAGTGGGTGAGACTAGGGGAATAAATCTTTGGGCACCTAGTGGTCATG

2 CATGACCACTAGGAGCATCTTTGGCGAGGAGACGGCTGAGAATGCCACGGGCGAAGCCTAGGGGAATAAATCTTTGGGCACCTAGTGGTCATG

2 CATGACCACTAGGAGCATCTTTGGCGAGGGGTAAGGGGCCGCTCGCGCCTGCGTGCGCTAGGGGAATAAATCTTTGGGCACCTAGTGGTCATG

2 CATGACCACTAGGAGCATCTTTGGCGATCAGGCCGCCAGGGCGTACGAAGTGTGGCTCTAGGGGAATAAATCTTTGGGCACCTAGTGGTCATG

2 CATGACCACTAGGAGCATCTTTGGCGACGGGGTGGGGACCGCAGGTTCGGCGGGTACCTAGGGGAATAAATCTTTGGGCACCTAGTGGTCATG

2 CATGACCACTAGGAGCATCTTTGGCGACACCTTCAGAGCGGCGGCATTGTTGCGTGCCTAGGGGAATAAATCTTTGGGCACCTAGTGGTCATG

2 CATGACCACTAGGAGCATCTTTGGCGAAGGTCCGGGGAGCGAACAAGCTTGAGCTTGCTAGGGGAATAAATCTTTGGGCACCTAGTGGTCATG

2 CATGACCACTAGGAGCATCTTTGGCGACACGTAGGACCGTACCGGGTGGTAAGACATCTAGGGGAATAAATCTTTGGGCACCTAGTGGTCATG

2 CATGACCACTAGGAGCATCTTTGGCGACACGGACCACAAAATGCAGCCGTAGTGGAGCTAGGGGAATAAATCTTTGGGCACCTAGTGGTCATG

2 CATGACCACTAGGAGCATCTTTGGCGACTAGAGGGTGGCTGAGCGTCTGTAAGGAGACTAGGGGAATAAATCTTTGGGCACCTAGTGGTCATG

2 CATGACCACTAGGAGCATCTTTGGCGAGAACGGGAGAATCGGTGGCATGGGTGTCTCCTAGGGGAATAAATCTTTGGGCACCTAGTGGTCATG

2 CATGACCACTAGGAGCATCTTTGGCGATCAGGCCGCCAGGGAGTACGAAGTGTGGTTCTAGGGGAATAAATCTTTGGGCACCTAGTGGTCATG

2 CATGACCACTAGGAGCATCTTTGGCGACATAATGATGTTAGTATAATTGTAGTGGAGCTAGGGGAATAAATCTTTGGGCACCTAGTGGTCATG

2 CATGACCACTAGGAGCATCTTTGGCGACTCCAGTTCATCTGTCGGGTAGTTTAAGGACTAGGGGAATAAATCTTTGGGCACCTAGTGGTCATG

2 CATGACCACTAGGAGCATCTTTGGCGACGGGCCGTGGTCAGCAGCATCCTACCGGAGCTAGGGGAATAAATCTTTGGGCACCTAGTGGTCATG

2 CATGACCACTAGGAGCATCTTTGGCGAGAAGCGGACAGGCGAACGTTATTAGGCCTGCTAGGGGAATAAATCTTTGGGCACCTAGTGGTCATG

2 CATGACCACTAGGAGCATCTTTGGCGAGCCGGATTTGGGGGTAGGGTAGAGGCCTAGCTAGGGGAATAAATCTTTGGGCACCTAGTGGTCATG

2 CATGACCACTAGGAGCATCTTTGGCGAGATCAGGAGAGTCGGTGGCATTGGTGTCTCCTAGGGGAATAAATCTTTGGGCACCTAGTGGTCATG

2 CATGACCACTAGGAGCATCTTTGGCGATGGGGCCGTGCTATCTGCACACTCCGCGGACTAGGGGAATAAATCTTTGGGCACCTAGTGGTCATG

2 CATGACCACTAGGAGCATCTTTGGCGACCAACAGATTGCGGTAGCGTGGATGGACGACTAGGGGAATAAATCTTTGGGCACCTAGTGGTCATG

2 CATGACCACTAGGAGCATCTTTGGCGAGCATAGGGAGCCATATCGCGCGCGTGTGTCCTAGGGGAATAAATCTTTGGGCACCTAGTGGTCATG

2 CATGACCACTAGGAGCATCTTTGGCGACGCCTGAGAATACATTAAAGAATAACGAGCCTAGGGGAATAAATCTTTGGGCACCTAGTGGTCATG

2 CATGACCACTAGGAGCATCTTTGGCGAAACGGTCCGGAGCGTTAATCAAGCTTTCGCCTAGGGGAATAAATCTTTGGGCACCTAGTGGTCATG

2 CATGACCACTAGGAGCATCTTTGGCGAGTTTCGCTGTGCTTGGAATGGGGGCACCCTCTAGGGGAATAAATCTTTGGGCACCTAGTGGTCATG

2 CATGACCACTAGGAGCATCTTTGGCGAAAGTAAGGAATGCGACCGGTATACTGAAGACTAGGGGAATAAATCTTTGGGCACCTAGTGGTCATG

2 CATGACCACTAGGAGCATCTTTGGCGACGAGTGAAAGTTAGCCTGTGGCCGAGCAGACTAGGGGAATAAATCTTTGGGCACCTAGTGGTCATG

2 CATGACCACTAGGAGCATCTTTGGCGAAGCATGAGGGTTGCCTAAGTTGCGCTTCGACTAGGGGAATAAATCTTTGGGCACCTAGTGGTCATG

2 CATGACCACTAGGAGCATCTTTGGCGATGGGAGCTGACGGCCGCCGCTGCGGCTACACTAGGGGAATAAATCTTTGGGCACCTAGTGGTCATG

2 CATGACCACTAGGAGCATCTTTGGCGAACGGCGGGTGTTGCGGTGGTTTGTGAATCCCTAGGGGAATAAATCTTTGGGCACCTAGTGGTCATG

2 CATGACCACTAGGAGCATCTTTGGCGAGATCGGGAGAATCGGTGGCATCGATGTCTCCTAGGGGAATAAATCTTTGGGCACCTAGTGGTCATG

2 CATGACCACTAGGAGCATCTTTGGCGAACAGGCCGTGGCTGCGGGTGGGTCCGCACCCTAGGGGAATAAATCTTTGGGCACCTAGTGGTCATG

2 CATGACCACTAGGAGCATCTTTGGCGACGCGGATGGGGCTGCATCGGCACAGGGAGCCTAGGGGAATAAATCTTTGGGCACCTAGTGGTCATG

2 CATGACCACTAGGAGCATCTTTGGCGACCCGATAGTAACCACTGTATGTCGGGTGGACTAGGGGAATAAATCTTTGGGCACCTAGTGGTCATG

2 CATGACCACTAGGAGCATCTTTGGCGAGACTAATGACCACATAAAAAACGCAGTTCTCTAGGGGAATAAATCTTTGGGCACCTAGTGGTCATG

2 CATGACCACTAGGAGCATCTTTGGCGAGAGGTCATGGGAATGTAATTGCGCTTAAGACTAGGGGAATAAATCTTTGGGCACCTAGTGGTCATG

2 CATGACCACTAGGAGCATCTTTGGCGATATGCGAGGCGGGCGTGCAGGCAGATCGATCTAGGGGAATAAATCTTTGGGCACCTAGTGGTCATG

2 CATGACCACTAGGAGCATCTTTGGCGAGGCTCCGCGTGAGGCAGAGCAGACGTTTGGCTAGGGGAATAAATCTTTGGGCACCTAGTGGTCATG

2 CATGACCACTAGGAGCATCTTTGGCGACGGTGTGGGGAACTTGTTTCGGCGGTGCAACTAGGGGAATAAATCTTTGGGCACCTAGTGGTCATG

2 CATGACCACTAGGAGCATCTTTGGCGATAAACGGATGTGACTAGTGGCAGAGTACGCCTAGGGGAATAAATCTTTGGGCACCTAGTGGTCATG

2 CATGACCACTAGGAGCATCTTTGGCGACGGCTGGCTGGTAGTGCGTACTAGTAGTCACTAGGGGAATAAATCTTTGGGCACCTAGTGGTCATG

2 CATGACCACTAGGAGCATCTTTGGCGATCTGAAGTGTAATGAAGGAAAGGTAGATAGCTAGGGGAATAAATCTTTGGGCACCTAGTGGTCATG

2 CATGACCACTAGGAGCATCTTTGGCGAGATAGGGAGAATCGGTGTCATTGGTGTCTCCTAGGGGAATAAATCTTTGGGCACCTAGTGGTCATG

2 CATGACCACTAGGAGCATCTTTGGCGAACTGCAGGTGTTGAGGTGGTCTGTGAATCCCTAGGGGAATAAATCTTTGGGCACCTAGTGGTCATG

2 CATGACCACTAGGAGCATCTTTGGCGAGGGAGGGCGCCGTCAGCGGTGTGAATGCGCCTAGGGGAATAAATCTTTGGGCACCTAGTGGTCATG

2 CATGACCACTAGGAGCATCTTTGGCGAGTGTGGGCTGGTAGCTTGGTATTCACTCTGCTAGGGGAATAAATCTTTGGGCACCTAGTGGTCATG

2 CATGACCACTAGGAGCATCTTTGGCGACGGTGTGGGGAACTTGTTTCGGCGGTGGTACTAGGGGAATAAATCTTTGGGCACCTAGTGGTCATG

2 CATGACCACTAGGAGCATCTTTGGCGACCCGTCGATCCAGAGCGCGACGTGGTGGCACTAGGGGAATAAATCTTTGGGCACCTAGTGGTCATG

2 CATGACCACTAGGAGCATCTTTGGCGAGAAGACTCTGGATTCGGTGACCAGTTGCTCCTAGGGGAATAAATCTTTGGGCACCTAGTGGTCATG

2 CATGACCACTAGGAGCATCTTTGGCGACGTGGGCCGGGAGGATGTGGTACTTCTCGACTAGGGGAATAAATCTTTGGGCACCTAGTGGTCATG

2 CATGACCACTAGGAGCATCTTTGGCGATCCTCGTCAGATAGTGAAGCAGACGTTTGGCTAGGGGAATAAATCTTTGGGCACCTAGTGGTCATG

2 CATGACCACTAGGAGCATCTTTGGCGAGTCGAGTAACGGCACAGCGCGACGGATCGCCTAGGGGAATAAATCTTTGGGCACCTAGTGGTCATG

2 CATGACCACTAGGAGCATCTTTGGCGACTGTCAAGATCGATGCAAAAAATTAAGGAGCTAGGGGAATAAATCTTTGGGCACCTAGTGGTCATG

2 CATGACCACTAGGAGCATCTTTGGCGAGCCTTGCTTGGGAGGTTGCTCCAACAGTTCCTAGGGGAATAAATCTTTGGGCACCTAGTGGTCATG

2 CATGACCACTAGGAGCATCTTTGGCGATATCGGGAGAATCGGTGTCATTGGTGTCTCCTAGGGGAATAAATCTTTGGGCACCTAGTGGTCATG

2 CATGACCACTAGGAGCATCTTTGGCGACATGTGGCAATCCTGGCCTCTGGCAAGCGCCTAGGGGAATAAATCTTTGGGCACCTAGTGGTCATG

2 CATGACCACTAGGAGCATCTTTGGCGACCAGGGAGCGAGAGCGAACCATGTGTGGAGCTAGGGGAATAAATCTTTGGGCACCTAGTGGTCATG

2 CATGACCACTAGGAGCATCTTTGGCGAGCGGGTGGGCATGCGTCAGGGGAGGCTCGACTAGGGGAATAAATCTTTGGGCACCTAGTGGTCATG

2 CATGACCACTAGGAGCATCTTTGGCGAGGATACGTCATAGGTGCGTGTGGTCGAGAACTAGGGGAATAAATCTTTGGGCACCTAGTGGTCATG

2 CATGACCACTAGGAGCATCTTTGGCGAACGCGTAGGAGCAGGATCCTAAAGGTAGATCTAGGGGAATAAATCTTTGGGCACCTAGTGGTCATG

2 CATGACCACTAGGAGCATCTTTGGCGACCCGGATGAAAGGTACCGCATCTGGGTGGACTAGGGGAATAAATCTTTGGGCACCTAGTGGTCATG

2 CATGACCACTAGGAGCATCTTTGGCGACGAAAGGTGGTCGGCTACGGAGAAGGTCGCCTAGGGGAATAAATCTTTGGGCACCTAGTGGTCATG

2 CATGACCACTAGGAGCATCTTTGGCGAAAGTCGGTAGAACAGGGTGGGGTGCGGTCCCTAGGGGAATAAATCTTTGGGCACCTAGTGGTCATG

2 CATGACCACTAGGAGCATCTTTGGCGACACGCACGCTGATTGAGGCCGCAGAGGCGACTAGGGGAATAAATCTTTGGGCACCTAGTGGTCATG

2 CATGACCACTAGGAGCATCTTTGGCGAAAGACTGCGATGATACGAAACGTTATTCGCCTAGGGGAATAAATCTTTGGGCACCTAGTGGTCATG

2 CATGACCACTAGGAGCATCTTTGGCGACGGGGGCAGTGCCAGGAAAGTTCGCGTCGACTAGGGGAATAAATCTTTGGGCACCTAGTGGTCATG

2 CATGACCACTAGGAGCATCTTTGGCGAGTGGCCTAAGTGCGTATCTGTGGTCGAGCTCTAGGGGAATAAATCTTTGGGCACCTAGTGGTCATG

2 CATGACCACTAGGAGCATCTTTGGCGATTTGGGAGTTGCAGTATGCAGGCAGTGAGACTAGGGGAATAAATCTTTGGGCACCTAGTGGTCATG

2 CATGACCACTAGGAGCATCTTTGGCGACCGCTTATAGCGAGGTCACTGGTCTGCGCCCTAGGGGAATAAATCTTTGGGCACCTAGTGGTCATG

2 CATGACCACTAGGAGCATCTTTGGCGAGATCGGGGGAATCGGTGGCATTGGTGTCACCTAGGGGAATAAATCTTTGGGCACCTAGTGGTCATG

2 CATGACCACTAGGAGCATCTTTGGCGATGGCGCCGGGGCTTGCCAGACCTCCGCGGCCTAGGGGAATAAATCTTTGGGCACCTAGTGGTCATG

2 CATGACCACTAGGAGCATCTTTGGCGAGAAGACTCCGGATTCGGGGACCAGTTGCCGCTAGGGGAATAAATCTTTGGGCACCTAGTGGTCATG

2 CATGACCACTAGGAGCATCTTTGGCGAAGCGACATGGGGCACACGGGCGAGTCTGAGCTAGGGGAATAAATCTTTGGGCACCTAGTGGTCATG

2 CATGACCACTAGGAGCATCTTTGGCGACATCGATTGCCGTACTGCAAGCGTTGTGGACTAGGGGAATAAATCTTTGGGCACCTAGTGGTCATG

2 CATGACCACTAGGAGCATCTTTGGCGAGCCGATTGTGCTACAATGTGTTCTGGGTAACTAGGGGAATAAATCTTTGGGCACCTAGTGGTCATG

2 CATGACCACTAGGAGCATCTTTGGCGACAATGTTAATGTGGTATGATGCTTAGAATTCTAGGGGAATAAATCTTTGGGCACCTAGTGGTCATG

2 CATGACCACTAGGAGCATCTTTGGCGACCCACATCGAAGGACTGTGGCCGTGGCGCTCTAGGGGAATAAATCTTTGGGCACCTAGTGGTCATG

2 CATGACCACTAGGAGCATCTTTGGCGACTCCAATCGGCTTGATAGTTGTGAGATCGACTAGGGGAATAAATCTTTGGGCACCTAGTGGTCATG

2 CATGACCACTAGGAGCATCTTTGGCGACAATGGGGCTGGTAGGAAGGTACTTATAGCCTAGGGGAATAAATCTTTGGGCACCTAGTGGTCATG

2 CATGACCACTAGGAGCATCTTTGGCGACGACGTGGTCGTGGTGATGTGGCCGAGTCGCTAGGGGAATAAATCTTTGGGCACCTAGTGGTCATG

2 CATGACCACTAGGAGCATCTTTGGCGACTGTGACAACGCAGACGAAGTGGGAAGCGCCTAGGGGAATAAATCTTTGGGCACCTAGTGGTCATG

2 CATGACCACTAGGAGCATCTTTGGCGATGGGCGTGTACGGTCGAGCGAAAGCGATAGCTAGGGGAATAAATCTTTGGGCACCTAGTGGTCATG

2 CATGACCACTAGGAGCATCTTTGGCGACGCAGTGGGGCCTGGTCACTATGGCGGAGACTAGGGGAATAAATCTTTGGGCACCTAGTGGTCATG

2 CATGACCACTAGGAGCATCTTTGGCGAGGGTGGGCGCCGGCAGCGGTGTGAATGTGCCTAGGGGAATAAATCTTTGGGCACCTAGTGGTCATG

2 CATGACCACTAGGAGCATCTTTGGCGAGATCGGGAGAATCGGTAACATTGGTGTCTCCTAGGGGAATAAATCTTTGGGCACCTAGTGGTCATG

2 CATGACCACTAGGAGCATCTTTGGCGATCCAACTATGCACGTGGTTGGTGTGGAAGCCTAGGGGAATAAATCTTTGGGCACCTAGTGGTCATG

2 CATGACCACTAGGAGCATCTTTGGCGACGCGCAGGGGACCGTGTGTACGGACGTCGACTAGGGGAATAAATCTTTGGGCACCTAGTGGTCATG

2 CATGACCACTAGGAGCATCTTTGGCGACGGGGGGTCAGGGGGTGTTGCGTACGGAGCCTAGGGGAATAAATCTTTGGGCACCTAGTGGTCATG

2 CATGACCACTAGGAGCATCTTTGGCGAAACCCATGTAATAGACGCCCGGGGTCTCTACTAGGGGAATAAATCTTTGGGCACCTAGTGGTCATG

2 CATGACCACTAGGAGCATCTTTGGCGATGGGTTCCGGAGATATGCCGAGCCTGAAGCCTAGGGGAATAAATCTTTGGGCACCTAGTGGTCATG

2 CATGACCACTAGGAGCATCTTTGGCGACCCTCAAGAGCGCAAGCGCAAAAGGGTGGACTAGGGGAATAAATCTTTGGGCACCTAGTGGTCATG

2 CATGACCACTAGGAGCATCTTTGGCGACGTGGGCAGTTTGAGGAGAATTAGTCGCCGCTAGGGGAATAAATCTTTGGGCACCTAGTGGTCATG

2 CATGACCACTAGGAGCATCTTTGGCGATCGGGTACCATCAACCTAAGAGGCGAGCGACTAGGGGAATAAATCTTTGGGCACCTAGTGGTCATG

2 CATGACCACTAGGAGCATCTTTGGCGAAAACCGCTCGTAGGGTGAGCAGACGTTGGGCTAGGGGAATAAATCTTTGGGCACCTAGTGGTCATG

2 CATGACCACTAGGAGCATCTTTGGCGATGGGTGCTGACGGGGCTAGGGAGCCCTACACTAGGGGAATAAATCTTTGGGCACCTAGTGGTCATG

2 CATGACCACTAGGAGCATCTTTGGCGACGAGGACAGGTTGGTACGATCGGGTGGCGCCTAGGGGAATAAATCTTTGGGCACCTAGTGGTCATG

2 CATGACCACTAGGAGCATCTTTGGCGACGACGGGTGGTCGGCATTGCTGAAGAGTCGCTAGGGGAATAAATCTTTGGGCACCTAGTGGTCATG

2 CATGACCACTAGGAGCATCTTTGGCGAGGTAAACACAGCTGGGATGTGGTAGTTCGCCTAGGGGAATAAATCTTTGGGCACCTAGTGGTCATG

2 CATGACCACTAGGAGCATCTTTGGCGAGGGGACAAGGGACGGTAATGAGGGCCGAGACTAGGGGAATAAATCTTTGGGCACCTAGTGGTCATG

2 CATGACCACTAGGAGCATCTTTGGCGACCCGCTCGGGCTACCGAAAGGAGTGTTGCTCTAGGGGAATAAATCTTTGGGCACCTAGTGGTCATG

2 CATGACCACTAGGAGCATCTTTGGCGAACCAGGAGGAGAGGTTATGAAAGTGTGAGACTAGGGGAATAAATCTTTGGGCACCTAGTGGTCATG

2 CATGACCACTAGGAGCATCTTTGGCGATGAGGTATGGGCGTCATGTTGGTAGGAGCTCTAGGGGAATAAATCTTTGGGCACCTAGTGGTCATG

2 CATGACCACTAGGAGCATCTTTGGCGAACGTGCGAGCACATTGTATGACGTCAGGGCCTAGGGGAATAAATCTTTGGGCACCTAGTGGTCATG

2 CATGACCACTAGGAGCATCTTTGGCGACGGCGTGGGGATATTGATGCGGCGGCACTCCTAGGGGAATAAATCTTTGGGCACCTAGTGGTCATG

2 CATGACCACTAGGAGCATCTTTGGCGAGTACGTGGGCGGGAATAGTACGTTGCGAGACTAGGGGAATAAATCTTTGGGCACCTAGTGGTCATG

2 CATGACCACTAGGAGCATCTTTGGCGAGATCTGGAGAATCGGAGGCATTGGTGTCTCCTAGGGGAATAAATCTTTGGGCACCTAGTGGTCATG

2 CATGACCACTAGGAGCATCTTTGGCGAAGCGTGTTGCAAGGTCCGAGAGTGTGGACCCTAGGGGAATAAATCTTTGGGCACCTAGTGGTCATG

2 CATGACCACTAGGAGCATCTTTGGCGAACGGCGGGTGTTGCGGTGGTCTGTGTATCCCTAGGGGAATAAATCTTTGGGCACCTAGTGGTCATG

2 CATGACCACTAGGAGCATCTTTGGCGAATCGCGGATCGCGACGAAGTGTAAATGAGACTAGGGGAATAAATCTTTGGGCACCTAGTGGTCATG

2 CATGACCACTAGGAGCATCTTTGGCGACTACGACGGGAGTGCCAGGCAAGGAGGCGACTAGGGGAATAAATCTTTGGGCACCTAGTGGTCATG

2 CATGACCACTAGGAGCATCTTTGGCGATCGTACGGGATCGGTTGAGGGGCCGCGAGCCTAGGGGAATAAATCTTTGGGCACCTAGTGGTCATG

2 CATGACCACTAGGAGCATCTTTGGCGAAAGTCGGTAGAAGAGGGTGGGGTGCTGTCCCTAGGGGAATAAATCTTTGGGCACCTAGTGGTCATG

2 CATGACCACTAGGAGCATCTTTGGCGAGATCGGGAGACTCGGTGGCATTGGTGTATCCTAGGGGAATAAATCTTTGGGCACCTAGTGGTCATG

2 CATGACCACTAGGAGCATCTTTGGCGACGACGGGTCAGGGCGTGACGATAATACTAGCTAGGGGAATAAATCTTTGGGCACCTAGTGGTCATG

2 CATGACCACTAGGAGCATCTTTGGCGAGGAACTTCGCTAAGGGTTATCGGTCCGAGACTAGGGGAATAAATCTTTGGGCACCTAGTGGTCATG

2 CATGACCACTAGGAGCATCTTTGGCGAGAGGTGGTCTTGCGGACGTCCACCCGAAGACTAGGGGAATAAATCTTTGGGCACCTAGTGGTCATG

2 CATGACCACTAGGAGCATCTTTGGCGAGTGTGGCGTAGCAGGTTAGTAATAAGTAGCCTAGGGGAATAAATCTTTGGGCACCTAGTGGTCATG

2 CATGACCACTAGGAGCATCTTTGGCGAAGCCGGAGAGTGATCTATAGTGGCTGAGACCTAGGGGAATAAATCTTTGGGCACCTAGTGGTCATG

2 CATGACCACTAGGAGCATCTTTGGCGAGAGGGGTTAGGCTGGAGCGCTGGTGGGGGACTAGGGGAATAAATCTTTGGGCACCTAGTGGTCATG

2 CATGACCACTAGGAGCATCTTTGGCGAGGTCCAGAGATGTGTACTGTGGCTGAGCAACTAGGGGAATAAATCTTTGGGCACCTAGTGGTCATG

2 CATGACCACTAGGAGCATCTTTGGCGAGGTGATTCGGTAGGAAAGAGTGCACCCCGACTAGGGGAATAAATCTTTGGGCACCTAGTGGTCATG

2 CATGACCACTAGGAGCATCTTTGGCGAGATCTTAACGAAGTGAATAGCGGCGGGGGCCTAGGGGAATAAATCTTTGGGCACCTAGTGGTCATG

2 CATGACCACTAGGAGCATCTTTGGCGACCCGAACAGACGCGGTCAGGTACGGGTGGACTAGGGGAATAAATCTTTGGGCACCTAGTGGTCATG

2 CATGACCACTAGGAGCATCTTTGGCGACGGTGTGGGGAACTAGTTTCGGCGGTGCTACTAGGGGAATAAATCTTTGGGCACCTAGTGGTCATG

2 CATGACCACTAGGAGCATCTTTGGCGATCGAACGAACCCGCGAAGGAATAGGGTAGCCTAGGGGAATAAATCTTTGGGCACCTAGTGGTCATG

2 CATGACCACTAGGAGCATCTTTGGCGACCAGCCTAGGTACCAGGAAAAAAAGGGCGACTAGGGGAATAAATCTTTGGGCACCTAGTGGTCATG

2 CATGACCACTAGGAGCATCTTTGGCGACGGCGTGGGGCCTCTAGTGCGGCGGGGATCCTAGGGGAATAAATCTTTGGGCACCTAGTGGTCATG

2 CATGACCACTAGGAGCATCTTTGGCGAGCCCCAAGCGCCGTGCGGGAGGTGTGTAGCCTAGGGGAATAAATCTTTGGGCACCTAGTGGTCATG

2 CATGACCACTAGGAGCATCTTTGGCGATCAGACGGGGTAGGCAGCTACAGAGATCGACTAGGGGAATAAATCTTTGGGCACCTAGTGGTCATG

2 CATGACCACTAGGAGCATCTTTGGCGACGTGGGCTGGTAGGATGTGGTACTTCTCGACTAGGGGAATAAATCTTTGGGCACCTAGTGGTCATG

2 CATGACCACTAGGAGCATCTTTGGCGAGGTGCGCAGATGTGAAGCAAGCAAGTCGGACTAGGGGAATAAATCTTTGGGCACCTAGTGGTCATG

2 CATGACCACTAGGAGCATCTTTGGCGAAAGTCGGTAGAGCAGGGTGGGGTGCTGCCCCTAGGGGAATAAATCTTTGGGCACCTAGTGGTCATG

2 CATGACCACTAGGAGCATCTTTGGCGACGCGGGGTGTCTCAAACATCACGACGTGGACTAGGGGAATAAATCTTTGGGCACCTAGTGGTCATG

2 CATGACCACTAGGAGCATCTTTGGCGACCCGTCTGCCTGGAAGGAGTGACGGGTGGACTAGGGGAATAAATCTTTGGGCACCTAGTGGTCATG

2 CATGACCACTAGGAGCATCTTTGGCGAACGGCAGGTGTTGCGGTGGTTTGTGAGTCCCTAGGGGAATAAATCTTTGGGCACCTAGTGGTCATG

2 CATGACCACTAGGAGCATCTTTGGCGATGAGTGGGCGCAGATCCCAAAACAGTACGCCTAGGGGAATAAATCTTTGGGCACCTAGTGGTCATG

2 CATGACCACTAGGAGCATCTTTGGCGACGAGGGGGGCAGCGATATCTGAAAAGTCGACTAGGGGAATAAATCTTTGGGCACCTAGTGGTCATG

2 CATGACCACTAGGAGCATCTTTGGCGACTGGGGTCCGATGCACCTGCCTACAGGCGACTAGGGGAATAAATCTTTGGGCACCTAGTGGTCATG

2 CATGACCACTAGGAGCATCTTTGGCGATAGGCATGTACGGTCGAGCGAAAGCGATAGCTAGGGGAATAAATCTTTGGGCACCTAGTGGTCATG

2 CATGACCACTAGGAGCATCTTTGGCGACCCGGCACCAGTCGGTGCTGAGAGTGGAGACTAGGGGAATAAATCTTTGGGCACCTAGTGGTCATG

2 CATGACCACTAGGAGCATCTTTGGCGAGTATTGGCTGGTAGGTTGCGTATGGGGGAGCTAGGGGAATAAATCTTTGGGCACCTAGTGGTCATG

2 CATGACCACTAGGAGCATCTTTGGCGAGATTGGGAGAATCGGTGGCATTAGTGTCTCCTAGGGGAATAAATCTTTGGGCACCTAGTGGTCATG

2 CATGACCACTAGGAGCATCTTTGGCGACACCGCTACTGTGGATACGGGGGGGGACGACTAGGGGAATAAATCTTTGGGCACCTAGTGGTCATG

2 CATGACCACTAGGAGCATCTTTGGCGACCCGAGCGGGAAGGGCCTAGATCGGGTGGACTAGGGGAATAAATCTTTGGGCACCTAGTGGTCATG

2 CATGACCACTAGGAGCATCTTTGGCGAGTTTGTAGCTCGCGCGTAGGGTCGGCAGTGCTAGGGGAATAAATCTTTGGGCACCTAGTGGTCATG

2 CATGACCACTAGGAGCATCTTTGGCGACGGTGTGGAATACGCCTGTAGGCGGACATGCTAGGGGAATAAATCTTTGGGCACCTAGTGGTCATG

2 CATGACCACTAGGAGCATCTTTGGCGATGGGGCCGTGCTATACGCACACTCCGCGGGCTAGGGGAATAAATCTTTGGGCACCTAGTGGTCATG

2 CATGACCACTAGGAGCATCTTTGGCGAGGACAGGTGGCGTGTCAGCCGGGGTGGTAGCTAGGGGAATAAATCTTTGGGCACCTAGTGGTCATG

2 CATGACCACTAGGAGCATCTTTGGCGAGGCGATTGTGCAACAATGTGTTCTGGGTAACTAGGGGAATAAATCTTTGGGCACCTAGTGGTCATG

2 CATGACCACTAGGAGCATCTTTGGCGAGTGGGGGGAGACGGCCGGTAGTGGGTGAGACTAGGGGAATAAATCTTTGGGCACCTAGTGGTCATG

2 CATGACCACTAGGAGCATCTTTGGCGAGGGGTGCTGACGGGGCTAGTGAGCCCTACACTAGGGGAATAAATCTTTGGGCACCTAGTGGTCATG

2 CATGACCACTAGGAGCATCTTTGGCGAGAGCGTCTGGCGCAGTCGGAAGGCCCGAGCCTAGGGGAATAAATCTTTGGGCACCTAGTGGTCATG

2 CATGACCACTAGGAGCATCTTTGGCGACCCGCTTGCGAGTGGCTAAAGTCGGAACGCCTAGGGGAATAAATCTTTGGGCACCTAGTGGTCATG

2 CATGACCACTAGGAGCATCTTTGGCGACGAGCAACATCAAGGTGCGGGCGGGATGTCCTAGGGGAATAAATCTTTGGGCACCTAGTGGTCATG

2 CATGACCACTAGGAGCATCTTTGGCGATCCGGATCGGCACTATGTGCTCGGTGGATCCTAGGGGAATAAATCTTTGGGCACCTAGTGGTCATG

2 CATGACCACTAGGAGCATCTTTGGCGATGTGTGGGCTGGTAGGTTAGTATTAAGGAGCTAGGGGAATAAATCTTTGGGCACCTAGTGGTCATG

2 CATGACCACTAGGAGCATCTTTGGCGACAATTGTATGCGTTATCAGAGTGGTCGACACTAGGGGAATAAATCTTTGGGCACCTAGTGGTCATG

2 CATGACCACTAGGAGCATCTTTGGCGACGTGGTCACTGGGAATGGCGCTGGCGTCGACTAGGGGAATAAATCTTTGGGCACCTAGTGGTCATG

2 CATGACCACTAGGAGCATCTTTGGCGAACCCATATGAATGCGCAGCATGGGTGTGGACTAGGGGAATAAATCTTTGGGCACCTAGTGGTCATG

2 CATGACCACTAGGAGCATCTTTGGCGACCCGTGCTCGGATACCGAGCCGAGTGTGGCCTAGGGGAATAAATCTTTGGGCACCTAGTGGTCATG

2 CATGACCACTAGGAGCATCTTTGGCGAGACGACCCTGTCGTTGGGGACCAGTTGCTGCTAGGGGAATAAATCTTTGGGCACCTAGTGGTCATG

2 CATGACCACTAGGAGCATCTTTGGCGACCCATGATTGGAATCAAGGGAGTGGGAGCTCTAGGGGAATAAATCTTTGGGCACCTAGTGGTCATG

2 CATGACCACTAGGAGCATCTTTGGCGAATGGCCGGTACGGCCCTCTAGTCCTCGGTACTAGGGGAATAAATCTTTGGGCACCTAGTGGTCATG

2 CATGACCACTAGGAGCATCTTTGGCGAGGCTACAGTGGAAGCAGAGCAGACGTTTGGCTAGGGGAATAAATCTTTGGGCACCTAGTGGTCATG

2 CATGACCACTAGGAGCATCTTTGGCGACTACGGACGGCGTGCTCCCAAGTGGTCGCTCTAGGGGAATAAATCTTTGGGCACCTAGTGGTCATG

2 CATGACCACTAGGAGCATCTTTGGCGATGGGGCCGTGCTATCTGAACACTCCGCGGGCTAGGGGAATAAATCTTTGGGCACCTAGTGGTCATG

2 CATGACCACTAGGAGCATCTTTGGCGACCGTGAACGGGTCGGTGTGCAGGAGGGCGACTAGGGGAATAAATCTTTGGGCACCTAGTGGTCATG

2 CATGACCACTAGGAGCATCTTTGGCGATGGGGCAGTGCTATCTGCACACTCCGCGGGCTAGGGGAATAAATCTTTGGGCACCTAGTGGTCATG

2 CATGACCACTAGGAGCATCTTTGGCGAGTCGAGGATGGGATCGGCGTGAGTCTTCGCCTAGGGGAATAAATCTTTGGGCACCTAGTGGTCATG

2 CATGACCACTAGGAGCATCTTTGGCGAGTGGATAGTCCAAGGCAGGTCCGAACTCGACTAGGGGAATAAATCTTTGGGCACCTAGTGGTCATG

2 CATGACCACTAGGAGCATCTTTGGCGAGCCTCGCTTGGGAAGTTGCTCCACCAGTTCCTAGGGGAATAAATCTTTGGGCACCTAGTGGTCATG

2 CATGACCACTAGGAGCATCTTTGGCGACGGCGTGGGGCCATCTGTGCGGCTGATCCCCTAGGGGAATAAATCTTTGGGCACCTAGTGGTCATG

2 CATGACCACTAGGAGCATCTTTGGCGAGTCGGCCCGGTCGTACGAGCAGACGTTTGGCTAGGGGAATAAATCTTTGGGCACCTAGTGGTCATG

2 CATGACCACTAGGAGCATCTTTGGCGACATGGATCATAGTAGATTCAGATAGTGGAGCTAGGGGAATAAATCTTTGGGCACCTAGTGGTCATG

2 CATGACCACTAGGAGCATCTTTGGCGATGACCAGGGACCGCGGCCCTGTGGTGGAGACTAGGGGAATAAATCTTTGGGCACCTAGTGGTCATG

2 CATGACCACTAGGAGCATCTTTGGCGACACGAGCAGCAATATAGCGATCGTTGTGGACTAGGGGAATAAATCTTTGGGCACCTAGTGGTCATG

2 CATGACCACTAGGAGCATCTTTGGCGACCCCGTATATCGACGTTATACCGGGGTGGACTAGGGGAATAAATCTTTGGGCACCTAGTGGTCATG

2 CATGACCACTAGGAGCATCTTTGGCGAACGCGTTAGATCGGGTGCTCTAGTGAAGATCTAGGGGAATAAATCTTTGGGCACCTAGTGGTCATG

2 CATGACCACTAGGAGCATCTTTGGCGATACGGCCCGTACAACGGCGAGCGTAGCGATCTAGGGGAATAAATCTTTGGGCACCTAGTGGTCATG

2 CATGACCACTAGGAGCATCTTTGGCGACAGTAAAGAAGCGATCTCCAAGATTGGAGCCTAGGGGAATAAATCTTTGGGCACCTAGTGGTCATG

2 CATGACCACTAGGAGCATCTTTGGCGACCCAATCGGTGCCTGGCACCACTGGGTGGACTAGGGGAATAAATCTTTGGGCACCTAGTGGTCATG

2 CATGACCACTAGGAGCATCTTTGGCGATGGAAACGCCGAGGCTCGCTCCGTGCATCGCTAGGGGAATAAATCTTTGGGCACCTAGTGGTCATG

1 CATGACCACTAGGAGCATCTTTGGCGACGGAGAGCGCAAGGGGTGGCCACGAGTCGCCTAGGGGAATAAATCTTTGGGCACCTAGTGGTCATG

1 CATGACCACTAGGAGCATCTTTGGCGACTCGTTGTCGGTAGCACGCAACGTAGTGGACTAGGGGAATAAATCTTTGGGCACCTAGTGGTCATG

1 CATGACCACTAGGAGCATCTTTGGCGAAATGGGACGCCTCGCCGTTGGAAGATACGCCTAGGGGAATAAATCTTTGGGCACCTAGTGGTCATG

1 CATGACCACTAGGAGCATCTTTGGCGACGTGGTGCGTCACGTAGGGGTTTCCGTCGACTAGGGGAATAAATCTTTGGGCACCTAGTGGTCATG

1 CATGACCACTAGGAGCATCTTTGGCGATCCGTCGTACGGTACGACCGGAGGTTGAGACTAGGGGAATAAATCTTTGGGCACCTAGTGGTCATG

1 CATGACCACTAGGAGCATCTTTGGCGACTCGTGGAGCTAGGGACCAAGAAGAGGCGACTAGGGGAATAAATCTTTGGGCACCTAGTGGTCATG

1 CATGACCACTAGGAGCATCTTTGGCGATGACGTGGGCTCTAGTGTCAAGAATCGCCTCTAGGGGAATAAATCTTTGGGCACCTAGTGGTCATG

1 CATGACCACTAGGAGCATCTTTGGCGAAAGGGGAACGGCAGGGACCGTCAGATTCGACTAGGGGAATAAATCTTTGGGCACCTAGTGGTCATG

1 CATGACCACTAGGAGCATCTTTGGCGAGAAGACTCAGAATTCGGGGACCAGTTGCTGCTAGGGGAATAAATCTTTGGGCACCTAGTGGTCATG

1 CATGACCACTAGGAGCATCTTTGGCGACACTGTGGGCCATGCTTTGCGAGGGATCGCCTAGGGGAATAAATCTTTGGGCACCTAGTGGTCATG

1 CATGACCACTAGGAGCATCTTTGGCGAGCCGGAGAGGTCACGGAACGAGGTGGTCGCCTAGGGGAATAAATCTTTGGGCACCTAGTGGTCATG

1 CATGACCACTAGGAGCATCTTTGGCGAGTGTGGTCAGCCGGCCCGCAAAAACCGAGCCTAGGGGAATAAATCTTTGGGCACCTAGTGGTCATG

1 CATGACCACTAGGAGCATCTTTGGCGAGGCCGTGGAAGACGGATTCCGCCGCGTCGCCTAGGGGAATAAATCTTTGGGCACCTAGTGGTCATG

1 CATGACCACTAGGAGCATCTTTGGCGACTCAGTACAAGGCGGGCTAAGTACGATAGCCTAGGGGAATAAATCTTTGGGCACCTAGTGGTCATG

1 CATGACCACTAGGAGCATCTTTGGCGAGTACAGCGAGAGTGGCCGAGCTAGGAGCTGCTAGGGGAATAAATCTTTGGGCACCTAGTGGTCATG

1 CATGACCACTAGGAGCATCTTTGGCGAATGAGGAGCAAAGGCCAGTGCGAATGGAGACTAGGGGAATAAATCTTTGGGCACCTAGTGGTCATG

1 CATGACCACTAGGAGCATCTTTGGCGAGTCGGAGACTTTGCGGTTGACACAGAGCGACTAGGGGAATAAATCTTTGGGCACCTAGTGGTCATG

1 CATGACCACTAGGAGCATCTTTGGCGACGGAGGTACACCGGGGACACAGATAGTCGACTAGGGGAATAAATCTTTGGGCACCTAGTGGTCATG

1 CATGACCACTAGGAGCATCTTTGGCGAGAAGAATCTGGATTCGTGGACCAGTTGCTGCTAGGGGAATAAATCTTTGGGCACCTAGTGGTCATG

1 CATGACCACTAGGAGCATCTTTGGCGACCCAGCGTAGGTACCCCGCAAATGGGTGGACTAGGGGAATAAATCTTTGGGCACCTAGTGGTCATG

1 CATGACCACTAGGAGCATCTTTGGCGACGAGGGCGAGATAATATGGTGATAAAGGAGCTAGGGGAATAAATCTTTGGGCACCTAGTGGTCATG

1 CATGACCACTAGGAGCATCTTTGGCGACGTAAGTGGGAAGTGTTGCTAGGCGAGTCGCTAGGGGAATAAATCTTTGGGCACCTAGTGGTCATG

1 CATGACCACTAGGAGCATCTTTGGCGATGTGGTAGGATCAGTCGAAACGGGAAACGCCTAGGGGAATAAATCTTTGGGCACCTAGTGGTCATG

1 CATGACCACTAGGAGCATCTTTGGCGAGCCACGAAAGAAAGCAGTCCTGTAGCGAGACTAGGGGAATAAATCTTTGGGCACCTAGTGGTCATG

1 CATGACCACTAGGAGCATCTTTGGCGAAGAGAACCGAGCGAATGATGGGGGACATCACTAGGGGAATAAATCTTTGGGCACCTAGTGGTCATG

1 CATGACCACTAGGAGCATCTTTGGCGACGACGGGCGGGGATCTGGGATGCGTCGAGACTAGGGGAATAAATCTTTGGGCACCTAGTGGTCATG

1 CATGACCACTAGGAGCATCTTTGGCGACCCACCCGGGAAAAACGCGGGTGAGGTGGACTAGGGGAATAAATCTTTGGGCACCTAGTGGTCATG

1 CATGACCACTAGGAGCATCTTTGGCGAGGATGCAGAAGACCACAAGAATTGTCCTCGCTAGGGGAATAAATCTTTGGGCACCTAGTGGTCATG

1 CATGACCACTAGGAGCATCTTTGGCGAACTAGGGTCCGTCCGCGGATGGGAAAGCGACTAGGGGAATAAATCTTTGGGCACCTAGTGGTCATG

1 CATGACCACTAGGAGCATCTTTGGCGAGGAGGGGTCTACTGGTGGGCACCACGCAGACTAGGGGAATAAATCTTTGGGCACCTAGTGGTCATG

1 CATGACCACTAGGAGCATCTTTGGCGAAACAGACGGCTTGTCTCGTGCTAGTGTAGACTAGGGGAATAAATCTTTGGGCACCTAGTGGTCATG

1 CATGACCACTAGGAGCATCTTTGGCGACGGTACAAAGCAATGCAGATCGATAGTCGACTAGGGGAATAAATCTTTGGGCACCTAGTGGTCATG

1 CATGACCACTAGGAGCATCTTTGGCGAGGTTGTAGCTCGCGCGAAGGGTCGGCAGTGCTAGGGGAATAAATCTTTGGGCACCTAGTGGTCATG

1 CATGACCACTAGGAGCATCTTTGGCGATAGCGCATGCTCCGTCGGACGACGTTAGCACTAGGGGAATAAATCTTTGGGCACCTAGTGGTCATG

1 CATGACCACTAGGAGCATCTTTGGCGACAAACGCGCAGCACGGGGGGAGATAGTCGACTAGGGGAATAAATCTTTGGGCACCTAGTGGTCATG

1 CATGACCACTAGGAGCATCTTTGGCGACCCGACAAGACTCTGGTACGTCGCGGTGGACTAGGGGAATAAATCTTTGGGCACCTAGTGGTCATG

1 CATGACCACTAGGAGCATCTTTGGCGACTGGCACGGATGGCAATGGGTTCCTAGGAGCTAGGGGAATAAATCTTTGGGCACCTAGTGGTCATG

1 CATGACCACTAGGAGCATCTTTGGCGAGGTCGGGAGAATCGGTGGCATTGGTGTCACCTAGGGGAATAAATCTTTGGGCACCTAGTGGTCATG

1 CATGACCACTAGGAGCATCTTTGGCGAGAACGGGAGAATCGGTGGCATTGGTGGCTCCTAGGGGAATAAATCTTTGGGCACCTAGTGGTCATG

1 CATGACCACTAGGAGCATCTTTGGCGAGTGGGTCATGAAGTTATGGGATGAACTCGACTAGGGGAATAAATCTTTGGGCACCTAGTGGTCATG

1 CATGACCACTAGGAGCATCTTTGGCGACTCGTCGAGGGACGGTTGGCGGTGAGGCGACTAGGGGAATAAATCTTTGGGCACCTAGTGGTCATG

1 CATGACCACTAGGAGCATCTTTGGCGACGTCTGTGGGCTGTGGTTATGGCGGACTCGCTAGGGGAATAAATCTTTGGGCACCTAGTGGTCATG

1 CATGACCACTAGGAGCATCTTTGGCGATCGGAACGAGAGCGACAGTATGTCCAAGCGCTAGGGGAATAAATCTTTGGGCACCTAGTGGTCATG

1 CATGACCACTAGGAGCATCTTTGGCGACCCGATGGGATACAAGACAGCTCGGGTGGACTAGGGGAATAAATCTTTGGGCACCTAGTGGTCATG

1 CATGACCACTAGGAGCATCTTTGGCGACACGATGGGTAAATAGAGGCACCGAGGCGACTAGGGGAATAAATCTTTGGGCACCTAGTGGTCATG

1 CATGACCACTAGGAGCATCTTTGGCGACGAGTGGGTAAATGGAGGCAAGTCTGTCGCCTAGGGGAATAAATCTTTGGGCACCTAGTGGTCATG

1 CATGACCACTAGGAGCATCTTTGGCGATAGCGGCGTCTCCCGCCGAAGGTCTATGGCCTAGGGGAATAAATCTTTGGGCACCTAGTGGTCATG

1 CATGACCACTAGGAGCATCTTTGGCGAACAGGCCGTGGCTGCGGGTGGGAGCGCACCCTAGGGGAATAAATCTTTGGGCACCTAGTGGTCATG

1 CATGACCACTAGGAGCATCTTTGGCGAGCCTTGATTGGGAGGTTGCTCCACCAGTTCCTAGGGGAATAAATCTTTGGGCACCTAGTGGTCATG

1 CATGACCACTAGGAGCATCTTTGGCGAGAGCTAGAAAAAGGTGCGTTGTTGTCGCATCTAGGGGAATAAATCTTTGGGCACCTAGTGGTCATG

1 CATGACCACTAGGAGCATCTTTGGCGATAGGGGGTCACTCAGGAAGAGAGGATTCGCCTAGGGGAATAAATCTTTGGGCACCTAGTGGTCATG

1 CATGACCACTAGGAGCATCTTTGGCGACGTGGCATATCAGCAATTGAGGACATTAGGCTAGGGGAATAAATCTTTGGGCACCTAGTGGTCATG

1 CATGACCACTAGGAGCATCTTTGGCGAGGCAAACGAACAGCATAGGGCTGTAAGATACTAGGGGAATAAATCTTTGGGCACCTAGTGGTCATG

1 CATGACCACTAGGAGCATCTTTGGCGATGGGTGCTGACGGGGCAAGTGAGCCCTACACTAGGGGAATAAATCTTTGGGCACCTAGTGGTCATG

1 CATGACCACTAGGAGCATCTTTGGCGAGATCGGGAGAATCTGTGGCATTGGTGTCGCCTAGGGGAATAAATCTTTGGGCACCTAGTGGTCATG

1 CATGACCACTAGGAGCATCTTTGGCGACGACGTGGCACTGAGGGAGGTCAGAGGAGACTAGGGGAATAAATCTTTGGGCACCTAGTGGTCATG

1 CATGACCACTAGGAGCATCTTTGGCGACTGTGGGAGAGAGTAGGGTAACAGGAGACGCTAGGGGAATAAATCTTTGGGCACCTAGTGGTCATG

1 CATGACCACTAGGAGCATCTTTGGCGACGCAGTACGTAAACCCGACACAGAAGGCGACTAGGGGAATAAATCTTTGGGCACCTAGTGGTCATG

1 CATGACCACTAGGAGCATCTTTGGCGACATGGTGGTTAGGGTACGTAGGGGTGACGCCTAGGGGAATAAATCTTTGGGCACCTAGTGGTCATG

1 CATGACCACTAGGAGCATCTTTGGCGAGGTTGTATCTCGCGCGTAGGGTCGGCAGTGCTAGGGGAATAAATCTTTGGGCACCTAGTGGTCATG

1 CATGACCACTAGGAGCATCTTTGGCGAGATGGTCCGACCAAACGCAATAGGACGAGCCTAGGGGAATAAATCTTTGGGCACCTAGTGGTCATG

1 CATGACCACTAGGAGCATCTTTGGCGAACACGAGAGTCAAGTGCGTATAGCGAGGAGCTAGGGGAATAAATCTTTGGGCACCTAGTGGTCATG

1 CATGACCACTAGGAGCATCTTTGGCGACGAGAGCGTCAAGACACGCACACAGACAGCCTAGGGGAATAAATCTTTGGGCACCTAGTGGTCATG

1 CATGACCACTAGGAGCATCTTTGGCGAATCGACCGGAATCCGAAATACGTGATGAGACTAGGGGAATAAATCTTTGGGCACCTAGTGGTCATG

1 CATGACCACTAGGAGCATCTTTGGCGACTGGAACGGGCTCTAGCGGAGGATCGTCGACTAGGGGAATAAATCTTTGGGCACCTAGTGGTCATG

1 CATGACCACTAGGAGCATCTTTGGCGACTAGTGGGACAGGAAGGCTCCGGAAGTCGCCTAGGGGAATAAATCTTTGGGCACCTAGTGGTCATG

1 CATGACCACTAGGAGCATCTTTGGCGAGAAGGCTCTCCTAGCTGGGAGATGTTTCTGCTAGGGGAATAAATCTTTGGGCACCTAGTGGTCATG

1 CATGACCACTAGGAGCATCTTTGGCGACAACCGGAGTAAGACTCTGGGATAGTGGAGCTAGGGGAATAAATCTTTGGGCACCTAGTGGTCATG

1 CATGACCACTAGGAGCATCTTTGGCGAGGCAATCGCGGAGTGCGCCGTGGTCGAGTCCTAGGGGAATAAATCTTTGGGCACCTAGTGGTCATG

1 CATGACCACTAGGAGCATCTTTGGCGAAGGGTTTCGGTCACGTGGGCCCTACGTAGCCTAGGGGAATAAATCTTTGGGCACCTAGTGGTCATG

1 CATGACCACTAGGAGCATCTTTGGCGATAAGGGTACCGTTGCGAAGTGGATAAGCGCCTAGGGGAATAAATCTTTGGGCACCTAGTGGTCATG

1 CATGACCACTAGGAGCATCTTTGGCGAAAGCCGTAGCCGTAATGTTGGTATAAGGCCCTAGGGGAATAAATCTTTGGGCACCTAGTGGTCATG

1 CATGACCACTAGGAGCATCTTTGGCGACGTCGTGGAAAGCGCATGCTACAGGCGCCTCTAGGGGAATAAATCTTTGGGCACCTAGTGGTCATG

1 CATGACCACTAGGAGCATCTTTGGCGATGAAAGTAGCGGCCGCACTTAGAACAGAGACTAGGGGAATAAATCTTTGGGCACCTAGTGGTCATG

1 CATGACCACTAGGAGCATCTTTGGCGAGGCAACGGCAAAGGTATGATGACGAGACAGCTAGGGGAATAAATCTTTGGGCACCTAGTGGTCATG

1 CATGACCACTAGGAGCATCTTTGGCGAGGTTCCACGGGTATCCTGCCTGGTCGCGAGCTAGGGGAATAAATCTTTGGGCACCTAGTGGTCATG

1 CATGACCACTAGGAGCATCTTTGGCGATCCGGGGCGAGCTTGCGAGGTGTTCATAGACTAGGGGAATAAATCTTTGGGCACCTAGTGGTCATG

1 CATGACCACTAGGAGCATCTTTGGCGATGCGTGTTGCAAGGTCCGCGAGTTTGGACCCTAGGGGAATAAATCTTTGGGCACCTAGTGGTCATG

1 CATGACCACTAGGAGCATCTTTGGCGATGGGTGCAGACGGGGCTAGTGAGCCCTACACTAGGGGAATAAATCTTTGGGCACCTAGTGGTCATG

1 CATGACCACTAGGAGCATCTTTGGCGATTGCGAACTGCCGGTCTGTGGAAGAAGCGCCTAGGGGAATAAATCTTTGGGCACCTAGTGGTCATG

1 CATGACCACTAGGAGCATCTTTGGCGAGGGTGGTCGTGTGGCGCGGACGGGTCGAGACTAGGGGAATAAATCTTTGGGCACCTAGTGGTCATG

1 CATGACCACTAGGAGCATCTTTGGCGACAGTGCCAGCGCGTAGTGGCTCAAGTGGAGCTAGGGGAATAAATCTTTGGGCACCTAGTGGTCATG

1 CATGACCACTAGGAGCATCTTTGGCGACGAAGGGGGCAACGGACATGTATCGCTCGACTAGGGGAATAAATCTTTGGGCACCTAGTGGTCATG

1 CATGACCACTAGGAGCATCTTTGGCGAACCGGGCCAGCCCCGCTGAGACGTCGGTGCCTAGGGGAATAAATCTTTGGGCACCTAGTGGTCATG

1 CATGACCACTAGGAGCATCTTTGGCGAGATCGGGAGAATCGGTGGCATTGGTGTATACTAGGGGAATAAATCTTTGGGCACCTAGTGGTCATG

1 CATGACCACTAGGAGCATCTTTGGCGATGAGGCGGCACAGGCGCCCAGGACGCGCGACTAGGGGAATAAATCTTTGGGCACCTAGTGGTCATG

1 CATGACCACTAGGAGCATCTTTGGCGAGAGCCGCCACGGGTTGGGCCGGCGCGACAGCTAGGGGAATAAATCTTTGGGCACCTAGTGGTCATG

1 CATGACCACTAGGAGCATCTTTGGCGAGTCCGTTGAGCGCAACGAATCATACTCTGCCTAGGGGAATAAATCTTTGGGCACCTAGTGGTCATG

1 CATGACCACTAGGAGCATCTTTGGCGAGGGAGGGGCGTCGGGACGGAGTGGTCGAGACTAGGGGAATAAATCTTTGGGCACCTAGTGGTCATG

1 CATGACCACTAGGAGCATCTTTGGCGATGGAGCGGAACCCGACACCCACCAGATCGACTAGGGGAATAAATCTTTGGGCACCTAGTGGTCATG

1 CATGACCACTAGGAGCATCTTTGGCGACGCAGAGGGCAGCGGTGATCGTTACGTCGACTAGGGGAATAAATCTTTGGGCACCTAGTGGTCATG

1 CATGACCACTAGGAGCATCTTTGGCGAGGCGGAACGACCGGGAGGCACTGTTGTCTCCTAGGGGAATAAATCTTTGGGCACCTAGTGGTCATG

1 CATGACCACTAGGAGCATCTTTGGCGATACAGACCTGGACAAGACTACGAGTATCGACTAGGGGAATAAATCTTTGGGCACCTAGTGGTCATG

1 CATGACCACTAGGAGCATCTTTGGCGATCTGAGTTGGTAGGTGAGTATTTGAGCGATCTAGGGGAATAAATCTTTGGGCACCTAGTGGTCATG

1 CATGACCACTAGGAGCATCTTTGGCGATGTTCGAACTCGGGTGTGGTCGGATTGCGACTAGGGGAATAAATCTTTGGGCACCTAGTGGTCATG

1 CATGACCACTAGGAGCATCTTTGGCGACTGTGTTAGACTGGGGCTTCGGGAAGGAGACTAGGGGAATAAATCTTTGGGCACCTAGTGGTCATG

1 CATGACCACTAGGAGCATCTTTGGCGACTATGTGGCATAGGCAATTAAATCTGTCGACTAGGGGAATAAATCTTTGGGCACCTAGTGGTCATG

1 CATGACCACTAGGAGCATCTTTGGCGAACGGCAGGTGTTGCGGAGGTTTGTGAATCCCTAGGGGAATAAATCTTTGGGCACCTAGTGGTCATG

1 CATGACCACTAGGAGCATCTTTGGCGAAGACAACCGGTGCGACCCCGTGGCTGAGATCTAGGGGAATAAATCTTTGGGCACCTAGTGGTCATG

1 CATGACCACTAGGAGCATCTTTGGCGACCACCCGAGGTCAATCGGGCGTGGAATGCTCTAGGGGAATAAATCTTTGGGCACCTAGTGGTCATG

1 CATGACCACTAGGAGCATCTTTGGCGAGTGAGGTCGGGAGTGGGCGTATTTGCGAGCCTAGGGGAATAAATCTTTGGGCACCTAGTGGTCATG

1 CATGACCACTAGGAGCATCTTTGGCGAACGGCAGGGGTTGCGGTGGTCTCTGAATCCCTAGGGGAATAAATCTTTGGGCACCTAGTGGTCATG

1 CATGACCACTAGGAGCATCTTTGGCGACGCAGCGTAGGCCATGTTCGTCCTGTCGCACTAGGGGAATAAATCTTTGGGCACCTAGTGGTCATG

1 CATGACCACTAGGAGCATCTTTGGCGACGGGGTGGGGAACTTGTTTCGGCGGTGCTACTAGGGGAATAAATCTTTGGGCACCTAGTGGTCATG

1 CATGACCACTAGGAGCATCTTTGGCGATACGAGGCTTGCTGGATGCGCGAGTTTCGCCTAGGGGAATAAATCTTTGGGCACCTAGTGGTCATG

1 CATGACCACTAGGAGCATCTTTGGCGACGTGGGCAGCTTGATTCACGCGAAGGCGCCCTAGGGGAATAAATCTTTGGGCACCTAGTGGTCATG

1 CATGACCACTAGGAGCATCTTTGGCGACTGGGGGTGGTCTGAGAGTCGAATCGTCGCCTAGGGGAATAAATCTTTGGGCACCTAGTGGTCATG

1 CATGACCACTAGGAGCATCTTTGGCGAATTCGACGGGCTTTTAACACGCGTATGAGACTAGGGGAATAAATCTTTGGGCACCTAGTGGTCATG

1 CATGACCACTAGGAGCATCTTTGGCGACCAAGGCGTCCGCGAAGGATAGCGTGGGAGCTAGGGGAATAAATCTTTGGGCACCTAGTGGTCATG

1 CATGACCACTAGGAGCATCTTTGGCGACCCGGCTTGCACCGCCAAGCGGGAGTGCGACTAGGGGAATAAATCTTTGGGCACCTAGTGGTCATG

1 CATGACCACTAGGAGCATCTTTGGCGATGGGGCCGTAGCATCGCTACACTGCGCGGCCTAGGGGAATAAATCTTTGGGCACCTAGTGGTCATG

1 CATGACCACTAGGAGCATCTTTGGCGACGAGTGGAGTCATGGTTAGGGCAACGTCGACTAGGGGAATAAATCTTTGGGCACCTAGTGGTCATG

1 CATGACCACTAGGAGCATCTTTGGCGACGGCAACGGAATAACCAAATTTACAGGCGACTAGGGGAATAAATCTTTGGGCACCTAGTGGTCATG

1 CATGACCACTAGGAGCATCTTTGGCGACAAGTGGGGTAGCGCATAGGGTTTGTCGCTCTAGGGGAATAAATCTTTGGGCACCTAGTGGTCATG

1 CATGACCACTAGGAGCATCTTTGGCGACTAGTGGGCTTCGCGAGTACACGTGTCAGCCTAGGGGAATAAATCTTTGGGCACCTAGTGGTCATG

1 CATGACCACTAGGAGCATCTTTGGCGATCCGACCTTGGCGGGCATGCAGAAGATCGACTAGGGGAATAAATCTTTGGGCACCTAGTGGTCATG

1 CATGACCACTAGGAGCATCTTTGGCGATCCACCCTAATGTGAGTGGTGGTGTGGAGACTAGGGGAATAAATCTTTGGGCACCTAGTGGTCATG

1 CATGACCACTAGGAGCATCTTTGGCGATTGAGGGACGGCACGTCATGTCGAAGTGGACTAGGGGAATAAATCTTTGGGCACCTAGTGGTCATG

1 CATGACCACTAGGAGCATCTTTGGCGAGACGGTTGGGCGACTAGAGTATGGTCGAGACTAGGGGAATAAATCTTTGGGCACCTAGTGGTCATG

1 CATGACCACTAGGAGCATCTTTGGCGACTACGGAAGAGCGTGGGGCAACAGAGTCGACTAGGGGAATAAATCTTTGGGCACCTAGTGGTCATG

1 CATGACCACTAGGAGCATCTTTGGCGACGATTCGTGGGCATGGGAATTGGCTGTCGCCTAGGGGAATAAATCTTTGGGCACCTAGTGGTCATG

1 CATGACCACTAGGAGCATCTTTGGCGACTAGGTAATAACCGGGAAACGAAGTTTAGCCTAGGGGAATAAATCTTTGGGCACCTAGTGGTCATG

1 CATGACCACTAGGAGCATCTTTGGCGACCCGTCCAACGAATGCAAGCGGCGTGGAAGCTAGGGGAATAAATCTTTGGGCACCTAGTGGTCATG

1 CATGACCACTAGGAGCATCTTTGGCGACCAAGTGCAAGAGGGCCAATCCGGTGTCGACTAGGGGAATAAATCTTTGGGCACCTAGTGGTCATG

1 CATGACCACTAGGAGCATCTTTGGCGAGGATCTACCGACCTTGATGGGGATAATCGACTAGGGGAATAAATCTTTGGGCACCTAGTGGTCATG

1 CATGACCACTAGGAGCATCTTTGGCGAAGGGTAAGGGGCCTACAGTTACACCGGAGACTAGGGGAATAAATCTTTGGGCACCTAGTGGTCATG

1 CATGACCACTAGGAGCATCTTTGGCGAGTAAGGCAAGCGCAGGCTTGTTCGGATCGACTAGGGGAATAAATCTTTGGGCACCTAGTGGTCATG

1 CATGACCACTAGGAGCATCTTTGGCGACGCAGGGGGCAGCTCGTTGGGCGAAGGAGCCTAGGGGAATAAATCTTTGGGCACCTAGTGGTCATG

1 CATGACCACTAGGAGCATCTTTGGCGACAAACGGAATCAGGGTATATCGTTGCAAGCCTAGGGGAATAAATCTTTGGGCACCTAGTGGTCATG

1 CATGACCACTAGGAGCATCTTTGGCGAGGGGAGGCGCGAAGCTGACGCCCCCTAAGGCTAGGGGAATAAATCTTTGGGCACCTAGTGGTCATG

1 CATGACCACTAGGAGCATCTTTGGCGAGGGTCAAGGCGCTGAAAATAAGGTCCAAGACTAGGGGAATAAATCTTTGGGCACCTAGTGGTCATG

1 CATGACCACTAGGAGCATCTTTGGCGACCCGCAGCCGTCAGCAGACGATGGGGTGGACTAGGGGAATAAATCTTTGGGCACCTAGTGGTCATG

1 CATGACCACTAGGAGCATCTTTGGCGATTGAACCGTGCGGGGCTCCAAATAACTCGCCTAGGGGAATAAATCTTTGGGCACCTAGTGGTCATG

1 CATGACCACTAGGAGCATCTTTGGCGACGCGGGTGGTGGCTGGATCGGTAGCGGAGACTAGGGGAATAAATCTTTGGGCACCTAGTGGTCATG

1 CATGACCACTAGGAGCATCTTTGGCGACTGGGGTCCGGTGGAAAAGGTAAACGTCGCCTAGGGGAATAAATCTTTGGGCACCTAGTGGTCATG

1 CATGACCACTAGGAGCATCTTTGGCGATATGTCAGATCATCAAATGGACGGCTTCGCCTAGGGGAATAAATCTTTGGGCACCTAGTGGTCATG

1 CATGACCACTAGGAGCATCTTTGGCGAGGAGCACGGTCAAGCTTAAGTGAGGAATAGCTAGGGGAATAAATCTTTGGGCACCTAGTGGTCATG

1 CATGACCACTAGGAGCATCTTTGGCGAGGCGCGGCCTGGGCTCAGGGCAACTCGGCACTAGGGGAATAAATCTTTGGGCACCTAGTGGTCATG

1 CATGACCACTAGGAGCATCTTTGGCGACGCGGGCACGTGCATCAGCTGAAAGGTCGACTAGGGGAATAAATCTTTGGGCACCTAGTGGTCATG

1 CATGACCACTAGGAGCATCTTTGGCGAGAGGACAGGTGCTCCCGTGGGCAATCTCGACTAGGGGAATAAATCTTTGGGCACCTAGTGGTCATG

1 CATGACCACTAGGAGCATCTTTGGCGATCCGGGGCGAAGGGCACTGAGGGTTAGTAGCTAGGGGAATAAATCTTTGGGCACCTAGTGGTCATG

1 CATGACCACTAGGAGCATCTTTGGCGACGATGGAGGTCAATGCGGGGCATTAGTCGACTAGGGGAATAAATCTTTGGGCACCTAGTGGTCATG

1 CATGACCACTAGGAGCATCTTTGGCGACAGGGGATGCCGATGTGCTGGACGACCGAGCTAGGGGAATAAATCTTTGGGCACCTAGTGGTCATG

1 CATGACCACTAGGAGCATCTTTGGCGACCAGAGAAGGCCACTCCGGCTGTCCAGTGACTAGGGGAATAAATCTTTGGGCACCTAGTGGTCATG

1 CATGACCACTAGGAGCATCTTTGGCGAATGGCCGGCACGGCCTTCTAGTCATCTGTACTAGGGGAATAAATCTTTGGGCACCTAGTGGTCATG

1 CATGACCACTAGGAGCATCTTTGGCGAGTCACAGAGGGCGTTGTCTGGTGGACTCGACTAGGGGAATAAATCTTTGGGCACCTAGTGGTCATG

1 CATGACCACTAGGAGCATCTTTGGCGAGATCGGGAGAATCGGGGGTATTGGTGTCTCCTAGGGGAATAAATCTTTGGGCACCTAGTGGTCATG

1 CATGACCACTAGGAGCATCTTTGGCGACAGGGACGGCGCTGTGAAATGCTGTCGCTACTAGGGGAATAAATCTTTGGGCACCTAGTGGTCATG

1 CATGACCACTAGGAGCATCTTTGGCGAACGGCTGATACCGGCAGGGGCTCGCGGTCGCTAGGGGAATAAATCTTTGGGCACCTAGTGGTCATG

1 CATGACCACTAGGAGCATCTTTGGCGACAGTTGGGTGCGAGATGGAAAATCGGCCAGCTAGGGGAATAAATCTTTGGGCACCTAGTGGTCATG

1 CATGACCACTAGGAGCATCTTTGGCGATATCGTATGGGGACGGCAGAGGAGATGAGACTAGGGGAATAAATCTTTGGGCACCTAGTGGTCATG

1 CATGACCACTAGGAGCATCTTTGGCGACGTCTACGACACGCTGGGCAGCAAGGAGCTCTAGGGGAATAAATCTTTGGGCACCTAGTGGTCATG

1 CATGACCACTAGGAGCATCTTTGGCGATCCGGTACGGCGTGGATAGGAGACGGTCGACTAGGGGAATAAATCTTTGGGCACCTAGTGGTCATG

1 CATGACCACTAGGAGCATCTTTGGCGATGGGTACGTTTCGGCCGACGATGGTCCAGCCTAGGGGAATAAATCTTTGGGCACCTAGTGGTCATG

1 CATGACCACTAGGAGCATCTTTGGCGACGTCAGTGGGCATCGTACATCGGAGGAAGACTAGGGGAATAAATCTTTGGGCACCTAGTGGTCATG

1 CATGACCACTAGGAGCATCTTTGGCGATTAGGCCGCTAGGGCGTACGAAGTGTGGTTCTAGGGGAATAAATCTTTGGGCACCTAGTGGTCATG

1 CATGACCACTAGGAGCATCTTTGGCGACGTCCAACGTAGGGACAGCCCTATGTCGCTCTAGGGGAATAAATCTTTGGGCACCTAGTGGTCATG

1 CATGACCACTAGGAGCATCTTTGGCGAGCGGAGCTGGAAGGGAGAGGTAGAGATCGCCTAGGGGAATAAATCTTTGGGCACCTAGTGGTCATG

1 CATGACCACTAGGAGCATCTTTGGCGACGGAGGAGGTAGTGATGAGAGACGCGTCGACTAGGGGAATAAATCTTTGGGCACCTAGTGGTCATG

1 CATGACCACTAGGAGCATCTTTGGCGAGAAAAGTAAGCTGACTGTAAAGTTAATAGCCTAGGGGAATAAATCTTTGGGCACCTAGTGGTCATG

1 CATGACCACTAGGAGCATCTTTGGCGACCCATCAGGCTATAGCATTGGTGGGTGGAGCTAGGGGAATAAATCTTTGGGCACCTAGTGGTCATG

1 CATGACCACTAGGAGCATCTTTGGCGAACGGTGTGGGCATGCTAGAATCGGGTTCGACTAGGGGAATAAATCTTTGGGCACCTAGTGGTCATG

1 CATGACCACTAGGAGCATCTTTGGCGAGCTGGGTCCGCAGAATGGGGCCCACCGAGACTAGGGGAATAAATCTTTGGGCACCTAGTGGTCATG

1 CATGACCACTAGGAGCATCTTTGGCGAATACGGTGGCAGGGGTCGCAGGACTCGTAGCTAGGGGAATAAATCTTTGGGCACCTAGTGGTCATG

1 CATGACCACTAGGAGCATCTTTGGCGAGCGTATGCCCAAGGGACGATGGACGGTCGACTAGGGGAATAAATCTTTGGGCACCTAGTGGTCATG

1 CATGACCACTAGGAGCATCTTTGGCGAGCAGGGAGCTGAGAGTAGGAGATGGCGAGACTAGGGGAATAAATCTTTGGGCACCTAGTGGTCATG

1 CATGACCACTAGGAGCATCTTTGGCGACCCGGTGGAGAAGGCAGCACGAACGGGAGACTAGGGGAATAAATCTTTGGGCACCTAGTGGTCATG

1 CATGACCACTAGGAGCATCTTTGGCGACGGCGTGGGGCTATTGATGGGGCGGCACTCCTAGGGGAATAAATCTTTGGGCACCTAGTGGTCATG

1 CATGACCACTAGGAGCATCTTTGGCGAGAACAGTATGATATCTGGGACATGTTTCTCCTAGGGGAATAAATCTTTGGGCACCTAGTGGTCATG

1 CATGACCACTAGGAGCATCTTTGGCGAGGGGTGGTCAGGGATGGCAGTAAACCGCGCCTAGGGGAATAAATCTTTGGGCACCTAGTGGTCATG

1 CATGACCACTAGGAGCATCTTTGGCGAGATAGTGGTCTCCGGAGTCAGTGGGGTCGCCTAGGGGAATAAATCTTTGGGCACCTAGTGGTCATG

1 CATGACCACTAGGAGCATCTTTGGCGATCTGGTACCAGAAGATGAGCATTGGATCGACTAGGGGAATAAATCTTTGGGCACCTAGTGGTCATG

1 CATGACCACTAGGAGCATCTTTGGCGACAGAGGCGTAGGCTGCCTTGGGTGGTGACCCTAGGGGAATAAATCTTTGGGCACCTAGTGGTCATG

1 CATGACCACTAGGAGCATCTTTGGCGACGGACTGCGACGGATGTACGTTGTCTGCGACTAGGGGAATAAATCTTTGGGCACCTAGTGGTCATG

1 CATGACCACTAGGAGCATCTTTGGCGAGAAGACTATGGATTAGGGGACCAGTTGATGCTAGGGGAATAAATCTTTGGGCACCTAGTGGTCATG

1 CATGACCACTAGGAGCATCTTTGGCGAGCGGGGGGCATGGCGCTAACGAATGCGAGACTAGGGGAATAAATCTTTGGGCACCTAGTGGTCATG

1 CATGACCACTAGGAGCATCTTTGGCGACGACACAAGGGAGGCAACCGTACTGTTCGCCTAGGGGAATAAATCTTTGGGCACCTAGTGGTCATG

1 CATGACCACTAGGAGCATCTTTGGCGAGGGGTCCTGGAACCGCTGCTCGGGGTGCGACTAGGGGAATAAATCTTTGGGCACCTAGTGGTCATG

1 CATGACCACTAGGAGCATCTTTGGCGACAAATGACGCAGTGAGCTGTGCGACATCGCCTAGGGGAATAAATCTTTGGGCACCTAGTGGTCATG

1 CATGACCACTAGGAGCATCTTTGGCGAACAAGGCGTTACACGGGGGTTGCGCTTCGCCTAGGGGAATAAATCTTTGGGCACCTAGTGGTCATG

1 CATGACCACTAGGAGCATCTTTGGCGACTAGGGGACATCGGGTCAGATCAATATCGCCTAGGGGAATAAATCTTTGGGCACCTAGTGGTCATG

1 CATGACCACTAGGAGCATCTTTGGCGAGCGGCAGGTGTTGCGGTGGTTTGTGAATCCCTAGGGGAATAAATCTTTGGGCACCTAGTGGTCATG

1 CATGACCACTAGGAGCATCTTTGGCGACATAGTGGCAGATGGAAGTACCGGAGTCGACTAGGGGAATAAATCTTTGGGCACCTAGTGGTCATG

1 CATGACCACTAGGAGCATCTTTGGCGACCTGCGGGTTTTGGAAAGACGAAGGATCGACTAGGGGAATAAATCTTTGGGCACCTAGTGGTCATG

1 CATGACCACTAGGAGCATCTTTGGCGATCTGCGTGGTCGTCGGGTCAAGTTACGCCGCTAGGGGAATAAATCTTTGGGCACCTAGTGGTCATG

1 CATGACCACTAGGAGCATCTTTGGCGAGAAGATTCCGGATTCGGGGACCAGTTGCTGCTAGGGGAATAAATCTTTGGGCACCTAGTGGTCATG

1 CATGACCACTAGGAGCATCTTTGGCGACGAGTGGACTGTGTCTACGAGAAAGATAGCCTAGGGGAATAAATCTTTGGGCACCTAGTGGTCATG

1 CATGACCACTAGGAGCATCTTTGGCGAAAGGTGGGCCGAGCCGACATCGTATTTCGACTAGGGGAATAAATCTTTGGGCACCTAGTGGTCATG

1 CATGACCACTAGGAGCATCTTTGGCGACGAACTGGGCATGTGCGCATACTAGTCGATCTAGGGGAATAAATCTTTGGGCACCTAGTGGTCATG

1 CATGACCACTAGGAGCATCTTTGGCGACGGTTGGGCAGGGTCCCACTATAGAGGCGACTAGGGGAATAAATCTTTGGGCACCTAGTGGTCATG

1 CATGACCACTAGGAGCATCTTTGGCGACTGCACCAGGGAGCTTAGTACAAGAGGAGCCTAGGGGAATAAATCTTTGGGCACCTAGTGGTCATG

1 CATGACCACTAGGAGCATCTTTGGCGATGGGGCGAGCGAATGCAGATCAACATCGCACTAGGGGAATAAATCTTTGGGCACCTAGTGGTCATG

1 CATGACCACTAGGAGCATCTTTGGCGAGCCTACGAGAATGCAGGTGGAGTTGGGGAGCTAGGGGAATAAATCTTTGGGCACCTAGTGGTCATG

1 CATGACCACTAGGAGCATCTTTGGCGAAGGAAGGGTGCTCTTGCTGAACCTCGGAGACTAGGGGAATAAATCTTTGGGCACCTAGTGGTCATG

1 CATGACCACTAGGAGCATCTTTGGCGACCGCTCGAGGTATTGACGATGTGGACTACGCTAGGGGAATAAATCTTTGGGCACCTAGTGGTCATG

1 CATGACCACTAGGAGCATCTTTGGCGAATTGAGCGGCACGACCGAGACACTGATGAGCTAGGGGAATAAATCTTTGGGCACCTAGTGGTCATG

1 CATGACCACTAGGAGCATCTTTGGCGACTTATGTGGTCAGACGGCGATATAAGGAGACTAGGGGAATAAATCTTTGGGCACCTAGTGGTCATG

1 CATGACCACTAGGAGCATCTTTGGCGAGTGGTGGCATGATCATCGTGTAGAACGAGACTAGGGGAATAAATCTTTGGGCACCTAGTGGTCATG

1 CATGACCACTAGGAGCATCTTTGGCGATCTGGAACTGGAGTAGCCTGGAGAGAGCGACTAGGGGAATAAATCTTTGGGCACCTAGTGGTCATG

1 CATGACCACTAGGAGCATCTTTGGCGACCCGGAAGGATGCAATCTTAATCGTGTGGACTAGGGGAATAAATCTTTGGGCACCTAGTGGTCATG

1 CATGACCACTAGGAGCATCTTTGGCGATTCGGTATAACGATTCGGCTGGTGGATCGCCTAGGGGAATAAATCTTTGGGCACCTAGTGGTCATG

1 CATGACCACTAGGAGCATCTTTGGCGACCCGAGTGGGCTTTAGCCGTCGTGGTGGAGCTAGGGGAATAAATCTTTGGGCACCTAGTGGTCATG

1 CATGACCACTAGGAGCATCTTTGGCGACCAAATCGGGGGGATGCAATAGTTGATCGCCTAGGGGAATAAATCTTTGGGCACCTAGTGGTCATG

1 CATGACCACTAGGAGCATCTTTGGCGACAATACGTGGTCAGGATGTCGGGCTGTCGCCTAGGGGAATAAATCTTTGGGCACCTAGTGGTCATG

1 CATGACCACTAGGAGCATCTTTGGCGAACGTTGTGGGCAGGCATGATAGAGTTCGCACTAGGGGAATAAATCTTTGGGCACCTAGTGGTCATG

1 CATGACCACTAGGAGCATCTTTGGCGACGGGACCAGGGTAGTCTACAAGTCCCGCTGCTAGGGGAATAAATCTTTGGGCACCTAGTGGTCATG

1 CATGACCACTAGGAGCATCTTTGGCGATTGCTGAAGGGATGGCCCTGACGTAGGCGACTAGGGGAATAAATCTTTGGGCACCTAGTGGTCATG

1 CATGACCACTAGGAGCATCTTTGGCGAGATCGGGAGACTCGGGGGCATTGGTGTCTCCTAGGGGAATAAATCTTTGGGCACCTAGTGGTCATG

1 CATGACCACTAGGAGCATCTTTGGCGATCCGACAAACGTGCGTGAGTGGTCGATTGCCTAGGGGAATAAATCTTTGGGCACCTAGTGGTCATG

1 CATGACCACTAGGAGCATCTTTGGCGACTGGGGTACCGGGCTTGCACTCAGTTCGCTCTAGGGGAATAAATCTTTGGGCACCTAGTGGTCATG

1 CATGACCACTAGGAGCATCTTTGGCGACTGATCGGCAGGGAGCGCTCGCTGGATCGCCTAGGGGAATAAATCTTTGGGCACCTAGTGGTCATG

1 CATGACCACTAGGAGCATCTTTGGCGAGTCGTGGGCATGAGAGAAGGGACGACGAGGCTAGGGGAATAAATCTTTGGGCACCTAGTGGTCATG

1 CATGACCACTAGGAGCATCTTTGGCGAAAAGAAGCGCTCGGGAGGATGACGTTGAGCCTAGGGGAATAAATCTTTGGGCACCTAGTGGTCATG

1 CATGACCACTAGGAGCATCTTTGGCGACGAGGCAAGGTTGGGCGTTGAGCGTTTCGCCTAGGGGAATAAATCTTTGGGCACCTAGTGGTCATG

1 CATGACCACTAGGAGCATCTTTGGCGAACTGCAGTTGTTGCGGTGGTCTGTGAATCCCTAGGGGAATAAATCTTTGGGCACCTAGTGGTCATG

1 CATGACCACTAGGAGCATCTTTGGCGATACGACGCAGGAGTGCATGAGTGTATCGAGCTAGGGGAATAAATCTTTGGGCACCTAGTGGTCATG

1 CATGACCACTAGGAGCATCTTTGGCGAACGATAGCTTGTGAAATCAGAGAAGTGGAGCTAGGGGAATAAATCTTTGGGCACCTAGTGGTCATG

1 CATGACCACTAGGAGCATCTTTGGCGAGATTGGGAGAATCGGTGGCATTGGTGCCTCCTAGGGGAATAAATCTTTGGGCACCTAGTGGTCATG

1 CATGACCACTAGGAGCATCTTTGGCGACTACGGCGTAAATGACGTGCTCGTAGTGGACTAGGGGAATAAATCTTTGGGCACCTAGTGGTCATG

1 CATGACCACTAGGAGCATCTTTGGCGAGAGGACGGTGGCAACGCGGAAGGCTCGAGACTAGGGGAATAAATCTTTGGGCACCTAGTGGTCATG

1 CATGACCACTAGGAGCATCTTTGGCGAGCAAGCGCGGTCCCTCGGACCTCACATTTGCTAGGGGAATAAATCTTTGGGCACCTAGTGGTCATG

1 CATGACCACTAGGAGCATCTTTGGCGATCTCGCAAGGATAGTTGTCGGGGAGGCGACCTAGGGGAATAAATCTTTGGGCACCTAGTGGTCATG

1 CATGACCACTAGGAGCATCTTTGGCGATCCTCGTCAGATGGTGAAGCAGGCGTTTGGCTAGGGGAATAAATCTTTGGGCACCTAGTGGTCATG

1 CATGACCACTAGGAGCATCTTTGGCGACCACGGAGTGGGGGGTTCGGCGGGTTGAGACTAGGGGAATAAATCTTTGGGCACCTAGTGGTCATG

1 CATGACCACTAGGAGCATCTTTGGCGACGTGGCGGGGAATGTTGGGGACCGTGTCGCCTAGGGGAATAAATCTTTGGGCACCTAGTGGTCATG

1 CATGACCACTAGGAGCATCTTTGGCGAGATAAGTGACGGCTTCCGTCGCGGCGGCGCCTAGGGGAATAAATCTTTGGGCACCTAGTGGTCATG

1 CATGACCACTAGGAGCATCTTTGGCGATCGTCACATGGATGCACGTACCGAGAGCCTCTAGGGGAATAAATCTTTGGGCACCTAGTGGTCATG

1 CATGACCACTAGGAGCATCTTTGGCGACAGAGTGGGCTCGTAGATCCGCAGAGTCGACTAGGGGAATAAATCTTTGGGCACCTAGTGGTCATG

1 CATGACCACTAGGAGCATCTTTGGCGAGAGGTCTCCGACACCGACGCAGACGTTTGGCTAGGGGAATAAATCTTTGGGCACCTAGTGGTCATG

1 CATGACCACTAGGAGCATCTTTGGCGATCGCTGGTGTCACTTTACGTATAAGCTCGCCTAGGGGAATAAATCTTTGGGCACCTAGTGGTCATG

1 CATGACCACTAGGAGCATCTTTGGCGACGGCGTGGGGGTCTTGATCCGGCGGTGGTCCTAGGGGAATAAATCTTTGGGCACCTAGTGGTCATG

1 CATGACCACTAGGAGCATCTTTGGCGAGCCGGTGGATTGCGGAGCAACACTGACAGCCTAGGGGAATAAATCTTTGGGCACCTAGTGGTCATG

1 CATGACCACTAGGAGCATCTTTGGCGAACGGCATGTGTTTCGGTGGTCTGTGAAACCCTAGGGGAATAAATCTTTGGGCACCTAGTGGTCATG

1 CATGACCACTAGGAGCATCTTTGGCGATATTGGCAGGCTCGGACTGTTTCGTAGCGACTAGGGGAATAAATCTTTGGGCACCTAGTGGTCATG

1 CATGACCACTAGGAGCATCTTTGGCGACGAAAACATAGTGATCAGCACAATAGGAGACTAGGGGAATAAATCTTTGGGCACCTAGTGGTCATG

1 CATGACCACTAGGAGCATCTTTGGCGACTGTGGGATATTTGTATAAGGGTGCAGCGACTAGGGGAATAAATCTTTGGGCACCTAGTGGTCATG

1 CATGACCACTAGGAGCATCTTTGGCGATGTCCACACACTGGATTACGCTGTCCGTGTCTAGGGGAATAAATCTTTGGGCACCTAGTGGTCATG

1 CATGACCACTAGGAGCATCTTTGGCGACCGTGGGCACAGAATGACATCTACGAAAGCCTAGGGGAATAAATCTTTGGGCACCTAGTGGTCATG

1 CATGACCACTAGGAGCATCTTTGGCGAGCGACGGGGAGGTGAAGTGATAAGCGACGACTAGGGGAATAAATCTTTGGGCACCTAGTGGTCATG

1 CATGACCACTAGGAGCATCTTTGGCGACGTTACTAGTTGGGAAGCATTCACGGTTAGCTAGGGGAATAAATCTTTGGGCACCTAGTGGTCATG

1 CATGACCACTAGGAGCATCTTTGGCGATTGACGGAGACGGTCAACAGTTATTGTCGCCTAGGGGAATAAATCTTTGGGCACCTAGTGGTCATG

1 CATGACCACTAGGAGCATCTTTGGCGACGAAGGGGAGGCTACGTGCAGGGAAGTCGACTAGGGGAATAAATCTTTGGGCACCTAGTGGTCATG

1 CATGACCACTAGGAGCATCTTTGGCGAGATCGCGAGAATCGGTGGCATTGGTGACTCCTAGGGGAATAAATCTTTGGGCACCTAGTGGTCATG

1 CATGACCACTAGGAGCATCTTTGGCGACCCCCTAAGCGAGCTGCTGGGGAGTGGAGACTAGGGGAATAAATCTTTGGGCACCTAGTGGTCATG

1 CATGACCACTAGGAGCATCTTTGGCGACATCGGACTGGGACTACAGTGCGTTGTGGACTAGGGGAATAAATCTTTGGGCACCTAGTGGTCATG

1 CATGACCACTAGGAGCATCTTTGGCGAGGTGCTCCGCGTTTGTGGGTTGTAGCCTCGCTAGGGGAATAAATCTTTGGGCACCTAGTGGTCATG

1 CATGACCACTAGGAGCATCTTTGGCGACGTGGTCTACGACTTCGGGAAATTAGACGCCTAGGGGAATAAATCTTTGGGCACCTAGTGGTCATG

1 CATGACCACTAGGAGCATCTTTGGCGACTGCCGGAAGATATAAAAAGAATTAGTCGCCTAGGGGAATAAATCTTTGGGCACCTAGTGGTCATG

1 CATGACCACTAGGAGCATCTTTGGCGACGACTTCGGAGTCGGTGCGCCGCTGGCATACTAGGGGAATAAATCTTTGGGCACCTAGTGGTCATG

1 CATGACCACTAGGAGCATCTTTGGCGAGCCTCGCTTGGGAGTTTGATCCACCAGTTCCTAGGGGAATAAATCTTTGGGCACCTAGTGGTCATG

1 CATGACCACTAGGAGCATCTTTGGCGACTGGTGGGTTGCGTCTCGGCCTGTTGGTTGCTAGGGGAATAAATCTTTGGGCACCTAGTGGTCATG

1 CATGACCACTAGGAGCATCTTTGGCGATATGGAACGGACGAACGTCCGAAGTGGCGCCTAGGGGAATAAATCTTTGGGCACCTAGTGGTCATG

1 CATGACCACTAGGAGCATCTTTGGCGATGAGAACGGCAAGGCTATCACAGCTCACGACTAGGGGAATAAATCTTTGGGCACCTAGTGGTCATG

1 CATGACCACTAGGAGCATCTTTGGCGAGGTCACGGTTTAGTACGTATGGACGAGCCGCTAGGGGAATAAATCTTTGGGCACCTAGTGGTCATG

1 CATGACCACTAGGAGCATCTTTGGCGAAACGGCTGTAGATGCCGGTGTGGATGGCGCCTAGGGGAATAAATCTTTGGGCACCTAGTGGTCATG

1 CATGACCACTAGGAGCATCTTTGGCGAATTGGTGAGACGGCGTCTCGAAAAAGCTCACTAGGGGAATAAATCTTTGGGCACCTAGTGGTCATG

1 CATGACCACTAGGAGCATCTTTGGCGAGGTGACGCGAGCGGACTGCGTGTCGAGCCGCTAGGGGAATAAATCTTTGGGCACCTAGTGGTCATG

1 CATGACCACTAGGAGCATCTTTGGCGACGTAGGACAGGTCATGGTTTCGTTGAGGAGCTAGGGGAATAAATCTTTGGGCACCTAGTGGTCATG

1 CATGACCACTAGGAGCATCTTTGGCGATCCGTTGTGCAGCGCTGACCGAGTCGCCAGCTAGGGGAATAAATCTTTGGGCACCTAGTGGTCATG

1 CATGACCACTAGGAGCATCTTTGGCGAACCGTTGGAGCAATTAGGCACTGGCTGAGCCTAGGGGAATAAATCTTTGGGCACCTAGTGGTCATG

1 CATGACCACTAGGAGCATCTTTGGCGATGGGTGTTGACGGCCGCCCCTGCGGCTACACTAGGGGAATAAATCTTTGGGCACCTAGTGGTCATG

1 CATGACCACTAGGAGCATCTTTGGCGAGAAGACTCTGGATTCGGGGACCATTTGCTTCTAGGGGAATAAATCTTTGGGCACCTAGTGGTCATG

1 CATGACCACTAGGAGCATCTTTGGCGAGATCGGCAGAATCGGTGGCATTGGAGTCTCCTAGGGGAATAAATCTTTGGGCACCTAGTGGTCATG

1 CATGACCACTAGGAGCATCTTTGGCGACCGTAGGAAAACAAAAGAGGCCTTGCTAGCCTAGGGGAATAAATCTTTGGGCACCTAGTGGTCATG

1 CATGACCACTAGGAGCATCTTTGGCGACGGTGGTCGGCCAGGCACACGCAAATGTCGCTAGGGGAATAAATCTTTGGGCACCTAGTGGTCATG

1 CATGACCACTAGGAGCATCTTTGGCGAATTGATAGTACATGTGTCTGGCGAAATCGACTAGGGGAATAAATCTTTGGGCACCTAGTGGTCATG

1 CATGACCACTAGGAGCATCTTTGGCGACGTGGTCAGGGAAGAAGTAGGCCGAGGAGACTAGGGGAATAAATCTTTGGGCACCTAGTGGTCATG

1 CATGACCACTAGGAGCATCTTTGGCGAGGTGGTGAGCATGGCAGGGATGCGTCGAGACTAGGGGAATAAATCTTTGGGCACCTAGTGGTCATG

1 CATGACCACTAGGAGCATCTTTGGCGAGTGCGATCTAAAGGCGTTTAGTGAACGAGACTAGGGGAATAAATCTTTGGGCACCTAGTGGTCATG

1 CATGACCACTAGGAGCATCTTTGGCGACGCTCGTGTAAGAGTGGTAGAGATTGTCGACTAGGGGAATAAATCTTTGGGCACCTAGTGGTCATG

1 CATGACCACTAGGAGCATCTTTGGCGACTAGGCATGTTAGTAGATCTGTAAACGTCGCTAGGGGAATAAATCTTTGGGCACCTAGTGGTCATG

1 CATGACCACTAGGAGCATCTTTGGCGAAGTACACAAAAGGGCCTGGGCGTACTTCGGCTAGGGGAATAAATCTTTGGGCACCTAGTGGTCATG

1 CATGACCACTAGGAGCATCTTTGGCGACTCAGGGACGCGGGCACGAGGACTAGATCGCTAGGGGAATAAATCTTTGGGCACCTAGTGGTCATG

1 CATGACCACTAGGAGCATCTTTGGCGACGTGGGTCGGGCAGGCTGATCAAACACGAGCTAGGGGAATAAATCTTTGGGCACCTAGTGGTCATG

1 CATGACCACTAGGAGCATCTTTGGCGACTTCCGTGATGTAAGCAATAAGGATAGAGACTAGGGGAATAAATCTTTGGGCACCTAGTGGTCATG

1 CATGACCACTAGGAGCATCTTTGGCGACATTTGGCTTTAACATTGCAACAGAGTCGACTAGGGGAATAAATCTTTGGGCACCTAGTGGTCATG

1 CATGACCACTAGGAGCATCTTTGGCGACTGAGTGGTTGGGGCTAGTATACCGGTCGACTAGGGGAATAAATCTTTGGGCACCTAGTGGTCATG

1 CATGACCACTAGGAGCATCTTTGGCGAGGGTAGCATGCCATCGGATTCGGCGACGAGCTAGGGGAATAAATCTTTGGGCACCTAGTGGTCATG

1 CATGACCACTAGGAGCATCTTTGGCGAGATAGTGGGTAAGTTAGATGGTTCTATCTCCTAGGGGAATAAATCTTTGGGCACCTAGTGGTCATG

1 CATGACCACTAGGAGCATCTTTGGCGACGGGTGGGCACGCGTTGATAGGAGGTCGCTCTAGGGGAATAAATCTTTGGGCACCTAGTGGTCATG

1 CATGACCACTAGGAGCATCTTTGGCGACTGTGGGCACGTGCCAAACTATAACGTCGACTAGGGGAATAAATCTTTGGGCACCTAGTGGTCATG

1 CATGACCACTAGGAGCATCTTTGGCGAATCATGGGTTGGCAGGGAGTATTTGGGCGACTAGGGGAATAAATCTTTGGGCACCTAGTGGTCATG

1 CATGACCACTAGGAGCATCTTTGGCGAGTGCCGGGGTGCGTGTCCAATGGGCCGAGACTAGGGGAATAAATCTTTGGGCACCTAGTGGTCATG

1 CATGACCACTAGGAGCATCTTTGGCGAGGGGTTGGGGAGCTCGGCGAACGTACGCGCCTAGGGGAATAAATCTTTGGGCACCTAGTGGTCATG

1 CATGACCACTAGGAGCATCTTTGGCGACGCAGTGGGCCAGGTCGGGTGAGAGCGCGCCTAGGGGAATAAATCTTTGGGCACCTAGTGGTCATG

1 CATGACCACTAGGAGCATCTTTGGCGAACGGAAGGTGTTGCCGTGGTCTTAGAAACCCTAGGGGAATAAATCTTTGGGCACCTAGTGGTCATG

1 CATGACCACTAGGAGCATCTTTGGCGACAAGTGGCTGAGCAGCGGAAAATTGGCTGACTAGGGGAATAAATCTTTGGGCACCTAGTGGTCATG

1 CATGACCACTAGGAGCATCTTTGGCGAGAGGACTCAATCATACGAGCAGACGTTTGGCTAGGGGAATAAATCTTTGGGCACCTAGTGGTCATG

1 CATGACCACTAGGAGCATCTTTGGCGACGTGTGCGGCCAGCGTGGTGACATAGTCGACTAGGGGAATAAATCTTTGGGCACCTAGTGGTCATG

1 CATGACCACTAGGAGCATCTTTGGCGAGCGGGGACAGCATGTTGGAAACGCTACGCGCTAGGGGAATAAATCTTTGGGCACCTAGTGGTCATG

1 CATGACCACTAGGAGCATCTTTGGCGATGACGTGGGCAGGGAGAAGACGATCGGAGCCTAGGGGAATAAATCTTTGGGCACCTAGTGGTCATG

1 CATGACCACTAGGAGCATCTTTGGCGAGATTCGACAGGAGGATAGGGAGCGGTTCGCCTAGGGGAATAAATCTTTGGGCACCTAGTGGTCATG

1 CATGACCACTAGGAGCATCTTTGGCGATGGGTGCTTACGGGGCTAGTGAGCCCTACACTAGGGGAATAAATCTTTGGGCACCTAGTGGTCATG

1 CATGACCACTAGGAGCATCTTTGGCGACGTAAGCAGAGAGCAGCGTTAGAAGGTCGACTAGGGGAATAAATCTTTGGGCACCTAGTGGTCATG

1 CATGACCACTAGGAGCATCTTTGGCGACGGCTAATCACGCGGGTCAGGCTGTCGGCACTAGGGGAATAAATCTTTGGGCACCTAGTGGTCATG

1 CATGACCACTAGGAGCATCTTTGGCGACAGGGCGAGCAGACGAACATAGAAGGGCGACTAGGGGAATAAATCTTTGGGCACCTAGTGGTCATG

1 CATGACCACTAGGAGCATCTTTGGCGATGTTCGTCCGGAGAGCATAGACGACCGAGACTAGGGGAATAAATCTTTGGGCACCTAGTGGTCATG

1 CATGACCACTAGGAGCATCTTTGGCGAATGAGCTTGTAGGCCAGAGCAGACGTTTGGCTAGGGGAATAAATCTTTGGGCACCTAGTGGTCATG

1 CATGACCACTAGGAGCATCTTTGGCGATAAAGTGGGCGTGACGGCTTCGCACATCGACTAGGGGAATAAATCTTTGGGCACCTAGTGGTCATG

1 CATGACCACTAGGAGCATCTTTGGCGAGGTTGGAGGCAAGGGTTTGCAGGTTCGAGACTAGGGGAATAAATCTTTGGGCACCTAGTGGTCATG

1 CATGACCACTAGGAGCATCTTTGGCGAATCCGTAAGGATGGCTAACACGGTAGTCGACTAGGGGAATAAATCTTTGGGCACCTAGTGGTCATG

1 CATGACCACTAGGAGCATCTTTGGCGAACGTGACGGGAGCGAACGGGCTATGCCTAGCTAGGGGAATAAATCTTTGGGCACCTAGTGGTCATG

1 CATGACCACTAGGAGCATCTTTGGCGACTGGTCATGTAGAGAGCTAGTTGGTGTCGCCTAGGGGAATAAATCTTTGGGCACCTAGTGGTCATG

1 CATGACCACTAGGAGCATCTTTGGCGATAAGTGGTCCGGGGATGCCGACTAACGCGCCTAGGGGAATAAATCTTTGGGCACCTAGTGGTCATG

1 CATGACCACTAGGAGCATCTTTGGCGAGTAGGTGTGAATGTTCGTTCGGTGTACTCGCTAGGGGAATAAATCTTTGGGCACCTAGTGGTCATG

1 CATGACCACTAGGAGCATCTTTGGCGACAACACCAGACATGTTATGGTGAATTGAAGCTAGGGGAATAAATCTTTGGGCACCTAGTGGTCATG

1 CATGACCACTAGGAGCATCTTTGGCGAAGGCCTAGATTGGTAGGATGTGCCTGGAGACTAGGGGAATAAATCTTTGGGCACCTAGTGGTCATG

1 CATGACCACTAGGAGCATCTTTGGCGATCGGGACACCGGGAGAAAAATGAAGATCGACTAGGGGAATAAATCTTTGGGCACCTAGTGGTCATG

1 CATGACCACTAGGAGCATCTTTGGCGAGATCGGGAGGATCGGTGGCGTTGGTGTCTCCTAGGGGAATAAATCTTTGGGCACCTAGTGGTCATG

1 CATGACCACTAGGAGCATCTTTGGCGACGGAGTACAGTCTTGGGACTGCGAAGTGGACTAGGGGAATAAATCTTTGGGCACCTAGTGGTCATG

1 CATGACCACTAGGAGCATCTTTGGCGACGGCAGTGGGCAGGTTCCAGCGCTGGCGCTCTAGGGGAATAAATCTTTGGGCACCTAGTGGTCATG

1 CATGACCACTAGGAGCATCTTTGGCGACCGAGCGTAGGAGGCAGGGGAGTCGGGCGACTAGGGGAATAAATCTTTGGGCACCTAGTGGTCATG

1 CATGACCACTAGGAGCATCTTTGGCGATCGGTTGGCATTGGTGTCTCCTTTGTGACGCTAGGGGAATAAATCTTTGGGCACCTAGTGGTCATG

1 CATGACCACTAGGAGCATCTTTGGCGAGGACCCACCCGGCATCGAGGTGTGACAAGACTAGGGGAATAAATCTTTGGGCACCTAGTGGTCATG

1 CATGACCACTAGGAGCATCTTTGGCGATCGCGCGAGTGCATCTAAAGCCGATAATCGCTAGGGGAATAAATCTTTGGGCACCTAGTGGTCATG

1 CATGACCACTAGGAGCATCTTTGGCGACCCTTAGCGTTGGCTAACGCAAGAGGTGGACTAGGGGAATAAATCTTTGGGCACCTAGTGGTCATG

1 CATGACCACTAGGAGCATCTTTGGCGACCGAGGCAGCGGGGTAAATATGCGCAGGTCCTAGGGGAATAAATCTTTGGGCACCTAGTGGTCATG

1 CATGACCACTAGGAGCATCTTTGGCGAAGGGAGTTCGGCAGTCTCGCAGACGCTCGCCTAGGGGAATAAATCTTTGGGCACCTAGTGGTCATG

1 CATGACCACTAGGAGCATCTTTGGCGATGAACATGGCAGGGAGATGTGTCACTCGACCTAGGGGAATAAATCTTTGGGCACCTAGTGGTCATG

1 CATGACCACTAGGAGCATCTTTGGCGAAGTGCGGCGTGTCGAGTCGTCCTGCGACTCCTAGGGGAATAAATCTTTGGGCACCTAGTGGTCATG

1 CATGACCACTAGGAGCATCTTTGGCGAGATCGGGAGAATCTGTGGAATTGGTGTCACCTAGGGGAATAAATCTTTGGGCACCTAGTGGTCATG

1 CATGACCACTAGGAGCATCTTTGGCGAGATCGCGAGAATCGGAGGCAGTGGTGTATCCTAGGGGAATAAATCTTTGGGCACCTAGTGGTCATG

1 CATGACCACTAGGAGCATCTTTGGCGATAGCGAATGCAGATGGTATGTCCGGATGCACTAGGGGAATAAATCTTTGGGCACCTAGTGGTCATG

1 CATGACCACTAGGAGCATCTTTGGCGAGCGGATAAATGGACGTCGAGCACTCGCGAGCTAGGGGAATAAATCTTTGGGCACCTAGTGGTCATG

1 CATGACCACTAGGAGCATCTTTGGCGACAAGGGGTTGCTAAGCAAATATAGGTCGCTCTAGGGGAATAAATCTTTGGGCACCTAGTGGTCATG

1 CATGACCACTAGGAGCATCTTTGGCGACGGTGTGGGGCCATATGTGCGGCGGATCCCCTAGGGGAATAAATCTTTGGGCACCTAGTGGTCATG

1 CATGACCACTAGGAGCATCTTTGGCGAGTCAACAGAAGATGTTGGGCAATGGCTCGACTAGGGGAATAAATCTTTGGGCACCTAGTGGTCATG

1 CATGACCACTAGGAGCATCTTTGGCGAACGGCAGGTGTTGCTGTTGTCTGTGAATCCCTAGGGGAATAAATCTTTGGGCACCTAGTGGTCATG

1 CATGACCACTAGGAGCATCTTTGGCGAAGGAGCTACGAGAGCGGGACAGAGCTTCGACTAGGGGAATAAATCTTTGGGCACCTAGTGGTCATG

1 CATGACCACTAGGAGCATCTTTGGCGATCAGGCCGCCAGGGCGTATGAAGTGTGGTTCTAGGGGAATAAATCTTTGGGCACCTAGTGGTCATG

1 CATGACCACTAGGAGCATCTTTGGCGACTCAGCGTGGGACGTTTGACTGGTGCTCGACTAGGGGAATAAATCTTTGGGCACCTAGTGGTCATG

1 CATGACCACTAGGAGCATCTTTGGCGAACGTCAGGTGTTTCGGTGGTCTGTGAATCCCTAGGGGAATAAATCTTTGGGCACCTAGTGGTCATG

1 CATGACCACTAGGAGCATCTTTGGCGAACGGATACATAACGGCATTACAAAGAGAGACTAGGGGAATAAATCTTTGGGCACCTAGTGGTCATG

1 CATGACCACTAGGAGCATCTTTGGCGAGTAGAAGCACTCGGCACTGGTGATCATCGCCTAGGGGAATAAATCTTTGGGCACCTAGTGGTCATG

1 CATGACCACTAGGAGCATCTTTGGCGATCCTCGCTTGGTATGTTGCTCCACCAGTTCCTAGGGGAATAAATCTTTGGGCACCTAGTGGTCATG

1 CATGACCACTAGGAGCATCTTTGGCGAAGATCAGGGGCATGTCAACGAGAGCTGAGACTAGGGGAATAAATCTTTGGGCACCTAGTGGTCATG

1 CATGACCACTAGGAGCATCTTTGGCGAGTGCTGGCGGCAAGGGGCGTCTGACCGAGCCTAGGGGAATAAATCTTTGGGCACCTAGTGGTCATG

1 CATGACCACTAGGAGCATCTTTGGCGACTCGTGGTCCTAGGATAGAGAACAAAACGCCTAGGGGAATAAATCTTTGGGCACCTAGTGGTCATG

1 CATGACCACTAGGAGCATCTTTGGCGAGATCGGAATAATCGGTGGCATTGGTGTCTCCTAGGGGAATAAATCTTTGGGCACCTAGTGGTCATG

1 CATGACCACTAGGAGCATCTTTGGCGAGATCGGGATAATATGTGGCATTGGTGTCTCCTAGGGGAATAAATCTTTGGGCACCTAGTGGTCATG

1 CATGACCACTAGGAGCATCTTTGGCGAATGGCCGGCACGACCTTATAGTCCTCGGTACTAGGGGAATAAATCTTTGGGCACCTAGTGGTCATG

1 CATGACCACTAGGAGCATCTTTGGCGAATGATATAGCGTATGTACGCTGAGGCTCGACTAGGGGAATAAATCTTTGGGCACCTAGTGGTCATG

1 CATGACCACTAGGAGCATCTTTGGCGACGATGTGTGGTCTCGTGTTGCTCGAGTCGACTAGGGGAATAAATCTTTGGGCACCTAGTGGTCATG

1 CATGACCACTAGGAGCATCTTTGGCGATGGACGACCGGACCAGATGAGGGAATGCGCCTAGGGGAATAAATCTTTGGGCACCTAGTGGTCATG

1 CATGACCACTAGGAGCATCTTTGGCGACGCAGTGGGCAGAAGCGGTGAGCGTGTCGCCTAGGGGAATAAATCTTTGGGCACCTAGTGGTCATG

1 CATGACCACTAGGAGCATCTTTGGCGACTGGTGGGTTGCAGCGGATGGACCTGTCGGCTAGGGGAATAAATCTTTGGGCACCTAGTGGTCATG

1 CATGACCACTAGGAGCATCTTTGGCGAGCGGGTGAGGCAGGGAGATGTGATGCTCGACTAGGGGAATAAATCTTTGGGCACCTAGTGGTCATG

1 CATGACCACTAGGAGCATCTTTGGCGACCCAAAACCATCGGGCCGATGGAGTGGAGACTAGGGGAATAAATCTTTGGGCACCTAGTGGTCATG

1 CATGACCACTAGGAGCATCTTTGGCGACCTGGGCTCCCGGGCTTACTTCGGTCGACACTAGGGGAATAAATCTTTGGGCACCTAGTGGTCATG

1 CATGACCACTAGGAGCATCTTTGGCGAGCACAAGAGGGCCCTACTGACACGGCGAGACTAGGGGAATAAATCTTTGGGCACCTAGTGGTCATG

1 CATGACCACTAGGAGCATCTTTGGCGACGAGTGGATAGAAGCCAGGTCACTGAGGTGCTAGGGGAATAAATCTTTGGGCACCTAGTGGTCATG

1 CATGACCACTAGGAGCATCTTTGGCGACATAGTGACGGAAGGCACGACGTAGTGGAGCTAGGGGAATAAATCTTTGGGCACCTAGTGGTCATG

1 CATGACCACTAGGAGCATCTTTGGCGACATCAGCTCCGCGGCCGGGGACATAAGGGACTAGGGGAATAAATCTTTGGGCACCTAGTGGTCATG

1 CATGACCACTAGGAGCATCTTTGGCGACTAGGGATTGTTGCGCCCGTACTCGGTCGACTAGGGGAATAAATCTTTGGGCACCTAGTGGTCATG

1 CATGACCACTAGGAGCATCTTTGGCGAGGAGCAGGCAGGCGAGTAAAGGCTTCGAGACTAGGGGAATAAATCTTTGGGCACCTAGTGGTCATG

1 CATGACCACTAGGAGCATCTTTGGCGATGGGGCCGTGCTATCTGCACACTCCGCTGGCTAGGGGAATAAATCTTTGGGCACCTAGTGGTCATG

1 CATGACCACTAGGAGCATCTTTGGCGAGCGTCGAAGGGGCCGGGAATTGACGAATCCCTAGGGGAATAAATCTTTGGGCACCTAGTGGTCATG

1 CATGACCACTAGGAGCATCTTTGGCGACATAATACGGTGCGAGCCACGGGAAGTCGACTAGGGGAATAAATCTTTGGGCACCTAGTGGTCATG

1 CATGACCACTAGGAGCATCTTTGGCGAACGTTTGTCAGAAGTGCGCGGGGAGTGAGACTAGGGGAATAAATCTTTGGGCACCTAGTGGTCATG

1 CATGACCACTAGGAGCATCTTTGGCGACACGTTAGAGTGATCGGTCGTGAAGTGGAGCTAGGGGAATAAATCTTTGGGCACCTAGTGGTCATG

1 CATGACCACTAGGAGCATCTTTGGCGACCTAGTGGACACGCGTTATCATTGGGAAGGCTAGGGGAATAAATCTTTGGGCACCTAGTGGTCATG

1 CATGACCACTAGGAGCATCTTTGGCGAGCTTGCAGCCACCAGTGGTGTGTCTGGCCGCTAGGGGAATAAATCTTTGGGCACCTAGTGGTCATG

1 CATGACCACTAGGAGCATCTTTGGCGAAGTACGGGGATGTGACAGTGGTTGAGTCGCCTAGGGGAATAAATCTTTGGGCACCTAGTGGTCATG

1 CATGACCACTAGGAGCATCTTTGGCGAGATCGGTAGAATCGGTGGCATTGGTGTCACCTAGGGGAATAAATCTTTGGGCACCTAGTGGTCATG

1 CATGACCACTAGGAGCATCTTTGGCGAGGGAGGGCGCCGGCATCGGTGTGAATGCGCCTAGGGGAATAAATCTTTGGGCACCTAGTGGTCATG

1 CATGACCACTAGGAGCATCTTTGGCGAGGAATGAGGAATGTGCGATTAATGTCGAGACTAGGGGAATAAATCTTTGGGCACCTAGTGGTCATG

1 CATGACCACTAGGAGCATCTTTGGCGACGTGCTGAGGCCTCGAGGGGGAAGAGGAGACTAGGGGAATAAATCTTTGGGCACCTAGTGGTCATG

1 CATGACCACTAGGAGCATCTTTGGCGAGTAAGCGAAGTCGTACGATGAGGATACGACCTAGGGGAATAAATCTTTGGGCACCTAGTGGTCATG

1 CATGACCACTAGGAGCATCTTTGGCGACGAGTGGAGTTTACGTAACTCATGGTGGAGCTAGGGGAATAAATCTTTGGGCACCTAGTGGTCATG

1 CATGACCACTAGGAGCATCTTTGGCGACGTACCGCGGAGTGTAAGTATGCGTCGCTACTAGGGGAATAAATCTTTGGGCACCTAGTGGTCATG

1 CATGACCACTAGGAGCATCTTTGGCGAGGGGGGTAAGCTCGTAGACGATCCTTCTCGCTAGGGGAATAAATCTTTGGGCACCTAGTGGTCATG

1 CATGACCACTAGGAGCATCTTTGGCGACGGGGTGGGCTCGGTAGATCGACAAGGAGACTAGGGGAATAAATCTTTGGGCACCTAGTGGTCATG

1 CATGACCACTAGGAGCATCTTTGGCGACGGGGGCTCCCATCATCCCGCAGTACGTCGCTAGGGGAATAAATCTTTGGGCACCTAGTGGTCATG

1 CATGACCACTAGGAGCATCTTTGGCGAGATCGGGAGAATCGGCGGCATCGGTGTCTCCTAGGGGAATAAATCTTTGGGCACCTAGTGGTCATG
[truncated: 1,701,961 more chars]
