# Supplementary material for: Selection of allosteric dnazymes that can sense phenylalanine by expression-SELEX
Source: Nucleic Acids Res. 2023 May 19;51(11):e66. doi: 10.1093/nar/gkad424 (PMC10287898; doi:10.1093/nar/gkad424)
Supplement: gkad424_Supplemental_Files [file gkad424_supplemental_files.zip › Supplementary file No. 4 count_most_represented_seqs_for_round-20.docx]

Note: total reads of the library are 1209093. The reads are on the left, and the sequence is on the right.

892954 CATGACCACTAGGAGCATCTTTGGCGAGATCGGGAGAATCGGTGGCATTGGTGTCTCCTAGGGGAATAAATCTTTGGGCACCTAGTGGTCATG

122302 CATGACCACTAGGAGCATCTTTGGCGAACGGCAGGTGTTGCGGTGGTCTGTGAATCCCTAGGGGAATAAATCTTTGGGCACCTAGTGGTCATG

57642 CATGACCACTAGGAGCATCTTTGGCGAGAAGACTCTGGATTCGGGGACCAGTTGCTGCTAGGGGAATAAATCTTTGGGCACCTAGTGGTCATG

10428 CATGACCACTAGGAGCATCTTTGGCGAAAGTCGGTAGAACAGGGTGGGGTGCTGTCCCTAGGGGAATAAATCTTTGGGCACCTAGTGGTCATG

7863 CATGACCACTAGGAGCATCTTTGGCGAGATCGGGAGAATCGGCGGCATTGGTGTCTCCTAGGGGAATAAATCTTTGGGCACCTAGTGGTCATG

4354 CATGACCACTAGGAGCATCTTTGGCGAGATTGGGAGAATCGGTGGCATTGGTGTCTCCTAGGGGAATAAATCTTTGGGCACCTAGTGGTCATG

3906 CATGACCACTAGGAGCATCTTTGGCGAGATCGGGAGAATCGGTGGCATTGGTGTCTTCTAGGGGAATAAATCTTTGGGCACCTAGTGGTCATG

3479 CATGACCACTAGGAGCATCTTTGGCGAGCCTTGCTTGGGAGGTTGCTCCACCAGTTCCTAGGGGAATAAATCTTTGGGCACCTAGTGGTCATG

3467 CATGACCACTAGGAGCATCTTTGGCGAATGGCCGGCACGGCCTTCTAGTCCTCGGTACTAGGGGAATAAATCTTTGGGCACCTAGTGGTCATG

3398 CATGACCACTAGGAGCATCTTTGGCGAGCCTCGCTTGGGAGGTTGCTCCACCAGTTCCTAGGGGAATAAATCTTTGGGCACCTAGTGGTCATG

3283 CATGACCACTAGGAGCATCTTTGGCGAGATCGGGAGAATTGGTGGCATTGGTGTCTCCTAGGGGAATAAATCTTTGGGCACCTAGTGGTCATG

2781 CATGACCACTAGGAGCATCTTTGGCGAGATCGTGAGAATCGGTGGCATTGGTGTCTCCTAGGGGAATAAATCTTTGGGCACCTAGTGGTCATG

2608 CATGACCACTAGGAGCATCTTTGGCGAGGGAGGGCGCCGGCAGCGGTGTGAATGCGACTAGGGGAATAAATCTTTGGGCACCTAGTGGTCATG

2397 CATGACCACTAGGAGCATCTTTGGCGAGATCGGGAGAATCGGTGGCATTGGTGTTTCCTAGGGGAATAAATCTTTGGGCACCTAGTGGTCATG

2111 CATGACCACTAGGAGCATCTTTGGCGAGATCGGGAGAATCGGTGGCATCGGTGTCTCCTAGGGGAATAAATCTTTGGGCACCTAGTGGTCATG

2015 CATGACCACTAGGAGCATCTTTGGCGATCCGGGGGCACCTATGTGCGACGCTGTGGGCTAGGGGAATAAATCTTTGGGCACCTAGTGGTCATG

1565 CATGACCACTAGGAGCATCTTTGGCGAGATCGGGGGAATCGGTGGCATTGGTGTCTCCTAGGGGAATAAATCTTTGGGCACCTAGTGGTCATG

1555 CATGACCACTAGGAGCATCTTTGGCGAGATCAGGAGAATCGGTGGCATTGGTGTCTCCTAGGGGAATAAATCTTTGGGCACCTAGTGGTCATG

1547 CATGACCACTAGGAGCATCTTTGGCGAGAAGACTCTGGATTCGGGGACCAGTTGCTCCTAGGGGAATAAATCTTTGGGCACCTAGTGGTCATG

1482 CATGACCACTAGGAGCATCTTTGGCGAGATCGGGAGGATCGGTGGCATTGGTGTCTCCTAGGGGAATAAATCTTTGGGCACCTAGTGGTCATG

1467 CATGACCACTAGGAGCATCTTTGGCGAACGGCAGGTGTTGCGGTGGTTTGTGAATCCCTAGGGGAATAAATCTTTGGGCACCTAGTGGTCATG

1449 CATGACCACTAGGAGCATCTTTGGCGAGATCGAGAGAATCGGTGGCATTGGTGTCTCCTAGGGGAATAAATCTTTGGGCACCTAGTGGTCATG

1394 CATGACCACTAGGAGCATCTTTGGCGAGTGGGGGGCGACGGCCGGTAGTGGGTGAGACTAGGGGAATAAATCTTTGGGCACCTAGTGGTCATG

1332 CATGACCACTAGGAGCATCTTTGGCGAGATCGGGAGAATCGGTGGCATTGGTGCCTCCTAGGGGAATAAATCTTTGGGCACCTAGTGGTCATG

1310 CATGACCACTAGGAGCATCTTTGGCGAACGGCAGGTGTTGCGGTGGTCTGTGGATCCCTAGGGGAATAAATCTTTGGGCACCTAGTGGTCATG

1257 CATGACCACTAGGAGCATCTTTGGCGAGATCGGGAGAATCGGTGGCATTGGCGTCTCCTAGGGGAATAAATCTTTGGGCACCTAGTGGTCATG

1204 CATGACCACTAGGAGCATCTTTGGCGAGATCGGGAGAATCGGTGGCATTGGTGTCCCCTAGGGGAATAAATCTTTGGGCACCTAGTGGTCATG

1108 CATGACCACTAGGAGCATCTTTGGCGATGGGTGCTGACGGCCGCCGCTGCGGCTACACTAGGGGAATAAATCTTTGGGCACCTAGTGGTCATG

1104 CATGACCACTAGGAGCATCTTTGGCGAGATCGGGAGAGTCGGTGGCATTGGTGTCTCCTAGGGGAATAAATCTTTGGGCACCTAGTGGTCATG

1081 CATGACCACTAGGAGCATCTTTGGCGAGATCGGGAGAATCGGTGGTATTGGTGTCTCCTAGGGGAATAAATCTTTGGGCACCTAGTGGTCATG

1060 CATGACCACTAGGAGCATCTTTGGCGAGGGAGGGCGCCGGCAGCGGTGTGAATGCGCCTAGGGGAATAAATCTTTGGGCACCTAGTGGTCATG

1013 CATGACCACTAGGAGCATCTTTGGCGAGATCGGAAGAATCGGTGGCATTGGTGTCTCCTAGGGGAATAAATCTTTGGGCACCTAGTGGTCATG

1011 CATGACCACTAGGAGCATCTTTGGCGAGATCGGGAGAATCGGTGGCGTTGGTGTCTCCTAGGGGAATAAATCTTTGGGCACCTAGTGGTCATG

963 CATGACCACTAGGAGCATCTTTGGCGAGATCGCGAGAATCGGTGGCATTGGTGTCTCCTAGGGGAATAAATCTTTGGGCACCTAGTGGTCATG

938 CATGACCACTAGGAGCATCTTTGGCGAGATCGGTAGAATCGGTGGCATTGGTGTCTCCTAGGGGAATAAATCTTTGGGCACCTAGTGGTCATG

935 CATGACCACTAGGAGCATCTTTGGCGAAGGGTGTAGGACTTCAAGTGGATCTCATAGCTAGGGGAATAAATCTTTGGGCACCTAGTGGTCATG

907 CATGACCACTAGGAGCATCTTTGGCGAGAAGACTTTGGATTCGGGGACCAGTTGCTGCTAGGGGAATAAATCTTTGGGCACCTAGTGGTCATG

754 CATGACCACTAGGAGCATCTTTGGCGAACGGCAGGTGTTGCGGTGGTCTGTGAATCTCTAGGGGAATAAATCTTTGGGCACCTAGTGGTCATG

748 CATGACCACTAGGAGCATCTTTGGCGAGATCGGGAGAATCGGTGGCATTGGTGTCTACTAGGGGAATAAATCTTTGGGCACCTAGTGGTCATG

706 CATGACCACTAGGAGCATCTTTGGCGAGATCTGGAGAATCGGTGGCATTGGTGTCTCCTAGGGGAATAAATCTTTGGGCACCTAGTGGTCATG

683 CATGACCACTAGGAGCATCTTTGGCGAGGTCGGGAGAATCGGTGGCATTGGTGTCTCCTAGGGGAATAAATCTTTGGGCACCTAGTGGTCATG

658 CATGACCACTAGGAGCATCTTTGGCGATATCGGGAGAATCGGTGGCATTGGTGTCTCCTAGGGGAATAAATCTTTGGGCACCTAGTGGTCATG

657 CATGACCACTAGGAGCATCTTTGGCGAGATCGGGAGAATCGGTGGCATTGGTGTCTGCTAGGGGAATAAATCTTTGGGCACCTAGTGGTCATG

649 CATGACCACTAGGAGCATCTTTGGCGACAATGGAGCGACGGTGTGGTCTGAGTCCTACTAGGGGAATAAATCTTTGGGCACCTAGTGGTCATG

629 CATGACCACTAGGAGCATCTTTGGCGAGATCGGGAGAATCGGTGGCACTGGTGTCTCCTAGGGGAATAAATCTTTGGGCACCTAGTGGTCATG

618 CATGACCACTAGGAGCATCTTTGGCGAGATCGGCAGAATCGGTGGCATTGGTGTCTCCTAGGGGAATAAATCTTTGGGCACCTAGTGGTCATG

587 CATGACCACTAGGAGCATCTTTGGCGAGCGGCAGGTGTTGCGGTGGTCTGTGAATCCCTAGGGGAATAAATCTTTGGGCACCTAGTGGTCATG

566 CATGACCACTAGGAGCATCTTTGGCGACGGTGTGGGGAACTTGTTTCGGCGGTGCTACTAGGGGAATAAATCTTTGGGCACCTAGTGGTCATG

559 CATGACCACTAGGAGCATCTTTGGCGAGATCGGGAGAATCGTTGGCATTGGTGTCTCCTAGGGGAATAAATCTTTGGGCACCTAGTGGTCATG

552 CATGACCACTAGGAGCATCTTTGGCGAGATCGGGAGAATCGGAGGCATTGGTGTCTCCTAGGGGAATAAATCTTTGGGCACCTAGTGGTCATG

542 CATGACCACTAGGAGCATCTTTGGCGAACGGCAGGTGTTGCGGTGGTCTGTGTATCCCTAGGGGAATAAATCTTTGGGCACCTAGTGGTCATG

534 CATGACCACTAGGAGCATCTTTGGCGAGATCGGGAGAATCGGTGGCATAGGTGTCTCCTAGGGGAATAAATCTTTGGGCACCTAGTGGTCATG

527 CATGACCACTAGGAGCATCTTTGGCGAGATCGGGAGAATCTGTGGCATTGGTGTCTCCTAGGGGAATAAATCTTTGGGCACCTAGTGGTCATG

526 CATGACCACTAGGAGCATCTTTGGCGACCAGATATGGTAGGTAATGTGCTGAGGAGGCTAGGGGAATAAATCTTTGGGCACCTAGTGGTCATG

526 CATGACCACTAGGAGCATCTTTGGCGAGATCGGGAGAATCAGTGGCATTGGTGTCTCCTAGGGGAATAAATCTTTGGGCACCTAGTGGTCATG

520 CATGACCACTAGGAGCATCTTTGGCGAGAAGACTCCGGATTCGGGGACCAGTTGCTGCTAGGGGAATAAATCTTTGGGCACCTAGTGGTCATG

518 CATGACCACTAGGAGCATCTTTGGCGAGACCGGGAGAATCGGTGGCATTGGTGTCTCCTAGGGGAATAAATCTTTGGGCACCTAGTGGTCATG

497 CATGACCACTAGGAGCATCTTTGGCGAATGGCAGGTGTTGCGGTGGTCTGTGAATCCCTAGGGGAATAAATCTTTGGGCACCTAGTGGTCATG

481 CATGACCACTAGGAGCATCTTTGGCGAGATCGGGAGAATCGGTGTCATTGGTGTCTCCTAGGGGAATAAATCTTTGGGCACCTAGTGGTCATG

479 CATGACCACTAGGAGCATCTTTGGCGAACGGCAGGTGTTGCGGTGGTCTGTGAAACCCTAGGGGAATAAATCTTTGGGCACCTAGTGGTCATG

454 CATGACCACTAGGAGCATCTTTGGCGAGATCGGGAGAATCGGTGGCATTAGTGTCTCCTAGGGGAATAAATCTTTGGGCACCTAGTGGTCATG

452 CATGACCACTAGGAGCATCTTTGGCGAGATCGGGAGAATCGGTGGCATTGATGTCTCCTAGGGGAATAAATCTTTGGGCACCTAGTGGTCATG

447 CATGACCACTAGGAGCATCTTTGGCGAGATCGGGAGAATCGGTAGCATTGGTGTCTCCTAGGGGAATAAATCTTTGGGCACCTAGTGGTCATG

440 CATGACCACTAGGAGCATCTTTGGCGAACGGCAGGTGTTGCGGTGGTCTATGAATCCCTAGGGGAATAAATCTTTGGGCACCTAGTGGTCATG

438 CATGACCACTAGGAGCATCTTTGGCGAACGGCAGGTGTTGCGGTGGTCTGTGAATTCCTAGGGGAATAAATCTTTGGGCACCTAGTGGTCATG

435 CATGACCACTAGGAGCATCTTTGGCGAGGAGTGGCTGAGGGCGGTGGGTAGGTCGCGCTAGGGGAATAAATCTTTGGGCACCTAGTGGTCATG

430 CATGACCACTAGGAGCATCTTTGGCGAGATCGGGAGAAACGGTGGCATTGGTGTCTCCTAGGGGAATAAATCTTTGGGCACCTAGTGGTCATG

428 CATGACCACTAGGAGCATCTTTGGCGACAGGGGATGCCGATGCGCTGGACGACCGAGCTAGGGGAATAAATCTTTGGGCACCTAGTGGTCATG

427 CATGACCACTAGGAGCATCTTTGGCGAGATCGGGAGAATCGGTGGCATTGTTGTCTCCTAGGGGAATAAATCTTTGGGCACCTAGTGGTCATG

417 CATGACCACTAGGAGCATCTTTGGCGAATGGCCGGCACGGCCTTCTAGTCCTTGGTACTAGGGGAATAAATCTTTGGGCACCTAGTGGTCATG

414 CATGACCACTAGGAGCATCTTTGGCGATGGGGCCGTGCTATCTGCACACTCCGCGGGCTAGGGGAATAAATCTTTGGGCACCTAGTGGTCATG

409 CATGACCACTAGGAGCATCTTTGGCGAAATCGGGAGAATCGGTGGCATTGGTGTCTCCTAGGGGAATAAATCTTTGGGCACCTAGTGGTCATG

396 CATGACCACTAGGAGCATCTTTGGCGAGATCGGGAGACTCGGTGGCATTGGTGTCTCCTAGGGGAATAAATCTTTGGGCACCTAGTGGTCATG

386 CATGACCACTAGGAGCATCTTTGGCGATCCTCGTCAGATGGTGAAGCAGACGTTTGGCTAGGGGAATAAATCTTTGGGCACCTAGTGGTCATG

386 CATGACCACTAGGAGCATCTTTGGCGAGATCGGGATAATCGGTGGCATTGGTGTCTCCTAGGGGAATAAATCTTTGGGCACCTAGTGGTCATG

383 CATGACCACTAGGAGCATCTTTGGCGAGGGGTCATGGGAATGTAATTGCGCTTAAGACTAGGGGAATAAATCTTTGGGCACCTAGTGGTCATG

378 CATGACCACTAGGAGCATCTTTGGCGAGATCGGGAGAATCGGTGACATTGGTGTCTCCTAGGGGAATAAATCTTTGGGCACCTAGTGGTCATG

370 CATGACCACTAGGAGCATCTTTGGCGAGATCGGGAGAATCGGTGGCATTTGTGTCTCCTAGGGGAATAAATCTTTGGGCACCTAGTGGTCATG

369 CATGACCACTAGGAGCATCTTTGGCGAGATAGGGAGAATCGGTGGCATTGGTGTCTCCTAGGGGAATAAATCTTTGGGCACCTAGTGGTCATG

366 CATGACCACTAGGAGCATCTTTGGCGAACGGCAGGTGTTGCGGTGGTCTGTGAACCCCTAGGGGAATAAATCTTTGGGCACCTAGTGGTCATG

364 CATGACCACTAGGAGCATCTTTGGCGAGATCGGGAGAATCGGTGGCAGTGGTGTCTCCTAGGGGAATAAATCTTTGGGCACCTAGTGGTCATG

358 CATGACCACTAGGAGCATCTTTGGCGAGATCGGGAGAATCGGTGGCATTGGTGTCACCTAGGGGAATAAATCTTTGGGCACCTAGTGGTCATG

354 CATGACCACTAGGAGCATCTTTGGCGAGAAGACTCTGGATTCGGGGACTAGTTGCTGCTAGGGGAATAAATCTTTGGGCACCTAGTGGTCATG

354 CATGACCACTAGGAGCATCTTTGGCGAGATCGGGAGAACCGGTGGCATTGGTGTCTCCTAGGGGAATAAATCTTTGGGCACCTAGTGGTCATG

352 CATGACCACTAGGAGCATCTTTGGCGAGTATTGGCTGGTAGGTTGCGTATTGGGGAGCTAGGGGAATAAATCTTTGGGCACCTAGTGGTCATG

347 CATGACCACTAGGAGCATCTTTGGCGACGGCGTGGGGCTATTGATGCGGCGGCACTCCTAGGGGAATAAATCTTTGGGCACCTAGTGGTCATG

343 CATGACCACTAGGAGCATCTTTGGCGAGATCGGGAGAATCGGTGGCATTGGAGTCTCCTAGGGGAATAAATCTTTGGGCACCTAGTGGTCATG

334 CATGACCACTAGGAGCATCTTTGGCGAAGCGCAGGGTGTGAGAGGGGCGTGTCCATGCTAGGGGAATAAATCTTTGGGCACCTAGTGGTCATG

330 CATGACCACTAGGAGCATCTTTGGCGAGATCGGGAGAATCGGTGGCATTGGTGACTCCTAGGGGAATAAATCTTTGGGCACCTAGTGGTCATG

327 CATGACCACTAGGAGCATCTTTGGCGAGATCGGGAGAATCGGTGGAATTGGTGTCTCCTAGGGGAATAAATCTTTGGGCACCTAGTGGTCATG

314 CATGACCACTAGGAGCATCTTTGGCGACGGCGTGGGGCCATCTGTGCGGCGGATCCCCTAGGGGAATAAATCTTTGGGCACCTAGTGGTCATG

310 CATGACCACTAGGAGCATCTTTGGCGAGATCGGGAGAATCGGTGGCATTGGTTTCTCCTAGGGGAATAAATCTTTGGGCACCTAGTGGTCATG

307 CATGACCACTAGGAGCATCTTTGGCGAGATCGGGAGAATAGGTGGCATTGGTGTCTCCTAGGGGAATAAATCTTTGGGCACCTAGTGGTCATG

296 CATGACCACTAGGAGCATCTTTGGCGAGATCGGGAGAATCGATGGCATTGGTGTCTCCTAGGGGAATAAATCTTTGGGCACCTAGTGGTCATG

285 CATGACCACTAGGAGCATCTTTGGCGAGATCGGGAGAATCGGTGGCATTGGTATCTCCTAGGGGAATAAATCTTTGGGCACCTAGTGGTCATG

283 CATGACCACTAGGAGCATCTTTGGCGAGATCGGGAGAATCGGTTGCATTGGTGTCTCCTAGGGGAATAAATCTTTGGGCACCTAGTGGTCATG

272 CATGACCACTAGGAGCATCTTTGGCGAGATCGGGAGAATCGCTGGCATTGGTGTCTCCTAGGGGAATAAATCTTTGGGCACCTAGTGGTCATG

270 CATGACCACTAGGAGCATCTTTGGCGACCGTAGGAAAACAAAAGTGGCCTTGCTAGCCTAGGGGAATAAATCTTTGGGCACCTAGTGGTCATG

259 CATGACCACTAGGAGCATCTTTGGCGAGATCGGGAGAATCGGTGGCAATGGTGTCTCCTAGGGGAATAAATCTTTGGGCACCTAGTGGTCATG

254 CATGACCACTAGGAGCATCTTTGGCGAGAAGACCCTGGATTCGGGGACCAGTTGCTGCTAGGGGAATAAATCTTTGGGCACCTAGTGGTCATG

252 CATGACCACTAGGAGCATCTTTGGCGAGAAGACTCTGTATTCGGGGACCAGTTGCTGCTAGGGGAATAAATCTTTGGGCACCTAGTGGTCATG

249 CATGACCACTAGGAGCATCTTTGGCGACCGAGTGGGCTGGGAGGAAGTATTTCGGAGCTAGGGGAATAAATCTTTGGGCACCTAGTGGTCATG

234 CATGACCACTAGGAGCATCTTTGGCGATCAGGGGGCCTTTGATGGCGACTCAGTGGGCTAGGGGAATAAATCTTTGGGCACCTAGTGGTCATG

230 CATGACCACTAGGAGCATCTTTGGCGAACGGTAGGTGTTGCGGTGGTCTGTGAATCCCTAGGGGAATAAATCTTTGGGCACCTAGTGGTCATG

226 CATGACCACTAGGAGCATCTTTGGCGAGATCGGGAAAATCGGTGGCATTGGTGTCTCCTAGGGGAATAAATCTTTGGGCACCTAGTGGTCATG

224 CATGACCACTAGGAGCATCTTTGGCGAACGTGCAAGATATGCCGCCTGGAGCGTAGGCTAGGGGAATAAATCTTTGGGCACCTAGTGGTCATG

222 CATGACCACTAGGAGCATCTTTGGCGAACGGCAGGTGTTGCGGTGGTCCGTGAATCCCTAGGGGAATAAATCTTTGGGCACCTAGTGGTCATG

222 CATGACCACTAGGAGCATCTTTGGCGAGATCGGGAGTATCGGTGGCATTGGTGTCTCCTAGGGGAATAAATCTTTGGGCACCTAGTGGTCATG

220 CATGACCACTAGGAGCATCTTTGGCGAGAAGACTCTGGATTTGGGGACCAGTTGCTGCTAGGGGAATAAATCTTTGGGCACCTAGTGGTCATG

216 CATGACCACTAGGAGCATCTTTGGCGAGAGAATCGGTAGGCAGAGTGCTCCAGCCAGCTAGGGGAATAAATCTTTGGGCACCTAGTGGTCATG

213 CATGACCACTAGGAGCATCTTTGGCGAGATCGGGAGAATCGGTGCCATTGGTGTCTCCTAGGGGAATAAATCTTTGGGCACCTAGTGGTCATG

210 CATGACCACTAGGAGCATCTTTGGCGAACGGCGGGTGTTGCGGTGGTCTGTGAATCCCTAGGGGAATAAATCTTTGGGCACCTAGTGGTCATG

208 CATGACCACTAGGAGCATCTTTGGCGAGATCGGGAGAATCGGTGGCCTTGGTGTCTCCTAGGGGAATAAATCTTTGGGCACCTAGTGGTCATG

196 CATGACCACTAGGAGCATCTTTGGCGATCCGGGGGCATGCTATGCGACGCTTATGGGCTAGGGGAATAAATCTTTGGGCACCTAGTGGTCATG

187 CATGACCACTAGGAGCATCTTTGGCGATCCGGGGGTCACAAGTGACGGCGCATAGGGCTAGGGGAATAAATCTTTGGGCACCTAGTGGTCATG

185 CATGACCACTAGGAGCATCTTTGGCGAGAAGACTCTGGATTCGGGGACCAGTTGTTGCTAGGGGAATAAATCTTTGGGCACCTAGTGGTCATG

179 CATGACCACTAGGAGCATCTTTGGCGAGATCCGGAGAATCGGTGGCATTGGTGTCTCCTAGGGGAATAAATCTTTGGGCACCTAGTGGTCATG

178 CATGACCACTAGGAGCATCTTTGGCGAAGCGTGTTGCAAGGTCCGCGAGTGTGGACCCTAGGGGAATAAATCTTTGGGCACCTAGTGGTCATG

177 CATGACCACTAGGAGCATCTTTGGCGAGAACGGGAGAATCGGTGGCATTGGTGTCTCCTAGGGGAATAAATCTTTGGGCACCTAGTGGTCATG

170 CATGACCACTAGGAGCATCTTTGGCGAGAAGACTCAGGATTCGGGGACCAGTTGCTGCTAGGGGAATAAATCTTTGGGCACCTAGTGGTCATG

170 CATGACCACTAGGAGCATCTTTGGCGAACGGCAGGGGTTGCGGTGGTCTGTGAATCCCTAGGGGAATAAATCTTTGGGCACCTAGTGGTCATG

170 CATGACCACTAGGAGCATCTTTGGCGAGGGGTAGGGGGCCGCTCGCGCCTGCGTGCGCTAGGGGAATAAATCTTTGGGCACCTAGTGGTCATG

169 CATGACCACTAGGAGCATCTTTGGCGAGATCGGGAGAATCGGTGGCATTGCTGTCTCCTAGGGGAATAAATCTTTGGGCACCTAGTGGTCATG

168 CATGACCACTAGGAGCATCTTTGGCGAGATCGGGAGAATCGGTGGCATTGGTGTATCCTAGGGGAATAAATCTTTGGGCACCTAGTGGTCATG

167 CATGACCACTAGGAGCATCTTTGGCGAACGGCAGGTGTTGCGGTGGTCTGTGAATCACTAGGGGAATAAATCTTTGGGCACCTAGTGGTCATG

163 CATGACCACTAGGAGCATCTTTGGCGATCAGGCCGCCAGGGCGTACGAAGTGTGGTTCTAGGGGAATAAATCTTTGGGCACCTAGTGGTCATG

161 CATGACCACTAGGAGCATCTTTGGCGATGGGTGCTGACGGGGCTAGTGAGCCCTACACTAGGGGAATAAATCTTTGGGCACCTAGTGGTCATG

161 CATGACCACTAGGAGCATCTTTGGCGATAGGCGTGTACGGTCGAGCGAAAGCGATAGCTAGGGGAATAAATCTTTGGGCACCTAGTGGTCATG

157 CATGACCACTAGGAGCATCTTTGGCGAACGGCAGGTGTTGCGGTGGCCTGTGAATCCCTAGGGGAATAAATCTTTGGGCACCTAGTGGTCATG

154 CATGACCACTAGGAGCATCTTTGGCGAGCCGATTGTGCAACAATGTGTTCTGGGTAACTAGGGGAATAAATCTTTGGGCACCTAGTGGTCATG

154 CATGACCACTAGGAGCATCTTTGGCGATAGCGAAGTGCTGTGAACAGTGGTTAGCACCTAGGGGAATAAATCTTTGGGCACCTAGTGGTCATG

147 CATGACCACTAGGAGCATCTTTGGCGAGATCGGGTGAATCGGTGGCATTGGTGTCTCCTAGGGGAATAAATCTTTGGGCACCTAGTGGTCATG

145 CATGACCACTAGGAGCATCTTTGGCGAACGGCAGGTGTTGTGGTGGTCTGTGAATCCCTAGGGGAATAAATCTTTGGGCACCTAGTGGTCATG

140 CATGACCACTAGGAGCATCTTTGGCGACCGGCAGGTGTTGCGGTGGTCTGTGAATCCCTAGGGGAATAAATCTTTGGGCACCTAGTGGTCATG

140 CATGACCACTAGGAGCATCTTTGGCGAACGGCAGGTGTTGCGGTGGTCTGCGAATCCCTAGGGGAATAAATCTTTGGGCACCTAGTGGTCATG

139 CATGACCACTAGGAGCATCTTTGGCGAACGGCAGGTGCTGCGGTGGTCTGTGAATCCCTAGGGGAATAAATCTTTGGGCACCTAGTGGTCATG

138 CATGACCACTAGGAGCATCTTTGGCGATAGTCGGTAGAACAGGGTGGGGTGCTGTCCCTAGGGGAATAAATCTTTGGGCACCTAGTGGTCATG

133 CATGACCACTAGGAGCATCTTTGGCGAGATCGGGCGAATCGGTGGCATTGGTGTCTCCTAGGGGAATAAATCTTTGGGCACCTAGTGGTCATG

132 CATGACCACTAGGAGCATCTTTGGCGAGATCGGGAGAATCGGTGGGATTGGTGTCTCCTAGGGGAATAAATCTTTGGGCACCTAGTGGTCATG

131 CATGACCACTAGGAGCATCTTTGGCGAAGTGCGGTGGGCATGTGGGTGGTAGCGACGCTAGGGGAATAAATCTTTGGGCACCTAGTGGTCATG

127 CATGACCACTAGGAGCATCTTTGGCGAGAAGATTCTGGATTCGGGGACCAGTTGCTGCTAGGGGAATAAATCTTTGGGCACCTAGTGGTCATG

125 CATGACCACTAGGAGCATCTTTGGCGAGAAGACTCTGGATTCGGGGATCAGTTGCTGCTAGGGGAATAAATCTTTGGGCACCTAGTGGTCATG

120 CATGACCACTAGGAGCATCTTTGGCGAGAGCGGGAGAATCGGTGGCATTGGTGTCTCCTAGGGGAATAAATCTTTGGGCACCTAGTGGTCATG

117 CATGACCACTAGGAGCATCTTTGGCGAGGCGAGTCCAGCGGCCTTCTTGGCCTGCGACTAGGGGAATAAATCTTTGGGCACCTAGTGGTCATG

115 CATGACCACTAGGAGCATCTTTGGCGAACACGAGAGTCAAGTGCGTATAGCGTGGAGCTAGGGGAATAAATCTTTGGGCACCTAGTGGTCATG

114 CATGACCACTAGGAGCATCTTTGGCGAACGGCAGGTGTTGCGGTGGTCTTTGAATCCCTAGGGGAATAAATCTTTGGGCACCTAGTGGTCATG

114 CATGACCACTAGGAGCATCTTTGGCGAGATCGGGAGAATCGGTGGCATTCGTGTCTCCTAGGGGAATAAATCTTTGGGCACCTAGTGGTCATG

111 CATGACCACTAGGAGCATCTTTGGCGAGATCGGGACAATCGGTGGCATTGGTGTCTCCTAGGGGAATAAATCTTTGGGCACCTAGTGGTCATG

111 CATGACCACTAGGAGCATCTTTGGCGAACGGCAGGCGTTGCGGTGGTCTGTGAATCCCTAGGGGAATAAATCTTTGGGCACCTAGTGGTCATG

107 CATGACCACTAGGAGCATCTTTGGCGATCGCACAGCGCACGAAGCCATATGAGGGGACTAGGGGAATAAATCTTTGGGCACCTAGTGGTCATG

107 CATGACCACTAGGAGCATCTTTGGCGATCCGGGGGCCACTGAGGCGACGCTAATGGGCTAGGGGAATAAATCTTTGGGCACCTAGTGGTCATG

106 CATGACCACTAGGAGCATCTTTGGCGAACGGCAGGTGTTGCGGCGGTCTGTGAATCCCTAGGGGAATAAATCTTTGGGCACCTAGTGGTCATG

105 CATGACCACTAGGAGCATCTTTGGCGAGATCGGGAGAATCGGTGGCATGGGTGTCTCCTAGGGGAATAAATCTTTGGGCACCTAGTGGTCATG

103 CATGACCACTAGGAGCATCTTTGGCGAACGGCAGGTGTTGCGGTGGTCTGTGAGTCCCTAGGGGAATAAATCTTTGGGCACCTAGTGGTCATG

102 CATGACCACTAGGAGCATCTTTGGCGAGATCGGGAGAATCGGTGGCATTGGTCTCTCCTAGGGGAATAAATCTTTGGGCACCTAGTGGTCATG

102 CATGACCACTAGGAGCATCTTTGGCGAGAAGACTCTGGATTCGGGGGCCAGTTGCTGCTAGGGGAATAAATCTTTGGGCACCTAGTGGTCATG

100 CATGACCACTAGGAGCATCTTTGGCGACTCCAGTTCATCTGTCGGGCAGTTTAAGGACTAGGGGAATAAATCTTTGGGCACCTAGTGGTCATG

100 CATGACCACTAGGAGCATCTTTGGCGACGGTGTGGGGCCATCTGTGCGGCGGATCCCCTAGGGGAATAAATCTTTGGGCACCTAGTGGTCATG

99 CATGACCACTAGGAGCATCTTTGGCGACGTAGTGGGCTGGCAGGGAGTACTTGTTGCCTAGGGGAATAAATCTTTGGGCACCTAGTGGTCATG

98 CATGACCACTAGGAGCATCTTTGGCGAGATCGGGAGAATCGGTGGCATTGGGGTCTCCTAGGGGAATAAATCTTTGGGCACCTAGTGGTCATG

96 CATGACCACTAGGAGCATCTTTGGCGAACGGCAGGTGTTGCGGTGGTCTGTGAATCGCTAGGGGAATAAATCTTTGGGCACCTAGTGGTCATG

92 CATGACCACTAGGAGCATCTTTGGCGAGTTCGGGAGAATCGGTGGCATTGGTGTCTCCTAGGGGAATAAATCTTTGGGCACCTAGTGGTCATG

91 CATGACCACTAGGAGCATCTTTGGCGAGAATACTCTGGATTCGGGGACCAGTTGCTGCTAGGGGAATAAATCTTTGGGCACCTAGTGGTCATG

90 CATGACCACTAGGAGCATCTTTGGCGACGGCGTGGGGCCATTTGTGCGGCGGATCCCCTAGGGGAATAAATCTTTGGGCACCTAGTGGTCATG

88 CATGACCACTAGGAGCATCTTTGGCGAGAAGACTCTGGGTTCGGGGACCAGTTGCTGCTAGGGGAATAAATCTTTGGGCACCTAGTGGTCATG

88 CATGACCACTAGGAGCATCTTTGGCGAACGGCAGGTGTCGCGGTGGTCTGTGAATCCCTAGGGGAATAAATCTTTGGGCACCTAGTGGTCATG

85 CATGACCACTAGGAGCATCTTTGGCGAAAGTCGGTAGAACAGGGTGGGGTGCTGTCTCTAGGGGAATAAATCTTTGGGCACCTAGTGGTCATG

85 CATGACCACTAGGAGCATCTTTGGCGAACGGCAGGTGTTGCTGTGGTCTGTGAATCCCTAGGGGAATAAATCTTTGGGCACCTAGTGGTCATG

84 CATGACCACTAGGAGCATCTTTGGCGAACGGCAGGTGTTGCGGTGGTATGTGAATCCCTAGGGGAATAAATCTTTGGGCACCTAGTGGTCATG

84 CATGACCACTAGGAGCATCTTTGGCGATTTGGGAGTCGCAGTATGCAGGCAGTGAGACTAGGGGAATAAATCTTTGGGCACCTAGTGGTCATG

83 CATGACCACTAGGAGCATCTTTGGCGAGATCGGGAGAATCGGTGGCATTGGTGGCTCCTAGGGGAATAAATCTTTGGGCACCTAGTGGTCATG

83 CATGACCACTAGGAGCATCTTTGGCGAGATCGGGAGAATCGGGGGCATTGGTGTCTCCTAGGGGAATAAATCTTTGGGCACCTAGTGGTCATG

82 CATGACCACTAGGAGCATCTTTGGCGACTAAGTGGTCAACGCGTTAAGTGGGGTAGACTAGGGGAATAAATCTTTGGGCACCTAGTGGTCATG

81 CATGACCACTAGGAGCATCTTTGGCGAAGCAGTGCGACGGGCTATGGGTTCCTCGAGCTAGGGGAATAAATCTTTGGGCACCTAGTGGTCATG

80 CATGACCACTAGGAGCATCTTTGGCGAAGACGACACAGTCGATCGGGTTAGAGGAAGCTAGGGGAATAAATCTTTGGGCACCTAGTGGTCATG

79 CATGACCACTAGGAGCATCTTTGGCGAATGGCCGGCACGGCCTCCTAGTCCTCGGTACTAGGGGAATAAATCTTTGGGCACCTAGTGGTCATG

79 CATGACCACTAGGAGCATCTTTGGCGAACGGCAGGTGTTGCGGTGGTCTCTGAATCCCTAGGGGAATAAATCTTTGGGCACCTAGTGGTCATG

77 CATGACCACTAGGAGCATCTTTGGCGAGATGGGGAGAATCGGTGGCATTGGTGTCTCCTAGGGGAATAAATCTTTGGGCACCTAGTGGTCATG

77 CATGACCACTAGGAGCATCTTTGGCGAGATCGGGAGAATCGGTGGCTTTGGTGTCTCCTAGGGGAATAAATCTTTGGGCACCTAGTGGTCATG

76 CATGACCACTAGGAGCATCTTTGGCGAGAAGGCTCTGGATTCGGGGACCAGTTGCTGCTAGGGGAATAAATCTTTGGGCACCTAGTGGTCATG

76 CATGACCACTAGGAGCATCTTTGGCGAACGGCAGGTGTTGCAGTGGTCTGTGAATCCCTAGGGGAATAAATCTTTGGGCACCTAGTGGTCATG

75 CATGACCACTAGGAGCATCTTTGGCGAGATCGGGAGAATCGGTCGCATTGGTGTCTCCTAGGGGAATAAATCTTTGGGCACCTAGTGGTCATG

75 CATGACCACTAGGAGCATCTTTGGCGAGAAGACTCTGGATTCGGGGACCAGTTGCTACTAGGGGAATAAATCTTTGGGCACCTAGTGGTCATG

74 CATGACCACTAGGAGCATCTTTGGCGAACGGCATGTGTTGCGGTGGTCTGTGAATCCCTAGGGGAATAAATCTTTGGGCACCTAGTGGTCATG

74 CATGACCACTAGGAGCATCTTTGGCGACGTCGTAAAGTGCGTAGAGTGGTAAGTCGACTAGGGGAATAAATCTTTGGGCACCTAGTGGTCATG

71 CATGACCACTAGGAGCATCTTTGGCGAAGACGACACAGTCGATCGGGATAGAGGAAGCTAGGGGAATAAATCTTTGGGCACCTAGTGGTCATG

71 CATGACCACTAGGAGCATCTTTGGCGAGAAGACTCTGGATTCGGGGACCGGTTGCTGCTAGGGGAATAAATCTTTGGGCACCTAGTGGTCATG

70 CATGACCACTAGGAGCATCTTTGGCGAGATCGGGAGAATCCGTGGCATTGGTGTCTCCTAGGGGAATAAATCTTTGGGCACCTAGTGGTCATG

70 CATGACCACTAGGAGCATCTTTGGCGACGTCGGCTGGTAGAGACTGTATTGAGGAACCTAGGGGAATAAATCTTTGGGCACCTAGTGGTCATG

69 CATGACCACTAGGAGCATCTTTGGCGACGACTTCGGAGTGGGTGCGCCGCTGGCATACTAGGGGAATAAATCTTTGGGCACCTAGTGGTCATG

69 CATGACCACTAGGAGCATCTTTGGCGAACGGCAGGTGTTGCGGTGGTCTGTGCATCCCTAGGGGAATAAATCTTTGGGCACCTAGTGGTCATG

67 CATGACCACTAGGAGCATCTTTGGCGACATCGGGAGAATCGGTGGCATTGGTGTCTCCTAGGGGAATAAATCTTTGGGCACCTAGTGGTCATG

66 CATGACCACTAGGAGCATCTTTGGCGAGCTCGGGAGAATCGGTGGCATTGGTGTCTCCTAGGGGAATAAATCTTTGGGCACCTAGTGGTCATG

65 CATGACCACTAGGAGCATCTTTGGCGAGAAGACTCTGGACTCGGGGACCAGTTGCTGCTAGGGGAATAAATCTTTGGGCACCTAGTGGTCATG

65 CATGACCACTAGGAGCATCTTTGGCGAACGGAAGGTGTTGCGGTGGTCTGTGAATCCCTAGGGGAATAAATCTTTGGGCACCTAGTGGTCATG

61 CATGACCACTAGGAGCATCTTTGGCGAACGGCTGGTGTTGCGGTGGTCTGTGAATCCCTAGGGGAATAAATCTTTGGGCACCTAGTGGTCATG

61 CATGACCACTAGGAGCATCTTTGGCGAACGGCAGTTGTTGCGGTGGTCTGTGAATCCCTAGGGGAATAAATCTTTGGGCACCTAGTGGTCATG

60 CATGACCACTAGGAGCATCTTTGGCGAACGGCAGGTGTTTCGGTGGTCTGTGAATCCCTAGGGGAATAAATCTTTGGGCACCTAGTGGTCATG

60 CATGACCACTAGGAGCATCTTTGGCGAACTGCAGGTGTTGCGGTGGTCTGTGAATCCCTAGGGGAATAAATCTTTGGGCACCTAGTGGTCATG

60 CATGACCACTAGGAGCATCTTTGGCGAACGGCAGGTGTTGCGGTGGTCTGTAAATCCCTAGGGGAATAAATCTTTGGGCACCTAGTGGTCATG

59 CATGACCACTAGGAGCATCTTTGGCGATCAAGTGCGCTTGGTAATTCCTGATGCGATCTAGGGGAATAAATCTTTGGGCACCTAGTGGTCATG

58 CATGACCACTAGGAGCATCTTTGGCGACGAGTGGGCACGGTACCACGCGACATAGTGCTAGGGGAATAAATCTTTGGGCACCTAGTGGTCATG

58 CATGACCACTAGGAGCATCTTTGGCGAACGGCAAGTGTTGCGGTGGTCTGTGAATCCCTAGGGGAATAAATCTTTGGGCACCTAGTGGTCATG

57 CATGACCACTAGGAGCATCTTTGGCGAACAGCAGGTGTTGCGGTGGTCTGTGAATCCCTAGGGGAATAAATCTTTGGGCACCTAGTGGTCATG

57 CATGACCACTAGGAGCATCTTTGGCGAACGTCAGGTGTTGCGGTGGTCTGTGAATCCCTAGGGGAATAAATCTTTGGGCACCTAGTGGTCATG

56 CATGACCACTAGGAGCATCTTTGGCGAACGTGCAAGAAATGCCGCCTGGAGCGTAGGCTAGGGGAATAAATCTTTGGGCACCTAGTGGTCATG

56 CATGACCACTAGGAGCATCTTTGGCGACGAGATTTGGTAGGTAGATGTGCTCTAGCACTAGGGGAATAAATCTTTGGGCACCTAGTGGTCATG

56 CATGACCACTAGGAGCATCTTTGGCGAGAAGACTCTGGATTCGGGGACCAGCTGCTGCTAGGGGAATAAATCTTTGGGCACCTAGTGGTCATG

55 CATGACCACTAGGAGCATCTTTGGCGAAAGTCGGTAGAACAGGGTGGGGTGCTGTTCCTAGGGGAATAAATCTTTGGGCACCTAGTGGTCATG

55 CATGACCACTAGGAGCATCTTTGGCGAACGGCAGGTGTTGAGGTGGTCTGTGAATCCCTAGGGGAATAAATCTTTGGGCACCTAGTGGTCATG

55 CATGACCACTAGGAGCATCTTTGGCGAAAGTCGATAGAACAGGGTGGGGTGCTGTCCCTAGGGGAATAAATCTTTGGGCACCTAGTGGTCATG

55 CATGACCACTAGGAGCATCTTTGGCGAGAAGACTCTGGATTCGGGTACCAGTTGCTGCTAGGGGAATAAATCTTTGGGCACCTAGTGGTCATG

55 CATGACCACTAGGAGCATCTTTGGCGAACGGCAGGAGTTGCGGTGGTCTGTGAATCCCTAGGGGAATAAATCTTTGGGCACCTAGTGGTCATG

53 CATGACCACTAGGAGCATCTTTGGCGAACGGCAGGTGTTACGGTGGTCTGTGAATCCCTAGGGGAATAAATCTTTGGGCACCTAGTGGTCATG

52 CATGACCACTAGGAGCATCTTTGGCGATCCGGGGGTCACATCAGGACGACGCTAGGGCTAGGGGAATAAATCTTTGGGCACCTAGTGGTCATG

52 CATGACCACTAGGAGCATCTTTGGCGAGAAGACTATGGATTCGGGGACCAGTTGCTGCTAGGGGAATAAATCTTTGGGCACCTAGTGGTCATG

52 CATGACCACTAGGAGCATCTTTGGCGAGAAGACTCTGGATTCGGGGACCAGTCGCTGCTAGGGGAATAAATCTTTGGGCACCTAGTGGTCATG

51 CATGACCACTAGGAGCATCTTTGGCGAACGGCAGGTGTTGCGGTGTTCTGTGAATCCCTAGGGGAATAAATCTTTGGGCACCTAGTGGTCATG

51 CATGACCACTAGGAGCATCTTTGGCGAACATCGTGGTCGGAAGGCGTCGGCGGAGTGCTAGGGGAATAAATCTTTGGGCACCTAGTGGTCATG

51 CATGACCACTAGGAGCATCTTTGGCGAACGGCAGGTGTTGCGGTGGTCAGTGAATCCCTAGGGGAATAAATCTTTGGGCACCTAGTGGTCATG

51 CATGACCACTAGGAGCATCTTTGGCGAGGGGGGGCGCCGGCAGCGGTGTGAATGCGCCTAGGGGAATAAATCTTTGGGCACCTAGTGGTCATG

50 CATGACCACTAGGAGCATCTTTGGCGAACGGCAGGTGTTGCGGTGGTCTGAGAATCCCTAGGGGAATAAATCTTTGGGCACCTAGTGGTCATG

49 CATGACCACTAGGAGCATCTTTGGCGAACGGCAGGTGTTGCGTTGGTCTGTGAATCCCTAGGGGAATAAATCTTTGGGCACCTAGTGGTCATG

49 CATGACCACTAGGAGCATCTTTGGCGAGAGGACTCTGGATTCGGGGACCAGTTGCTGCTAGGGGAATAAATCTTTGGGCACCTAGTGGTCATG

48 CATGACCACTAGGAGCATCTTTGGCGAGATTGGGAGAATCGGCGGCATTGGTGTCTCCTAGGGGAATAAATCTTTGGGCACCTAGTGGTCATG

48 CATGACCACTAGGAGCATCTTTGGCGATCGGCAGGTGTTGCGGTGGTCTGTGAATCCCTAGGGGAATAAATCTTTGGGCACCTAGTGGTCATG

47 CATGACCACTAGGAGCATCTTTGGCGAGGGAGTGGTCGGCCATGTGGGGGGTCGGGGCTAGGGGAATAAATCTTTGGGCACCTAGTGGTCATG

47 CATGACCACTAGGAGCATCTTTGGCGAGATCGGGAGCATCGGTGGCATTGGTGTCTCCTAGGGGAATAAATCTTTGGGCACCTAGTGGTCATG

47 CATGACCACTAGGAGCATCTTTGGCGAAAGGCAGGTGTTGCGGTGGTCTGTGAATCCCTAGGGGAATAAATCTTTGGGCACCTAGTGGTCATG

47 CATGACCACTAGGAGCATCTTTGGCGAACGGCAGATGTTGCGGTGGTCTGTGAATCCCTAGGGGAATAAATCTTTGGGCACCTAGTGGTCATG

47 CATGACCACTAGGAGCATCTTTGGCGATCCGGGGGTCAGATAAGACGGCGCGTTGGGCTAGGGGAATAAATCTTTGGGCACCTAGTGGTCATG

47 CATGACCACTAGGAGCATCTTTGGCGAACGGCAGGTGTTGCGGAGGTCTGTGAATCCCTAGGGGAATAAATCTTTGGGCACCTAGTGGTCATG

47 CATGACCACTAGGAGCATCTTTGGCGAACGGCAGGTGTTGCGGTGGTCGGTGAATCCCTAGGGGAATAAATCTTTGGGCACCTAGTGGTCATG

46 CATGACCACTAGGAGCATCTTTGGCGAGGGTGGGCGCCGGCAGCGGTGTGAATGCGCCTAGGGGAATAAATCTTTGGGCACCTAGTGGTCATG

46 CATGACCACTAGGAGCATCTTTGGCGAGTGGGGGGCGCCGGCCGGTAGTGGGTGAGACTAGGGGAATAAATCTTTGGGCACCTAGTGGTCATG

44 CATGACCACTAGGAGCATCTTTGGCGACGGTGTGGGGAACTTGTTTCGGCGGTGCTCCTAGGGGAATAAATCTTTGGGCACCTAGTGGTCATG

44 CATGACCACTAGGAGCATCTTTGGCGAGAAGACTCTGGATTCGGGGATTAGTTGCTGCTAGGGGAATAAATCTTTGGGCACCTAGTGGTCATG

43 CATGACCACTAGGAGCATCTTTGGCGAAAGTTGGTAGAACAGGGTGGGGTGCTGTCCCTAGGGGAATAAATCTTTGGGCACCTAGTGGTCATG

43 CATGACCACTAGGAGCATCTTTGGCGAGGGGGGGCGCCGGCAGCGGTGTGAATGCGACTAGGGGAATAAATCTTTGGGCACCTAGTGGTCATG

42 CATGACCACTAGGAGCATCTTTGGCGAGGAGACTCTGGATTCGGGGACCAGTTGCTGCTAGGGGAATAAATCTTTGGGCACCTAGTGGTCATG

42 CATGACCACTAGGAGCATCTTTGGCGAGAAGACTCTGGATTCAGGGACCAGTTGCTGCTAGGGGAATAAATCTTTGGGCACCTAGTGGTCATG

42 CATGACCACTAGGAGCATCTTTGGCGAGAAGACTCTGAATTCGGGGACCAGTTGCTGCTAGGGGAATAAATCTTTGGGCACCTAGTGGTCATG

41 CATGACCACTAGGAGCATCTTTGGCGAACGGCAGGTGTTGCGGTGGTCTGTTAATCCCTAGGGGAATAAATCTTTGGGCACCTAGTGGTCATG

41 CATGACCACTAGGAGCATCTTTGGCGAGATCGGGAGAATGGGTGGCATTGGTGTCTCCTAGGGGAATAAATCTTTGGGCACCTAGTGGTCATG

40 CATGACCACTAGGAGCATCTTTGGCGAGATCGGGAGAATCGGTGGCATTGGTGTGTCCTAGGGGAATAAATCTTTGGGCACCTAGTGGTCATG

40 CATGACCACTAGGAGCATCTTTGGCGAGAAGACACTGGATTCGGGGACCAGTTGCTGCTAGGGGAATAAATCTTTGGGCACCTAGTGGTCATG

40 CATGACCACTAGGAGCATCTTTGGCGAGAAGACTCTGGATTCGGGGACCAGTTGCCGCTAGGGGAATAAATCTTTGGGCACCTAGTGGTCATG

39 CATGACCACTAGGAGCATCTTTGGCGACGAGGGGCACGGGTGGTCATTGCGAGAAGACTAGGGGAATAAATCTTTGGGCACCTAGTGGTCATG

39 CATGACCACTAGGAGCATCTTTGGCGACGGTGTGGGGACCGCAGGTTCGGCGGGTACCTAGGGGAATAAATCTTTGGGCACCTAGTGGTCATG

39 CATGACCACTAGGAGCATCTTTGGCGAACGGCAGGTGTTGCGATGGTCTGTGAATCCCTAGGGGAATAAATCTTTGGGCACCTAGTGGTCATG

39 CATGACCACTAGGAGCATCTTTGGCGAGAAGACTCTGGATTCGTGGACCAGTTGCTGCTAGGGGAATAAATCTTTGGGCACCTAGTGGTCATG

38 CATGACCACTAGGAGCATCTTTGGCGAACGGCAGGTGTTGCGGTTGTCTGTGAATCCCTAGGGGAATAAATCTTTGGGCACCTAGTGGTCATG

38 CATGACCACTAGGAGCATCTTTGGCGAACGGCAGGTGTTGCGGTGGACTGTGAATCCCTAGGGGAATAAATCTTTGGGCACCTAGTGGTCATG

37 CATGACCACTAGGAGCATCTTTGGCGAGAAGACGCTGGATTCGGGGACCAGTTGCTGCTAGGGGAATAAATCTTTGGGCACCTAGTGGTCATG

37 CATGACCACTAGGAGCATCTTTGGCGAACGGCAGGTGTTGCGGTGGTCTGTGAATACCTAGGGGAATAAATCTTTGGGCACCTAGTGGTCATG

37 CATGACCACTAGGAGCATCTTTGGCGAGAAGACTTTGGATTCGGGGACCAGTTGCTCCTAGGGGAATAAATCTTTGGGCACCTAGTGGTCATG

37 CATGACCACTAGGAGCATCTTTGGCGAGAAGACTCTGGATTCGGGGACCAGTTGCTTCTAGGGGAATAAATCTTTGGGCACCTAGTGGTCATG

36 CATGACCACTAGGAGCATCTTTGGCGAACGGCAGGTGTTGCGGGGGTCTGTGAATCCCTAGGGGAATAAATCTTTGGGCACCTAGTGGTCATG

36 CATGACCACTAGGAGCATCTTTGGCGACGAGTGGGCGGAGCTTAACGCGGCATAGTGCTAGGGGAATAAATCTTTGGGCACCTAGTGGTCATG

36 CATGACCACTAGGAGCATCTTTGGCGAACGGCAGGTATTGCGGTGGTCTGTGAATCCCTAGGGGAATAAATCTTTGGGCACCTAGTGGTCATG

36 CATGACCACTAGGAGCATCTTTGGCGAACGACAGGTGTTGCGGTGGTCTGTGAATCCCTAGGGGAATAAATCTTTGGGCACCTAGTGGTCATG

36 CATGACCACTAGGAGCATCTTTGGCGAGAAGACTCTGGATTCGGGGACCAGATGCTGCTAGGGGAATAAATCTTTGGGCACCTAGTGGTCATG

36 CATGACCACTAGGAGCATCTTTGGCGAACGGCAGGTGTTGCGGTAGTCTGTGAATCCCTAGGGGAATAAATCTTTGGGCACCTAGTGGTCATG

35 CATGACCACTAGGAGCATCTTTGGCGAACGGCAGGTGATGCGGTGGTCTGTGAATCCCTAGGGGAATAAATCTTTGGGCACCTAGTGGTCATG

35 CATGACCACTAGGAGCATCTTTGGCGAAAGTCGTTAGAACAGGGTGGGGTGCTGTCCCTAGGGGAATAAATCTTTGGGCACCTAGTGGTCATG

35 CATGACCACTAGGAGCATCTTTGGCGACCGTAGGAAAACAAAAGTGCCCTTGCTAGCCTAGGGGAATAAATCTTTGGGCACCTAGTGGTCATG

35 CATGACCACTAGGAGCATCTTTGGCGAAAGTCGGCAGAACAGGGTGGGGTGCTGTCCCTAGGGGAATAAATCTTTGGGCACCTAGTGGTCATG

35 CATGACCACTAGGAGCATCTTTGGCGAGATCGAAAGAATCGGTGGCATTGGTGTCTCCTAGGGGAATAAATCTTTGGGCACCTAGTGGTCATG

35 CATGACCACTAGGAGCATCTTTGGCGACGGAGTGGGGCTCATTGTGCGGCGGTAATCCTAGGGGAATAAATCTTTGGGCACCTAGTGGTCATG

34 CATGACCACTAGGAGCATCTTTGGCGACGGCGTGGGGCTATAGATGCGGCGGCACTCCTAGGGGAATAAATCTTTGGGCACCTAGTGGTCATG

34 CATGACCACTAGGAGCATCTTTGGCGATCCGGGGGGCTGTGCAGCCGACGCAAAGGGCTAGGGGAATAAATCTTTGGGCACCTAGTGGTCATG

34 CATGACCACTAGGAGCATCTTTGGCGAGATCAGGAGAATCGGCGGCATTGGTGTCTCCTAGGGGAATAAATCTTTGGGCACCTAGTGGTCATG

34 CATGACCACTAGGAGCATCTTTGGCGAGAAGACTCTAGATTCGGGGACCAGTTGCTGCTAGGGGAATAAATCTTTGGGCACCTAGTGGTCATG

34 CATGACCACTAGGAGCATCTTTGGCGAGAAAACTCTGGATTCGGGGACCAGTTGCTGCTAGGGGAATAAATCTTTGGGCACCTAGTGGTCATG

34 CATGACCACTAGGAGCATCTTTGGCGAACGGCCGGCACGGCCTTCTAGTCCTCGGTACTAGGGGAATAAATCTTTGGGCACCTAGTGGTCATG

34 CATGACCACTAGGAGCATCTTTGGCGATGGGTTGCTGGTAGGTGGTACGACCTCGATCTAGGGGAATAAATCTTTGGGCACCTAGTGGTCATG

34 CATGACCACTAGGAGCATCTTTGGCGAGCCTTGCTTGGGAGGTTGCTCTACCAGTTCCTAGGGGAATAAATCTTTGGGCACCTAGTGGTCATG

34 CATGACCACTAGGAGCATCTTTGGCGAGGGGCCTAAGTGCGTATCTGTGGTCGAGCTCTAGGGGAATAAATCTTTGGGCACCTAGTGGTCATG

34 CATGACCACTAGGAGCATCTTTGGCGAGAAGACTGTGGATTCGGGGACCAGTTGCTGCTAGGGGAATAAATCTTTGGGCACCTAGTGGTCATG

33 CATGACCACTAGGAGCATCTTTGGCGAGAGTCGGTAGAACAGGGTGGGGTGCTGTCCCTAGGGGAATAAATCTTTGGGCACCTAGTGGTCATG

33 CATGACCACTAGGAGCATCTTTGGCGATCCGGGGGGACTGGGTCCGACGCAATAGGGCTAGGGGAATAAATCTTTGGGCACCTAGTGGTCATG

33 CATGACCACTAGGAGCATCTTTGGCGAGAAGACTCTGGATTCGGTGACCAGTTGCTGCTAGGGGAATAAATCTTTGGGCACCTAGTGGTCATG

33 CATGACCACTAGGAGCATCTTTGGCGAGAAGACTCTGGATTCTGGGACCAGTTGCTGCTAGGGGAATAAATCTTTGGGCACCTAGTGGTCATG

33 CATGACCACTAGGAGCATCTTTGGCGAACGGCAGGTGTAGCGGTGGTCTGTGAATCCCTAGGGGAATAAATCTTTGGGCACCTAGTGGTCATG

32 CATGACCACTAGGAGCATCTTTGGCGAAAGTCGGTAGAACAGGGTGGGGTGTTGTCCCTAGGGGAATAAATCTTTGGGCACCTAGTGGTCATG

32 CATGACCACTAGGAGCATCTTTGGCGACGGCGTGGGGCTATTGATGCGGCGGTACTCCTAGGGGAATAAATCTTTGGGCACCTAGTGGTCATG

32 CATGACCACTAGGAGCATCTTTGGCGACTGTGGTCTCGTCCTAATTGTGCGGGCGTCCTAGGGGAATAAATCTTTGGGCACCTAGTGGTCATG

32 CATGACCACTAGGAGCATCTTTGGCGAGAAGACTCTGGAGTCGGGGACCAGTTGCTGCTAGGGGAATAAATCTTTGGGCACCTAGTGGTCATG

32 CATGACCACTAGGAGCATCTTTGGCGACAGCCGAAGGGACCAGAAGGGAGTCACTGTCTAGGGGAATAAATCTTTGGGCACCTAGTGGTCATG

32 CATGACCACTAGGAGCATCTTTGGCGAACGTCGGTAGAACAGGGTGGGGTGCTGTCCCTAGGGGAATAAATCTTTGGGCACCTAGTGGTCATG

32 CATGACCACTAGGAGCATCTTTGGCGAGATCGTGAGAATCGGCGGCATTGGTGTCTCCTAGGGGAATAAATCTTTGGGCACCTAGTGGTCATG

32 CATGACCACTAGGAGCATCTTTGGCGAGTGTGGGCTGGCAGTGCGTACTTGATCGCTCTAGGGGAATAAATCTTTGGGCACCTAGTGGTCATG

31 CATGACCACTAGGAGCATCTTTGGCGAGAAGACTCTGGATTCGGGGACCAGTAGCTGCTAGGGGAATAAATCTTTGGGCACCTAGTGGTCATG

31 CATGACCACTAGGAGCATCTTTGGCGAGCGACAAATGGAAATGCGACGTAGTGGACGCTAGGGGAATAAATCTTTGGGCACCTAGTGGTCATG

31 CATGACCACTAGGAGCATCTTTGGCGATCAGGCCGCCAGGGCGTACGAAGCGTGGTTCTAGGGGAATAAATCTTTGGGCACCTAGTGGTCATG

31 CATGACCACTAGGAGCATCTTTGGCGAGAAGACTCTGGATCCGGGGACCAGTTGCTGCTAGGGGAATAAATCTTTGGGCACCTAGTGGTCATG

30 CATGACCACTAGGAGCATCTTTGGCGAGATCGGGAGAATCGGCGGCATTGGTGTCTTCTAGGGGAATAAATCTTTGGGCACCTAGTGGTCATG

30 CATGACCACTAGGAGCATCTTTGGCGAGGTTGTAGCTCGCGCGTAGGGTCGGCAGTGCTAGGGGAATAAATCTTTGGGCACCTAGTGGTCATG

30 CATGACCACTAGGAGCATCTTTGGCGATAAGACTCTGGATTCGGGGACCAGTTGCTGCTAGGGGAATAAATCTTTGGGCACCTAGTGGTCATG

29 CATGACCACTAGGAGCATCTTTGGCGATTGGTAGTCGCGGATGCAGGCAGTGATGCTCTAGGGGAATAAATCTTTGGGCACCTAGTGGTCATG

29 CATGACCACTAGGAGCATCTTTGGCGAGAAGACTCTGGATTCGAGGACCAGTTGCTGCTAGGGGAATAAATCTTTGGGCACCTAGTGGTCATG

29 CATGACCACTAGGAGCATCTTTGGCGACTAAGTGGCCAACGCGTTAAATGGGGTAGACTAGGGGAATAAATCTTTGGGCACCTAGTGGTCATG

29 CATGACCACTAGGAGCATCTTTGGCGATCCGGGGGCCAAAGGCGACGCAAATTAGGGCTAGGGGAATAAATCTTTGGGCACCTAGTGGTCATG

29 CATGACCACTAGGAGCATCTTTGGCGAGCCTTGCTTGGGAGGTTGCTCCACTAGTTCCTAGGGGAATAAATCTTTGGGCACCTAGTGGTCATG

29 CATGACCACTAGGAGCATCTTTGGCGAGAAGACTCTGGATTCGGGGACCAGTTTCTGCTAGGGGAATAAATCTTTGGGCACCTAGTGGTCATG

28 CATGACCACTAGGAGCATCTTTGGCGATTGAAGCCGGTGCGGTGCGGCGAGATGCGACTAGGGGAATAAATCTTTGGGCACCTAGTGGTCATG

28 CATGACCACTAGGAGCATCTTTGGCGAACGGCAGGTGGTGCGGTGGTCTGTGAATCCCTAGGGGAATAAATCTTTGGGCACCTAGTGGTCATG

28 CATGACCACTAGGAGCATCTTTGGCGATCCGGGGGGCAGAGAGGCCGACGCACTGGGCTAGGGGAATAAATCTTTGGGCACCTAGTGGTCATG

28 CATGACCACTAGGAGCATCTTTGGCGAGATCGGGAGAATTGGCGGCATTGGTGTCTCCTAGGGGAATAAATCTTTGGGCACCTAGTGGTCATG

28 CATGACCACTAGGAGCATCTTTGGCGAACGGCAGGTGTTGCGGTGGTCTGTGAATTTCTAGGGGAATAAATCTTTGGGCACCTAGTGGTCATG

28 CATGACCACTAGGAGCATCTTTGGCGAGAAGACTCTGGATTCGGGGACAAGTTGCTGCTAGGGGAATAAATCTTTGGGCACCTAGTGGTCATG

28 CATGACCACTAGGAGCATCTTTGGCGAGATCGGGAGAAGCGGTGGCATTGGTGTCTCCTAGGGGAATAAATCTTTGGGCACCTAGTGGTCATG

27 CATGACCACTAGGAGCATCTTTGGCGACTCGTGGTCCTTACCCCCGTAGAGGGTTGTCTAGGGGAATAAATCTTTGGGCACCTAGTGGTCATG

27 CATGACCACTAGGAGCATCTTTGGCGAGAAGACTCTGGATTCGGGGACCAGTTGATGCTAGGGGAATAAATCTTTGGGCACCTAGTGGTCATG

27 CATGACCACTAGGAGCATCTTTGGCGAACGGCAGGTTTTGCGGTGGTCTGTGAATCCCTAGGGGAATAAATCTTTGGGCACCTAGTGGTCATG

27 CATGACCACTAGGAGCATCTTTGGCGAGGGATGGGAGCCGATGGGGGTTCGGGGTTTCTAGGGGAATAAATCTTTGGGCACCTAGTGGTCATG

27 CATGACCACTAGGAGCATCTTTGGCGAGAAGACTCTGGATTCGGGGACCATTTGCTGCTAGGGGAATAAATCTTTGGGCACCTAGTGGTCATG

27 CATGACCACTAGGAGCATCTTTGGCGACGTGGGCTGGAAGGATGTGGTACTTCTCGACTAGGGGAATAAATCTTTGGGCACCTAGTGGTCATG

27 CATGACCACTAGGAGCATCTTTGGCGATTTGGGAGTCGCAGTATGCAGGCAGTGAGGCTAGGGGAATAAATCTTTGGGCACCTAGTGGTCATG

26 CATGACCACTAGGAGCATCTTTGGCGAGTTGTCGTAGAGCCACGGGACACCCGTCAGCTAGGGGAATAAATCTTTGGGCACCTAGTGGTCATG

26 CATGACCACTAGGAGCATCTTTGGCGAAGTGAAGAGTGCGTGTAGTGGTCTGTTCGCCTAGGGGAATAAATCTTTGGGCACCTAGTGGTCATG

26 CATGACCACTAGGAGCATCTTTGGCGACGGCGAAAGTTCGGGCTGGGGAGTGTTGCTCTAGGGGAATAAATCTTTGGGCACCTAGTGGTCATG

26 CATGACCACTAGGAGCATCTTTGGCGAGATCGGGAGAATCGGCGGCATTGGTGTTTCCTAGGGGAATAAATCTTTGGGCACCTAGTGGTCATG

26 CATGACCACTAGGAGCATCTTTGGCGAGAAGACTCTGGATTCGGGAACCAGTTGCTGCTAGGGGAATAAATCTTTGGGCACCTAGTGGTCATG

25 CATGACCACTAGGAGCATCTTTGGCGAGCTTTGCTTGGGAGGTTGCTCCACCAGTTCCTAGGGGAATAAATCTTTGGGCACCTAGTGGTCATG

25 CATGACCACTAGGAGCATCTTTGGCGATGGGTGCAGACGAGTGCGCTTGCAATTACACTAGGGGAATAAATCTTTGGGCACCTAGTGGTCATG

25 CATGACCACTAGGAGCATCTTTGGCGAGAAGACTCTGGATTCGGAGACCAGTTGCTGCTAGGGGAATAAATCTTTGGGCACCTAGTGGTCATG

25 CATGACCACTAGGAGCATCTTTGGCGAATGGCCGGCACGGCCTTCTAGTCTTCGGTACTAGGGGAATAAATCTTTGGGCACCTAGTGGTCATG

25 CATGACCACTAGGAGCATCTTTGGCGAGGGGGCGAGTGGGCGAGGTGTTATAGGGAGCTAGGGGAATAAATCTTTGGGCACCTAGTGGTCATG

25 CATGACCACTAGGAGCATCTTTGGCGACAGCCGTAGCCGTAAGGTTGGTATAAGGCCCTAGGGGAATAAATCTTTGGGCACCTAGTGGTCATG

24 CATGACCACTAGGAGCATCTTTGGCGAGATCGGGAGAATCGGTGGCATTGGTGTCGCCTAGGGGAATAAATCTTTGGGCACCTAGTGGTCATG

24 CATGACCACTAGGAGCATCTTTGGCGAGCCTCGCTTGGGAGGTTGCTTCACCAGTTCCTAGGGGAATAAATCTTTGGGCACCTAGTGGTCATG

24 CATGACCACTAGGAGCATCTTTGGCGAGATCAAGAGAATCGGTGGCATTGGTGTCTCCTAGGGGAATAAATCTTTGGGCACCTAGTGGTCATG

24 CATGACCACTAGGAGCATCTTTGGCGATCGAGTGGGCTGGGAGGAAGTATTTCGGAGCTAGGGGAATAAATCTTTGGGCACCTAGTGGTCATG

24 CATGACCACTAGGAGCATCTTTGGCGACGATGGTTGGGAGGGATGTATTTCGGGCGACTAGGGGAATAAATCTTTGGGCACCTAGTGGTCATG

24 CATGACCACTAGGAGCATCTTTGGCGACATAGTGGATCGCAGTCCCGAAAAGGACGCCTAGGGGAATAAATCTTTGGGCACCTAGTGGTCATG

23 CATGACCACTAGGAGCATCTTTGGCGAGAAGAATCTGGATTCGGGGACCAGTTGCTGCTAGGGGAATAAATCTTTGGGCACCTAGTGGTCATG

23 CATGACCACTAGGAGCATCTTTGGCGATCCGGGGACACGCAGTGGGACGCACTTGGGCTAGGGGAATAAATCTTTGGGCACCTAGTGGTCATG

23 CATGACCACTAGGAGCATCTTTGGCGAACGGCAGGTGTTGCGGTGGGCTGTGAATCCCTAGGGGAATAAATCTTTGGGCACCTAGTGGTCATG

23 CATGACCACTAGGAGCATCTTTGGCGAGCACTACGTAGCGGTAGGCATTGTTGTCTCCTAGGGGAATAAATCTTTGGGCACCTAGTGGTCATG

23 CATGACCACTAGGAGCATCTTTGGCGAGATTGGGAGAATCGGTGGCATTGGTGTCTTCTAGGGGAATAAATCTTTGGGCACCTAGTGGTCATG

23 CATGACCACTAGGAGCATCTTTGGCGAAGGGCAGGTGTTGCGGTGGTCTGTGAATCCCTAGGGGAATAAATCTTTGGGCACCTAGTGGTCATG

22 CATGACCACTAGGAGCATCTTTGGCGATGGGGGGCGAGAGGACTGAGTAGGAATAAGCTAGGGGAATAAATCTTTGGGCACCTAGTGGTCATG

22 CATGACCACTAGGAGCATCTTTGGCGATCCGGGGGTCCGGGTGGACGACGCGACGGGCTAGGGGAATAAATCTTTGGGCACCTAGTGGTCATG

22 CATGACCACTAGGAGCATCTTTGGCGACAGCGGCGTAGGCTGCCTTGGGTGGTGGCCCTAGGGGAATAAATCTTTGGGCACCTAGTGGTCATG

22 CATGACCACTAGGAGCATCTTTGGCGAAGTGTGGAACATGCCGGTTTTGGTTGCACCCTAGGGGAATAAATCTTTGGGCACCTAGTGGTCATG

22 CATGACCACTAGGAGCATCTTTGGCGAAAAGACTCTGGATTCGGGGACCAGTTGCTGCTAGGGGAATAAATCTTTGGGCACCTAGTGGTCATG

22 CATGACCACTAGGAGCATCTTTGGCGAACGGCAGGTGTTGGGGTGGTCTGTGAATCCCTAGGGGAATAAATCTTTGGGCACCTAGTGGTCATG

21 CATGACCACTAGGAGCATCTTTGGCGAATGGCCGGCACGGCTTTCTAGTCCTCGGTACTAGGGGAATAAATCTTTGGGCACCTAGTGGTCATG

21 CATGACCACTAGGAGCATCTTTGGCGAGATCGGGGGAATCGGCGGCATTGGTGTCTCCTAGGGGAATAAATCTTTGGGCACCTAGTGGTCATG

21 CATGACCACTAGGAGCATCTTTGGCGAGAGCTTGAAAAAGGTGCGTTGTTGTCGCATCTAGGGGAATAAATCTTTGGGCACCTAGTGGTCATG

21 CATGACCACTAGGAGCATCTTTGGCGAGAAGACTCTGGATTCGGGGACCAATTGCTGCTAGGGGAATAAATCTTTGGGCACCTAGTGGTCATG

21 CATGACCACTAGGAGCATCTTTGGCGAACGGCAGGTGTTGCGGTGATCTGTGAATCCCTAGGGGAATAAATCTTTGGGCACCTAGTGGTCATG

21 CATGACCACTAGGAGCATCTTTGGCGAGGGTGGGCGCCGGCAGCGGTGTGAATGCGACTAGGGGAATAAATCTTTGGGCACCTAGTGGTCATG

21 CATGACCACTAGGAGCATCTTTGGCGAGAAGACTCTGGATTCGGGGACCAGTGTCTCCTAGGGGAATAAATCTTTGGGCACCTAGTGGTCATG

21 CATGACCACTAGGAGCATCTTTGGCGAAAGTCGGTAGAACAGGGTGGGGCGCTGTCCCTAGGGGAATAAATCTTTGGGCACCTAGTGGTCATG

21 CATGACCACTAGGAGCATCTTTGGCGAGATCGGGAGAATCGGCGGCATCGGTGTCTCCTAGGGGAATAAATCTTTGGGCACCTAGTGGTCATG

20 CATGACCACTAGGAGCATCTTTGGCGAATGGCCGGCACGGCCTTTTAGTCCTCGGTACTAGGGGAATAAATCTTTGGGCACCTAGTGGTCATG

20 CATGACCACTAGGAGCATCTTTGGCGAACGCCAGGTGTTGCGGTGGTCTGTGAATCCCTAGGGGAATAAATCTTTGGGCACCTAGTGGTCATG

20 CATGACCACTAGGAGCATCTTTGGCGAAAGTCGGTAGAACAGGGTGGGGTGCTGCCCCTAGGGGAATAAATCTTTGGGCACCTAGTGGTCATG

20 CATGACCACTAGGAGCATCTTTGGCGAACGGCACGTGTTGCGGTGGTCTGTGAATCCCTAGGGGAATAAATCTTTGGGCACCTAGTGGTCATG

20 CATGACCACTAGGAGCATCTTTGGCGAGCCTTGCTTGGGAGGTTGCTTCACCAGTTCCTAGGGGAATAAATCTTTGGGCACCTAGTGGTCATG

20 CATGACCACTAGGAGCATCTTTGGCGAACGGTAACGTAGTAACGTCGAAGTAGGCGCCTAGGGGAATAAATCTTTGGGCACCTAGTGGTCATG

20 CATGACCACTAGGAGCATCTTTGGCGACGATTGGTCGGAAGGTAAGTGTTCGGATGACTAGGGGAATAAATCTTTGGGCACCTAGTGGTCATG

19 CATGACCACTAGGAGCATCTTTGGCGAGAAGACTCTGGAATCGGGGACCAGTTGCTGCTAGGGGAATAAATCTTTGGGCACCTAGTGGTCATG

19 CATGACCACTAGGAGCATCTTTGGCGAATGGCTGGCACGGCCTTCTAGTCCTCGGTACTAGGGGAATAAATCTTTGGGCACCTAGTGGTCATG

19 CATGACCACTAGGAGCATCTTTGGCGAGATCGGGAGAGTCGGCGGCATTGGTGTCTCCTAGGGGAATAAATCTTTGGGCACCTAGTGGTCATG

19 CATGACCACTAGGAGCATCTTTGGCGACTAAGTGGCCAGCGCGTTAAATGGGGTAGACTAGGGGAATAAATCTTTGGGCACCTAGTGGTCATG

19 CATGACCACTAGGAGCATCTTTGGCGAGATCGGGAGAATCGGCGGTATTGGTGTCTCCTAGGGGAATAAATCTTTGGGCACCTAGTGGTCATG

18 CATGACCACTAGGAGCATCTTTGGCGAGAAGACTCCGGATTCGGGGACCAGTTGCTCCTAGGGGAATAAATCTTTGGGCACCTAGTGGTCATG

18 CATGACCACTAGGAGCATCTTTGGCGAAGCGTGTTGCGAGGTCCGCGAGTGTGGACCCTAGGGGAATAAATCTTTGGGCACCTAGTGGTCATG

18 CATGACCACTAGGAGCATCTTTGGCGAGAAGACTCTTGATTCGGGGACCAGTTGCTGCTAGGGGAATAAATCTTTGGGCACCTAGTGGTCATG

18 CATGACCACTAGGAGCATCTTTGGCGACGTGGGCTGGGAGGATGTGGTACTTCTCGACTAGGGGAATAAATCTTTGGGCACCTAGTGGTCATG

18 CATGACCACTAGGAGCATCTTTGGCGAACGGCAGGTGTTGCGGTGGTCTGTGACTCCCTAGGGGAATAAATCTTTGGGCACCTAGTGGTCATG

18 CATGACCACTAGGAGCATCTTTGGCGAGATTGTGAGAATCGGTGGCATTGGTGTCTCCTAGGGGAATAAATCTTTGGGCACCTAGTGGTCATG

18 CATGACCACTAGGAGCATCTTTGGCGAGATCTTAACGAAGTGAATAGCGGCAGGGGCCTAGGGGAATAAATCTTTGGGCACCTAGTGGTCATG

18 CATGACCACTAGGAGCATCTTTGGCGAAGATCCGATGGGCTTGTGACCCGCCTCGGCCTAGGGGAATAAATCTTTGGGCACCTAGTGGTCATG

18 CATGACCACTAGGAGCATCTTTGGCGAGAATGGAGCGACGGTGTGGTCTGAGTCCTACTAGGGGAATAAATCTTTGGGCACCTAGTGGTCATG

18 CATGACCACTAGGAGCATCTTTGGCGATACAGGTGATCGGTGCTCGGGTGCCTGGCCCTAGGGGAATAAATCTTTGGGCACCTAGTGGTCATG

18 CATGACCACTAGGAGCATCTTTGGCGAGCCTCGCTTGGGAGGTTGCTCCACTAGTTCCTAGGGGAATAAATCTTTGGGCACCTAGTGGTCATG

18 CATGACCACTAGGAGCATCTTTGGCGATGACGTCGTCGTAAATAGAGGCCTCGCGACCTAGGGGAATAAATCTTTGGGCACCTAGTGGTCATG

17 CATGACCACTAGGAGCATCTTTGGCGACAGTCGGTAGAACAGGGTGGGGTGCTGTCCCTAGGGGAATAAATCTTTGGGCACCTAGTGGTCATG

17 CATGACCACTAGGAGCATCTTTGGCGACAAGTAACGCATGCGGAACGCGACGTAGTGCTAGGGGAATAAATCTTTGGGCACCTAGTGGTCATG

17 CATGACCACTAGGAGCATCTTTGGCGAAAGTCGGTAGAACGGGGTGGGGTGCTGTCCCTAGGGGAATAAATCTTTGGGCACCTAGTGGTCATG

17 CATGACCACTAGGAGCATCTTTGGCGATCGCATAGCGTACCGGCAGTGGTCGGGGGCCTAGGGGAATAAATCTTTGGGCACCTAGTGGTCATG

17 CATGACCACTAGGAGCATCTTTGGCGAATAGGCCGTGGCTGCGGGTGGGTGCGCACCCTAGGGGAATAAATCTTTGGGCACCTAGTGGTCATG

17 CATGACCACTAGGAGCATCTTTGGCGAACGGCAGGTGTGGCGGTGGTCTGTGAATCCCTAGGGGAATAAATCTTTGGGCACCTAGTGGTCATG

17 CATGACCACTAGGAGCATCTTTGGCGAAAGTCGGTAGAACAGGGTGGGGTGCTGTCACTAGGGGAATAAATCTTTGGGCACCTAGTGGTCATG

17 CATGACCACTAGGAGCATCTTTGGCGAGATCGGGAGAATCGGCGGCATTGGTGCCTCCTAGGGGAATAAATCTTTGGGCACCTAGTGGTCATG

17 CATGACCACTAGGAGCATCTTTGGCGATCCGGGGGTCCGGGACGGGCGACGCTTGGGCTAGGGGAATAAATCTTTGGGCACCTAGTGGTCATG

17 CATGACCACTAGGAGCATCTTTGGCGACCGTAGGAGAACAAAAGTGGCCTTGCTAGCCTAGGGGAATAAATCTTTGGGCACCTAGTGGTCATG

16 CATGACCACTAGGAGCATCTTTGGCGAGTATACCGGCAAGGAAGGGAAACGTTGCGACTAGGGGAATAAATCTTTGGGCACCTAGTGGTCATG

16 CATGACCACTAGGAGCATCTTTGGCGAGAAGACTCTGGATTCGGGGAACAGTTGCTGCTAGGGGAATAAATCTTTGGGCACCTAGTGGTCATG

16 CATGACCACTAGGAGCATCTTTGGCGATCCGGGGGTCTAAGACGACGCAATTCTGGGCTAGGGGAATAAATCTTTGGGCACCTAGTGGTCATG

16 CATGACCACTAGGAGCATCTTTGGCGAAAGTCGGTAGAATAGGGTGGGGTGCTGTCCCTAGGGGAATAAATCTTTGGGCACCTAGTGGTCATG

16 CATGACCACTAGGAGCATCTTTGGCGAATGGCCGGTACGGCCTTCTAGTCCTCGGTACTAGGGGAATAAATCTTTGGGCACCTAGTGGTCATG

16 CATGACCACTAGGAGCATCTTTGGCGAGGAACGCGGCAGCAGTGGGACTAGAAGGTACTAGGGGAATAAATCTTTGGGCACCTAGTGGTCATG

16 CATGACCACTAGGAGCATCTTTGGCGATAGACAAGCGGTAGCCCGTAGTGGGTTGTTCTAGGGGAATAAATCTTTGGGCACCTAGTGGTCATG

16 CATGACCACTAGGAGCATCTTTGGCGAAGGTCGGTAGAACAGGGTGGGGTGCTGTCCCTAGGGGAATAAATCTTTGGGCACCTAGTGGTCATG

16 CATGACCACTAGGAGCATCTTTGGCGAGAAGACTCGGGATTCGGGGACCAGTTGCTGCTAGGGGAATAAATCTTTGGGCACCTAGTGGTCATG

16 CATGACCACTAGGAGCATCTTTGGCGAAAGCGCAGGGCATGCGACCTCCAATTGGTCCTAGGGGAATAAATCTTTGGGCACCTAGTGGTCATG

16 CATGACCACTAGGAGCATCTTTGGCGATCCGGGGGCCGTGGATAGGCGACGTTTAGGCTAGGGGAATAAATCTTTGGGCACCTAGTGGTCATG

15 CATGACCACTAGGAGCATCTTTGGCGAACGGCAGGTGTTGCGGTGGTCTGTGTACCCCTAGGGGAATAAATCTTTGGGCACCTAGTGGTCATG

15 CATGACCACTAGGAGCATCTTTGGCGAGATCGGGAGAATCGGTGGCATTGGTGTTTTCTAGGGGAATAAATCTTTGGGCACCTAGTGGTCATG

15 CATGACCACTAGGAGCATCTTTGGCGAGATCGGGAGAATCGGCGGCATTGGTGTCTACTAGGGGAATAAATCTTTGGGCACCTAGTGGTCATG

15 CATGACCACTAGGAGCATCTTTGGCGAGAAGACTCTGGATTCGGGGACCAGTTACTGCTAGGGGAATAAATCTTTGGGCACCTAGTGGTCATG

15 CATGACCACTAGGAGCATCTTTGGCGATGGGTGTTGACGGCCGCCGCTGCGGCTACACTAGGGGAATAAATCTTTGGGCACCTAGTGGTCATG

15 CATGACCACTAGGAGCATCTTTGGCGAGGTGCAGCGACCGGCAAGGGTTGAGGTGGGCTAGGGGAATAAATCTTTGGGCACCTAGTGGTCATG

15 CATGACCACTAGGAGCATCTTTGGCGAAAGTCGGTAGAGCAGGGTGGGGTGCTGTCCCTAGGGGAATAAATCTTTGGGCACCTAGTGGTCATG

15 CATGACCACTAGGAGCATCTTTGGCGATATCGGCGTTAGCTAACATCGTAGGATGTTCTAGGGGAATAAATCTTTGGGCACCTAGTGGTCATG

15 CATGACCACTAGGAGCATCTTTGGCGAGAAGACCTTGGATTCGGGGACCAGTTGCTGCTAGGGGAATAAATCTTTGGGCACCTAGTGGTCATG

15 CATGACCACTAGGAGCATCTTTGGCGAACCGCAGGTGTTGCGGTGGTCTGTGAATCCCTAGGGGAATAAATCTTTGGGCACCTAGTGGTCATG

15 CATGACCACTAGGAGCATCTTTGGCGAGATTGGGAGAATTGGTGGCATTGGTGTCTCCTAGGGGAATAAATCTTTGGGCACCTAGTGGTCATG

15 CATGACCACTAGGAGCATCTTTGGCGAACGGCAGGTCTTGCGGTGGTCTGTGAATCCCTAGGGGAATAAATCTTTGGGCACCTAGTGGTCATG

15 CATGACCACTAGGAGCATCTTTGGCGAGATCGGGAGATTCGGTGGCATTGGTGTCTCCTAGGGGAATAAATCTTTGGGCACCTAGTGGTCATG

15 CATGACCACTAGGAGCATCTTTGGCGAACGGCAGGTGTTGCGGTGCTCTGTGAATCCCTAGGGGAATAAATCTTTGGGCACCTAGTGGTCATG

15 CATGACCACTAGGAGCATCTTTGGCGAGGGAGGGCGCCGGTAGCGGTGTGAATGCGACTAGGGGAATAAATCTTTGGGCACCTAGTGGTCATG

15 CATGACCACTAGGAGCATCTTTGGCGAGAAGACTCTGGATTAGGGGACCAGTTGCTGCTAGGGGAATAAATCTTTGGGCACCTAGTGGTCATG

15 CATGACCACTAGGAGCATCTTTGGCGAGCGAAGGTAACGGTGTGCGTAAAGGAGCTCCTAGGGGAATAAATCTTTGGGCACCTAGTGGTCATG

15 CATGACCACTAGGAGCATCTTTGGCGAATGGCCGGCACGGTCTTCTAGTCCTCGGTACTAGGGGAATAAATCTTTGGGCACCTAGTGGTCATG

15 CATGACCACTAGGAGCATCTTTGGCGACAAGGGCATGTAATATACCGCGACATAGTGCTAGGGGAATAAATCTTTGGGCACCTAGTGGTCATG

15 CATGACCACTAGGAGCATCTTTGGCGATCAGGGGGCATGCCAAGGCGACTCGATGGGCTAGGGGAATAAATCTTTGGGCACCTAGTGGTCATG

15 CATGACCACTAGGAGCATCTTTGGCGAGTTGGGGGCACGACCTTGGTAGGTATAAAGCTAGGGGAATAAATCTTTGGGCACCTAGTGGTCATG

15 CATGACCACTAGGAGCATCTTTGGCGAAAGTCGGTGGAACAGGGTGGGGTGCTGTCCCTAGGGGAATAAATCTTTGGGCACCTAGTGGTCATG

15 CATGACCACTAGGAGCATCTTTGGCGACGGGTGGGCGCCCTAGGGTGTGAGTGTTGCCTAGGGGAATAAATCTTTGGGCACCTAGTGGTCATG

15 CATGACCACTAGGAGCATCTTTGGCGAGGGGTCGGTTGGAAGGACGTATTCCTGAGGCTAGGGGAATAAATCTTTGGGCACCTAGTGGTCATG

15 CATGACCACTAGGAGCATCTTTGGCGAAAGTCAGTAGAACAGGGTGGGGTGCTGTCCCTAGGGGAATAAATCTTTGGGCACCTAGTGGTCATG

15 CATGACCACTAGGAGCATCTTTGGCGAGATCGTGAGAATTGGTGGCATTGGTGTCTCCTAGGGGAATAAATCTTTGGGCACCTAGTGGTCATG

15 CATGACCACTAGGAGCATCTTTGGCGAGATCGGGAGAATCGGTAACATTGGTGTCTCCTAGGGGAATAAATCTTTGGGCACCTAGTGGTCATG

15 CATGACCACTAGGAGCATCTTTGGCGATCCGGGGGCTCCGGAGGGGCGACGCGTGGGCTAGGGGAATAAATCTTTGGGCACCTAGTGGTCATG

15 CATGACCACTAGGAGCATCTTTGGCGAGCCACGGGCGTAGACCGCTGTTCTGGGTAACTAGGGGAATAAATCTTTGGGCACCTAGTGGTCATG

14 CATGACCACTAGGAGCATCTTTGGCGAATGGCCGGCACGGTCTTCTAGTCCTTGGTACTAGGGGAATAAATCTTTGGGCACCTAGTGGTCATG

14 CATGACCACTAGGAGCATCTTTGGCGACGTCTGTCCTTGGTAGTTACGGCCAATCTCCTAGGGGAATAAATCTTTGGGCACCTAGTGGTCATG

14 CATGACCACTAGGAGCATCTTTGGCGACTAAGTGGACAACGCGTTAAATGGGGTAGACTAGGGGAATAAATCTTTGGGCACCTAGTGGTCATG

14 CATGACCACTAGGAGCATCTTTGGCGACTAAGTGGCCAACGCGTTAAGTGGGGTAGACTAGGGGAATAAATCTTTGGGCACCTAGTGGTCATG

14 CATGACCACTAGGAGCATCTTTGGCGAAAGCCGGTAGAACAGGGTGGGGTGCTGTCCCTAGGGGAATAAATCTTTGGGCACCTAGTGGTCATG

14 CATGACCACTAGGAGCATCTTTGGCGATGGGGCCGTGCTATCTGCACACTTCGCGGGCTAGGGGAATAAATCTTTGGGCACCTAGTGGTCATG

14 CATGACCACTAGGAGCATCTTTGGCGAACGGCAGGTGTTGCCGTGGTCTGTGAATCCCTAGGGGAATAAATCTTTGGGCACCTAGTGGTCATG

14 CATGACCACTAGGAGCATCTTTGGCGATTTGGGAGTCGCATTATGCAGGCAGTGAGACTAGGGGAATAAATCTTTGGGCACCTAGTGGTCATG

14 CATGACCACTAGGAGCATCTTTGGCGATGGGTGCAGACGAGTGCGCATGCAATTACACTAGGGGAATAAATCTTTGGGCACCTAGTGGTCATG

14 CATGACCACTAGGAGCATCTTTGGCGAGAAGACTCTGGATACGGGGACCAGTTGCTGCTAGGGGAATAAATCTTTGGGCACCTAGTGGTCATG

14 CATGACCACTAGGAGCATCTTTGGCGAACGGCAGGTGTTGCGGTGGTCTGTCAATCCCTAGGGGAATAAATCTTTGGGCACCTAGTGGTCATG

14 CATGACCACTAGGAGCATCTTTGGCGACGTTATGAGTTGGGAAGCCTGGCGGCCAGACTAGGGGAATAAATCTTTGGGCACCTAGTGGTCATG

14 CATGACCACTAGGAGCATCTTTGGCGAGATCGTGAGAATCGGTGGCATTGGTGTTTCCTAGGGGAATAAATCTTTGGGCACCTAGTGGTCATG

14 CATGACCACTAGGAGCATCTTTGGCGAGACGACTCTGGATTCGGGGACCAGTTGCTGCTAGGGGAATAAATCTTTGGGCACCTAGTGGTCATG

14 CATGACCACTAGGAGCATCTTTGGCGAGATCGAGAGAATCGGCGGCATTGGTGTCTCCTAGGGGAATAAATCTTTGGGCACCTAGTGGTCATG

14 CATGACCACTAGGAGCATCTTTGGCGACCCACGGTAAAGCAGTGTACGTGTGGTGGACTAGGGGAATAAATCTTTGGGCACCTAGTGGTCATG

14 CATGACCACTAGGAGCATCTTTGGCGAGATCGTGAGAATCGGTGGCATTGGTGTCTTCTAGGGGAATAAATCTTTGGGCACCTAGTGGTCATG

14 CATGACCACTAGGAGCATCTTTGGCGACGGGCGGGCGCCCTAGGGTGTGAGTGTTGCCTAGGGGAATAAATCTTTGGGCACCTAGTGGTCATG

14 CATGACCACTAGGAGCATCTTTGGCGAGCCTCGCTTGGGAGGTTGCTCTACCAGTTCCTAGGGGAATAAATCTTTGGGCACCTAGTGGTCATG

13 CATGACCACTAGGAGCATCTTTGGCGAACGTGCATGATATGCCGCCTGGAGCGTAGGCTAGGGGAATAAATCTTTGGGCACCTAGTGGTCATG

13 CATGACCACTAGGAGCATCTTTGGCGATCCGGGGGCCTGAGTAGGCGACGCCCAGGGCTAGGGGAATAAATCTTTGGGCACCTAGTGGTCATG

13 CATGACCACTAGGAGCATCTTTGGCGAGCTTCGCTTGGGAGGTTGCTCCACCAGTTCCTAGGGGAATAAATCTTTGGGCACCTAGTGGTCATG

13 CATGACCACTAGGAGCATCTTTGGCGAATGGCCGGCACGGCCTTCTAGTCTTTGGTACTAGGGGAATAAATCTTTGGGCACCTAGTGGTCATG

13 CATGACCACTAGGAGCATCTTTGGCGACGGTGTGGGTGCAGGGCCAGGCGGTTGATCCTAGGGGAATAAATCTTTGGGCACCTAGTGGTCATG

13 CATGACCACTAGGAGCATCTTTGGCGAACGGCAGGTGTTGCGGTGGTGTGTGAATCCCTAGGGGAATAAATCTTTGGGCACCTAGTGGTCATG

13 CATGACCACTAGGAGCATCTTTGGCGACGGTGTGGGGACCGTAGGTTCGGCGGGTACCTAGGGGAATAAATCTTTGGGCACCTAGTGGTCATG

13 CATGACCACTAGGAGCATCTTTGGCGAGGGGGTCTGTCGGGCCGGAGTGCTCCAACGCTAGGGGAATAAATCTTTGGGCACCTAGTGGTCATG

13 CATGACCACTAGGAGCATCTTTGGCGAACGGCCGGTGTTGCGGTGGTCTGTGAATCCCTAGGGGAATAAATCTTTGGGCACCTAGTGGTCATG

13 CATGACCACTAGGAGCATCTTTGGCGACGGTGTGGGGAAGTTATATCGGCGGATGCTCTAGGGGAATAAATCTTTGGGCACCTAGTGGTCATG

13 CATGACCACTAGGAGCATCTTTGGCGAGATCTGGAGAATCGGCGGCATTGGTGTCTCCTAGGGGAATAAATCTTTGGGCACCTAGTGGTCATG

13 CATGACCACTAGGAGCATCTTTGGCGAGGAGTGGCTGAGGGCGGTGGTTAGGTCGCGCTAGGGGAATAAATCTTTGGGCACCTAGTGGTCATG

13 CATGACCACTAGGAGCATCTTTGGCGAAGGGGGTCTCGGGGTGATAACGATGTAGCTCTAGGGGAATAAATCTTTGGGCACCTAGTGGTCATG

13 CATGACCACTAGGAGCATCTTTGGCGAGGTACGAAGCCGAGGCTGTGGCTGAGTTTACTAGGGGAATAAATCTTTGGGCACCTAGTGGTCATG

13 CATGACCACTAGGAGCATCTTTGGCGATCGCATAGCGGAACTACGAAGTGTCGGGCTCTAGGGGAATAAATCTTTGGGCACCTAGTGGTCATG

13 CATGACCACTAGGAGCATCTTTGGCGATCCGGGGGCCCTCGCGGGCGACGCTATGGGCTAGGGGAATAAATCTTTGGGCACCTAGTGGTCATG

13 CATGACCACTAGGAGCATCTTTGGCGAAAGTCGGTAGGACAGGGTGGGGTGCTGTCCCTAGGGGAATAAATCTTTGGGCACCTAGTGGTCATG

13 CATGACCACTAGGAGCATCTTTGGCGATACATGTGTATTCCGAAGTGGAACGAGTCCCTAGGGGAATAAATCTTTGGGCACCTAGTGGTCATG

13 CATGACCACTAGGAGCATCTTTGGCGATCCGGGGGTCCATGCGGACGACGTCTAGGGCTAGGGGAATAAATCTTTGGGCACCTAGTGGTCATG

13 CATGACCACTAGGAGCATCTTTGGCGAGAAGACTCTGGATTCGGGGACCAGTTGCAGCTAGGGGAATAAATCTTTGGGCACCTAGTGGTCATG

12 CATGACCACTAGGAGCATCTTTGGCGACAAGGGAGTTGAAGTGACCGCGACGTAGTGCTAGGGGAATAAATCTTTGGGCACCTAGTGGTCATG

12 CATGACCACTAGGAGCATCTTTGGCGATTTGGGAGTCGCGGTATGCAGGCAGTGAGACTAGGGGAATAAATCTTTGGGCACCTAGTGGTCATG

12 CATGACCACTAGGAGCATCTTTGGCGAGAAGACTCTGTATTCGGGGACCAGTTGCTCCTAGGGGAATAAATCTTTGGGCACCTAGTGGTCATG

12 CATGACCACTAGGAGCATCTTTGGCGACTGTGGGTTGGTAGGGGTGTACCTGTGGCGCTAGGGGAATAAATCTTTGGGCACCTAGTGGTCATG

12 CATGACCACTAGGAGCATCTTTGGCGAACGGCAGGTGTTCCGGTGGTCTGTGAATCCCTAGGGGAATAAATCTTTGGGCACCTAGTGGTCATG

12 CATGACCACTAGGAGCATCTTTGGCGAGGTCGGGAGAACCGGTGGCATTGGTGTCTCCTAGGGGAATAAATCTTTGGGCACCTAGTGGTCATG

12 CATGACCACTAGGAGCATCTTTGGCGAATGGCCGACACGGCCTTCTAGTCCTCGGTACTAGGGGAATAAATCTTTGGGCACCTAGTGGTCATG

12 CATGACCACTAGGAGCATCTTTGGCGATGGGGAGCAAGAGGACTGAGTAGGAATAAGCTAGGGGAATAAATCTTTGGGCACCTAGTGGTCATG

12 CATGACCACTAGGAGCATCTTTGGCGATTGGGGGGCGACGGCCGGTAGTGGGTGAGACTAGGGGAATAAATCTTTGGGCACCTAGTGGTCATG

12 CATGACCACTAGGAGCATCTTTGGCGAGTGGTGGGTGAGCAACGTGAGGGATGTCGACTAGGGGAATAAATCTTTGGGCACCTAGTGGTCATG

12 CATGACCACTAGGAGCATCTTTGGCGAGAAGACTCTGGATTCGGGGACCTGTTGCTGCTAGGGGAATAAATCTTTGGGCACCTAGTGGTCATG

12 CATGACCACTAGGAGCATCTTTGGCGAGAAGACTCTGGATTCGGGCACCAGTTGCTGCTAGGGGAATAAATCTTTGGGCACCTAGTGGTCATG

12 CATGACCACTAGGAGCATCTTTGGCGAATGGCCGGCATGGCCTTCTAGTCCTCGGTACTAGGGGAATAAATCTTTGGGCACCTAGTGGTCATG

12 CATGACCACTAGGAGCATCTTTGGCGAAGTACAACGGCAGGCATTGTGTCTCGGTAGCTAGGGGAATAAATCTTTGGGCACCTAGTGGTCATG

12 CATGACCACTAGGAGCATCTTTGGCGACGATGGTGGGCTAGAGACCTCCGAAGTGGACTAGGGGAATAAATCTTTGGGCACCTAGTGGTCATG

12 CATGACCACTAGGAGCATCTTTGGCGACTGAGTGGGCTGGGAGGAAGTATTTCGGAGCTAGGGGAATAAATCTTTGGGCACCTAGTGGTCATG

12 CATGACCACTAGGAGCATCTTTGGCGACGGCTGGCTGGCAGTGCGTACTAGTAGTCACTAGGGGAATAAATCTTTGGGCACCTAGTGGTCATG

12 CATGACCACTAGGAGCATCTTTGGCGAGCAAGCGCGGTCCCTCGGACCTCACAGTTGCTAGGGGAATAAATCTTTGGGCACCTAGTGGTCATG

12 CATGACCACTAGGAGCATCTTTGGCGAGCCTCGCTTGGGAGGTTGCTCCATCAGTTCCTAGGGGAATAAATCTTTGGGCACCTAGTGGTCATG

11 CATGACCACTAGGAGCATCTTTGGCGATGGAGGGCGCCGGCAGCGGTGTGAATGCGACTAGGGGAATAAATCTTTGGGCACCTAGTGGTCATG

11 CATGACCACTAGGAGCATCTTTGGCGAGGTCCGAGCGATGCGTGCCGTGGTCGAGCTCTAGGGGAATAAATCTTTGGGCACCTAGTGGTCATG

11 CATGACCACTAGGAGCATCTTTGGCGATCCTTGCTTGGGAGGTTGCTCCACCAGTTCCTAGGGGAATAAATCTTTGGGCACCTAGTGGTCATG

11 CATGACCACTAGGAGCATCTTTGGCGAACGGGAGGTGTTGCGGTGGTCTGTGAATCCCTAGGGGAATAAATCTTTGGGCACCTAGTGGTCATG

11 CATGACCACTAGGAGCATCTTTGGCGAGATCGGTAGAATCGGCGGCATTGGTGTCTCCTAGGGGAATAAATCTTTGGGCACCTAGTGGTCATG

11 CATGACCACTAGGAGCATCTTTGGCGATGGGGCCGTGCTATTTGCACACTCCGCGGGCTAGGGGAATAAATCTTTGGGCACCTAGTGGTCATG

11 CATGACCACTAGGAGCATCTTTGGCGACGGCGTGGGGCCATCTGTGCGGCGGATCTCCTAGGGGAATAAATCTTTGGGCACCTAGTGGTCATG

11 CATGACCACTAGGAGCATCTTTGGCGATCAGCATAGCATATGCGCCTGGAGCGTAGGCTAGGGGAATAAATCTTTGGGCACCTAGTGGTCATG

11 CATGACCACTAGGAGCATCTTTGGCGATCCGGGGGCACCTAAGTGCGACGCTGTGGGCTAGGGGAATAAATCTTTGGGCACCTAGTGGTCATG

11 CATGACCACTAGGAGCATCTTTGGCGACGGCGTGGGGCCATCTGTGCGGCGGATCCTCTAGGGGAATAAATCTTTGGGCACCTAGTGGTCATG

11 CATGACCACTAGGAGCATCTTTGGCGAGATCGGGAGAATCAATGGCATTGGTGTCTCCTAGGGGAATAAATCTTTGGGCACCTAGTGGTCATG

11 CATGACCACTAGGAGCATCTTTGGCGAGAAGACTTCGGATTCGGGGACCAGTTGCTGCTAGGGGAATAAATCTTTGGGCACCTAGTGGTCATG

11 CATGACCACTAGGAGCATCTTTGGCGAGATGACTCTGGATTCGGGGACCAGTTGCTGCTAGGGGAATAAATCTTTGGGCACCTAGTGGTCATG

11 CATGACCACTAGGAGCATCTTTGGCGAGATCGGGAGGATCGGTGGCATTGGTGTCTTCTAGGGGAATAAATCTTTGGGCACCTAGTGGTCATG

11 CATGACCACTAGGAGCATCTTTGGCGAGAAGACTCTGGATTCGCGGACCAGTTGCTGCTAGGGGAATAAATCTTTGGGCACCTAGTGGTCATG

11 CATGACCACTAGGAGCATCTTTGGCGAGCGTAGCGTGGGCACGGTGCGGGTTTCCTCCTAGGGGAATAAATCTTTGGGCACCTAGTGGTCATG

11 CATGACCACTAGGAGCATCTTTGGCGAGATCGGGAGGATCGGCGGCATTGGTGTCTCCTAGGGGAATAAATCTTTGGGCACCTAGTGGTCATG

11 CATGACCACTAGGAGCATCTTTGGCGAGGGGTGTAGGACTTCAAGTGGATCTCATAGCTAGGGGAATAAATCTTTGGGCACCTAGTGGTCATG

11 CATGACCACTAGGAGCATCTTTGGCGAATGGCAGGTGTTGCGGTGGTTTGTGAATCCCTAGGGGAATAAATCTTTGGGCACCTAGTGGTCATG

11 CATGACCACTAGGAGCATCTTTGGCGACGGGCGGGCGCCCAAGGGTGTGAGTGTTGCCTAGGGGAATAAATCTTTGGGCACCTAGTGGTCATG

11 CATGACCACTAGGAGCATCTTTGGCGAGATCGAGAGAATCGGTGGCATTGGTGTCTTCTAGGGGAATAAATCTTTGGGCACCTAGTGGTCATG

11 CATGACCACTAGGAGCATCTTTGGCGAGATCGGTAGAATCGGTGGCATTGGTGTCTTCTAGGGGAATAAATCTTTGGGCACCTAGTGGTCATG

11 CATGACCACTAGGAGCATCTTTGGCGATCCGGGGGCACCAATGTGCGACGCTGTGGGCTAGGGGAATAAATCTTTGGGCACCTAGTGGTCATG

10 CATGACCACTAGGAGCATCTTTGGCGAGAAGACTCTGGATTCCGGGACCAGTTGCTGCTAGGGGAATAAATCTTTGGGCACCTAGTGGTCATG

10 CATGACCACTAGGAGCATCTTTGGCGACTCGTGTAGCGACCTGGGTGCGGCCGGTGACTAGGGGAATAAATCTTTGGGCACCTAGTGGTCATG

10 CATGACCACTAGGAGCATCTTTGGCGAGGGAGGGCGCTGGCAGCGGTGTGAATGCGCCTAGGGGAATAAATCTTTGGGCACCTAGTGGTCATG

10 CATGACCACTAGGAGCATCTTTGGCGATCGAGTATCGGTGGTCTGATTCAGATGCGCCTAGGGGAATAAATCTTTGGGCACCTAGTGGTCATG

10 CATGACCACTAGGAGCATCTTTGGCGATCCGGGGGCACTTATGTGCGACGCTGTGGGCTAGGGGAATAAATCTTTGGGCACCTAGTGGTCATG

10 CATGACCACTAGGAGCATCTTTGGCGAACGGCAGGTGTTGCGGTGGTCTGGGAATCCCTAGGGGAATAAATCTTTGGGCACCTAGTGGTCATG

10 CATGACCACTAGGAGCATCTTTGGCGAACCTGTACGGTGATGAGGTCGGTATCAGCCCTAGGGGAATAAATCTTTGGGCACCTAGTGGTCATG

10 CATGACCACTAGGAGCATCTTTGGCGATTAGCGTCTTGCGAGCGGGTGGGTTCGCTCCTAGGGGAATAAATCTTTGGGCACCTAGTGGTCATG

10 CATGACCACTAGGAGCATCTTTGGCGAGAAGACTCTGGTTTCGGGGACCAGTTGCTGCTAGGGGAATAAATCTTTGGGCACCTAGTGGTCATG

10 CATGACCACTAGGAGCATCTTTGGCGAAAGTCGGTAGAACAGGGTGTGGTGCTGTCCCTAGGGGAATAAATCTTTGGGCACCTAGTGGTCATG

10 CATGACCACTAGGAGCATCTTTGGCGACTGAGGGACGGCACGTCATGTCGAAGTGGACTAGGGGAATAAATCTTTGGGCACCTAGTGGTCATG

10 CATGACCACTAGGAGCATCTTTGGCGAGAAGACTCTGGATTCGGGGACCAGTTGCGGCTAGGGGAATAAATCTTTGGGCACCTAGTGGTCATG

10 CATGACCACTAGGAGCATCTTTGGCGAGAAGTCTCTGGATTCGGGGACCAGTTGCTGCTAGGGGAATAAATCTTTGGGCACCTAGTGGTCATG

10 CATGACCACTAGGAGCATCTTTGGCGATTAGCGTCTTGCGAGCGGGTGGTTCGCTCCCTAGGGGAATAAATCTTTGGGCACCTAGTGGTCATG

10 CATGACCACTAGGAGCATCTTTGGCGAGCCTCGCTTGGGAGGTTGCTCCACCAGTTTCTAGGGGAATAAATCTTTGGGCACCTAGTGGTCATG

10 CATGACCACTAGGAGCATCTTTGGCGAGGGGATCTGTCGGGCCGGAGTGCTCCAACGCTAGGGGAATAAATCTTTGGGCACCTAGTGGTCATG

10 CATGACCACTAGGAGCATCTTTGGCGAGAAGACTCTGGATTCGGGGACTAGTTGCTCCTAGGGGAATAAATCTTTGGGCACCTAGTGGTCATG

10 CATGACCACTAGGAGCATCTTTGGCGATACCAGAGGTGAGCGAAGGTGTGCGAGCTACTAGGGGAATAAATCTTTGGGCACCTAGTGGTCATG

10 CATGACCACTAGGAGCATCTTTGGCGATAGCAGAAGGTGTGAATGAAAAGGGATCTACTAGGGGAATAAATCTTTGGGCACCTAGTGGTCATG

10 CATGACCACTAGGAGCATCTTTGGCGAGGGGTAGGGGGCCGCTCGCGCCTGTGTGCGCTAGGGGAATAAATCTTTGGGCACCTAGTGGTCATG

10 CATGACCACTAGGAGCATCTTTGGCGAAAGTCGGTAGAAAAGGGTGGGGTGCTGTCCCTAGGGGAATAAATCTTTGGGCACCTAGTGGTCATG

10 CATGACCACTAGGAGCATCTTTGGCGATCCGGGGGCCTGAAGGGCGACGCGCTCGGGCTAGGGGAATAAATCTTTGGGCACCTAGTGGTCATG

10 CATGACCACTAGGAGCATCTTTGGCGAGATCGGGAGAATTGGTGGCATCGGTGTCTCCTAGGGGAATAAATCTTTGGGCACCTAGTGGTCATG

10 CATGACCACTAGGAGCATCTTTGGCGAGAAGACTCTGGCTTCGGGGACCAGTTGCTGCTAGGGGAATAAATCTTTGGGCACCTAGTGGTCATG

10 CATGACCACTAGGAGCATCTTTGGCGATTAGGCCGCCAGGGCGTACGAAGTGTGGTTCTAGGGGAATAAATCTTTGGGCACCTAGTGGTCATG

10 CATGACCACTAGGAGCATCTTTGGCGAATGGCCGGCACGGCTTTCTAGTCCTTGGTACTAGGGGAATAAATCTTTGGGCACCTAGTGGTCATG

10 CATGACCACTAGGAGCATCTTTGGCGATCCGGGGGCCGCGGCCGGCGACGCAATGGGCTAGGGGAATAAATCTTTGGGCACCTAGTGGTCATG

10 CATGACCACTAGGAGCATCTTTGGCGAGATCGGGAGACTTGGTGGCATTGGTGTCTCCTAGGGGAATAAATCTTTGGGCACCTAGTGGTCATG

10 CATGACCACTAGGAGCATCTTTGGCGACAATGGGGCTGGTAGGAAAGTACTTATAGCCTAGGGGAATAAATCTTTGGGCACCTAGTGGTCATG

10 CATGACCACTAGGAGCATCTTTGGCGAGCTCCGAGCAAGCGAACCGCGAAAAGTCGGCTAGGGGAATAAATCTTTGGGCACCTAGTGGTCATG

9 CATGACCACTAGGAGCATCTTTGGCGATGGGGAGCGAGAGGACTGAGTAGGAATAAGCTAGGGGAATAAATCTTTGGGCACCTAGTGGTCATG

9 CATGACCACTAGGAGCATCTTTGGCGAGATCGGGAGAATTGGTGGCATTGGTGTCTTCTAGGGGAATAAATCTTTGGGCACCTAGTGGTCATG

9 CATGACCACTAGGAGCATCTTTGGCGAGATTGGGAGAATCGGTGGCATCGGTGTCTCCTAGGGGAATAAATCTTTGGGCACCTAGTGGTCATG

9 CATGACCACTAGGAGCATCTTTGGCGAGAAGACTCTGGATTCGGCGACCAGTTGCTGCTAGGGGAATAAATCTTTGGGCACCTAGTGGTCATG

9 CATGACCACTAGGAGCATCTTTGGCGAAAGTCTGTAGAACAGGGTGGGGTGCTGTCCCTAGGGGAATAAATCTTTGGGCACCTAGTGGTCATG

9 CATGACCACTAGGAGCATCTTTGGCGAAACGGATCGGGAGGCGTGGCGGATTGTGTGCTAGGGGAATAAATCTTTGGGCACCTAGTGGTCATG

9 CATGACCACTAGGAGCATCTTTGGCGAATGGCCGGCACGGCCTTCTAGTTCTCGGTACTAGGGGAATAAATCTTTGGGCACCTAGTGGTCATG

9 CATGACCACTAGGAGCATCTTTGGCGAACGGCAGGTGTTGCGCTGGTCTGTGAATCCCTAGGGGAATAAATCTTTGGGCACCTAGTGGTCATG

9 CATGACCACTAGGAGCATCTTTGGCGAACAGGCCTTGGCTGCGGGTGGGTGCGCACCCTAGGGGAATAAATCTTTGGGCACCTAGTGGTCATG

9 CATGACCACTAGGAGCATCTTTGGCGAGGGAGGGCGCCGACAGCGGTGTGAATGCGACTAGGGGAATAAATCTTTGGGCACCTAGTGGTCATG

9 CATGACCACTAGGAGCATCTTTGGCGAGCCTTGCTTGGGAGGTTGCTACACCAGTTCCTAGGGGAATAAATCTTTGGGCACCTAGTGGTCATG

9 CATGACCACTAGGAGCATCTTTGGCGACAACGTGGGCACAGCAAAGTGGAAGTGGAGCTAGGGGAATAAATCTTTGGGCACCTAGTGGTCATG

9 CATGACCACTAGGAGCATCTTTGGCGACGAAGTGGGCTCTTGGCGAGTGGCGACCGCCTAGGGGAATAAATCTTTGGGCACCTAGTGGTCATG

9 CATGACCACTAGGAGCATCTTTGGCGAGAAGACTCTGGATTTGGGGACCAGTTGCTCCTAGGGGAATAAATCTTTGGGCACCTAGTGGTCATG

9 CATGACCACTAGGAGCATCTTTGGCGACAGTGGGCGGACCGGGACCCAAGGGGCGGACTAGGGGAATAAATCTTTGGGCACCTAGTGGTCATG

9 CATGACCACTAGGAGCATCTTTGGCGAGATAGGGAGAATCGGTGGCATTTGTGTCTCCTAGGGGAATAAATCTTTGGGCACCTAGTGGTCATG

9 CATGACCACTAGGAGCATCTTTGGCGAAAGTCGGTAGAACAGGGGGGGGTGCTGTCCCTAGGGGAATAAATCTTTGGGCACCTAGTGGTCATG

9 CATGACCACTAGGAGCATCTTTGGCGAGGGAGGGCGCCGGCAGCGGTGTGTATGCGACTAGGGGAATAAATCTTTGGGCACCTAGTGGTCATG

9 CATGACCACTAGGAGCATCTTTGGCGATCGCATAGCGAAGGATAAGATGTCCCGGGCCTAGGGGAATAAATCTTTGGGCACCTAGTGGTCATG

9 CATGACCACTAGGAGCATCTTTGGCGATTGGTGCTGACGGCCGCCGCTGCGGCTACACTAGGGGAATAAATCTTTGGGCACCTAGTGGTCATG

9 CATGACCACTAGGAGCATCTTTGGCGAGATCGTGAGAATCGGTGGCATCGGTGTCTCCTAGGGGAATAAATCTTTGGGCACCTAGTGGTCATG

9 CATGACCACTAGGAGCATCTTTGGCGAGTAGACTCTGGATTCGGGGACCAGTTGCTGCTAGGGGAATAAATCTTTGGGCACCTAGTGGTCATG

9 CATGACCACTAGGAGCATCTTTGGCGAAAGTCGGTAGAACAGGGTGGGGTGATGTCCCTAGGGGAATAAATCTTTGGGCACCTAGTGGTCATG

9 CATGACCACTAGGAGCATCTTTGGCGAGCCTTGCTTGGGAGGTTGCTCCATCAGTTCCTAGGGGAATAAATCTTTGGGCACCTAGTGGTCATG

9 CATGACCACTAGGAGCATCTTTGGCGACCTGGTGGGACATGCTCAAACCAGTGTGGACTAGGGGAATAAATCTTTGGGCACCTAGTGGTCATG

9 CATGACCACTAGGAGCATCTTTGGCGATCCGGGGGCATCTATGTGCGACGCTGTGGGCTAGGGGAATAAATCTTTGGGCACCTAGTGGTCATG

8 CATGACCACTAGGAGCATCTTTGGCGATCCGGGGGCCAGCATTGGCGACGCTCAGGGCTAGGGGAATAAATCTTTGGGCACCTAGTGGTCATG

8 CATGACCACTAGGAGCATCTTTGGCGAGAAGACTCTGGATTCGGGGCCCAGTTGCTGCTAGGGGAATAAATCTTTGGGCACCTAGTGGTCATG

8 CATGACCACTAGGAGCATCTTTGGCGAGATCGTAAGAATCGGTGGCATTGGTGTCTCCTAGGGGAATAAATCTTTGGGCACCTAGTGGTCATG

8 CATGACCACTAGGAGCATCTTTGGCGAGATTGGGAGAATCGGTGGTATTGGTGTCTCCTAGGGGAATAAATCTTTGGGCACCTAGTGGTCATG

8 CATGACCACTAGGAGCATCTTTGGCGAGCCTCGCTTGGGAGGTTGCTACACCAGTTCCTAGGGGAATAAATCTTTGGGCACCTAGTGGTCATG

8 CATGACCACTAGGAGCATCTTTGGCGAGAAGTGGGCAAGGGGCAAAGTGTGTCGAGCCTAGGGGAATAAATCTTTGGGCACCTAGTGGTCATG

8 CATGACCACTAGGAGCATCTTTGGCGAACGGCAGCTGTTGCGGTGGTCTGTGAATCCCTAGGGGAATAAATCTTTGGGCACCTAGTGGTCATG

8 CATGACCACTAGGAGCATCTTTGGCGATCGCACAGCGGACGACTATCAGGGAGGGGACTAGGGGAATAAATCTTTGGGCACCTAGTGGTCATG

8 CATGACCACTAGGAGCATCTTTGGCGACGATGATGATGGGCAGGCTGGGGAGTGTTGCTAGGGGAATAAATCTTTGGGCACCTAGTGGTCATG

8 CATGACCACTAGGAGCATCTTTGGCGATAGCGAAGGGTACCAGTGTGGGAGTCCGTCCTAGGGGAATAAATCTTTGGGCACCTAGTGGTCATG

8 CATGACCACTAGGAGCATCTTTGGCGAGCTCTACGCGTCCTTGAGTGCCGACGTACACTAGGGGAATAAATCTTTGGGCACCTAGTGGTCATG

8 CATGACCACTAGGAGCATCTTTGGCGAGGAGTGGCTAAGGGCGGTGGGTAGGTCGCGCTAGGGGAATAAATCTTTGGGCACCTAGTGGTCATG

8 CATGACCACTAGGAGCATCTTTGGCGAGGGAGGGCGCCGGTAGCGGTGTGAATGCGCCTAGGGGAATAAATCTTTGGGCACCTAGTGGTCATG

8 CATGACCACTAGGAGCATCTTTGGCGAGAAGACTTTGGATTCGGGGACTAGTTGCTGCTAGGGGAATAAATCTTTGGGCACCTAGTGGTCATG

8 CATGACCACTAGGAGCATCTTTGGCGATTCGGGGGCACCTATGTGCGACGCTGTGGGCTAGGGGAATAAATCTTTGGGCACCTAGTGGTCATG

8 CATGACCACTAGGAGCATCTTTGGCGATCCGGGGGCCGACCGCGGCGACGTCTAGGGCTAGGGGAATAAATCTTTGGGCACCTAGTGGTCATG

8 CATGACCACTAGGAGCATCTTTGGCGATAGCGAACATGCGACCAGAGTGGTTAAGTGCTAGGGGAATAAATCTTTGGGCACCTAGTGGTCATG

8 CATGACCACTAGGAGCATCTTTGGCGACCAGCTGAGGCGTCATGCAGCGGAGTGGAACTAGGGGAATAAATCTTTGGGCACCTAGTGGTCATG

8 CATGACCACTAGGAGCATCTTTGGCGATCGCATAGCGCACGACAGGTCTGGAGGGGACTAGGGGAATAAATCTTTGGGCACCTAGTGGTCATG

8 CATGACCACTAGGAGCATCTTTGGCGATCAGCATAGTATATGCGCCTGGAGCGTAGGCTAGGGGAATAAATCTTTGGGCACCTAGTGGTCATG

8 CATGACCACTAGGAGCATCTTTGGCGAACGGCAGGTGTTGCGGTGGTTTGTGAATCTCTAGGGGAATAAATCTTTGGGCACCTAGTGGTCATG

8 CATGACCACTAGGAGCATCTTTGGCGAAAGTCGGTAGAACAGGGTGAGGTGCTGTCCCTAGGGGAATAAATCTTTGGGCACCTAGTGGTCATG

8 CATGACCACTAGGAGCATCTTTGGCGAGCCTTGCTTGGGAGGTTGTTCCACCAGTTCCTAGGGGAATAAATCTTTGGGCACCTAGTGGTCATG

8 CATGACCACTAGGAGCATCTTTGGCGATGGGTGCTGACGGCCGCTGCTGCGGCTACACTAGGGGAATAAATCTTTGGGCACCTAGTGGTCATG

8 CATGACCACTAGGAGCATCTTTGGCGATCCGGGGGACGAGCTAGTCGACGCTTTGGGCTAGGGGAATAAATCTTTGGGCACCTAGTGGTCATG

8 CATGACCACTAGGAGCATCTTTGGCGAGATCGGGAGAATCGTCGGCATTGGTGTCTCCTAGGGGAATAAATCTTTGGGCACCTAGTGGTCATG

8 CATGACCACTAGGAGCATCTTTGGCGATCCGGGGGCCTCGCAAAGGCGACGCCAGGGCTAGGGGAATAAATCTTTGGGCACCTAGTGGTCATG

8 CATGACCACTAGGAGCATCTTTGGCGACTAAGTGGTCAACGCGTTAAATGGGGTAGACTAGGGGAATAAATCTTTGGGCACCTAGTGGTCATG

8 CATGACCACTAGGAGCATCTTTGGCGACGGCGTGGGGCTATTGATGCGGCGGCACCCCTAGGGGAATAAATCTTTGGGCACCTAGTGGTCATG

8 CATGACCACTAGGAGCATCTTTGGCGATCGCACGGCGGAGTTAGAGACTAACCGGGACTAGGGGAATAAATCTTTGGGCACCTAGTGGTCATG

8 CATGACCACTAGGAGCATCTTTGGCGACGGGATCGTGTCGGCGGATAGCGTACTTGGCTAGGGGAATAAATCTTTGGGCACCTAGTGGTCATG

8 CATGACCACTAGGAGCATCTTTGGCGAAGCGTGTTGCAAGGTCCGTGAGTGTGGACCCTAGGGGAATAAATCTTTGGGCACCTAGTGGTCATG

8 CATGACCACTAGGAGCATCTTTGGCGAGATCGGGAGAATCGGTGGCATCGGTGTCTTCTAGGGGAATAAATCTTTGGGCACCTAGTGGTCATG

8 CATGACCACTAGGAGCATCTTTGGCGAGGAGACGGGATGAGTCGGGACTAGAAGGTACTAGGGGAATAAATCTTTGGGCACCTAGTGGTCATG

8 CATGACCACTAGGAGCATCTTTGGCGAAAGTCGGTAGAACAGGGTGGTGTGCTGTCCCTAGGGGAATAAATCTTTGGGCACCTAGTGGTCATG

8 CATGACCACTAGGAGCATCTTTGGCGAGGGAGGGCGCTGGCAGCGGTGTGAATGCGACTAGGGGAATAAATCTTTGGGCACCTAGTGGTCATG

8 CATGACCACTAGGAGCATCTTTGGCGATAGCGTAGGACCGTCGGAATGAGGGTGGATCTAGGGGAATAAATCTTTGGGCACCTAGTGGTCATG

8 CATGACCACTAGGAGCATCTTTGGCGAAAGTGGGCGATCTGGGAGAGGTCAATCATGCTAGGGGAATAAATCTTTGGGCACCTAGTGGTCATG

8 CATGACCACTAGGAGCATCTTTGGCGACGACGAAGGTGCAATGACTGAGGATTCGCCCTAGGGGAATAAATCTTTGGGCACCTAGTGGTCATG

7 CATGACCACTAGGAGCATCTTTGGCGAAAGTCGGTCGAACAGGGTGGGGTGCTGTCCCTAGGGGAATAAATCTTTGGGCACCTAGTGGTCATG

7 CATGACCACTAGGAGCATCTTTGGCGAGCCTTGCTAGGGAGGTTGCTCCACCAGTTCCTAGGGGAATAAATCTTTGGGCACCTAGTGGTCATG

7 CATGACCACTAGGAGCATCTTTGGCGAGATCGGAGGAATCGGTGGCATTGGTGTCTCCTAGGGGAATAAATCTTTGGGCACCTAGTGGTCATG

7 CATGACCACTAGGAGCATCTTTGGCGAGCCTTGCTTGGGAGGTTGCTCCACCAGTTACTAGGGGAATAAATCTTTGGGCACCTAGTGGTCATG

7 CATGACCACTAGGAGCATCTTTGGCGAAAGTAGGTAGAACAGGGTGGGGTGCTGTCCCTAGGGGAATAAATCTTTGGGCACCTAGTGGTCATG

7 CATGACCACTAGGAGCATCTTTGGCGACGTAGTGGGCTGGCAGGGAGTACTTGTAGCCTAGGGGAATAAATCTTTGGGCACCTAGTGGTCATG

7 CATGACCACTAGGAGCATCTTTGGCGATCGCAGAGCGTACAGTGGCATCAGTGGGGCCTAGGGGAATAAATCTTTGGGCACCTAGTGGTCATG

7 CATGACCACTAGGAGCATCTTTGGCGAAAGTCGGTAGAACAGGTTGGGGTGCTGTCCCTAGGGGAATAAATCTTTGGGCACCTAGTGGTCATG

7 CATGACCACTAGGAGCATCTTTGGCGAAAGTCGGTAGAACAAGGTGGGGTGCTGTCCCTAGGGGAATAAATCTTTGGGCACCTAGTGGTCATG

7 CATGACCACTAGGAGCATCTTTGGCGAAGGGTGTAGGACTTTAAGTGGATCTCATAGCTAGGGGAATAAATCTTTGGGCACCTAGTGGTCATG

7 CATGACCACTAGGAGCATCTTTGGCGAGATCAGGAGAATTGGTGGCATTGGTGTCTCCTAGGGGAATAAATCTTTGGGCACCTAGTGGTCATG

7 CATGACCACTAGGAGCATCTTTGGCGAATGGGGGGCGACGGCCGGTAGTGGGTGAGACTAGGGGAATAAATCTTTGGGCACCTAGTGGTCATG

7 CATGACCACTAGGAGCATCTTTGGCGATCCGGGGGCGTTACGACGCGACGCTGTGGGCTAGGGGAATAAATCTTTGGGCACCTAGTGGTCATG

7 CATGACCACTAGGAGCATCTTTGGCGAGATCGTGAGGATCGGTGGCATTGGTGTCTCCTAGGGGAATAAATCTTTGGGCACCTAGTGGTCATG

7 CATGACCACTAGGAGCATCTTTGGCGAAAGTCGGAAGAACAGGGTGGGGTGCTGTCCCTAGGGGAATAAATCTTTGGGCACCTAGTGGTCATG

7 CATGACCACTAGGAGCATCTTTGGCGAGGGAGGGCGCCGACAGCGGTGTGAATGCGCCTAGGGGAATAAATCTTTGGGCACCTAGTGGTCATG

7 CATGACCACTAGGAGCATCTTTGGCGAGATCGTGAGAATCGGTGGCATAGGTGTCTCCTAGGGGAATAAATCTTTGGGCACCTAGTGGTCATG

7 CATGACCACTAGGAGCATCTTTGGCGAGATCGGGAGAAACGGTGGCATAGGTGTCTCCTAGGGGAATAAATCTTTGGGCACCTAGTGGTCATG

7 CATGACCACTAGGAGCATCTTTGGCGAAAGTCGGTAGAACAGGGCGGGGTGCTGTCCCTAGGGGAATAAATCTTTGGGCACCTAGTGGTCATG

7 CATGACCACTAGGAGCATCTTTGGCGAAGCGTGTTGCAAGGTCTGCGAGTGTGGACCCTAGGGGAATAAATCTTTGGGCACCTAGTGGTCATG

7 CATGACCACTAGGAGCATCTTTGGCGATGGGTGCTGACGGCCGCCGTTGCGGCTACACTAGGGGAATAAATCTTTGGGCACCTAGTGGTCATG

7 CATGACCACTAGGAGCATCTTTGGCGAATGGCAGGTGTTGCGGTGGTCTGTGGATCCCTAGGGGAATAAATCTTTGGGCACCTAGTGGTCATG

7 CATGACCACTAGGAGCATCTTTGGCGAGGGGTAGGGGGCCGCTCGCGCCTACGTGCGCTAGGGGAATAAATCTTTGGGCACCTAGTGGTCATG

7 CATGACCACTAGGAGCATCTTTGGCGAAAGTCGGTAGAACAGGGTGGGTTGCTGTCCCTAGGGGAATAAATCTTTGGGCACCTAGTGGTCATG

7 CATGACCACTAGGAGCATCTTTGGCGAGCCTTGCTTGGGAGGTTGCTCCACCAGTTTCTAGGGGAATAAATCTTTGGGCACCTAGTGGTCATG

7 CATGACCACTAGGAGCATCTTTGGCGAGCCTTGCTTGGGAGGTTGCTCCGCCAGTTCCTAGGGGAATAAATCTTTGGGCACCTAGTGGTCATG

7 CATGACCACTAGGAGCATCTTTGGCGACCCGAGAAGCGACCGAAGAGCAACCAGTGACTAGGGGAATAAATCTTTGGGCACCTAGTGGTCATG

7 CATGACCACTAGGAGCATCTTTGGCGAAAGTCGGTAGAACAGGGTGGAGTGCTGTCCCTAGGGGAATAAATCTTTGGGCACCTAGTGGTCATG

7 CATGACCACTAGGAGCATCTTTGGCGAAAGTCGGTAGAACAGGGTGGGGTGCTGTTTCTAGGGGAATAAATCTTTGGGCACCTAGTGGTCATG

7 CATGACCACTAGGAGCATCTTTGGCGAGATCGGGAGAATCGGCGTCATTGGTGTCTCCTAGGGGAATAAATCTTTGGGCACCTAGTGGTCATG

7 CATGACCACTAGGAGCATCTTTGGCGACGGTGTGGGGCATCTCTCGCGGCGGTTATTCTAGGGGAATAAATCTTTGGGCACCTAGTGGTCATG

7 CATGACCACTAGGAGCATCTTTGGCGAGTCTTGCTTGGGAGGTTGCTCCACCAGTTCCTAGGGGAATAAATCTTTGGGCACCTAGTGGTCATG

7 CATGACCACTAGGAGCATCTTTGGCGAGATTGGGAGAATCGGTGGCATTGGTGCCTCCTAGGGGAATAAATCTTTGGGCACCTAGTGGTCATG

7 CATGACCACTAGGAGCATCTTTGGCGAGCGGCAGGTGTTGCGGTGGTCTGTGAAACCCTAGGGGAATAAATCTTTGGGCACCTAGTGGTCATG

7 CATGACCACTAGGAGCATCTTTGGCGAGGCAAGGTGTGCGGCGGGCGGGTACCACGGCTAGGGGAATAAATCTTTGGGCACCTAGTGGTCATG

7 CATGACCACTAGGAGCATCTTTGGCGAGATCGGGAGAATCGGCGGCATTGGTGTCCCCTAGGGGAATAAATCTTTGGGCACCTAGTGGTCATG

7 CATGACCACTAGGAGCATCTTTGGCGAGGGAGGGCGCCGGCAGCGGTGTGAATGCGGCTAGGGGAATAAATCTTTGGGCACCTAGTGGTCATG

7 CATGACCACTAGGAGCATCTTTGGCGAACGTGCAATATATGCCGCCTGGAGCGTAGGCTAGGGGAATAAATCTTTGGGCACCTAGTGGTCATG

7 CATGACCACTAGGAGCATCTTTGGCGAAATTCGGTAGAACAGGGTGGGGTGCTGTCCCTAGGGGAATAAATCTTTGGGCACCTAGTGGTCATG

7 CATGACCACTAGGAGCATCTTTGGCGAATGGCCAGCACGGCCTTCTAGTCCTCGGTACTAGGGGAATAAATCTTTGGGCACCTAGTGGTCATG

7 CATGACCACTAGGAGCATCTTTGGCGAGATCAGGAGAATCGGTGGCATTGGTGTCTTCTAGGGGAATAAATCTTTGGGCACCTAGTGGTCATG

7 CATGACCACTAGGAGCATCTTTGGCGAGATCGTGAGAATCGGTGGCATTGGCGTCTCCTAGGGGAATAAATCTTTGGGCACCTAGTGGTCATG

7 CATGACCACTAGGAGCATCTTTGGCGACTAGGCGTCAGCACCGGGTGGGTTCGGTCCCTAGGGGAATAAATCTTTGGGCACCTAGTGGTCATG

7 CATGACCACTAGGAGCATCTTTGGCGAGAGAATCCGAGCGATAGGTGTGTGCTCCCACTAGGGGAATAAATCTTTGGGCACCTAGTGGTCATG

7 CATGACCACTAGGAGCATCTTTGGCGAGAAGACTCTGGATTCGGGGACCAGGTGCTGCTAGGGGAATAAATCTTTGGGCACCTAGTGGTCATG

7 CATGACCACTAGGAGCATCTTTGGCGAGCCCTGCTTGGGAGGTTGCTCCACCAGTTCCTAGGGGAATAAATCTTTGGGCACCTAGTGGTCATG

7 CATGACCACTAGGAGCATCTTTGGCGACAGCCGTAGCCGTAAGGTTGGTATAAGGTCCTAGGGGAATAAATCTTTGGGCACCTAGTGGTCATG

7 CATGACCACTAGGAGCATCTTTGGCGAGTGCGGGCAACACGCAGTCCTGGTGTGCGACTAGGGGAATAAATCTTTGGGCACCTAGTGGTCATG

7 CATGACCACTAGGAGCATCTTTGGCGATAGCGTCTTGCGAGCGGGTGGGTTCGCTCCCTAGGGGAATAAATCTTTGGGCACCTAGTGGTCATG

7 CATGACCACTAGGAGCATCTTTGGCGAAAGTCGGTAGAACATGGTGGGGTGCTGTCCCTAGGGGAATAAATCTTTGGGCACCTAGTGGTCATG

7 CATGACCACTAGGAGCATCTTTGGCGATATCGGGAGAATCTGTGGCATTGGTGTCTCCTAGGGGAATAAATCTTTGGGCACCTAGTGGTCATG

7 CATGACCACTAGGAGCATCTTTGGCGAGGGATCGGCTAGGTGTCGTGTAGGGTGAGACTAGGGGAATAAATCTTTGGGCACCTAGTGGTCATG

7 CATGACCACTAGGAGCATCTTTGGCGAGGGAGGGCGCCGGCAGCGGTGTGAATGCGTCTAGGGGAATAAATCTTTGGGCACCTAGTGGTCATG

7 CATGACCACTAGGAGCATCTTTGGCGAGATCGCGAGAATCGGTGGCATTGGTGTCTTCTAGGGGAATAAATCTTTGGGCACCTAGTGGTCATG

7 CATGACCACTAGGAGCATCTTTGGCGATGGGGAGCTAGAGGACTGAGTAGGAATAAGCTAGGGGAATAAATCTTTGGGCACCTAGTGGTCATG

7 CATGACCACTAGGAGCATCTTTGGCGAGATCGGGAGAATCGGCGGCATTGGCGTCTCCTAGGGGAATAAATCTTTGGGCACCTAGTGGTCATG

7 CATGACCACTAGGAGCATCTTTGGCGACGATGGTTGGGAGGGAAGTATTTCGGGCGACTAGGGGAATAAATCTTTGGGCACCTAGTGGTCATG

7 CATGACCACTAGGAGCATCTTTGGCGACGGAGTGGGGCTCATTGTGCGGCGGTAACCCTAGGGGAATAAATCTTTGGGCACCTAGTGGTCATG

7 CATGACCACTAGGAGCATCTTTGGCGAGATCGGGAGAATCGGTGGCATTGGTGCTTCCTAGGGGAATAAATCTTTGGGCACCTAGTGGTCATG

7 CATGACCACTAGGAGCATCTTTGGCGACGTCGTGGCTGGTAGAGTGTATTTGACAAGCTAGGGGAATAAATCTTTGGGCACCTAGTGGTCATG

7 CATGACCACTAGGAGCATCTTTGGCGAAAGTCGGTAGAACAGAGTGGGGTGCTGTCCCTAGGGGAATAAATCTTTGGGCACCTAGTGGTCATG

7 CATGACCACTAGGAGCATCTTTGGCGAGTGGGGGGCGACGGCTGGTAGTGGGTGAGACTAGGGGAATAAATCTTTGGGCACCTAGTGGTCATG

7 CATGACCACTAGGAGCATCTTTGGCGACTAAGTGGGCAACGCGTTAAATGGGGTAGACTAGGGGAATAAATCTTTGGGCACCTAGTGGTCATG

6 CATGACCACTAGGAGCATCTTTGGCGAGATCGGGAGAGTCGGTGGCATTGGTGTCTACTAGGGGAATAAATCTTTGGGCACCTAGTGGTCATG

6 CATGACCACTAGGAGCATCTTTGGCGAGATCGGGAGGATCGGTGGTATTGGTGTCTCCTAGGGGAATAAATCTTTGGGCACCTAGTGGTCATG

6 CATGACCACTAGGAGCATCTTTGGCGAGATCGGGAGAATCGGTGGCATAGGTGTCTTCTAGGGGAATAAATCTTTGGGCACCTAGTGGTCATG

6 CATGACCACTAGGAGCATCTTTGGCGATCCTGTAGGAAGAGTAGCGGCTTCGTGAGACTAGGGGAATAAATCTTTGGGCACCTAGTGGTCATG

6 CATGACCACTAGGAGCATCTTTGGCGACGAAGGTCTTTAGTGGGCCCGAGGGCCGACCTAGGGGAATAAATCTTTGGGCACCTAGTGGTCATG

6 CATGACCACTAGGAGCATCTTTGGCGAATGGCCGGCACGGCCTTCTAGTCCTCGGTGCTAGGGGAATAAATCTTTGGGCACCTAGTGGTCATG

6 CATGACCACTAGGAGCATCTTTGGCGAGATCGGGAGAATTGGTGGCATTGGTGCCTCCTAGGGGAATAAATCTTTGGGCACCTAGTGGTCATG

6 CATGACCACTAGGAGCATCTTTGGCGAGATTGGGAGAATCGGTGGCATTGGTGTCTGCTAGGGGAATAAATCTTTGGGCACCTAGTGGTCATG

6 CATGACCACTAGGAGCATCTTTGGCGATATCGGGAGAAACGGTGGCATTGGTGTCTCCTAGGGGAATAAATCTTTGGGCACCTAGTGGTCATG

6 CATGACCACTAGGAGCATCTTTGGCGAGAAGACTCAGGATTCGGGGACCAGTTGCTCCTAGGGGAATAAATCTTTGGGCACCTAGTGGTCATG

6 CATGACCACTAGGAGCATCTTTGGCGAGATCGGGAGAATTGGTGGCATTGATGTCTCCTAGGGGAATAAATCTTTGGGCACCTAGTGGTCATG

6 CATGACCACTAGGAGCATCTTTGGCGAGATCGTGAGAATCGGTGTCATTGGTGTCTCCTAGGGGAATAAATCTTTGGGCACCTAGTGGTCATG

6 CATGACCACTAGGAGCATCTTTGGCGAACGGCAGGTGTTGCGGTGGTCTGTGATTCCCTAGGGGAATAAATCTTTGGGCACCTAGTGGTCATG

6 CATGACCACTAGGAGCATCTTTGGCGAGCCTCGCTTGGGAGGTAGCTCCACCAGTTCCTAGGGGAATAAATCTTTGGGCACCTAGTGGTCATG

6 CATGACCACTAGGAGCATCTTTGGCGAGAAGACTCTGGATTCGGGGACGAGTTGCTGCTAGGGGAATAAATCTTTGGGCACCTAGTGGTCATG

6 CATGACCACTAGGAGCATCTTTGGCGAGATCGAGAGGATCGGTGGCATTGGTGTCTCCTAGGGGAATAAATCTTTGGGCACCTAGTGGTCATG

6 CATGACCACTAGGAGCATCTTTGGCGAATGGCAGGTGTTGCGGTGGTCTGTGTATCCCTAGGGGAATAAATCTTTGGGCACCTAGTGGTCATG

6 CATGACCACTAGGAGCATCTTTGGCGATAGCGGCGTAGGCTGCCTTGGGTGGTGGCCCTAGGGGAATAAATCTTTGGGCACCTAGTGGTCATG

6 CATGACCACTAGGAGCATCTTTGGCGATCGCATAGCGGAAGCAGGTAGTGCTCGGGCCTAGGGGAATAAATCTTTGGGCACCTAGTGGTCATG

6 CATGACCACTAGGAGCATCTTTGGCGACTCGACCGGGGCCCTAGGGTTGCGGCCCACCTAGGGGAATAAATCTTTGGGCACCTAGTGGTCATG

6 CATGACCACTAGGAGCATCTTTGGCGAGATCGGGAGAATCGGTGTCATTGGTGTCTACTAGGGGAATAAATCTTTGGGCACCTAGTGGTCATG

6 CATGACCACTAGGAGCATCTTTGGCGAGCCTTGCTTGGGAGGCTGCTCCACCAGTTCCTAGGGGAATAAATCTTTGGGCACCTAGTGGTCATG

6 CATGACCACTAGGAGCATCTTTGGCGAGATTGGGAGAATCGGTGGCATTGGTGTCCCCTAGGGGAATAAATCTTTGGGCACCTAGTGGTCATG

6 CATGACCACTAGGAGCATCTTTGGCGACGGTGTGGGGAACTCGTTTCGGCGGTGCTACTAGGGGAATAAATCTTTGGGCACCTAGTGGTCATG

6 CATGACCACTAGGAGCATCTTTGGCGATAGCCGAAGGGACCAGAAGGGAGTCACTGTCTAGGGGAATAAATCTTTGGGCACCTAGTGGTCATG

6 CATGACCACTAGGAGCATCTTTGGCGAGGGAGGGCGCCGGCAGCGGTGTGAATGAGACTAGGGGAATAAATCTTTGGGCACCTAGTGGTCATG

6 CATGACCACTAGGAGCATCTTTGGCGACTGTGGCTGGTAGGTTCGTACTATAGGCGGCTAGGGGAATAAATCTTTGGGCACCTAGTGGTCATG

6 CATGACCACTAGGAGCATCTTTGGCGAGATTGGGAGAATCGGTGGCACTGGTGTCTCCTAGGGGAATAAATCTTTGGGCACCTAGTGGTCATG

6 CATGACCACTAGGAGCATCTTTGGCGACGGTGTGGGGACCGCAGGTTCGGCGGCTACCTAGGGGAATAAATCTTTGGGCACCTAGTGGTCATG

6 CATGACCACTAGGAGCATCTTTGGCGAGTCGGGGATTGGCAGGGGCGTTGACTGGCACTAGGGGAATAAATCTTTGGGCACCTAGTGGTCATG

6 CATGACCACTAGGAGCATCTTTGGCGAGGGAGGGCGTCGGCAGCGGTGTGAATGCGACTAGGGGAATAAATCTTTGGGCACCTAGTGGTCATG

6 CATGACCACTAGGAGCATCTTTGGCGAGATCGAGAGAATCGGTGGCGTTGGTGTCTCCTAGGGGAATAAATCTTTGGGCACCTAGTGGTCATG

6 CATGACCACTAGGAGCATCTTTGGCGAGATCGGGGGAATCGGTGGCATTGGTGTCTTCTAGGGGAATAAATCTTTGGGCACCTAGTGGTCATG

6 CATGACCACTAGGAGCATCTTTGGCGACGGGTTGCTGGTAGGTGGTACGACCTCGATCTAGGGGAATAAATCTTTGGGCACCTAGTGGTCATG

6 CATGACCACTAGGAGCATCTTTGGCGAGATCGGTAGAATCGGTGGCATTGGAGTCTCCTAGGGGAATAAATCTTTGGGCACCTAGTGGTCATG

6 CATGACCACTAGGAGCATCTTTGGCGACGGTGTGGGGAACTTGTTTCGGCGGTGTTACTAGGGGAATAAATCTTTGGGCACCTAGTGGTCATG

6 CATGACCACTAGGAGCATCTTTGGCGATCCGGGGGCACCTATGTGCGGCGCTGTGGGCTAGGGGAATAAATCTTTGGGCACCTAGTGGTCATG

6 CATGACCACTAGGAGCATCTTTGGCGAACGTGCAAAATATGCCGCCTGGAGCGTAGGCTAGGGGAATAAATCTTTGGGCACCTAGTGGTCATG

6 CATGACCACTAGGAGCATCTTTGGCGATCCGGGGGTGCGCGAGAACGACGACTTGGGCTAGGGGAATAAATCTTTGGGCACCTAGTGGTCATG

6 CATGACCACTAGGAGCATCTTTGGCGAAAGTCGGTATAACAGGGTGGGGTGCTGTCCCTAGGGGAATAAATCTTTGGGCACCTAGTGGTCATG

6 CATGACCACTAGGAGCATCTTTGGCGAAAGTCGGTAGAACAGGGTGGGGTGCCGTCCCTAGGGGAATAAATCTTTGGGCACCTAGTGGTCATG

6 CATGACCACTAGGAGCATCTTTGGCGAATGGCCGGCACGGCCCTCTAGTCCTCGGTACTAGGGGAATAAATCTTTGGGCACCTAGTGGTCATG

6 CATGACCACTAGGAGCATCTTTGGCGAGCGGCAGGTGTTGCGGTGGTTTGTGAATCCCTAGGGGAATAAATCTTTGGGCACCTAGTGGTCATG

6 CATGACCACTAGGAGCATCTTTGGCGAGAAGACTCTGGATTCGGGGTCCAGTTGCTGCTAGGGGAATAAATCTTTGGGCACCTAGTGGTCATG

6 CATGACCACTAGGAGCATCTTTGGCGAGGGCCGGTCGCGGAGGTGGTCTGGCGAACCCTAGGGGAATAAATCTTTGGGCACCTAGTGGTCATG

6 CATGACCACTAGGAGCATCTTTGGCGAGCCTAGCTTGGGAGGTTGCTCCACCAGTTCCTAGGGGAATAAATCTTTGGGCACCTAGTGGTCATG

6 CATGACCACTAGGAGCATCTTTGGCGAGATCGGGAGAATCGTAGGCATTGGTGTCTCCTAGGGGAATAAATCTTTGGGCACCTAGTGGTCATG

6 CATGACCACTAGGAGCATCTTTGGCGAGATCGGGAGAAACGGAGGCATTGGTGTCTCCTAGGGGAATAAATCTTTGGGCACCTAGTGGTCATG

6 CATGACCACTAGGAGCATCTTTGGCGAGCCTCGCTTGGGAGGTTGCTCCACCAGCTCCTAGGGGAATAAATCTTTGGGCACCTAGTGGTCATG

6 CATGACCACTAGGAGCATCTTTGGCGAGATCGGGAGAATCGGATGCATTGGTGTCTCCTAGGGGAATAAATCTTTGGGCACCTAGTGGTCATG

6 CATGACCACTAGGAGCATCTTTGGCGATCGCATAGCGTACACTTCAAAAAGTGCGGACTAGGGGAATAAATCTTTGGGCACCTAGTGGTCATG

6 CATGACCACTAGGAGCATCTTTGGCGAGGTCGTGAGAATCGGTGGCATTGGTGTCTCCTAGGGGAATAAATCTTTGGGCACCTAGTGGTCATG

6 CATGACCACTAGGAGCATCTTTGGCGAGATTGGGAGAATCGGAGGCATTGGTGTCTCCTAGGGGAATAAATCTTTGGGCACCTAGTGGTCATG

6 CATGACCACTAGGAGCATCTTTGGCGACGGCGTGGGGCCATATGTGCGGCGGATCCCCTAGGGGAATAAATCTTTGGGCACCTAGTGGTCATG

6 CATGACCACTAGGAGCATCTTTGGCGAAGCGAAGGAGCTCGTAAGGCGGCGTACCGGCTAGGGGAATAAATCTTTGGGCACCTAGTGGTCATG

6 CATGACCACTAGGAGCATCTTTGGCGAGGAGTGGTTGAGGGCGGTGGGTAGGTCGCGCTAGGGGAATAAATCTTTGGGCACCTAGTGGTCATG

6 CATGACCACTAGGAGCATCTTTGGCGAGATCGGGAGGATTGGTGGCATTGGTGTCTCCTAGGGGAATAAATCTTTGGGCACCTAGTGGTCATG

6 CATGACCACTAGGAGCATCTTTGGCGAGATCGAGAGAATTGGTGGCATTGGTGTCTCCTAGGGGAATAAATCTTTGGGCACCTAGTGGTCATG

6 CATGACCACTAGGAGCATCTTTGGCGAGATCAGGAGAATCGGTGGCATTGGTGTCCCCTAGGGGAATAAATCTTTGGGCACCTAGTGGTCATG

6 CATGACCACTAGGAGCATCTTTGGCGAGAGGGGGACAAAGTGCGGGTGGTCGAGCCTCTAGGGGAATAAATCTTTGGGCACCTAGTGGTCATG

6 CATGACCACTAGGAGCATCTTTGGCGAGCGGCAGGTGTTGCGGTGGTCTGTGGATCCCTAGGGGAATAAATCTTTGGGCACCTAGTGGTCATG

6 CATGACCACTAGGAGCATCTTTGGCGAACGGCAGGTGTTGCGGTGGTCTGTGGATTCCTAGGGGAATAAATCTTTGGGCACCTAGTGGTCATG

6 CATGACCACTAGGAGCATCTTTGGCGAACGGCAGGTGTTGCGGTGGTCTGTGGAACCCTAGGGGAATAAATCTTTGGGCACCTAGTGGTCATG

6 CATGACCACTAGGAGCATCTTTGGCGAGAAGACTTTGGATTTGGGGACCAGTTGCTGCTAGGGGAATAAATCTTTGGGCACCTAGTGGTCATG

6 CATGACCACTAGGAGCATCTTTGGCGATTTGGGAGTCGCAGTAAGCAGGCAGTGAGACTAGGGGAATAAATCTTTGGGCACCTAGTGGTCATG

6 CATGACCACTAGGAGCATCTTTGGCGATATCGGGAGAATTGGTGGCATTGGTGTCTCCTAGGGGAATAAATCTTTGGGCACCTAGTGGTCATG

6 CATGACCACTAGGAGCATCTTTGGCGAGATCGGGAGAATTGGTGGCATTGGTGTTTCCTAGGGGAATAAATCTTTGGGCACCTAGTGGTCATG

6 CATGACCACTAGGAGCATCTTTGGCGAGATCGTGAGAATCGGTGGCGTTGGTGTCTCCTAGGGGAATAAATCTTTGGGCACCTAGTGGTCATG

6 CATGACCACTAGGAGCATCTTTGGCGACGGTGTGGGGCTATTGATGCGGCGGCACTCCTAGGGGAATAAATCTTTGGGCACCTAGTGGTCATG

6 CATGACCACTAGGAGCATCTTTGGCGAGATCGGGAGAATCGGAGGCATAGGTGTCTCCTAGGGGAATAAATCTTTGGGCACCTAGTGGTCATG

6 CATGACCACTAGGAGCATCTTTGGCGAAGCGCAGGGTGCGAGAGGGGCGTGTCCATGCTAGGGGAATAAATCTTTGGGCACCTAGTGGTCATG

6 CATGACCACTAGGAGCATCTTTGGCGATCCGGGGGCCAACGGCGACGCACTTAAGGGCTAGGGGAATAAATCTTTGGGCACCTAGTGGTCATG

6 CATGACCACTAGGAGCATCTTTGGCGAGACCAGCAAGGGCTTCGAGGGACGGGAAGGCTAGGGGAATAAATCTTTGGGCACCTAGTGGTCATG

6 CATGACCACTAGGAGCATCTTTGGCGAGAAGAGTCTGGATTCGGGGACCAGTTGCTGCTAGGGGAATAAATCTTTGGGCACCTAGTGGTCATG

6 CATGACCACTAGGAGCATCTTTGGCGAGATCGGGAGAATCGGTGGCATTAATGTCTCCTAGGGGAATAAATCTTTGGGCACCTAGTGGTCATG

6 CATGACCACTAGGAGCATCTTTGGCGACATTGACAGCTAGGGTGAGGTTGTGTGCGACTAGGGGAATAAATCTTTGGGCACCTAGTGGTCATG

6 CATGACCACTAGGAGCATCTTTGGCGAGATTGGGAGAATCGGTGGCATTGGTGTTTCCTAGGGGAATAAATCTTTGGGCACCTAGTGGTCATG

6 CATGACCACTAGGAGCATCTTTGGCGAACGGCAGGTGTTGCGGTGGTCTGTGGATCTCTAGGGGAATAAATCTTTGGGCACCTAGTGGTCATG

6 CATGACCACTAGGAGCATCTTTGGCGAGGGAGGGCGCCGGCAGCGGTGTGAATGTGACTAGGGGAATAAATCTTTGGGCACCTAGTGGTCATG

6 CATGACCACTAGGAGCATCTTTGGCGAGAACACTCTGGATTCGGGGACCAGTTGCTGCTAGGGGAATAAATCTTTGGGCACCTAGTGGTCATG

6 CATGACCACTAGGAGCATCTTTGGCGAGATCGGGAGAATCGGTGGCATAGGAGTCTCCTAGGGGAATAAATCTTTGGGCACCTAGTGGTCATG

6 CATGACCACTAGGAGCATCTTTGGCGAGATCGGAAGAATCGGTGGCATTGGTGCCTCCTAGGGGAATAAATCTTTGGGCACCTAGTGGTCATG

6 CATGACCACTAGGAGCATCTTTGGCGAAAGACGGTAGAACAGGGTGGGGTGCTGTCCCTAGGGGAATAAATCTTTGGGCACCTAGTGGTCATG

6 CATGACCACTAGGAGCATCTTTGGCGAGATCAGGAGAATCGGTGGCATCGGTGTCTCCTAGGGGAATAAATCTTTGGGCACCTAGTGGTCATG

6 CATGACCACTAGGAGCATCTTTGGCGAGAGCCGCCACGGGTTGGGCCGGTGCGACAGCTAGGGGAATAAATCTTTGGGCACCTAGTGGTCATG

5 CATGACCACTAGGAGCATCTTTGGCGAGTGGGGGGCGACGGCCGGTAGTGGGTGAGGCTAGGGGAATAAATCTTTGGGCACCTAGTGGTCATG

5 CATGACCACTAGGAGCATCTTTGGCGATCTGGGGGCACCTATGTGCGACGCTGTGGGCTAGGGGAATAAATCTTTGGGCACCTAGTGGTCATG

5 CATGACCACTAGGAGCATCTTTGGCGAGATCGTGAGAATCGGTGGCATTGGTGCCTCCTAGGGGAATAAATCTTTGGGCACCTAGTGGTCATG

5 CATGACCACTAGGAGCATCTTTGGCGACTAAGTGGGCAACGCGTTAAGTGGGGTAGACTAGGGGAATAAATCTTTGGGCACCTAGTGGTCATG

5 CATGACCACTAGGAGCATCTTTGGCGATCCGGGGGCACCTGTGTGCGACGCTGTGGGCTAGGGGAATAAATCTTTGGGCACCTAGTGGTCATG

5 CATGACCACTAGGAGCATCTTTGGCGAGATCGGGAGAATCGGTGTCATTGTTGTCTCCTAGGGGAATAAATCTTTGGGCACCTAGTGGTCATG

5 CATGACCACTAGGAGCATCTTTGGCGAAGACGACGCAGTCGATCGGGTTAGAGGAAGCTAGGGGAATAAATCTTTGGGCACCTAGTGGTCATG

5 CATGACCACTAGGAGCATCTTTGGCGAGCCTCGCTCGGGAGGTTGCTCCACCAGTTCCTAGGGGAATAAATCTTTGGGCACCTAGTGGTCATG

5 CATGACCACTAGGAGCATCTTTGGCGAGATTGGGAGAATCGGTGACATTGGTGTCTCCTAGGGGAATAAATCTTTGGGCACCTAGTGGTCATG

5 CATGACCACTAGGAGCATCTTTGGCGATCCGGGGGCTGCATGCGGCGACGCGACGGGCTAGGGGAATAAATCTTTGGGCACCTAGTGGTCATG

5 CATGACCACTAGGAGCATCTTTGGCGATCCGGGGGCGCCTATGTGCGACGCTGTGGGCTAGGGGAATAAATCTTTGGGCACCTAGTGGTCATG

5 CATGACCACTAGGAGCATCTTTGGCGAGATCGGGAGAATCTGTGGCATTGGTGTTTCCTAGGGGAATAAATCTTTGGGCACCTAGTGGTCATG

5 CATGACCACTAGGAGCATCTTTGGCGAGATCGCGAGAATCGGTGGCATTGGTGTTTCCTAGGGGAATAAATCTTTGGGCACCTAGTGGTCATG

5 CATGACCACTAGGAGCATCTTTGGCGATTAGCGTCTGCGAGCGGGTGGGTTCGCTCCCTAGGGGAATAAATCTTTGGGCACCTAGTGGTCATG

5 CATGACCACTAGGAGCATCTTTGGCGAGATCGGGAGAATCGGCGGCATAGGTGTCTCCTAGGGGAATAAATCTTTGGGCACCTAGTGGTCATG

5 CATGACCACTAGGAGCATCTTTGGCGAGATCGTGAGAATCGGTGGTATTGGTGTCTCCTAGGGGAATAAATCTTTGGGCACCTAGTGGTCATG

5 CATGACCACTAGGAGCATCTTTGGCGACCCGAGAAGGACAAACGTGCCGTCCAGTGACTAGGGGAATAAATCTTTGGGCACCTAGTGGTCATG

5 CATGACCACTAGGAGCATCTTTGGCGAGATCGGGGGACTCGGTGGCATTGGTGTCTCCTAGGGGAATAAATCTTTGGGCACCTAGTGGTCATG

5 CATGACCACTAGGAGCATCTTTGGCGAGCGGCAGGTGTTGCGGTGGTCTGTGAATCTCTAGGGGAATAAATCTTTGGGCACCTAGTGGTCATG

5 CATGACCACTAGGAGCATCTTTGGCGAGATCAGAAGAATCGGTGGCATTGGTGTCTCCTAGGGGAATAAATCTTTGGGCACCTAGTGGTCATG

5 CATGACCACTAGGAGCATCTTTGGCGAGATCGGGAGAGTCGGTGGCATTGGTGTCTTCTAGGGGAATAAATCTTTGGGCACCTAGTGGTCATG

5 CATGACCACTAGGAGCATCTTTGGCGAGATCGGTAGAATCGGTGGCATTGGTGTTTCCTAGGGGAATAAATCTTTGGGCACCTAGTGGTCATG

5 CATGACCACTAGGAGCATCTTTGGCGAGATCGGAAGAATCGGTGGCATTGGTGTCTTCTAGGGGAATAAATCTTTGGGCACCTAGTGGTCATG

5 CATGACCACTAGGAGCATCTTTGGCGAGATCGGGGGAATTGGTGGCATTGGTGTCTCCTAGGGGAATAAATCTTTGGGCACCTAGTGGTCATG

5 CATGACCACTAGGAGCATCTTTGGCGATATCGTGAGAATCGGTGGCATTGGTGTCTCCTAGGGGAATAAATCTTTGGGCACCTAGTGGTCATG

5 CATGACCACTAGGAGCATCTTTGGCGAGATCGGGAGAATCGGTGGCATAGGTGTCTACTAGGGGAATAAATCTTTGGGCACCTAGTGGTCATG

5 CATGACCACTAGGAGCATCTTTGGCGAACGGCAGGTGTTGCGGTGGTCTATGAATCTCTAGGGGAATAAATCTTTGGGCACCTAGTGGTCATG

5 CATGACCACTAGGAGCATCTTTGGCGAGATCGGGAGAATCGGTGGAATTGGTGACTCCTAGGGGAATAAATCTTTGGGCACCTAGTGGTCATG

5 CATGACCACTAGGAGCATCTTTGGCGATCGCACAGCGCACGAAAATTGTGTAGGGGACTAGGGGAATAAATCTTTGGGCACCTAGTGGTCATG

5 CATGACCACTAGGAGCATCTTTGGCGACGGCGTGGGGCTATCTGTGCGGCGGATCCCCTAGGGGAATAAATCTTTGGGCACCTAGTGGTCATG

5 CATGACCACTAGGAGCATCTTTGGCGAATGGCCGGCACGGCCTTCTAGTCCTCAGTACTAGGGGAATAAATCTTTGGGCACCTAGTGGTCATG

5 CATGACCACTAGGAGCATCTTTGGCGACGGGATCGTGTCGGCTGATAGCGTACTTGGCTAGGGGAATAAATCTTTGGGCACCTAGTGGTCATG

5 CATGACCACTAGGAGCATCTTTGGCGAGCCTCGCTTGGTAGGTTGCTCCACCAGTTCCTAGGGGAATAAATCTTTGGGCACCTAGTGGTCATG

5 CATGACCACTAGGAGCATCTTTGGCGAGATTGGGAGAATCGGTGGCATTAGTGTCTCCTAGGGGAATAAATCTTTGGGCACCTAGTGGTCATG

5 CATGACCACTAGGAGCATCTTTGGCGACTAAGTGGACAACGCGTTAAGTGGGGTAGACTAGGGGAATAAATCTTTGGGCACCTAGTGGTCATG

5 CATGACCACTAGGAGCATCTTTGGCGACTACTGTAAGCGAAAGAAGCAACAGTGGAGCTAGGGGAATAAATCTTTGGGCACCTAGTGGTCATG

5 CATGACCACTAGGAGCATCTTTGGCGAAAGTCGGTAGAACAGGGTGGGGTGCAGTCCCTAGGGGAATAAATCTTTGGGCACCTAGTGGTCATG

5 CATGACCACTAGGAGCATCTTTGGCGAGATCGGGAGAATCTGTGGCATAGGTGTCTCCTAGGGGAATAAATCTTTGGGCACCTAGTGGTCATG

5 CATGACCACTAGGAGCATCTTTGGCGATCGCACAGCAGGCAAAGAAGTTGACTAGGCCTAGGGGAATAAATCTTTGGGCACCTAGTGGTCATG

5 CATGACCACTAGGAGCATCTTTGGCGAGATTGGTAGAATCGGTGGCATTGGTGTCTCCTAGGGGAATAAATCTTTGGGCACCTAGTGGTCATG

5 CATGACCACTAGGAGCATCTTTGGCGACTAGATATGGTAGGTAATGTGCTGAGGAGGCTAGGGGAATAAATCTTTGGGCACCTAGTGGTCATG

5 CATGACCACTAGGAGCATCTTTGGCGAACCTTGCTTGGGAGGTTGCTCCACCAGTTCCTAGGGGAATAAATCTTTGGGCACCTAGTGGTCATG

5 CATGACCACTAGGAGCATCTTTGGCGAGATCTGGAGAATCTGTGGCATTGGTGTCTCCTAGGGGAATAAATCTTTGGGCACCTAGTGGTCATG

5 CATGACCACTAGGAGCATCTTTGGCGAGCCTCGATTGGGAGGTTGCTCCACCAGTTCCTAGGGGAATAAATCTTTGGGCACCTAGTGGTCATG

5 CATGACCACTAGGAGCATCTTTGGCGATGGGTGCTGACGGTCGCCGCTGCGGCTACACTAGGGGAATAAATCTTTGGGCACCTAGTGGTCATG

5 CATGACCACTAGGAGCATCTTTGGCGACCCAGCTAAAATGAGTCACGGTTCGGAAGTCTAGGGGAATAAATCTTTGGGCACCTAGTGGTCATG

5 CATGACCACTAGGAGCATCTTTGGCGAGTAGTGGCTGAGAGCACCAAGTGTCCGAGACTAGGGGAATAAATCTTTGGGCACCTAGTGGTCATG

5 CATGACCACTAGGAGCATCTTTGGCGAGATCGCGAGAATCGGCGGCATTGGTGTCTCCTAGGGGAATAAATCTTTGGGCACCTAGTGGTCATG

5 CATGACCACTAGGAGCATCTTTGGCGAGTATCGGCTGGTAGGTTGCGTATTGGGGAGCTAGGGGAATAAATCTTTGGGCACCTAGTGGTCATG

5 CATGACCACTAGGAGCATCTTTGGCGATCCGGGGGTCCCCCAGGGACGACGCTGGGGCTAGGGGAATAAATCTTTGGGCACCTAGTGGTCATG

5 CATGACCACTAGGAGCATCTTTGGCGAGATCGGGAGAATCGGCGACATTGGTGTCTCCTAGGGGAATAAATCTTTGGGCACCTAGTGGTCATG

5 CATGACCACTAGGAGCATCTTTGGCGAGGGAAGTGGTCAAGCGGCAGCTCCCGTAGACTAGGGGAATAAATCTTTGGGCACCTAGTGGTCATG

5 CATGACCACTAGGAGCATCTTTGGCGAGATCGGGAGAATCGGTGGCAATGGAGTCTCCTAGGGGAATAAATCTTTGGGCACCTAGTGGTCATG

5 CATGACCACTAGGAGCATCTTTGGCGAGATCTGGAGAATCGGTGGCATTTGTGTCTCCTAGGGGAATAAATCTTTGGGCACCTAGTGGTCATG

5 CATGACCACTAGGAGCATCTTTGGCGAGCCTTGCTTGAGAGGTTGCTCCACCAGTTCCTAGGGGAATAAATCTTTGGGCACCTAGTGGTCATG

5 CATGACCACTAGGAGCATCTTTGGCGAGGAAGGGCGCCGGCAGCGGTGTGAATGCGACTAGGGGAATAAATCTTTGGGCACCTAGTGGTCATG

5 CATGACCACTAGGAGCATCTTTGGCGATCGCATAGCGGACCAGTCAGAAGTGGGGGCCTAGGGGAATAAATCTTTGGGCACCTAGTGGTCATG

5 CATGACCACTAGGAGCATCTTTGGCGAGATCGGGAGAATCAGCGGCATTGGTGTCTCCTAGGGGAATAAATCTTTGGGCACCTAGTGGTCATG

5 CATGACCACTAGGAGCATCTTTGGCGAGAAGACTCTGGATTCGGGGACCCGTTGCTGCTAGGGGAATAAATCTTTGGGCACCTAGTGGTCATG

5 CATGACCACTAGGAGCATCTTTGGCGATCAAGTGCGCTTGGTAATTCCTGATGCGACCTAGGGGAATAAATCTTTGGGCACCTAGTGGTCATG

5 CATGACCACTAGGAGCATCTTTGGCGAGAAGCCTCTGGATTCGGGGACCAGTTGCTGCTAGGGGAATAAATCTTTGGGCACCTAGTGGTCATG

5 CATGACCACTAGGAGCATCTTTGGCGACTCGTGAGGCATGGGTATGCTGTCCGGTGACTAGGGGAATAAATCTTTGGGCACCTAGTGGTCATG

5 CATGACCACTAGGAGCATCTTTGGCGATGGGGCCGTGCTATCTGCACACTCTGCGGGCTAGGGGAATAAATCTTTGGGCACCTAGTGGTCATG

5 CATGACCACTAGGAGCATCTTTGGCGACAGCAAAGGGTGTGAGCAAGGCGAGTCCTACTAGGGGAATAAATCTTTGGGCACCTAGTGGTCATG

5 CATGACCACTAGGAGCATCTTTGGCGAGATTGGGAGGATCGGTGGCATTGGTGTCTCCTAGGGGAATAAATCTTTGGGCACCTAGTGGTCATG

5 CATGACCACTAGGAGCATCTTTGGCGAGAAGACTCTGGATTCGGGGACCAGTTGGTGCTAGGGGAATAAATCTTTGGGCACCTAGTGGTCATG

5 CATGACCACTAGGAGCATCTTTGGCGAGATCTGGGGAATCGGTGGCATTGGTGTCTCCTAGGGGAATAAATCTTTGGGCACCTAGTGGTCATG

5 CATGACCACTAGGAGCATCTTTGGCGAGATTGGAAGAATCGGTGGCATTGGTGTCTCCTAGGGGAATAAATCTTTGGGCACCTAGTGGTCATG

5 CATGACCACTAGGAGCATCTTTGGCGACAATGGAGCGACGGTGTGGTCTGAGTCTTACTAGGGGAATAAATCTTTGGGCACCTAGTGGTCATG

5 CATGACCACTAGGAGCATCTTTGGCGAGAAGACTCTGGATTCGGGGACCACTTGCTGCTAGGGGAATAAATCTTTGGGCACCTAGTGGTCATG

5 CATGACCACTAGGAGCATCTTTGGCGAGATCGGGAGAATCGGCTGCATTGGTGTCTCCTAGGGGAATAAATCTTTGGGCACCTAGTGGTCATG

5 CATGACCACTAGGAGCATCTTTGGCGAGCCTTGATTGGGAGGTTGCTCCACCAGTTCCTAGGGGAATAAATCTTTGGGCACCTAGTGGTCATG

5 CATGACCACTAGGAGCATCTTTGGCGATCGCATAGCGGACGACGAATGTGTCGGGGCCTAGGGGAATAAATCTTTGGGCACCTAGTGGTCATG

5 CATGACCACTAGGAGCATCTTTGGCGAGATCGGGAGAATCGGCGGCATTTGTGTCTCCTAGGGGAATAAATCTTTGGGCACCTAGTGGTCATG

5 CATGACCACTAGGAGCATCTTTGGCGAGCCCCGCTTGGGAGGTTGCTCCACCAGTTCCTAGGGGAATAAATCTTTGGGCACCTAGTGGTCATG

5 CATGACCACTAGGAGCATCTTTGGCGACATGGGCGACCTAAGAGAGGAGAAGTCGAACTAGGGGAATAAATCTTTGGGCACCTAGTGGTCATG

5 CATGACCACTAGGAGCATCTTTGGCGACAAGCGATGGAACTATAGCGAGACGTAGTGCTAGGGGAATAAATCTTTGGGCACCTAGTGGTCATG

5 CATGACCACTAGGAGCATCTTTGGCGAGGTCGGGAGAATCGGCGGCATTGGTGTCTCCTAGGGGAATAAATCTTTGGGCACCTAGTGGTCATG

5 CATGACCACTAGGAGCATCTTTGGCGAGCCTTGCTTGGGAGGTTGCTCCACCAGTACCTAGGGGAATAAATCTTTGGGCACCTAGTGGTCATG

5 CATGACCACTAGGAGCATCTTTGGCGAGATCGGGAGAATCGGCGGCAGTGGTGTCTCCTAGGGGAATAAATCTTTGGGCACCTAGTGGTCATG

5 CATGACCACTAGGAGCATCTTTGGCGAGATCGGGAGAATCGGCGGCGTTGGTGTCTCCTAGGGGAATAAATCTTTGGGCACCTAGTGGTCATG

5 CATGACCACTAGGAGCATCTTTGGCGAGGTGGGGTGTGGATAAGCGAGTGGTCGGCACTAGGGGAATAAATCTTTGGGCACCTAGTGGTCATG

5 CATGACCACTAGGAGCATCTTTGGCGACGAGCTGGGTGCGCGTTAGATTGGGATAGCCTAGGGGAATAAATCTTTGGGCACCTAGTGGTCATG

5 CATGACCACTAGGAGCATCTTTGGCGAGATCGAGAGAAACGGTGGCATTGGTGTCTCCTAGGGGAATAAATCTTTGGGCACCTAGTGGTCATG

5 CATGACCACTAGGAGCATCTTTGGCGAATGAGCCTGTAGGCCAGAGCAGACGTTTGGCTAGGGGAATAAATCTTTGGGCACCTAGTGGTCATG

5 CATGACCACTAGGAGCATCTTTGGCGACATCAGCTCCGCGGTCGGGGACGTAAGGGACTAGGGGAATAAATCTTTGGGCACCTAGTGGTCATG

5 CATGACCACTAGGAGCATCTTTGGCGAGCCTTGCTTGGGAGATTGCTCCACCAGTTCCTAGGGGAATAAATCTTTGGGCACCTAGTGGTCATG

5 CATGACCACTAGGAGCATCTTTGGCGACGTGGCTGGTAGGGTAAGTACTGTGTCGATCTAGGGGAATAAATCTTTGGGCACCTAGTGGTCATG

5 CATGACCACTAGGAGCATCTTTGGCGAATGGCCGGCGCGGCCTTCTAGTCCTCGGTACTAGGGGAATAAATCTTTGGGCACCTAGTGGTCATG

5 CATGACCACTAGGAGCATCTTTGGCGAGATCGGGAGAATCGGTGGTATTGGTGTCTTCTAGGGGAATAAATCTTTGGGCACCTAGTGGTCATG

5 CATGACCACTAGGAGCATCTTTGGCGAAGCAGAAGGTGTGATGTTGTAAGAGAACTACTAGGGGAATAAATCTTTGGGCACCTAGTGGTCATG

5 CATGACCACTAGGAGCATCTTTGGCGATCCGGGGGCGCTCAGGGCGCGACGCTGGGGCTAGGGGAATAAATCTTTGGGCACCTAGTGGTCATG

5 CATGACCACTAGGAGCATCTTTGGCGAGATCGGAAGAATCGGTGGCATCGGTGTCTCCTAGGGGAATAAATCTTTGGGCACCTAGTGGTCATG

5 CATGACCACTAGGAGCATCTTTGGCGATCCGGGGGGTCGGGAAGACCGACGCTAGGGCTAGGGGAATAAATCTTTGGGCACCTAGTGGTCATG

5 CATGACCACTAGGAGCATCTTTGGCGAGATTGGGAGAATCGGTGGCATTGGCGTCTCCTAGGGGAATAAATCTTTGGGCACCTAGTGGTCATG

5 CATGACCACTAGGAGCATCTTTGGCGACGGCGTGGGGCATCTCTCGCGGCGGTTATTCTAGGGGAATAAATCTTTGGGCACCTAGTGGTCATG

5 CATGACCACTAGGAGCATCTTTGGCGAGATCGGGAGAATCGGTGGCATCGGTGTTTCCTAGGGGAATAAATCTTTGGGCACCTAGTGGTCATG

5 CATGACCACTAGGAGCATCTTTGGCGAATTGCCGGCACGGCCTTCTAGTCCTCGGTACTAGGGGAATAAATCTTTGGGCACCTAGTGGTCATG

5 CATGACCACTAGGAGCATCTTTGGCGATCCGGGGGCCTGAATTGGCGACGACTTGGGCTAGGGGAATAAATCTTTGGGCACCTAGTGGTCATG

5 CATGACCACTAGGAGCATCTTTGGCGATATCTGGAGAATCGGTGGCATTGGTGTCTCCTAGGGGAATAAATCTTTGGGCACCTAGTGGTCATG

5 CATGACCACTAGGAGCATCTTTGGCGAGATCGGGAGAAACGGTGGCATTGGAGTCTCCTAGGGGAATAAATCTTTGGGCACCTAGTGGTCATG

5 CATGACCACTAGGAGCATCTTTGGCGAGATCGGGAGAATCGGTGGACTTGGTGTCTCCTAGGGGAATAAATCTTTGGGCACCTAGTGGTCATG

5 CATGACCACTAGGAGCATCTTTGGCGATATCGGGAGAATCGGTGGCATTGGTGTCTACTAGGGGAATAAATCTTTGGGCACCTAGTGGTCATG

5 CATGACCACTAGGAGCATCTTTGGCGAGTGGGGGGCGACGGCCGTTAGTGGGTGAGACTAGGGGAATAAATCTTTGGGCACCTAGTGGTCATG

5 CATGACCACTAGGAGCATCTTTGGCGATTAGGGAGTCGCAGTATGCAGGCAGTGAGACTAGGGGAATAAATCTTTGGGCACCTAGTGGTCATG

5 CATGACCACTAGGAGCATCTTTGGCGAGATCGAGAGAATCGGTGGCATTGGTGTCCCCTAGGGGAATAAATCTTTGGGCACCTAGTGGTCATG

5 CATGACCACTAGGAGCATCTTTGGCGACGGTGTGGGGACCTTGTTTCGGCGGTGCTACTAGGGGAATAAATCTTTGGGCACCTAGTGGTCATG

5 CATGACCACTAGGAGCATCTTTGGCGACGATTGGTCGGAAGGCAAGTGTTCGGATGACTAGGGGAATAAATCTTTGGGCACCTAGTGGTCATG

5 CATGACCACTAGGAGCATCTTTGGCGAGCCTTGCTTGGGAGGTTGCCCCACCAGTTCCTAGGGGAATAAATCTTTGGGCACCTAGTGGTCATG

5 CATGACCACTAGGAGCATCTTTGGCGACGGTGTGGGGAACTTGTTTCGGCGGTGCTGCTAGGGGAATAAATCTTTGGGCACCTAGTGGTCATG

5 CATGACCACTAGGAGCATCTTTGGCGACTAGGTTCCGCTCCGGACCGCGACGTAGTGCTAGGGGAATAAATCTTTGGGCACCTAGTGGTCATG

5 CATGACCACTAGGAGCATCTTTGGCGACGGCGTGGGGCTATTGATGCGGCGGCACTTCTAGGGGAATAAATCTTTGGGCACCTAGTGGTCATG

5 CATGACCACTAGGAGCATCTTTGGCGATATGCAGGAGCAGGATCCGGGTGGGCAGGCCTAGGGGAATAAATCTTTGGGCACCTAGTGGTCATG

5 CATGACCACTAGGAGCATCTTTGGCGAACGGCAGTGTCTAAGTGGCTGCATTGGAACCTAGGGGAATAAATCTTTGGGCACCTAGTGGTCATG

5 CATGACCACTAGGAGCATCTTTGGCGACGTGGTGTGTTGCCCAAGTGGCTGAGTTGACTAGGGGAATAAATCTTTGGGCACCTAGTGGTCATG

5 CATGACCACTAGGAGCATCTTTGGCGAGTGGGGGGCGACGGCCGGTAGTGGGCGAGACTAGGGGAATAAATCTTTGGGCACCTAGTGGTCATG

5 CATGACCACTAGGAGCATCTTTGGCGAGATCGGCAGAATCGGTGGCATTGGTGTCTTCTAGGGGAATAAATCTTTGGGCACCTAGTGGTCATG

5 CATGACCACTAGGAGCATCTTTGGCGAGATCGCGAGAATTGGTGGCATTGGTGTCTCCTAGGGGAATAAATCTTTGGGCACCTAGTGGTCATG

5 CATGACCACTAGGAGCATCTTTGGCGATCGCATAGCGTACGACGTTGAGGACGGGGACTAGGGGAATAAATCTTTGGGCACCTAGTGGTCATG

5 CATGACCACTAGGAGCATCTTTGGCGACGGTGTGGGGACCGCAGGCTCGGCGGGTACCTAGGGGAATAAATCTTTGGGCACCTAGTGGTCATG

5 CATGACCACTAGGAGCATCTTTGGCGACTACGGGCTAGAGTTACTCGTGTTAATAAGCTAGGGGAATAAATCTTTGGGCACCTAGTGGTCATG

5 CATGACCACTAGGAGCATCTTTGGCGAAGCGAGGGTGTGAAAGGCACTGAAGTACTACTAGGGGAATAAATCTTTGGGCACCTAGTGGTCATG

5 CATGACCACTAGGAGCATCTTTGGCGAGGGTGTGGGGAACTTGTTTCGGCGGTGCTACTAGGGGAATAAATCTTTGGGCACCTAGTGGTCATG

5 CATGACCACTAGGAGCATCTTTGGCGACCCGGGGGCACCTATGTGCGACGCTGTGGGCTAGGGGAATAAATCTTTGGGCACCTAGTGGTCATG

5 CATGACCACTAGGAGCATCTTTGGCGAGATCGTGAGAGTCGGTGGCATTGGTGTCTCCTAGGGGAATAAATCTTTGGGCACCTAGTGGTCATG

5 CATGACCACTAGGAGCATCTTTGGCGAGCCTCGCTTAGGAGGTTGCTCCACCAGTTCCTAGGGGAATAAATCTTTGGGCACCTAGTGGTCATG

5 CATGACCACTAGGAGCATCTTTGGCGAGATCGGGAGAGTTGGTGGCATTGGTGTCTCCTAGGGGAATAAATCTTTGGGCACCTAGTGGTCATG

4 CATGACCACTAGGAGCATCTTTGGCGATCGCATAGCTATAAGGGAGTGAATACGGGCCTAGGGGAATAAATCTTTGGGCACCTAGTGGTCATG

4 CATGACCACTAGGAGCATCTTTGGCGAGAAGACTTTGGATTCGGGGATCAGTTGCTGCTAGGGGAATAAATCTTTGGGCACCTAGTGGTCATG

4 CATGACCACTAGGAGCATCTTTGGCGAGATCGGGAGACTCGGTGGCATTGGTGTCCCCTAGGGGAATAAATCTTTGGGCACCTAGTGGTCATG

4 CATGACCACTAGGAGCATCTTTGGCGACCCGGTCTTACGAAGGAATGCCGTGGTGGACTAGGGGAATAAATCTTTGGGCACCTAGTGGTCATG

4 CATGACCACTAGGAGCATCTTTGGCGAGGGAGGGCTCCGGCAGCGGTGTGAATGCGACTAGGGGAATAAATCTTTGGGCACCTAGTGGTCATG

4 CATGACCACTAGGAGCATCTTTGGCGAGATCGGGAGAATCTGTGGCATTGGTTTCTCCTAGGGGAATAAATCTTTGGGCACCTAGTGGTCATG

4 CATGACCACTAGGAGCATCTTTGGCGATAACGGGAGAATCGGTGGCATTGGTGTCTCCTAGGGGAATAAATCTTTGGGCACCTAGTGGTCATG

4 CATGACCACTAGGAGCATCTTTGGCGAAAGTCGGTAGAACAGGGTGGGGTGCTGTCGCTAGGGGAATAAATCTTTGGGCACCTAGTGGTCATG

4 CATGACCACTAGGAGCATCTTTGGCGAACGGCAGGTGTTGCGGTGGTCTGTGGATCACTAGGGGAATAAATCTTTGGGCACCTAGTGGTCATG

4 CATGACCACTAGGAGCATCTTTGGCGAGATCGGGAGAATCGGTGGCATAGGTGTATCCTAGGGGAATAAATCTTTGGGCACCTAGTGGTCATG

4 CATGACCACTAGGAGCATCTTTGGCGAGATCGGGAGAATCGGCGGCATTGGGGTCTCCTAGGGGAATAAATCTTTGGGCACCTAGTGGTCATG

4 CATGACCACTAGGAGCATCTTTGGCGACAAGTGACAGAGGTGGAACGTGACGTAGTGCTAGGGGAATAAATCTTTGGGCACCTAGTGGTCATG

4 CATGACCACTAGGAGCATCTTTGGCGACCAGCAGTCCCAGGAATGCGAGACGTAGTGCTAGGGGAATAAATCTTTGGGCACCTAGTGGTCATG

4 CATGACCACTAGGAGCATCTTTGGCGATGGGTGCAGACGAGTGCGCTAGCAATTACACTAGGGGAATAAATCTTTGGGCACCTAGTGGTCATG

4 CATGACCACTAGGAGCATCTTTGGCGATATCGGGAGAATAGGTGGCATTGGTGTCTCCTAGGGGAATAAATCTTTGGGCACCTAGTGGTCATG

4 CATGACCACTAGGAGCATCTTTGGCGAGATCGGGAGAATCGGTGGCATAGGTGTCACCTAGGGGAATAAATCTTTGGGCACCTAGTGGTCATG

4 CATGACCACTAGGAGCATCTTTGGCGAGAAGACTCCGGATTCGGGGACCAGTTACTGCTAGGGGAATAAATCTTTGGGCACCTAGTGGTCATG

4 CATGACCACTAGGAGCATCTTTGGCGAGCCTTGCTTGGGAGGTTGCTCCAACAGTTCCTAGGGGAATAAATCTTTGGGCACCTAGTGGTCATG

4 CATGACCACTAGGAGCATCTTTGGCGAGCACTACGTAGCGGTAGGCATTGGTGTCTCCTAGGGGAATAAATCTTTGGGCACCTAGTGGTCATG

4 CATGACCACTAGGAGCATCTTTGGCGACGTGGGCGTTTCGGCCGTCTCGGCGTGTGCCTAGGGGAATAAATCTTTGGGCACCTAGTGGTCATG

4 CATGACCACTAGGAGCATCTTTGGCGACGAGATTTGGTAGGCAGATGTGCTCTAGCACTAGGGGAATAAATCTTTGGGCACCTAGTGGTCATG

4 CATGACCACTAGGAGCATCTTTGGCGACTAAGTGGCCCACGCGTTAAATGGGGTAGACTAGGGGAATAAATCTTTGGGCACCTAGTGGTCATG

4 CATGACCACTAGGAGCATCTTTGGCGAGATCGGGAGAATCGGTGGCATTGGTGCCTTCTAGGGGAATAAATCTTTGGGCACCTAGTGGTCATG

4 CATGACCACTAGGAGCATCTTTGGCGAGGGAGGTCGCCGGCAGCGGTGTGAATGCGACTAGGGGAATAAATCTTTGGGCACCTAGTGGTCATG

4 CATGACCACTAGGAGCATCTTTGGCGAGATCGGGAGAATCGGTGGAATTGGTGTCTACTAGGGGAATAAATCTTTGGGCACCTAGTGGTCATG

4 CATGACCACTAGGAGCATCTTTGGCGAGATCGGGAGAATCGGTGGAATTGTTGTCTCCTAGGGGAATAAATCTTTGGGCACCTAGTGGTCATG

4 CATGACCACTAGGAGCATCTTTGGCGAGAGGCCTAAGTGCGTATCTGTGGTCGAGCTCTAGGGGAATAAATCTTTGGGCACCTAGTGGTCATG

4 CATGACCACTAGGAGCATCTTTGGCGAATGGACGGCACGGCCTTCTAGTCCTCGGTACTAGGGGAATAAATCTTTGGGCACCTAGTGGTCATG

4 CATGACCACTAGGAGCATCTTTGGCGACGGGGCCGGACGGGACGGACACATCGCGGCCTAGGGGAATAAATCTTTGGGCACCTAGTGGTCATG

4 CATGACCACTAGGAGCATCTTTGGCGAAGACTGGTTGAGATGCGGGGTGGTCGAGCTCTAGGGGAATAAATCTTTGGGCACCTAGTGGTCATG

4 CATGACCACTAGGAGCATCTTTGGCGAGATCGGTAGAATCGGTGTCATTGGTGTCTCCTAGGGGAATAAATCTTTGGGCACCTAGTGGTCATG

4 CATGACCACTAGGAGCATCTTTGGCGATGGGTGCTGACGGCTGCCGCTGCGGCTACACTAGGGGAATAAATCTTTGGGCACCTAGTGGTCATG

4 CATGACCACTAGGAGCATCTTTGGCGACGTTGGGAGCGAAGTCCCAGCGAAGTGGACCTAGGGGAATAAATCTTTGGGCACCTAGTGGTCATG

4 CATGACCACTAGGAGCATCTTTGGCGACTCGTGGGCATATATGGGCCACGTAGTGGACTAGGGGAATAAATCTTTGGGCACCTAGTGGTCATG

4 CATGACCACTAGGAGCATCTTTGGCGAGCCTTGCATGGGAGGTTGCTCCACCAGTTCCTAGGGGAATAAATCTTTGGGCACCTAGTGGTCATG

4 CATGACCACTAGGAGCATCTTTGGCGAGATCGGGAGAATCTGTGGCATTGGTGACTCCTAGGGGAATAAATCTTTGGGCACCTAGTGGTCATG

4 CATGACCACTAGGAGCATCTTTGGCGATCCGGGGGCACCTATGTGCGACGCTGCGGGCTAGGGGAATAAATCTTTGGGCACCTAGTGGTCATG

4 CATGACCACTAGGAGCATCTTTGGCGAGATCGGGAGAATCGGTGGCAGTGGTGTCCCCTAGGGGAATAAATCTTTGGGCACCTAGTGGTCATG

4 CATGACCACTAGGAGCATCTTTGGCGAGATCCGGAGAATCGGCGGCATTGGTGTCTCCTAGGGGAATAAATCTTTGGGCACCTAGTGGTCATG

4 CATGACCACTAGGAGCATCTTTGGCGAGATCGTGAGAATCGGTGGCACTGGTGTCTCCTAGGGGAATAAATCTTTGGGCACCTAGTGGTCATG

4 CATGACCACTAGGAGCATCTTTGGCGAGATCGTTAGAATCGGTGGCATTGGTGTCTCCTAGGGGAATAAATCTTTGGGCACCTAGTGGTCATG

4 CATGACCACTAGGAGCATCTTTGGCGAAGACGACGCAGTCGATCGGGATAGAGGAAGCTAGGGGAATAAATCTTTGGGCACCTAGTGGTCATG

4 CATGACCACTAGGAGCATCTTTGGCGACAATGGAGCGACGGTGTGGTCTGAGTTCTACTAGGGGAATAAATCTTTGGGCACCTAGTGGTCATG

4 CATGACCACTAGGAGCATCTTTGGCGAGATCGGGAGAATCGTTGGCATTGGAGTCTCCTAGGGGAATAAATCTTTGGGCACCTAGTGGTCATG

4 CATGACCACTAGGAGCATCTTTGGCGATATCGGGAGAATCGGTGGAATTGGTGTCTCCTAGGGGAATAAATCTTTGGGCACCTAGTGGTCATG

4 CATGACCACTAGGAGCATCTTTGGCGATCCGGGGGCCATGCAGTGGCGACGTCTGGGCTAGGGGAATAAATCTTTGGGCACCTAGTGGTCATG

4 CATGACCACTAGGAGCATCTTTGGCGAGAAGACTCTGGATTCGGGGAGCAGTTGCTGCTAGGGGAATAAATCTTTGGGCACCTAGTGGTCATG

4 CATGACCACTAGGAGCATCTTTGGCGAGATCGGGAGGATCGGTGGCATTGGTGCCTCCTAGGGGAATAAATCTTTGGGCACCTAGTGGTCATG

4 CATGACCACTAGGAGCATCTTTGGCGAGGAGTGGCTGAGGGCGGTGGGTAGGTCGCTCTAGGGGAATAAATCTTTGGGCACCTAGTGGTCATG

4 CATGACCACTAGGAGCATCTTTGGCGAGATCGGAAGAATTGGTGGCATTGGTGTCTCCTAGGGGAATAAATCTTTGGGCACCTAGTGGTCATG

4 CATGACCACTAGGAGCATCTTTGGCGAGGGAGGGCGCCAGCAGCGGTGTGAATGCGACTAGGGGAATAAATCTTTGGGCACCTAGTGGTCATG

4 CATGACCACTAGGAGCATCTTTGGCGAGATCGGGAGAAACGGTGGCATTGGTGACTCCTAGGGGAATAAATCTTTGGGCACCTAGTGGTCATG

4 CATGACCACTAGGAGCATCTTTGGCGAGGGAGGGCGCCGGCAGCGGTGTGAATACGCCTAGGGGAATAAATCTTTGGGCACCTAGTGGTCATG

4 CATGACCACTAGGAGCATCTTTGGCGAGATCGGGAGAATCGGCGGAATTGGTGTCTCCTAGGGGAATAAATCTTTGGGCACCTAGTGGTCATG

4 CATGACCACTAGGAGCATCTTTGGCGAAAGTCGGTAGAACAGGGTGGGGTGCTATCCCTAGGGGAATAAATCTTTGGGCACCTAGTGGTCATG

4 CATGACCACTAGGAGCATCTTTGGCGACCGTAGGAAAACAAAGGTGGCCTTGCTAGCCTAGGGGAATAAATCTTTGGGCACCTAGTGGTCATG

4 CATGACCACTAGGAGCATCTTTGGCGAGGGAGGGCGCCGGCAGAGGTGTGAATGCGACTAGGGGAATAAATCTTTGGGCACCTAGTGGTCATG

4 CATGACCACTAGGAGCATCTTTGGCGAGCCTTGCTTGGGAGGTTGCTCCACCAGTCCCTAGGGGAATAAATCTTTGGGCACCTAGTGGTCATG

4 CATGACCACTAGGAGCATCTTTGGCGACAAGTGGTCCTCTGGCCGCGCGACGTAGTGCTAGGGGAATAAATCTTTGGGCACCTAGTGGTCATG

4 CATGACCACTAGGAGCATCTTTGGCGACTGAGTGGGCTCGTCCGTGGGTGGTGTCGGCTAGGGGAATAAATCTTTGGGCACCTAGTGGTCATG

4 CATGACCACTAGGAGCATCTTTGGCGACTCGTGAAGTGCTACCAAAACGGCCTGTGACTAGGGGAATAAATCTTTGGGCACCTAGTGGTCATG

4 CATGACCACTAGGAGCATCTTTGGCGAGATCGGGAGAATCGGTGGCATAGGTGACTCCTAGGGGAATAAATCTTTGGGCACCTAGTGGTCATG

4 CATGACCACTAGGAGCATCTTTGGCGACGAAGGAAACTTTGTTGACTGATAGTGCGACTAGGGGAATAAATCTTTGGGCACCTAGTGGTCATG

4 CATGACCACTAGGAGCATCTTTGGCGAGCCTCGCTTGGGAGGTTGCTCCACCAGTTGCTAGGGGAATAAATCTTTGGGCACCTAGTGGTCATG

4 CATGACCACTAGGAGCATCTTTGGCGAGATAGGGAGAATCGGCGGCATTGGTGTCTCCTAGGGGAATAAATCTTTGGGCACCTAGTGGTCATG

4 CATGACCACTAGGAGCATCTTTGGCGAGGAGTGGCTGAGGGCGGTGGATAGGTCGCGCTAGGGGAATAAATCTTTGGGCACCTAGTGGTCATG

4 CATGACCACTAGGAGCATCTTTGGCGAGATCGGGAGAATCGGGGGTATTGGTGTCTCCTAGGGGAATAAATCTTTGGGCACCTAGTGGTCATG

4 CATGACCACTAGGAGCATCTTTGGCGAGATCGGGAGAATCGGTGGCATTGCAGTCTCCTAGGGGAATAAATCTTTGGGCACCTAGTGGTCATG

4 CATGACCACTAGGAGCATCTTTGGCGAGGAGTGGCTGAGGGCGGTGGCTAGGTCGCGCTAGGGGAATAAATCTTTGGGCACCTAGTGGTCATG

4 CATGACCACTAGGAGCATCTTTGGCGAGATCAGGAGAATCGGTAGCATTGGTGTCTCCTAGGGGAATAAATCTTTGGGCACCTAGTGGTCATG

4 CATGACCACTAGGAGCATCTTTGGCGAGATCGAGAGAATCGGTGGCATCGGTGTCTCCTAGGGGAATAAATCTTTGGGCACCTAGTGGTCATG

4 CATGACCACTAGGAGCATCTTTGGCGAGATCGGGAGAATCGGTGGCATTGTTGTCTACTAGGGGAATAAATCTTTGGGCACCTAGTGGTCATG

4 CATGACCACTAGGAGCATCTTTGGCGACGGTCGGTCGGTAGGATAGTATTCCGTTGGCTAGGGGAATAAATCTTTGGGCACCTAGTGGTCATG

4 CATGACCACTAGGAGCATCTTTGGCGAGATAGGTAGAATCGGTGGCATTGGTGTCTCCTAGGGGAATAAATCTTTGGGCACCTAGTGGTCATG

4 CATGACCACTAGGAGCATCTTTGGCGACTGGCCGGCACGGCCTTCTAGTCCTCGGTACTAGGGGAATAAATCTTTGGGCACCTAGTGGTCATG

4 CATGACCACTAGGAGCATCTTTGGCGAATGGCCGGAACGGCCTTCTAGTCCTCGGTACTAGGGGAATAAATCTTTGGGCACCTAGTGGTCATG

4 CATGACCACTAGGAGCATCTTTGGCGAGATCTTGAGAATCGGTGGCATTGGTGTCTCCTAGGGGAATAAATCTTTGGGCACCTAGTGGTCATG

4 CATGACCACTAGGAGCATCTTTGGCGAGATCATGAGAATCGGTGGCATTGGTGTCTCCTAGGGGAATAAATCTTTGGGCACCTAGTGGTCATG

4 CATGACCACTAGGAGCATCTTTGGCGATCAGCCGAGGGCACCAATGTTGGGATCAATCTAGGGGAATAAATCTTTGGGCACCTAGTGGTCATG

4 CATGACCACTAGGAGCATCTTTGGCGAACGGCAGGTGTCGCGGTGGTCTGTGTATCCCTAGGGGAATAAATCTTTGGGCACCTAGTGGTCATG

4 CATGACCACTAGGAGCATCTTTGGCGAACGGCAGGTGTTGCGGTGGTTCGTGAATCCCTAGGGGAATAAATCTTTGGGCACCTAGTGGTCATG

4 CATGACCACTAGGAGCATCTTTGGCGAGATCGGGAGAATTGGTGGTATTGGTGTCTCCTAGGGGAATAAATCTTTGGGCACCTAGTGGTCATG

4 CATGACCACTAGGAGCATCTTTGGCGAACACAGGTGTCGTAGTGTCCTCACAGGGTGCTAGGGGAATAAATCTTTGGGCACCTAGTGGTCATG

4 CATGACCACTAGGAGCATCTTTGGCGAGATCGGGAGAATCGGTGGCATTGGAGACTCCTAGGGGAATAAATCTTTGGGCACCTAGTGGTCATG

4 CATGACCACTAGGAGCATCTTTGGCGACGGTGTGGGGCCATCTGTGCGGCGGATCCTCTAGGGGAATAAATCTTTGGGCACCTAGTGGTCATG

4 CATGACCACTAGGAGCATCTTTGGCGAAGTGATCGGAGATTACAAGCAGACGTTGGGCTAGGGGAATAAATCTTTGGGCACCTAGTGGTCATG

4 CATGACCACTAGGAGCATCTTTGGCGATTAGCACAGCGGCGGACGAAGAGGAGGGGCCTAGGGGAATAAATCTTTGGGCACCTAGTGGTCATG

4 CATGACCACTAGGAGCATCTTTGGCGAGCCTCGCTTGGGAGGTTGTTCCACCAGTTCCTAGGGGAATAAATCTTTGGGCACCTAGTGGTCATG

4 CATGACCACTAGGAGCATCTTTGGCGATGGGGCCGTGCTACCTGCACACTCCGCGGGCTAGGGGAATAAATCTTTGGGCACCTAGTGGTCATG

4 CATGACCACTAGGAGCATCTTTGGCGATCCGGGGGCGCCCTGTGCGCGACGCAAGGGCTAGGGGAATAAATCTTTGGGCACCTAGTGGTCATG

4 CATGACCACTAGGAGCATCTTTGGCGAACGACAAATGGAAATGCGACGTAGTGGACGCTAGGGGAATAAATCTTTGGGCACCTAGTGGTCATG

4 CATGACCACTAGGAGCATCTTTGGCGAATGGTCGGCACGGCCTTCTAGTCCTCGGTACTAGGGGAATAAATCTTTGGGCACCTAGTGGTCATG

4 CATGACCACTAGGAGCATCTTTGGCGAGATTGGGAGAGTCGGTGGCATTGGTGTCTCCTAGGGGAATAAATCTTTGGGCACCTAGTGGTCATG

4 CATGACCACTAGGAGCATCTTTGGCGACGTAGGGGGCATGGGTCAGAAAGTCGCCAGCTAGGGGAATAAATCTTTGGGCACCTAGTGGTCATG

4 CATGACCACTAGGAGCATCTTTGGCGAGCCTTGCTTGGGAGGTTGCTCCACCAGATCCTAGGGGAATAAATCTTTGGGCACCTAGTGGTCATG

4 CATGACCACTAGGAGCATCTTTGGCGAATGTCGGTAGAACAGGGTGGGGTGCTGTCCCTAGGGGAATAAATCTTTGGGCACCTAGTGGTCATG

4 CATGACCACTAGGAGCATCTTTGGCGAGATCAGGAGAATCGGTGGCATTGGTGTTTCCTAGGGGAATAAATCTTTGGGCACCTAGTGGTCATG

4 CATGACCACTAGGAGCATCTTTGGCGAGGGAGGGTGCCGGCAGCGGTGTGAATGCGCCTAGGGGAATAAATCTTTGGGCACCTAGTGGTCATG

4 CATGACCACTAGGAGCATCTTTGGCGAGTAGTGCTGGCTAACTAAGTGTGGAACTCGCTAGGGGAATAAATCTTTGGGCACCTAGTGGTCATG

4 CATGACCACTAGGAGCATCTTTGGCGAGAGTGGGAGAATCGGTGGCATTGGTGTCTCCTAGGGGAATAAATCTTTGGGCACCTAGTGGTCATG

4 CATGACCACTAGGAGCATCTTTGGCGAGAAGACCCTGGATTCGGGGACTAGTTGCTGCTAGGGGAATAAATCTTTGGGCACCTAGTGGTCATG

4 CATGACCACTAGGAGCATCTTTGGCGAGATCGGGAGACTCGGTGGCATTGGTGCCTCCTAGGGGAATAAATCTTTGGGCACCTAGTGGTCATG

4 CATGACCACTAGGAGCATCTTTGGCGAATGGCAGGTGTTGCGGTGGTCTGTGAATCTCTAGGGGAATAAATCTTTGGGCACCTAGTGGTCATG

4 CATGACCACTAGGAGCATCTTTGGCGAGATCGGGAGAATCGGTGGCACCGGTGTCTCCTAGGGGAATAAATCTTTGGGCACCTAGTGGTCATG

4 CATGACCACTAGGAGCATCTTTGGCGAGTGTTGCCCGGTACACGAGCAGACGTTTGGCTAGGGGAATAAATCTTTGGGCACCTAGTGGTCATG

4 CATGACCACTAGGAGCATCTTTGGCGAGCCTCGCTTGGGAGGTTGCCCCACCAGTTCCTAGGGGAATAAATCTTTGGGCACCTAGTGGTCATG

4 CATGACCACTAGGAGCATCTTTGGCGAGATCGGGAGAATCTGTGGCATTGTTGTCTCCTAGGGGAATAAATCTTTGGGCACCTAGTGGTCATG

4 CATGACCACTAGGAGCATCTTTGGCGAGATCGTGAGACTCGGTGGCATTGGTGTCTCCTAGGGGAATAAATCTTTGGGCACCTAGTGGTCATG

4 CATGACCACTAGGAGCATCTTTGGCGATCGCTGAGCGAACTGTGGTAAATAGTGGGCCTAGGGGAATAAATCTTTGGGCACCTAGTGGTCATG

4 CATGACCACTAGGAGCATCTTTGGCGATGGGGCCGTGCCATCTGCACACTCCGCGGGCTAGGGGAATAAATCTTTGGGCACCTAGTGGTCATG

4 CATGACCACTAGGAGCATCTTTGGCGAGATTGGGAGAATCGGTGGCATAGGTGTCTCCTAGGGGAATAAATCTTTGGGCACCTAGTGGTCATG

4 CATGACCACTAGGAGCATCTTTGGCGAGGGAGGGCGCCGTCAGCGGTGTGAATGCGACTAGGGGAATAAATCTTTGGGCACCTAGTGGTCATG

4 CATGACCACTAGGAGCATCTTTGGCGACAGCGAAGTGCTGTGAACAGTGGTTAGCACCTAGGGGAATAAATCTTTGGGCACCTAGTGGTCATG

4 CATGACCACTAGGAGCATCTTTGGCGAATGGCCGGCACGGCCTTATAGTCCTCGGTACTAGGGGAATAAATCTTTGGGCACCTAGTGGTCATG

4 CATGACCACTAGGAGCATCTTTGGCGAAGATCGCTCGTCGACTCGGGTCTGAGGAAGCTAGGGGAATAAATCTTTGGGCACCTAGTGGTCATG

4 CATGACCACTAGGAGCATCTTTGGCGAGAAGACTCTGGATTCGGGGACCAGTTCCTGCTAGGGGAATAAATCTTTGGGCACCTAGTGGTCATG

4 CATGACCACTAGGAGCATCTTTGGCGATGGATACGGGAGGACAGTGTGCCAGAATGCCTAGGGGAATAAATCTTTGGGCACCTAGTGGTCATG

4 CATGACCACTAGGAGCATCTTTGGCGATATCGGGAGAATCGGTGGTATTGGTGTCTCCTAGGGGAATAAATCTTTGGGCACCTAGTGGTCATG

4 CATGACCACTAGGAGCATCTTTGGCGATCGCAGAGCGGACGACCTCGGGGCGCGGGACTAGGGGAATAAATCTTTGGGCACCTAGTGGTCATG

4 CATGACCACTAGGAGCATCTTTGGCGAGATCGGGAGAATCGGTGTCAATGGTGTCTCCTAGGGGAATAAATCTTTGGGCACCTAGTGGTCATG

4 CATGACCACTAGGAGCATCTTTGGCGAGATCGGAAGAATCGGTGGTATTGGTGTCTCCTAGGGGAATAAATCTTTGGGCACCTAGTGGTCATG

4 CATGACCACTAGGAGCATCTTTGGCGATCCGGGGGGCTGTCTAGCCGACGCCAGGGGCTAGGGGAATAAATCTTTGGGCACCTAGTGGTCATG

4 CATGACCACTAGGAGCATCTTTGGCGAGATCGGTAGAATCGGTGGCATTGGTGTCTACTAGGGGAATAAATCTTTGGGCACCTAGTGGTCATG

4 CATGACCACTAGGAGCATCTTTGGCGAAAGGGGATGCCGATGCGCTGGACGACCGAGCTAGGGGAATAAATCTTTGGGCACCTAGTGGTCATG

4 CATGACCACTAGGAGCATCTTTGGCGAGAGACTGAGATGTGCAATGTGGTCGAGCATCTAGGGGAATAAATCTTTGGGCACCTAGTGGTCATG

4 CATGACCACTAGGAGCATCTTTGGCGATATCGGGAGAATCGGTGGCATTGGTGTATCCTAGGGGAATAAATCTTTGGGCACCTAGTGGTCATG

4 CATGACCACTAGGAGCATCTTTGGCGACGGTGGGCTGGAAGGGCGTATTCTCGACGCCTAGGGGAATAAATCTTTGGGCACCTAGTGGTCATG

4 CATGACCACTAGGAGCATCTTTGGCGAGCCTCGCTTGGGAGGTTGCTCCAACAGTTCCTAGGGGAATAAATCTTTGGGCACCTAGTGGTCATG

4 CATGACCACTAGGAGCATCTTTGGCGAGATTAGGAGAATCGGTGGCATTGGTGTCTCCTAGGGGAATAAATCTTTGGGCACCTAGTGGTCATG

4 CATGACCACTAGGAGCATCTTTGGCGACGCAACAAGTACGTAGGACGTTGTCGTGGACTAGGGGAATAAATCTTTGGGCACCTAGTGGTCATG

4 CATGACCACTAGGAGCATCTTTGGCGACGGGCCTAAGTGCGTATCTGTGGTCGAGCTCTAGGGGAATAAATCTTTGGGCACCTAGTGGTCATG

4 CATGACCACTAGGAGCATCTTTGGCGAGATTGGGAGACTCGGTGGCATTGGTGTCTCCTAGGGGAATAAATCTTTGGGCACCTAGTGGTCATG

4 CATGACCACTAGGAGCATCTTTGGCGAGATCGGGGGAATCGGTGGTATTGGTGTCTCCTAGGGGAATAAATCTTTGGGCACCTAGTGGTCATG

4 CATGACCACTAGGAGCATCTTTGGCGAGATCGGGAGAATCGGTGGTATTGGTTTCTCCTAGGGGAATAAATCTTTGGGCACCTAGTGGTCATG

4 CATGACCACTAGGAGCATCTTTGGCGAGATCGCGAGAATCGGTGGCATTGGCGTCTCCTAGGGGAATAAATCTTTGGGCACCTAGTGGTCATG

4 CATGACCACTAGGAGCATCTTTGGCGAGATCGGGAGAATCGGTGGCATTGGCGTTTCCTAGGGGAATAAATCTTTGGGCACCTAGTGGTCATG

4 CATGACCACTAGGAGCATCTTTGGCGATCCGGGGGCACCTATGTGCGACGCAGTGGGCTAGGGGAATAAATCTTTGGGCACCTAGTGGTCATG

4 CATGACCACTAGGAGCATCTTTGGCGAACGGCAGGTGTTGCGGTGGTCTATGGATCCCTAGGGGAATAAATCTTTGGGCACCTAGTGGTCATG

4 CATGACCACTAGGAGCATCTTTGGCGAGAAGACTTAGGATTCGGGGACCAGTTGCTGCTAGGGGAATAAATCTTTGGGCACCTAGTGGTCATG

4 CATGACCACTAGGAGCATCTTTGGCGAGATCGGGGGAATCGGTGGCATAGGTGTCTCCTAGGGGAATAAATCTTTGGGCACCTAGTGGTCATG

4 CATGACCACTAGGAGCATCTTTGGCGAGGTGTCTCAGTTCGACATAAGGGGCGAGCACTAGGGGAATAAATCTTTGGGCACCTAGTGGTCATG

4 CATGACCACTAGGAGCATCTTTGGCGAATGGTCGGCACGGCCTTCTAGTCCTTGGTACTAGGGGAATAAATCTTTGGGCACCTAGTGGTCATG

4 CATGACCACTAGGAGCATCTTTGGCGATCCGGGGGCACCTATGTGCGACGCTGTGGACTAGGGGAATAAATCTTTGGGCACCTAGTGGTCATG

4 CATGACCACTAGGAGCATCTTTGGCGAGAAGACTCTCGATTCGGGGACCAGTTGCTGCTAGGGGAATAAATCTTTGGGCACCTAGTGGTCATG

4 CATGACCACTAGGAGCATCTTTGGCGACCCCTGTCGCTGGACATGCGACGGAGTGGACTAGGGGAATAAATCTTTGGGCACCTAGTGGTCATG

4 CATGACCACTAGGAGCATCTTTGGCGAGCCGATTGTGCAGCAATGTGTTCTGGGTAACTAGGGGAATAAATCTTTGGGCACCTAGTGGTCATG

4 CATGACCACTAGGAGCATCTTTGGCGAGATCGTGAGAATCTGTGGCATTGGTGTCTCCTAGGGGAATAAATCTTTGGGCACCTAGTGGTCATG

4 CATGACCACTAGGAGCATCTTTGGCGACTTTGGGAGCAAGTGCGTCGGAGTGGTCGCCTAGGGGAATAAATCTTTGGGCACCTAGTGGTCATG

4 CATGACCACTAGGAGCATCTTTGGCGACAAGTGGCGCTTGCGTCACGAGACATAGTGCTAGGGGAATAAATCTTTGGGCACCTAGTGGTCATG

4 CATGACCACTAGGAGCATCTTTGGCGAAGGGTGTAGGACTTCAAGTGGATCTCGTAGCTAGGGGAATAAATCTTTGGGCACCTAGTGGTCATG

4 CATGACCACTAGGAGCATCTTTGGCGAGATCGGGAGAATCTGTGGCATTGGTGTCTTCTAGGGGAATAAATCTTTGGGCACCTAGTGGTCATG

4 CATGACCACTAGGAGCATCTTTGGCGAGATCGGGAGAATCGGCGGCATTGGTGTCTGCTAGGGGAATAAATCTTTGGGCACCTAGTGGTCATG

4 CATGACCACTAGGAGCATCTTTGGCGAGATCGGGAGAATCGGTGGCATTGGTGTTCCCTAGGGGAATAAATCTTTGGGCACCTAGTGGTCATG

4 CATGACCACTAGGAGCATCTTTGGCGAGGTCGAGAGAATCGGTGGCATTGGTGTCTCCTAGGGGAATAAATCTTTGGGCACCTAGTGGTCATG

4 CATGACCACTAGGAGCATCTTTGGCGATTAGCTGAGCGGGAAACGGATAGTGGGGGCCTAGGGGAATAAATCTTTGGGCACCTAGTGGTCATG

4 CATGACCACTAGGAGCATCTTTGGCGAGAACGTGAGAATCGGTGGCATTGGTGTCTCCTAGGGGAATAAATCTTTGGGCACCTAGTGGTCATG

4 CATGACCACTAGGAGCATCTTTGGCGACGAGGGGCATGGGTGGTCATTGCGAGAAGACTAGGGGAATAAATCTTTGGGCACCTAGTGGTCATG

4 CATGACCACTAGGAGCATCTTTGGCGACTAGGGTTGGTCCATACCCGCGACGTAGTGCTAGGGGAATAAATCTTTGGGCACCTAGTGGTCATG

4 CATGACCACTAGGAGCATCTTTGGCGACGGTGTGGGGAGCTTGTTTCGGCGGTGCTACTAGGGGAATAAATCTTTGGGCACCTAGTGGTCATG

4 CATGACCACTAGGAGCATCTTTGGCGAGATCGCGAGAATCGGTGGCATTTGTGTCTCCTAGGGGAATAAATCTTTGGGCACCTAGTGGTCATG

4 CATGACCACTAGGAGCATCTTTGGCGATCCGGGGAGTGGGAACACTGACGCATAGGGCTAGGGGAATAAATCTTTGGGCACCTAGTGGTCATG

4 CATGACCACTAGGAGCATCTTTGGCGAGGACCGGTAAACTAGCATTAGCGTTGCGCTCTAGGGGAATAAATCTTTGGGCACCTAGTGGTCATG

4 CATGACCACTAGGAGCATCTTTGGCGAGGGCGGGCGCCGGCAGCGGTGTGAATGCGACTAGGGGAATAAATCTTTGGGCACCTAGTGGTCATG

4 CATGACCACTAGGAGCATCTTTGGCGAGATCGGGAGAATAGGTGGCATTGTTGTCTCCTAGGGGAATAAATCTTTGGGCACCTAGTGGTCATG

4 CATGACCACTAGGAGCATCTTTGGCGACGGTGTGGGGAACTTGTCTCGGCGGTGCTACTAGGGGAATAAATCTTTGGGCACCTAGTGGTCATG

4 CATGACCACTAGGAGCATCTTTGGCGACGGGGTGGGGCCTAAGTGCGGCGGCTGATTCTAGGGGAATAAATCTTTGGGCACCTAGTGGTCATG

4 CATGACCACTAGGAGCATCTTTGGCGAGTTTCGCTGTGCTTGGAATGGGGCCACCCTCTAGGGGAATAAATCTTTGGGCACCTAGTGGTCATG

4 CATGACCACTAGGAGCATCTTTGGCGACGGTGTGGGGTCTACTAGCACGGCGGTGATCTAGGGGAATAAATCTTTGGGCACCTAGTGGTCATG

4 CATGACCACTAGGAGCATCTTTGGCGAGATCGGGAGAATCGGTGGTATTGGCGTCTCCTAGGGGAATAAATCTTTGGGCACCTAGTGGTCATG

4 CATGACCACTAGGAGCATCTTTGGCGACCGGCGGAGACATGCATGCGCGTTGTGGAGCTAGGGGAATAAATCTTTGGGCACCTAGTGGTCATG

4 CATGACCACTAGGAGCATCTTTGGCGAGGAGTGGCTGAGGGCGGTGGCTAGGTCGCACTAGGGGAATAAATCTTTGGGCACCTAGTGGTCATG

4 CATGACCACTAGGAGCATCTTTGGCGAGAAGACCCTGGATTCGGGGACCAGTTGTTGCTAGGGGAATAAATCTTTGGGCACCTAGTGGTCATG

4 CATGACCACTAGGAGCATCTTTGGCGATTAGCTGAGCGGACGAGTCATAAAGTGGGCCTAGGGGAATAAATCTTTGGGCACCTAGTGGTCATG

4 CATGACCACTAGGAGCATCTTTGGCGAGATCGGGAGAATCGGTGGCATCGGTGCCTCCTAGGGGAATAAATCTTTGGGCACCTAGTGGTCATG

4 CATGACCACTAGGAGCATCTTTGGCGACGGGATCGTGTCGGCAGATTGCGTACTTGGCTAGGGGAATAAATCTTTGGGCACCTAGTGGTCATG

4 CATGACCACTAGGAGCATCTTTGGCGAGACTGGGAGAATCGGTGGCATTGGTGTCTCCTAGGGGAATAAATCTTTGGGCACCTAGTGGTCATG

4 CATGACCACTAGGAGCATCTTTGGCGAGATCGGCAGAATCGATGGCATTGGTGTCTCCTAGGGGAATAAATCTTTGGGCACCTAGTGGTCATG

4 CATGACCACTAGGAGCATCTTTGGCGAATGGCTGGCACGGCCTTCTAGTCCTTGGTACTAGGGGAATAAATCTTTGGGCACCTAGTGGTCATG

4 CATGACCACTAGGAGCATCTTTGGCGAGCCTCGCTAGGGAGGTTGCTCCACCAGTTCCTAGGGGAATAAATCTTTGGGCACCTAGTGGTCATG

4 CATGACCACTAGGAGCATCTTTGGCGAGGGTTGCGGGCTCCTCGGAGTGTGGGTGGCCTAGGGGAATAAATCTTTGGGCACCTAGTGGTCATG

4 CATGACCACTAGGAGCATCTTTGGCGACGGGATTCGGTAGGAAAGAGTGCACGCCGACTAGGGGAATAAATCTTTGGGCACCTAGTGGTCATG

4 CATGACCACTAGGAGCATCTTTGGCGAACCGTTAGGGGGGGAAATAGAGCTCGTAGACTAGGGGAATAAATCTTTGGGCACCTAGTGGTCATG

4 CATGACCACTAGGAGCATCTTTGGCGAATGGCCGGCACGGCCTTCTAATCCTCGGTACTAGGGGAATAAATCTTTGGGCACCTAGTGGTCATG

4 CATGACCACTAGGAGCATCTTTGGCGAGTGGTGGTAGGCCGCTAGAAGCCGTCAAGACTAGGGGAATAAATCTTTGGGCACCTAGTGGTCATG

4 CATGACCACTAGGAGCATCTTTGGCGAGGGAGGGCGCCGGCAGCGGTGTAAATGCGACTAGGGGAATAAATCTTTGGGCACCTAGTGGTCATG

4 CATGACCACTAGGAGCATCTTTGGCGATCCGGGGGCACCTACGTGCGACGCTGTGGGCTAGGGGAATAAATCTTTGGGCACCTAGTGGTCATG

4 CATGACCACTAGGAGCATCTTTGGCGAAGGAGGGCGCCGGCAGCGGTGTGAATGCGACTAGGGGAATAAATCTTTGGGCACCTAGTGGTCATG

4 CATGACCACTAGGAGCATCTTTGGCGAGATCTGGAGAATCGGTGGCATTGGAGTCTCCTAGGGGAATAAATCTTTGGGCACCTAGTGGTCATG

4 CATGACCACTAGGAGCATCTTTGGCGAATGGCCGGCACGGCCTTCTAGTCCTCGGCACTAGGGGAATAAATCTTTGGGCACCTAGTGGTCATG

4 CATGACCACTAGGAGCATCTTTGGCGAAAGTCGGTAGAACAGGATGGGGTGCTGTCCCTAGGGGAATAAATCTTTGGGCACCTAGTGGTCATG

4 CATGACCACTAGGAGCATCTTTGGCGAAAGTCGGTAGACCAGGGTGGGGTGCTGTCCCTAGGGGAATAAATCTTTGGGCACCTAGTGGTCATG

4 CATGACCACTAGGAGCATCTTTGGCGAGAAGACTCTGGATTGGGGGACCAGTTGCTGCTAGGGGAATAAATCTTTGGGCACCTAGTGGTCATG

4 CATGACCACTAGGAGCATCTTTGGCGAATGGCCGGCACGGCCTCCTAGTCCTTGGTACTAGGGGAATAAATCTTTGGGCACCTAGTGGTCATG

4 CATGACCACTAGGAGCATCTTTGGCGACTCGTGGGCTGGTAGGTGGTACTTGAGTCACTAGGGGAATAAATCTTTGGGCACCTAGTGGTCATG

4 CATGACCACTAGGAGCATCTTTGGCGAGGGAGGGCGCCGGCAGCGGTGTGGATGCGACTAGGGGAATAAATCTTTGGGCACCTAGTGGTCATG

4 CATGACCACTAGGAGCATCTTTGGCGAACGGCAGGTGTTGCGGTGGTTTGTGAATTCCTAGGGGAATAAATCTTTGGGCACCTAGTGGTCATG

4 CATGACCACTAGGAGCATCTTTGGCGAGATCGGGAGAATCGGCAGCATTGGTGTCTCCTAGGGGAATAAATCTTTGGGCACCTAGTGGTCATG

4 CATGACCACTAGGAGCATCTTTGGCGAGATCGGGAGAATCGGTGGCATAAGTGTCTCCTAGGGGAATAAATCTTTGGGCACCTAGTGGTCATG

4 CATGACCACTAGGAGCATCTTTGGCGACCAGTGAAGCTTAGGTAGTGCGACCAGTGACTAGGGGAATAAATCTTTGGGCACCTAGTGGTCATG

4 CATGACCACTAGGAGCATCTTTGGCGAACGGCAGGTGTTGCGGTCGTCTGTGAATCCCTAGGGGAATAAATCTTTGGGCACCTAGTGGTCATG

4 CATGACCACTAGGAGCATCTTTGGCGAGGGCGTGGGGCTATTGATGCGGCGGCACTCCTAGGGGAATAAATCTTTGGGCACCTAGTGGTCATG

4 CATGACCACTAGGAGCATCTTTGGCGAGATTGGGAGAATCAGTGGCATTGGTGTCTCCTAGGGGAATAAATCTTTGGGCACCTAGTGGTCATG

4 CATGACCACTAGGAGCATCTTTGGCGAATGGCCGGCACGGCCTACTAGTCCTCGGTACTAGGGGAATAAATCTTTGGGCACCTAGTGGTCATG

4 CATGACCACTAGGAGCATCTTTGGCGACCTCGGCTGGTAGGTAATGTATTGGGCGATCTAGGGGAATAAATCTTTGGGCACCTAGTGGTCATG

4 CATGACCACTAGGAGCATCTTTGGCGAGATCTGGAGAATCGGTGGTATTGGTGTCTCCTAGGGGAATAAATCTTTGGGCACCTAGTGGTCATG

4 CATGACCACTAGGAGCATCTTTGGCGAGATCGGTAGAATCGGTGGCATCGGTGTCTCCTAGGGGAATAAATCTTTGGGCACCTAGTGGTCATG

4 CATGACCACTAGGAGCATCTTTGGCGAGATCGGGATAATCGGCGGCATTGGTGTCTCCTAGGGGAATAAATCTTTGGGCACCTAGTGGTCATG

4 CATGACCACTAGGAGCATCTTTGGCGAAAGTCGGTAGAACAGGGTTGGGTGCTGTCCCTAGGGGAATAAATCTTTGGGCACCTAGTGGTCATG

4 CATGACCACTAGGAGCATCTTTGGCGAATGGCCGGCACGGCCTTCTAGTCCTAGGTACTAGGGGAATAAATCTTTGGGCACCTAGTGGTCATG

3 CATGACCACTAGGAGCATCTTTGGCGACGTCATAAAGTGCGTAGAGTGGTAAGTCGACTAGGGGAATAAATCTTTGGGCACCTAGTGGTCATG

3 CATGACCACTAGGAGCATCTTTGGCGAGATCGGGAGAATCGGTGGCATTTGTGTATCCTAGGGGAATAAATCTTTGGGCACCTAGTGGTCATG

3 CATGACCACTAGGAGCATCTTTGGCGATGGGGCCGTGCTGTCTGCACACTCCGCGGGCTAGGGGAATAAATCTTTGGGCACCTAGTGGTCATG

3 CATGACCACTAGGAGCATCTTTGGCGAGAAGACTCCGGATTCGGGTACCAGTTGCTGCTAGGGGAATAAATCTTTGGGCACCTAGTGGTCATG

3 CATGACCACTAGGAGCATCTTTGGCGAGATCGGGAGGATCGGTGGCATTGGTGTTTCCTAGGGGAATAAATCTTTGGGCACCTAGTGGTCATG

3 CATGACCACTAGGAGCATCTTTGGCGATCGCACAGCGAAGACGATGTACTGTCGGGACTAGGGGAATAAATCTTTGGGCACCTAGTGGTCATG

3 CATGACCACTAGGAGCATCTTTGGCGATCGCGGAGCGCACGACTAAAAGGTGGGGGCCTAGGGGAATAAATCTTTGGGCACCTAGTGGTCATG

3 CATGACCACTAGGAGCATCTTTGGCGATAGCCAGACAATTGTGGGTTGCGGAATGCTCTAGGGGAATAAATCTTTGGGCACCTAGTGGTCATG

3 CATGACCACTAGGAGCATCTTTGGCGAGAATACTCCGGATTCGGGGACCAGTTGCTGCTAGGGGAATAAATCTTTGGGCACCTAGTGGTCATG

3 CATGACCACTAGGAGCATCTTTGGCGAGGCGCAGGGTGTGAGAGGGGCGTGTCCATGCTAGGGGAATAAATCTTTGGGCACCTAGTGGTCATG

3 CATGACCACTAGGAGCATCTTTGGCGACCCAAGTGGGGAAATGTTCGGTGGAAGTGCCTAGGGGAATAAATCTTTGGGCACCTAGTGGTCATG

3 CATGACCACTAGGAGCATCTTTGGCGAGCCTTGCTTGGGAGGTAGCTCCACCAGTTCCTAGGGGAATAAATCTTTGGGCACCTAGTGGTCATG

3 CATGACCACTAGGAGCATCTTTGGCGAGATCGGGAGAAACGGTGGCAGTGGTGTCTCCTAGGGGAATAAATCTTTGGGCACCTAGTGGTCATG

3 CATGACCACTAGGAGCATCTTTGGCGAGATCGGGGGAATCGGTGGCATTGGTGTTTCCTAGGGGAATAAATCTTTGGGCACCTAGTGGTCATG

3 CATGACCACTAGGAGCATCTTTGGCGATCCTCGCTTGGGAGGTTGCTCCACCAGTTCCTAGGGGAATAAATCTTTGGGCACCTAGTGGTCATG

3 CATGACCACTAGGAGCATCTTTGGCGAGATCGGGAGAATCGGCGGCATTGATGTCTCCTAGGGGAATAAATCTTTGGGCACCTAGTGGTCATG

3 CATGACCACTAGGAGCATCTTTGGCGAGATCGTGAGAATCGGTGGCATTGGTGTCCCCTAGGGGAATAAATCTTTGGGCACCTAGTGGTCATG

3 CATGACCACTAGGAGCATCTTTGGCGAAAGTCGGTAGAACAGGGAGGGGTGCTGTCCCTAGGGGAATAAATCTTTGGGCACCTAGTGGTCATG

3 CATGACCACTAGGAGCATCTTTGGCGACTACTTCCGGGGGGTCGAGGCTTGCCTCCACTAGGGGAATAAATCTTTGGGCACCTAGTGGTCATG

3 CATGACCACTAGGAGCATCTTTGGCGAGATCGGGAGAATCGGTGGCATCGGTGTCTGCTAGGGGAATAAATCTTTGGGCACCTAGTGGTCATG

3 CATGACCACTAGGAGCATCTTTGGCGAGACCGCGAGAATCGGTGGCATTGGTGTCTCCTAGGGGAATAAATCTTTGGGCACCTAGTGGTCATG

3 CATGACCACTAGGAGCATCTTTGGCGAACGGTAGGTGTTGCGGTGGTCTTTGAATCCCTAGGGGAATAAATCTTTGGGCACCTAGTGGTCATG

3 CATGACCACTAGGAGCATCTTTGGCGAGAACGGGAGAATCGGTGGCATTGGTGTCACCTAGGGGAATAAATCTTTGGGCACCTAGTGGTCATG

3 CATGACCACTAGGAGCATCTTTGGCGAACGGCGGGTGTTGCGGTGGTCTGTGAATCTCTAGGGGAATAAATCTTTGGGCACCTAGTGGTCATG

3 CATGACCACTAGGAGCATCTTTGGCGAGGCAGGTGGTCATAATAACCAGGGTGGTAGCTAGGGGAATAAATCTTTGGGCACCTAGTGGTCATG

3 CATGACCACTAGGAGCATCTTTGGCGATCGCATAGCGAACAACAGTGGGGTTGGGGCCTAGGGGAATAAATCTTTGGGCACCTAGTGGTCATG

3 CATGACCACTAGGAGCATCTTTGGCGAGCCTCGCTTGGGAGATTGCTCCACCAGTTCCTAGGGGAATAAATCTTTGGGCACCTAGTGGTCATG

3 CATGACCACTAGGAGCATCTTTGGCGATTGGGCCGTGCTATCTGCACACTCCGCGGGCTAGGGGAATAAATCTTTGGGCACCTAGTGGTCATG

3 CATGACCACTAGGAGCATCTTTGGCGAGATCGGGAGAATCGGTGGCATCGGTGTCACCTAGGGGAATAAATCTTTGGGCACCTAGTGGTCATG

3 CATGACCACTAGGAGCATCTTTGGCGAACGGCAGGTGTTGCTGTGGTCTATGAATCCCTAGGGGAATAAATCTTTGGGCACCTAGTGGTCATG

3 CATGACCACTAGGAGCATCTTTGGCGAGATCGTGATAATCGGTGGCATTGGTGTCTCCTAGGGGAATAAATCTTTGGGCACCTAGTGGTCATG

3 CATGACCACTAGGAGCATCTTTGGCGAGCCTTGCTTGGGAGGTTGCTCCATTAGTTCCTAGGGGAATAAATCTTTGGGCACCTAGTGGTCATG

3 CATGACCACTAGGAGCATCTTTGGCGATCGCAGAGCGGACTTTGCATAGAAGTGGGCCTAGGGGAATAAATCTTTGGGCACCTAGTGGTCATG

3 CATGACCACTAGGAGCATCTTTGGCGAGATCGGGAGAATCGGTGGCATTGGTGCCTGCTAGGGGAATAAATCTTTGGGCACCTAGTGGTCATG

3 CATGACCACTAGGAGCATCTTTGGCGAGATCGGGAGAATCGACGGCATTGGTGTCTCCTAGGGGAATAAATCTTTGGGCACCTAGTGGTCATG

3 CATGACCACTAGGAGCATCTTTGGCGAGATCGGCAGAATCGGTGGCATTGGTGTCTGCTAGGGGAATAAATCTTTGGGCACCTAGTGGTCATG

3 CATGACCACTAGGAGCATCTTTGGCGATCCTCGTCAGATGGTGAAGCAGACTTTTGGCTAGGGGAATAAATCTTTGGGCACCTAGTGGTCATG

3 CATGACCACTAGGAGCATCTTTGGCGACTCCACTAGGGTTCCCTCGGATGGACCCAACTAGGGGAATAAATCTTTGGGCACCTAGTGGTCATG

3 CATGACCACTAGGAGCATCTTTGGCGACTGGGCTGGCAGGATGGTATTGTGTTCGCACTAGGGGAATAAATCTTTGGGCACCTAGTGGTCATG

3 CATGACCACTAGGAGCATCTTTGGCGAGAAGACTTTGGGTTCGGGGACCAGTTGCTGCTAGGGGAATAAATCTTTGGGCACCTAGTGGTCATG

3 CATGACCACTAGGAGCATCTTTGGCGAAAGTCGACAGAACAGGGTGGGGTGCTGTCCCTAGGGGAATAAATCTTTGGGCACCTAGTGGTCATG

3 CATGACCACTAGGAGCATCTTTGGCGAAAGGCGTGTACGGTCGAGCGAAAGCGATAGCTAGGGGAATAAATCTTTGGGCACCTAGTGGTCATG

3 CATGACCACTAGGAGCATCTTTGGCGATCGCAAAGCGGATTCCAGTGTGGAACGGGACTAGGGGAATAAATCTTTGGGCACCTAGTGGTCATG

3 CATGACCACTAGGAGCATCTTTGGCGAGAAGACTCCGGACTCGGGGACCAGTTGCTGCTAGGGGAATAAATCTTTGGGCACCTAGTGGTCATG

3 CATGACCACTAGGAGCATCTTTGGCGAGATCGGGAGAGTCGGTGGCAATGGTGTCTCCTAGGGGAATAAATCTTTGGGCACCTAGTGGTCATG

3 CATGACCACTAGGAGCATCTTTGGCGACGGTGTGGGGCCTAAGTGCGGCGGTTGATTCTAGGGGAATAAATCTTTGGGCACCTAGTGGTCATG

3 CATGACCACTAGGAGCATCTTTGGCGACGTAGTGGGCTGGTAGGGAGTACTTGTTGCCTAGGGGAATAAATCTTTGGGCACCTAGTGGTCATG

3 CATGACCACTAGGAGCATCTTTGGCGAGATCGGGAGAATCGGTGGCATTTGTGTCTTCTAGGGGAATAAATCTTTGGGCACCTAGTGGTCATG

3 CATGACCACTAGGAGCATCTTTGGCGAGATCGGAAGAATCGGTGGCATTGGTGTCCCCTAGGGGAATAAATCTTTGGGCACCTAGTGGTCATG

3 CATGACCACTAGGAGCATCTTTGGCGAGATTGGGAGAATCGGTGGCATTGATGTCTCCTAGGGGAATAAATCTTTGGGCACCTAGTGGTCATG

3 CATGACCACTAGGAGCATCTTTGGCGATAGCTTAAGCGTGTGGCTGGTGGTCCGAGTCTAGGGGAATAAATCTTTGGGCACCTAGTGGTCATG

3 CATGACCACTAGGAGCATCTTTGGCGAGGGGTCATGGGACTGTAATTGCGCTTAAGACTAGGGGAATAAATCTTTGGGCACCTAGTGGTCATG

3 CATGACCACTAGGAGCATCTTTGGCGATCAGATATGGTAGGTAATGTGCTGAGGAGGCTAGGGGAATAAATCTTTGGGCACCTAGTGGTCATG

3 CATGACCACTAGGAGCATCTTTGGCGAGAAGACTTTGTATTCGGGGACCAGTTGCTGCTAGGGGAATAAATCTTTGGGCACCTAGTGGTCATG

3 CATGACCACTAGGAGCATCTTTGGCGATATAGGGAGAATCGGTGGCATTGGTGTCTCCTAGGGGAATAAATCTTTGGGCACCTAGTGGTCATG

3 CATGACCACTAGGAGCATCTTTGGCGAGGACAAAGTACTGGGCATGTGGTCGAGCTCCTAGGGGAATAAATCTTTGGGCACCTAGTGGTCATG

3 CATGACCACTAGGAGCATCTTTGGCGAAGTGCGGCGTGCCGAGTCGTCCTGCGACTCCTAGGGGAATAAATCTTTGGGCACCTAGTGGTCATG

3 CATGACCACTAGGAGCATCTTTGGCGAGATCGGTAGAATCGGTGGCATTGGCGTCTCCTAGGGGAATAAATCTTTGGGCACCTAGTGGTCATG

3 CATGACCACTAGGAGCATCTTTGGCGAGCCTCGCTTGAGAGGTTGCTCCACCAGTTCCTAGGGGAATAAATCTTTGGGCACCTAGTGGTCATG

3 CATGACCACTAGGAGCATCTTTGGCGAAAATCGGTAGAACAGGGTGGGGTGCTGTCCCTAGGGGAATAAATCTTTGGGCACCTAGTGGTCATG

3 CATGACCACTAGGAGCATCTTTGGCGAGATCTGGAGAATCGGAGGCATTGGTGTCTCCTAGGGGAATAAATCTTTGGGCACCTAGTGGTCATG

3 CATGACCACTAGGAGCATCTTTGGCGACTGTGGCTGAGTGGGAGGGCGTGGCTGAGACTAGGGGAATAAATCTTTGGGCACCTAGTGGTCATG

3 CATGACCACTAGGAGCATCTTTGGCGAGATCTGGAGAAACGGTGGCATTGGTGTCTCCTAGGGGAATAAATCTTTGGGCACCTAGTGGTCATG

3 CATGACCACTAGGAGCATCTTTGGCGAGCCTTGCTTGGTAGGTTGCTCCACCAGTTCCTAGGGGAATAAATCTTTGGGCACCTAGTGGTCATG

3 CATGACCACTAGGAGCATCTTTGGCGAAACGACCTTGTCGTTGGGGACCAGTTGCTGCTAGGGGAATAAATCTTTGGGCACCTAGTGGTCATG

3 CATGACCACTAGGAGCATCTTTGGCGAGATCGTGAGAATCGGTGGCATTGTTGTCTCCTAGGGGAATAAATCTTTGGGCACCTAGTGGTCATG

3 CATGACCACTAGGAGCATCTTTGGCGAGGTCGGGAGAATCGGTGGCATTGGCGTCTCCTAGGGGAATAAATCTTTGGGCACCTAGTGGTCATG

3 CATGACCACTAGGAGCATCTTTGGCGAGAGACGGAGATGTGCAATGTGGTCGAGCATCTAGGGGAATAAATCTTTGGGCACCTAGTGGTCATG

3 CATGACCACTAGGAGCATCTTTGGCGATAAGTGGCATGGTTCTGCCGGTAGTCTCGACTAGGGGAATAAATCTTTGGGCACCTAGTGGTCATG

3 CATGACCACTAGGAGCATCTTTGGCGACTCGACTAAGCACAGTGATGTCGTAGTGGACTAGGGGAATAAATCTTTGGGCACCTAGTGGTCATG

3 CATGACCACTAGGAGCATCTTTGGCGAGAAGATTCTGGATTCGGGGACCAGTTGCTCCTAGGGGAATAAATCTTTGGGCACCTAGTGGTCATG

3 CATGACCACTAGGAGCATCTTTGGCGAGCCTCGCTTGGGAGGTTGCTCCACCGGTTCCTAGGGGAATAAATCTTTGGGCACCTAGTGGTCATG

3 CATGACCACTAGGAGCATCTTTGGCGAGATCGGGAGAATCGGTCGCATCGGTGTCTCCTAGGGGAATAAATCTTTGGGCACCTAGTGGTCATG

3 CATGACCACTAGGAGCATCTTTGGCGATCCGGGGGTGCGTGGGCACGACGTAACAGGCTAGGGGAATAAATCTTTGGGCACCTAGTGGTCATG

3 CATGACCACTAGGAGCATCTTTGGCGATCCGGGGGCACCTATGTGCGACGCTGTGAGCTAGGGGAATAAATCTTTGGGCACCTAGTGGTCATG

3 CATGACCACTAGGAGCATCTTTGGCGACGGAGATGTCTAAAGGCTGGGGAGTGTTGACTAGGGGAATAAATCTTTGGGCACCTAGTGGTCATG

3 CATGACCACTAGGAGCATCTTTGGCGATCAGGCCGCCAGGGCGTACGAAGTGTGGTACTAGGGGAATAAATCTTTGGGCACCTAGTGGTCATG

3 CATGACCACTAGGAGCATCTTTGGCGATCGCACAGCGGATGTGATGCAGCACAGGGACTAGGGGAATAAATCTTTGGGCACCTAGTGGTCATG

3 CATGACCACTAGGAGCATCTTTGGCGAGGGGTCGGTTGGAAGGGCGTATTCCTGAGGCTAGGGGAATAAATCTTTGGGCACCTAGTGGTCATG

3 CATGACCACTAGGAGCATCTTTGGCGAGATCGGGAGAGTCGGTGGCATTGGTGCCTCCTAGGGGAATAAATCTTTGGGCACCTAGTGGTCATG

3 CATGACCACTAGGAGCATCTTTGGCGACGGTGTGGGGCCATCTGTGCGGCGGATCTCCTAGGGGAATAAATCTTTGGGCACCTAGTGGTCATG

3 CATGACCACTAGGAGCATCTTTGGCGACCGGCAGGTGTTGCGGTGGTCTGTGAATCTCTAGGGGAATAAATCTTTGGGCACCTAGTGGTCATG

3 CATGACCACTAGGAGCATCTTTGGCGACTCGAGAAGTGGTCATAAACGTCCGGTGCTCTAGGGGAATAAATCTTTGGGCACCTAGTGGTCATG

3 CATGACCACTAGGAGCATCTTTGGCGATCCGGGGGTCTCTATAGACGGCGCGTAGGGCTAGGGGAATAAATCTTTGGGCACCTAGTGGTCATG

3 CATGACCACTAGGAGCATCTTTGGCGAGGTTGGTGCAGGCTCTGCCTGTCGTCGAGACTAGGGGAATAAATCTTTGGGCACCTAGTGGTCATG

3 CATGACCACTAGGAGCATCTTTGGCGAAGAATCGTCGCGCGCTTGCAGTAATCGCACCTAGGGGAATAAATCTTTGGGCACCTAGTGGTCATG

3 CATGACCACTAGGAGCATCTTTGGCGAGAGCCGTAGCCGTAAGGTTGGTATAAGGCCCTAGGGGAATAAATCTTTGGGCACCTAGTGGTCATG

3 CATGACCACTAGGAGCATCTTTGGCGAAAGTCGGTAGAACAGTGTGGGGTGCTGTCCCTAGGGGAATAAATCTTTGGGCACCTAGTGGTCATG

3 CATGACCACTAGGAGCATCTTTGGCGAGATCGGGATAATCGGTGGCATTGGTGTCTTCTAGGGGAATAAATCTTTGGGCACCTAGTGGTCATG

3 CATGACCACTAGGAGCATCTTTGGCGAATGTGCAAGATATGCCGCCTGGAGCGTAGGCTAGGGGAATAAATCTTTGGGCACCTAGTGGTCATG

3 CATGACCACTAGGAGCATCTTTGGCGATCCGGGGGCACCTATGTGCGACGCTGTGGTCTAGGGGAATAAATCTTTGGGCACCTAGTGGTCATG

3 CATGACCACTAGGAGCATCTTTGGCGAGGGAGGGCGCCGGCGGCGGTGTGAATGCGACTAGGGGAATAAATCTTTGGGCACCTAGTGGTCATG

3 CATGACCACTAGGAGCATCTTTGGCGATATCGGCGTAGCCAACCAATGCTAATGGTTCTAGGGGAATAAATCTTTGGGCACCTAGTGGTCATG

3 CATGACCACTAGGAGCATCTTTGGCGAACGCAGATCTGGCGTAGATCGGATTGGAACCTAGGGGAATAAATCTTTGGGCACCTAGTGGTCATG

3 CATGACCACTAGGAGCATCTTTGGCGACGACGTGGGCAAGTGTGGGTATGTCGGAGCCTAGGGGAATAAATCTTTGGGCACCTAGTGGTCATG

3 CATGACCACTAGGAGCATCTTTGGCGAGATCGGGAGAATCGGTGGCATTGTTGTATCCTAGGGGAATAAATCTTTGGGCACCTAGTGGTCATG

3 CATGACCACTAGGAGCATCTTTGGCGAGATCGGGCGACTCGGTGGCATTGGTGTCTCCTAGGGGAATAAATCTTTGGGCACCTAGTGGTCATG

3 CATGACCACTAGGAGCATCTTTGGCGAACGGCAGGTGTTGCGGTGGTTTGTGAGTCCCTAGGGGAATAAATCTTTGGGCACCTAGTGGTCATG

3 CATGACCACTAGGAGCATCTTTGGCGATAGCCGTAGCCGTAAGGTTGGTATAAGGTCCTAGGGGAATAAATCTTTGGGCACCTAGTGGTCATG

3 CATGACCACTAGGAGCATCTTTGGCGATCCGGGGGTCTTGTAAGGCGACGCTATGGGCTAGGGGAATAAATCTTTGGGCACCTAGTGGTCATG

3 CATGACCACTAGGAGCATCTTTGGCGAAAGTCGGTAAAACAGGGTGGGGTGCTGTCCCTAGGGGAATAAATCTTTGGGCACCTAGTGGTCATG

3 CATGACCACTAGGAGCATCTTTGGCGAACAGGAACTGGGGGGCGAAATGTTCATAGACTAGGGGAATAAATCTTTGGGCACCTAGTGGTCATG

3 CATGACCACTAGGAGCATCTTTGGCGAGGAGTGGCTGAGGGCGGTGGGTAGGTCGCACTAGGGGAATAAATCTTTGGGCACCTAGTGGTCATG

3 CATGACCACTAGGAGCATCTTTGGCGAGAAGACTCTGGATGCGGGGACCAGTTGCTGCTAGGGGAATAAATCTTTGGGCACCTAGTGGTCATG

3 CATGACCACTAGGAGCATCTTTGGCGAGATCGGGAGAATCGGTGGCAGTGTTGTCTCCTAGGGGAATAAATCTTTGGGCACCTAGTGGTCATG

3 CATGACCACTAGGAGCATCTTTGGCGAGATCGGGAGAATAGGTGGCAATGGTGTCTCCTAGGGGAATAAATCTTTGGGCACCTAGTGGTCATG

3 CATGACCACTAGGAGCATCTTTGGCGAGCAGACTCTGGATTCGGGGACCAGTTGCTGCTAGGGGAATAAATCTTTGGGCACCTAGTGGTCATG

3 CATGACCACTAGGAGCATCTTTGGCGAGGACCAAGGTGTGCATCCCGTACAGGCTGCCTAGGGGAATAAATCTTTGGGCACCTAGTGGTCATG

3 CATGACCACTAGGAGCATCTTTGGCGAGCCTTGCTTTGGAGGTTGCTCCACCAGTTCCTAGGGGAATAAATCTTTGGGCACCTAGTGGTCATG

3 CATGACCACTAGGAGCATCTTTGGCGAGCATTGCTTGGGAGGTTGCTCCACCAGTTCCTAGGGGAATAAATCTTTGGGCACCTAGTGGTCATG

3 CATGACCACTAGGAGCATCTTTGGCGAACGGCAGGTGTTGCGGTGGTCTACGAATCCCTAGGGGAATAAATCTTTGGGCACCTAGTGGTCATG

3 CATGACCACTAGGAGCATCTTTGGCGAGATCGCGAGAATCGGAGGCATTGGTGTCTCCTAGGGGAATAAATCTTTGGGCACCTAGTGGTCATG

3 CATGACCACTAGGAGCATCTTTGGCGACAATGTTAATGTGGTGTGATGCTTAGAATTCTAGGGGAATAAATCTTTGGGCACCTAGTGGTCATG

3 CATGACCACTAGGAGCATCTTTGGCGACGGGGTGGGGCCTAAGTGCGGCGGTTGATTCTAGGGGAATAAATCTTTGGGCACCTAGTGGTCATG

3 CATGACCACTAGGAGCATCTTTGGCGAAGCGGAGGGCGCCTGTGGTCGCTGTGTGGTCTAGGGGAATAAATCTTTGGGCACCTAGTGGTCATG

3 CATGACCACTAGGAGCATCTTTGGCGATATCGGGAGAATCGGTGGCATTGGTGTCCCCTAGGGGAATAAATCTTTGGGCACCTAGTGGTCATG

3 CATGACCACTAGGAGCATCTTTGGCGAGAAGACTCTGGGTTCGGGGACCAGTTGCTCCTAGGGGAATAAATCTTTGGGCACCTAGTGGTCATG

3 CATGACCACTAGGAGCATCTTTGGCGAGGAAGATGGTAGGGTAATGTGTCGGCGCCGCTAGGGGAATAAATCTTTGGGCACCTAGTGGTCATG

3 CATGACCACTAGGAGCATCTTTGGCGATAGGCGTGTACGGTCGAGCGAAGGCGATAGCTAGGGGAATAAATCTTTGGGCACCTAGTGGTCATG

3 CATGACCACTAGGAGCATCTTTGGCGAGAACGAGAGAATCGGTGGCATTGGTGTCTCCTAGGGGAATAAATCTTTGGGCACCTAGTGGTCATG

3 CATGACCACTAGGAGCATCTTTGGCGAGAAGACTCTGGATTCGAGGACCAGTTGCTCCTAGGGGAATAAATCTTTGGGCACCTAGTGGTCATG

3 CATGACCACTAGGAGCATCTTTGGCGACTAGGCGTCAGCACCGGGTGGGCTCGGTCCCTAGGGGAATAAATCTTTGGGCACCTAGTGGTCATG

3 CATGACCACTAGGAGCATCTTTGGCGAGATCGGGAGAATCGGTGGCAATGGTGTCTTCTAGGGGAATAAATCTTTGGGCACCTAGTGGTCATG

3 CATGACCACTAGGAGCATCTTTGGCGATCGCACAGCGCACGAAGCCACATGAGGGGACTAGGGGAATAAATCTTTGGGCACCTAGTGGTCATG

3 CATGACCACTAGGAGCATCTTTGGCGACCCTGGCGCGGGGATCCTGGTTGCGGCGACCTAGGGGAATAAATCTTTGGGCACCTAGTGGTCATG

3 CATGACCACTAGGAGCATCTTTGGCGAGATCGGGAGAATAGGCGGCATTGGTGTCTCCTAGGGGAATAAATCTTTGGGCACCTAGTGGTCATG

3 CATGACCACTAGGAGCATCTTTGGCGAGGGAGGGCGCCGGCAGCGGTGTGAGTGCGCCTAGGGGAATAAATCTTTGGGCACCTAGTGGTCATG

3 CATGACCACTAGGAGCATCTTTGGCGATCCGGGGGGTCAACGACCGACGCCTCTGGGCTAGGGGAATAAATCTTTGGGCACCTAGTGGTCATG

3 CATGACCACTAGGAGCATCTTTGGCGAGCCTTGTTTGGGAGGTTGCTCCACCAGTTCCTAGGGGAATAAATCTTTGGGCACCTAGTGGTCATG

3 CATGACCACTAGGAGCATCTTTGGCGAAAGTCGGTAGAACAGGGTGGGGTGCTGACCCTAGGGGAATAAATCTTTGGGCACCTAGTGGTCATG

3 CATGACCACTAGGAGCATCTTTGGCGACACGGCCCAAGGACGCCGTGGATGGTGCGCCTAGGGGAATAAATCTTTGGGCACCTAGTGGTCATG

3 CATGACCACTAGGAGCATCTTTGGCGACGTGGGCAGTTTAGAAGATCGCCTAGTACGCTAGGGGAATAAATCTTTGGGCACCTAGTGGTCATG

3 CATGACCACTAGGAGCATCTTTGGCGAGATCGGGATAATCGGTGGAATTGGTGTCTCCTAGGGGAATAAATCTTTGGGCACCTAGTGGTCATG

3 CATGACCACTAGGAGCATCTTTGGCGAGATCGGGAGAAAAGGTGGCATTGGTGTCTCCTAGGGGAATAAATCTTTGGGCACCTAGTGGTCATG

3 CATGACCACTAGGAGCATCTTTGGCGAGATCGGGAGAATCGGCGCCATTGGTGTCTCCTAGGGGAATAAATCTTTGGGCACCTAGTGGTCATG

3 CATGACCACTAGGAGCATCTTTGGCGAGATCGGGAGAATCGGTGTAATTGGTGTCTCCTAGGGGAATAAATCTTTGGGCACCTAGTGGTCATG

3 CATGACCACTAGGAGCATCTTTGGCGATCCGGGGGTCACGAGTGACGGCGCATAGGGCTAGGGGAATAAATCTTTGGGCACCTAGTGGTCATG

3 CATGACCACTAGGAGCATCTTTGGCGAGAAGACGCTGGATTCGGGGACCAGTTGCTCCTAGGGGAATAAATCTTTGGGCACCTAGTGGTCATG

3 CATGACCACTAGGAGCATCTTTGGCGAACGGCATGTGTTGCGGTGGTCTGTGGATCCCTAGGGGAATAAATCTTTGGGCACCTAGTGGTCATG

3 CATGACCACTAGGAGCATCTTTGGCGACAGGGGATGCCGATGCGCTGGACGATCGAGCTAGGGGAATAAATCTTTGGGCACCTAGTGGTCATG

3 CATGACCACTAGGAGCATCTTTGGCGAGATCGTGAGAATCGGTAGCATTGGTGTCTCCTAGGGGAATAAATCTTTGGGCACCTAGTGGTCATG

3 CATGACCACTAGGAGCATCTTTGGCGACGGCGTGGGGCCATCAGTGCGGCGGATCCCCTAGGGGAATAAATCTTTGGGCACCTAGTGGTCATG

3 CATGACCACTAGGAGCATCTTTGGCGAATGGCCGGCACGGACTTCTAGTCCTCGGTACTAGGGGAATAAATCTTTGGGCACCTAGTGGTCATG

3 CATGACCACTAGGAGCATCTTTGGCGAAAGTCCGTAGAACAGGGTGGGGTGCTGTCCCTAGGGGAATAAATCTTTGGGCACCTAGTGGTCATG

3 CATGACCACTAGGAGCATCTTTGGCGAGATCGGGAGAATTGGTGGCATTGGTGACTCCTAGGGGAATAAATCTTTGGGCACCTAGTGGTCATG

3 CATGACCACTAGGAGCATCTTTGGCGACTCATGTGGTCCCGTACCACAGGTAGTGGACTAGGGGAATAAATCTTTGGGCACCTAGTGGTCATG

3 CATGACCACTAGGAGCATCTTTGGCGAGAAGACTCTGGATTCGGGGACCAGTTGCCCCTAGGGGAATAAATCTTTGGGCACCTAGTGGTCATG

3 CATGACCACTAGGAGCATCTTTGGCGAAAGTCGGTAGAACAGGGTGGGGAGCTGTCCCTAGGGGAATAAATCTTTGGGCACCTAGTGGTCATG

3 CATGACCACTAGGAGCATCTTTGGCGAACGGCAGGTGTTGCGGTGGTTTGTGGATCCCTAGGGGAATAAATCTTTGGGCACCTAGTGGTCATG

3 CATGACCACTAGGAGCATCTTTGGCGACGAGTCCTGGACTTGTCCCTGAGGGGAGATCTAGGGGAATAAATCTTTGGGCACCTAGTGGTCATG

3 CATGACCACTAGGAGCATCTTTGGCGATACGTCGTGTGGTGGTTCAAGGTGAGCTAGCTAGGGGAATAAATCTTTGGGCACCTAGTGGTCATG

3 CATGACCACTAGGAGCATCTTTGGCGAGCCTCGCTTGGGAGTTTGCTCCACCAGTTCCTAGGGGAATAAATCTTTGGGCACCTAGTGGTCATG

3 CATGACCACTAGGAGCATCTTTGGCGAGATCGGGAGAATCGGCGGCATTGGTGTCACCTAGGGGAATAAATCTTTGGGCACCTAGTGGTCATG

3 CATGACCACTAGGAGCATCTTTGGCGACGGGTGCTGACGGCCGCCGCTGCGGCTACACTAGGGGAATAAATCTTTGGGCACCTAGTGGTCATG

3 CATGACCACTAGGAGCATCTTTGGCGAGATCGTGAGTATCGGTGGCATTGGTGTCTCCTAGGGGAATAAATCTTTGGGCACCTAGTGGTCATG

3 CATGACCACTAGGAGCATCTTTGGCGAGATCGGGAGAATTGGTAGCATTGGTGTCTCCTAGGGGAATAAATCTTTGGGCACCTAGTGGTCATG

3 CATGACCACTAGGAGCATCTTTGGCGATATCGGTAGAATCGGTGGCATTGGTGTCTCCTAGGGGAATAAATCTTTGGGCACCTAGTGGTCATG

3 CATGACCACTAGGAGCATCTTTGGCGACGTTATGAGTTGGGAAGCTTGGCGGCCAGACTAGGGGAATAAATCTTTGGGCACCTAGTGGTCATG

3 CATGACCACTAGGAGCATCTTTGGCGAGATAGTGAGAATCGGTGGCATTGGTGTCTCCTAGGGGAATAAATCTTTGGGCACCTAGTGGTCATG

3 CATGACCACTAGGAGCATCTTTGGCGAGATCCCCAGAATCGGTGGCATTGGTGTCTCCTAGGGGAATAAATCTTTGGGCACCTAGTGGTCATG

3 CATGACCACTAGGAGCATCTTTGGCGAGATCGGGAGAATCTGAGGCATTGGTGTCTCCTAGGGGAATAAATCTTTGGGCACCTAGTGGTCATG

3 CATGACCACTAGGAGCATCTTTGGCGAGGGATGGCGCCGGCAGCGGTGTGAATGCGACTAGGGGAATAAATCTTTGGGCACCTAGTGGTCATG

3 CATGACCACTAGGAGCATCTTTGGCGAAAGTCGGTAGAACAGGGTGGGATGCTGTCCCTAGGGGAATAAATCTTTGGGCACCTAGTGGTCATG

3 CATGACCACTAGGAGCATCTTTGGCGATATTGGGAGAATCGGTGGCATTGGTGTCTCCTAGGGGAATAAATCTTTGGGCACCTAGTGGTCATG

3 CATGACCACTAGGAGCATCTTTGGCGAGTGGTCGGGTTGGGAGGGCGTGGCTATCGGCTAGGGGAATAAATCTTTGGGCACCTAGTGGTCATG

3 CATGACCACTAGGAGCATCTTTGGCGATCAGGGGGCTTTTGATGGCGACTCAGTGGGCTAGGGGAATAAATCTTTGGGCACCTAGTGGTCATG

3 CATGACCACTAGGAGCATCTTTGGCGAGCCTTGCTTGGGAGGTTACTCCACCAGTTCCTAGGGGAATAAATCTTTGGGCACCTAGTGGTCATG

3 CATGACCACTAGGAGCATCTTTGGCGACTAAGTGGGCTGGGAGGAAGTATTTCGGAGCTAGGGGAATAAATCTTTGGGCACCTAGTGGTCATG

3 CATGACCACTAGGAGCATCTTTGGCGATGGGTGCTGACGGCCGCGGCTGCGGCTACACTAGGGGAATAAATCTTTGGGCACCTAGTGGTCATG

3 CATGACCACTAGGAGCATCTTTGGCGAGGGAGGGCGCCGGCAGCGGTGCGAATGCGACTAGGGGAATAAATCTTTGGGCACCTAGTGGTCATG

3 CATGACCACTAGGAGCATCTTTGGCGAGATAGGGAGAATCGGAGGCATTGGTGTCTCCTAGGGGAATAAATCTTTGGGCACCTAGTGGTCATG

3 CATGACCACTAGGAGCATCTTTGGCGAAGCGCAGGGTGTGAGAGGGGCGTGTCTATGCTAGGGGAATAAATCTTTGGGCACCTAGTGGTCATG

3 CATGACCACTAGGAGCATCTTTGGCGACCGGTGCAGACTTAGAGGCGACTAGTGAAGCTAGGGGAATAAATCTTTGGGCACCTAGTGGTCATG

3 CATGACCACTAGGAGCATCTTTGGCGATCCGGGGGTGCAGGGGGCACGACGCGTGGGCTAGGGGAATAAATCTTTGGGCACCTAGTGGTCATG

3 CATGACCACTAGGAGCATCTTTGGCGAGATTGGGGGAATCGGTGGCATTGGTGTCTCCTAGGGGAATAAATCTTTGGGCACCTAGTGGTCATG

3 CATGACCACTAGGAGCATCTTTGGCGAACAGGCCGTGGCTGCGGGTGGGTGCGCACCCTAGGGGAATAAATCTTTGGGCACCTAGTGGTCATG

3 CATGACCACTAGGAGCATCTTTGGCGAGATCGGGAGAATTTGTGGCATTGGTGTCTCCTAGGGGAATAAATCTTTGGGCACCTAGTGGTCATG

3 CATGACCACTAGGAGCATCTTTGGCGAGATCGGGATAATTGGTGGCATTGGTGTCTCCTAGGGGAATAAATCTTTGGGCACCTAGTGGTCATG

3 CATGACCACTAGGAGCATCTTTGGCGAGTGGGGGGCAACGGCCGGTAGTGGGTGAGACTAGGGGAATAAATCTTTGGGCACCTAGTGGTCATG

3 CATGACCACTAGGAGCATCTTTGGCGAGAGCGGGAGAATCGGCGGCATTGGTGTCTCCTAGGGGAATAAATCTTTGGGCACCTAGTGGTCATG

3 CATGACCACTAGGAGCATCTTTGGCGAGATCGGGGGAGTCGGTGGCATTGGTGTCTCCTAGGGGAATAAATCTTTGGGCACCTAGTGGTCATG

3 CATGACCACTAGGAGCATCTTTGGCGATCGCAGAGCGTAAGTTGGTTGAGACTGGGCCTAGGGGAATAAATCTTTGGGCACCTAGTGGTCATG

3 CATGACCACTAGGAGCATCTTTGGCGATCCGGGGGCCTACGAAGGGCGACGCTTGGACTAGGGGAATAAATCTTTGGGCACCTAGTGGTCATG

3 CATGACCACTAGGAGCATCTTTGGCGAGCCATGCTTGGGAGGTTGCTCCACCAGTTCCTAGGGGAATAAATCTTTGGGCACCTAGTGGTCATG

3 CATGACCACTAGGAGCATCTTTGGCGACGGGATCGTGTCGGCAGATAGCGTACTTGGCTAGGGGAATAAATCTTTGGGCACCTAGTGGTCATG

3 CATGACCACTAGGAGCATCTTTGGCGAGCCTCTCTTGGGAGGTTGCTCCACCAGTTCCTAGGGGAATAAATCTTTGGGCACCTAGTGGTCATG

3 CATGACCACTAGGAGCATCTTTGGCGAAAGTCGGTAGAACAGGGTGGGGTGCTTTCCCTAGGGGAATAAATCTTTGGGCACCTAGTGGTCATG

3 CATGACCACTAGGAGCATCTTTGGCGAGATCGGGAGAGTCGGTGGCGTTGGTGTCTCCTAGGGGAATAAATCTTTGGGCACCTAGTGGTCATG

3 CATGACCACTAGGAGCATCTTTGGCGAATGGCCGGCTCGGCCTTCTAGTCCTCGGTACTAGGGGAATAAATCTTTGGGCACCTAGTGGTCATG

3 CATGACCACTAGGAGCATCTTTGGCGAGATCGGTAGAATCGGTGGCATAGGTGTCTCCTAGGGGAATAAATCTTTGGGCACCTAGTGGTCATG

3 CATGACCACTAGGAGCATCTTTGGCGAGGGAGTGCGCCGGCAGCGGTGTGAATGCGACTAGGGGAATAAATCTTTGGGCACCTAGTGGTCATG

3 CATGACCACTAGGAGCATCTTTGGCGATCCGGGGGGTCTGCGATCCGACGCTGTGGGCTAGGGGAATAAATCTTTGGGCACCTAGTGGTCATG

3 CATGACCACTAGGAGCATCTTTGGCGAGCTCTACGCGTCTTTGAGTGCCGACGTACACTAGGGGAATAAATCTTTGGGCACCTAGTGGTCATG

3 CATGACCACTAGGAGCATCTTTGGCGACAAGCTGGTATTACCAGTCCTGAAGTGGACCTAGGGGAATAAATCTTTGGGCACCTAGTGGTCATG

3 CATGACCACTAGGAGCATCTTTGGCGAGCCTCGCTTGGGAGGTTGATCCACCAGTTCCTAGGGGAATAAATCTTTGGGCACCTAGTGGTCATG

3 CATGACCACTAGGAGCATCTTTGGCGAACGGCGGGTGTTGCGGTGGTCTGTGAAACCCTAGGGGAATAAATCTTTGGGCACCTAGTGGTCATG

3 CATGACCACTAGGAGCATCTTTGGCGAGGGAGGGCGCCTGCAGCGGTGTGAATGCGACTAGGGGAATAAATCTTTGGGCACCTAGTGGTCATG

3 CATGACCACTAGGAGCATCTTTGGCGAGATCGGGAGAATCGGTGGCATTGGTGTCAACTAGGGGAATAAATCTTTGGGCACCTAGTGGTCATG

3 CATGACCACTAGGAGCATCTTTGGCGACGGGAATATTCATCGGTGATTCGGCGAGATCTAGGGGAATAAATCTTTGGGCACCTAGTGGTCATG

3 CATGACCACTAGGAGCATCTTTGGCGAGCACACAGTTGCTTCGAAGTAATGGGAAGGCTAGGGGAATAAATCTTTGGGCACCTAGTGGTCATG

3 CATGACCACTAGGAGCATCTTTGGCGAAAGTCGGTAGAACAGGGTGGGGTTCTGTCCCTAGGGGAATAAATCTTTGGGCACCTAGTGGTCATG

3 CATGACCACTAGGAGCATCTTTGGCGAAGGGCGAACGATCTAGATCTGAGGAAATGTCTAGGGGAATAAATCTTTGGGCACCTAGTGGTCATG

3 CATGACCACTAGGAGCATCTTTGGCGAGATCGGTAGAATCGGTGGCATTGGTTTCTCCTAGGGGAATAAATCTTTGGGCACCTAGTGGTCATG

3 CATGACCACTAGGAGCATCTTTGGCGATCCGGGGGTCTGGACAGACGACGCTAGGGTCTAGGGGAATAAATCTTTGGGCACCTAGTGGTCATG

3 CATGACCACTAGGAGCATCTTTGGCGATCCGGGGGCCGAAGGCGACGCAAATTAGGGCTAGGGGAATAAATCTTTGGGCACCTAGTGGTCATG

3 CATGACCACTAGGAGCATCTTTGGCGACAGGTTCGTTAGGCGGTGGCCGCGGGAGATCTAGGGGAATAAATCTTTGGGCACCTAGTGGTCATG

3 CATGACCACTAGGAGCATCTTTGGCGAATGGTGTAGGACTTCAAGTGGATCTCATAGCTAGGGGAATAAATCTTTGGGCACCTAGTGGTCATG

3 CATGACCACTAGGAGCATCTTTGGCGAGACCGGGAGAATCGGTGGCATTGGTGTTTCCTAGGGGAATAAATCTTTGGGCACCTAGTGGTCATG

3 CATGACCACTAGGAGCATCTTTGGCGAGATCGCGAGAATCGGTGGCATCGGTGTCTCCTAGGGGAATAAATCTTTGGGCACCTAGTGGTCATG

3 CATGACCACTAGGAGCATCTTTGGCGATCCGGGGGCACCTATGTGCGACGCGACGGGCTAGGGGAATAAATCTTTGGGCACCTAGTGGTCATG

3 CATGACCACTAGGAGCATCTTTGGCGATCCGGGGGCACCTATGTGCGACACTGTGGGCTAGGGGAATAAATCTTTGGGCACCTAGTGGTCATG

3 CATGACCACTAGGAGCATCTTTGGCGACGGCGTGGGGCTATTGGTGCGGCGGCACTCCTAGGGGAATAAATCTTTGGGCACCTAGTGGTCATG

3 CATGACCACTAGGAGCATCTTTGGCGAGATCGGGAGAATCGGAGGCATTGGTGTCTACTAGGGGAATAAATCTTTGGGCACCTAGTGGTCATG

3 CATGACCACTAGGAGCATCTTTGGCGAAAGACGGTTCGGTGCGTAAGATGTTTGAGACTAGGGGAATAAATCTTTGGGCACCTAGTGGTCATG

3 CATGACCACTAGGAGCATCTTTGGCGAATGGCCGGCACGGCCTTCTAGTCCTCGGTCCTAGGGGAATAAATCTTTGGGCACCTAGTGGTCATG

3 CATGACCACTAGGAGCATCTTTGGCGATCGCAAGGCGAAGATACAGAGGATCCGGGCCTAGGGGAATAAATCTTTGGGCACCTAGTGGTCATG

3 CATGACCACTAGGAGCATCTTTGGCGATCCGGGGGGCGTGCAGCCGACGCGAAAGGGCTAGGGGAATAAATCTTTGGGCACCTAGTGGTCATG

3 CATGACCACTAGGAGCATCTTTGGCGAGATCCGAAGAATCGGTGGCATTGGTGTCTCCTAGGGGAATAAATCTTTGGGCACCTAGTGGTCATG

3 CATGACCACTAGGAGCATCTTTGGCGAGCCTCGCTTGGGAGGTTGCTCAACCAGTTCCTAGGGGAATAAATCTTTGGGCACCTAGTGGTCATG

3 CATGACCACTAGGAGCATCTTTGGCGAGATCGGGAGAATCGGAGGCAATGGTGTCTCCTAGGGGAATAAATCTTTGGGCACCTAGTGGTCATG

3 CATGACCACTAGGAGCATCTTTGGCGAGAACGGGATAATCGGTGGCATTGGTGTCTCCTAGGGGAATAAATCTTTGGGCACCTAGTGGTCATG

3 CATGACCACTAGGAGCATCTTTGGCGAGATCGGGAAAATTGGTGGCATTGGTGTCTCCTAGGGGAATAAATCTTTGGGCACCTAGTGGTCATG

3 CATGACCACTAGGAGCATCTTTGGCGAGATCGTGAGAATCGGTGGCATTGGGGTCTCCTAGGGGAATAAATCTTTGGGCACCTAGTGGTCATG

3 CATGACCACTAGGAGCATCTTTGGCGAGAACGGGAGAATCGGAGGCATTGGTGTCTCCTAGGGGAATAAATCTTTGGGCACCTAGTGGTCATG

3 CATGACCACTAGGAGCATCTTTGGCGATCCGGGGGCACCTATGTGCGACGCGGTGGGCTAGGGGAATAAATCTTTGGGCACCTAGTGGTCATG

3 CATGACCACTAGGAGCATCTTTGGCGACGGTGTGGGGAATTTGTTTCGGCGGTGCTACTAGGGGAATAAATCTTTGGGCACCTAGTGGTCATG

3 CATGACCACTAGGAGCATCTTTGGCGAGATCGAGAGAATCGGTAGCATTGGTGTCTCCTAGGGGAATAAATCTTTGGGCACCTAGTGGTCATG

3 CATGACCACTAGGAGCATCTTTGGCGATCGCAGTGCGGACGATTAAAACAGAGGGGCCTAGGGGAATAAATCTTTGGGCACCTAGTGGTCATG

3 CATGACCACTAGGAGCATCTTTGGCGAGATCGGGAGAATCGGAGGCATTGTTGTCTCCTAGGGGAATAAATCTTTGGGCACCTAGTGGTCATG

3 CATGACCACTAGGAGCATCTTTGGCGAAAGTCGGTAGAACAGGGTGGGGGGCTGTCCCTAGGGGAATAAATCTTTGGGCACCTAGTGGTCATG

3 CATGACCACTAGGAGCATCTTTGGCGAATGGCCGGCACCGCCTTCTAGTCCTCGGTACTAGGGGAATAAATCTTTGGGCACCTAGTGGTCATG

3 CATGACCACTAGGAGCATCTTTGGCGAGAATACTCTGGATTCGGGGACTAGTTGCTGCTAGGGGAATAAATCTTTGGGCACCTAGTGGTCATG

3 CATGACCACTAGGAGCATCTTTGGCGATCGCATAGCGTACTAGTGTAAAGGAGGGGCCTAGGGGAATAAATCTTTGGGCACCTAGTGGTCATG

3 CATGACCACTAGGAGCATCTTTGGCGAGATCGGGATAATCGGTGGCATTTGTGTCTCCTAGGGGAATAAATCTTTGGGCACCTAGTGGTCATG

3 CATGACCACTAGGAGCATCTTTGGCGATGGGTTGCTGGTAGGTGGTACGACTTCGATCTAGGGGAATAAATCTTTGGGCACCTAGTGGTCATG

3 CATGACCACTAGGAGCATCTTTGGCGACCGACAAATGGAAATGCGACGTAGTGGACGCTAGGGGAATAAATCTTTGGGCACCTAGTGGTCATG

3 CATGACCACTAGGAGCATCTTTGGCGACGGTGTGGGGCTATCTGTGCGGCGGATCCCCTAGGGGAATAAATCTTTGGGCACCTAGTGGTCATG

3 CATGACCACTAGGAGCATCTTTGGCGAGTGGGGGGTGACGGCCGGTAGTGGGTGAGACTAGGGGAATAAATCTTTGGGCACCTAGTGGTCATG

3 CATGACCACTAGGAGCATCTTTGGCGACGATCGAAGGTGCATGTAGTGGACGAGCCTCTAGGGGAATAAATCTTTGGGCACCTAGTGGTCATG

3 CATGACCACTAGGAGCATCTTTGGCGACGTGGTGTGTTGCCCATGTGGCTGAGTTGACTAGGGGAATAAATCTTTGGGCACCTAGTGGTCATG

3 CATGACCACTAGGAGCATCTTTGGCGACTCCAGTTTATCTGTCGGGCAGTTTAAGGACTAGGGGAATAAATCTTTGGGCACCTAGTGGTCATG

3 CATGACCACTAGGAGCATCTTTGGCGATCAGGGGGCTCAGTGGAGCGACTCCTAGGGCTAGGGGAATAAATCTTTGGGCACCTAGTGGTCATG

3 CATGACCACTAGGAGCATCTTTGGCGAGACCAGGAGAATCGGTGGCATTGGTGTCTCCTAGGGGAATAAATCTTTGGGCACCTAGTGGTCATG

3 CATGACCACTAGGAGCATCTTTGGCGACGAGGGGGCATTGGGAAATGTAAACGTCGACTAGGGGAATAAATCTTTGGGCACCTAGTGGTCATG

3 CATGACCACTAGGAGCATCTTTGGCGACTTCAGTTCATCTGTCGGGCAGTTTAAGGACTAGGGGAATAAATCTTTGGGCACCTAGTGGTCATG

3 CATGACCACTAGGAGCATCTTTGGCGACGGTGGTACGGGGGATGGGGAGTGTTGCCTCTAGGGGAATAAATCTTTGGGCACCTAGTGGTCATG

3 CATGACCACTAGGAGCATCTTTGGCGAGATCGGCAGAATCGGCGGCATTGGTGTCTCCTAGGGGAATAAATCTTTGGGCACCTAGTGGTCATG

3 CATGACCACTAGGAGCATCTTTGGCGATAGCGAAGTGCTGTGAATAGTGGTTAGCACCTAGGGGAATAAATCTTTGGGCACCTAGTGGTCATG

3 CATGACCACTAGGAGCATCTTTGGCGACAGGGGATGCCGATGCGCTAGACGACCGAGCTAGGGGAATAAATCTTTGGGCACCTAGTGGTCATG

3 CATGACCACTAGGAGCATCTTTGGCGAGATCGGGAGAATCGGTGGCAATGGTGTCTACTAGGGGAATAAATCTTTGGGCACCTAGTGGTCATG

3 CATGACCACTAGGAGCATCTTTGGCGAATCGATTATATCATTACCCTACGGTCCCGCCTAGGGGAATAAATCTTTGGGCACCTAGTGGTCATG

3 CATGACCACTAGGAGCATCTTTGGCGAGATCGTGAGAATCGGTGGCAGTGGTGTCTCCTAGGGGAATAAATCTTTGGGCACCTAGTGGTCATG

3 CATGACCACTAGGAGCATCTTTGGCGACCGCAGGAAAACAAAAGTGGCCTTGCTAGCCTAGGGGAATAAATCTTTGGGCACCTAGTGGTCATG

3 CATGACCACTAGGAGCATCTTTGGCGAGATCGGTAGAATCGGTTGCATTGGTGTCTCCTAGGGGAATAAATCTTTGGGCACCTAGTGGTCATG

3 CATGACCACTAGGAGCATCTTTGGCGATTAAGTGGTCAACGCGTTAAGTGGGGTAGACTAGGGGAATAAATCTTTGGGCACCTAGTGGTCATG

3 CATGACCACTAGGAGCATCTTTGGCGATGGGTGCTGACGGCCGCCGCTGCGGTTACACTAGGGGAATAAATCTTTGGGCACCTAGTGGTCATG

3 CATGACCACTAGGAGCATCTTTGGCGAGGGAGGGCGCCGGCAGTGGTGTGAATGCGCCTAGGGGAATAAATCTTTGGGCACCTAGTGGTCATG

3 CATGACCACTAGGAGCATCTTTGGCGAGATTGGGAGAATCGGTGCCATTGGTGTCTCCTAGGGGAATAAATCTTTGGGCACCTAGTGGTCATG

3 CATGACCACTAGGAGCATCTTTGGCGAACGGCAGGTGTTGCGGTGGTCTGTGAAACTCTAGGGGAATAAATCTTTGGGCACCTAGTGGTCATG

3 CATGACCACTAGGAGCATCTTTGGCGAACGGCAAGTGTTGCGGTGGTCTGTGGATCCCTAGGGGAATAAATCTTTGGGCACCTAGTGGTCATG

3 CATGACCACTAGGAGCATCTTTGGCGATCGCATAGCGCACGAAGCCATATGAGGGGACTAGGGGAATAAATCTTTGGGCACCTAGTGGTCATG

3 CATGACCACTAGGAGCATCTTTGGCGAGTGGTGGTCGGACGGAAAGCATACACGAGACTAGGGGAATAAATCTTTGGGCACCTAGTGGTCATG

3 CATGACCACTAGGAGCATCTTTGGCGAGATCGGGAGAAACGGTGGAATTGGTGTCTCCTAGGGGAATAAATCTTTGGGCACCTAGTGGTCATG

3 CATGACCACTAGGAGCATCTTTGGCGAGATCGGGAGAATCGCTGGCATTGGTGTCTTCTAGGGGAATAAATCTTTGGGCACCTAGTGGTCATG

3 CATGACCACTAGGAGCATCTTTGGCGAGCCTTGCTTGGGAGGTTGCTCCACCTGTTCCTAGGGGAATAAATCTTTGGGCACCTAGTGGTCATG

3 CATGACCACTAGGAGCATCTTTGGCGAGATTTGGAGAATCGGTGGCATTGGTGTCTCCTAGGGGAATAAATCTTTGGGCACCTAGTGGTCATG

3 CATGACCACTAGGAGCATCTTTGGCGAGAACGGGAGAAACGGTGGCATTGGTGTCTCCTAGGGGAATAAATCTTTGGGCACCTAGTGGTCATG

3 CATGACCACTAGGAGCATCTTTGGCGAGATCGGGAGAGTCGGTGGCATCGGTGTCTCCTAGGGGAATAAATCTTTGGGCACCTAGTGGTCATG

3 CATGACCACTAGGAGCATCTTTGGCGAGCCTTGCTTGGGGGGTTGCTCCACCAGTTCCTAGGGGAATAAATCTTTGGGCACCTAGTGGTCATG

3 CATGACCACTAGGAGCATCTTTGGCGAGCCTTGCTCGGGAGGTTGCTCCACCAGTTCCTAGGGGAATAAATCTTTGGGCACCTAGTGGTCATG

3 CATGACCACTAGGAGCATCTTTGGCGACGGGTGTAGGACTTCAAGTGGATCTCATAGCTAGGGGAATAAATCTTTGGGCACCTAGTGGTCATG

3 CATGACCACTAGGAGCATCTTTGGCGAGATCGCGAGTATCGGTGGCATTGGTGTCTCCTAGGGGAATAAATCTTTGGGCACCTAGTGGTCATG

3 CATGACCACTAGGAGCATCTTTGGCGACGTAGGTGGCATCGTGGTAGAGCTACTAGACTAGGGGAATAAATCTTTGGGCACCTAGTGGTCATG

3 CATGACCACTAGGAGCATCTTTGGCGAACGGCAGGTGTTGCGGTGGTCTGTGAATGCCTAGGGGAATAAATCTTTGGGCACCTAGTGGTCATG

3 CATGACCACTAGGAGCATCTTTGGCGAATGTGGGAATGCCTGAGGATAATAATTCGACTAGGGGAATAAATCTTTGGGCACCTAGTGGTCATG

3 CATGACCACTAGGAGCATCTTTGGCGAGCCTTACTTGGGAGGTTGCTCCACCAGTTCCTAGGGGAATAAATCTTTGGGCACCTAGTGGTCATG

3 CATGACCACTAGGAGCATCTTTGGCGAACGACTGTGAAGACAGAGCAGTCTTGGAACCTAGGGGAATAAATCTTTGGGCACCTAGTGGTCATG

3 CATGACCACTAGGAGCATCTTTGGCGAGATTGCGAGAATCGGTGGCATTGGTGTCTCCTAGGGGAATAAATCTTTGGGCACCTAGTGGTCATG

3 CATGACCACTAGGAGCATCTTTGGCGAGCCTTGCCTGGGAGGTTGCTCCACCAGTTCCTAGGGGAATAAATCTTTGGGCACCTAGTGGTCATG

3 CATGACCACTAGGAGCATCTTTGGCGAATGGCCGGCAGGGCCTTCTAGTCCTCGGTACTAGGGGAATAAATCTTTGGGCACCTAGTGGTCATG

3 CATGACCACTAGGAGCATCTTTGGCGAAAGCAGAAGGTGTGAATAACAAGAGTCCTACTAGGGGAATAAATCTTTGGGCACCTAGTGGTCATG

3 CATGACCACTAGGAGCATCTTTGGCGACTCAGTGGTCAACGCGTTAAGTGGGGTAGACTAGGGGAATAAATCTTTGGGCACCTAGTGGTCATG

3 CATGACCACTAGGAGCATCTTTGGCGACGTAGTGGCGAACGTGTGGCTCTGCACACGCTAGGGGAATAAATCTTTGGGCACCTAGTGGTCATG

3 CATGACCACTAGGAGCATCTTTGGCGAGATCGGGAGAATTGGAGGCATTGGTGTCTCCTAGGGGAATAAATCTTTGGGCACCTAGTGGTCATG

3 CATGACCACTAGGAGCATCTTTGGCGAGATTGAGAGAATCGGTGGCATTGGTGTCTCCTAGGGGAATAAATCTTTGGGCACCTAGTGGTCATG

3 CATGACCACTAGGAGCATCTTTGGCGAGCGGCAGGTGTTGCGGTGGTCTGTGAACCCCTAGGGGAATAAATCTTTGGGCACCTAGTGGTCATG

3 CATGACCACTAGGAGCATCTTTGGCGAAATCGTGAGAATCGGTGGCATTGGTGTCTCCTAGGGGAATAAATCTTTGGGCACCTAGTGGTCATG

3 CATGACCACTAGGAGCATCTTTGGCGAGCCTTGCTTGGGAGGTTGCTCAACCAGTTCCTAGGGGAATAAATCTTTGGGCACCTAGTGGTCATG

3 CATGACCACTAGGAGCATCTTTGGCGAGGGAGGGCGCCGGCAGCGGTGTCAATGCGACTAGGGGAATAAATCTTTGGGCACCTAGTGGTCATG

3 CATGACCACTAGGAGCATCTTTGGCGATAGTCTGTAGAACAGGGTGGGGTGCTGTCCCTAGGGGAATAAATCTTTGGGCACCTAGTGGTCATG

3 CATGACCACTAGGAGCATCTTTGGCGACGTGGGCAACGTGACGGTACTGGATGTCGACTAGGGGAATAAATCTTTGGGCACCTAGTGGTCATG

3 CATGACCACTAGGAGCATCTTTGGCGACGGCGTGGGGCTATAGATGCGGCGGTACTCCTAGGGGAATAAATCTTTGGGCACCTAGTGGTCATG

3 CATGACCACTAGGAGCATCTTTGGCGAGATCGGGAGGGTCGGTGGCATTGGTGTCTCCTAGGGGAATAAATCTTTGGGCACCTAGTGGTCATG

3 CATGACCACTAGGAGCATCTTTGGCGAGAAGACTCTGGATTCGGGGATCAGTTGCTCCTAGGGGAATAAATCTTTGGGCACCTAGTGGTCATG

3 CATGACCACTAGGAGCATCTTTGGCGAGATCGGGAGAATCTGCGGCATTGGTGTCTCCTAGGGGAATAAATCTTTGGGCACCTAGTGGTCATG

3 CATGACCACTAGGAGCATCTTTGGCGACGTTGGCTGGTAGAGACTGTATTGAGGAACCTAGGGGAATAAATCTTTGGGCACCTAGTGGTCATG

3 CATGACCACTAGGAGCATCTTTGGCGACTGGCTGGACATTCGAGGGTTTTTATGTTGCTAGGGGAATAAATCTTTGGGCACCTAGTGGTCATG

3 CATGACCACTAGGAGCATCTTTGGCGAGGGGTCATGGGAATGTAATTGCGCTTAAGGCTAGGGGAATAAATCTTTGGGCACCTAGTGGTCATG

3 CATGACCACTAGGAGCATCTTTGGCGAGATCGGGAGAAACGGTGGCATTGGTGTTTCCTAGGGGAATAAATCTTTGGGCACCTAGTGGTCATG

3 CATGACCACTAGGAGCATCTTTGGCGAGATCGGGAGAATCGTTGGCATTGTTGTCTCCTAGGGGAATAAATCTTTGGGCACCTAGTGGTCATG

3 CATGACCACTAGGAGCATCTTTGGCGAGAAGACTCTGGATTCGGGGACCGGTTGCTCCTAGGGGAATAAATCTTTGGGCACCTAGTGGTCATG

3 CATGACCACTAGGAGCATCTTTGGCGATCCGGGGGTCATAAGTGACGGCGCATAGGGCTAGGGGAATAAATCTTTGGGCACCTAGTGGTCATG

3 CATGACCACTAGGAGCATCTTTGGCGAACGTCGTCGGCAACGAGGCGATAAGTGGAGCTAGGGGAATAAATCTTTGGGCACCTAGTGGTCATG

3 CATGACCACTAGGAGCATCTTTGGCGATATCGGGAGAATCGGCGGCATTGGTGTCTCCTAGGGGAATAAATCTTTGGGCACCTAGTGGTCATG

3 CATGACCACTAGGAGCATCTTTGGCGATCGCACAGCGGACGGTAGGAACGGAGGGGCCTAGGGGAATAAATCTTTGGGCACCTAGTGGTCATG

3 CATGACCACTAGGAGCATCTTTGGCGAGATAGGGAGAAACGGTGGCATTGGTGTCTCCTAGGGGAATAAATCTTTGGGCACCTAGTGGTCATG

3 CATGACCACTAGGAGCATCTTTGGCGAGTAGTGGGTCTGACAAGGGTAAAAACGAGACTAGGGGAATAAATCTTTGGGCACCTAGTGGTCATG

3 CATGACCACTAGGAGCATCTTTGGCGATATCGGGAGAATCGGTGGCATTGGTGTCTTCTAGGGGAATAAATCTTTGGGCACCTAGTGGTCATG

3 CATGACCACTAGGAGCATCTTTGGCGACTCACAGACAGCATGTCACTGTGTAGTGGACTAGGGGAATAAATCTTTGGGCACCTAGTGGTCATG

3 CATGACCACTAGGAGCATCTTTGGCGAACGGCAGGTGTTGTGGTGGTCTGTGAATCTCTAGGGGAATAAATCTTTGGGCACCTAGTGGTCATG

3 CATGACCACTAGGAGCATCTTTGGCGAGACAAGACGGGAGTGGCTTGAAGATTCCCCCTAGGGGAATAAATCTTTGGGCACCTAGTGGTCATG

3 CATGACCACTAGGAGCATCTTTGGCGAGACGGCAGTCTTCGAAGGGATCTGGGAAGGCTAGGGGAATAAATCTTTGGGCACCTAGTGGTCATG

3 CATGACCACTAGGAGCATCTTTGGCGACTCGACCGGGGCCCTAGGGCTGCGGCCCACCTAGGGGAATAAATCTTTGGGCACCTAGTGGTCATG

3 CATGACCACTAGGAGCATCTTTGGCGACGTGGGCGTGTCGGCCGTGTGGCGTAGTAGCTAGGGGAATAAATCTTTGGGCACCTAGTGGTCATG

3 CATGACCACTAGGAGCATCTTTGGCGAGTAGAGGTGGTGCGTTGGCGGAGCGATGCTCTAGGGGAATAAATCTTTGGGCACCTAGTGGTCATG

2 CATGACCACTAGGAGCATCTTTGGCGATCCGGGGGCACCTATGTGCGACGCCGTGGGCTAGGGGAATAAATCTTTGGGCACCTAGTGGTCATG

2 CATGACCACTAGGAGCATCTTTGGCGATCGCAGAGCGTACAGTGGTATCAGTGGGGCCTAGGGGAATAAATCTTTGGGCACCTAGTGGTCATG

2 CATGACCACTAGGAGCATCTTTGGCGAGATCGGGAGAATCGGTGGCATTGGAGTCTTCTAGGGGAATAAATCTTTGGGCACCTAGTGGTCATG

2 CATGACCACTAGGAGCATCTTTGGCGACAACGTCGATGAGATAGCGACGCCGTGGAGCTAGGGGAATAAATCTTTGGGCACCTAGTGGTCATG

2 CATGACCACTAGGAGCATCTTTGGCGATGGGGCCGTGCTATCTGCACACTTTGCGGGCTAGGGGAATAAATCTTTGGGCACCTAGTGGTCATG

2 CATGACCACTAGGAGCATCTTTGGCGATGGGTGCTGAAGGCCGCCGCTGCGGCTACACTAGGGGAATAAATCTTTGGGCACCTAGTGGTCATG

2 CATGACCACTAGGAGCATCTTTGGCGAGATCGGGAGAATCGGTGGCATTGGTGTCAGCTAGGGGAATAAATCTTTGGGCACCTAGTGGTCATG

2 CATGACCACTAGGAGCATCTTTGGCGAGATTGGGAGAATCTGTGGCATTGGTGTCTCCTAGGGGAATAAATCTTTGGGCACCTAGTGGTCATG

2 CATGACCACTAGGAGCATCTTTGGCGATCAGGGGGACGTTGTGGTCGACTCTACGGGCTAGGGGAATAAATCTTTGGGCACCTAGTGGTCATG

2 CATGACCACTAGGAGCATCTTTGGCGACGTGGGCTGGGAGGATATGGTACTTCTCGACTAGGGGAATAAATCTTTGGGCACCTAGTGGTCATG

2 CATGACCACTAGGAGCATCTTTGGCGAGATCGGGAGAATCGCAGGCATTGGTGTCTCCTAGGGGAATAAATCTTTGGGCACCTAGTGGTCATG

2 CATGACCACTAGGAGCATCTTTGGCGAGTTGTCGTAGAGCTACGGGACACCCGTCAGCTAGGGGAATAAATCTTTGGGCACCTAGTGGTCATG

2 CATGACCACTAGGAGCATCTTTGGCGAGATCGGGAGAATCGTTGTCATTGGTGTCTCCTAGGGGAATAAATCTTTGGGCACCTAGTGGTCATG

2 CATGACCACTAGGAGCATCTTTGGCGAAGGGTGCAGGACTTCAAGTGGATCTCATAGCTAGGGGAATAAATCTTTGGGCACCTAGTGGTCATG

2 CATGACCACTAGGAGCATCTTTGGCGAAGCGGAAGGAGATGCGTATGGTGGCGCGAGCTAGGGGAATAAATCTTTGGGCACCTAGTGGTCATG

2 CATGACCACTAGGAGCATCTTTGGCGAATCTTGGTGGGAAGCGGTTGTGTGCTCCGCCTAGGGGAATAAATCTTTGGGCACCTAGTGGTCATG

2 CATGACCACTAGGAGCATCTTTGGCGACGTGTGGCCGAGTAGCATGGGTGAGATCGACTAGGGGAATAAATCTTTGGGCACCTAGTGGTCATG

2 CATGACCACTAGGAGCATCTTTGGCGATCGGCGCGGATCAGTCAGGGTTGCGAACTGCTAGGGGAATAAATCTTTGGGCACCTAGTGGTCATG

2 CATGACCACTAGGAGCATCTTTGGCGAGCCTTGCTTGGGAGGTTGCTCCACCAGTTGCTAGGGGAATAAATCTTTGGGCACCTAGTGGTCATG

2 CATGACCACTAGGAGCATCTTTGGCGAGGTCGGGGGAATCGGTGGCATTGGTGTCTCCTAGGGGAATAAATCTTTGGGCACCTAGTGGTCATG

2 CATGACCACTAGGAGCATCTTTGGCGAGCCTCGCTTGGGAGGTTGCTCCACCTGTTCCTAGGGGAATAAATCTTTGGGCACCTAGTGGTCATG

2 CATGACCACTAGGAGCATCTTTGGCGAACGGAAGGTGTTGCGGTGGTTTGTGAATCCCTAGGGGAATAAATCTTTGGGCACCTAGTGGTCATG

2 CATGACCACTAGGAGCATCTTTGGCGATCGCCCACGTGGTTCGGGAATCTAAAACCCCTAGGGGAATAAATCTTTGGGCACCTAGTGGTCATG

2 CATGACCACTAGGAGCATCTTTGGCGAGCACTACGTAGCGGTTGGCATTGTTGTCTCCTAGGGGAATAAATCTTTGGGCACCTAGTGGTCATG

2 CATGACCACTAGGAGCATCTTTGGCGACTTGTGGGAGTGGGAAGGGTAGGGCGGAGACTAGGGGAATAAATCTTTGGGCACCTAGTGGTCATG

2 CATGACCACTAGGAGCATCTTTGGCGAAGCGTAGGGTGTGAGAGGGGCGTGTCCATGCTAGGGGAATAAATCTTTGGGCACCTAGTGGTCATG

2 CATGACCACTAGGAGCATCTTTGGCGACCCGGGACGCACTTGATGGACCGTGGTGGACTAGGGGAATAAATCTTTGGGCACCTAGTGGTCATG

2 CATGACCACTAGGAGCATCTTTGGCGAGACCGGGAGAATCGTTGGCATTGGTGTCTCCTAGGGGAATAAATCTTTGGGCACCTAGTGGTCATG

2 CATGACCACTAGGAGCATCTTTGGCGAGCCTTGCTTAGGAGGTTGCTCCACCAGTTCCTAGGGGAATAAATCTTTGGGCACCTAGTGGTCATG

2 CATGACCACTAGGAGCATCTTTGGCGAGGTCGGGAGAATCGGTGGCATTGGTGTCTGCTAGGGGAATAAATCTTTGGGCACCTAGTGGTCATG

2 CATGACCACTAGGAGCATCTTTGGCGAGAAGACTTTGGATTCGGGGACCAGTGTCTCCTAGGGGAATAAATCTTTGGGCACCTAGTGGTCATG

2 CATGACCACTAGGAGCATCTTTGGCGAGATCGCGAGAATCGGTGGCATAGGTGTCTCCTAGGGGAATAAATCTTTGGGCACCTAGTGGTCATG

2 CATGACCACTAGGAGCATCTTTGGCGAGGGAAGGCGCCGGCAGCGGTGTGAATGCGACTAGGGGAATAAATCTTTGGGCACCTAGTGGTCATG

2 CATGACCACTAGGAGCATCTTTGGCGACGGGGTGGGGCCGAAGTGCGGCGGTGACTCCTAGGGGAATAAATCTTTGGGCACCTAGTGGTCATG

2 CATGACCACTAGGAGCATCTTTGGCGAGATTGGGAGAATCGGTGGCCTTGGTGTCTCCTAGGGGAATAAATCTTTGGGCACCTAGTGGTCATG

2 CATGACCACTAGGAGCATCTTTGGCGAGATAGGGAGAATCGGTGGAATTGGTGTCTCCTAGGGGAATAAATCTTTGGGCACCTAGTGGTCATG

2 CATGACCACTAGGAGCATCTTTGGCGACGGTGTGGGGCCTAAGTGCGGCGGCTGATTCTAGGGGAATAAATCTTTGGGCACCTAGTGGTCATG

2 CATGACCACTAGGAGCATCTTTGGCGATCCGGGGGTCCTACGTGACGACGCAAAGGGCTAGGGGAATAAATCTTTGGGCACCTAGTGGTCATG

2 CATGACCACTAGGAGCATCTTTGGCGAGATCTGGAGAATCGGTGGCATTCGTGTCTCCTAGGGGAATAAATCTTTGGGCACCTAGTGGTCATG

2 CATGACCACTAGGAGCATCTTTGGCGAACGGAAGGTGTTGCGGTGGTCTGTGGATCCCTAGGGGAATAAATCTTTGGGCACCTAGTGGTCATG

2 CATGACCACTAGGAGCATCTTTGGCGAGATCGGGAGAATCGGTGGCAGTGGAGTCTCCTAGGGGAATAAATCTTTGGGCACCTAGTGGTCATG

2 CATGACCACTAGGAGCATCTTTGGCGAGATCGCGAGAATCGGTGGTATTGGTGTCTCCTAGGGGAATAAATCTTTGGGCACCTAGTGGTCATG

2 CATGACCACTAGGAGCATCTTTGGCGATCCGGGGGCATGCTATGCGACGCTTAAGGGCTAGGGGAATAAATCTTTGGGCACCTAGTGGTCATG

2 CATGACCACTAGGAGCATCTTTGGCGAACGGCAGGTGTTGCGGTGGACTGAGAATCCCTAGGGGAATAAATCTTTGGGCACCTAGTGGTCATG

2 CATGACCACTAGGAGCATCTTTGGCGACCCATATCTGCCGTGCAGTCATGTGGTGGACTAGGGGAATAAATCTTTGGGCACCTAGTGGTCATG

2 CATGACCACTAGGAGCATCTTTGGCGACCTGGTGGGGCATGCTCAAACCAGTGTGGACTAGGGGAATAAATCTTTGGGCACCTAGTGGTCATG

2 CATGACCACTAGGAGCATCTTTGGCGAGAAGACTCCGGATTCGGGGACCAGTTGCCGCTAGGGGAATAAATCTTTGGGCACCTAGTGGTCATG

2 CATGACCACTAGGAGCATCTTTGGCGAGATCGACAGAATCGGTGGCATTGGTGTCTCCTAGGGGAATAAATCTTTGGGCACCTAGTGGTCATG

2 CATGACCACTAGGAGCATCTTTGGCGAGATCTTAACGAAGTGAATAGCGGCGGGGGCCTAGGGGAATAAATCTTTGGGCACCTAGTGGTCATG

2 CATGACCACTAGGAGCATCTTTGGCGACAAGCAATGCAATTGCGAGACATAGTGCTTCTAGGGGAATAAATCTTTGGGCACCTAGTGGTCATG

2 CATGACCACTAGGAGCATCTTTGGCGACATTCGTGCAAGGCATCGAAAGAAGTGGAGCTAGGGGAATAAATCTTTGGGCACCTAGTGGTCATG

2 CATGACCACTAGGAGCATCTTTGGCGAGATCGGGAGAATCGGTGGCATTGGTATCTTCTAGGGGAATAAATCTTTGGGCACCTAGTGGTCATG

2 CATGACCACTAGGAGCATCTTTGGCGATCGCACAGCTAAGGTATCAAATAACTGGGCCTAGGGGAATAAATCTTTGGGCACCTAGTGGTCATG

2 CATGACCACTAGGAGCATCTTTGGCGAGATCGGGGGAATCGGTGGCATCGGTGTCTCCTAGGGGAATAAATCTTTGGGCACCTAGTGGTCATG

2 CATGACCACTAGGAGCATCTTTGGCGAGATCGGGAGAATCGGTGGCATTGGGGTCTTCTAGGGGAATAAATCTTTGGGCACCTAGTGGTCATG

2 CATGACCACTAGGAGCATCTTTGGCGACCAGATATGGTAGGTAATGTGCTGTGGAGGCTAGGGGAATAAATCTTTGGGCACCTAGTGGTCATG

2 CATGACCACTAGGAGCATCTTTGGCGACGACAGAGGGGAAGATGACGACGGAGTGGACTAGGGGAATAAATCTTTGGGCACCTAGTGGTCATG

2 CATGACCACTAGGAGCATCTTTGGCGACGGTGTGGGGAACTTGTTTCGGCGGTTCTACTAGGGGAATAAATCTTTGGGCACCTAGTGGTCATG

2 CATGACCACTAGGAGCATCTTTGGCGATAAGCACAGCGATTGTGTTACACAGGGGGCCTAGGGGAATAAATCTTTGGGCACCTAGTGGTCATG

2 CATGACCACTAGGAGCATCTTTGGCGACGTGGGCGTTTCGGCCGTTTCGGCGTGTGCCTAGGGGAATAAATCTTTGGGCACCTAGTGGTCATG

2 CATGACCACTAGGAGCATCTTTGGCGACACAGACATAAGTATGCGAGTGGTGGCGTCCTAGGGGAATAAATCTTTGGGCACCTAGTGGTCATG

2 CATGACCACTAGGAGCATCTTTGGCGACAGTGCGAGGTTGGTTCAGTGGTCTGCGATCTAGGGGAATAAATCTTTGGGCACCTAGTGGTCATG

2 CATGACCACTAGGAGCATCTTTGGCGAGATCGGGAAAATCGGCGGCATTGGTGTCTCCTAGGGGAATAAATCTTTGGGCACCTAGTGGTCATG

2 CATGACCACTAGGAGCATCTTTGGCGAGATTGGGAGAATCGGTGGCATTGGTATCTCCTAGGGGAATAAATCTTTGGGCACCTAGTGGTCATG

2 CATGACCACTAGGAGCATCTTTGGCGATACAGGTGATCGGTGCTTGGGTGCCTGGCCCTAGGGGAATAAATCTTTGGGCACCTAGTGGTCATG

2 CATGACCACTAGGAGCATCTTTGGCGAGATCGGGAGAATCGGCGGCATTGGTGGCTCCTAGGGGAATAAATCTTTGGGCACCTAGTGGTCATG

2 CATGACCACTAGGAGCATCTTTGGCGAATGGCCGGCACGGCCTTCTAGTCCTCGGAACTAGGGGAATAAATCTTTGGGCACCTAGTGGTCATG

2 CATGACCACTAGGAGCATCTTTGGCGAACATCGTGGTCGGTAGGCGTCGGCGGAGTGCTAGGGGAATAAATCTTTGGGCACCTAGTGGTCATG

2 CATGACCACTAGGAGCATCTTTGGCGAGAAGACTCTGCATTCGGGGACCAGTTGCTGCTAGGGGAATAAATCTTTGGGCACCTAGTGGTCATG

2 CATGACCACTAGGAGCATCTTTGGCGAGATCGAGAGAATCGGTGGCATTGGTGTCTGCTAGGGGAATAAATCTTTGGGCACCTAGTGGTCATG

2 CATGACCACTAGGAGCATCTTTGGCGACACTACCCAGTGGCGTCACTCTGCTCATATCTAGGGGAATAAATCTTTGGGCACCTAGTGGTCATG

2 CATGACCACTAGGAGCATCTTTGGCGAAATCGGGAGAATCGGTGGCGTTGGTGTCTCCTAGGGGAATAAATCTTTGGGCACCTAGTGGTCATG

2 CATGACCACTAGGAGCATCTTTGGCGAAGATCGCTCGTCGACTCGGGTCCGAGGAAGCTAGGGGAATAAATCTTTGGGCACCTAGTGGTCATG

2 CATGACCACTAGGAGCATCTTTGGCGACTAACTCTCGGGTTATGCCGGTATCGGGAACTAGGGGAATAAATCTTTGGGCACCTAGTGGTCATG

2 CATGACCACTAGGAGCATCTTTGGCGAGGGAGGGCGCCGGCCGCGGTGTGAATGCGCCTAGGGGAATAAATCTTTGGGCACCTAGTGGTCATG

2 CATGACCACTAGGAGCATCTTTGGCGAACGGCAGGTGTTGCGGTGGTTTTTGAATCCCTAGGGGAATAAATCTTTGGGCACCTAGTGGTCATG

2 CATGACCACTAGGAGCATCTTTGGCGAGATCTGGAGAATCGGTGGCATCGGTGTCTCCTAGGGGAATAAATCTTTGGGCACCTAGTGGTCATG

2 CATGACCACTAGGAGCATCTTTGGCGAGATCGGGAGAATAGGTTGCATTGGTGTCTCCTAGGGGAATAAATCTTTGGGCACCTAGTGGTCATG

2 CATGACCACTAGGAGCATCTTTGGCGACGACCTCGGAGTGGGTGCGCCGCTGGCATACTAGGGGAATAAATCTTTGGGCACCTAGTGGTCATG

2 CATGACCACTAGGAGCATCTTTGGCGAACGGTAGGTGCTGCGGTGGTCTGTGAATCCCTAGGGGAATAAATCTTTGGGCACCTAGTGGTCATG

2 CATGACCACTAGGAGCATCTTTGGCGAATGGCCGGCACGGCCTTCTAGTTCTTGGTACTAGGGGAATAAATCTTTGGGCACCTAGTGGTCATG

2 CATGACCACTAGGAGCATCTTTGGCGACCGAGGATGCGTGTGATGTGGTCGAGCAAGCTAGGGGAATAAATCTTTGGGCACCTAGTGGTCATG

2 CATGACCACTAGGAGCATCTTTGGCGAAAGCGCAGGGCATGCGACCTCTAGTTGGTCCTAGGGGAATAAATCTTTGGGCACCTAGTGGTCATG

2 CATGACCACTAGGAGCATCTTTGGCGAGAAGACTCTGGATTCGTGGACCAGTTGCTCCTAGGGGAATAAATCTTTGGGCACCTAGTGGTCATG

2 CATGACCACTAGGAGCATCTTTGGCGACTAGCGGTCCCACCACCGCGCGGCGTAGTGCTAGGGGAATAAATCTTTGGGCACCTAGTGGTCATG

2 CATGACCACTAGGAGCATCTTTGGCGAATGTCCGGCACGGCCTTCTAGTCCTCGGTACTAGGGGAATAAATCTTTGGGCACCTAGTGGTCATG

2 CATGACCACTAGGAGCATCTTTGGCGACAATGGAGCGACGGTGTGGTCCGAGTCCTACTAGGGGAATAAATCTTTGGGCACCTAGTGGTCATG

2 CATGACCACTAGGAGCATCTTTGGCGAGATCTGGAGAATCGGTGGCATTGGTGTTTCCTAGGGGAATAAATCTTTGGGCACCTAGTGGTCATG

2 CATGACCACTAGGAGCATCTTTGGCGAGCCTCGTTTGGGAGGTTGCTCCACCAGTTCCTAGGGGAATAAATCTTTGGGCACCTAGTGGTCATG

2 CATGACCACTAGGAGCATCTTTGGCGAGATCTAGAGAATCGGTGGCATTGGTGTCTCCTAGGGGAATAAATCTTTGGGCACCTAGTGGTCATG

2 CATGACCACTAGGAGCATCTTTGGCGACGGTGGGCCGAACAAGAAAAATGGCCCGTTCTAGGGGAATAAATCTTTGGGCACCTAGTGGTCATG

2 CATGACCACTAGGAGCATCTTTGGCGAGGTTGTTCGAAGTTATCACCCTTAAGGGTGCTAGGGGAATAAATCTTTGGGCACCTAGTGGTCATG

2 CATGACCACTAGGAGCATCTTTGGCGATCGCAGAGCGGACCCGGTGAGTGGGCGGGCCTAGGGGAATAAATCTTTGGGCACCTAGTGGTCATG

2 CATGACCACTAGGAGCATCTTTGGCGAGTGGTCCTGTTCAAACCGGAGGACGTTGGGCTAGGGGAATAAATCTTTGGGCACCTAGTGGTCATG

2 CATGACCACTAGGAGCATCTTTGGCGAAGGGTGTAGGACTTCAAGTGGATCTCACAGCTAGGGGAATAAATCTTTGGGCACCTAGTGGTCATG

2 CATGACCACTAGGAGCATCTTTGGCGAGATCGGGAGAATCGGTGGCACTGGTGTCTACTAGGGGAATAAATCTTTGGGCACCTAGTGGTCATG

2 CATGACCACTAGGAGCATCTTTGGCGAGTCAGACTATGATGCAAGTGGAAACTCGCACTAGGGGAATAAATCTTTGGGCACCTAGTGGTCATG

2 CATGACCACTAGGAGCATCTTTGGCGATCTGCGTGGTCGTCGGATTAAGTTACGCCGCTAGGGGAATAAATCTTTGGGCACCTAGTGGTCATG

2 CATGACCACTAGGAGCATCTTTGGCGAACGGCAGATGTTGCGGTGGTTTGTGAATCCCTAGGGGAATAAATCTTTGGGCACCTAGTGGTCATG

2 CATGACCACTAGGAGCATCTTTGGCGAACGGAAGGTGTTGCGGTGGTCCGTGAATCCCTAGGGGAATAAATCTTTGGGCACCTAGTGGTCATG

2 CATGACCACTAGGAGCATCTTTGGCGATATCGGGAGAATCGGTGGCATTGGAGTCTCCTAGGGGAATAAATCTTTGGGCACCTAGTGGTCATG

2 CATGACCACTAGGAGCATCTTTGGCGAGTCGTACTTACCACTAGCGAGTGATGCTCTCTAGGGGAATAAATCTTTGGGCACCTAGTGGTCATG

2 CATGACCACTAGGAGCATCTTTGGCGAGCCTTGGTTGGGAGGTTGCTCCACCAGTTCCTAGGGGAATAAATCTTTGGGCACCTAGTGGTCATG

2 CATGACCACTAGGAGCATCTTTGGCGAGTTGGGGGCGACGGCCGGTAGTGGGTGAGACTAGGGGAATAAATCTTTGGGCACCTAGTGGTCATG

2 CATGACCACTAGGAGCATCTTTGGCGATCAGGGGGCCTTTGATGGCAACTCAGTGGGCTAGGGGAATAAATCTTTGGGCACCTAGTGGTCATG

2 CATGACCACTAGGAGCATCTTTGGCGAGATCGTGAGAAACGGTGGCATTGGTGTCTCCTAGGGGAATAAATCTTTGGGCACCTAGTGGTCATG

2 CATGACCACTAGGAGCATCTTTGGCGATAACGGGAGAATCGGTGGCAATGGTGTCTCCTAGGGGAATAAATCTTTGGGCACCTAGTGGTCATG

2 CATGACCACTAGGAGCATCTTTGGCGACGTAAGTGGGCATGCAACTGCATAGCTCCGCTAGGGGAATAAATCTTTGGGCACCTAGTGGTCATG

2 CATGACCACTAGGAGCATCTTTGGCGACGGGATTCGGTAGGAAAGAGTGCACGCTGACTAGGGGAATAAATCTTTGGGCACCTAGTGGTCATG

2 CATGACCACTAGGAGCATCTTTGGCGAGATCGGGAGAATTGGTGGCATTGTTGTCTCCTAGGGGAATAAATCTTTGGGCACCTAGTGGTCATG

2 CATGACCACTAGGAGCATCTTTGGCGATTTCGATGGCCACGACCTTGCGGCCGCAACCTAGGGGAATAAATCTTTGGGCACCTAGTGGTCATG

2 CATGACCACTAGGAGCATCTTTGGCGAGGGGTCATGGGAATGTAATTGCGCTTAATACTAGGGGAATAAATCTTTGGGCACCTAGTGGTCATG

2 CATGACCACTAGGAGCATCTTTGGCGAGATCGGGAGAATCTGTGGCATTTGTGTCTCCTAGGGGAATAAATCTTTGGGCACCTAGTGGTCATG

2 CATGACCACTAGGAGCATCTTTGGCGACAGGGGATGCCGATGCGCCGGACGACCGAGCTAGGGGAATAAATCTTTGGGCACCTAGTGGTCATG

2 CATGACCACTAGGAGCATCTTTGGCGACCCGAAAGGAGTGGGAGAGGCGAAGGTCGACTAGGGGAATAAATCTTTGGGCACCTAGTGGTCATG

2 CATGACCACTAGGAGCATCTTTGGCGAGAAGCCTCTGGATTCTGGGACCAGTTGCTGCTAGGGGAATAAATCTTTGGGCACCTAGTGGTCATG

2 CATGACCACTAGGAGCATCTTTGGCGACCAGTGAAATAAAGATTCGACCAGCGAAGACTAGGGGAATAAATCTTTGGGCACCTAGTGGTCATG

2 CATGACCACTAGGAGCATCTTTGGCGACGAGTGGGCTCAGAGGAATATTGTAGTCGACTAGGGGAATAAATCTTTGGGCACCTAGTGGTCATG

2 CATGACCACTAGGAGCATCTTTGGCGAGATTGGGAGAATCGGTGGCATTGTTGTCTCCTAGGGGAATAAATCTTTGGGCACCTAGTGGTCATG

2 CATGACCACTAGGAGCATCTTTGGCGAGATTGGGAGAATCGGTGGCGTTGGTGTCTCCTAGGGGAATAAATCTTTGGGCACCTAGTGGTCATG

2 CATGACCACTAGGAGCATCTTTGGCGAAGGGTGTAGGACTTCAAGTGGATCCCATAGCTAGGGGAATAAATCTTTGGGCACCTAGTGGTCATG

2 CATGACCACTAGGAGCATCTTTGGCGAATTCTGTTTTATTTCGACGTGTCCCTTTATCTAGGGGAATAAATCTTTGGGCACCTAGTGGTCATG

2 CATGACCACTAGGAGCATCTTTGGCGAACGGCAGGTGTTGCTGTGGTCTGTGGATCCCTAGGGGAATAAATCTTTGGGCACCTAGTGGTCATG

2 CATGACCACTAGGAGCATCTTTGGCGAGAAGACTCTGGATTCGGGGACCGGTGTCTCCTAGGGGAATAAATCTTTGGGCACCTAGTGGTCATG

2 CATGACCACTAGGAGCATCTTTGGCGAACGGCAGGTGTTGCGGTGGCTTGTGAATCCCTAGGGGAATAAATCTTTGGGCACCTAGTGGTCATG

2 CATGACCACTAGGAGCATCTTTGGCGAGATTGTAGCTCGCGCGTAGGGTCGGCAGTGCTAGGGGAATAAATCTTTGGGCACCTAGTGGTCATG

2 CATGACCACTAGGAGCATCTTTGGCGAGAAGACTATGGATTCGGGGACCAGTCGCTGCTAGGGGAATAAATCTTTGGGCACCTAGTGGTCATG

2 CATGACCACTAGGAGCATCTTTGGCGAGATCTGGAGAATCGGTGGCATTGTTGTCTCCTAGGGGAATAAATCTTTGGGCACCTAGTGGTCATG

2 CATGACCACTAGGAGCATCTTTGGCGAGGTCGATATGGAACTGGCGAACCGTTGGTCCTAGGGGAATAAATCTTTGGGCACCTAGTGGTCATG

2 CATGACCACTAGGAGCATCTTTGGCGATTTGGGAGTCGCTGTATGCAGGCAGTGAGACTAGGGGAATAAATCTTTGGGCACCTAGTGGTCATG

2 CATGACCACTAGGAGCATCTTTGGCGAGATCGGGAGAATCGGTGGCAAAGGTGTCTCCTAGGGGAATAAATCTTTGGGCACCTAGTGGTCATG

2 CATGACCACTAGGAGCATCTTTGGCGAGATCGGTAGAATCTGTGGCATTGGTGTCTCCTAGGGGAATAAATCTTTGGGCACCTAGTGGTCATG

2 CATGACCACTAGGAGCATCTTTGGCGACGGTGTGGGGAACTAGTTTCGGCGGTGCTACTAGGGGAATAAATCTTTGGGCACCTAGTGGTCATG

2 CATGACCACTAGGAGCATCTTTGGCGAACCGTTAGTGGGGGAAATAGAGCTCGTAGACTAGGGGAATAAATCTTTGGGCACCTAGTGGTCATG

2 CATGACCACTAGGAGCATCTTTGGCGAGATCGGGATAATCGGAGGCATTGGTGTCTCCTAGGGGAATAAATCTTTGGGCACCTAGTGGTCATG

2 CATGACCACTAGGAGCATCTTTGGCGATTCGTATGGCTACGTAAGTTAAGCGTCGTGCTAGGGGAATAAATCTTTGGGCACCTAGTGGTCATG

2 CATGACCACTAGGAGCATCTTTGGCGAAAGTCGGTAGAACAGGGTGGGGTGCGGTCCCTAGGGGAATAAATCTTTGGGCACCTAGTGGTCATG

2 CATGACCACTAGGAGCATCTTTGGCGATCGCATAGCGTACTGGTGTAAAGGAGGGGCCTAGGGGAATAAATCTTTGGGCACCTAGTGGTCATG

2 CATGACCACTAGGAGCATCTTTGGCGAGGGAGGGCGCCGGCAGCGGTGTGAATGGGACTAGGGGAATAAATCTTTGGGCACCTAGTGGTCATG

2 CATGACCACTAGGAGCATCTTTGGCGAGACCGGAAGAATCGGTGGCATTGGTGTCTCCTAGGGGAATAAATCTTTGGGCACCTAGTGGTCATG

2 CATGACCACTAGGAGCATCTTTGGCGACCGTGTGGGGAACTTGTTTCGGCGGTGCTACTAGGGGAATAAATCTTTGGGCACCTAGTGGTCATG

2 CATGACCACTAGGAGCATCTTTGGCGAATGGCCGGCACGGCCTTCCAGTCCTCGGTACTAGGGGAATAAATCTTTGGGCACCTAGTGGTCATG

2 CATGACCACTAGGAGCATCTTTGGCGATCCCCTCCGTTGCGGGTTTGACGAGGTTTGCTAGGGGAATAAATCTTTGGGCACCTAGTGGTCATG

2 CATGACCACTAGGAGCATCTTTGGCGAGGCGCGCGTGGTGGTCTGACAAGATCCCGGCTAGGGGAATAAATCTTTGGGCACCTAGTGGTCATG

2 CATGACCACTAGGAGCATCTTTGGCGACTAGTGAGGCTTGCGTCACGCGGCGTAGTGCTAGGGGAATAAATCTTTGGGCACCTAGTGGTCATG

2 CATGACCACTAGGAGCATCTTTGGCGAATAGCCGGCACGGCCTTCTAGTCCTCGGTACTAGGGGAATAAATCTTTGGGCACCTAGTGGTCATG

2 CATGACCACTAGGAGCATCTTTGGCGAGATCGCGAGAATCGGTGGCATTGGTGTCTGCTAGGGGAATAAATCTTTGGGCACCTAGTGGTCATG

2 CATGACCACTAGGAGCATCTTTGGCGAATGATCCCCTGCGAATTGAGAGCTGAAGGCCTAGGGGAATAAATCTTTGGGCACCTAGTGGTCATG

2 CATGACCACTAGGAGCATCTTTGGCGAGGGTGGGCCAAGGTGCGGTGTGGTCGAGCTCTAGGGGAATAAATCTTTGGGCACCTAGTGGTCATG

2 CATGACCACTAGGAGCATCTTTGGCGAGATCGAGAGAATCGGTGGAATTGGTGTCTCCTAGGGGAATAAATCTTTGGGCACCTAGTGGTCATG

2 CATGACCACTAGGAGCATCTTTGGCGACGGCGTGGGGCAATTGATGCGGCGGCACTCCTAGGGGAATAAATCTTTGGGCACCTAGTGGTCATG

2 CATGACCACTAGGAGCATCTTTGGCGAACGGCAGGTGTTGCGGTGGTCTGCGAACCCCTAGGGGAATAAATCTTTGGGCACCTAGTGGTCATG

2 CATGACCACTAGGAGCATCTTTGGCGAACGGCAGGTGTCGCGGTGGTTTGTGAATCCCTAGGGGAATAAATCTTTGGGCACCTAGTGGTCATG

2 CATGACCACTAGGAGCATCTTTGGCGATAGCAAGAGGTGTGAGCACAGCGAGTACTACTAGGGGAATAAATCTTTGGGCACCTAGTGGTCATG

2 CATGACCACTAGGAGCATCTTTGGCGAGATCGGGAGAATCGGTGGCATTGGTGCCCCCTAGGGGAATAAATCTTTGGGCACCTAGTGGTCATG

2 CATGACCACTAGGAGCATCTTTGGCGAGATCGGGAGAATTGGTGGAATTGGTGTCTCCTAGGGGAATAAATCTTTGGGCACCTAGTGGTCATG

2 CATGACCACTAGGAGCATCTTTGGCGAGATCGGGAGACTCGGAGGCATTGGTGTCTCCTAGGGGAATAAATCTTTGGGCACCTAGTGGTCATG

2 CATGACCACTAGGAGCATCTTTGGCGAGTATTAGGGATGCCTGTTAAAAGCTGGTCTCTAGGGGAATAAATCTTTGGGCACCTAGTGGTCATG

2 CATGACCACTAGGAGCATCTTTGGCGACCGGTAACGTAGTAACGTCGAAGTAGGCGCCTAGGGGAATAAATCTTTGGGCACCTAGTGGTCATG

2 CATGACCACTAGGAGCATCTTTGGCGAGATCGGGAGGATCGGTGGCATTGGTGTCCCCTAGGGGAATAAATCTTTGGGCACCTAGTGGTCATG

2 CATGACCACTAGGAGCATCTTTGGCGAGATCAGGAGAATCGGTGACATTGGTGTCTCCTAGGGGAATAAATCTTTGGGCACCTAGTGGTCATG

2 CATGACCACTAGGAGCATCTTTGGCGAACGGCAGGGGTTGCGGTGGTTTGTGAATCTCTAGGGGAATAAATCTTTGGGCACCTAGTGGTCATG

2 CATGACCACTAGGAGCATCTTTGGCGAGATCTGGAGAATCGGTGGCTTTGGTGTCTCCTAGGGGAATAAATCTTTGGGCACCTAGTGGTCATG

2 CATGACCACTAGGAGCATCTTTGGCGAGATCGGGAGAATCGTTGGCATTGGCGTCTCCTAGGGGAATAAATCTTTGGGCACCTAGTGGTCATG

2 CATGACCACTAGGAGCATCTTTGGCGACACCAGACGTCTAACGATGGTCGTGGAAAGCTAGGGGAATAAATCTTTGGGCACCTAGTGGTCATG

2 CATGACCACTAGGAGCATCTTTGGCGAGATCGGGAGAGTCGGTGGCATTGGTGTTTCCTAGGGGAATAAATCTTTGGGCACCTAGTGGTCATG

2 CATGACCACTAGGAGCATCTTTGGCGACGGAGGGCGCCGGCAGCGGTGTGAATGCGACTAGGGGAATAAATCTTTGGGCACCTAGTGGTCATG

2 CATGACCACTAGGAGCATCTTTGGCGAGATCGGGAGAAACGGTGGCATTTGTGTCTCCTAGGGGAATAAATCTTTGGGCACCTAGTGGTCATG

2 CATGACCACTAGGAGCATCTTTGGCGAGATCGGGAGAATCGGTGGCATAGTTGTCTCCTAGGGGAATAAATCTTTGGGCACCTAGTGGTCATG

2 CATGACCACTAGGAGCATCTTTGGCGAGATCGGGCGAATAGGTGGCATTGGTGTCTCCTAGGGGAATAAATCTTTGGGCACCTAGTGGTCATG

2 CATGACCACTAGGAGCATCTTTGGCGATCAGGCCGTGGCTGCGGGTGGGTGCGCACCCTAGGGGAATAAATCTTTGGGCACCTAGTGGTCATG

2 CATGACCACTAGGAGCATCTTTGGCGAACGGCAGGTGTTGCGGTGGTCTATGAATTCCTAGGGGAATAAATCTTTGGGCACCTAGTGGTCATG

2 CATGACCACTAGGAGCATCTTTGGCGAGATCGGGAGACTCGGTGGCCTTGGTGTCTCCTAGGGGAATAAATCTTTGGGCACCTAGTGGTCATG

2 CATGACCACTAGGAGCATCTTTGGCGAGATCGGGAGAATCTGAGGCATAGGTGTCTCCTAGGGGAATAAATCTTTGGGCACCTAGTGGTCATG

2 CATGACCACTAGGAGCATCTTTGGCGAGAAGATTCTGGATTTGGGGACCAGTTGCTGCTAGGGGAATAAATCTTTGGGCACCTAGTGGTCATG

2 CATGACCACTAGGAGCATCTTTGGCGATTAAGTGGTTAACGCGTTAAGTGGGGTAGACTAGGGGAATAAATCTTTGGGCACCTAGTGGTCATG

2 CATGACCACTAGGAGCATCTTTGGCGAGATCGGGAGAATCGGAGGCAGTGGTGTCTCCTAGGGGAATAAATCTTTGGGCACCTAGTGGTCATG

2 CATGACCACTAGGAGCATCTTTGGCGAGATCTGGAGGATCGGTGGCATTGGTGTCTCCTAGGGGAATAAATCTTTGGGCACCTAGTGGTCATG

2 CATGACCACTAGGAGCATCTTTGGCGAGTGGGGGGCGACGGCCGGTAGTGGATGAGACTAGGGGAATAAATCTTTGGGCACCTAGTGGTCATG

2 CATGACCACTAGGAGCATCTTTGGCGATCCGGGGGCCACAAGTGACGGCGCATAGGGCTAGGGGAATAAATCTTTGGGCACCTAGTGGTCATG

2 CATGACCACTAGGAGCATCTTTGGCGACATACTCGGTTGCTCTTCCATTTGAGTGAACTAGGGGAATAAATCTTTGGGCACCTAGTGGTCATG

2 CATGACCACTAGGAGCATCTTTGGCGAGATCGGGAGAATCGGTGGTATTGATGTCTCCTAGGGGAATAAATCTTTGGGCACCTAGTGGTCATG

2 CATGACCACTAGGAGCATCTTTGGCGATGTGATCGACGCGCTGTAGGTTGCGGTAAGCTAGGGGAATAAATCTTTGGGCACCTAGTGGTCATG

2 CATGACCACTAGGAGCATCTTTGGCGACGTGGGTGGTCAAAGCAGTTTGGTTGTCGCCTAGGGGAATAAATCTTTGGGCACCTAGTGGTCATG

2 CATGACCACTAGGAGCATCTTTGGCGATGGGTGCTTACGGCCGCCGCTGCGGCTACACTAGGGGAATAAATCTTTGGGCACCTAGTGGTCATG

2 CATGACCACTAGGAGCATCTTTGGCGACGACTTTGGAGTGGGTGCGCCGCTGGCATACTAGGGGAATAAATCTTTGGGCACCTAGTGGTCATG

2 CATGACCACTAGGAGCATCTTTGGCGAACGGCAGGAGTTGCGGTGGTCTATGAATCCCTAGGGGAATAAATCTTTGGGCACCTAGTGGTCATG

2 CATGACCACTAGGAGCATCTTTGGCGAGTCTCGCTTGGGAGGTTGCTCCACCAGTTCCTAGGGGAATAAATCTTTGGGCACCTAGTGGTCATG

2 CATGACCACTAGGAGCATCTTTGGCGACCAGTGGATGTTAGTCCGACCCGCGAAGGTCTAGGGGAATAAATCTTTGGGCACCTAGTGGTCATG

2 CATGACCACTAGGAGCATCTTTGGCGAGATCTGGAGAATCGGTGGCGTTGGTGTCTCCTAGGGGAATAAATCTTTGGGCACCTAGTGGTCATG

2 CATGACCACTAGGAGCATCTTTGGCGAGATAGGGAGAATCGGTGTCATTGGTGTCTCCTAGGGGAATAAATCTTTGGGCACCTAGTGGTCATG

2 CATGACCACTAGGAGCATCTTTGGCGACAGTGGTCCGGGGCACTCGGAATGCTAAGACTAGGGGAATAAATCTTTGGGCACCTAGTGGTCATG

2 CATGACCACTAGGAGCATCTTTGGCGAGACATGATGCTTCGAGACCGACGTACAGGTCTAGGGGAATAAATCTTTGGGCACCTAGTGGTCATG

2 CATGACCACTAGGAGCATCTTTGGCGAAAGCGCAGGGCATGCGACCTCCAGTTGGTCCTAGGGGAATAAATCTTTGGGCACCTAGTGGTCATG

2 CATGACCACTAGGAGCATCTTTGGCGAATCGCCGGCACGGCCTTCTAGTCCTCGGTACTAGGGGAATAAATCTTTGGGCACCTAGTGGTCATG

2 CATGACCACTAGGAGCATCTTTGGCGATCCGGTGGCACCTATGTGCGACGCTGTGGGCTAGGGGAATAAATCTTTGGGCACCTAGTGGTCATG

2 CATGACCACTAGGAGCATCTTTGGCGATCGATTACTGGCTAACTAAAAGTCTACTGTCTAGGGGAATAAATCTTTGGGCACCTAGTGGTCATG

2 CATGACCACTAGGAGCATCTTTGGCGAGGGAGGGCGCCGGCACCGGTGTGAATGCGACTAGGGGAATAAATCTTTGGGCACCTAGTGGTCATG

2 CATGACCACTAGGAGCATCTTTGGCGAACTTGAACGTCGAAGTGGCAGAGAAAGCGACTAGGGGAATAAATCTTTGGGCACCTAGTGGTCATG

2 CATGACCACTAGGAGCATCTTTGGCGATCGCACAGCGCACGACTGCTTAGAAGGGGACTAGGGGAATAAATCTTTGGGCACCTAGTGGTCATG

2 CATGACCACTAGGAGCATCTTTGGCGATCGCAGAGCGGATGACGTGGAGGAACGGGACTAGGGGAATAAATCTTTGGGCACCTAGTGGTCATG

2 CATGACCACTAGGAGCATCTTTGGCGAGATCGGGAGAATCGGCGGCATTGTTGTCTCCTAGGGGAATAAATCTTTGGGCACCTAGTGGTCATG

2 CATGACCACTAGGAGCATCTTTGGCGACAGGTGCTGCCGGATGTACCTAAAAGTCGACTAGGGGAATAAATCTTTGGGCACCTAGTGGTCATG

2 CATGACCACTAGGAGCATCTTTGGCGACCGAACGACAGTGCGTTAAGTGGGGTAGATCTAGGGGAATAAATCTTTGGGCACCTAGTGGTCATG

2 CATGACCACTAGGAGCATCTTTGGCGAGATCGGGAGGATCGGTGGCGTTGGTGTCTCCTAGGGGAATAAATCTTTGGGCACCTAGTGGTCATG

2 CATGACCACTAGGAGCATCTTTGGCGAGATCGGGGGAATCGGTGGCACTGGTGTCTCCTAGGGGAATAAATCTTTGGGCACCTAGTGGTCATG

2 CATGACCACTAGGAGCATCTTTGGCGAGAAGACTCTGGATTCAAGGACCAGTTGCTGCTAGGGGAATAAATCTTTGGGCACCTAGTGGTCATG

2 CATGACCACTAGGAGCATCTTTGGCGATCGCACAGCGGGTAGAGTCATCGGACGGGACTAGGGGAATAAATCTTTGGGCACCTAGTGGTCATG

2 CATGACCACTAGGAGCATCTTTGGCGACCGGCTTGTCCTAATGTTAAAGTGTCGACGCTAGGGGAATAAATCTTTGGGCACCTAGTGGTCATG

2 CATGACCACTAGGAGCATCTTTGGCGAAAGGCGGTAGAACAGGGTGGGGTGCTGTCCCTAGGGGAATAAATCTTTGGGCACCTAGTGGTCATG

2 CATGACCACTAGGAGCATCTTTGGCGAACGGCAGGTGTTGCGGTGGTTTGTGTATCCCTAGGGGAATAAATCTTTGGGCACCTAGTGGTCATG

2 CATGACCACTAGGAGCATCTTTGGCGAAGACTCGTGGTTGGTTGTGTGGATCGATAGCTAGGGGAATAAATCTTTGGGCACCTAGTGGTCATG

2 CATGACCACTAGGAGCATCTTTGGCGAAGCAGTGCGACGGGCTATGGGTCCCTCGAGCTAGGGGAATAAATCTTTGGGCACCTAGTGGTCATG

2 CATGACCACTAGGAGCATCTTTGGCGAGATCGGGAGAGTCGGTGGCATTGGTGTCCCCTAGGGGAATAAATCTTTGGGCACCTAGTGGTCATG

2 CATGACCACTAGGAGCATCTTTGGCGAGATCGTGAGAATCGGTGGCATTGGTGTCTGCTAGGGGAATAAATCTTTGGGCACCTAGTGGTCATG

2 CATGACCACTAGGAGCATCTTTGGCGAGATCGGGAGAATCTGTGGCACTGGTGTCTCCTAGGGGAATAAATCTTTGGGCACCTAGTGGTCATG

2 CATGACCACTAGGAGCATCTTTGGCGACATCAGCTCTGCGGTCGGGGACGTAAGGGACTAGGGGAATAAATCTTTGGGCACCTAGTGGTCATG

2 CATGACCACTAGGAGCATCTTTGGCGATATCGGGAGAATCGGTGGCAATGGTGTCTCCTAGGGGAATAAATCTTTGGGCACCTAGTGGTCATG

2 CATGACCACTAGGAGCATCTTTGGCGAGATCGTCAGAATCGGTGGCATTGGTGTCTCCTAGGGGAATAAATCTTTGGGCACCTAGTGGTCATG

2 CATGACCACTAGGAGCATCTTTGGCGACTAACAGTGGTCAGGTACGGCACAGTGGAGCTAGGGGAATAAATCTTTGGGCACCTAGTGGTCATG

2 CATGACCACTAGGAGCATCTTTGGCGAGACCGGGAGAATCGGTGGCGTTGGTGTCTCCTAGGGGAATAAATCTTTGGGCACCTAGTGGTCATG

2 CATGACCACTAGGAGCATCTTTGGCGAGATCGGGAGAATCGGTTGCATTGGTTTCTCCTAGGGGAATAAATCTTTGGGCACCTAGTGGTCATG

2 CATGACCACTAGGAGCATCTTTGGCGAGATCGGGAGAATCTGTGGCATCGGTGTCTCCTAGGGGAATAAATCTTTGGGCACCTAGTGGTCATG

2 CATGACCACTAGGAGCATCTTTGGCGAGGTCGGGAGAATTGGTGGCATTGGTGTCTCCTAGGGGAATAAATCTTTGGGCACCTAGTGGTCATG

2 CATGACCACTAGGAGCATCTTTGGCGAGATAGGGAGAATCGGTGGCATTGGTGTCCCCTAGGGGAATAAATCTTTGGGCACCTAGTGGTCATG

2 CATGACCACTAGGAGCATCTTTGGCGAGGGAGGGCTCCGGCAGCGGTGTGAATGCGCCTAGGGGAATAAATCTTTGGGCACCTAGTGGTCATG

2 CATGACCACTAGGAGCATCTTTGGCGAACGGCAGGTGTTCCGGTGGTCTGTGAATCTCTAGGGGAATAAATCTTTGGGCACCTAGTGGTCATG

2 CATGACCACTAGGAGCATCTTTGGCGAACGGCAGGTGTTGCGGTGGTATGTGTATCCCTAGGGGAATAAATCTTTGGGCACCTAGTGGTCATG

2 CATGACCACTAGGAGCATCTTTGGCGAAGGGTGTAGGACTTCAAGTGGATCTCATGGCTAGGGGAATAAATCTTTGGGCACCTAGTGGTCATG

2 CATGACCACTAGGAGCATCTTTGGCGAGCTCACCCGGCATCTCTCGTACATTCGGGTCTAGGGGAATAAATCTTTGGGCACCTAGTGGTCATG

2 CATGACCACTAGGAGCATCTTTGGCGACGAGGTATGGTCAGCGATCTCCGAAGTGGACTAGGGGAATAAATCTTTGGGCACCTAGTGGTCATG

2 CATGACCACTAGGAGCATCTTTGGCGAGATCGGGAGAATAGGTGGCATTGGTGACTCCTAGGGGAATAAATCTTTGGGCACCTAGTGGTCATG

2 CATGACCACTAGGAGCATCTTTGGCGAGATCGGGAGAATCGGCGGCATTGGTGTGTCCTAGGGGAATAAATCTTTGGGCACCTAGTGGTCATG

2 CATGACCACTAGGAGCATCTTTGGCGAGATCGGGAGAATCGGTGGCATCCGTGTCTCCTAGGGGAATAAATCTTTGGGCACCTAGTGGTCATG

2 CATGACCACTAGGAGCATCTTTGGCGAGTTCGGGAGAATCGGTGGCATTGGTGTCTTCTAGGGGAATAAATCTTTGGGCACCTAGTGGTCATG

2 CATGACCACTAGGAGCATCTTTGGCGATGGAGGGCGCCGGCAGCGGTGTGAATGCGCCTAGGGGAATAAATCTTTGGGCACCTAGTGGTCATG

2 CATGACCACTAGGAGCATCTTTGGCGATCAGGCCGCCAGGGCGTACGAGGTGTGGTTCTAGGGGAATAAATCTTTGGGCACCTAGTGGTCATG

2 CATGACCACTAGGAGCATCTTTGGCGAGATCGGGAGAATCGGTGGCATTGGAGTCACCTAGGGGAATAAATCTTTGGGCACCTAGTGGTCATG

2 CATGACCACTAGGAGCATCTTTGGCGATCCGGGGAGGGCAGTGCCCTGACGCTTGGGCTAGGGGAATAAATCTTTGGGCACCTAGTGGTCATG

2 CATGACCACTAGGAGCATCTTTGGCGAGAAGACTTTGGATTCGGGGACCCGTTGCTGCTAGGGGAATAAATCTTTGGGCACCTAGTGGTCATG

2 CATGACCACTAGGAGCATCTTTGGCGACACAGTCGGCTGGTAGAATGTATTTGTGAGCTAGGGGAATAAATCTTTGGGCACCTAGTGGTCATG

2 CATGACCACTAGGAGCATCTTTGGCGAGAAGATTTTGGATTCGGGGACCAGTTGCTGCTAGGGGAATAAATCTTTGGGCACCTAGTGGTCATG

2 CATGACCACTAGGAGCATCTTTGGCGAACGGCAGGTGTTGCGGTGGGCTGTGGATCCCTAGGGGAATAAATCTTTGGGCACCTAGTGGTCATG

2 CATGACCACTAGGAGCATCTTTGGCGAGATCGGGAGAATCGGCGGCATTGGTGTCGCCTAGGGGAATAAATCTTTGGGCACCTAGTGGTCATG

2 CATGACCACTAGGAGCATCTTTGGCGAATGGCAGGTGTTGCGGTGGTCTGTGAAACCCTAGGGGAATAAATCTTTGGGCACCTAGTGGTCATG

2 CATGACCACTAGGAGCATCTTTGGCGACCGAGTGGGCTGGGAGGGAGTATTTCGGAGCTAGGGGAATAAATCTTTGGGCACCTAGTGGTCATG

2 CATGACCACTAGGAGCATCTTTGGCGATATCGGGAGAATCGGCGGCATTAGTGTCTCCTAGGGGAATAAATCTTTGGGCACCTAGTGGTCATG

2 CATGACCACTAGGAGCATCTTTGGCGAGTGGGGGGCGACGGCCGGTAGTGGGAGAGACTAGGGGAATAAATCTTTGGGCACCTAGTGGTCATG

2 CATGACCACTAGGAGCATCTTTGGCGAGATCGTGAGAATCGGTGCCATTGGTGTCTCCTAGGGGAATAAATCTTTGGGCACCTAGTGGTCATG

2 CATGACCACTAGGAGCATCTTTGGCGAGTGGGGTGCGACGGCCGGTAGTGGGTGAGACTAGGGGAATAAATCTTTGGGCACCTAGTGGTCATG

2 CATGACCACTAGGAGCATCTTTGGCGACGTTGTAAAGTGCGTAGAGTGGTAAGTCGACTAGGGGAATAAATCTTTGGGCACCTAGTGGTCATG

2 CATGACCACTAGGAGCATCTTTGGCGAGTGTGGGGCGACGGCCGGTAGTGGGTGAGACTAGGGGAATAAATCTTTGGGCACCTAGTGGTCATG

2 CATGACCACTAGGAGCATCTTTGGCGAGATCGGGAGAATCGGTTGCATTGGTGACTCCTAGGGGAATAAATCTTTGGGCACCTAGTGGTCATG

2 CATGACCACTAGGAGCATCTTTGGCGAGATCGGGAGAAACGGTGGCATTGTTGTCTCCTAGGGGAATAAATCTTTGGGCACCTAGTGGTCATG

2 CATGACCACTAGGAGCATCTTTGGCGAGAACGGGAGAATCGGTGGCATTGGTGCCTCCTAGGGGAATAAATCTTTGGGCACCTAGTGGTCATG

2 CATGACCACTAGGAGCATCTTTGGCGAGATTGGGAGAATCGGTAGCATTGGTGTCTCCTAGGGGAATAAATCTTTGGGCACCTAGTGGTCATG

2 CATGACCACTAGGAGCATCTTTGGCGAAAGTTGGTAGAACATGGTGGGGTGCTGTCCCTAGGGGAATAAATCTTTGGGCACCTAGTGGTCATG

2 CATGACCACTAGGAGCATCTTTGGCGACGATGTGGGCAGGCGGTTCGAAATCGGAGACTAGGGGAATAAATCTTTGGGCACCTAGTGGTCATG

2 CATGACCACTAGGAGCATCTTTGGCGAGACTCGCTTGGGAGGTTGCTCCACCAGTTCCTAGGGGAATAAATCTTTGGGCACCTAGTGGTCATG

2 CATGACCACTAGGAGCATCTTTGGCGAGGCGAGTCCAGCGGCCCTCTTGGCCTGCGACTAGGGGAATAAATCTTTGGGCACCTAGTGGTCATG

2 CATGACCACTAGGAGCATCTTTGGCGATCAAGTGCGCTTGGTAATTCTTGATGCGATCTAGGGGAATAAATCTTTGGGCACCTAGTGGTCATG

2 CATGACCACTAGGAGCATCTTTGGCGAACGGCAGGTGTTGCGGGGGTCTGTGAATCACTAGGGGAATAAATCTTTGGGCACCTAGTGGTCATG

2 CATGACCACTAGGAGCATCTTTGGCGAGGGAGGGCGCCGGCAGCGGCGTGAATGCGACTAGGGGAATAAATCTTTGGGCACCTAGTGGTCATG

2 CATGACCACTAGGAGCATCTTTGGCGATTCCTGATGGGACTTCCCGGTTCGTCTCTTCTAGGGGAATAAATCTTTGGGCACCTAGTGGTCATG

2 CATGACCACTAGGAGCATCTTTGGCGAGATCGGGAGAATCGGTGGCATTGTTGCCTCCTAGGGGAATAAATCTTTGGGCACCTAGTGGTCATG

2 CATGACCACTAGGAGCATCTTTGGCGAGATCGGGAGAATCGGTTGCATAGGTGTCTCCTAGGGGAATAAATCTTTGGGCACCTAGTGGTCATG

2 CATGACCACTAGGAGCATCTTTGGCGAGGGGCCATGGGAATGTAATTGCGCTTAAGACTAGGGGAATAAATCTTTGGGCACCTAGTGGTCATG

2 CATGACCACTAGGAGCATCTTTGGCGAACGGCAGGGGTTGCAGTGGTCTGTGAATCCCTAGGGGAATAAATCTTTGGGCACCTAGTGGTCATG

2 CATGACCACTAGGAGCATCTTTGGCGACGGTGTGGGGCCTCTAGCGCGGCGGGGATCCTAGGGGAATAAATCTTTGGGCACCTAGTGGTCATG

2 CATGACCACTAGGAGCATCTTTGGCGACTTGTGGCTGAGGCATGCTAGAAGCATCGACTAGGGGAATAAATCTTTGGGCACCTAGTGGTCATG

2 CATGACCACTAGGAGCATCTTTGGCGATCGCATAGCGGATGTCTGTCGTGCACGGGCCTAGGGGAATAAATCTTTGGGCACCTAGTGGTCATG

2 CATGACCACTAGGAGCATCTTTGGCGAGATCGAGAGAATCGGTGGCATAGGTGTCTCCTAGGGGAATAAATCTTTGGGCACCTAGTGGTCATG

2 CATGACCACTAGGAGCATCTTTGGCGAGAATGGGAGAATCGGTGGCATTGGTGTCTCCTAGGGGAATAAATCTTTGGGCACCTAGTGGTCATG

2 CATGACCACTAGGAGCATCTTTGGCGATAGGCGTGTACGGTCGAGCGTAAGCGATAGCTAGGGGAATAAATCTTTGGGCACCTAGTGGTCATG

2 CATGACCACTAGGAGCATCTTTGGCGAGATCGCGAGAATCGGTGGCCTTGGTGTCTCCTAGGGGAATAAATCTTTGGGCACCTAGTGGTCATG

2 CATGACCACTAGGAGCATCTTTGGCGAAGCGTGTTGTAAGGTCCGCGAGTGTGGACCCTAGGGGAATAAATCTTTGGGCACCTAGTGGTCATG

2 CATGACCACTAGGAGCATCTTTGGCGAGATCTCGAGAATCGGTGGCATTGGTGTCTCCTAGGGGAATAAATCTTTGGGCACCTAGTGGTCATG

2 CATGACCACTAGGAGCATCTTTGGCGACGAGGGGGCGTGAGATGTGGGGAACGGAGACTAGGGGAATAAATCTTTGGGCACCTAGTGGTCATG

2 CATGACCACTAGGAGCATCTTTGGCGATCGCATAGCGAAGGGACATGGACCCTGGGACTAGGGGAATAAATCTTTGGGCACCTAGTGGTCATG

2 CATGACCACTAGGAGCATCTTTGGCGAACGGCAGGTGTTGCGGTGGTCTGAGGATCCCTAGGGGAATAAATCTTTGGGCACCTAGTGGTCATG

2 CATGACCACTAGGAGCATCTTTGGCGACTCGTGAGGCGAACAAAATGGTTGCGGGCGCTAGGGGAATAAATCTTTGGGCACCTAGTGGTCATG

2 CATGACCACTAGGAGCATCTTTGGCGAAGGGGGTCTCGGGGTGATAACGATGTAGCCCTAGGGGAATAAATCTTTGGGCACCTAGTGGTCATG

2 CATGACCACTAGGAGCATCTTTGGCGAACTAGGGGGAAGTGTAAATGCCTTCGAAGACTAGGGGAATAAATCTTTGGGCACCTAGTGGTCATG

2 CATGACCACTAGGAGCATCTTTGGCGAGTAGTGGCTGAGCAGTTAAACAACTCGAGGCTAGGGGAATAAATCTTTGGGCACCTAGTGGTCATG

2 CATGACCACTAGGAGCATCTTTGGCGAACGTAGAACGGGGGGCAGATTACTCGTAGACTAGGGGAATAAATCTTTGGGCACCTAGTGGTCATG

2 CATGACCACTAGGAGCATCTTTGGCGATGTGGGCTGGAAGGATGTGGTACTTCTCGACTAGGGGAATAAATCTTTGGGCACCTAGTGGTCATG

2 CATGACCACTAGGAGCATCTTTGGCGACGGCGTGGCTGGCACCAATAGTTTACGCACCTAGGGGAATAAATCTTTGGGCACCTAGTGGTCATG

2 CATGACCACTAGGAGCATCTTTGGCGACGTGGGTTGGCTGGTAGTATTGCGTGCGCTCTAGGGGAATAAATCTTTGGGCACCTAGTGGTCATG

2 CATGACCACTAGGAGCATCTTTGGCGACTAATTTCGCTTTCGAAATGCGACGTAGTGCTAGGGGAATAAATCTTTGGGCACCTAGTGGTCATG

2 CATGACCACTAGGAGCATCTTTGGCGAGATCGGGAGAATCGGTTGAATTGGTGTCTCCTAGGGGAATAAATCTTTGGGCACCTAGTGGTCATG

2 CATGACCACTAGGAGCATCTTTGGCGATAGCGCAGGGCATGCGACCTCCAATTGGTCCTAGGGGAATAAATCTTTGGGCACCTAGTGGTCATG

2 CATGACCACTAGGAGCATCTTTGGCGACTCAGTGGGCGAAATGCCCTGGAAGTGGAGCTAGGGGAATAAATCTTTGGGCACCTAGTGGTCATG

2 CATGACCACTAGGAGCATCTTTGGCGAAGCGCGGGGAGGACAATGTGGCCGAGTTGGCTAGGGGAATAAATCTTTGGGCACCTAGTGGTCATG

2 CATGACCACTAGGAGCATCTTTGGCGAGCAAGCGCGGTCCCCCGGACCTCACAGTTGCTAGGGGAATAAATCTTTGGGCACCTAGTGGTCATG

2 CATGACCACTAGGAGCATCTTTGGCGATGGGTGCTGACGACCGTCCATACGGTTACTCTAGGGGAATAAATCTTTGGGCACCTAGTGGTCATG

2 CATGACCACTAGGAGCATCTTTGGCGACAGCCGTTGGGGGCGCAAGGTAGGGGGCGACTAGGGGAATAAATCTTTGGGCACCTAGTGGTCATG

2 CATGACCACTAGGAGCATCTTTGGCGAATGGCAGGTGTTGCGGTGGTCTGTGAATTCCTAGGGGAATAAATCTTTGGGCACCTAGTGGTCATG

2 CATGACCACTAGGAGCATCTTTGGCGAGAGAATCGGTAGGCAGAGTGCTCCAGCTAGCTAGGGGAATAAATCTTTGGGCACCTAGTGGTCATG

2 CATGACCACTAGGAGCATCTTTGGCGAGATCGGGAGAATTGGTGGCATTGGCGTCTCCTAGGGGAATAAATCTTTGGGCACCTAGTGGTCATG

2 CATGACCACTAGGAGCATCTTTGGCGAAACGTGCCCGGGCGCGCTCGTATAAAGGGGCTAGGGGAATAAATCTTTGGGCACCTAGTGGTCATG

2 CATGACCACTAGGAGCATCTTTGGCGACGGATAGCGACACGAGGCTAATGGGATGGCCTAGGGGAATAAATCTTTGGGCACCTAGTGGTCATG

2 CATGACCACTAGGAGCATCTTTGGCGAGATCGGGAGAATCGTTTGCATTGGTGTCTCCTAGGGGAATAAATCTTTGGGCACCTAGTGGTCATG

2 CATGACCACTAGGAGCATCTTTGGCGAGATCGGGAGAATCGGTGGCATCGATGTCTCCTAGGGGAATAAATCTTTGGGCACCTAGTGGTCATG

2 CATGACCACTAGGAGCATCTTTGGCGAGGGAGGGCGCCGGCAGCGGAGTGAATGCGACTAGGGGAATAAATCTTTGGGCACCTAGTGGTCATG

2 CATGACCACTAGGAGCATCTTTGGCGAGTGGGGGGCGACGGCCGCTAGTGGGTGAGACTAGGGGAATAAATCTTTGGGCACCTAGTGGTCATG

2 CATGACCACTAGGAGCATCTTTGGCGACCCGTTAGGTGTGGAATTAGGAAGTGTCGACTAGGGGAATAAATCTTTGGGCACCTAGTGGTCATG

2 CATGACCACTAGGAGCATCTTTGGCGACCAAGGCGCACTTAATCATGTGGACTTCTTCTAGGGGAATAAATCTTTGGGCACCTAGTGGTCATG

2 CATGACCACTAGGAGCATCTTTGGCGACGGTGTGGGGCCATCTGCGCGGCGGATCCCCTAGGGGAATAAATCTTTGGGCACCTAGTGGTCATG

2 CATGACCACTAGGAGCATCTTTGGCGAAGTTCAATTTCAACAAACATATAGGTAAAGCTAGGGGAATAAATCTTTGGGCACCTAGTGGTCATG

2 CATGACCACTAGGAGCATCTTTGGCGATCAGGGGGCCTCTGATGGCGACTCAGTGGGCTAGGGGAATAAATCTTTGGGCACCTAGTGGTCATG

2 CATGACCACTAGGAGCATCTTTGGCGAGATCGGCGGAATCGGTGGCATTGGTGTCTCCTAGGGGAATAAATCTTTGGGCACCTAGTGGTCATG

2 CATGACCACTAGGAGCATCTTTGGCGAGATCGGGAGAATCGGAGGCATTAGTGTCTCCTAGGGGAATAAATCTTTGGGCACCTAGTGGTCATG

2 CATGACCACTAGGAGCATCTTTGGCGAGATTGGGAGTATCGGTGGCATTGGTGTCTCCTAGGGGAATAAATCTTTGGGCACCTAGTGGTCATG

2 CATGACCACTAGGAGCATCTTTGGCGAGATCCGTAGAATCGGTGGCATTGGTGTCTCCTAGGGGAATAAATCTTTGGGCACCTAGTGGTCATG

2 CATGACCACTAGGAGCATCTTTGGCGAAATCGGGAGAATCGGCGGCATTGGTGTCTCCTAGGGGAATAAATCTTTGGGCACCTAGTGGTCATG

2 CATGACCACTAGGAGCATCTTTGGCGACTTGTCGAGCCGTTCGGCGTTGAGGAAGTGCTAGGGGAATAAATCTTTGGGCACCTAGTGGTCATG

2 CATGACCACTAGGAGCATCTTTGGCGAGGGAGGACGCCGGCAGCGGTGTGAATGCGACTAGGGGAATAAATCTTTGGGCACCTAGTGGTCATG

2 CATGACCACTAGGAGCATCTTTGGCGACGAGGGGCACGGGTGGTTATTGCGAGAAGACTAGGGGAATAAATCTTTGGGCACCTAGTGGTCATG

2 CATGACCACTAGGAGCATCTTTGGCGACAGGGGATGCCGATGCGCTGGACCACCGAGCTAGGGGAATAAATCTTTGGGCACCTAGTGGTCATG

2 CATGACCACTAGGAGCATCTTTGGCGAGATCGCGAGAATCGGTGGCATTAGTGTCTCCTAGGGGAATAAATCTTTGGGCACCTAGTGGTCATG

2 CATGACCACTAGGAGCATCTTTGGCGATCTAGTGGATACGCGTTATCATTGGGAAGGCTAGGGGAATAAATCTTTGGGCACCTAGTGGTCATG

2 CATGACCACTAGGAGCATCTTTGGCGATTTGGCTGGTAGGTCGTACTGGGTAGCATACTAGGGGAATAAATCTTTGGGCACCTAGTGGTCATG

2 CATGACCACTAGGAGCATCTTTGGCGAGAAGACTTTGGATTCGGGGACCAGTTGCTTCTAGGGGAATAAATCTTTGGGCACCTAGTGGTCATG

2 CATGACCACTAGGAGCATCTTTGGCGAACGGCAGGTGTTGCGGTGGTCTGTGAAATCCTAGGGGAATAAATCTTTGGGCACCTAGTGGTCATG

2 CATGACCACTAGGAGCATCTTTGGCGAGAAGTGGCTGAGGGCGATGGGTAGGTCGCGCTAGGGGAATAAATCTTTGGGCACCTAGTGGTCATG

2 CATGACCACTAGGAGCATCTTTGGCGAGATCTGGAGAATCGGTGGAATTGGTGTCTCCTAGGGGAATAAATCTTTGGGCACCTAGTGGTCATG

2 CATGACCACTAGGAGCATCTTTGGCGATCCGGGGGCCAAAGGCTACGCAAATTAGGGCTAGGGGAATAAATCTTTGGGCACCTAGTGGTCATG

2 CATGACCACTAGGAGCATCTTTGGCGAGAAGACTCCGTATTCGGGGACCAGTTGCTGCTAGGGGAATAAATCTTTGGGCACCTAGTGGTCATG

2 CATGACCACTAGGAGCATCTTTGGCGAGATCAGGAGAGTCGGTGGCATTGGTGTCTCCTAGGGGAATAAATCTTTGGGCACCTAGTGGTCATG

2 CATGACCACTAGGAGCATCTTTGGCGAGAAGACTCTGGATTTGGGGACCAGTTGTTGCTAGGGGAATAAATCTTTGGGCACCTAGTGGTCATG

2 CATGACCACTAGGAGCATCTTTGGCGAGATCGGAAGAATCGGCGGCATTGGTGTCTCCTAGGGGAATAAATCTTTGGGCACCTAGTGGTCATG

2 CATGACCACTAGGAGCATCTTTGGCGATTCGGGGGGCAGAGAGGCCGACGCACTGGGCTAGGGGAATAAATCTTTGGGCACCTAGTGGTCATG

2 CATGACCACTAGGAGCATCTTTGGCGAGTGGCGCTCTGGGTGAGACCTTCATTGGTCCTAGGGGAATAAATCTTTGGGCACCTAGTGGTCATG

2 CATGACCACTAGGAGCATCTTTGGCGAGCTATGGATATGCTTCGAGGTACGGGAAGGCTAGGGGAATAAATCTTTGGGCACCTAGTGGTCATG

2 CATGACCACTAGGAGCATCTTTGGCGACCGGGGATGCCGATGCGCTGGACGACCGAGCTAGGGGAATAAATCTTTGGGCACCTAGTGGTCATG

2 CATGACCACTAGGAGCATCTTTGGCGATGCGAATTTCATAAGTGTGTTAGAGAGAATCTAGGGGAATAAATCTTTGGGCACCTAGTGGTCATG

2 CATGACCACTAGGAGCATCTTTGGCGAGATCGGGAGAATCGGTGGCACTGGTGTCCCCTAGGGGAATAAATCTTTGGGCACCTAGTGGTCATG

2 CATGACCACTAGGAGCATCTTTGGCGATCCGGGGGCACCTATGCGCGACGCTGTGGGCTAGGGGAATAAATCTTTGGGCACCTAGTGGTCATG

2 CATGACCACTAGGAGCATCTTTGGCGAACGGCAGGTGTTGCGGTAATCTGTGAACCCCTAGGGGAATAAATCTTTGGGCACCTAGTGGTCATG

2 CATGACCACTAGGAGCATCTTTGGCGACCCGCGTAGCAAGCTCTGATGCGACCTGTGCTAGGGGAATAAATCTTTGGGCACCTAGTGGTCATG

2 CATGACCACTAGGAGCATCTTTGGCGAGCCGATTGTGCAACAATGTGTCCTGGGTAACTAGGGGAATAAATCTTTGGGCACCTAGTGGTCATG

2 CATGACCACTAGGAGCATCTTTGGCGAAGACGACACAGTCGATCGGGGTAGAGGAAGCTAGGGGAATAAATCTTTGGGCACCTAGTGGTCATG

2 CATGACCACTAGGAGCATCTTTGGCGAGAGCTAGGGTCTCCCAATGTCTGCCCTCCTCTAGGGGAATAAATCTTTGGGCACCTAGTGGTCATG

2 CATGACCACTAGGAGCATCTTTGGCGAGACAATACGTCGAGTTCGTCGTGCCGGCATCTAGGGGAATAAATCTTTGGGCACCTAGTGGTCATG

2 CATGACCACTAGGAGCATCTTTGGCGAGATCGTGAGAATCGGTGGCATTGGTATCTCCTAGGGGAATAAATCTTTGGGCACCTAGTGGTCATG

2 CATGACCACTAGGAGCATCTTTGGCGAGATCGGGGGAATCGGTGGCATTGGTTTCTCCTAGGGGAATAAATCTTTGGGCACCTAGTGGTCATG

2 CATGACCACTAGGAGCATCTTTGGCGATACTTGTGGGCTTTTGTAGCTAGCGCGTACCTAGGGGAATAAATCTTTGGGCACCTAGTGGTCATG

2 CATGACCACTAGGAGCATCTTTGGCGAGGTTGGGAGAATCGGTGGCATTGGTGTCTCCTAGGGGAATAAATCTTTGGGCACCTAGTGGTCATG

2 CATGACCACTAGGAGCATCTTTGGCGATTAGCACGGCGGGAGTTGCTAAACGGGGGCCTAGGGGAATAAATCTTTGGGCACCTAGTGGTCATG

2 CATGACCACTAGGAGCATCTTTGGCGATCCGGGGGGAGCGTAGATCCGACGCTTGGGCTAGGGGAATAAATCTTTGGGCACCTAGTGGTCATG

2 CATGACCACTAGGAGCATCTTTGGCGATGGGTGCTGACGGCCGCCGCAGCGGCTACACTAGGGGAATAAATCTTTGGGCACCTAGTGGTCATG

2 CATGACCACTAGGAGCATCTTTGGCGAGATCGGGAGAATCGGTGGCATTGGCGTCCCCTAGGGGAATAAATCTTTGGGCACCTAGTGGTCATG

2 CATGACCACTAGGAGCATCTTTGGCGATCGCACAGCGGGGATCGGGAGAGTACGGGCCTAGGGGAATAAATCTTTGGGCACCTAGTGGTCATG

2 CATGACCACTAGGAGCATCTTTGGCGACTGAAATGGCTGCCCGGAGGGGACATTGTCCTAGGGGAATAAATCTTTGGGCACCTAGTGGTCATG

2 CATGACCACTAGGAGCATCTTTGGCGACCGTAGGAAAACAAAAGTGGCTTTGCTAGCCTAGGGGAATAAATCTTTGGGCACCTAGTGGTCATG

2 CATGACCACTAGGAGCATCTTTGGCGATCGCAGTGGCAACGGGTAGCGGTACCGGGCCTAGGGGAATAAATCTTTGGGCACCTAGTGGTCATG

2 CATGACCACTAGGAGCATCTTTGGCGAGACCGGGGGAATCGGTGGCATTGGTGTCTCCTAGGGGAATAAATCTTTGGGCACCTAGTGGTCATG

2 CATGACCACTAGGAGCATCTTTGGCGAGAACGGGAGAATCGGTGGCATTGGTGACTCCTAGGGGAATAAATCTTTGGGCACCTAGTGGTCATG

2 CATGACCACTAGGAGCATCTTTGGCGAGATCGGGAGAATCGGAGGCATTGGTGCCTCCTAGGGGAATAAATCTTTGGGCACCTAGTGGTCATG

2 CATGACCACTAGGAGCATCTTTGGCGAGATTGGGAGAATCGGTGGCATTTGTGTCTCCTAGGGGAATAAATCTTTGGGCACCTAGTGGTCATG

2 CATGACCACTAGGAGCATCTTTGGCGAGATCGGGAGAATCGGTTGTATTGGTGTCTCCTAGGGGAATAAATCTTTGGGCACCTAGTGGTCATG

2 CATGACCACTAGGAGCATCTTTGGCGAGGGGTCATGGGAATGTAAATGCGCTTAAGACTAGGGGAATAAATCTTTGGGCACCTAGTGGTCATG

2 CATGACCACTAGGAGCATCTTTGGCGACTGTGGCTACGTCCCGGACTTAGTAAGCGCCTAGGGGAATAAATCTTTGGGCACCTAGTGGTCATG

2 CATGACCACTAGGAGCATCTTTGGCGAGATCGGGAGACTCGGTGTCATTGGTGTCTCCTAGGGGAATAAATCTTTGGGCACCTAGTGGTCATG

2 CATGACCACTAGGAGCATCTTTGGCGATCAGGGGGCACCTATGTGCGACGCTGTGGGCTAGGGGAATAAATCTTTGGGCACCTAGTGGTCATG

2 CATGACCACTAGGAGCATCTTTGGCGAGTTCGTAAGGCCGCGCTCCTTGTGGCCGTGCTAGGGGAATAAATCTTTGGGCACCTAGTGGTCATG

2 CATGACCACTAGGAGCATCTTTGGCGAACGGCAGGTGTTGCGGTGGTCCGTGGATCCCTAGGGGAATAAATCTTTGGGCACCTAGTGGTCATG

2 CATGACCACTAGGAGCATCTTTGGCGATCCGACACGGCGTGGTAGTCAAGGTAGCGACTAGGGGAATAAATCTTTGGGCACCTAGTGGTCATG

2 CATGACCACTAGGAGCATCTTTGGCGAGCCTCGCTTGGGGGGTTGCTCCACCAGTTCCTAGGGGAATAAATCTTTGGGCACCTAGTGGTCATG

2 CATGACCACTAGGAGCATCTTTGGCGACGAGTGGGCGGAGCTTAACGCGTCATAGTGCTAGGGGAATAAATCTTTGGGCACCTAGTGGTCATG

2 CATGACCACTAGGAGCATCTTTGGCGATATCGCGAGAATCGGTGGCATTGGTGTCTCCTAGGGGAATAAATCTTTGGGCACCTAGTGGTCATG

2 CATGACCACTAGGAGCATCTTTGGCGAGATCGGGGGAATAGGTGGCATTGGTGTCTCCTAGGGGAATAAATCTTTGGGCACCTAGTGGTCATG

2 CATGACCACTAGGAGCATCTTTGGCGACAAGGGATGCCGATGCGCTGGACGACTGAGCTAGGGGAATAAATCTTTGGGCACCTAGTGGTCATG

2 CATGACCACTAGGAGCATCTTTGGCGATCCGGGGGCACCTATGTGCGACGTTGTGGGCTAGGGGAATAAATCTTTGGGCACCTAGTGGTCATG

2 CATGACCACTAGGAGCATCTTTGGCGAGATCGGGAGAAACGGTGTCATTGGTGTCTCCTAGGGGAATAAATCTTTGGGCACCTAGTGGTCATG

2 CATGACCACTAGGAGCATCTTTGGCGAAATTTTCGCGTTTGAGATCATACTCTTTGGCTAGGGGAATAAATCTTTGGGCACCTAGTGGTCATG

2 CATGACCACTAGGAGCATCTTTGGCGAACGACGTAGATGCAATAAAAGTCTTGGAACCTAGGGGAATAAATCTTTGGGCACCTAGTGGTCATG

2 CATGACCACTAGGAGCATCTTTGGCGATCCGGGGGTCACATTAGGACGACGCTAGGGCTAGGGGAATAAATCTTTGGGCACCTAGTGGTCATG

2 CATGACCACTAGGAGCATCTTTGGCGAATGGCCGGCACGGCCATCTAGTCCTCGGTACTAGGGGAATAAATCTTTGGGCACCTAGTGGTCATG

2 CATGACCACTAGGAGCATCTTTGGCGAATGCGGGCAACACGCAGTCCTGGTGTGCGACTAGGGGAATAAATCTTTGGGCACCTAGTGGTCATG

2 CATGACCACTAGGAGCATCTTTGGCGAGATCGGGCGACTCGGCGGCATTGGTGTCTCCTAGGGGAATAAATCTTTGGGCACCTAGTGGTCATG

2 CATGACCACTAGGAGCATCTTTGGCGATGAAATCTGATAAGATGCGTAACGGTGTCGCTAGGGGAATAAATCTTTGGGCACCTAGTGGTCATG

2 CATGACCACTAGGAGCATCTTTGGCGAACGGCAGGTGTTGCGGTGGTCAGTGGATCCCTAGGGGAATAAATCTTTGGGCACCTAGTGGTCATG

2 CATGACCACTAGGAGCATCTTTGGCGAGATCGGGATAATCGGTGGCATTGGTGTCTACTAGGGGAATAAATCTTTGGGCACCTAGTGGTCATG

2 CATGACCACTAGGAGCATCTTTGGCGATCCTCGTCAGATGGAGAAGCAGACGTTTGGCTAGGGGAATAAATCTTTGGGCACCTAGTGGTCATG

2 CATGACCACTAGGAGCATCTTTGGCGAGATCGTGAGAATCGTTGGCATTGGTGTCTCCTAGGGGAATAAATCTTTGGGCACCTAGTGGTCATG

2 CATGACCACTAGGAGCATCTTTGGCGAGAAGACTTTGGATTCGGGGACCAGTTGCCGCTAGGGGAATAAATCTTTGGGCACCTAGTGGTCATG

2 CATGACCACTAGGAGCATCTTTGGCGAGATCGTGAGAATCGGTGGCATTGGTTTCTCCTAGGGGAATAAATCTTTGGGCACCTAGTGGTCATG

2 CATGACCACTAGGAGCATCTTTGGCGACCCGGCAAAACGTGTTATTGGCAGGGTGGACTAGGGGAATAAATCTTTGGGCACCTAGTGGTCATG

2 CATGACCACTAGGAGCATCTTTGGCGAGGGGTCATGGGAACGTAATTGCGCTTAAGACTAGGGGAATAAATCTTTGGGCACCTAGTGGTCATG

2 CATGACCACTAGGAGCATCTTTGGCGAGATCGGGAGAATCGGGGGCGTTGGTGTCTCCTAGGGGAATAAATCTTTGGGCACCTAGTGGTCATG

2 CATGACCACTAGGAGCATCTTTGGCGAGAAGACTCTGGATTCGGGGATTAGTTGCTCCTAGGGGAATAAATCTTTGGGCACCTAGTGGTCATG

2 CATGACCACTAGGAGCATCTTTGGCGAACGGTAGGTGTTGCGGTGGTCTGTGGATCCCTAGGGGAATAAATCTTTGGGCACCTAGTGGTCATG

2 CATGACCACTAGGAGCATCTTTGGCGACCAGTGAAGTAAGGAGGATTACGCCCAGTGCTAGGGGAATAAATCTTTGGGCACCTAGTGGTCATG

2 CATGACCACTAGGAGCATCTTTGGCGACCCATGCGAACGTAAGGGAGCAAGGTGTGACTAGGGGAATAAATCTTTGGGCACCTAGTGGTCATG

2 CATGACCACTAGGAGCATCTTTGGCGATGGCGTGGGGCCATCTGTGCGGCGGATCCCCTAGGGGAATAAATCTTTGGGCACCTAGTGGTCATG

2 CATGACCACTAGGAGCATCTTTGGCGAGCTCGGGAGGATCGGTGGCATTGGTGTCTCCTAGGGGAATAAATCTTTGGGCACCTAGTGGTCATG

2 CATGACCACTAGGAGCATCTTTGGCGAGTCGCGATTAGTTATCAGCAGTGCTACCCCCTAGGGGAATAAATCTTTGGGCACCTAGTGGTCATG

2 CATGACCACTAGGAGCATCTTTGGCGAATCTGTCCGATGGGTCGGAAATGTACCGGGCTAGGGGAATAAATCTTTGGGCACCTAGTGGTCATG

2 CATGACCACTAGGAGCATCTTTGGCGAGATCGGCTGGTAGAGACTGTATTGAGGAACCTAGGGGAATAAATCTTTGGGCACCTAGTGGTCATG

2 CATGACCACTAGGAGCATCTTTGGCGATGGGGGGCGCCGGCAGCGGTGTGAATGCGACTAGGGGAATAAATCTTTGGGCACCTAGTGGTCATG

2 CATGACCACTAGGAGCATCTTTGGCGAGTGGGGGGCGACGGCCGGTAGTGGGTGAGTCTAGGGGAATAAATCTTTGGGCACCTAGTGGTCATG

2 CATGACCACTAGGAGCATCTTTGGCGACAATGGAGCGACGGTGTGGTCTGAGTCCAACTAGGGGAATAAATCTTTGGGCACCTAGTGGTCATG

2 CATGACCACTAGGAGCATCTTTGGCGACCGTAGGGAAACAAAAGTGGCCTTGCTAGCCTAGGGGAATAAATCTTTGGGCACCTAGTGGTCATG

2 CATGACCACTAGGAGCATCTTTGGCGAATGGCCGGCACGGCCTTCTAGACCTCGGTACTAGGGGAATAAATCTTTGGGCACCTAGTGGTCATG

2 CATGACCACTAGGAGCATCTTTGGCGACAAGCTATCTGCGATGAGCGCGACATAGTGCTAGGGGAATAAATCTTTGGGCACCTAGTGGTCATG

2 CATGACCACTAGGAGCATCTTTGGCGAAGTGCGGTGGGCATGTGGGTGGTAGTGACGCTAGGGGAATAAATCTTTGGGCACCTAGTGGTCATG

2 CATGACCACTAGGAGCATCTTTGGCGAGATCGGGACAATCGGCGGCATTGGTGTCTCCTAGGGGAATAAATCTTTGGGCACCTAGTGGTCATG

2 CATGACCACTAGGAGCATCTTTGGCGAGGGTGGACGCCGGCAGCGGTGTGAATGCGACTAGGGGAATAAATCTTTGGGCACCTAGTGGTCATG

2 CATGACCACTAGGAGCATCTTTGGCGAGATCGGGAGAATCGCTGGCATTTGTGTCTCCTAGGGGAATAAATCTTTGGGCACCTAGTGGTCATG

2 CATGACCACTAGGAGCATCTTTGGCGAAGCAGTGTGACGGGCTATGGGTTCCTCGAGCTAGGGGAATAAATCTTTGGGCACCTAGTGGTCATG

2 CATGACCACTAGGAGCATCTTTGGCGAGATCGGGCGAATCGGTGGCAGTGGTGTCTCCTAGGGGAATAAATCTTTGGGCACCTAGTGGTCATG

2 CATGACCACTAGGAGCATCTTTGGCGAAGGGTGTAGGACTTCAAGTGGATCTCAAAGCTAGGGGAATAAATCTTTGGGCACCTAGTGGTCATG

2 CATGACCACTAGGAGCATCTTTGGCGATTAGCGTCTTGCGAGCGGGTGGGTTGCTCCCTAGGGGAATAAATCTTTGGGCACCTAGTGGTCATG

2 CATGACCACTAGGAGCATCTTTGGCGAGGGTATACACGGCCGACCAGATTCGAATTGCTAGGGGAATAAATCTTTGGGCACCTAGTGGTCATG

2 CATGACCACTAGGAGCATCTTTGGCGACGGCGTGGGGTTATTGATGCGGCGGCACTCCTAGGGGAATAAATCTTTGGGCACCTAGTGGTCATG

2 CATGACCACTAGGAGCATCTTTGGCGATCGCACAGCGTACGAACATCGAGGAGGGGTCTAGGGGAATAAATCTTTGGGCACCTAGTGGTCATG

2 CATGACCACTAGGAGCATCTTTGGCGACGACGAGGTCTGGGGTCAAGAGAAGTCGATCTAGGGGAATAAATCTTTGGGCACCTAGTGGTCATG

2 CATGACCACTAGGAGCATCTTTGGCGAACGGCAGGTGTTGCTGTGGTCTGTGAATCACTAGGGGAATAAATCTTTGGGCACCTAGTGGTCATG

2 CATGACCACTAGGAGCATCTTTGGCGAGATCGGGAGAATCGGTGGCATTGGCGTCTGCTAGGGGAATAAATCTTTGGGCACCTAGTGGTCATG

2 CATGACCACTAGGAGCATCTTTGGCGAGATCGGGAGAATCGGTGGCGTTGGTGTCTTCTAGGGGAATAAATCTTTGGGCACCTAGTGGTCATG

2 CATGACCACTAGGAGCATCTTTGGCGAGATCGGGAGAATCGGTGGTATTGGTGTCCCCTAGGGGAATAAATCTTTGGGCACCTAGTGGTCATG

2 CATGACCACTAGGAGCATCTTTGGCGAGATCGGGAGGATCGTTGGCATTGGTGTCTCCTAGGGGAATAAATCTTTGGGCACCTAGTGGTCATG

2 CATGACCACTAGGAGCATCTTTGGCGACGGCGTGGGGCCATCTGCGCGGCGGATCCCCTAGGGGAATAAATCTTTGGGCACCTAGTGGTCATG

2 CATGACCACTAGGAGCATCTTTGGCGACCGATCGTCAGGTTGGAGACCGAGGGGCTACTAGGGGAATAAATCTTTGGGCACCTAGTGGTCATG

2 CATGACCACTAGGAGCATCTTTGGCGACCGGCCCAGGCGATTACACCCGGGAAGGAGCTAGGGGAATAAATCTTTGGGCACCTAGTGGTCATG

2 CATGACCACTAGGAGCATCTTTGGCGAGATCGGGAGAATCGGTGGCATTGGTGATTCCTAGGGGAATAAATCTTTGGGCACCTAGTGGTCATG

2 CATGACCACTAGGAGCATCTTTGGCGACTGTACTACTCGGGCTCGGTCGAGGCATCTCTAGGGGAATAAATCTTTGGGCACCTAGTGGTCATG

2 CATGACCACTAGGAGCATCTTTGGCGAGCCTCGCTTGGGAGGCTGCTCCACCAGTTCCTAGGGGAATAAATCTTTGGGCACCTAGTGGTCATG

2 CATGACCACTAGGAGCATCTTTGGCGATCGCACAGCGGAAATGGTATCATTCGTGGACTAGGGGAATAAATCTTTGGGCACCTAGTGGTCATG

2 CATGACCACTAGGAGCATCTTTGGCGAGATCGGGAGAATCGGCGGCACTGGTGTCTCCTAGGGGAATAAATCTTTGGGCACCTAGTGGTCATG

2 CATGACCACTAGGAGCATCTTTGGCGAGATCGGGAGAATCGGTGGCATAGGCGTCTCCTAGGGGAATAAATCTTTGGGCACCTAGTGGTCATG

2 CATGACCACTAGGAGCATCTTTGGCGAGATCGGGAGAAACGGTGGCATCGGTGTCTCCTAGGGGAATAAATCTTTGGGCACCTAGTGGTCATG

2 CATGACCACTAGGAGCATCTTTGGCGATGGGGCCGTGCTATCAGCACACTCCGCGGGCTAGGGGAATAAATCTTTGGGCACCTAGTGGTCATG

2 CATGACCACTAGGAGCATCTTTGGCGAATGGCCGGCACGGTCTTCTAGTCCTCGGTGCTAGGGGAATAAATCTTTGGGCACCTAGTGGTCATG

2 CATGACCACTAGGAGCATCTTTGGCGATCCGGGGGCCCTCGATGGCGACGTTACAGGCTAGGGGAATAAATCTTTGGGCACCTAGTGGTCATG

2 CATGACCACTAGGAGCATCTTTGGCGACGAGCAACATCAAGGTGCGGGCGGGATGTCCTAGGGGAATAAATCTTTGGGCACCTAGTGGTCATG

2 CATGACCACTAGGAGCATCTTTGGCGAACGAAAGTGAGGCATGGCGGGTGGTGCGACCTAGGGGAATAAATCTTTGGGCACCTAGTGGTCATG

2 CATGACCACTAGGAGCATCTTTGGCGAAGCGTGTTGCAAGGTTTGCGAGTGTGGACCCTAGGGGAATAAATCTTTGGGCACCTAGTGGTCATG

2 CATGACCACTAGGAGCATCTTTGGCGAATGGCCGGCACGGCCTTCTAGCCCTCGGTACTAGGGGAATAAATCTTTGGGCACCTAGTGGTCATG

2 CATGACCACTAGGAGCATCTTTGGCGAGATTGGGAGAATCGGTGGCATTGGTGACTCCTAGGGGAATAAATCTTTGGGCACCTAGTGGTCATG

2 CATGACCACTAGGAGCATCTTTGGCGATTTGGGAGTCGCGTTATGCAGGCAGTGAGACTAGGGGAATAAATCTTTGGGCACCTAGTGGTCATG

2 CATGACCACTAGGAGCATCTTTGGCGAAAGTCGGTAGAACACGGTGGGGTGCTGTCCCTAGGGGAATAAATCTTTGGGCACCTAGTGGTCATG

2 CATGACCACTAGGAGCATCTTTGGCGAGATCGGGATAATCGGTGGCATTGTTGTCTCCTAGGGGAATAAATCTTTGGGCACCTAGTGGTCATG

2 CATGACCACTAGGAGCATCTTTGGCGAGGACGCTCGAGAGGAGGTTGTTAGTCTAGACTAGGGGAATAAATCTTTGGGCACCTAGTGGTCATG

2 CATGACCACTAGGAGCATCTTTGGCGACCGGCAGGTGTTGCGGTGGTCTATGAATCCCTAGGGGAATAAATCTTTGGGCACCTAGTGGTCATG

2 CATGACCACTAGGAGCATCTTTGGCGAGATCGGGAGAATCGGTGGCATTGATGTTTCCTAGGGGAATAAATCTTTGGGCACCTAGTGGTCATG

2 CATGACCACTAGGAGCATCTTTGGCGACGTTTGTCCTTGGTAGTTACGGCCAATCTCCTAGGGGAATAAATCTTTGGGCACCTAGTGGTCATG

2 CATGACCACTAGGAGCATCTTTGGCGAGATCGTGAGAATCGCTGGCATTGGTGTCTCCTAGGGGAATAAATCTTTGGGCACCTAGTGGTCATG

2 CATGACCACTAGGAGCATCTTTGGCGAACGGCAGGTGTTGCGGTGGTATGAGAATCCCTAGGGGAATAAATCTTTGGGCACCTAGTGGTCATG

2 CATGACCACTAGGAGCATCTTTGGCGAGATCGGGAGACTAGGTGGCATTGGTGTCTCCTAGGGGAATAAATCTTTGGGCACCTAGTGGTCATG

2 CATGACCACTAGGAGCATCTTTGGCGAGATCGGGAGAATCGGTGGCATTGGTGTATACTAGGGGAATAAATCTTTGGGCACCTAGTGGTCATG

2 CATGACCACTAGGAGCATCTTTGGCGAGATCTGGAGAATCGGTGGCATTGGTGCCTCCTAGGGGAATAAATCTTTGGGCACCTAGTGGTCATG

2 CATGACCACTAGGAGCATCTTTGGCGAGATCGGGAGAATCGGTAGCATCGGTGTCTCCTAGGGGAATAAATCTTTGGGCACCTAGTGGTCATG

2 CATGACCACTAGGAGCATCTTTGGCGACCTCTGCATGGTACATGTAAAGAGTGTGGACTAGGGGAATAAATCTTTGGGCACCTAGTGGTCATG

2 CATGACCACTAGGAGCATCTTTGGCGAGATCGGGAGGATCGGTGGCATAGGTGTCTCCTAGGGGAATAAATCTTTGGGCACCTAGTGGTCATG

2 CATGACCACTAGGAGCATCTTTGGCGAGTAGTGGTTACGGGGGGTTCACTGCAGAGACTAGGGGAATAAATCTTTGGGCACCTAGTGGTCATG

2 CATGACCACTAGGAGCATCTTTGGCGAACGGCCGGCACGGCCTTCTAGTCCTTGGTACTAGGGGAATAAATCTTTGGGCACCTAGTGGTCATG

2 CATGACCACTAGGAGCATCTTTGGCGAGATCTGTAGAATCGGTGGCATTGGTGTCTCCTAGGGGAATAAATCTTTGGGCACCTAGTGGTCATG

2 CATGACCACTAGGAGCATCTTTGGCGAAGGGTGTAGGACTTCAAGTGGGTCTCATAGCTAGGGGAATAAATCTTTGGGCACCTAGTGGTCATG

2 CATGACCACTAGGAGCATCTTTGGCGAGATCGGTAGAATAGGTGGCATTGGTGTCTCCTAGGGGAATAAATCTTTGGGCACCTAGTGGTCATG

2 CATGACCACTAGGAGCATCTTTGGCGAGAGCGGGAGAATCGGTGGCAGTGGTGTCTCCTAGGGGAATAAATCTTTGGGCACCTAGTGGTCATG

2 CATGACCACTAGGAGCATCTTTGGCGAGGACCGGTGTGTCATTCCAAGATGCCGAACCTAGGGGAATAAATCTTTGGGCACCTAGTGGTCATG

2 CATGACCACTAGGAGCATCTTTGGCGAGGAGTGGCTGAGGGCGGTGGGTCGGTCGCGCTAGGGGAATAAATCTTTGGGCACCTAGTGGTCATG

2 CATGACCACTAGGAGCATCTTTGGCGAACGGCGGGTGTTGCGGTGGTCTGTGAATTCCTAGGGGAATAAATCTTTGGGCACCTAGTGGTCATG

2 CATGACCACTAGGAGCATCTTTGGCGAGATCGCGAGAAACGGTGGCATTGGTGTCTCCTAGGGGAATAAATCTTTGGGCACCTAGTGGTCATG

2 CATGACCACTAGGAGCATCTTTGGCGAGCCTCGCTTGGGAGGTTGCTCCACAAGTTCCTAGGGGAATAAATCTTTGGGCACCTAGTGGTCATG

2 CATGACCACTAGGAGCATCTTTGGCGACAATGGAGCGACGGTGCGGTCTGAGTCCTACTAGGGGAATAAATCTTTGGGCACCTAGTGGTCATG

2 CATGACCACTAGGAGCATCTTTGGCGACGAAAGTGGCCATCTCAAGCGCGTGTGATCCTAGGGGAATAAATCTTTGGGCACCTAGTGGTCATG

2 CATGACCACTAGGAGCATCTTTGGCGAGATCGTGGGAATCGGTGGCATTGGTGTCTCCTAGGGGAATAAATCTTTGGGCACCTAGTGGTCATG

2 CATGACCACTAGGAGCATCTTTGGCGACGTGGGCACAAGGAACAGAGAACACAGAGACTAGGGGAATAAATCTTTGGGCACCTAGTGGTCATG

2 CATGACCACTAGGAGCATCTTTGGCGAGATCGGGAGAATCGGTGGAATTGGTGTCTTCTAGGGGAATAAATCTTTGGGCACCTAGTGGTCATG

2 CATGACCACTAGGAGCATCTTTGGCGAGCCTTGCTTGGGAGGTTGCTCCTCCAGTTCCTAGGGGAATAAATCTTTGGGCACCTAGTGGTCATG

2 CATGACCACTAGGAGCATCTTTGGCGAGATCTGGAGAATCGGTGGCATAGGTGTCTCCTAGGGGAATAAATCTTTGGGCACCTAGTGGTCATG

2 CATGACCACTAGGAGCATCTTTGGCGATGGGTGCTGACGGCCGCAGCTGCGGCTACACTAGGGGAATAAATCTTTGGGCACCTAGTGGTCATG

2 CATGACCACTAGGAGCATCTTTGGCGAGAACGGGAGGATCGGTGGCATTGGTGTCTCCTAGGGGAATAAATCTTTGGGCACCTAGTGGTCATG

2 CATGACCACTAGGAGCATCTTTGGCGAACGGCAGGTGCTGCGGTGGTCTGTGAGTCCCTAGGGGAATAAATCTTTGGGCACCTAGTGGTCATG

2 CATGACCACTAGGAGCATCTTTGGCGAGGTCGCGAGAATCGGTGGCATTGGTGTCTCCTAGGGGAATAAATCTTTGGGCACCTAGTGGTCATG

2 CATGACCACTAGGAGCATCTTTGGCGACCCCCTCCGTTGCGGGTTTGACGAGGTTTGCTAGGGGAATAAATCTTTGGGCACCTAGTGGTCATG

2 CATGACCACTAGGAGCATCTTTGGCGAACGGCAGGACTTGCGGTGGTCTGTGAATCCCTAGGGGAATAAATCTTTGGGCACCTAGTGGTCATG

2 CATGACCACTAGGAGCATCTTTGGCGATCCGGAATAGTGTTGGTTTTAACACGTGAGCTAGGGGAATAAATCTTTGGGCACCTAGTGGTCATG

2 CATGACCACTAGGAGCATCTTTGGCGAGGTGTCATAGAGCGATCAGGGGGCGAGACACTAGGGGAATAAATCTTTGGGCACCTAGTGGTCATG

2 CATGACCACTAGGAGCATCTTTGGCGAGATCGGGTGAGTCGGTGGCATTGGTGTCTCCTAGGGGAATAAATCTTTGGGCACCTAGTGGTCATG

2 CATGACCACTAGGAGCATCTTTGGCGACGAGTCAGGGCATGCACTGCGGTAGTGGAGCTAGGGGAATAAATCTTTGGGCACCTAGTGGTCATG

2 CATGACCACTAGGAGCATCTTTGGCGACACAGATGTTGATAGATGTGACAAGTGGAGCTAGGGGAATAAATCTTTGGGCACCTAGTGGTCATG

2 CATGACCACTAGGAGCATCTTTGGCGAGATCGGGGGAATCGGTGGCATTGGCGTCTCCTAGGGGAATAAATCTTTGGGCACCTAGTGGTCATG

2 CATGACCACTAGGAGCATCTTTGGCGAGATCAGGAGAATCGGTTGCATTGGTGTCTCCTAGGGGAATAAATCTTTGGGCACCTAGTGGTCATG

2 CATGACCACTAGGAGCATCTTTGGCGAGATCGGGAGAATCAGTGGCATTGGTGTTTCCTAGGGGAATAAATCTTTGGGCACCTAGTGGTCATG

2 CATGACCACTAGGAGCATCTTTGGCGATCCGGGGGCATGCTATGCGACGATTATGGGCTAGGGGAATAAATCTTTGGGCACCTAGTGGTCATG

2 CATGACCACTAGGAGCATCTTTGGCGATGGGTGCTGACTGCCGCCGCTGCGGCTACACTAGGGGAATAAATCTTTGGGCACCTAGTGGTCATG

2 CATGACCACTAGGAGCATCTTTGGCGACAAGTGGGGGTATGAAAGTTTTCGTTGAGACTAGGGGAATAAATCTTTGGGCACCTAGTGGTCATG

2 CATGACCACTAGGAGCATCTTTGGCGAACGGCAGGTGTTGCGTTGGTCTGTGAATTCCTAGGGGAATAAATCTTTGGGCACCTAGTGGTCATG

2 CATGACCACTAGGAGCATCTTTGGCGACAATGGAGCAACGGTGTGGTCTGAGTCCTACTAGGGGAATAAATCTTTGGGCACCTAGTGGTCATG

2 CATGACCACTAGGAGCATCTTTGGCGAGGGTCCCTAGGGCCCTATAGAGTTTGTGAGCTAGGGGAATAAATCTTTGGGCACCTAGTGGTCATG

2 CATGACCACTAGGAGCATCTTTGGCGACGTCTAATGGATGTTCGTGATCGCTGAGCTCTAGGGGAATAAATCTTTGGGCACCTAGTGGTCATG

2 CATGACCACTAGGAGCATCTTTGGCGAGATCGGGAGAAACGTTGGCATTGGTGTCTCCTAGGGGAATAAATCTTTGGGCACCTAGTGGTCATG

2 CATGACCACTAGGAGCATCTTTGGCGATCCGGGGGCATTCTATGCGACGCTTATGGGCTAGGGGAATAAATCTTTGGGCACCTAGTGGTCATG

2 CATGACCACTAGGAGCATCTTTGGCGAGATCGGGAGAATTGGTGGCATTGGTGTCTACTAGGGGAATAAATCTTTGGGCACCTAGTGGTCATG

2 CATGACCACTAGGAGCATCTTTGGCGAACGGCAGGTGCTGCTGTGGTCTGTGAATCCCTAGGGGAATAAATCTTTGGGCACCTAGTGGTCATG

2 CATGACCACTAGGAGCATCTTTGGCGAGGTCTACGACGAGCGAAGGTGTGCGACCTACTAGGGGAATAAATCTTTGGGCACCTAGTGGTCATG

2 CATGACCACTAGGAGCATCTTTGGCGAGGTCGGGAGAATCGGTGGCATTGATGTCTCCTAGGGGAATAAATCTTTGGGCACCTAGTGGTCATG

2 CATGACCACTAGGAGCATCTTTGGCGATCCGGGGGGCCATCTAGGACGACGCTAGGGCTAGGGGAATAAATCTTTGGGCACCTAGTGGTCATG

2 CATGACCACTAGGAGCATCTTTGGCGAGTCGGGGTCGTGAAGTGGGTCGTTGACTGGCTAGGGGAATAAATCTTTGGGCACCTAGTGGTCATG

2 CATGACCACTAGGAGCATCTTTGGCGAAGCGCAGGGTGTGAGAGGGGCGCGTCCATGCTAGGGGAATAAATCTTTGGGCACCTAGTGGTCATG

2 CATGACCACTAGGAGCATCTTTGGCGAAAGTCGGTAGAACAGGGTAGGGTGCTGTCCCTAGGGGAATAAATCTTTGGGCACCTAGTGGTCATG

2 CATGACCACTAGGAGCATCTTTGGCGAGATCGCTAGAATCGGTGGCATTGGTGTCTCCTAGGGGAATAAATCTTTGGGCACCTAGTGGTCATG

2 CATGACCACTAGGAGCATCTTTGGCGAGTCGGGGATTTCCGGAGTCGTTGACTGGTCCTAGGGGAATAAATCTTTGGGCACCTAGTGGTCATG

2 CATGACCACTAGGAGCATCTTTGGCGAGATCGGTAGAATCGGAGGCATTGGTGTCTCCTAGGGGAATAAATCTTTGGGCACCTAGTGGTCATG

2 CATGACCACTAGGAGCATCTTTGGCGACTAAGTGGCCGACGCGTTAAATGGGGTAGACTAGGGGAATAAATCTTTGGGCACCTAGTGGTCATG

2 CATGACCACTAGGAGCATCTTTGGCGAAGCGTGTTGCAAGGTCCGCGACTGTGGACCCTAGGGGAATAAATCTTTGGGCACCTAGTGGTCATG

2 CATGACCACTAGGAGCATCTTTGGCGAAATCGGGAGAATTGGTGGCATTGGTGTCTCCTAGGGGAATAAATCTTTGGGCACCTAGTGGTCATG

2 CATGACCACTAGGAGCATCTTTGGCGAGATCGGGAGAATCGGTGGAATTGGTTTCTCCTAGGGGAATAAATCTTTGGGCACCTAGTGGTCATG

2 CATGACCACTAGGAGCATCTTTGGCGAATGGCCGGCACAGCCTTCTAGTCCTCGGTACTAGGGGAATAAATCTTTGGGCACCTAGTGGTCATG

2 CATGACCACTAGGAGCATCTTTGGCGAACGAAGTGGTCGGGAGGCGTCGGCGGAGTGCTAGGGGAATAAATCTTTGGGCACCTAGTGGTCATG

2 CATGACCACTAGGAGCATCTTTGGCGAGCCTTGCTTGGGAGGTTGCTCCACAAGTTCCTAGGGGAATAAATCTTTGGGCACCTAGTGGTCATG

2 CATGACCACTAGGAGCATCTTTGGCGAGATCAGTAGAATCGGTGGCATTGGTGTCTCCTAGGGGAATAAATCTTTGGGCACCTAGTGGTCATG

2 CATGACCACTAGGAGCATCTTTGGCGACGAAGGGCACGGGTGGTCATTGCGAGAAGACTAGGGGAATAAATCTTTGGGCACCTAGTGGTCATG

2 CATGACCACTAGGAGCATCTTTGGCGATCCGGGGGCTGATGTGCAGCGACGCCTGGGCTAGGGGAATAAATCTTTGGGCACCTAGTGGTCATG

2 CATGACCACTAGGAGCATCTTTGGCGATATGCATAGCTGGATCCGTTGTGGGCAGGCCTAGGGGAATAAATCTTTGGGCACCTAGTGGTCATG

2 CATGACCACTAGGAGCATCTTTGGCGACAGGGGATGCCGATGCGCTGGACGACTGAGCTAGGGGAATAAATCTTTGGGCACCTAGTGGTCATG

2 CATGACCACTAGGAGCATCTTTGGCGAACGGCTGGCACGGCCTTCTAGTCCTCGGTACTAGGGGAATAAATCTTTGGGCACCTAGTGGTCATG

2 CATGACCACTAGGAGCATCTTTGGCGACCTGGACGTGGGCGGTCAGACGGAGGGCGACTAGGGGAATAAATCTTTGGGCACCTAGTGGTCATG

2 CATGACCACTAGGAGCATCTTTGGCGAGATCGGGAGAATCGGTGGCATAGGTCTCTCCTAGGGGAATAAATCTTTGGGCACCTAGTGGTCATG

2 CATGACCACTAGGAGCATCTTTGGCGATCGCACAGCGGACGATATATAGAGCGGGGCCTAGGGGAATAAATCTTTGGGCACCTAGTGGTCATG

2 CATGACCACTAGGAGCATCTTTGGCGACAATGGAGCGACGGTGTGGTCTGAGTCCCACTAGGGGAATAAATCTTTGGGCACCTAGTGGTCATG

2 CATGACCACTAGGAGCATCTTTGGCGATGGGTGCTGACGACCGTTCATACGGTTACTCTAGGGGAATAAATCTTTGGGCACCTAGTGGTCATG

2 CATGACCACTAGGAGCATCTTTGGCGAGGAGCCTAAGTGCGTATCTGTGGTCGAGCTCTAGGGGAATAAATCTTTGGGCACCTAGTGGTCATG

2 CATGACCACTAGGAGCATCTTTGGCGAGATCGGGAGAATCGGTTGCATTGGTGTCTTCTAGGGGAATAAATCTTTGGGCACCTAGTGGTCATG

2 CATGACCACTAGGAGCATCTTTGGCGAGATCGGGAGACTCGGTGGCAGTGGTGTCTCCTAGGGGAATAAATCTTTGGGCACCTAGTGGTCATG

2 CATGACCACTAGGAGCATCTTTGGCGACGGTGCGGGGCCTAAGTGCGGCGGCTGATTCTAGGGGAATAAATCTTTGGGCACCTAGTGGTCATG

2 CATGACCACTAGGAGCATCTTTGGCGAGGTTGTAGCTCGCGTGTAGGGTCGGCAGTGCTAGGGGAATAAATCTTTGGGCACCTAGTGGTCATG

2 CATGACCACTAGGAGCATCTTTGGCGAACGTGCAAGATATGCCGCTTGGAGCGTAGGCTAGGGGAATAAATCTTTGGGCACCTAGTGGTCATG

2 CATGACCACTAGGAGCATCTTTGGCGACGTTAGTGGGCACTGTGAAGAAAACGGAGACTAGGGGAATAAATCTTTGGGCACCTAGTGGTCATG

2 CATGACCACTAGGAGCATCTTTGGCGAGCACGAGAGTCAAGTGCGTATAGCGTGGAGCTAGGGGAATAAATCTTTGGGCACCTAGTGGTCATG

2 CATGACCACTAGGAGCATCTTTGGCGATAGGCGTGTACGGTCGAGCGAAAGCGGTAGCTAGGGGAATAAATCTTTGGGCACCTAGTGGTCATG

2 CATGACCACTAGGAGCATCTTTGGCGAGAAGACCCCGGATTCGGGGACCAGTTGCTGCTAGGGGAATAAATCTTTGGGCACCTAGTGGTCATG

2 CATGACCACTAGGAGCATCTTTGGCGATGGGTGCTGACGGCCGCCTCTGCGGCTACACTAGGGGAATAAATCTTTGGGCACCTAGTGGTCATG

2 CATGACCACTAGGAGCATCTTTGGCGAAGGCGAAGCACGCTGGTTGTGGTGGCGTCCCTAGGGGAATAAATCTTTGGGCACCTAGTGGTCATG

2 CATGACCACTAGGAGCATCTTTGGCGAGATCGGCAGAATCGGTGGCAATGGTGTCTCCTAGGGGAATAAATCTTTGGGCACCTAGTGGTCATG

2 CATGACCACTAGGAGCATCTTTGGCGAGATCGCGGGAATCGGTGGCATTGGTGTCTCCTAGGGGAATAAATCTTTGGGCACCTAGTGGTCATG

2 CATGACCACTAGGAGCATCTTTGGCGAACGGCAGGTGTTGCGGTGGTCTCTTAATCCCTAGGGGAATAAATCTTTGGGCACCTAGTGGTCATG

2 CATGACCACTAGGAGCATCTTTGGCGAGAAGACTCTGGATCCGGGGACCAGTTGCTCCTAGGGGAATAAATCTTTGGGCACCTAGTGGTCATG

2 CATGACCACTAGGAGCATCTTTGGCGAGATCGGGAGAATCGGTGTCATTGGTGACTCCTAGGGGAATAAATCTTTGGGCACCTAGTGGTCATG

2 CATGACCACTAGGAGCATCTTTGGCGAACGGCAGGTGTTGCGGTGGTCTGTGCATCTCTAGGGGAATAAATCTTTGGGCACCTAGTGGTCATG

2 CATGACCACTAGGAGCATCTTTGGCGACCGGCATGTGTTGCGGTGGTCTGTGAATCCCTAGGGGAATAAATCTTTGGGCACCTAGTGGTCATG

2 CATGACCACTAGGAGCATCTTTGGCGAACGGCAGGTTTTGCGGTGGTCTGTGGATCCCTAGGGGAATAAATCTTTGGGCACCTAGTGGTCATG

2 CATGACCACTAGGAGCATCTTTGGCGATCGCACAGCGAAGGGGCACTGCAACCGGGACTAGGGGAATAAATCTTTGGGCACCTAGTGGTCATG

2 CATGACCACTAGGAGCATCTTTGGCGAGGAGTGGCTGAGGGCGGTGGGTAGGTTGCGCTAGGGGAATAAATCTTTGGGCACCTAGTGGTCATG

2 CATGACCACTAGGAGCATCTTTGGCGACACATTGACGTCAACCAATGCGAAGTGGAGCTAGGGGAATAAATCTTTGGGCACCTAGTGGTCATG

2 CATGACCACTAGGAGCATCTTTGGCGAGGGAGGGTGCCGGCAGCGGTGTGAATGCGACTAGGGGAATAAATCTTTGGGCACCTAGTGGTCATG

2 CATGACCACTAGGAGCATCTTTGGCGACCCAGCTTAGGATCATGCTGGAGTGTTGCACTAGGGGAATAAATCTTTGGGCACCTAGTGGTCATG

2 CATGACCACTAGGAGCATCTTTGGCGACCCTCAATCTCCGAGAGGAATAGGGGTGGACTAGGGGAATAAATCTTTGGGCACCTAGTGGTCATG

2 CATGACCACTAGGAGCATCTTTGGCGACGGCGTGGGGCCATCTGTGCGGCGGATTCCCTAGGGGAATAAATCTTTGGGCACCTAGTGGTCATG

2 CATGACCACTAGGAGCATCTTTGGCGATCCGGGGGTCACATCGGGACGACGCTAGGGCTAGGGGAATAAATCTTTGGGCACCTAGTGGTCATG

2 CATGACCACTAGGAGCATCTTTGGCGAGATCGGGAGAATCAGTGGCCTTGGTGTCTCCTAGGGGAATAAATCTTTGGGCACCTAGTGGTCATG

2 CATGACCACTAGGAGCATCTTTGGCGAGATCGGTAGAATCGGTGGCGTTGGTGTCTCCTAGGGGAATAAATCTTTGGGCACCTAGTGGTCATG

2 CATGACCACTAGGAGCATCTTTGGCGAGATAGGGAGAATAGGTGGCATTGGTGTCTCCTAGGGGAATAAATCTTTGGGCACCTAGTGGTCATG

2 CATGACCACTAGGAGCATCTTTGGCGAAGTATCGACTAGATTCGAGCAGACGTTTGGCTAGGGGAATAAATCTTTGGGCACCTAGTGGTCATG

2 CATGACCACTAGGAGCATCTTTGGCGAGATCGGAAGAATCGGTGGCATTGGTGTTTCCTAGGGGAATAAATCTTTGGGCACCTAGTGGTCATG

2 CATGACCACTAGGAGCATCTTTGGCGAGATCGGGAGAATCGGCGGCATTGGTGTATCCTAGGGGAATAAATCTTTGGGCACCTAGTGGTCATG

2 CATGACCACTAGGAGCATCTTTGGCGATCCGGGGGACTTGTTAGTCGGCGCTTTGGGCTAGGGGAATAAATCTTTGGGCACCTAGTGGTCATG

2 CATGACCACTAGGAGCATCTTTGGCGACAGGCGTGGTCAGGGCACTAGCGACGGAGACTAGGGGAATAAATCTTTGGGCACCTAGTGGTCATG

2 CATGACCACTAGGAGCATCTTTGGCGAACGGGAGCAAGGCGGGTCAAATAATGTCGACTAGGGGAATAAATCTTTGGGCACCTAGTGGTCATG

2 CATGACCACTAGGAGCATCTTTGGCGAGGCAAGGCGTGCGGCGGGCGGGTACCACGGCTAGGGGAATAAATCTTTGGGCACCTAGTGGTCATG

2 CATGACCACTAGGAGCATCTTTGGCGAGATCGGGAGAACCTGTGGCATTGGTGTCTCCTAGGGGAATAAATCTTTGGGCACCTAGTGGTCATG

2 CATGACCACTAGGAGCATCTTTGGCGAGTGGGGGGCGACGGCCGGTAGTGTGTGAGACTAGGGGAATAAATCTTTGGGCACCTAGTGGTCATG

2 CATGACCACTAGGAGCATCTTTGGCGAGATCGGGAGAGTCGGTGGCCTTGGTGTCTCCTAGGGGAATAAATCTTTGGGCACCTAGTGGTCATG

2 CATGACCACTAGGAGCATCTTTGGCGAGATCGAGAGAATCGGTGGCATTGGTGTTTCCTAGGGGAATAAATCTTTGGGCACCTAGTGGTCATG

2 CATGACCACTAGGAGCATCTTTGGCGAGTCGCCAGGCTCCGAGAGACATCAGAATCCCTAGGGGAATAAATCTTTGGGCACCTAGTGGTCATG

2 CATGACCACTAGGAGCATCTTTGGCGATCGCAGAGCGGGGAATGTATGTGAACGGGCCTAGGGGAATAAATCTTTGGGCACCTAGTGGTCATG

2 CATGACCACTAGGAGCATCTTTGGCGAGATCGGGAGGATCGGTGGCATTGGCGTCTCCTAGGGGAATAAATCTTTGGGCACCTAGTGGTCATG

2 CATGACCACTAGGAGCATCTTTGGCGAGACTTGCTTGGGAGGTTGCTCCACCAGTTCCTAGGGGAATAAATCTTTGGGCACCTAGTGGTCATG

2 CATGACCACTAGGAGCATCTTTGGCGAGATAGGAAGAATCGGTGGCATTGGTGTCTCCTAGGGGAATAAATCTTTGGGCACCTAGTGGTCATG

2 CATGACCACTAGGAGCATCTTTGGCGAGCCTCGCTTGGGAGGTTGCTCCACCAGTCCCTAGGGGAATAAATCTTTGGGCACCTAGTGGTCATG

2 CATGACCACTAGGAGCATCTTTGGCGATATTGAATAGACCCAGATCACAATCGTAGACTAGGGGAATAAATCTTTGGGCACCTAGTGGTCATG

2 CATGACCACTAGGAGCATCTTTGGCGAGAAGACTCCGGATTCGGGGATCAGTTGCTGCTAGGGGAATAAATCTTTGGGCACCTAGTGGTCATG

2 CATGACCACTAGGAGCATCTTTGGCGACGGTGTGGGGTCTACCAGCACGGCGGTGATCTAGGGGAATAAATCTTTGGGCACCTAGTGGTCATG

2 CATGACCACTAGGAGCATCTTTGGCGAGATCGGGGGAATCGGTGGCATTGATGTCTCCTAGGGGAATAAATCTTTGGGCACCTAGTGGTCATG

2 CATGACCACTAGGAGCATCTTTGGCGAACCCGCGCGCGCTATAGGTTGCTCGAGCTCCTAGGGGAATAAATCTTTGGGCACCTAGTGGTCATG

2 CATGACCACTAGGAGCATCTTTGGCGAACGGCAAGTGTTGCGGTGGTCTGTGTATCCCTAGGGGAATAAATCTTTGGGCACCTAGTGGTCATG

2 CATGACCACTAGGAGCATCTTTGGCGAGATGGGGACAAAGTGCGGGTGGTCGAGCCTCTAGGGGAATAAATCTTTGGGCACCTAGTGGTCATG

2 CATGACCACTAGGAGCATCTTTGGCGAGATCGGGAGAATCGGTGGCAGTGGCGTCTCCTAGGGGAATAAATCTTTGGGCACCTAGTGGTCATG

2 CATGACCACTAGGAGCATCTTTGGCGACCGTAGCCTCCGGGTCCCGGTTGAAGACCACTAGGGGAATAAATCTTTGGGCACCTAGTGGTCATG

2 CATGACCACTAGGAGCATCTTTGGCGAGTGGGGGGTGACGGCCGGTTGTGGGTGAGACTAGGGGAATAAATCTTTGGGCACCTAGTGGTCATG

2 CATGACCACTAGGAGCATCTTTGGCGACCCGGCAGGGTGGAAACGACGGAGTGGCGACTAGGGGAATAAATCTTTGGGCACCTAGTGGTCATG

2 CATGACCACTAGGAGCATCTTTGGCGACATAGTGGACAACGAATACGTAGATAGTCGCTAGGGGAATAAATCTTTGGGCACCTAGTGGTCATG

2 CATGACCACTAGGAGCATCTTTGGCGAGATCGGGAGAATCGTTGGCATTGGTGTCTACTAGGGGAATAAATCTTTGGGCACCTAGTGGTCATG

2 CATGACCACTAGGAGCATCTTTGGCGACCCTGCCTTGAGTCTTAGACCTGCGCGATGCTAGGGGAATAAATCTTTGGGCACCTAGTGGTCATG

2 CATGACCACTAGGAGCATCTTTGGCGAGGTGTTTCAGTTCGACATAAGGGGCGAGCACTAGGGGAATAAATCTTTGGGCACCTAGTGGTCATG

2 CATGACCACTAGGAGCATCTTTGGCGACCCGCTGTTATGCAGGGCCGATCGTTTCGTCTAGGGGAATAAATCTTTGGGCACCTAGTGGTCATG

2 CATGACCACTAGGAGCATCTTTGGCGAGATCGGTAGAATTGGTGGCATTGGTGTCTCCTAGGGGAATAAATCTTTGGGCACCTAGTGGTCATG

2 CATGACCACTAGGAGCATCTTTGGCGAAGGGTGTAGGATTTCAAGTGGATCTCATAGCTAGGGGAATAAATCTTTGGGCACCTAGTGGTCATG

2 CATGACCACTAGGAGCATCTTTGGCGAGATCGGGAGACTCGTTGGCATTGGTGTCTCCTAGGGGAATAAATCTTTGGGCACCTAGTGGTCATG

2 CATGACCACTAGGAGCATCTTTGGCGAATGACCGAAGACATGTGGCAGAGAGGTCGCCTAGGGGAATAAATCTTTGGGCACCTAGTGGTCATG

2 CATGACCACTAGGAGCATCTTTGGCGAGAAGTGTTGACTCGGTCGAGATAAGTAACACTAGGGGAATAAATCTTTGGGCACCTAGTGGTCATG

2 CATGACCACTAGGAGCATCTTTGGCGATCATCGTCAGATGGTGAAGCAGACGTTTGGCTAGGGGAATAAATCTTTGGGCACCTAGTGGTCATG

2 CATGACCACTAGGAGCATCTTTGGCGACAGCCGTAGCCGTGAGGTTGGTATAAGGTCCTAGGGGAATAAATCTTTGGGCACCTAGTGGTCATG

2 CATGACCACTAGGAGCATCTTTGGCGAGATCGGGGGAATCGGAGGCATTGGTGTCTCCTAGGGGAATAAATCTTTGGGCACCTAGTGGTCATG

2 CATGACCACTAGGAGCATCTTTGGCGACGGCGTGGGGCTATTGATGCGGCGGCATTCCTAGGGGAATAAATCTTTGGGCACCTAGTGGTCATG

2 CATGACCACTAGGAGCATCTTTGGCGAACTGCAGGTGTTGCGGTGGTCTGTGAATCTCTAGGGGAATAAATCTTTGGGCACCTAGTGGTCATG

2 CATGACCACTAGGAGCATCTTTGGCGAGATCGGGAGAATCGGCGGCATTGGAGTCTCCTAGGGGAATAAATCTTTGGGCACCTAGTGGTCATG

2 CATGACCACTAGGAGCATCTTTGGCGAGAGGGGGGCGACGGCCGGTAGTGGGTGAGACTAGGGGAATAAATCTTTGGGCACCTAGTGGTCATG

2 CATGACCACTAGGAGCATCTTTGGCGAGATCGGAAGAATCGGTGGCATTGTTGTCTCCTAGGGGAATAAATCTTTGGGCACCTAGTGGTCATG

2 CATGACCACTAGGAGCATCTTTGGCGAGATCGGGAGGATCGGTGGCACTGGTGTCTCCTAGGGGAATAAATCTTTGGGCACCTAGTGGTCATG

2 CATGACCACTAGGAGCATCTTTGGCGACCCGCGTAGACAATGTAGAGTCGACCAGTGCTAGGGGAATAAATCTTTGGGCACCTAGTGGTCATG

2 CATGACCACTAGGAGCATCTTTGGCGAGATCGGGAGAATCGGTGGCGTTGGCGTCTCCTAGGGGAATAAATCTTTGGGCACCTAGTGGTCATG

2 CATGACCACTAGGAGCATCTTTGGCGAAGGGTGTAGGACTTCAAGTGGATCTAATAGCTAGGGGAATAAATCTTTGGGCACCTAGTGGTCATG

2 CATGACCACTAGGAGCATCTTTGGCGAGATCGAGAGAATCGGTGGCAGTGGTGTCTCCTAGGGGAATAAATCTTTGGGCACCTAGTGGTCATG

2 CATGACCACTAGGAGCATCTTTGGCGAGATCGGAAGGATCGGTGGCATTGGTGTCTCCTAGGGGAATAAATCTTTGGGCACCTAGTGGTCATG

2 CATGACCACTAGGAGCATCTTTGGCGAGGGATGGGAGCCAGTGGGGTTCGGGGTCTGCTAGGGGAATAAATCTTTGGGCACCTAGTGGTCATG

2 CATGACCACTAGGAGCATCTTTGGCGACGGCGTGGGGCATCTTTCGCGGCGGTTATTCTAGGGGAATAAATCTTTGGGCACCTAGTGGTCATG

2 CATGACCACTAGGAGCATCTTTGGCGAGGGAGGGCGCCGGCAGCGGTGTGAATGTGCCTAGGGGAATAAATCTTTGGGCACCTAGTGGTCATG

2 CATGACCACTAGGAGCATCTTTGGCGACTGAGTGGGCAACGCGTTAAATGGGGTAGACTAGGGGAATAAATCTTTGGGCACCTAGTGGTCATG

2 CATGACCACTAGGAGCATCTTTGGCGAGGGAGGGCGCCGGCTGCGGTGTGAATGCGCCTAGGGGAATAAATCTTTGGGCACCTAGTGGTCATG

2 CATGACCACTAGGAGCATCTTTGGCGATCAGGGGGCCGCACAGGGGCGACTTCAGGGCTAGGGGAATAAATCTTTGGGCACCTAGTGGTCATG

2 CATGACCACTAGGAGCATCTTTGGCGACTAAGTGGGCCACGCGTTAAATGGGGTAGACTAGGGGAATAAATCTTTGGGCACCTAGTGGTCATG

2 CATGACCACTAGGAGCATCTTTGGCGAGATCTGGATAATCGGTGGCATTGGTGTCTCCTAGGGGAATAAATCTTTGGGCACCTAGTGGTCATG

2 CATGACCACTAGGAGCATCTTTGGCGAACGGCAGGTGTTGCGGTGGACTGTGGATCCCTAGGGGAATAAATCTTTGGGCACCTAGTGGTCATG

2 CATGACCACTAGGAGCATCTTTGGCGAGTGGTGGTTGCCTGGAACCGTAGGCCTAGACTAGGGGAATAAATCTTTGGGCACCTAGTGGTCATG

2 CATGACCACTAGGAGCATCTTTGGCGAGATTGGGAGAATCGGTGGCATTGGAGTCTCCTAGGGGAATAAATCTTTGGGCACCTAGTGGTCATG

2 CATGACCACTAGGAGCATCTTTGGCGAGACGACTAATCCTACGGACGTCCAGTGGAGCTAGGGGAATAAATCTTTGGGCACCTAGTGGTCATG

2 CATGACCACTAGGAGCATCTTTGGCGAGAAAACTTTGGATTCGGGGACCAGTTGCTGCTAGGGGAATAAATCTTTGGGCACCTAGTGGTCATG

2 CATGACCACTAGGAGCATCTTTGGCGACAGTGGGCGAATAGTCAAATGGGAGTCGCTCTAGGGGAATAAATCTTTGGGCACCTAGTGGTCATG

2 CATGACCACTAGGAGCATCTTTGGCGAGATCGGGAGAATCGGTGGCAGTGGGGTCTCCTAGGGGAATAAATCTTTGGGCACCTAGTGGTCATG

2 CATGACCACTAGGAGCATCTTTGGCGAGATCGGGAGAATCGGTGGCAATTGTGTCTCCTAGGGGAATAAATCTTTGGGCACCTAGTGGTCATG

2 CATGACCACTAGGAGCATCTTTGGCGATATTAGATTGGATATCTTAGATGTTTCGTTCTAGGGGAATAAATCTTTGGGCACCTAGTGGTCATG

2 CATGACCACTAGGAGCATCTTTGGCGAGATCGGGAGGATCGGTGGCATCGGTGTCTCCTAGGGGAATAAATCTTTGGGCACCTAGTGGTCATG

2 CATGACCACTAGGAGCATCTTTGGCGACGGTGTGGGTGCAGGGCCAGGCGGTTGATTCTAGGGGAATAAATCTTTGGGCACCTAGTGGTCATG

2 CATGACCACTAGGAGCATCTTTGGCGAGATCGGGAGAATCGGTGGCATTGGTGTTTGCTAGGGGAATAAATCTTTGGGCACCTAGTGGTCATG

2 CATGACCACTAGGAGCATCTTTGGCGAAGACGACACAGTCGAGCGGGTTAGAGGAAGCTAGGGGAATAAATCTTTGGGCACCTAGTGGTCATG

2 CATGACCACTAGGAGCATCTTTGGCGAGATCGGGAGAATCGGTGGAAATGGTGTCTCCTAGGGGAATAAATCTTTGGGCACCTAGTGGTCATG

2 CATGACCACTAGGAGCATCTTTGGCGAGATCGGGAGAAACGGTGGCATTGGTGTCTACTAGGGGAATAAATCTTTGGGCACCTAGTGGTCATG

2 CATGACCACTAGGAGCATCTTTGGCGAGATTGGGATAATCGGTGGCATTGGTGTCTCCTAGGGGAATAAATCTTTGGGCACCTAGTGGTCATG

2 CATGACCACTAGGAGCATCTTTGGCGAGGGGTAGGGGGCCGCTCGCGCCAGCGTGCGCTAGGGGAATAAATCTTTGGGCACCTAGTGGTCATG

2 CATGACCACTAGGAGCATCTTTGGCGAATGGCCGGCACGGCCTTCTAGTCCTTGGTCCTAGGGGAATAAATCTTTGGGCACCTAGTGGTCATG

2 CATGACCACTAGGAGCATCTTTGGCGAACGTCAGGTGTTGCGGTGGTTTGTGAATCCCTAGGGGAATAAATCTTTGGGCACCTAGTGGTCATG

2 CATGACCACTAGGAGCATCTTTGGCGAGATCGGTACAATCGGTGGCATTGGTGTCTCCTAGGGGAATAAATCTTTGGGCACCTAGTGGTCATG

2 CATGACCACTAGGAGCATCTTTGGCGATCCTCGTCAGACGGTGAAGCAGACGTTTGGCTAGGGGAATAAATCTTTGGGCACCTAGTGGTCATG

2 CATGACCACTAGGAGCATCTTTGGCGAGAACGGGAGAATCGGTGGCAATGGTGTCTCCTAGGGGAATAAATCTTTGGGCACCTAGTGGTCATG

2 CATGACCACTAGGAGCATCTTTGGCGAGTACTGCTGCCGTCAAGGTAGAACAGTGTGCTAGGGGAATAAATCTTTGGGCACCTAGTGGTCATG

2 CATGACCACTAGGAGCATCTTTGGCGAAAGTCGGTTGAACAGGGTGGGGTGCTGTCCCTAGGGGAATAAATCTTTGGGCACCTAGTGGTCATG

2 CATGACCACTAGGAGCATCTTTGGCGAGAGAATCGGTAGGCAGAGTGTTCCAGCCAGCTAGGGGAATAAATCTTTGGGCACCTAGTGGTCATG

2 CATGACCACTAGGAGCATCTTTGGCGAATAGTTGTCGGGTATATCATGTAGATAAAACTAGGGGAATAAATCTTTGGGCACCTAGTGGTCATG

2 CATGACCACTAGGAGCATCTTTGGCGACCAGTGGTGGCACACTAACGAGACGTAGTGCTAGGGGAATAAATCTTTGGGCACCTAGTGGTCATG

2 CATGACCACTAGGAGCATCTTTGGCGAGATCGGGAGAATCATTGGCATTGGTGTCTCCTAGGGGAATAAATCTTTGGGCACCTAGTGGTCATG

2 CATGACCACTAGGAGCATCTTTGGCGACAAAAGGGATGCAAATAGTGGCAGAGACGCCTAGGGGAATAAATCTTTGGGCACCTAGTGGTCATG

2 CATGACCACTAGGAGCATCTTTGGCGAGATAGGGAGAATCGGTGGCATTGGTGTATCCTAGGGGAATAAATCTTTGGGCACCTAGTGGTCATG

2 CATGACCACTAGGAGCATCTTTGGCGAACCTCGCTTGGGAGGTTGCTCCACCAGTTCCTAGGGGAATAAATCTTTGGGCACCTAGTGGTCATG

2 CATGACCACTAGGAGCATCTTTGGCGAGATCGAGAGAATCGGTGGCCTTGGTGTCTCCTAGGGGAATAAATCTTTGGGCACCTAGTGGTCATG

2 CATGACCACTAGGAGCATCTTTGGCGACAATGTGGTCAGTGCCTGACGGAGCTAGCCCTAGGGGAATAAATCTTTGGGCACCTAGTGGTCATG

2 CATGACCACTAGGAGCATCTTTGGCGAACGGTAGGTGTTGCGGTGGTCTGTGAATTCCTAGGGGAATAAATCTTTGGGCACCTAGTGGTCATG

2 CATGACCACTAGGAGCATCTTTGGCGAGTTCGGGAGAATCGGTGGCAGTGGTGTCTCCTAGGGGAATAAATCTTTGGGCACCTAGTGGTCATG

2 CATGACCACTAGGAGCATCTTTGGCGAGATCAGGAGAATCGGTGGCACTGGTGTCTCCTAGGGGAATAAATCTTTGGGCACCTAGTGGTCATG

2 CATGACCACTAGGAGCATCTTTGGCGAGATCGGGAGAATCCCAGGCATTGGTGTCTCCTAGGGGAATAAATCTTTGGGCACCTAGTGGTCATG

2 CATGACCACTAGGAGCATCTTTGGCGAGAGGACTCTGGATTCGGGGACCAGTTGCTCCTAGGGGAATAAATCTTTGGGCACCTAGTGGTCATG

2 CATGACCACTAGGAGCATCTTTGGCGATCCGGGGGGGCTACGGGCCCGACGCTAGGGCTAGGGGAATAAATCTTTGGGCACCTAGTGGTCATG

2 CATGACCACTAGGAGCATCTTTGGCGACAAGTCGAGCAGAGCCGACGAGACGTAGTGCTAGGGGAATAAATCTTTGGGCACCTAGTGGTCATG

2 CATGACCACTAGGAGCATCTTTGGCGAGATCGGGAGAATCGGTGGAATTGGGGTCTCCTAGGGGAATAAATCTTTGGGCACCTAGTGGTCATG

2 CATGACCACTAGGAGCATCTTTGGCGATCCGGGGGACCTACGGTCGACGCTAAGGGGCTAGGGGAATAAATCTTTGGGCACCTAGTGGTCATG

2 CATGACCACTAGGAGCATCTTTGGCGATGGTGGGTGGAACCATGCTAGGGTCGTCGACTAGGGGAATAAATCTTTGGGCACCTAGTGGTCATG

2 CATGACCACTAGGAGCATCTTTGGCGAAGGGCAGGTGCTGCGGTGGTCTGTGAATCCCTAGGGGAATAAATCTTTGGGCACCTAGTGGTCATG

2 CATGACCACTAGGAGCATCTTTGGCGAGATCGAGAGAATCGGTGGCATTTGTGTCTCCTAGGGGAATAAATCTTTGGGCACCTAGTGGTCATG

2 CATGACCACTAGGAGCATCTTTGGCGATTGGTAGTCGCGGATGCAGGTAGTGATGCTCTAGGGGAATAAATCTTTGGGCACCTAGTGGTCATG

2 CATGACCACTAGGAGCATCTTTGGCGACGAGGAGCGGACGTTTATTGCGCTAGTCGACTAGGGGAATAAATCTTTGGGCACCTAGTGGTCATG

2 CATGACCACTAGGAGCATCTTTGGCGAGATCGGGAGAATCGGTGGCATTAGTGCCTCCTAGGGGAATAAATCTTTGGGCACCTAGTGGTCATG

2 CATGACCACTAGGAGCATCTTTGGCGACGTAGAGGGCATGGAGTAGGGACGCGTCGACTAGGGGAATAAATCTTTGGGCACCTAGTGGTCATG

2 CATGACCACTAGGAGCATCTTTGGCGAGGGAGGGCGCCGGCAGCTGTGTGAATGCGACTAGGGGAATAAATCTTTGGGCACCTAGTGGTCATG

2 CATGACCACTAGGAGCATCTTTGGCGAGCCTCGCTTGGGAGGTTGCTCCACCAGTTACTAGGGGAATAAATCTTTGGGCACCTAGTGGTCATG

2 CATGACCACTAGGAGCATCTTTGGCGATCCTCGTCAGGTGGTGAAGCAGACGTTTGGCTAGGGGAATAAATCTTTGGGCACCTAGTGGTCATG

2 CATGACCACTAGGAGCATCTTTGGCGAGATCGAGAGAATCGGTGGTATTGGTGTCTCCTAGGGGAATAAATCTTTGGGCACCTAGTGGTCATG

2 CATGACCACTAGGAGCATCTTTGGCGAGAAGACTCTGGAATCGGGGACCAGTTGCAGCTAGGGGAATAAATCTTTGGGCACCTAGTGGTCATG

2 CATGACCACTAGGAGCATCTTTGGCGATTGGGGTCGGCAGGTGAGTACCTTGGAGCTCTAGGGGAATAAATCTTTGGGCACCTAGTGGTCATG

2 CATGACCACTAGGAGCATCTTTGGCGAGATCGGGAGAATCGGTGGCAGTGGTGTCACCTAGGGGAATAAATCTTTGGGCACCTAGTGGTCATG

2 CATGACCACTAGGAGCATCTTTGGCGAGATCGGCAGAATCGGTGGCATCGGTGTCTCCTAGGGGAATAAATCTTTGGGCACCTAGTGGTCATG

2 CATGACCACTAGGAGCATCTTTGGCGACCAGATATGGTAGGTAATGTGATGAGGAGGCTAGGGGAATAAATCTTTGGGCACCTAGTGGTCATG

2 CATGACCACTAGGAGCATCTTTGGCGAGGTCCGAGCAAGCGAACCGCGAAAAGTCGGCTAGGGGAATAAATCTTTGGGCACCTAGTGGTCATG

2 CATGACCACTAGGAGCATCTTTGGCGAAGGGTGTAGGACTTCAAGTGGACCTCATAGCTAGGGGAATAAATCTTTGGGCACCTAGTGGTCATG

2 CATGACCACTAGGAGCATCTTTGGCGATCGCATAGCGTAGGACGAAAGCGTCAGGGACTAGGGGAATAAATCTTTGGGCACCTAGTGGTCATG

2 CATGACCACTAGGAGCATCTTTGGCGACGGCGTGGGGCCATTGATGCGGCGGCACTCCTAGGGGAATAAATCTTTGGGCACCTAGTGGTCATG

2 CATGACCACTAGGAGCATCTTTGGCGATTGGCTGGGAGAAGGGCTCTAAGTATTCAGCTAGGGGAATAAATCTTTGGGCACCTAGTGGTCATG

2 CATGACCACTAGGAGCATCTTTGGCGATTCGGGGGCCACTGAGGCGACGCTAATGGGCTAGGGGAATAAATCTTTGGGCACCTAGTGGTCATG

2 CATGACCACTAGGAGCATCTTTGGCGAAGATGTGGGCTAGGATGGAGAAGACGTCGACTAGGGGAATAAATCTTTGGGCACCTAGTGGTCATG

2 CATGACCACTAGGAGCATCTTTGGCGATTTCTGAATCCGTTTAATCAAAATGGAATTCTAGGGGAATAAATCTTTGGGCACCTAGTGGTCATG

2 CATGACCACTAGGAGCATCTTTGGCGAGATCGTGAGAATCGGAGGCATTGGTGTCTCCTAGGGGAATAAATCTTTGGGCACCTAGTGGTCATG

2 CATGACCACTAGGAGCATCTTTGGCGAGGGAGGGCGCCGGCAGCGGTGTTAATGCGACTAGGGGAATAAATCTTTGGGCACCTAGTGGTCATG

2 CATGACCACTAGGAGCATCTTTGGCGAGGGAGGGCGCAGGCAGCGGTGTGAATGCGCCTAGGGGAATAAATCTTTGGGCACCTAGTGGTCATG

2 CATGACCACTAGGAGCATCTTTGGCGAACGGCAGGAGTTGCGGTGGTTTGTGAATCCCTAGGGGAATAAATCTTTGGGCACCTAGTGGTCATG

2 CATGACCACTAGGAGCATCTTTGGCGACCAGGGGGGGCATGGACGATATGAGGTCGACTAGGGGAATAAATCTTTGGGCACCTAGTGGTCATG

2 CATGACCACTAGGAGCATCTTTGGCGACCGAGTGGGCTGGGAGGAAGTATTTTGGAGCTAGGGGAATAAATCTTTGGGCACCTAGTGGTCATG

2 CATGACCACTAGGAGCATCTTTGGCGAGTGGGGGGCGACGGCCGGTTGTGGGTGAGACTAGGGGAATAAATCTTTGGGCACCTAGTGGTCATG

2 CATGACCACTAGGAGCATCTTTGGCGAGATCGGGAGAATCGGTGGCATTGGAGTATCCTAGGGGAATAAATCTTTGGGCACCTAGTGGTCATG

2 CATGACCACTAGGAGCATCTTTGGCGATAGCAGAAGGTGTGAATGAAGAGGGATCTACTAGGGGAATAAATCTTTGGGCACCTAGTGGTCATG

2 CATGACCACTAGGAGCATCTTTGGCGAGATCGGGAGACTCGGTGGCATTGGTGTCTACTAGGGGAATAAATCTTTGGGCACCTAGTGGTCATG

2 CATGACCACTAGGAGCATCTTTGGCGATCGCACAGCGCACGAAGCCATACGAGGGGACTAGGGGAATAAATCTTTGGGCACCTAGTGGTCATG

2 CATGACCACTAGGAGCATCTTTGGCGACTAACGGCTGCTCTGTAACTTGGTCCGCCGCTAGGGGAATAAATCTTTGGGCACCTAGTGGTCATG

2 CATGACCACTAGGAGCATCTTTGGCGAGATCGGGAGAATCGGCGGCATTGGTTTCTCCTAGGGGAATAAATCTTTGGGCACCTAGTGGTCATG

2 CATGACCACTAGGAGCATCTTTGGCGAGATCGGGAGAATCGGCGGCTTTGGTGTCTCCTAGGGGAATAAATCTTTGGGCACCTAGTGGTCATG

2 CATGACCACTAGGAGCATCTTTGGCGAGTGGGCATGTCCTGGAGTTGTGGTACGAGACTAGGGGAATAAATCTTTGGGCACCTAGTGGTCATG

2 CATGACCACTAGGAGCATCTTTGGCGATCCCCCTTGGCACGGGTCTTAATGGATTTGCTAGGGGAATAAATCTTTGGGCACCTAGTGGTCATG

2 CATGACCACTAGGAGCATCTTTGGCGACCAACGGTGGAGATGGGACTAAGTGGGCAACTAGGGGAATAAATCTTTGGGCACCTAGTGGTCATG

2 CATGACCACTAGGAGCATCTTTGGCGAATCGTAGTTCATAATGTGTCAATTGGTGCTCTAGGGGAATAAATCTTTGGGCACCTAGTGGTCATG

2 CATGACCACTAGGAGCATCTTTGGCGATGGGTGCTGACGACCGTTCACACGGTTACTCTAGGGGAATAAATCTTTGGGCACCTAGTGGTCATG

2 CATGACCACTAGGAGCATCTTTGGCGAGATCGGGAGAATCGCCGGCATTGGTGTCTCCTAGGGGAATAAATCTTTGGGCACCTAGTGGTCATG

2 CATGACCACTAGGAGCATCTTTGGCGAGATCGGGAGACTCGGTGGCATGGGTGTCTCCTAGGGGAATAAATCTTTGGGCACCTAGTGGTCATG

2 CATGACCACTAGGAGCATCTTTGGCGAGGTTCGAGCGATGCGTGCCGTGGTCGAGCTCTAGGGGAATAAATCTTTGGGCACCTAGTGGTCATG

2 CATGACCACTAGGAGCATCTTTGGCGAGCCTTGCTTGGGAGGTTGCTCCACCAGCTCCTAGGGGAATAAATCTTTGGGCACCTAGTGGTCATG

2 CATGACCACTAGGAGCATCTTTGGCGAATGGCCGCCACGGCCTTCTAGTCCTCGGTACTAGGGGAATAAATCTTTGGGCACCTAGTGGTCATG

2 CATGACCACTAGGAGCATCTTTGGCGACGCAGGGGGCAAGGTCGGGGCAAACGTCGACTAGGGGAATAAATCTTTGGGCACCTAGTGGTCATG

2 CATGACCACTAGGAGCATCTTTGGCGATTAAGTGGTCAACGCGTTAAATGGGGTAGACTAGGGGAATAAATCTTTGGGCACCTAGTGGTCATG

2 CATGACCACTAGGAGCATCTTTGGCGAACGGCAGGTGTTGCGGTGGTCTGTGAATGTCTAGGGGAATAAATCTTTGGGCACCTAGTGGTCATG

2 CATGACCACTAGGAGCATCTTTGGCGAAGGGTGATGTTTAGCGATGTAATTTCTCTTCTAGGGGAATAAATCTTTGGGCACCTAGTGGTCATG

2 CATGACCACTAGGAGCATCTTTGGCGAGATCGCGAGAATCGGTGGCACTGGTGTCTCCTAGGGGAATAAATCTTTGGGCACCTAGTGGTCATG

2 CATGACCACTAGGAGCATCTTTGGCGAGATCGGGAGAATCGGCGGCAATGGTGTCTCCTAGGGGAATAAATCTTTGGGCACCTAGTGGTCATG

2 CATGACCACTAGGAGCATCTTTGGCGATAGGCGTGTACGGTCGAGCGAATGCGATAGCTAGGGGAATAAATCTTTGGGCACCTAGTGGTCATG

2 CATGACCACTAGGAGCATCTTTGGCGATCCGGGGGCACCTATGTGCGACTCTGTGGGCTAGGGGAATAAATCTTTGGGCACCTAGTGGTCATG

2 CATGACCACTAGGAGCATCTTTGGCGAATGGCCGGCACGGCTTCCTAGTCCTCGGTACTAGGGGAATAAATCTTTGGGCACCTAGTGGTCATG

2 CATGACCACTAGGAGCATCTTTGGCGAGGGTTCGGGATACGGTCCACAATTATTTGGCTAGGGGAATAAATCTTTGGGCACCTAGTGGTCATG

2 CATGACCACTAGGAGCATCTTTGGCGATCCGGGGGCACCTATGTGCGATGCTGTGGGCTAGGGGAATAAATCTTTGGGCACCTAGTGGTCATG

2 CATGACCACTAGGAGCATCTTTGGCGATCTAGCATAGCGGGAACGTCATGTGGGGGCCTAGGGGAATAAATCTTTGGGCACCTAGTGGTCATG

2 CATGACCACTAGGAGCATCTTTGGCGAGATCGGGAGAATCGGTGGCATCTGTGTCTCCTAGGGGAATAAATCTTTGGGCACCTAGTGGTCATG

2 CATGACCACTAGGAGCATCTTTGGCGATCGCATAGCGGCACGTAGAAGAGTGCGGGACTAGGGGAATAAATCTTTGGGCACCTAGTGGTCATG

2 CATGACCACTAGGAGCATCTTTGGCGAGATCGGGAGAATCGGTGGCATAGGTGTTTCCTAGGGGAATAAATCTTTGGGCACCTAGTGGTCATG

2 CATGACCACTAGGAGCATCTTTGGCGAGCCTCGCTTGGGAGGTTGCTCCACCAGTACCTAGGGGAATAAATCTTTGGGCACCTAGTGGTCATG

2 CATGACCACTAGGAGCATCTTTGGCGAAGTGGTATGCATTGTGGTCTTGGTTCAACCCTAGGGGAATAAATCTTTGGGCACCTAGTGGTCATG

2 CATGACCACTAGGAGCATCTTTGGCGAGATCGGGAGACTCGGTGGCATAGGTGTCTCCTAGGGGAATAAATCTTTGGGCACCTAGTGGTCATG

2 CATGACCACTAGGAGCATCTTTGGCGACAATGGAGCGACGGTGTGGTTTGAGTCCTACTAGGGGAATAAATCTTTGGGCACCTAGTGGTCATG

2 CATGACCACTAGGAGCATCTTTGGCGACACGACATTAGGAACGAATGCCGCTGTGGACTAGGGGAATAAATCTTTGGGCACCTAGTGGTCATG

2 CATGACCACTAGGAGCATCTTTGGCGACTCGAGTAGCGCAGTCTACCTGTGGTGGACCTAGGGGAATAAATCTTTGGGCACCTAGTGGTCATG

2 CATGACCACTAGGAGCATCTTTGGCGACTCATGGGTTGGCAGGGGGTATTTGGGCGACTAGGGGAATAAATCTTTGGGCACCTAGTGGTCATG

2 CATGACCACTAGGAGCATCTTTGGCGAGAACGCGAACGCGTAAAAGCAGACGTTTGGCTAGGGGAATAAATCTTTGGGCACCTAGTGGTCATG

2 CATGACCACTAGGAGCATCTTTGGCGAGGCTGGAGACCGTAGCGAAGAGCAACGCTCCTAGGGGAATAAATCTTTGGGCACCTAGTGGTCATG

2 CATGACCACTAGGAGCATCTTTGGCGAGAGATGAACCGTCAATACCCAGGCCGTCGCCTAGGGGAATAAATCTTTGGGCACCTAGTGGTCATG

2 CATGACCACTAGGAGCATCTTTGGCGAGATCGGGAGAATCGGTGGCAGTGGTGTCTTCTAGGGGAATAAATCTTTGGGCACCTAGTGGTCATG

2 CATGACCACTAGGAGCATCTTTGGCGAGATCGGGAGAATTGGTGGCACTGGTGTCTCCTAGGGGAATAAATCTTTGGGCACCTAGTGGTCATG

2 CATGACCACTAGGAGCATCTTTGGCGAACGGCAGTAGTTGCGGTGGTCTGTGAATCCCTAGGGGAATAAATCTTTGGGCACCTAGTGGTCATG

2 CATGACCACTAGGAGCATCTTTGGCGATTGTGGGAATGGCCGTTCCGCCCAACGACCCTAGGGGAATAAATCTTTGGGCACCTAGTGGTCATG

2 CATGACCACTAGGAGCATCTTTGGCGAGTGGTCGATGGGTCCGCTACAGTACTCGCTCTAGGGGAATAAATCTTTGGGCACCTAGTGGTCATG

2 CATGACCACTAGGAGCATCTTTGGCGACTAAGTGGCCCGCGCGTTAAATGGGGTAGACTAGGGGAATAAATCTTTGGGCACCTAGTGGTCATG

2 CATGACCACTAGGAGCATCTTTGGCGAGAAGACTCCGGATTCGGGGACCAGTTGTTGCTAGGGGAATAAATCTTTGGGCACCTAGTGGTCATG

2 CATGACCACTAGGAGCATCTTTGGCGACGACCAGTGGATTTAACGACCCGCGAAGTCCTAGGGGAATAAATCTTTGGGCACCTAGTGGTCATG

2 CATGACCACTAGGAGCATCTTTGGCGAGATCGGGAGAATCGGTAGCATTGGTGTTTCCTAGGGGAATAAATCTTTGGGCACCTAGTGGTCATG

2 CATGACCACTAGGAGCATCTTTGGCGAGTGGGGGGCGACGGCCTGTAGTGGGTGAGACTAGGGGAATAAATCTTTGGGCACCTAGTGGTCATG

2 CATGACCACTAGGAGCATCTTTGGCGACCAGAGAAGCTTCGTCGATGCGACCAGTGCCTAGGGGAATAAATCTTTGGGCACCTAGTGGTCATG

2 CATGACCACTAGGAGCATCTTTGGCGAAAGTCGGTAGAACAGGGTAAGGTGCTGTCCCTAGGGGAATAAATCTTTGGGCACCTAGTGGTCATG

2 CATGACCACTAGGAGCATCTTTGGCGACAGCGGCGTAGGCCGCCTTGGGTGGTGGCCCTAGGGGAATAAATCTTTGGGCACCTAGTGGTCATG

2 CATGACCACTAGGAGCATCTTTGGCGAGATCGGGGGAATCGGTGGCATTGGTGTCTGCTAGGGGAATAAATCTTTGGGCACCTAGTGGTCATG

2 CATGACCACTAGGAGCATCTTTGGCGATCGTAGGAAAACAAAAGTGGCCTTGCTAGCCTAGGGGAATAAATCTTTGGGCACCTAGTGGTCATG

2 CATGACCACTAGGAGCATCTTTGGCGACGTAGGGGGCATTGGGATTCAGAGAGTCGACTAGGGGAATAAATCTTTGGGCACCTAGTGGTCATG

2 CATGACCACTAGGAGCATCTTTGGCGAAGGCGAAGCACGCTGGTCCTGGTGGCGTCCCTAGGGGAATAAATCTTTGGGCACCTAGTGGTCATG

2 CATGACCACTAGGAGCATCTTTGGCGAACGTCGGTGGATGGTCCCACGAATTGGAACCTAGGGGAATAAATCTTTGGGCACCTAGTGGTCATG

2 CATGACCACTAGGAGCATCTTTGGCGAGATCGGGAGAATCGGTGGCATTGGTTTCTACTAGGGGAATAAATCTTTGGGCACCTAGTGGTCATG

2 CATGACCACTAGGAGCATCTTTGGCGAACGGCAGGGGATGTAGAAATAGTGAGACAGCTAGGGGAATAAATCTTTGGGCACCTAGTGGTCATG

2 CATGACCACTAGGAGCATCTTTGGCGATCGCTTAGCACAGATAGTGGATCTGTAGGCCTAGGGGAATAAATCTTTGGGCACCTAGTGGTCATG

2 CATGACCACTAGGAGCATCTTTGGCGAGATCGGGAGAAACGGTGGCAATGGTGTCTCCTAGGGGAATAAATCTTTGGGCACCTAGTGGTCATG

2 CATGACCACTAGGAGCATCTTTGGCGACGGGTGGGCGCCCAAGGGTGTGAGTGTTGCCTAGGGGAATAAATCTTTGGGCACCTAGTGGTCATG

2 CATGACCACTAGGAGCATCTTTGGCGACCGGCAGGTGTTGCGGTGGTCTGTGGATCCCTAGGGGAATAAATCTTTGGGCACCTAGTGGTCATG

2 CATGACCACTAGGAGCATCTTTGGCGAATGGGCGGCACGGCCTTCTAGTCCTCGGTACTAGGGGAATAAATCTTTGGGCACCTAGTGGTCATG

2 CATGACCACTAGGAGCATCTTTGGCGAACTCATTGGAGTGGGTGTAACGAATCCGTACTAGGGGAATAAATCTTTGGGCACCTAGTGGTCATG

2 CATGACCACTAGGAGCATCTTTGGCGAACGGCAGGTGTTGCGGTGGTTTGTTAATCCCTAGGGGAATAAATCTTTGGGCACCTAGTGGTCATG

2 CATGACCACTAGGAGCATCTTTGGCGAACGGCAGGAGTTGCGGTGGTCTGTGAAACCCTAGGGGAATAAATCTTTGGGCACCTAGTGGTCATG

2 CATGACCACTAGGAGCATCTTTGGCGATATCGGGAGAATCGGTGGCATTGGTGTCACCTAGGGGAATAAATCTTTGGGCACCTAGTGGTCATG

2 CATGACCACTAGGAGCATCTTTGGCGAGATTGGGAGAATCGGTGGCATTGGTTTCTCCTAGGGGAATAAATCTTTGGGCACCTAGTGGTCATG

2 CATGACCACTAGGAGCATCTTTGGCGAAAAGACTCCGGATTCGGGGACCAGTTGCTGCTAGGGGAATAAATCTTTGGGCACCTAGTGGTCATG

2 CATGACCACTAGGAGCATCTTTGGCGAGATAGGGAGAATCGGTGGCATTGTTGTCTCCTAGGGGAATAAATCTTTGGGCACCTAGTGGTCATG

2 CATGACCACTAGGAGCATCTTTGGCGAACGGCAGGGGTTGCGGTGGTCTGTGAAACCCTAGGGGAATAAATCTTTGGGCACCTAGTGGTCATG

2 CATGACCACTAGGAGCATCTTTGGCGAGGGGTCATGGGAATGTAACTGCGCTTAAGACTAGGGGAATAAATCTTTGGGCACCTAGTGGTCATG

2 CATGACCACTAGGAGCATCTTTGGCGAGAGTTTGTAATCTTCGTATGTTTTTAAGTGCTAGGGGAATAAATCTTTGGGCACCTAGTGGTCATG

2 CATGACCACTAGGAGCATCTTTGGCGAGCGCAACGTGCCGGATTGGGGGGTCCAGCCCTAGGGGAATAAATCTTTGGGCACCTAGTGGTCATG

2 CATGACCACTAGGAGCATCTTTGGCGACGTAGTGGGGTGGTAGGGAGTACTTGTTGCCTAGGGGAATAAATCTTTGGGCACCTAGTGGTCATG

2 CATGACCACTAGGAGCATCTTTGGCGATGAGGGCTCGACCCATTCGTGACACGAAGCCTAGGGGAATAAATCTTTGGGCACCTAGTGGTCATG

2 CATGACCACTAGGAGCATCTTTGGCGAACGGCAGGTGTTGCGGTTGTCTGTGGATCCCTAGGGGAATAAATCTTTGGGCACCTAGTGGTCATG

2 CATGACCACTAGGAGCATCTTTGGCGAGAGCGGGAGAATCGGTGGCATCGGTGTCTCCTAGGGGAATAAATCTTTGGGCACCTAGTGGTCATG

2 CATGACCACTAGGAGCATCTTTGGCGAGGGAGGGCACCGGCAGCGGTGTGAATGCGACTAGGGGAATAAATCTTTGGGCACCTAGTGGTCATG

2 CATGACCACTAGGAGCATCTTTGGCGAGACCGGGAGAATCGGCGGCATTGGTGTCTCCTAGGGGAATAAATCTTTGGGCACCTAGTGGTCATG

2 CATGACCACTAGGAGCATCTTTGGCGAGTTTGGTGATGTGGGCGGTCTCTTGGGCTACTAGGGGAATAAATCTTTGGGCACCTAGTGGTCATG

2 CATGACCACTAGGAGCATCTTTGGCGAAAGTCGGTAGAACAGGGTGGGGTGTTGTTCCTAGGGGAATAAATCTTTGGGCACCTAGTGGTCATG

2 CATGACCACTAGGAGCATCTTTGGCGACAGGGGATGCTGATGCGCTGGACGACCGAGCTAGGGGAATAAATCTTTGGGCACCTAGTGGTCATG

2 CATGACCACTAGGAGCATCTTTGGCGACGTTGGCTGGTAGGCGATCGTATTAAGTGCCTAGGGGAATAAATCTTTGGGCACCTAGTGGTCATG

2 CATGACCACTAGGAGCATCTTTGGCGAACGGCAGGTGTTGCGGTGGTTTGTGAACCCCTAGGGGAATAAATCTTTGGGCACCTAGTGGTCATG

2 CATGACCACTAGGAGCATCTTTGGCGAGGATCGGCACTCGGAACGTGTCCCGGATTACTAGGGGAATAAATCTTTGGGCACCTAGTGGTCATG

2 CATGACCACTAGGAGCATCTTTGGCGAGATGGAACGAGAGCGACCAGGAATCGATGCCTAGGGGAATAAATCTTTGGGCACCTAGTGGTCATG

2 CATGACCACTAGGAGCATCTTTGGCGAGTCCGTGAAAGTTGCGTATGAGTGGCACGCCTAGGGGAATAAATCTTTGGGCACCTAGTGGTCATG

2 CATGACCACTAGGAGCATCTTTGGCGAACATTTTAGCAAACGGTCTTTGTTGGGGGTCTAGGGGAATAAATCTTTGGGCACCTAGTGGTCATG

2 CATGACCACTAGGAGCATCTTTGGCGAGGGGATCTGAGGCATGGCGGTAAGTGCGACCTAGGGGAATAAATCTTTGGGCACCTAGTGGTCATG

2 CATGACCACTAGGAGCATCTTTGGCGATCATCGAAGTGGACGAAGCTAAACAAGCGCCTAGGGGAATAAATCTTTGGGCACCTAGTGGTCATG

2 CATGACCACTAGGAGCATCTTTGGCGAGATCGGGAGAATCTGTGGCATTGGTCTCTCCTAGGGGAATAAATCTTTGGGCACCTAGTGGTCATG

2 CATGACCACTAGGAGCATCTTTGGCGAGCCTTGCGTGGGAGGTTGCTCCACCAGTTCCTAGGGGAATAAATCTTTGGGCACCTAGTGGTCATG

2 CATGACCACTAGGAGCATCTTTGGCGAGTAAGGTGTGGGCGGTGAGAAACAATACGACTAGGGGAATAAATCTTTGGGCACCTAGTGGTCATG

2 CATGACCACTAGGAGCATCTTTGGCGAAGCAGTGCGACGGGCTATGGGTTCTTCGAGCTAGGGGAATAAATCTTTGGGCACCTAGTGGTCATG

2 CATGACCACTAGGAGCATCTTTGGCGACCAGTGGTGGTGCGAAGTGGGTTGGTTCGGCTAGGGGAATAAATCTTTGGGCACCTAGTGGTCATG

2 CATGACCACTAGGAGCATCTTTGGCGACTCGGGTAGCGCAATAGCGCGGCAGGTGCTCTAGGGGAATAAATCTTTGGGCACCTAGTGGTCATG

2 CATGACCACTAGGAGCATCTTTGGCGACGAGGTCAAGGGAATCCGTATCGAAGTGGACTAGGGGAATAAATCTTTGGGCACCTAGTGGTCATG

2 CATGACCACTAGGAGCATCTTTGGCGATCCGGGGGGACCGTGTGTCCGACGCATGGGCTAGGGGAATAAATCTTTGGGCACCTAGTGGTCATG

2 CATGACCACTAGGAGCATCTTTGGCGATCCGGGGGTCACAAGTGACGTCGCATAGGGCTAGGGGAATAAATCTTTGGGCACCTAGTGGTCATG

2 CATGACCACTAGGAGCATCTTTGGCGAGATCGCGAGGATCGGTGGCATTGGTGTCTCCTAGGGGAATAAATCTTTGGGCACCTAGTGGTCATG

2 CATGACCACTAGGAGCATCTTTGGCGAACGACGATCGTCATAGTGGCGTCTTGGAACCTAGGGGAATAAATCTTTGGGCACCTAGTGGTCATG

2 CATGACCACTAGGAGCATCTTTGGCGAGAACGGGAGAATCGGTGGAATTGGTGTCTCCTAGGGGAATAAATCTTTGGGCACCTAGTGGTCATG

2 CATGACCACTAGGAGCATCTTTGGCGACTGCCGAGGATGAGTTAAGTGGTCGAGCGCCTAGGGGAATAAATCTTTGGGCACCTAGTGGTCATG

2 CATGACCACTAGGAGCATCTTTGGCGAGATCGGGAGAATCGGTGGCATTGTTGACTCCTAGGGGAATAAATCTTTGGGCACCTAGTGGTCATG

2 CATGACCACTAGGAGCATCTTTGGCGAGATCGGGAGTATCGGTGGCAGTGGTGTCTCCTAGGGGAATAAATCTTTGGGCACCTAGTGGTCATG

2 CATGACCACTAGGAGCATCTTTGGCGAGGGTAGGCGTCAGGATGATGTGTATTTGCTCTAGGGGAATAAATCTTTGGGCACCTAGTGGTCATG

2 CATGACCACTAGGAGCATCTTTGGCGACGTGGTGTGTTGCCCAAGTGGCTGAGTCGACTAGGGGAATAAATCTTTGGGCACCTAGTGGTCATG

2 CATGACCACTAGGAGCATCTTTGGCGATGGGCCTAAGTGCGTATCTGTGGTCGAGCTCTAGGGGAATAAATCTTTGGGCACCTAGTGGTCATG

2 CATGACCACTAGGAGCATCTTTGGCGAGGTCGAGAAGAAGTGAGGATACAGGTGCCTCTAGGGGAATAAATCTTTGGGCACCTAGTGGTCATG

2 CATGACCACTAGGAGCATCTTTGGCGAGATCGGGAGAATCGGAGTCATTGGTGTCTCCTAGGGGAATAAATCTTTGGGCACCTAGTGGTCATG

2 CATGACCACTAGGAGCATCTTTGGCGAGATCTGGAGAATCGGTGGCATTGATGTCTCCTAGGGGAATAAATCTTTGGGCACCTAGTGGTCATG

2 CATGACCACTAGGAGCATCTTTGGCGACCGTAGGAAAACAAAAGTGTCCTTGCTAGCCTAGGGGAATAAATCTTTGGGCACCTAGTGGTCATG

2 CATGACCACTAGGAGCATCTTTGGCGAGATCGGGAGAATAGGTGGAATTGGTGTCTCCTAGGGGAATAAATCTTTGGGCACCTAGTGGTCATG

2 CATGACCACTAGGAGCATCTTTGGCGAGATCGGGAGAATCGGTGTCATTGGTGTTTCCTAGGGGAATAAATCTTTGGGCACCTAGTGGTCATG

2 CATGACCACTAGGAGCATCTTTGGCGATACGCATAGCGAGACGAAGAAAGTCTGGGCCTAGGGGAATAAATCTTTGGGCACCTAGTGGTCATG

2 CATGACCACTAGGAGCATCTTTGGCGAGGGGGCGAGTGCGCGAGGTGTTATAGGGAGCTAGGGGAATAAATCTTTGGGCACCTAGTGGTCATG

2 CATGACCACTAGGAGCATCTTTGGCGACTCGTGTAGGTTCCAGGGACCGGCCTGTGACTAGGGGAATAAATCTTTGGGCACCTAGTGGTCATG

2 CATGACCACTAGGAGCATCTTTGGCGAGATTGGGAGAACCGGTGGCATTGGTGTCTCCTAGGGGAATAAATCTTTGGGCACCTAGTGGTCATG

2 CATGACCACTAGGAGCATCTTTGGCGATTCCTCGAATCGGGAACTCGGGGACGGTGCCTAGGGGAATAAATCTTTGGGCACCTAGTGGTCATG

2 CATGACCACTAGGAGCATCTTTGGCGAGTCGTCGGGTACGATGTGATTAAGATGAGACTAGGGGAATAAATCTTTGGGCACCTAGTGGTCATG

2 CATGACCACTAGGAGCATCTTTGGCGAGATCTGGAGAATCGGTGGCATTGGTGTCTTCTAGGGGAATAAATCTTTGGGCACCTAGTGGTCATG

2 CATGACCACTAGGAGCATCTTTGGCGAGGGAGGGCGCCGGCAGCGGTGTGGATGCGCCTAGGGGAATAAATCTTTGGGCACCTAGTGGTCATG

2 CATGACCACTAGGAGCATCTTTGGCGAGATCGGGAGAATTGGTGGCGTTGGTGTCTCCTAGGGGAATAAATCTTTGGGCACCTAGTGGTCATG

2 CATGACCACTAGGAGCATCTTTGGCGACTGTGGGCACTACGCAATACCTCGGTCCGCCTAGGGGAATAAATCTTTGGGCACCTAGTGGTCATG

2 CATGACCACTAGGAGCATCTTTGGCGAACACCGAGCGCCGTGGGCACGGGTTGGAACCTAGGGGAATAAATCTTTGGGCACCTAGTGGTCATG

2 CATGACCACTAGGAGCATCTTTGGCGATCGCATAGCGGACGGATTCCGAAGAGGGGCCTAGGGGAATAAATCTTTGGGCACCTAGTGGTCATG

2 CATGACCACTAGGAGCATCTTTGGCGAACTTGATCGAGACGATCGAGCGAAGTTGGACTAGGGGAATAAATCTTTGGGCACCTAGTGGTCATG

2 CATGACCACTAGGAGCATCTTTGGCGATTAGCTGAGCGGTTTGTTGAAAAAAGGGGCCTAGGGGAATAAATCTTTGGGCACCTAGTGGTCATG

2 CATGACCACTAGGAGCATCTTTGGCGATATCGGGAGAATCAGTGGCATTGGTGTCTCCTAGGGGAATAAATCTTTGGGCACCTAGTGGTCATG

2 CATGACCACTAGGAGCATCTTTGGCGAGCCACGCTTGGGAGGTTGCTCCACCAGTTCCTAGGGGAATAAATCTTTGGGCACCTAGTGGTCATG

2 CATGACCACTAGGAGCATCTTTGGCGACCAGATATGGTCGGTAATGTGCTGAGGAGGCTAGGGGAATAAATCTTTGGGCACCTAGTGGTCATG

2 CATGACCACTAGGAGCATCTTTGGCGAGATCGGGAGAAACGGTGGCATTGGTGTCACCTAGGGGAATAAATCTTTGGGCACCTAGTGGTCATG

2 CATGACCACTAGGAGCATCTTTGGCGAAATCGGGAGAATCGGTGGCATTGGTGTCTTCTAGGGGAATAAATCTTTGGGCACCTAGTGGTCATG

2 CATGACCACTAGGAGCATCTTTGGCGAACGGCAGGTGTTGCGGTGGTCTGTGTAACCCTAGGGGAATAAATCTTTGGGCACCTAGTGGTCATG

2 CATGACCACTAGGAGCATCTTTGGCGAGTATTGGCTGGTAGGTTGCGTATTGGGAAGCTAGGGGAATAAATCTTTGGGCACCTAGTGGTCATG

2 CATGACCACTAGGAGCATCTTTGGCGACCGGCAGGGGTTGCGGTGGTCTGTGAATCCCTAGGGGAATAAATCTTTGGGCACCTAGTGGTCATG

2 CATGACCACTAGGAGCATCTTTGGCGAGAACTCGACTGGCGGGATCTATGGGGCGACCTAGGGGAATAAATCTTTGGGCACCTAGTGGTCATG

2 CATGACCACTAGGAGCATCTTTGGCGATTTAGGTTTTTATGTGCTTTTAACCATTAGCTAGGGGAATAAATCTTTGGGCACCTAGTGGTCATG

2 CATGACCACTAGGAGCATCTTTGGCGATTCGCTAAGATATGTCAGCTTTCCGGCCACCTAGGGGAATAAATCTTTGGGCACCTAGTGGTCATG

2 CATGACCACTAGGAGCATCTTTGGCGACGGTCGATGTGGGTTAGAATTAGATTCGAGCTAGGGGAATAAATCTTTGGGCACCTAGTGGTCATG

2 CATGACCACTAGGAGCATCTTTGGCGAGAACGGGAGAATCGGTGGCATAGGTGTCTCCTAGGGGAATAAATCTTTGGGCACCTAGTGGTCATG

2 CATGACCACTAGGAGCATCTTTGGCGAACGGCAGGTGTTGCGGTGTTCTGTGTATCCCTAGGGGAATAAATCTTTGGGCACCTAGTGGTCATG

2 CATGACCACTAGGAGCATCTTTGGCGAGATCCGGAGAGTCGGTGGCATTGGTGTCTCCTAGGGGAATAAATCTTTGGGCACCTAGTGGTCATG

2 CATGACCACTAGGAGCATCTTTGGCGAGAAGATTCTGGATTCGGGGGCCAGTTGCTGCTAGGGGAATAAATCTTTGGGCACCTAGTGGTCATG

2 CATGACCACTAGGAGCATCTTTGGCGAGATCGGGAGAATCTGTGGCATTGGTGTCCCCTAGGGGAATAAATCTTTGGGCACCTAGTGGTCATG

2 CATGACCACTAGGAGCATCTTTGGCGATCGCAGAGCGGAGACGTTCTACGGCCGGGACTAGGGGAATAAATCTTTGGGCACCTAGTGGTCATG

2 CATGACCACTAGGAGCATCTTTGGCGAACGTCAGGTTTTGCGGTGGTCTGTGAATCCCTAGGGGAATAAATCTTTGGGCACCTAGTGGTCATG

2 CATGACCACTAGGAGCATCTTTGGCGAGATAGGGAGAATCGGTTGCATTGGTGTCTCCTAGGGGAATAAATCTTTGGGCACCTAGTGGTCATG

2 CATGACCACTAGGAGCATCTTTGGCGATGGGGCCGTGTTATCTGCACACTCCGCGGGCTAGGGGAATAAATCTTTGGGCACCTAGTGGTCATG

2 CATGACCACTAGGAGCATCTTTGGCGAAAGTCGGTAGAACAGGGTGGGGTACTGTCCCTAGGGGAATAAATCTTTGGGCACCTAGTGGTCATG

2 CATGACCACTAGGAGCATCTTTGGCGAGAAGAATTTGGATTCGGGGACCAGTTGCTGCTAGGGGAATAAATCTTTGGGCACCTAGTGGTCATG

2 CATGACCACTAGGAGCATCTTTGGCGAGTGGGGTCGTGGTTAAATCCGTGCTCGAGCCTAGGGGAATAAATCTTTGGGCACCTAGTGGTCATG

2 CATGACCACTAGGAGCATCTTTGGCGAGCCGATTGTGTAACAATGTGTTCTGGGTAACTAGGGGAATAAATCTTTGGGCACCTAGTGGTCATG

2 CATGACCACTAGGAGCATCTTTGGCGACGGCGTGGGGGAGAAATCCGGCGGTGACTGCTAGGGGAATAAATCTTTGGGCACCTAGTGGTCATG

2 CATGACCACTAGGAGCATCTTTGGCGAGAAGACTCTGGATTCGGGTACCAGTTGCTCCTAGGGGAATAAATCTTTGGGCACCTAGTGGTCATG

2 CATGACCACTAGGAGCATCTTTGGCGAGATTGGCAGAATCGGTGGCATTGGTGTCTCCTAGGGGAATAAATCTTTGGGCACCTAGTGGTCATG

2 CATGACCACTAGGAGCATCTTTGGCGAACGGCAGGTGTTGCGGTGGGTTGTGAATCCCTAGGGGAATAAATCTTTGGGCACCTAGTGGTCATG

2 CATGACCACTAGGAGCATCTTTGGCGACGGTGTGGGGCTCCATGATGCGGCGGATTCCTAGGGGAATAAATCTTTGGGCACCTAGTGGTCATG

2 CATGACCACTAGGAGCATCTTTGGCGAGATCAGGAGAATCAGTGGCATTGGTGTCTCCTAGGGGAATAAATCTTTGGGCACCTAGTGGTCATG

2 CATGACCACTAGGAGCATCTTTGGCGATATCGGGAGAATCGTTGGCATTGGTGTCTCCTAGGGGAATAAATCTTTGGGCACCTAGTGGTCATG

2 CATGACCACTAGGAGCATCTTTGGCGAGAAGACTCTGGATTCGGGGACCAGCTGCTCCTAGGGGAATAAATCTTTGGGCACCTAGTGGTCATG

2 CATGACCACTAGGAGCATCTTTGGCGAACGGCAGGTGTTGCGGTGGTTTCTGAATCCCTAGGGGAATAAATCTTTGGGCACCTAGTGGTCATG

2 CATGACCACTAGGAGCATCTTTGGCGAGCCCGACTGGACGTCCTGAGATCTGGGATTCTAGGGGAATAAATCTTTGGGCACCTAGTGGTCATG

2 CATGACCACTAGGAGCATCTTTGGCGAGATCGGGAGACTCGGTGGCATTGGTGACTCCTAGGGGAATAAATCTTTGGGCACCTAGTGGTCATG

2 CATGACCACTAGGAGCATCTTTGGCGATATCGGGATAATCGGTGGCATTGGTGTCTCCTAGGGGAATAAATCTTTGGGCACCTAGTGGTCATG

2 CATGACCACTAGGAGCATCTTTGGCGAGTCATCATAAACGGTTGTATTGGACCCCGGCTAGGGGAATAAATCTTTGGGCACCTAGTGGTCATG

2 CATGACCACTAGGAGCATCTTTGGCGAACGGCAGGTGTTGCGGTGGTCTTTGAAACCCTAGGGGAATAAATCTTTGGGCACCTAGTGGTCATG

2 CATGACCACTAGGAGCATCTTTGGCGATCGCACAGCGCACGGGATCGAAGGAGGGGCCTAGGGGAATAAATCTTTGGGCACCTAGTGGTCATG

2 CATGACCACTAGGAGCATCTTTGGCGAACGGCAGGTGATGCGGTGGTCTGTGAATACCTAGGGGAATAAATCTTTGGGCACCTAGTGGTCATG

2 CATGACCACTAGGAGCATCTTTGGCGAGGCGGAGGGTCCGCCGGGGACCAGTTGCTGCTAGGGGAATAAATCTTTGGGCACCTAGTGGTCATG

2 CATGACCACTAGGAGCATCTTTGGCGAGCCGATTGTGCAACAATATGTTCTGGGTAACTAGGGGAATAAATCTTTGGGCACCTAGTGGTCATG

2 CATGACCACTAGGAGCATCTTTGGCGAGATTGAAAGAATCGGTGGCATTGGTGTCTCCTAGGGGAATAAATCTTTGGGCACCTAGTGGTCATG

2 CATGACCACTAGGAGCATCTTTGGCGAACGGCAGGAGATGCGGTGGTCTGTGAATCCCTAGGGGAATAAATCTTTGGGCACCTAGTGGTCATG

2 CATGACCACTAGGAGCATCTTTGGCGATCGCAAAAGCGGCAGGAGAAACCGTCGGGCCTAGGGGAATAAATCTTTGGGCACCTAGTGGTCATG

2 CATGACCACTAGGAGCATCTTTGGCGACGGAGTGGGGCTCATTGTGCGGCGGTGATCCTAGGGGAATAAATCTTTGGGCACCTAGTGGTCATG

2 CATGACCACTAGGAGCATCTTTGGCGAGATCGGGAGAATCGGTGGCGTTGGTGTCCCCTAGGGGAATAAATCTTTGGGCACCTAGTGGTCATG

2 CATGACCACTAGGAGCATCTTTGGCGACCTAGTGGGCTGGGGTGTTAGGTAAGGCGCCTAGGGGAATAAATCTTTGGGCACCTAGTGGTCATG

2 CATGACCACTAGGAGCATCTTTGGCGACCAACAATCACGGGATTTAGTGAGTTAGTCCTAGGGGAATAAATCTTTGGGCACCTAGTGGTCATG

2 CATGACCACTAGGAGCATCTTTGGCGACAATGGAGCGACGGTGTGGTCTGAGTCATACTAGGGGAATAAATCTTTGGGCACCTAGTGGTCATG

2 CATGACCACTAGGAGCATCTTTGGCGACGGCGTGGGACTATAGATGCGGCGGCACTCCTAGGGGAATAAATCTTTGGGCACCTAGTGGTCATG

2 CATGACCACTAGGAGCATCTTTGGCGATTGCGGGTGTAAGGGTGCTGTTTTTTACGTCTAGGGGAATAAATCTTTGGGCACCTAGTGGTCATG

2 CATGACCACTAGGAGCATCTTTGGCGAGATAGGGAGAATCGGTGGCATTGGAGTCTCCTAGGGGAATAAATCTTTGGGCACCTAGTGGTCATG

2 CATGACCACTAGGAGCATCTTTGGCGAGAAGACTCTGGATTCGGGGACCAGTTGCGACTAGGGGAATAAATCTTTGGGCACCTAGTGGTCATG

2 CATGACCACTAGGAGCATCTTTGGCGAGATCGGGAGAATTGGTGGCATTGGTTTCTCCTAGGGGAATAAATCTTTGGGCACCTAGTGGTCATG

2 CATGACCACTAGGAGCATCTTTGGCGACGAGGAAAGAGAGAGATGGGCGAGTACTGACTAGGGGAATAAATCTTTGGGCACCTAGTGGTCATG

2 CATGACCACTAGGAGCATCTTTGGCGACAATGGAGCGATGGTGTGGTCTGAGTCCTACTAGGGGAATAAATCTTTGGGCACCTAGTGGTCATG

2 CATGACCACTAGGAGCATCTTTGGCGACAATGGAGCGACGGTGTGGCCTGAGTCCTACTAGGGGAATAAATCTTTGGGCACCTAGTGGTCATG

2 CATGACCACTAGGAGCATCTTTGGCGACCCGACATCACGTAAGGATGTAGAGGTGGACTAGGGGAATAAATCTTTGGGCACCTAGTGGTCATG

2 CATGACCACTAGGAGCATCTTTGGCGACCGAGTGGGCTGGGAGGAAGTATTTCGTAGCTAGGGGAATAAATCTTTGGGCACCTAGTGGTCATG

2 CATGACCACTAGGAGCATCTTTGGCGAGATCGGGAGAGTCGGTGGCATTGGTGTCTGCTAGGGGAATAAATCTTTGGGCACCTAGTGGTCATG

2 CATGACCACTAGGAGCATCTTTGGCGAACGGCAAATGTTGCGGTGGTCTGTGAATCCCTAGGGGAATAAATCTTTGGGCACCTAGTGGTCATG

2 CATGACCACTAGGAGCATCTTTGGCGAGAAGACTCTGGAGTCGGGGACCAGTTGCTCCTAGGGGAATAAATCTTTGGGCACCTAGTGGTCATG

2 CATGACCACTAGGAGCATCTTTGGCGATGGGTGCTGACGGGGCAAGTGAGCCCTACACTAGGGGAATAAATCTTTGGGCACCTAGTGGTCATG

2 CATGACCACTAGGAGCATCTTTGGCGAGCCTTGCTTGGGAGGTTGCTCCACCGGTTCCTAGGGGAATAAATCTTTGGGCACCTAGTGGTCATG

2 CATGACCACTAGGAGCATCTTTGGCGAGATCGGGGGAATCAGTGGCATTGGTGTCTCCTAGGGGAATAAATCTTTGGGCACCTAGTGGTCATG

2 CATGACCACTAGGAGCATCTTTGGCGATCCGGGGGCCAGAGGCGACGCAAATTAGGGCTAGGGGAATAAATCTTTGGGCACCTAGTGGTCATG

2 CATGACCACTAGGAGCATCTTTGGCGATCAGCATAGCGAAGTCAGAGGAGACGGGGCCTAGGGGAATAAATCTTTGGGCACCTAGTGGTCATG

2 CATGACCACTAGGAGCATCTTTGGCGAGATCGGGAGAATCGGTGGCATCGGTGTCTACTAGGGGAATAAATCTTTGGGCACCTAGTGGTCATG

2 CATGACCACTAGGAGCATCTTTGGCGAACGGAAGGGGTTGCGGTGGTCTGTGAATCCCTAGGGGAATAAATCTTTGGGCACCTAGTGGTCATG

2 CATGACCACTAGGAGCATCTTTGGCGAGGAGTGGCTCAGGGCGGTGGGTAGGTCGCGCTAGGGGAATAAATCTTTGGGCACCTAGTGGTCATG

2 CATGACCACTAGGAGCATCTTTGGCGATATCGGGAGAATCGGTGGCATTTGTGTCTCCTAGGGGAATAAATCTTTGGGCACCTAGTGGTCATG

2 CATGACCACTAGGAGCATCTTTGGCGAGAAGACTCTAAATTCGGGGACCAGTTGCTGCTAGGGGAATAAATCTTTGGGCACCTAGTGGTCATG

2 CATGACCACTAGGAGCATCTTTGGCGATTGGTCAGGAACGAGAGGAGGAGAACGAGACTAGGGGAATAAATCTTTGGGCACCTAGTGGTCATG

2 CATGACCACTAGGAGCATCTTTGGCGATCGCACAGCGGAACTGGCAGCAGTCGGGCTCTAGGGGAATAAATCTTTGGGCACCTAGTGGTCATG

2 CATGACCACTAGGAGCATCTTTGGCGAACGGCAGGAGTTGCGGTGGTCAGTGAATCCCTAGGGGAATAAATCTTTGGGCACCTAGTGGTCATG

2 CATGACCACTAGGAGCATCTTTGGCGAGATCGTGAGAATCGGTGGAATTGGTGTCTCCTAGGGGAATAAATCTTTGGGCACCTAGTGGTCATG

2 CATGACCACTAGGAGCATCTTTGGCGAAATGGAATCGGGTCACGTGGTATCGCTCCCCTAGGGGAATAAATCTTTGGGCACCTAGTGGTCATG

2 CATGACCACTAGGAGCATCTTTGGCGAAAGGCAGGTGTTGCGGTGGTCTGTGAATCACTAGGGGAATAAATCTTTGGGCACCTAGTGGTCATG

2 CATGACCACTAGGAGCATCTTTGGCGAGATCGGGAGAATCGGTCCCAAAGGTGTCTCCTAGGGGAATAAATCTTTGGGCACCTAGTGGTCATG

1 CATGACCACTAGGAGCATCTTTGGCGATGGGTTCTGACGGCCGCCGCTGCGGCTACACTAGGGGAATAAATCTTTGGGCACCTAGTGGTCATG

1 CATGACCACTAGGAGCATCTTTGGCGAGGGAGGGCGCCGGCAGCAGTGTGAATGCGACTAGGGGAATAAATCTTTGGGCACCTAGTGGTCATG

1 CATGACCACTAGGAGCATCTTTGGCGACGGTGTGGGGAACTTGTTTCGGCGGAGCTACTAGGGGAATAAATCTTTGGGCACCTAGTGGTCATG

1 CATGACCACTAGGAGCATCTTTGGCGAGTTGTCATAGAGCCACGGGACACCCGTCAGCTAGGGGAATAAATCTTTGGGCACCTAGTGGTCATG

1 CATGACCACTAGGAGCATCTTTGGCGAAGGTGCCTCTGGAAAATGGGTCGGTCCGTCCTAGGGGAATAAATCTTTGGGCACCTAGTGGTCATG

1 CATGACCACTAGGAGCATCTTTGGCGAGCCATTGGTTTCAAGGAAATCCATTGGGCACTAGGGGAATAAATCTTTGGGCACCTAGTGGTCATG

1 CATGACCACTAGGAGCATCTTTGGCGACCTGTAAGACGCGGATGCGCGTTAGTGGAGCTAGGGGAATAAATCTTTGGGCACCTAGTGGTCATG

1 CATGACCACTAGGAGCATCTTTGGCGAGACTGCCACGTGGTACAGGCCTCCGTGTTTCTAGGGGAATAAATCTTTGGGCACCTAGTGGTCATG

1 CATGACCACTAGGAGCATCTTTGGCGATAGACTCCCCAAGCGGGGCACACTGGGCTCCTAGGGGAATAAATCTTTGGGCACCTAGTGGTCATG

1 CATGACCACTAGGAGCATCTTTGGCGATGGGCGCTCTGGGTGAGACCTTCATTGGTCCTAGGGGAATAAATCTTTGGGCACCTAGTGGTCATG

1 CATGACCACTAGGAGCATCTTTGGCGACTTGGGGGGCTGGTCCCCCCTGATTATCGACTAGGGGAATAAATCTTTGGGCACCTAGTGGTCATG

1 CATGACCACTAGGAGCATCTTTGGCGACGATGGCGGGCTAGAGACCTCCGAAGTGGACTAGGGGAATAAATCTTTGGGCACCTAGTGGTCATG

1 CATGACCACTAGGAGCATCTTTGGCGAGATCGGGAGAATCAGTGGCATTGGTGTCTACTAGGGGAATAAATCTTTGGGCACCTAGTGGTCATG

1 CATGACCACTAGGAGCATCTTTGGCGAGGGAGGGCGCCGGCATCGGTGTGAATGCGACTAGGGGAATAAATCTTTGGGCACCTAGTGGTCATG

1 CATGACCACTAGGAGCATCTTTGGCGATATCGGGAGAATCGCTGGCATTGCTGACTCCTAGGGGAATAAATCTTTGGGCACCTAGTGGTCATG

1 CATGACCACTAGGAGCATCTTTGGCGAGATCGGGAGAATCGGTAGCATTGGTATCTCCTAGGGGAATAAATCTTTGGGCACCTAGTGGTCATG

1 CATGACCACTAGGAGCATCTTTGGCGAGGGTGGGTAGAACAGGGTGGGGTGCTGTCCCTAGGGGAATAAATCTTTGGGCACCTAGTGGTCATG

1 CATGACCACTAGGAGCATCTTTGGCGAGATCGGGAGAATCGGAGGCATTGGTGTCTTCTAGGGGAATAAATCTTTGGGCACCTAGTGGTCATG

1 CATGACCACTAGGAGCATCTTTGGCGAGAGTCGGTAGAACAGGGTGGGGTGCTGTCTCTAGGGGAATAAATCTTTGGGCACCTAGTGGTCATG

1 CATGACCACTAGGAGCATCTTTGGCGAGGGGTCATGGGAATGTGATTGCGCTTAAGACTAGGGGAATAAATCTTTGGGCACCTAGTGGTCATG

1 CATGACCACTAGGAGCATCTTTGGCGAGTGGGGGGCGACGGCCGGTAGCGGGTGAGACTAGGGGAATAAATCTTTGGGCACCTAGTGGTCATG

1 CATGACCACTAGGAGCATCTTTGGCGATCCGGTCTCGATCAGTTCTCGTGGGTCTCTCTAGGGGAATAAATCTTTGGGCACCTAGTGGTCATG

1 CATGACCACTAGGAGCATCTTTGGCGAGATCGGGAGAATAGGTGGCATTGGTGTCACCTAGGGGAATAAATCTTTGGGCACCTAGTGGTCATG

1 CATGACCACTAGGAGCATCTTTGGCGACTAAGTGGGCACCGCGTTAAGTGGGGTAGACTAGGGGAATAAATCTTTGGGCACCTAGTGGTCATG

1 CATGACCACTAGGAGCATCTTTGGCGATCGGGGTCCACAGGTTGAAATTAGATGCGCCTAGGGGAATAAATCTTTGGGCACCTAGTGGTCATG

1 CATGACCACTAGGAGCATCTTTGGCGACTAAGTGGTCAACGCGTTAAGTGGGGTACACTAGGGGAATAAATCTTTGGGCACCTAGTGGTCATG

1 CATGACCACTAGGAGCATCTTTGGCGACCAGATCTGGTAGGTAATGTGCTGAGGAGGCTAGGGGAATAAATCTTTGGGCACCTAGTGGTCATG

1 CATGACCACTAGGAGCATCTTTGGCGACCGGCAGGTGTTGCGGGGGTCTGTGAATCCCTAGGGGAATAAATCTTTGGGCACCTAGTGGTCATG

1 CATGACCACTAGGAGCATCTTTGGCGACGGGGAATCAAGGAGGGCTGGGGAGTGATGCTAGGGGAATAAATCTTTGGGCACCTAGTGGTCATG

1 CATGACCACTAGGAGCATCTTTGGCGACGAGGTAAAGTGCGTAGAGTGGTAAGTCGACTAGGGGAATAAATCTTTGGGCACCTAGTGGTCATG

1 CATGACCACTAGGAGCATCTTTGGCGAGAAGACTACGGATTTGGGGACCAGTTGCTGCTAGGGGAATAAATCTTTGGGCACCTAGTGGTCATG

1 CATGACCACTAGGAGCATCTTTGGCGAACGGCAGGTGTTGCGTTGGTCTGTGTATACCTAGGGGAATAAATCTTTGGGCACCTAGTGGTCATG

1 CATGACCACTAGGAGCATCTTTGGCGATGGGTTGTAATCGATTTGTGCTGGATCGAACTAGGGGAATAAATCTTTGGGCACCTAGTGGTCATG

1 CATGACCACTAGGAGCATCTTTGGCGAGAAAGGGAGAATCCGTGGCATTGGTGTATCCTAGGGGAATAAATCTTTGGGCACCTAGTGGTCATG

1 CATGACCACTAGGAGCATCTTTGGCGAGAAGACTCCGGATTCGGGGACAAGTTGCTGCTAGGGGAATAAATCTTTGGGCACCTAGTGGTCATG

1 CATGACCACTAGGAGCATCTTTGGCGATTGTGTTCTATACTCTTTCAATATGCTTCACTAGGGGAATAAATCTTTGGGCACCTAGTGGTCATG

1 CATGACCACTAGGAGCATCTTTGGCGATCCGGGGGTCCGCGACGGACGACGTTCGGGCTAGGGGAATAAATCTTTGGGCACCTAGTGGTCATG

1 CATGACCACTAGGAGCATCTTTGGCGAGATCGGGAGAATCGGTGGCCTTGGTGTCTTCTAGGGGAATAAATCTTTGGGCACCTAGTGGTCATG

1 CATGACCACTAGGAGCATCTTTGGCGATGGGTGCTGACGGCCGCCCCCGCGGCTACACTAGGGGAATAAATCTTTGGGCACCTAGTGGTCATG

1 CATGACCACTAGGAGCATCTTTGGCGAGGCTGGCCGCGTCCCGGTCGTCTCTCCTGCCTAGGGGAATAAATCTTTGGGCACCTAGTGGTCATG

1 CATGACCACTAGGAGCATCTTTGGCGATCCGTGGGCACCTATGTGCGACGCTGTGGGCTAGGGGAATAAATCTTTGGGCACCTAGTGGTCATG

1 CATGACCACTAGGAGCATCTTTGGCGACGAGTGCGGCAACGGTTATAAGTAAGTCGACTAGGGGAATAAATCTTTGGGCACCTAGTGGTCATG

1 CATGACCACTAGGAGCATCTTTGGCGAGCCTCGCTTGGGAGGTTCCTCCACCAGTTCCTAGGGGAATAAATCTTTGGGCACCTAGTGGTCATG

1 CATGACCACTAGGAGCATCTTTGGCGATCTGGGGGCCAAAGGCGACGCAAATTAGGGCTAGGGGAATAAATCTTTGGGCACCTAGTGGTCATG

1 CATGACCACTAGGAGCATCTTTGGCGACCGTCAAATTATCATATTGTGGACGAGCCTCTAGGGGAATAAATCTTTGGGCACCTAGTGGTCATG

1 CATGACCACTAGGAGCATCTTTGGCGATGATCGCTTAGCGAACCTACAGGGTGGTCCCTAGGGGAATAAATCTTTGGGCACCTAGTGGTCATG

1 CATGACCACTAGGAGCATCTTTGGCGAGATCGGGAGAATCGGTGGCATTGCTTACACCTAGGGGAATAAATCTTTGGGCACCTAGTGGTCATG

1 CATGACCACTAGGAGCATCTTTGGCGAATGGCCGGCACGGCCTTCTGGTCCTCGGTACTAGGGGAATAAATCTTTGGGCACCTAGTGGTCATG

1 CATGACCACTAGGAGCATCTTTGGCGATATCTGGAGAATCGGTGGCATTTGTGTCTCCTAGGGGAATAAATCTTTGGGCACCTAGTGGTCATG

1 CATGACCACTAGGAGCATCTTTGGCGAGATCTGGATAATCGGTGGCATAGGTGTCTCCTAGGGGAATAAATCTTTGGGCACCTAGTGGTCATG

1 CATGACCACTAGGAGCATCTTTGGCGAGAAGACTCTGGATTCTGGGACCAGTTGCTCCTAGGGGAATAAATCTTTGGGCACCTAGTGGTCATG

1 CATGACCACTAGGAGCATCTTTGGCGACATTGGTTAATTTGATCTCGTATGTTTGGGCTAGGGGAATAAATCTTTGGGCACCTAGTGGTCATG

1 CATGACCACTAGGAGCATCTTTGGCGATCGTCAAAGGTACGAGTGGTGGTAGAGCGACTAGGGGAATAAATCTTTGGGCACCTAGTGGTCATG

1 CATGACCACTAGGAGCATCTTTGGCGAGCAGATATGGTAGGTAATGTGCTGAGGAGGCTAGGGGAATAAATCTTTGGGCACCTAGTGGTCATG

1 CATGACCACTAGGAGCATCTTTGGCGACTACGGGCTAGAGTTACTCGTATTAATAAGCTAGGGGAATAAATCTTTGGGCACCTAGTGGTCATG

1 CATGACCACTAGGAGCATCTTTGGCGAGCTCGGGAGAATCGGTGGCATGGGTGTCTCCTAGGGGAATAAATCTTTGGGCACCTAGTGGTCATG

1 CATGACCACTAGGAGCATCTTTGGCGAATCGGCGGCAAGTCGTTGAAGGATGTCAGTCTAGGGGAATAAATCTTTGGGCACCTAGTGGTCATG

1 CATGACCACTAGGAGCATCTTTGGCGAGATTGGGAGAATCGGTTGCATTGGTGTCTCCTAGGGGAATAAATCTTTGGGCACCTAGTGGTCATG

1 CATGACCACTAGGAGCATCTTTGGCGAGATCGGGAGAATCGGTGGCATTTGTGTCACCTAGGGGAATAAATCTTTGGGCACCTAGTGGTCATG

1 CATGACCACTAGGAGCATCTTTGGCGAGATCGGGAGAATCGGACCCAATGGTGTCTCCTAGGGGAATAAATCTTTGGGCACCTAGTGGTCATG

1 CATGACCACTAGGAGCATCTTTGGCGAGCCTCGCTTGGGAGGTTGCTACACTAGTTCCTAGGGGAATAAATCTTTGGGCACCTAGTGGTCATG

1 CATGACCACTAGGAGCATCTTTGGCGAGGGAGGGAGCCGGCAGCGGTGTGAATGCGACTAGGGGAATAAATCTTTGGGCACCTAGTGGTCATG

1 CATGACCACTAGGAGCATCTTTGGCGATCAGGCCGCCAGGGCGTACAAAGTGTGGTTCTAGGGGAATAAATCTTTGGGCACCTAGTGGTCATG

1 CATGACCACTAGGAGCATCTTTGGCGAACGGAAGGAGTTGCGGTGGTCTGTGAATCCCTAGGGGAATAAATCTTTGGGCACCTAGTGGTCATG

1 CATGACCACTAGGAGCATCTTTGGCGAGGTGCGGTGGGCATGTGGGTGGTAGCGACGCTAGGGGAATAAATCTTTGGGCACCTAGTGGTCATG

1 CATGACCACTAGGAGCATCTTTGGCGAACTGTTTCCCCCCTCTGCACTATTGCCGTTCTAGGGGAATAAATCTTTGGGCACCTAGTGGTCATG

1 CATGACCACTAGGAGCATCTTTGGCGAGTGAGGGCGCCGGCAGCGGTGTGAATGCGCCTAGGGGAATAAATCTTTGGGCACCTAGTGGTCATG

1 CATGACCACTAGGAGCATCTTTGGCGAACGGCAGGTGTTGCCGTGGCCTGTGAATCCCTAGGGGAATAAATCTTTGGGCACCTAGTGGTCATG

1 CATGACCACTAGGAGCATCTTTGGCGACGAGATTTGGTAGGTAGATGTGCTCTATCACTAGGGGAATAAATCTTTGGGCACCTAGTGGTCATG

1 CATGACCACTAGGAGCATCTTTGGCGATAGTCGGTAGAACAGGGTGTGGTGCTGTCCCTAGGGGAATAAATCTTTGGGCACCTAGTGGTCATG

1 CATGACCACTAGGAGCATCTTTGGCGATTCCCTTGGCCGTCGCTCGCTCGGGCTGATCTAGGGGAATAAATCTTTGGGCACCTAGTGGTCATG

1 CATGACCACTAGGAGCATCTTTGGCGATTACTGACGACAGGTTGCGGGAACTGGGAGCTAGGGGAATAAATCTTTGGGCACCTAGTGGTCATG

1 CATGACCACTAGGAGCATCTTTGGCGAGATCGGGAGAATCGGTCGCATAGGTGTCTCCTAGGGGAATAAATCTTTGGGCACCTAGTGGTCATG

1 CATGACCACTAGGAGCATCTTTGGCGAAGTTCCTGACGGCCTTTCGTCATGGATTTTCTAGGGGAATAAATCTTTGGGCACCTAGTGGTCATG

1 CATGACCACTAGGAGCATCTTTGGCGATGGGTCCGTGCTATCTGCACACTCCGCGGGCTAGGGGAATAAATCTTTGGGCACCTAGTGGTCATG

1 CATGACCACTAGGAGCATCTTTGGCGAGATCGGGAGACTCGGTGGGATTGGTGTCTCCTAGGGGAATAAATCTTTGGGCACCTAGTGGTCATG

1 CATGACCACTAGGAGCATCTTTGGCGAAGCAGTGTAGTCTGATTGCTTTGGCATATTCTAGGGGAATAAATCTTTGGGCACCTAGTGGTCATG

1 CATGACCACTAGGAGCATCTTTGGCGACTATCGATGTGTTCGTCAGTGCTAGTGGAGCTAGGGGAATAAATCTTTGGGCACCTAGTGGTCATG

1 CATGACCACTAGGAGCATCTTTGGCGAGAGGGTGGGGGATGAATCACTGTCGCATTTCTAGGGGAATAAATCTTTGGGCACCTAGTGGTCATG

1 CATGACCACTAGGAGCATCTTTGGCGACGTCGGCTGGTAGAGATTGTATTGAGGAACCTAGGGGAATAAATCTTTGGGCACCTAGTGGTCATG

1 CATGACCACTAGGAGCATCTTTGGCGAAGCGTGTTGCAAGGTCCGCTAGTGTGGACCCTAGGGGAATAAATCTTTGGGCACCTAGTGGTCATG

1 CATGACCACTAGGAGCATCTTTGGCGAGATCGGGAGAATCGGAGGAATTGGAGTCTCCTAGGGGAATAAATCTTTGGGCACCTAGTGGTCATG

1 CATGACCACTAGGAGCATCTTTGGCGACCTGCAGGGGATGCGGTGGACTGTGGAAACCTAGGGGAATAAATCTTTGGGCACCTAGTGGTCATG

1 CATGACCACTAGGAGCATCTTTGGCGAACGTGCAGGAAATGCCGCCTGGAGCGTAGGCTAGGGGAATAAATCTTTGGGCACCTAGTGGTCATG

1 CATGACCACTAGGAGCATCTTTGGCGAGCCGGTTGTGCAACAATGTGTTCTGGGTAACTAGGGGAATAAATCTTTGGGCACCTAGTGGTCATG

1 CATGACCACTAGGAGCATCTTTGGCGAGCCCGGGAGAATCGGTGGCATTGGTGTCTCCTAGGGGAATAAATCTTTGGGCACCTAGTGGTCATG

1 CATGACCACTAGGAGCATCTTTGGCGAGTGGCTGAGGTGCGAGAGACACGGTCGAGACTAGGGGAATAAATCTTTGGGCACCTAGTGGTCATG

1 CATGACCACTAGGAGCATCTTTGGCGAGGGGCGCTCTGGGTGAGACCTTCATTGGTCCTAGGGGAATAAATCTTTGGGCACCTAGTGGTCATG

1 CATGACCACTAGGAGCATCTTTGGCGATCGCATAGCGCACGACAGGTTTGGAGGGGACTAGGGGAATAAATCTTTGGGCACCTAGTGGTCATG

1 CATGACCACTAGGAGCATCTTTGGCGATATCGGGAGAATCGGTCGCATTGGTGTCTCCTAGGGGAATAAATCTTTGGGCACCTAGTGGTCATG

1 CATGACCACTAGGAGCATCTTTGGCGACCACAGAGAGGTGAAGGTGAGAACAGTCGACTAGGGGAATAAATCTTTGGGCACCTAGTGGTCATG

1 CATGACCACTAGGAGCATCTTTGGCGATGAAGATGGTAGGGTAATGTGTCGGCGCCGCTAGGGGAATAAATCTTTGGGCACCTAGTGGTCATG

1 CATGACCACTAGGAGCATCTTTGGCGAGATCGGGAGAATCCGTGGCATTGGTGTCTTCTAGGGGAATAAATCTTTGGGCACCTAGTGGTCATG

1 CATGACCACTAGGAGCATCTTTGGCGATCCGGGGGCATGCTATGCGACGCTTACGGGCTAGGGGAATAAATCTTTGGGCACCTAGTGGTCATG

1 CATGACCACTAGGAGCATCTTTGGCGAGATCGGGAGGATCGGTGGCATTAGTGTCTCCTAGGGGAATAAATCTTTGGGCACCTAGTGGTCATG

1 CATGACCACTAGGAGCATCTTTGGCGAGATCGGGAGAATCGGTGGAATTGCTGTCTCCTAGGGGAATAAATCTTTGGGCACCTAGTGGTCATG

1 CATGACCACTAGGAGCATCTTTGGCGAAGCGCAGGGTGTAAGAGGGGCGTGTCCATGCTAGGGGAATAAATCTTTGGGCACCTAGTGGTCATG

1 CATGACCACTAGGAGCATCTTTGGCGAACGTCAGGTGTATCGGTGGTCTGTGAATCCCTAGGGGAATAAATCTTTGGGCACCTAGTGGTCATG

1 CATGACCACTAGGAGCATCTTTGGCGAGCTCGGGGGGATCCGTTGCAGTGTTGAAACCTAGGGGAATAAATCTTTGGGCACCTAGTGGTCATG

1 CATGACCACTAGGAGCATCTTTGGCGACTCGAGTGGTCAAGCGCGGCATTGGATCGCCTAGGGGAATAAATCTTTGGGCACCTAGTGGTCATG

1 CATGACCACTAGGAGCATCTTTGGCGAGATCGGGAGAATCGGTGGCATTGGTGTCATCTAGGGGAATAAATCTTTGGGCACCTAGTGGTCATG

1 CATGACCACTAGGAGCATCTTTGGCGACTCGGATCGGCGTGCCGTATGCGTAGTGGACTAGGGGAATAAATCTTTGGGCACCTAGTGGTCATG

1 CATGACCACTAGGAGCATCTTTGGCGAGAGGACTCTGTATTCGGGGACCAGTTGCTGCTAGGGGAATAAATCTTTGGGCACCTAGTGGTCATG

1 CATGACCACTAGGAGCATCTTTGGCGATCCGGGGGCACCTATGTTCGACGCTGTGGGCTAGGGGAATAAATCTTTGGGCACCTAGTGGTCATG

1 CATGACCACTAGGAGCATCTTTGGCGAGATCGGGAGAATCGGTGGCGTTGGTTTCTCCTAGGGGAATAAATCTTTGGGCACCTAGTGGTCATG

1 CATGACCACTAGGAGCATCTTTGGCGAACGGCAGGTGTTGTGGTGGTCTGTGTATCCCTAGGGGAATAAATCTTTGGGCACCTAGTGGTCATG

1 CATGACCACTAGGAGCATCTTTGGCGAGGGAGGGCGCCGGCAGCGGTGTGAGTGCGACTAGGGGAATAAATCTTTGGGCACCTAGTGGTCATG

1 CATGACCACTAGGAGCATCTTTGGCGAGAAGACTCTGGGTTCGGGGACCAGTCGCTGCTAGGGGAATAAATCTTTGGGCACCTAGTGGTCATG

1 CATGACCACTAGGAGCATCTTTGGCGACGTCGGCTTGTAGAGACTGTATTGAGGAACCTAGGGGAATAAATCTTTGGGCACCTAGTGGTCATG

1 CATGACCACTAGGAGCATCTTTGGCGAGCCGATTGTGCAAAAATGTGTTCTGGGTAACTAGGGGAATAAATCTTTGGGCACCTAGTGGTCATG

1 CATGACCACTAGGAGCATCTTTGGCGAGATCTGGAGAATCGGTGGCATTGCTGTCTCCTAGGGGAATAAATCTTTGGGCACCTAGTGGTCATG

1 CATGACCACTAGGAGCATCTTTGGCGAAGCGAACAGCATCAGCAAGTGGACTGTCTGCTAGGGGAATAAATCTTTGGGCACCTAGTGGTCATG

1 CATGACCACTAGGAGCATCTTTGGCGACGGTGTGGGGAACTTGATTCGGCGGTGCTACTAGGGGAATAAATCTTTGGGCACCTAGTGGTCATG

1 CATGACCACTAGGAGCATCTTTGGCGAGCGGCAGGTGTTGCGGTGGCCTGTGAATCCCTAGGGGAATAAATCTTTGGGCACCTAGTGGTCATG

1 CATGACCACTAGGAGCATCTTTGGCGAACGGCAGGTGTTGCGGTGGACTGTGAAACCCTAGGGGAATAAATCTTTGGGCACCTAGTGGTCATG

1 CATGACCACTAGGAGCATCTTTGGCGAACGGCAGGTGTTGCGGTGGTCTGAGTCCTACTAGGGGAATAAATCTTTGGGCACCTAGTGGTCATG

1 CATGACCACTAGGAGCATCTTTGGCGACCGTAGGTAAACAAAAGTGGCCTTGCTAGCCTAGGGGAATAAATCTTTGGGCACCTAGTGGTCATG

1 CATGACCACTAGGAGCATCTTTGGCGAGAAGACTATGGATTCGGGGACCAGTTGCTACTAGGGGAATAAATCTTTGGGCACCTAGTGGTCATG

1 CATGACCACTAGGAGCATCTTTGGCGAATTTCGCATCTCTCGCAGTAGTGGCCTCGTCTAGGGGAATAAATCTTTGGGCACCTAGTGGTCATG

1 CATGACCACTAGGAGCATCTTTGGCGATTTCGTGTATTGACTCTACGTCGTAGCCAGCTAGGGGAATAAATCTTTGGGCACCTAGTGGTCATG

1 CATGACCACTAGGAGCATCTTTGGCGAGGGAGTGGTCGGCCAGGTGGGGGGTCGGGGCTAGGGGAATAAATCTTTGGGCACCTAGTGGTCATG

1 CATGACCACTAGGAGCATCTTTGGCGAGCCTTGCTTGGGAGGTTGCTCCACCATTTCCTAGGGGAATAAATCTTTGGGCACCTAGTGGTCATG

1 CATGACCACTAGGAGCATCTTTGGCGACGGCGTGGGGCTATTGATGAGGCGGCACTCCTAGGGGAATAAATCTTTGGGCACCTAGTGGTCATG

1 CATGACCACTAGGAGCATCTTTGGCGAGAGCTTGAGAATCGGTGGCATTGGTGTCTCCTAGGGGAATAAATCTTTGGGCACCTAGTGGTCATG

1 CATGACCACTAGGAGCATCTTTGGCGAGATCGGGAGAATCGGAGGCCTTGGTGTCTCCTAGGGGAATAAATCTTTGGGCACCTAGTGGTCATG

1 CATGACCACTAGGAGCATCTTTGGCGAGAAGTCTCTGGATTCGGGGACCATTTGCTGCTAGGGGAATAAATCTTTGGGCACCTAGTGGTCATG

1 CATGACCACTAGGAGCATCTTTGGCGAAAGCTTGAAAAAGGTGCGTTGTTGTCGCATCTAGGGGAATAAATCTTTGGGCACCTAGTGGTCATG

1 CATGACCACTAGGAGCATCTTTGGCGAGTGGGGGGCGACGGCCGGGAGTGGGTGAGACTAGGGGAATAAATCTTTGGGCACCTAGTGGTCATG

1 CATGACCACTAGGAGCATCTTTGGCGACAGGGAATGCCGATGCGCTGGACGACCGAGCTAGGGGAATAAATCTTTGGGCACCTAGTGGTCATG

1 CATGACCACTAGGAGCATCTTTGGCGAACGGCAGAGCGCACGGCGATGCATTGGAACCTAGGGGAATAAATCTTTGGGCACCTAGTGGTCATG

1 CATGACCACTAGGAGCATCTTTGGCGAGATCGGGAGAATCTGTGTCATTGGTGTCTCCTAGGGGAATAAATCTTTGGGCACCTAGTGGTCATG

1 CATGACCACTAGGAGCATCTTTGGCGAAGTGCGGCGTGCCGAGTCTTCCTGCGACTCCTAGGGGAATAAATCTTTGGGCACCTAGTGGTCATG

1 CATGACCACTAGGAGCATCTTTGGCGAACGGAAGGTGTTTCGGTGGTCTGTGGATCCCTAGGGGAATAAATCTTTGGGCACCTAGTGGTCATG

1 CATGACCACTAGGAGCATCTTTGGCGAGATCGGGATAATCGTTGGCATCGGTGTCTCCTAGGGGAATAAATCTTTGGGCACCTAGTGGTCATG

1 CATGACCACTAGGAGCATCTTTGGCGATAGCGGACACTAAGGGTTCTTTAGTATGGGCTAGGGGAATAAATCTTTGGGCACCTAGTGGTCATG

1 CATGACCACTAGGAGCATCTTTGGCGAGATCGGGAGGATCGGTGGCATTGGTGTCTACTAGGGGAATAAATCTTTGGGCACCTAGTGGTCATG

1 CATGACCACTAGGAGCATCTTTGGCGACAAGATAGGCAACGGAGCAGTAAATGTCGACTAGGGGAATAAATCTTTGGGCACCTAGTGGTCATG

1 CATGACCACTAGGAGCATCTTTGGCGAACGGCTGGTGTTGCGGTGGTTTGTGAATCCCTAGGGGAATAAATCTTTGGGCACCTAGTGGTCATG

1 CATGACCACTAGGAGCATCTTTGGCGAGTCAGTGGGAATGGGAGACAGGCGACGAGACTAGGGGAATAAATCTTTGGGCACCTAGTGGTCATG

1 CATGACCACTAGGAGCATCTTTGGCGAACGGCAGGTGTTGCGGTGGTCTGTGGATGCCTAGGGGAATAAATCTTTGGGCACCTAGTGGTCATG

1 CATGACCACTAGGAGCATCTTTGGCGAGAAGACTTTGGATTCGGGGACCGGTTGCTGCTAGGGGAATAAATCTTTGGGCACCTAGTGGTCATG

1 CATGACCACTAGGAGCATCTTTGGCGAGATCGGGACAATCGGTGGCATTGGTGTCTACTAGGGGAATAAATCTTTGGGCACCTAGTGGTCATG

1 CATGACCACTAGGAGCATCTTTGGCGACGAGTGGAGCTGGAACGGCTCCAAAGTGGACTAGGGGAATAAATCTTTGGGCACCTAGTGGTCATG

1 CATGACCACTAGGAGCATCTTTGGCGAGAAGTGGCTGAGGGCGGTGGGTAGGTCGCGCTAGGGGAATAAATCTTTGGGCACCTAGTGGTCATG

1 CATGACCACTAGGAGCATCTTTGGCGATACAGGTGATCGGTGCTCGGGTGCCAGGCCCTAGGGGAATAAATCTTTGGGCACCTAGTGGTCATG

1 CATGACCACTAGGAGCATCTTTGGCGAGATGGGGAGAATCGCTGGCATTGGTGTCTCCTAGGGGAATAAATCTTTGGGCACCTAGTGGTCATG

1 CATGACCACTAGGAGCATCTTTGGCGATCCTTCCCGCTCGTGCGTGGTTGGGAGCATCTAGGGGAATAAATCTTTGGGCACCTAGTGGTCATG

1 CATGACCACTAGGAGCATCTTTGGCGAGATCGGGAGCATCGGTGGCATGGGTGTCTCCTAGGGGAATAAATCTTTGGGCACCTAGTGGTCATG

1 CATGACCACTAGGAGCATCTTTGGCGACCAGCGTAGCCATAGAGTTGGCGACCAGTGCTAGGGGAATAAATCTTTGGGCACCTAGTGGTCATG

1 CATGACCACTAGGAGCATCTTTGGCGAGATCGGGAGAATCGGTGGCATGGGTGTCTTCTAGGGGAATAAATCTTTGGGCACCTAGTGGTCATG

1 CATGACCACTAGGAGCATCTTTGGCGATGGGACCCCGTCCTCCAACGGGCAACGGTCCTAGGGGAATAAATCTTTGGGCACCTAGTGGTCATG

1 CATGACCACTAGGAGCATCTTTGGCGATCGCACAGCGAACAAGGAGCGACTTGGGGCCTAGGGGAATAAATCTTTGGGCACCTAGTGGTCATG

1 CATGACCACTAGGAGCATCTTTGGCGATTCGGGGGTCACAAGTGACGGCGCATAGGGCTAGGGGAATAAATCTTTGGGCACCTAGTGGTCATG

1 CATGACCACTAGGAGCATCTTTGGCGAGAAGACTTTGGATTCGGTGACCCGTTGCTGCTAGGGGAATAAATCTTTGGGCACCTAGTGGTCATG

1 CATGACCACTAGGAGCATCTTTGGCGATCTCGCCTCCGTCGAGTGTTTTGTTGAGTGCTAGGGGAATAAATCTTTGGGCACCTAGTGGTCATG

1 CATGACCACTAGGAGCATCTTTGGCGACTACTGATGGCCGGAGAGAGCTAACCGTCCCTAGGGGAATAAATCTTTGGGCACCTAGTGGTCATG

1 CATGACCACTAGGAGCATCTTTGGCGATTGGGGAGTCGCAGTATGCAGGCAGTGAGACTAGGGGAATAAATCTTTGGGCACCTAGTGGTCATG

1 CATGACCACTAGGAGCATCTTTGGCGAAAGTCGGTAGAAAAGGGTGGGGTGCTGTCTCTAGGGGAATAAATCTTTGGGCACCTAGTGGTCATG

1 CATGACCACTAGGAGCATCTTTGGCGAGATCGGGAGAATCGGTGGCATTGGCGACTCCTAGGGGAATAAATCTTTGGGCACCTAGTGGTCATG

1 CATGACCACTAGGAGCATCTTTGGCGATTGTATAGGGATCTCCTACGATAGTATGTGCTAGGGGAATAAATCTTTGGGCACCTAGTGGTCATG

1 CATGACCACTAGGAGCATCTTTGGCGAGATCGGGAGAATCGGCGGCATGGGTGTCTCCTAGGGGAATAAATCTTTGGGCACCTAGTGGTCATG

1 CATGACCACTAGGAGCATCTTTGGCGACCTTCTAGGAGTCCTATGAGCAAATATTACCTAGGGGAATAAATCTTTGGGCACCTAGTGGTCATG

1 CATGACCACTAGGAGCATCTTTGGCGAGAACGCGAGAATCGGTGGCATTGGTGTCTCCTAGGGGAATAAATCTTTGGGCACCTAGTGGTCATG

1 CATGACCACTAGGAGCATCTTTGGCGAAAGTCGGTAGTACAGGGTGGGGTGCTGACCCTAGGGGAATAAATCTTTGGGCACCTAGTGGTCATG

1 CATGACCACTAGGAGCATCTTTGGCGATCCGGGGGCCACTGAGGTGACGCTAATGGGCTAGGGGAATAAATCTTTGGGCACCTAGTGGTCATG

1 CATGACCACTAGGAGCATCTTTGGCGATGATTTTTAGCAAAGTTGTTCTTCAATCTGCTAGGGGAATAAATCTTTGGGCACCTAGTGGTCATG

1 CATGACCACTAGGAGCATCTTTGGCGAGACACATCGTGACCGTTTAAATTAGGCTCTCTAGGGGAATAAATCTTTGGGCACCTAGTGGTCATG

1 CATGACCACTAGGAGCATCTTTGGCGATCATGGGGCCTTTGATGGCGACTCAGTGGGCTAGGGGAATAAATCTTTGGGCACCTAGTGGTCATG

1 CATGACCACTAGGAGCATCTTTGGCGAGAGAGGGCGCCGGCAGCGGTGTGAATGCGCCTAGGGGAATAAATCTTTGGGCACCTAGTGGTCATG

1 CATGACCACTAGGAGCATCTTTGGCGAACGGCAGTTGTTGCGGTGGTCTGTGACTCCCTAGGGGAATAAATCTTTGGGCACCTAGTGGTCATG

1 CATGACCACTAGGAGCATCTTTGGCGAGATCGGGAGAATCGGTGGCATTGGTTTATCCTAGGGGAATAAATCTTTGGGCACCTAGTGGTCATG

1 CATGACCACTAGGAGCATCTTTGGCGACGACGTTGGTATGCTCTCGGGGATACGTGCCTAGGGGAATAAATCTTTGGGCACCTAGTGGTCATG

1 CATGACCACTAGGAGCATCTTTGGCGAGATCTGGAGAATCTGTTGCATTGGTGTCTCCTAGGGGAATAAATCTTTGGGCACCTAGTGGTCATG

1 CATGACCACTAGGAGCATCTTTGGCGAGATCGGGAGAATCGGTGGCATTGGTTCCTCCTAGGGGAATAAATCTTTGGGCACCTAGTGGTCATG

1 CATGACCACTAGGAGCATCTTTGGCGACTCCAGTTCATCTGTCGGGCAGTTTATGGACTAGGGGAATAAATCTTTGGGCACCTAGTGGTCATG

1 CATGACCACTAGGAGCATCTTTGGCGACTGTGGCTGGTAGGTTCGTACTGTAGGCGGCTAGGGGAATAAATCTTTGGGCACCTAGTGGTCATG

1 CATGACCACTAGGAGCATCTTTGGCGACGAAAGTAGGGTGGAGCTATTTGATGTGATCTAGGGGAATAAATCTTTGGGCACCTAGTGGTCATG

1 CATGACCACTAGGAGCATCTTTGGCGAATGACTCTTTGTCCTATAAGATATGAAAGACTAGGGGAATAAATCTTTGGGCACCTAGTGGTCATG

1 CATGACCACTAGGAGCATCTTTGGCGAGGGTTTGTTTTAACTGGGTGCATTCCTCACCTAGGGGAATAAATCTTTGGGCACCTAGTGGTCATG

1 CATGACCACTAGGAGCATCTTTGGCGATAGCGGGTGGCGGTTGGTCGTTTTCCGATACTAGGGGAATAAATCTTTGGGCACCTAGTGGTCATG

1 CATGACCACTAGGAGCATCTTTGGCGAGCAGTCAGACATTTTTCGTTGACGGGAGTACTAGGGGAATAAATCTTTGGGCACCTAGTGGTCATG

1 CATGACCACTAGGAGCATCTTTGGCGAACGGCAGGTGCTGCGGTGGTCTGTGAATCTCTAGGGGAATAAATCTTTGGGCACCTAGTGGTCATG

1 CATGACCACTAGGAGCATCTTTGGCGACATTGGAGCGACGGTGTGGTCTGAGTCCTACTAGGGGAATAAATCTTTGGGCACCTAGTGGTCATG

1 CATGACCACTAGGAGCATCTTTGGCGAGAAGACCCTGGATTCGGGGACAAGTTGCTGCTAGGGGAATAAATCTTTGGGCACCTAGTGGTCATG

1 CATGACCACTAGGAGCATCTTTGGCGATAGGGGAATCAGAGGTGTGGATGGCAGAGGCTAGGGGAATAAATCTTTGGGCACCTAGTGGTCATG

1 CATGACCACTAGGAGCATCTTTGGCGAGCGTAGAGGACGCAGCAGCGTATCCGTCTACTAGGGGAATAAATCTTTGGGCACCTAGTGGTCATG

1 CATGACCACTAGGAGCATCTTTGGCGAGCCCTAAGGGTCGTGTCTGTAGTGAGCAGCCTAGGGGAATAAATCTTTGGGCACCTAGTGGTCATG

1 CATGACCACTAGGAGCATCTTTGGCGATATCGGGAGAATCGGTGGCATTTTAGTATCCTAGGGGAATAAATCTTTGGGCACCTAGTGGTCATG

1 CATGACCACTAGGAGCATCTTTGGCGAGAAGACTCCGGATTCGAGGACCAGTTGCTGCTAGGGGAATAAATCTTTGGGCACCTAGTGGTCATG

1 CATGACCACTAGGAGCATCTTTGGCGACCCACACTGGGTGGACGTGATCGCATAGCTCTAGGGGAATAAATCTTTGGGCACCTAGTGGTCATG

1 CATGACCACTAGGAGCATCTTTGGCGAGATCGGGAGAATCGTTGGCATTGATGTCTCCTAGGGGAATAAATCTTTGGGCACCTAGTGGTCATG

1 CATGACCACTAGGAGCATCTTTGGCGAGATCGGGAGAATCGTAGGGTGAATAGTCGACTAGGGGAATAAATCTTTGGGCACCTAGTGGTCATG

1 CATGACCACTAGGAGCATCTTTGGCGAGATCGGGAGAATCGGTGGAAGTGGTGTCTCCTAGGGGAATAAATCTTTGGGCACCTAGTGGTCATG

1 CATGACCACTAGGAGCATCTTTGGCGAGAGGTAGGGGGCCGCTCGCGCCTGCGTGCGCTAGGGGAATAAATCTTTGGGCACCTAGTGGTCATG

1 CATGACCACTAGGAGCATCTTTGGCGAGGGAGTGGTCGGCTATGTGGGGGGTCGGGGCTAGGGGAATAAATCTTTGGGCACCTAGTGGTCATG

1 CATGACCACTAGGAGCATCTTTGGCGAGAACGGAAGAATCGGTGGCATTGGTGTCTCCTAGGGGAATAAATCTTTGGGCACCTAGTGGTCATG

1 CATGACCACTAGGAGCATCTTTGGCGATCCGAACAGCTTCTTCTGCGAGTTCGGGTCCTAGGGGAATAAATCTTTGGGCACCTAGTGGTCATG

1 CATGACCACTAGGAGCATCTTTGGCGATCCGACGAAGTGGCAGAGGCAGAATATCGACTAGGGGAATAAATCTTTGGGCACCTAGTGGTCATG

1 CATGACCACTAGGAGCATCTTTGGCGATTTGGGAGTCGCATTATGCAGGCAGTGAGGCTAGGGGAATAAATCTTTGGGCACCTAGTGGTCATG

1 CATGACCACTAGGAGCATCTTTGGCGAGATCGGGAGAATCGGTGGCAATGGTGTTTCCTAGGGGAATAAATCTTTGGGCACCTAGTGGTCATG

1 CATGACCACTAGGAGCATCTTTGGCGACGGCGTGGGGCCATTTGTGCGGCGGATCCTCTAGGGGAATAAATCTTTGGGCACCTAGTGGTCATG

1 CATGACCACTAGGAGCATCTTTGGCGATTTGGGAGTCGCATTATGCAGGCAGTGAAGCTAGGGGAATAAATCTTTGGGCACCTAGTGGTCATG

1 CATGACCACTAGGAGCATCTTTGGCGATGAATACTGCACCAGTGTTCGTGCTATCGACTAGGGGAATAAATCTTTGGGCACCTAGTGGTCATG

1 CATGACCACTAGGAGCATCTTTGGCGACCCATTGTACGGCGAAGCGATATGGGTGGACTAGGGGAATAAATCTTTGGGCACCTAGTGGTCATG

1 CATGACCACTAGGAGCATCTTTGGCGAAGCAACAGGAACTGGACCCTTAGACTTGAACTAGGGGAATAAATCTTTGGGCACCTAGTGGTCATG

1 CATGACCACTAGGAGCATCTTTGGCGAGAAGACTATGGATTCGGGGACCAGTTGCTCCTAGGGGAATAAATCTTTGGGCACCTAGTGGTCATG

1 CATGACCACTAGGAGCATCTTTGGCGAAAGTCGGTAGTACAGGGTGGGGTGCTGTCCCTAGGGGAATAAATCTTTGGGCACCTAGTGGTCATG

1 CATGACCACTAGGAGCATCTTTGGCGAGTTCGGGAGCATCGGTGGCATTGGTGTCTCCTAGGGGAATAAATCTTTGGGCACCTAGTGGTCATG

1 CATGACCACTAGGAGCATCTTTGGCGAGATCGGAAGACTCGGTGGCATTGGTGTCTCCTAGGGGAATAAATCTTTGGGCACCTAGTGGTCATG

1 CATGACCACTAGGAGCATCTTTGGCGATTGGGGAGTCGCAGTATGCAGGCAGTGAGGCTAGGGGAATAAATCTTTGGGCACCTAGTGGTCATG

1 CATGACCACTAGGAGCATCTTTGGCGATCCGGGGGGCAGAGAGTCCGACGCACTGGGCTAGGGGAATAAATCTTTGGGCACCTAGTGGTCATG

1 CATGACCACTAGGAGCATCTTTGGCGATGGGTGCATACGAGTGCGCTAGCAATTACACTAGGGGAATAAATCTTTGGGCACCTAGTGGTCATG

1 CATGACCACTAGGAGCATCTTTGGCGAAGGGTAGGGGGCCGCTCGCGCCTGCGTGCGCTAGGGGAATAAATCTTTGGGCACCTAGTGGTCATG

1 CATGACCACTAGGAGCATCTTTGGCGAGAGTCGGTAGAACAGGGTGGGGTGTTGTCCCTAGGGGAATAAATCTTTGGGCACCTAGTGGTCATG

1 CATGACCACTAGGAGCATCTTTGGCGAGCCGATTGTTCAACAATGTGTTCTGGGTAACTAGGGGAATAAATCTTTGGGCACCTAGTGGTCATG

1 CATGACCACTAGGAGCATCTTTGGCGATGAACACTTTAGTGGCGTCCCGGTGTTGCTCTAGGGGAATAAATCTTTGGGCACCTAGTGGTCATG

1 CATGACCACTAGGAGCATCTTTGGCGAGGGGTCATGGGAATGAAATTGCGCTTAAGACTAGGGGAATAAATCTTTGGGCACCTAGTGGTCATG

1 CATGACCACTAGGAGCATCTTTGGCGAACGGCAGGCGTTGCGGTGGTCTGTGAATCTCTAGGGGAATAAATCTTTGGGCACCTAGTGGTCATG

1 CATGACCACTAGGAGCATCTTTGGCGAAGCAGTGCGACGGGCTATGGGTGCCTCGAGCTAGGGGAATAAATCTTTGGGCACCTAGTGGTCATG

1 CATGACCACTAGGAGCATCTTTGGCGAGATCGGTAGTATCGGTGGCATAGGTGTCTCCTAGGGGAATAAATCTTTGGGCACCTAGTGGTCATG

1 CATGACCACTAGGAGCATCTTTGGCGACTAAGTGGTCCACGCGTTAAATGGGGTAGACTAGGGGAATAAATCTTTGGGCACCTAGTGGTCATG

1 CATGACCACTAGGAGCATCTTTGGCGAGATCGGGGTAATCGGTGGCATTGGTGTCTCCTAGGGGAATAAATCTTTGGGCACCTAGTGGTCATG

1 CATGACCACTAGGAGCATCTTTGGCGATCGCTTAGCAGGTCGGAACGTGGCTCGGGCCTAGGGGAATAAATCTTTGGGCACCTAGTGGTCATG

1 CATGACCACTAGGAGCATCTTTGGCGAGAATACTCTGGAATCGGGGACCAGTTGCTGCTAGGGGAATAAATCTTTGGGCACCTAGTGGTCATG

1 CATGACCACTAGGAGCATCTTTGGCGAACTGCAGGTGTTGAGGTGGTCTGTGAATCCCTAGGGGAATAAATCTTTGGGCACCTAGTGGTCATG

1 CATGACCACTAGGAGCATCTTTGGCGAGATCGGGATAATCGTTTGCATTGTTGTCTCCTAGGGGAATAAATCTTTGGGCACCTAGTGGTCATG

1 CATGACCACTAGGAGCATCTTTGGCGACTCGTGACGGTGCCGAGAGCACCCCGGTGACTAGGGGAATAAATCTTTGGGCACCTAGTGGTCATG

1 CATGACCACTAGGAGCATCTTTGGCGACAGGGGATGCCGATGCGCTGGACGATTGAGCTAGGGGAATAAATCTTTGGGCACCTAGTGGTCATG

1 CATGACCACTAGGAGCATCTTTGGCGAGCCTTGCTTGGGAGGTTGCTTTACCAGTTCCTAGGGGAATAAATCTTTGGGCACCTAGTGGTCATG

1 CATGACCACTAGGAGCATCTTTGGCGATTAAGTGGTCCACGCGTTAAGTGGGGTAGACTAGGGGAATAAATCTTTGGGCACCTAGTGGTCATG

1 CATGACCACTAGGAGCATCTTTGGCGACAAGACTCTGGATTCGGGGACCAGTTGCTGCTAGGGGAATAAATCTTTGGGCACCTAGTGGTCATG

1 CATGACCACTAGGAGCATCTTTGGCGACCGTAGGAAAACAAAAGTGGGCTTGCTAGCCTAGGGGAATAAATCTTTGGGCACCTAGTGGTCATG

1 CATGACCACTAGGAGCATCTTTGGCGAACGTGCAAGATATGCTGCCTGGAGCGTAGGCTAGGGGAATAAATCTTTGGGCACCTAGTGGTCATG

1 CATGACCACTAGGAGCATCTTTGGCGACCAGATACGGTAGGTAATGTGCTGAGGAGGCTAGGGGAATAAATCTTTGGGCACCTAGTGGTCATG

1 CATGACCACTAGGAGCATCTTTGGCGAGGGTGGGCGCTGGCAGCGGTGTGAATGCGCCTAGGGGAATAAATCTTTGGGCACCTAGTGGTCATG

1 CATGACCACTAGGAGCATCTTTGGCGACCCGGCGAAGACGCGGCCTGGTGTGGGGGCCTAGGGGAATAAATCTTTGGGCACCTAGTGGTCATG

1 CATGACCACTAGGAGCATCTTTGGCGAGCCTTGCTTGGGAGGATGCTCCACCAGTTCCTAGGGGAATAAATCTTTGGGCACCTAGTGGTCATG

1 CATGACCACTAGGAGCATCTTTGGCGAGGAGACTTTGGATTCGGGGACCAGTTGCTGCTAGGGGAATAAATCTTTGGGCACCTAGTGGTCATG

1 CATGACCACTAGGAGCATCTTTGGCGATCCGGGGGCACCTATGTGCGACGCTGAGGGCTAGGGGAATAAATCTTTGGGCACCTAGTGGTCATG

1 CATGACCACTAGGAGCATCTTTGGCGATAGACAAGGGGTAGCCCGTAGTGGGTTGTTCTAGGGGAATAAATCTTTGGGCACCTAGTGGTCATG

1 CATGACCACTAGGAGCATCTTTGGCGAGATTAGGAGAATCGGTGGCATTGGAGTCTCCTAGGGGAATAAATCTTTGGGCACCTAGTGGTCATG

1 CATGACCACTAGGAGCATCTTTGGCGAGATCGGCAGAATAGGTGGCATTGGTGTCTCCTAGGGGAATAAATCTTTGGGCACCTAGTGGTCATG

1 CATGACCACTAGGAGCATCTTTGGCGACAAGGGACCCCGGCCCGAGACATAGTGCTGCTAGGGGAATAAATCTTTGGGCACCTAGTGGTCATG

1 CATGACCACTAGGAGCATCTTTGGCGACCGAGTGGGTTGGAAGGAAGTATTTCGGAGCTAGGGGAATAAATCTTTGGGCACCTAGTGGTCATG

1 CATGACCACTAGGAGCATCTTTGGCGAGATCTGGAGAATCGGTGGCATAGGTGACTCCTAGGGGAATAAATCTTTGGGCACCTAGTGGTCATG

1 CATGACCACTAGGAGCATCTTTGGCGATGGGCTGGAAGGAGCGCCGTGGTACTTCGACTAGGGGAATAAATCTTTGGGCACCTAGTGGTCATG

1 CATGACCACTAGGAGCATCTTTGGCGATAGTCGGTAGAACGGGGTGGGGTGCTGTCCCTAGGGGAATAAATCTTTGGGCACCTAGTGGTCATG

1 CATGACCACTAGGAGCATCTTTGGCGATCCAATGGGATCTTAACCCGGGTCGAGGGACTAGGGGAATAAATCTTTGGGCACCTAGTGGTCATG

1 CATGACCACTAGGAGCATCTTTGGCGAATCGTCGTGAGAAGCCTCGCGTGAACAGGTCTAGGGGAATAAATCTTTGGGCACCTAGTGGTCATG

1 CATGACCACTAGGAGCATCTTTGGCGAGACACAATGTATCTTCGAGGAACGGGAAGGCTAGGGGAATAAATCTTTGGGCACCTAGTGGTCATG

1 CATGACCACTAGGAGCATCTTTGGCGACTCTCAATGCCTTGATATCAGCGAACTCGTCTAGGGGAATAAATCTTTGGGCACCTAGTGGTCATG

1 CATGACCACTAGGAGCATCTTTGGCGACCGTAGGAAAACAAAAGTAGCCTTGCTAGCCTAGGGGAATAAATCTTTGGGCACCTAGTGGTCATG

1 CATGACCACTAGGAGCATCTTTGGCGAGTTCGGGAGAATCGGTGGCAATGGTGTCTCCTAGGGGAATAAATCTTTGGGCACCTAGTGGTCATG

1 CATGACCACTAGGAGCATCTTTGGCGATCCGGGGGCATGCCATGCGACGCTTATGGGCTAGGGGAATAAATCTTTGGGCACCTAGTGGTCATG

1 CATGACCACTAGGAGCATCTTTGGCGAGGATGATCCGATTGCTAGAGTGGTCGAGTACTAGGGGAATAAATCTTTGGGCACCTAGTGGTCATG

1 CATGACCACTAGGAGCATCTTTGGCGATAGCGAAGTGCTGTGGACAGTGGTCAGCACCTAGGGGAATAAATCTTTGGGCACCTAGTGGTCATG

1 CATGACCACTAGGAGCATCTTTGGCGAGTGCTAAAGTGTCGGGAGTGGTCGAGCCTCCTAGGGGAATAAATCTTTGGGCACCTAGTGGTCATG

1 CATGACCACTAGGAGCATCTTTGGCGACGGAGTGGGGCTCATTATGCGGCGGTAATCCTAGGGGAATAAATCTTTGGGCACCTAGTGGTCATG

1 CATGACCACTAGGAGCATCTTTGGCGATAGTCAGGTGTTGCGGTGGTCTGTGAATCCCTAGGGGAATAAATCTTTGGGCACCTAGTGGTCATG

1 CATGACCACTAGGAGCATCTTTGGCGATGGGTAGTATACGTGTGGCCGAGTATTCGACTAGGGGAATAAATCTTTGGGCACCTAGTGGTCATG

1 CATGACCACTAGGAGCATCTTTGGCGAGGCATCACGCCCTCTTTTGTTGTGGTTGAGCTAGGGGAATAAATCTTTGGGCACCTAGTGGTCATG

1 CATGACCACTAGGAGCATCTTTGGCGAATGGCCGGCACGGCCTTCTAGTCCCCGGTACTAGGGGAATAAATCTTTGGGCACCTAGTGGTCATG

1 CATGACCACTAGGAGCATCTTTGGCGACGTGACGAGTGCGTGTAGTGGTCTGTTCGTCTAGGGGAATAAATCTTTGGGCACCTAGTGGTCATG

1 CATGACCACTAGGAGCATCTTTGGCGATCCGGGGGCACAGCTGTGCGACTCTACGGGCTAGGGGAATAAATCTTTGGGCACCTAGTGGTCATG

1 CATGACCACTAGGAGCATCTTTGGCGATAGTCGGTAGAACAGGGTGGGATGCTGTCCCTAGGGGAATAAATCTTTGGGCACCTAGTGGTCATG

1 CATGACCACTAGGAGCATCTTTGGCGAGAAGACTATGGATTCGGGGACCAGTTACTGCTAGGGGAATAAATCTTTGGGCACCTAGTGGTCATG

1 CATGACCACTAGGAGCATCTTTGGCGAAATCGGGAGAATCGGTGGCATTGGTATCTCCTAGGGGAATAAATCTTTGGGCACCTAGTGGTCATG

1 CATGACCACTAGGAGCATCTTTGGCGAACACCACTAAGGGTTTCCAAAGGGACATGACTAGGGGAATAAATCTTTGGGCACCTAGTGGTCATG

1 CATGACCACTAGGAGCATCTTTGGCGATGATGGTGGGCTAGAGACCTCCGAAGTGGACTAGGGGAATAAATCTTTGGGCACCTAGTGGTCATG

1 CATGACCACTAGGAGCATCTTTGGCGAACTGCGCAGAGCGAGTGGCTGAGGTTACGCCTAGGGGAATAAATCTTTGGGCACCTAGTGGTCATG

1 CATGACCACTAGGAGCATCTTTGGCGAGTGGGGGGCGACGGCCGGGAGGGGGTGAGACTAGGGGAATAAATCTTTGGGCACCTAGTGGTCATG

1 CATGACCACTAGGAGCATCTTTGGCGAGATCGGGAGAATAGGTGGCATAGGTGTCTCCTAGGGGAATAAATCTTTGGGCACCTAGTGGTCATG

1 CATGACCACTAGGAGCATCTTTGGCGACGGTGTGGGGCTCCATGATGCGGCGGTCTCCTAGGGGAATAAATCTTTGGGCACCTAGTGGTCATG

1 CATGACCACTAGGAGCATCTTTGGCGACGTCGAAACAAAGGTGGGCATGGAAGGAGACTAGGGGAATAAATCTTTGGGCACCTAGTGGTCATG

1 CATGACCACTAGGAGCATCTTTGGCGATCAGGCCGCCAGGGCGAACGAAGTGTGGTTCTAGGGGAATAAATCTTTGGGCACCTAGTGGTCATG

1 CATGACCACTAGGAGCATCTTTGGCGAGATCGGGAGAATAGGAGGCATTGGTGTCTCCTAGGGGAATAAATCTTTGGGCACCTAGTGGTCATG

1 CATGACCACTAGGAGCATCTTTGGCGAGCTTCCGTCCCGTCTCTTGTAGGGATCCCCCTAGGGGAATAAATCTTTGGGCACCTAGTGGTCATG

1 CATGACCACTAGGAGCATCTTTGGCGACTCGTATCATTCTTTTTAAGGTCACTACTTCTAGGGGAATAAATCTTTGGGCACCTAGTGGTCATG

1 CATGACCACTAGGAGCATCTTTGGCGACTAAGTGGTCAGCGCGTTAAGTGGGGTAGACTAGGGGAATAAATCTTTGGGCACCTAGTGGTCATG

1 CATGACCACTAGGAGCATCTTTGGCGAGAGCCGAAGGGACCAGAAGGGAGTCTCTGTCTAGGGGAATAAATCTTTGGGCACCTAGTGGTCATG

1 CATGACCACTAGGAGCATCTTTGGCGAGATCGGGAGAAACCGTGGCATTGGTGTCTCCTAGGGGAATAAATCTTTGGGCACCTAGTGGTCATG

1 CATGACCACTAGGAGCATCTTTGGCGAGATCGGGAGAATCGTTGGCATTGGTTTCTCCTAGGGGAATAAATCTTTGGGCACCTAGTGGTCATG

1 CATGACCACTAGGAGCATCTTTGGCGAGATCATATGCCTGCAAGGATGTTAGTGGAGCTAGGGGAATAAATCTTTGGGCACCTAGTGGTCATG

1 CATGACCACTAGGAGCATCTTTGGCGATCGGTAGGAGAGTGCAACGGCGTGGTGAGACTAGGGGAATAAATCTTTGGGCACCTAGTGGTCATG

1 CATGACCACTAGGAGCATCTTTGGCGAAGCGTGTTGCAAGGTTCGTGAGTGTGGACCCTAGGGGAATAAATCTTTGGGCACCTAGTGGTCATG

1 CATGACCACTAGGAGCATCTTTGGCGATAGCGAAGGGCTGTGAACAGTGGTTAGCACCTAGGGGAATAAATCTTTGGGCACCTAGTGGTCATG

1 CATGACCACTAGGAGCATCTTTGGCGAGATCGGGAGAATAGGTGGCATTGGTGTCTTCTAGGGGAATAAATCTTTGGGCACCTAGTGGTCATG

1 CATGACCACTAGGAGCATCTTTGGCGATCAGGGGGTCTTTGATGGCGACTCAGTGGGCTAGGGGAATAAATCTTTGGGCACCTAGTGGTCATG

1 CATGACCACTAGGAGCATCTTTGGCGACGGTGTGGGGCCATCAGTGCGGCGGATCCCCTAGGGGAATAAATCTTTGGGCACCTAGTGGTCATG

1 CATGACCACTAGGAGCATCTTTGGCGACAAGGTGATACCTATCATCGCGACGTAGTGCTAGGGGAATAAATCTTTGGGCACCTAGTGGTCATG

1 CATGACCACTAGGAGCATCTTTGGCGACGGCGAAAGTTCGGGCTGGGGAGTGCTGCTCTAGGGGAATAAATCTTTGGGCACCTAGTGGTCATG

1 CATGACCACTAGGAGCATCTTTGGCGAGATCGCGAGAATAGGTGGCATTGGTGACTCCTAGGGGAATAAATCTTTGGGCACCTAGTGGTCATG

1 CATGACCACTAGGAGCATCTTTGGCGAGATCGGGAGAAACCGTGGCAGTGGTGTCTCCTAGGGGAATAAATCTTTGGGCACCTAGTGGTCATG

1 CATGACCACTAGGAGCATCTTTGGCGAACTGGAAAGGGGGTTAATATGGATCGTAGACTAGGGGAATAAATCTTTGGGCACCTAGTGGTCATG

1 CATGACCACTAGGAGCATCTTTGGCGATTGGGATCGAGGGAACGCTAGTTTTCCCTACTAGGGGAATAAATCTTTGGGCACCTAGTGGTCATG

1 CATGACCACTAGGAGCATCTTTGGCGATTCGCATAGCGGAGTCGTGAGACCGGGCTTCTAGGGGAATAAATCTTTGGGCACCTAGTGGTCATG

1 CATGACCACTAGGAGCATCTTTGGCGAGATCGGGAGAATCGTTGGCATTGGTGTATCCTAGGGGAATAAATCTTTGGGCACCTAGTGGTCATG

1 CATGACCACTAGGAGCATCTTTGGCGAGATCGGGAGAATCGGTGGTATTGGTGTTTCCTAGGGGAATAAATCTTTGGGCACCTAGTGGTCATG

1 CATGACCACTAGGAGCATCTTTGGCGACGGGATCGTGTCGGCGGATAGCGTACTTGTCTAGGGGAATAAATCTTTGGGCACCTAGTGGTCATG

1 CATGACCACTAGGAGCATCTTTGGCGAGAAGACTCTGGATTCGGGGATTAGTTGTTGCTAGGGGAATAAATCTTTGGGCACCTAGTGGTCATG

1 CATGACCACTAGGAGCATCTTTGGCGAAGTGCGGTGGGCCTGTGGGTGGTAGCGACGCTAGGGGAATAAATCTTTGGGCACCTAGTGGTCATG

1 CATGACCACTAGGAGCATCTTTGGCGATTGGGACTAGGGAAGTGCCCCAGGTGATTACTAGGGGAATAAATCTTTGGGCACCTAGTGGTCATG

1 CATGACCACTAGGAGCATCTTTGGCGATGGGGCTGTGCTATCTGCACACTCCGCGGGCTAGGGGAATAAATCTTTGGGCACCTAGTGGTCATG

1 CATGACCACTAGGAGCATCTTTGGCGAGTAGACTCTGGATTCGGGGACCAGTTGCTCCTAGGGGAATAAATCTTTGGGCACCTAGTGGTCATG

1 CATGACCACTAGGAGCATCTTTGGCGATTGATTTCTTCCAGATATAACTACCGGAAGCTAGGGGAATAAATCTTTGGGCACCTAGTGGTCATG

1 CATGACCACTAGGAGCATCTTTGGCGAGGTCGCGTTAGCGATTGCGGCGGGCCTCGACTAGGGGAATAAATCTTTGGGCACCTAGTGGTCATG

1 CATGACCACTAGGAGCATCTTTGGCGAGAAGACTTTGGATCCGGGGACCAGTTGCTGCTAGGGGAATAAATCTTTGGGCACCTAGTGGTCATG

1 CATGACCACTAGGAGCATCTTTGGCGAGAACACTATAGAGTGTGGGACACGTTGCTCCTAGGGGAATAAATCTTTGGGCACCTAGTGGTCATG

1 CATGACCACTAGGAGCATCTTTGGCGACGTGGGAGTGGGAAAGGTGGTGGTGTGGAGCTAGGGGAATAAATCTTTGGGCACCTAGTGGTCATG

1 CATGACCACTAGGAGCATCTTTGGCGATGGAAATGGACGGTACGTAACGGTGATAACCTAGGGGAATAAATCTTTGGGCACCTAGTGGTCATG

1 CATGACCACTAGGAGCATCTTTGGCGACGGCGTGGGGTTATCTGTGCGGCGGATCCCCTAGGGGAATAAATCTTTGGGCACCTAGTGGTCATG

1 CATGACCACTAGGAGCATCTTTGGCGACTAGCGAATAACTTACAGTTGACCGGTGCACTAGGGGAATAAATCTTTGGGCACCTAGTGGTCATG

1 CATGACCACTAGGAGCATCTTTGGCGAGATCGGGAGAATCGGTGTCCTGGGTGTCTCCTAGGGGAATAAATCTTTGGGCACCTAGTGGTCATG

1 CATGACCACTAGGAGCATCTTTGGCGACGTTAGTGGTGTGGTTGGTACATCTGTCGACTAGGGGAATAAATCTTTGGGCACCTAGTGGTCATG

1 CATGACCACTAGGAGCATCTTTGGCGACGGCGTGGGGCAATTGATGCGGCGGCACCCCTAGGGGAATAAATCTTTGGGCACCTAGTGGTCATG

1 CATGACCACTAGGAGCATCTTTGGCGATCCGGGGGTCCATGCGGACGACGTTTAGGGCTAGGGGAATAAATCTTTGGGCACCTAGTGGTCATG

1 CATGACCACTAGGAGCATCTTTGGCGACCAGATAGGGTAGGTAATGTGCTGAGGAGGCTAGGGGAATAAATCTTTGGGCACCTAGTGGTCATG

1 CATGACCACTAGGAGCATCTTTGGCGACATCAGCTCCGCGGTCGGGGACATAAGGGACTAGGGGAATAAATCTTTGGGCACCTAGTGGTCATG

1 CATGACCACTAGGAGCATCTTTGGCGAGATCGGGAGAATCGGTGGCAATGTTGTCTCCTAGGGGAATAAATCTTTGGGCACCTAGTGGTCATG

1 CATGACCACTAGGAGCATCTTTGGCGAACGGCAGGTGGTGCGGGGGTCTGTGAATCCCTAGGGGAATAAATCTTTGGGCACCTAGTGGTCATG

1 CATGACCACTAGGAGCATCTTTGGCGACTCGGGTTAACTGCGTTAACCGATGTGGAGCTAGGGGAATAAATCTTTGGGCACCTAGTGGTCATG

1 CATGACCACTAGGAGCATCTTTGGCGAGATCGGGGGAATCGGTGTCATTGGTGTCTCCTAGGGGAATAAATCTTTGGGCACCTAGTGGTCATG

1 CATGACCACTAGGAGCATCTTTGGCGAAAGCACCTGTCTTTGGCCGGCCTCCGTGCCCTAGGGGAATAAATCTTTGGGCACCTAGTGGTCATG

1 CATGACCACTAGGAGCATCTTTGGCGAGATCGGGAGAATCGCTGGCATTGGTGTCTACTAGGGGAATAAATCTTTGGGCACCTAGTGGTCATG

1 CATGACCACTAGGAGCATCTTTGGCGATTATGGCTATTTGAAGTGTTGTGTGCTGCTCTAGGGGAATAAATCTTTGGGCACCTAGTGGTCATG

1 CATGACCACTAGGAGCATCTTTGGCGAACGGCAGGTGTTGCGGTGGTCTGCCAATCCCTAGGGGAATAAATCTTTGGGCACCTAGTGGTCATG

1 CATGACCACTAGGAGCATCTTTGGCGATATCGGGATAAACGGTGGCATTGGTGTCTCCTAGGGGAATAAATCTTTGGGCACCTAGTGGTCATG

1 CATGACCACTAGGAGCATCTTTGGCGACCCGTGAAGGTGCGAAGCTAGCGACCAGTGCTAGGGGAATAAATCTTTGGGCACCTAGTGGTCATG

1 CATGACCACTAGGAGCATCTTTGGCGAGATCGGGAGAATCAGTGGCATTGGTGTCTTCTAGGGGAATAAATCTTTGGGCACCTAGTGGTCATG

1 CATGACCACTAGGAGCATCTTTGGCGAGATCGGGAGAATCGGTGGTATTAGTGTCTCCTAGGGGAATAAATCTTTGGGCACCTAGTGGTCATG

1 CATGACCACTAGGAGCATCTTTGGCGAGATCGTGACAATCGGTGTCATTGGTGTCTCCTAGGGGAATAAATCTTTGGGCACCTAGTGGTCATG

1 CATGACCACTAGGAGCATCTTTGGCGACACTGGGGGCATGGAACAAAGTAGGGTCGACTAGGGGAATAAATCTTTGGGCACCTAGTGGTCATG

1 CATGACCACTAGGAGCATCTTTGGCGAGATCGGCAGAATCGGTGGCCTTGGTGTCTCCTAGGGGAATAAATCTTTGGGCACCTAGTGGTCATG

1 CATGACCACTAGGAGCATCTTTGGCGAAAGTAGTCGACCGTGCTATGTAATCGGGATCTAGGGGAATAAATCTTTGGGCACCTAGTGGTCATG

1 CATGACCACTAGGAGCATCTTTGGCGATATCGGGAGAACCGGTGGCATTGGTGTCTCCTAGGGGAATAAATCTTTGGGCACCTAGTGGTCATG

1 CATGACCACTAGGAGCATCTTTGGCGAAAAGACTCTGTATTCGGGGACCAGTTGCTGCTAGGGGAATAAATCTTTGGGCACCTAGTGGTCATG

1 CATGACCACTAGGAGCATCTTTGGCGAAGTGTGGAACATGCCGGTCTTGGTTGCACCCTAGGGGAATAAATCTTTGGGCACCTAGTGGTCATG

1 CATGACCACTAGGAGCATCTTTGGCGAGATCAGGAGAATCGGTGGCATTGATGTCTCCTAGGGGAATAAATCTTTGGGCACCTAGTGGTCATG

1 CATGACCACTAGGAGCATCTTTGGCGAACTGCAGTAGTTGCGGTGGTCTGTGAATCCCTAGGGGAATAAATCTTTGGGCACCTAGTGGTCATG

1 CATGACCACTAGGAGCATCTTTGGCGACCGGTAGTGGTTTTTTACTCACTGGGACTTCTAGGGGAATAAATCTTTGGGCACCTAGTGGTCATG

1 CATGACCACTAGGAGCATCTTTGGCGAGATCGGGAGAATCGGTGGCATAGGTTTCTCCTAGGGGAATAAATCTTTGGGCACCTAGTGGTCATG

1 CATGACCACTAGGAGCATCTTTGGCGAGATGCGGAGAAACGGTGGCGTGGGATTTACCTAGGGGAATAAATCTTTGGGCACCTAGTGGTCATG

1 CATGACCACTAGGAGCATCTTTGGCGACGACATGGGCATGCGTAAGAAGGGTCGAGACTAGGGGAATAAATCTTTGGGCACCTAGTGGTCATG

1 CATGACCACTAGGAGCATCTTTGGCGACTCCCACGGGGTGGGGACGAAGTGGAGCGACTAGGGGAATAAATCTTTGGGCACCTAGTGGTCATG

1 CATGACCACTAGGAGCATCTTTGGCGAGATCGGAACGAGTGGCAACAAGAATATCGACTAGGGGAATAAATCTTTGGGCACCTAGTGGTCATG

1 CATGACCACTAGGAGCATCTTTGGCGAAGGCTCATTTCGCTGGGAGGACGATGCTAACTAGGGGAATAAATCTTTGGGCACCTAGTGGTCATG

1 CATGACCACTAGGAGCATCTTTGGCGAACCGAAACGGGGGGGTAGTAGACTCGTAGACTAGGGGAATAAATCTTTGGGCACCTAGTGGTCATG

1 CATGACCACTAGGAGCATCTTTGGCGATAGGGACTTTCAAGATGGTGGAGAGTTGTTCTAGGGGAATAAATCTTTGGGCACCTAGTGGTCATG

1 CATGACCACTAGGAGCATCTTTGGCGATGGGAGTTGGCAGGCAGGCTAGGTGGGAAGCTAGGGGAATAAATCTTTGGGCACCTAGTGGTCATG

1 CATGACCACTAGGAGCATCTTTGGCGAGAAGACTACGGATTCGGGGACCAGTTGCTGCTAGGGGAATAAATCTTTGGGCACCTAGTGGTCATG

1 CATGACCACTAGGAGCATCTTTGGCGAGTGGTACGCGTGTTACGAACTGTGTGTTGTCTAGGGGAATAAATCTTTGGGCACCTAGTGGTCATG

1 CATGACCACTAGGAGCATCTTTGGCGAGCTTTGATGGGTCAAACTGGTTGCGTGGGTCTAGGGGAATAAATCTTTGGGCACCTAGTGGTCATG

1 CATGACCACTAGGAGCATCTTTGGCGACGCGGCTGGAGGTTTAGGCTCTGCTGTTTACTAGGGGAATAAATCTTTGGGCACCTAGTGGTCATG

1 CATGACCACTAGGAGCATCTTTGGCGAGATCAGCAGAATCGGCGGCATTGGTGTCTCCTAGGGGAATAAATCTTTGGGCACCTAGTGGTCATG

1 CATGACCACTAGGAGCATCTTTGGCGACAATGGAGCGACGGTGTGGTCTGAGCCCTACTAGGGGAATAAATCTTTGGGCACCTAGTGGTCATG

1 CATGACCACTAGGAGCATCTTTGGCGACGAGGGCATGCTAAGACGATTAAAGAGGAGCTAGGGGAATAAATCTTTGGGCACCTAGTGGTCATG

1 CATGACCACTAGGAGCATCTTTGGCGAGAACGGGAGAATCGGTGGCATTGGTGTCTACTAGGGGAATAAATCTTTGGGCACCTAGTGGTCATG

1 CATGACCACTAGGAGCATCTTTGGCGAGCGACAAAGGTGAGCATGTGGTCGAGCCAGCTAGGGGAATAAATCTTTGGGCACCTAGTGGTCATG

1 CATGACCACTAGGAGCATCTTTGGCGAACGGTGTAGGACTTCAAGTGGATCTCATAGCTAGGGGAATAAATCTTTGGGCACCTAGTGGTCATG

1 CATGACCACTAGGAGCATCTTTGGCGAGATCGGGAGAATTGGTTGCATTGGTGTCTCCTAGGGGAATAAATCTTTGGGCACCTAGTGGTCATG

1 CATGACCACTAGGAGCATCTTTGGCGATTGGTCATGTTCTGCCTGCAATTTCATTCACTAGGGGAATAAATCTTTGGGCACCTAGTGGTCATG

1 CATGACCACTAGGAGCATCTTTGGCGATCAGGGGGGCGTCAGACGCCGACTCACGGGCTAGGGGAATAAATCTTTGGGCACCTAGTGGTCATG

1 CATGACCACTAGGAGCATCTTTGGCGAGATCGGGAGAATCGGTTGCATTGGTGTCTACTAGGGGAATAAATCTTTGGGCACCTAGTGGTCATG

1 CATGACCACTAGGAGCATCTTTGGCGACCATCCAAAGGTAGGGCGGTATGGTGGGGCCTAGGGGAATAAATCTTTGGGCACCTAGTGGTCATG

1 CATGACCACTAGGAGCATCTTTGGCGACTAAGTGGCCCACGCGGTAAATGGGGTAGACTAGGGGAATAAATCTTTGGGCACCTAGTGGTCATG

1 CATGACCACTAGGAGCATCTTTGGCGATCCGGGGGCGTCACGACGCGACGCTGTGGGCTAGGGGAATAAATCTTTGGGCACCTAGTGGTCATG

1 CATGACCACTAGGAGCATCTTTGGCGACTGAGTTGGAAACGCGTTAAGTTGGCGAAGCTAGGGGAATAAATCTTTGGGCACCTAGTGGTCATG

1 CATGACCACTAGGAGCATCTTTGGCGAGGAGTGGCTGAGGGCGGTGGGTAGGCCGCGCTAGGGGAATAAATCTTTGGGCACCTAGTGGTCATG

1 CATGACCACTAGGAGCATCTTTGGCGAGCCTGGCTTGGGAGGTTGCTCCACTAGTTCCTAGGGGAATAAATCTTTGGGCACCTAGTGGTCATG

1 CATGACCACTAGGAGCATCTTTGGCGAGATCGGGAGAATCGGTGTCATTGGAGTCTCCTAGGGGAATAAATCTTTGGGCACCTAGTGGTCATG

1 CATGACCACTAGGAGCATCTTTGGCGACGGTGTGGGGACCGCAGGTTCGGCGGTTACCTAGGGGAATAAATCTTTGGGCACCTAGTGGTCATG

1 CATGACCACTAGGAGCATCTTTGGCGATACGGGGTGTTGCGCTGGTGGTTGGGGTCGCTAGGGGAATAAATCTTTGGGCACCTAGTGGTCATG

1 CATGACCACTAGGAGCATCTTTGGCGAGATCGAGAGAATCGGTGGCATTGGCGTCTCCTAGGGGAATAAATCTTTGGGCACCTAGTGGTCATG

1 CATGACCACTAGGAGCATCTTTGGCGAGAAGACTATGGATTCGGGTACCAGTTGCTTCTAGGGGAATAAATCTTTGGGCACCTAGTGGTCATG

1 CATGACCACTAGGAGCATCTTTGGCGAGATCGGGAGAATCGGAGGCATTGGTGTATCCTAGGGGAATAAATCTTTGGGCACCTAGTGGTCATG

1 CATGACCACTAGGAGCATCTTTGGCGAGATCTGTAGAAACGGTGGCATTGGTGTCTCCTAGGGGAATAAATCTTTGGGCACCTAGTGGTCATG

1 CATGACCACTAGGAGCATCTTTGGCGAGATCGGTAGAATCGCTGGAATTGGTGTCTCCTAGGGGAATAAATCTTTGGGCACCTAGTGGTCATG

1 CATGACCACTAGGAGCATCTTTGGCGATCAAAGCACGCCGTGGCTGAGAAAAGTCGACTAGGGGAATAAATCTTTGGGCACCTAGTGGTCATG

1 CATGACCACTAGGAGCATCTTTGGCGATTTCCGGCTGGATAGGGTTGCCGCCCTGTGCTAGGGGAATAAATCTTTGGGCACCTAGTGGTCATG

1 CATGACCACTAGGAGCATCTTTGGCGAAGACGACACGGTCGATCGGGTTAGAGGAAGCTAGGGGAATAAATCTTTGGGCACCTAGTGGTCATG

1 CATGACCACTAGGAGCATCTTTGGCGAGATCAGGAGAATCGGTGCCATTGGTGTCTCCTAGGGGAATAAATCTTTGGGCACCTAGTGGTCATG

1 CATGACCACTAGGAGCATCTTTGGCGACAAAGGTGTTCCGCAAGTGGCACTGTGGAGCTAGGGGAATAAATCTTTGGGCACCTAGTGGTCATG

1 CATGACCACTAGGAGCATCTTTGGCGAATACGGGAACTAGGTATGTGGTATGCTCCTCTAGGGGAATAAATCTTTGGGCACCTAGTGGTCATG

1 CATGACCACTAGGAGCATCTTTGGCGATCCTTGCTTGGGAGTTTGCTCCACCAGTTCCTAGGGGAATAAATCTTTGGGCACCTAGTGGTCATG

1 CATGACCACTAGGAGCATCTTTGGCGATTCTCGTCAGATGGTGAAGCAGACGTTTGGCTAGGGGAATAAATCTTTGGGCACCTAGTGGTCATG

1 CATGACCACTAGGAGCATCTTTGGCGAGTAGTGGCTGAGGTATGTTCACGTACGAAACTAGGGGAATAAATCTTTGGGCACCTAGTGGTCATG

1 CATGACCACTAGGAGCATCTTTGGCGAGGAAACCGGGCACGGGCGCGACGGGCGAGACTAGGGGAATAAATCTTTGGGCACCTAGTGGTCATG

1 CATGACCACTAGGAGCATCTTTGGCGAGAGCGGGAGAATCGGTGGCATTTGTGTCTCCTAGGGGAATAAATCTTTGGGCACCTAGTGGTCATG

1 CATGACCACTAGGAGCATCTTTGGCGAGATCGGGAGACTCGGCGGCATTGGTGTCTCCTAGGGGAATAAATCTTTGGGCACCTAGTGGTCATG

1 CATGACCACTAGGAGCATCTTTGGCGACGATTGGTCGGAAGGTAAGTGTTCGGATGGCTAGGGGAATAAATCTTTGGGCACCTAGTGGTCATG

1 CATGACCACTAGGAGCATCTTTGGCGATCCGGGGGCGTGCTATGCGACGCTTATGGGCTAGGGGAATAAATCTTTGGGCACCTAGTGGTCATG

1 CATGACCACTAGGAGCATCTTTGGCGACCCCGGCTATGCATGGCCAGGTAGTCCTATCTAGGGGAATAAATCTTTGGGCACCTAGTGGTCATG

1 CATGACCACTAGGAGCATCTTTGGCGATAAGACTCTGGATGCGGGGACCAGTTGCTGCTAGGGGAATAAATCTTTGGGCACCTAGTGGTCATG

1 CATGACCACTAGGAGCATCTTTGGCGATCGCATAGCGTACCAACATCGGGAGGGGGCCTAGGGGAATAAATCTTTGGGCACCTAGTGGTCATG

1 CATGACCACTAGGAGCATCTTTGGCGAGATCGGGACAATCGGTCGCATTGGTGTCTCCTAGGGGAATAAATCTTTGGGCACCTAGTGGTCATG

1 CATGACCACTAGGAGCATCTTTGGCGATTTTCCCCGTGGGAGGGGAGCCATGCGCTCCTAGGGGAATAAATCTTTGGGCACCTAGTGGTCATG

1 CATGACCACTAGGAGCATCTTTGGCGAAATCGGGAGAATCGGTGGCATTTGTGTCTCCTAGGGGAATAAATCTTTGGGCACCTAGTGGTCATG

1 CATGACCACTAGGAGCATCTTTGGCGAGGTCGGCAGAATCGGTGGCATTGGTGTCTCCTAGGGGAATAAATCTTTGGGCACCTAGTGGTCATG

1 CATGACCACTAGGAGCATCTTTGGCGAGATCGGGAGAGTCTGTGGCATTGGTGTCTCCTAGGGGAATAAATCTTTGGGCACCTAGTGGTCATG

1 CATGACCACTAGGAGCATCTTTGGCGAAAATTAACCACGCAGTGTACTCGTCCTGAGCTAGGGGAATAAATCTTTGGGCACCTAGTGGTCATG

1 CATGACCACTAGGAGCATCTTTGGCGAGATCGGTAGAATCGTTGGAATTGGTGTCTCCTAGGGGAATAAATCTTTGGGCACCTAGTGGTCATG

1 CATGACCACTAGGAGCATCTTTGGCGATCCGGGGGTGCGTGGGCACGACTTAACAGGCTAGGGGAATAAATCTTTGGGCACCTAGTGGTCATG

1 CATGACCACTAGGAGCATCTTTGGCGAGAGAATCGGTAGGCAGAGTGCTCCAGCGAGCTAGGGGAATAAATCTTTGGGCACCTAGTGGTCATG

1 CATGACCACTAGGAGCATCTTTGGCGACTAAGTGGTCAACGCGTTAAGTGGTGTCTCCTAGGGGAATAAATCTTTGGGCACCTAGTGGTCATG

1 CATGACCACTAGGAGCATCTTTGGCGAACAATATATTGGGGGGCAGATGTCTCGTAGCTAGGGGAATAAATCTTTGGGCACCTAGTGGTCATG

1 CATGACCACTAGGAGCATCTTTGGCGAGATCCGGAGAATCGGAGGCATTGGTGTCTCCTAGGGGAATAAATCTTTGGGCACCTAGTGGTCATG

1 CATGACCACTAGGAGCATCTTTGGCGATACCAATTTAGCGCGACGCGTGTGATTACTCTAGGGGAATAAATCTTTGGGCACCTAGTGGTCATG

1 CATGACCACTAGGAGCATCTTTGGCGACATAGTAACTCAGCGGGATTTATAACGGGTCTAGGGGAATAAATCTTTGGGCACCTAGTGGTCATG

1 CATGACCACTAGGAGCATCTTTGGCGAGATCGGGAGTATCGGTGGCACTGGTGTCTCCTAGGGGAATAAATCTTTGGGCACCTAGTGGTCATG

1 CATGACCACTAGGAGCATCTTTGGCGACGGTGTGGGGAACCTGTTTCGGCGGTGCTACTAGGGGAATAAATCTTTGGGCACCTAGTGGTCATG

1 CATGACCACTAGGAGCATCTTTGGCGATGGCGTGGGGCCATTTGTGCGGCGGATCCCCTAGGGGAATAAATCTTTGGGCACCTAGTGGTCATG

1 CATGACCACTAGGAGCATCTTTGGCGATCCGAGAATGCAGTGATGCCGGTGTGGAGCCTAGGGGAATAAATCTTTGGGCACCTAGTGGTCATG

1 CATGACCACTAGGAGCATCTTTGGCGAGATAGGGAGAATCGGTGTCGGAGTGCTCTCCTAGGGGAATAAATCTTTGGGCACCTAGTGGTCATG

1 CATGACCACTAGGAGCATCTTTGGCGAGGGGTAGGGGGCCGCTCGCGCCTCCGTGCGCTAGGGGAATAAATCTTTGGGCACCTAGTGGTCATG

1 CATGACCACTAGGAGCATCTTTGGCGAAAGTGCGATAGCAGGTTTACGAAGTGGAGCCTAGGGGAATAAATCTTTGGGCACCTAGTGGTCATG

1 CATGACCACTAGGAGCATCTTTGGCGAACGCCAGGTGTTGCGGTGGAGTGTGAAACCCTAGGGGAATAAATCTTTGGGCACCTAGTGGTCATG

1 CATGACCACTAGGAGCATCTTTGGCGAGACCGGGAGACTCGGTGGTATTGGTGTCTCCTAGGGGAATAAATCTTTGGGCACCTAGTGGTCATG

1 CATGACCACTAGGAGCATCTTTGGCGAGTATTGGCTGGTAGGTTGCGTATGGGGGAGCTAGGGGAATAAATCTTTGGGCACCTAGTGGTCATG

1 CATGACCACTAGGAGCATCTTTGGCGAGAAGACTTTGGATTCGGAGACCAGTTGCTGCTAGGGGAATAAATCTTTGGGCACCTAGTGGTCATG

1 CATGACCACTAGGAGCATCTTTGGCGATAAGACTCTGGATTCGGGGACCAGTTGATGCTAGGGGAATAAATCTTTGGGCACCTAGTGGTCATG

1 CATGACCACTAGGAGCATCTTTGGCGAGCCTCGGTTGGGAGGTTGCTCCACCAGTTCCTAGGGGAATAAATCTTTGGGCACCTAGTGGTCATG

1 CATGACCACTAGGAGCATCTTTGGCGAAAGTCGGTAGAACAGGGTGGGGCGCTGTCTCTAGGGGAATAAATCTTTGGGCACCTAGTGGTCATG

1 CATGACCACTAGGAGCATCTTTGGCGAGGATCGTTGGTGCGTAAGCGTGGACAAAGACTAGGGGAATAAATCTTTGGGCACCTAGTGGTCATG

1 CATGACCACTAGGAGCATCTTTGGCGAGATCGTCCTGGCACAGCGCGGGGCTGTTACCTAGGGGAATAAATCTTTGGGCACCTAGTGGTCATG

1 CATGACCACTAGGAGCATCTTTGGCGAGATCGGGAGAATCGGCGGCATTAGTGTCTCCTAGGGGAATAAATCTTTGGGCACCTAGTGGTCATG

1 CATGACCACTAGGAGCATCTTTGGCGAGAAGACTCTGAATTCGGGGACCAGTTGCTCCTAGGGGAATAAATCTTTGGGCACCTAGTGGTCATG

1 CATGACCACTAGGAGCATCTTTGGCGAGCGATTTTTTCGTCTCCGAAGGTCCATTCTCTAGGGGAATAAATCTTTGGGCACCTAGTGGTCATG

1 CATGACCACTAGGAGCATCTTTGGCGACTAAGTGGCCAATGCGTTAAATGGGGTAGACTAGGGGAATAAATCTTTGGGCACCTAGTGGTCATG

1 CATGACCACTAGGAGCATCTTTGGCGAGATCGGGAGAACCGGAGGCATTGGTGTCTCCTAGGGGAATAAATCTTTGGGCACCTAGTGGTCATG

1 CATGACCACTAGGAGCATCTTTGGCGATCCGGGCGGCTGTGCAGCCGACGCAAAGGGCTAGGGGAATAAATCTTTGGGCACCTAGTGGTCATG

1 CATGACCACTAGGAGCATCTTTGGCGAGGCTCGAGCAGTAGTTGGTCTCGTTGTACGCTAGGGGAATAAATCTTTGGGCACCTAGTGGTCATG

1 CATGACCACTAGGAGCATCTTTGGCGACGTCGTGTGTGAGAGCGTTCAAACTGTAAACTAGGGGAATAAATCTTTGGGCACCTAGTGGTCATG

1 CATGACCACTAGGAGCATCTTTGGCGATCCGGGGGTCAGCTAAGACGGCGCGTTGGGCTAGGGGAATAAATCTTTGGGCACCTAGTGGTCATG

1 CATGACCACTAGGAGCATCTTTGGCGAGATCGAGAGACTCGGTGGCATTGGTGTCTCCTAGGGGAATAAATCTTTGGGCACCTAGTGGTCATG

1 CATGACCACTAGGAGCATCTTTGGCGACAAGTAGTGCTGGCTCTACGAGTCATAGTGCTAGGGGAATAAATCTTTGGGCACCTAGTGGTCATG

1 CATGACCACTAGGAGCATCTTTGGCGAGATCGGGAGAATCGGTGGCGCTGGTGTCTCCTAGGGGAATAAATCTTTGGGCACCTAGTGGTCATG

1 CATGACCACTAGGAGCATCTTTGGCGAGATCGGGAGAATCTTTGGCATTGGTGTCTCCTAGGGGAATAAATCTTTGGGCACCTAGTGGTCATG

1 CATGACCACTAGGAGCATCTTTGGCGAGGGGCCTAAGTGCGTATCTTTGGTCGAGCTCTAGGGGAATAAATCTTTGGGCACCTAGTGGTCATG

1 CATGACCACTAGGAGCATCTTTGGCGAGGCATTAGGGGTTGGCTGCCGTCATCAGGACTAGGGGAATAAATCTTTGGGCACCTAGTGGTCATG

1 CATGACCACTAGGAGCATCTTTGGCGAGGGAGGGCGCCTGCAGCGGTGTGAATGCGCCTAGGGGAATAAATCTTTGGGCACCTAGTGGTCATG

1 CATGACCACTAGGAGCATCTTTGGCGAGCGGTTCGTCCCGGCCGATGCTCGGACAAGCTAGGGGAATAAATCTTTGGGCACCTAGTGGTCATG

1 CATGACCACTAGGAGCATCTTTGGCGAGAAGACGCTGGAGTCGGGGACCAGTTGCTGCTAGGGGAATAAATCTTTGGGCACCTAGTGGTCATG

1 CATGACCACTAGGAGCATCTTTGGCGAGATCGGGAGTATCGGTGGCATCGGTGTCTCCTAGGGGAATAAATCTTTGGGCACCTAGTGGTCATG

1 CATGACCACTAGGAGCATCTTTGGCGAAAGTCGGTAGAACAGGGTGGGGTGCTGGCCCTAGGGGAATAAATCTTTGGGCACCTAGTGGTCATG

1 CATGACCACTAGGAGCATCTTTGGCGATCCTCGTCAGATGGTGAAGCAGACGTTTGACTAGGGGAATAAATCTTTGGGCACCTAGTGGTCATG

1 CATGACCACTAGGAGCATCTTTGGCGAACGGCTGGTGTTGCGGTGGTCTGTGGATCCCTAGGGGAATAAATCTTTGGGCACCTAGTGGTCATG

1 CATGACCACTAGGAGCATCTTTGGCGAGATCGGGAGAATCCGTGGCATTGGTTTCTCCTAGGGGAATAAATCTTTGGGCACCTAGTGGTCATG

1 CATGACCACTAGGAGCATCTTTGGCGAGATCGGGACAATCGGTGGCATTGGTGCCTCCTAGGGGAATAAATCTTTGGGCACCTAGTGGTCATG

1 CATGACCACTAGGAGCATCTTTGGCGACCGGCAGGTGTTGCGGTGGCCTGTGAATCCCTAGGGGAATAAATCTTTGGGCACCTAGTGGTCATG

1 CATGACCACTAGGAGCATCTTTGGCGAAAGGCAGGTGTTGCGGTTGTCTGTGAATCCCTAGGGGAATAAATCTTTGGGCACCTAGTGGTCATG

1 CATGACCACTAGGAGCATCTTTGGCGAGGGAGGGCGCCGGCAGCGGTGAGAATGCGCCTAGGGGAATAAATCTTTGGGCACCTAGTGGTCATG

1 CATGACCACTAGGAGCATCTTTGGCGAACGTGCAAGAAATGCTGCCTGGAGCGTAGGCTAGGGGAATAAATCTTTGGGCACCTAGTGGTCATG

1 CATGACCACTAGGAGCATCTTTGGCGATCGCTGTATGGTTCTTGGATTTCTGTTTTACTAGGGGAATAAATCTTTGGGCACCTAGTGGTCATG

1 CATGACCACTAGGAGCATCTTTGGCGAGTTACGCTCCGTTCGGCGTTCTCCGGGGCCCTAGGGGAATAAATCTTTGGGCACCTAGTGGTCATG

1 CATGACCACTAGGAGCATCTTTGGCGAGATCGGGAGAATCGGGCGCATTGTTGTCTCCTAGGGGAATAAATCTTTGGGCACCTAGTGGTCATG

1 CATGACCACTAGGAGCATCTTTGGCGACCCGAGAACGTATGTGTAAATCCGGGTGGACTAGGGGAATAAATCTTTGGGCACCTAGTGGTCATG

1 CATGACCACTAGGAGCATCTTTGGCGAATCGGCCTCATCTCTCGAGGATCCGCGGTGCTAGGGGAATAAATCTTTGGGCACCTAGTGGTCATG

1 CATGACCACTAGGAGCATCTTTGGCGACTCGTGGTCAATGATTAAGGTCTTGTCTGGCTAGGGGAATAAATCTTTGGGCACCTAGTGGTCATG

1 CATGACCACTAGGAGCATCTTTGGCGAGGCGGCTTGTGCTGGCTAGGTAAAGCTTCGCTAGGGGAATAAATCTTTGGGCACCTAGTGGTCATG

1 CATGACCACTAGGAGCATCTTTGGCGACGGCTAGTGCGTCGAGCAGAGTGGACCCAGCTAGGGGAATAAATCTTTGGGCACCTAGTGGTCATG

1 CATGACCACTAGGAGCATCTTTGGCGAACGGCAGGTGTTGTGGTGGTCTGTTAATCCCTAGGGGAATAAATCTTTGGGCACCTAGTGGTCATG

1 CATGACCACTAGGAGCATCTTTGGCGAGGAGTGCGTGGTGGTCGGTGTACGACCCGGCTAGGGGAATAAATCTTTGGGCACCTAGTGGTCATG

1 CATGACCACTAGGAGCATCTTTGGCGACCCACCGGGAGTCGATGTGTTCTGTCTTCTCTAGGGGAATAAATCTTTGGGCACCTAGTGGTCATG

1 CATGACCACTAGGAGCATCTTTGGCGAGAGAATAGGTAGGCAGAGTGCTCCAGCCAGCTAGGGGAATAAATCTTTGGGCACCTAGTGGTCATG

1 CATGACCACTAGGAGCATCTTTGGCGAGATCGGGAGAATAGGTGGCATAGGAGTCTCCTAGGGGAATAAATCTTTGGGCACCTAGTGGTCATG

1 CATGACCACTAGGAGCATCTTTGGCGAGGGGACCCGCTTCTAGGATGGGCTTTGTCACTAGGGGAATAAATCTTTGGGCACCTAGTGGTCATG

1 CATGACCACTAGGAGCATCTTTGGCGATGGGTGCTGACGGCCTCCGCTGCGGCTACACTAGGGGAATAAATCTTTGGGCACCTAGTGGTCATG

1 CATGACCACTAGGAGCATCTTTGGCGATATCGGGAGAATCGGTGTCATTGGTGTATCCTAGGGGAATAAATCTTTGGGCACCTAGTGGTCATG

1 CATGACCACTAGGAGCATCTTTGGCGAGCCTCGCTTGGGAGGTTGCTTTACCAGTTCCTAGGGGAATAAATCTTTGGGCACCTAGTGGTCATG

1 CATGACCACTAGGAGCATCTTTGGCGAGATCGGGTGAATCGGTGGCATTGGTGCCTCCTAGGGGAATAAATCTTTGGGCACCTAGTGGTCATG

1 CATGACCACTAGGAGCATCTTTGGCGAAAGTCGGTAGAACAGGGCGAGGTGCTGTCCCTAGGGGAATAAATCTTTGGGCACCTAGTGGTCATG

1 CATGACCACTAGGAGCATCTTTGGCGAGAAGACTATGGATTTGGGGACCAGTTGCTGCTAGGGGAATAAATCTTTGGGCACCTAGTGGTCATG

1 CATGACCACTAGGAGCATCTTTGGCGATGGGTGCTGACGGCCGCCGCTGAGGCTACACTAGGGGAATAAATCTTTGGGCACCTAGTGGTCATG

1 CATGACCACTAGGAGCATCTTTGGCGAGATCGGGAGAATCGGTGGTATCGGTGTCTCCTAGGGGAATAAATCTTTGGGCACCTAGTGGTCATG

1 CATGACCACTAGGAGCATCTTTGGCGAGACGTAGTAGTCCACTTCTCTAATGCCCGACTAGGGGAATAAATCTTTGGGCACCTAGTGGTCATG

1 CATGACCACTAGGAGCATCTTTGGCGATAAATGCTGGTAGGCATTATTCTTTGATTTCTAGGGGAATAAATCTTTGGGCACCTAGTGGTCATG

1 CATGACCACTAGGAGCATCTTTGGCGACCAGATTTGGTAGGTAATGTGCTGAGGAGGCTAGGGGAATAAATCTTTGGGCACCTAGTGGTCATG

1 CATGACCACTAGGAGCATCTTTGGCGAAAGTCGGTAGAACAGGGTGTGGTGATGTCCCTAGGGGAATAAATCTTTGGGCACCTAGTGGTCATG

1 CATGACCACTAGGAGCATCTTTGGCGAACGAGGGTGCCAAGTGGTCGAGTACATTGCCTAGGGGAATAAATCTTTGGGCACCTAGTGGTCATG

1 CATGACCACTAGGAGCATCTTTGGCGAGATCGGGAGAATCGCTGGCATTGGTGTTTCCTAGGGGAATAAATCTTTGGGCACCTAGTGGTCATG

1 CATGACCACTAGGAGCATCTTTGGCGAACGGCAGGTGTTGCGGTGGGCTGTGAAACCCTAGGGGAATAAATCTTTGGGCACCTAGTGGTCATG

1 CATGACCACTAGGAGCATCTTTGGCGATCCGGGGGGCTTTGAGGCCGACGCAATGGGCTAGGGGAATAAATCTTTGGGCACCTAGTGGTCATG

1 CATGACCACTAGGAGCATCTTTGGCGAGTACACCGGCAAGGAAGGGAAACGTTGCGACTAGGGGAATAAATCTTTGGGCACCTAGTGGTCATG

1 CATGACCACTAGGAGCATCTTTGGCGATGCGGTTCACCGTGTACGGGTTGGCGAAGTCTAGGGGAATAAATCTTTGGGCACCTAGTGGTCATG

1 CATGACCACTAGGAGCATCTTTGGCGAACTGCAGGTGTTGCGGTGGTCTGTGAAACACTAGGGGAATAAATCTTTGGGCACCTAGTGGTCATG

1 CATGACCACTAGGAGCATCTTTGGCGATCCGGCCGAACCAAGGTGCGAGGAGTGGAGCTAGGGGAATAAATCTTTGGGCACCTAGTGGTCATG

1 CATGACCACTAGGAGCATCTTTGGCGACGATTGGTCAGAAGGCAAGTGTTCGGATGACTAGGGGAATAAATCTTTGGGCACCTAGTGGTCATG

1 CATGACCACTAGGAGCATCTTTGGCGAACGGCAGGTGTTGCGGTGGTCTATAAATCCCTAGGGGAATAAATCTTTGGGCACCTAGTGGTCATG

1 CATGACCACTAGGAGCATCTTTGGCGAGAGGACATCGGCCCAAAGCTTTCTCAGCGCCTAGGGGAATAAATCTTTGGGCACCTAGTGGTCATG

1 CATGACCACTAGGAGCATCTTTGGCGAGAGTCCAAGTATGCGGTAGTGGTCGAGCCTCTAGGGGAATAAATCTTTGGGCACCTAGTGGTCATG

1 CATGACCACTAGGAGCATCTTTGGCGAGCATCGGAGGAGGGGCGGTCTCAGACGTGCCTAGGGGAATAAATCTTTGGGCACCTAGTGGTCATG

1 CATGACCACTAGGAGCATCTTTGGCGAGATAGGGATAATCGGTGGCATAGGTGTCTCCTAGGGGAATAAATCTTTGGGCACCTAGTGGTCATG

1 CATGACCACTAGGAGCATCTTTGGCGAGGCGGGCGGTGGACATCCCTCGAACCTTAACTAGGGGAATAAATCTTTGGGCACCTAGTGGTCATG

1 CATGACCACTAGGAGCATCTTTGGCGAGGTAGCGAACTCTCTCCAAGGCGCGCTCTACTAGGGGAATAAATCTTTGGGCACCTAGTGGTCATG

1 CATGACCACTAGGAGCATCTTTGGCGAGATCGGAAGAATCGGTGGCATTGGTGGCTCCTAGGGGAATAAATCTTTGGGCACCTAGTGGTCATG

1 CATGACCACTAGGAGCATCTTTGGCGAAGGGTGTATGACTTCAAGTGGATCTCATAGCTAGGGGAATAAATCTTTGGGCACCTAGTGGTCATG

1 CATGACCACTAGGAGCATCTTTGGCGAAATGAACTCAGGTTGTAGGTTCACTGTCTCCTAGGGGAATAAATCTTTGGGCACCTAGTGGTCATG

1 CATGACCACTAGGAGCATCTTTGGCGAGATCGTGAGAATCTGTGGCAATGGTGTCTCCTAGGGGAATAAATCTTTGGGCACCTAGTGGTCATG

1 CATGACCACTAGGAGCATCTTTGGCGAATGGCCGGTGTTGCGGTGGTCTGTGAATCCCTAGGGGAATAAATCTTTGGGCACCTAGTGGTCATG

1 CATGACCACTAGGAGCATCTTTGGCGATGGGGCCGTGCTATCTGCACACACCGCGGGCTAGGGGAATAAATCTTTGGGCACCTAGTGGTCATG

1 CATGACCACTAGGAGCATCTTTGGCGACAGGGAAGAGCAATGGAAGAGAATAGTCGACTAGGGGAATAAATCTTTGGGCACCTAGTGGTCATG

1 CATGACCACTAGGAGCATCTTTGGCGAGAAGCCAGGAGTGTGTGTTCATTTGATTGTCTAGGGGAATAAATCTTTGGGCACCTAGTGGTCATG

1 CATGACCACTAGGAGCATCTTTGGCGAGATCGGGAGAATCGGTGGCATTGGTGTTTACTAGGGGAATAAATCTTTGGGCACCTAGTGGTCATG

1 CATGACCACTAGGAGCATCTTTGGCGAGATTGGGAGAATCGGTGGCATGGGTGTCTCCTAGGGGAATAAATCTTTGGGCACCTAGTGGTCATG

1 CATGACCACTAGGAGCATCTTTGGCGACTAAGTGGCCAGCGCGTTAAGTGGGGTAGACTAGGGGAATAAATCTTTGGGCACCTAGTGGTCATG

1 CATGACCACTAGGAGCATCTTTGGCGACGGGGGCATGCACAGGATCGAGACAGTCGACTAGGGGAATAAATCTTTGGGCACCTAGTGGTCATG

1 CATGACCACTAGGAGCATCTTTGGCGAACGGCAGGTGTTGCGGTGGTATGTGAATCACTAGGGGAATAAATCTTTGGGCACCTAGTGGTCATG

1 CATGACCACTAGGAGCATCTTTGGCGAGAAAGGGAGAATCGGAGGCATTGGAGTCTCCTAGGGGAATAAATCTTTGGGCACCTAGTGGTCATG

1 CATGACCACTAGGAGCATCTTTGGCGATAAAGGTGATCGGTGCTCGGGTGCCTGGCCCTAGGGGAATAAATCTTTGGGCACCTAGTGGTCATG

1 CATGACCACTAGGAGCATCTTTGGCGAACCACACTCGGCGTTAGCACAAGTTTGAGACTAGGGGAATAAATCTTTGGGCACCTAGTGGTCATG

1 CATGACCACTAGGAGCATCTTTGGCGAACGGTACCGTGGACATTCGGGGCTCCAGAACTAGGGGAATAAATCTTTGGGCACCTAGTGGTCATG

1 CATGACCACTAGGAGCATCTTTGGCGAGATCGGGAGAATCGGTGTCATTGGTGTCCCCTAGGGGAATAAATCTTTGGGCACCTAGTGGTCATG

1 CATGACCACTAGGAGCATCTTTGGCGAGATTGGGAGAATCGGTGGCTTTGGTGTCTCCTAGGGGAATAAATCTTTGGGCACCTAGTGGTCATG

1 CATGACCACTAGGAGCATCTTTGGCGAGATCGGTAGAATCGGTGGCATGGGTGTCTCCTAGGGGAATAAATCTTTGGGCACCTAGTGGTCATG

1 CATGACCACTAGGAGCATCTTTGGCGACTCGCGAAGCACACAATTGCGACCTTGTGGCTAGGGGAATAAATCTTTGGGCACCTAGTGGTCATG

1 CATGACCACTAGGAGCATCTTTGGCGAATGGCCGGCACGGCGTTCTAGTCCTCGGTACTAGGGGAATAAATCTTTGGGCACCTAGTGGTCATG

1 CATGACCACTAGGAGCATCTTTGGCGAGATCGGTAGAATCGGCGGCATTGGTGTCTGCTAGGGGAATAAATCTTTGGGCACCTAGTGGTCATG

1 CATGACCACTAGGAGCATCTTTGGCGAGATCGGGAGAATAGGTTGCATTGGTTTCTCCTAGGGGAATAAATCTTTGGGCACCTAGTGGTCATG

1 CATGACCACTAGGAGCATCTTTGGCGAGCACCCGACGAGGGGGGAGCAGACGTTTGGCTAGGGGAATAAATCTTTGGGCACCTAGTGGTCATG

1 CATGACCACTAGGAGCATCTTTGGCGAGCCTCGCTTGGGAGGTTGCTCCACCCGTTCCTAGGGGAATAAATCTTTGGGCACCTAGTGGTCATG

1 CATGACCACTAGGAGCATCTTTGGCGAAAGTCGGTATAACAGGGTGGGGCGCTGTCACTAGGGGAATAAATCTTTGGGCACCTAGTGGTCATG

1 CATGACCACTAGGAGCATCTTTGGCGAACGGCAGGTGTTGCGGTGGTCTGAGAATACCTAGGGGAATAAATCTTTGGGCACCTAGTGGTCATG

1 CATGACCACTAGGAGCATCTTTGGCGAGATCGGGAGAATCGGTGGCAGTGGTATCTCCTAGGGGAATAAATCTTTGGGCACCTAGTGGTCATG

1 CATGACCACTAGGAGCATCTTTGGCGAGTTTTGCTTGGGAGGTTGCTCCACCAGTTCCTAGGGGAATAAATCTTTGGGCACCTAGTGGTCATG

1 CATGACCACTAGGAGCATCTTTGGCGACCGTATGAAAACAAAAGTGGCCTTGCTAGCCTAGGGGAATAAATCTTTGGGCACCTAGTGGTCATG

1 CATGACCACTAGGAGCATCTTTGGCGAGATCGGGGGAATCGGTGGCATTGGTGTCTACTAGGGGAATAAATCTTTGGGCACCTAGTGGTCATG

1 CATGACCACTAGGAGCATCTTTGGCGAACGGCAGGTGTTTTGGTGGTCTGTGAATCCCTAGGGGAATAAATCTTTGGGCACCTAGTGGTCATG

1 CATGACCACTAGGAGCATCTTTGGCGACGAGTTGATGAGGTCGAGCGTGCGGGAAGGCTAGGGGAATAAATCTTTGGGCACCTAGTGGTCATG

1 CATGACCACTAGGAGCATCTTTGGCGACAGTCAGGGGTGTGGAGATGTGGTGAGTCGCTAGGGGAATAAATCTTTGGGCACCTAGTGGTCATG

1 CATGACCACTAGGAGCATCTTTGGCGACGCGATGGAACGTAGGCACATCGACGTGGACTAGGGGAATAAATCTTTGGGCACCTAGTGGTCATG

1 CATGACCACTAGGAGCATCTTTGGCGAGGGCGTGGGGCCATCTGTGCGGCGGATCCCCTAGGGGAATAAATCTTTGGGCACCTAGTGGTCATG

1 CATGACCACTAGGAGCATCTTTGGCGACAAGGGAGTTGAAGTGACTGCGACGTAGTGCTAGGGGAATAAATCTTTGGGCACCTAGTGGTCATG

1 CATGACCACTAGGAGCATCTTTGGCGAACGGCATGTGTTGCGTTGGTCTGTGAATCCCTAGGGGAATAAATCTTTGGGCACCTAGTGGTCATG

1 CATGACCACTAGGAGCATCTTTGGCGAAGGGTGTAGGACTTCTAGTGGATCTCATAGCTAGGGGAATAAATCTTTGGGCACCTAGTGGTCATG

1 CATGACCACTAGGAGCATCTTTGGCGAACTGCAAGTGTTGCGGTGGTCTGTGAATCCCTAGGGGAATAAATCTTTGGGCACCTAGTGGTCATG

1 CATGACCACTAGGAGCATCTTTGGCGAGATCGGGAGAATCGGTGGCATTTGCGTCTCCTAGGGGAATAAATCTTTGGGCACCTAGTGGTCATG

1 CATGACCACTAGGAGCATCTTTGGCGACCGTAGGAAAACAAAAGTGGCCTTGCTAGTCTAGGGGAATAAATCTTTGGGCACCTAGTGGTCATG

1 CATGACCACTAGGAGCATCTTTGGCGAGATCGGGAGAATCGGTGTCATTGGTGTATCCTAGGGGAATAAATCTTTGGGCACCTAGTGGTCATG

1 CATGACCACTAGGAGCATCTTTGGCGAGCTCGGGAGAATCGGTGGCATTTGTGTCTCCTAGGGGAATAAATCTTTGGGCACCTAGTGGTCATG

1 CATGACCACTAGGAGCATCTTTGGCGAGAAGGGCTAGAGGTGACAGAGTGGACTCGGCTAGGGGAATAAATCTTTGGGCACCTAGTGGTCATG

1 CATGACCACTAGGAGCATCTTTGGCGACGGATTTTCTTGAAGGGCCCTAGTTCTGAACTAGGGGAATAAATCTTTGGGCACCTAGTGGTCATG

1 CATGACCACTAGGAGCATCTTTGGCGAGATCGCGAGAATCGGTGGCATTGGTGCCTCCTAGGGGAATAAATCTTTGGGCACCTAGTGGTCATG

1 CATGACCACTAGGAGCATCTTTGGCGATCGCACAGCGGACTGAGGAACAAGACGGGCCTAGGGGAATAAATCTTTGGGCACCTAGTGGTCATG

1 CATGACCACTAGGAGCATCTTTGGCGAGATCGCGAGAATCTGTGGCATTGGTGTCTCCTAGGGGAATAAATCTTTGGGCACCTAGTGGTCATG

1 CATGACCACTAGGAGCATCTTTGGCGAGGGGTCATGCGAATGTAATTGCGCTTAAGACTAGGGGAATAAATCTTTGGGCACCTAGTGGTCATG

1 CATGACCACTAGGAGCATCTTTGGCGAGATCGGCAGAATCGGGGGCATTGGTGTCTCCTAGGGGAATAAATCTTTGGGCACCTAGTGGTCATG

1 CATGACCACTAGGAGCATCTTTGGCGAATAGCCGGCACGGCCTTCTAGTCCTTGGTACTAGGGGAATAAATCTTTGGGCACCTAGTGGTCATG

1 CATGACCACTAGGAGCATCTTTGGCGAGATCGGGAGAATCGGTGGCATGTGTGTCTCCTAGGGGAATAAATCTTTGGGCACCTAGTGGTCATG

1 CATGACCACTAGGAGCATCTTTGGCGAACGGCAGGTGTAGCGGTGGTCTGTGAAACCCTAGGGGAATAAATCTTTGGGCACCTAGTGGTCATG

1 CATGACCACTAGGAGCATCTTTGGCGAGAAGACTCCTGATTCGGGGACCAGTTGCTGCTAGGGGAATAAATCTTTGGGCACCTAGTGGTCATG

1 CATGACCACTAGGAGCATCTTTGGCGAGTTTGCGGCAATGCCACTGCGTTCCTGGCCCTAGGGGAATAAATCTTTGGGCACCTAGTGGTCATG

1 CATGACCACTAGGAGCATCTTTGGCGAAGTGCGGTGGGCATGTGGGCGGTAGCGACGCTAGGGGAATAAATCTTTGGGCACCTAGTGGTCATG

1 CATGACCACTAGGAGCATCTTTGGCGAGTGGTCGTTAGGGGATGGTTAAGGTCGAGACTAGGGGAATAAATCTTTGGGCACCTAGTGGTCATG

1 CATGACCACTAGGAGCATCTTTGGCGAAACGAATGTGTAGACGTGGTCGGGTGGAAGCTAGGGGAATAAATCTTTGGGCACCTAGTGGTCATG

1 CATGACCACTAGGAGCATCTTTGGCGAGAACGGGAGAATCGGCGGCACTGGTGTCTCCTAGGGGAATAAATCTTTGGGCACCTAGTGGTCATG

1 CATGACCACTAGGAGCATCTTTGGCGAGGAGGACTGCAGGTTGGAACTGGAACTTAACTAGGGGAATAAATCTTTGGGCACCTAGTGGTCATG

1 CATGACCACTAGGAGCATCTTTGGCGAAGTGCGGTGGGCATGTGGGTGGTAGCGAGGCTAGGGGAATAAATCTTTGGGCACCTAGTGGTCATG

1 CATGACCACTAGGAGCATCTTTGGCGATCCTCGTCAGATGGTGAAGCAGACGCTTGGCTAGGGGAATAAATCTTTGGGCACCTAGTGGTCATG

1 CATGACCACTAGGAGCATCTTTGGCGAAAGTCGGTAGAAAATGGTGGGGTGCTGTCCCTAGGGGAATAAATCTTTGGGCACCTAGTGGTCATG

1 CATGACCACTAGGAGCATCTTTGGCGACCCATGGACGTGGAACGGCTATGCGGTGGACTAGGGGAATAAATCTTTGGGCACCTAGTGGTCATG

1 CATGACCACTAGGAGCATCTTTGGCGACCGTAGGAAGACAAAAGTGGCCTTGCTAGCCTAGGGGAATAAATCTTTGGGCACCTAGTGGTCATG

1 CATGACCACTAGGAGCATCTTTGGCGAAGGGTGTAGGACTTCAAGTGGATCTTATAGCTAGGGGAATAAATCTTTGGGCACCTAGTGGTCATG

1 CATGACCACTAGGAGCATCTTTGGCGAGGGCTGTTAGTAGCTGTGACATTAACGGATCTAGGGGAATAAATCTTTGGGCACCTAGTGGTCATG

1 CATGACCACTAGGAGCATCTTTGGCGACGGCGTGGGGCCAATTGTGCGGCGGATCCCCTAGGGGAATAAATCTTTGGGCACCTAGTGGTCATG

1 CATGACCACTAGGAGCATCTTTGGCGACGACGGGGTTGTGGCTTTGTGGGGAGGCGACTAGGGGAATAAATCTTTGGGCACCTAGTGGTCATG

1 CATGACCACTAGGAGCATCTTTGGCGATCCGGGGGTCCGACCGGACGGCGCTCTGGGCTAGGGGAATAAATCTTTGGGCACCTAGTGGTCATG

1 CATGACCACTAGGAGCATCTTTGGCGATCCGGGGGCCACTGAGGCGACGCTAACGGGCTAGGGGAATAAATCTTTGGGCACCTAGTGGTCATG

1 CATGACCACTAGGAGCATCTTTGGCGAGATCGGGAGAATCGGTGACATTGGTGTTTCCTAGGGGAATAAATCTTTGGGCACCTAGTGGTCATG

1 CATGACCACTAGGAGCATCTTTGGCGACAGGGGATGCCGATGCGCTGGACGGCCGAGCTAGGGGAATAAATCTTTGGGCACCTAGTGGTCATG

1 CATGACCACTAGGAGCATCTTTGGCGAGATCGGGAGAAGCGGTGGCATTGGTGTTTCCTAGGGGAATAAATCTTTGGGCACCTAGTGGTCATG

1 CATGACCACTAGGAGCATCTTTGGCGAGTGGGGGGCGATGGCCGGTAGTGGGTGAGACTAGGGGAATAAATCTTTGGGCACCTAGTGGTCATG

1 CATGACCACTAGGAGCATCTTTGGCGATCCGGGTGTCCGGGTGGACGACGCGACGGGCTAGGGGAATAAATCTTTGGGCACCTAGTGGTCATG

1 CATGACCACTAGGAGCATCTTTGGCGACTAAGTGGCCAGCGCGGTAAATGGGGTAGACTAGGGGAATAAATCTTTGGGCACCTAGTGGTCATG

1 CATGACCACTAGGAGCATCTTTGGCGAGAACGCGAGAATCGGTGCCATTGGTGTCTCCTAGGGGAATAAATCTTTGGGCACCTAGTGGTCATG

1 CATGACCACTAGGAGCATCTTTGGCGAACGGCAGGTGTTGCGGTGGTATGTGAATACCTAGGGGAATAAATCTTTGGGCACCTAGTGGTCATG

1 CATGACCACTAGGAGCATCTTTGGCGAATGGCCGGCACGGCCTCCCAGTCCTCGGTACTAGGGGAATAAATCTTTGGGCACCTAGTGGTCATG

1 CATGACCACTAGGAGCATCTTTGGCGAATAGGCCTTGGCTGCGGGTGGGTGCGCACCCTAGGGGAATAAATCTTTGGGCACCTAGTGGTCATG

1 CATGACCACTAGGAGCATCTTTGGCGAGATCGGGAGAATAGGTGGCATTGGAGTCTCCTAGGGGAATAAATCTTTGGGCACCTAGTGGTCATG

1 CATGACCACTAGGAGCATCTTTGGCGACGGTGTGGGGAGCTTGTTTCGGCGGTGCTCCTAGGGGAATAAATCTTTGGGCACCTAGTGGTCATG

1 CATGACCACTAGGAGCATCTTTGGCGAGGGAGGGCGCCAGCAGCGGTGTGAATGCGCCTAGGGGAATAAATCTTTGGGCACCTAGTGGTCATG

1 CATGACCACTAGGAGCATCTTTGGCGAGAATACTCTGGAATCGCGGACCAGTTGCTGCTAGGGGAATAAATCTTTGGGCACCTAGTGGTCATG

1 CATGACCACTAGGAGCATCTTTGGCGACCCCTGGCTGGTAGTGCGTACTCTGTCGAACTAGGGGAATAAATCTTTGGGCACCTAGTGGTCATG

1 CATGACCACTAGGAGCATCTTTGGCGAGATCGGGAGACTCGGTGGGAGTGGTGTCTCCTAGGGGAATAAATCTTTGGGCACCTAGTGGTCATG

1 CATGACCACTAGGAGCATCTTTGGCGATCGCATAGCGGACGGATTCCGAGGAGGGGCCTAGGGGAATAAATCTTTGGGCACCTAGTGGTCATG

1 CATGACCACTAGGAGCATCTTTGGCGAGAAGACTCTGGATTCTGGGACAAGTTGCTGCTAGGGGAATAAATCTTTGGGCACCTAGTGGTCATG

1 CATGACCACTAGGAGCATCTTTGGCGATCAGGCCTTGGCTGCGGGTGGGTGCGCACCCTAGGGGAATAAATCTTTGGGCACCTAGTGGTCATG

1 CATGACCACTAGGAGCATCTTTGGCGAGATCGGGTGAATCGGTGGCATTGGTTTCTCCTAGGGGAATAAATCTTTGGGCACCTAGTGGTCATG

1 CATGACCACTAGGAGCATCTTTGGCGAGGGGTGGCTGAGGGCGGTGGGTAGGTCGCGCTAGGGGAATAAATCTTTGGGCACCTAGTGGTCATG

1 CATGACCACTAGGAGCATCTTTGGCGATTAGCGTCTTGCGAGCGGGTGGGTCGCTCCCTAGGGGAATAAATCTTTGGGCACCTAGTGGTCATG

1 CATGACCACTAGGAGCATCTTTGGCGAGACTAGTTATGACATGACCTGTGTGTTTATCTAGGGGAATAAATCTTTGGGCACCTAGTGGTCATG

1 CATGACCACTAGGAGCATCTTTGGCGACGGCATGGGGCCATTTGTGCGGCGGATCCCCTAGGGGAATAAATCTTTGGGCACCTAGTGGTCATG

1 CATGACCACTAGGAGCATCTTTGGCGACAGTGGTTGGCCTCAGCTATGCTCCGTCGACTAGGGGAATAAATCTTTGGGCACCTAGTGGTCATG

1 CATGACCACTAGGAGCATCTTTGGCGAACAGCAGGTGTTGCGGTGGTCTGTGGATCCCTAGGGGAATAAATCTTTGGGCACCTAGTGGTCATG

1 CATGACCACTAGGAGCATCTTTGGCGAGCTTTGCTGGGGAGGTTGCTACACCAGTTCCTAGGGGAATAAATCTTTGGGCACCTAGTGGTCATG

1 CATGACCACTAGGAGCATCTTTGGCGAGTGGCTCTGCAGGTTTCAGAGTCCTCGACGCTAGGGGAATAAATCTTTGGGCACCTAGTGGTCATG

1 CATGACCACTAGGAGCATCTTTGGCGACGCGGTGTCATCGTTTGGGGCAAGTTGCTGCTAGGGGAATAAATCTTTGGGCACCTAGTGGTCATG

1 CATGACCACTAGGAGCATCTTTGGCGAAAGTCGCTAGAACAGGGTGGGGTGCTGTCCCTAGGGGAATAAATCTTTGGGCACCTAGTGGTCATG

1 CATGACCACTAGGAGCATCTTTGGCGAGATCGGGAGAATCGCTGGCATTGGTGTATCCTAGGGGAATAAATCTTTGGGCACCTAGTGGTCATG

1 CATGACCACTAGGAGCATCTTTGGCGAGATCGCGAGAATCGCTGGCATTGGTTTCTCCTAGGGGAATAAATCTTTGGGCACCTAGTGGTCATG

1 CATGACCACTAGGAGCATCTTTGGCGAGACCGGGAGAATCGGTGGCATAGGTGTCTCCTAGGGGAATAAATCTTTGGGCACCTAGTGGTCATG

1 CATGACCACTAGGAGCATCTTTGGCGAGATCGGGAGTAACGGTGGCATTGGTGTCTCCTAGGGGAATAAATCTTTGGGCACCTAGTGGTCATG

1 CATGACCACTAGGAGCATCTTTGGCGACCGCATAGCGAAGGATAAGATGTCCCGGGCCTAGGGGAATAAATCTTTGGGCACCTAGTGGTCATG

1 CATGACCACTAGGAGCATCTTTGGCGACTCGTGAATCGGCTGGCCGGATGTCCTGTGCTAGGGGAATAAATCTTTGGGCACCTAGTGGTCATG

1 CATGACCACTAGGAGCATCTTTGGCGAGATCGGGGGAATCAGCGGCATTGGTGTCTCCTAGGGGAATAAATCTTTGGGCACCTAGTGGTCATG

1 CATGACCACTAGGAGCATCTTTGGCGACGTGGGGAGGTGAGGTTGTGGCCAGGCAGACTAGGGGAATAAATCTTTGGGCACCTAGTGGTCATG

1 CATGACCACTAGGAGCATCTTTGGCGACCTGTAGGTATGTGCAAACGGCCTTGCTAGCTAGGGGAATAAATCTTTGGGCACCTAGTGGTCATG

1 CATGACCACTAGGAGCATCTTTGGCGAGGAGTGGCTGAGGGCGGTGGGTAGGTCGAGCTAGGGGAATAAATCTTTGGGCACCTAGTGGTCATG

1 CATGACCACTAGGAGCATCTTTGGCGATATCGGGAGTATCCGTTGCTGTGGTGAAACCTAGGGGAATAAATCTTTGGGCACCTAGTGGTCATG

1 CATGACCACTAGGAGCATCTTTGGCGACCGGCAGGTGTTGCGGTGGTTTGTGAATCCCTAGGGGAATAAATCTTTGGGCACCTAGTGGTCATG

1 CATGACCACTAGGAGCATCTTTGGCGACCGAGTGGGCTGGGAGGAAGTATCTCGGAGCTAGGGGAATAAATCTTTGGGCACCTAGTGGTCATG

1 CATGACCACTAGGAGCATCTTTGGCGAGAGAATCGGTAGGTAGAGTGCTCCAGCCAGCTAGGGGAATAAATCTTTGGGCACCTAGTGGTCATG

1 CATGACCACTAGGAGCATCTTTGGCGAGAAGACACTGTATTCGGGGACCAGTTGCTGCTAGGGGAATAAATCTTTGGGCACCTAGTGGTCATG

1 CATGACCACTAGGAGCATCTTTGGCGAACGTCAGGTGTTGCGGTGGTCTGTCAATCCCTAGGGGAATAAATCTTTGGGCACCTAGTGGTCATG

1 CATGACCACTAGGAGCATCTTTGGCGAGGGCGGGCACCGGCAGCGGTGTGAATGCGACTAGGGGAATAAATCTTTGGGCACCTAGTGGTCATG

1 CATGACCACTAGGAGCATCTTTGGCGAGATCGGGCGAATCGGTGGCCTTGGTGTCTCCTAGGGGAATAAATCTTTGGGCACCTAGTGGTCATG

1 CATGACCACTAGGAGCATCTTTGGCGAGGGGTAGGGGGCCGCTCGTGCCTGCGTGCGCTAGGGGAATAAATCTTTGGGCACCTAGTGGTCATG

1 CATGACCACTAGGAGCATCTTTGGCGAGAGTGGTCGTTAGAGTAGAGTGGGTCGAGACTAGGGGAATAAATCTTTGGGCACCTAGTGGTCATG

1 CATGACCACTAGGAGCATCTTTGGCGAACGACAGGTGTTGCGGTGGTCTGTATATCCCTAGGGGAATAAATCTTTGGGCACCTAGTGGTCATG

1 CATGACCACTAGGAGCATCTTTGGCGAACAGCAGGTGATGCGGTGGTCTGTGAATCCCTAGGGGAATAAATCTTTGGGCACCTAGTGGTCATG

1 CATGACCACTAGGAGCATCTTTGGCGAACCCCGACAGCGCTCCGAACGGGCGCATGCCTAGGGGAATAAATCTTTGGGCACCTAGTGGTCATG

1 CATGACCACTAGGAGCATCTTTGGCGAGAGAATCTGTATGCAGAGTTCTCAATCCAGCTAGGGGAATAAATCTTTGGGCACCTAGTGGTCATG

1 CATGACCACTAGGAGCATCTTTGGCGAGGTCTACCCGTCTGCCGACAGGCTTTAGTGCTAGGGGAATAAATCTTTGGGCACCTAGTGGTCATG

1 CATGACCACTAGGAGCATCTTTGGCGAGATCGGGAGAATCGGTGGCATCGCTGTCTCCTAGGGGAATAAATCTTTGGGCACCTAGTGGTCATG

1 CATGACCACTAGGAGCATCTTTGGCGAATGGCCGGAACGGCCTTCTAGTCCTTGGTACTAGGGGAATAAATCTTTGGGCACCTAGTGGTCATG

1 CATGACCACTAGGAGCATCTTTGGCGAACGACGTGGTCACAGATGGAGGAAGTGGAGCTAGGGGAATAAATCTTTGGGCACCTAGTGGTCATG

1 CATGACCACTAGGAGCATCTTTGGCGACGGTCCCTCGGAGGTACGATCTCGGGTGTACTAGGGGAATAAATCTTTGGGCACCTAGTGGTCATG

1 CATGACCACTAGGAGCATCTTTGGCGAGTTCGGGAGCATCGGTGGCATTGGTGTCTGCTAGGGGAATAAATCTTTGGGCACCTAGTGGTCATG

1 CATGACCACTAGGAGCATCTTTGGCGACGTATGTGGGCTTGTGCGATGGCTGGGCGCCTAGGGGAATAAATCTTTGGGCACCTAGTGGTCATG

1 CATGACCACTAGGAGCATCTTTGGCGATCCGGGGGTTACAAGTGACGGCGCATAGGGCTAGGGGAATAAATCTTTGGGCACCTAGTGGTCATG

1 CATGACCACTAGGAGCATCTTTGGCGAACGGAAGGTGTTGCGGTGGTCAGTGAATCCCTAGGGGAATAAATCTTTGGGCACCTAGTGGTCATG

1 CATGACCACTAGGAGCATCTTTGGCGAACGGCAGGTGTTGCGGTGGTTTGAGAATCCCTAGGGGAATAAATCTTTGGGCACCTAGTGGTCATG

1 CATGACCACTAGGAGCATCTTTGGCGACGGTGGGTGACGAGAGTTGGAAGGAGACCTCTAGGGGAATAAATCTTTGGGCACCTAGTGGTCATG

1 CATGACCACTAGGAGCATCTTTGGCGAGATCGGGAGAGTCGGTTGCATTGGTGTCTACTAGGGGAATAAATCTTTGGGCACCTAGTGGTCATG

1 CATGACCACTAGGAGCATCTTTGGCGATCAGCGTCTTGCGAGCGGGGGGTTCGCTCCCTAGGGGAATAAATCTTTGGGCACCTAGTGGTCATG

1 CATGACCACTAGGAGCATCTTTGGCGAGGTTGCAGCTCGCGCGTAGGGTCGGCAGTGCTAGGGGAATAAATCTTTGGGCACCTAGTGGTCATG

1 CATGACCACTAGGAGCATCTTTGGCGAGATCGGGAGAAACGGTGGCATTGGTGTCTTCTAGGGGAATAAATCTTTGGGCACCTAGTGGTCATG

1 CATGACCACTAGGAGCATCTTTGGCGAGAGCCCGGCAATGCCTTCGAGTCCTGTCTCCTAGGGGAATAAATCTTTGGGCACCTAGTGGTCATG

1 CATGACCACTAGGAGCATCTTTGGCGAGATCAGGAGTATCGGTGGCATTGGTGTCTCCTAGGGGAATAAATCTTTGGGCACCTAGTGGTCATG

1 CATGACCACTAGGAGCATCTTTGGCGAAATCGGGAGAATCGGTGTCATTGGTGTCTCCTAGGGGAATAAATCTTTGGGCACCTAGTGGTCATG

1 CATGACCACTAGGAGCATCTTTGGCGACCCAAAATCCGAAGGATAGATTTGGGTGGACTAGGGGAATAAATCTTTGGGCACCTAGTGGTCATG

1 CATGACCACTAGGAGCATCTTTGGCGACCCTCGTCAGATGGTGAAGCAGACGTTTGGCTAGGGGAATAAATCTTTGGGCACCTAGTGGTCATG

1 CATGACCACTAGGAGCATCTTTGGCGATCGGCAGCGACGACGCATGTATGCAGGTTGCTAGGGGAATAAATCTTTGGGCACCTAGTGGTCATG

1 CATGACCACTAGGAGCATCTTTGGCGACCAGGTGTGGTGCCACACCGCGACGTAGTGCTAGGGGAATAAATCTTTGGGCACCTAGTGGTCATG

1 CATGACCACTAGGAGCATCTTTGGCGAGAATACTCTGGATTCGGGGACCAGTAGCTGCTAGGGGAATAAATCTTTGGGCACCTAGTGGTCATG

1 CATGACCACTAGGAGCATCTTTGGCGATTCTGCAGCAAGCTATTACCCTCTAACATTCTAGGGGAATAAATCTTTGGGCACCTAGTGGTCATG

1 CATGACCACTAGGAGCATCTTTGGCGAATGGGGGGCGACGGCCGGTAGGGGGTGAGACTAGGGGAATAAATCTTTGGGCACCTAGTGGTCATG

1 CATGACCACTAGGAGCATCTTTGGCGAACGGCAGGTGTTGCTGTGGTCTGTGAATACCTAGGGGAATAAATCTTTGGGCACCTAGTGGTCATG

1 CATGACCACTAGGAGCATCTTTGGCGAGATCGGGAGAACCGGTGTCATTGGTGTCTCCTAGGGGAATAAATCTTTGGGCACCTAGTGGTCATG

1 CATGACCACTAGGAGCATCTTTGGCGAGATCGGGAGAATCGGTGGCATTGTCGTCTCCTAGGGGAATAAATCTTTGGGCACCTAGTGGTCATG

1 CATGACCACTAGGAGCATCTTTGGCGAGTGGCCATCAGTACTGTCATACATCTTACACTAGGGGAATAAATCTTTGGGCACCTAGTGGTCATG

1 CATGACCACTAGGAGCATCTTTGGCGACGTAGTGGGCTGGCAGGGTGTACTTGTTGCCTAGGGGAATAAATCTTTGGGCACCTAGTGGTCATG

1 CATGACCACTAGGAGCATCTTTGGCGATGGGTGCTGACGGGAGGTTTAACTCCTACACTAGGGGAATAAATCTTTGGGCACCTAGTGGTCATG

1 CATGACCACTAGGAGCATCTTTGGCGAGGGGTAGGGGGCTGCTCGCGCCTGCGTGCGCTAGGGGAATAAATCTTTGGGCACCTAGTGGTCATG

1 CATGACCACTAGGAGCATCTTTGGCGACACGAATGCAAGTGTTTGTGATTGTCCGCGCTAGGGGAATAAATCTTTGGGCACCTAGTGGTCATG

1 CATGACCACTAGGAGCATCTTTGGCGAGATCGGGAGAATCCGTGGCATTGGTGTAACCTAGGGGAATAAATCTTTGGGCACCTAGTGGTCATG

1 CATGACCACTAGGAGCATCTTTGGCGAGATCGGGAGAATCGTTGGCAGTGGTGTCTCCTAGGGGAATAAATCTTTGGGCACCTAGTGGTCATG

1 CATGACCACTAGGAGCATCTTTGGCGAGAAGACTCTGGATTCAGGGACCAGTTGCTCCTAGGGGAATAAATCTTTGGGCACCTAGTGGTCATG

1 CATGACCACTAGGAGCATCTTTGGCGACCTGCAGGTGTTCCGGTGGTCTGTGAATCCCTAGGGGAATAAATCTTTGGGCACCTAGTGGTCATG

1 CATGACCACTAGGAGCATCTTTGGCGATGGGTGCTGACGGCCGCCGCTCCGGCTACACTAGGGGAATAAATCTTTGGGCACCTAGTGGTCATG

1 CATGACCACTAGGAGCATCTTTGGCGAACGGCAGGTGTTGCGGTGGTTTGTGCATCCCTAGGGGAATAAATCTTTGGGCACCTAGTGGTCATG

1 CATGACCACTAGGAGCATCTTTGGCGAGAAGACTCTGGAGTTGGGGACCAGTTGCTGCTAGGGGAATAAATCTTTGGGCACCTAGTGGTCATG

1 CATGACCACTAGGAGCATCTTTGGCGAAGACGGCACAGTCGATCGGGTTAGAGGAAGCTAGGGGAATAAATCTTTGGGCACCTAGTGGTCATG

1 CATGACCACTAGGAGCATCTTTGGCGACCAGTGATAACTCGTAACGCGTCGTAGTGCCTAGGGGAATAAATCTTTGGGCACCTAGTGGTCATG

1 CATGACCACTAGGAGCATCTTTGGCGAGAACGGGAGGATCTGTGGCAGAGGTGTCTCCTAGGGGAATAAATCTTTGGGCACCTAGTGGTCATG

1 CATGACCACTAGGAGCATCTTTGGCGATCCAGAGTCAATGTGTTACGGTCCGGGAGTCTAGGGGAATAAATCTTTGGGCACCTAGTGGTCATG

1 CATGACCACTAGGAGCATCTTTGGCGATAGTCAGTAGAACAGGGTGGGGTGCTGTCCCTAGGGGAATAAATCTTTGGGCACCTAGTGGTCATG

1 CATGACCACTAGGAGCATCTTTGGCGAGATCGGGAGAACCGGTGGCATTGGTTTCTCCTAGGGGAATAAATCTTTGGGCACCTAGTGGTCATG

1 CATGACCACTAGGAGCATCTTTGGCGACGGAGTGGGTGTGCGAGAGGATACCGTCAGCTAGGGGAATAAATCTTTGGGCACCTAGTGGTCATG

1 CATGACCACTAGGAGCATCTTTGGCGAGATCGTGAGAATCGGTGACATTGGTGTCTCCTAGGGGAATAAATCTTTGGGCACCTAGTGGTCATG

1 CATGACCACTAGGAGCATCTTTGGCGACAACGGAGTGTCTGACACCTTTGATGGAGACTAGGGGAATAAATCTTTGGGCACCTAGTGGTCATG

1 CATGACCACTAGGAGCATCTTTGGCGAATAGCAGGTGTTGCGGTGGTCTGTGAATCCCTAGGGGAATAAATCTTTGGGCACCTAGTGGTCATG

1 CATGACCACTAGGAGCATCTTTGGCGAGGGAGGGCGCCGGCAGCGGTATGAATGCGCCTAGGGGAATAAATCTTTGGGCACCTAGTGGTCATG

1 CATGACCACTAGGAGCATCTTTGGCGAAGGTTTCTACTTGTAGGAGTCGGTTTACCTCTAGGGGAATAAATCTTTGGGCACCTAGTGGTCATG

1 CATGACCACTAGGAGCATCTTTGGCGATCCTCGTCAAATGGTGAAGCAGACGTTTGGCTAGGGGAATAAATCTTTGGGCACCTAGTGGTCATG

1 CATGACCACTAGGAGCATCTTTGGCGAGCCGGCCGCTATAGTTGGCGTGTCTGGGTCCTAGGGGAATAAATCTTTGGGCACCTAGTGGTCATG

1 CATGACCACTAGGAGCATCTTTGGCGAGTGGTCATGTCGAGACACGTTACTTTTCTGCTAGGGGAATAAATCTTTGGGCACCTAGTGGTCATG

1 CATGACCACTAGGAGCATCTTTGGCGAACGGCAGGTGTTGCGGTGGCCTGTTAATCCCTAGGGGAATAAATCTTTGGGCACCTAGTGGTCATG

1 CATGACCACTAGGAGCATCTTTGGCGAGAGCGGGAGAATCGGCGGCCTTGGTGTCTCCTAGGGGAATAAATCTTTGGGCACCTAGTGGTCATG

1 CATGACCACTAGGAGCATCTTTGGCGAGAAGACTCTCGATTCGGGGACCTGTTGCTGCTAGGGGAATAAATCTTTGGGCACCTAGTGGTCATG

1 CATGACCACTAGGAGCATCTTTGGCGATAGTCGGTAGAACAGGGTGGCGTGCTGTCCCTAGGGGAATAAATCTTTGGGCACCTAGTGGTCATG

1 CATGACCACTAGGAGCATCTTTGGCGACGAGTGGCAGAGCAACGTATCTTTGTACGCCTAGGGGAATAAATCTTTGGGCACCTAGTGGTCATG

1 CATGACCACTAGGAGCATCTTTGGCGACAAGCAGTCTGCGCACTGCGTGACATAGTGCTAGGGGAATAAATCTTTGGGCACCTAGTGGTCATG

1 CATGACCACTAGGAGCATCTTTGGCGACGATGTATTTGGCGCACGATGTTCCAGCGGCTAGGGGAATAAATCTTTGGGCACCTAGTGGTCATG

1 CATGACCACTAGGAGCATCTTTGGCGAGATCGGCAGAATCGGAGGCATTGGTGTCTCCTAGGGGAATAAATCTTTGGGCACCTAGTGGTCATG

1 CATGACCACTAGGAGCATCTTTGGCGAGACATAGTGCCGATATAAGAGCGACTAGCCCTAGGGGAATAAATCTTTGGGCACCTAGTGGTCATG

1 CATGACCACTAGGAGCATCTTTGGCGAGCGGCAGGTGTTACGGTGGTCTGTGAATCCCTAGGGGAATAAATCTTTGGGCACCTAGTGGTCATG

1 CATGACCACTAGGAGCATCTTTGGCGAAAGTCGGTAGAACAGGCTGGGGTGCTGTCCCTAGGGGAATAAATCTTTGGGCACCTAGTGGTCATG

1 CATGACCACTAGGAGCATCTTTGGCGACTCGTGAAGAACCCATTGTGCGACCCGTGACTAGGGGAATAAATCTTTGGGCACCTAGTGGTCATG

1 CATGACCACTAGGAGCATCTTTGGCGAGAACTTGAGAATCGGAGGCATTGGTGTCTCCTAGGGGAATAAATCTTTGGGCACCTAGTGGTCATG

1 CATGACCACTAGGAGCATCTTTGGCGAAAGTCGGTAGAACAGGTTGTGGTGCTGTCCCTAGGGGAATAAATCTTTGGGCACCTAGTGGTCATG

1 CATGACCACTAGGAGCATCTTTGGCGATGCTGCAGCGGCTGGTAGTCCTTTGCTGGCCTAGGGGAATAAATCTTTGGGCACCTAGTGGTCATG

1 CATGACCACTAGGAGCATCTTTGGCGATCTTACGTGGATCATGCCTGTGTCTTTTATCTAGGGGAATAAATCTTTGGGCACCTAGTGGTCATG

1 CATGACCACTAGGAGCATCTTTGGCGAGATTGGGAGAATCGGTGGCATTGGTGTCTACTAGGGGAATAAATCTTTGGGCACCTAGTGGTCATG

1 CATGACCACTAGGAGCATCTTTGGCGACGACAGTGGTGAGTGGCCTCCGGGAGGAGACTAGGGGAATAAATCTTTGGGCACCTAGTGGTCATG

1 CATGACCACTAGGAGCATCTTTGGCGATCCCGTCTCTGTTTCAAGATTAGTGGCATTCTAGGGGAATAAATCTTTGGGCACCTAGTGGTCATG

1 CATGACCACTAGGAGCATCTTTGGCGATCCGGGGGCCGCAGCCGGCGACGCAATGGGCTAGGGGAATAAATCTTTGGGCACCTAGTGGTCATG

1 CATGACCACTAGGAGCATCTTTGGCGACCCCGATGGCGTGTGGGGACTGAGATGCGACTAGGGGAATAAATCTTTGGGCACCTAGTGGTCATG

1 CATGACCACTAGGAGCATCTTTGGCGATATACGGTCGCGTCCGGAGAGGGACGTTCGCTAGGGGAATAAATCTTTGGGCACCTAGTGGTCATG

1 CATGACCACTAGGAGCATCTTTGGCGAGAACCCGTGGGAATTAAGTCTTCGCCGTACCTAGGGGAATAAATCTTTGGGCACCTAGTGGTCATG

1 CATGACCACTAGGAGCATCTTTGGCGAGTGGCCGGCACGGCCTTCTAGTCCTCGGTACTAGGGGAATAAATCTTTGGGCACCTAGTGGTCATG

1 CATGACCACTAGGAGCATCTTTGGCGAAGGGTGTAGGACTTCAAGTGGATCTCCTAGCTAGGGGAATAAATCTTTGGGCACCTAGTGGTCATG

1 CATGACCACTAGGAGCATCTTTGGCGAGAACGGGAAAATCGGTGGCATTGGTGTCTCCTAGGGGAATAAATCTTTGGGCACCTAGTGGTCATG

1 CATGACCACTAGGAGCATCTTTGGCGACTCGGGTGGACACGGTTCACATGTATTGTCCTAGGGGAATAAATCTTTGGGCACCTAGTGGTCATG

1 CATGACCACTAGGAGCATCTTTGGCGATATTGTATGCTGACCGGGTGATCCTTCGCACTAGGGGAATAAATCTTTGGGCACCTAGTGGTCATG

1 CATGACCACTAGGAGCATCTTTGGCGAACGGCAGGTGTTGCGGTAGTCTGTGAAACCCTAGGGGAATAAATCTTTGGGCACCTAGTGGTCATG

1 CATGACCACTAGGAGCATCTTTGGCGAGATCGGCAGAATCTGTGGCATTGGTGTCTCCTAGGGGAATAAATCTTTGGGCACCTAGTGGTCATG

1 CATGACCACTAGGAGCATCTTTGGCGAAGGTGGCCGAGATCCGATTGGTAGTGTTGCCTAGGGGAATAAATCTTTGGGCACCTAGTGGTCATG

1 CATGACCACTAGGAGCATCTTTGGCGACTCCAGTTCATCTGTCGGGCGGTTTAAGGACTAGGGGAATAAATCTTTGGGCACCTAGTGGTCATG

1 CATGACCACTAGGAGCATCTTTGGCGACAGGGGATGCCGATGCGCTGAACGACCGAGCTAGGGGAATAAATCTTTGGGCACCTAGTGGTCATG

1 CATGACCACTAGGAGCATCTTTGGCGAGAACGGGATACTAGGTGGCATTCGTGTCTCCTAGGGGAATAAATCTTTGGGCACCTAGTGGTCATG

1 CATGACCACTAGGAGCATCTTTGGCGAAGAGGACCCTTGAGGCTGCTCTTATAGACTCTAGGGGAATAAATCTTTGGGCACCTAGTGGTCATG

1 CATGACCACTAGGAGCATCTTTGGCGACAATGTAACAAAGCGAAGGTTTATACGTCGCTAGGGGAATAAATCTTTGGGCACCTAGTGGTCATG

1 CATGACCACTAGGAGCATCTTTGGCGACGGCGTGGGGCTATTGATGCGGAGGCACTCCTAGGGGAATAAATCTTTGGGCACCTAGTGGTCATG

1 CATGACCACTAGGAGCATCTTTGGCGAAATCGGGAGAATCGGTGGCATTGGTGTTTCCTAGGGGAATAAATCTTTGGGCACCTAGTGGTCATG

1 CATGACCACTAGGAGCATCTTTGGCGATGCACCAGGGTGCTTATGGTCGCTAAGCAGCTAGGGGAATAAATCTTTGGGCACCTAGTGGTCATG

1 CATGACCACTAGGAGCATCTTTGGCGATTCCGGGGGTGCAGTGCACGACGCAAAGGGCTAGGGGAATAAATCTTTGGGCACCTAGTGGTCATG

1 CATGACCACTAGGAGCATCTTTGGCGAAATCGGGAGAATCGGTGGCATCGGTGTCTCCTAGGGGAATAAATCTTTGGGCACCTAGTGGTCATG

1 CATGACCACTAGGAGCATCTTTGGCGAACGGCACGTGTTGCGGTGGTCTATGAATCCCTAGGGGAATAAATCTTTGGGCACCTAGTGGTCATG

1 CATGACCACTAGGAGCATCTTTGGCGAGATCGGGAGAATATGTGGCAGTGGTGTCTCCTAGGGGAATAAATCTTTGGGCACCTAGTGGTCATG

1 CATGACCACTAGGAGCATCTTTGGCGAGAAGACACCGGATTCGGGGACCAGTTGCTGCTAGGGGAATAAATCTTTGGGCACCTAGTGGTCATG

1 CATGACCACTAGGAGCATCTTTGGCGAACGGCAGGTATTGCGGTGGTCTGTGAACCCCTAGGGGAATAAATCTTTGGGCACCTAGTGGTCATG

1 CATGACCACTAGGAGCATCTTTGGCGACCCGAGTGGTCAGACGAGACGACGGGTGGACTAGGGGAATAAATCTTTGGGCACCTAGTGGTCATG

1 CATGACCACTAGGAGCATCTTTGGCGAGCGGCGGGTGTTGCGGTGGTCTGTGAATCCCTAGGGGAATAAATCTTTGGGCACCTAGTGGTCATG

1 CATGACCACTAGGAGCATCTTTGGCGAGGGCTGGGTAGGCAAACGGCTATACGTGGCCTAGGGGAATAAATCTTTGGGCACCTAGTGGTCATG

1 CATGACCACTAGGAGCATCTTTGGCGAGATTGGGAGAATCGGTGGCAATGGTGTCTCCTAGGGGAATAAATCTTTGGGCACCTAGTGGTCATG

1 CATGACCACTAGGAGCATCTTTGGCGAACGGCAGGGGTTGAGGGGGTCTGTGAATCCCTAGGGGAATAAATCTTTGGGCACCTAGTGGTCATG

1 CATGACCACTAGGAGCATCTTTGGCGACTCGTGGTTCTTACCCCCGTAGAGGGTAGTCTAGGGGAATAAATCTTTGGGCACCTAGTGGTCATG

1 CATGACCACTAGGAGCATCTTTGGCGAAGACGACACAGTCGATCGGGTTAGATGTAGCTAGGGGAATAAATCTTTGGGCACCTAGTGGTCATG

1 CATGACCACTAGGAGCATCTTTGGCGAACGGCAGGTGTTGCGGTGTTCTGTGAAACCCTAGGGGAATAAATCTTTGGGCACCTAGTGGTCATG

1 CATGACCACTAGGAGCATCTTTGGCGAAAGCAGAAGGTGTGAGTGTAATGCGGCCTACTAGGGGAATAAATCTTTGGGCACCTAGTGGTCATG

1 CATGACCACTAGGAGCATCTTTGGCGAACGGCAGGTGTTGCGGTGGTCTCTGAATCACTAGGGGAATAAATCTTTGGGCACCTAGTGGTCATG

1 CATGACCACTAGGAGCATCTTTGGCGAACGGCAGGAGATGCGGTGGTCTGTTAATCCCTAGGGGAATAAATCTTTGGGCACCTAGTGGTCATG

1 CATGACCACTAGGAGCATCTTTGGCGACGTGGTGTGTTGCCTAAGTGGCTGAGTTGACTAGGGGAATAAATCTTTGGGCACCTAGTGGTCATG

1 CATGACCACTAGGAGCATCTTTGGCGAGATCGGGAGAATCGGTGGCATTGGTGTAACCTAGGGGAATAAATCTTTGGGCACCTAGTGGTCATG

1 CATGACCACTAGGAGCATCTTTGGCGAGATCGGGAGAATCGGTAGCCTTGGTGTCTCCTAGGGGAATAAATCTTTGGGCACCTAGTGGTCATG

1 CATGACCACTAGGAGCATCTTTGGCGATCCAGCTGCGCGAGCGACCTGGAGTGGAGCCTAGGGGAATAAATCTTTGGGCACCTAGTGGTCATG

1 CATGACCACTAGGAGCATCTTTGGCGAACGTGCGATAGCAGGTTTACGAAGTGGAGCCTAGGGGAATAAATCTTTGGGCACCTAGTGGTCATG

1 CATGACCACTAGGAGCATCTTTGGCGACATCGGGAGAATCGGTGGCATTGGCGTCTCCTAGGGGAATAAATCTTTGGGCACCTAGTGGTCATG

1 CATGACCACTAGGAGCATCTTTGGCGACAAGAGGCCGTGTAAGGGTCTTGAAGTGGACTAGGGGAATAAATCTTTGGGCACCTAGTGGTCATG

1 CATGACCACTAGGAGCATCTTTGGCGAGAATACTCTGGATTCGGGGACAAGTTGCTGCTAGGGGAATAAATCTTTGGGCACCTAGTGGTCATG

1 CATGACCACTAGGAGCATCTTTGGCGAGCCTAGCTTTCTAGATTTCTTCCATATCGTCTAGGGGAATAAATCTTTGGGCACCTAGTGGTCATG

1 CATGACCACTAGGAGCATCTTTGGCGACGAAACGCAGATCCGACAGACTGGAGGCGCCTAGGGGAATAAATCTTTGGGCACCTAGTGGTCATG

1 CATGACCACTAGGAGCATCTTTGGCGAGGTAGGGCGCCGGCAGCGGTGTGAATGCGACTAGGGGAATAAATCTTTGGGCACCTAGTGGTCATG

1 CATGACCACTAGGAGCATCTTTGGCGACTCGGAGGGTTTGTAAAAGACGGTGTGTGCCTAGGGGAATAAATCTTTGGGCACCTAGTGGTCATG

1 CATGACCACTAGGAGCATCTTTGGCGAGTAGTGGCTGAGCAGACGCCTACGGATTGCCTAGGGGAATAAATCTTTGGGCACCTAGTGGTCATG

1 CATGACCACTAGGAGCATCTTTGGCGAGCTCGGGAGAATCGGTGGCAGTGGTGTCTCCTAGGGGAATAAATCTTTGGGCACCTAGTGGTCATG

1 CATGACCACTAGGAGCATCTTTGGCGATTCTGAATCGACAGACAGAATTTTCTTTCTCTAGGGGAATAAATCTTTGGGCACCTAGTGGTCATG

1 CATGACCACTAGGAGCATCTTTGGCGACGCAGTGGGCAGATAGGCGGACATACGTCGCTAGGGGAATAAATCTTTGGGCACCTAGTGGTCATG

1 CATGACCACTAGGAGCATCTTTGGCGAGCGTGTCAAGGCGTACGATCTGGACATTCCCTAGGGGAATAAATCTTTGGGCACCTAGTGGTCATG

1 CATGACCACTAGGAGCATCTTTGGCGAGATCGGGAGAATCGTTGGCATAGGTGTCTCCTAGGGGAATAAATCTTTGGGCACCTAGTGGTCATG

1 CATGACCACTAGGAGCATCTTTGGCGAACGGCAGGTGTTGCGGTGGTCTGCGGATCCCTAGGGGAATAAATCTTTGGGCACCTAGTGGTCATG

1 CATGACCACTAGGAGCATCTTTGGCGACAGGAATGTGTAGGCGTGGTCCTTGAAACCCTAGGGGAATAAATCTTTGGGCACCTAGTGGTCATG

1 CATGACCACTAGGAGCATCTTTGGCGACGGCGTAAGGCCATCTGTGCGGCGGATCCCCTAGGGGAATAAATCTTTGGGCACCTAGTGGTCATG

1 CATGACCACTAGGAGCATCTTTGGCGAGAAGACTTTAGATTCGGGGACCAGTTGCTGCTAGGGGAATAAATCTTTGGGCACCTAGTGGTCATG

1 CATGACCACTAGGAGCATCTTTGGCGAGGCATGCTAAGGTCCGCTCTGATGCGCTCTCTAGGGGAATAAATCTTTGGGCACCTAGTGGTCATG

1 CATGACCACTAGGAGCATCTTTGGCGAGATGGTGTACACCGTCGAGCAGACGTTGGGCTAGGGGAATAAATCTTTGGGCACCTAGTGGTCATG

1 CATGACCACTAGGAGCATCTTTGGCGACTTGGGGGCACGACCTTGGTAGGTATAAAGCTAGGGGAATAAATCTTTGGGCACCTAGTGGTCATG

1 CATGACCACTAGGAGCATCTTTGGCGAGCCGATTGAGCAACAATGTGTTCTGGGTAACTAGGGGAATAAATCTTTGGGCACCTAGTGGTCATG

1 CATGACCACTAGGAGCATCTTTGGCGATCTGTGTACGCATCATGTGGGTAGCACATTCTAGGGGAATAAATCTTTGGGCACCTAGTGGTCATG

1 CATGACCACTAGGAGCATCTTTGGCGAACGGCAGGTTTTGCGGTGGTCTGAGAATCCCTAGGGGAATAAATCTTTGGGCACCTAGTGGTCATG

1 CATGACCACTAGGAGCATCTTTGGCGAGGCGTGTTGCGCGGGACCGTTCGGCCTAGACTAGGGGAATAAATCTTTGGGCACCTAGTGGTCATG

1 CATGACCACTAGGAGCATCTTTGGCGATAGGCGTGTACGGTCGAGCGAAAGCGATGGCTAGGGGAATAAATCTTTGGGCACCTAGTGGTCATG

1 CATGACCACTAGGAGCATCTTTGGCGAGATCGGGCGAATCGGTGGCATTGGTGACTCCTAGGGGAATAAATCTTTGGGCACCTAGTGGTCATG

1 CATGACCACTAGGAGCATCTTTGGCGAAGAGGCCTTCGAATGGCTGTTCGGGATGCCCTAGGGGAATAAATCTTTGGGCACCTAGTGGTCATG

1 CATGACCACTAGGAGCATCTTTGGCGAGAACACTATGCAAGCTGGGAGGGGTTTCTGCTAGGGGAATAAATCTTTGGGCACCTAGTGGTCATG

1 CATGACCACTAGGAGCATCTTTGGCGATCCGGGGGGTCCGCGATCCGACGCTGTGGGCTAGGGGAATAAATCTTTGGGCACCTAGTGGTCATG

1 CATGACCACTAGGAGCATCTTTGGCGAGATCGGGCGGTTGGGTTGCTCTGGTGACACCTAGGGGAATAAATCTTTGGGCACCTAGTGGTCATG

1 CATGACCACTAGGAGCATCTTTGGCGATGGAGTGGTCGGCCATGTGGGGGGTCGGGGCTAGGGGAATAAATCTTTGGGCACCTAGTGGTCATG

1 CATGACCACTAGGAGCATCTTTGGCGATGGGACCCCGTCCTCTAACGGGCAACGGTCCTAGGGGAATAAATCTTTGGGCACCTAGTGGTCATG

1 CATGACCACTAGGAGCATCTTTGGCGAGAAGACTTTGGATTCGGGGGCCAGTTGCTCCTAGGGGAATAAATCTTTGGGCACCTAGTGGTCATG

1 CATGACCACTAGGAGCATCTTTGGCGATATTCTTTCGTGACGTATCTTATTTATATCCTAGGGGAATAAATCTTTGGGCACCTAGTGGTCATG

1 CATGACCACTAGGAGCATCTTTGGCGACGGTGTGGGAAACTTGTTTCGGCGGTGCTACTAGGGGAATAAATCTTTGGGCACCTAGTGGTCATG

1 CATGACCACTAGGAGCATCTTTGGCGAGATCCGTGACGAGCGAAGGTGTGCGACCTACTAGGGGAATAAATCTTTGGGCACCTAGTGGTCATG

1 CATGACCACTAGGAGCATCTTTGGCGAAGGGTGTAGGACCTCAAGTGGATCTCATAGCTAGGGGAATAAATCTTTGGGCACCTAGTGGTCATG

1 CATGACCACTAGGAGCATCTTTGGCGATCCTCGTCAGATGGTGAAGCAGACGTTTGTCTAGGGGAATAAATCTTTGGGCACCTAGTGGTCATG

1 CATGACCACTAGGAGCATCTTTGGCGAGATCGAGAGAATCGGAGTCATTGGTGTCTCCTAGGGGAATAAATCTTTGGGCACCTAGTGGTCATG

1 CATGACCACTAGGAGCATCTTTGGCGAGAAGACTATGGATGCTGGGAACAGTTGCTGCTAGGGGAATAAATCTTTGGGCACCTAGTGGTCATG

1 CATGACCACTAGGAGCATCTTTGGCGAACGGAAGGTGTAGCGGTGCTCTGTGAATCCCTAGGGGAATAAATCTTTGGGCACCTAGTGGTCATG

1 CATGACCACTAGGAGCATCTTTGGCGACCGGCAGGTGTTGCGGTGGTCTGTGAACTCCTAGGGGAATAAATCTTTGGGCACCTAGTGGTCATG

1 CATGACCACTAGGAGCATCTTTGGCGACGGGGGATGCCGATGCGCTGGACGACCGAGCTAGGGGAATAAATCTTTGGGCACCTAGTGGTCATG

1 CATGACCACTAGGAGCATCTTTGGCGAAAACTCGTTGGGCATCGCACTCCCATACGTCTAGGGGAATAAATCTTTGGGCACCTAGTGGTCATG

1 CATGACCACTAGGAGCATCTTTGGCGAACGGCAGGTGTTGCAGTGGTCTATGAATCCCTAGGGGAATAAATCTTTGGGCACCTAGTGGTCATG

1 CATGACCACTAGGAGCATCTTTGGCGACATCCCGGTAAAGATTCACGAAATGGTCGACTAGGGGAATAAATCTTTGGGCACCTAGTGGTCATG

1 CATGACCACTAGGAGCATCTTTGGCGAACGGCAGGTGTTGCAGTGGTCTGTGTATCCCTAGGGGAATAAATCTTTGGGCACCTAGTGGTCATG

1 CATGACCACTAGGAGCATCTTTGGCGAATACAGAGATCTGGCCTACGGTGTCATCGACTAGGGGAATAAATCTTTGGGCACCTAGTGGTCATG

1 CATGACCACTAGGAGCATCTTTGGCGACGAAGGGGTCTGTGATGGTGCATCCGTCGACTAGGGGAATAAATCTTTGGGCACCTAGTGGTCATG

1 CATGACCACTAGGAGCATCTTTGGCGAACGGCAGGTGTTGCGGAGGTCTGTGGATCCCTAGGGGAATAAATCTTTGGGCACCTAGTGGTCATG

1 CATGACCACTAGGAGCATCTTTGGCGATATCGGGAGAATCGGTGGCATTGGTTTCTCCTAGGGGAATAAATCTTTGGGCACCTAGTGGTCATG

1 CATGACCACTAGGAGCATCTTTGGCGACGGTGTGGGCGAGAGGCGTGGTTGAGGGCGCTAGGGGAATAAATCTTTGGGCACCTAGTGGTCATG

1 CATGACCACTAGGAGCATCTTTGGCGAACGTCACAGTGTCCTCCCGTGAATTGGAACCTAGGGGAATAAATCTTTGGGCACCTAGTGGTCATG

1 CATGACCACTAGGAGCATCTTTGGCGAGAGCGGAAGAATCGGTGGCATTGGTGTCTCCTAGGGGAATAAATCTTTGGGCACCTAGTGGTCATG

1 CATGACCACTAGGAGCATCTTTGGCGATCCGGGGGTCACTGAGGCGACGCTAATTGGCTAGGGGAATAAATCTTTGGGCACCTAGTGGTCATG

1 CATGACCACTAGGAGCATCTTTGGCGAGATCAGGAGAATCGGTGGCATTGGTGTCTACTAGGGGAATAAATCTTTGGGCACCTAGTGGTCATG

1 CATGACCACTAGGAGCATCTTTGGCGAGATCGGGAGAATCGGTGGCACTGGTGCCTCCTAGGGGAATAAATCTTTGGGCACCTAGTGGTCATG

1 CATGACCACTAGGAGCATCTTTGGCGACTCCCACCGGGTGGGGACGAAGTGGAGCGACTAGGGGAATAAATCTTTGGGCACCTAGTGGTCATG

1 CATGACCACTAGGAGCATCTTTGGCGATAGCGAAGTGCTGTGAACAATGGTTAGCACCTAGGGGAATAAATCTTTGGGCACCTAGTGGTCATG

1 CATGACCACTAGGAGCATCTTTGGCGAATTGGTAGATTAGTCTAATTCCGGTATGGCCTAGGGGAATAAATCTTTGGGCACCTAGTGGTCATG

1 CATGACCACTAGGAGCATCTTTGGCGATCTTCGTCAGATGGTGAAGCAGACGTTTGGCTAGGGGAATAAATCTTTGGGCACCTAGTGGTCATG

1 CATGACCACTAGGAGCATCTTTGGCGACTGTGGCTGGCAGGTTCGTACTATAGGCGGCTAGGGGAATAAATCTTTGGGCACCTAGTGGTCATG

1 CATGACCACTAGGAGCATCTTTGGCGAACTAGGGGGGTGCTGTTAAGCGTTCGAAGACTAGGGGAATAAATCTTTGGGCACCTAGTGGTCATG

1 CATGACCACTAGGAGCATCTTTGGCGAAGATCTAATGGGCTTGTGACCCGCCTCGGCCTAGGGGAATAAATCTTTGGGCACCTAGTGGTCATG

1 CATGACCACTAGGAGCATCTTTGGCGATCGCACAGCGGAAGACCATCGGGCTCGGGGCTAGGGGAATAAATCTTTGGGCACCTAGTGGTCATG

1 CATGACCACTAGGAGCATCTTTGGCGACTAAGTGGCCAGCGCGTTAAATTGGGTAGACTAGGGGAATAAATCTTTGGGCACCTAGTGGTCATG

1 CATGACCACTAGGAGCATCTTTGGCGAGATCGGGAGAATAGGTGGTATTGGTGTCTCCTAGGGGAATAAATCTTTGGGCACCTAGTGGTCATG

1 CATGACCACTAGGAGCATCTTTGGCGATACGGACTACGGTGCGTGGGTTGCCGGCGACTAGGGGAATAAATCTTTGGGCACCTAGTGGTCATG

1 CATGACCACTAGGAGCATCTTTGGCGAACCTTATGCCAGTGAAGAAGTCCTTCGTTGCTAGGGGAATAAATCTTTGGGCACCTAGTGGTCATG

1 CATGACCACTAGGAGCATCTTTGGCGAATGGCCGGTACGGCCTCCTAGTCCTCGGTACTAGGGGAATAAATCTTTGGGCACCTAGTGGTCATG

1 CATGACCACTAGGAGCATCTTTGGCGACGAGGGGCACGGGTGGTCATTGCGAGAAGTCTAGGGGAATAAATCTTTGGGCACCTAGTGGTCATG

1 CATGACCACTAGGAGCATCTTTGGCGACATGTGCAAACAAGCGGGTTAAAGTGGCGACTAGGGGAATAAATCTTTGGGCACCTAGTGGTCATG

1 CATGACCACTAGGAGCATCTTTGGCGACGGTGTGGGGTCATCAATGCGGCGGTGACCCTAGGGGAATAAATCTTTGGGCACCTAGTGGTCATG

1 CATGACCACTAGGAGCATCTTTGGCGAACCAAGGGGGGGGACGAAGTGCTTCGAAGACTAGGGGAATAAATCTTTGGGCACCTAGTGGTCATG

1 CATGACCACTAGGAGCATCTTTGGCGAGTTTGGGTGGCGGTATGGCGCTATATTTTCCTAGGGGAATAAATCTTTGGGCACCTAGTGGTCATG

1 CATGACCACTAGGAGCATCTTTGGCGAGACATGGTAGGCATAACACCAATAGTGGAGCTAGGGGAATAAATCTTTGGGCACCTAGTGGTCATG

1 CATGACCACTAGGAGCATCTTTGGCGAACGTCAGGAGTTGCGGTGGTTTGTGAATCCCTAGGGGAATAAATCTTTGGGCACCTAGTGGTCATG

1 CATGACCACTAGGAGCATCTTTGGCGAGATCCCACGGGTGTAGGGCCCAGGCTGCAGCTAGGGGAATAAATCTTTGGGCACCTAGTGGTCATG

1 CATGACCACTAGGAGCATCTTTGGCGAGGAGAACTGGTAGTTCGGGCCTAGAAGGTACTAGGGGAATAAATCTTTGGGCACCTAGTGGTCATG

1 CATGACCACTAGGAGCATCTTTGGCGAGAAGACTCTGGATTCGGGCACCAGTTGCTCCTAGGGGAATAAATCTTTGGGCACCTAGTGGTCATG

1 CATGACCACTAGGAGCATCTTTGGCGATCCACATTGGCCGTTGTTCGGTTACGTGACCTAGGGGAATAAATCTTTGGGCACCTAGTGGTCATG

1 CATGACCACTAGGAGCATCTTTGGCGAAAGTCGGTAGAACAGGGTGGGGCGCCGTCCCTAGGGGAATAAATCTTTGGGCACCTAGTGGTCATG

1 CATGACCACTAGGAGCATCTTTGGCGAAGGCGACACAGTCGATCGGGTTAGAGGAAGCTAGGGGAATAAATCTTTGGGCACCTAGTGGTCATG

1 CATGACCACTAGGAGCATCTTTGGCGAAATCGGGAGAATCGGTGGCACTGGTGTCTCCTAGGGGAATAAATCTTTGGGCACCTAGTGGTCATG

1 CATGACCACTAGGAGCATCTTTGGCGAGGCTGGCGTCAAAGTGATGTGGTCGAGACACTAGGGGAATAAATCTTTGGGCACCTAGTGGTCATG

1 CATGACCACTAGGAGCATCTTTGGCGAGATCGGGAGAATCTGTGGAATTGGTGTCTCCTAGGGGAATAAATCTTTGGGCACCTAGTGGTCATG

1 CATGACCACTAGGAGCATCTTTGGCGATGCGAGTCCAGCGGCCTTCTTGGCCTGCGACTAGGGGAATAAATCTTTGGGCACCTAGTGGTCATG

1 CATGACCACTAGGAGCATCTTTGGCGATCGCTGAGCGGCGAGGGTTAGTCGCGTGGCCTAGGGGAATAAATCTTTGGGCACCTAGTGGTCATG

1 CATGACCACTAGGAGCATCTTTGGCGAGATCGGGAGAACCGGTGGTATTGGTGTCTCCTAGGGGAATAAATCTTTGGGCACCTAGTGGTCATG

1 CATGACCACTAGGAGCATCTTTGGCGACAAATCCTGTGATCGAGGGTCACTAGGTGCCTAGGGGAATAAATCTTTGGGCACCTAGTGGTCATG

1 CATGACCACTAGGAGCATCTTTGGCGAGATTGGGAGAATTGGTGGCATTGTTGTCTCCTAGGGGAATAAATCTTTGGGCACCTAGTGGTCATG

1 CATGACCACTAGGAGCATCTTTGGCGATCCGGGGGTCACAAGTGACGGCGCACAGGGCTAGGGGAATAAATCTTTGGGCACCTAGTGGTCATG

1 CATGACCACTAGGAGCATCTTTGGCGACGATGGTGGTCGTGGGATCGGAAAAGTCGACTAGGGGAATAAATCTTTGGGCACCTAGTGGTCATG

1 CATGACCACTAGGAGCATCTTTGGCGATTCATATAACGACAAATGGGCAGGATCTGACTAGGGGAATAAATCTTTGGGCACCTAGTGGTCATG

1 CATGACCACTAGGAGCATCTTTGGCGAGATCGGGATAACCGGTGGCATTGGTGTCTCCTAGGGGAATAAATCTTTGGGCACCTAGTGGTCATG

1 CATGACCACTAGGAGCATCTTTGGCGAGGCGAGTTCAGCGGCCTTCTTGGCCTGCGACTAGGGGAATAAATCTTTGGGCACCTAGTGGTCATG

1 CATGACCACTAGGAGCATCTTTGGCGAACGGCAGGGGTTGCGGTGGTTTGTGAATCCCTAGGGGAATAAATCTTTGGGCACCTAGTGGTCATG

1 CATGACCACTAGGAGCATCTTTGGCGATGAGGAAGTCGGGGTCGGGTTTTGCAATTTCTAGGGGAATAAATCTTTGGGCACCTAGTGGTCATG

1 CATGACCACTAGGAGCATCTTTGGCGAGATCGGGAATATCGGTGGAAGTTGATGAACCTAGGGGAATAAATCTTTGGGCACCTAGTGGTCATG

1 CATGACCACTAGGAGCATCTTTGGCGAACGGCATGTGTTGCGGTGGTCTGTGAATACCTAGGGGAATAAATCTTTGGGCACCTAGTGGTCATG

1 CATGACCACTAGGAGCATCTTTGGCGACCCGCGAAATGATGAATGAATGTCCTGTGACTAGGGGAATAAATCTTTGGGCACCTAGTGGTCATG

1 CATGACCACTAGGAGCATCTTTGGCGAAAGTCGGTATAACAGGGTGGGGTGCTTTCCCTAGGGGAATAAATCTTTGGGCACCTAGTGGTCATG

1 CATGACCACTAGGAGCATCTTTGGCGAAAGGTACCTGTCGATGTATGGAGGCGCCAGCTAGGGGAATAAATCTTTGGGCACCTAGTGGTCATG

1 CATGACCACTAGGAGCATCTTTGGCGAGTATTGGCTGGTAGGTTGCGTATTGCGGAGCTAGGGGAATAAATCTTTGGGCACCTAGTGGTCATG

1 CATGACCACTAGGAGCATCTTTGGCGACCCTCACTGGGACCAGTGCCTTCGGGCAGACTAGGGGAATAAATCTTTGGGCACCTAGTGGTCATG

1 CATGACCACTAGGAGCATCTTTGGCGAGATCGGTAGAATCTCTGGGAGAGGTGTCTCCTAGGGGAATAAATCTTTGGGCACCTAGTGGTCATG

1 CATGACCACTAGGAGCATCTTTGGCGAGATCGGAAGAATCGGTGCCATTGGTGTCTCCTAGGGGAATAAATCTTTGGGCACCTAGTGGTCATG

1 CATGACCACTAGGAGCATCTTTGGCGAGATCGGGAGAAACGCTGGCAATGGTTAATCCTAGGGGAATAAATCTTTGGGCACCTAGTGGTCATG

1 CATGACCACTAGGAGCATCTTTGGCGATGGGTGTTGACGGCCGCCGCCGCGGCTACACTAGGGGAATAAATCTTTGGGCACCTAGTGGTCATG

1 CATGACCACTAGGAGCATCTTTGGCGAGTGCGGGAGAATCGGTGGCAGTGGGGTCTCCTAGGGGAATAAATCTTTGGGCACCTAGTGGTCATG

1 CATGACCACTAGGAGCATCTTTGGCGACGTAGTGGGCTGGCAGGGAGTACTAGTTGCCTAGGGGAATAAATCTTTGGGCACCTAGTGGTCATG

1 CATGACCACTAGGAGCATCTTTGGCGACCGGATATGGTAGGTAATGTGCTGAGGAGGCTAGGGGAATAAATCTTTGGGCACCTAGTGGTCATG

1 CATGACCACTAGGAGCATCTTTGGCGACGAACGGTGCCGGTATGTCAAAGTCGTGCCCTAGGGGAATAAATCTTTGGGCACCTAGTGGTCATG

1 CATGACCACTAGGAGCATCTTTGGCGACCCAGTGCGCGAAGAGCAAGGCGTGGAAGACTAGGGGAATAAATCTTTGGGCACCTAGTGGTCATG

1 CATGACCACTAGGAGCATCTTTGGCGATATCGGGAGAATCGGCGGCATTGTTGTCTCCTAGGGGAATAAATCTTTGGGCACCTAGTGGTCATG

1 CATGACCACTAGGAGCATCTTTGGCGAGCGGCAGGTGTTGCGGTGGTCCGTGAATCCCTAGGGGAATAAATCTTTGGGCACCTAGTGGTCATG

1 CATGACCACTAGGAGCATCTTTGGCGATCCGGGGGCCTGAAGGGAGACGCGCTCGGGCTAGGGGAATAAATCTTTGGGCACCTAGTGGTCATG

1 CATGACCACTAGGAGCATCTTTGGCGAGAAGGCTCTGGATACGGGGACCAGTTGCTGCTAGGGGAATAAATCTTTGGGCACCTAGTGGTCATG

1 CATGACCACTAGGAGCATCTTTGGCGACGGGGTGGGGCCGAAGTGCGGCGGCGATTCCTAGGGGAATAAATCTTTGGGCACCTAGTGGTCATG

1 CATGACCACTAGGAGCATCTTTGGCGAGATCGGGAGAATCTGTGGCAGTGGTGTCTGCTAGGGGAATAAATCTTTGGGCACCTAGTGGTCATG

1 CATGACCACTAGGAGCATCTTTGGCGATCGGTGGTGCGCCTAAGGGCGTAGTGGACGCTAGGGGAATAAATCTTTGGGCACCTAGTGGTCATG

1 CATGACCACTAGGAGCATCTTTGGCGATTAGGTGGGCACGGGAGCAACAAAAGTCGACTAGGGGAATAAATCTTTGGGCACCTAGTGGTCATG

1 CATGACCACTAGGAGCATCTTTGGCGACTCAGGGACGCAGGCACGGAGAAGTGGAGCCTAGGGGAATAAATCTTTGGGCACCTAGTGGTCATG

1 CATGACCACTAGGAGCATCTTTGGCGAAGCGTGGCTTTCAATGCGAAGGAGGTGCGACTAGGGGAATAAATCTTTGGGCACCTAGTGGTCATG

1 CATGACCACTAGGAGCATCTTTGGCGAGATCGGGAGAATTGGTGGCATTGGTATCTCCTAGGGGAATAAATCTTTGGGCACCTAGTGGTCATG

1 CATGACCACTAGGAGCATCTTTGGCGAGGACGAGTGGAATCGTAGTAAACACTTATCCTAGGGGAATAAATCTTTGGGCACCTAGTGGTCATG

1 CATGACCACTAGGAGCATCTTTGGCGAGATCGGGAGAATCTGTGGCAGTGGCGTCTCCTAGGGGAATAAATCTTTGGGCACCTAGTGGTCATG

1 CATGACCACTAGGAGCATCTTTGGCGAGTAGGGGGCACCGGTAATCGACAGACGAGCCTAGGGGAATAAATCTTTGGGCACCTAGTGGTCATG

1 CATGACCACTAGGAGCATCTTTGGCGACCAGGACGGGACGTTCCGAGACATAGTGCCCTAGGGGAATAAATCTTTGGGCACCTAGTGGTCATG

1 CATGACCACTAGGAGCATCTTTGGCGAGGGGGGGCGCCGGCAGCGGCGTGAATGCGACTAGGGGAATAAATCTTTGGGCACCTAGTGGTCATG

1 CATGACCACTAGGAGCATCTTTGGCGAGGCCCGGTGTGCGGAGGAAGATGTCGACGACTAGGGGAATAAATCTTTGGGCACCTAGTGGTCATG

1 CATGACCACTAGGAGCATCTTTGGCGAGAAGACTCAGGATTTGGGGACCAGTTGCTGCTAGGGGAATAAATCTTTGGGCACCTAGTGGTCATG

1 CATGACCACTAGGAGCATCTTTGGCGAATATAGCTACTCACATCTGCTGATTTACTTCTAGGGGAATAAATCTTTGGGCACCTAGTGGTCATG

1 CATGACCACTAGGAGCATCTTTGGCGAAACGCTGTAGTCGGCAGATTGGTCGAGGTCCTAGGGGAATAAATCTTTGGGCACCTAGTGGTCATG

1 CATGACCACTAGGAGCATCTTTGGCGAACGTCAGGTGTTGCGGTGGACAGTGAATCCCTAGGGGAATAAATCTTTGGGCACCTAGTGGTCATG

1 CATGACCACTAGGAGCATCTTTGGCGATCGGCAGGTGTTGCGGTGGTCTGTGAATCTCTAGGGGAATAAATCTTTGGGCACCTAGTGGTCATG

1 CATGACCACTAGGAGCATCTTTGGCGACGCAGCATGCTTCGAGGGGACCGGGAAGGACTAGGGGAATAAATCTTTGGGCACCTAGTGGTCATG

1 CATGACCACTAGGAGCATCTTTGGCGACTAGAGGGGGCATGTAAAGTTGCGAATAGACTAGGGGAATAAATCTTTGGGCACCTAGTGGTCATG

1 CATGACCACTAGGAGCATCTTTGGCGAACAATGAGGGTATCAGTCCCCGGTCGGGAGCTAGGGGAATAAATCTTTGGGCACCTAGTGGTCATG

1 CATGACCACTAGGAGCATCTTTGGCGATCGAGTATCGGTGGTCTGATTCAGATGCGTCTAGGGGAATAAATCTTTGGGCACCTAGTGGTCATG

1 CATGACCACTAGGAGCATCTTTGGCGAACGGCAGTTGTTGCGGTGGTCTGCGAAACCCTAGGGGAATAAATCTTTGGGCACCTAGTGGTCATG

1 CATGACCACTAGGAGCATCTTTGGCGAGATCGGTAGAATCGGTGGCAGTGGTGTCTCCTAGGGGAATAAATCTTTGGGCACCTAGTGGTCATG

1 CATGACCACTAGGAGCATCTTTGGCGACTGAACCTGCCAGGAATGGCAGGCAGTGGACTAGGGGAATAAATCTTTGGGCACCTAGTGGTCATG

1 CATGACCACTAGGAGCATCTTTGGCGACAGGTGATCGGCGTGTTGAGCTGGTTGTATCTAGGGGAATAAATCTTTGGGCACCTAGTGGTCATG

1 CATGACCACTAGGAGCATCTTTGGCGAACGGCAGGTGTTGAGGTGGTATGTGAATCCCTAGGGGAATAAATCTTTGGGCACCTAGTGGTCATG

1 CATGACCACTAGGAGCATCTTTGGCGACGCAGGGGGCAGCTATGAGGTAAAAGTCGACTAGGGGAATAAATCTTTGGGCACCTAGTGGTCATG

1 CATGACCACTAGGAGCATCTTTGGCGAGAAGACTCTGGATTCTTGGACCAGTTGCTGCTAGGGGAATAAATCTTTGGGCACCTAGTGGTCATG

1 CATGACCACTAGGAGCATCTTTGGCGAGCGGGGGGCGACGGCCGGTAGTGGGTGAGACTAGGGGAATAAATCTTTGGGCACCTAGTGGTCATG

1 CATGACCACTAGGAGCATCTTTGGCGAACGGAAGGTGTTGCGGTGGTCTGTGAATCACTAGGGGAATAAATCTTTGGGCACCTAGTGGTCATG

1 CATGACCACTAGGAGCATCTTTGGCGAGCGACAAATGGAAATGCGACGTAGTGGATGCTAGGGGAATAAATCTTTGGGCACCTAGTGGTCATG

1 CATGACCACTAGGAGCATCTTTGGCGATCGCAGAGCGGAAGACTGTTGTCTCGGGCCCTAGGGGAATAAATCTTTGGGCACCTAGTGGTCATG

1 CATGACCACTAGGAGCATCTTTGGCGAGATCGGTAGAACCGGTGGCATTTGTGTCTCCTAGGGGAATAAATCTTTGGGCACCTAGTGGTCATG

1 CATGACCACTAGGAGCATCTTTGGCGAGATCTGGAGAATCGGTGGCAAAGGTGTCTCCTAGGGGAATAAATCTTTGGGCACCTAGTGGTCATG

1 CATGACCACTAGGAGCATCTTTGGCGACTAAGTGGACAGCGCGTTAAATGGGGTAGACTAGGGGAATAAATCTTTGGGCACCTAGTGGTCATG

1 CATGACCACTAGGAGCATCTTTGGCGAGAGTGGGCGGTCTGGAGCTAGGTCTGTCGACTAGGGGAATAAATCTTTGGGCACCTAGTGGTCATG

1 CATGACCACTAGGAGCATCTTTGGCGAGAACACTCGGGAGTCTGGGACGAGTTTCTGCTAGGGGAATAAATCTTTGGGCACCTAGTGGTCATG

1 CATGACCACTAGGAGCATCTTTGGCGACTAGATCGGTCGGAGGTAGTGTTCTGGAAGCTAGGGGAATAAATCTTTGGGCACCTAGTGGTCATG

1 CATGACCACTAGGAGCATCTTTGGCGAGACCTGGAGAATCGGTGGCATTGGTGTCTCCTAGGGGAATAAATCTTTGGGCACCTAGTGGTCATG

1 CATGACCACTAGGAGCATCTTTGGCGAATCGCAGGGTGTGAGAGGGGCGTGTCCATGCTAGGGGAATAAATCTTTGGGCACCTAGTGGTCATG

1 CATGACCACTAGGAGCATCTTTGGCGAGATCGGGGGAATCGGTGGCATTGGTGTATCCTAGGGGAATAAATCTTTGGGCACCTAGTGGTCATG

1 CATGACCACTAGGAGCATCTTTGGCGATTGTGTAAGGTTGGAGTACTCTGCTACCAGCTAGGGGAATAAATCTTTGGGCACCTAGTGGTCATG

1 CATGACCACTAGGAGCATCTTTGGCGACTCAAGGGGGTCGGTATGTGGCAGAGGCGACTAGGGGAATAAATCTTTGGGCACCTAGTGGTCATG

1 CATGACCACTAGGAGCATCTTTGGCGAGTGGGGGGCGACGGCCGGTAGTGGTTGAGACTAGGGGAATAAATCTTTGGGCACCTAGTGGTCATG

1 CATGACCACTAGGAGCATCTTTGGCGAGATCGGAAGAATCGGTAGCATTGGTGTCTCCTAGGGGAATAAATCTTTGGGCACCTAGTGGTCATG

1 CATGACCACTAGGAGCATCTTTGGCGAGATCTGGAGAATCGGTTGCATTGGTGTCTTCTAGGGGAATAAATCTTTGGGCACCTAGTGGTCATG

1 CATGACCACTAGGAGCATCTTTGGCGAGATCGCGAGAATCGGTGGCATTGGTGTCACCTAGGGGAATAAATCTTTGGGCACCTAGTGGTCATG

1 CATGACCACTAGGAGCATCTTTGGCGAAAGCATAGCTGTGCGTGTCGGTCTAGCATCCTAGGGGAATAAATCTTTGGGCACCTAGTGGTCATG

1 CATGACCACTAGGAGCATCTTTGGCGAGCATACTTGTGAGCGGAGGTGTGCGATCTACTAGGGGAATAAATCTTTGGGCACCTAGTGGTCATG

1 CATGACCACTAGGAGCATCTTTGGCGATCCGGGGGTCAGACAAGACGGCGCGTTGGGCTAGGGGAATAAATCTTTGGGCACCTAGTGGTCATG

1 CATGACCACTAGGAGCATCTTTGGCGAGATCGGGAGAATCGGTGGCATTGGTGTTAACTAGGGGAATAAATCTTTGGGCACCTAGTGGTCATG

1 CATGACCACTAGGAGCATCTTTGGCGAGATCGGTATAATCGGTGGCATTGGTGTCCCCTAGGGGAATAAATCTTTGGGCACCTAGTGGTCATG

1 CATGACCACTAGGAGCATCTTTGGCGAGAAGTCTCTGGATTCGGGGACCAGTTGCTCCTAGGGGAATAAATCTTTGGGCACCTAGTGGTCATG

1 CATGACCACTAGGAGCATCTTTGGCGAACGTCAGGTGTTGCGGTGGTCTGTGGATCCCTAGGGGAATAAATCTTTGGGCACCTAGTGGTCATG

1 CATGACCACTAGGAGCATCTTTGGCGACGATGGTGGGCAGTAGGAATTCAATGTCGACTAGGGGAATAAATCTTTGGGCACCTAGTGGTCATG

1 CATGACCACTAGGAGCATCTTTGGCGATGAATATTGCGGAGTATCTTTAGTCAGGCCCTAGGGGAATAAATCTTTGGGCACCTAGTGGTCATG

1 CATGACCACTAGGAGCATCTTTGGCGAGTCCAAGGAGGGGTGGGTCGAGGAAGCCTGCTAGGGGAATAAATCTTTGGGCACCTAGTGGTCATG

1 CATGACCACTAGGAGCATCTTTGGCGACCCGCACGCAGGATGCGACGCAAGGGTGGACTAGGGGAATAAATCTTTGGGCACCTAGTGGTCATG

1 CATGACCACTAGGAGCATCTTTGGCGATCCAGGGGCATGCTATGCGACGCTTATGGGCTAGGGGAATAAATCTTTGGGCACCTAGTGGTCATG

1 CATGACCACTAGGAGCATCTTTGGCGAATGGCGTGGCTCAGCCTACGTGAGGACTGGCTAGGGGAATAAATCTTTGGGCACCTAGTGGTCATG

1 CATGACCACTAGGAGCATCTTTGGCGAGATCAGGATAATCGGAGGCATAGGTGTCTCCTAGGGGAATAAATCTTTGGGCACCTAGTGGTCATG

1 CATGACCACTAGGAGCATCTTTGGCGACGGTTGGTCGGTAGGATAGTATTCCGTTGGCTAGGGGAATAAATCTTTGGGCACCTAGTGGTCATG

1 CATGACCACTAGGAGCATCTTTGGCGAACGTGCGAGATATGCCGCCTGGAGCGTAGGCTAGGGGAATAAATCTTTGGGCACCTAGTGGTCATG

1 CATGACCACTAGGAGCATCTTTGGCGAAATGGAGGTGATCCGGTGGACTGTGAAAACCTAGGGGAATAAATCTTTGGGCACCTAGTGGTCATG

1 CATGACCACTAGGAGCATCTTTGGCGACTGTAGTGGGCTTGGAAATATGGCAGGCGACTAGGGGAATAAATCTTTGGGCACCTAGTGGTCATG

1 CATGACCACTAGGAGCATCTTTGGCGACATTGACAGCTAGGGTGAGGATGTGTGCGACTAGGGGAATAAATCTTTGGGCACCTAGTGGTCATG

1 CATGACCACTAGGAGCATCTTTGGCGAGTATTGGCTGGTAGTTTGCGTATTGGGGAGCTAGGGGAATAAATCTTTGGGCACCTAGTGGTCATG

1 CATGACCACTAGGAGCATCTTTGGCGAATGGTTGGTCTGACTTCCAGTCACAGGAGACTAGGGGAATAAATCTTTGGGCACCTAGTGGTCATG

1 CATGACCACTAGGAGCATCTTTGGCGATCGCATAGCGTACGAAGAGTTTACGGGGGCCTAGGGGAATAAATCTTTGGGCACCTAGTGGTCATG

1 CATGACCACTAGGAGCATCTTTGGCGAGATCGGTAGAATCTGTGGAATTGGTGTCTCCTAGGGGAATAAATCTTTGGGCACCTAGTGGTCATG

1 CATGACCACTAGGAGCATCTTTGGCGAGATCGGGAGAATCGGAAGCAGTGGTGTCTCCTAGGGGAATAAATCTTTGGGCACCTAGTGGTCATG

1 CATGACCACTAGGAGCATCTTTGGCGATCGCACAGCGGAAATGGTATCATTCGTAGACTAGGGGAATAAATCTTTGGGCACCTAGTGGTCATG

1 CATGACCACTAGGAGCATCTTTGGCGACGATGAGAGTTAATGTGCGGAAGCGAGTATCTAGGGGAATAAATCTTTGGGCACCTAGTGGTCATG

1 CATGACCACTAGGAGCATCTTTGGCGAACGGCAGGCGTTGCGGTGGTCCGTGAATCCCTAGGGGAATAAATCTTTGGGCACCTAGTGGTCATG

1 CATGACCACTAGGAGCATCTTTGGCGACGTAGTGGCGAAGGACCAGTGCACAGGTCCCTAGGGGAATAAATCTTTGGGCACCTAGTGGTCATG

1 CATGACCACTAGGAGCATCTTTGGCGAGAAGCTGGTATTACCAGTCCTGAAGTGGACCTAGGGGAATAAATCTTTGGGCACCTAGTGGTCATG

1 CATGACCACTAGGAGCATCTTTGGCGAGGGAGGGCGCCGGCAACGGTGTGAATGCGACTAGGGGAATAAATCTTTGGGCACCTAGTGGTCATG

1 CATGACCACTAGGAGCATCTTTGGCGAGATCGGGAGAATCGGTTGCATTGGTGTCACCTAGGGGAATAAATCTTTGGGCACCTAGTGGTCATG

1 CATGACCACTAGGAGCATCTTTGGCGAGATCGGGAGAGTCGGTGGCATTGGTGGCTCCTAGGGGAATAAATCTTTGGGCACCTAGTGGTCATG

1 CATGACCACTAGGAGCATCTTTGGCGACTGGTGTGCCTAGTGGGAAGGGGAGTGTTGCTAGGGGAATAAATCTTTGGGCACCTAGTGGTCATG

1 CATGACCACTAGGAGCATCTTTGGCGAGATCGGGAGAATCGGAGCAATAGGTGTCTCCTAGGGGAATAAATCTTTGGGCACCTAGTGGTCATG

1 CATGACCACTAGGAGCATCTTTGGCGACTCCGGGGGTCTGGTAGGACGACGCTTGGGCTAGGGGAATAAATCTTTGGGCACCTAGTGGTCATG

1 CATGACCACTAGGAGCATCTTTGGCGAGATCGAGAGAATCGGTGGCATTGGTGTCTACTAGGGGAATAAATCTTTGGGCACCTAGTGGTCATG

1 CATGACCACTAGGAGCATCTTTGGCGAAGCAGAAGGTGTGAGTTATAAAGAGATCAACTAGGGGAATAAATCTTTGGGCACCTAGTGGTCATG

1 CATGACCACTAGGAGCATCTTTGGCGAGGCGGGAAGTTCTTGTTCTAGTCGAATGTCCTAGGGGAATAAATCTTTGGGCACCTAGTGGTCATG

1 CATGACCACTAGGAGCATCTTTGGCGAGACCATTTCTAGTTTCGGCTAAGTGCATGCCTAGGGGAATAAATCTTTGGGCACCTAGTGGTCATG

1 CATGACCACTAGGAGCATCTTTGGCGAGCGGTAGGTGTTGCGGTGGTCTGTGAATCCCTAGGGGAATAAATCTTTGGGCACCTAGTGGTCATG

1 CATGACCACTAGGAGCATCTTTGGCGATAGAAGCACAGCGGTCACAAGGTGGGGGGCCTAGGGGAATAAATCTTTGGGCACCTAGTGGTCATG

1 CATGACCACTAGGAGCATCTTTGGCGAACTGTTGAATAGATTGTACAAGGTAGGCCTCTAGGGGAATAAATCTTTGGGCACCTAGTGGTCATG

1 CATGACCACTAGGAGCATCTTTGGCGAACGGAAGGTTTGGCGGTGGTCTGTGAATCCCTAGGGGAATAAATCTTTGGGCACCTAGTGGTCATG

1 CATGACCACTAGGAGCATCTTTGGCGATAAGGGGGTAAAAAGTGACGTCGCATACGGCTAGGGGAATAAATCTTTGGGCACCTAGTGGTCATG

1 CATGACCACTAGGAGCATCTTTGGCGAATGGCCGACACGGCCTTCTAGTCCTTGGTACTAGGGGAATAAATCTTTGGGCACCTAGTGGTCATG

1 CATGACCACTAGGAGCATCTTTGGCGATGGAACGGTATATGCGCGTACGCGGATCGCCTAGGGGAATAAATCTTTGGGCACCTAGTGGTCATG

1 CATGACCACTAGGAGCATCTTTGGCGAGGTGGAATCGAAGCTGTTCACATTCTCTTTCTAGGGGAATAAATCTTTGGGCACCTAGTGGTCATG

1 CATGACCACTAGGAGCATCTTTGGCGAGGGGTAGGGGGCCGCTTGCGCCTGCGTGCGCTAGGGGAATAAATCTTTGGGCACCTAGTGGTCATG

1 CATGACCACTAGGAGCATCTTTGGCGACTGCTAAGACGAAATAAGGCAAAGGCGCTGCTAGGGGAATAAATCTTTGGGCACCTAGTGGTCATG

1 CATGACCACTAGGAGCATCTTTGGCGAGAAGACTCTGTATTCGGGGACCAGTTGCCGCTAGGGGAATAAATCTTTGGGCACCTAGTGGTCATG

1 CATGACCACTAGGAGCATCTTTGGCGAGATCGGGAGAATCGGTGGCATTGATGCCTCCTAGGGGAATAAATCTTTGGGCACCTAGTGGTCATG

1 CATGACCACTAGGAGCATCTTTGGCGACAATGGATCGACGGTGTGGTCTGAGTCCTACTAGGGGAATAAATCTTTGGGCACCTAGTGGTCATG

1 CATGACCACTAGGAGCATCTTTGGCGAGGACAGTAGGGGGCGAGCCCGGTACCCGCACTAGGGGAATAAATCTTTGGGCACCTAGTGGTCATG

1 CATGACCACTAGGAGCATCTTTGGCGAGAAGACTCTGGATTCGGGGGCCAGTAGCTGCTAGGGGAATAAATCTTTGGGCACCTAGTGGTCATG

1 CATGACCACTAGGAGCATCTTTGGCGATTAGGCCGCCAGGGCGTACGAAGGGTGGTTCTAGGGGAATAAATCTTTGGGCACCTAGTGGTCATG

1 CATGACCACTAGGAGCATCTTTGGCGAGATCGGGAGAATCGGTGGCATTGCTGTCTACTAGGGGAATAAATCTTTGGGCACCTAGTGGTCATG

1 CATGACCACTAGGAGCATCTTTGGCGAGAAGACTCTGTATTCGGGGACCAGATGCTGCTAGGGGAATAAATCTTTGGGCACCTAGTGGTCATG

1 CATGACCACTAGGAGCATCTTTGGCGACGACGATAGGATGGCGCTGATCGCTTAGCCCTAGGGGAATAAATCTTTGGGCACCTAGTGGTCATG

1 CATGACCACTAGGAGCATCTTTGGCGATCCTCGTCAGATGGTGAAGCAGACGTGTGGCTAGGGGAATAAATCTTTGGGCACCTAGTGGTCATG

1 CATGACCACTAGGAGCATCTTTGGCGAAACGGCCTGTGACCTGATAAGTCCCGCTGGCTAGGGGAATAAATCTTTGGGCACCTAGTGGTCATG

1 CATGACCACTAGGAGCATCTTTGGCGATAACTGGGGCGTACATGTGGATTTAGCCGGCTAGGGGAATAAATCTTTGGGCACCTAGTGGTCATG

1 CATGACCACTAGGAGCATCTTTGGCGAAGCAGTGCGACTGGCTATGGGTTCCTCGAGCTAGGGGAATAAATCTTTGGGCACCTAGTGGTCATG

1 CATGACCACTAGGAGCATCTTTGGCGACCAGATATGGTAGTTAATGTGCTGAGGAGGCTAGGGGAATAAATCTTTGGGCACCTAGTGGTCATG

1 CATGACCACTAGGAGCATCTTTGGCGAACGGCAGTTGTTGCGGTGGTCTGTGAAACCCTAGGGGAATAAATCTTTGGGCACCTAGTGGTCATG

1 CATGACCACTAGGAGCATCTTTGGCGATGGCTGCTGAATGCCGCCGCTGCGGCTAAACTAGGGGAATAAATCTTTGGGCACCTAGTGGTCATG

1 CATGACCACTAGGAGCATCTTTGGCGAACGGCAAGTGTTGCGGTGGTCTTTGAATCCCTAGGGGAATAAATCTTTGGGCACCTAGTGGTCATG

1 CATGACCACTAGGAGCATCTTTGGCGAAAGGCACGTGTTGCGGTGGTCTGTGAATCCCTAGGGGAATAAATCTTTGGGCACCTAGTGGTCATG

1 CATGACCACTAGGAGCATCTTTGGCGAGAAGACTCTGGCTTCGGGGAGCAGTTGCTGCTAGGGGAATAAATCTTTGGGCACCTAGTGGTCATG

1 CATGACCACTAGGAGCATCTTTGGCGAGAAGACTTTGGATTCGGGGACCAGTTGCGGCTAGGGGAATAAATCTTTGGGCACCTAGTGGTCATG

1 CATGACCACTAGGAGCATCTTTGGCGACGGTGGGCTGGGAGGCTAGTATTTCGTCTCCTAGGGGAATAAATCTTTGGGCACCTAGTGGTCATG

1 CATGACCACTAGGAGCATCTTTGGCGAGATCGGGAGAATCGGTGGCATTGGTGAAACCTAGGGGAATAAATCTTTGGGCACCTAGTGGTCATG

1 CATGACCACTAGGAGCATCTTTGGCGAAGGGTGTAGGACTTCAGGTGGATCTCATAGCTAGGGGAATAAATCTTTGGGCACCTAGTGGTCATG

1 CATGACCACTAGGAGCATCTTTGGCGAACGGCAGGTGTTGCGGCGGTCTGTGGATCCCTAGGGGAATAAATCTTTGGGCACCTAGTGGTCATG

1 CATGACCACTAGGAGCATCTTTGGCGACTTCCTTCGTTTTGGACTTTTGCGCTGTTTCTAGGGGAATAAATCTTTGGGCACCTAGTGGTCATG

1 CATGACCACTAGGAGCATCTTTGGCGACGCGGGTGCAACAAGCTAGCAGACGTTTGGCTAGGGGAATAAATCTTTGGGCACCTAGTGGTCATG

1 CATGACCACTAGGAGCATCTTTGGCGAGTATTGGCTGGTAGGTTGCGTATTAGGGAGCTAGGGGAATAAATCTTTGGGCACCTAGTGGTCATG

1 CATGACCACTAGGAGCATCTTTGGCGAGAACGCTAGAATCGCTGGGAGAGGTGTCTCCTAGGGGAATAAATCTTTGGGCACCTAGTGGTCATG

1 CATGACCACTAGGAGCATCTTTGGCGAACGGCAGGAGTTGCGGTGGTCTGTGAATCTCTAGGGGAATAAATCTTTGGGCACCTAGTGGTCATG

1 CATGACCACTAGGAGCATCTTTGGCGAGATAGTCCATCGATGTTGGGGGAGAAGGGCCTAGGGGAATAAATCTTTGGGCACCTAGTGGTCATG

1 CATGACCACTAGGAGCATCTTTGGCGAACGGCAGGTGTTGCGGTGGTCTGTGTATCTCTAGGGGAATAAATCTTTGGGCACCTAGTGGTCATG

1 CATGACCACTAGGAGCATCTTTGGCGAGATCGGGAGAATCGGCGGCATTGGTGACTCCTAGGGGAATAAATCTTTGGGCACCTAGTGGTCATG

1 CATGACCACTAGGAGCATCTTTGGCGAGCCTCGCTTGGGAGGTTTCTCCACCAGTTCCTAGGGGAATAAATCTTTGGGCACCTAGTGGTCATG

1 CATGACCACTAGGAGCATCTTTGGCGAGATCGGGAGAATCGGTGGCACGGGTGTCTCCTAGGGGAATAAATCTTTGGGCACCTAGTGGTCATG

1 CATGACCACTAGGAGCATCTTTGGCGAAAGTCGGTAGAACAGGGTGGGTTGCAGTACCTAGGGGAATAAATCTTTGGGCACCTAGTGGTCATG

1 CATGACCACTAGGAGCATCTTTGGCGAACGGGGCGATGCAGTACCGTTTTCCCTGGTCTAGGGGAATAAATCTTTGGGCACCTAGTGGTCATG

1 CATGACCACTAGGAGCATCTTTGGCGAAGCGCATAGGCACAGTGGCTGCGTGGACTACTAGGGGAATAAATCTTTGGGCACCTAGTGGTCATG

1 CATGACCACTAGGAGCATCTTTGGCGAATCTTCATTGATTCGGGCATCGTAGACTTACTAGGGGAATAAATCTTTGGGCACCTAGTGGTCATG

1 CATGACCACTAGGAGCATCTTTGGCGAACGTCCAAGTGTGGTCATTGGAATTGGAACCTAGGGGAATAAATCTTTGGGCACCTAGTGGTCATG

1 CATGACCACTAGGAGCATCTTTGGCGAGGGCCTAATACTTCACTCCGGTTGCGCTGCCTAGGGGAATAAATCTTTGGGCACCTAGTGGTCATG

1 CATGACCACTAGGAGCATCTTTGGCGACAAGCTAAATGATAAGCGCGCCGTAGTGCCCTAGGGGAATAAATCTTTGGGCACCTAGTGGTCATG

1 CATGACCACTAGGAGCATCTTTGGCGAATCAGCAAGATCGGAGGGAAATTACTGTTGCTAGGGGAATAAATCTTTGGGCACCTAGTGGTCATG

1 CATGACCACTAGGAGCATCTTTGGCGACGCGAGGGGTCATGCGTATATGGCGGCGCACTAGGGGAATAAATCTTTGGGCACCTAGTGGTCATG

1 CATGACCACTAGGAGCATCTTTGGCGAGGGATGGGAGCCGGTGGGGGTTCGGGGTTTCTAGGGGAATAAATCTTTGGGCACCTAGTGGTCATG

1 CATGACCACTAGGAGCATCTTTGGCGAACTGCAGGTGTTGCGGTGGACTGTGAATCCCTAGGGGAATAAATCTTTGGGCACCTAGTGGTCATG

1 CATGACCACTAGGAGCATCTTTGGCGACCAGATATGGTTGGTAATGTGCTGAGGAGGCTAGGGGAATAAATCTTTGGGCACCTAGTGGTCATG

1 CATGACCACTAGGAGCATCTTTGGCGATCGTATGGGACGTATTACATACACGTCTTCCTAGGGGAATAAATCTTTGGGCACCTAGTGGTCATG

1 CATGACCACTAGGAGCATCTTTGGCGATTACAGGTTTGGTGTCATCTTCATAAATAGCTAGGGGAATAAATCTTTGGGCACCTAGTGGTCATG

1 CATGACCACTAGGAGCATCTTTGGCGAACGGCAGGCGTTGCGGTGGTCTTTGAATCCCTAGGGGAATAAATCTTTGGGCACCTAGTGGTCATG

1 CATGACCACTAGGAGCATCTTTGGCGATGGTTGAAAGCGAATCCCTTTCGGGCTGCCCTAGGGGAATAAATCTTTGGGCACCTAGTGGTCATG

1 CATGACCACTAGGAGCATCTTTGGCGAGATCGGGAGAATCTGTGGCCTTGGTGTCTCCTAGGGGAATAAATCTTTGGGCACCTAGTGGTCATG

1 CATGACCACTAGGAGCATCTTTGGCGACCGTAGGAAAACAAAAGTGGCCTAGCTAGCCTAGGGGAATAAATCTTTGGGCACCTAGTGGTCATG

1 CATGACCACTAGGAGCATCTTTGGCGATATCGGGAGAATCGGTTGCATTGGTGTCTCCTAGGGGAATAAATCTTTGGGCACCTAGTGGTCATG

1 CATGACCACTAGGAGCATCTTTGGCGAGAAGTGGTCGTGTGGACAGGTGCAGTCGAGCTAGGGGAATAAATCTTTGGGCACCTAGTGGTCATG

1 CATGACCACTAGGAGCATCTTTGGCGACTCGCGTAATGAGGCTCGAATGTCCGGTGACTAGGGGAATAAATCTTTGGGCACCTAGTGGTCATG

1 CATGACCACTAGGAGCATCTTTGGCGAGATCGGGAGAACCGGTGGCATTGGTGTTTCCTAGGGGAATAAATCTTTGGGCACCTAGTGGTCATG

1 CATGACCACTAGGAGCATCTTTGGCGAGATCGGGAGAAACGTTGGAATTGGTGTCTCCTAGGGGAATAAATCTTTGGGCACCTAGTGGTCATG

1 CATGACCACTAGGAGCATCTTTGGCGAGAGAATCGGTAGGCAGGGTGCTCCAGCCAGCTAGGGGAATAAATCTTTGGGCACCTAGTGGTCATG

1 CATGACCACTAGGAGCATCTTTGGCGAGATCGGTGGAATCGGTGGCATTGGTGTCTCCTAGGGGAATAAATCTTTGGGCACCTAGTGGTCATG

1 CATGACCACTAGGAGCATCTTTGGCGAGAAGACTTTGGATTCGGGGACCAGTTGCTACTAGGGGAATAAATCTTTGGGCACCTAGTGGTCATG

1 CATGACCACTAGGAGCATCTTTGGCGAGATCGGTAGGATCGGTGGCATTGGTGTCTCCTAGGGGAATAAATCTTTGGGCACCTAGTGGTCATG

1 CATGACCACTAGGAGCATCTTTGGCGACGACGGGGGAATGGACTCACAAAATGTCGCCTAGGGGAATAAATCTTTGGGCACCTAGTGGTCATG

1 CATGACCACTAGGAGCATCTTTGGCGACGGGTGGGCCCTAACCGAATGGACTCGTCACTAGGGGAATAAATCTTTGGGCACCTAGTGGTCATG

1 CATGACCACTAGGAGCATCTTTGGCGAGACGACTCTGGATTCGGGGACCAGTTGCGGCTAGGGGAATAAATCTTTGGGCACCTAGTGGTCATG

1 CATGACCACTAGGAGCATCTTTGGCGACCCTATGTTACAGCGTAAAGTAAGGGTGGACTAGGGGAATAAATCTTTGGGCACCTAGTGGTCATG

1 CATGACCACTAGGAGCATCTTTGGCGAGGTCGGGAGAATCGGTTGCATTGGTGTCCCCTAGGGGAATAAATCTTTGGGCACCTAGTGGTCATG

1 CATGACCACTAGGAGCATCTTTGGCGAATGGCCGGCACGGTTTTCTAGTCCTCGGTACTAGGGGAATAAATCTTTGGGCACCTAGTGGTCATG

1 CATGACCACTAGGAGCATCTTTGGCGAGATCGGGAGGATCGGTGGCATTGGTGGCTCCTAGGGGAATAAATCTTTGGGCACCTAGTGGTCATG

1 CATGACCACTAGGAGCATCTTTGGCGAGATCAGGAGAAAAGCTGGCATTGGTTTCTCCTAGGGGAATAAATCTTTGGGCACCTAGTGGTCATG

1 CATGACCACTAGGAGCATCTTTGGCGAGGGGGGGGCGACGGCCGGTAGTGGGTGAGACTAGGGGAATAAATCTTTGGGCACCTAGTGGTCATG

1 CATGACCACTAGGAGCATCTTTGGCGATTGGCCGGCACGGTCTTCTAGTCCTCGGTACTAGGGGAATAAATCTTTGGGCACCTAGTGGTCATG

1 CATGACCACTAGGAGCATCTTTGGCGACCAGATATGGTAGGTAATGTGCCGAGGAGGCTAGGGGAATAAATCTTTGGGCACCTAGTGGTCATG

1 CATGACCACTAGGAGCATCTTTGGCGATAGCGAAGTGCTGTGAACAGTGGTTAGCGCCTAGGGGAATAAATCTTTGGGCACCTAGTGGTCATG

1 CATGACCACTAGGAGCATCTTTGGCGAGATTGGGACAATCGGTGGCATTGGTGTCTCCTAGGGGAATAAATCTTTGGGCACCTAGTGGTCATG

1 CATGACCACTAGGAGCATCTTTGGCGAGATCGGGCGAATCGGTGGCATTGGTGTATCCTAGGGGAATAAATCTTTGGGCACCTAGTGGTCATG

1 CATGACCACTAGGAGCATCTTTGGCGAGTCAAGATTAACGTTGGTGCAGTCAAACCACTAGGGGAATAAATCTTTGGGCACCTAGTGGTCATG

1 CATGACCACTAGGAGCATCTTTGGCGAGCCTCGCTTGGGAAGTTGCTCCACCAGTTCCTAGGGGAATAAATCTTTGGGCACCTAGTGGTCATG

1 CATGACCACTAGGAGCATCTTTGGCGAACTCACGGACGAGGATATTCTGCCGCTACCCTAGGGGAATAAATCTTTGGGCACCTAGTGGTCATG

1 CATGACCACTAGGAGCATCTTTGGCGAAGACTCGCCGTATCAAGCCGTAGGCAGCCCCTAGGGGAATAAATCTTTGGGCACCTAGTGGTCATG

1 CATGACCACTAGGAGCATCTTTGGCGAGAAGATCCTGGATTCGGGGACCAGTTGCTGCTAGGGGAATAAATCTTTGGGCACCTAGTGGTCATG

1 CATGACCACTAGGAGCATCTTTGGCGAACGGCAGGCGTTGCGGTGCTCTGTGAATCCCTAGGGGAATAAATCTTTGGGCACCTAGTGGTCATG

1 CATGACCACTAGGAGCATCTTTGGCGAACACGAGAGTCAAGTGCGTATAGCTTGGAGCTAGGGGAATAAATCTTTGGGCACCTAGTGGTCATG

1 CATGACCACTAGGAGCATCTTTGGCGAGCCTTGCTTGTGAGGTTGCTCCACCAGTTCCTAGGGGAATAAATCTTTGGGCACCTAGTGGTCATG

1 CATGACCACTAGGAGCATCTTTGGCGACTCGTATTGGGATGTCAGGGTAACACTTTCCTAGGGGAATAAATCTTTGGGCACCTAGTGGTCATG

1 CATGACCACTAGGAGCATCTTTGGCGACGTCCATGTGATGGCTAAATCGTGCTCAGCCTAGGGGAATAAATCTTTGGGCACCTAGTGGTCATG

1 CATGACCACTAGGAGCATCTTTGGCGAGTGTGGGCTGGTAGTGCGTACTTGATCGCTCTAGGGGAATAAATCTTTGGGCACCTAGTGGTCATG

1 CATGACCACTAGGAGCATCTTTGGCGATATCTGGAGAATCGGTGCCATTGGTGTCTCCTAGGGGAATAAATCTTTGGGCACCTAGTGGTCATG

1 CATGACCACTAGGAGCATCTTTGGCGACGTATGTGGGCATCGGTAGGCGGAGGAGCTCTAGGGGAATAAATCTTTGGGCACCTAGTGGTCATG

1 CATGACCACTAGGAGCATCTTTGGCGACAAGTGGTACTGGTCCAACGCGACATAGTGCTAGGGGAATAAATCTTTGGGCACCTAGTGGTCATG

1 CATGACCACTAGGAGCATCTTTGGCGAGCCTTGCTTGGGAGGTTGCTGCACCAGTTCCTAGGGGAATAAATCTTTGGGCACCTAGTGGTCATG

1 CATGACCACTAGGAGCATCTTTGGCGATCGCAAAGCAAATCTCGTAGTGGATTAGGCCTAGGGGAATAAATCTTTGGGCACCTAGTGGTCATG

1 CATGACCACTAGGAGCATCTTTGGCGAGATCGGGCTAGTCTGTGGCAGAGGTGTCTCCTAGGGGAATAAATCTTTGGGCACCTAGTGGTCATG

1 CATGACCACTAGGAGCATCTTTGGCGATAGCGAAGTGCTGTGGACAGTGGTTAGCACCTAGGGGAATAAATCTTTGGGCACCTAGTGGTCATG

1 CATGACCACTAGGAGCATCTTTGGCGAGAGCGGGAGACTCGGTGGCATTGGTGTCTCCTAGGGGAATAAATCTTTGGGCACCTAGTGGTCATG

1 CATGACCACTAGGAGCATCTTTGGCGAGGTTTATTAGAGGGACGGATGTAGCTGATTCTAGGGGAATAAATCTTTGGGCACCTAGTGGTCATG

1 CATGACCACTAGGAGCATCTTTGGCGAGAACGGGATAGTCGGTGGCATAGGTGTCTCCTAGGGGAATAAATCTTTGGGCACCTAGTGGTCATG

1 CATGACCACTAGGAGCATCTTTGGCGAACGGCAGGTGTTGCGGTGGTCAGAGAATCCCTAGGGGAATAAATCTTTGGGCACCTAGTGGTCATG

1 CATGACCACTAGGAGCATCTTTGGCGAGATCGGGAGAGTCGGTGGCACTGGTGTCTCCTAGGGGAATAAATCTTTGGGCACCTAGTGGTCATG

1 CATGACCACTAGGAGCATCTTTGGCGAGATCGGGAGAATCGATGGCATAGGTGTCTCCTAGGGGAATAAATCTTTGGGCACCTAGTGGTCATG

1 CATGACCACTAGGAGCATCTTTGGCGAACCTACAGGAGCACCGCGAATCGGATGAGACTAGGGGAATAAATCTTTGGGCACCTAGTGGTCATG

1 CATGACCACTAGGAGCATCTTTGGCGAATGGCCGGCACGGCCTTCTAGTTTTCGGTACTAGGGGAATAAATCTTTGGGCACCTAGTGGTCATG

1 CATGACCACTAGGAGCATCTTTGGCGAGGTACGAAGCCGAGGCTATGGCTGAGTTTACTAGGGGAATAAATCTTTGGGCACCTAGTGGTCATG

1 CATGACCACTAGGAGCATCTTTGGCGATCCGGGGGTTAGATAAGACGGCGCGTTGGGCTAGGGGAATAAATCTTTGGGCACCTAGTGGTCATG

1 CATGACCACTAGGAGCATCTTTGGCGAGAAGACTATGGAATCGGGGACCAGTTGCTGCTAGGGGAATAAATCTTTGGGCACCTAGTGGTCATG

1 CATGACCACTAGGAGCATCTTTGGCGAGACCACGATGGTGGGCGTTTCTGTCTATTCCTAGGGGAATAAATCTTTGGGCACCTAGTGGTCATG

1 CATGACCACTAGGAGCATCTTTGGCGACCCTTACGGATGGGAAGAAAATGATCGATACTAGGGGAATAAATCTTTGGGCACCTAGTGGTCATG

1 CATGACCACTAGGAGCATCTTTGGCGACGGTGTGGGGCTATAGATGCGGCGGCACTCCTAGGGGAATAAATCTTTGGGCACCTAGTGGTCATG

1 CATGACCACTAGGAGCATCTTTGGCGAGGGAGTGGTCGTCCATGTGGGGGGTCGGGGCTAGGGGAATAAATCTTTGGGCACCTAGTGGTCATG

1 CATGACCACTAGGAGCATCTTTGGCGAGGGAGGGCGCCGGAAGCGGTGTGAATGCGACTAGGGGAATAAATCTTTGGGCACCTAGTGGTCATG

1 CATGACCACTAGGAGCATCTTTGGCGACAGGGGACGCCGATGCGCTGGACGACCGAGCTAGGGGAATAAATCTTTGGGCACCTAGTGGTCATG

1 CATGACCACTAGGAGCATCTTTGGCGAACGGCAGGTGTTGCGGTGGTCTGTAGATCCCTAGGGGAATAAATCTTTGGGCACCTAGTGGTCATG

1 CATGACCACTAGGAGCATCTTTGGCGAAGTGTGGAACATGCCGGTTTTGGTTGCAGCCTAGGGGAATAAATCTTTGGGCACCTAGTGGTCATG

1 CATGACCACTAGGAGCATCTTTGGCGAGATCGGGAGAATCGCTGGCATTGGTGTCACCTAGGGGAATAAATCTTTGGGCACCTAGTGGTCATG

1 CATGACCACTAGGAGCATCTTTGGCGAGAAGACTCCGGATTCGTGGACCAGTTGCTGCTAGGGGAATAAATCTTTGGGCACCTAGTGGTCATG

1 CATGACCACTAGGAGCATCTTTGGCGACTCGTGGTCTTTACCCCCGTAGAGGGTTGTCTAGGGGAATAAATCTTTGGGCACCTAGTGGTCATG

1 CATGACCACTAGGAGCATCTTTGGCGAGGGAGGGCGCCGGCAGCGGTGTGTATGCGCCTAGGGGAATAAATCTTTGGGCACCTAGTGGTCATG

1 CATGACCACTAGGAGCATCTTTGGCGACATAGGATGCGACGCTGAGTGGTAAGTCGACTAGGGGAATAAATCTTTGGGCACCTAGTGGTCATG

1 CATGACCACTAGGAGCATCTTTGGCGACTTAGTGGACTGCGATTCAAAAAAGGGAGCCTAGGGGAATAAATCTTTGGGCACCTAGTGGTCATG

1 CATGACCACTAGGAGCATCTTTGGCGAGAAGACTCAGGATTCGGGGACAAGTTGCTGCTAGGGGAATAAATCTTTGGGCACCTAGTGGTCATG

1 CATGACCACTAGGAGCATCTTTGGCGACGTAGTGGGCTGGCAGGGGGTACTTGTTGCCTAGGGGAATAAATCTTTGGGCACCTAGTGGTCATG

1 CATGACCACTAGGAGCATCTTTGGCGACAGTGGGCAGCAGAGGCGCAGGAAGGTCGACTAGGGGAATAAATCTTTGGGCACCTAGTGGTCATG

1 CATGACCACTAGGAGCATCTTTGGCGAATCTCTCTGGGTGCACGCAATTTGGCAGCTCTAGGGGAATAAATCTTTGGGCACCTAGTGGTCATG

1 CATGACCACTAGGAGCATCTTTGGCGACCCAGACTGATGTAATTTGTAATGGGTGGACTAGGGGAATAAATCTTTGGGCACCTAGTGGTCATG

1 CATGACCACTAGGAGCATCTTTGGCGAGATCAGGAGAATCGGTGGCATTGTTGTCTCCTAGGGGAATAAATCTTTGGGCACCTAGTGGTCATG

1 CATGACCACTAGGAGCATCTTTGGCGACGGCGGAGCCCGCTCTGGTGGTCGAGAGATCTAGGGGAATAAATCTTTGGGCACCTAGTGGTCATG

1 CATGACCACTAGGAGCATCTTTGGCGACGTGGGTGCGTCTGCAACCGTTGGGTGGAGCTAGGGGAATAAATCTTTGGGCACCTAGTGGTCATG

1 CATGACCACTAGGAGCATCTTTGGCGACGCGTGATCTTCGTAGTCAGCCAAGTGTTCCTAGGGGAATAAATCTTTGGGCACCTAGTGGTCATG

1 CATGACCACTAGGAGCATCTTTGGCGATATTGAGGGGGAGTTATACACCTTTCTCAACTAGGGGAATAAATCTTTGGGCACCTAGTGGTCATG

1 CATGACCACTAGGAGCATCTTTGGCGACGGTGCGGGGACCGCAGGTTCGGCGGGTACCTAGGGGAATAAATCTTTGGGCACCTAGTGGTCATG

1 CATGACCACTAGGAGCATCTTTGGCGACTCGTGGGCATATATGGGCCGCGTAGTGGACTAGGGGAATAAATCTTTGGGCACCTAGTGGTCATG

1 CATGACCACTAGGAGCATCTTTGGCGAGTAGAAGGGGGCATGGATCTGTATACGCGACTAGGGGAATAAATCTTTGGGCACCTAGTGGTCATG

1 CATGACCACTAGGAGCATCTTTGGCGACGGTGTGGGGCACTTCTCGCGGCGGTTATTCTAGGGGAATAAATCTTTGGGCACCTAGTGGTCATG

1 CATGACCACTAGGAGCATCTTTGGCGACAGTGGTGTAGGCAGGGTTGGTGGGACTAGCTAGGGGAATAAATCTTTGGGCACCTAGTGGTCATG

1 CATGACCACTAGGAGCATCTTTGGCGAAAGTCGGTAGAACAGGGTTCGGTGCTGTCCCTAGGGGAATAAATCTTTGGGCACCTAGTGGTCATG

1 CATGACCACTAGGAGCATCTTTGGCGAATGGTGGATTACCGCGAGCGTTTCCACGGGCTAGGGGAATAAATCTTTGGGCACCTAGTGGTCATG

1 CATGACCACTAGGAGCATCTTTGGCGAATGAGCTACGCGCCACGTCTAATCGGTTCACTAGGGGAATAAATCTTTGGGCACCTAGTGGTCATG

1 CATGACCACTAGGAGCATCTTTGGCGAATGGCAGGGGTTGCGGTGGTCTGTGAATCCCTAGGGGAATAAATCTTTGGGCACCTAGTGGTCATG

1 CATGACCACTAGGAGCATCTTTGGCGAGATCGGGAGAATCGGTGGCATTGGCGTCTTCTAGGGGAATAAATCTTTGGGCACCTAGTGGTCATG

1 CATGACCACTAGGAGCATCTTTGGCGAGGATAATCCTTCCCGTTTAGTCGGCCTCGACTAGGGGAATAAATCTTTGGGCACCTAGTGGTCATG

1 CATGACCACTAGGAGCATCTTTGGCGAGATCGGGAGAATCGGTGGCCTTGGTGTCTGCTAGGGGAATAAATCTTTGGGCACCTAGTGGTCATG

1 CATGACCACTAGGAGCATCTTTGGCGAGAAGATTCTGGATTCGGGGACTAGTTGCTGCTAGGGGAATAAATCTTTGGGCACCTAGTGGTCATG

1 CATGACCACTAGGAGCATCTTTGGCGAGCCGATTGTGCAACAATGTGTTTTGGGTAACTAGGGGAATAAATCTTTGGGCACCTAGTGGTCATG

1 CATGACCACTAGGAGCATCTTTGGCGAACGGCAGGTGTTGCGGTGGCCTGTGGATCCCTAGGGGAATAAATCTTTGGGCACCTAGTGGTCATG

1 CATGACCACTAGGAGCATCTTTGGCGAGCGGCAGGTGTTGCGTTGGTCTGTGAATCCCTAGGGGAATAAATCTTTGGGCACCTAGTGGTCATG

1 CATGACCACTAGGAGCATCTTTGGCGAACTAGGGGGAAGCGCAGTGCTTTCGTAGCTCTAGGGGAATAAATCTTTGGGCACCTAGTGGTCATG

1 CATGACCACTAGGAGCATCTTTGGCGAATGGCCGGCACGGCCTTCTAGTCCACGGTACTAGGGGAATAAATCTTTGGGCACCTAGTGGTCATG

1 CATGACCACTAGGAGCATCTTTGGCGAGGTAGAAAATGCATAGCAGCGAAAGAACAACTAGGGGAATAAATCTTTGGGCACCTAGTGGTCATG

1 CATGACCACTAGGAGCATCTTTGGCGACCCGAGGGGGCATGTTGTTCATAGGGTAGACTAGGGGAATAAATCTTTGGGCACCTAGTGGTCATG

1 CATGACCACTAGGAGCATCTTTGGCGAGATCTGGAGAATCGGTCGCATTGGTGTCTCCTAGGGGAATAAATCTTTGGGCACCTAGTGGTCATG

1 CATGACCACTAGGAGCATCTTTGGCGAGAAGACTCTGTCTTCGGGGACCAGTTGCTGCTAGGGGAATAAATCTTTGGGCACCTAGTGGTCATG

1 CATGACCACTAGGAGCATCTTTGGCGACGACGTGGGCATTGCCTAAAGTTAAAACGCCTAGGGGAATAAATCTTTGGGCACCTAGTGGTCATG

1 CATGACCACTAGGAGCATCTTTGGCGAGATCGGGAGAATCGGTGCCAATGGTGTCTCCTAGGGGAATAAATCTTTGGGCACCTAGTGGTCATG

1 CATGACCACTAGGAGCATCTTTGGCGACGGTGTGGGGCCAACTGTGCGGCGGATCCCCTAGGGGAATAAATCTTTGGGCACCTAGTGGTCATG

1 CATGACCACTAGGAGCATCTTTGGCGAGAGAATTCGAGCGATAGGTGTGTGCTCCCACTAGGGGAATAAATCTTTGGGCACCTAGTGGTCATG

1 CATGACCACTAGGAGCATCTTTGGCGAGATCGGGAGAATCGGAGGCATTGGTGACTCCTAGGGGAATAAATCTTTGGGCACCTAGTGGTCATG

1 CATGACCACTAGGAGCATCTTTGGCGACAACGGGAGAATCGGTGGCATTGGTGTCTCCTAGGGGAATAAATCTTTGGGCACCTAGTGGTCATG

1 CATGACCACTAGGAGCATCTTTGGCGATGGGTGCTGACGGCTGCTGCTGCGGCTACACTAGGGGAATAAATCTTTGGGCACCTAGTGGTCATG

1 CATGACCACTAGGAGCATCTTTGGCGAGAAGACCCCGGATTCGGGGACTAGTTGCTGCTAGGGGAATAAATCTTTGGGCACCTAGTGGTCATG

1 CATGACCACTAGGAGCATCTTTGGCGAGATAGGTAGAATAGGTGGCATTGGTGTCTCCTAGGGGAATAAATCTTTGGGCACCTAGTGGTCATG

1 CATGACCACTAGGAGCATCTTTGGCGAGATCTGGAGACTCGGTGGCATTGGTGTCTCCTAGGGGAATAAATCTTTGGGCACCTAGTGGTCATG

1 CATGACCACTAGGAGCATCTTTGGCGACGAGTGGGATGCATATCTCAGTGAAGTGGACTAGGGGAATAAATCTTTGGGCACCTAGTGGTCATG

1 CATGACCACTAGGAGCATCTTTGGCGAGATCGGGAGTATCGGAGGCATTGGTGTCTCCTAGGGGAATAAATCTTTGGGCACCTAGTGGTCATG

1 CATGACCACTAGGAGCATCTTTGGCGAGATCAGGAGAATCGGTGGCATTCGTGTCTCCTAGGGGAATAAATCTTTGGGCACCTAGTGGTCATG

1 CATGACCACTAGGAGCATCTTTGGCGACCGGGGAGGTACGGACTTGGTGGTATCAGACTAGGGGAATAAATCTTTGGGCACCTAGTGGTCATG

1 CATGACCACTAGGAGCATCTTTGGCGATGGGTGCTGACGTCCGCCGCTGCGGCTACACTAGGGGAATAAATCTTTGGGCACCTAGTGGTCATG

1 CATGACCACTAGGAGCATCTTTGGCGAGATCGGGAGAATCGGTGGCGTTGGTGACTCCTAGGGGAATAAATCTTTGGGCACCTAGTGGTCATG

1 CATGACCACTAGGAGCATCTTTGGCGAAAGTCGGTAGAACATGGTGGGGTGCCGTCCCTAGGGGAATAAATCTTTGGGCACCTAGTGGTCATG

1 CATGACCACTAGGAGCATCTTTGGCGACCTGCAGGTGTTGCGGTGGTCTGTGAATCCCTAGGGGAATAAATCTTTGGGCACCTAGTGGTCATG

1 CATGACCACTAGGAGCATCTTTGGCGAAAGGCTGGGGTTGGGGTGGACTGGTGAACCCTAGGGGAATAAATCTTTGGGCACCTAGTGGTCATG

1 CATGACCACTAGGAGCATCTTTGGCGAGATCGGGAGAATTGTTGGCATTGGTGTCTCCTAGGGGAATAAATCTTTGGGCACCTAGTGGTCATG

1 CATGACCACTAGGAGCATCTTTGGCGAACGTCAGGTGGTGCGGTGGTCTGTGAATCCCTAGGGGAATAAATCTTTGGGCACCTAGTGGTCATG

1 CATGACCACTAGGAGCATCTTTGGCGAGCGGCAGGTGTTGTGGTGGTCTGTGAATCCCTAGGGGAATAAATCTTTGGGCACCTAGTGGTCATG

1 CATGACCACTAGGAGCATCTTTGGCGAGATCGGTAGAATCGGTGGCATTGATGTCTCCTAGGGGAATAAATCTTTGGGCACCTAGTGGTCATG

1 CATGACCACTAGGAGCATCTTTGGCGAGTACACTCCTTTGGTTGGTTCTGGTCAAGGCTAGGGGAATAAATCTTTGGGCACCTAGTGGTCATG

1 CATGACCACTAGGAGCATCTTTGGCGATCCGGGGGCGCAAATGCGAGACGACTAGGGCTAGGGGAATAAATCTTTGGGCACCTAGTGGTCATG

1 CATGACCACTAGGAGCATCTTTGGCGAGATCGGGAGAATCGGTGCCATTGGTGTCTACTAGGGGAATAAATCTTTGGGCACCTAGTGGTCATG

1 CATGACCACTAGGAGCATCTTTGGCGAAGATCCGATGGGCTTGCGACCCGCCTCGGCCTAGGGGAATAAATCTTTGGGCACCTAGTGGTCATG

1 CATGACCACTAGGAGCATCTTTGGCGAACGGCAGGCGTTGCGGTGGTTTGTGAATCCCTAGGGGAATAAATCTTTGGGCACCTAGTGGTCATG

1 CATGACCACTAGGAGCATCTTTGGCGAGATCGGGAGAATCGGTGGCATTGTGGTCTCCTAGGGGAATAAATCTTTGGGCACCTAGTGGTCATG

1 CATGACCACTAGGAGCATCTTTGGCGACGTTGTGGGCCCACATGTGGCACCGTGGAGCTAGGGGAATAAATCTTTGGGCACCTAGTGGTCATG

1 CATGACCACTAGGAGCATCTTTGGCGAGATCAGGGGAATCGGTGGCATTGGTGTCTCCTAGGGGAATAAATCTTTGGGCACCTAGTGGTCATG

1 CATGACCACTAGGAGCATCTTTGGCGACGGCGTGGGGCCATCTGTGCGGAGGATCCCCTAGGGGAATAAATCTTTGGGCACCTAGTGGTCATG

1 CATGACCACTAGGAGCATCTTTGGCGAACTATGTTGCCTAGCGCGTTGCCGATGCCTCTAGGGGAATAAATCTTTGGGCACCTAGTGGTCATG

1 CATGACCACTAGGAGCATCTTTGGCGAGGGGTCCTGGGAATGTAATTGCGCTTAAGACTAGGGGAATAAATCTTTGGGCACCTAGTGGTCATG

1 CATGACCACTAGGAGCATCTTTGGCGACGGCGTGGGGCTATTGACGCAGCGGCACTCCTAGGGGAATAAATCTTTGGGCACCTAGTGGTCATG

1 CATGACCACTAGGAGCATCTTTGGCGAGAAGACTCTGGATTCGGGGACCAGTTGCACCTAGGGGAATAAATCTTTGGGCACCTAGTGGTCATG

1 CATGACCACTAGGAGCATCTTTGGCGAGAAGACTCTGGATTCGGGGACCAGTTGTTCCTAGGGGAATAAATCTTTGGGCACCTAGTGGTCATG

1 CATGACCACTAGGAGCATCTTTGGCGAGAAGACTCTGGATTCGGGGACCAGTCGCAGCTAGGGGAATAAATCTTTGGGCACCTAGTGGTCATG

1 CATGACCACTAGGAGCATCTTTGGCGACAAGTGACAGAGGTAGAACGTGACGTCGTGCTAGGGGAATAAATCTTTGGGCACCTAGTGGTCATG

1 CATGACCACTAGGAGCATCTTTGGCGACTACTCACGTTGTGGCGCCAATTGGTTCGTCTAGGGGAATAAATCTTTGGGCACCTAGTGGTCATG

1 CATGACCACTAGGAGCATCTTTGGCGAGATCGTTAGATTCGGTGGCATTGGTGTCTCCTAGGGGAATAAATCTTTGGGCACCTAGTGGTCATG

1 CATGACCACTAGGAGCATCTTTGGCGAGGCGGGGGGAGGCCGGCCTATGCTCCGAGACTAGGGGAATAAATCTTTGGGCACCTAGTGGTCATG

1 CATGACCACTAGGAGCATCTTTGGCGATATCGGGAGAATCGGAGGCATTGGTGTCTCCTAGGGGAATAAATCTTTGGGCACCTAGTGGTCATG

1 CATGACCACTAGGAGCATCTTTGGCGAGATCGGGAGAATCGGTGGCCGTGGTGTCTCCTAGGGGAATAAATCTTTGGGCACCTAGTGGTCATG

1 CATGACCACTAGGAGCATCTTTGGCGAATGGCCGGCACGGCCTTCAAGTCCTCGGTACTAGGGGAATAAATCTTTGGGCACCTAGTGGTCATG

1 CATGACCACTAGGAGCATCTTTGGCGATTCTTCTGGGTTTTAGAAATATGGGGCTGACTAGGGGAATAAATCTTTGGGCACCTAGTGGTCATG

1 CATGACCACTAGGAGCATCTTTGGCGATCGCTGAGCGGCTGACCAAAGGTCTCGGGCCTAGGGGAATAAATCTTTGGGCACCTAGTGGTCATG

1 CATGACCACTAGGAGCATCTTTGGCGATACCAGAGGTGAGCGAAGGTGTGCGATCTACTAGGGGAATAAATCTTTGGGCACCTAGTGGTCATG

1 CATGACCACTAGGAGCATCTTTGGCGACAAAGAGGTGTAAATGTGGCAGAGTCGCCGCTAGGGGAATAAATCTTTGGGCACCTAGTGGTCATG

1 CATGACCACTAGGAGCATCTTTGGCGATGGGGCCGTGCTATCCGCACACTCCGCGGGCTAGGGGAATAAATCTTTGGGCACCTAGTGGTCATG

1 CATGACCACTAGGAGCATCTTTGGCGAGGTCGGGAGAATCGGTGGCATTTGTGTCTCCTAGGGGAATAAATCTTTGGGCACCTAGTGGTCATG

1 CATGACCACTAGGAGCATCTTTGGCGAAAGTAGGTAGAACAGGGTGGGGTGTTGTCCCTAGGGGAATAAATCTTTGGGCACCTAGTGGTCATG

1 CATGACCACTAGGAGCATCTTTGGCGATACCGGGCGCGAAGCGCGCGTGGTGGAAGACTAGGGGAATAAATCTTTGGGCACCTAGTGGTCATG

1 CATGACCACTAGGAGCATCTTTGGCGAGATCGGGAGAATCGGTGGCATTGGTGAATCCTAGGGGAATAAATCTTTGGGCACCTAGTGGTCATG

1 CATGACCACTAGGAGCATCTTTGGCGAAAGTCGGTAGAGCAGTGTGGGGTGCTGTCCCTAGGGGAATAAATCTTTGGGCACCTAGTGGTCATG

1 CATGACCACTAGGAGCATCTTTGGCGAACGTCCGTAGATGGCTACAGGAATTGGAACCTAGGGGAATAAATCTTTGGGCACCTAGTGGTCATG

1 CATGACCACTAGGAGCATCTTTGGCGATCCGGGGGCATGCACGTGCGACGTCCTGGGCTAGGGGAATAAATCTTTGGGCACCTAGTGGTCATG

1 CATGACCACTAGGAGCATCTTTGGCGAGATCGGGAGAATCGGTGGCATTGGTGACACCTAGGGGAATAAATCTTTGGGCACCTAGTGGTCATG

1 CATGACCACTAGGAGCATCTTTGGCGATGGGGCCGTGCTATCTGCAGACTCCGCGGGCTAGGGGAATAAATCTTTGGGCACCTAGTGGTCATG

1 CATGACCACTAGGAGCATCTTTGGCGATCCGGGGGCCCCTGTGTGCGACGCTGTGGGCTAGGGGAATAAATCTTTGGGCACCTAGTGGTCATG

1 CATGACCACTAGGAGCATCTTTGGCGACCCAGATTGCAAAGAAGGGTGGTGGATCGCCTAGGGGAATAAATCTTTGGGCACCTAGTGGTCATG

1 CATGACCACTAGGAGCATCTTTGGCGACGACAGGGGATGCAGGTGTGGTTGAGGAGACTAGGGGAATAAATCTTTGGGCACCTAGTGGTCATG

1 CATGACCACTAGGAGCATCTTTGGCGAGATCGTGAGAATCGGTGGCATTGGAGTCTCCTAGGGGAATAAATCTTTGGGCACCTAGTGGTCATG

1 CATGACCACTAGGAGCATCTTTGGCGAGGTCGTGGGCGTGCGTGAAATGGAAGTCGACTAGGGGAATAAATCTTTGGGCACCTAGTGGTCATG

1 CATGACCACTAGGAGCATCTTTGGCGAACGGCAGGTGTAGCGGTGGTCTTTGAATCCCTAGGGGAATAAATCTTTGGGCACCTAGTGGTCATG

1 CATGACCACTAGGAGCATCTTTGGCGAGCGGTTCGCCCGGTAGAGGGGTCTCTTACGCTAGGGGAATAAATCTTTGGGCACCTAGTGGTCATG

1 CATGACCACTAGGAGCATCTTTGGCGAGAAGAATCTGGATTCGGTGACCAGTTGCTGCTAGGGGAATAAATCTTTGGGCACCTAGTGGTCATG

1 CATGACCACTAGGAGCATCTTTGGCGAGATCGCGAGAATCGGTGGCATTGGTGTCTACTAGGGGAATAAATCTTTGGGCACCTAGTGGTCATG

1 CATGACCACTAGGAGCATCTTTGGCGAGAAGACTCTGGAATCGGGGACCAGATGATTCTAGGGGAATAAATCTTTGGGCACCTAGTGGTCATG

1 CATGACCACTAGGAGCATCTTTGGCGACCGTAGGAAAACAAAAGTGGCCTTGTTAGCCTAGGGGAATAAATCTTTGGGCACCTAGTGGTCATG

1 CATGACCACTAGGAGCATCTTTGGCGAAGCCTACCCCGTCGTTGATGAGGTTTGATGCTAGGGGAATAAATCTTTGGGCACCTAGTGGTCATG

1 CATGACCACTAGGAGCATCTTTGGCGAACGGCAGGTGTTGCGGTGGTCGGTGGATCCCTAGGGGAATAAATCTTTGGGCACCTAGTGGTCATG

1 CATGACCACTAGGAGCATCTTTGGCGAGAACACTCGTATATCTGGCACAGGTTTCTGCTAGGGGAATAAATCTTTGGGCACCTAGTGGTCATG

1 CATGACCACTAGGAGCATCTTTGGCGACCGAGTGGTCTGGGAGGAAGTATTTCGGAGCTAGGGGAATAAATCTTTGGGCACCTAGTGGTCATG

1 CATGACCACTAGGAGCATCTTTGGCGAGCCTCGCTTGGGAGGTTGCTCCACCAGATCCTAGGGGAATAAATCTTTGGGCACCTAGTGGTCATG

1 CATGACCACTAGGAGCATCTTTGGCGAAGCGCATGGTGTGAGAGCGGCGTGTCCATGCTAGGGGAATAAATCTTTGGGCACCTAGTGGTCATG

1 CATGACCACTAGGAGCATCTTTGGCGAGATCGGGAGAATCGGCCGCATTGGTGTCTCCTAGGGGAATAAATCTTTGGGCACCTAGTGGTCATG

1 CATGACCACTAGGAGCATCTTTGGCGAACGGAAGGTGTTTCGGTTGTCTGTGAATCCCTAGGGGAATAAATCTTTGGGCACCTAGTGGTCATG

1 CATGACCACTAGGAGCATCTTTGGCGAAAATTAGAATATGAGTATCGTTTTCTCTGACTAGGGGAATAAATCTTTGGGCACCTAGTGGTCATG

1 CATGACCACTAGGAGCATCTTTGGCGATGGGTGCTGACGGCCGCCACTGCGGCTACACTAGGGGAATAAATCTTTGGGCACCTAGTGGTCATG

1 CATGACCACTAGGAGCATCTTTGGCGATCCCCGGCTTGTCTAACCCTCTCAGTTGACCTAGGGGAATAAATCTTTGGGCACCTAGTGGTCATG

1 CATGACCACTAGGAGCATCTTTGGCGACAATGTTAATGTGGTGTGATGCTTAGAATGCTAGGGGAATAAATCTTTGGGCACCTAGTGGTCATG

1 CATGACCACTAGGAGCATCTTTGGCGACTCGTGACCAGCCATAATGGCTGCCGGTGACTAGGGGAATAAATCTTTGGGCACCTAGTGGTCATG

1 CATGACCACTAGGAGCATCTTTGGCGAGATCGGGAGAATCGTTGGCATTGGTGCCTCCTAGGGGAATAAATCTTTGGGCACCTAGTGGTCATG

1 CATGACCACTAGGAGCATCTTTGGCGACTCTTAATGTACAGTGTTTAGGGTATATCCCTAGGGGAATAAATCTTTGGGCACCTAGTGGTCATG

1 CATGACCACTAGGAGCATCTTTGGCGAGAGAATCGGTAGGCAGAGTGCTCCGGCCAGCTAGGGGAATAAATCTTTGGGCACCTAGTGGTCATG

1 CATGACCACTAGGAGCATCTTTGGCGAGGTTAGGGGCTTCCTAGGATTACCGGGCCCCTAGGGGAATAAATCTTTGGGCACCTAGTGGTCATG

1 CATGACCACTAGGAGCATCTTTGGCGAGATCGCGAGAATCGGTGGCATTGGTGTCCCCTAGGGGAATAAATCTTTGGGCACCTAGTGGTCATG

1 CATGACCACTAGGAGCATCTTTGGCGACCCTGTAAGCTGACCAGCGACGAGGGTGGACTAGGGGAATAAATCTTTGGGCACCTAGTGGTCATG

1 CATGACCACTAGGAGCATCTTTGGCGAGACGACTCCGGATTCGGGGACCAGTTGCTGCTAGGGGAATAAATCTTTGGGCACCTAGTGGTCATG

1 CATGACCACTAGGAGCATCTTTGGCGAGATCGGTAGAATCGGTGGCATTAGTGTCTCCTAGGGGAATAAATCTTTGGGCACCTAGTGGTCATG

1 CATGACCACTAGGAGCATCTTTGGCGAGACCGGGAGAATCGGTGGCATTGGTGTCCCCTAGGGGAATAAATCTTTGGGCACCTAGTGGTCATG

1 CATGACCACTAGGAGCATCTTTGGCGACGGCGTGGGGCTATAGATGCGGCGGCATTCCTAGGGGAATAAATCTTTGGGCACCTAGTGGTCATG

1 CATGACCACTAGGAGCATCTTTGGCGACATCCGTTGATGACTCAGCAATGGTGTGGACTAGGGGAATAAATCTTTGGGCACCTAGTGGTCATG

1 CATGACCACTAGGAGCATCTTTGGCGACGATGGAGGTCATGCTTAGGGAGAAGGAGACTAGGGGAATAAATCTTTGGGCACCTAGTGGTCATG

1 CATGACCACTAGGAGCATCTTTGGCGATCGCAGGTCGGATGCAGCGTTTTGCCTTCCCTAGGGGAATAAATCTTTGGGCACCTAGTGGTCATG

1 CATGACCACTAGGAGCATCTTTGGCGAGATCAGGAGAATCGGTGGCAGTGGTGTCTCCTAGGGGAATAAATCTTTGGGCACCTAGTGGTCATG

1 CATGACCACTAGGAGCATCTTTGGCGAGGTGACCCCCGTGCGCTGGGGAGTGCACCTCTAGGGGAATAAATCTTTGGGCACCTAGTGGTCATG

1 CATGACCACTAGGAGCATCTTTGGCGAGATCGGGAGAATCGTTGGCATTGGTGACTCCTAGGGGAATAAATCTTTGGGCACCTAGTGGTCATG

1 CATGACCACTAGGAGCATCTTTGGCGAGAACTGGAGAATCGGTGGCATTGCTGTCTCCTAGGGGAATAAATCTTTGGGCACCTAGTGGTCATG

1 CATGACCACTAGGAGCATCTTTGGCGACAGCGTAGCCGTTAAAGCTGTAAAGTGGAGCTAGGGGAATAAATCTTTGGGCACCTAGTGGTCATG

1 CATGACCACTAGGAGCATCTTTGGCGAACGACTACGATACTGCCGATGTCTTGGAACCTAGGGGAATAAATCTTTGGGCACCTAGTGGTCATG

1 CATGACCACTAGGAGCATCTTTGGCGACGTGGCTGGTAGGGTCAGTACTGTGTCGATCTAGGGGAATAAATCTTTGGGCACCTAGTGGTCATG

1 CATGACCACTAGGAGCATCTTTGGCGAGGCATCGTGGAAGGATCCATCGTGGCGTCTCTAGGGGAATAAATCTTTGGGCACCTAGTGGTCATG

1 CATGACCACTAGGAGCATCTTTGGCGATGGGTGCTGACGGCCGCCGCTGTGGCTACACTAGGGGAATAAATCTTTGGGCACCTAGTGGTCATG

1 CATGACCACTAGGAGCATCTTTGGCGACGGGATGCGCGGTGGTCTTTGGGAGTGTTGCTAGGGGAATAAATCTTTGGGCACCTAGTGGTCATG

1 CATGACCACTAGGAGCATCTTTGGCGAACGTCGGTAGAACGGGGTGGGGTGCTGTCCCTAGGGGAATAAATCTTTGGGCACCTAGTGGTCATG

1 CATGACCACTAGGAGCATCTTTGGCGAAGGGTGTAGGACTTCAAGTGGATTTCATAGCTAGGGGAATAAATCTTTGGGCACCTAGTGGTCATG

1 CATGACCACTAGGAGCATCTTTGGCGAACGGCAGGGGTTGCGCTGGTCTGTGAATCCCTAGGGGAATAAATCTTTGGGCACCTAGTGGTCATG

1 CATGACCACTAGGAGCATCTTTGGCGAGAACGGCGTCAGGCGCGCAGGTCCCAGGTTCTAGGGGAATAAATCTTTGGGCACCTAGTGGTCATG

1 CATGACCACTAGGAGCATCTTTGGCGAGCTCGGGAGAATCGGCGGCATTGGTGTCTCCTAGGGGAATAAATCTTTGGGCACCTAGTGGTCATG

1 CATGACCACTAGGAGCATCTTTGGCGAACGGCAGGTGTTGCGGTGGTCTATGAATCGCTAGGGGAATAAATCTTTGGGCACCTAGTGGTCATG

1 CATGACCACTAGGAGCATCTTTGGCGAGATCGGGAGAATTGGTGGCATTGGTGTTTTCTAGGGGAATAAATCTTTGGGCACCTAGTGGTCATG

1 CATGACCACTAGGAGCATCTTTGGCGACCGTAGGAAAACAAAGGTGGCCTTTCTAGCCTAGGGGAATAAATCTTTGGGCACCTAGTGGTCATG

1 CATGACCACTAGGAGCATCTTTGGCGATCCGGGGGTCAAATAAGACGGCGCGTTGGGCTAGGGGAATAAATCTTTGGGCACCTAGTGGTCATG

1 CATGACCACTAGGAGCATCTTTGGCGACCGGGGTAGCGGGGGAAATATGCGCAGGTCCTAGGGGAATAAATCTTTGGGCACCTAGTGGTCATG

1 CATGACCACTAGGAGCATCTTTGGCGAAGTCCCGCGGAAGGTGATGCGTCAGCGTCCCTAGGGGAATAAATCTTTGGGCACCTAGTGGTCATG

1 CATGACCACTAGGAGCATCTTTGGCGACACGAGTGGTCCCATCTCGTAAAAGTGGAGCTAGGGGAATAAATCTTTGGGCACCTAGTGGTCATG

1 CATGACCACTAGGAGCATCTTTGGCGAGATCGGGAGAATCGCTGGTATTGGTGTCTCCTAGGGGAATAAATCTTTGGGCACCTAGTGGTCATG

1 CATGACCACTAGGAGCATCTTTGGCGACGTCGTGTTCGGCATTCACTCATTTGAGAACTAGGGGAATAAATCTTTGGGCACCTAGTGGTCATG

1 CATGACCACTAGGAGCATCTTTGGCGAACGTCAGGTGTTGCGGAGGTCTGTGAATCCCTAGGGGAATAAATCTTTGGGCACCTAGTGGTCATG

1 CATGACCACTAGGAGCATCTTTGGCGACGAAGTGGTCAACGCGTTAAGTGGGGTAGACTAGGGGAATAAATCTTTGGGCACCTAGTGGTCATG

1 CATGACCACTAGGAGCATCTTTGGCGAAGGAGTGGTCGGCCATGTGGGGGGTCGGGGCTAGGGGAATAAATCTTTGGGCACCTAGTGGTCATG

1 CATGACCACTAGGAGCATCTTTGGCGACCTGGCTGGCAGGATAGTACCGAGTAGTCTCTAGGGGAATAAATCTTTGGGCACCTAGTGGTCATG

1 CATGACCACTAGGAGCATCTTTGGCGATCCGGGTGAAACCTGGCGTGGATTACGAGACTAGGGGAATAAATCTTTGGGCACCTAGTGGTCATG

1 CATGACCACTAGGAGCATCTTTGGCGATCCGGGGGTCAGATAAGAAGGCGCGTTGGGCTAGGGGAATAAATCTTTGGGCACCTAGTGGTCATG

1 CATGACCACTAGGAGCATCTTTGGCGAGCCGCGCTTGGGAGGTTGCTCCACCAGTTCCTAGGGGAATAAATCTTTGGGCACCTAGTGGTCATG

1 CATGACCACTAGGAGCATCTTTGGCGACCGTGGGCATGGGACAAGTATAAAGGACGCCTAGGGGAATAAATCTTTGGGCACCTAGTGGTCATG

1 CATGACCACTAGGAGCATCTTTGGCGACGGTGTGGGTACTTTAGTTAGGCGGTTAGTCTAGGGGAATAAATCTTTGGGCACCTAGTGGTCATG

1 CATGACCACTAGGAGCATCTTTGGCGAACGGCGGGTGTTGCGGTGGTCCGTGAATCCCTAGGGGAATAAATCTTTGGGCACCTAGTGGTCATG

1 CATGACCACTAGGAGCATCTTTGGCGACTAAGTGGCCCACGCGTTAAGTGGGGTAGACTAGGGGAATAAATCTTTGGGCACCTAGTGGTCATG

1 CATGACCACTAGGAGCATCTTTGGCGAGAAGACTCTGGAGACGGGGACCAGTTGCTGCTAGGGGAATAAATCTTTGGGCACCTAGTGGTCATG

1 CATGACCACTAGGAGCATCTTTGGCGAGGGAGGGCGCCGGCAGCGGTATGAATGCGACTAGGGGAATAAATCTTTGGGCACCTAGTGGTCATG

1 CATGACCACTAGGAGCATCTTTGGCGAAGGGTGTAGGACTTCAAGTGGCTCTCATAGCTAGGGGAATAAATCTTTGGGCACCTAGTGGTCATG

1 CATGACCACTAGGAGCATCTTTGGCGAGATCGGGAGAATCGGTGGTATTGTTGTCTCCTAGGGGAATAAATCTTTGGGCACCTAGTGGTCATG

1 CATGACCACTAGGAGCATCTTTGGCGATCAGGGGGCCTTTGATGGCGATTCAGTGGGCTAGGGGAATAAATCTTTGGGCACCTAGTGGTCATG

1 CATGACCACTAGGAGCATCTTTGGCGAGCGTCTTAGGGAGTATCCTTCCTTAGATACCTAGGGGAATAAATCTTTGGGCACCTAGTGGTCATG

1 CATGACCACTAGGAGCATCTTTGGCGAAGCGTGGTCGTTAGATTGGTTAGGTTGATCCTAGGGGAATAAATCTTTGGGCACCTAGTGGTCATG

1 CATGACCACTAGGAGCATCTTTGGCGAACCTTGTTACTCGGGATCCAAATCAGTTCTCTAGGGGAATAAATCTTTGGGCACCTAGTGGTCATG

1 CATGACCACTAGGAGCATCTTTGGCGAAGGCGCCCGAGTGGTCTCGTCGTGCCAAGACTAGGGGAATAAATCTTTGGGCACCTAGTGGTCATG

1 CATGACCACTAGGAGCATCTTTGGCGACTCCAGTTCACCTGTCGGGCAGCTTAAGGACTAGGGGAATAAATCTTTGGGCACCTAGTGGTCATG

1 CATGACCACTAGGAGCATCTTTGGCGAGCCTTGCTTGGGAGATTGCTCCACTAGTTCCTAGGGGAATAAATCTTTGGGCACCTAGTGGTCATG

1 CATGACCACTAGGAGCATCTTTGGCGAGACCGGGAGAATCGGTGGCATTGATGTCTCCTAGGGGAATAAATCTTTGGGCACCTAGTGGTCATG

1 CATGACCACTAGGAGCATCTTTGGCGACGGCGTGGGGCCATTTGTGCGGCGGATCTCCTAGGGGAATAAATCTTTGGGCACCTAGTGGTCATG

1 CATGACCACTAGGAGCATCTTTGGCGAGGTGCGACAGAGTGCGTGTGGTGGTCGAGACTAGGGGAATAAATCTTTGGGCACCTAGTGGTCATG

1 CATGACCACTAGGAGCATCTTTGGCGAACGACAGGTGTTGCGGTGGTTTGTGAATCCCTAGGGGAATAAATCTTTGGGCACCTAGTGGTCATG

1 CATGACCACTAGGAGCATCTTTGGCGACAGGGGATGCCGGTGCGCTGGACGACCGAGCTAGGGGAATAAATCTTTGGGCACCTAGTGGTCATG

1 CATGACCACTAGGAGCATCTTTGGCGATATCGGCAGAATCGGTGGCATTGGTGTCTCCTAGGGGAATAAATCTTTGGGCACCTAGTGGTCATG

1 CATGACCACTAGGAGCATCTTTGGCGAGAAGACTCTGGATTCGGGGGCTAGTTGCTGCTAGGGGAATAAATCTTTGGGCACCTAGTGGTCATG

1 CATGACCACTAGGAGCATCTTTGGCGAGATCGGGAGAGTCGGTGGCATAGGTGTCTCCTAGGGGAATAAATCTTTGGGCACCTAGTGGTCATG

1 CATGACCACTAGGAGCATCTTTGGCGAGATCGGGAGAATCGGTGGTATTGGAGTCTCCTAGGGGAATAAATCTTTGGGCACCTAGTGGTCATG

1 CATGACCACTAGGAGCATCTTTGGCGAACGGCTGGTGTAGCGGTGGTCTGTGAATCCCTAGGGGAATAAATCTTTGGGCACCTAGTGGTCATG

1 CATGACCACTAGGAGCATCTTTGGCGAGAAGACTCTGGAATCGGGGACCAGTTGCTCCTAGGGGAATAAATCTTTGGGCACCTAGTGGTCATG

1 CATGACCACTAGGAGCATCTTTGGCGATGGGTGCTGACGGCCGCCGCTGCGACTACACTAGGGGAATAAATCTTTGGGCACCTAGTGGTCATG

1 CATGACCACTAGGAGCATCTTTGGCGAATGGCCGGCACGCCCTTCTAGTCCTCGGTACTAGGGGAATAAATCTTTGGGCACCTAGTGGTCATG

1 CATGACCACTAGGAGCATCTTTGGCGAGCCTTGCTTGGGAGGTTGCTCCACCACTTCCTAGGGGAATAAATCTTTGGGCACCTAGTGGTCATG

1 CATGACCACTAGGAGCATCTTTGGCGAGATCGGGAGAATCGGTGGCATTGGACACTCCTAGGGGAATAAATCTTTGGGCACCTAGTGGTCATG

1 CATGACCACTAGGAGCATCTTTGGCGAACGGCGGGTGTTTCGGTGGTCTGTGAATCCCTAGGGGAATAAATCTTTGGGCACCTAGTGGTCATG

1 CATGACCACTAGGAGCATCTTTGGCGACGGGCAAGTCAGTGAAGAAGATTAACTCGTCTAGGGGAATAAATCTTTGGGCACCTAGTGGTCATG

1 CATGACCACTAGGAGCATCTTTGGCGAGATCTAGAGAATCGGTGGCATTGGTGTCTACTAGGGGAATAAATCTTTGGGCACCTAGTGGTCATG

1 CATGACCACTAGGAGCATCTTTGGCGAAAGGCAGGTGTTGCGGTGGTCTGTGACTCCCTAGGGGAATAAATCTTTGGGCACCTAGTGGTCATG

1 CATGACCACTAGGAGCATCTTTGGCGAGAAGACTTTGGATTCGGGGACAAGTTGCTGCTAGGGGAATAAATCTTTGGGCACCTAGTGGTCATG

1 CATGACCACTAGGAGCATCTTTGGCGAGCACTACGTAGCGGTAGGCACTGGTGTCTCCTAGGGGAATAAATCTTTGGGCACCTAGTGGTCATG

1 CATGACCACTAGGAGCATCTTTGGCGACCTTAGGTCCTCTGACACCGTGAGTGTCCCCTAGGGGAATAAATCTTTGGGCACCTAGTGGTCATG

1 CATGACCACTAGGAGCATCTTTGGCGAGAAGACTCTGGATTCAGGGACTAGTTGCTGCTAGGGGAATAAATCTTTGGGCACCTAGTGGTCATG

1 CATGACCACTAGGAGCATCTTTGGCGAGATCGGGAGAAACGGTGGCATAGGTGTCACCTAGGGGAATAAATCTTTGGGCACCTAGTGGTCATG

1 CATGACCACTAGGAGCATCTTTGGCGACAGCGGATGACTATGAGCTGGACGACACACCTAGGGGAATAAATCTTTGGGCACCTAGTGGTCATG

1 CATGACCACTAGGAGCATCTTTGGCGAAGGACAGAGATCGACATAGTGGCCGAGAGACTAGGGGAATAAATCTTTGGGCACCTAGTGGTCATG

1 CATGACCACTAGGAGCATCTTTGGCGACCCGTGTAGAGCAATAAGCTTGTCCTGTGACTAGGGGAATAAATCTTTGGGCACCTAGTGGTCATG

1 CATGACCACTAGGAGCATCTTTGGCGACAAGTGGGCGGAGCTTAACGCGGCATAGTGCTAGGGGAATAAATCTTTGGGCACCTAGTGGTCATG

1 CATGACCACTAGGAGCATCTTTGGCGAACAGGAACTGGGGGGCGAAATGTTTATAGACTAGGGGAATAAATCTTTGGGCACCTAGTGGTCATG

1 CATGACCACTAGGAGCATCTTTGGCGACGACAAAGGTTGCGCGTTGTGGAGGAGACACTAGGGGAATAAATCTTTGGGCACCTAGTGGTCATG

1 CATGACCACTAGGAGCATCTTTGGCGAACGCGCTATGTATGTCGATGCGATTGGAACCTAGGGGAATAAATCTTTGGGCACCTAGTGGTCATG

1 CATGACCACTAGGAGCATCTTTGGCGAGATAGGGAGAATCGGTGGCATTGGTTTCTACTAGGGGAATAAATCTTTGGGCACCTAGTGGTCATG

1 CATGACCACTAGGAGCATCTTTGGCGAGATCGGGAGAATCTGTGGAATTGGTGTATCCTAGGGGAATAAATCTTTGGGCACCTAGTGGTCATG

1 CATGACCACTAGGAGCATCTTTGGCGATCTAGTGGACACGCGTTATCATTGGGAACGCTAGGGGAATAAATCTTTGGGCACCTAGTGGTCATG

1 CATGACCACTAGGAGCATCTTTGGCGAGATCGGAAGAATCGGTGGCATAGGTGTCTCCTAGGGGAATAAATCTTTGGGCACCTAGTGGTCATG

1 CATGACCACTAGGAGCATCTTTGGCGACGGCGTGGGGCCATCTGTGCGGCGGGTCCCCTAGGGGAATAAATCTTTGGGCACCTAGTGGTCATG

1 CATGACCACTAGGAGCATCTTTGGCGAGAACGTGAGAATCGTTGGCATTGGTGTCTCCTAGGGGAATAAATCTTTGGGCACCTAGTGGTCATG

1 CATGACCACTAGGAGCATCTTTGGCGATCCGGGGGCCACTGAGGCGACGCTTATGGGCTAGGGGAATAAATCTTTGGGCACCTAGTGGTCATG

1 CATGACCACTAGGAGCATCTTTGGCGACCCAGGATGGCAATGTTGCAACGGGGTGGACTAGGGGAATAAATCTTTGGGCACCTAGTGGTCATG

1 CATGACCACTAGGAGCATCTTTGGCGAACGTGCAAGATATGTCGCCTGGAGCGTAGGCTAGGGGAATAAATCTTTGGGCACCTAGTGGTCATG

1 CATGACCACTAGGAGCATCTTTGGCGACGGCGTGGGGCTAAAGATGCGGCGGCACTCCTAGGGGAATAAATCTTTGGGCACCTAGTGGTCATG

1 CATGACCACTAGGAGCATCTTTGGCGACGGTGTGGGGACCTTAGGTTCGGCGGGTACCTAGGGGAATAAATCTTTGGGCACCTAGTGGTCATG

1 CATGACCACTAGGAGCATCTTTGGCGAGATCGGGCTAATCTGAGGCATTGGTGTCTCCTAGGGGAATAAATCTTTGGGCACCTAGTGGTCATG

1 CATGACCACTAGGAGCATCTTTGGCGACAAGGGTATGTAATATACCGCGACATAGTGCTAGGGGAATAAATCTTTGGGCACCTAGTGGTCATG

1 CATGACCACTAGGAGCATCTTTGGCGACCTTGGCGCGGGGATCCTGGTTGCGGCGACCTAGGGGAATAAATCTTTGGGCACCTAGTGGTCATG

1 CATGACCACTAGGAGCATCTTTGGCGAGATCGGGAGAACCGGTGGCATTGGTGTCCCCTAGGGGAATAAATCTTTGGGCACCTAGTGGTCATG

1 CATGACCACTAGGAGCATCTTTGGCGACGTTTATCTGGCATGTGTTGGCGCATCTATCTAGGGGAATAAATCTTTGGGCACCTAGTGGTCATG

1 CATGACCACTAGGAGCATCTTTGGCGAAAGTCGGTAGAACAGGGTGGCGTGCTGTCCCTAGGGGAATAAATCTTTGGGCACCTAGTGGTCATG

1 CATGACCACTAGGAGCATCTTTGGCGAGATCGGAAGAATCGGTGGCACTGGTGTCTCCTAGGGGAATAAATCTTTGGGCACCTAGTGGTCATG

1 CATGACCACTAGGAGCATCTTTGGCGACGCGTTTGGGTGTGTGCCCATTGTACTCCGCTAGGGGAATAAATCTTTGGGCACCTAGTGGTCATG

1 CATGACCACTAGGAGCATCTTTGGCGACAGCCGGAGGGACCAGAAGGGAGTCACTGTCTAGGGGAATAAATCTTTGGGCACCTAGTGGTCATG

1 CATGACCACTAGGAGCATCTTTGGCGAACGCGGAGGGTGTCACCATACGATTGGAACCTAGGGGAATAAATCTTTGGGCACCTAGTGGTCATG

1 CATGACCACTAGGAGCATCTTTGGCGAGAGGAAAGCTCCTCCAAATATGGCCCCGGCCTAGGGGAATAAATCTTTGGGCACCTAGTGGTCATG

1 CATGACCACTAGGAGCATCTTTGGCGAACGTCAGGTGTTGCGGTGGTCTGTGAATCACTAGGGGAATAAATCTTTGGGCACCTAGTGGTCATG

1 CATGACCACTAGGAGCATCTTTGGCGATGTGATCGACGCGCTATAGGTTGCGGTAAGCTAGGGGAATAAATCTTTGGGCACCTAGTGGTCATG

1 CATGACCACTAGGAGCATCTTTGGCGACTGTGGTCTCGTCCTGATTGTGCGGGCGTCCTAGGGGAATAAATCTTTGGGCACCTAGTGGTCATG

1 CATGACCACTAGGAGCATCTTTGGCGAGATCGGTAGAATCGGTGGCAATGGGGTCTCCTAGGGGAATAAATCTTTGGGCACCTAGTGGTCATG

1 CATGACCACTAGGAGCATCTTTGGCGATGACGTCGTCGTAAATAGAGGCCTTGCGACCTAGGGGAATAAATCTTTGGGCACCTAGTGGTCATG

1 CATGACCACTAGGAGCATCTTTGGCGAGAACGGGAGAATCGGTGCCATTGGTGTCTCCTAGGGGAATAAATCTTTGGGCACCTAGTGGTCATG

1 CATGACCACTAGGAGCATCTTTGGCGACGAAGGGCATGGGTGGTCATTGCGAGAAGACTAGGGGAATAAATCTTTGGGCACCTAGTGGTCATG

1 CATGACCACTAGGAGCATCTTTGGCGAGAAGACTCTGGATTCGGGTACCAGATGCTGCTAGGGGAATAAATCTTTGGGCACCTAGTGGTCATG

1 CATGACCACTAGGAGCATCTTTGGCGAGATCTGGAGAAAAGGTGGCAATGGTGTCTCCTAGGGGAATAAATCTTTGGGCACCTAGTGGTCATG

1 CATGACCACTAGGAGCATCTTTGGCGAACGGCAGGTTTTGCGGTGGTCTGTGAATTCCTAGGGGAATAAATCTTTGGGCACCTAGTGGTCATG

1 CATGACCACTAGGAGCATCTTTGGCGAATGGCCGGCACGGCCTACTAGTCCTTGGTACTAGGGGAATAAATCTTTGGGCACCTAGTGGTCATG

1 CATGACCACTAGGAGCATCTTTGGCGACAACGTGGTCTTCCAGTAAGTGTAGTGGAGCTAGGGGAATAAATCTTTGGGCACCTAGTGGTCATG

1 CATGACCACTAGGAGCATCTTTGGCGAACGGCAGGTGTTGCGGTGGTCTGTGACTCTCTAGGGGAATAAATCTTTGGGCACCTAGTGGTCATG

1 CATGACCACTAGGAGCATCTTTGGCGACGATTGTTGGGAGGGATGTATTTCGGGCGACTAGGGGAATAAATCTTTGGGCACCTAGTGGTCATG

1 CATGACCACTAGGAGCATCTTTGGCGACGTCGCCTTCCCGTATCGCCGGGAAGTTGGCTAGGGGAATAAATCTTTGGGCACCTAGTGGTCATG

1 CATGACCACTAGGAGCATCTTTGGCGATTAGAGTAGTATGATCGATACGGCCGGTGACTAGGGGAATAAATCTTTGGGCACCTAGTGGTCATG

1 CATGACCACTAGGAGCATCTTTGGCGAAGTGGGCTGGTAGTTTGGTACTTGCGTAGACTAGGGGAATAAATCTTTGGGCACCTAGTGGTCATG

1 CATGACCACTAGGAGCATCTTTGGCGAAATTAAATTCCAAAGAGACATCCTGTCAGTCTAGGGGAATAAATCTTTGGGCACCTAGTGGTCATG

1 CATGACCACTAGGAGCATCTTTGGCGACTAGCGTAGGACGGTACCGGGTGAAGGTGACTAGGGGAATAAATCTTTGGGCACCTAGTGGTCATG

1 CATGACCACTAGGAGCATCTTTGGCGAGTATTGGCTGGTAGGTTGTGTATTGGGGAGCTAGGGGAATAAATCTTTGGGCACCTAGTGGTCATG

1 CATGACCACTAGGAGCATCTTTGGCGACTGTAGGAAAACAAAAGTGGCCTTGCTAGCCTAGGGGAATAAATCTTTGGGCACCTAGTGGTCATG

1 CATGACCACTAGGAGCATCTTTGGCGATCGGTGGTCAAGACTATAGGCTCTCGTCGCCTAGGGGAATAAATCTTTGGGCACCTAGTGGTCATG

1 CATGACCACTAGGAGCATCTTTGGCGAACGGGAGGTGTTGCGGTGGTCTATGAATCCCTAGGGGAATAAATCTTTGGGCACCTAGTGGTCATG

1 CATGACCACTAGGAGCATCTTTGGCGAGATCTGGAGACTCGGTGGCAGTGGTGTCTCCTAGGGGAATAAATCTTTGGGCACCTAGTGGTCATG

1 CATGACCACTAGGAGCATCTTTGGCGACCAGATATGGTAGGAAATGTGCTGAGGAGGCTAGGGGAATAAATCTTTGGGCACCTAGTGGTCATG

1 CATGACCACTAGGAGCATCTTTGGCGAACGGCAGGTTTTGCGGAGGTCTGTGAATCCCTAGGGGAATAAATCTTTGGGCACCTAGTGGTCATG

1 CATGACCACTAGGAGCATCTTTGGCGATGGAGTCTCGACGCGGAATGTTACCTCGGACTAGGGGAATAAATCTTTGGGCACCTAGTGGTCATG

1 CATGACCACTAGGAGCATCTTTGGCGACGATGGTTGGAAGTGATGTATTTCGGGCGACTAGGGGAATAAATCTTTGGGCACCTAGTGGTCATG

1 CATGACCACTAGGAGCATCTTTGGCGAACGGTAACGGGGGGTAGATGTCGCTCATAGCTAGGGGAATAAATCTTTGGGCACCTAGTGGTCATG

1 CATGACCACTAGGAGCATCTTTGGCGAGGCGTTTTCAAACACTCCCCAAGAGGCGGTCTAGGGGAATAAATCTTTGGGCACCTAGTGGTCATG

1 CATGACCACTAGGAGCATCTTTGGCGAGATCGGGAGAATCGGAGGCATTGGTGTTTCCTAGGGGAATAAATCTTTGGGCACCTAGTGGTCATG

1 CATGACCACTAGGAGCATCTTTGGCGAGAAGACTATGGATTCGGGGACCAGATGCTCCTAGGGGAATAAATCTTTGGGCACCTAGTGGTCATG

1 CATGACCACTAGGAGCATCTTTGGCGATATCGGGAGAATCGGTGGCATTGTTGTCTCCTAGGGGAATAAATCTTTGGGCACCTAGTGGTCATG

1 CATGACCACTAGGAGCATCTTTGGCGACGTGGGCGTTTCGGCCGTTCCGGCGTGTGCCTAGGGGAATAAATCTTTGGGCACCTAGTGGTCATG

1 CATGACCACTAGGAGCATCTTTGGCGAGAATGGTCGTTTTGCGCTATACGCCCTTGGCTAGGGGAATAAATCTTTGGGCACCTAGTGGTCATG

1 CATGACCACTAGGAGCATCTTTGGCGAAGTGCGGAGGGCATGTGGGTGGTAGCGACGCTAGGGGAATAAATCTTTGGGCACCTAGTGGTCATG

1 CATGACCACTAGGAGCATCTTTGGCGATCCGACGTCAAGGTTGACGAACTAGTGGAGCTAGGGGAATAAATCTTTGGGCACCTAGTGGTCATG

1 CATGACCACTAGGAGCATCTTTGGCGAAAGCGAGTGCAACAGTGGAAGGATCTGGCGCTAGGGGAATAAATCTTTGGGCACCTAGTGGTCATG

1 CATGACCACTAGGAGCATCTTTGGCGAGGTCGGGAGAATCGGTGGCATTGGTGTTTCCTAGGGGAATAAATCTTTGGGCACCTAGTGGTCATG

1 CATGACCACTAGGAGCATCTTTGGCGAGATCGGGAGAAACGGTGGCATTGGTTTCTCCTAGGGGAATAAATCTTTGGGCACCTAGTGGTCATG

1 CATGACCACTAGGAGCATCTTTGGCGAGAGCTTGAAAATGGTGCGTTGTTGTCGCATCTAGGGGAATAAATCTTTGGGCACCTAGTGGTCATG

1 CATGACCACTAGGAGCATCTTTGGCGACGAAGGTCTTTTAGTGGCCCGAGGGCCGACCTAGGGGAATAAATCTTTGGGCACCTAGTGGTCATG

1 CATGACCACTAGGAGCATCTTTGGCGAACACAGATGGGGGGGGTCATAGCTCGTAGCCTAGGGGAATAAATCTTTGGGCACCTAGTGGTCATG

1 CATGACCACTAGGAGCATCTTTGGCGAAGCGTGTTGCGAGGTCCGCGTGTGTGGACCCTAGGGGAATAAATCTTTGGGCACCTAGTGGTCATG

1 CATGACCACTAGGAGCATCTTTGGCGAGACCGGGAGAATCGGTGGCATTGGTGTCTACTAGGGGAATAAATCTTTGGGCACCTAGTGGTCATG

1 CATGACCACTAGGAGCATCTTTGGCGAGGGGACGTAGGGCACGCAGCCGGGGGCGAGCTAGGGGAATAAATCTTTGGGCACCTAGTGGTCATG

1 CATGACCACTAGGAGCATCTTTGGCGACTGTGGGGCTGGTAGGAAAGTACTTATAGCCTAGGGGAATAAATCTTTGGGCACCTAGTGGTCATG

1 CATGACCACTAGGAGCATCTTTGGCGAGAAGACCCTGGATTTGGGGACCAGTTGCTGCTAGGGGAATAAATCTTTGGGCACCTAGTGGTCATG

1 CATGACCACTAGGAGCATCTTTGGCGATTACAGGGGGGTTGGCTCATGAGGTCTCGTCTAGGGGAATAAATCTTTGGGCACCTAGTGGTCATG

1 CATGACCACTAGGAGCATCTTTGGCGACGGGGGTTCCCCTCATCCAGGATGTCCCTCCTAGGGGAATAAATCTTTGGGCACCTAGTGGTCATG

1 CATGACCACTAGGAGCATCTTTGGCGAGCCTTGCTTGGGAGTTTGCTCCACCAGTTCCTAGGGGAATAAATCTTTGGGCACCTAGTGGTCATG

1 CATGACCACTAGGAGCATCTTTGGCGAGAAGACTCTGTATTCGGGGACCAGTAGCTGCTAGGGGAATAAATCTTTGGGCACCTAGTGGTCATG

1 CATGACCACTAGGAGCATCTTTGGCGAGATCGGTAGAATAGGTGGCATTCGTGTCTCCTAGGGGAATAAATCTTTGGGCACCTAGTGGTCATG

1 CATGACCACTAGGAGCATCTTTGGCGAGATCGGGATAATCGGTGGCAATGGTGTCTCCTAGGGGAATAAATCTTTGGGCACCTAGTGGTCATG

1 CATGACCACTAGGAGCATCTTTGGCGATCGCAGTGGACATGGAGCTGGGGAGATCGACTAGGGGAATAAATCTTTGGGCACCTAGTGGTCATG

1 CATGACCACTAGGAGCATCTTTGGCGATCCGTGGGTCACAAGTGACGGCGCATAGGGCTAGGGGAATAAATCTTTGGGCACCTAGTGGTCATG

1 CATGACCACTAGGAGCATCTTTGGCGACAGGGGATGCCGATGCGATGGACGACCGAGCTAGGGGAATAAATCTTTGGGCACCTAGTGGTCATG

1 CATGACCACTAGGAGCATCTTTGGCGAAACAGATAGTTGAGAGTGGGTTTCTGACGCCTAGGGGAATAAATCTTTGGGCACCTAGTGGTCATG

1 CATGACCACTAGGAGCATCTTTGGCGACAGTGGGCGGACCGGGGTTCAAGGGGCGGACTAGGGGAATAAATCTTTGGGCACCTAGTGGTCATG

1 CATGACCACTAGGAGCATCTTTGGCGAGATCGGGAGAGTCGGTTGCATTGGTGTCTCCTAGGGGAATAAATCTTTGGGCACCTAGTGGTCATG

1 CATGACCACTAGGAGCATCTTTGGCGAGGAGTGCCCGGCGGTGGCCTGGTAGGTCGTCTAGGGGAATAAATCTTTGGGCACCTAGTGGTCATG

1 CATGACCACTAGGAGCATCTTTGGCGAGTACAGAAAATTAACTGTATTAGTCCCAGTCTAGGGGAATAAATCTTTGGGCACCTAGTGGTCATG

1 CATGACCACTAGGAGCATCTTTGGCGAGATCGGGAGAATCGGAGGCATTGGAGTCTCCTAGGGGAATAAATCTTTGGGCACCTAGTGGTCATG

1 CATGACCACTAGGAGCATCTTTGGCGATGTCGGGAGAATCGGTGGCATTGGTGTCTCCTAGGGGAATAAATCTTTGGGCACCTAGTGGTCATG

1 CATGACCACTAGGAGCATCTTTGGCGACGTCGTAAAGTGCGTAGAGTGGTACGTCGACTAGGGGAATAAATCTTTGGGCACCTAGTGGTCATG

1 CATGACCACTAGGAGCATCTTTGGCGACGGTACGGTTATCCCAAATGCAGCGCACCGCTAGGGGAATAAATCTTTGGGCACCTAGTGGTCATG

1 CATGACCACTAGGAGCATCTTTGGCGACGTTATGAGTTGGGAAGCCTGGCGGCCAGGCTAGGGGAATAAATCTTTGGGCACCTAGTGGTCATG

1 CATGACCACTAGGAGCATCTTTGGCGAGGGCCCATGGGCGGGGTAGTCGTCACTCTGCTAGGGGAATAAATCTTTGGGCACCTAGTGGTCATG

1 CATGACCACTAGGAGCATCTTTGGCGAGATCGGGAGAATCGGTGGCATCGGCGTCTCCTAGGGGAATAAATCTTTGGGCACCTAGTGGTCATG

1 CATGACCACTAGGAGCATCTTTGGCGAGCCGGGTTTGGAAGTGCGGGAAGTAGTCATCTAGGGGAATAAATCTTTGGGCACCTAGTGGTCATG

1 CATGACCACTAGGAGCATCTTTGGCGAGAGGGCGCTAGTGGTCGTGGTTGCCCGTCCCTAGGGGAATAAATCTTTGGGCACCTAGTGGTCATG

1 CATGACCACTAGGAGCATCTTTGGCGAGATCGGGAGAATCGGTGGCAGTGGAGTCCCCTAGGGGAATAAATCTTTGGGCACCTAGTGGTCATG

1 CATGACCACTAGGAGCATCTTTGGCGAACGGTAACGTAGTAACGTCGAAGTAGGCTCCTAGGGGAATAAATCTTTGGGCACCTAGTGGTCATG

1 CATGACCACTAGGAGCATCTTTGGCGACCCTGGCGCGGGGATCCTGGTGGCGGCGACCTAGGGGAATAAATCTTTGGGCACCTAGTGGTCATG

1 CATGACCACTAGGAGCATCTTTGGCGACGTGTTGAGCGGTGAGCGACAACAAGTGGACTAGGGGAATAAATCTTTGGGCACCTAGTGGTCATG

1 CATGACCACTAGGAGCATCTTTGGCGACCCGCGAAGGGCATTTGGCCAGACCTGTGACTAGGGGAATAAATCTTTGGGCACCTAGTGGTCATG

1 CATGACCACTAGGAGCATCTTTGGCGAAGCTTGGTTAGGCGACGGGCTTGGGCCACTCTAGGGGAATAAATCTTTGGGCACCTAGTGGTCATG

1 CATGACCACTAGGAGCATCTTTGGCGAACTGCAGGTGTTGCGGTGGTCTGTGAAACCCTAGGGGAATAAATCTTTGGGCACCTAGTGGTCATG

1 CATGACCACTAGGAGCATCTTTGGCGACGCGAGGTGATAGTAGCATCTCGACGTGGACTAGGGGAATAAATCTTTGGGCACCTAGTGGTCATG

1 CATGACCACTAGGAGCATCTTTGGCGACCGGGGTCATGCTAAGCAAACGGGGGTAGACTAGGGGAATAAATCTTTGGGCACCTAGTGGTCATG

1 CATGACCACTAGGAGCATCTTTGGCGACCCGGGGGTCAGATAAGACGGCGCGTTGGGCTAGGGGAATAAATCTTTGGGCACCTAGTGGTCATG

1 CATGACCACTAGGAGCATCTTTGGCGAAGAGAGGAAAGGTCGGGAACGGTGCGTTCACTAGGGGAATAAATCTTTGGGCACCTAGTGGTCATG

1 CATGACCACTAGGAGCATCTTTGGCGATCGCCTACGTTGTGGTCAAGTACTAGCAGCCTAGGGGAATAAATCTTTGGGCACCTAGTGGTCATG

1 CATGACCACTAGGAGCATCTTTGGCGAGAAGACTCTGTATTCAGGGACCAGTTGCTGCTAGGGGAATAAATCTTTGGGCACCTAGTGGTCATG

1 CATGACCACTAGGAGCATCTTTGGCGAGTCGTAATCCTTCGGTTGGCTCGACAAGCCCTAGGGGAATAAATCTTTGGGCACCTAGTGGTCATG

1 CATGACCACTAGGAGCATCTTTGGCGAGATCGGAAAAATCGGTGGCATTGGTGTCTCCTAGGGGAATAAATCTTTGGGCACCTAGTGGTCATG

1 CATGACCACTAGGAGCATCTTTGGCGAGGCTTAGTGGGAGTAGTGAGGAGCTCGGGACTAGGGGAATAAATCTTTGGGCACCTAGTGGTCATG

1 CATGACCACTAGGAGCATCTTTGGCGAACGGTCTTGGGCCGGGGGGATGCTCGTAGACTAGGGGAATAAATCTTTGGGCACCTAGTGGTCATG

1 CATGACCACTAGGAGCATCTTTGGCGACAGGAGAACACGGCACGTCGAGGACGTCGACTAGGGGAATAAATCTTTGGGCACCTAGTGGTCATG

1 CATGACCACTAGGAGCATCTTTGGCGAGGAGTGGCTGAGGGCGGCGGGTAGGTCGCGCTAGGGGAATAAATCTTTGGGCACCTAGTGGTCATG

1 CATGACCACTAGGAGCATCTTTGGCGAACGGCAGGGGTTGCGGTGGTCTGCGAATCCCTAGGGGAATAAATCTTTGGGCACCTAGTGGTCATG

1 CATGACCACTAGGAGCATCTTTGGCGAACTAGGGGGGTAAAAATGCTTTCGAAGCCTCTAGGGGAATAAATCTTTGGGCACCTAGTGGTCATG

1 CATGACCACTAGGAGCATCTTTGGCGAGAAGACTCTGGATTCGGTGAACAGTTGCTGCTAGGGGAATAAATCTTTGGGCACCTAGTGGTCATG

1 CATGACCACTAGGAGCATCTTTGGCGAGAACGGGAGAATCGGTGGCATTGGTGTTTCCTAGGGGAATAAATCTTTGGGCACCTAGTGGTCATG

1 CATGACCACTAGGAGCATCTTTGGCGACGGGAAATCAAGGAGGGCTGGGGAGTGTTGCTAGGGGAATAAATCTTTGGGCACCTAGTGGTCATG

1 CATGACCACTAGGAGCATCTTTGGCGATACGCATAGCGAGACGAAGGAAGTCTGGGCCTAGGGGAATAAATCTTTGGGCACCTAGTGGTCATG

1 CATGACCACTAGGAGCATCTTTGGCGAGAACTCTCGGGAGTCTGGGACAGGTTTCTGCTAGGGGAATAAATCTTTGGGCACCTAGTGGTCATG

1 CATGACCACTAGGAGCATCTTTGGCGACTCAGTGGCCAGCGCGTTAAATGGGGTAGACTAGGGGAATAAATCTTTGGGCACCTAGTGGTCATG

1 CATGACCACTAGGAGCATCTTTGGCGAGATCTGGAGAATCGGTGGCATTGGTGTCACCTAGGGGAATAAATCTTTGGGCACCTAGTGGTCATG

1 CATGACCACTAGGAGCATCTTTGGCGACTTGTGGCAGGGTGATAGGGAATGTCGACGCTAGGGGAATAAATCTTTGGGCACCTAGTGGTCATG

1 CATGACCACTAGGAGCATCTTTGGCGAGATCGCGAGAATCGGTGGCATTCGTGTCTCCTAGGGGAATAAATCTTTGGGCACCTAGTGGTCATG

1 CATGACCACTAGGAGCATCTTTGGCGAACGGCAGGTGTTGAGGTGGTTTGTGAATCCCTAGGGGAATAAATCTTTGGGCACCTAGTGGTCATG

1 CATGACCACTAGGAGCATCTTTGGCGATCACCGGACTCGGCCAGAGTGAAAGTGGAGCTAGGGGAATAAATCTTTGGGCACCTAGTGGTCATG

1 CATGACCACTAGGAGCATCTTTGGCGATGTATTATCATCTGGCCAAACGTAAACAATCTAGGGGAATAAATCTTTGGGCACCTAGTGGTCATG

1 CATGACCACTAGGAGCATCTTTGGCGACGAGCAGTTCCCGGAACCTCGCGAAGTGGACTAGGGGAATAAATCTTTGGGCACCTAGTGGTCATG

1 CATGACCACTAGGAGCATCTTTGGCGATCCGGGGGCCTGAGTAGGCGACGCCCCGGGCTAGGGGAATAAATCTTTGGGCACCTAGTGGTCATG

1 CATGACCACTAGGAGCATCTTTGGCGAAAGGATGTGGGCGGTGGGAACTGGATAGGGCTAGGGGAATAAATCTTTGGGCACCTAGTGGTCATG

1 CATGACCACTAGGAGCATCTTTGGCGAGATCGGAAGAATCGGTGGCATTGGTGTCTACTAGGGGAATAAATCTTTGGGCACCTAGTGGTCATG

1 CATGACCACTAGGAGCATCTTTGGCGATTCGGTTACGGTCCCCCCAAGCAACGCATACTAGGGGAATAAATCTTTGGGCACCTAGTGGTCATG

1 CATGACCACTAGGAGCATCTTTGGCGATGTGGGCTGGTAGGAGTGGTACTTGTTGAGCTAGGGGAATAAATCTTTGGGCACCTAGTGGTCATG

1 CATGACCACTAGGAGCATCTTTGGCGAACGGCAGGTGTTGCGGTGGTCTGTGGACCCCTAGGGGAATAAATCTTTGGGCACCTAGTGGTCATG

1 CATGACCACTAGGAGCATCTTTGGCGAACGGCAGGTGTTGCGGTGGTCTGTGAATAACTAGGGGAATAAATCTTTGGGCACCTAGTGGTCATG

1 CATGACCACTAGGAGCATCTTTGGCGAACAGCAGGTGTTGCGGTGGTCTGTGAATCACTAGGGGAATAAATCTTTGGGCACCTAGTGGTCATG

1 CATGACCACTAGGAGCATCTTTGGCGAGATCGGTAGAATCGGTGGCCTTGGAGTCTCCTAGGGGAATAAATCTTTGGGCACCTAGTGGTCATG

1 CATGACCACTAGGAGCATCTTTGGCGAGTATTGGCTGGTAGGTTGCGGATTGGGGAGCTAGGGGAATAAATCTTTGGGCACCTAGTGGTCATG

1 CATGACCACTAGGAGCATCTTTGGCGAGTGGGGGGCGACGGCCGGTGGTGGGTGAGACTAGGGGAATAAATCTTTGGGCACCTAGTGGTCATG

1 CATGACCACTAGGAGCATCTTTGGCGAATGGCCGGCACGGCCTTCTAGTCATCGGTACTAGGGGAATAAATCTTTGGGCACCTAGTGGTCATG

1 CATGACCACTAGGAGCATCTTTGGCGACCCAGACATGTTGGCGCAACACTGGGTGGACTAGGGGAATAAATCTTTGGGCACCTAGTGGTCATG

1 CATGACCACTAGGAGCATCTTTGGCGAGATCGGGAGAATCGGCGGCATTGGTATCTCCTAGGGGAATAAATCTTTGGGCACCTAGTGGTCATG

1 CATGACCACTAGGAGCATCTTTGGCGAGATCAGGAGAATCGGTGGCATTAGTGTCTCCTAGGGGAATAAATCTTTGGGCACCTAGTGGTCATG

1 CATGACCACTAGGAGCATCTTTGGCGAGATCGGGAGAAACCCTGGCATTGGTGTCTCCTAGGGGAATAAATCTTTGGGCACCTAGTGGTCATG

1 CATGACCACTAGGAGCATCTTTGGCGAGGGGTCATGGGAATGTAATTGCCCTTAAGACTAGGGGAATAAATCTTTGGGCACCTAGTGGTCATG

1 CATGACCACTAGGAGCATCTTTGGCGAACTGCAGGTGTTGCGGTGGTCTGTGAATCACTAGGGGAATAAATCTTTGGGCACCTAGTGGTCATG

1 CATGACCACTAGGAGCATCTTTGGCGAGTGGTGGTAGGAAGCGGGAGCTCCGTCAAGCTAGGGGAATAAATCTTTGGGCACCTAGTGGTCATG

1 CATGACCACTAGGAGCATCTTTGGCGAGTATTGGCTGGTAGGTTGCGTATTGAGGAGCTAGGGGAATAAATCTTTGGGCACCTAGTGGTCATG

1 CATGACCACTAGGAGCATCTTTGGCGAGATCGGGAGAATCGGTCGCATTGGTGTCTACTAGGGGAATAAATCTTTGGGCACCTAGTGGTCATG

1 CATGACCACTAGGAGCATCTTTGGCGACCAGTGGTTTGTGGCCCAATGGGATGTACTCTAGGGGAATAAATCTTTGGGCACCTAGTGGTCATG

1 CATGACCACTAGGAGCATCTTTGGCGATTAGGGGACTCTACAAGGAGGGACTATGGGCTAGGGGAATAAATCTTTGGGCACCTAGTGGTCATG

1 CATGACCACTAGGAGCATCTTTGGCGACGGGATCGTGTCGGCTGATAGCGTACTTGACTAGGGGAATAAATCTTTGGGCACCTAGTGGTCATG

1 CATGACCACTAGGAGCATCTTTGGCGAACGGGTTGTGGATCCAAAATGGTGCTATCCCTAGGGGAATAAATCTTTGGGCACCTAGTGGTCATG

1 CATGACCACTAGGAGCATCTTTGGCGAGATAGGGAGAATCGGTGGCATTGGTGCCTCCTAGGGGAATAAATCTTTGGGCACCTAGTGGTCATG

1 CATGACCACTAGGAGCATCTTTGGCGAACAGGCGGTGGCTGCGGGTGGGTGCGCACCCTAGGGGAATAAATCTTTGGGCACCTAGTGGTCATG

1 CATGACCACTAGGAGCATCTTTGGCGACGTGGGCTGGGAGGATGCGGTACTTCTCGACTAGGGGAATAAATCTTTGGGCACCTAGTGGTCATG

1 CATGACCACTAGGAGCATCTTTGGCGAGTCGTGTACGGGTGGCGAACCTTTCGTAGGCTAGGGGAATAAATCTTTGGGCACCTAGTGGTCATG

1 CATGACCACTAGGAGCATCTTTGGCGAGATCGCCACAAACGGTGGCATTGGTGTCTCCTAGGGGAATAAATCTTTGGGCACCTAGTGGTCATG

1 CATGACCACTAGGAGCATCTTTGGCGAAAGTCGTCAGAACAGGGTGGGGTGCTGTCCCTAGGGGAATAAATCTTTGGGCACCTAGTGGTCATG

1 CATGACCACTAGGAGCATCTTTGGCGAACGGCAGGTTTTGAGGTGGTCTGTGAATACCTAGGGGAATAAATCTTTGGGCACCTAGTGGTCATG

1 CATGACCACTAGGAGCATCTTTGGCGAGGGAGGGCGCCGGCAGCGGTGTGAATGCAACTAGGGGAATAAATCTTTGGGCACCTAGTGGTCATG

1 CATGACCACTAGGAGCATCTTTGGCGAGATAGTGAGAATCGGTGGAATTGGTGTCTCCTAGGGGAATAAATCTTTGGGCACCTAGTGGTCATG

1 CATGACCACTAGGAGCATCTTTGGCGAGAACGGTATAATCTGTGGCAAAGGTGTCTCCTAGGGGAATAAATCTTTGGGCACCTAGTGGTCATG

1 CATGACCACTAGGAGCATCTTTGGCGAGAAGACTCTGGATACTGGGACCAGTTGCTGCTAGGGGAATAAATCTTTGGGCACCTAGTGGTCATG

1 CATGACCACTAGGAGCATCTTTGGCGACGGCGTGGGGCATCTCTCACGGCGGTTATTCTAGGGGAATAAATCTTTGGGCACCTAGTGGTCATG

1 CATGACCACTAGGAGCATCTTTGGCGATATTCCATGATAGGTTTTTCTTGCAGGAGACTAGGGGAATAAATCTTTGGGCACCTAGTGGTCATG

1 CATGACCACTAGGAGCATCTTTGGCGAGATCCGGAGAAACGGTGGCAATGGATTCTCCTAGGGGAATAAATCTTTGGGCACCTAGTGGTCATG

1 CATGACCACTAGGAGCATCTTTGGCGAGACCGGGAGAATTGGTGGCATTGGTGTCTCCTAGGGGAATAAATCTTTGGGCACCTAGTGGTCATG

1 CATGACCACTAGGAGCATCTTTGGCGAGATCGGGAGAATCTGTGGCATTGGGGTCTCCTAGGGGAATAAATCTTTGGGCACCTAGTGGTCATG

1 CATGACCACTAGGAGCATCTTTGGCGAGAAGACTCTGGATTCGGGGACCAGTCGCGGCTAGGGGAATAAATCTTTGGGCACCTAGTGGTCATG

1 CATGACCACTAGGAGCATCTTTGGCGAGATCGGGAGAATTGCTGGCATTGGTGTCTCCTAGGGGAATAAATCTTTGGGCACCTAGTGGTCATG

1 CATGACCACTAGGAGCATCTTTGGCGACTTCTGTTCGCCTAACCTGGACTTGGTGCCCTAGGGGAATAAATCTTTGGGCACCTAGTGGTCATG

1 CATGACCACTAGGAGCATCTTTGGCGACGGTGGTAGGAAAAGAACAGCTTACGTCGACTAGGGGAATAAATCTTTGGGCACCTAGTGGTCATG

1 CATGACCACTAGGAGCATCTTTGGCGAGATCGGGAGAATCGTCTGCATTGGTGTCTCCTAGGGGAATAAATCTTTGGGCACCTAGTGGTCATG

1 CATGACCACTAGGAGCATCTTTGGCGATCAAGTGCGCTTGCTAATTCCTGATGCGATCTAGGGGAATAAATCTTTGGGCACCTAGTGGTCATG

1 CATGACCACTAGGAGCATCTTTGGCGAAAGTCGGTAGAACAGGGTGCGGTGCTGTCCCTAGGGGAATAAATCTTTGGGCACCTAGTGGTCATG

1 CATGACCACTAGGAGCATCTTTGGCGAGATCGGCAGAATCGGTGGCATTGGTCTCACCTAGGGGAATAAATCTTTGGGCACCTAGTGGTCATG

1 CATGACCACTAGGAGCATCTTTGGCGACGCGGTCACTACGGTACTGACCGTCGTGGACTAGGGGAATAAATCTTTGGGCACCTAGTGGTCATG

1 CATGACCACTAGGAGCATCTTTGGCGACATAGTGGGCTGGCAGGGAGTACTTGTTGCCTAGGGGAATAAATCTTTGGGCACCTAGTGGTCATG

1 CATGACCACTAGGAGCATCTTTGGCGATAGTCGGTAGTACAGGGTGGGGTGCTGTCCCTAGGGGAATAAATCTTTGGGCACCTAGTGGTCATG

1 CATGACCACTAGGAGCATCTTTGGCGAATGCGAAAGAGGCAAGCTGTGGCTGACTCTCTAGGGGAATAAATCTTTGGGCACCTAGTGGTCATG

1 CATGACCACTAGGAGCATCTTTGGCGATCCGGGGGACCGTGTGGTCGACGCTTCGGGCTAGGGGAATAAATCTTTGGGCACCTAGTGGTCATG

1 CATGACCACTAGGAGCATCTTTGGCGAGTAGTGGGCCAGGAAATGGCAGCGCCCGAGCTAGGGGAATAAATCTTTGGGCACCTAGTGGTCATG

1 CATGACCACTAGGAGCATCTTTGGCGAGAAGACTCTTGATTCGGGGACTAGTTGCTGCTAGGGGAATAAATCTTTGGGCACCTAGTGGTCATG

1 CATGACCACTAGGAGCATCTTTGGCGAAGTGTGGTGGGCATGTGGGTGGTAGCGACGCTAGGGGAATAAATCTTTGGGCACCTAGTGGTCATG

1 CATGACCACTAGGAGCATCTTTGGCGAGGGGGGGCGCCGGCAGCGGTGTGAATACGACTAGGGGAATAAATCTTTGGGCACCTAGTGGTCATG

1 CATGACCACTAGGAGCATCTTTGGCGAGATCGGGAAAATCGGTGGCGTTGGTGTCTCCTAGGGGAATAAATCTTTGGGCACCTAGTGGTCATG

1 CATGACCACTAGGAGCATCTTTGGCGACGGGTGGGGTGTGGTCAAGCGTGGGCGTGCCTAGGGGAATAAATCTTTGGGCACCTAGTGGTCATG

1 CATGACCACTAGGAGCATCTTTGGCGAGATCGGGAGAATCGTTGGCATCGGTGTCTCCTAGGGGAATAAATCTTTGGGCACCTAGTGGTCATG

1 CATGACCACTAGGAGCATCTTTGGCGAGATCGGGAGAATCGTTGGCAATGGTTTCTCCTAGGGGAATAAATCTTTGGGCACCTAGTGGTCATG

1 CATGACCACTAGGAGCATCTTTGGCGAGATCTGGAGAATCGGTGGCATTGGTTTCTCCTAGGGGAATAAATCTTTGGGCACCTAGTGGTCATG

1 CATGACCACTAGGAGCATCTTTGGCGAAAGTTGATAGAACAGGGTGGGGTGCTGTCCCTAGGGGAATAAATCTTTGGGCACCTAGTGGTCATG

1 CATGACCACTAGGAGCATCTTTGGCGATGGGGCCGTGCTATCTGCACATTCCGCGGGCTAGGGGAATAAATCTTTGGGCACCTAGTGGTCATG

1 CATGACCACTAGGAGCATCTTTGGCGAAAGTCGGTAGAACAGGGTGGGGACCTGTCCCTAGGGGAATAAATCTTTGGGCACCTAGTGGTCATG

1 CATGACCACTAGGAGCATCTTTGGCGAGTATTGGCGGGTAGGTTGCGTATTGGGGAGCTAGGGGAATAAATCTTTGGGCACCTAGTGGTCATG

1 CATGACCACTAGGAGCATCTTTGGCGACACGTGTCTCGAAAGATGTCCGCCGTGGAACTAGGGGAATAAATCTTTGGGCACCTAGTGGTCATG

1 CATGACCACTAGGAGCATCTTTGGCGAATGGCCGGCACGGCCGTCTAGTCATCGGTACTAGGGGAATAAATCTTTGGGCACCTAGTGGTCATG

1 CATGACCACTAGGAGCATCTTTGGCGAGATCGGGAGAGTCGGTGGTATTGGTGTCTCCTAGGGGAATAAATCTTTGGGCACCTAGTGGTCATG

1 CATGACCACTAGGAGCATCTTTGGCGACGGGGGTCGTGAGGCACTATCGGCAGTCGACTAGGGGAATAAATCTTTGGGCACCTAGTGGTCATG

1 CATGACCACTAGGAGCATCTTTGGCGACCCGTGTGAGCAGAAAGGCTACCGGGTGGACTAGGGGAATAAATCTTTGGGCACCTAGTGGTCATG

1 CATGACCACTAGGAGCATCTTTGGCGAGATCGGGAGAATCGGTGGAATTGGTCTCTCCTAGGGGAATAAATCTTTGGGCACCTAGTGGTCATG

1 CATGACCACTAGGAGCATCTTTGGCGAGATCTGGAGAATCGGTGGCAGTGGTGTCTCCTAGGGGAATAAATCTTTGGGCACCTAGTGGTCATG

1 CATGACCACTAGGAGCATCTTTGGCGAGATCGGAAGAATCGGTGGCATTGGAGTCTCCTAGGGGAATAAATCTTTGGGCACCTAGTGGTCATG

1 CATGACCACTAGGAGCATCTTTGGCGACGACGTGGTCGTGGTGATGTGGCCGAGTCGCTAGGGGAATAAATCTTTGGGCACCTAGTGGTCATG

1 CATGACCACTAGGAGCATCTTTGGCGAAACATGTGACGAAGTTGCAATGAAGTGGAGCTAGGGGAATAAATCTTTGGGCACCTAGTGGTCATG

1 CATGACCACTAGGAGCATCTTTGGCGAGAAGACTCTGGATTCGGGAACCAGGTGCTGCTAGGGGAATAAATCTTTGGGCACCTAGTGGTCATG

1 CATGACCACTAGGAGCATCTTTGGCGAAGAGCAGAGACTACGCAAAGGTAAGTTCGCCTAGGGGAATAAATCTTTGGGCACCTAGTGGTCATG

1 CATGACCACTAGGAGCATCTTTGGCGACGGCGTGGGGCCATCTGCGCGGCGGATCTCCTAGGGGAATAAATCTTTGGGCACCTAGTGGTCATG

1 CATGACCACTAGGAGCATCTTTGGCGAGCGTAGTGTGGGCACGGTGCGGGTTTCCTCCTAGGGGAATAAATCTTTGGGCACCTAGTGGTCATG

1 CATGACCACTAGGAGCATCTTTGGCGACAAGCTCTGTTGTAGAGGCGCGACGTAGTGCTAGGGGAATAAATCTTTGGGCACCTAGTGGTCATG

1 CATGACCACTAGGAGCATCTTTGGCGACCGTGTCCCGAAGGAATAGTGGCCGAGCGCCTAGGGGAATAAATCTTTGGGCACCTAGTGGTCATG

1 CATGACCACTAGGAGCATCTTTGGCGAAGCGTGTTGCAAGGTCCGCGAGTGTGGGCCCTAGGGGAATAAATCTTTGGGCACCTAGTGGTCATG

1 CATGACCACTAGGAGCATCTTTGGCGAGCCTTGCTTGGGAGGTTGCGCCACCAGTTCCTAGGGGAATAAATCTTTGGGCACCTAGTGGTCATG

1 CATGACCACTAGGAGCATCTTTGGCGAGTGTGGGCGGTCGGGTGTAAGTGGTCGAGACTAGGGGAATAAATCTTTGGGCACCTAGTGGTCATG

1 CATGACCACTAGGAGCATCTTTGGCGATCAGCTGAGCGGCACACGTTGGTGGGGGGCCTAGGGGAATAAATCTTTGGGCACCTAGTGGTCATG

1 CATGACCACTAGGAGCATCTTTGGCGAACGGCAGGTGTTGCGTTTGTCTGTGAATCCCTAGGGGAATAAATCTTTGGGCACCTAGTGGTCATG

1 CATGACCACTAGGAGCATCTTTGGCGAGAAGACTCAGGATTCGGGGACCAGTAGCTGCTAGGGGAATAAATCTTTGGGCACCTAGTGGTCATG

1 CATGACCACTAGGAGCATCTTTGGCGAACGGCAGGTGTTGCGGTGGTCCGTGAATCACTAGGGGAATAAATCTTTGGGCACCTAGTGGTCATG

1 CATGACCACTAGGAGCATCTTTGGCGAGGGAGGGCGCCGGCAGCGGTGTGAATACGACTAGGGGAATAAATCTTTGGGCACCTAGTGGTCATG

1 CATGACCACTAGGAGCATCTTTGGCGAGATCGGGAGAAACGGTGTCATTGGAGTCTCCTAGGGGAATAAATCTTTGGGCACCTAGTGGTCATG

1 CATGACCACTAGGAGCATCTTTGGCGACTAGGGGTTGGCTGGTAGGGCCGGTGGTGACTAGGGGAATAAATCTTTGGGCACCTAGTGGTCATG

1 CATGACCACTAGGAGCATCTTTGGCGAGGTTGCGGGAATCCGACCAGCTGGTCGTCTCTAGGGGAATAAATCTTTGGGCACCTAGTGGTCATG

1 CATGACCACTAGGAGCATCTTTGGCGAGATCGGGAGAATCGGAGGCATTGGTGTCACCTAGGGGAATAAATCTTTGGGCACCTAGTGGTCATG

1 CATGACCACTAGGAGCATCTTTGGCGAGATCAGGAGAATCTGTGGCATTGGTGTCTCCTAGGGGAATAAATCTTTGGGCACCTAGTGGTCATG

1 CATGACCACTAGGAGCATCTTTGGCGATGGGGCTGTGCTATCTGCACACTCTGCGGGCTAGGGGAATAAATCTTTGGGCACCTAGTGGTCATG

1 CATGACCACTAGGAGCATCTTTGGCGACCCGGTTCAGGCAAGCCGACACTGGGTGGACTAGGGGAATAAATCTTTGGGCACCTAGTGGTCATG

1 CATGACCACTAGGAGCATCTTTGGCGAGAACGTGAGAATCGGAGGCATTGGTGTCTCCTAGGGGAATAAATCTTTGGGCACCTAGTGGTCATG

1 CATGACCACTAGGAGCATCTTTGGCGAAGAGATTGGACGTGAGTGGTGTGTATCGTTCTAGGGGAATAAATCTTTGGGCACCTAGTGGTCATG

1 CATGACCACTAGGAGCATCTTTGGCGACGGCGTGGGGCCATTTGCGCGGCGGATCCCCTAGGGGAATAAATCTTTGGGCACCTAGTGGTCATG

1 CATGACCACTAGGAGCATCTTTGGCGAGAGCGTGAGAATCGGTGGCATTGGTGTCTCCTAGGGGAATAAATCTTTGGGCACCTAGTGGTCATG

1 CATGACCACTAGGAGCATCTTTGGCGATCACGTTGCAAACGGAGTGGGGGGTTGCGACTAGGGGAATAAATCTTTGGGCACCTAGTGGTCATG

1 CATGACCACTAGGAGCATCTTTGGCGAGATCGGGAGACTAGGTGGCAGTGGTGTCTTCTAGGGGAATAAATCTTTGGGCACCTAGTGGTCATG

1 CATGACCACTAGGAGCATCTTTGGCGAGATCGGGAGAATAGGTGGCATTGGAGACTCCTAGGGGAATAAATCTTTGGGCACCTAGTGGTCATG

1 CATGACCACTAGGAGCATCTTTGGCGAGCGGCTGGTTTTCCGGGGGTGTGGGAAAACCTAGGGGAATAAATCTTTGGGCACCTAGTGGTCATG

1 CATGACCACTAGGAGCATCTTTGGCGAGTGGTGGTAGTAATCGAAGATTGCGTCAAGCTAGGGGAATAAATCTTTGGGCACCTAGTGGTCATG

1 CATGACCACTAGGAGCATCTTTGGCGAGCGATGTTTCTTCGCGTGTACGCTCTATGTCTAGGGGAATAAATCTTTGGGCACCTAGTGGTCATG

1 CATGACCACTAGGAGCATCTTTGGCGACGACCCGAGACGCGAATGCCCAGTGTCGAACTAGGGGAATAAATCTTTGGGCACCTAGTGGTCATG

1 CATGACCACTAGGAGCATCTTTGGCGAGATCGGGAGAAACGGTGGCATTTGTGACTCCTAGGGGAATAAATCTTTGGGCACCTAGTGGTCATG

1 CATGACCACTAGGAGCATCTTTGGCGAGATCGGGAGAGTCGGTGGCAGTGGTGTCACCTAGGGGAATAAATCTTTGGGCACCTAGTGGTCATG

1 CATGACCACTAGGAGCATCTTTGGCGAGTGTTGCCCGGTAAACGAGCAGACGTTTGGCTAGGGGAATAAATCTTTGGGCACCTAGTGGTCATG

1 CATGACCACTAGGAGCATCTTTGGCGACGAGGGGGCGGGAGATGTGGGGAACGGAGACTAGGGGAATAAATCTTTGGGCACCTAGTGGTCATG

1 CATGACCACTAGGAGCATCTTTGGCGACGACCGTGGGCATAGTAGGAAAGGAGACGCCTAGGGGAATAAATCTTTGGGCACCTAGTGGTCATG

1 CATGACCACTAGGAGCATCTTTGGCGAGATCGGGAGAATCGGTGGAATTTGTGACTACTAGGGGAATAAATCTTTGGGCACCTAGTGGTCATG

1 CATGACCACTAGGAGCATCTTTGGCGACGACCCTTTGGGTAATCACTAAATGCAGGCCTAGGGGAATAAATCTTTGGGCACCTAGTGGTCATG

1 CATGACCACTAGGAGCATCTTTGGCGAACTGCTGTTCCGGCGCTCCAGAAGATAAAGCTAGGGGAATAAATCTTTGGGCACCTAGTGGTCATG

1 CATGACCACTAGGAGCATCTTTGGCGAACGGCAGGTGTTGTGGTGGTCTGTGAACCCCTAGGGGAATAAATCTTTGGGCACCTAGTGGTCATG

1 CATGACCACTAGGAGCATCTTTGGCGATCCTCGTCCGATGGTGAAGCAGACGTTTGGCTAGGGGAATAAATCTTTGGGCACCTAGTGGTCATG

1 CATGACCACTAGGAGCATCTTTGGCGAGATCGGGAGAATCGGTGGCTGTGGTGTCTCCTAGGGGAATAAATCTTTGGGCACCTAGTGGTCATG

1 CATGACCACTAGGAGCATCTTTGGCGAGATCGGTAGAATCGGTGGAATTGGTGTTTCCTAGGGGAATAAATCTTTGGGCACCTAGTGGTCATG

1 CATGACCACTAGGAGCATCTTTGGCGACATGCAGGGTATGCGTTGGAATGTGAAAACCTAGGGGAATAAATCTTTGGGCACCTAGTGGTCATG

1 CATGACCACTAGGAGCATCTTTGGCGAGATCGGGAGAATCGGTGGCATCAGTGTCTCCTAGGGGAATAAATCTTTGGGCACCTAGTGGTCATG

1 CATGACCACTAGGAGCATCTTTGGCGAGATCGGGAGAATCGTTGGAATAGGTGTCTCCTAGGGGAATAAATCTTTGGGCACCTAGTGGTCATG

1 CATGACCACTAGGAGCATCTTTGGCGAGCCTTGCTTGGGAGGTTGCTCCACCAGAACCTAGGGGAATAAATCTTTGGGCACCTAGTGGTCATG

1 CATGACCACTAGGAGCATCTTTGGCGACGGTGTGGGGCTCTATGATGCGGCGGATTCCTAGGGGAATAAATCTTTGGGCACCTAGTGGTCATG

1 CATGACCACTAGGAGCATCTTTGGCGAGGGAGGGCGCCGGCAGCGGTTTGAATGCGACTAGGGGAATAAATCTTTGGGCACCTAGTGGTCATG

1 CATGACCACTAGGAGCATCTTTGGCGAGACTAGAGGAGAGCAGCGATCTCGAGTGGCCTAGGGGAATAAATCTTTGGGCACCTAGTGGTCATG

1 CATGACCACTAGGAGCATCTTTGGCGAGACATACTTGCTGGCTAGGAATGAATGCATCTAGGGGAATAAATCTTTGGGCACCTAGTGGTCATG

1 CATGACCACTAGGAGCATCTTTGGCGAGAGCGGGCGAATCGGTGGCATAGGTGTCTCCTAGGGGAATAAATCTTTGGGCACCTAGTGGTCATG

1 CATGACCACTAGGAGCATCTTTGGCGAGCCTCGCTTGGGAGGTTGCTCCACCAATTCCTAGGGGAATAAATCTTTGGGCACCTAGTGGTCATG

1 CATGACCACTAGGAGCATCTTTGGCGATAAACGGATGTGACTAGTGGCAGAGTACGCCTAGGGGAATAAATCTTTGGGCACCTAGTGGTCATG

1 CATGACCACTAGGAGCATCTTTGGCGAAATTTTGTTTCCAGCTGGTCGAGTGCACCACTAGGGGAATAAATCTTTGGGCACCTAGTGGTCATG

1 CATGACCACTAGGAGCATCTTTGGCGATCTAACAGAATGAGGGCTCACGGTAGACGCCTAGGGGAATAAATCTTTGGGCACCTAGTGGTCATG

1 CATGACCACTAGGAGCATCTTTGGCGAAGCAGTGCGACGGGCTTTGGGTTCCTCGAGCTAGGGGAATAAATCTTTGGGCACCTAGTGGTCATG

1 CATGACCACTAGGAGCATCTTTGGCGAGGGGATCCGAGGCATGGCGGTAAGTGCGACCTAGGGGAATAAATCTTTGGGCACCTAGTGGTCATG

1 CATGACCACTAGGAGCATCTTTGGCGATAGTCGGTAGAACAGGGTGGGGTGCTGTTCCTAGGGGAATAAATCTTTGGGCACCTAGTGGTCATG

1 CATGACCACTAGGAGCATCTTTGGCGAGATCGGGAGAATCGGTGCAATTGGTGTCTCCTAGGGGAATAAATCTTTGGGCACCTAGTGGTCATG

1 CATGACCACTAGGAGCATCTTTGGCGACCGGCAGGTGTTGCGGTGGTCTGTGCATCCCTAGGGGAATAAATCTTTGGGCACCTAGTGGTCATG

1 CATGACCACTAGGAGCATCTTTGGCGAGATCGAGAGAATCGTTGGCATTGGTGTCTCCTAGGGGAATAAATCTTTGGGCACCTAGTGGTCATG

1 CATGACCACTAGGAGCATCTTTGGCGACGATGGATGGGAGGGATGTATTTCGGGCGACTAGGGGAATAAATCTTTGGGCACCTAGTGGTCATG

1 CATGACCACTAGGAGCATCTTTGGCGAGGGTGGGCGCCGGCAGCGGTTTGAATGCGCCTAGGGGAATAAATCTTTGGGCACCTAGTGGTCATG

1 CATGACCACTAGGAGCATCTTTGGCGATGGATGATCGCTGAGCTAGTGGTCCTCGGCCTAGGGGAATAAATCTTTGGGCACCTAGTGGTCATG

1 CATGACCACTAGGAGCATCTTTGGCGAGAAGACTCTGTATTCGGGGAACAGTTGCTGCTAGGGGAATAAATCTTTGGGCACCTAGTGGTCATG

1 CATGACCACTAGGAGCATCTTTGGCGACGATGGTTGGGAGGGACGTATTTCGGGCGACTAGGGGAATAAATCTTTGGGCACCTAGTGGTCATG

1 CATGACCACTAGGAGCATCTTTGGCGAGGAGTGGCAGAGGGCGGTGGTTAGGTCGCGCTAGGGGAATAAATCTTTGGGCACCTAGTGGTCATG

1 CATGACCACTAGGAGCATCTTTGGCGAGATCGTGAGAATCGGTGTCATTGGTGACTCCTAGGGGAATAAATCTTTGGGCACCTAGTGGTCATG

1 CATGACCACTAGGAGCATCTTTGGCGACCCTGGCGTGGGGATCCTGGTTGCGGCGACCTAGGGGAATAAATCTTTGGGCACCTAGTGGTCATG

1 CATGACCACTAGGAGCATCTTTGGCGACGGTGTGGGGAACTTGTTTCGGCGGTGCCACTAGGGGAATAAATCTTTGGGCACCTAGTGGTCATG

1 CATGACCACTAGGAGCATCTTTGGCGAGATCGAGAGAATCGCTGGCATTGGTGTCTCCTAGGGGAATAAATCTTTGGGCACCTAGTGGTCATG

1 CATGACCACTAGGAGCATCTTTGGCGATCGCATAGCGGAGTGGTGCGGCACCGTGGCCTAGGGGAATAAATCTTTGGGCACCTAGTGGTCATG

1 CATGACCACTAGGAGCATCTTTGGCGAGGGAGGGCGCCGGCGGCGGTGTGAATGCGCCTAGGGGAATAAATCTTTGGGCACCTAGTGGTCATG

1 CATGACCACTAGGAGCATCTTTGGCGACGTGAGTAGCTTAGTGGCTGAGCTCCTAGACTAGGGGAATAAATCTTTGGGCACCTAGTGGTCATG

1 CATGACCACTAGGAGCATCTTTGGCGAGAAGACTCTGGATTCGGGGACCAGTTCCTACTAGGGGAATAAATCTTTGGGCACCTAGTGGTCATG

1 CATGACCACTAGGAGCATCTTTGGCGAACTGCAGGAGTTGCGGTGGTCTGTGAATCCCTAGGGGAATAAATCTTTGGGCACCTAGTGGTCATG

1 CATGACCACTAGGAGCATCTTTGGCGACAACTGATCGCTAAGCTCCCGGGTTGCTCCCTAGGGGAATAAATCTTTGGGCACCTAGTGGTCATG

1 CATGACCACTAGGAGCATCTTTGGCGAGTGGTCAGAGGGAGTCGGTCCGGGTCAAGACTAGGGGAATAAATCTTTGGGCACCTAGTGGTCATG

1 CATGACCACTAGGAGCATCTTTGGCGAGCGTTCAGGATTCGATTGGAGTTGCCAACGCTAGGGGAATAAATCTTTGGGCACCTAGTGGTCATG

1 CATGACCACTAGGAGCATCTTTGGCGAAGCGCGGTGGATCCACGGCAGTGGTAGAGGCTAGGGGAATAAATCTTTGGGCACCTAGTGGTCATG

1 CATGACCACTAGGAGCATCTTTGGCGAGATCGGGAGACTCGGTGGCACTGGTGTCTCCTAGGGGAATAAATCTTTGGGCACCTAGTGGTCATG

1 CATGACCACTAGGAGCATCTTTGGCGAGGGGTCATGGGAATGTAATTGCACTTAAGACTAGGGGAATAAATCTTTGGGCACCTAGTGGTCATG

1 CATGACCACTAGGAGCATCTTTGGCGAGAAGACTCTGGAATCTGGGACCAGTTGCTGCTAGGGGAATAAATCTTTGGGCACCTAGTGGTCATG

1 CATGACCACTAGGAGCATCTTTGGCGAGATCGGGAGAATCGGTGGCCATGGTGTCTACTAGGGGAATAAATCTTTGGGCACCTAGTGGTCATG

1 CATGACCACTAGGAGCATCTTTGGCGAGAACGGTAGGATCGGTGGCATTGGTGTCTCCTAGGGGAATAAATCTTTGGGCACCTAGTGGTCATG

1 CATGACCACTAGGAGCATCTTTGGCGAGGAGTGGCTGAGGGCGGTGGGTAGGTCGTGCTAGGGGAATAAATCTTTGGGCACCTAGTGGTCATG

1 CATGACCACTAGGAGCATCTTTGGCGAACGTCAGGTGATGCGGTTGTCTGTGAATCCCTAGGGGAATAAATCTTTGGGCACCTAGTGGTCATG

1 CATGACCACTAGGAGCATCTTTGGCGAGTGAGTGTAACGGTCGTCAAGGTGTACTGCCTAGGGGAATAAATCTTTGGGCACCTAGTGGTCATG

1 CATGACCACTAGGAGCATCTTTGGCGAAATCGGGAGAATCGGTGGCATTGGCGTCTCCTAGGGGAATAAATCTTTGGGCACCTAGTGGTCATG

1 CATGACCACTAGGAGCATCTTTGGCGAAGCGTGTCTTCGTAGTGCTCTCACAGGGTGCTAGGGGAATAAATCTTTGGGCACCTAGTGGTCATG

1 CATGACCACTAGGAGCATCTTTGGCGAACGGTAGGTGCTGCGGTGGTCTGTGGATCCCTAGGGGAATAAATCTTTGGGCACCTAGTGGTCATG

1 CATGACCACTAGGAGCATCTTTGGCGACTGGTTAGGGCACGTTCTCAGTCGCAGACACTAGGGGAATAAATCTTTGGGCACCTAGTGGTCATG

1 CATGACCACTAGGAGCATCTTTGGCGAGATCGGGAGAATCGGTGGCATACCACACTCCTAGGGGAATAAATCTTTGGGCACCTAGTGGTCATG

1 CATGACCACTAGGAGCATCTTTGGCGAACGGCAGGTTTTGCGTTGGTCTGTGAATCCCTAGGGGAATAAATCTTTGGGCACCTAGTGGTCATG

1 CATGACCACTAGGAGCATCTTTGGCGAGCCGATAGTGCAACAATGTGTTCTGGGTAACTAGGGGAATAAATCTTTGGGCACCTAGTGGTCATG

1 CATGACCACTAGGAGCATCTTTGGCGAGATCTGGAGAATCGGTGGCATTGGTGTCTACTAGGGGAATAAATCTTTGGGCACCTAGTGGTCATG

1 CATGACCACTAGGAGCATCTTTGGCGAATGGCCGGCACGGCCCTCTAGTCCTTGGTACTAGGGGAATAAATCTTTGGGCACCTAGTGGTCATG

1 CATGACCACTAGGAGCATCTTTGGCGAGATCGTGAGAATCGGTGGCATTGGTTTCACCTAGGGGAATAAATCTTTGGGCACCTAGTGGTCATG

1 CATGACCACTAGGAGCATCTTTGGCGATCCGGGGGGATTGGGTCCGACGCAATAGGGCTAGGGGAATAAATCTTTGGGCACCTAGTGGTCATG

1 CATGACCACTAGGAGCATCTTTGGCGACCCTTTGGGAGTGGATCGGCAGAGAGCCGACTAGGGGAATAAATCTTTGGGCACCTAGTGGTCATG

1 CATGACCACTAGGAGCATCTTTGGCGATCCTCGTAAGAATGTGAAGCAGACGTTTGGCTAGGGGAATAAATCTTTGGGCACCTAGTGGTCATG

1 CATGACCACTAGGAGCATCTTTGGCGACTAGTAGCGCACGGCGCGAGGTGTGTTGGACTAGGGGAATAAATCTTTGGGCACCTAGTGGTCATG

1 CATGACCACTAGGAGCATCTTTGGCGATCGCATAGCGGATGCTTACGGGCACGTGGCCTAGGGGAATAAATCTTTGGGCACCTAGTGGTCATG

1 CATGACCACTAGGAGCATCTTTGGCGAACGGTGGTCGAGGGATACAGAGGGCATATGCTAGGGGAATAAATCTTTGGGCACCTAGTGGTCATG

1 CATGACCACTAGGAGCATCTTTGGCGAACGGCAGGTGTTGCGGTGGTCTGTCAATCACTAGGGGAATAAATCTTTGGGCACCTAGTGGTCATG

1 CATGACCACTAGGAGCATCTTTGGCGAACGTCAGGTGTTGCGGTGGTCTGTGAACCCCTAGGGGAATAAATCTTTGGGCACCTAGTGGTCATG

1 CATGACCACTAGGAGCATCTTTGGCGAGGGAGGTCGCCGGCAGCGGTGTGAATGAGACTAGGGGAATAAATCTTTGGGCACCTAGTGGTCATG

1 CATGACCACTAGGAGCATCTTTGGCGATCCGGGGGCATGCTATGCGATGCTTATGGGCTAGGGGAATAAATCTTTGGGCACCTAGTGGTCATG

1 CATGACCACTAGGAGCATCTTTGGCGAGGGGCATTTGGAGGAATGAGAATATTTATGCTAGGGGAATAAATCTTTGGGCACCTAGTGGTCATG

1 CATGACCACTAGGAGCATCTTTGGCGACTGAAGCACTGTGAGAACGGTGCCAGTGGACTAGGGGAATAAATCTTTGGGCACCTAGTGGTCATG

1 CATGACCACTAGGAGCATCTTTGGCGAGCCTCGCTTGGGATGTTGCTCCACCAGTTCCTAGGGGAATAAATCTTTGGGCACCTAGTGGTCATG

1 CATGACCACTAGGAGCATCTTTGGCGAGGTCACCGTATCTCTCACAGGTGTGTTCATCTAGGGGAATAAATCTTTGGGCACCTAGTGGTCATG

1 CATGACCACTAGGAGCATCTTTGGCGACCGGCCGGTGTTGCGGTGGTCTGTGAATCCCTAGGGGAATAAATCTTTGGGCACCTAGTGGTCATG

1 CATGACCACTAGGAGCATCTTTGGCGATCGGGCCGCCAGGGCGTACGAAGTGTGGTTCTAGGGGAATAAATCTTTGGGCACCTAGTGGTCATG

1 CATGACCACTAGGAGCATCTTTGGCGAATCAGAGCATTATTCCTGCGTAGTTAAAGTCTAGGGGAATAAATCTTTGGGCACCTAGTGGTCATG

1 CATGACCACTAGGAGCATCTTTGGCGACGGAGTGGGGCTTATTGTGCGGCGGTAATCCTAGGGGAATAAATCTTTGGGCACCTAGTGGTCATG

1 CATGACCACTAGGAGCATCTTTGGCGAGAAGACTTTGGATTCGGGGGCCAGTTGCTGCTAGGGGAATAAATCTTTGGGCACCTAGTGGTCATG

1 CATGACCACTAGGAGCATCTTTGGCGAGAAGACTCTGGAGTCGGGGACCAGTTTCTGCTAGGGGAATAAATCTTTGGGCACCTAGTGGTCATG

1 CATGACCACTAGGAGCATCTTTGGCGAACGGCAGGTGTTTCGGTGGTCTGAGAAACCCTAGGGGAATAAATCTTTGGGCACCTAGTGGTCATG

1 CATGACCACTAGGAGCATCTTTGGCGAGATCGGCAGAATCGGTGGCAGTGGTGTCTCCTAGGGGAATAAATCTTTGGGCACCTAGTGGTCATG

1 CATGACCACTAGGAGCATCTTTGGCGATATCGGGAGAAACGGTTGCATTGGTGTCTCCTAGGGGAATAAATCTTTGGGCACCTAGTGGTCATG

1 CATGACCACTAGGAGCATCTTTGGCGACGCGTGTTGCAAGGTCCGCGAGTGTGGACCCTAGGGGAATAAATCTTTGGGCACCTAGTGGTCATG

1 CATGACCACTAGGAGCATCTTTGGCGATATAGGGAGAATCCGTGGCATTGGTGTCTCCTAGGGGAATAAATCTTTGGGCACCTAGTGGTCATG

1 CATGACCACTAGGAGCATCTTTGGCGACCCAATTTGTAAGAATTACGAGTGGGTGGACTAGGGGAATAAATCTTTGGGCACCTAGTGGTCATG

1 CATGACCACTAGGAGCATCTTTGGCGAAATCGGGAGAATCGGTGGCATTGGAGTCTCCTAGGGGAATAAATCTTTGGGCACCTAGTGGTCATG

1 CATGACCACTAGGAGCATCTTTGGCGAGATCGGGAGAATCGGTGGCATTATTGTCTACTAGGGGAATAAATCTTTGGGCACCTAGTGGTCATG

1 CATGACCACTAGGAGCATCTTTGGCGACTGGCGTTTAGAGGTAGCCATGGAATTAACCTAGGGGAATAAATCTTTGGGCACCTAGTGGTCATG

1 CATGACCACTAGGAGCATCTTTGGCGACTAAGTGGACCACGCGTTAAGTGGGGTAGACTAGGGGAATAAATCTTTGGGCACCTAGTGGTCATG

1 CATGACCACTAGGAGCATCTTTGGCGAGATCCAGAGAATCGTTGGCAATGGTGTCTCCTAGGGGAATAAATCTTTGGGCACCTAGTGGTCATG

1 CATGACCACTAGGAGCATCTTTGGCGACGAGATTTGGTAGGTAGATGAGCTCTAGCACTAGGGGAATAAATCTTTGGGCACCTAGTGGTCATG

1 CATGACCACTAGGAGCATCTTTGGCGATGATGGGGACTATGTATTTATGCGCCTTGACTAGGGGAATAAATCTTTGGGCACCTAGTGGTCATG

1 CATGACCACTAGGAGCATCTTTGGCGAGATCGGGAGAATCGGTTGCATTTGTGACTCCTAGGGGAATAAATCTTTGGGCACCTAGTGGTCATG

1 CATGACCACTAGGAGCATCTTTGGCGATCGGATCTGCCCGCGGGACCGGTCCTTGCTCTAGGGGAATAAATCTTTGGGCACCTAGTGGTCATG

1 CATGACCACTAGGAGCATCTTTGGCGAGAAGATTCCGGATTCGGGGACCAGTTGCTCCTAGGGGAATAAATCTTTGGGCACCTAGTGGTCATG

1 CATGACCACTAGGAGCATCTTTGGCGATGTGTTGTTAAAGTGGTAATTGTTTTTTATCTAGGGGAATAAATCTTTGGGCACCTAGTGGTCATG

1 CATGACCACTAGGAGCATCTTTGGCGATTAGCTAAGCGACCCGAGACACGGGTGGGCCTAGGGGAATAAATCTTTGGGCACCTAGTGGTCATG

1 CATGACCACTAGGAGCATCTTTGGCGACTCCTTACGCCGGTAGTCTGGGGTGCTTCCCTAGGGGAATAAATCTTTGGGCACCTAGTGGTCATG

1 CATGACCACTAGGAGCATCTTTGGCGAGGTCGGGAGAGTCGGTGGCATTGGTGTCTCCTAGGGGAATAAATCTTTGGGCACCTAGTGGTCATG

1 CATGACCACTAGGAGCATCTTTGGCGATTCGTTATTCCAGCACCCTGATTGTGACCTCTAGGGGAATAAATCTTTGGGCACCTAGTGGTCATG

1 CATGACCACTAGGAGCATCTTTGGCGAGATCTGGAGAATAGGTGTCATTGGTGTCTCCTAGGGGAATAAATCTTTGGGCACCTAGTGGTCATG

1 CATGACCACTAGGAGCATCTTTGGCGATGGGTGCTGACGGCCGTCGCTGCGGCTACACTAGGGGAATAAATCTTTGGGCACCTAGTGGTCATG

1 CATGACCACTAGGAGCATCTTTGGCGAGATCGGGATAATCGGTGTCATTGGTGTCTCCTAGGGGAATAAATCTTTGGGCACCTAGTGGTCATG

1 CATGACCACTAGGAGCATCTTTGGCGACAAGTGGTGATAACATACGCGACGTAGTGACTAGGGGAATAAATCTTTGGGCACCTAGTGGTCATG

1 CATGACCACTAGGAGCATCTTTGGCGAAAGTCGGTAGAACAGGGTGGGGTGCTGTGCCTAGGGGAATAAATCTTTGGGCACCTAGTGGTCATG

1 CATGACCACTAGGAGCATCTTTGGCGAACGGCAAGTGTTGCGGTGGTATGTGAATCCCTAGGGGAATAAATCTTTGGGCACCTAGTGGTCATG

1 CATGACCACTAGGAGCATCTTTGGCGAGATCGCGAGAATCGGTGGAATTGGTGTCTCCTAGGGGAATAAATCTTTGGGCACCTAGTGGTCATG

1 CATGACCACTAGGAGCATCTTTGGCGAGATCGGTCGAATCGGTGGCATTGGTGTCTCCTAGGGGAATAAATCTTTGGGCACCTAGTGGTCATG

1 CATGACCACTAGGAGCATCTTTGGCGAGGGAGGGCGCCGGCAGCGGTGTGAATTCGCCTAGGGGAATAAATCTTTGGGCACCTAGTGGTCATG

1 CATGACCACTAGGAGCATCTTTGGCGACGCGGAACCCAGTGATGGGGTCGAAGTGGACTAGGGGAATAAATCTTTGGGCACCTAGTGGTCATG

1 CATGACCACTAGGAGCATCTTTGGCGAGATCGGGAGAATCGGAGGCAATGGAGTCTACTAGGGGAATAAATCTTTGGGCACCTAGTGGTCATG

1 CATGACCACTAGGAGCATCTTTGGCGAGATCGGGAGGATCGGTGGCATTGTTGTCTCCTAGGGGAATAAATCTTTGGGCACCTAGTGGTCATG

1 CATGACCACTAGGAGCATCTTTGGCGAAGGGGGTAGGACTTCAAGTGGCTCTCATAGCTAGGGGAATAAATCTTTGGGCACCTAGTGGTCATG

1 CATGACCACTAGGAGCATCTTTGGCGAAGTACGAAGGTGCCCTGGCGTGGTTGAGCCCTAGGGGAATAAATCTTTGGGCACCTAGTGGTCATG

1 CATGACCACTAGGAGCATCTTTGGCGACGGTGGGCAGGTGCTACGTGGCTGAGGAGACTAGGGGAATAAATCTTTGGGCACCTAGTGGTCATG

1 CATGACCACTAGGAGCATCTTTGGCGAGAAGACTCTGGAATCGGGGGCCAGTTGCTGCTAGGGGAATAAATCTTTGGGCACCTAGTGGTCATG

1 CATGACCACTAGGAGCATCTTTGGCGATTAGCACAGCGAAGACTGCAGTGTCGGGGCCTAGGGGAATAAATCTTTGGGCACCTAGTGGTCATG

1 CATGACCACTAGGAGCATCTTTGGCGAGATGGGGTGTATCGGTGGGAGTGTTGAAACCTAGGGGAATAAATCTTTGGGCACCTAGTGGTCATG

1 CATGACCACTAGGAGCATCTTTGGCGAATGAGTGTCGGCACCCCGTCTTTGGTTATACTAGGGGAATAAATCTTTGGGCACCTAGTGGTCATG

1 CATGACCACTAGGAGCATCTTTGGCGACCGTAGGAAAATAAAAGTGGCCTTGCTAGCCTAGGGGAATAAATCTTTGGGCACCTAGTGGTCATG

1 CATGACCACTAGGAGCATCTTTGGCGATCCTCGTCAGATGGTGAAGTAGACGTTTGGCTAGGGGAATAAATCTTTGGGCACCTAGTGGTCATG

1 CATGACCACTAGGAGCATCTTTGGCGACCCGGTGTGGTGTGGTAGTGACGAAGAAGACTAGGGGAATAAATCTTTGGGCACCTAGTGGTCATG

1 CATGACCACTAGGAGCATCTTTGGCGAGATCGGGAGGATCGGTTGCATTGGTGTCTCCTAGGGGAATAAATCTTTGGGCACCTAGTGGTCATG

1 CATGACCACTAGGAGCATCTTTGGCGAGATCGGTAGAATCGGTGGCACTGGTGTCTCCTAGGGGAATAAATCTTTGGGCACCTAGTGGTCATG

1 CATGACCACTAGGAGCATCTTTGGCGATGGGTGCTGACGGGGCTAGTGAGCCTTACACTAGGGGAATAAATCTTTGGGCACCTAGTGGTCATG

1 CATGACCACTAGGAGCATCTTTGGCGAGGACTTATGGTATTTGCCTGGCTTTGCGCCCTAGGGGAATAAATCTTTGGGCACCTAGTGGTCATG

1 CATGACCACTAGGAGCATCTTTGGCGAGGGGAGATGGATGGCTATCCAGTATGAGTTCTAGGGGAATAAATCTTTGGGCACCTAGTGGTCATG

1 CATGACCACTAGGAGCATCTTTGGCGAACACACATGTGGGGGGGCTAATTTCGATGCCTAGGGGAATAAATCTTTGGGCACCTAGTGGTCATG

1 CATGACCACTAGGAGCATCTTTGGCGATACTTGACAGCAGACTCGGTGTTTGTTCTACTAGGGGAATAAATCTTTGGGCACCTAGTGGTCATG

1 CATGACCACTAGGAGCATCTTTGGCGAGAAGACTCTGGAGTCTGGGACCAGTTTCTGCTAGGGGAATAAATCTTTGGGCACCTAGTGGTCATG

1 CATGACCACTAGGAGCATCTTTGGCGAGATCGGGAGAATCGGTGGAATTCGTGTCTCCTAGGGGAATAAATCTTTGGGCACCTAGTGGTCATG

1 CATGACCACTAGGAGCATCTTTGGCGATCGCACAGCGGATACAGTCAAGGTACGGGACTAGGGGAATAAATCTTTGGGCACCTAGTGGTCATG

1 CATGACCACTAGGAGCATCTTTGGCGAACGGCAGGTGTTGCGGTTGTCTGTAAATCCCTAGGGGAATAAATCTTTGGGCACCTAGTGGTCATG

1 CATGACCACTAGGAGCATCTTTGGCGACGTGGGCCGGGAGGATGTGGTACTTCTCGACTAGGGGAATAAATCTTTGGGCACCTAGTGGTCATG

1 CATGACCACTAGGAGCATCTTTGGCGAACCGTTTGGAGAGCGGAGTGGCTGAGCTACCTAGGGGAATAAATCTTTGGGCACCTAGTGGTCATG

1 CATGACCACTAGGAGCATCTTTGGCGAACGGCAGGTGTTGCGGTGGACTGTGTATCCCTAGGGGAATAAATCTTTGGGCACCTAGTGGTCATG

1 CATGACCACTAGGAGCATCTTTGGCGAGTCGACAAACGTGCGTGAGTGGTCGATTGCCTAGGGGAATAAATCTTTGGGCACCTAGTGGTCATG

1 CATGACCACTAGGAGCATCTTTGGCGAACGGCAGGTGTAGCGGTGGTCTATGAATCCCTAGGGGAATAAATCTTTGGGCACCTAGTGGTCATG

1 CATGACCACTAGGAGCATCTTTGGCGAGATCGGGAGACTCGGTGGCATCGGTGTCTCCTAGGGGAATAAATCTTTGGGCACCTAGTGGTCATG

1 CATGACCACTAGGAGCATCTTTGGCGAGGAGCTACGGTGCGCGGTCAGGTATGTCTGCTAGGGGAATAAATCTTTGGGCACCTAGTGGTCATG

1 CATGACCACTAGGAGCATCTTTGGCGAGATCTGGAGAATCGGTGGCATTGGCGTCTCCTAGGGGAATAAATCTTTGGGCACCTAGTGGTCATG

1 CATGACCACTAGGAGCATCTTTGGCGAATGGTGGGTGAGCGACGTGAGGGATGTCGACTAGGGGAATAAATCTTTGGGCACCTAGTGGTCATG

1 CATGACCACTAGGAGCATCTTTGGCGAGATCGGGAGAATCGGTCCCATTGGTGTCTCCTAGGGGAATAAATCTTTGGGCACCTAGTGGTCATG

1 CATGACCACTAGGAGCATCTTTGGCGAACACGAGAGTCAAGGGCGTATAGCGTGGAGCTAGGGGAATAAATCTTTGGGCACCTAGTGGTCATG

1 CATGACCACTAGGAGCATCTTTGGCGATCCTCGTCAGATGGCGAAGCAGACGTTTGGCTAGGGGAATAAATCTTTGGGCACCTAGTGGTCATG

1 CATGACCACTAGGAGCATCTTTGGCGAGGGAGGGCGCGGGCAGCGGTGTGAATGCGACTAGGGGAATAAATCTTTGGGCACCTAGTGGTCATG

1 CATGACCACTAGGAGCATCTTTGGCGATGGGTGCTGACGACCGTCTACACGGTTACTCTAGGGGAATAAATCTTTGGGCACCTAGTGGTCATG

1 CATGACCACTAGGAGCATCTTTGGCGAGATCGGGAGAATCTGCGGCATTTGTGTCTCCTAGGGGAATAAATCTTTGGGCACCTAGTGGTCATG

1 CATGACCACTAGGAGCATCTTTGGCGATCCGGGGGCATCTATGTGCGAAGCTGTGGGCTAGGGGAATAAATCTTTGGGCACCTAGTGGTCATG

1 CATGACCACTAGGAGCATCTTTGGCGAAAGGCAGGTGTTGCGGTGTTCTGTGAATCCCTAGGGGAATAAATCTTTGGGCACCTAGTGGTCATG

1 CATGACCACTAGGAGCATCTTTGGCGACAGTGGGGCTGGTAGGAAAGTACTTATAGCCTAGGGGAATAAATCTTTGGGCACCTAGTGGTCATG

1 CATGACCACTAGGAGCATCTTTGGCGAACGGCAGGTGTTGCGGTGGTCCGTGAATTCCTAGGGGAATAAATCTTTGGGCACCTAGTGGTCATG

1 CATGACCACTAGGAGCATCTTTGGCGAACGGCAGATGTAGCGGTGGTCTGTGAATCCCTAGGGGAATAAATCTTTGGGCACCTAGTGGTCATG

1 CATGACCACTAGGAGCATCTTTGGCGACCAAATATGGGTTTACAGAAGGCGCATTACCTAGGGGAATAAATCTTTGGGCACCTAGTGGTCATG

1 CATGACCACTAGGAGCATCTTTGGCGAGTTACACTCTGCTGTCTTACTTTGCGGTGCCTAGGGGAATAAATCTTTGGGCACCTAGTGGTCATG

1 CATGACCACTAGGAGCATCTTTGGCGAGATAACAGAGTGAGGTGTTAAATTGAATTTCTAGGGGAATAAATCTTTGGGCACCTAGTGGTCATG

1 CATGACCACTAGGAGCATCTTTGGCGAAGCAGAAGGTGTGAGGTTGTAAGAGAACTACTAGGGGAATAAATCTTTGGGCACCTAGTGGTCATG

1 CATGACCACTAGGAGCATCTTTGGCGAATCTCCCTGCGTACGTCGGAGTTCTCATCACTAGGGGAATAAATCTTTGGGCACCTAGTGGTCATG

1 CATGACCACTAGGAGCATCTTTGGCGAGCGAAGGTAACGGTGTGCGTAAAGGAGCTTCTAGGGGAATAAATCTTTGGGCACCTAGTGGTCATG

1 CATGACCACTAGGAGCATCTTTGGCGATCAGGGGGCCTTTGATGGCGACTAAGTGGGCTAGGGGAATAAATCTTTGGGCACCTAGTGGTCATG

1 CATGACCACTAGGAGCATCTTTGGCGAGATCGGGATAATCGGTGGCATTGGTGTATCCTAGGGGAATAAATCTTTGGGCACCTAGTGGTCATG

1 CATGACCACTAGGAGCATCTTTGGCGATCGCATAGCGGAACAAGGCATCGTGTGGGCCTAGGGGAATAAATCTTTGGGCACCTAGTGGTCATG

1 CATGACCACTAGGAGCATCTTTGGCGAACGTAGTCGAAGTGGAGGTGCTACTGAGCTCTAGGGGAATAAATCTTTGGGCACCTAGTGGTCATG

1 CATGACCACTAGGAGCATCTTTGGCGACGGGGAATCAAGGAGGGCTGGGGAGTGTTGCTAGGGGAATAAATCTTTGGGCACCTAGTGGTCATG

1 CATGACCACTAGGAGCATCTTTGGCGAGGCGCGATGGGTGGCCCGTGGCTTTCAGCCCTAGGGGAATAAATCTTTGGGCACCTAGTGGTCATG

1 CATGACCACTAGGAGCATCTTTGGCGACCCGCGAAGGACGTGACGGACAACCTGTGACTAGGGGAATAAATCTTTGGGCACCTAGTGGTCATG

1 CATGACCACTAGGAGCATCTTTGGCGAGGGAGGGCGCCGGCAGCGGTTTGAATGCGCCTAGGGGAATAAATCTTTGGGCACCTAGTGGTCATG

1 CATGACCACTAGGAGCATCTTTGGCGACAAGGGAGCGACGGTGTGGTCTGAGTCCTACTAGGGGAATAAATCTTTGGGCACCTAGTGGTCATG

1 CATGACCACTAGGAGCATCTTTGGCGAACGGCAGGTGTTGCGGTGGTCTTTGAGTCCCTAGGGGAATAAATCTTTGGGCACCTAGTGGTCATG

1 CATGACCACTAGGAGCATCTTTGGCGAACGGCAGGTGATCCGGTGGTCTGTGAATCCCTAGGGGAATAAATCTTTGGGCACCTAGTGGTCATG

1 CATGACCACTAGGAGCATCTTTGGCGAGATCGGTAGAATCTCTGGAATTGGTGTCTCCTAGGGGAATAAATCTTTGGGCACCTAGTGGTCATG

1 CATGACCACTAGGAGCATCTTTGGCGAGATCGGGATAGTCGGTGGCATTGGTGTCTCCTAGGGGAATAAATCTTTGGGCACCTAGTGGTCATG

1 CATGACCACTAGGAGCATCTTTGGCGAGATCGGTATTAACTCTGGGAGAGGTGTCTCCTAGGGGAATAAATCTTTGGGCACCTAGTGGTCATG

1 CATGACCACTAGGAGCATCTTTGGCGACGTCATCGGGGGGTGAATCAACGTTACCCCCTAGGGGAATAAATCTTTGGGCACCTAGTGGTCATG

1 CATGACCACTAGGAGCATCTTTGGCGACCACGGAGCGATGCGTTGAGTGTGGTAAGCCTAGGGGAATAAATCTTTGGGCACCTAGTGGTCATG

1 CATGACCACTAGGAGCATCTTTGGCGAGGGTGGGCCAGGGGCTGTCGTACCCCAGTCCTAGGGGAATAAATCTTTGGGCACCTAGTGGTCATG

1 CATGACCACTAGGAGCATCTTTGGCGAACGGCAGGTGTTGCGGCGGTCTGTGAATCGCTAGGGGAATAAATCTTTGGGCACCTAGTGGTCATG

1 CATGACCACTAGGAGCATCTTTGGCGAGATGGGGAGAATCGGTGGCATTGGTGTTTCCTAGGGGAATAAATCTTTGGGCACCTAGTGGTCATG

1 CATGACCACTAGGAGCATCTTTGGCGATCAGGGGGCCTTTGATGGAGACTCAGTGGGCTAGGGGAATAAATCTTTGGGCACCTAGTGGTCATG

1 CATGACCACTAGGAGCATCTTTGGCGACTTTGCCGGGCAGACCGCGCCGTTAGTTGTCTAGGGGAATAAATCTTTGGGCACCTAGTGGTCATG

1 CATGACCACTAGGAGCATCTTTGGCGAGTCGTGTGGGTTCGATAGTGCTGAGTCCCCCTAGGGGAATAAATCTTTGGGCACCTAGTGGTCATG

1 CATGACCACTAGGAGCATCTTTGGCGAGAGCTTGAAAAAGGTGCGTTGTTGCCGCATCTAGGGGAATAAATCTTTGGGCACCTAGTGGTCATG

1 CATGACCACTAGGAGCATCTTTGGCGAGATCGGGAGTATCGGCGGCATTGGTGCCTCCTAGGGGAATAAATCTTTGGGCACCTAGTGGTCATG

1 CATGACCACTAGGAGCATCTTTGGCGACGGCGTGGGGCCATCTGTGCGGCAGATCCCCTAGGGGAATAAATCTTTGGGCACCTAGTGGTCATG

1 CATGACCACTAGGAGCATCTTTGGCGAGATCGGGAGAATCGGTGGCATTGGAATCTCCTAGGGGAATAAATCTTTGGGCACCTAGTGGTCATG

1 CATGACCACTAGGAGCATCTTTGGCGACTTTATAAACACGGACGTGTATGAAGTGGACTAGGGGAATAAATCTTTGGGCACCTAGTGGTCATG

1 CATGACCACTAGGAGCATCTTTGGCGAGTGGGGGTCGACGGCCGGTAGTGGGTGAGACTAGGGGAATAAATCTTTGGGCACCTAGTGGTCATG

1 CATGACCACTAGGAGCATCTTTGGCGAATATCGGTAGCGGTGTCTTAAGAGATGGCCCTAGGGGAATAAATCTTTGGGCACCTAGTGGTCATG

1 CATGACCACTAGGAGCATCTTTGGCGACGATCGGTCGGAAGGTAAGTGTTCGGATGACTAGGGGAATAAATCTTTGGGCACCTAGTGGTCATG

1 CATGACCACTAGGAGCATCTTTGGCGATTTTCCATGATCTCGCCGGTCCGGGGTTTACTAGGGGAATAAATCTTTGGGCACCTAGTGGTCATG

1 CATGACCACTAGGAGCATCTTTGGCGAAAGGCAGGTGTTGCGGTGGTCTGTGAATCGCTAGGGGAATAAATCTTTGGGCACCTAGTGGTCATG

1 CATGACCACTAGGAGCATCTTTGGCGAGGAATGGCTGAGGGCGGTGGGTAGGTCGCGCTAGGGGAATAAATCTTTGGGCACCTAGTGGTCATG

1 CATGACCACTAGGAGCATCTTTGGCGAGATCGTGAGAATCGGTGGCATTGGTGTATCCTAGGGGAATAAATCTTTGGGCACCTAGTGGTCATG

1 CATGACCACTAGGAGCATCTTTGGCGAGCCTCGCTTGGGAGGTTGCTCCTCCAGTTCCTAGGGGAATAAATCTTTGGGCACCTAGTGGTCATG

1 CATGACCACTAGGAGCATCTTTGGCGAACCGGCAGCGCCGGGTGGAAATATGTTAGCCTAGGGGAATAAATCTTTGGGCACCTAGTGGTCATG

1 CATGACCACTAGGAGCATCTTTGGCGATCAGGGGGCCTTTGATGGCGACTCAGGGGGCTAGGGGAATAAATCTTTGGGCACCTAGTGGTCATG

1 CATGACCACTAGGAGCATCTTTGGCGAGATCAGGAGAATCGGTGGCATTGGTGTCACCTAGGGGAATAAATCTTTGGGCACCTAGTGGTCATG

1 CATGACCACTAGGAGCATCTTTGGCGAGCCTTTCTTGGGAGGTTGCTCCACCAGATCCTAGGGGAATAAATCTTTGGGCACCTAGTGGTCATG

1 CATGACCACTAGGAGCATCTTTGGCGATCCGGGGACACGCAGTGGGACGCACTAGGGCTAGGGGAATAAATCTTTGGGCACCTAGTGGTCATG

1 CATGACCACTAGGAGCATCTTTGGCGAACGGTAGGTGTTGCGGTGGTCTGTGAATTTCTAGGGGAATAAATCTTTGGGCACCTAGTGGTCATG

1 CATGACCACTAGGAGCATCTTTGGCGAACGGCAGGTGTTGCGGTGGTTTGTGAATCACTAGGGGAATAAATCTTTGGGCACCTAGTGGTCATG

1 CATGACCACTAGGAGCATCTTTGGCGAGATCGGGAGAGTCGGTGGCATTGGTGTATCCTAGGGGAATAAATCTTTGGGCACCTAGTGGTCATG

1 CATGACCACTAGGAGCATCTTTGGCGATGGCCACGGCCCTCAATCCGGGTGCTGCAGCTAGGGGAATAAATCTTTGGGCACCTAGTGGTCATG

1 CATGACCACTAGGAGCATCTTTGGCGAGAAGACTCCGGATTCGGGGGCCAGTTGCTGCTAGGGGAATAAATCTTTGGGCACCTAGTGGTCATG

1 CATGACCACTAGGAGCATCTTTGGCGACCAGTGCGAAGTATGCAAACGGAGGTGGAGCTAGGGGAATAAATCTTTGGGCACCTAGTGGTCATG

1 CATGACCACTAGGAGCATCTTTGGCGAGTGGGGGGCGACGGCCGGTAGAGGGTGAGACTAGGGGAATAAATCTTTGGGCACCTAGTGGTCATG

1 CATGACCACTAGGAGCATCTTTGGCGAATGCGAAGGGCTCTGGGACGTAATGAGCCCCTAGGGGAATAAATCTTTGGGCACCTAGTGGTCATG

1 CATGACCACTAGGAGCATCTTTGGCGAGGGTTCCAGAAAGTGGACGTGGTGTCAAGACTAGGGGAATAAATCTTTGGGCACCTAGTGGTCATG

1 CATGACCACTAGGAGCATCTTTGGCGACGGTGTGGGGAACCTGTTTCGGCGGTGCTCCTAGGGGAATAAATCTTTGGGCACCTAGTGGTCATG

1 CATGACCACTAGGAGCATCTTTGGCGAGATGGGCCGGAAGGATGGTATTTGTAGCCGCTAGGGGAATAAATCTTTGGGCACCTAGTGGTCATG

1 CATGACCACTAGGAGCATCTTTGGCGACGGCGTGCGGCCATCTGTGCGGCGGATCCCCTAGGGGAATAAATCTTTGGGCACCTAGTGGTCATG

1 CATGACCACTAGGAGCATCTTTGGCGACGTGGGGTCAGGCGATCAGAGGGAAGTCGACTAGGGGAATAAATCTTTGGGCACCTAGTGGTCATG

1 CATGACCACTAGGAGCATCTTTGGCGAACGGCAGGTGTTGCTGTGGTATGTGAATCCCTAGGGGAATAAATCTTTGGGCACCTAGTGGTCATG

1 CATGACCACTAGGAGCATCTTTGGCGAGTGGGGGGCGACGGCCGGTAGTGGGTTAGACTAGGGGAATAAATCTTTGGGCACCTAGTGGTCATG

1 CATGACCACTAGGAGCATCTTTGGCGAGATCATCAAGGGGGTGATACCATCTAGCGCCTAGGGGAATAAATCTTTGGGCACCTAGTGGTCATG

1 CATGACCACTAGGAGCATCTTTGGCGATGGTGGGTGGAACCATGCTAGCGTCGTCGACTAGGGGAATAAATCTTTGGGCACCTAGTGGTCATG

1 CATGACCACTAGGAGCATCTTTGGCGAAAGTCGGTAGAACAGGGTGGGCTGCTGTCCCTAGGGGAATAAATCTTTGGGCACCTAGTGGTCATG

1 CATGACCACTAGGAGCATCTTTGGCGAGACCAGCCGCTCGAACCCGGAATTCTTGCTCTAGGGGAATAAATCTTTGGGCACCTAGTGGTCATG

1 CATGACCACTAGGAGCATCTTTGGCGAATGGCCGGCACGGCCTTCTAGTCCTCCGTACTAGGGGAATAAATCTTTGGGCACCTAGTGGTCATG

1 CATGACCACTAGGAGCATCTTTGGCGAACGGCAGGAGTAGCGGTGGTCTGTGAATCCCTAGGGGAATAAATCTTTGGGCACCTAGTGGTCATG

1 CATGACCACTAGGAGCATCTTTGGCGAGGGTGTGGGGAAGTTATATCGGCGGATGCTCTAGGGGAATAAATCTTTGGGCACCTAGTGGTCATG

1 CATGACCACTAGGAGCATCTTTGGCGATCCGGGGGTCAAAAGTGACGGCGCATAGGGCTAGGGGAATAAATCTTTGGGCACCTAGTGGTCATG

1 CATGACCACTAGGAGCATCTTTGGCGAGTAAGATTGTCGTACGGATACTGCACCATGCTAGGGGAATAAATCTTTGGGCACCTAGTGGTCATG

1 CATGACCACTAGGAGCATCTTTGGCGAGATCGGGAGAATTGGTGGCATCGGTGTTTCCTAGGGGAATAAATCTTTGGGCACCTAGTGGTCATG

1 CATGACCACTAGGAGCATCTTTGGCGAGATCGTGAGAATCAGTGGCATTGGTGTCTCCTAGGGGAATAAATCTTTGGGCACCTAGTGGTCATG

1 CATGACCACTAGGAGCATCTTTGGCGAGGTGCACCGGCCGGGTGGGTACGTCGCGTCCTAGGGGAATAAATCTTTGGGCACCTAGTGGTCATG

1 CATGACCACTAGGAGCATCTTTGGCGACTAGCTGGAAAACCAGCGCGACATAGTGCTCTAGGGGAATAAATCTTTGGGCACCTAGTGGTCATG

1 CATGACCACTAGGAGCATCTTTGGCGATCCGGGGGTTACATATGAACGACGCTTGGGCTAGGGGAATAAATCTTTGGGCACCTAGTGGTCATG

1 CATGACCACTAGGAGCATCTTTGGCGAGGTCGGGAGAATCGGTGGTATTGGTGTCTCCTAGGGGAATAAATCTTTGGGCACCTAGTGGTCATG

1 CATGACCACTAGGAGCATCTTTGGCGACAGTGGGCAGTCGGCACACGTGGTGGAGCTCTAGGGGAATAAATCTTTGGGCACCTAGTGGTCATG

1 CATGACCACTAGGAGCATCTTTGGCGACAAGGCTCGATAGTAGTCCGCGACGTAGTGCTAGGGGAATAAATCTTTGGGCACCTAGTGGTCATG

1 CATGACCACTAGGAGCATCTTTGGCGATGGGGGGCGAGAGGACTGAGTAGGAATAGGCTAGGGGAATAAATCTTTGGGCACCTAGTGGTCATG

1 CATGACCACTAGGAGCATCTTTGGCGACACAGTGTAATCCATGTAGCTGTCGTGGAGCTAGGGGAATAAATCTTTGGGCACCTAGTGGTCATG

1 CATGACCACTAGGAGCATCTTTGGCGAACGGCAGGTGGTGCGGTGGTCCGTGAATCCCTAGGGGAATAAATCTTTGGGCACCTAGTGGTCATG

1 CATGACCACTAGGAGCATCTTTGGCGATCGATCCGACGATCGGGAGCAGACGTTTGGCTAGGGGAATAAATCTTTGGGCACCTAGTGGTCATG

1 CATGACCACTAGGAGCATCTTTGGCGACCGGGCTTCCGGGTAAGGTACTCTCCGACTCTAGGGGAATAAATCTTTGGGCACCTAGTGGTCATG

1 CATGACCACTAGGAGCATCTTTGGCGAGGATCTATGGTGTGCTGGCTTAGAGCTCAACTAGGGGAATAAATCTTTGGGCACCTAGTGGTCATG

1 CATGACCACTAGGAGCATCTTTGGCGAGATGGGCGAACGCAGTGTGAGGAAACCTCCCTAGGGGAATAAATCTTTGGGCACCTAGTGGTCATG

1 CATGACCACTAGGAGCATCTTTGGCGAGACCGGGAGAATCTGTGGCATTGGTGTCTCCTAGGGGAATAAATCTTTGGGCACCTAGTGGTCATG

1 CATGACCACTAGGAGCATCTTTGGCGATACGGAGGTCACAAGTGACGGCGCATAGGGCTAGGGGAATAAATCTTTGGGCACCTAGTGGTCATG

1 CATGACCACTAGGAGCATCTTTGGCGATAGCGTCTTGCGAGCGGGTGGGTTCGCTTCCTAGGGGAATAAATCTTTGGGCACCTAGTGGTCATG

1 CATGACCACTAGGAGCATCTTTGGCGATATCGGGAGAATCGGTGGCATAGGTGTCTACTAGGGGAATAAATCTTTGGGCACCTAGTGGTCATG

1 CATGACCACTAGGAGCATCTTTGGCGATATCAGGAGAATCGGTGGCATTGGTGTCTCCTAGGGGAATAAATCTTTGGGCACCTAGTGGTCATG

1 CATGACCACTAGGAGCATCTTTGGCGAATGGCCGGCACGGCCTTCTAGTCCTCGTTACTAGGGGAATAAATCTTTGGGCACCTAGTGGTCATG

1 CATGACCACTAGGAGCATCTTTGGCGAGGTCGGTAGAATCGGTGGCATTGGTGTCTCCTAGGGGAATAAATCTTTGGGCACCTAGTGGTCATG

1 CATGACCACTAGGAGCATCTTTGGCGAGGAGACTCTGAATTCGGGGACCAGTTGCTGCTAGGGGAATAAATCTTTGGGCACCTAGTGGTCATG

1 CATGACCACTAGGAGCATCTTTGGCGACTTACCGTATTACCTAGGTTAACTAACCCGCTAGGGGAATAAATCTTTGGGCACCTAGTGGTCATG

1 CATGACCACTAGGAGCATCTTTGGCGAGATCGGGAGAATCCGTGGCATTTGTGTCTCCTAGGGGAATAAATCTTTGGGCACCTAGTGGTCATG

1 CATGACCACTAGGAGCATCTTTGGCGACGTAGGTGGCCTCGTGGTAGAGCTACTAGACTAGGGGAATAAATCTTTGGGCACCTAGTGGTCATG

1 CATGACCACTAGGAGCATCTTTGGCGACCTCGATGTAGTCACTGACAATCTCCGCCACTAGGGGAATAAATCTTTGGGCACCTAGTGGTCATG

1 CATGACCACTAGGAGCATCTTTGGCGATTAGCATAGCGAATCTCTACGAAGATGGGCCTAGGGGAATAAATCTTTGGGCACCTAGTGGTCATG

1 CATGACCACTAGGAGCATCTTTGGCGAACGAGTGGGCAGCGCAAATACGTGGGTAGACTAGGGGAATAAATCTTTGGGCACCTAGTGGTCATG

1 CATGACCACTAGGAGCATCTTTGGCGAACGGCAAGAGATAACATTGCATTGGAACCTCTAGGGGAATAAATCTTTGGGCACCTAGTGGTCATG

1 CATGACCACTAGGAGCATCTTTGGCGAATTGTGATTCGTTTAGGTTGCACACAGGCGCTAGGGGAATAAATCTTTGGGCACCTAGTGGTCATG

1 CATGACCACTAGGAGCATCTTTGGCGACGAGAGTGGGTCATGGTGGAATAAAGTCGACTAGGGGAATAAATCTTTGGGCACCTAGTGGTCATG

1 CATGACCACTAGGAGCATCTTTGGCGACTCCTGGTGCAGTATAGCACAAGGTGTGGACTAGGGGAATAAATCTTTGGGCACCTAGTGGTCATG

1 CATGACCACTAGGAGCATCTTTGGCGAGATCGGGAGAATCTGTGGCATTGGTGTCTGCTAGGGGAATAAATCTTTGGGCACCTAGTGGTCATG

1 CATGACCACTAGGAGCATCTTTGGCGAGCCGATTGTGCAAAAAAGTGATCTGGGTAACTAGGGGAATAAATCTTTGGGCACCTAGTGGTCATG

1 CATGACCACTAGGAGCATCTTTGGCGAGATTGGGAGAATCGGTGGCATTGGTGTCACCTAGGGGAATAAATCTTTGGGCACCTAGTGGTCATG

1 CATGACCACTAGGAGCATCTTTGGCGAGATCGGGAGAATCGGTGGCATTGGTGACCCCTAGGGGAATAAATCTTTGGGCACCTAGTGGTCATG

1 CATGACCACTAGGAGCATCTTTGGCGAGATCGGTAGAATCGGTGGCATTGGTGCCTCCTAGGGGAATAAATCTTTGGGCACCTAGTGGTCATG

1 CATGACCACTAGGAGCATCTTTGGCGAGATCGGAAGAAACGGTGGCATAGGTGTCTCCTAGGGGAATAAATCTTTGGGCACCTAGTGGTCATG

1 CATGACCACTAGGAGCATCTTTGGCGACACCGTGTAACGTCGTGGACAGGGCTGCGCCTAGGGGAATAAATCTTTGGGCACCTAGTGGTCATG

1 CATGACCACTAGGAGCATCTTTGGCGACCGGCAGGTGTTGCGGTGGTCTGTGAAAACCTAGGGGAATAAATCTTTGGGCACCTAGTGGTCATG

1 CATGACCACTAGGAGCATCTTTGGCGAGTATTGGCTGGTAGATTGCGTATTGGGGAGCTAGGGGAATAAATCTTTGGGCACCTAGTGGTCATG

1 CATGACCACTAGGAGCATCTTTGGCGAGATCGGGAGAATAGGTGGCACTGGTGTCTCCTAGGGGAATAAATCTTTGGGCACCTAGTGGTCATG

1 CATGACCACTAGGAGCATCTTTGGCGACTCCATGGCGAGCGTGGATCGGGTCCCCCACTAGGGGAATAAATCTTTGGGCACCTAGTGGTCATG

1 CATGACCACTAGGAGCATCTTTGGCGACATAAATTCTGGGGGAATATAGCTCATTCCCTAGGGGAATAAATCTTTGGGCACCTAGTGGTCATG

1 CATGACCACTAGGAGCATCTTTGGCGACTAGCGCTGATCAGATGTCAACGCGTTCATCTAGGGGAATAAATCTTTGGGCACCTAGTGGTCATG

1 CATGACCACTAGGAGCATCTTTGGCGAGATCTGGAGACTCGGTGGCATTGGTGTTTCCTAGGGGAATAAATCTTTGGGCACCTAGTGGTCATG

1 CATGACCACTAGGAGCATCTTTGGCGAGGGGTAGGGGGCCGCACGCGCCTGCGTGCGCTAGGGGAATAAATCTTTGGGCACCTAGTGGTCATG

1 CATGACCACTAGGAGCATCTTTGGCGAGATCGGGAGAATTGGTGGCATTGGTGTGTCCTAGGGGAATAAATCTTTGGGCACCTAGTGGTCATG

1 CATGACCACTAGGAGCATCTTTGGCGATGTATACGGAGCAGTGTATTCTACCGTTTCCTAGGGGAATAAATCTTTGGGCACCTAGTGGTCATG

1 CATGACCACTAGGAGCATCTTTGGCGAGCTCCGGAGGATCGGTGGCAGTGGTGAAGCCTAGGGGAATAAATCTTTGGGCACCTAGTGGTCATG

1 CATGACCACTAGGAGCATCTTTGGCGACTCATTTTGAACGTTGCAAAATGTAGTGGACTAGGGGAATAAATCTTTGGGCACCTAGTGGTCATG

1 CATGACCACTAGGAGCATCTTTGGCGACCGGCAGGTGTTGCGGAGGTCTGTGAATCCCTAGGGGAATAAATCTTTGGGCACCTAGTGGTCATG

1 CATGACCACTAGGAGCATCTTTGGCGAACGGCAGGTGTCGCGGCGGTCTGTGAATCCCTAGGGGAATAAATCTTTGGGCACCTAGTGGTCATG

1 CATGACCACTAGGAGCATCTTTGGCGAGATCGGGAGAATCGGTGGCATTGCTGCCTCCTAGGGGAATAAATCTTTGGGCACCTAGTGGTCATG

1 CATGACCACTAGGAGCATCTTTGGCGACTAGAGACCCTGTCACGACACTGCCGGACCCTAGGGGAATAAATCTTTGGGCACCTAGTGGTCATG

1 CATGACCACTAGGAGCATCTTTGGCGACTCCCAGTCATTTTATTTGAGGCCCTATAACTAGGGGAATAAATCTTTGGGCACCTAGTGGTCATG

1 CATGACCACTAGGAGCATCTTTGGCGAACGCAGATGGGCCGTGGTTGAGCGTGCAGACTAGGGGAATAAATCTTTGGGCACCTAGTGGTCATG

1 CATGACCACTAGGAGCATCTTTGGCGAACGGCAGGTGCTGCGGTGGGCTGTGAATCCCTAGGGGAATAAATCTTTGGGCACCTAGTGGTCATG

1 CATGACCACTAGGAGCATCTTTGGCGAACGGTAGGTGTTGCTGTGGTATTTGAATCCCTAGGGGAATAAATCTTTGGGCACCTAGTGGTCATG

1 CATGACCACTAGGAGCATCTTTGGCGACGCGGTGTGTGGATAGGGTATGGTACGCGACTAGGGGAATAAATCTTTGGGCACCTAGTGGTCATG
[truncated: 224,166 more chars]
