# Supplementary material for: Selection of allosteric dnazymes that can sense phenylalanine by expression-SELEX
Source: Nucleic Acids Res. 2023 May 19;51(11):e66. doi: 10.1093/nar/gkad424 (PMC10287898; doi:10.1093/nar/gkad424)
Supplement: gkad424_Supplemental_Files [file gkad424_supplemental_files.zip › Supplementary file No. 5 top1000-enriched-sequences-30nt-random_for_round20.docx]

Random region of the round_20^th^ library

>1_892954

GATCGGGAGAATCGGTGGCATTGGTGTCTC

>2_122302

ACGGCAGGTGTTGCGGTGGTCTGTGAATCC

>3_57642

GAAGACTCTGGATTCGGGGACCAGTTGCTG

>4_10428

AAGTCGGTAGAACAGGGTGGGGTGCTGTCC

>5_7863

GATCGGGAGAATCGGCGGCATTGGTGTCTC

>6_4354

GATTGGGAGAATCGGTGGCATTGGTGTCTC

>7_3906

GATCGGGAGAATCGGTGGCATTGGTGTCTT

>8_3479

GCCTTGCTTGGGAGGTTGCTCCACCAGTTC

>9_3467

ATGGCCGGCACGGCCTTCTAGTCCTCGGTA

>10_3398

GCCTCGCTTGGGAGGTTGCTCCACCAGTTC

>11_3283

GATCGGGAGAATTGGTGGCATTGGTGTCTC

>12_2781

GATCGTGAGAATCGGTGGCATTGGTGTCTC

>13_2608

GGGAGGGCGCCGGCAGCGGTGTGAATGCGA

>14_2397

GATCGGGAGAATCGGTGGCATTGGTGTTTC

>15_2111

GATCGGGAGAATCGGTGGCATCGGTGTCTC

>16_2015

TCCGGGGGCACCTATGTGCGACGCTGTGGG

>17_1565

GATCGGGGGAATCGGTGGCATTGGTGTCTC

>18_1555

GATCAGGAGAATCGGTGGCATTGGTGTCTC

>19_1547

GAAGACTCTGGATTCGGGGACCAGTTGCTC

>20_1482

GATCGGGAGGATCGGTGGCATTGGTGTCTC

>21_1467

ACGGCAGGTGTTGCGGTGGTTTGTGAATCC

>22_1449

GATCGAGAGAATCGGTGGCATTGGTGTCTC

>23_1394

GTGGGGGGCGACGGCCGGTAGTGGGTGAGA

>24_1332

GATCGGGAGAATCGGTGGCATTGGTGCCTC

>25_1310

ACGGCAGGTGTTGCGGTGGTCTGTGGATCC

>26_1257

GATCGGGAGAATCGGTGGCATTGGCGTCTC

>27_1204

GATCGGGAGAATCGGTGGCATTGGTGTCCC

>28_1108

TGGGTGCTGACGGCCGCCGCTGCGGCTACA

>29_1104

GATCGGGAGAGTCGGTGGCATTGGTGTCTC

>30_1081

GATCGGGAGAATCGGTGGTATTGGTGTCTC

>31_1060

GGGAGGGCGCCGGCAGCGGTGTGAATGCGC

>32_1013

GATCGGAAGAATCGGTGGCATTGGTGTCTC

>33_1011

GATCGGGAGAATCGGTGGCGTTGGTGTCTC

>34_963

GATCGCGAGAATCGGTGGCATTGGTGTCTC

>35_938

GATCGGTAGAATCGGTGGCATTGGTGTCTC

>36_935

AGGGTGTAGGACTTCAAGTGGATCTCATAG

>37_907

GAAGACTTTGGATTCGGGGACCAGTTGCTG

>38_754

ACGGCAGGTGTTGCGGTGGTCTGTGAATCT

>39_748

GATCGGGAGAATCGGTGGCATTGGTGTCTA

>40_706

GATCTGGAGAATCGGTGGCATTGGTGTCTC

>41_683

GGTCGGGAGAATCGGTGGCATTGGTGTCTC

>42_658

TATCGGGAGAATCGGTGGCATTGGTGTCTC

>43_657

GATCGGGAGAATCGGTGGCATTGGTGTCTG

>44_649

CAATGGAGCGACGGTGTGGTCTGAGTCCTA

>45_629

GATCGGGAGAATCGGTGGCACTGGTGTCTC

>46_618

GATCGGCAGAATCGGTGGCATTGGTGTCTC

>47_587

GCGGCAGGTGTTGCGGTGGTCTGTGAATCC

>48_566

CGGTGTGGGGAACTTGTTTCGGCGGTGCTA

>49_559

GATCGGGAGAATCGTTGGCATTGGTGTCTC

>50_552

GATCGGGAGAATCGGAGGCATTGGTGTCTC

>51_542

ACGGCAGGTGTTGCGGTGGTCTGTGTATCC

>52_534

GATCGGGAGAATCGGTGGCATAGGTGTCTC

>53_527

GATCGGGAGAATCTGTGGCATTGGTGTCTC

>54_526

CCAGATATGGTAGGTAATGTGCTGAGGAGG

>55_526

GATCGGGAGAATCAGTGGCATTGGTGTCTC

>56_520

GAAGACTCCGGATTCGGGGACCAGTTGCTG

>57_518

GACCGGGAGAATCGGTGGCATTGGTGTCTC

>58_497

ATGGCAGGTGTTGCGGTGGTCTGTGAATCC

>59_481

GATCGGGAGAATCGGTGTCATTGGTGTCTC

>60_479

ACGGCAGGTGTTGCGGTGGTCTGTGAAACC

>61_454

GATCGGGAGAATCGGTGGCATTAGTGTCTC

>62_452

GATCGGGAGAATCGGTGGCATTGATGTCTC

>63_447

GATCGGGAGAATCGGTAGCATTGGTGTCTC

>64_440

ACGGCAGGTGTTGCGGTGGTCTATGAATCC

>65_438

ACGGCAGGTGTTGCGGTGGTCTGTGAATTC

>66_435

GGAGTGGCTGAGGGCGGTGGGTAGGTCGCG

>67_430

GATCGGGAGAAACGGTGGCATTGGTGTCTC

>68_428

CAGGGGATGCCGATGCGCTGGACGACCGAG

>69_427

GATCGGGAGAATCGGTGGCATTGTTGTCTC

>70_417

ATGGCCGGCACGGCCTTCTAGTCCTTGGTA

>71_414

TGGGGCCGTGCTATCTGCACACTCCGCGGG

>72_409

AATCGGGAGAATCGGTGGCATTGGTGTCTC

>73_396

GATCGGGAGACTCGGTGGCATTGGTGTCTC

>74_386

TCCTCGTCAGATGGTGAAGCAGACGTTTGG

>75_386

GATCGGGATAATCGGTGGCATTGGTGTCTC

>76_383

GGGGTCATGGGAATGTAATTGCGCTTAAGA

>77_378

GATCGGGAGAATCGGTGACATTGGTGTCTC

>78_370

GATCGGGAGAATCGGTGGCATTTGTGTCTC

>79_369

GATAGGGAGAATCGGTGGCATTGGTGTCTC

>80_366

ACGGCAGGTGTTGCGGTGGTCTGTGAACCC

>81_364

GATCGGGAGAATCGGTGGCAGTGGTGTCTC

>82_358

GATCGGGAGAATCGGTGGCATTGGTGTCAC

>83_354

GAAGACTCTGGATTCGGGGACTAGTTGCTG

>84_354

GATCGGGAGAACCGGTGGCATTGGTGTCTC

>85_352

GTATTGGCTGGTAGGTTGCGTATTGGGGAG

>86_347

CGGCGTGGGGCTATTGATGCGGCGGCACTC

>87_343

GATCGGGAGAATCGGTGGCATTGGAGTCTC

>88_334

AGCGCAGGGTGTGAGAGGGGCGTGTCCATG

>89_330

GATCGGGAGAATCGGTGGCATTGGTGACTC

>90_327

GATCGGGAGAATCGGTGGAATTGGTGTCTC

>91_314

CGGCGTGGGGCCATCTGTGCGGCGGATCCC

>92_310

GATCGGGAGAATCGGTGGCATTGGTTTCTC

>93_307

GATCGGGAGAATAGGTGGCATTGGTGTCTC

>94_296

GATCGGGAGAATCGATGGCATTGGTGTCTC

>95_285

GATCGGGAGAATCGGTGGCATTGGTATCTC

>96_283

GATCGGGAGAATCGGTTGCATTGGTGTCTC

>97_272

GATCGGGAGAATCGCTGGCATTGGTGTCTC

>98_270

CCGTAGGAAAACAAAAGTGGCCTTGCTAGC

>99_259

GATCGGGAGAATCGGTGGCAATGGTGTCTC

>100_254

GAAGACCCTGGATTCGGGGACCAGTTGCTG

>101_252

GAAGACTCTGTATTCGGGGACCAGTTGCTG

>102_249

CCGAGTGGGCTGGGAGGAAGTATTTCGGAG

>103_234

TCAGGGGGCCTTTGATGGCGACTCAGTGGG

>104_230

ACGGTAGGTGTTGCGGTGGTCTGTGAATCC

>105_226

GATCGGGAAAATCGGTGGCATTGGTGTCTC

>106_224

ACGTGCAAGATATGCCGCCTGGAGCGTAGG

>107_222

ACGGCAGGTGTTGCGGTGGTCCGTGAATCC

>108_222

GATCGGGAGTATCGGTGGCATTGGTGTCTC

>109_220

GAAGACTCTGGATTTGGGGACCAGTTGCTG

>110_216

GAGAATCGGTAGGCAGAGTGCTCCAGCCAG

>111_213

GATCGGGAGAATCGGTGCCATTGGTGTCTC

>112_210

ACGGCGGGTGTTGCGGTGGTCTGTGAATCC

>113_208

GATCGGGAGAATCGGTGGCCTTGGTGTCTC

>114_196

TCCGGGGGCATGCTATGCGACGCTTATGGG

>115_187

TCCGGGGGTCACAAGTGACGGCGCATAGGG

>116_185

GAAGACTCTGGATTCGGGGACCAGTTGTTG

>117_179

GATCCGGAGAATCGGTGGCATTGGTGTCTC

>118_178

AGCGTGTTGCAAGGTCCGCGAGTGTGGACC

>119_177

GAACGGGAGAATCGGTGGCATTGGTGTCTC

>120_170

GAAGACTCAGGATTCGGGGACCAGTTGCTG

>121_170

ACGGCAGGGGTTGCGGTGGTCTGTGAATCC

>122_170

GGGGTAGGGGGCCGCTCGCGCCTGCGTGCG

>123_169

GATCGGGAGAATCGGTGGCATTGCTGTCTC

>124_168

GATCGGGAGAATCGGTGGCATTGGTGTATC

>125_167

ACGGCAGGTGTTGCGGTGGTCTGTGAATCA

>126_163

TCAGGCCGCCAGGGCGTACGAAGTGTGGTT

>127_161

TGGGTGCTGACGGGGCTAGTGAGCCCTACA

>128_161

TAGGCGTGTACGGTCGAGCGAAAGCGATAG

>129_157

ACGGCAGGTGTTGCGGTGGCCTGTGAATCC

>130_154

GCCGATTGTGCAACAATGTGTTCTGGGTAA

>131_154

TAGCGAAGTGCTGTGAACAGTGGTTAGCAC

>132_147

GATCGGGTGAATCGGTGGCATTGGTGTCTC

>133_145

ACGGCAGGTGTTGTGGTGGTCTGTGAATCC

>134_140

CCGGCAGGTGTTGCGGTGGTCTGTGAATCC

>135_140

ACGGCAGGTGTTGCGGTGGTCTGCGAATCC

>136_139

ACGGCAGGTGCTGCGGTGGTCTGTGAATCC

>137_138

TAGTCGGTAGAACAGGGTGGGGTGCTGTCC

>138_133

GATCGGGCGAATCGGTGGCATTGGTGTCTC

>139_132

GATCGGGAGAATCGGTGGGATTGGTGTCTC

>140_131

AGTGCGGTGGGCATGTGGGTGGTAGCGACG

>141_127

GAAGATTCTGGATTCGGGGACCAGTTGCTG

>142_125

GAAGACTCTGGATTCGGGGATCAGTTGCTG

>143_120

GAGCGGGAGAATCGGTGGCATTGGTGTCTC

>144_117

GGCGAGTCCAGCGGCCTTCTTGGCCTGCGA

>145_115

ACACGAGAGTCAAGTGCGTATAGCGTGGAG

>146_114

ACGGCAGGTGTTGCGGTGGTCTTTGAATCC

>147_114

GATCGGGAGAATCGGTGGCATTCGTGTCTC

>148_111

GATCGGGACAATCGGTGGCATTGGTGTCTC

>149_111

ACGGCAGGCGTTGCGGTGGTCTGTGAATCC

>150_107

TCGCACAGCGCACGAAGCCATATGAGGGGA

>151_107

TCCGGGGGCCACTGAGGCGACGCTAATGGG

>152_106

ACGGCAGGTGTTGCGGCGGTCTGTGAATCC

>153_105

GATCGGGAGAATCGGTGGCATGGGTGTCTC

>154_103

ACGGCAGGTGTTGCGGTGGTCTGTGAGTCC

>155_102

GATCGGGAGAATCGGTGGCATTGGTCTCTC

>156_102

GAAGACTCTGGATTCGGGGGCCAGTTGCTG

>157_100

CTCCAGTTCATCTGTCGGGCAGTTTAAGGA

>158_100

CGGTGTGGGGCCATCTGTGCGGCGGATCCC

>159_99

CGTAGTGGGCTGGCAGGGAGTACTTGTTGC

>160_98

GATCGGGAGAATCGGTGGCATTGGGGTCTC

>161_96

ACGGCAGGTGTTGCGGTGGTCTGTGAATCG

>162_92

GTTCGGGAGAATCGGTGGCATTGGTGTCTC

>163_91

GAATACTCTGGATTCGGGGACCAGTTGCTG

>164_90

CGGCGTGGGGCCATTTGTGCGGCGGATCCC

>165_88

GAAGACTCTGGGTTCGGGGACCAGTTGCTG

>166_88

ACGGCAGGTGTCGCGGTGGTCTGTGAATCC

>167_85

AAGTCGGTAGAACAGGGTGGGGTGCTGTCT

>168_85

ACGGCAGGTGTTGCTGTGGTCTGTGAATCC

>169_84

ACGGCAGGTGTTGCGGTGGTATGTGAATCC

>170_84

TTTGGGAGTCGCAGTATGCAGGCAGTGAGA

>171_83

GATCGGGAGAATCGGTGGCATTGGTGGCTC

>172_83

GATCGGGAGAATCGGGGGCATTGGTGTCTC

>173_82

CTAAGTGGTCAACGCGTTAAGTGGGGTAGA

>174_81

AGCAGTGCGACGGGCTATGGGTTCCTCGAG

>175_80

AGACGACACAGTCGATCGGGTTAGAGGAAG

>176_79

ATGGCCGGCACGGCCTCCTAGTCCTCGGTA

>177_79

ACGGCAGGTGTTGCGGTGGTCTCTGAATCC

>178_77

GATGGGGAGAATCGGTGGCATTGGTGTCTC

>179_77

GATCGGGAGAATCGGTGGCTTTGGTGTCTC

>180_76

GAAGGCTCTGGATTCGGGGACCAGTTGCTG

>181_76

ACGGCAGGTGTTGCAGTGGTCTGTGAATCC

>182_75

GATCGGGAGAATCGGTCGCATTGGTGTCTC

>183_75

GAAGACTCTGGATTCGGGGACCAGTTGCTA

>184_74

ACGGCATGTGTTGCGGTGGTCTGTGAATCC

>185_74

CGTCGTAAAGTGCGTAGAGTGGTAAGTCGA

>186_71

AGACGACACAGTCGATCGGGATAGAGGAAG

>187_71

GAAGACTCTGGATTCGGGGACCGGTTGCTG

>188_70

GATCGGGAGAATCCGTGGCATTGGTGTCTC

>189_70

CGTCGGCTGGTAGAGACTGTATTGAGGAAC

>190_69

CGACTTCGGAGTGGGTGCGCCGCTGGCATA

>191_69

ACGGCAGGTGTTGCGGTGGTCTGTGCATCC

>192_67

CATCGGGAGAATCGGTGGCATTGGTGTCTC

>193_66

GCTCGGGAGAATCGGTGGCATTGGTGTCTC

>194_65

GAAGACTCTGGACTCGGGGACCAGTTGCTG

>195_65

ACGGAAGGTGTTGCGGTGGTCTGTGAATCC

>196_61

ACGGCTGGTGTTGCGGTGGTCTGTGAATCC

>197_61

ACGGCAGTTGTTGCGGTGGTCTGTGAATCC

>198_60

ACGGCAGGTGTTTCGGTGGTCTGTGAATCC

>199_60

ACTGCAGGTGTTGCGGTGGTCTGTGAATCC

>200_60

ACGGCAGGTGTTGCGGTGGTCTGTAAATCC

>201_59

TCAAGTGCGCTTGGTAATTCCTGATGCGAT

>202_58

CGAGTGGGCACGGTACCACGCGACATAGTG

>203_58

ACGGCAAGTGTTGCGGTGGTCTGTGAATCC

>204_57

ACAGCAGGTGTTGCGGTGGTCTGTGAATCC

>205_57

ACGTCAGGTGTTGCGGTGGTCTGTGAATCC

>206_56

ACGTGCAAGAAATGCCGCCTGGAGCGTAGG

>207_56

CGAGATTTGGTAGGTAGATGTGCTCTAGCA

>208_56

GAAGACTCTGGATTCGGGGACCAGCTGCTG

>209_55

AAGTCGGTAGAACAGGGTGGGGTGCTGTTC

>210_55

ACGGCAGGTGTTGAGGTGGTCTGTGAATCC

>211_55

AAGTCGATAGAACAGGGTGGGGTGCTGTCC

>212_55

GAAGACTCTGGATTCGGGTACCAGTTGCTG

>213_55

ACGGCAGGAGTTGCGGTGGTCTGTGAATCC

>214_53

ACGGCAGGTGTTACGGTGGTCTGTGAATCC

>215_52

TCCGGGGGTCACATCAGGACGACGCTAGGG

>216_52

GAAGACTATGGATTCGGGGACCAGTTGCTG

>217_52

GAAGACTCTGGATTCGGGGACCAGTCGCTG

>218_51

ACGGCAGGTGTTGCGGTGTTCTGTGAATCC

>219_51

ACATCGTGGTCGGAAGGCGTCGGCGGAGTG

>220_51

ACGGCAGGTGTTGCGGTGGTCAGTGAATCC

>221_51

GGGGGGGCGCCGGCAGCGGTGTGAATGCGC

>222_50

ACGGCAGGTGTTGCGGTGGTCTGAGAATCC

>223_49

ACGGCAGGTGTTGCGTTGGTCTGTGAATCC

>224_49

GAGGACTCTGGATTCGGGGACCAGTTGCTG

>225_48

GATTGGGAGAATCGGCGGCATTGGTGTCTC

>226_48

TCGGCAGGTGTTGCGGTGGTCTGTGAATCC

>227_47

GGGAGTGGTCGGCCATGTGGGGGGTCGGGG

>228_47

GATCGGGAGCATCGGTGGCATTGGTGTCTC

>229_47

AAGGCAGGTGTTGCGGTGGTCTGTGAATCC

>230_47

ACGGCAGATGTTGCGGTGGTCTGTGAATCC

>231_47

TCCGGGGGTCAGATAAGACGGCGCGTTGGG

>232_47

ACGGCAGGTGTTGCGGAGGTCTGTGAATCC

>233_47

ACGGCAGGTGTTGCGGTGGTCGGTGAATCC

>234_46

GGGTGGGCGCCGGCAGCGGTGTGAATGCGC

>235_46

GTGGGGGGCGCCGGCCGGTAGTGGGTGAGA

>236_44

CGGTGTGGGGAACTTGTTTCGGCGGTGCTC

>237_44

GAAGACTCTGGATTCGGGGATTAGTTGCTG

>238_43

AAGTTGGTAGAACAGGGTGGGGTGCTGTCC

>239_43

GGGGGGGCGCCGGCAGCGGTGTGAATGCGA

>240_42

GGAGACTCTGGATTCGGGGACCAGTTGCTG

>241_42

GAAGACTCTGGATTCAGGGACCAGTTGCTG

>242_42

GAAGACTCTGAATTCGGGGACCAGTTGCTG

>243_41

ACGGCAGGTGTTGCGGTGGTCTGTTAATCC

>244_41

GATCGGGAGAATGGGTGGCATTGGTGTCTC

>245_40

GATCGGGAGAATCGGTGGCATTGGTGTGTC

>246_40

GAAGACACTGGATTCGGGGACCAGTTGCTG

>247_40

GAAGACTCTGGATTCGGGGACCAGTTGCCG

>248_39

CGAGGGGCACGGGTGGTCATTGCGAGAAGA

>249_39

CGGTGTGGGGACCGCAGGTTCGGCGGGTAC

>250_39

ACGGCAGGTGTTGCGATGGTCTGTGAATCC

>251_39

GAAGACTCTGGATTCGTGGACCAGTTGCTG

>252_38

ACGGCAGGTGTTGCGGTTGTCTGTGAATCC

>253_38

ACGGCAGGTGTTGCGGTGGACTGTGAATCC

>254_37

GAAGACGCTGGATTCGGGGACCAGTTGCTG

>255_37

ACGGCAGGTGTTGCGGTGGTCTGTGAATAC

>256_37

GAAGACTTTGGATTCGGGGACCAGTTGCTC

>257_37

GAAGACTCTGGATTCGGGGACCAGTTGCTT

>258_36

ACGGCAGGTGTTGCGGGGGTCTGTGAATCC

>259_36

CGAGTGGGCGGAGCTTAACGCGGCATAGTG

>260_36

ACGGCAGGTATTGCGGTGGTCTGTGAATCC

>261_36

ACGACAGGTGTTGCGGTGGTCTGTGAATCC

>262_36

GAAGACTCTGGATTCGGGGACCAGATGCTG

>263_36

ACGGCAGGTGTTGCGGTAGTCTGTGAATCC

>264_35

ACGGCAGGTGATGCGGTGGTCTGTGAATCC

>265_35

AAGTCGTTAGAACAGGGTGGGGTGCTGTCC

>266_35

CCGTAGGAAAACAAAAGTGCCCTTGCTAGC

>267_35

AAGTCGGCAGAACAGGGTGGGGTGCTGTCC

>268_35

GATCGAAAGAATCGGTGGCATTGGTGTCTC

>269_35

CGGAGTGGGGCTCATTGTGCGGCGGTAATC

>270_34

CGGCGTGGGGCTATAGATGCGGCGGCACTC

>271_34

TCCGGGGGGCTGTGCAGCCGACGCAAAGGG

>272_34

GATCAGGAGAATCGGCGGCATTGGTGTCTC

>273_34

GAAGACTCTAGATTCGGGGACCAGTTGCTG

>274_34

GAAAACTCTGGATTCGGGGACCAGTTGCTG

>275_34

ACGGCCGGCACGGCCTTCTAGTCCTCGGTA

>276_34

TGGGTTGCTGGTAGGTGGTACGACCTCGAT

>277_34

GCCTTGCTTGGGAGGTTGCTCTACCAGTTC

>278_34

GGGGCCTAAGTGCGTATCTGTGGTCGAGCT

>279_34

GAAGACTGTGGATTCGGGGACCAGTTGCTG

>280_33

GAGTCGGTAGAACAGGGTGGGGTGCTGTCC

>281_33

TCCGGGGGGACTGGGTCCGACGCAATAGGG

>282_33

GAAGACTCTGGATTCGGTGACCAGTTGCTG

>283_33

GAAGACTCTGGATTCTGGGACCAGTTGCTG

>284_33

ACGGCAGGTGTAGCGGTGGTCTGTGAATCC

>285_32

AAGTCGGTAGAACAGGGTGGGGTGTTGTCC

>286_32

CGGCGTGGGGCTATTGATGCGGCGGTACTC

>287_32

CTGTGGTCTCGTCCTAATTGTGCGGGCGTC

>288_32

GAAGACTCTGGAGTCGGGGACCAGTTGCTG

>289_32

CAGCCGAAGGGACCAGAAGGGAGTCACTGT

>290_32

ACGTCGGTAGAACAGGGTGGGGTGCTGTCC

>291_32

GATCGTGAGAATCGGCGGCATTGGTGTCTC

>292_32

GTGTGGGCTGGCAGTGCGTACTTGATCGCT

>293_31

GAAGACTCTGGATTCGGGGACCAGTAGCTG

>294_31

GCGACAAATGGAAATGCGACGTAGTGGACG

>295_31

TCAGGCCGCCAGGGCGTACGAAGCGTGGTT

>296_31

GAAGACTCTGGATCCGGGGACCAGTTGCTG

>297_30

GATCGGGAGAATCGGCGGCATTGGTGTCTT

>298_30

GGTTGTAGCTCGCGCGTAGGGTCGGCAGTG

>299_30

TAAGACTCTGGATTCGGGGACCAGTTGCTG

>300_29

TTGGTAGTCGCGGATGCAGGCAGTGATGCT

>301_29

GAAGACTCTGGATTCGAGGACCAGTTGCTG

>302_29

CTAAGTGGCCAACGCGTTAAATGGGGTAGA

>303_29

TCCGGGGGCCAAAGGCGACGCAAATTAGGG

>304_29

GCCTTGCTTGGGAGGTTGCTCCACTAGTTC

>305_29

GAAGACTCTGGATTCGGGGACCAGTTTCTG

>306_28

TTGAAGCCGGTGCGGTGCGGCGAGATGCGA

>307_28

ACGGCAGGTGGTGCGGTGGTCTGTGAATCC

>308_28

TCCGGGGGGCAGAGAGGCCGACGCACTGGG

>309_28

GATCGGGAGAATTGGCGGCATTGGTGTCTC

>310_28

ACGGCAGGTGTTGCGGTGGTCTGTGAATTT

>311_28

GAAGACTCTGGATTCGGGGACAAGTTGCTG

>312_28

GATCGGGAGAAGCGGTGGCATTGGTGTCTC

>313_27

CTCGTGGTCCTTACCCCCGTAGAGGGTTGT

>314_27

GAAGACTCTGGATTCGGGGACCAGTTGATG

>315_27

ACGGCAGGTTTTGCGGTGGTCTGTGAATCC

>316_27

GGGATGGGAGCCGATGGGGGTTCGGGGTTT

>317_27

GAAGACTCTGGATTCGGGGACCATTTGCTG

>318_27

CGTGGGCTGGAAGGATGTGGTACTTCTCGA

>319_27

TTTGGGAGTCGCAGTATGCAGGCAGTGAGG

>320_26

GTTGTCGTAGAGCCACGGGACACCCGTCAG

>321_26

AGTGAAGAGTGCGTGTAGTGGTCTGTTCGC

>322_26

CGGCGAAAGTTCGGGCTGGGGAGTGTTGCT

>323_26

GATCGGGAGAATCGGCGGCATTGGTGTTTC

>324_26

GAAGACTCTGGATTCGGGAACCAGTTGCTG

>325_25

GCTTTGCTTGGGAGGTTGCTCCACCAGTTC

>326_25

TGGGTGCAGACGAGTGCGCTTGCAATTACA

>327_25

GAAGACTCTGGATTCGGAGACCAGTTGCTG

>328_25

ATGGCCGGCACGGCCTTCTAGTCTTCGGTA

>329_25

GGGGGCGAGTGGGCGAGGTGTTATAGGGAG

>330_25

CAGCCGTAGCCGTAAGGTTGGTATAAGGCC

>331_24

GATCGGGAGAATCGGTGGCATTGGTGTCGC

>332_24

GCCTCGCTTGGGAGGTTGCTTCACCAGTTC

>333_24

GATCAAGAGAATCGGTGGCATTGGTGTCTC

>334_24

TCGAGTGGGCTGGGAGGAAGTATTTCGGAG

>335_24

CGATGGTTGGGAGGGATGTATTTCGGGCGA

>336_24

CATAGTGGATCGCAGTCCCGAAAAGGACGC

>337_23

GAAGAATCTGGATTCGGGGACCAGTTGCTG

>338_23

TCCGGGGACACGCAGTGGGACGCACTTGGG

>339_23

ACGGCAGGTGTTGCGGTGGGCTGTGAATCC

>340_23

GCACTACGTAGCGGTAGGCATTGTTGTCTC

>341_23

GATTGGGAGAATCGGTGGCATTGGTGTCTT

>342_23

AGGGCAGGTGTTGCGGTGGTCTGTGAATCC

>343_22

TGGGGGGCGAGAGGACTGAGTAGGAATAAG

>344_22

TCCGGGGGTCCGGGTGGACGACGCGACGGG

>345_22

CAGCGGCGTAGGCTGCCTTGGGTGGTGGCC

>346_22

AGTGTGGAACATGCCGGTTTTGGTTGCACC

>347_22

AAAGACTCTGGATTCGGGGACCAGTTGCTG

>348_22

ACGGCAGGTGTTGGGGTGGTCTGTGAATCC

>349_21

ATGGCCGGCACGGCTTTCTAGTCCTCGGTA

>350_21

GATCGGGGGAATCGGCGGCATTGGTGTCTC

>351_21

GAGCTTGAAAAAGGTGCGTTGTTGTCGCAT

>352_21

GAAGACTCTGGATTCGGGGACCAATTGCTG

>353_21

ACGGCAGGTGTTGCGGTGATCTGTGAATCC

>354_21

GGGTGGGCGCCGGCAGCGGTGTGAATGCGA

>355_21

GAAGACTCTGGATTCGGGGACCAGTGTCTC

>356_21

AAGTCGGTAGAACAGGGTGGGGCGCTGTCC

>357_21

GATCGGGAGAATCGGCGGCATCGGTGTCTC

>358_20

ATGGCCGGCACGGCCTTTTAGTCCTCGGTA

>359_20

ACGCCAGGTGTTGCGGTGGTCTGTGAATCC

>360_20

AAGTCGGTAGAACAGGGTGGGGTGCTGCCC

>361_20

ACGGCACGTGTTGCGGTGGTCTGTGAATCC

>362_20

GCCTTGCTTGGGAGGTTGCTTCACCAGTTC

>363_20

ACGGTAACGTAGTAACGTCGAAGTAGGCGC

>364_20

CGATTGGTCGGAAGGTAAGTGTTCGGATGA

>365_19

GAAGACTCTGGAATCGGGGACCAGTTGCTG

>366_19

ATGGCTGGCACGGCCTTCTAGTCCTCGGTA

>367_19

GATCGGGAGAGTCGGCGGCATTGGTGTCTC

>368_19

CTAAGTGGCCAGCGCGTTAAATGGGGTAGA

>369_19

GATCGGGAGAATCGGCGGTATTGGTGTCTC

>370_18

GAAGACTCCGGATTCGGGGACCAGTTGCTC

>371_18

AGCGTGTTGCGAGGTCCGCGAGTGTGGACC

>372_18

GAAGACTCTTGATTCGGGGACCAGTTGCTG

>373_18

CGTGGGCTGGGAGGATGTGGTACTTCTCGA

>374_18

ACGGCAGGTGTTGCGGTGGTCTGTGACTCC

>375_18

GATTGTGAGAATCGGTGGCATTGGTGTCTC

>376_18

GATCTTAACGAAGTGAATAGCGGCAGGGGC

>377_18

AGATCCGATGGGCTTGTGACCCGCCTCGGC

>378_18

GAATGGAGCGACGGTGTGGTCTGAGTCCTA

>379_18

TACAGGTGATCGGTGCTCGGGTGCCTGGCC

>380_18

GCCTCGCTTGGGAGGTTGCTCCACTAGTTC

>381_18

TGACGTCGTCGTAAATAGAGGCCTCGCGAC

>382_17

CAGTCGGTAGAACAGGGTGGGGTGCTGTCC

>383_17

CAAGTAACGCATGCGGAACGCGACGTAGTG

>384_17

AAGTCGGTAGAACGGGGTGGGGTGCTGTCC

>385_17

TCGCATAGCGTACCGGCAGTGGTCGGGGGC

>386_17

ATAGGCCGTGGCTGCGGGTGGGTGCGCACC

>387_17

ACGGCAGGTGTGGCGGTGGTCTGTGAATCC

>388_17

AAGTCGGTAGAACAGGGTGGGGTGCTGTCA

>389_17

GATCGGGAGAATCGGCGGCATTGGTGCCTC

>390_17

TCCGGGGGTCCGGGACGGGCGACGCTTGGG

>391_17

CCGTAGGAGAACAAAAGTGGCCTTGCTAGC

>392_16

GTATACCGGCAAGGAAGGGAAACGTTGCGA

>393_16

GAAGACTCTGGATTCGGGGAACAGTTGCTG

>394_16

TCCGGGGGTCTAAGACGACGCAATTCTGGG

>395_16

AAGTCGGTAGAATAGGGTGGGGTGCTGTCC

>396_16

ATGGCCGGTACGGCCTTCTAGTCCTCGGTA

>397_16

GGAACGCGGCAGCAGTGGGACTAGAAGGTA

>398_16

TAGACAAGCGGTAGCCCGTAGTGGGTTGTT

>399_16

AGGTCGGTAGAACAGGGTGGGGTGCTGTCC

>400_16

GAAGACTCGGGATTCGGGGACCAGTTGCTG

>401_16

AAGCGCAGGGCATGCGACCTCCAATTGGTC

>402_16

TCCGGGGGCCGTGGATAGGCGACGTTTAGG

>403_15

ACGGCAGGTGTTGCGGTGGTCTGTGTACCC

>404_15

GATCGGGAGAATCGGTGGCATTGGTGTTTT

>405_15

GATCGGGAGAATCGGCGGCATTGGTGTCTA

>406_15

GAAGACTCTGGATTCGGGGACCAGTTACTG

>407_15

TGGGTGTTGACGGCCGCCGCTGCGGCTACA

>408_15

GGTGCAGCGACCGGCAAGGGTTGAGGTGGG

>409_15

AAGTCGGTAGAGCAGGGTGGGGTGCTGTCC

>410_15

TATCGGCGTTAGCTAACATCGTAGGATGTT

>411_15

GAAGACCTTGGATTCGGGGACCAGTTGCTG

>412_15

ACCGCAGGTGTTGCGGTGGTCTGTGAATCC

>413_15

GATTGGGAGAATTGGTGGCATTGGTGTCTC

>414_15

ACGGCAGGTCTTGCGGTGGTCTGTGAATCC

>415_15

GATCGGGAGATTCGGTGGCATTGGTGTCTC

>416_15

ACGGCAGGTGTTGCGGTGCTCTGTGAATCC

>417_15

GGGAGGGCGCCGGTAGCGGTGTGAATGCGA

>418_15

GAAGACTCTGGATTAGGGGACCAGTTGCTG

>419_15

GCGAAGGTAACGGTGTGCGTAAAGGAGCTC

>420_15

ATGGCCGGCACGGTCTTCTAGTCCTCGGTA

>421_15

CAAGGGCATGTAATATACCGCGACATAGTG

>422_15

TCAGGGGGCATGCCAAGGCGACTCGATGGG

>423_15

GTTGGGGGCACGACCTTGGTAGGTATAAAG

>424_15

AAGTCGGTGGAACAGGGTGGGGTGCTGTCC

>425_15

CGGGTGGGCGCCCTAGGGTGTGAGTGTTGC

>426_15

GGGGTCGGTTGGAAGGACGTATTCCTGAGG

>427_15

AAGTCAGTAGAACAGGGTGGGGTGCTGTCC

>428_15

GATCGTGAGAATTGGTGGCATTGGTGTCTC

>429_15

GATCGGGAGAATCGGTAACATTGGTGTCTC

>430_15

TCCGGGGGCTCCGGAGGGGCGACGCGTGGG

>431_15

GCCACGGGCGTAGACCGCTGTTCTGGGTAA

>432_14

ATGGCCGGCACGGTCTTCTAGTCCTTGGTA

>433_14

CGTCTGTCCTTGGTAGTTACGGCCAATCTC

>434_14

CTAAGTGGACAACGCGTTAAATGGGGTAGA

>435_14

CTAAGTGGCCAACGCGTTAAGTGGGGTAGA

>436_14

AAGCCGGTAGAACAGGGTGGGGTGCTGTCC

>437_14

TGGGGCCGTGCTATCTGCACACTTCGCGGG

>438_14

ACGGCAGGTGTTGCCGTGGTCTGTGAATCC

>439_14

TTTGGGAGTCGCATTATGCAGGCAGTGAGA

>440_14

TGGGTGCAGACGAGTGCGCATGCAATTACA

>441_14

GAAGACTCTGGATACGGGGACCAGTTGCTG

>442_14

ACGGCAGGTGTTGCGGTGGTCTGTCAATCC

>443_14

CGTTATGAGTTGGGAAGCCTGGCGGCCAGA

>444_14

GATCGTGAGAATCGGTGGCATTGGTGTTTC

>445_14

GACGACTCTGGATTCGGGGACCAGTTGCTG

>446_14

GATCGAGAGAATCGGCGGCATTGGTGTCTC

>447_14

CCCACGGTAAAGCAGTGTACGTGTGGTGGA

>448_14

GATCGTGAGAATCGGTGGCATTGGTGTCTT

>449_14

CGGGCGGGCGCCCTAGGGTGTGAGTGTTGC

>450_14

GCCTCGCTTGGGAGGTTGCTCTACCAGTTC

>451_13

ACGTGCATGATATGCCGCCTGGAGCGTAGG

>452_13

TCCGGGGGCCTGAGTAGGCGACGCCCAGGG

>453_13

GCTTCGCTTGGGAGGTTGCTCCACCAGTTC

>454_13

ATGGCCGGCACGGCCTTCTAGTCTTTGGTA

>455_13

CGGTGTGGGTGCAGGGCCAGGCGGTTGATC

>456_13

ACGGCAGGTGTTGCGGTGGTGTGTGAATCC

>457_13

CGGTGTGGGGACCGTAGGTTCGGCGGGTAC

>458_13

GGGGGTCTGTCGGGCCGGAGTGCTCCAACG

>459_13

ACGGCCGGTGTTGCGGTGGTCTGTGAATCC

>460_13

CGGTGTGGGGAAGTTATATCGGCGGATGCT

>461_13

GATCTGGAGAATCGGCGGCATTGGTGTCTC

>462_13

GGAGTGGCTGAGGGCGGTGGTTAGGTCGCG

>463_13

AGGGGGTCTCGGGGTGATAACGATGTAGCT

>464_13

GGTACGAAGCCGAGGCTGTGGCTGAGTTTA

>465_13

TCGCATAGCGGAACTACGAAGTGTCGGGCT

>466_13

TCCGGGGGCCCTCGCGGGCGACGCTATGGG

>467_13

AAGTCGGTAGGACAGGGTGGGGTGCTGTCC

>468_13

TACATGTGTATTCCGAAGTGGAACGAGTCC

>469_13

TCCGGGGGTCCATGCGGACGACGTCTAGGG

>470_13

GAAGACTCTGGATTCGGGGACCAGTTGCAG

>471_12

CAAGGGAGTTGAAGTGACCGCGACGTAGTG

>472_12

TTTGGGAGTCGCGGTATGCAGGCAGTGAGA

>473_12

GAAGACTCTGTATTCGGGGACCAGTTGCTC

>474_12

CTGTGGGTTGGTAGGGGTGTACCTGTGGCG

>475_12

ACGGCAGGTGTTCCGGTGGTCTGTGAATCC

>476_12

GGTCGGGAGAACCGGTGGCATTGGTGTCTC

>477_12

ATGGCCGACACGGCCTTCTAGTCCTCGGTA

>478_12

TGGGGAGCAAGAGGACTGAGTAGGAATAAG

>479_12

TTGGGGGGCGACGGCCGGTAGTGGGTGAGA

>480_12

GTGGTGGGTGAGCAACGTGAGGGATGTCGA

>481_12

GAAGACTCTGGATTCGGGGACCTGTTGCTG

>482_12

GAAGACTCTGGATTCGGGCACCAGTTGCTG

>483_12

ATGGCCGGCATGGCCTTCTAGTCCTCGGTA

>484_12

AGTACAACGGCAGGCATTGTGTCTCGGTAG

>485_12

CGATGGTGGGCTAGAGACCTCCGAAGTGGA

>486_12

CTGAGTGGGCTGGGAGGAAGTATTTCGGAG

>487_12

CGGCTGGCTGGCAGTGCGTACTAGTAGTCA

>488_12

GCAAGCGCGGTCCCTCGGACCTCACAGTTG

>489_12

GCCTCGCTTGGGAGGTTGCTCCATCAGTTC

>490_11

TGGAGGGCGCCGGCAGCGGTGTGAATGCGA

>491_11

GGTCCGAGCGATGCGTGCCGTGGTCGAGCT

>492_11

TCCTTGCTTGGGAGGTTGCTCCACCAGTTC

>493_11

ACGGGAGGTGTTGCGGTGGTCTGTGAATCC

>494_11

GATCGGTAGAATCGGCGGCATTGGTGTCTC

>495_11

TGGGGCCGTGCTATTTGCACACTCCGCGGG

>496_11

CGGCGTGGGGCCATCTGTGCGGCGGATCTC

>497_11

TCAGCATAGCATATGCGCCTGGAGCGTAGG

>498_11

TCCGGGGGCACCTAAGTGCGACGCTGTGGG

>499_11

CGGCGTGGGGCCATCTGTGCGGCGGATCCT

>500_11

GATCGGGAGAATCAATGGCATTGGTGTCTC

>501_11

GAAGACTTCGGATTCGGGGACCAGTTGCTG

>502_11

GATGACTCTGGATTCGGGGACCAGTTGCTG

>503_11

GATCGGGAGGATCGGTGGCATTGGTGTCTT

>504_11

GAAGACTCTGGATTCGCGGACCAGTTGCTG

>505_11

GCGTAGCGTGGGCACGGTGCGGGTTTCCTC

>506_11

GATCGGGAGGATCGGCGGCATTGGTGTCTC

>507_11

GGGGTGTAGGACTTCAAGTGGATCTCATAG

>508_11

ATGGCAGGTGTTGCGGTGGTTTGTGAATCC

>509_11

CGGGCGGGCGCCCAAGGGTGTGAGTGTTGC

>510_11

GATCGAGAGAATCGGTGGCATTGGTGTCTT

>511_11

GATCGGTAGAATCGGTGGCATTGGTGTCTT

>512_11

TCCGGGGGCACCAATGTGCGACGCTGTGGG

>513_10

GAAGACTCTGGATTCCGGGACCAGTTGCTG

>514_10

CTCGTGTAGCGACCTGGGTGCGGCCGGTGA

>515_10

GGGAGGGCGCTGGCAGCGGTGTGAATGCGC

>516_10

TCGAGTATCGGTGGTCTGATTCAGATGCGC

>517_10

TCCGGGGGCACTTATGTGCGACGCTGTGGG

>518_10

ACGGCAGGTGTTGCGGTGGTCTGGGAATCC

>519_10

ACCTGTACGGTGATGAGGTCGGTATCAGCC

>520_10

TTAGCGTCTTGCGAGCGGGTGGGTTCGCTC

>521_10

GAAGACTCTGGTTTCGGGGACCAGTTGCTG

>522_10

AAGTCGGTAGAACAGGGTGTGGTGCTGTCC

>523_10

CTGAGGGACGGCACGTCATGTCGAAGTGGA

>524_10

GAAGACTCTGGATTCGGGGACCAGTTGCGG

>525_10

GAAGTCTCTGGATTCGGGGACCAGTTGCTG

>526_10

TTAGCGTCTTGCGAGCGGGTGGTTCGCTCC

>527_10

GCCTCGCTTGGGAGGTTGCTCCACCAGTTT

>528_10

GGGGATCTGTCGGGCCGGAGTGCTCCAACG

>529_10

GAAGACTCTGGATTCGGGGACTAGTTGCTC

>530_10

TACCAGAGGTGAGCGAAGGTGTGCGAGCTA

>531_10

TAGCAGAAGGTGTGAATGAAAAGGGATCTA

>532_10

GGGGTAGGGGGCCGCTCGCGCCTGTGTGCG

>533_10

AAGTCGGTAGAAAAGGGTGGGGTGCTGTCC

>534_10

TCCGGGGGCCTGAAGGGCGACGCGCTCGGG

>535_10

GATCGGGAGAATTGGTGGCATCGGTGTCTC

>536_10

GAAGACTCTGGCTTCGGGGACCAGTTGCTG

>537_10

TTAGGCCGCCAGGGCGTACGAAGTGTGGTT

>538_10

ATGGCCGGCACGGCTTTCTAGTCCTTGGTA

>539_10

TCCGGGGGCCGCGGCCGGCGACGCAATGGG

>540_10

GATCGGGAGACTTGGTGGCATTGGTGTCTC

>541_10

CAATGGGGCTGGTAGGAAAGTACTTATAGC

>542_10

GCTCCGAGCAAGCGAACCGCGAAAAGTCGG

>543_9

TGGGGAGCGAGAGGACTGAGTAGGAATAAG

>544_9

GATCGGGAGAATTGGTGGCATTGGTGTCTT

>545_9

GATTGGGAGAATCGGTGGCATCGGTGTCTC

>546_9

GAAGACTCTGGATTCGGCGACCAGTTGCTG

>547_9

AAGTCTGTAGAACAGGGTGGGGTGCTGTCC

>548_9

AACGGATCGGGAGGCGTGGCGGATTGTGTG

>549_9

ATGGCCGGCACGGCCTTCTAGTTCTCGGTA

>550_9

ACGGCAGGTGTTGCGCTGGTCTGTGAATCC

>551_9

ACAGGCCTTGGCTGCGGGTGGGTGCGCACC

>552_9

GGGAGGGCGCCGACAGCGGTGTGAATGCGA

>553_9

GCCTTGCTTGGGAGGTTGCTACACCAGTTC

>554_9

CAACGTGGGCACAGCAAAGTGGAAGTGGAG

>555_9

CGAAGTGGGCTCTTGGCGAGTGGCGACCGC

>556_9

GAAGACTCTGGATTTGGGGACCAGTTGCTC

>557_9

CAGTGGGCGGACCGGGACCCAAGGGGCGGA

>558_9

GATAGGGAGAATCGGTGGCATTTGTGTCTC

>559_9

AAGTCGGTAGAACAGGGGGGGGTGCTGTCC

>560_9

GGGAGGGCGCCGGCAGCGGTGTGTATGCGA

>561_9

TCGCATAGCGAAGGATAAGATGTCCCGGGC

>562_9

TTGGTGCTGACGGCCGCCGCTGCGGCTACA

>563_9

GATCGTGAGAATCGGTGGCATCGGTGTCTC

>564_9

GTAGACTCTGGATTCGGGGACCAGTTGCTG

>565_9

AAGTCGGTAGAACAGGGTGGGGTGATGTCC

>566_9

GCCTTGCTTGGGAGGTTGCTCCATCAGTTC

>567_9

CCTGGTGGGACATGCTCAAACCAGTGTGGA

>568_9

TCCGGGGGCATCTATGTGCGACGCTGTGGG

>569_8

TCCGGGGGCCAGCATTGGCGACGCTCAGGG

>570_8

GAAGACTCTGGATTCGGGGCCCAGTTGCTG

>571_8

GATCGTAAGAATCGGTGGCATTGGTGTCTC

>572_8

GATTGGGAGAATCGGTGGTATTGGTGTCTC

>573_8

GCCTCGCTTGGGAGGTTGCTACACCAGTTC

>574_8

GAAGTGGGCAAGGGGCAAAGTGTGTCGAGC

>575_8

ACGGCAGCTGTTGCGGTGGTCTGTGAATCC

>576_8

TCGCACAGCGGACGACTATCAGGGAGGGGA

>577_8

CGATGATGATGGGCAGGCTGGGGAGTGTTG

>578_8

TAGCGAAGGGTACCAGTGTGGGAGTCCGTC

>579_8

GCTCTACGCGTCCTTGAGTGCCGACGTACA

>580_8

GGAGTGGCTAAGGGCGGTGGGTAGGTCGCG

>581_8

GGGAGGGCGCCGGTAGCGGTGTGAATGCGC

>582_8

GAAGACTTTGGATTCGGGGACTAGTTGCTG

>583_8

TTCGGGGGCACCTATGTGCGACGCTGTGGG

>584_8

TCCGGGGGCCGACCGCGGCGACGTCTAGGG

>585_8

TAGCGAACATGCGACCAGAGTGGTTAAGTG

>586_8

CCAGCTGAGGCGTCATGCAGCGGAGTGGAA

>587_8

TCGCATAGCGCACGACAGGTCTGGAGGGGA

>588_8

TCAGCATAGTATATGCGCCTGGAGCGTAGG

>589_8

ACGGCAGGTGTTGCGGTGGTTTGTGAATCT

>590_8

AAGTCGGTAGAACAGGGTGAGGTGCTGTCC

>591_8

GCCTTGCTTGGGAGGTTGTTCCACCAGTTC

>592_8

TGGGTGCTGACGGCCGCTGCTGCGGCTACA

>593_8

TCCGGGGGACGAGCTAGTCGACGCTTTGGG

>594_8

GATCGGGAGAATCGTCGGCATTGGTGTCTC

>595_8

TCCGGGGGCCTCGCAAAGGCGACGCCAGGG

>596_8

CTAAGTGGTCAACGCGTTAAATGGGGTAGA

>597_8

CGGCGTGGGGCTATTGATGCGGCGGCACCC

>598_8

TCGCACGGCGGAGTTAGAGACTAACCGGGA

>599_8

CGGGATCGTGTCGGCGGATAGCGTACTTGG

>600_8

AGCGTGTTGCAAGGTCCGTGAGTGTGGACC

>601_8

GATCGGGAGAATCGGTGGCATCGGTGTCTT

>602_8

GGAGACGGGATGAGTCGGGACTAGAAGGTA

>603_8

AAGTCGGTAGAACAGGGTGGTGTGCTGTCC

>604_8

GGGAGGGCGCTGGCAGCGGTGTGAATGCGA

>605_8

TAGCGTAGGACCGTCGGAATGAGGGTGGAT

>606_8

AAGTGGGCGATCTGGGAGAGGTCAATCATG

>607_8

CGACGAAGGTGCAATGACTGAGGATTCGCC

>608_7

AAGTCGGTCGAACAGGGTGGGGTGCTGTCC

>609_7

GCCTTGCTAGGGAGGTTGCTCCACCAGTTC

>610_7

GATCGGAGGAATCGGTGGCATTGGTGTCTC

>611_7

GCCTTGCTTGGGAGGTTGCTCCACCAGTTA

>612_7

AAGTAGGTAGAACAGGGTGGGGTGCTGTCC

>613_7

CGTAGTGGGCTGGCAGGGAGTACTTGTAGC

>614_7

TCGCAGAGCGTACAGTGGCATCAGTGGGGC

>615_7

AAGTCGGTAGAACAGGTTGGGGTGCTGTCC

>616_7

AAGTCGGTAGAACAAGGTGGGGTGCTGTCC

>617_7

AGGGTGTAGGACTTTAAGTGGATCTCATAG

>618_7

GATCAGGAGAATTGGTGGCATTGGTGTCTC

>619_7

ATGGGGGGCGACGGCCGGTAGTGGGTGAGA

>620_7

TCCGGGGGCGTTACGACGCGACGCTGTGGG

>621_7

GATCGTGAGGATCGGTGGCATTGGTGTCTC

>622_7

AAGTCGGAAGAACAGGGTGGGGTGCTGTCC

>623_7

GGGAGGGCGCCGACAGCGGTGTGAATGCGC

>624_7

GATCGTGAGAATCGGTGGCATAGGTGTCTC

>625_7

GATCGGGAGAAACGGTGGCATAGGTGTCTC

>626_7

AAGTCGGTAGAACAGGGCGGGGTGCTGTCC

>627_7

AGCGTGTTGCAAGGTCTGCGAGTGTGGACC

>628_7

TGGGTGCTGACGGCCGCCGTTGCGGCTACA

>629_7

ATGGCAGGTGTTGCGGTGGTCTGTGGATCC

>630_7

GGGGTAGGGGGCCGCTCGCGCCTACGTGCG

>631_7

AAGTCGGTAGAACAGGGTGGGTTGCTGTCC

>632_7

GCCTTGCTTGGGAGGTTGCTCCACCAGTTT

>633_7

GCCTTGCTTGGGAGGTTGCTCCGCCAGTTC

>634_7

CCCGAGAAGCGACCGAAGAGCAACCAGTGA

>635_7

AAGTCGGTAGAACAGGGTGGAGTGCTGTCC

>636_7

AAGTCGGTAGAACAGGGTGGGGTGCTGTTT

>637_7

GATCGGGAGAATCGGCGTCATTGGTGTCTC

>638_7

CGGTGTGGGGCATCTCTCGCGGCGGTTATT

>639_7

GTCTTGCTTGGGAGGTTGCTCCACCAGTTC

>640_7

GATTGGGAGAATCGGTGGCATTGGTGCCTC

>641_7

GCGGCAGGTGTTGCGGTGGTCTGTGAAACC

>642_7

GGCAAGGTGTGCGGCGGGCGGGTACCACGG

>643_7

GATCGGGAGAATCGGCGGCATTGGTGTCCC

>644_7

GGGAGGGCGCCGGCAGCGGTGTGAATGCGG

>645_7

ACGTGCAATATATGCCGCCTGGAGCGTAGG

>646_7

AATTCGGTAGAACAGGGTGGGGTGCTGTCC

>647_7

ATGGCCAGCACGGCCTTCTAGTCCTCGGTA

>648_7

GATCAGGAGAATCGGTGGCATTGGTGTCTT

>649_7

GATCGTGAGAATCGGTGGCATTGGCGTCTC

>650_7

CTAGGCGTCAGCACCGGGTGGGTTCGGTCC

>651_7

GAGAATCCGAGCGATAGGTGTGTGCTCCCA

>652_7

GAAGACTCTGGATTCGGGGACCAGGTGCTG

>653_7

GCCCTGCTTGGGAGGTTGCTCCACCAGTTC

>654_7

CAGCCGTAGCCGTAAGGTTGGTATAAGGTC

>655_7

GTGCGGGCAACACGCAGTCCTGGTGTGCGA

>656_7

TAGCGTCTTGCGAGCGGGTGGGTTCGCTCC

>657_7

AAGTCGGTAGAACATGGTGGGGTGCTGTCC

>658_7

TATCGGGAGAATCTGTGGCATTGGTGTCTC

>659_7

GGGATCGGCTAGGTGTCGTGTAGGGTGAGA

>660_7

GGGAGGGCGCCGGCAGCGGTGTGAATGCGT

>661_7

GATCGCGAGAATCGGTGGCATTGGTGTCTT

>662_7

TGGGGAGCTAGAGGACTGAGTAGGAATAAG

>663_7

GATCGGGAGAATCGGCGGCATTGGCGTCTC

>664_7

CGATGGTTGGGAGGGAAGTATTTCGGGCGA

>665_7

CGGAGTGGGGCTCATTGTGCGGCGGTAACC

>666_7

GATCGGGAGAATCGGTGGCATTGGTGCTTC

>667_7

CGTCGTGGCTGGTAGAGTGTATTTGACAAG

>668_7

AAGTCGGTAGAACAGAGTGGGGTGCTGTCC

>669_7

GTGGGGGGCGACGGCTGGTAGTGGGTGAGA

>670_7

CTAAGTGGGCAACGCGTTAAATGGGGTAGA

>671_6

GATCGGGAGAGTCGGTGGCATTGGTGTCTA

>672_6

GATCGGGAGGATCGGTGGTATTGGTGTCTC

>673_6

GATCGGGAGAATCGGTGGCATAGGTGTCTT

>674_6

TCCTGTAGGAAGAGTAGCGGCTTCGTGAGA

>675_6

CGAAGGTCTTTAGTGGGCCCGAGGGCCGAC

>676_6

ATGGCCGGCACGGCCTTCTAGTCCTCGGTG

>677_6

GATCGGGAGAATTGGTGGCATTGGTGCCTC

>678_6

GATTGGGAGAATCGGTGGCATTGGTGTCTG

>679_6

TATCGGGAGAAACGGTGGCATTGGTGTCTC

>680_6

GAAGACTCAGGATTCGGGGACCAGTTGCTC

>681_6

GATCGGGAGAATTGGTGGCATTGATGTCTC

>682_6

GATCGTGAGAATCGGTGTCATTGGTGTCTC

>683_6

ACGGCAGGTGTTGCGGTGGTCTGTGATTCC

>684_6

GCCTCGCTTGGGAGGTAGCTCCACCAGTTC

>685_6

GAAGACTCTGGATTCGGGGACGAGTTGCTG

>686_6

GATCGAGAGGATCGGTGGCATTGGTGTCTC

>687_6

ATGGCAGGTGTTGCGGTGGTCTGTGTATCC

>688_6

TAGCGGCGTAGGCTGCCTTGGGTGGTGGCC

>689_6

TCGCATAGCGGAAGCAGGTAGTGCTCGGGC

>690_6

CTCGACCGGGGCCCTAGGGTTGCGGCCCAC

>691_6

GATCGGGAGAATCGGTGTCATTGGTGTCTA

>692_6

GCCTTGCTTGGGAGGCTGCTCCACCAGTTC

>693_6

GATTGGGAGAATCGGTGGCATTGGTGTCCC

>694_6

CGGTGTGGGGAACTCGTTTCGGCGGTGCTA

>695_6

TAGCCGAAGGGACCAGAAGGGAGTCACTGT

>696_6

GGGAGGGCGCCGGCAGCGGTGTGAATGAGA

>697_6

CTGTGGCTGGTAGGTTCGTACTATAGGCGG

>698_6

GATTGGGAGAATCGGTGGCACTGGTGTCTC

>699_6

CGGTGTGGGGACCGCAGGTTCGGCGGCTAC

>700_6

GTCGGGGATTGGCAGGGGCGTTGACTGGCA

>701_6

GGGAGGGCGTCGGCAGCGGTGTGAATGCGA

>702_6

GATCGAGAGAATCGGTGGCGTTGGTGTCTC

>703_6

GATCGGGGGAATCGGTGGCATTGGTGTCTT

>704_6

CGGGTTGCTGGTAGGTGGTACGACCTCGAT

>705_6

GATCGGTAGAATCGGTGGCATTGGAGTCTC

>706_6

CGGTGTGGGGAACTTGTTTCGGCGGTGTTA

>707_6

TCCGGGGGCACCTATGTGCGGCGCTGTGGG

>708_6

ACGTGCAAAATATGCCGCCTGGAGCGTAGG

>709_6

TCCGGGGGTGCGCGAGAACGACGACTTGGG

>710_6

AAGTCGGTATAACAGGGTGGGGTGCTGTCC

>711_6

AAGTCGGTAGAACAGGGTGGGGTGCCGTCC

>712_6

ATGGCCGGCACGGCCCTCTAGTCCTCGGTA

>713_6

GCGGCAGGTGTTGCGGTGGTTTGTGAATCC

>714_6

GAAGACTCTGGATTCGGGGTCCAGTTGCTG

>715_6

GGGCCGGTCGCGGAGGTGGTCTGGCGAACC

>716_6

GCCTAGCTTGGGAGGTTGCTCCACCAGTTC

>717_6

GATCGGGAGAATCGTAGGCATTGGTGTCTC

>718_6

GATCGGGAGAAACGGAGGCATTGGTGTCTC

>719_6

GCCTCGCTTGGGAGGTTGCTCCACCAGCTC

>720_6

GATCGGGAGAATCGGATGCATTGGTGTCTC

>721_6

TCGCATAGCGTACACTTCAAAAAGTGCGGA

>722_6

GGTCGTGAGAATCGGTGGCATTGGTGTCTC

>723_6

GATTGGGAGAATCGGAGGCATTGGTGTCTC

>724_6

CGGCGTGGGGCCATATGTGCGGCGGATCCC

>725_6

AGCGAAGGAGCTCGTAAGGCGGCGTACCGG

>726_6

GGAGTGGTTGAGGGCGGTGGGTAGGTCGCG

>727_6

GATCGGGAGGATTGGTGGCATTGGTGTCTC

>728_6

GATCGAGAGAATTGGTGGCATTGGTGTCTC

>729_6

GATCAGGAGAATCGGTGGCATTGGTGTCCC

>730_6

GAGGGGGACAAAGTGCGGGTGGTCGAGCCT

>731_6

GCGGCAGGTGTTGCGGTGGTCTGTGGATCC

>732_6

ACGGCAGGTGTTGCGGTGGTCTGTGGATTC

>733_6

ACGGCAGGTGTTGCGGTGGTCTGTGGAACC

>734_6

GAAGACTTTGGATTTGGGGACCAGTTGCTG

>735_6

TTTGGGAGTCGCAGTAAGCAGGCAGTGAGA

>736_6

TATCGGGAGAATTGGTGGCATTGGTGTCTC

>737_6

GATCGGGAGAATTGGTGGCATTGGTGTTTC

>738_6

GATCGTGAGAATCGGTGGCGTTGGTGTCTC

>739_6

CGGTGTGGGGCTATTGATGCGGCGGCACTC

>740_6

GATCGGGAGAATCGGAGGCATAGGTGTCTC

>741_6

AGCGCAGGGTGCGAGAGGGGCGTGTCCATG

>742_6

TCCGGGGGCCAACGGCGACGCACTTAAGGG

>743_6

GACCAGCAAGGGCTTCGAGGGACGGGAAGG

>744_6

GAAGAGTCTGGATTCGGGGACCAGTTGCTG

>745_6

GATCGGGAGAATCGGTGGCATTAATGTCTC

>746_6

CATTGACAGCTAGGGTGAGGTTGTGTGCGA

>747_6

GATTGGGAGAATCGGTGGCATTGGTGTTTC

>748_6

ACGGCAGGTGTTGCGGTGGTCTGTGGATCT

>749_6

GGGAGGGCGCCGGCAGCGGTGTGAATGTGA

>750_6

GAACACTCTGGATTCGGGGACCAGTTGCTG

>751_6

GATCGGGAGAATCGGTGGCATAGGAGTCTC

>752_6

GATCGGAAGAATCGGTGGCATTGGTGCCTC

>753_6

AAGACGGTAGAACAGGGTGGGGTGCTGTCC

>754_6

GATCAGGAGAATCGGTGGCATCGGTGTCTC

>755_6

GAGCCGCCACGGGTTGGGCCGGTGCGACAG

>756_5

GTGGGGGGCGACGGCCGGTAGTGGGTGAGG

>757_5

TCTGGGGGCACCTATGTGCGACGCTGTGGG

>758_5

GATCGTGAGAATCGGTGGCATTGGTGCCTC

>759_5

CTAAGTGGGCAACGCGTTAAGTGGGGTAGA

>760_5

TCCGGGGGCACCTGTGTGCGACGCTGTGGG

>761_5

GATCGGGAGAATCGGTGTCATTGTTGTCTC

>762_5

AGACGACGCAGTCGATCGGGTTAGAGGAAG

>763_5

GCCTCGCTCGGGAGGTTGCTCCACCAGTTC

>764_5

GATTGGGAGAATCGGTGACATTGGTGTCTC

>765_5

TCCGGGGGCTGCATGCGGCGACGCGACGGG

>766_5

TCCGGGGGCGCCTATGTGCGACGCTGTGGG

>767_5

GATCGGGAGAATCTGTGGCATTGGTGTTTC

>768_5

GATCGCGAGAATCGGTGGCATTGGTGTTTC

>769_5

TTAGCGTCTGCGAGCGGGTGGGTTCGCTCC

>770_5

GATCGGGAGAATCGGCGGCATAGGTGTCTC

>771_5

GATCGTGAGAATCGGTGGTATTGGTGTCTC

>772_5

CCCGAGAAGGACAAACGTGCCGTCCAGTGA

>773_5

GATCGGGGGACTCGGTGGCATTGGTGTCTC

>774_5

GCGGCAGGTGTTGCGGTGGTCTGTGAATCT

>775_5

GATCAGAAGAATCGGTGGCATTGGTGTCTC

>776_5

GATCGGGAGAGTCGGTGGCATTGGTGTCTT

>777_5

GATCGGTAGAATCGGTGGCATTGGTGTTTC

>778_5

GATCGGAAGAATCGGTGGCATTGGTGTCTT

>779_5

GATCGGGGGAATTGGTGGCATTGGTGTCTC

>780_5

TATCGTGAGAATCGGTGGCATTGGTGTCTC

>781_5

GATCGGGAGAATCGGTGGCATAGGTGTCTA

>782_5

ACGGCAGGTGTTGCGGTGGTCTATGAATCT

>783_5

GATCGGGAGAATCGGTGGAATTGGTGACTC

>784_5

TCGCACAGCGCACGAAAATTGTGTAGGGGA

>785_5

CGGCGTGGGGCTATCTGTGCGGCGGATCCC

>786_5

ATGGCCGGCACGGCCTTCTAGTCCTCAGTA

>787_5

CGGGATCGTGTCGGCTGATAGCGTACTTGG

>788_5

GCCTCGCTTGGTAGGTTGCTCCACCAGTTC

>789_5

GATTGGGAGAATCGGTGGCATTAGTGTCTC

>790_5

CTAAGTGGACAACGCGTTAAGTGGGGTAGA

>791_5

CTACTGTAAGCGAAAGAAGCAACAGTGGAG

>792_5

AAGTCGGTAGAACAGGGTGGGGTGCAGTCC

>793_5

GATCGGGAGAATCTGTGGCATAGGTGTCTC

>794_5

TCGCACAGCAGGCAAAGAAGTTGACTAGGC

>795_5

GATTGGTAGAATCGGTGGCATTGGTGTCTC

>796_5

CTAGATATGGTAGGTAATGTGCTGAGGAGG

>797_5

ACCTTGCTTGGGAGGTTGCTCCACCAGTTC

>798_5

GATCTGGAGAATCTGTGGCATTGGTGTCTC

>799_5

GCCTCGATTGGGAGGTTGCTCCACCAGTTC

>800_5

TGGGTGCTGACGGTCGCCGCTGCGGCTACA

>801_5

CCCAGCTAAAATGAGTCACGGTTCGGAAGT

>802_5

GTAGTGGCTGAGAGCACCAAGTGTCCGAGA

>803_5

GATCGCGAGAATCGGCGGCATTGGTGTCTC

>804_5

GTATCGGCTGGTAGGTTGCGTATTGGGGAG

>805_5

TCCGGGGGTCCCCCAGGGACGACGCTGGGG

>806_5

GATCGGGAGAATCGGCGACATTGGTGTCTC

>807_5

GGGAAGTGGTCAAGCGGCAGCTCCCGTAGA

>808_5

GATCGGGAGAATCGGTGGCAATGGAGTCTC

>809_5

GATCTGGAGAATCGGTGGCATTTGTGTCTC

>810_5

GCCTTGCTTGAGAGGTTGCTCCACCAGTTC

>811_5

GGAAGGGCGCCGGCAGCGGTGTGAATGCGA

>812_5

TCGCATAGCGGACCAGTCAGAAGTGGGGGC

>813_5

GATCGGGAGAATCAGCGGCATTGGTGTCTC

>814_5

GAAGACTCTGGATTCGGGGACCCGTTGCTG

>815_5

TCAAGTGCGCTTGGTAATTCCTGATGCGAC

>816_5

GAAGCCTCTGGATTCGGGGACCAGTTGCTG

>817_5

CTCGTGAGGCATGGGTATGCTGTCCGGTGA

>818_5

TGGGGCCGTGCTATCTGCACACTCTGCGGG

>819_5

CAGCAAAGGGTGTGAGCAAGGCGAGTCCTA

>820_5

GATTGGGAGGATCGGTGGCATTGGTGTCTC

>821_5

GAAGACTCTGGATTCGGGGACCAGTTGGTG

>822_5

GATCTGGGGAATCGGTGGCATTGGTGTCTC

>823_5

GATTGGAAGAATCGGTGGCATTGGTGTCTC

>824_5

CAATGGAGCGACGGTGTGGTCTGAGTCTTA

>825_5

GAAGACTCTGGATTCGGGGACCACTTGCTG

>826_5

GATCGGGAGAATCGGCTGCATTGGTGTCTC

>827_5

GCCTTGATTGGGAGGTTGCTCCACCAGTTC

>828_5

TCGCATAGCGGACGACGAATGTGTCGGGGC

>829_5

GATCGGGAGAATCGGCGGCATTTGTGTCTC

>830_5

GCCCCGCTTGGGAGGTTGCTCCACCAGTTC

>831_5

CATGGGCGACCTAAGAGAGGAGAAGTCGAA

>832_5

CAAGCGATGGAACTATAGCGAGACGTAGTG

>833_5

GGTCGGGAGAATCGGCGGCATTGGTGTCTC

>834_5

GCCTTGCTTGGGAGGTTGCTCCACCAGTAC

>835_5

GATCGGGAGAATCGGCGGCAGTGGTGTCTC

>836_5

GATCGGGAGAATCGGCGGCGTTGGTGTCTC

>837_5

GGTGGGGTGTGGATAAGCGAGTGGTCGGCA

>838_5

CGAGCTGGGTGCGCGTTAGATTGGGATAGC

>839_5

GATCGAGAGAAACGGTGGCATTGGTGTCTC

>840_5

ATGAGCCTGTAGGCCAGAGCAGACGTTTGG

>841_5

CATCAGCTCCGCGGTCGGGGACGTAAGGGA

>842_5

GCCTTGCTTGGGAGATTGCTCCACCAGTTC

>843_5

CGTGGCTGGTAGGGTAAGTACTGTGTCGAT

>844_5

ATGGCCGGCGCGGCCTTCTAGTCCTCGGTA

>845_5

GATCGGGAGAATCGGTGGTATTGGTGTCTT

>846_5

AGCAGAAGGTGTGATGTTGTAAGAGAACTA

>847_5

TCCGGGGGCGCTCAGGGCGCGACGCTGGGG

>848_5

GATCGGAAGAATCGGTGGCATCGGTGTCTC

>849_5

TCCGGGGGGTCGGGAAGACCGACGCTAGGG

>850_5

GATTGGGAGAATCGGTGGCATTGGCGTCTC

>851_5

CGGCGTGGGGCATCTCTCGCGGCGGTTATT

>852_5

GATCGGGAGAATCGGTGGCATCGGTGTTTC

>853_5

ATTGCCGGCACGGCCTTCTAGTCCTCGGTA

>854_5

TCCGGGGGCCTGAATTGGCGACGACTTGGG

>855_5

TATCTGGAGAATCGGTGGCATTGGTGTCTC

>856_5

GATCGGGAGAAACGGTGGCATTGGAGTCTC

>857_5

GATCGGGAGAATCGGTGGACTTGGTGTCTC

>858_5

TATCGGGAGAATCGGTGGCATTGGTGTCTA

>859_5

GTGGGGGGCGACGGCCGTTAGTGGGTGAGA

>860_5

TTAGGGAGTCGCAGTATGCAGGCAGTGAGA

>861_5

GATCGAGAGAATCGGTGGCATTGGTGTCCC

>862_5

CGGTGTGGGGACCTTGTTTCGGCGGTGCTA

>863_5

CGATTGGTCGGAAGGCAAGTGTTCGGATGA

>864_5

GCCTTGCTTGGGAGGTTGCCCCACCAGTTC

>865_5

CGGTGTGGGGAACTTGTTTCGGCGGTGCTG

>866_5

CTAGGTTCCGCTCCGGACCGCGACGTAGTG

>867_5

CGGCGTGGGGCTATTGATGCGGCGGCACTT

>868_5

TATGCAGGAGCAGGATCCGGGTGGGCAGGC

>869_5

ACGGCAGTGTCTAAGTGGCTGCATTGGAAC

>870_5

CGTGGTGTGTTGCCCAAGTGGCTGAGTTGA

>871_5

GTGGGGGGCGACGGCCGGTAGTGGGCGAGA

>872_5

GATCGGCAGAATCGGTGGCATTGGTGTCTT

>873_5

GATCGCGAGAATTGGTGGCATTGGTGTCTC

>874_5

TCGCATAGCGTACGACGTTGAGGACGGGGA

>875_5

CGGTGTGGGGACCGCAGGCTCGGCGGGTAC

>876_5

CTACGGGCTAGAGTTACTCGTGTTAATAAG

>877_5

AGCGAGGGTGTGAAAGGCACTGAAGTACTA

>878_5

GGGTGTGGGGAACTTGTTTCGGCGGTGCTA

>879_5

CCCGGGGGCACCTATGTGCGACGCTGTGGG

>880_5

GATCGTGAGAGTCGGTGGCATTGGTGTCTC

>881_5

GCCTCGCTTAGGAGGTTGCTCCACCAGTTC

>882_5

GATCGGGAGAGTTGGTGGCATTGGTGTCTC

>883_4

TCGCATAGCTATAAGGGAGTGAATACGGGC

>884_4

GAAGACTTTGGATTCGGGGATCAGTTGCTG

>885_4

GATCGGGAGACTCGGTGGCATTGGTGTCCC

>886_4

CCCGGTCTTACGAAGGAATGCCGTGGTGGA

>887_4

GGGAGGGCTCCGGCAGCGGTGTGAATGCGA

>888_4

GATCGGGAGAATCTGTGGCATTGGTTTCTC

>889_4

TAACGGGAGAATCGGTGGCATTGGTGTCTC

>890_4

AAGTCGGTAGAACAGGGTGGGGTGCTGTCG

>891_4

ACGGCAGGTGTTGCGGTGGTCTGTGGATCA

>892_4

GATCGGGAGAATCGGTGGCATAGGTGTATC

>893_4

GATCGGGAGAATCGGCGGCATTGGGGTCTC

>894_4

CAAGTGACAGAGGTGGAACGTGACGTAGTG

>895_4

CCAGCAGTCCCAGGAATGCGAGACGTAGTG

>896_4

TGGGTGCAGACGAGTGCGCTAGCAATTACA

>897_4

TATCGGGAGAATAGGTGGCATTGGTGTCTC

>898_4

GATCGGGAGAATCGGTGGCATAGGTGTCAC

>899_4

GAAGACTCCGGATTCGGGGACCAGTTACTG

>900_4

GCCTTGCTTGGGAGGTTGCTCCAACAGTTC

>901_4

GCACTACGTAGCGGTAGGCATTGGTGTCTC

>902_4

CGTGGGCGTTTCGGCCGTCTCGGCGTGTGC

>903_4

CGAGATTTGGTAGGCAGATGTGCTCTAGCA

>904_4

CTAAGTGGCCCACGCGTTAAATGGGGTAGA

>905_4

GATCGGGAGAATCGGTGGCATTGGTGCCTT

>906_4

GGGAGGTCGCCGGCAGCGGTGTGAATGCGA

>907_4

GATCGGGAGAATCGGTGGAATTGGTGTCTA

>908_4

GATCGGGAGAATCGGTGGAATTGTTGTCTC

>909_4

GAGGCCTAAGTGCGTATCTGTGGTCGAGCT

>910_4

ATGGACGGCACGGCCTTCTAGTCCTCGGTA

>911_4

CGGGGCCGGACGGGACGGACACATCGCGGC

>912_4

AGACTGGTTGAGATGCGGGGTGGTCGAGCT

>913_4

GATCGGTAGAATCGGTGTCATTGGTGTCTC

>914_4

TGGGTGCTGACGGCTGCCGCTGCGGCTACA

>915_4

CGTTGGGAGCGAAGTCCCAGCGAAGTGGAC

>916_4

CTCGTGGGCATATATGGGCCACGTAGTGGA

>917_4

GCCTTGCATGGGAGGTTGCTCCACCAGTTC

>918_4

GATCGGGAGAATCTGTGGCATTGGTGACTC

>919_4

TCCGGGGGCACCTATGTGCGACGCTGCGGG

>920_4

GATCGGGAGAATCGGTGGCAGTGGTGTCCC

>921_4

GATCCGGAGAATCGGCGGCATTGGTGTCTC

>922_4

GATCGTGAGAATCGGTGGCACTGGTGTCTC

>923_4

GATCGTTAGAATCGGTGGCATTGGTGTCTC

>924_4

AGACGACGCAGTCGATCGGGATAGAGGAAG

>925_4

CAATGGAGCGACGGTGTGGTCTGAGTTCTA

>926_4

GATCGGGAGAATCGTTGGCATTGGAGTCTC

>927_4

TATCGGGAGAATCGGTGGAATTGGTGTCTC

>928_4

TCCGGGGGCCATGCAGTGGCGACGTCTGGG

>929_4

GAAGACTCTGGATTCGGGGAGCAGTTGCTG

>930_4

GATCGGGAGGATCGGTGGCATTGGTGCCTC

>931_4

GGAGTGGCTGAGGGCGGTGGGTAGGTCGCT

>932_4

GATCGGAAGAATTGGTGGCATTGGTGTCTC

>933_4

GGGAGGGCGCCAGCAGCGGTGTGAATGCGA

>934_4

GATCGGGAGAAACGGTGGCATTGGTGACTC

>935_4

GGGAGGGCGCCGGCAGCGGTGTGAATACGC

>936_4

GATCGGGAGAATCGGCGGAATTGGTGTCTC

>937_4

AAGTCGGTAGAACAGGGTGGGGTGCTATCC

>938_4

CCGTAGGAAAACAAAGGTGGCCTTGCTAGC

>939_4

GGGAGGGCGCCGGCAGAGGTGTGAATGCGA

>940_4

GCCTTGCTTGGGAGGTTGCTCCACCAGTCC

>941_4

CAAGTGGTCCTCTGGCCGCGCGACGTAGTG

>942_4

CTGAGTGGGCTCGTCCGTGGGTGGTGTCGG

>943_4

CTCGTGAAGTGCTACCAAAACGGCCTGTGA

>944_4

GATCGGGAGAATCGGTGGCATAGGTGACTC

>945_4

CGAAGGAAACTTTGTTGACTGATAGTGCGA

>946_4

GCCTCGCTTGGGAGGTTGCTCCACCAGTTG

>947_4

GATAGGGAGAATCGGCGGCATTGGTGTCTC

>948_4

GGAGTGGCTGAGGGCGGTGGATAGGTCGCG

>949_4

GATCGGGAGAATCGGGGGTATTGGTGTCTC

>950_4

GATCGGGAGAATCGGTGGCATTGCAGTCTC

>951_4

GGAGTGGCTGAGGGCGGTGGCTAGGTCGCG

>952_4

GATCAGGAGAATCGGTAGCATTGGTGTCTC

>953_4

GATCGAGAGAATCGGTGGCATCGGTGTCTC

>954_4

GATCGGGAGAATCGGTGGCATTGTTGTCTA

>955_4

CGGTCGGTCGGTAGGATAGTATTCCGTTGG

>956_4

GATAGGTAGAATCGGTGGCATTGGTGTCTC

>957_4

CTGGCCGGCACGGCCTTCTAGTCCTCGGTA

>958_4

ATGGCCGGAACGGCCTTCTAGTCCTCGGTA

>959_4

GATCTTGAGAATCGGTGGCATTGGTGTCTC

>960_4

GATCATGAGAATCGGTGGCATTGGTGTCTC

>961_4

TCAGCCGAGGGCACCAATGTTGGGATCAAT

>962_4

ACGGCAGGTGTCGCGGTGGTCTGTGTATCC

>963_4

ACGGCAGGTGTTGCGGTGGTTCGTGAATCC

>964_4

GATCGGGAGAATTGGTGGTATTGGTGTCTC

>965_4

ACACAGGTGTCGTAGTGTCCTCACAGGGTG

>966_4

GATCGGGAGAATCGGTGGCATTGGAGACTC

>967_4

CGGTGTGGGGCCATCTGTGCGGCGGATCCT

>968_4

AGTGATCGGAGATTACAAGCAGACGTTGGG

>969_4

TTAGCACAGCGGCGGACGAAGAGGAGGGGC

>970_4

GCCTCGCTTGGGAGGTTGTTCCACCAGTTC

>971_4

TGGGGCCGTGCTACCTGCACACTCCGCGGG

>972_4

TCCGGGGGCGCCCTGTGCGCGACGCAAGGG

>973_4

ACGACAAATGGAAATGCGACGTAGTGGACG

>974_4

ATGGTCGGCACGGCCTTCTAGTCCTCGGTA

>975_4

GATTGGGAGAGTCGGTGGCATTGGTGTCTC

>976_4

CGTAGGGGGCATGGGTCAGAAAGTCGCCAG

>977_4

GCCTTGCTTGGGAGGTTGCTCCACCAGATC

>978_4

ATGTCGGTAGAACAGGGTGGGGTGCTGTCC

>979_4

GATCAGGAGAATCGGTGGCATTGGTGTTTC

>980_4

GGGAGGGTGCCGGCAGCGGTGTGAATGCGC

>981_4

GTAGTGCTGGCTAACTAAGTGTGGAACTCG

>982_4

GAGTGGGAGAATCGGTGGCATTGGTGTCTC

>983_4

GAAGACCCTGGATTCGGGGACTAGTTGCTG

>984_4

GATCGGGAGACTCGGTGGCATTGGTGCCTC

>985_4

ATGGCAGGTGTTGCGGTGGTCTGTGAATCT

>986_4

GATCGGGAGAATCGGTGGCACCGGTGTCTC

>987_4

GTGTTGCCCGGTACACGAGCAGACGTTTGG

>988_4

GCCTCGCTTGGGAGGTTGCCCCACCAGTTC

>989_4

GATCGGGAGAATCTGTGGCATTGTTGTCTC

>990_4

GATCGTGAGACTCGGTGGCATTGGTGTCTC

>991_4

TCGCTGAGCGAACTGTGGTAAATAGTGGGC

>992_4

TGGGGCCGTGCCATCTGCACACTCCGCGGG

>993_4

GATTGGGAGAATCGGTGGCATAGGTGTCTC

>994_4

GGGAGGGCGCCGTCAGCGGTGTGAATGCGA

>995_4

CAGCGAAGTGCTGTGAACAGTGGTTAGCAC

>996_4

ATGGCCGGCACGGCCTTATAGTCCTCGGTA

>997_4

AGATCGCTCGTCGACTCGGGTCTGAGGAAG

>998_4

GAAGACTCTGGATTCGGGGACCAGTTCCTG

>999_4

TGGATACGGGAGGACAGTGTGCCAGAATGC

>1000_4

TATCGGGAGAATCGGTGGTATTGGTGTCTC
