# Supplementary material for: Selection of allosteric dnazymes that can sense phenylalanine by expression-SELEX
Source: Nucleic Acids Res. 2023 May 19;51(11):e66. doi: 10.1093/nar/gkad424 (PMC10287898; doi:10.1093/nar/gkad424)
Supplement: gkad424_Supplemental_Files [file gkad424_supplemental_files.zip › Supplementary file No. 6 three-top-enriched-sequences-blast_round20.docx]

**1. Using** **II-R1-1 (1_892954) to blast round_20^th^ top 1000 sequences**

>1_892954

GATCGGGAGAATCGGTGGCATTGGTGTCTC

>415_15

GATCGGGAGATTCGGTGGCATTGGTGTCTC

>312_28

GATCGGGAGAAGCGGTGGCATTGGTGTCTC

>245_40

GATCGGGAGAATCGGTGGCATTGGTGTGTC

>244_41

GATCGGGAGAATGGGTGGCATTGGTGTCTC

>228_47

GATCGGGAGCATCGGTGGCATTGGTGTCTC

>188_70

GATCGGGAGAATCCGTGGCATTGGTGTCTC

>182_75

GATCGGGAGAATCGGTCGCATTGGTGTCTC

>179_77

GATCGGGAGAATCGGTGGCTTTGGTGTCTC

>178_77

GATGGGGAGAATCGGTGGCATTGGTGTCTC

>172_83

GATCGGGAGAATCGGGGGCATTGGTGTCTC

>171_83

GATCGGGAGAATCGGTGGCATTGGTGGCTC

>160_98

GATCGGGAGAATCGGTGGCATTGGGGTCTC

>155_102

GATCGGGAGAATCGGTGGCATTGGTCTCTC

>153_105

GATCGGGAGAATCGGTGGCATGGGTGTCTC

>148_111

GATCGGGACAATCGGTGGCATTGGTGTCTC

>147_114

GATCGGGAGAATCGGTGGCATTCGTGTCTC

>143_120

GAGCGGGAGAATCGGTGGCATTGGTGTCTC

>139_132

GATCGGGAGAATCGGTGGGATTGGTGTCTC

>138_133

GATCGGGCGAATCGGTGGCATTGGTGTCTC

>132_147

GATCGGGTGAATCGGTGGCATTGGTGTCTC

>124_168

GATCGGGAGAATCGGTGGCATTGGTGTATC

>123_169

GATCGGGAGAATCGGTGGCATTGCTGTCTC

>119_177

GAACGGGAGAATCGGTGGCATTGGTGTCTC

>117_179

GATCCGGAGAATCGGTGGCATTGGTGTCTC

>113_208

GATCGGGAGAATCGGTGGCCTTGGTGTCTC

>111_213

GATCGGGAGAATCGGTGCCATTGGTGTCTC

>108_222

GATCGGGAGTATCGGTGGCATTGGTGTCTC

>105_226

GATCGGGAAAATCGGTGGCATTGGTGTCTC

>99_259

GATCGGGAGAATCGGTGGCAATGGTGTCTC

>97_272

GATCGGGAGAATCGCTGGCATTGGTGTCTC

>96_283

GATCGGGAGAATCGGTTGCATTGGTGTCTC

>95_285

GATCGGGAGAATCGGTGGCATTGGTATCTC

>94_296

GATCGGGAGAATCGATGGCATTGGTGTCTC

>93_307

GATCGGGAGAATAGGTGGCATTGGTGTCTC

>92_310

GATCGGGAGAATCGGTGGCATTGGTTTCTC

>90_327

GATCGGGAGAATCGGTGGAATTGGTGTCTC

>89_330

GATCGGGAGAATCGGTGGCATTGGTGACTC

>87_343

GATCGGGAGAATCGGTGGCATTGGAGTCTC

>84_354

GATCGGGAGAACCGGTGGCATTGGTGTCTC

>81_364

GATCGGGAGAATCGGTGGCAGTGGTGTCTC

>79_369

GATAGGGAGAATCGGTGGCATTGGTGTCTC

>78_370

GATCGGGAGAATCGGTGGCATTTGTGTCTC

>77_378

GATCGGGAGAATCGGTGACATTGGTGTCTC

>75_386

GATCGGGATAATCGGTGGCATTGGTGTCTC

>73_396

GATCGGGAGACTCGGTGGCATTGGTGTCTC

>69_427

GATCGGGAGAATCGGTGGCATTGTTGTCTC

>67_430

GATCGGGAGAAACGGTGGCATTGGTGTCTC

>63_447

GATCGGGAGAATCGGTAGCATTGGTGTCTC

>62_452

GATCGGGAGAATCGGTGGCATTGATGTCTC

>61_454

GATCGGGAGAATCGGTGGCATTAGTGTCTC

>59_481

GATCGGGAGAATCGGTGTCATTGGTGTCTC

>57_518

GACCGGGAGAATCGGTGGCATTGGTGTCTC

>55_526

GATCGGGAGAATCAGTGGCATTGGTGTCTC

>53_527

GATCGGGAGAATCTGTGGCATTGGTGTCTC

>52_534

GATCGGGAGAATCGGTGGCATAGGTGTCTC

>50_552

GATCGGGAGAATCGGAGGCATTGGTGTCTC

>49_559

GATCGGGAGAATCGTTGGCATTGGTGTCTC

>46_618

GATCGGCAGAATCGGTGGCATTGGTGTCTC

>45_629

GATCGGGAGAATCGGTGGCACTGGTGTCTC

>40_706

GATCTGGAGAATCGGTGGCATTGGTGTCTC

>35_938

GATCGGTAGAATCGGTGGCATTGGTGTCTC

>34_963

GATCGCGAGAATCGGTGGCATTGGTGTCTC

>33_1011

GATCGGGAGAATCGGTGGCGTTGGTGTCTC

>32_1013

GATCGGAAGAATCGGTGGCATTGGTGTCTC

>30_1081

GATCGGGAGAATCGGTGGTATTGGTGTCTC

>29_1104

GATCGGGAGAGTCGGTGGCATTGGTGTCTC

>26_1257

GATCGGGAGAATCGGTGGCATTGGCGTCTC

>24_1332

GATCGGGAGAATCGGTGGCATTGGTGCCTC

>22_1449

GATCGAGAGAATCGGTGGCATTGGTGTCTC

>20_1482

GATCGGGAGGATCGGTGGCATTGGTGTCTC

>18_1555

GATCAGGAGAATCGGTGGCATTGGTGTCTC

>17_1565

GATCGGGGGAATCGGTGGCATTGGTGTCTC

>15_2111

GATCGGGAGAATCGGTGGCATCGGTGTCTC

>14_2397

GATCGGGAGAATCGGTGGCATTGGTGTTTC

>12_2781

GATCGTGAGAATCGGTGGCATTGGTGTCTC

>11_3283

GATCGGGAGAATTGGTGGCATTGGTGTCTC

>6_4354

GATTGGGAGAATCGGTGGCATTGGTGTCTC

>5_7863

GATCGGGAGAATCGGCGGCATTGGTGTCTC

>993_4

GATTGGGAGAATCGGTGGCATAGGTGTCTC

>990_4

GATCGTGAGACTCGGTGGCATTGGTGTCTC

>989_4

GATCGGGAGAATCTGTGGCATTGTTGTCTC

>986_4

GATCGGGAGAATCGGTGGCACCGGTGTCTC

>984_4

GATCGGGAGACTCGGTGGCATTGGTGCCTC

>979_4

GATCAGGAGAATCGGTGGCATTGGTGTTTC

>975_4

GATTGGGAGAGTCGGTGGCATTGGTGTCTC

>966_4

GATCGGGAGAATCGGTGGCATTGGAGACTC

>964_4

GATCGGGAGAATTGGTGGTATTGGTGTCTC

>960_4

GATCATGAGAATCGGTGGCATTGGTGTCTC

>959_4

GATCTTGAGAATCGGTGGCATTGGTGTCTC

>956_4

GATAGGTAGAATCGGTGGCATTGGTGTCTC

>953_4

GATCGAGAGAATCGGTGGCATCGGTGTCTC

>952_4

GATCAGGAGAATCGGTAGCATTGGTGTCTC

>950_4

GATCGGGAGAATCGGTGGCATTGCAGTCTC

>949_4

GATCGGGAGAATCGGGGGTATTGGTGTCTC

>947_4

GATAGGGAGAATCGGCGGCATTGGTGTCTC

>944_4

GATCGGGAGAATCGGTGGCATAGGTGACTC

>936_4

GATCGGGAGAATCGGCGGAATTGGTGTCTC

>934_4

GATCGGGAGAAACGGTGGCATTGGTGACTC

>932_4

GATCGGAAGAATTGGTGGCATTGGTGTCTC

>930_4

GATCGGGAGGATCGGTGGCATTGGTGCCTC

>926_4

GATCGGGAGAATCGTTGGCATTGGAGTCTC

>923_4

GATCGTTAGAATCGGTGGCATTGGTGTCTC

>922_4

GATCGTGAGAATCGGTGGCACTGGTGTCTC

>921_4

GATCCGGAGAATCGGCGGCATTGGTGTCTC

>918_4

GATCGGGAGAATCTGTGGCATTGGTGACTC

>913_4

GATCGGTAGAATCGGTGTCATTGGTGTCTC

>908_4

GATCGGGAGAATCGGTGGAATTGTTGTCTC

>893_4

GATCGGGAGAATCGGCGGCATTGGGGTCTC

>892_4

GATCGGGAGAATCGGTGGCATAGGTGTATC

>888_4

GATCGGGAGAATCTGTGGCATTGGTTTCTC

>882_5

GATCGGGAGAGTTGGTGGCATTGGTGTCTC

>880_5

GATCGTGAGAGTCGGTGGCATTGGTGTCTC

>873_5

GATCGCGAGAATTGGTGGCATTGGTGTCTC

>857_5

GATCGGGAGAATCGGTGGACTTGGTGTCTC

>856_5

GATCGGGAGAAACGGTGGCATTGGAGTCTC

>852_5

GATCGGGAGAATCGGTGGCATCGGTGTTTC

>850_5

GATTGGGAGAATCGGTGGCATTGGCGTCTC

>848_5

GATCGGAAGAATCGGTGGCATCGGTGTCTC

>839_5

GATCGAGAGAAACGGTGGCATTGGTGTCTC

>836_5

GATCGGGAGAATCGGCGGCGTTGGTGTCTC

>835_5

GATCGGGAGAATCGGCGGCAGTGGTGTCTC

>829_5

GATCGGGAGAATCGGCGGCATTTGTGTCTC

>826_5

GATCGGGAGAATCGGCTGCATTGGTGTCTC

>823_5

GATTGGAAGAATCGGTGGCATTGGTGTCTC

>822_5

GATCTGGGGAATCGGTGGCATTGGTGTCTC

>820_5

GATTGGGAGGATCGGTGGCATTGGTGTCTC

>813_5

GATCGGGAGAATCAGCGGCATTGGTGTCTC

>809_5

GATCTGGAGAATCGGTGGCATTTGTGTCTC

>808_5

GATCGGGAGAATCGGTGGCAATGGAGTCTC

>806_5

GATCGGGAGAATCGGCGACATTGGTGTCTC

>803_5

GATCGCGAGAATCGGCGGCATTGGTGTCTC

>798_5

GATCTGGAGAATCTGTGGCATTGGTGTCTC

>795_5

GATTGGTAGAATCGGTGGCATTGGTGTCTC

>793_5

GATCGGGAGAATCTGTGGCATAGGTGTCTC

>789_5

GATTGGGAGAATCGGTGGCATTAGTGTCTC

>783_5

GATCGGGAGAATCGGTGGAATTGGTGACTC

>779_5

GATCGGGGGAATTGGTGGCATTGGTGTCTC

>777_5

GATCGGTAGAATCGGTGGCATTGGTGTTTC

>775_5

GATCAGAAGAATCGGTGGCATTGGTGTCTC

>773_5

GATCGGGGGACTCGGTGGCATTGGTGTCTC

>771_5

GATCGTGAGAATCGGTGGTATTGGTGTCTC

>770_5

GATCGGGAGAATCGGCGGCATAGGTGTCTC

>768_5

GATCGCGAGAATCGGTGGCATTGGTGTTTC

>767_5

GATCGGGAGAATCTGTGGCATTGGTGTTTC

>764_5

GATTGGGAGAATCGGTGACATTGGTGTCTC

>761_5

GATCGGGAGAATCGGTGTCATTGTTGTCTC

>758_5

GATCGTGAGAATCGGTGGCATTGGTGCCTC

>754_6

GATCAGGAGAATCGGTGGCATCGGTGTCTC

>752_6

GATCGGAAGAATCGGTGGCATTGGTGCCTC

>751_6

GATCGGGAGAATCGGTGGCATAGGAGTCTC

>747_6

GATTGGGAGAATCGGTGGCATTGGTGTTTC

>745_6

GATCGGGAGAATCGGTGGCATTAATGTCTC

>740_6

GATCGGGAGAATCGGAGGCATAGGTGTCTC

>738_6

GATCGTGAGAATCGGTGGCGTTGGTGTCTC

>737_6

GATCGGGAGAATTGGTGGCATTGGTGTTTC

>728_6

GATCGAGAGAATTGGTGGCATTGGTGTCTC

>727_6

GATCGGGAGGATTGGTGGCATTGGTGTCTC

>723_6

GATTGGGAGAATCGGAGGCATTGGTGTCTC

>720_6

GATCGGGAGAATCGGATGCATTGGTGTCTC

>718_6

GATCGGGAGAAACGGAGGCATTGGTGTCTC

>717_6

GATCGGGAGAATCGTAGGCATTGGTGTCTC

>705_6

GATCGGTAGAATCGGTGGCATTGGAGTCTC

>702_6

GATCGAGAGAATCGGTGGCGTTGGTGTCTC

>698_6

GATTGGGAGAATCGGTGGCACTGGTGTCTC

>686_6

GATCGAGAGGATCGGTGGCATTGGTGTCTC

>682_6

GATCGTGAGAATCGGTGTCATTGGTGTCTC

>681_6

GATCGGGAGAATTGGTGGCATTGATGTCTC

>677_6

GATCGGGAGAATTGGTGGCATTGGTGCCTC

>672_6

GATCGGGAGGATCGGTGGTATTGGTGTCTC

>663_7

GATCGGGAGAATCGGCGGCATTGGCGTCTC

>649_7

GATCGTGAGAATCGGTGGCATTGGCGTCTC

>640_7

GATTGGGAGAATCGGTGGCATTGGTGCCTC

>637_7

GATCGGGAGAATCGGCGTCATTGGTGTCTC

>625_7

GATCGGGAGAAACGGTGGCATAGGTGTCTC

>624_7

GATCGTGAGAATCGGTGGCATAGGTGTCTC

>621_7

GATCGTGAGGATCGGTGGCATTGGTGTCTC

>618_7

GATCAGGAGAATTGGTGGCATTGGTGTCTC

>610_7

GATCGGAGGAATCGGTGGCATTGGTGTCTC

>594_8

GATCGGGAGAATCGTCGGCATTGGTGTCTC

>572_8

GATTGGGAGAATCGGTGGTATTGGTGTCTC

>571_8

GATCGTAAGAATCGGTGGCATTGGTGTCTC

>563_9

GATCGTGAGAATCGGTGGCATCGGTGTCTC

>558_9

GATAGGGAGAATCGGTGGCATTTGTGTCTC

>545_9

GATTGGGAGAATCGGTGGCATCGGTGTCTC

>540_10

GATCGGGAGACTTGGTGGCATTGGTGTCTC

>535_10

GATCGGGAGAATTGGTGGCATCGGTGTCTC

>506_11

GATCGGGAGGATCGGCGGCATTGGTGTCTC

>500_11

GATCGGGAGAATCAATGGCATTGGTGTCTC

>494_11

GATCGGTAGAATCGGCGGCATTGGTGTCTC

>461_13

GATCTGGAGAATCGGCGGCATTGGTGTCTC

>446_14

GATCGAGAGAATCGGCGGCATTGGTGTCTC

>444_14

GATCGTGAGAATCGGTGGCATTGGTGTTTC

>429_15

GATCGGGAGAATCGGTAACATTGGTGTCTC

>428_15

GATCGTGAGAATTGGTGGCATTGGTGTCTC

>413_15

GATTGGGAGAATTGGTGGCATTGGTGTCTC

>389_17

GATCGGGAGAATCGGCGGCATTGGTGCCTC

>375_18

GATTGTGAGAATCGGTGGCATTGGTGTCTC

>369_19

GATCGGGAGAATCGGCGGTATTGGTGTCTC

>367_19

GATCGGGAGAGTCGGCGGCATTGGTGTCTC

>357_21

GATCGGGAGAATCGGCGGCATCGGTGTCTC

>350_21

GATCGGGGGAATCGGCGGCATTGGTGTCTC

>333_24

GATCAAGAGAATCGGTGGCATTGGTGTCTC

>323_26

GATCGGGAGAATCGGCGGCATTGGTGTTTC

>309_28

GATCGGGAGAATTGGCGGCATTGGTGTCTC

>291_32

GATCGTGAGAATCGGCGGCATTGGTGTCTC

>272_34

GATCAGGAGAATCGGCGGCATTGGTGTCTC

>268_35

GATCGAAAGAATCGGTGGCATTGGTGTCTC

>225_48

GATTGGGAGAATCGGCGGCATTGGTGTCTC

**2. Using II-R1-3 (****3_57642) to blast round_20^th^ top 1000 sequences**

>3_57642

GAAGACTCTGGATTCGGGGACCAGTTGCTG

>998_4

GAAGACTCTGGATTCGGGGACCAGTTCCTG

>929_4

GAAGACTCTGGATTCGGGGAGCAGTTGCTG

>825_5

GAAGACTCTGGATTCGGGGACCACTTGCTG

>821_5

GAAGACTCTGGATTCGGGGACCAGTTGGTG

>816_5

GAAGCCTCTGGATTCGGGGACCAGTTGCTG

>814_5

GAAGACTCTGGATTCGGGGACCCGTTGCTG

>750_6

GAACACTCTGGATTCGGGGACCAGTTGCTG

>744_6

GAAGAGTCTGGATTCGGGGACCAGTTGCTG

>714_6

GAAGACTCTGGATTCGGGGTCCAGTTGCTG

>685_6

GAAGACTCTGGATTCGGGGACGAGTTGCTG

>652_7

GAAGACTCTGGATTCGGGGACCAGGTGCTG

>570_8

GAAGACTCTGGATTCGGGGCCCAGTTGCTG

>546_9

GAAGACTCTGGATTCGGCGACCAGTTGCTG

>536_10

GAAGACTCTGGCTTCGGGGACCAGTTGCTG

>525_10

GAAGTCTCTGGATTCGGGGACCAGTTGCTG

>521_10

GAAGACTCTGGTTTCGGGGACCAGTTGCTG

>513_10

GAAGACTCTGGATTCCGGGACCAGTTGCTG

>504_11

GAAGACTCTGGATTCGCGGACCAGTTGCTG

>502_11

GATGACTCTGGATTCGGGGACCAGTTGCTG

>482_12

GAAGACTCTGGATTCGGGCACCAGTTGCTG

>481_12

GAAGACTCTGGATTCGGGGACCTGTTGCTG

>445_14

GACGACTCTGGATTCGGGGACCAGTTGCTG

>441_14

GAAGACTCTGGATACGGGGACCAGTTGCTG

>418_15

GAAGACTCTGGATTAGGGGACCAGTTGCTG

>406_15

GAAGACTCTGGATTCGGGGACCAGTTACTG

>400_16

GAAGACTCGGGATTCGGGGACCAGTTGCTG

>393_16

GAAGACTCTGGATTCGGGGAACAGTTGCTG

>372_18

GAAGACTCTTGATTCGGGGACCAGTTGCTG

>365_19

GAAGACTCTGGAATCGGGGACCAGTTGCTG

>352_21

GAAGACTCTGGATTCGGGGACCAATTGCTG

>337_23

GAAGAATCTGGATTCGGGGACCAGTTGCTG

>327_25

GAAGACTCTGGATTCGGAGACCAGTTGCTG

>324_26

GAAGACTCTGGATTCGGGAACCAGTTGCTG

>317_27

GAAGACTCTGGATTCGGGGACCATTTGCTG

>314_27

GAAGACTCTGGATTCGGGGACCAGTTGATG

>311_28

GAAGACTCTGGATTCGGGGACAAGTTGCTG

>305_29

GAAGACTCTGGATTCGGGGACCAGTTTCTG

>301_29

GAAGACTCTGGATTCGAGGACCAGTTGCTG

>296_31

GAAGACTCTGGATCCGGGGACCAGTTGCTG

>293_31

GAAGACTCTGGATTCGGGGACCAGTAGCTG

>288_32

GAAGACTCTGGAGTCGGGGACCAGTTGCTG

>283_33

GAAGACTCTGGATTCTGGGACCAGTTGCTG

>282_33

GAAGACTCTGGATTCGGTGACCAGTTGCTG

>279_34

GAAGACTGTGGATTCGGGGACCAGTTGCTG

>274_34

GAAAACTCTGGATTCGGGGACCAGTTGCTG

>273_34

GAAGACTCTAGATTCGGGGACCAGTTGCTG

>262_36

GAAGACTCTGGATTCGGGGACCAGATGCTG

>254_37

GAAGACGCTGGATTCGGGGACCAGTTGCTG

>251_39

GAAGACTCTGGATTCGTGGACCAGTTGCTG

>246_40

GAAGACACTGGATTCGGGGACCAGTTGCTG

>242_42

GAAGACTCTGAATTCGGGGACCAGTTGCTG

>241_42

GAAGACTCTGGATTCAGGGACCAGTTGCTG

>224_49

GAGGACTCTGGATTCGGGGACCAGTTGCTG

>217_52

GAAGACTCTGGATTCGGGGACCAGTCGCTG

>216_52

GAAGACTATGGATTCGGGGACCAGTTGCTG

>212_55

GAAGACTCTGGATTCGGGTACCAGTTGCTG

>208_56

GAAGACTCTGGATTCGGGGACCAGCTGCTG

>194_65

GAAGACTCTGGACTCGGGGACCAGTTGCTG

>187_71

GAAGACTCTGGATTCGGGGACCGGTTGCTG

>180_76

GAAGGCTCTGGATTCGGGGACCAGTTGCTG

>165_88

GAAGACTCTGGGTTCGGGGACCAGTTGCTG

>163_91

GAATACTCTGGATTCGGGGACCAGTTGCTG

>156_102

GAAGACTCTGGATTCGGGGGCCAGTTGCTG

>142_125

GAAGACTCTGGATTCGGGGATCAGTTGCTG

>141_127

GAAGATTCTGGATTCGGGGACCAGTTGCTG

>120_170

GAAGACTCAGGATTCGGGGACCAGTTGCTG

>116_185

GAAGACTCTGGATTCGGGGACCAGTTGTTG

>109_220

GAAGACTCTGGATTTGGGGACCAGTTGCTG

>101_252

GAAGACTCTGTATTCGGGGACCAGTTGCTG

>100_254

GAAGACCCTGGATTCGGGGACCAGTTGCTG

>83_354

GAAGACTCTGGATTCGGGGACTAGTTGCTG

>56_520

GAAGACTCCGGATTCGGGGACCAGTTGCTG

>37_907

GAAGACTTTGGATTCGGGGACCAGTTGCTG

>983_4

GAAGACCCTGGATTCGGGGACTAGTTGCTG

>899_4

GAAGACTCCGGATTCGGGGACCAGTTACTG

>884_4

GAAGACTTTGGATTCGGGGATCAGTTGCTG

>734_6

GAAGACTTTGGATTTGGGGACCAGTTGCTG

>582_8

GAAGACTTTGGATTCGGGGACTAGTTGCTG

>501_11

GAAGACTTCGGATTCGGGGACCAGTTGCTG

>411_15

GAAGACCTTGGATTCGGGGACCAGTTGCTG

>237_44

GAAGACTCTGGATTCGGGGATTAGTTGCTG

**3. Using II-R1-7 (****7_3906) to blast round_20^th^ top 1000 sequences**

>7_3906

GATCGGGAGAATCGGTGGCATTGGTGTCTT

>905_4

GATCGGGAGAATCGGTGGCATTGGTGCCTT

>872_5

GATCGGCAGAATCGGTGGCATTGGTGTCTT

>845_5

GATCGGGAGAATCGGTGGTATTGGTGTCTT

>778_5

GATCGGAAGAATCGGTGGCATTGGTGTCTT

>776_5

GATCGGGAGAGTCGGTGGCATTGGTGTCTT

>703_6

GATCGGGGGAATCGGTGGCATTGGTGTCTT

>673_6

GATCGGGAGAATCGGTGGCATAGGTGTCTT

>661_7

GATCGCGAGAATCGGTGGCATTGGTGTCTT

>648_7

GATCAGGAGAATCGGTGGCATTGGTGTCTT

>601_8

GATCGGGAGAATCGGTGGCATCGGTGTCTT

>544_9

GATCGGGAGAATTGGTGGCATTGGTGTCTT

>511_11

GATCGGTAGAATCGGTGGCATTGGTGTCTT

>510_11

GATCGAGAGAATCGGTGGCATTGGTGTCTT

>503_11

GATCGGGAGGATCGGTGGCATTGGTGTCTT

>448_14

GATCGTGAGAATCGGTGGCATTGGTGTCTT

>404_15

GATCGGGAGAATCGGTGGCATTGGTGTTTT

>341_23

GATTGGGAGAATCGGTGGCATTGGTGTCTT

>297_30

GATCGGGAGAATCGGCGGCATTGGTGTCTT
