# Supplementary material for: Selection of allosteric dnazymes that can sense phenylalanine by expression-SELEX
Source: Nucleic Acids Res. 2023 May 19;51(11):e66. doi: 10.1093/nar/gkad424 (PMC10287898; doi:10.1093/nar/gkad424)
Supplement: gkad424_Supplemental_Files [file gkad424_supplemental_files.zip › Supplementary file No. 7 three-top-enriched-sequences-blast_round20_full_length.docx]

**1. Using II-R1-1 (1_892954) to blast round_20^th^ top 1000 full_length sequences**

>1_892954

CATGACCACTAGGAGCATCTTTGGCGAGATCGGGAGAATCGGTGGCATTGGTGTCTCCTAGGGGAATAAATCTTTGGGCACCTAGTGGTCATG

>415_15

CATGACCACTAGGAGCATCTTTGGCGAGATCGGGAGATTCGGTGGCATTGGTGTCTCCTAGGGGAATAAATCTTTGGGCACCTAGTGGTCATG

>312_28

CATGACCACTAGGAGCATCTTTGGCGAGATCGGGAGAAGCGGTGGCATTGGTGTCTCCTAGGGGAATAAATCTTTGGGCACCTAGTGGTCATG

>245_40

CATGACCACTAGGAGCATCTTTGGCGAGATCGGGAGAATCGGTGGCATTGGTGTGTCCTAGGGGAATAAATCTTTGGGCACCTAGTGGTCATG

>244_41

CATGACCACTAGGAGCATCTTTGGCGAGATCGGGAGAATGGGTGGCATTGGTGTCTCCTAGGGGAATAAATCTTTGGGCACCTAGTGGTCATG

>228_47

CATGACCACTAGGAGCATCTTTGGCGAGATCGGGAGCATCGGTGGCATTGGTGTCTCCTAGGGGAATAAATCTTTGGGCACCTAGTGGTCATG

>188_70

CATGACCACTAGGAGCATCTTTGGCGAGATCGGGAGAATCCGTGGCATTGGTGTCTCCTAGGGGAATAAATCTTTGGGCACCTAGTGGTCATG

>182_75

CATGACCACTAGGAGCATCTTTGGCGAGATCGGGAGAATCGGTCGCATTGGTGTCTCCTAGGGGAATAAATCTTTGGGCACCTAGTGGTCATG

>179_77

CATGACCACTAGGAGCATCTTTGGCGAGATCGGGAGAATCGGTGGCTTTGGTGTCTCCTAGGGGAATAAATCTTTGGGCACCTAGTGGTCATG

>178_77

CATGACCACTAGGAGCATCTTTGGCGAGATGGGGAGAATCGGTGGCATTGGTGTCTCCTAGGGGAATAAATCTTTGGGCACCTAGTGGTCATG

>172_83

CATGACCACTAGGAGCATCTTTGGCGAGATCGGGAGAATCGGGGGCATTGGTGTCTCCTAGGGGAATAAATCTTTGGGCACCTAGTGGTCATG

>171_83

CATGACCACTAGGAGCATCTTTGGCGAGATCGGGAGAATCGGTGGCATTGGTGGCTCCTAGGGGAATAAATCTTTGGGCACCTAGTGGTCATG

>160_98

CATGACCACTAGGAGCATCTTTGGCGAGATCGGGAGAATCGGTGGCATTGGGGTCTCCTAGGGGAATAAATCTTTGGGCACCTAGTGGTCATG

>155_102

CATGACCACTAGGAGCATCTTTGGCGAGATCGGGAGAATCGGTGGCATTGGTCTCTCCTAGGGGAATAAATCTTTGGGCACCTAGTGGTCATG

>153_105

CATGACCACTAGGAGCATCTTTGGCGAGATCGGGAGAATCGGTGGCATGGGTGTCTCCTAGGGGAATAAATCTTTGGGCACCTAGTGGTCATG

>148_111

CATGACCACTAGGAGCATCTTTGGCGAGATCGGGACAATCGGTGGCATTGGTGTCTCCTAGGGGAATAAATCTTTGGGCACCTAGTGGTCATG

>147_114

CATGACCACTAGGAGCATCTTTGGCGAGATCGGGAGAATCGGTGGCATTCGTGTCTCCTAGGGGAATAAATCTTTGGGCACCTAGTGGTCATG

>143_120

CATGACCACTAGGAGCATCTTTGGCGAGAGCGGGAGAATCGGTGGCATTGGTGTCTCCTAGGGGAATAAATCTTTGGGCACCTAGTGGTCATG

>139_132

CATGACCACTAGGAGCATCTTTGGCGAGATCGGGAGAATCGGTGGGATTGGTGTCTCCTAGGGGAATAAATCTTTGGGCACCTAGTGGTCATG

>138_133

CATGACCACTAGGAGCATCTTTGGCGAGATCGGGCGAATCGGTGGCATTGGTGTCTCCTAGGGGAATAAATCTTTGGGCACCTAGTGGTCATG

>132_147

CATGACCACTAGGAGCATCTTTGGCGAGATCGGGTGAATCGGTGGCATTGGTGTCTCCTAGGGGAATAAATCTTTGGGCACCTAGTGGTCATG

>124_168

CATGACCACTAGGAGCATCTTTGGCGAGATCGGGAGAATCGGTGGCATTGGTGTATCCTAGGGGAATAAATCTTTGGGCACCTAGTGGTCATG

>123_169

CATGACCACTAGGAGCATCTTTGGCGAGATCGGGAGAATCGGTGGCATTGCTGTCTCCTAGGGGAATAAATCTTTGGGCACCTAGTGGTCATG

>119_177

CATGACCACTAGGAGCATCTTTGGCGAGAACGGGAGAATCGGTGGCATTGGTGTCTCCTAGGGGAATAAATCTTTGGGCACCTAGTGGTCATG

>117_179

CATGACCACTAGGAGCATCTTTGGCGAGATCCGGAGAATCGGTGGCATTGGTGTCTCCTAGGGGAATAAATCTTTGGGCACCTAGTGGTCATG

>113_208

CATGACCACTAGGAGCATCTTTGGCGAGATCGGGAGAATCGGTGGCCTTGGTGTCTCCTAGGGGAATAAATCTTTGGGCACCTAGTGGTCATG

>111_213

CATGACCACTAGGAGCATCTTTGGCGAGATCGGGAGAATCGGTGCCATTGGTGTCTCCTAGGGGAATAAATCTTTGGGCACCTAGTGGTCATG

>108_222

CATGACCACTAGGAGCATCTTTGGCGAGATCGGGAGTATCGGTGGCATTGGTGTCTCCTAGGGGAATAAATCTTTGGGCACCTAGTGGTCATG

>105_226

CATGACCACTAGGAGCATCTTTGGCGAGATCGGGAAAATCGGTGGCATTGGTGTCTCCTAGGGGAATAAATCTTTGGGCACCTAGTGGTCATG

>99_259

CATGACCACTAGGAGCATCTTTGGCGAGATCGGGAGAATCGGTGGCAATGGTGTCTCCTAGGGGAATAAATCTTTGGGCACCTAGTGGTCATG

>97_272

CATGACCACTAGGAGCATCTTTGGCGAGATCGGGAGAATCGCTGGCATTGGTGTCTCCTAGGGGAATAAATCTTTGGGCACCTAGTGGTCATG

>96_283

CATGACCACTAGGAGCATCTTTGGCGAGATCGGGAGAATCGGTTGCATTGGTGTCTCCTAGGGGAATAAATCTTTGGGCACCTAGTGGTCATG

>95_285

CATGACCACTAGGAGCATCTTTGGCGAGATCGGGAGAATCGGTGGCATTGGTATCTCCTAGGGGAATAAATCTTTGGGCACCTAGTGGTCATG

>94_296

CATGACCACTAGGAGCATCTTTGGCGAGATCGGGAGAATCGATGGCATTGGTGTCTCCTAGGGGAATAAATCTTTGGGCACCTAGTGGTCATG

>93_307

CATGACCACTAGGAGCATCTTTGGCGAGATCGGGAGAATAGGTGGCATTGGTGTCTCCTAGGGGAATAAATCTTTGGGCACCTAGTGGTCATG

>92_310

CATGACCACTAGGAGCATCTTTGGCGAGATCGGGAGAATCGGTGGCATTGGTTTCTCCTAGGGGAATAAATCTTTGGGCACCTAGTGGTCATG

>90_327

CATGACCACTAGGAGCATCTTTGGCGAGATCGGGAGAATCGGTGGAATTGGTGTCTCCTAGGGGAATAAATCTTTGGGCACCTAGTGGTCATG

>89_330

CATGACCACTAGGAGCATCTTTGGCGAGATCGGGAGAATCGGTGGCATTGGTGACTCCTAGGGGAATAAATCTTTGGGCACCTAGTGGTCATG

>87_343

CATGACCACTAGGAGCATCTTTGGCGAGATCGGGAGAATCGGTGGCATTGGAGTCTCCTAGGGGAATAAATCTTTGGGCACCTAGTGGTCATG

>84_354

CATGACCACTAGGAGCATCTTTGGCGAGATCGGGAGAACCGGTGGCATTGGTGTCTCCTAGGGGAATAAATCTTTGGGCACCTAGTGGTCATG

>81_364

CATGACCACTAGGAGCATCTTTGGCGAGATCGGGAGAATCGGTGGCAGTGGTGTCTCCTAGGGGAATAAATCTTTGGGCACCTAGTGGTCATG

>79_369

CATGACCACTAGGAGCATCTTTGGCGAGATAGGGAGAATCGGTGGCATTGGTGTCTCCTAGGGGAATAAATCTTTGGGCACCTAGTGGTCATG

>78_370

CATGACCACTAGGAGCATCTTTGGCGAGATCGGGAGAATCGGTGGCATTTGTGTCTCCTAGGGGAATAAATCTTTGGGCACCTAGTGGTCATG

>77_378

CATGACCACTAGGAGCATCTTTGGCGAGATCGGGAGAATCGGTGACATTGGTGTCTCCTAGGGGAATAAATCTTTGGGCACCTAGTGGTCATG

>75_386

CATGACCACTAGGAGCATCTTTGGCGAGATCGGGATAATCGGTGGCATTGGTGTCTCCTAGGGGAATAAATCTTTGGGCACCTAGTGGTCATG

>73_396

CATGACCACTAGGAGCATCTTTGGCGAGATCGGGAGACTCGGTGGCATTGGTGTCTCCTAGGGGAATAAATCTTTGGGCACCTAGTGGTCATG

>69_427

CATGACCACTAGGAGCATCTTTGGCGAGATCGGGAGAATCGGTGGCATTGTTGTCTCCTAGGGGAATAAATCTTTGGGCACCTAGTGGTCATG

>67_430

CATGACCACTAGGAGCATCTTTGGCGAGATCGGGAGAAACGGTGGCATTGGTGTCTCCTAGGGGAATAAATCTTTGGGCACCTAGTGGTCATG

>63_447

CATGACCACTAGGAGCATCTTTGGCGAGATCGGGAGAATCGGTAGCATTGGTGTCTCCTAGGGGAATAAATCTTTGGGCACCTAGTGGTCATG

>62_452

CATGACCACTAGGAGCATCTTTGGCGAGATCGGGAGAATCGGTGGCATTGATGTCTCCTAGGGGAATAAATCTTTGGGCACCTAGTGGTCATG

>61_454

CATGACCACTAGGAGCATCTTTGGCGAGATCGGGAGAATCGGTGGCATTAGTGTCTCCTAGGGGAATAAATCTTTGGGCACCTAGTGGTCATG

>59_481

CATGACCACTAGGAGCATCTTTGGCGAGATCGGGAGAATCGGTGTCATTGGTGTCTCCTAGGGGAATAAATCTTTGGGCACCTAGTGGTCATG

>57_518

CATGACCACTAGGAGCATCTTTGGCGAGACCGGGAGAATCGGTGGCATTGGTGTCTCCTAGGGGAATAAATCTTTGGGCACCTAGTGGTCATG

>55_526

CATGACCACTAGGAGCATCTTTGGCGAGATCGGGAGAATCAGTGGCATTGGTGTCTCCTAGGGGAATAAATCTTTGGGCACCTAGTGGTCATG

>53_527

CATGACCACTAGGAGCATCTTTGGCGAGATCGGGAGAATCTGTGGCATTGGTGTCTCCTAGGGGAATAAATCTTTGGGCACCTAGTGGTCATG

>52_534

CATGACCACTAGGAGCATCTTTGGCGAGATCGGGAGAATCGGTGGCATAGGTGTCTCCTAGGGGAATAAATCTTTGGGCACCTAGTGGTCATG

>50_552

CATGACCACTAGGAGCATCTTTGGCGAGATCGGGAGAATCGGAGGCATTGGTGTCTCCTAGGGGAATAAATCTTTGGGCACCTAGTGGTCATG

>49_559

CATGACCACTAGGAGCATCTTTGGCGAGATCGGGAGAATCGTTGGCATTGGTGTCTCCTAGGGGAATAAATCTTTGGGCACCTAGTGGTCATG

>46_618

CATGACCACTAGGAGCATCTTTGGCGAGATCGGCAGAATCGGTGGCATTGGTGTCTCCTAGGGGAATAAATCTTTGGGCACCTAGTGGTCATG

>45_629

CATGACCACTAGGAGCATCTTTGGCGAGATCGGGAGAATCGGTGGCACTGGTGTCTCCTAGGGGAATAAATCTTTGGGCACCTAGTGGTCATG

>40_706

CATGACCACTAGGAGCATCTTTGGCGAGATCTGGAGAATCGGTGGCATTGGTGTCTCCTAGGGGAATAAATCTTTGGGCACCTAGTGGTCATG

>35_938

CATGACCACTAGGAGCATCTTTGGCGAGATCGGTAGAATCGGTGGCATTGGTGTCTCCTAGGGGAATAAATCTTTGGGCACCTAGTGGTCATG

>34_963

CATGACCACTAGGAGCATCTTTGGCGAGATCGCGAGAATCGGTGGCATTGGTGTCTCCTAGGGGAATAAATCTTTGGGCACCTAGTGGTCATG

>33_1011

CATGACCACTAGGAGCATCTTTGGCGAGATCGGGAGAATCGGTGGCGTTGGTGTCTCCTAGGGGAATAAATCTTTGGGCACCTAGTGGTCATG

>32_1013

CATGACCACTAGGAGCATCTTTGGCGAGATCGGAAGAATCGGTGGCATTGGTGTCTCCTAGGGGAATAAATCTTTGGGCACCTAGTGGTCATG

>30_1081

CATGACCACTAGGAGCATCTTTGGCGAGATCGGGAGAATCGGTGGTATTGGTGTCTCCTAGGGGAATAAATCTTTGGGCACCTAGTGGTCATG

>29_1104

CATGACCACTAGGAGCATCTTTGGCGAGATCGGGAGAGTCGGTGGCATTGGTGTCTCCTAGGGGAATAAATCTTTGGGCACCTAGTGGTCATG

>26_1257

CATGACCACTAGGAGCATCTTTGGCGAGATCGGGAGAATCGGTGGCATTGGCGTCTCCTAGGGGAATAAATCTTTGGGCACCTAGTGGTCATG

>24_1332

CATGACCACTAGGAGCATCTTTGGCGAGATCGGGAGAATCGGTGGCATTGGTGCCTCCTAGGGGAATAAATCTTTGGGCACCTAGTGGTCATG

>22_1449

CATGACCACTAGGAGCATCTTTGGCGAGATCGAGAGAATCGGTGGCATTGGTGTCTCCTAGGGGAATAAATCTTTGGGCACCTAGTGGTCATG

>20_1482

CATGACCACTAGGAGCATCTTTGGCGAGATCGGGAGGATCGGTGGCATTGGTGTCTCCTAGGGGAATAAATCTTTGGGCACCTAGTGGTCATG

>18_1555

CATGACCACTAGGAGCATCTTTGGCGAGATCAGGAGAATCGGTGGCATTGGTGTCTCCTAGGGGAATAAATCTTTGGGCACCTAGTGGTCATG

>17_1565

CATGACCACTAGGAGCATCTTTGGCGAGATCGGGGGAATCGGTGGCATTGGTGTCTCCTAGGGGAATAAATCTTTGGGCACCTAGTGGTCATG

>15_2111

CATGACCACTAGGAGCATCTTTGGCGAGATCGGGAGAATCGGTGGCATCGGTGTCTCCTAGGGGAATAAATCTTTGGGCACCTAGTGGTCATG

>14_2397

CATGACCACTAGGAGCATCTTTGGCGAGATCGGGAGAATCGGTGGCATTGGTGTTTCCTAGGGGAATAAATCTTTGGGCACCTAGTGGTCATG

>12_2781

CATGACCACTAGGAGCATCTTTGGCGAGATCGTGAGAATCGGTGGCATTGGTGTCTCCTAGGGGAATAAATCTTTGGGCACCTAGTGGTCATG

>11_3283

CATGACCACTAGGAGCATCTTTGGCGAGATCGGGAGAATTGGTGGCATTGGTGTCTCCTAGGGGAATAAATCTTTGGGCACCTAGTGGTCATG

>6_4354

CATGACCACTAGGAGCATCTTTGGCGAGATTGGGAGAATCGGTGGCATTGGTGTCTCCTAGGGGAATAAATCTTTGGGCACCTAGTGGTCATG

>5_7863

CATGACCACTAGGAGCATCTTTGGCGAGATCGGGAGAATCGGCGGCATTGGTGTCTCCTAGGGGAATAAATCTTTGGGCACCTAGTGGTCATG

>993_4

CATGACCACTAGGAGCATCTTTGGCGAGATTGGGAGAATCGGTGGCATAGGTGTCTCCTAGGGGAATAAATCTTTGGGCACCTAGTGGTCATG

>990_4

CATGACCACTAGGAGCATCTTTGGCGAGATCGTGAGACTCGGTGGCATTGGTGTCTCCTAGGGGAATAAATCTTTGGGCACCTAGTGGTCATG

>989_4

CATGACCACTAGGAGCATCTTTGGCGAGATCGGGAGAATCTGTGGCATTGTTGTCTCCTAGGGGAATAAATCTTTGGGCACCTAGTGGTCATG

>986_4

CATGACCACTAGGAGCATCTTTGGCGAGATCGGGAGAATCGGTGGCACCGGTGTCTCCTAGGGGAATAAATCTTTGGGCACCTAGTGGTCATG

>984_4

CATGACCACTAGGAGCATCTTTGGCGAGATCGGGAGACTCGGTGGCATTGGTGCCTCCTAGGGGAATAAATCTTTGGGCACCTAGTGGTCATG

>979_4

CATGACCACTAGGAGCATCTTTGGCGAGATCAGGAGAATCGGTGGCATTGGTGTTTCCTAGGGGAATAAATCTTTGGGCACCTAGTGGTCATG

>975_4

CATGACCACTAGGAGCATCTTTGGCGAGATTGGGAGAGTCGGTGGCATTGGTGTCTCCTAGGGGAATAAATCTTTGGGCACCTAGTGGTCATG

>966_4

CATGACCACTAGGAGCATCTTTGGCGAGATCGGGAGAATCGGTGGCATTGGAGACTCCTAGGGGAATAAATCTTTGGGCACCTAGTGGTCATG

>964_4

CATGACCACTAGGAGCATCTTTGGCGAGATCGGGAGAATTGGTGGTATTGGTGTCTCCTAGGGGAATAAATCTTTGGGCACCTAGTGGTCATG

>960_4

CATGACCACTAGGAGCATCTTTGGCGAGATCATGAGAATCGGTGGCATTGGTGTCTCCTAGGGGAATAAATCTTTGGGCACCTAGTGGTCATG

>959_4

CATGACCACTAGGAGCATCTTTGGCGAGATCTTGAGAATCGGTGGCATTGGTGTCTCCTAGGGGAATAAATCTTTGGGCACCTAGTGGTCATG

>956_4

CATGACCACTAGGAGCATCTTTGGCGAGATAGGTAGAATCGGTGGCATTGGTGTCTCCTAGGGGAATAAATCTTTGGGCACCTAGTGGTCATG

>953_4

CATGACCACTAGGAGCATCTTTGGCGAGATCGAGAGAATCGGTGGCATCGGTGTCTCCTAGGGGAATAAATCTTTGGGCACCTAGTGGTCATG

>952_4

CATGACCACTAGGAGCATCTTTGGCGAGATCAGGAGAATCGGTAGCATTGGTGTCTCCTAGGGGAATAAATCTTTGGGCACCTAGTGGTCATG

>950_4

CATGACCACTAGGAGCATCTTTGGCGAGATCGGGAGAATCGGTGGCATTGCAGTCTCCTAGGGGAATAAATCTTTGGGCACCTAGTGGTCATG

>949_4

CATGACCACTAGGAGCATCTTTGGCGAGATCGGGAGAATCGGGGGTATTGGTGTCTCCTAGGGGAATAAATCTTTGGGCACCTAGTGGTCATG

>947_4

CATGACCACTAGGAGCATCTTTGGCGAGATAGGGAGAATCGGCGGCATTGGTGTCTCCTAGGGGAATAAATCTTTGGGCACCTAGTGGTCATG

>944_4

CATGACCACTAGGAGCATCTTTGGCGAGATCGGGAGAATCGGTGGCATAGGTGACTCCTAGGGGAATAAATCTTTGGGCACCTAGTGGTCATG

>936_4

CATGACCACTAGGAGCATCTTTGGCGAGATCGGGAGAATCGGCGGAATTGGTGTCTCCTAGGGGAATAAATCTTTGGGCACCTAGTGGTCATG

>934_4

CATGACCACTAGGAGCATCTTTGGCGAGATCGGGAGAAACGGTGGCATTGGTGACTCCTAGGGGAATAAATCTTTGGGCACCTAGTGGTCATG

>932_4

CATGACCACTAGGAGCATCTTTGGCGAGATCGGAAGAATTGGTGGCATTGGTGTCTCCTAGGGGAATAAATCTTTGGGCACCTAGTGGTCATG

>930_4

CATGACCACTAGGAGCATCTTTGGCGAGATCGGGAGGATCGGTGGCATTGGTGCCTCCTAGGGGAATAAATCTTTGGGCACCTAGTGGTCATG

>926_4

CATGACCACTAGGAGCATCTTTGGCGAGATCGGGAGAATCGTTGGCATTGGAGTCTCCTAGGGGAATAAATCTTTGGGCACCTAGTGGTCATG

>923_4

CATGACCACTAGGAGCATCTTTGGCGAGATCGTTAGAATCGGTGGCATTGGTGTCTCCTAGGGGAATAAATCTTTGGGCACCTAGTGGTCATG

>922_4

CATGACCACTAGGAGCATCTTTGGCGAGATCGTGAGAATCGGTGGCACTGGTGTCTCCTAGGGGAATAAATCTTTGGGCACCTAGTGGTCATG

>921_4

CATGACCACTAGGAGCATCTTTGGCGAGATCCGGAGAATCGGCGGCATTGGTGTCTCCTAGGGGAATAAATCTTTGGGCACCTAGTGGTCATG

>918_4

CATGACCACTAGGAGCATCTTTGGCGAGATCGGGAGAATCTGTGGCATTGGTGACTCCTAGGGGAATAAATCTTTGGGCACCTAGTGGTCATG

>913_4

CATGACCACTAGGAGCATCTTTGGCGAGATCGGTAGAATCGGTGTCATTGGTGTCTCCTAGGGGAATAAATCTTTGGGCACCTAGTGGTCATG

>908_4

CATGACCACTAGGAGCATCTTTGGCGAGATCGGGAGAATCGGTGGAATTGTTGTCTCCTAGGGGAATAAATCTTTGGGCACCTAGTGGTCATG

>893_4

CATGACCACTAGGAGCATCTTTGGCGAGATCGGGAGAATCGGCGGCATTGGGGTCTCCTAGGGGAATAAATCTTTGGGCACCTAGTGGTCATG

>892_4

CATGACCACTAGGAGCATCTTTGGCGAGATCGGGAGAATCGGTGGCATAGGTGTATCCTAGGGGAATAAATCTTTGGGCACCTAGTGGTCATG

>888_4

CATGACCACTAGGAGCATCTTTGGCGAGATCGGGAGAATCTGTGGCATTGGTTTCTCCTAGGGGAATAAATCTTTGGGCACCTAGTGGTCATG

>882_5

CATGACCACTAGGAGCATCTTTGGCGAGATCGGGAGAGTTGGTGGCATTGGTGTCTCCTAGGGGAATAAATCTTTGGGCACCTAGTGGTCATG

>880_5

CATGACCACTAGGAGCATCTTTGGCGAGATCGTGAGAGTCGGTGGCATTGGTGTCTCCTAGGGGAATAAATCTTTGGGCACCTAGTGGTCATG

>873_5

CATGACCACTAGGAGCATCTTTGGCGAGATCGCGAGAATTGGTGGCATTGGTGTCTCCTAGGGGAATAAATCTTTGGGCACCTAGTGGTCATG

>857_5

CATGACCACTAGGAGCATCTTTGGCGAGATCGGGAGAATCGGTGGACTTGGTGTCTCCTAGGGGAATAAATCTTTGGGCACCTAGTGGTCATG

>856_5

CATGACCACTAGGAGCATCTTTGGCGAGATCGGGAGAAACGGTGGCATTGGAGTCTCCTAGGGGAATAAATCTTTGGGCACCTAGTGGTCATG

>852_5

CATGACCACTAGGAGCATCTTTGGCGAGATCGGGAGAATCGGTGGCATCGGTGTTTCCTAGGGGAATAAATCTTTGGGCACCTAGTGGTCATG

>850_5

CATGACCACTAGGAGCATCTTTGGCGAGATTGGGAGAATCGGTGGCATTGGCGTCTCCTAGGGGAATAAATCTTTGGGCACCTAGTGGTCATG

>848_5

CATGACCACTAGGAGCATCTTTGGCGAGATCGGAAGAATCGGTGGCATCGGTGTCTCCTAGGGGAATAAATCTTTGGGCACCTAGTGGTCATG

>839_5

CATGACCACTAGGAGCATCTTTGGCGAGATCGAGAGAAACGGTGGCATTGGTGTCTCCTAGGGGAATAAATCTTTGGGCACCTAGTGGTCATG

>836_5

CATGACCACTAGGAGCATCTTTGGCGAGATCGGGAGAATCGGCGGCGTTGGTGTCTCCTAGGGGAATAAATCTTTGGGCACCTAGTGGTCATG

>835_5

CATGACCACTAGGAGCATCTTTGGCGAGATCGGGAGAATCGGCGGCAGTGGTGTCTCCTAGGGGAATAAATCTTTGGGCACCTAGTGGTCATG

>829_5

CATGACCACTAGGAGCATCTTTGGCGAGATCGGGAGAATCGGCGGCATTTGTGTCTCCTAGGGGAATAAATCTTTGGGCACCTAGTGGTCATG

>826_5

CATGACCACTAGGAGCATCTTTGGCGAGATCGGGAGAATCGGCTGCATTGGTGTCTCCTAGGGGAATAAATCTTTGGGCACCTAGTGGTCATG

>823_5

CATGACCACTAGGAGCATCTTTGGCGAGATTGGAAGAATCGGTGGCATTGGTGTCTCCTAGGGGAATAAATCTTTGGGCACCTAGTGGTCATG

>822_5

CATGACCACTAGGAGCATCTTTGGCGAGATCTGGGGAATCGGTGGCATTGGTGTCTCCTAGGGGAATAAATCTTTGGGCACCTAGTGGTCATG

>820_5

CATGACCACTAGGAGCATCTTTGGCGAGATTGGGAGGATCGGTGGCATTGGTGTCTCCTAGGGGAATAAATCTTTGGGCACCTAGTGGTCATG

>813_5

CATGACCACTAGGAGCATCTTTGGCGAGATCGGGAGAATCAGCGGCATTGGTGTCTCCTAGGGGAATAAATCTTTGGGCACCTAGTGGTCATG

>809_5

CATGACCACTAGGAGCATCTTTGGCGAGATCTGGAGAATCGGTGGCATTTGTGTCTCCTAGGGGAATAAATCTTTGGGCACCTAGTGGTCATG

>808_5

CATGACCACTAGGAGCATCTTTGGCGAGATCGGGAGAATCGGTGGCAATGGAGTCTCCTAGGGGAATAAATCTTTGGGCACCTAGTGGTCATG

>806_5

CATGACCACTAGGAGCATCTTTGGCGAGATCGGGAGAATCGGCGACATTGGTGTCTCCTAGGGGAATAAATCTTTGGGCACCTAGTGGTCATG

>803_5

CATGACCACTAGGAGCATCTTTGGCGAGATCGCGAGAATCGGCGGCATTGGTGTCTCCTAGGGGAATAAATCTTTGGGCACCTAGTGGTCATG

>798_5

CATGACCACTAGGAGCATCTTTGGCGAGATCTGGAGAATCTGTGGCATTGGTGTCTCCTAGGGGAATAAATCTTTGGGCACCTAGTGGTCATG

>795_5

CATGACCACTAGGAGCATCTTTGGCGAGATTGGTAGAATCGGTGGCATTGGTGTCTCCTAGGGGAATAAATCTTTGGGCACCTAGTGGTCATG

>793_5

CATGACCACTAGGAGCATCTTTGGCGAGATCGGGAGAATCTGTGGCATAGGTGTCTCCTAGGGGAATAAATCTTTGGGCACCTAGTGGTCATG

>789_5

CATGACCACTAGGAGCATCTTTGGCGAGATTGGGAGAATCGGTGGCATTAGTGTCTCCTAGGGGAATAAATCTTTGGGCACCTAGTGGTCATG

>783_5

CATGACCACTAGGAGCATCTTTGGCGAGATCGGGAGAATCGGTGGAATTGGTGACTCCTAGGGGAATAAATCTTTGGGCACCTAGTGGTCATG

>779_5

CATGACCACTAGGAGCATCTTTGGCGAGATCGGGGGAATTGGTGGCATTGGTGTCTCCTAGGGGAATAAATCTTTGGGCACCTAGTGGTCATG

>777_5

CATGACCACTAGGAGCATCTTTGGCGAGATCGGTAGAATCGGTGGCATTGGTGTTTCCTAGGGGAATAAATCTTTGGGCACCTAGTGGTCATG

>775_5

CATGACCACTAGGAGCATCTTTGGCGAGATCAGAAGAATCGGTGGCATTGGTGTCTCCTAGGGGAATAAATCTTTGGGCACCTAGTGGTCATG

>773_5

CATGACCACTAGGAGCATCTTTGGCGAGATCGGGGGACTCGGTGGCATTGGTGTCTCCTAGGGGAATAAATCTTTGGGCACCTAGTGGTCATG

>771_5

CATGACCACTAGGAGCATCTTTGGCGAGATCGTGAGAATCGGTGGTATTGGTGTCTCCTAGGGGAATAAATCTTTGGGCACCTAGTGGTCATG

>770_5

CATGACCACTAGGAGCATCTTTGGCGAGATCGGGAGAATCGGCGGCATAGGTGTCTCCTAGGGGAATAAATCTTTGGGCACCTAGTGGTCATG

>768_5

CATGACCACTAGGAGCATCTTTGGCGAGATCGCGAGAATCGGTGGCATTGGTGTTTCCTAGGGGAATAAATCTTTGGGCACCTAGTGGTCATG

>767_5

CATGACCACTAGGAGCATCTTTGGCGAGATCGGGAGAATCTGTGGCATTGGTGTTTCCTAGGGGAATAAATCTTTGGGCACCTAGTGGTCATG

>764_5

CATGACCACTAGGAGCATCTTTGGCGAGATTGGGAGAATCGGTGACATTGGTGTCTCCTAGGGGAATAAATCTTTGGGCACCTAGTGGTCATG

>761_5

CATGACCACTAGGAGCATCTTTGGCGAGATCGGGAGAATCGGTGTCATTGTTGTCTCCTAGGGGAATAAATCTTTGGGCACCTAGTGGTCATG

>758_5

CATGACCACTAGGAGCATCTTTGGCGAGATCGTGAGAATCGGTGGCATTGGTGCCTCCTAGGGGAATAAATCTTTGGGCACCTAGTGGTCATG

>754_6

CATGACCACTAGGAGCATCTTTGGCGAGATCAGGAGAATCGGTGGCATCGGTGTCTCCTAGGGGAATAAATCTTTGGGCACCTAGTGGTCATG

>752_6

CATGACCACTAGGAGCATCTTTGGCGAGATCGGAAGAATCGGTGGCATTGGTGCCTCCTAGGGGAATAAATCTTTGGGCACCTAGTGGTCATG

>751_6

CATGACCACTAGGAGCATCTTTGGCGAGATCGGGAGAATCGGTGGCATAGGAGTCTCCTAGGGGAATAAATCTTTGGGCACCTAGTGGTCATG

>747_6

CATGACCACTAGGAGCATCTTTGGCGAGATTGGGAGAATCGGTGGCATTGGTGTTTCCTAGGGGAATAAATCTTTGGGCACCTAGTGGTCATG

>745_6

CATGACCACTAGGAGCATCTTTGGCGAGATCGGGAGAATCGGTGGCATTAATGTCTCCTAGGGGAATAAATCTTTGGGCACCTAGTGGTCATG

>740_6

CATGACCACTAGGAGCATCTTTGGCGAGATCGGGAGAATCGGAGGCATAGGTGTCTCCTAGGGGAATAAATCTTTGGGCACCTAGTGGTCATG

>738_6

CATGACCACTAGGAGCATCTTTGGCGAGATCGTGAGAATCGGTGGCGTTGGTGTCTCCTAGGGGAATAAATCTTTGGGCACCTAGTGGTCATG

>737_6

CATGACCACTAGGAGCATCTTTGGCGAGATCGGGAGAATTGGTGGCATTGGTGTTTCCTAGGGGAATAAATCTTTGGGCACCTAGTGGTCATG

>728_6

CATGACCACTAGGAGCATCTTTGGCGAGATCGAGAGAATTGGTGGCATTGGTGTCTCCTAGGGGAATAAATCTTTGGGCACCTAGTGGTCATG

>727_6

CATGACCACTAGGAGCATCTTTGGCGAGATCGGGAGGATTGGTGGCATTGGTGTCTCCTAGGGGAATAAATCTTTGGGCACCTAGTGGTCATG

>723_6

CATGACCACTAGGAGCATCTTTGGCGAGATTGGGAGAATCGGAGGCATTGGTGTCTCCTAGGGGAATAAATCTTTGGGCACCTAGTGGTCATG

>720_6

CATGACCACTAGGAGCATCTTTGGCGAGATCGGGAGAATCGGATGCATTGGTGTCTCCTAGGGGAATAAATCTTTGGGCACCTAGTGGTCATG

>718_6

CATGACCACTAGGAGCATCTTTGGCGAGATCGGGAGAAACGGAGGCATTGGTGTCTCCTAGGGGAATAAATCTTTGGGCACCTAGTGGTCATG

>717_6

CATGACCACTAGGAGCATCTTTGGCGAGATCGGGAGAATCGTAGGCATTGGTGTCTCCTAGGGGAATAAATCTTTGGGCACCTAGTGGTCATG

>705_6

CATGACCACTAGGAGCATCTTTGGCGAGATCGGTAGAATCGGTGGCATTGGAGTCTCCTAGGGGAATAAATCTTTGGGCACCTAGTGGTCATG

>702_6

CATGACCACTAGGAGCATCTTTGGCGAGATCGAGAGAATCGGTGGCGTTGGTGTCTCCTAGGGGAATAAATCTTTGGGCACCTAGTGGTCATG

>698_6

CATGACCACTAGGAGCATCTTTGGCGAGATTGGGAGAATCGGTGGCACTGGTGTCTCCTAGGGGAATAAATCTTTGGGCACCTAGTGGTCATG

>686_6

CATGACCACTAGGAGCATCTTTGGCGAGATCGAGAGGATCGGTGGCATTGGTGTCTCCTAGGGGAATAAATCTTTGGGCACCTAGTGGTCATG

>682_6

CATGACCACTAGGAGCATCTTTGGCGAGATCGTGAGAATCGGTGTCATTGGTGTCTCCTAGGGGAATAAATCTTTGGGCACCTAGTGGTCATG

>681_6

CATGACCACTAGGAGCATCTTTGGCGAGATCGGGAGAATTGGTGGCATTGATGTCTCCTAGGGGAATAAATCTTTGGGCACCTAGTGGTCATG

>677_6

CATGACCACTAGGAGCATCTTTGGCGAGATCGGGAGAATTGGTGGCATTGGTGCCTCCTAGGGGAATAAATCTTTGGGCACCTAGTGGTCATG

>672_6

CATGACCACTAGGAGCATCTTTGGCGAGATCGGGAGGATCGGTGGTATTGGTGTCTCCTAGGGGAATAAATCTTTGGGCACCTAGTGGTCATG

>663_7

CATGACCACTAGGAGCATCTTTGGCGAGATCGGGAGAATCGGCGGCATTGGCGTCTCCTAGGGGAATAAATCTTTGGGCACCTAGTGGTCATG

>649_7

CATGACCACTAGGAGCATCTTTGGCGAGATCGTGAGAATCGGTGGCATTGGCGTCTCCTAGGGGAATAAATCTTTGGGCACCTAGTGGTCATG

>640_7

CATGACCACTAGGAGCATCTTTGGCGAGATTGGGAGAATCGGTGGCATTGGTGCCTCCTAGGGGAATAAATCTTTGGGCACCTAGTGGTCATG

>637_7

CATGACCACTAGGAGCATCTTTGGCGAGATCGGGAGAATCGGCGTCATTGGTGTCTCCTAGGGGAATAAATCTTTGGGCACCTAGTGGTCATG

>625_7

CATGACCACTAGGAGCATCTTTGGCGAGATCGGGAGAAACGGTGGCATAGGTGTCTCCTAGGGGAATAAATCTTTGGGCACCTAGTGGTCATG

>624_7

CATGACCACTAGGAGCATCTTTGGCGAGATCGTGAGAATCGGTGGCATAGGTGTCTCCTAGGGGAATAAATCTTTGGGCACCTAGTGGTCATG

>621_7

CATGACCACTAGGAGCATCTTTGGCGAGATCGTGAGGATCGGTGGCATTGGTGTCTCCTAGGGGAATAAATCTTTGGGCACCTAGTGGTCATG

>618_7

CATGACCACTAGGAGCATCTTTGGCGAGATCAGGAGAATTGGTGGCATTGGTGTCTCCTAGGGGAATAAATCTTTGGGCACCTAGTGGTCATG

>610_7

CATGACCACTAGGAGCATCTTTGGCGAGATCGGAGGAATCGGTGGCATTGGTGTCTCCTAGGGGAATAAATCTTTGGGCACCTAGTGGTCATG

>594_8

CATGACCACTAGGAGCATCTTTGGCGAGATCGGGAGAATCGTCGGCATTGGTGTCTCCTAGGGGAATAAATCTTTGGGCACCTAGTGGTCATG

>572_8

CATGACCACTAGGAGCATCTTTGGCGAGATTGGGAGAATCGGTGGTATTGGTGTCTCCTAGGGGAATAAATCTTTGGGCACCTAGTGGTCATG

>571_8

CATGACCACTAGGAGCATCTTTGGCGAGATCGTAAGAATCGGTGGCATTGGTGTCTCCTAGGGGAATAAATCTTTGGGCACCTAGTGGTCATG

>563_9

CATGACCACTAGGAGCATCTTTGGCGAGATCGTGAGAATCGGTGGCATCGGTGTCTCCTAGGGGAATAAATCTTTGGGCACCTAGTGGTCATG

>558_9

CATGACCACTAGGAGCATCTTTGGCGAGATAGGGAGAATCGGTGGCATTTGTGTCTCCTAGGGGAATAAATCTTTGGGCACCTAGTGGTCATG

>545_9

CATGACCACTAGGAGCATCTTTGGCGAGATTGGGAGAATCGGTGGCATCGGTGTCTCCTAGGGGAATAAATCTTTGGGCACCTAGTGGTCATG

>540_10

CATGACCACTAGGAGCATCTTTGGCGAGATCGGGAGACTTGGTGGCATTGGTGTCTCCTAGGGGAATAAATCTTTGGGCACCTAGTGGTCATG

>535_10

CATGACCACTAGGAGCATCTTTGGCGAGATCGGGAGAATTGGTGGCATCGGTGTCTCCTAGGGGAATAAATCTTTGGGCACCTAGTGGTCATG

>506_11

CATGACCACTAGGAGCATCTTTGGCGAGATCGGGAGGATCGGCGGCATTGGTGTCTCCTAGGGGAATAAATCTTTGGGCACCTAGTGGTCATG

>500_11

CATGACCACTAGGAGCATCTTTGGCGAGATCGGGAGAATCAATGGCATTGGTGTCTCCTAGGGGAATAAATCTTTGGGCACCTAGTGGTCATG

>494_11

CATGACCACTAGGAGCATCTTTGGCGAGATCGGTAGAATCGGCGGCATTGGTGTCTCCTAGGGGAATAAATCTTTGGGCACCTAGTGGTCATG

>461_13

CATGACCACTAGGAGCATCTTTGGCGAGATCTGGAGAATCGGCGGCATTGGTGTCTCCTAGGGGAATAAATCTTTGGGCACCTAGTGGTCATG

>446_14

CATGACCACTAGGAGCATCTTTGGCGAGATCGAGAGAATCGGCGGCATTGGTGTCTCCTAGGGGAATAAATCTTTGGGCACCTAGTGGTCATG

>444_14

CATGACCACTAGGAGCATCTTTGGCGAGATCGTGAGAATCGGTGGCATTGGTGTTTCCTAGGGGAATAAATCTTTGGGCACCTAGTGGTCATG

>429_15

CATGACCACTAGGAGCATCTTTGGCGAGATCGGGAGAATCGGTAACATTGGTGTCTCCTAGGGGAATAAATCTTTGGGCACCTAGTGGTCATG

>428_15

CATGACCACTAGGAGCATCTTTGGCGAGATCGTGAGAATTGGTGGCATTGGTGTCTCCTAGGGGAATAAATCTTTGGGCACCTAGTGGTCATG

>413_15

CATGACCACTAGGAGCATCTTTGGCGAGATTGGGAGAATTGGTGGCATTGGTGTCTCCTAGGGGAATAAATCTTTGGGCACCTAGTGGTCATG

>389_17

CATGACCACTAGGAGCATCTTTGGCGAGATCGGGAGAATCGGCGGCATTGGTGCCTCCTAGGGGAATAAATCTTTGGGCACCTAGTGGTCATG

>375_18

CATGACCACTAGGAGCATCTTTGGCGAGATTGTGAGAATCGGTGGCATTGGTGTCTCCTAGGGGAATAAATCTTTGGGCACCTAGTGGTCATG

>369_19

CATGACCACTAGGAGCATCTTTGGCGAGATCGGGAGAATCGGCGGTATTGGTGTCTCCTAGGGGAATAAATCTTTGGGCACCTAGTGGTCATG

>367_19

CATGACCACTAGGAGCATCTTTGGCGAGATCGGGAGAGTCGGCGGCATTGGTGTCTCCTAGGGGAATAAATCTTTGGGCACCTAGTGGTCATG

>357_21

CATGACCACTAGGAGCATCTTTGGCGAGATCGGGAGAATCGGCGGCATCGGTGTCTCCTAGGGGAATAAATCTTTGGGCACCTAGTGGTCATG

>350_21

CATGACCACTAGGAGCATCTTTGGCGAGATCGGGGGAATCGGCGGCATTGGTGTCTCCTAGGGGAATAAATCTTTGGGCACCTAGTGGTCATG

>333_24

CATGACCACTAGGAGCATCTTTGGCGAGATCAAGAGAATCGGTGGCATTGGTGTCTCCTAGGGGAATAAATCTTTGGGCACCTAGTGGTCATG

>323_26

CATGACCACTAGGAGCATCTTTGGCGAGATCGGGAGAATCGGCGGCATTGGTGTTTCCTAGGGGAATAAATCTTTGGGCACCTAGTGGTCATG

>309_28

CATGACCACTAGGAGCATCTTTGGCGAGATCGGGAGAATTGGCGGCATTGGTGTCTCCTAGGGGAATAAATCTTTGGGCACCTAGTGGTCATG

>291_32

CATGACCACTAGGAGCATCTTTGGCGAGATCGTGAGAATCGGCGGCATTGGTGTCTCCTAGGGGAATAAATCTTTGGGCACCTAGTGGTCATG

>272_34

CATGACCACTAGGAGCATCTTTGGCGAGATCAGGAGAATCGGCGGCATTGGTGTCTCCTAGGGGAATAAATCTTTGGGCACCTAGTGGTCATG

>268_35

CATGACCACTAGGAGCATCTTTGGCGAGATCGAAAGAATCGGTGGCATTGGTGTCTCCTAGGGGAATAAATCTTTGGGCACCTAGTGGTCATG

>225_48

CATGACCACTAGGAGCATCTTTGGCGAGATTGGGAGAATCGGCGGCATTGGTGTCTCCTAGGGGAATAAATCTTTGGGCACCTAGTGGTCATG

**2. Using II-R1-3 (3_57642) to blast round_20^th^ top 1000 full_length sequences**

>3_57642

CATGACCACTAGGAGCATCTTTGGCGAGAAGACTCTGGATTCGGGGACCAGTTGCTGCTAGGGGAATAAATCTTTGGGCACCTAGTGGTCATG

>998_4

CATGACCACTAGGAGCATCTTTGGCGAGAAGACTCTGGATTCGGGGACCAGTTCCTGCTAGGGGAATAAATCTTTGGGCACCTAGTGGTCATG

>929_4

CATGACCACTAGGAGCATCTTTGGCGAGAAGACTCTGGATTCGGGGAGCAGTTGCTGCTAGGGGAATAAATCTTTGGGCACCTAGTGGTCATG

>825_5

CATGACCACTAGGAGCATCTTTGGCGAGAAGACTCTGGATTCGGGGACCACTTGCTGCTAGGGGAATAAATCTTTGGGCACCTAGTGGTCATG

>821_5

CATGACCACTAGGAGCATCTTTGGCGAGAAGACTCTGGATTCGGGGACCAGTTGGTGCTAGGGGAATAAATCTTTGGGCACCTAGTGGTCATG

>816_5

CATGACCACTAGGAGCATCTTTGGCGAGAAGCCTCTGGATTCGGGGACCAGTTGCTGCTAGGGGAATAAATCTTTGGGCACCTAGTGGTCATG

>814_5

CATGACCACTAGGAGCATCTTTGGCGAGAAGACTCTGGATTCGGGGACCCGTTGCTGCTAGGGGAATAAATCTTTGGGCACCTAGTGGTCATG

>750_6

CATGACCACTAGGAGCATCTTTGGCGAGAACACTCTGGATTCGGGGACCAGTTGCTGCTAGGGGAATAAATCTTTGGGCACCTAGTGGTCATG

>744_6

CATGACCACTAGGAGCATCTTTGGCGAGAAGAGTCTGGATTCGGGGACCAGTTGCTGCTAGGGGAATAAATCTTTGGGCACCTAGTGGTCATG

>714_6

CATGACCACTAGGAGCATCTTTGGCGAGAAGACTCTGGATTCGGGGTCCAGTTGCTGCTAGGGGAATAAATCTTTGGGCACCTAGTGGTCATG

>685_6

CATGACCACTAGGAGCATCTTTGGCGAGAAGACTCTGGATTCGGGGACGAGTTGCTGCTAGGGGAATAAATCTTTGGGCACCTAGTGGTCATG

>652_7

CATGACCACTAGGAGCATCTTTGGCGAGAAGACTCTGGATTCGGGGACCAGGTGCTGCTAGGGGAATAAATCTTTGGGCACCTAGTGGTCATG

>570_8

CATGACCACTAGGAGCATCTTTGGCGAGAAGACTCTGGATTCGGGGCCCAGTTGCTGCTAGGGGAATAAATCTTTGGGCACCTAGTGGTCATG

>546_9

CATGACCACTAGGAGCATCTTTGGCGAGAAGACTCTGGATTCGGCGACCAGTTGCTGCTAGGGGAATAAATCTTTGGGCACCTAGTGGTCATG

>536_10

CATGACCACTAGGAGCATCTTTGGCGAGAAGACTCTGGCTTCGGGGACCAGTTGCTGCTAGGGGAATAAATCTTTGGGCACCTAGTGGTCATG

>525_10

CATGACCACTAGGAGCATCTTTGGCGAGAAGTCTCTGGATTCGGGGACCAGTTGCTGCTAGGGGAATAAATCTTTGGGCACCTAGTGGTCATG

>521_10

CATGACCACTAGGAGCATCTTTGGCGAGAAGACTCTGGTTTCGGGGACCAGTTGCTGCTAGGGGAATAAATCTTTGGGCACCTAGTGGTCATG

>513_10

CATGACCACTAGGAGCATCTTTGGCGAGAAGACTCTGGATTCCGGGACCAGTTGCTGCTAGGGGAATAAATCTTTGGGCACCTAGTGGTCATG

>504_11

CATGACCACTAGGAGCATCTTTGGCGAGAAGACTCTGGATTCGCGGACCAGTTGCTGCTAGGGGAATAAATCTTTGGGCACCTAGTGGTCATG

>502_11

CATGACCACTAGGAGCATCTTTGGCGAGATGACTCTGGATTCGGGGACCAGTTGCTGCTAGGGGAATAAATCTTTGGGCACCTAGTGGTCATG

>482_12

CATGACCACTAGGAGCATCTTTGGCGAGAAGACTCTGGATTCGGGCACCAGTTGCTGCTAGGGGAATAAATCTTTGGGCACCTAGTGGTCATG

>481_12

CATGACCACTAGGAGCATCTTTGGCGAGAAGACTCTGGATTCGGGGACCTGTTGCTGCTAGGGGAATAAATCTTTGGGCACCTAGTGGTCATG

>445_14

CATGACCACTAGGAGCATCTTTGGCGAGACGACTCTGGATTCGGGGACCAGTTGCTGCTAGGGGAATAAATCTTTGGGCACCTAGTGGTCATG

>441_14

CATGACCACTAGGAGCATCTTTGGCGAGAAGACTCTGGATACGGGGACCAGTTGCTGCTAGGGGAATAAATCTTTGGGCACCTAGTGGTCATG

>418_15

CATGACCACTAGGAGCATCTTTGGCGAGAAGACTCTGGATTAGGGGACCAGTTGCTGCTAGGGGAATAAATCTTTGGGCACCTAGTGGTCATG

>406_15

CATGACCACTAGGAGCATCTTTGGCGAGAAGACTCTGGATTCGGGGACCAGTTACTGCTAGGGGAATAAATCTTTGGGCACCTAGTGGTCATG

>400_16

CATGACCACTAGGAGCATCTTTGGCGAGAAGACTCGGGATTCGGGGACCAGTTGCTGCTAGGGGAATAAATCTTTGGGCACCTAGTGGTCATG

>393_16

CATGACCACTAGGAGCATCTTTGGCGAGAAGACTCTGGATTCGGGGAACAGTTGCTGCTAGGGGAATAAATCTTTGGGCACCTAGTGGTCATG

>372_18

CATGACCACTAGGAGCATCTTTGGCGAGAAGACTCTTGATTCGGGGACCAGTTGCTGCTAGGGGAATAAATCTTTGGGCACCTAGTGGTCATG

>365_19

CATGACCACTAGGAGCATCTTTGGCGAGAAGACTCTGGAATCGGGGACCAGTTGCTGCTAGGGGAATAAATCTTTGGGCACCTAGTGGTCATG

>352_21

CATGACCACTAGGAGCATCTTTGGCGAGAAGACTCTGGATTCGGGGACCAATTGCTGCTAGGGGAATAAATCTTTGGGCACCTAGTGGTCATG

>337_23

CATGACCACTAGGAGCATCTTTGGCGAGAAGAATCTGGATTCGGGGACCAGTTGCTGCTAGGGGAATAAATCTTTGGGCACCTAGTGGTCATG

>327_25

CATGACCACTAGGAGCATCTTTGGCGAGAAGACTCTGGATTCGGAGACCAGTTGCTGCTAGGGGAATAAATCTTTGGGCACCTAGTGGTCATG

>324_26

CATGACCACTAGGAGCATCTTTGGCGAGAAGACTCTGGATTCGGGAACCAGTTGCTGCTAGGGGAATAAATCTTTGGGCACCTAGTGGTCATG

>317_27

CATGACCACTAGGAGCATCTTTGGCGAGAAGACTCTGGATTCGGGGACCATTTGCTGCTAGGGGAATAAATCTTTGGGCACCTAGTGGTCATG

>314_27

CATGACCACTAGGAGCATCTTTGGCGAGAAGACTCTGGATTCGGGGACCAGTTGATGCTAGGGGAATAAATCTTTGGGCACCTAGTGGTCATG

>311_28

CATGACCACTAGGAGCATCTTTGGCGAGAAGACTCTGGATTCGGGGACAAGTTGCTGCTAGGGGAATAAATCTTTGGGCACCTAGTGGTCATG

>305_29

CATGACCACTAGGAGCATCTTTGGCGAGAAGACTCTGGATTCGGGGACCAGTTTCTGCTAGGGGAATAAATCTTTGGGCACCTAGTGGTCATG

>301_29

CATGACCACTAGGAGCATCTTTGGCGAGAAGACTCTGGATTCGAGGACCAGTTGCTGCTAGGGGAATAAATCTTTGGGCACCTAGTGGTCATG

>296_31

CATGACCACTAGGAGCATCTTTGGCGAGAAGACTCTGGATCCGGGGACCAGTTGCTGCTAGGGGAATAAATCTTTGGGCACCTAGTGGTCATG

>293_31

CATGACCACTAGGAGCATCTTTGGCGAGAAGACTCTGGATTCGGGGACCAGTAGCTGCTAGGGGAATAAATCTTTGGGCACCTAGTGGTCATG

>288_32

CATGACCACTAGGAGCATCTTTGGCGAGAAGACTCTGGAGTCGGGGACCAGTTGCTGCTAGGGGAATAAATCTTTGGGCACCTAGTGGTCATG

>283_33

CATGACCACTAGGAGCATCTTTGGCGAGAAGACTCTGGATTCTGGGACCAGTTGCTGCTAGGGGAATAAATCTTTGGGCACCTAGTGGTCATG

>282_33

CATGACCACTAGGAGCATCTTTGGCGAGAAGACTCTGGATTCGGTGACCAGTTGCTGCTAGGGGAATAAATCTTTGGGCACCTAGTGGTCATG

>279_34

CATGACCACTAGGAGCATCTTTGGCGAGAAGACTGTGGATTCGGGGACCAGTTGCTGCTAGGGGAATAAATCTTTGGGCACCTAGTGGTCATG

>274_34

CATGACCACTAGGAGCATCTTTGGCGAGAAAACTCTGGATTCGGGGACCAGTTGCTGCTAGGGGAATAAATCTTTGGGCACCTAGTGGTCATG

>273_34

CATGACCACTAGGAGCATCTTTGGCGAGAAGACTCTAGATTCGGGGACCAGTTGCTGCTAGGGGAATAAATCTTTGGGCACCTAGTGGTCATG

>262_36

CATGACCACTAGGAGCATCTTTGGCGAGAAGACTCTGGATTCGGGGACCAGATGCTGCTAGGGGAATAAATCTTTGGGCACCTAGTGGTCATG

>254_37

CATGACCACTAGGAGCATCTTTGGCGAGAAGACGCTGGATTCGGGGACCAGTTGCTGCTAGGGGAATAAATCTTTGGGCACCTAGTGGTCATG

>251_39

CATGACCACTAGGAGCATCTTTGGCGAGAAGACTCTGGATTCGTGGACCAGTTGCTGCTAGGGGAATAAATCTTTGGGCACCTAGTGGTCATG

>246_40

CATGACCACTAGGAGCATCTTTGGCGAGAAGACACTGGATTCGGGGACCAGTTGCTGCTAGGGGAATAAATCTTTGGGCACCTAGTGGTCATG

>242_42

CATGACCACTAGGAGCATCTTTGGCGAGAAGACTCTGAATTCGGGGACCAGTTGCTGCTAGGGGAATAAATCTTTGGGCACCTAGTGGTCATG

>241_42

CATGACCACTAGGAGCATCTTTGGCGAGAAGACTCTGGATTCAGGGACCAGTTGCTGCTAGGGGAATAAATCTTTGGGCACCTAGTGGTCATG

>224_49

CATGACCACTAGGAGCATCTTTGGCGAGAGGACTCTGGATTCGGGGACCAGTTGCTGCTAGGGGAATAAATCTTTGGGCACCTAGTGGTCATG

>217_52

CATGACCACTAGGAGCATCTTTGGCGAGAAGACTCTGGATTCGGGGACCAGTCGCTGCTAGGGGAATAAATCTTTGGGCACCTAGTGGTCATG

>216_52

CATGACCACTAGGAGCATCTTTGGCGAGAAGACTATGGATTCGGGGACCAGTTGCTGCTAGGGGAATAAATCTTTGGGCACCTAGTGGTCATG

>212_55

CATGACCACTAGGAGCATCTTTGGCGAGAAGACTCTGGATTCGGGTACCAGTTGCTGCTAGGGGAATAAATCTTTGGGCACCTAGTGGTCATG

>208_56

CATGACCACTAGGAGCATCTTTGGCGAGAAGACTCTGGATTCGGGGACCAGCTGCTGCTAGGGGAATAAATCTTTGGGCACCTAGTGGTCATG

>194_65

CATGACCACTAGGAGCATCTTTGGCGAGAAGACTCTGGACTCGGGGACCAGTTGCTGCTAGGGGAATAAATCTTTGGGCACCTAGTGGTCATG

>187_71

CATGACCACTAGGAGCATCTTTGGCGAGAAGACTCTGGATTCGGGGACCGGTTGCTGCTAGGGGAATAAATCTTTGGGCACCTAGTGGTCATG

>180_76

CATGACCACTAGGAGCATCTTTGGCGAGAAGGCTCTGGATTCGGGGACCAGTTGCTGCTAGGGGAATAAATCTTTGGGCACCTAGTGGTCATG

>165_88

CATGACCACTAGGAGCATCTTTGGCGAGAAGACTCTGGGTTCGGGGACCAGTTGCTGCTAGGGGAATAAATCTTTGGGCACCTAGTGGTCATG

>163_91

CATGACCACTAGGAGCATCTTTGGCGAGAATACTCTGGATTCGGGGACCAGTTGCTGCTAGGGGAATAAATCTTTGGGCACCTAGTGGTCATG

>156_102

CATGACCACTAGGAGCATCTTTGGCGAGAAGACTCTGGATTCGGGGGCCAGTTGCTGCTAGGGGAATAAATCTTTGGGCACCTAGTGGTCATG

>142_125

CATGACCACTAGGAGCATCTTTGGCGAGAAGACTCTGGATTCGGGGATCAGTTGCTGCTAGGGGAATAAATCTTTGGGCACCTAGTGGTCATG

>141_127

CATGACCACTAGGAGCATCTTTGGCGAGAAGATTCTGGATTCGGGGACCAGTTGCTGCTAGGGGAATAAATCTTTGGGCACCTAGTGGTCATG

>120_170

CATGACCACTAGGAGCATCTTTGGCGAGAAGACTCAGGATTCGGGGACCAGTTGCTGCTAGGGGAATAAATCTTTGGGCACCTAGTGGTCATG

>116_185

CATGACCACTAGGAGCATCTTTGGCGAGAAGACTCTGGATTCGGGGACCAGTTGTTGCTAGGGGAATAAATCTTTGGGCACCTAGTGGTCATG

>109_220

CATGACCACTAGGAGCATCTTTGGCGAGAAGACTCTGGATTTGGGGACCAGTTGCTGCTAGGGGAATAAATCTTTGGGCACCTAGTGGTCATG

>101_252

CATGACCACTAGGAGCATCTTTGGCGAGAAGACTCTGTATTCGGGGACCAGTTGCTGCTAGGGGAATAAATCTTTGGGCACCTAGTGGTCATG

>100_254

CATGACCACTAGGAGCATCTTTGGCGAGAAGACCCTGGATTCGGGGACCAGTTGCTGCTAGGGGAATAAATCTTTGGGCACCTAGTGGTCATG

>83_354

CATGACCACTAGGAGCATCTTTGGCGAGAAGACTCTGGATTCGGGGACTAGTTGCTGCTAGGGGAATAAATCTTTGGGCACCTAGTGGTCATG

>56_520

CATGACCACTAGGAGCATCTTTGGCGAGAAGACTCCGGATTCGGGGACCAGTTGCTGCTAGGGGAATAAATCTTTGGGCACCTAGTGGTCATG

>37_907

CATGACCACTAGGAGCATCTTTGGCGAGAAGACTTTGGATTCGGGGACCAGTTGCTGCTAGGGGAATAAATCTTTGGGCACCTAGTGGTCATG

>983_4

CATGACCACTAGGAGCATCTTTGGCGAGAAGACCCTGGATTCGGGGACTAGTTGCTGCTAGGGGAATAAATCTTTGGGCACCTAGTGGTCATG

>899_4

CATGACCACTAGGAGCATCTTTGGCGAGAAGACTCCGGATTCGGGGACCAGTTACTGCTAGGGGAATAAATCTTTGGGCACCTAGTGGTCATG

>884_4

CATGACCACTAGGAGCATCTTTGGCGAGAAGACTTTGGATTCGGGGATCAGTTGCTGCTAGGGGAATAAATCTTTGGGCACCTAGTGGTCATG

>734_6

CATGACCACTAGGAGCATCTTTGGCGAGAAGACTTTGGATTTGGGGACCAGTTGCTGCTAGGGGAATAAATCTTTGGGCACCTAGTGGTCATG

>582_8

CATGACCACTAGGAGCATCTTTGGCGAGAAGACTTTGGATTCGGGGACTAGTTGCTGCTAGGGGAATAAATCTTTGGGCACCTAGTGGTCATG

>501_11

CATGACCACTAGGAGCATCTTTGGCGAGAAGACTTCGGATTCGGGGACCAGTTGCTGCTAGGGGAATAAATCTTTGGGCACCTAGTGGTCATG

>411_15

CATGACCACTAGGAGCATCTTTGGCGAGAAGACCTTGGATTCGGGGACCAGTTGCTGCTAGGGGAATAAATCTTTGGGCACCTAGTGGTCATG

>237_44

CATGACCACTAGGAGCATCTTTGGCGAGAAGACTCTGGATTCGGGGATTAGTTGCTGCTAGGGGAATAAATCTTTGGGCACCTAGTGGTCATG

**3. Using II-R1-7 (7_3906) to blast round_20^th^ top 1000 full_length sequences**

>7_3906

CATGACCACTAGGAGCATCTTTGGCGAGATCGGGAGAATCGGTGGCATTGGTGTCTTCTAGGGGAATAAATCTTTGGGCACCTAGTGGTCATG

>905_4

CATGACCACTAGGAGCATCTTTGGCGAGATCGGGAGAATCGGTGGCATTGGTGCCTTCTAGGGGAATAAATCTTTGGGCACCTAGTGGTCATG

>872_5

CATGACCACTAGGAGCATCTTTGGCGAGATCGGCAGAATCGGTGGCATTGGTGTCTTCTAGGGGAATAAATCTTTGGGCACCTAGTGGTCATG

>845_5

CATGACCACTAGGAGCATCTTTGGCGAGATCGGGAGAATCGGTGGTATTGGTGTCTTCTAGGGGAATAAATCTTTGGGCACCTAGTGGTCATG

>778_5

CATGACCACTAGGAGCATCTTTGGCGAGATCGGAAGAATCGGTGGCATTGGTGTCTTCTAGGGGAATAAATCTTTGGGCACCTAGTGGTCATG

>776_5

CATGACCACTAGGAGCATCTTTGGCGAGATCGGGAGAGTCGGTGGCATTGGTGTCTTCTAGGGGAATAAATCTTTGGGCACCTAGTGGTCATG

>703_6

CATGACCACTAGGAGCATCTTTGGCGAGATCGGGGGAATCGGTGGCATTGGTGTCTTCTAGGGGAATAAATCTTTGGGCACCTAGTGGTCATG

>673_6

CATGACCACTAGGAGCATCTTTGGCGAGATCGGGAGAATCGGTGGCATAGGTGTCTTCTAGGGGAATAAATCTTTGGGCACCTAGTGGTCATG

>661_7

CATGACCACTAGGAGCATCTTTGGCGAGATCGCGAGAATCGGTGGCATTGGTGTCTTCTAGGGGAATAAATCTTTGGGCACCTAGTGGTCATG

>648_7

CATGACCACTAGGAGCATCTTTGGCGAGATCAGGAGAATCGGTGGCATTGGTGTCTTCTAGGGGAATAAATCTTTGGGCACCTAGTGGTCATG

>601_8

CATGACCACTAGGAGCATCTTTGGCGAGATCGGGAGAATCGGTGGCATCGGTGTCTTCTAGGGGAATAAATCTTTGGGCACCTAGTGGTCATG

>544_9

CATGACCACTAGGAGCATCTTTGGCGAGATCGGGAGAATTGGTGGCATTGGTGTCTTCTAGGGGAATAAATCTTTGGGCACCTAGTGGTCATG

>511_11

CATGACCACTAGGAGCATCTTTGGCGAGATCGGTAGAATCGGTGGCATTGGTGTCTTCTAGGGGAATAAATCTTTGGGCACCTAGTGGTCATG

>510_11

CATGACCACTAGGAGCATCTTTGGCGAGATCGAGAGAATCGGTGGCATTGGTGTCTTCTAGGGGAATAAATCTTTGGGCACCTAGTGGTCATG

>503_11

CATGACCACTAGGAGCATCTTTGGCGAGATCGGGAGGATCGGTGGCATTGGTGTCTTCTAGGGGAATAAATCTTTGGGCACCTAGTGGTCATG

>448_14

CATGACCACTAGGAGCATCTTTGGCGAGATCGTGAGAATCGGTGGCATTGGTGTCTTCTAGGGGAATAAATCTTTGGGCACCTAGTGGTCATG

>404_15

CATGACCACTAGGAGCATCTTTGGCGAGATCGGGAGAATCGGTGGCATTGGTGTTTTCTAGGGGAATAAATCTTTGGGCACCTAGTGGTCATG

>341_23

CATGACCACTAGGAGCATCTTTGGCGAGATTGGGAGAATCGGTGGCATTGGTGTCTTCTAGGGGAATAAATCTTTGGGCACCTAGTGGTCATG

>297_30

CATGACCACTAGGAGCATCTTTGGCGAGATCGGGAGAATCGGCGGCATTGGTGTCTTCTAGGGGAATAAATCTTTGGGCACCTAGTGGTCATG
