# Supplementary material for: Selection of allosteric dnazymes that can sense phenylalanine by expression-SELEX
Source: Nucleic Acids Res. 2023 May 19;51(11):e66. doi: 10.1093/nar/gkad424 (PMC10287898; doi:10.1093/nar/gkad424)
Supplement: gkad424_Supplemental_Files [file gkad424_supplemental_files.zip › Supplementary file No. 8 three-top-enriched-sequences-clustal.docx]

**1. CLUSTAL multiple sequence alignment for II-R1-1(1_892954)**

5_7863 CATGACCACTAGGAGCATCTTTGGCGAGATCGGGAGAATCGGCGGCATTGGTGTCTCCTA 60

6_4354 CATGACCACTAGGAGCATCTTTGGCGAGATTGGGAGAATCGGTGGCATTGGTGTCTCCTA 60

11_3283 CATGACCACTAGGAGCATCTTTGGCGAGATCGGGAGAATTGGTGGCATTGGTGTCTCCTA 60

12_2781 CATGACCACTAGGAGCATCTTTGGCGAGATCGTGAGAATCGGTGGCATTGGTGTCTCCTA 60

14_2397 CATGACCACTAGGAGCATCTTTGGCGAGATCGGGAGAATCGGTGGCATTGGTGTTTCCTA 60

15_2111 CATGACCACTAGGAGCATCTTTGGCGAGATCGGGAGAATCGGTGGCATCGGTGTCTCCTA 60

17_1565 CATGACCACTAGGAGCATCTTTGGCGAGATCGGGGGAATCGGTGGCATTGGTGTCTCCTA 60

18_1555 CATGACCACTAGGAGCATCTTTGGCGAGATCAGGAGAATCGGTGGCATTGGTGTCTCCTA 60

20_1482 CATGACCACTAGGAGCATCTTTGGCGAGATCGGGAGGATCGGTGGCATTGGTGTCTCCTA 60

22_1449 CATGACCACTAGGAGCATCTTTGGCGAGATCGAGAGAATCGGTGGCATTGGTGTCTCCTA 60

24_1332 CATGACCACTAGGAGCATCTTTGGCGAGATCGGGAGAATCGGTGGCATTGGTGCCTCCTA 60

26_1257 CATGACCACTAGGAGCATCTTTGGCGAGATCGGGAGAATCGGTGGCATTGGCGTCTCCTA 60

30_1081 CATGACCACTAGGAGCATCTTTGGCGAGATCGGGAGAATCGGTGGTATTGGTGTCTCCTA 60

32_1013 CATGACCACTAGGAGCATCTTTGGCGAGATCGGAAGAATCGGTGGCATTGGTGTCTCCTA 60

33_1011 CATGACCACTAGGAGCATCTTTGGCGAGATCGGGAGAATCGGTGGCGTTGGTGTCTCCTA 60

34_963 CATGACCACTAGGAGCATCTTTGGCGAGATCGCGAGAATCGGTGGCATTGGTGTCTCCTA 60

35_938 CATGACCACTAGGAGCATCTTTGGCGAGATCGGTAGAATCGGTGGCATTGGTGTCTCCTA 60

40_706 CATGACCACTAGGAGCATCTTTGGCGAGATCTGGAGAATCGGTGGCATTGGTGTCTCCTA 60

45_629 CATGACCACTAGGAGCATCTTTGGCGAGATCGGGAGAATCGGTGGCACTGGTGTCTCCTA 60

46_618 CATGACCACTAGGAGCATCTTTGGCGAGATCGGCAGAATCGGTGGCATTGGTGTCTCCTA 60

49_559 CATGACCACTAGGAGCATCTTTGGCGAGATCGGGAGAATCGTTGGCATTGGTGTCTCCTA 60

50_552 CATGACCACTAGGAGCATCTTTGGCGAGATCGGGAGAATCGGAGGCATTGGTGTCTCCTA 60

52_534 CATGACCACTAGGAGCATCTTTGGCGAGATCGGGAGAATCGGTGGCATAGGTGTCTCCTA 60

53_527 CATGACCACTAGGAGCATCTTTGGCGAGATCGGGAGAATCTGTGGCATTGGTGTCTCCTA 60

55_526 CATGACCACTAGGAGCATCTTTGGCGAGATCGGGAGAATCAGTGGCATTGGTGTCTCCTA 60

57_518 CATGACCACTAGGAGCATCTTTGGCGAGACCGGGAGAATCGGTGGCATTGGTGTCTCCTA 60

59_481 CATGACCACTAGGAGCATCTTTGGCGAGATCGGGAGAATCGGTGTCATTGGTGTCTCCTA 60

61_454 CATGACCACTAGGAGCATCTTTGGCGAGATCGGGAGAATCGGTGGCATTAGTGTCTCCTA 60

62_452 CATGACCACTAGGAGCATCTTTGGCGAGATCGGGAGAATCGGTGGCATTGATGTCTCCTA 60

63_447 CATGACCACTAGGAGCATCTTTGGCGAGATCGGGAGAATCGGTAGCATTGGTGTCTCCTA 60

67_430 CATGACCACTAGGAGCATCTTTGGCGAGATCGGGAGAAACGGTGGCATTGGTGTCTCCTA 60

69_427 CATGACCACTAGGAGCATCTTTGGCGAGATCGGGAGAATCGGTGGCATTGTTGTCTCCTA 60

75_386 CATGACCACTAGGAGCATCTTTGGCGAGATCGGGATAATCGGTGGCATTGGTGTCTCCTA 60

77_378 CATGACCACTAGGAGCATCTTTGGCGAGATCGGGAGAATCGGTGACATTGGTGTCTCCTA 60

78_370 CATGACCACTAGGAGCATCTTTGGCGAGATCGGGAGAATCGGTGGCATTTGTGTCTCCTA 60

79_369 CATGACCACTAGGAGCATCTTTGGCGAGATAGGGAGAATCGGTGGCATTGGTGTCTCCTA 60

81_364 CATGACCACTAGGAGCATCTTTGGCGAGATCGGGAGAATCGGTGGCAGTGGTGTCTCCTA 60

84_354 CATGACCACTAGGAGCATCTTTGGCGAGATCGGGAGAACCGGTGGCATTGGTGTCTCCTA 60

87_343 CATGACCACTAGGAGCATCTTTGGCGAGATCGGGAGAATCGGTGGCATTGGAGTCTCCTA 60

89_330 CATGACCACTAGGAGCATCTTTGGCGAGATCGGGAGAATCGGTGGCATTGGTGACTCCTA 60

90_327 CATGACCACTAGGAGCATCTTTGGCGAGATCGGGAGAATCGGTGGAATTGGTGTCTCCTA 60

92_310 CATGACCACTAGGAGCATCTTTGGCGAGATCGGGAGAATCGGTGGCATTGGTTTCTCCTA 60

93_307 CATGACCACTAGGAGCATCTTTGGCGAGATCGGGAGAATAGGTGGCATTGGTGTCTCCTA 60

94_296 CATGACCACTAGGAGCATCTTTGGCGAGATCGGGAGAATCGATGGCATTGGTGTCTCCTA 60

95_285 CATGACCACTAGGAGCATCTTTGGCGAGATCGGGAGAATCGGTGGCATTGGTATCTCCTA 60

96_283 CATGACCACTAGGAGCATCTTTGGCGAGATCGGGAGAATCGGTTGCATTGGTGTCTCCTA 60

97_272 CATGACCACTAGGAGCATCTTTGGCGAGATCGGGAGAATCGCTGGCATTGGTGTCTCCTA 60

99_259 CATGACCACTAGGAGCATCTTTGGCGAGATCGGGAGAATCGGTGGCAATGGTGTCTCCTA 60

105_226 CATGACCACTAGGAGCATCTTTGGCGAGATCGGGAAAATCGGTGGCATTGGTGTCTCCTA 60

108_222 CATGACCACTAGGAGCATCTTTGGCGAGATCGGGAGTATCGGTGGCATTGGTGTCTCCTA 60

111_213 CATGACCACTAGGAGCATCTTTGGCGAGATCGGGAGAATCGGTGCCATTGGTGTCTCCTA 60

113_208 CATGACCACTAGGAGCATCTTTGGCGAGATCGGGAGAATCGGTGGCCTTGGTGTCTCCTA 60

117_179 CATGACCACTAGGAGCATCTTTGGCGAGATCCGGAGAATCGGTGGCATTGGTGTCTCCTA 60

119_177 CATGACCACTAGGAGCATCTTTGGCGAGAACGGGAGAATCGGTGGCATTGGTGTCTCCTA 60

123_169 CATGACCACTAGGAGCATCTTTGGCGAGATCGGGAGAATCGGTGGCATTGCTGTCTCCTA 60

124_168 CATGACCACTAGGAGCATCTTTGGCGAGATCGGGAGAATCGGTGGCATTGGTGTATCCTA 60

132_147 CATGACCACTAGGAGCATCTTTGGCGAGATCGGGTGAATCGGTGGCATTGGTGTCTCCTA 60

138_133 CATGACCACTAGGAGCATCTTTGGCGAGATCGGGCGAATCGGTGGCATTGGTGTCTCCTA 60

139_132 CATGACCACTAGGAGCATCTTTGGCGAGATCGGGAGAATCGGTGGGATTGGTGTCTCCTA 60

143_120 CATGACCACTAGGAGCATCTTTGGCGAGAGCGGGAGAATCGGTGGCATTGGTGTCTCCTA 60

147_114 CATGACCACTAGGAGCATCTTTGGCGAGATCGGGAGAATCGGTGGCATTCGTGTCTCCTA 60

148_111 CATGACCACTAGGAGCATCTTTGGCGAGATCGGGACAATCGGTGGCATTGGTGTCTCCTA 60

153_105 CATGACCACTAGGAGCATCTTTGGCGAGATCGGGAGAATCGGTGGCATGGGTGTCTCCTA 60

155_102 CATGACCACTAGGAGCATCTTTGGCGAGATCGGGAGAATCGGTGGCATTGGTCTCTCCTA 60

160_98 CATGACCACTAGGAGCATCTTTGGCGAGATCGGGAGAATCGGTGGCATTGGGGTCTCCTA 60

171_83 CATGACCACTAGGAGCATCTTTGGCGAGATCGGGAGAATCGGTGGCATTGGTGGCTCCTA 60

172_83 CATGACCACTAGGAGCATCTTTGGCGAGATCGGGAGAATCGGGGGCATTGGTGTCTCCTA 60

178_77 CATGACCACTAGGAGCATCTTTGGCGAGATGGGGAGAATCGGTGGCATTGGTGTCTCCTA 60

179_77 CATGACCACTAGGAGCATCTTTGGCGAGATCGGGAGAATCGGTGGCTTTGGTGTCTCCTA 60

182_75 CATGACCACTAGGAGCATCTTTGGCGAGATCGGGAGAATCGGTCGCATTGGTGTCTCCTA 60

188_70 CATGACCACTAGGAGCATCTTTGGCGAGATCGGGAGAATCCGTGGCATTGGTGTCTCCTA 60

228_47 CATGACCACTAGGAGCATCTTTGGCGAGATCGGGAGCATCGGTGGCATTGGTGTCTCCTA 60

244_41 CATGACCACTAGGAGCATCTTTGGCGAGATCGGGAGAATGGGTGGCATTGGTGTCTCCTA 60

245_40 CATGACCACTAGGAGCATCTTTGGCGAGATCGGGAGAATCGGTGGCATTGGTGTGTCCTA 60

312_28 CATGACCACTAGGAGCATCTTTGGCGAGATCGGGAGAAGCGGTGGCATTGGTGTCTCCTA 60

1_892954 CATGACCACTAGGAGCATCTTTGGCGAGATCGGGAGAATCGGTGGCATTGGTGTCTCCTA 60

415_15 CATGACCACTAGGAGCATCTTTGGCGAGATCGGGAGATTCGGTGGCATTGGTGTCTCCTA 60

73_396 CATGACCACTAGGAGCATCTTTGGCGAGATCGGGAGACTCGGTGGCATTGGTGTCTCCTA 60

29_1104 CATGACCACTAGGAGCATCTTTGGCGAGATCGGGAGAGTCGGTGGCATTGGTGTCTCCTA 60

773_5 CATGACCACTAGGAGCATCTTTGGCGAGATCGGGGGACTCGGTGGCATTGGTGTCTCCTA 60

822_5 CATGACCACTAGGAGCATCTTTGGCGAGATCTGGGGAATCGGTGGCATTGGTGTCTCCTA 60

779_5 CATGACCACTAGGAGCATCTTTGGCGAGATCGGGGGAATTGGTGGCATTGGTGTCTCCTA 60

350_21 CATGACCACTAGGAGCATCTTTGGCGAGATCGGGGGAATCGGCGGCATTGGTGTCTCCTA 60

610_7 CATGACCACTAGGAGCATCTTTGGCGAGATCGGAGGAATCGGTGGCATTGGTGTCTCCTA 60

986_4 CATGACCACTAGGAGCATCTTTGGCGAGATCGGGAGAATCGGTGGCACCGGTGTCTCCTA 60

852_5 CATGACCACTAGGAGCATCTTTGGCGAGATCGGGAGAATCGGTGGCATCGGTGTTTCCTA 60

848_5 CATGACCACTAGGAGCATCTTTGGCGAGATCGGAAGAATCGGTGGCATCGGTGTCTCCTA 60

429_15 CATGACCACTAGGAGCATCTTTGGCGAGATCGGGAGAATCGGTAACATTGGTGTCTCCTA 60

952_4 CATGACCACTAGGAGCATCTTTGGCGAGATCAGGAGAATCGGTAGCATTGGTGTCTCCTA 60

754_6 CATGACCACTAGGAGCATCTTTGGCGAGATCAGGAGAATCGGTGGCATCGGTGTCTCCTA 60

717_6 CATGACCACTAGGAGCATCTTTGGCGAGATCGGGAGAATCGTAGGCATTGGTGTCTCCTA 60

594_8 CATGACCACTAGGAGCATCTTTGGCGAGATCGGGAGAATCGTCGGCATTGGTGTCTCCTA 60

718_6 CATGACCACTAGGAGCATCTTTGGCGAGATCGGGAGAAACGGAGGCATTGGTGTCTCCTA 60

966_4 CATGACCACTAGGAGCATCTTTGGCGAGATCGGGAGAATCGGTGGCATTGGAGACTCCTA 60

950_4 CATGACCACTAGGAGCATCTTTGGCGAGATCGGGAGAATCGGTGGCATTGCAGTCTCCTA 60

926_4 CATGACCACTAGGAGCATCTTTGGCGAGATCGGGAGAATCGTTGGCATTGGAGTCTCCTA 60

856_5 CATGACCACTAGGAGCATCTTTGGCGAGATCGGGAGAAACGGTGGCATTGGAGTCTCCTA 60

808_5 CATGACCACTAGGAGCATCTTTGGCGAGATCGGGAGAATCGGTGGCAATGGAGTCTCCTA 60

705_6 CATGACCACTAGGAGCATCTTTGGCGAGATCGGTAGAATCGGTGGCATTGGAGTCTCCTA 60

494_11 CATGACCACTAGGAGCATCTTTGGCGAGATCGGTAGAATCGGCGGCATTGGTGTCTCCTA 60

558_9 CATGACCACTAGGAGCATCTTTGGCGAGATAGGGAGAATCGGTGGCATTTGTGTCTCCTA 60

823_5 CATGACCACTAGGAGCATCTTTGGCGAGATTGGAAGAATCGGTGGCATTGGTGTCTCCTA 60

956_4 CATGACCACTAGGAGCATCTTTGGCGAGATAGGTAGAATCGGTGGCATTGGTGTCTCCTA 60

795_5 CATGACCACTAGGAGCATCTTTGGCGAGATTGGTAGAATCGGTGGCATTGGTGTCTCCTA 60

367_19 CATGACCACTAGGAGCATCTTTGGCGAGATCGGGAGAGTCGGCGGCATTGGTGTCTCCTA 60

413_15 CATGACCACTAGGAGCATCTTTGGCGAGATTGGGAGAATTGGTGGCATTGGTGTCTCCTA 60

572_8 CATGACCACTAGGAGCATCTTTGGCGAGATTGGGAGAATCGGTGGTATTGGTGTCTCCTA 60

625_7 CATGACCACTAGGAGCATCTTTGGCGAGATCGGGAGAAACGGTGGCATAGGTGTCTCCTA 60

640_7 CATGACCACTAGGAGCATCTTTGGCGAGATTGGGAGAATCGGTGGCATTGGTGCCTCCTA 60

745_6 CATGACCACTAGGAGCATCTTTGGCGAGATCGGGAGAATCGGTGGCATTAATGTCTCCTA 60

751_6 CATGACCACTAGGAGCATCTTTGGCGAGATCGGGAGAATCGGTGGCATAGGAGTCTCCTA 60

789_5 CATGACCACTAGGAGCATCTTTGGCGAGATTGGGAGAATCGGTGGCATTAGTGTCTCCTA 60

892_4 CATGACCACTAGGAGCATCTTTGGCGAGATCGGGAGAATCGGTGGCATAGGTGTATCCTA 60

993_4 CATGACCACTAGGAGCATCTTTGGCGAGATTGGGAGAATCGGTGGCATAGGTGTCTCCTA 60

545_9 CATGACCACTAGGAGCATCTTTGGCGAGATTGGGAGAATCGGTGGCATCGGTGTCTCCTA 60

975_4 CATGACCACTAGGAGCATCTTTGGCGAGATTGGGAGAGTCGGTGGCATTGGTGTCTCCTA 60

850_5 CATGACCACTAGGAGCATCTTTGGCGAGATTGGGAGAATCGGTGGCATTGGCGTCTCCTA 60

820_5 CATGACCACTAGGAGCATCTTTGGCGAGATTGGGAGGATCGGTGGCATTGGTGTCTCCTA 60

764_5 CATGACCACTAGGAGCATCTTTGGCGAGATTGGGAGAATCGGTGACATTGGTGTCTCCTA 60

747_6 CATGACCACTAGGAGCATCTTTGGCGAGATTGGGAGAATCGGTGGCATTGGTGTTTCCTA 60

698_6 CATGACCACTAGGAGCATCTTTGGCGAGATTGGGAGAATCGGTGGCACTGGTGTCTCCTA 60

826_5 CATGACCACTAGGAGCATCTTTGGCGAGATCGGGAGAATCGGCTGCATTGGTGTCTCCTA 60

720_6 CATGACCACTAGGAGCATCTTTGGCGAGATCGGGAGAATCGGATGCATTGGTGTCTCCTA 60

357_21 CATGACCACTAGGAGCATCTTTGGCGAGATCGGGAGAATCGGCGGCATCGGTGTCTCCTA 60

770_5 CATGACCACTAGGAGCATCTTTGGCGAGATCGGGAGAATCGGCGGCATAGGTGTCTCCTA 60

740_6 CATGACCACTAGGAGCATCTTTGGCGAGATCGGGAGAATCGGAGGCATAGGTGTCTCCTA 60

803_5 CATGACCACTAGGAGCATCTTTGGCGAGATCGCGAGAATCGGCGGCATTGGTGTCTCCTA 60

936_4 CATGACCACTAGGAGCATCTTTGGCGAGATCGGGAGAATCGGCGGAATTGGTGTCTCCTA 60

949_4 CATGACCACTAGGAGCATCTTTGGCGAGATCGGGAGAATCGGGGGTATTGGTGTCTCCTA 60

369_19 CATGACCACTAGGAGCATCTTTGGCGAGATCGGGAGAATCGGCGGTATTGGTGTCTCCTA 60

309_28 CATGACCACTAGGAGCATCTTTGGCGAGATCGGGAGAATTGGCGGCATTGGTGTCTCCTA 60

323_26 CATGACCACTAGGAGCATCTTTGGCGAGATCGGGAGAATCGGCGGCATTGGTGTTTCCTA 60

389_17 CATGACCACTAGGAGCATCTTTGGCGAGATCGGGAGAATCGGCGGCATTGGTGCCTCCTA 60

506_11 CATGACCACTAGGAGCATCTTTGGCGAGATCGGGAGGATCGGCGGCATTGGTGTCTCCTA 60

806_5 CATGACCACTAGGAGCATCTTTGGCGAGATCGGGAGAATCGGCGACATTGGTGTCTCCTA 60

637_7 CATGACCACTAGGAGCATCTTTGGCGAGATCGGGAGAATCGGCGTCATTGGTGTCTCCTA 60

813_5 CATGACCACTAGGAGCATCTTTGGCGAGATCGGGAGAATCAGCGGCATTGGTGTCTCCTA 60

829_5 CATGACCACTAGGAGCATCTTTGGCGAGATCGGGAGAATCGGCGGCATTTGTGTCTCCTA 60

835_5 CATGACCACTAGGAGCATCTTTGGCGAGATCGGGAGAATCGGCGGCAGTGGTGTCTCCTA 60

836_5 CATGACCACTAGGAGCATCTTTGGCGAGATCGGGAGAATCGGCGGCGTTGGTGTCTCCTA 60

893_4 CATGACCACTAGGAGCATCTTTGGCGAGATCGGGAGAATCGGCGGCATTGGGGTCTCCTA 60

663_7 CATGACCACTAGGAGCATCTTTGGCGAGATCGGGAGAATCGGCGGCATTGGCGTCTCCTA 60

921_4 CATGACCACTAGGAGCATCTTTGGCGAGATCCGGAGAATCGGCGGCATTGGTGTCTCCTA 60

461_13 CATGACCACTAGGAGCATCTTTGGCGAGATCTGGAGAATCGGCGGCATTGGTGTCTCCTA 60

272_34 CATGACCACTAGGAGCATCTTTGGCGAGATCAGGAGAATCGGCGGCATTGGTGTCTCCTA 60

723_6 CATGACCACTAGGAGCATCTTTGGCGAGATTGGGAGAATCGGAGGCATTGGTGTCTCCTA 60

947_4 CATGACCACTAGGAGCATCTTTGGCGAGATAGGGAGAATCGGCGGCATTGGTGTCTCCTA 60

225_48 CATGACCACTAGGAGCATCTTTGGCGAGATTGGGAGAATCGGCGGCATTGGTGTCTCCTA 60

793_5 CATGACCACTAGGAGCATCTTTGGCGAGATCGGGAGAATCTGTGGCATAGGTGTCTCCTA 60

944_4 CATGACCACTAGGAGCATCTTTGGCGAGATCGGGAGAATCGGTGGCATAGGTGACTCCTA 60

934_4 CATGACCACTAGGAGCATCTTTGGCGAGATCGGGAGAAACGGTGGCATTGGTGACTCCTA 60

783_5 CATGACCACTAGGAGCATCTTTGGCGAGATCGGGAGAATCGGTGGAATTGGTGACTCCTA 60

535_10 CATGACCACTAGGAGCATCTTTGGCGAGATCGGGAGAATTGGTGGCATCGGTGTCTCCTA 60

672_6 CATGACCACTAGGAGCATCTTTGGCGAGATCGGGAGGATCGGTGGTATTGGTGTCTCCTA 60

964_4 CATGACCACTAGGAGCATCTTTGGCGAGATCGGGAGAATTGGTGGTATTGGTGTCTCCTA 60

932_4 CATGACCACTAGGAGCATCTTTGGCGAGATCGGAAGAATTGGTGGCATTGGTGTCTCCTA 60

882_5 CATGACCACTAGGAGCATCTTTGGCGAGATCGGGAGAGTTGGTGGCATTGGTGTCTCCTA 60

540_10 CATGACCACTAGGAGCATCTTTGGCGAGATCGGGAGACTTGGTGGCATTGGTGTCTCCTA 60

873_5 CATGACCACTAGGAGCATCTTTGGCGAGATCGCGAGAATTGGTGGCATTGGTGTCTCCTA 60

728_6 CATGACCACTAGGAGCATCTTTGGCGAGATCGAGAGAATTGGTGGCATTGGTGTCTCCTA 60

428_15 CATGACCACTAGGAGCATCTTTGGCGAGATCGTGAGAATTGGTGGCATTGGTGTCTCCTA 60

737_6 CATGACCACTAGGAGCATCTTTGGCGAGATCGGGAGAATTGGTGGCATTGGTGTTTCCTA 60

727_6 CATGACCACTAGGAGCATCTTTGGCGAGATCGGGAGGATTGGTGGCATTGGTGTCTCCTA 60

681_6 CATGACCACTAGGAGCATCTTTGGCGAGATCGGGAGAATTGGTGGCATTGATGTCTCCTA 60

677_6 CATGACCACTAGGAGCATCTTTGGCGAGATCGGGAGAATTGGTGGCATTGGTGCCTCCTA 60

500_11 CATGACCACTAGGAGCATCTTTGGCGAGATCGGGAGAATCAATGGCATTGGTGTCTCCTA 60

761_5 CATGACCACTAGGAGCATCTTTGGCGAGATCGGGAGAATCGGTGTCATTGTTGTCTCCTA 60

857_5 CATGACCACTAGGAGCATCTTTGGCGAGATCGGGAGAATCGGTGGACTTGGTGTCTCCTA 60

888_4 CATGACCACTAGGAGCATCTTTGGCGAGATCGGGAGAATCTGTGGCATTGGTTTCTCCTA 60

908_4 CATGACCACTAGGAGCATCTTTGGCGAGATCGGGAGAATCGGTGGAATTGTTGTCTCCTA 60

989_4 CATGACCACTAGGAGCATCTTTGGCGAGATCGGGAGAATCTGTGGCATTGTTGTCTCCTA 60

918_4 CATGACCACTAGGAGCATCTTTGGCGAGATCGGGAGAATCTGTGGCATTGGTGACTCCTA 60

798_5 CATGACCACTAGGAGCATCTTTGGCGAGATCTGGAGAATCTGTGGCATTGGTGTCTCCTA 60

767_5 CATGACCACTAGGAGCATCTTTGGCGAGATCGGGAGAATCTGTGGCATTGGTGTTTCCTA 60

839_5 CATGACCACTAGGAGCATCTTTGGCGAGATCGAGAGAAACGGTGGCATTGGTGTCTCCTA 60

624_7 CATGACCACTAGGAGCATCTTTGGCGAGATCGTGAGAATCGGTGGCATAGGTGTCTCCTA 60

953_4 CATGACCACTAGGAGCATCTTTGGCGAGATCGAGAGAATCGGTGGCATCGGTGTCTCCTA 60

563_9 CATGACCACTAGGAGCATCTTTGGCGAGATCGTGAGAATCGGTGGCATCGGTGTCTCCTA 60

446_14 CATGACCACTAGGAGCATCTTTGGCGAGATCGAGAGAATCGGCGGCATTGGTGTCTCCTA 60

291_32 CATGACCACTAGGAGCATCTTTGGCGAGATCGTGAGAATCGGCGGCATTGGTGTCTCCTA 60

777_5 CATGACCACTAGGAGCATCTTTGGCGAGATCGGTAGAATCGGTGGCATTGGTGTTTCCTA 60

913_4 CATGACCACTAGGAGCATCTTTGGCGAGATCGGTAGAATCGGTGTCATTGGTGTCTCCTA 60

268_35 CATGACCACTAGGAGCATCTTTGGCGAGATCGAAAGAATCGGTGGCATTGGTGTCTCCTA 60

923_4 CATGACCACTAGGAGCATCTTTGGCGAGATCGTTAGAATCGGTGGCATTGGTGTCTCCTA 60

571_8 CATGACCACTAGGAGCATCTTTGGCGAGATCGTAAGAATCGGTGGCATTGGTGTCTCCTA 60

738_6 CATGACCACTAGGAGCATCTTTGGCGAGATCGTGAGAATCGGTGGCGTTGGTGTCTCCTA 60

702_6 CATGACCACTAGGAGCATCTTTGGCGAGATCGAGAGAATCGGTGGCGTTGGTGTCTCCTA 60

375_18 CATGACCACTAGGAGCATCTTTGGCGAGATTGTGAGAATCGGTGGCATTGGTGTCTCCTA 60

618_7 CATGACCACTAGGAGCATCTTTGGCGAGATCAGGAGAATTGGTGGCATTGGTGTCTCCTA 60

649_7 CATGACCACTAGGAGCATCTTTGGCGAGATCGTGAGAATCGGTGGCATTGGCGTCTCCTA 60

682_6 CATGACCACTAGGAGCATCTTTGGCGAGATCGTGAGAATCGGTGTCATTGGTGTCTCCTA 60

686_6 CATGACCACTAGGAGCATCTTTGGCGAGATCGAGAGGATCGGTGGCATTGGTGTCTCCTA 60

621_7 CATGACCACTAGGAGCATCTTTGGCGAGATCGTGAGGATCGGTGGCATTGGTGTCTCCTA 60

752_6 CATGACCACTAGGAGCATCTTTGGCGAGATCGGAAGAATCGGTGGCATTGGTGCCTCCTA 60

758_5 CATGACCACTAGGAGCATCTTTGGCGAGATCGTGAGAATCGGTGGCATTGGTGCCTCCTA 60

768_5 CATGACCACTAGGAGCATCTTTGGCGAGATCGCGAGAATCGGTGGCATTGGTGTTTCCTA 60

444_14 CATGACCACTAGGAGCATCTTTGGCGAGATCGTGAGAATCGGTGGCATTGGTGTTTCCTA 60

771_5 CATGACCACTAGGAGCATCTTTGGCGAGATCGTGAGAATCGGTGGTATTGGTGTCTCCTA 60

775_5 CATGACCACTAGGAGCATCTTTGGCGAGATCAGAAGAATCGGTGGCATTGGTGTCTCCTA 60

809_5 CATGACCACTAGGAGCATCTTTGGCGAGATCTGGAGAATCGGTGGCATTTGTGTCTCCTA 60

922_4 CATGACCACTAGGAGCATCTTTGGCGAGATCGTGAGAATCGGTGGCACTGGTGTCTCCTA 60

930_4 CATGACCACTAGGAGCATCTTTGGCGAGATCGGGAGGATCGGTGGCATTGGTGCCTCCTA 60

979_4 CATGACCACTAGGAGCATCTTTGGCGAGATCAGGAGAATCGGTGGCATTGGTGTTTCCTA 60

984_4 CATGACCACTAGGAGCATCTTTGGCGAGATCGGGAGACTCGGTGGCATTGGTGCCTCCTA 60

990_4 CATGACCACTAGGAGCATCTTTGGCGAGATCGTGAGACTCGGTGGCATTGGTGTCTCCTA 60

880_5 CATGACCACTAGGAGCATCTTTGGCGAGATCGTGAGAGTCGGTGGCATTGGTGTCTCCTA 60

333_24 CATGACCACTAGGAGCATCTTTGGCGAGATCAAGAGAATCGGTGGCATTGGTGTCTCCTA 60

960_4 CATGACCACTAGGAGCATCTTTGGCGAGATCATGAGAATCGGTGGCATTGGTGTCTCCTA 60

959_4 CATGACCACTAGGAGCATCTTTGGCGAGATCTTGAGAATCGGTGGCATTGGTGTCTCCTA 60

***************************** *****

5_7863 GGGGAATAAATCTTTGGGCACCTAGTGGTCATG 93

6_4354 GGGGAATAAATCTTTGGGCACCTAGTGGTCATG 93

11_3283 GGGGAATAAATCTTTGGGCACCTAGTGGTCATG 93

12_2781 GGGGAATAAATCTTTGGGCACCTAGTGGTCATG 93

14_2397 GGGGAATAAATCTTTGGGCACCTAGTGGTCATG 93

15_2111 GGGGAATAAATCTTTGGGCACCTAGTGGTCATG 93

17_1565 GGGGAATAAATCTTTGGGCACCTAGTGGTCATG 93

18_1555 GGGGAATAAATCTTTGGGCACCTAGTGGTCATG 93

20_1482 GGGGAATAAATCTTTGGGCACCTAGTGGTCATG 93

22_1449 GGGGAATAAATCTTTGGGCACCTAGTGGTCATG 93

24_1332 GGGGAATAAATCTTTGGGCACCTAGTGGTCATG 93

26_1257 GGGGAATAAATCTTTGGGCACCTAGTGGTCATG 93

30_1081 GGGGAATAAATCTTTGGGCACCTAGTGGTCATG 93

32_1013 GGGGAATAAATCTTTGGGCACCTAGTGGTCATG 93

33_1011 GGGGAATAAATCTTTGGGCACCTAGTGGTCATG 93

34_963 GGGGAATAAATCTTTGGGCACCTAGTGGTCATG 93

35_938 GGGGAATAAATCTTTGGGCACCTAGTGGTCATG 93

40_706 GGGGAATAAATCTTTGGGCACCTAGTGGTCATG 93

45_629 GGGGAATAAATCTTTGGGCACCTAGTGGTCATG 93

46_618 GGGGAATAAATCTTTGGGCACCTAGTGGTCATG 93

49_559 GGGGAATAAATCTTTGGGCACCTAGTGGTCATG 93

50_552 GGGGAATAAATCTTTGGGCACCTAGTGGTCATG 93

52_534 GGGGAATAAATCTTTGGGCACCTAGTGGTCATG 93

53_527 GGGGAATAAATCTTTGGGCACCTAGTGGTCATG 93

55_526 GGGGAATAAATCTTTGGGCACCTAGTGGTCATG 93

57_518 GGGGAATAAATCTTTGGGCACCTAGTGGTCATG 93

59_481 GGGGAATAAATCTTTGGGCACCTAGTGGTCATG 93

61_454 GGGGAATAAATCTTTGGGCACCTAGTGGTCATG 93

62_452 GGGGAATAAATCTTTGGGCACCTAGTGGTCATG 93

63_447 GGGGAATAAATCTTTGGGCACCTAGTGGTCATG 93

67_430 GGGGAATAAATCTTTGGGCACCTAGTGGTCATG 93

69_427 GGGGAATAAATCTTTGGGCACCTAGTGGTCATG 93

75_386 GGGGAATAAATCTTTGGGCACCTAGTGGTCATG 93

77_378 GGGGAATAAATCTTTGGGCACCTAGTGGTCATG 93

78_370 GGGGAATAAATCTTTGGGCACCTAGTGGTCATG 93

79_369 GGGGAATAAATCTTTGGGCACCTAGTGGTCATG 93

81_364 GGGGAATAAATCTTTGGGCACCTAGTGGTCATG 93

84_354 GGGGAATAAATCTTTGGGCACCTAGTGGTCATG 93

87_343 GGGGAATAAATCTTTGGGCACCTAGTGGTCATG 93

89_330 GGGGAATAAATCTTTGGGCACCTAGTGGTCATG 93

90_327 GGGGAATAAATCTTTGGGCACCTAGTGGTCATG 93

92_310 GGGGAATAAATCTTTGGGCACCTAGTGGTCATG 93

93_307 GGGGAATAAATCTTTGGGCACCTAGTGGTCATG 93

94_296 GGGGAATAAATCTTTGGGCACCTAGTGGTCATG 93

95_285 GGGGAATAAATCTTTGGGCACCTAGTGGTCATG 93

96_283 GGGGAATAAATCTTTGGGCACCTAGTGGTCATG 93

97_272 GGGGAATAAATCTTTGGGCACCTAGTGGTCATG 93

99_259 GGGGAATAAATCTTTGGGCACCTAGTGGTCATG 93

105_226 GGGGAATAAATCTTTGGGCACCTAGTGGTCATG 93

108_222 GGGGAATAAATCTTTGGGCACCTAGTGGTCATG 93

111_213 GGGGAATAAATCTTTGGGCACCTAGTGGTCATG 93

113_208 GGGGAATAAATCTTTGGGCACCTAGTGGTCATG 93

117_179 GGGGAATAAATCTTTGGGCACCTAGTGGTCATG 93

119_177 GGGGAATAAATCTTTGGGCACCTAGTGGTCATG 93

123_169 GGGGAATAAATCTTTGGGCACCTAGTGGTCATG 93

124_168 GGGGAATAAATCTTTGGGCACCTAGTGGTCATG 93

132_147 GGGGAATAAATCTTTGGGCACCTAGTGGTCATG 93

138_133 GGGGAATAAATCTTTGGGCACCTAGTGGTCATG 93

139_132 GGGGAATAAATCTTTGGGCACCTAGTGGTCATG 93

143_120 GGGGAATAAATCTTTGGGCACCTAGTGGTCATG 93

147_114 GGGGAATAAATCTTTGGGCACCTAGTGGTCATG 93

148_111 GGGGAATAAATCTTTGGGCACCTAGTGGTCATG 93

153_105 GGGGAATAAATCTTTGGGCACCTAGTGGTCATG 93

155_102 GGGGAATAAATCTTTGGGCACCTAGTGGTCATG 93

160_98 GGGGAATAAATCTTTGGGCACCTAGTGGTCATG 93

171_83 GGGGAATAAATCTTTGGGCACCTAGTGGTCATG 93

172_83 GGGGAATAAATCTTTGGGCACCTAGTGGTCATG 93

178_77 GGGGAATAAATCTTTGGGCACCTAGTGGTCATG 93

179_77 GGGGAATAAATCTTTGGGCACCTAGTGGTCATG 93

182_75 GGGGAATAAATCTTTGGGCACCTAGTGGTCATG 93

188_70 GGGGAATAAATCTTTGGGCACCTAGTGGTCATG 93

228_47 GGGGAATAAATCTTTGGGCACCTAGTGGTCATG 93

244_41 GGGGAATAAATCTTTGGGCACCTAGTGGTCATG 93

245_40 GGGGAATAAATCTTTGGGCACCTAGTGGTCATG 93

312_28 GGGGAATAAATCTTTGGGCACCTAGTGGTCATG 93

1_892954 GGGGAATAAATCTTTGGGCACCTAGTGGTCATG 93

415_15 GGGGAATAAATCTTTGGGCACCTAGTGGTCATG 93

73_396 GGGGAATAAATCTTTGGGCACCTAGTGGTCATG 93

29_1104 GGGGAATAAATCTTTGGGCACCTAGTGGTCATG 93

773_5 GGGGAATAAATCTTTGGGCACCTAGTGGTCATG 93

822_5 GGGGAATAAATCTTTGGGCACCTAGTGGTCATG 93

779_5 GGGGAATAAATCTTTGGGCACCTAGTGGTCATG 93

350_21 GGGGAATAAATCTTTGGGCACCTAGTGGTCATG 93

610_7 GGGGAATAAATCTTTGGGCACCTAGTGGTCATG 93

986_4 GGGGAATAAATCTTTGGGCACCTAGTGGTCATG 93

852_5 GGGGAATAAATCTTTGGGCACCTAGTGGTCATG 93

848_5 GGGGAATAAATCTTTGGGCACCTAGTGGTCATG 93

429_15 GGGGAATAAATCTTTGGGCACCTAGTGGTCATG 93

952_4 GGGGAATAAATCTTTGGGCACCTAGTGGTCATG 93

754_6 GGGGAATAAATCTTTGGGCACCTAGTGGTCATG 93

717_6 GGGGAATAAATCTTTGGGCACCTAGTGGTCATG 93

594_8 GGGGAATAAATCTTTGGGCACCTAGTGGTCATG 93

718_6 GGGGAATAAATCTTTGGGCACCTAGTGGTCATG 93

966_4 GGGGAATAAATCTTTGGGCACCTAGTGGTCATG 93

950_4 GGGGAATAAATCTTTGGGCACCTAGTGGTCATG 93

926_4 GGGGAATAAATCTTTGGGCACCTAGTGGTCATG 93

856_5 GGGGAATAAATCTTTGGGCACCTAGTGGTCATG 93

808_5 GGGGAATAAATCTTTGGGCACCTAGTGGTCATG 93

705_6 GGGGAATAAATCTTTGGGCACCTAGTGGTCATG 93

494_11 GGGGAATAAATCTTTGGGCACCTAGTGGTCATG 93

558_9 GGGGAATAAATCTTTGGGCACCTAGTGGTCATG 93

823_5 GGGGAATAAATCTTTGGGCACCTAGTGGTCATG 93

956_4 GGGGAATAAATCTTTGGGCACCTAGTGGTCATG 93

795_5 GGGGAATAAATCTTTGGGCACCTAGTGGTCATG 93

367_19 GGGGAATAAATCTTTGGGCACCTAGTGGTCATG 93

413_15 GGGGAATAAATCTTTGGGCACCTAGTGGTCATG 93

572_8 GGGGAATAAATCTTTGGGCACCTAGTGGTCATG 93

625_7 GGGGAATAAATCTTTGGGCACCTAGTGGTCATG 93

640_7 GGGGAATAAATCTTTGGGCACCTAGTGGTCATG 93

745_6 GGGGAATAAATCTTTGGGCACCTAGTGGTCATG 93

751_6 GGGGAATAAATCTTTGGGCACCTAGTGGTCATG 93

789_5 GGGGAATAAATCTTTGGGCACCTAGTGGTCATG 93

892_4 GGGGAATAAATCTTTGGGCACCTAGTGGTCATG 93

993_4 GGGGAATAAATCTTTGGGCACCTAGTGGTCATG 93

545_9 GGGGAATAAATCTTTGGGCACCTAGTGGTCATG 93

975_4 GGGGAATAAATCTTTGGGCACCTAGTGGTCATG 93

850_5 GGGGAATAAATCTTTGGGCACCTAGTGGTCATG 93

820_5 GGGGAATAAATCTTTGGGCACCTAGTGGTCATG 93

764_5 GGGGAATAAATCTTTGGGCACCTAGTGGTCATG 93

747_6 GGGGAATAAATCTTTGGGCACCTAGTGGTCATG 93

698_6 GGGGAATAAATCTTTGGGCACCTAGTGGTCATG 93

826_5 GGGGAATAAATCTTTGGGCACCTAGTGGTCATG 93

720_6 GGGGAATAAATCTTTGGGCACCTAGTGGTCATG 93

357_21 GGGGAATAAATCTTTGGGCACCTAGTGGTCATG 93

770_5 GGGGAATAAATCTTTGGGCACCTAGTGGTCATG 93

740_6 GGGGAATAAATCTTTGGGCACCTAGTGGTCATG 93

803_5 GGGGAATAAATCTTTGGGCACCTAGTGGTCATG 93

936_4 GGGGAATAAATCTTTGGGCACCTAGTGGTCATG 93

949_4 GGGGAATAAATCTTTGGGCACCTAGTGGTCATG 93

369_19 GGGGAATAAATCTTTGGGCACCTAGTGGTCATG 93

309_28 GGGGAATAAATCTTTGGGCACCTAGTGGTCATG 93

323_26 GGGGAATAAATCTTTGGGCACCTAGTGGTCATG 93

389_17 GGGGAATAAATCTTTGGGCACCTAGTGGTCATG 93

506_11 GGGGAATAAATCTTTGGGCACCTAGTGGTCATG 93

806_5 GGGGAATAAATCTTTGGGCACCTAGTGGTCATG 93

637_7 GGGGAATAAATCTTTGGGCACCTAGTGGTCATG 93

813_5 GGGGAATAAATCTTTGGGCACCTAGTGGTCATG 93

829_5 GGGGAATAAATCTTTGGGCACCTAGTGGTCATG 93

835_5 GGGGAATAAATCTTTGGGCACCTAGTGGTCATG 93

836_5 GGGGAATAAATCTTTGGGCACCTAGTGGTCATG 93

893_4 GGGGAATAAATCTTTGGGCACCTAGTGGTCATG 93

663_7 GGGGAATAAATCTTTGGGCACCTAGTGGTCATG 93

921_4 GGGGAATAAATCTTTGGGCACCTAGTGGTCATG 93

461_13 GGGGAATAAATCTTTGGGCACCTAGTGGTCATG 93

272_34 GGGGAATAAATCTTTGGGCACCTAGTGGTCATG 93

723_6 GGGGAATAAATCTTTGGGCACCTAGTGGTCATG 93

947_4 GGGGAATAAATCTTTGGGCACCTAGTGGTCATG 93

225_48 GGGGAATAAATCTTTGGGCACCTAGTGGTCATG 93

793_5 GGGGAATAAATCTTTGGGCACCTAGTGGTCATG 93

944_4 GGGGAATAAATCTTTGGGCACCTAGTGGTCATG 93

934_4 GGGGAATAAATCTTTGGGCACCTAGTGGTCATG 93

783_5 GGGGAATAAATCTTTGGGCACCTAGTGGTCATG 93

535_10 GGGGAATAAATCTTTGGGCACCTAGTGGTCATG 93

672_6 GGGGAATAAATCTTTGGGCACCTAGTGGTCATG 93

964_4 GGGGAATAAATCTTTGGGCACCTAGTGGTCATG 93

932_4 GGGGAATAAATCTTTGGGCACCTAGTGGTCATG 93

882_5 GGGGAATAAATCTTTGGGCACCTAGTGGTCATG 93

540_10 GGGGAATAAATCTTTGGGCACCTAGTGGTCATG 93

873_5 GGGGAATAAATCTTTGGGCACCTAGTGGTCATG 93

728_6 GGGGAATAAATCTTTGGGCACCTAGTGGTCATG 93

428_15 GGGGAATAAATCTTTGGGCACCTAGTGGTCATG 93

737_6 GGGGAATAAATCTTTGGGCACCTAGTGGTCATG 93

727_6 GGGGAATAAATCTTTGGGCACCTAGTGGTCATG 93

681_6 GGGGAATAAATCTTTGGGCACCTAGTGGTCATG 93

677_6 GGGGAATAAATCTTTGGGCACCTAGTGGTCATG 93

500_11 GGGGAATAAATCTTTGGGCACCTAGTGGTCATG 93

761_5 GGGGAATAAATCTTTGGGCACCTAGTGGTCATG 93

857_5 GGGGAATAAATCTTTGGGCACCTAGTGGTCATG 93

888_4 GGGGAATAAATCTTTGGGCACCTAGTGGTCATG 93

908_4 GGGGAATAAATCTTTGGGCACCTAGTGGTCATG 93

989_4 GGGGAATAAATCTTTGGGCACCTAGTGGTCATG 93

918_4 GGGGAATAAATCTTTGGGCACCTAGTGGTCATG 93

798_5 GGGGAATAAATCTTTGGGCACCTAGTGGTCATG 93

767_5 GGGGAATAAATCTTTGGGCACCTAGTGGTCATG 93

839_5 GGGGAATAAATCTTTGGGCACCTAGTGGTCATG 93

624_7 GGGGAATAAATCTTTGGGCACCTAGTGGTCATG 93

953_4 GGGGAATAAATCTTTGGGCACCTAGTGGTCATG 93

563_9 GGGGAATAAATCTTTGGGCACCTAGTGGTCATG 93

446_14 GGGGAATAAATCTTTGGGCACCTAGTGGTCATG 93

291_32 GGGGAATAAATCTTTGGGCACCTAGTGGTCATG 93

777_5 GGGGAATAAATCTTTGGGCACCTAGTGGTCATG 93

913_4 GGGGAATAAATCTTTGGGCACCTAGTGGTCATG 93

268_35 GGGGAATAAATCTTTGGGCACCTAGTGGTCATG 93

923_4 GGGGAATAAATCTTTGGGCACCTAGTGGTCATG 93

571_8 GGGGAATAAATCTTTGGGCACCTAGTGGTCATG 93

738_6 GGGGAATAAATCTTTGGGCACCTAGTGGTCATG 93

702_6 GGGGAATAAATCTTTGGGCACCTAGTGGTCATG 93

375_18 GGGGAATAAATCTTTGGGCACCTAGTGGTCATG 93

618_7 GGGGAATAAATCTTTGGGCACCTAGTGGTCATG 93

649_7 GGGGAATAAATCTTTGGGCACCTAGTGGTCATG 93

682_6 GGGGAATAAATCTTTGGGCACCTAGTGGTCATG 93

686_6 GGGGAATAAATCTTTGGGCACCTAGTGGTCATG 93

621_7 GGGGAATAAATCTTTGGGCACCTAGTGGTCATG 93

752_6 GGGGAATAAATCTTTGGGCACCTAGTGGTCATG 93

758_5 GGGGAATAAATCTTTGGGCACCTAGTGGTCATG 93

768_5 GGGGAATAAATCTTTGGGCACCTAGTGGTCATG 93

444_14 GGGGAATAAATCTTTGGGCACCTAGTGGTCATG 93

771_5 GGGGAATAAATCTTTGGGCACCTAGTGGTCATG 93

775_5 GGGGAATAAATCTTTGGGCACCTAGTGGTCATG 93

809_5 GGGGAATAAATCTTTGGGCACCTAGTGGTCATG 93

922_4 GGGGAATAAATCTTTGGGCACCTAGTGGTCATG 93

930_4 GGGGAATAAATCTTTGGGCACCTAGTGGTCATG 93

979_4 GGGGAATAAATCTTTGGGCACCTAGTGGTCATG 93

984_4 GGGGAATAAATCTTTGGGCACCTAGTGGTCATG 93

990_4 GGGGAATAAATCTTTGGGCACCTAGTGGTCATG 93

880_5 GGGGAATAAATCTTTGGGCACCTAGTGGTCATG 93

333_24 GGGGAATAAATCTTTGGGCACCTAGTGGTCATG 93

960_4 GGGGAATAAATCTTTGGGCACCTAGTGGTCATG 93

959_4 GGGGAATAAATCTTTGGGCACCTAGTGGTCATG 93

*********************************

**2. CLUSTAL multiple sequence alignment for II-R1-3 (3_57642)**

CLUSTAL multiple sequence alignment

237_44 CATGACCACTAGGAGCATCTTTGGCGAGAAGACTCTGGATTCGGGGATTAGTTGCTGCTA 60

411_15 CATGACCACTAGGAGCATCTTTGGCGAGAAGACCTTGGATTCGGGGACCAGTTGCTGCTA 60

501_11 CATGACCACTAGGAGCATCTTTGGCGAGAAGACTTCGGATTCGGGGACCAGTTGCTGCTA 60

582_8 CATGACCACTAGGAGCATCTTTGGCGAGAAGACTTTGGATTCGGGGACTAGTTGCTGCTA 60

734_6 CATGACCACTAGGAGCATCTTTGGCGAGAAGACTTTGGATTTGGGGACCAGTTGCTGCTA 60

884_4 CATGACCACTAGGAGCATCTTTGGCGAGAAGACTTTGGATTCGGGGATCAGTTGCTGCTA 60

899_4 CATGACCACTAGGAGCATCTTTGGCGAGAAGACTCCGGATTCGGGGACCAGTTACTGCTA 60

983_4 CATGACCACTAGGAGCATCTTTGGCGAGAAGACCCTGGATTCGGGGACTAGTTGCTGCTA 60

37_907 CATGACCACTAGGAGCATCTTTGGCGAGAAGACTTTGGATTCGGGGACCAGTTGCTGCTA 60

56_520 CATGACCACTAGGAGCATCTTTGGCGAGAAGACTCCGGATTCGGGGACCAGTTGCTGCTA 60

83_354 CATGACCACTAGGAGCATCTTTGGCGAGAAGACTCTGGATTCGGGGACTAGTTGCTGCTA 60

100_254 CATGACCACTAGGAGCATCTTTGGCGAGAAGACCCTGGATTCGGGGACCAGTTGCTGCTA 60

101_252 CATGACCACTAGGAGCATCTTTGGCGAGAAGACTCTGTATTCGGGGACCAGTTGCTGCTA 60

109_220 CATGACCACTAGGAGCATCTTTGGCGAGAAGACTCTGGATTTGGGGACCAGTTGCTGCTA 60

116_185 CATGACCACTAGGAGCATCTTTGGCGAGAAGACTCTGGATTCGGGGACCAGTTGTTGCTA 60

120_170 CATGACCACTAGGAGCATCTTTGGCGAGAAGACTCAGGATTCGGGGACCAGTTGCTGCTA 60

141_127 CATGACCACTAGGAGCATCTTTGGCGAGAAGATTCTGGATTCGGGGACCAGTTGCTGCTA 60

142_125 CATGACCACTAGGAGCATCTTTGGCGAGAAGACTCTGGATTCGGGGATCAGTTGCTGCTA 60

156_102 CATGACCACTAGGAGCATCTTTGGCGAGAAGACTCTGGATTCGGGGGCCAGTTGCTGCTA 60

163_91 CATGACCACTAGGAGCATCTTTGGCGAGAATACTCTGGATTCGGGGACCAGTTGCTGCTA 60

165_88 CATGACCACTAGGAGCATCTTTGGCGAGAAGACTCTGGGTTCGGGGACCAGTTGCTGCTA 60

180_76 CATGACCACTAGGAGCATCTTTGGCGAGAAGGCTCTGGATTCGGGGACCAGTTGCTGCTA 60

187_71 CATGACCACTAGGAGCATCTTTGGCGAGAAGACTCTGGATTCGGGGACCGGTTGCTGCTA 60

194_65 CATGACCACTAGGAGCATCTTTGGCGAGAAGACTCTGGACTCGGGGACCAGTTGCTGCTA 60

208_56 CATGACCACTAGGAGCATCTTTGGCGAGAAGACTCTGGATTCGGGGACCAGCTGCTGCTA 60

212_55 CATGACCACTAGGAGCATCTTTGGCGAGAAGACTCTGGATTCGGGTACCAGTTGCTGCTA 60

216_52 CATGACCACTAGGAGCATCTTTGGCGAGAAGACTATGGATTCGGGGACCAGTTGCTGCTA 60

217_52 CATGACCACTAGGAGCATCTTTGGCGAGAAGACTCTGGATTCGGGGACCAGTCGCTGCTA 60

224_49 CATGACCACTAGGAGCATCTTTGGCGAGAGGACTCTGGATTCGGGGACCAGTTGCTGCTA 60

241_42 CATGACCACTAGGAGCATCTTTGGCGAGAAGACTCTGGATTCAGGGACCAGTTGCTGCTA 60

242_42 CATGACCACTAGGAGCATCTTTGGCGAGAAGACTCTGAATTCGGGGACCAGTTGCTGCTA 60

246_40 CATGACCACTAGGAGCATCTTTGGCGAGAAGACACTGGATTCGGGGACCAGTTGCTGCTA 60

251_39 CATGACCACTAGGAGCATCTTTGGCGAGAAGACTCTGGATTCGTGGACCAGTTGCTGCTA 60

254_37 CATGACCACTAGGAGCATCTTTGGCGAGAAGACGCTGGATTCGGGGACCAGTTGCTGCTA 60

262_36 CATGACCACTAGGAGCATCTTTGGCGAGAAGACTCTGGATTCGGGGACCAGATGCTGCTA 60

273_34 CATGACCACTAGGAGCATCTTTGGCGAGAAGACTCTAGATTCGGGGACCAGTTGCTGCTA 60

274_34 CATGACCACTAGGAGCATCTTTGGCGAGAAAACTCTGGATTCGGGGACCAGTTGCTGCTA 60

279_34 CATGACCACTAGGAGCATCTTTGGCGAGAAGACTGTGGATTCGGGGACCAGTTGCTGCTA 60

282_33 CATGACCACTAGGAGCATCTTTGGCGAGAAGACTCTGGATTCGGTGACCAGTTGCTGCTA 60

283_33 CATGACCACTAGGAGCATCTTTGGCGAGAAGACTCTGGATTCTGGGACCAGTTGCTGCTA 60

288_32 CATGACCACTAGGAGCATCTTTGGCGAGAAGACTCTGGAGTCGGGGACCAGTTGCTGCTA 60

293_31 CATGACCACTAGGAGCATCTTTGGCGAGAAGACTCTGGATTCGGGGACCAGTAGCTGCTA 60

296_31 CATGACCACTAGGAGCATCTTTGGCGAGAAGACTCTGGATCCGGGGACCAGTTGCTGCTA 60

301_29 CATGACCACTAGGAGCATCTTTGGCGAGAAGACTCTGGATTCGAGGACCAGTTGCTGCTA 60

311_28 CATGACCACTAGGAGCATCTTTGGCGAGAAGACTCTGGATTCGGGGACAAGTTGCTGCTA 60

314_27 CATGACCACTAGGAGCATCTTTGGCGAGAAGACTCTGGATTCGGGGACCAGTTGATGCTA 60

317_27 CATGACCACTAGGAGCATCTTTGGCGAGAAGACTCTGGATTCGGGGACCATTTGCTGCTA 60

324_26 CATGACCACTAGGAGCATCTTTGGCGAGAAGACTCTGGATTCGGGAACCAGTTGCTGCTA 60

327_25 CATGACCACTAGGAGCATCTTTGGCGAGAAGACTCTGGATTCGGAGACCAGTTGCTGCTA 60

337_23 CATGACCACTAGGAGCATCTTTGGCGAGAAGAATCTGGATTCGGGGACCAGTTGCTGCTA 60

352_21 CATGACCACTAGGAGCATCTTTGGCGAGAAGACTCTGGATTCGGGGACCAATTGCTGCTA 60

365_19 CATGACCACTAGGAGCATCTTTGGCGAGAAGACTCTGGAATCGGGGACCAGTTGCTGCTA 60

372_18 CATGACCACTAGGAGCATCTTTGGCGAGAAGACTCTTGATTCGGGGACCAGTTGCTGCTA 60

393_16 CATGACCACTAGGAGCATCTTTGGCGAGAAGACTCTGGATTCGGGGAACAGTTGCTGCTA 60

400_16 CATGACCACTAGGAGCATCTTTGGCGAGAAGACTCGGGATTCGGGGACCAGTTGCTGCTA 60

418_15 CATGACCACTAGGAGCATCTTTGGCGAGAAGACTCTGGATTAGGGGACCAGTTGCTGCTA 60

441_14 CATGACCACTAGGAGCATCTTTGGCGAGAAGACTCTGGATACGGGGACCAGTTGCTGCTA 60

445_14 CATGACCACTAGGAGCATCTTTGGCGAGACGACTCTGGATTCGGGGACCAGTTGCTGCTA 60

481_12 CATGACCACTAGGAGCATCTTTGGCGAGAAGACTCTGGATTCGGGGACCTGTTGCTGCTA 60

482_12 CATGACCACTAGGAGCATCTTTGGCGAGAAGACTCTGGATTCGGGCACCAGTTGCTGCTA 60

502_11 CATGACCACTAGGAGCATCTTTGGCGAGATGACTCTGGATTCGGGGACCAGTTGCTGCTA 60

504_11 CATGACCACTAGGAGCATCTTTGGCGAGAAGACTCTGGATTCGCGGACCAGTTGCTGCTA 60

513_10 CATGACCACTAGGAGCATCTTTGGCGAGAAGACTCTGGATTCCGGGACCAGTTGCTGCTA 60

521_10 CATGACCACTAGGAGCATCTTTGGCGAGAAGACTCTGGTTTCGGGGACCAGTTGCTGCTA 60

525_10 CATGACCACTAGGAGCATCTTTGGCGAGAAGTCTCTGGATTCGGGGACCAGTTGCTGCTA 60

536_10 CATGACCACTAGGAGCATCTTTGGCGAGAAGACTCTGGCTTCGGGGACCAGTTGCTGCTA 60

546_9 CATGACCACTAGGAGCATCTTTGGCGAGAAGACTCTGGATTCGGCGACCAGTTGCTGCTA 60

570_8 CATGACCACTAGGAGCATCTTTGGCGAGAAGACTCTGGATTCGGGGCCCAGTTGCTGCTA 60

652_7 CATGACCACTAGGAGCATCTTTGGCGAGAAGACTCTGGATTCGGGGACCAGGTGCTGCTA 60

685_6 CATGACCACTAGGAGCATCTTTGGCGAGAAGACTCTGGATTCGGGGACGAGTTGCTGCTA 60

714_6 CATGACCACTAGGAGCATCTTTGGCGAGAAGACTCTGGATTCGGGGTCCAGTTGCTGCTA 60

744_6 CATGACCACTAGGAGCATCTTTGGCGAGAAGAGTCTGGATTCGGGGACCAGTTGCTGCTA 60

750_6 CATGACCACTAGGAGCATCTTTGGCGAGAACACTCTGGATTCGGGGACCAGTTGCTGCTA 60

814_5 CATGACCACTAGGAGCATCTTTGGCGAGAAGACTCTGGATTCGGGGACCCGTTGCTGCTA 60

816_5 CATGACCACTAGGAGCATCTTTGGCGAGAAGCCTCTGGATTCGGGGACCAGTTGCTGCTA 60

821_5 CATGACCACTAGGAGCATCTTTGGCGAGAAGACTCTGGATTCGGGGACCAGTTGGTGCTA 60

825_5 CATGACCACTAGGAGCATCTTTGGCGAGAAGACTCTGGATTCGGGGACCACTTGCTGCTA 60

929_4 CATGACCACTAGGAGCATCTTTGGCGAGAAGACTCTGGATTCGGGGAGCAGTTGCTGCTA 60

3_57642 CATGACCACTAGGAGCATCTTTGGCGAGAAGACTCTGGATTCGGGGACCAGTTGCTGCTA 60

998_4 CATGACCACTAGGAGCATCTTTGGCGAGAAGACTCTGGATTCGGGGACCAGTTCCTGCTA 60

406_15 CATGACCACTAGGAGCATCTTTGGCGAGAAGACTCTGGATTCGGGGACCAGTTACTGCTA 60

305_29 CATGACCACTAGGAGCATCTTTGGCGAGAAGACTCTGGATTCGGGGACCAGTTTCTGCTA 60

***************************** *****

237_44 GGGGAATAAATCTTTGGGCACCTAGTGGTCATG 93

411_15 GGGGAATAAATCTTTGGGCACCTAGTGGTCATG 93

501_11 GGGGAATAAATCTTTGGGCACCTAGTGGTCATG 93

582_8 GGGGAATAAATCTTTGGGCACCTAGTGGTCATG 93

734_6 GGGGAATAAATCTTTGGGCACCTAGTGGTCATG 93

884_4 GGGGAATAAATCTTTGGGCACCTAGTGGTCATG 93

899_4 GGGGAATAAATCTTTGGGCACCTAGTGGTCATG 93

983_4 GGGGAATAAATCTTTGGGCACCTAGTGGTCATG 93

37_907 GGGGAATAAATCTTTGGGCACCTAGTGGTCATG 93

56_520 GGGGAATAAATCTTTGGGCACCTAGTGGTCATG 93

83_354 GGGGAATAAATCTTTGGGCACCTAGTGGTCATG 93

100_254 GGGGAATAAATCTTTGGGCACCTAGTGGTCATG 93

101_252 GGGGAATAAATCTTTGGGCACCTAGTGGTCATG 93

109_220 GGGGAATAAATCTTTGGGCACCTAGTGGTCATG 93

116_185 GGGGAATAAATCTTTGGGCACCTAGTGGTCATG 93

120_170 GGGGAATAAATCTTTGGGCACCTAGTGGTCATG 93

141_127 GGGGAATAAATCTTTGGGCACCTAGTGGTCATG 93

142_125 GGGGAATAAATCTTTGGGCACCTAGTGGTCATG 93

156_102 GGGGAATAAATCTTTGGGCACCTAGTGGTCATG 93

163_91 GGGGAATAAATCTTTGGGCACCTAGTGGTCATG 93

165_88 GGGGAATAAATCTTTGGGCACCTAGTGGTCATG 93

180_76 GGGGAATAAATCTTTGGGCACCTAGTGGTCATG 93

187_71 GGGGAATAAATCTTTGGGCACCTAGTGGTCATG 93

194_65 GGGGAATAAATCTTTGGGCACCTAGTGGTCATG 93

208_56 GGGGAATAAATCTTTGGGCACCTAGTGGTCATG 93

212_55 GGGGAATAAATCTTTGGGCACCTAGTGGTCATG 93

216_52 GGGGAATAAATCTTTGGGCACCTAGTGGTCATG 93

217_52 GGGGAATAAATCTTTGGGCACCTAGTGGTCATG 93

224_49 GGGGAATAAATCTTTGGGCACCTAGTGGTCATG 93

241_42 GGGGAATAAATCTTTGGGCACCTAGTGGTCATG 93

242_42 GGGGAATAAATCTTTGGGCACCTAGTGGTCATG 93

246_40 GGGGAATAAATCTTTGGGCACCTAGTGGTCATG 93

251_39 GGGGAATAAATCTTTGGGCACCTAGTGGTCATG 93

254_37 GGGGAATAAATCTTTGGGCACCTAGTGGTCATG 93

262_36 GGGGAATAAATCTTTGGGCACCTAGTGGTCATG 93

273_34 GGGGAATAAATCTTTGGGCACCTAGTGGTCATG 93

274_34 GGGGAATAAATCTTTGGGCACCTAGTGGTCATG 93

279_34 GGGGAATAAATCTTTGGGCACCTAGTGGTCATG 93

282_33 GGGGAATAAATCTTTGGGCACCTAGTGGTCATG 93

283_33 GGGGAATAAATCTTTGGGCACCTAGTGGTCATG 93

288_32 GGGGAATAAATCTTTGGGCACCTAGTGGTCATG 93

293_31 GGGGAATAAATCTTTGGGCACCTAGTGGTCATG 93

296_31 GGGGAATAAATCTTTGGGCACCTAGTGGTCATG 93

301_29 GGGGAATAAATCTTTGGGCACCTAGTGGTCATG 93

311_28 GGGGAATAAATCTTTGGGCACCTAGTGGTCATG 93

314_27 GGGGAATAAATCTTTGGGCACCTAGTGGTCATG 93

317_27 GGGGAATAAATCTTTGGGCACCTAGTGGTCATG 93

324_26 GGGGAATAAATCTTTGGGCACCTAGTGGTCATG 93

327_25 GGGGAATAAATCTTTGGGCACCTAGTGGTCATG 93

337_23 GGGGAATAAATCTTTGGGCACCTAGTGGTCATG 93

352_21 GGGGAATAAATCTTTGGGCACCTAGTGGTCATG 93

365_19 GGGGAATAAATCTTTGGGCACCTAGTGGTCATG 93

372_18 GGGGAATAAATCTTTGGGCACCTAGTGGTCATG 93

393_16 GGGGAATAAATCTTTGGGCACCTAGTGGTCATG 93

400_16 GGGGAATAAATCTTTGGGCACCTAGTGGTCATG 93

418_15 GGGGAATAAATCTTTGGGCACCTAGTGGTCATG 93

441_14 GGGGAATAAATCTTTGGGCACCTAGTGGTCATG 93

445_14 GGGGAATAAATCTTTGGGCACCTAGTGGTCATG 93

481_12 GGGGAATAAATCTTTGGGCACCTAGTGGTCATG 93

482_12 GGGGAATAAATCTTTGGGCACCTAGTGGTCATG 93

502_11 GGGGAATAAATCTTTGGGCACCTAGTGGTCATG 93

504_11 GGGGAATAAATCTTTGGGCACCTAGTGGTCATG 93

513_10 GGGGAATAAATCTTTGGGCACCTAGTGGTCATG 93

521_10 GGGGAATAAATCTTTGGGCACCTAGTGGTCATG 93

525_10 GGGGAATAAATCTTTGGGCACCTAGTGGTCATG 93

536_10 GGGGAATAAATCTTTGGGCACCTAGTGGTCATG 93

546_9 GGGGAATAAATCTTTGGGCACCTAGTGGTCATG 93

570_8 GGGGAATAAATCTTTGGGCACCTAGTGGTCATG 93

652_7 GGGGAATAAATCTTTGGGCACCTAGTGGTCATG 93

685_6 GGGGAATAAATCTTTGGGCACCTAGTGGTCATG 93

714_6 GGGGAATAAATCTTTGGGCACCTAGTGGTCATG 93

744_6 GGGGAATAAATCTTTGGGCACCTAGTGGTCATG 93

750_6 GGGGAATAAATCTTTGGGCACCTAGTGGTCATG 93

814_5 GGGGAATAAATCTTTGGGCACCTAGTGGTCATG 93

816_5 GGGGAATAAATCTTTGGGCACCTAGTGGTCATG 93

821_5 GGGGAATAAATCTTTGGGCACCTAGTGGTCATG 93

825_5 GGGGAATAAATCTTTGGGCACCTAGTGGTCATG 93

929_4 GGGGAATAAATCTTTGGGCACCTAGTGGTCATG 93

3_57642 GGGGAATAAATCTTTGGGCACCTAGTGGTCATG 93

998_4 GGGGAATAAATCTTTGGGCACCTAGTGGTCATG 93

406_15 GGGGAATAAATCTTTGGGCACCTAGTGGTCATG 93

305_29 GGGGAATAAATCTTTGGGCACCTAGTGGTCATG 93

*********************************

**3. CLUSTAL multiple sequence alignment for II-R1-7 (7_3906)**

CLUSTAL multiple sequence alignment

297_30 CATGACCACTAGGAGCATCTTTGGCGAGATCGGGAGAATCGGCGGCATTGGTGTCTTCTA 60

341_23 CATGACCACTAGGAGCATCTTTGGCGAGATTGGGAGAATCGGTGGCATTGGTGTCTTCTA 60

404_15 CATGACCACTAGGAGCATCTTTGGCGAGATCGGGAGAATCGGTGGCATTGGTGTTTTCTA 60

448_14 CATGACCACTAGGAGCATCTTTGGCGAGATCGTGAGAATCGGTGGCATTGGTGTCTTCTA 60

503_11 CATGACCACTAGGAGCATCTTTGGCGAGATCGGGAGGATCGGTGGCATTGGTGTCTTCTA 60

510_11 CATGACCACTAGGAGCATCTTTGGCGAGATCGAGAGAATCGGTGGCATTGGTGTCTTCTA 60

511_11 CATGACCACTAGGAGCATCTTTGGCGAGATCGGTAGAATCGGTGGCATTGGTGTCTTCTA 60

544_9 CATGACCACTAGGAGCATCTTTGGCGAGATCGGGAGAATTGGTGGCATTGGTGTCTTCTA 60

601_8 CATGACCACTAGGAGCATCTTTGGCGAGATCGGGAGAATCGGTGGCATCGGTGTCTTCTA 60

648_7 CATGACCACTAGGAGCATCTTTGGCGAGATCAGGAGAATCGGTGGCATTGGTGTCTTCTA 60

661_7 CATGACCACTAGGAGCATCTTTGGCGAGATCGCGAGAATCGGTGGCATTGGTGTCTTCTA 60

673_6 CATGACCACTAGGAGCATCTTTGGCGAGATCGGGAGAATCGGTGGCATAGGTGTCTTCTA 60

703_6 CATGACCACTAGGAGCATCTTTGGCGAGATCGGGGGAATCGGTGGCATTGGTGTCTTCTA 60

776_5 CATGACCACTAGGAGCATCTTTGGCGAGATCGGGAGAGTCGGTGGCATTGGTGTCTTCTA 60

778_5 CATGACCACTAGGAGCATCTTTGGCGAGATCGGAAGAATCGGTGGCATTGGTGTCTTCTA 60

845_5 CATGACCACTAGGAGCATCTTTGGCGAGATCGGGAGAATCGGTGGTATTGGTGTCTTCTA 60

872_5 CATGACCACTAGGAGCATCTTTGGCGAGATCGGCAGAATCGGTGGCATTGGTGTCTTCTA 60

7_3906 CATGACCACTAGGAGCATCTTTGGCGAGATCGGGAGAATCGGTGGCATTGGTGTCTTCTA 60

905_4 CATGACCACTAGGAGCATCTTTGGCGAGATCGGGAGAATCGGTGGCATTGGTGCCTTCTA 60

****************************** * * ** ** ** **** *****

297_30 GGGGAATAAATCTTTGGGCACCTAGTGGTCATG 93

341_23 GGGGAATAAATCTTTGGGCACCTAGTGGTCATG 93

404_15 GGGGAATAAATCTTTGGGCACCTAGTGGTCATG 93

448_14 GGGGAATAAATCTTTGGGCACCTAGTGGTCATG 93

503_11 GGGGAATAAATCTTTGGGCACCTAGTGGTCATG 93

510_11 GGGGAATAAATCTTTGGGCACCTAGTGGTCATG 93

511_11 GGGGAATAAATCTTTGGGCACCTAGTGGTCATG 93

544_9 GGGGAATAAATCTTTGGGCACCTAGTGGTCATG 93

601_8 GGGGAATAAATCTTTGGGCACCTAGTGGTCATG 93

648_7 GGGGAATAAATCTTTGGGCACCTAGTGGTCATG 93

661_7 GGGGAATAAATCTTTGGGCACCTAGTGGTCATG 93

673_6 GGGGAATAAATCTTTGGGCACCTAGTGGTCATG 93

703_6 GGGGAATAAATCTTTGGGCACCTAGTGGTCATG 93

776_5 GGGGAATAAATCTTTGGGCACCTAGTGGTCATG 93

778_5 GGGGAATAAATCTTTGGGCACCTAGTGGTCATG 93

845_5 GGGGAATAAATCTTTGGGCACCTAGTGGTCATG 93

872_5 GGGGAATAAATCTTTGGGCACCTAGTGGTCATG 93

7_3906 GGGGAATAAATCTTTGGGCACCTAGTGGTCATG 93

905_4 GGGGAATAAATCTTTGGGCACCTAGTGGTCATG 93

*********************************
