# Supplementary material for: Selection of allosteric dnazymes that can sense phenylalanine by expression-SELEX
Source: Nucleic Acids Res. 2023 May 19;51(11):e66. doi: 10.1093/nar/gkad424 (PMC10287898; doi:10.1093/nar/gkad424)
Supplement: gkad424_Supplemental_Files [file gkad424_supplemental_files.zip › Supplementary file No. 9 three-top-enriched-sequences-stockholm_files.docx]

1. **II-R1-1 (1_892954) Stockholm file**

# STOCKHOLM 1.0

#=GF SS RNAalifold prediction

5_7863 CATGACCACTAGGAGCATCTTTGGCGAGATCGGGAGAATCGGCGGCATTGGTGTCTCCTAGGGGAATAAATCTTTGGGCACCTAGTGGTCATG

6_4354 CATGACCACTAGGAGCATCTTTGGCGAGATTGGGAGAATCGGTGGCATTGGTGTCTCCTAGGGGAATAAATCTTTGGGCACCTAGTGGTCATG

11_3283 CATGACCACTAGGAGCATCTTTGGCGAGATCGGGAGAATTGGTGGCATTGGTGTCTCCTAGGGGAATAAATCTTTGGGCACCTAGTGGTCATG

12_2781 CATGACCACTAGGAGCATCTTTGGCGAGATCGTGAGAATCGGTGGCATTGGTGTCTCCTAGGGGAATAAATCTTTGGGCACCTAGTGGTCATG

14_2397 CATGACCACTAGGAGCATCTTTGGCGAGATCGGGAGAATCGGTGGCATTGGTGTTTCCTAGGGGAATAAATCTTTGGGCACCTAGTGGTCATG

15_2111 CATGACCACTAGGAGCATCTTTGGCGAGATCGGGAGAATCGGTGGCATCGGTGTCTCCTAGGGGAATAAATCTTTGGGCACCTAGTGGTCATG

17_1565 CATGACCACTAGGAGCATCTTTGGCGAGATCGGGGGAATCGGTGGCATTGGTGTCTCCTAGGGGAATAAATCTTTGGGCACCTAGTGGTCATG

18_1555 CATGACCACTAGGAGCATCTTTGGCGAGATCAGGAGAATCGGTGGCATTGGTGTCTCCTAGGGGAATAAATCTTTGGGCACCTAGTGGTCATG

20_1482 CATGACCACTAGGAGCATCTTTGGCGAGATCGGGAGGATCGGTGGCATTGGTGTCTCCTAGGGGAATAAATCTTTGGGCACCTAGTGGTCATG

22_1449 CATGACCACTAGGAGCATCTTTGGCGAGATCGAGAGAATCGGTGGCATTGGTGTCTCCTAGGGGAATAAATCTTTGGGCACCTAGTGGTCATG

24_1332 CATGACCACTAGGAGCATCTTTGGCGAGATCGGGAGAATCGGTGGCATTGGTGCCTCCTAGGGGAATAAATCTTTGGGCACCTAGTGGTCATG

26_1257 CATGACCACTAGGAGCATCTTTGGCGAGATCGGGAGAATCGGTGGCATTGGCGTCTCCTAGGGGAATAAATCTTTGGGCACCTAGTGGTCATG

30_1081 CATGACCACTAGGAGCATCTTTGGCGAGATCGGGAGAATCGGTGGTATTGGTGTCTCCTAGGGGAATAAATCTTTGGGCACCTAGTGGTCATG

32_1013 CATGACCACTAGGAGCATCTTTGGCGAGATCGGAAGAATCGGTGGCATTGGTGTCTCCTAGGGGAATAAATCTTTGGGCACCTAGTGGTCATG

33_1011 CATGACCACTAGGAGCATCTTTGGCGAGATCGGGAGAATCGGTGGCGTTGGTGTCTCCTAGGGGAATAAATCTTTGGGCACCTAGTGGTCATG

34_963 CATGACCACTAGGAGCATCTTTGGCGAGATCGCGAGAATCGGTGGCATTGGTGTCTCCTAGGGGAATAAATCTTTGGGCACCTAGTGGTCATG

35_938 CATGACCACTAGGAGCATCTTTGGCGAGATCGGTAGAATCGGTGGCATTGGTGTCTCCTAGGGGAATAAATCTTTGGGCACCTAGTGGTCATG

40_706 CATGACCACTAGGAGCATCTTTGGCGAGATCTGGAGAATCGGTGGCATTGGTGTCTCCTAGGGGAATAAATCTTTGGGCACCTAGTGGTCATG

45_629 CATGACCACTAGGAGCATCTTTGGCGAGATCGGGAGAATCGGTGGCACTGGTGTCTCCTAGGGGAATAAATCTTTGGGCACCTAGTGGTCATG

46_618 CATGACCACTAGGAGCATCTTTGGCGAGATCGGCAGAATCGGTGGCATTGGTGTCTCCTAGGGGAATAAATCTTTGGGCACCTAGTGGTCATG

49_559 CATGACCACTAGGAGCATCTTTGGCGAGATCGGGAGAATCGTTGGCATTGGTGTCTCCTAGGGGAATAAATCTTTGGGCACCTAGTGGTCATG

50_552 CATGACCACTAGGAGCATCTTTGGCGAGATCGGGAGAATCGGAGGCATTGGTGTCTCCTAGGGGAATAAATCTTTGGGCACCTAGTGGTCATG

52_534 CATGACCACTAGGAGCATCTTTGGCGAGATCGGGAGAATCGGTGGCATAGGTGTCTCCTAGGGGAATAAATCTTTGGGCACCTAGTGGTCATG

53_527 CATGACCACTAGGAGCATCTTTGGCGAGATCGGGAGAATCTGTGGCATTGGTGTCTCCTAGGGGAATAAATCTTTGGGCACCTAGTGGTCATG

55_526 CATGACCACTAGGAGCATCTTTGGCGAGATCGGGAGAATCAGTGGCATTGGTGTCTCCTAGGGGAATAAATCTTTGGGCACCTAGTGGTCATG

57_518 CATGACCACTAGGAGCATCTTTGGCGAGACCGGGAGAATCGGTGGCATTGGTGTCTCCTAGGGGAATAAATCTTTGGGCACCTAGTGGTCATG

59_481 CATGACCACTAGGAGCATCTTTGGCGAGATCGGGAGAATCGGTGTCATTGGTGTCTCCTAGGGGAATAAATCTTTGGGCACCTAGTGGTCATG

61_454 CATGACCACTAGGAGCATCTTTGGCGAGATCGGGAGAATCGGTGGCATTAGTGTCTCCTAGGGGAATAAATCTTTGGGCACCTAGTGGTCATG

62_452 CATGACCACTAGGAGCATCTTTGGCGAGATCGGGAGAATCGGTGGCATTGATGTCTCCTAGGGGAATAAATCTTTGGGCACCTAGTGGTCATG

63_447 CATGACCACTAGGAGCATCTTTGGCGAGATCGGGAGAATCGGTAGCATTGGTGTCTCCTAGGGGAATAAATCTTTGGGCACCTAGTGGTCATG

67_430 CATGACCACTAGGAGCATCTTTGGCGAGATCGGGAGAAACGGTGGCATTGGTGTCTCCTAGGGGAATAAATCTTTGGGCACCTAGTGGTCATG

69_427 CATGACCACTAGGAGCATCTTTGGCGAGATCGGGAGAATCGGTGGCATTGTTGTCTCCTAGGGGAATAAATCTTTGGGCACCTAGTGGTCATG

75_386 CATGACCACTAGGAGCATCTTTGGCGAGATCGGGATAATCGGTGGCATTGGTGTCTCCTAGGGGAATAAATCTTTGGGCACCTAGTGGTCATG

77_378 CATGACCACTAGGAGCATCTTTGGCGAGATCGGGAGAATCGGTGACATTGGTGTCTCCTAGGGGAATAAATCTTTGGGCACCTAGTGGTCATG

78_370 CATGACCACTAGGAGCATCTTTGGCGAGATCGGGAGAATCGGTGGCATTTGTGTCTCCTAGGGGAATAAATCTTTGGGCACCTAGTGGTCATG

79_369 CATGACCACTAGGAGCATCTTTGGCGAGATAGGGAGAATCGGTGGCATTGGTGTCTCCTAGGGGAATAAATCTTTGGGCACCTAGTGGTCATG

81_364 CATGACCACTAGGAGCATCTTTGGCGAGATCGGGAGAATCGGTGGCAGTGGTGTCTCCTAGGGGAATAAATCTTTGGGCACCTAGTGGTCATG

84_354 CATGACCACTAGGAGCATCTTTGGCGAGATCGGGAGAACCGGTGGCATTGGTGTCTCCTAGGGGAATAAATCTTTGGGCACCTAGTGGTCATG

87_343 CATGACCACTAGGAGCATCTTTGGCGAGATCGGGAGAATCGGTGGCATTGGAGTCTCCTAGGGGAATAAATCTTTGGGCACCTAGTGGTCATG

89_330 CATGACCACTAGGAGCATCTTTGGCGAGATCGGGAGAATCGGTGGCATTGGTGACTCCTAGGGGAATAAATCTTTGGGCACCTAGTGGTCATG

90_327 CATGACCACTAGGAGCATCTTTGGCGAGATCGGGAGAATCGGTGGAATTGGTGTCTCCTAGGGGAATAAATCTTTGGGCACCTAGTGGTCATG

92_310 CATGACCACTAGGAGCATCTTTGGCGAGATCGGGAGAATCGGTGGCATTGGTTTCTCCTAGGGGAATAAATCTTTGGGCACCTAGTGGTCATG

93_307 CATGACCACTAGGAGCATCTTTGGCGAGATCGGGAGAATAGGTGGCATTGGTGTCTCCTAGGGGAATAAATCTTTGGGCACCTAGTGGTCATG

94_296 CATGACCACTAGGAGCATCTTTGGCGAGATCGGGAGAATCGATGGCATTGGTGTCTCCTAGGGGAATAAATCTTTGGGCACCTAGTGGTCATG

95_285 CATGACCACTAGGAGCATCTTTGGCGAGATCGGGAGAATCGGTGGCATTGGTATCTCCTAGGGGAATAAATCTTTGGGCACCTAGTGGTCATG

96_283 CATGACCACTAGGAGCATCTTTGGCGAGATCGGGAGAATCGGTTGCATTGGTGTCTCCTAGGGGAATAAATCTTTGGGCACCTAGTGGTCATG

97_272 CATGACCACTAGGAGCATCTTTGGCGAGATCGGGAGAATCGCTGGCATTGGTGTCTCCTAGGGGAATAAATCTTTGGGCACCTAGTGGTCATG

99_259 CATGACCACTAGGAGCATCTTTGGCGAGATCGGGAGAATCGGTGGCAATGGTGTCTCCTAGGGGAATAAATCTTTGGGCACCTAGTGGTCATG

105_226 CATGACCACTAGGAGCATCTTTGGCGAGATCGGGAAAATCGGTGGCATTGGTGTCTCCTAGGGGAATAAATCTTTGGGCACCTAGTGGTCATG

108_222 CATGACCACTAGGAGCATCTTTGGCGAGATCGGGAGTATCGGTGGCATTGGTGTCTCCTAGGGGAATAAATCTTTGGGCACCTAGTGGTCATG

111_213 CATGACCACTAGGAGCATCTTTGGCGAGATCGGGAGAATCGGTGCCATTGGTGTCTCCTAGGGGAATAAATCTTTGGGCACCTAGTGGTCATG

113_208 CATGACCACTAGGAGCATCTTTGGCGAGATCGGGAGAATCGGTGGCCTTGGTGTCTCCTAGGGGAATAAATCTTTGGGCACCTAGTGGTCATG

117_179 CATGACCACTAGGAGCATCTTTGGCGAGATCCGGAGAATCGGTGGCATTGGTGTCTCCTAGGGGAATAAATCTTTGGGCACCTAGTGGTCATG

119_177 CATGACCACTAGGAGCATCTTTGGCGAGAACGGGAGAATCGGTGGCATTGGTGTCTCCTAGGGGAATAAATCTTTGGGCACCTAGTGGTCATG

123_169 CATGACCACTAGGAGCATCTTTGGCGAGATCGGGAGAATCGGTGGCATTGCTGTCTCCTAGGGGAATAAATCTTTGGGCACCTAGTGGTCATG

124_168 CATGACCACTAGGAGCATCTTTGGCGAGATCGGGAGAATCGGTGGCATTGGTGTATCCTAGGGGAATAAATCTTTGGGCACCTAGTGGTCATG

132_147 CATGACCACTAGGAGCATCTTTGGCGAGATCGGGTGAATCGGTGGCATTGGTGTCTCCTAGGGGAATAAATCTTTGGGCACCTAGTGGTCATG

138_133 CATGACCACTAGGAGCATCTTTGGCGAGATCGGGCGAATCGGTGGCATTGGTGTCTCCTAGGGGAATAAATCTTTGGGCACCTAGTGGTCATG

139_132 CATGACCACTAGGAGCATCTTTGGCGAGATCGGGAGAATCGGTGGGATTGGTGTCTCCTAGGGGAATAAATCTTTGGGCACCTAGTGGTCATG

143_120 CATGACCACTAGGAGCATCTTTGGCGAGAGCGGGAGAATCGGTGGCATTGGTGTCTCCTAGGGGAATAAATCTTTGGGCACCTAGTGGTCATG

147_114 CATGACCACTAGGAGCATCTTTGGCGAGATCGGGAGAATCGGTGGCATTCGTGTCTCCTAGGGGAATAAATCTTTGGGCACCTAGTGGTCATG

148_111 CATGACCACTAGGAGCATCTTTGGCGAGATCGGGACAATCGGTGGCATTGGTGTCTCCTAGGGGAATAAATCTTTGGGCACCTAGTGGTCATG

153_105 CATGACCACTAGGAGCATCTTTGGCGAGATCGGGAGAATCGGTGGCATGGGTGTCTCCTAGGGGAATAAATCTTTGGGCACCTAGTGGTCATG

155_102 CATGACCACTAGGAGCATCTTTGGCGAGATCGGGAGAATCGGTGGCATTGGTCTCTCCTAGGGGAATAAATCTTTGGGCACCTAGTGGTCATG

160_98 CATGACCACTAGGAGCATCTTTGGCGAGATCGGGAGAATCGGTGGCATTGGGGTCTCCTAGGGGAATAAATCTTTGGGCACCTAGTGGTCATG

171_83 CATGACCACTAGGAGCATCTTTGGCGAGATCGGGAGAATCGGTGGCATTGGTGGCTCCTAGGGGAATAAATCTTTGGGCACCTAGTGGTCATG

172_83 CATGACCACTAGGAGCATCTTTGGCGAGATCGGGAGAATCGGGGGCATTGGTGTCTCCTAGGGGAATAAATCTTTGGGCACCTAGTGGTCATG

178_77 CATGACCACTAGGAGCATCTTTGGCGAGATGGGGAGAATCGGTGGCATTGGTGTCTCCTAGGGGAATAAATCTTTGGGCACCTAGTGGTCATG

179_77 CATGACCACTAGGAGCATCTTTGGCGAGATCGGGAGAATCGGTGGCTTTGGTGTCTCCTAGGGGAATAAATCTTTGGGCACCTAGTGGTCATG

182_75 CATGACCACTAGGAGCATCTTTGGCGAGATCGGGAGAATCGGTCGCATTGGTGTCTCCTAGGGGAATAAATCTTTGGGCACCTAGTGGTCATG

188_70 CATGACCACTAGGAGCATCTTTGGCGAGATCGGGAGAATCCGTGGCATTGGTGTCTCCTAGGGGAATAAATCTTTGGGCACCTAGTGGTCATG

228_47 CATGACCACTAGGAGCATCTTTGGCGAGATCGGGAGCATCGGTGGCATTGGTGTCTCCTAGGGGAATAAATCTTTGGGCACCTAGTGGTCATG

244_41 CATGACCACTAGGAGCATCTTTGGCGAGATCGGGAGAATGGGTGGCATTGGTGTCTCCTAGGGGAATAAATCTTTGGGCACCTAGTGGTCATG

245_40 CATGACCACTAGGAGCATCTTTGGCGAGATCGGGAGAATCGGTGGCATTGGTGTGTCCTAGGGGAATAAATCTTTGGGCACCTAGTGGTCATG

312_28 CATGACCACTAGGAGCATCTTTGGCGAGATCGGGAGAAGCGGTGGCATTGGTGTCTCCTAGGGGAATAAATCTTTGGGCACCTAGTGGTCATG

1_892954 CATGACCACTAGGAGCATCTTTGGCGAGATCGGGAGAATCGGTGGCATTGGTGTCTCCTAGGGGAATAAATCTTTGGGCACCTAGTGGTCATG

415_15 CATGACCACTAGGAGCATCTTTGGCGAGATCGGGAGATTCGGTGGCATTGGTGTCTCCTAGGGGAATAAATCTTTGGGCACCTAGTGGTCATG

73_396 CATGACCACTAGGAGCATCTTTGGCGAGATCGGGAGACTCGGTGGCATTGGTGTCTCCTAGGGGAATAAATCTTTGGGCACCTAGTGGTCATG

29_1104 CATGACCACTAGGAGCATCTTTGGCGAGATCGGGAGAGTCGGTGGCATTGGTGTCTCCTAGGGGAATAAATCTTTGGGCACCTAGTGGTCATG

773_5 CATGACCACTAGGAGCATCTTTGGCGAGATCGGGGGACTCGGTGGCATTGGTGTCTCCTAGGGGAATAAATCTTTGGGCACCTAGTGGTCATG

822_5 CATGACCACTAGGAGCATCTTTGGCGAGATCTGGGGAATCGGTGGCATTGGTGTCTCCTAGGGGAATAAATCTTTGGGCACCTAGTGGTCATG

779_5 CATGACCACTAGGAGCATCTTTGGCGAGATCGGGGGAATTGGTGGCATTGGTGTCTCCTAGGGGAATAAATCTTTGGGCACCTAGTGGTCATG

350_21 CATGACCACTAGGAGCATCTTTGGCGAGATCGGGGGAATCGGCGGCATTGGTGTCTCCTAGGGGAATAAATCTTTGGGCACCTAGTGGTCATG

610_7 CATGACCACTAGGAGCATCTTTGGCGAGATCGGAGGAATCGGTGGCATTGGTGTCTCCTAGGGGAATAAATCTTTGGGCACCTAGTGGTCATG

986_4 CATGACCACTAGGAGCATCTTTGGCGAGATCGGGAGAATCGGTGGCACCGGTGTCTCCTAGGGGAATAAATCTTTGGGCACCTAGTGGTCATG

852_5 CATGACCACTAGGAGCATCTTTGGCGAGATCGGGAGAATCGGTGGCATCGGTGTTTCCTAGGGGAATAAATCTTTGGGCACCTAGTGGTCATG

848_5 CATGACCACTAGGAGCATCTTTGGCGAGATCGGAAGAATCGGTGGCATCGGTGTCTCCTAGGGGAATAAATCTTTGGGCACCTAGTGGTCATG

429_15 CATGACCACTAGGAGCATCTTTGGCGAGATCGGGAGAATCGGTAACATTGGTGTCTCCTAGGGGAATAAATCTTTGGGCACCTAGTGGTCATG

952_4 CATGACCACTAGGAGCATCTTTGGCGAGATCAGGAGAATCGGTAGCATTGGTGTCTCCTAGGGGAATAAATCTTTGGGCACCTAGTGGTCATG

754_6 CATGACCACTAGGAGCATCTTTGGCGAGATCAGGAGAATCGGTGGCATCGGTGTCTCCTAGGGGAATAAATCTTTGGGCACCTAGTGGTCATG

717_6 CATGACCACTAGGAGCATCTTTGGCGAGATCGGGAGAATCGTAGGCATTGGTGTCTCCTAGGGGAATAAATCTTTGGGCACCTAGTGGTCATG

594_8 CATGACCACTAGGAGCATCTTTGGCGAGATCGGGAGAATCGTCGGCATTGGTGTCTCCTAGGGGAATAAATCTTTGGGCACCTAGTGGTCATG

718_6 CATGACCACTAGGAGCATCTTTGGCGAGATCGGGAGAAACGGAGGCATTGGTGTCTCCTAGGGGAATAAATCTTTGGGCACCTAGTGGTCATG

966_4 CATGACCACTAGGAGCATCTTTGGCGAGATCGGGAGAATCGGTGGCATTGGAGACTCCTAGGGGAATAAATCTTTGGGCACCTAGTGGTCATG

950_4 CATGACCACTAGGAGCATCTTTGGCGAGATCGGGAGAATCGGTGGCATTGCAGTCTCCTAGGGGAATAAATCTTTGGGCACCTAGTGGTCATG

926_4 CATGACCACTAGGAGCATCTTTGGCGAGATCGGGAGAATCGTTGGCATTGGAGTCTCCTAGGGGAATAAATCTTTGGGCACCTAGTGGTCATG

856_5 CATGACCACTAGGAGCATCTTTGGCGAGATCGGGAGAAACGGTGGCATTGGAGTCTCCTAGGGGAATAAATCTTTGGGCACCTAGTGGTCATG

808_5 CATGACCACTAGGAGCATCTTTGGCGAGATCGGGAGAATCGGTGGCAATGGAGTCTCCTAGGGGAATAAATCTTTGGGCACCTAGTGGTCATG

705_6 CATGACCACTAGGAGCATCTTTGGCGAGATCGGTAGAATCGGTGGCATTGGAGTCTCCTAGGGGAATAAATCTTTGGGCACCTAGTGGTCATG

494_11 CATGACCACTAGGAGCATCTTTGGCGAGATCGGTAGAATCGGCGGCATTGGTGTCTCCTAGGGGAATAAATCTTTGGGCACCTAGTGGTCATG

558_9 CATGACCACTAGGAGCATCTTTGGCGAGATAGGGAGAATCGGTGGCATTTGTGTCTCCTAGGGGAATAAATCTTTGGGCACCTAGTGGTCATG

823_5 CATGACCACTAGGAGCATCTTTGGCGAGATTGGAAGAATCGGTGGCATTGGTGTCTCCTAGGGGAATAAATCTTTGGGCACCTAGTGGTCATG

956_4 CATGACCACTAGGAGCATCTTTGGCGAGATAGGTAGAATCGGTGGCATTGGTGTCTCCTAGGGGAATAAATCTTTGGGCACCTAGTGGTCATG

795_5 CATGACCACTAGGAGCATCTTTGGCGAGATTGGTAGAATCGGTGGCATTGGTGTCTCCTAGGGGAATAAATCTTTGGGCACCTAGTGGTCATG

367_19 CATGACCACTAGGAGCATCTTTGGCGAGATCGGGAGAGTCGGCGGCATTGGTGTCTCCTAGGGGAATAAATCTTTGGGCACCTAGTGGTCATG

413_15 CATGACCACTAGGAGCATCTTTGGCGAGATTGGGAGAATTGGTGGCATTGGTGTCTCCTAGGGGAATAAATCTTTGGGCACCTAGTGGTCATG

572_8 CATGACCACTAGGAGCATCTTTGGCGAGATTGGGAGAATCGGTGGTATTGGTGTCTCCTAGGGGAATAAATCTTTGGGCACCTAGTGGTCATG

625_7 CATGACCACTAGGAGCATCTTTGGCGAGATCGGGAGAAACGGTGGCATAGGTGTCTCCTAGGGGAATAAATCTTTGGGCACCTAGTGGTCATG

640_7 CATGACCACTAGGAGCATCTTTGGCGAGATTGGGAGAATCGGTGGCATTGGTGCCTCCTAGGGGAATAAATCTTTGGGCACCTAGTGGTCATG

745_6 CATGACCACTAGGAGCATCTTTGGCGAGATCGGGAGAATCGGTGGCATTAATGTCTCCTAGGGGAATAAATCTTTGGGCACCTAGTGGTCATG

751_6 CATGACCACTAGGAGCATCTTTGGCGAGATCGGGAGAATCGGTGGCATAGGAGTCTCCTAGGGGAATAAATCTTTGGGCACCTAGTGGTCATG

789_5 CATGACCACTAGGAGCATCTTTGGCGAGATTGGGAGAATCGGTGGCATTAGTGTCTCCTAGGGGAATAAATCTTTGGGCACCTAGTGGTCATG

892_4 CATGACCACTAGGAGCATCTTTGGCGAGATCGGGAGAATCGGTGGCATAGGTGTATCCTAGGGGAATAAATCTTTGGGCACCTAGTGGTCATG

993_4 CATGACCACTAGGAGCATCTTTGGCGAGATTGGGAGAATCGGTGGCATAGGTGTCTCCTAGGGGAATAAATCTTTGGGCACCTAGTGGTCATG

545_9 CATGACCACTAGGAGCATCTTTGGCGAGATTGGGAGAATCGGTGGCATCGGTGTCTCCTAGGGGAATAAATCTTTGGGCACCTAGTGGTCATG

975_4 CATGACCACTAGGAGCATCTTTGGCGAGATTGGGAGAGTCGGTGGCATTGGTGTCTCCTAGGGGAATAAATCTTTGGGCACCTAGTGGTCATG

850_5 CATGACCACTAGGAGCATCTTTGGCGAGATTGGGAGAATCGGTGGCATTGGCGTCTCCTAGGGGAATAAATCTTTGGGCACCTAGTGGTCATG

820_5 CATGACCACTAGGAGCATCTTTGGCGAGATTGGGAGGATCGGTGGCATTGGTGTCTCCTAGGGGAATAAATCTTTGGGCACCTAGTGGTCATG

764_5 CATGACCACTAGGAGCATCTTTGGCGAGATTGGGAGAATCGGTGACATTGGTGTCTCCTAGGGGAATAAATCTTTGGGCACCTAGTGGTCATG

747_6 CATGACCACTAGGAGCATCTTTGGCGAGATTGGGAGAATCGGTGGCATTGGTGTTTCCTAGGGGAATAAATCTTTGGGCACCTAGTGGTCATG

698_6 CATGACCACTAGGAGCATCTTTGGCGAGATTGGGAGAATCGGTGGCACTGGTGTCTCCTAGGGGAATAAATCTTTGGGCACCTAGTGGTCATG

826_5 CATGACCACTAGGAGCATCTTTGGCGAGATCGGGAGAATCGGCTGCATTGGTGTCTCCTAGGGGAATAAATCTTTGGGCACCTAGTGGTCATG

720_6 CATGACCACTAGGAGCATCTTTGGCGAGATCGGGAGAATCGGATGCATTGGTGTCTCCTAGGGGAATAAATCTTTGGGCACCTAGTGGTCATG

357_21 CATGACCACTAGGAGCATCTTTGGCGAGATCGGGAGAATCGGCGGCATCGGTGTCTCCTAGGGGAATAAATCTTTGGGCACCTAGTGGTCATG

770_5 CATGACCACTAGGAGCATCTTTGGCGAGATCGGGAGAATCGGCGGCATAGGTGTCTCCTAGGGGAATAAATCTTTGGGCACCTAGTGGTCATG

740_6 CATGACCACTAGGAGCATCTTTGGCGAGATCGGGAGAATCGGAGGCATAGGTGTCTCCTAGGGGAATAAATCTTTGGGCACCTAGTGGTCATG

803_5 CATGACCACTAGGAGCATCTTTGGCGAGATCGCGAGAATCGGCGGCATTGGTGTCTCCTAGGGGAATAAATCTTTGGGCACCTAGTGGTCATG

936_4 CATGACCACTAGGAGCATCTTTGGCGAGATCGGGAGAATCGGCGGAATTGGTGTCTCCTAGGGGAATAAATCTTTGGGCACCTAGTGGTCATG

949_4 CATGACCACTAGGAGCATCTTTGGCGAGATCGGGAGAATCGGGGGTATTGGTGTCTCCTAGGGGAATAAATCTTTGGGCACCTAGTGGTCATG

369_19 CATGACCACTAGGAGCATCTTTGGCGAGATCGGGAGAATCGGCGGTATTGGTGTCTCCTAGGGGAATAAATCTTTGGGCACCTAGTGGTCATG

309_28 CATGACCACTAGGAGCATCTTTGGCGAGATCGGGAGAATTGGCGGCATTGGTGTCTCCTAGGGGAATAAATCTTTGGGCACCTAGTGGTCATG

323_26 CATGACCACTAGGAGCATCTTTGGCGAGATCGGGAGAATCGGCGGCATTGGTGTTTCCTAGGGGAATAAATCTTTGGGCACCTAGTGGTCATG

389_17 CATGACCACTAGGAGCATCTTTGGCGAGATCGGGAGAATCGGCGGCATTGGTGCCTCCTAGGGGAATAAATCTTTGGGCACCTAGTGGTCATG

506_11 CATGACCACTAGGAGCATCTTTGGCGAGATCGGGAGGATCGGCGGCATTGGTGTCTCCTAGGGGAATAAATCTTTGGGCACCTAGTGGTCATG

806_5 CATGACCACTAGGAGCATCTTTGGCGAGATCGGGAGAATCGGCGACATTGGTGTCTCCTAGGGGAATAAATCTTTGGGCACCTAGTGGTCATG

637_7 CATGACCACTAGGAGCATCTTTGGCGAGATCGGGAGAATCGGCGTCATTGGTGTCTCCTAGGGGAATAAATCTTTGGGCACCTAGTGGTCATG

813_5 CATGACCACTAGGAGCATCTTTGGCGAGATCGGGAGAATCAGCGGCATTGGTGTCTCCTAGGGGAATAAATCTTTGGGCACCTAGTGGTCATG

829_5 CATGACCACTAGGAGCATCTTTGGCGAGATCGGGAGAATCGGCGGCATTTGTGTCTCCTAGGGGAATAAATCTTTGGGCACCTAGTGGTCATG

835_5 CATGACCACTAGGAGCATCTTTGGCGAGATCGGGAGAATCGGCGGCAGTGGTGTCTCCTAGGGGAATAAATCTTTGGGCACCTAGTGGTCATG

836_5 CATGACCACTAGGAGCATCTTTGGCGAGATCGGGAGAATCGGCGGCGTTGGTGTCTCCTAGGGGAATAAATCTTTGGGCACCTAGTGGTCATG

893_4 CATGACCACTAGGAGCATCTTTGGCGAGATCGGGAGAATCGGCGGCATTGGGGTCTCCTAGGGGAATAAATCTTTGGGCACCTAGTGGTCATG

663_7 CATGACCACTAGGAGCATCTTTGGCGAGATCGGGAGAATCGGCGGCATTGGCGTCTCCTAGGGGAATAAATCTTTGGGCACCTAGTGGTCATG

921_4 CATGACCACTAGGAGCATCTTTGGCGAGATCCGGAGAATCGGCGGCATTGGTGTCTCCTAGGGGAATAAATCTTTGGGCACCTAGTGGTCATG

461_13 CATGACCACTAGGAGCATCTTTGGCGAGATCTGGAGAATCGGCGGCATTGGTGTCTCCTAGGGGAATAAATCTTTGGGCACCTAGTGGTCATG

272_34 CATGACCACTAGGAGCATCTTTGGCGAGATCAGGAGAATCGGCGGCATTGGTGTCTCCTAGGGGAATAAATCTTTGGGCACCTAGTGGTCATG

723_6 CATGACCACTAGGAGCATCTTTGGCGAGATTGGGAGAATCGGAGGCATTGGTGTCTCCTAGGGGAATAAATCTTTGGGCACCTAGTGGTCATG

947_4 CATGACCACTAGGAGCATCTTTGGCGAGATAGGGAGAATCGGCGGCATTGGTGTCTCCTAGGGGAATAAATCTTTGGGCACCTAGTGGTCATG

225_48 CATGACCACTAGGAGCATCTTTGGCGAGATTGGGAGAATCGGCGGCATTGGTGTCTCCTAGGGGAATAAATCTTTGGGCACCTAGTGGTCATG

793_5 CATGACCACTAGGAGCATCTTTGGCGAGATCGGGAGAATCTGTGGCATAGGTGTCTCCTAGGGGAATAAATCTTTGGGCACCTAGTGGTCATG

944_4 CATGACCACTAGGAGCATCTTTGGCGAGATCGGGAGAATCGGTGGCATAGGTGACTCCTAGGGGAATAAATCTTTGGGCACCTAGTGGTCATG

934_4 CATGACCACTAGGAGCATCTTTGGCGAGATCGGGAGAAACGGTGGCATTGGTGACTCCTAGGGGAATAAATCTTTGGGCACCTAGTGGTCATG

783_5 CATGACCACTAGGAGCATCTTTGGCGAGATCGGGAGAATCGGTGGAATTGGTGACTCCTAGGGGAATAAATCTTTGGGCACCTAGTGGTCATG

535_10 CATGACCACTAGGAGCATCTTTGGCGAGATCGGGAGAATTGGTGGCATCGGTGTCTCCTAGGGGAATAAATCTTTGGGCACCTAGTGGTCATG

672_6 CATGACCACTAGGAGCATCTTTGGCGAGATCGGGAGGATCGGTGGTATTGGTGTCTCCTAGGGGAATAAATCTTTGGGCACCTAGTGGTCATG

964_4 CATGACCACTAGGAGCATCTTTGGCGAGATCGGGAGAATTGGTGGTATTGGTGTCTCCTAGGGGAATAAATCTTTGGGCACCTAGTGGTCATG

932_4 CATGACCACTAGGAGCATCTTTGGCGAGATCGGAAGAATTGGTGGCATTGGTGTCTCCTAGGGGAATAAATCTTTGGGCACCTAGTGGTCATG

882_5 CATGACCACTAGGAGCATCTTTGGCGAGATCGGGAGAGTTGGTGGCATTGGTGTCTCCTAGGGGAATAAATCTTTGGGCACCTAGTGGTCATG

540_10 CATGACCACTAGGAGCATCTTTGGCGAGATCGGGAGACTTGGTGGCATTGGTGTCTCCTAGGGGAATAAATCTTTGGGCACCTAGTGGTCATG

873_5 CATGACCACTAGGAGCATCTTTGGCGAGATCGCGAGAATTGGTGGCATTGGTGTCTCCTAGGGGAATAAATCTTTGGGCACCTAGTGGTCATG

728_6 CATGACCACTAGGAGCATCTTTGGCGAGATCGAGAGAATTGGTGGCATTGGTGTCTCCTAGGGGAATAAATCTTTGGGCACCTAGTGGTCATG

428_15 CATGACCACTAGGAGCATCTTTGGCGAGATCGTGAGAATTGGTGGCATTGGTGTCTCCTAGGGGAATAAATCTTTGGGCACCTAGTGGTCATG

737_6 CATGACCACTAGGAGCATCTTTGGCGAGATCGGGAGAATTGGTGGCATTGGTGTTTCCTAGGGGAATAAATCTTTGGGCACCTAGTGGTCATG

727_6 CATGACCACTAGGAGCATCTTTGGCGAGATCGGGAGGATTGGTGGCATTGGTGTCTCCTAGGGGAATAAATCTTTGGGCACCTAGTGGTCATG

681_6 CATGACCACTAGGAGCATCTTTGGCGAGATCGGGAGAATTGGTGGCATTGATGTCTCCTAGGGGAATAAATCTTTGGGCACCTAGTGGTCATG

677_6 CATGACCACTAGGAGCATCTTTGGCGAGATCGGGAGAATTGGTGGCATTGGTGCCTCCTAGGGGAATAAATCTTTGGGCACCTAGTGGTCATG

500_11 CATGACCACTAGGAGCATCTTTGGCGAGATCGGGAGAATCAATGGCATTGGTGTCTCCTAGGGGAATAAATCTTTGGGCACCTAGTGGTCATG

761_5 CATGACCACTAGGAGCATCTTTGGCGAGATCGGGAGAATCGGTGTCATTGTTGTCTCCTAGGGGAATAAATCTTTGGGCACCTAGTGGTCATG

857_5 CATGACCACTAGGAGCATCTTTGGCGAGATCGGGAGAATCGGTGGACTTGGTGTCTCCTAGGGGAATAAATCTTTGGGCACCTAGTGGTCATG

888_4 CATGACCACTAGGAGCATCTTTGGCGAGATCGGGAGAATCTGTGGCATTGGTTTCTCCTAGGGGAATAAATCTTTGGGCACCTAGTGGTCATG

908_4 CATGACCACTAGGAGCATCTTTGGCGAGATCGGGAGAATCGGTGGAATTGTTGTCTCCTAGGGGAATAAATCTTTGGGCACCTAGTGGTCATG

989_4 CATGACCACTAGGAGCATCTTTGGCGAGATCGGGAGAATCTGTGGCATTGTTGTCTCCTAGGGGAATAAATCTTTGGGCACCTAGTGGTCATG

918_4 CATGACCACTAGGAGCATCTTTGGCGAGATCGGGAGAATCTGTGGCATTGGTGACTCCTAGGGGAATAAATCTTTGGGCACCTAGTGGTCATG

798_5 CATGACCACTAGGAGCATCTTTGGCGAGATCTGGAGAATCTGTGGCATTGGTGTCTCCTAGGGGAATAAATCTTTGGGCACCTAGTGGTCATG

767_5 CATGACCACTAGGAGCATCTTTGGCGAGATCGGGAGAATCTGTGGCATTGGTGTTTCCTAGGGGAATAAATCTTTGGGCACCTAGTGGTCATG

839_5 CATGACCACTAGGAGCATCTTTGGCGAGATCGAGAGAAACGGTGGCATTGGTGTCTCCTAGGGGAATAAATCTTTGGGCACCTAGTGGTCATG

624_7 CATGACCACTAGGAGCATCTTTGGCGAGATCGTGAGAATCGGTGGCATAGGTGTCTCCTAGGGGAATAAATCTTTGGGCACCTAGTGGTCATG

953_4 CATGACCACTAGGAGCATCTTTGGCGAGATCGAGAGAATCGGTGGCATCGGTGTCTCCTAGGGGAATAAATCTTTGGGCACCTAGTGGTCATG

563_9 CATGACCACTAGGAGCATCTTTGGCGAGATCGTGAGAATCGGTGGCATCGGTGTCTCCTAGGGGAATAAATCTTTGGGCACCTAGTGGTCATG

446_14 CATGACCACTAGGAGCATCTTTGGCGAGATCGAGAGAATCGGCGGCATTGGTGTCTCCTAGGGGAATAAATCTTTGGGCACCTAGTGGTCATG

291_32 CATGACCACTAGGAGCATCTTTGGCGAGATCGTGAGAATCGGCGGCATTGGTGTCTCCTAGGGGAATAAATCTTTGGGCACCTAGTGGTCATG

777_5 CATGACCACTAGGAGCATCTTTGGCGAGATCGGTAGAATCGGTGGCATTGGTGTTTCCTAGGGGAATAAATCTTTGGGCACCTAGTGGTCATG

913_4 CATGACCACTAGGAGCATCTTTGGCGAGATCGGTAGAATCGGTGTCATTGGTGTCTCCTAGGGGAATAAATCTTTGGGCACCTAGTGGTCATG

268_35 CATGACCACTAGGAGCATCTTTGGCGAGATCGAAAGAATCGGTGGCATTGGTGTCTCCTAGGGGAATAAATCTTTGGGCACCTAGTGGTCATG

923_4 CATGACCACTAGGAGCATCTTTGGCGAGATCGTTAGAATCGGTGGCATTGGTGTCTCCTAGGGGAATAAATCTTTGGGCACCTAGTGGTCATG

571_8 CATGACCACTAGGAGCATCTTTGGCGAGATCGTAAGAATCGGTGGCATTGGTGTCTCCTAGGGGAATAAATCTTTGGGCACCTAGTGGTCATG

738_6 CATGACCACTAGGAGCATCTTTGGCGAGATCGTGAGAATCGGTGGCGTTGGTGTCTCCTAGGGGAATAAATCTTTGGGCACCTAGTGGTCATG

702_6 CATGACCACTAGGAGCATCTTTGGCGAGATCGAGAGAATCGGTGGCGTTGGTGTCTCCTAGGGGAATAAATCTTTGGGCACCTAGTGGTCATG

375_18 CATGACCACTAGGAGCATCTTTGGCGAGATTGTGAGAATCGGTGGCATTGGTGTCTCCTAGGGGAATAAATCTTTGGGCACCTAGTGGTCATG

618_7 CATGACCACTAGGAGCATCTTTGGCGAGATCAGGAGAATTGGTGGCATTGGTGTCTCCTAGGGGAATAAATCTTTGGGCACCTAGTGGTCATG

649_7 CATGACCACTAGGAGCATCTTTGGCGAGATCGTGAGAATCGGTGGCATTGGCGTCTCCTAGGGGAATAAATCTTTGGGCACCTAGTGGTCATG

682_6 CATGACCACTAGGAGCATCTTTGGCGAGATCGTGAGAATCGGTGTCATTGGTGTCTCCTAGGGGAATAAATCTTTGGGCACCTAGTGGTCATG

686_6 CATGACCACTAGGAGCATCTTTGGCGAGATCGAGAGGATCGGTGGCATTGGTGTCTCCTAGGGGAATAAATCTTTGGGCACCTAGTGGTCATG

621_7 CATGACCACTAGGAGCATCTTTGGCGAGATCGTGAGGATCGGTGGCATTGGTGTCTCCTAGGGGAATAAATCTTTGGGCACCTAGTGGTCATG

752_6 CATGACCACTAGGAGCATCTTTGGCGAGATCGGAAGAATCGGTGGCATTGGTGCCTCCTAGGGGAATAAATCTTTGGGCACCTAGTGGTCATG

758_5 CATGACCACTAGGAGCATCTTTGGCGAGATCGTGAGAATCGGTGGCATTGGTGCCTCCTAGGGGAATAAATCTTTGGGCACCTAGTGGTCATG

768_5 CATGACCACTAGGAGCATCTTTGGCGAGATCGCGAGAATCGGTGGCATTGGTGTTTCCTAGGGGAATAAATCTTTGGGCACCTAGTGGTCATG

444_14 CATGACCACTAGGAGCATCTTTGGCGAGATCGTGAGAATCGGTGGCATTGGTGTTTCCTAGGGGAATAAATCTTTGGGCACCTAGTGGTCATG

771_5 CATGACCACTAGGAGCATCTTTGGCGAGATCGTGAGAATCGGTGGTATTGGTGTCTCCTAGGGGAATAAATCTTTGGGCACCTAGTGGTCATG

775_5 CATGACCACTAGGAGCATCTTTGGCGAGATCAGAAGAATCGGTGGCATTGGTGTCTCCTAGGGGAATAAATCTTTGGGCACCTAGTGGTCATG

809_5 CATGACCACTAGGAGCATCTTTGGCGAGATCTGGAGAATCGGTGGCATTTGTGTCTCCTAGGGGAATAAATCTTTGGGCACCTAGTGGTCATG

922_4 CATGACCACTAGGAGCATCTTTGGCGAGATCGTGAGAATCGGTGGCACTGGTGTCTCCTAGGGGAATAAATCTTTGGGCACCTAGTGGTCATG

930_4 CATGACCACTAGGAGCATCTTTGGCGAGATCGGGAGGATCGGTGGCATTGGTGCCTCCTAGGGGAATAAATCTTTGGGCACCTAGTGGTCATG

979_4 CATGACCACTAGGAGCATCTTTGGCGAGATCAGGAGAATCGGTGGCATTGGTGTTTCCTAGGGGAATAAATCTTTGGGCACCTAGTGGTCATG

984_4 CATGACCACTAGGAGCATCTTTGGCGAGATCGGGAGACTCGGTGGCATTGGTGCCTCCTAGGGGAATAAATCTTTGGGCACCTAGTGGTCATG

990_4 CATGACCACTAGGAGCATCTTTGGCGAGATCGTGAGACTCGGTGGCATTGGTGTCTCCTAGGGGAATAAATCTTTGGGCACCTAGTGGTCATG

880_5 CATGACCACTAGGAGCATCTTTGGCGAGATCGTGAGAGTCGGTGGCATTGGTGTCTCCTAGGGGAATAAATCTTTGGGCACCTAGTGGTCATG

333_24 CATGACCACTAGGAGCATCTTTGGCGAGATCAAGAGAATCGGTGGCATTGGTGTCTCCTAGGGGAATAAATCTTTGGGCACCTAGTGGTCATG

960_4 CATGACCACTAGGAGCATCTTTGGCGAGATCATGAGAATCGGTGGCATTGGTGTCTCCTAGGGGAATAAATCTTTGGGCACCTAGTGGTCATG

959_4 CATGACCACTAGGAGCATCTTTGGCGAGATCTTGAGAATCGGTGGCATTGGTGTCTCCTAGGGGAATAAATCTTTGGGCACCTAGTGGTCATG

#=GC RF CAUGACCACUAGGAGCAUCUUUGGCGAGAUCGGGAGAAUCGGUGGCAUUGGUGUCUCCUAGGGGAAUAAAUCUUUGGGCACCUAGUGGUCAUG

#=GC SS_cons (((((((((((((.((.(((((((.(((..........................)))))))))).............)).)))))))))))))

//

1. **II-R1-3 (3_57642) Stockholm file**

# STOCKHOLM 1.0

#=GF SS RNAalifold prediction

237_44 CATGACCACTAGGAGCATCTTTGGCGAGAAGACTCTGGATTCGGGGATTAGTTGCTGCTAGGGGAATAAATCTTTGGGCACCTAGTGGTCATG

411_15 CATGACCACTAGGAGCATCTTTGGCGAGAAGACCTTGGATTCGGGGACCAGTTGCTGCTAGGGGAATAAATCTTTGGGCACCTAGTGGTCATG

501_11 CATGACCACTAGGAGCATCTTTGGCGAGAAGACTTCGGATTCGGGGACCAGTTGCTGCTAGGGGAATAAATCTTTGGGCACCTAGTGGTCATG

582_8 CATGACCACTAGGAGCATCTTTGGCGAGAAGACTTTGGATTCGGGGACTAGTTGCTGCTAGGGGAATAAATCTTTGGGCACCTAGTGGTCATG

734_6 CATGACCACTAGGAGCATCTTTGGCGAGAAGACTTTGGATTTGGGGACCAGTTGCTGCTAGGGGAATAAATCTTTGGGCACCTAGTGGTCATG

884_4 CATGACCACTAGGAGCATCTTTGGCGAGAAGACTTTGGATTCGGGGATCAGTTGCTGCTAGGGGAATAAATCTTTGGGCACCTAGTGGTCATG

899_4 CATGACCACTAGGAGCATCTTTGGCGAGAAGACTCCGGATTCGGGGACCAGTTACTGCTAGGGGAATAAATCTTTGGGCACCTAGTGGTCATG

983_4 CATGACCACTAGGAGCATCTTTGGCGAGAAGACCCTGGATTCGGGGACTAGTTGCTGCTAGGGGAATAAATCTTTGGGCACCTAGTGGTCATG

37_907 CATGACCACTAGGAGCATCTTTGGCGAGAAGACTTTGGATTCGGGGACCAGTTGCTGCTAGGGGAATAAATCTTTGGGCACCTAGTGGTCATG

56_520 CATGACCACTAGGAGCATCTTTGGCGAGAAGACTCCGGATTCGGGGACCAGTTGCTGCTAGGGGAATAAATCTTTGGGCACCTAGTGGTCATG

83_354 CATGACCACTAGGAGCATCTTTGGCGAGAAGACTCTGGATTCGGGGACTAGTTGCTGCTAGGGGAATAAATCTTTGGGCACCTAGTGGTCATG

100_254 CATGACCACTAGGAGCATCTTTGGCGAGAAGACCCTGGATTCGGGGACCAGTTGCTGCTAGGGGAATAAATCTTTGGGCACCTAGTGGTCATG

101_252 CATGACCACTAGGAGCATCTTTGGCGAGAAGACTCTGTATTCGGGGACCAGTTGCTGCTAGGGGAATAAATCTTTGGGCACCTAGTGGTCATG

109_220 CATGACCACTAGGAGCATCTTTGGCGAGAAGACTCTGGATTTGGGGACCAGTTGCTGCTAGGGGAATAAATCTTTGGGCACCTAGTGGTCATG

116_185 CATGACCACTAGGAGCATCTTTGGCGAGAAGACTCTGGATTCGGGGACCAGTTGTTGCTAGGGGAATAAATCTTTGGGCACCTAGTGGTCATG

120_170 CATGACCACTAGGAGCATCTTTGGCGAGAAGACTCAGGATTCGGGGACCAGTTGCTGCTAGGGGAATAAATCTTTGGGCACCTAGTGGTCATG

141_127 CATGACCACTAGGAGCATCTTTGGCGAGAAGATTCTGGATTCGGGGACCAGTTGCTGCTAGGGGAATAAATCTTTGGGCACCTAGTGGTCATG

142_125 CATGACCACTAGGAGCATCTTTGGCGAGAAGACTCTGGATTCGGGGATCAGTTGCTGCTAGGGGAATAAATCTTTGGGCACCTAGTGGTCATG

156_102 CATGACCACTAGGAGCATCTTTGGCGAGAAGACTCTGGATTCGGGGGCCAGTTGCTGCTAGGGGAATAAATCTTTGGGCACCTAGTGGTCATG

163_91 CATGACCACTAGGAGCATCTTTGGCGAGAATACTCTGGATTCGGGGACCAGTTGCTGCTAGGGGAATAAATCTTTGGGCACCTAGTGGTCATG

165_88 CATGACCACTAGGAGCATCTTTGGCGAGAAGACTCTGGGTTCGGGGACCAGTTGCTGCTAGGGGAATAAATCTTTGGGCACCTAGTGGTCATG

180_76 CATGACCACTAGGAGCATCTTTGGCGAGAAGGCTCTGGATTCGGGGACCAGTTGCTGCTAGGGGAATAAATCTTTGGGCACCTAGTGGTCATG

187_71 CATGACCACTAGGAGCATCTTTGGCGAGAAGACTCTGGATTCGGGGACCGGTTGCTGCTAGGGGAATAAATCTTTGGGCACCTAGTGGTCATG

194_65 CATGACCACTAGGAGCATCTTTGGCGAGAAGACTCTGGACTCGGGGACCAGTTGCTGCTAGGGGAATAAATCTTTGGGCACCTAGTGGTCATG

208_56 CATGACCACTAGGAGCATCTTTGGCGAGAAGACTCTGGATTCGGGGACCAGCTGCTGCTAGGGGAATAAATCTTTGGGCACCTAGTGGTCATG

212_55 CATGACCACTAGGAGCATCTTTGGCGAGAAGACTCTGGATTCGGGTACCAGTTGCTGCTAGGGGAATAAATCTTTGGGCACCTAGTGGTCATG

216_52 CATGACCACTAGGAGCATCTTTGGCGAGAAGACTATGGATTCGGGGACCAGTTGCTGCTAGGGGAATAAATCTTTGGGCACCTAGTGGTCATG

217_52 CATGACCACTAGGAGCATCTTTGGCGAGAAGACTCTGGATTCGGGGACCAGTCGCTGCTAGGGGAATAAATCTTTGGGCACCTAGTGGTCATG

224_49 CATGACCACTAGGAGCATCTTTGGCGAGAGGACTCTGGATTCGGGGACCAGTTGCTGCTAGGGGAATAAATCTTTGGGCACCTAGTGGTCATG

241_42 CATGACCACTAGGAGCATCTTTGGCGAGAAGACTCTGGATTCAGGGACCAGTTGCTGCTAGGGGAATAAATCTTTGGGCACCTAGTGGTCATG

242_42 CATGACCACTAGGAGCATCTTTGGCGAGAAGACTCTGAATTCGGGGACCAGTTGCTGCTAGGGGAATAAATCTTTGGGCACCTAGTGGTCATG

246_40 CATGACCACTAGGAGCATCTTTGGCGAGAAGACACTGGATTCGGGGACCAGTTGCTGCTAGGGGAATAAATCTTTGGGCACCTAGTGGTCATG

251_39 CATGACCACTAGGAGCATCTTTGGCGAGAAGACTCTGGATTCGTGGACCAGTTGCTGCTAGGGGAATAAATCTTTGGGCACCTAGTGGTCATG

254_37 CATGACCACTAGGAGCATCTTTGGCGAGAAGACGCTGGATTCGGGGACCAGTTGCTGCTAGGGGAATAAATCTTTGGGCACCTAGTGGTCATG

262_36 CATGACCACTAGGAGCATCTTTGGCGAGAAGACTCTGGATTCGGGGACCAGATGCTGCTAGGGGAATAAATCTTTGGGCACCTAGTGGTCATG

273_34 CATGACCACTAGGAGCATCTTTGGCGAGAAGACTCTAGATTCGGGGACCAGTTGCTGCTAGGGGAATAAATCTTTGGGCACCTAGTGGTCATG

274_34 CATGACCACTAGGAGCATCTTTGGCGAGAAAACTCTGGATTCGGGGACCAGTTGCTGCTAGGGGAATAAATCTTTGGGCACCTAGTGGTCATG

279_34 CATGACCACTAGGAGCATCTTTGGCGAGAAGACTGTGGATTCGGGGACCAGTTGCTGCTAGGGGAATAAATCTTTGGGCACCTAGTGGTCATG

282_33 CATGACCACTAGGAGCATCTTTGGCGAGAAGACTCTGGATTCGGTGACCAGTTGCTGCTAGGGGAATAAATCTTTGGGCACCTAGTGGTCATG

283_33 CATGACCACTAGGAGCATCTTTGGCGAGAAGACTCTGGATTCTGGGACCAGTTGCTGCTAGGGGAATAAATCTTTGGGCACCTAGTGGTCATG

288_32 CATGACCACTAGGAGCATCTTTGGCGAGAAGACTCTGGAGTCGGGGACCAGTTGCTGCTAGGGGAATAAATCTTTGGGCACCTAGTGGTCATG

293_31 CATGACCACTAGGAGCATCTTTGGCGAGAAGACTCTGGATTCGGGGACCAGTAGCTGCTAGGGGAATAAATCTTTGGGCACCTAGTGGTCATG

296_31 CATGACCACTAGGAGCATCTTTGGCGAGAAGACTCTGGATCCGGGGACCAGTTGCTGCTAGGGGAATAAATCTTTGGGCACCTAGTGGTCATG

301_29 CATGACCACTAGGAGCATCTTTGGCGAGAAGACTCTGGATTCGAGGACCAGTTGCTGCTAGGGGAATAAATCTTTGGGCACCTAGTGGTCATG

311_28 CATGACCACTAGGAGCATCTTTGGCGAGAAGACTCTGGATTCGGGGACAAGTTGCTGCTAGGGGAATAAATCTTTGGGCACCTAGTGGTCATG

314_27 CATGACCACTAGGAGCATCTTTGGCGAGAAGACTCTGGATTCGGGGACCAGTTGATGCTAGGGGAATAAATCTTTGGGCACCTAGTGGTCATG

317_27 CATGACCACTAGGAGCATCTTTGGCGAGAAGACTCTGGATTCGGGGACCATTTGCTGCTAGGGGAATAAATCTTTGGGCACCTAGTGGTCATG

324_26 CATGACCACTAGGAGCATCTTTGGCGAGAAGACTCTGGATTCGGGAACCAGTTGCTGCTAGGGGAATAAATCTTTGGGCACCTAGTGGTCATG

327_25 CATGACCACTAGGAGCATCTTTGGCGAGAAGACTCTGGATTCGGAGACCAGTTGCTGCTAGGGGAATAAATCTTTGGGCACCTAGTGGTCATG

337_23 CATGACCACTAGGAGCATCTTTGGCGAGAAGAATCTGGATTCGGGGACCAGTTGCTGCTAGGGGAATAAATCTTTGGGCACCTAGTGGTCATG

352_21 CATGACCACTAGGAGCATCTTTGGCGAGAAGACTCTGGATTCGGGGACCAATTGCTGCTAGGGGAATAAATCTTTGGGCACCTAGTGGTCATG

365_19 CATGACCACTAGGAGCATCTTTGGCGAGAAGACTCTGGAATCGGGGACCAGTTGCTGCTAGGGGAATAAATCTTTGGGCACCTAGTGGTCATG

372_18 CATGACCACTAGGAGCATCTTTGGCGAGAAGACTCTTGATTCGGGGACCAGTTGCTGCTAGGGGAATAAATCTTTGGGCACCTAGTGGTCATG

393_16 CATGACCACTAGGAGCATCTTTGGCGAGAAGACTCTGGATTCGGGGAACAGTTGCTGCTAGGGGAATAAATCTTTGGGCACCTAGTGGTCATG

400_16 CATGACCACTAGGAGCATCTTTGGCGAGAAGACTCGGGATTCGGGGACCAGTTGCTGCTAGGGGAATAAATCTTTGGGCACCTAGTGGTCATG

418_15 CATGACCACTAGGAGCATCTTTGGCGAGAAGACTCTGGATTAGGGGACCAGTTGCTGCTAGGGGAATAAATCTTTGGGCACCTAGTGGTCATG

441_14 CATGACCACTAGGAGCATCTTTGGCGAGAAGACTCTGGATACGGGGACCAGTTGCTGCTAGGGGAATAAATCTTTGGGCACCTAGTGGTCATG

445_14 CATGACCACTAGGAGCATCTTTGGCGAGACGACTCTGGATTCGGGGACCAGTTGCTGCTAGGGGAATAAATCTTTGGGCACCTAGTGGTCATG

481_12 CATGACCACTAGGAGCATCTTTGGCGAGAAGACTCTGGATTCGGGGACCTGTTGCTGCTAGGGGAATAAATCTTTGGGCACCTAGTGGTCATG

482_12 CATGACCACTAGGAGCATCTTTGGCGAGAAGACTCTGGATTCGGGCACCAGTTGCTGCTAGGGGAATAAATCTTTGGGCACCTAGTGGTCATG

502_11 CATGACCACTAGGAGCATCTTTGGCGAGATGACTCTGGATTCGGGGACCAGTTGCTGCTAGGGGAATAAATCTTTGGGCACCTAGTGGTCATG

504_11 CATGACCACTAGGAGCATCTTTGGCGAGAAGACTCTGGATTCGCGGACCAGTTGCTGCTAGGGGAATAAATCTTTGGGCACCTAGTGGTCATG

513_10 CATGACCACTAGGAGCATCTTTGGCGAGAAGACTCTGGATTCCGGGACCAGTTGCTGCTAGGGGAATAAATCTTTGGGCACCTAGTGGTCATG

521_10 CATGACCACTAGGAGCATCTTTGGCGAGAAGACTCTGGTTTCGGGGACCAGTTGCTGCTAGGGGAATAAATCTTTGGGCACCTAGTGGTCATG

525_10 CATGACCACTAGGAGCATCTTTGGCGAGAAGTCTCTGGATTCGGGGACCAGTTGCTGCTAGGGGAATAAATCTTTGGGCACCTAGTGGTCATG

536_10 CATGACCACTAGGAGCATCTTTGGCGAGAAGACTCTGGCTTCGGGGACCAGTTGCTGCTAGGGGAATAAATCTTTGGGCACCTAGTGGTCATG

546_9 CATGACCACTAGGAGCATCTTTGGCGAGAAGACTCTGGATTCGGCGACCAGTTGCTGCTAGGGGAATAAATCTTTGGGCACCTAGTGGTCATG

570_8 CATGACCACTAGGAGCATCTTTGGCGAGAAGACTCTGGATTCGGGGCCCAGTTGCTGCTAGGGGAATAAATCTTTGGGCACCTAGTGGTCATG

652_7 CATGACCACTAGGAGCATCTTTGGCGAGAAGACTCTGGATTCGGGGACCAGGTGCTGCTAGGGGAATAAATCTTTGGGCACCTAGTGGTCATG

685_6 CATGACCACTAGGAGCATCTTTGGCGAGAAGACTCTGGATTCGGGGACGAGTTGCTGCTAGGGGAATAAATCTTTGGGCACCTAGTGGTCATG

714_6 CATGACCACTAGGAGCATCTTTGGCGAGAAGACTCTGGATTCGGGGTCCAGTTGCTGCTAGGGGAATAAATCTTTGGGCACCTAGTGGTCATG

744_6 CATGACCACTAGGAGCATCTTTGGCGAGAAGAGTCTGGATTCGGGGACCAGTTGCTGCTAGGGGAATAAATCTTTGGGCACCTAGTGGTCATG

750_6 CATGACCACTAGGAGCATCTTTGGCGAGAACACTCTGGATTCGGGGACCAGTTGCTGCTAGGGGAATAAATCTTTGGGCACCTAGTGGTCATG

814_5 CATGACCACTAGGAGCATCTTTGGCGAGAAGACTCTGGATTCGGGGACCCGTTGCTGCTAGGGGAATAAATCTTTGGGCACCTAGTGGTCATG

816_5 CATGACCACTAGGAGCATCTTTGGCGAGAAGCCTCTGGATTCGGGGACCAGTTGCTGCTAGGGGAATAAATCTTTGGGCACCTAGTGGTCATG

821_5 CATGACCACTAGGAGCATCTTTGGCGAGAAGACTCTGGATTCGGGGACCAGTTGGTGCTAGGGGAATAAATCTTTGGGCACCTAGTGGTCATG

825_5 CATGACCACTAGGAGCATCTTTGGCGAGAAGACTCTGGATTCGGGGACCACTTGCTGCTAGGGGAATAAATCTTTGGGCACCTAGTGGTCATG

929_4 CATGACCACTAGGAGCATCTTTGGCGAGAAGACTCTGGATTCGGGGAGCAGTTGCTGCTAGGGGAATAAATCTTTGGGCACCTAGTGGTCATG

3_57642 CATGACCACTAGGAGCATCTTTGGCGAGAAGACTCTGGATTCGGGGACCAGTTGCTGCTAGGGGAATAAATCTTTGGGCACCTAGTGGTCATG

998_4 CATGACCACTAGGAGCATCTTTGGCGAGAAGACTCTGGATTCGGGGACCAGTTCCTGCTAGGGGAATAAATCTTTGGGCACCTAGTGGTCATG

406_15 CATGACCACTAGGAGCATCTTTGGCGAGAAGACTCTGGATTCGGGGACCAGTTACTGCTAGGGGAATAAATCTTTGGGCACCTAGTGGTCATG

305_29 CATGACCACTAGGAGCATCTTTGGCGAGAAGACTCTGGATTCGGGGACCAGTTTCTGCTAGGGGAATAAATCTTTGGGCACCTAGTGGTCATG

#=GC RF CAUGACCACUAGGAGCAUCUUUGGCGAGAAGACUCUGGAUUCGGGGACCAGUUGCUGCUAGGGGAAUAAAUCUUUGGGCACCUAGUGGUCAUG

#=GC SS_cons (((((((((((((.((.((((....))))....((((....))))............((((((........)))))))).)))))))))))))

//

1. **II-R1-7 (7_3906) Stockholm file**

# STOCKHOLM 1.0

#=GF SS RNAalifold prediction

297_30 CATGACCACTAGGAGCATCTTTGGCGAGATCGGGAGAATCGGCGGCATTGGTGTCTTCTAGGGGAATAAATCTTTGGGCACCTAGTGGTCATG

341_23 CATGACCACTAGGAGCATCTTTGGCGAGATTGGGAGAATCGGTGGCATTGGTGTCTTCTAGGGGAATAAATCTTTGGGCACCTAGTGGTCATG

404_15 CATGACCACTAGGAGCATCTTTGGCGAGATCGGGAGAATCGGTGGCATTGGTGTTTTCTAGGGGAATAAATCTTTGGGCACCTAGTGGTCATG

448_14 CATGACCACTAGGAGCATCTTTGGCGAGATCGTGAGAATCGGTGGCATTGGTGTCTTCTAGGGGAATAAATCTTTGGGCACCTAGTGGTCATG

503_11 CATGACCACTAGGAGCATCTTTGGCGAGATCGGGAGGATCGGTGGCATTGGTGTCTTCTAGGGGAATAAATCTTTGGGCACCTAGTGGTCATG

510_11 CATGACCACTAGGAGCATCTTTGGCGAGATCGAGAGAATCGGTGGCATTGGTGTCTTCTAGGGGAATAAATCTTTGGGCACCTAGTGGTCATG

511_11 CATGACCACTAGGAGCATCTTTGGCGAGATCGGTAGAATCGGTGGCATTGGTGTCTTCTAGGGGAATAAATCTTTGGGCACCTAGTGGTCATG

544_9 CATGACCACTAGGAGCATCTTTGGCGAGATCGGGAGAATTGGTGGCATTGGTGTCTTCTAGGGGAATAAATCTTTGGGCACCTAGTGGTCATG

601_8 CATGACCACTAGGAGCATCTTTGGCGAGATCGGGAGAATCGGTGGCATCGGTGTCTTCTAGGGGAATAAATCTTTGGGCACCTAGTGGTCATG

648_7 CATGACCACTAGGAGCATCTTTGGCGAGATCAGGAGAATCGGTGGCATTGGTGTCTTCTAGGGGAATAAATCTTTGGGCACCTAGTGGTCATG

661_7 CATGACCACTAGGAGCATCTTTGGCGAGATCGCGAGAATCGGTGGCATTGGTGTCTTCTAGGGGAATAAATCTTTGGGCACCTAGTGGTCATG

673_6 CATGACCACTAGGAGCATCTTTGGCGAGATCGGGAGAATCGGTGGCATAGGTGTCTTCTAGGGGAATAAATCTTTGGGCACCTAGTGGTCATG

703_6 CATGACCACTAGGAGCATCTTTGGCGAGATCGGGGGAATCGGTGGCATTGGTGTCTTCTAGGGGAATAAATCTTTGGGCACCTAGTGGTCATG

776_5 CATGACCACTAGGAGCATCTTTGGCGAGATCGGGAGAGTCGGTGGCATTGGTGTCTTCTAGGGGAATAAATCTTTGGGCACCTAGTGGTCATG

778_5 CATGACCACTAGGAGCATCTTTGGCGAGATCGGAAGAATCGGTGGCATTGGTGTCTTCTAGGGGAATAAATCTTTGGGCACCTAGTGGTCATG

845_5 CATGACCACTAGGAGCATCTTTGGCGAGATCGGGAGAATCGGTGGTATTGGTGTCTTCTAGGGGAATAAATCTTTGGGCACCTAGTGGTCATG

872_5 CATGACCACTAGGAGCATCTTTGGCGAGATCGGCAGAATCGGTGGCATTGGTGTCTTCTAGGGGAATAAATCTTTGGGCACCTAGTGGTCATG

7_3906 CATGACCACTAGGAGCATCTTTGGCGAGATCGGGAGAATCGGTGGCATTGGTGTCTTCTAGGGGAATAAATCTTTGGGCACCTAGTGGTCATG

905_4 CATGACCACTAGGAGCATCTTTGGCGAGATCGGGAGAATCGGTGGCATTGGTGCCTTCTAGGGGAATAAATCTTTGGGCACCTAGTGGTCATG

#=GC RF CAUGACCACUAGGAGCAUCUUUGGCGAGAUCGGGAGAAUCGGUGGCAUUGGUGUCUUCUAGGGGAAUAAAUCUUUGGGCACCUAGUGGUCAUG

#=GC SS_cons (((((((((((((.(((((..((.((.((((....).)))..)).))..)))))..(((((((........)))))))..)))))))))))))

//
